# Measuring progress and projecting attainment on the basis of past trends of the health-related Sustainable Development Goals in 188 countries: an analysis from the Global Burden of Disease Study 2016

**DOI:** 10.1016/S0140-6736(17)32336-X

**Published:** 2017-09-16

**Authors:** GBD 2016 SDG Collaborators, Amanuel Alemu Abajobir, Kalkidan Hassen Abate, Cristiana Abbafati, Kaja M Abbas, Foad Abd-Allah, Rizwan Suliankatchi Abdulkader, Abdishakur M Abdulle, Semaw Ferede Abera, Victor Aboyans, Laith J Abu-Raddad, Niveen M E Abu-Rmeileh, Isaac Akinkunmi Adedeji, Olatunji Adetokunboh, Ashkan Afshin, Anurag Agrawal, Sutapa Agrawal, Aliasghar Ahmad Kiadaliri, Hamid Ahmadieh, Muktar Beshir Ahmed, Miloud Taki Eddine Aichour, Amani Nidhal Aichour, Ibtihel Aichour, Sneha Aiyar, Rufus Olusola Akinyemi, Nadia Akseer, Ziyad Al-Aly, Khurshid Alam, Noore Alam, Deena Alasfoor, Kefyalew Addis Alene, Reza Alizadeh-Navaei, Ala'a Alkerwi, François Alla, Peter Allebeck, Christine Allen, Rajaa Al-Raddadi, Ubai Alsharif, Khalid A Altirkawi, Nelson Alvis-Guzman, Azmeraw T Amare, Erfan Amini, Walid Ammar, Hossein Ansari, Carl Abelardo T Antonio, Palwasha Anwari, Megha Arora, Al Artaman, Krishna Kumar Aryal, Hamid Asayesh, Solomon Weldegebreal Asgedom, Reza Assadi, Tesfay Mehari Atey, Sachin R Atre, Leticia Avila-Burgos, Euripide Frinel G Arthur Avokpaho, Ashish Awasthi, Peter Azzopardi, Umar Bacha, Alaa Badawi, Kalpana Balakrishnan, Marlena S Bannick, Aleksandra Barac, Ryan M Barber, Suzanne L Barker-Collo, Till Bärnighausen, Lope H Barrero, Sanjay Basu, Katherine E Battle, Bernhard T Baune, Justin Beardsley, Neeraj Bedi, Ettore Beghi, Yannick Béjot, Michelle L Bell, Derrick A Bennett, James R Bennett, Isabela M Bensenor, Adugnaw Berhane, Derbew Fikadu Berhe, Eduardo Bernabé, Balem Demtsu Betsu, Mircea Beuran, Addisu Shunu Beyene, Neeraj Bhala, Anil Bhansali, Samir Bhatt, Zulfiqar A Bhutta, Burcu Kucuk Bicer, Hassan Haghparast Bidgoli, Boris Bikbov, Arebu I Bilal, Charles Birungi, Stan Biryukov, Habtamu Mellie Bizuayehu, Christopher D Blosser, Dube Jara Boneya, Dipan Bose, Ibrahim R Bou-Orm, Michael Brauer, Nicholas J K Breitborde, Traolach S Brugha, Lemma Negesa Bulto Bulto, Zahid A Butt, Lucero Cahuana-Hurtado, Ewan Cameron, Julio Cesar Campuzano, Hélène Carabin, Rosario Cárdenas, Juan Jesus Carrero, Austin Carter, Daniel C Casey, Carlos A Castañeda-Orjuela, Ruben Estanislao Castro, Ferrán Catalá-López, Kelly Cercy, Hsing-Yi Chang, Jung-Chen Chang, Fiona J Charlson, Adrienne Chew, Vesper Hichilombwe Chisumpa, Abdulaal A Chitheer, Hanne Christensen, Devasahayam Jesudas Christopher, Massimo Cirillo, Cyrus Cooper, Michael H Criqui, Elizabeth A Cromwell, John A Crump, Lalit Dandona, Rakhi Dandona, Paul I Dargan, José das Neves, Dragos V Davitoiu, Barbora de Courten, Hans De Steur, Barthelemy Kuate Defo, Louisa Degenhardt, Selina Deiparine, Kebede Deribe, Gabrielle A deVeber, Eric L Ding, Shirin Djalalinia, Huyen Phuc Do, Klara Dokova, David Teye Doku, Aaron van Donkelaar, E Ray Dorsey, Tim R Driscoll, Manisha Dubey, Bruce Bartholow Duncan, Beth E Ebel, Hedyeh Ebrahimi, Ziad Ziad El-Khatib, Ahmadali Enayati, Aman Yesuf Endries, Sergey Petrovich Ermakov, Holly E Erskine, Babak Eshrati, Sharareh Eskandarieh, Alireza Esteghamati, Kara Estep, Emerito Jose Aquino Faraon, Carla Sofia e Sa Farinha, André Faro, Farshad Farzadfar, Mir Sohail Fazeli, Valery L Feigin, Andrea B Feigl, Seyed-Mohammad Fereshtehnejad, João C Fernandes, Alize J Ferrari, Tesfaye Regassa Feyissa, Irina Filip, Florian Fischer, Christina Fitzmaurice, Abraham D Flaxman, Nataliya Foigt, Kyle J Foreman, Tahvi Frank, Richard C Franklin, Joseph Friedman, Joseph J Frostad, Nancy Fullman, Thomas Fürst, Joao M Furtado, Emmanuela Gakidou, Alberto L Garcia-Basteiro, Tsegaye Tewelde Gebrehiwot, Johanna M Geleijnse, Ayele Geleto, Bikila Lencha Gemechu, Peter W Gething, Katherine B Gibney, Paramjit Singh Gill, Richard F Gillum, Ababi Zergaw Giref, Melkamu Dedefo Gishu, Giorgia Giussani, Scott D Glenn, William W Godwin, Ellen M Goldberg, Philimon N Gona, Amador Goodridge, Sameer Vali Gopalani, Yevgeniy Goryakin, Max Griswold, Harish Chander Gugnani, Rajeev Gupta, Tanush Gupta, Vipin Gupta, Nima Hafezi-Nejad, Gessessew Bugssa Hailu, Randah Ribhi Hamadeh, Mouhanad Hammami, Graeme J Hankey, Hilda L Harb, Habtamu Abera Hareri, Mohammad Sadegh Hassanvand, Rasmus Havmoeller, Caitlin Hawley, Simon I Hay, Jiawei He, Delia Hendrie, Nathaniel J Henry, Ileana Beatriz Heredia-Pi, Hans W Hoek, Mollie Holmberg, Nobuyuki Horita, H Dean Hosgood, Sorin Hostiuc, Damian G Hoy, Mohamed Hsairi, Aung Soe Htet, John J Huang, Hsiang Huang, Chantal Huynh, Kim Moesgaard Iburg, Chad Ikeda, Manami Inoue, Caleb Mackay Salpeter Irvine, Kathryn H Jacobsen, Nader Jahanmehr, Mihajlo B Jakovljevic, Alejandra Jauregui, Mehdi Javanbakht, Panniyammakal Jeemon, Vivekanand Jha, Denny John, Catherine O Johnson, Sarah Charlotte Johnson, Jost B Jonas, Mikk Jürisson, Zubair Kabir, Rajendra Kadel, Amaha Kahsay, Ritul Kamal, André Karch, Corine Kakizi Karema, Amir Kasaeian, Nicholas J Kassebaum, Anshul Kastor, Srinivasa Vittal Katikireddi, Norito Kawakami, Peter Njenga Keiyoro, Sefonias Getachew Kelbore, Laura Kemmer, Andre Pascal Kengne, Chandrasekharan Nair Kesavachandran, Yousef Saleh Khader, Ibrahim A Khalil, Ejaz Ahmad Khan, Young-Ho Khang, Ardeshir Khosravi, Jagdish Khubchandani, Christian Kieling, Jun Y Kim, Yun Jin Kim, Daniel Kim, Ruth W Kimokoti, Yohannes Kinfu, Adnan Kisa, Katarzyna A Kissimova-Skarbek, Mika Kivimaki, Yoshihiro Kokubo, Jacek A Kopec, Soewarta Kosen, Parvaiz A Koul, Ai Koyanagi, Michael Kravchenko, Kristopher J Krohn, Xie Rachel Kulikoff, G Anil Kumar, Dharmesh Kumar Lal, Michael J Kutz, Hmwe H Kyu, Ratilal Lalloo, Van C Lansingh, Anders Larsson, Jeffrey Victor Lazarus, Paul H Lee, James Leigh, Janni Leung, Ricky Leung, Miriam Levi, Yongmei Li, Misgan Legesse Liben, Stephen S Lim, Shai Linn, Patrick Y Liu, Shiwei Liu, Rakesh Lodha, Katharine J Looker, Alan D Lopez, Stefan Lorkowski, Paulo A Lotufo, Rafael Lozano, Timothy C D Lucas, Raimundas Lunevicius, Mark T Mackay, Emilie R Maddison, Hassan Magdy Abd El Razek, Mohammed Magdy Abd El Razek, Marek Majdan, Reza Majdzadeh, Azeem Majeed, Reza Malekzadeh, Rajesh Malhotra, Deborah Carvalho Malta, Abdullah A Mamun, Helena Manguerra, Lorenzo G Mantovani, Tsegahun Manyazewal, Chabila C Mapoma, Guy B Marks, Randall V Martin, Jose Martinez-Raga, Francisco Rogerlândio Martins-Melo, Ira Martopullo, Manu Raj Mathur, Mohsen Mazidi, Colm McAlinden, Madeline McGaughey, John J McGrath, Martin McKee, Suresh Mehata, Man Mohan Mehndiratta, Toni Meier, Kidanu Gebremariam Meles, Ziad A Memish, Walter Mendoza, Melkamu Merid Mengesha, Mubarek Abera Mengistie, George A Mensah, Gert B M Mensink, Seid Tiku Mereta, Tuomo J Meretoja, Atte Meretoja, Haftay Berhane Mezgebe, Renata Micha, Anoushka Millear, Ted R Miller, Shawn Minnig, Mojde Mirarefin, Erkin M Mirrakhimov, Awoke Misganaw, Shiva Raj Mishra, Philip B Mitchell, Karzan Abdulmuhsin Mohammad, Kedir Endris Mohammed, Shafiu Mohammed, Murali B V Mohan, Ali H Mokdad, Sarah K Mollenkopf, Lorenzo Monasta, Julio Cesar Montañez Hernandez, Marcella Montico, Maziar Moradi-Lakeh, Paula Moraga, Lidia Morawska, Shane D Morrison, Mark W Moses, Cliff Mountjoy-Venning, Ulrich O Mueller, Kate Muller, Christopher J L Murray, Gudlavalleti Venkata Satyanarayana Murthy, Kamarul Imran Musa, Mohsen Naghavi, Aliya Naheed, Kovin S Naidoo, Vinay Nangia, Gopalakrishnan Natarajan, Ruxandra Irina Negoi, Ionut Negoi, Cuong Tat Nguyen, Quyen Le Nguyen, Trang Huyen Nguyen, Grant Nguyen, Minh Nguyen, Emma Nichols, Dina Nur Anggraini Ningrum, Marika Nomura, Vuong Minh Nong, Ole F Norheim, Jean Jacques N Noubiap, Carla Makhlouf Obermeyer, Felix Akpojene Ogbo, In-Hwan Oh, Olanrewaju Oladimeji, Andrew Toyin Olagunju, Tinuke Oluwasefunmi Olagunju, Pedro R Olivares, Helen E Olsen, Bolajoko Olubukunola Olusanya, Jacob Olusegun Olusanya, Kanyin Ong, Eyal Oren, Alberto Ortiz, Mayowa O Owolabi, Mahesh PA, Adrian Pana, Basant Kumar Panda, Songhomitra Panda-Jonas, Christina Papachristou, Eun-Kee Park, George C Patton, Katherine Paulson, David M Pereira, David Norberto Perico, Konrad Pesudovs, Max Petzold, Michael Robert Phillips, David M Pigott, Julian David Pillay, Christine Pinho, Michael A Piradov, Farhad Pishgar, Richie G Poulton, Farshad Pourmalek, Mostafa Qorbani, Amir Radfar, Anwar Rafay, Vafa Rahimi-Movaghar, Mohammad Hifz Ur Rahman, Muhammad Aziz Rahman, Mahfuzar Rahman, Rajesh Kumar Rai, Sasa Rajsic, Usha Ram, Chhabi Lal Ranabhat, Puja C Rao, Salman Rawaf, Patrick Reidy, Robert C Reiner, Nikolas Reinig, Marissa B Reitsma, Giuseppe Remuzzi, Andre M N Renzaho, Serge Resnikoff, Satar Rezaei, Maria Jesus Rios Blancas, Jacqueline Castillo Rivas, Kedir Teji Roba, David Rojas-Rueda, Mohammad Bagher Rokni, Gholamreza Roshandel, Gregory A Roth, Ambuj Roy, Enrico Rubagotti, Nafis Sadat, Mahdi Safdarian, Sare Safi, Saeid Safiri, Rajesh Sagar, Joseph Salama, Joshua A Salomon, Abdallah M Samy, Juan Ramon Sanabria, Damian Santomauro, Itamar S Santos, João Vasco Santos, Milena M Santric Milicevic, Benn Sartorius, Maheswar Satpathy, Monika Sawhney, Sonia Saxena, Mete I Saylan, Maria Inês Schmidt, Ione J C Schneider, Matthew T Schneider, Ben Schöttker, Aletta E Schutte, David C Schwebel, Falk Schwendicke, Soraya Seedat, Sadaf G Sepanlou, Edson E Servan-Mori, Katya Anne Shackelford, Amira Shaheen, Saeid Shahraz, Masood Ali Shaikh, Mansour Shamsipour, Morteza Shamsizadeh, Sheikh Mohammed Shariful Islam, Jayendra Sharma, Rajesh Sharma, Jun She, Peilin Shi, Kenji Shibuya, Chloe Shields, Girma Temam Shifa, Mekonnen Sisay Shiferaw, Mika Shigematsu, Min-Jeong Shin, Rahman Shiri, Reza Shirkoohi, Shreya Shirude, Kawkab Shishani, Haitham Shoman, Mark G Shrime, Donald H Silberberg, Diego Augusto Santos Silva, João Pedro Silva, Dayane Gabriele Alves Silveira, Jasvinder A Singh, Virendra Singh, Dhirendra Narain Sinha, Eirini Skiadaresi, Erica Leigh Slepak, Amber Sligar, David L Smith, Alison Smith, Mari Smith, Badr H A Sobaih, Eugene Sobngwi, Michael Soljak, Samir Soneji, Reed J D Sorensen, Luciano A Sposato, Chandrashekhar T Sreeramareddy, Vinay Srinivasan, Jeffrey D Stanaway, Dan J Stein, Caitlyn Steiner, Sabine Steinke, Mark Andrew Stokes, Bryan Strub, Muawiyyah Babale Sufiyan, Bruno F Sunguya, Patrick J Sur, Soumya Swaminathan, Bryan L Sykes, Dillon O Sylte, Cassandra E I Szoeke, Rafael Tabarés-Seisdedos, Santosh Kumar Tadakamadla, Nikhil Tandon, Tianchan Tao, Yihunie L Tarekegn, Mohammad Tavakkoli, Nuno Taveira, Teketo Kassaw Tegegne, Abdullah Sulieman Terkawi, Gizachew Assefa Tessema, JS Thakur, Kavumpurathu Raman Thankappan, Amanda G Thrift, Tenaw Yimer Tiruye, Ruoyan Tobe-Gai, Roman Topor-Madry, Anna Torre, Miguel Tortajada, Bach Xuan Tran, Christopher Troeger, Thomas Truelsen, Derrick Tsoi, Kald Beshir Tuem, Emin Murat Tuzcu, Stefanos Tyrovolas, Kingsley N Ukwaja, Chigozie Jesse Uneke, Rachel Updike, Olalekan A Uthman, Job F M van Boven, Santosh Varughese, Tommi Vasankari, Narayanaswamy Venketasubramanian, Ramesh Vidavalur, Francesco S Violante, Sergey K Vladimirov, Vasiliy Victorovich Vlassov, Stein Emil Vollset, Theo Vos, Fiseha Wadilo, Tolassa Wakayo, Mitchell T Wallin, Yuan-Pang Wang, Scott Weichenthal, Elisabete Weiderpass, Robert G Weintraub, Daniel J Weiss, Andrea Werdecker, Ronny Westerman, Harvey A Whiteford, Tissa Wijeratne, Charles Shey Wiysonge, Belete Getahun Woldeyes, Charles D A Wolfe, Rachel Woodbrook, Denis Xavier, Gelin Xu, Simon Yadgir, Bereket Yakob, Lijing L Yan, Yuichiro Yano, Mehdi Yaseri, Pengpeng Ye, Hassen Hamid Yimam, Paul Yip, Naohiro Yonemoto, Seok-Jun Yoon, Marcel Yotebieng, Mustafa Z Younis, Zoubida Zaidi, Maysaa El Sayed Zaki, Luis Zavala-Arciniega, Xueying Zhang, Ben Zipkin, Sanjay Zodpey

## Abstract

**Background:**

The UN's Sustainable Development Goals (SDGs) are grounded in the global ambition of “leaving no one behind”. Understanding today's gains and gaps for the health-related SDGs is essential for decision makers as they aim to improve the health of populations. As part of the Global Burden of Diseases, Injuries, and Risk Factors Study 2016 (GBD 2016), we measured 37 of the 50 health-related SDG indicators over the period 1990–2016 for 188 countries, and then on the basis of these past trends, we projected indicators to 2030.

**Methods:**

We used standardised GBD 2016 methods to measure 37 health-related indicators from 1990 to 2016, an increase of four indicators since GBD 2015. We substantially revised the universal health coverage (UHC) measure, which focuses on coverage of essential health services, to also represent personal health-care access and quality for several non-communicable diseases. We transformed each indicator on a scale of 0–100, with 0 as the 2·5th percentile estimated between 1990 and 2030, and 100 as the 97·5th percentile during that time. An index representing all 37 health-related SDG indicators was constructed by taking the geometric mean of scaled indicators by target. On the basis of past trends, we produced projections of indicator values, using a weighted average of the indicator and country-specific annualised rates of change from 1990 to 2016 with weights for each annual rate of change based on out-of-sample validity. 24 of the currently measured health-related SDG indicators have defined SDG targets, against which we assessed attainment.

**Findings:**

Globally, the median health-related SDG index was 56·7 (IQR 31·9–66·8) in 2016 and country-level performance markedly varied, with Singapore (86·8, 95% uncertainty interval 84·6–88·9), Iceland (86·0, 84·1–87·6), and Sweden (85·6, 81·8–87·8) having the highest levels in 2016 and Afghanistan (10·9, 9·6–11·9), the Central African Republic (11·0, 8·8–13·8), and Somalia (11·3, 9·5–13·1) recording the lowest. Between 2000 and 2016, notable improvements in the UHC index were achieved by several countries, including Cambodia, Rwanda, Equatorial Guinea, Laos, Turkey, and China; however, a number of countries, such as Lesotho and the Central African Republic, but also high-income countries, such as the USA, showed minimal gains. Based on projections of past trends, the median number of SDG targets attained in 2030 was five (IQR 2–8) of the 24 defined targets currently measured. Globally, projected target attainment considerably varied by SDG indicator, ranging from more than 60% of countries projected to reach targets for under-5 mortality, neonatal mortality, maternal mortality ratio, and malaria, to less than 5% of countries projected to achieve targets linked to 11 indicator targets, including those for childhood overweight, tuberculosis, and road injury mortality. For several of the health-related SDGs, meeting defined targets hinges upon substantially faster progress than what most countries have achieved in the past.

**Interpretation:**

GBD 2016 provides an updated and expanded evidence base on where the world currently stands in terms of the health-related SDGs. Our improved measure of UHC offers a basis to monitor the expansion of health services necessary to meet the SDGs. Based on past rates of progress, many places are facing challenges in meeting defined health-related SDG targets, particularly among countries that are the worst off. In view of the early stages of SDG implementation, however, opportunity remains to take actions to accelerate progress, as shown by the catalytic effects of adopting the Millennium Development Goals after 2000. With the SDGs' broader, bolder development agenda, multisectoral commitments and investments are vital to make the health-related SDGs within reach of all populations.

**Funding:**

Bill & Melinda Gates Foundation.

**Research in context**

**Evidence before this study**

Since the establishment of the Sustainable Development Goals (SDGs) in September, 2015, an increasing number of global efforts have sought to measure levels and progress in achieving the health-related SDGs. International agencies such as WHO currently report on a subset of the 50 health-related SDG indicators, but inconsistencies in the years reported and countries represented for each SDG indicator provide an incomplete understanding of health priorities in the SDG era. Drawing on the Global Burden of Diseases, Injuries, and Risk Factors Study 2015 (GBD 2015), we measured 33 health-related indicators and an overall health-related SDG index for 188 countries from 1990 to 2015. A number of indicators were not included in this baseline assessment, and some indicators such as universal health coverage (UHC; SDG indicator 3.8.1) had substantial measurement limitations. Demand for initial projections of SDG achievement in 2030, based on past trends, has increased as national and global institutions alike aim to solidify actionable strategies and concrete policy agendas. To date, however, no studies have produced projections across health-related SDG indicators and locations.

**Added value of this study**

Based on work by more than 2500 collaborators from more than 135 countries and territories, GBD 2016 provides an independent and systematic assessment of 37 of the 50 health-related indicators. This represents an increase of four indicators since GBD 2015: vaccine coverage for targeted populations by vaccines in national programmes (SDG indicator 3.b.1); two violence indicators (prevalence of physical or sexual violence [SDG indicator 16.1.3] and childhood sexual abuse [SDG indicator 16.2.3]); and well-certified death registration (SDG indicator 17.19.2c). For the UHC index (SDG indicator 3.8.1), to better represent a full range of essential health services, we combined risk-standardised mortality rates from 32 causes from which death should not occur in the presence of high-quality health care with estimates of nine types of intervention coverage for infectious diseases and maternal and child health outcomes. Based on past trends measured from 1990 to 2016, this study provides projections of each health-related indicator through 2030 and an assessment of attainment against defined SDG targets.

**Implications of all available evidence**

Country-level performance for the health-related SDG index varied greatly in 2016, emphasising health inequalities by location and levels of sociodemographic development. Our improved measure of UHC showed a divide across the sociodemographic spectrum, which might be associated with major differences in access to high-quality health services focused on non-communicable diseases and complex conditions in higher-income countries. Nonetheless, considerable progress occurred for many countries on the UHC index between 2000 and 2016, especially in Cambodia, Rwanda, Equatorial Guinea, Laos, Turkey, and China. Based on projections of past trends, meeting a subset of established SDG targets by 2030 might be possible for some areas of the world, with more than 60% of countries projected to meet targets on under-5 mortality, neonatal mortality, maternal mortality ratio, and malaria. At the same time, on the basis of past trends, much of western and central sub-Saharan Africa was projected to attain very few—if any—defined targets in 2030. Furthermore, at current rates of progress, fewer than 5% of countries were projected to reach 2030 targets for 11 indicators, including childhood overweight, tuberculosis, and road injury mortality. Translation of the global SDG framework into investments and policy remains in its infancy, offering decision makers the opportunity to address both long-standing and emerging health challenges in the SDG era.

## Introduction

“Leaving no one behind” is the cornerstone of the Sustainable Development Goals (SDGs), the international development agenda formally adopted by the UN and its member states in September, 2015.^1^ To deliver on this aim, it is essential to measure where advances have been achieved—and where challenges or new threats are occurring—through routinely updated, comparable monitoring and evaluation.^2^, ^3^ After the SDGs's adoption, debate continued around the SDG indicator framework, implementation, and monitoring,^4^ which ultimately led to an open call for revision proposals overseen by the Inter-Agency and Expert Group on Sustainable Development Goal Indicators (IAEG-SDGs) in 2016. In March, 2017, the UN Statistical Commission agreed on several indicator revisions and established formal mechanisms for ongoing indicator refinement and additions.^5^ At this time, 232 individual SDG indicators are included in the global SDG indicator framework,^5^ aligned with the original 17 goals and 169 targets. 50 health-related indicators (ie, indicators that directly involve health services, health outcomes, and risk factors with well established causal connections to health) exist within 29 health-related targets and 11 goals, including SDG 3, which aims to “ensure healthy lives and promote wellbeing for all at all ages”.

As part of the Global Burden of Diseases, Injuries, and Risk Factors Study 2015 (GBD 2015),^6^ we generated a baseline assessment for 33 health-related SDG indicators, producing an overall summary indicator (the health-related SDG index), and examined historical trends for the overall index and individual indicators for 188 countries from 1990 to 2015. Other efforts have also sought to measure the health-related SDGs across countries, including assessments by the WHO,^7^, ^8^ the Sustainable Development Solutions Network (SDSN),^9^, ^10^ and the World Bank;^11^ however, they experience limitations in terms of the years covered and countries included for each indicator. By contrast, the GBD study uses highly standardised analytical approaches to produce comprehensive and comparable estimates across countries and over time. A collaboration of more than 2500 global health researchers and experts from more than 135 countries and territories enables GBD to incorporate the latest data, reflect regional and local knowledge, and to facilitate policy translation at local levels. Additionally, established mechanisms, including a Scientific Council and Independent Advisory Committee,^12^ ensure scientific rigour and independence from undue political influence.

A key component of the health-related SDGs is universal health coverage (UHC).^13^, ^14^, ^15^, ^16^, ^17^, ^18^ SDG target 3.8 explicitly highlights the importance of UHC, aiming to “achieve universal health coverage, including financial risk protection, access to quality essential health-care services and access to safe, effective, quality, and affordable essential medicines and vaccines for all”.^5^ SDG indicator 3.8.1 focuses on coverage of essential health services, capturing the role of health systems in delivering effective interventions to improve a wide range of health outcomes. On the basis of GBD 2015 results, we developed a proxy measure of UHC based on the coverage of maternal, child, and selected communicable disease interventions.^6^ WHO has proposed a similar proxy UHC measure,^7^, ^19^ although the WHO UHC indicator also seeks to incorporate the absence of selected risk factors at the population level (eg, blood pressure, cholesterol, and smoking). However, measures of risk exposure might not optimally capture access to high-quality health care or broader health system functioning; rather, they might represent behavioural, cultural, or environmental determinants (eg, diet, air pollution) that are less directly addressed by health systems. Considerable opportunity exists to improve current UHC measures by combining more traditional measures of intervention coverage with analyses of amenable mortality, such as those used in the Healthcare Access and Quality (HAQ) Index;^20^ this would allow the incorporation of a broader set of health services as well as reflect both access to and quality of care.

Understanding of how past rates of progress translate into future trajectories for the SDGs is an important input for decision makers, particularly during these initial years of SDG policy development and implementation. Health-related SDG targets and their corresponding indicators represent a substantially broader range of health needs than those represented in the Millennium Development Goals (MDGs), which primarily concentrated on maternal and child health outcomes and infectious diseases;^21^ furthermore, the SDGs are meant to apply to all countries, irrespective of their development status, whereas the MDGs were viewed as lower priority or less applicable to higher-income countries. Subsequently, it is crucial to know where—and how much—progress needs to be accelerated during the next years of SDG implementation to reach stated targets. Selected studies have generated projections based on past trends, but have generally been restricted to specific SDG indicators (eg, under-5 mortality,^22^ maternal mortality,^23^ non-communicable disease [NCD] mortality,^24^ and met need for family planning^25^) or focused on individual countries and indicators (eg, premature mortality from NCDs in Mexico^26^ and child mortality in India^27^). A comprehensive assessment of how past progress could translate into SDG performance in 2030 across health-related indicators is essential to help global, regional, and national decision makers identify the countries and areas of greatest need and align current and future investment plans accordingly.

In this study, we provide updated estimates from 1990 to 2016 for each health-related SDG indicator and the overall health-related SDG index. In doing so, we also improve the measurement for several indicators, most notably the UHC index (SDG indicator 3.8.1) by incorporating components of the HAQ Index. We also include four additional health-related indicators since GBD 2015: vaccine coverage for targeted populations by vaccines in national programmes (SDG indicator 3.b.1), two violence indicators (prevalence of physical or sexual violence [SDG indicator 16.1.3] and childhood sexual abuse [SDG indicator 16.2.3]), and well-certified death registration (SDG indicator 17.19.2c). Based on past trends, we produce indicator-by-indicator projections for 188 countries from 2017 to 2030. It is important to note that these projections are not intended to predict what progress would be achieved as a result of the SDGs; instead, these projections are meant to shed light on potential gaps and gains on the health-related SDGs by 2030, and where countries are likely to be, based on past progress, in relation to defined SDG targets.

## Methods

### Overview of GBD

This analysis of the health-related SDGs is based on the GBD study, which measures the health of populations on an annual basis. GBD produces age-specific, sex-specific, and country-specific estimates (including selected subnational units) of cause-specific mortality and morbidity, risk factor exposure, mortality and morbidity attributable to these risks, and a range of health system characteristics, from 1990 to the most recent year. Various summary measures are computed, including disability-adjusted life-years (DALYs) and healthy life expectancy. GBD uses highly standardised and validated approaches applied to all available data sources adjusted for major sources of bias. Further details on GBD 2016, which covers 1990–2016, are available elsewhere.^28^, ^29^, ^30^, ^31^, ^32^

As with all revisions of the GBD study, GBD 2016 provides an update of the full time series from 1990–2016 based on methodological improvements and newly identified data sources; subsequently, the full time series on the health-related SDGs published here as part of GBD 2016 supersedes previous GBD studies. The GBD 2016 study and this analysis comply with the Guidelines for Accurate and Transparent Health Estimates Reporting (GATHER).^33^ Further detail on the estimation and data sources used for all indicators are available in appendix 1.

### Indicators, definitions, and measurement approach

In this updated analysis we cover 37 of 50 health-related SDG indicators (table). Additional details on data and methods for estimating each indicator are in appendix 1. Appendix 2 outlines the 13 indicators not presently measured (pp 10–12). The addition of new causes, risks, and health indicators are considered by the GBD Scientific Council for each annual cycle of the GBD. For GBD 2016, four health-related SDG indicators were added: vaccine coverage (SDG indicator 3.b.1); two violence indicators (prevalence of physical or sexual violence [SDG indicator 16.1.3] and childhood sexual abuse [SDG indicator 16.2.3]); and well-certified death registration (SDG indicator 17.19.2c).

**Table:** Health-related goals, targets, and health-related SDG indicators used in the present analysis and further details regarding any indicator modifications, and inclusion in the health-related MDG index or health-related non-MDG index

	**Health-related SDG indicator**	**Definition used in this analysis**	**Further details**	**SDG target**	**SDG target used in this analysis**	**Inclusion in MDG or non-MDG index**
**Goal 1: End poverty in all its forms everywhere**						
Target 1.5: By 2030, build the resilience of the poor and those in vulnerable situations and reduce their exposure and vulnerability to climate-related extreme events and other economic, social, and environmental shocks, and disasters	Disaster mortality (1.5.1; same as indicators 11.5.1 and 13.1.1)	Death rate due to exposure to forces of nature (per 100 000 population)	Existing datasets do not comprehensively measure missing persons and people affected by natural disasters; we thus report on deaths due to exposure to forces of nature.	Undefined	··	Non-MDG
**Goal 2: End hunger, achieve food security and improved nutrition, and promote sustainable agriculture**						
Target 2.2: By 2030, end all forms of malnutrition, including achieving, by 2025, the internationally agreed targets on stunting and wasting in children younger than 5 years of age, and address the nutritional needs of adolescent girls, pregnant and lactating women, and older people	Child stunting (2.2.1)	Prevalence of stunting in children younger than 5 years, %	Stunting is defined as below −2 SDs from the median height-for-age of the WHO reference population. No indicator modifications are required.	Eliminate by 2030	≤0·5%	MDG
Target 2.2 (as above)	Child wasting (2.2.2a)	Prevalence of wasting in children younger than 5 years, %	We have separated reporting for indicator 2.2.2 into wasting (2.2.2a) and overweight (2.2.2b). Wasting is defined as below −2 SDs from the median weight-for-height of the WHO reference population.	Eliminate by 2030	≤0·5%	MDG
Target 2.2 (as above)	Child overweight (2.2.2b)	Prevalence of overweight in children aged 2–4 years, %	We used the IOTF thresholds because the WHO cutoff at age 5 years can lead to an artificial shift in prevalence estimates when the analysis covers more age groups. Furthermore, considerably more studies use IOTF cutoffs, which allowed us to build a larger database for estimating child overweight.	Eliminate by 2030	≤0·5%	Non-MDG
**Goal 3: Ensure healthy lives and promote wellbeing for all at all ages**						
Target 3.1: By 2030, reduce the global maternal mortality ratio to less than 70 per 100 000 livebirths	Maternal mortality ratio (3.1.1)	Maternal deaths per 100 000 livebirths in women aged 10–54 years	No indicator modifications required	Reduce to <70 deaths per 100 000 livebirths by 2030	<70 deaths per 100 000 livebirths	MDG
Target 3.1 (as above)	Skilled birth attendance (3.1.2)	Proportion of births attended by skilled health personnel (doctors, nurses, midwives, or country-specific medical staff [eg, clinical officers]), %	No indicator modifications required	Universal access (100%)	≥99%	MDG
Target 3.2: By 2030, end preventable deaths of newborns and children younger than 5 years of age, with all countries aiming to reduce neonatal mortality to at least as low as 12 per 1000 livebirths and under-5 mortality to at least as low as 25 per 1000 livebirths	Under-5 mortality (3.2.1)	Probability of dying before the age of 5 years per 1000 livebirths	No indicator modifications required	Reduce to 25 deaths per 1000 livebirths or lower by 2030	≤25 deaths per 1000 livebirths	MDG
Target 3.2 (as above)	Neonatal mortality (3.2.2)	Probability of dying during the first 28 days of life per 1000 livebirths	No indicator modifications required	Reduce to 12 deaths per 1000 livebirths or lower by 2030	≤12 deaths per 1000 livebirths	MDG
Target 3.3: By 2030, end the epidemics of AIDS, tuberculosis, malaria, and neglected tropical diseases and combat hepatitis, water-borne diseases, and other communicable diseases	HIV incidence (3.3.1)	Age-standardised rate of new HIV infections per 1000 population	We report HIV incidence of all populations and in terms of age-standardised rates	Eliminate by 2030	≤0·005 per 1000 population	MDG
Target 3.3 (as above)	Tuberculosis incidence (3.3.2)	Age-standardised rate of tuberculosis cases per 100 000 population	No indicator modifications required	Eliminate by 2030	≤0·5 per 100 000 population	MDG
Target 3.3 (as above)	Malaria incidence (3.3.3)	Age-standardised rate of malaria cases per 1000 population	No indicator modifications required	Eliminate by 2030	≤0·005 per 1000 population	MDG
Target 3.3 (as above)	Hepatitis B incidence (3.3.4)	Age-standardised rate of hepatitis B incidence per 100 000 population	No indicator modifications required	Undefined	··	Non-MDG
Target 3.3 (as above)	Prevalence of 15 neglected tropical diseases (3.3.5)	Age-standardised prevalence of the sum of 15 neglected tropical diseases, %	People requiring interventions against neglected tropical diseases is not well defined; thus this indicator is revised to the sum of the prevalence of 15 neglected tropical diseases currently measured in the GBD study: African trypanosomiasis, Chagas disease, cystic echinococcosis, cysticerosis, dengue, food-borne trematodiases, Guinea worm, intestinal nematode infections, leishmaniasis, leprosy, lymphatic filariasis, onchocerciasis, rabies, schistosomiasis, and trachoma.	Eliminate by 2030	≤0·5%	Non-MDG
Target 3.4: By 2030, reduce by one-third premature mortality from NCDs through prevention and treatment and promote mental health and wellbeing	Mortality due to a subset of NCDs (3.4·1)	Age-standardised death rate due to cardiovascular disease, cancer, diabetes, and chronic respiratory disease in populations aged 30–70 years per 100 000 population	No indicator modifications required	Reduce by one-third by 2030	Reduce by one-third	Non-MDG
Target 3.4 (as above)	Suicide mortality (3.4.2)	Age-standardised death rate due to self-harm per 100 000 population	No indicator modifications required	Reduce by one-third by 2030	Reduce by one-third	Non-MDG
Target 3.5: Strengthen the prevention and treatment of substance abuse, including narcotic drug abuse and harmful use of alcohol	Alcohol use (3.5.2)	Risk-weighted prevalence of alcohol consumption, as measured by the SEV for alcohol use, %	For this indicator, we include three categories of alcohol consumption because national alcohol consumption per capita does not capture the distribution of use. The SEV for alcohol use is based on two primary dimensions: individual-level drinking (current drinkers and lifetime abstainers, and alcohol consumption by age and sex) and population-level consumption (L per capita of pure alcohol stock). The SEV then weights these categories with their corresponding relative risks, which translate to risk-weighted prevalences on a scale of 0% (no risk in the population) to 100% (the entire population experiences maximum risk associated with alcohol consumption).	Undefined	··	Non-MDG
Target 3.6: By 2020, halve the number of global deaths and injuries from road traffic accidents	Road injury mortality (3.6.1)	Age-standardised death rate due to road injuries per 100 000 population	No indicator modifications required	Reduce by one-half by 2020	Reduce by 50%	Non-MDG
Target 3·7: By 2030, ensure universal access to sexual and reproductive health-care services, including for family planning, information and education, and the integration of reproductive health into national strategies and programmes	Family planning need met, modern contraception methods (3.7.1)	Proportion of women of reproductive age (15–49 years) who have their need for family planning satisfied with modern methods, %	No indicator modifications required	Universal access (100%)	≥99%	MDG
Target 3.7 (as above)	Adolescent birth rate (3.7.2)	Number of livebirths per 1000 women aged 10–14 years and women aged 15–19 years	No indicator modifications required	Undefined	··	MDG
Target 3.8: Achieve universal health coverage, including financial risk protection, access to quality essential health-care services, and access to safe, effective, quality, and affordable essential medicines and vaccines for all	Universal health coverage index (3.8.1)	Coverage of essential health services, as defined by a universal health coverage index of the coverage of nine tracer interventions and risk-standardised death rates from 32 causes amenable to personal health care	Tracer interventions included vaccination coverage (coverage of three doses of diphtheria-pertussis-tetanus, measles vaccine, and three doses of the oral polio vaccine or inactivated polio vaccine); met need for modern contraception; antenatal care coverage (one or more visits and four or more visits); skilled birth attendence coverage; in-facility delivery rates; and coverage of antiretroviral therapy among people living with HIV. The 32 causes amenable to personal health care, which compose the HAQ Index, included tuberculosis, diarrhoeal diseases, lower respiratory infections, upper respiratory infections, diphtheria, whooping cough, tetanus, measles, maternal disorders, neonatal disorders, colon and rectum cancer, non-melanoma cancer, breast cancer, cervical cancer, uterine cancer, testicular cancer, Hodgkin's lymphoma, leukaemia, rheumatic heart disease, ischaemic heart disease, cerebrovascular disease, hypertensive heart disease, peptic ulcer disease, appendicitis, hernia, gallbladder and biliary diseases, epilepsy, diabetes, chronic kidney disease, congenital heart anomalies, and adverse effects of medical treatment. We then scaled these 41 individual inputs on a scale of 0–100, with 0 reflecting the worst levels observed between 1990 and 2016 and 100 reflecting the best observed during this time. We took the arithmetic mean of these 41 scaled indicators so as to collectively capture a wide range of essential health services pertaining to reproductive, maternal, newborn, and child health; infectious diseases; NCDs; and service capacity and access.	Universal access (100%)	≥99%	Non-MDG
Target 3.9: By 2030, substantially reduce the number of deaths and illnesses from hazardous chemicals and air, water, and soil pollution and contamination	Mortality attributable to air pollution (3.9.1)	Age-standardised death rate attributable to household air pollution and ambient air pollution, per 100 000 population	No indicator modifications required	Undefined	··	Non-MDG
Target 3.9 (as above)	Mortality attributable to WaSH (3.9.2)	Age-standardised death rate attributable to unsafe WaSH, per 100 000 population	No indicator modifications required	Undefined	··	Non-MDG
Target 3.9 (as above)	Poisoning mortality (3.9.3)	Age-standardised death rate due to unintentional poisonings, per 100 000 population	No indicator modifications required	Undefined	··	Non-MDG
Target 3.a: Strengthen the implementation of the WHO Framework Convention on Tobacco Control in all countries, as appropriate	Smoking prevalence (3.a.1)	Age-standardised prevalence of daily smoking in populations aged 10 years and older, %	We report daily smoking due to data limitations regarding the systematic measurement of current smoking and to reflect populations aged 10 years and older.	Undefined	··	Non-MDG
Target 3.b: Support the research and development of vaccines and medicines for the communicable and non-communicable diseases that primarily affect developing countries, provide access to affordable essential medicines and vaccines, in accordance with the Doha Declaration on the TRIPS Agreement and Public Health, which affirms the right of developing countries to use to the full the provisions in the Agreement on Trade-Related Aspects of Intellectual Property Rights regarding flexibilities to protect public health, and, in particular, provide access to medicines for all	Vaccine coverage (3.b.1)	Coverage of eight vaccines, conditional on inclusion in national vaccine schedules, in target populations, %	Vaccines included diphtheria-pertussis-tetanus (three doses), measles (one dose), BCG, polio vaccine (three doses), hepatitis B (three doses), *Haemophilus influenzae* type b (three doses), pneumococcal conjugate vaccine (three doses), and rotavirus vaccine (two or three doses). We then used the geometric mean of coverage of these eight vaccines, based on their inclusion in the national vaccine schedule, to compute overall vaccine coverage.	Coverage of all target populations (100%)	≥99%	Non-MDG
**Goal 5: Achieve gender equality and empower all women and girls**						
Target 5.2: Eliminate all forms of violence against all women and girls in the public and private spheres, including trafficking and sexual and other types of exploitation	Intimate partner violence (5.2.1)	Age-standardised prevalence of women aged 15 years and older who experienced physical or sexual violence by an intimate partner in the past 12 months, %	Data for exposure to subtypes of violence are not systematically available across locations and over time; we thus report on physical or sexual violence by an intimate partner.	Eliminate by 2030	≤0·5%	Non-MDG
**Goal 6: Ensure availability and sustainable management of water and sanitation for all**						
Target 6.1: By 2030, achieve universal and equitable access to safe and affordable drinking water for all	Water (6.1·1)	Risk-weighted prevalence of populations using unsafe or unimproved water sources, as measured by the SEV for unsafe water, %	Different types of unsafe water sources have correspondingly different relative risks associated with poor health outcomes; we thus report on the SEV for water, which captures the relative risk of different types of unsafe water sources and then combines them into a risk-weighted prevalence on a scale of 0% (no risk in the population) to 100% (the entire population experiences maximum risk associated with unsafe water).	Universal access to safe water (100%); 0% on the SEV for unsafe water	≤1%	MDG
Target 6.2: By 2030, achieve access to adequate and equitable sanitation and hygiene for all and end open defecation, paying special attention to the needs of women and girls and those in vulnerable situations	Sanitation (6.2·1a)	Risk-weighted prevalence of populations using unsafe or unimproved sanitation, as measured by the SEV for unsafe sanitation, %	We have separated reporting for indicator 6.2.1 into sanitation (6.2.1a) and hygiene (6·2·1b). We had three mutually exclusive, collectively exhaustive categories for sanitation at the household level: households with piped sanitation (with a sewer connection); households with improved sanitation without a sewer connection (pit latrine, ventilated improved latrine, pit latrine with slab, composting toilet), as defined by the JMP; and households without improved sanitation (flush toilet that is not piped to sewer or septic tank, pit latrine without a slab or open pit, bucket, hanging toilet or hanging latrine, shared facilities, no facilities), as defined by the JMP.	Universal access to safe sanitation (100%); 0% on the SEV for unsafe sanitation	≤1%	MDG
Target 6.2 (as above)	Hygiene (6.2.1b)	Risk-weighted prevalence of populations without access to a handwashing facility, as measured by the SEV for unsafe hygiene, %	We have separated reporting for indicator 6.2.1 into sanitation (6.2.1a) and hygiene (6.2.1b). Access to a handwashing facility was defined as having an observed handwashing station with soap and water available in the household.	Universal access to handwashing facility (100%); 0% on the SEV for hygiene	≤1%	Non-MDG
**Goal 7: Ensure access to affordable, reliable, sustainable, and modern energy for all**						
Target 7.1: By 2030, ensure universal access to affordable, reliable, and modern energy services	Household air pollution (7.1.2)	Risk-weighted prevalence of household air pollution, as measured by the SEV for household air pollution, %	Existing datasets do not comprehensively measure population use of clean fuels and technology for heating and lighting across geographies; we thus report on the exposure to clean (or unclean) fuels used for cooking.	Universal access to improved fuels (100%); 0% on the SEV for household air pollution	≤1%	MDG
**Goal 8: Promote sustained, inclusive, and sustainable economic growth, full and productive employment, and decent work for all**						
Target 8.8: Protect labour rights and promote safe and secure working environments for all workers, including migrant workers, in particular women migrants, and those in precarious employment	Disease burden attributable to occupational risks (8.8.1)	Age-standardised all-cause DALY rate attributable to occupational risks per 100 000 population	This indicator is reported as DALY rates attributable to occupational risks because DALYs combine measures of mortality and non-fatal outcomes into a singular summary measure, and occupational risks represent the full range of safety hazards that might be encountered in working environments.	Undefined	··	Non-MDG
**Goal 11: Make cities and human settlements inclusive, safe, resilient, and sustainable**						
Target 11.5: By 2030, significantly reduce the number of deaths and the number of people affected and substantially decrease the direct economic losses relative to global gross domestic product caused by disasters, including water-related disasters, with a focus on protecting the poor and people in vulnerable situations	Disaster mortality (11.5.1; same as indicators 1.5.1 and 13.1.1)	Death rate due to exposure to forces of nature per 100 000 population	Existing datasets do not comprehensively measure missing persons and people affected by natural disasters; we thus report on deaths due to exposure to forces of nature.	Undefined	··	Non-MDG
Target 11.6: By 2030, reduce the adverse per capita environmental impact of cities, including by paying special attention to air quality and municipal and other waste management	Mean PM_2·5_ (11.6.2)	Population-weighted mean levels of PM_2·5_, μg/m^3^	No indicator modifications required	Undefined	··	Non-MDG
**Goal 13: Take urgent action to combat climate change and its impacts**						
Target 13.1: Strengthen resilience and adaptive capacity to climate-related hazards and natural disasters in all countries	Disaster mortality (13.1.1; same as indicators 1.5.1 and 11.5.1)	Death rate due to exposure to forces of nature (per 100 000 population)	Existing datasets do not comprehensively measure missing persons and persons affected by natural disasters; we thus report on deaths due to exposure to forces of nature.	Undefined	··	Non-MDG
**Goal 16: Promote peaceful and inclusive societies for sustainable development, provide access to justice for all, and build effective, accountable, and inclusive institutions at all levels**						
Target 16.1: Significantly reduce all forms of violence and related death rates everywhere	Homicide (16.1.1)	Age-standardised death rate due to interpersonal violence per 100 000 population	No indicator modifications required	Undefined	··	Non-MDG
Target 16.1 (as above)	Conflict and terrorism mortality (16.1·2)	Death rate due to conflict and terrorism per 100 000 population	No indicator modifications required	Undefined	··	Non-MDG
Target 16.1 (as above)	Violence prevalence (16.1.3)	Age-standardised prevalence of physical or sexual violence experienced by populations in the past 12 months, %	Data for exposure to psychological violence are not systematically available across locations and over time; we thus report on prevalence of physical or sexual violence.	Undefined	··	Non-MDG
Target 16.2: End abuse, exploitations, trafficking and all forms of violence against and torture of children	Childhood sexual abuse (16.2.3)	Age-standardised prevalence of women and men aged 18–29 years who experienced sexual violence by age 18 years, %	No indicator modifications required	Eliminate by 2030	≤0·5%	Non-MDG
**Goal 17: Strengthen the means of implementation and revitalise the global partnership for sustainable development**						
Target 17.19: By 2030, build on existing initiatives to develop measurements of progress on sustainable development that complement gross domestic product, and support statistical capacity-building in developing countries	Well-certified death registration (17.19.2c)	Well-certified deaths by a vital registration system among a country's total population, %	Indicator 17.19.2 involves three separate country-level components pertaining to demographic and health data collection and monitoring: status of conducting at least one population and housing census in the past 10 years; birth registration; and death registration. Although these data collection and monitoring systems are inter-connected, their actual status or functionality at a given time can vary. Subsequently, we have separated reporting on 17.19.2 into three indicators, and thus report death registration as 17.19.2c. Well-certified deaths were determined by three measures: completeness of death registration; fraction of deaths not assigned to major garbage codes (ie, causes that cannot or should not be underlying causes of death); and fraction of deaths assigned to detailed GBD causes.	80% of total deaths	≥80%	Non-MDG

Detailed descriptions of the data and methods used to estimate each health-related SDG indicator are in appendix 1. DALY=disability-adjusted life-year. GBD=Global Burden of Disease. HAQ Index=Healthcare Access and Quality Index. IOTF=International Obesity Task Force. JMP=Joint Monitoring Programme. MDG=Millennium Development Goal. NCDs=non-communicable diseases. SDG=Sustainable Development Goal. SEV=summary exposure value. WaSH=water, sanitation, and hygiene. PM_2·5_=fine particulate matter smaller than 2.5 μm.

Vaccine coverage (SDG indicator 3.b.1), defined as “proportion of the target population covered by all vaccines included in their national programme”, became a separate indicator as part of the March, 2017, revision to the SDG framework.^5^ We report on this indicator by using the geometric mean of the coverage of three-dose diphtheria, pertussis, and tetanus (DPT3); three-dose polio; first-dose measles vaccine; and for countries where the vaccine(s) are included in the national schedule: BCG vaccine, three-dose pneumococcal conjugate vaccine (PCV3), three-dose *Haemophilus influenzae* type b vaccine (Hib3), three-dose hepatitis B vaccine (delivered as part of pentavalent vaccines), and two-dose or three-dose rotavirus vaccine. To account for the scale-up period for newly introduced vaccines, we include new vaccines in the geometric mean only 3 years after the introduction year in each country.

We also added two violence indicators in GBD 2016: age-standardised prevalence of physical or sexual violence experienced by populations in the last 12 months (SDG indicator 16.1.3) and age-standardised prevalence of women and men aged 18–29 years who experienced sexual violence by age 18 years (SDG indicator 16.2.3). The UN definition for SDG indicator 16.1.3 includes psychological violence, but due to limited data availability and highly variable definitions of self-reported psychological violence, we restricted this measurement to physical and sexual violence.

As part of GBD 2016, we developed a data quality measure to reflect the proportion of well-certified deaths by a vital registration (VR) system among a country's total population, which corresponds with the third component of 17.19.2 (referred to as SDG indicator 17.19.2c). Well-certified deaths were determined by three measures: (1) completeness of death registration; (2) fraction of deaths not assigned to major garbage codes (ie, causes that cannot or should not be underlying causes of death); and (3) fraction of deaths assigned to detailed GBD causes. More detail on this measure can be found elsewhere ^29^ and in appendix 1.

We also refined the measurement of several previously included health-related indicators. First, SDG indicator 16.1.2 (conflict mortality) now exclusively focuses on deaths due to conflict and terrorism. Second, we revised the exposure period from lifetime to 12 months for SDG indicator 5.2.1 (intimate partner violence) to match the UN SDG definition. Third, we limited our measurement of SDG indicator 6.2.1b (hygiene) to access to a handwashing facility, which also aligns more directly with the UN SDG target. Fourth, we extended the measurement of SDG indicator 3.8.1 (coverage of essential health services, or UHC tracer interventions) to include the individual components of the HAQ Index,^20^ which is based on risk-standardised death rates from 32 causes amenable to personal health care.^34^, ^35^ This revised approach expands the range of potential health services, particularly those for NCDs, captured by this summary measure. The previous UHC tracer indicator included only maternal and child health and selected infectious disease interventions (malaria, HIV, and tuberculosis).^6^ Last, a subset of indicators have undergone substantial revision due to data improvements, methodological improvements, or both, implemented in GBD 2016, including alcohol consumption and child growth failure (ie, under-5 stunting and wasting). Further detail on these updates can be found in appendix 1, as well as accompanying GBD 2016 papers.^28^, ^29^, ^30^, ^31^, ^32^

### Projection of health-related SDG indicators to 2030

We projected the health-related SDG indicators on the basis of past trends. We first calculated for each location the annual rate of change between 1990 and 2016 for each individual year in natural-log space or, for indicators bounded between 0 and 1 (eg, intervention coverage, percentage of population) in logit-space. We then calculated the weighted median annualised rate of change for each country using the following weighting function:

$$weight_{year}=\frac{{\left(\text{year}-1990\right)}^{\omega }}{\sum_{t=1991}^{T}{\left(t-1990\right)}^{\omega }}$$

The value of ω denotes how much weight is given to recent years compared with past years when calculating the median annualised rate of change. To determine the appropriate value of ω for each SDG indicator, we did an out-of-sample predictive validity test in which we held out data for all countries from 2008 to 2016 and predicted values for this time period using the data from 1990 to 2007. We tested values of ω ranging from 0 to 2 in increments of 0·2 and chose the indicator-specific value of ω that minimised the root mean squared error (RMSE) in the held out data (2008–16). This was used to project each indicator to 2030. Appendix 1 provides the indicator specific values of ω used and further details on methods.

For HIV, we used an alternative approach. In many countries, antiretroviral therapy (ART) coverage, through large internal investments, substantial development assistance via programmes such as the President's Emergency Plan for AIDS Relief (PEPFAR),^36^ and reductions in drug prices, has been scaled up considerably. If past trends are used to project future coverage, many countries would be projected to achieve 100% coverage by 2030. This ignores health system constraints in scaling up ART. For ART coverage, our projections were a function of projected ART price based on data from the Global Price Reporting Mechanism (GPRM),^37^ projected government health expenditure as source,^38^ and projected development assistance for health (DAH) for HIV or AIDS.^38^ We bounded ART projections with an ART coverage frontier produced on the basis of income per capita to reflect health system constraints. We then used projected ART coverage to project HIV incidence hazard and HIV incidence using Spectrum.^39^ Further detail on this method is in appendix 1.

### Health-related SDG indices, health-related MDG indices, and health-related non-MDG indices

As in GBD 2015, we developed an overall health-related SDG index that is a function of the 37 health-related SDG indicators (referred to as the health-related SDG index), an index reflecting the 14 SDG health-related indicators previously included in the MDG monitoring framework (referred to as the MDG index), and one reflecting the 23 SDG health-related indicators not included in the MDGs (referred to as the non-MDG index).

A variety of approaches exist to create indices from multidimensional data. As in GBD 2015,^6^ we adopted a preference-weighted approach that weights each indicator by expressed preferences for the relative importance of different indicators. We interpret the SDG targets to represent the expressed preferences of UN member states and thus assume that each SDG target should be treated equally.

To combine indicators, we first transformed each indicator on a scale from 0 to 100. Scaling indicator values in this way allows comparisons to be made on the relative performance on very different SDG indicators and allows us to produce an overall health-related SDG index by calculating an arithmetic or geometric mean of the scaled values. For GBD 2016, we transformed each health-related SDG indicator on a scale from 0 to 100, in order from worst to best, with 0 being the 2·5th percentile value observed over the time period 1990–2030 (ie, including projected values) and 100 the 97·5th percentile value observed during this time. This was implemented in log-space for mortality and incidence rates.

To estimate the health-related SDG index, we first computed the geometric mean of each scaled health-related SDG indicator for a given target, followed by the geometric mean of resulting values across all SDG targets (reflecting the preference-weighted approach described above). The geometric mean allows indicators with very high values to partly compensate for low values on other indicators (referred to as partial substitutability). To avoid problems with indicator values close to 0, when computing indices we applied a floor of 1 to all indicators. The same process was used to construct the MDG and non-MDG indices. Results of sensitivity analyses based on alternative approaches to create the SDG, MDG, and non-MDG indices are detailed in appendix 1.

### SDG indicator attainment

Of the 37 health-related indicators measured in GBD 2016, 24 had defined targets, with 21 having absolute targets to reach by 2030, and three featuring targets relative to 2015 levels (ie, SDG target 3.4, “By 2030, reduce by one third premature mortality from NCDs”). For these 24 indicators, we applied these thresholds to determine achievement by 2030 (or 2020, in the case of road injury mortality [SDG indicator 3.6.1]). 17 health-related indicators had targets citing “achieving elimination”, “ending epidemics”, or “reaching universal coverage or access”. For these indicators we set target thresholds as at least 99% for universal coverage or access and achieving a rate of 0·000005 or less for measures of morbidity (ie, ≤0·005 per 1000 or ≤0·5 per 100 000) and 0·5% for prevalence. The table details the target thresholds or relative reductions applied for each indicator with a defined target.

Because some of these elimination targets have been operationalised in terms of reducing incidence or prevalence by 2030,^40^ we applied a more conservative 80% reduction threshold from 2015 to 2030 for indicators with established elimination SDG targets and compared these results. We also used a threshold of 90% or more for indicators with universal coverage or access in this conservative target attainment scenario.^41^

### Comparing performance on the health-related SDGs across the development spectrum

In addition to examining global patterns in SDG performance, we report on differences in the health-related SDG index and individual indicators across levels of development. To do this, we use the Socio-demographic Index (SDI), a summary measure of overall development that was originally introduced as part of GBD 2015.^30^ SDI is based on income per capita, mean years of education among populations 15 years and older, and total fertility rates, on a scale of 0 to 1. We use the SDI quintiles established in the GBD study to compare performance and progress on the health-related SDGs. More details on the estimation of SDI can be found in accompanying GBD 2016 publications.^28^, ^29^, ^30^, ^31^, ^32^

### Uncertainty analysis

GBD produces 1000 draws for all indicator estimates by location, age, and sex (when relevant) and for all years from 1990 to 2016. These draws from the posterior distribution represent uncertainty in the underlying data sources as well as the various steps in the estimation process. Further details on this are provided in the accompanying GBD 2016 papers^28^, ^29^, ^30^, ^31^, ^32^ and in appendix 1 for each indicator. These 1000 draws are used to determine 95% uncertainty intervals (UIs) in each of the scaled SDG indicators, as well as the three indices, using simulation analysis.

To estimate uncertainty in SDG indicators and indices for the projected values, we applied the median rate of change chosen from the out-of-sample validity test to each of the 1000 draws of the indicator to estimate 1000 draws of each indicator for the time period 2017–30. Additionally, for each of the 1000 draws we allow for year-to-year deviation from the median rate of change on the basis of the variance across all draws.

### Role of the funding source

The funder of the study had no role in the study design, data collection, data analysis, data interpretation, or writing of the report. The corresponding author had full access to all the data in the study and had final responsibility for the decision to submit for publication.

## Results

### Health-related SDGs in 2016

**Figure 1:**
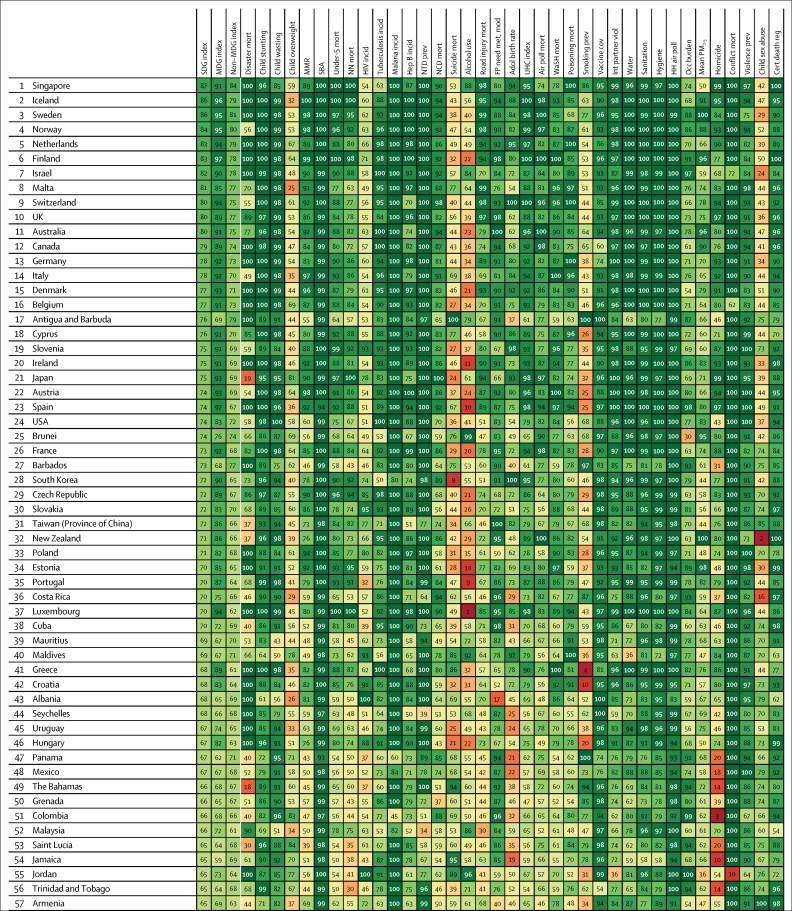

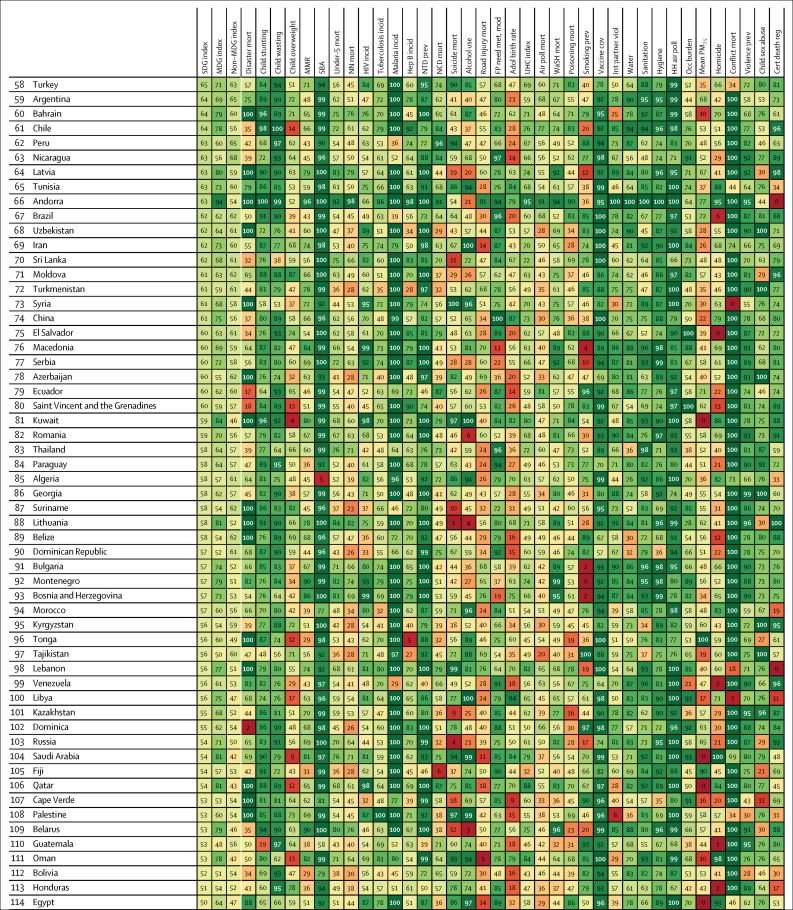

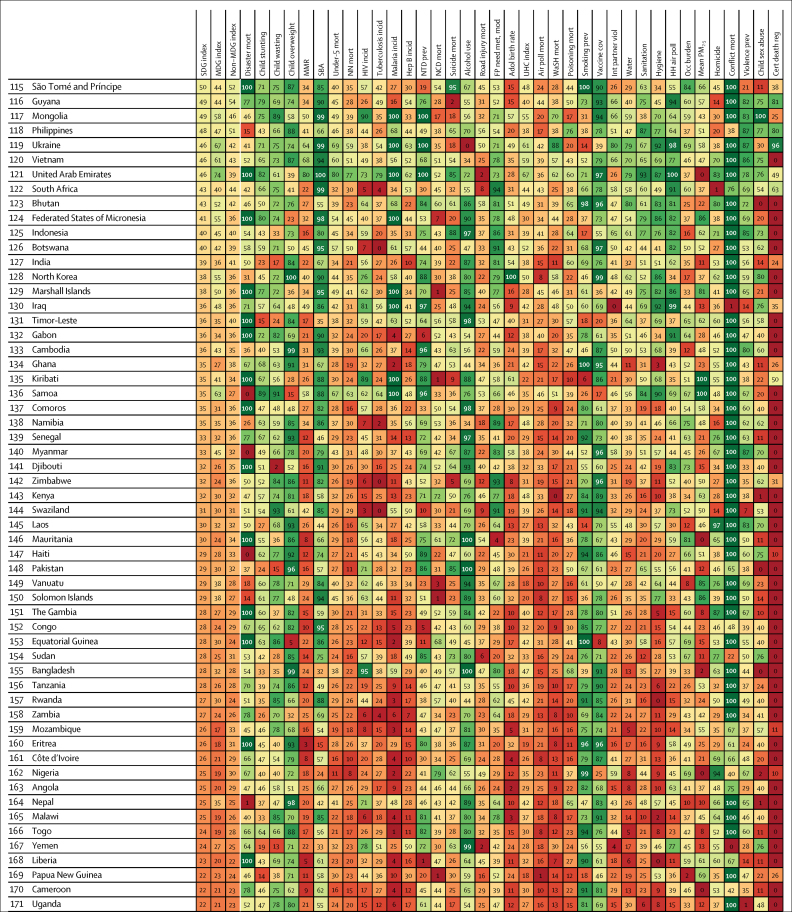

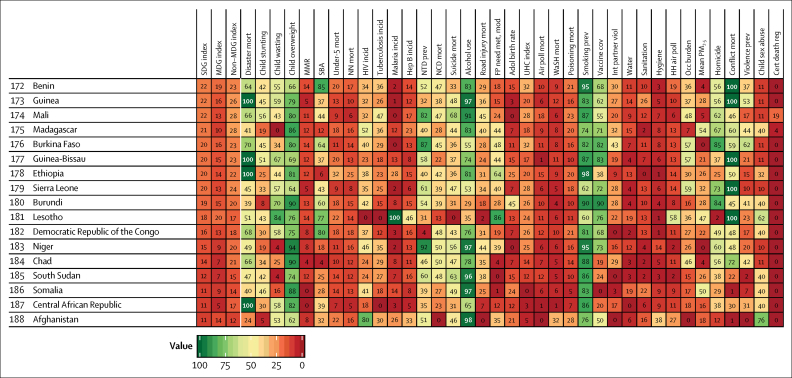
Performance on the health-related SDG index, MDG index, and non-MDG index, and 37 individual health-related indicators, by country, 2016 Countries are ranked by their health-related SDG index from highest to lowest in 2016. Indices and individual indicators are reported on a scale of 0 to 100, with 0 representing the worst levels from 1990 to 2030 and 100 reflecting the best during that time. Definitions of health-related SDG indicators are shown in the table. SDG=Sustainable Development Goal. MDG=Millennium Development Goal. Disaster mort=mortality due to exposure to forces of nature. MMR=maternal mortality ratio. SBA=skilled birth attendance. NN mort=neonatal mortality. Mort=mortality. Incid=incidence. NTD prev=prevalence of 15 neglected tropical diseases. NCD mort=mortality due to a subset of non-communicable diseases (cardiovascular disease, cancer, diabetes, and chronic respiratory diseases). FP need met, mod=family planning need met with modern contraception methods. Adol birth rate=adolescent birth rate. UHC index=universal health coverage index. Air poll mort=mortality attributable to household air pollution and ambient air pollution. WaSH mort=mortality attributable to unsafe water, sanitation, and hygiene. Poisoning mort=mortality due to unintentional poisonings. Smoking prev=prevalence of daily smoking. Vaccine cov=vaccine coverage of target populations based on national vaccine schedules. Int partner viol=prevalence of intimate partner violence. HH air poll=prevalence of household air pollution. Occ burden=disease burden attributable to occupational risks. Mean PM2.5=fine particulate matter smaller than 2·5 μm. Homicide=mortality due to interpersonal violence. Conflict mort=mortality due to conflict and terrorism. Violence prev=prevalence of physical or sexual violence. Child sex abuse=prevalence of childhood sexual abuse. Cert death reg=well-certified death registration.

Globally, the median health-related SDG index was 56·7 (IQR 31·9–66·8) in 2016, with marked country-level variation. Countries with the highest values on the health-related SDG index in 2016 were Singapore (86·8, 95% UI 84·6–88·9), Iceland (86·0, 84·1–87·6), and Sweden (85·6, 81·8–87·8). Countries with the lowest were Afghanistan (10·9, 9·6–11·9), the Central African Republic (11·0, 8·8–13·8), and Somalia (11·3, 9·5–13·1; figure 1). For many health-related indicators, in particular those associated with the MDG era, such as the maternal mortality ratio (MMR), child stunting and wasting, malaria, and environmental risks, higher-SDI countries as expected performed better than lower-SDI countries. For other indicators, including childhood overweight, suicide mortality, harmful alcohol use, smoking, and interpersonal violence mortality, performance was much more heterogeneous across levels of SDI, with many high-SDI countries performing relatively poorly. These findings were exemplified by the USA, which fell below 50 on suicide mortality, harmful alcohol use, and interpersonal violence mortality in 2016. Notably, performance on vaccine coverage, a new indicator as part of the GBD SDG assessment, was generally high across the development spectrum with the exception of the lowest SDI countries; in fact, several middle-SDI countries such as Brazil had among the highest scores.

**Figure 2:**
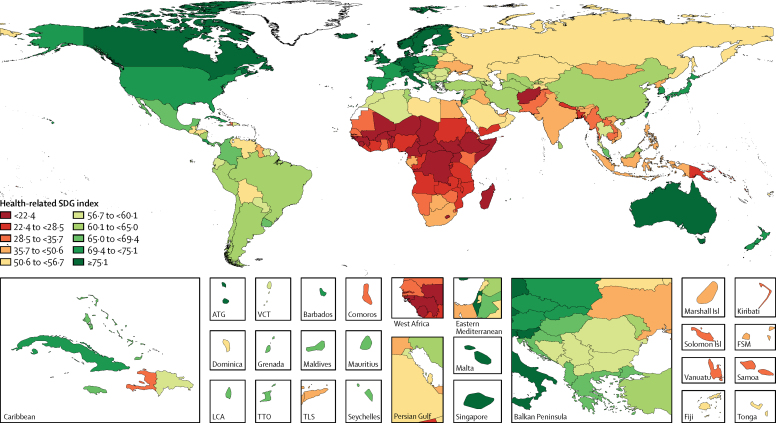
Map of the health-related SDG index, by decile, in 2016 Deciles ranged from less than 22·4 (first decile) to at least 75·1 (tenth decile) in 2016. SDG=Sustainable Development Goal. ATG=Antigua and Barbuda. VCT=Saint Vincent and the Grenadines. LCA=Saint Lucia. TTO=Trinidad and Tobago. Isl=Islands. FSM=Federated States of Micronesia. TLS=Timor-Leste.

In 2016, the highest scores on the health-related SDG index were found among Nordic countries, the UK, a subset of western European countries, Singapore, Australia, Canada, and Israel, with these countries comprising the tenth decile of performance (figure 2). Several western European countries (eg, France, Spain, and Portugal), the USA, New Zealand, Japan, and South Korea occupied the next decile of highest performance on the health-related SDG index. The vast majority of countries in the first decile—places with the lowest scores on the health-related SDG index—were in sub-Saharan Africa, particularly western sub-Saharan Africa, as well as a subset of central and eastern sub-Saharan African countries. Afghanistan was the only country in the first decile outside of sub-Saharan Africa. Other regions showed sizeable differences on the health-related SDG index in 2016; for instance, in Latin America, Costa Rica scored as high as the ninth decile and several countries (ie, Colombia, Mexico, Panama, and Uruguay) were in the eighth decile, whereas Guyana scored as low as the fourth decile. A similar range was found in North Africa and the Middle East, spanning from Jordan in the eighth decile to Yemen in the second decile. China was in the seventh decile on the health-related SDG index in 2016, while Russia was in the fifth decile and India was in the fourth decile.

**Figure 3:**
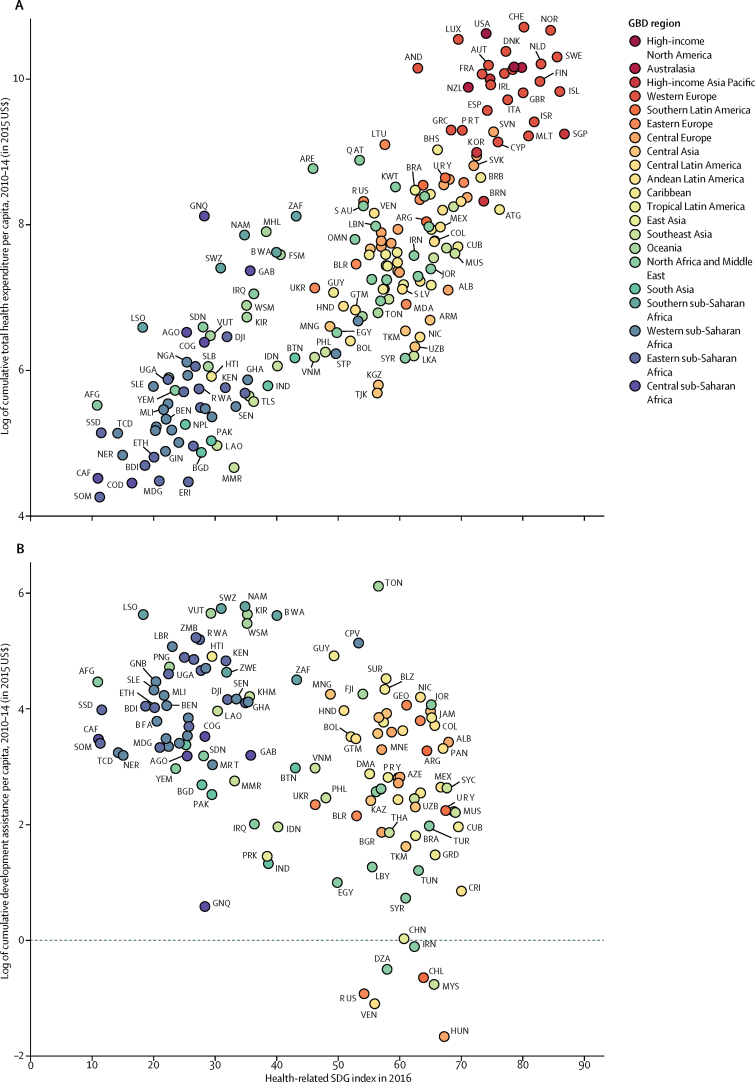
Comparing the health-related SDG index in 2016 to cumulative total health expenditure per capita (A) and cumulative development assistance for health per capita (B), from 2010–14, by GBD region The health-related SDG index is reported on a scale of 0–100, with 0 representing the worst levels from 1990 to 2030 and 100 reflecting the best during that time. Total health expenditure includes development assistance for health and government, out-of-pocket, and pre-paid private health spending. Of the 188 countries in this analysis, 184 had estimates of total health expenditure per capita; North Korea, Palestine, Taiwan (Province of China), and Zimbabwe were excluded due to missing data on national health expenditure. 130 countries were recipients of development assistance for health from 2010 to 2014. Countries are abbreviated according to the ISO3 code. GBD=Global Burden of Disease. SDG=Sustainable Development Goal.

By comparing performance on the health-related SDG index in 2016 with total health expenditure and DAH per capita received from 2010 to 2014 (figure 3),^28^, ^31^ insights might be gleaned regarding the association between overall health funding and performance on the health-related SDG index and whether DAH is being directed toward those countries with the greatest need. Generally, total health expenditure is positively correlated with performance on the health-related SDG index; however, considerable variation exists at the same level of expenditure. For example, among countries with a health-related SDG index of 30 to 70, the association between total health expenditure per capita and performance varied massively, spanning at least a 7 times difference in spending with similar levels of performance on the health-related SDG index. For countries that received DAH between 2010–14, some of the most pronounced differences in cumulative DAH per capita received in the 2016 index were in sub-Saharan Africa, with several countries in southern sub-Saharan Africa posting nearly 3 times more cumulative DAH per capita than a number of countries in central and western sub-Saharan Africa. Most notably, some of the poorest performers on the health-related SDG index, such as the Central African Republic, South Sudan, Somalia, and Niger, received relatively little DAH.

### Progress on UHC

**Figure 4:**
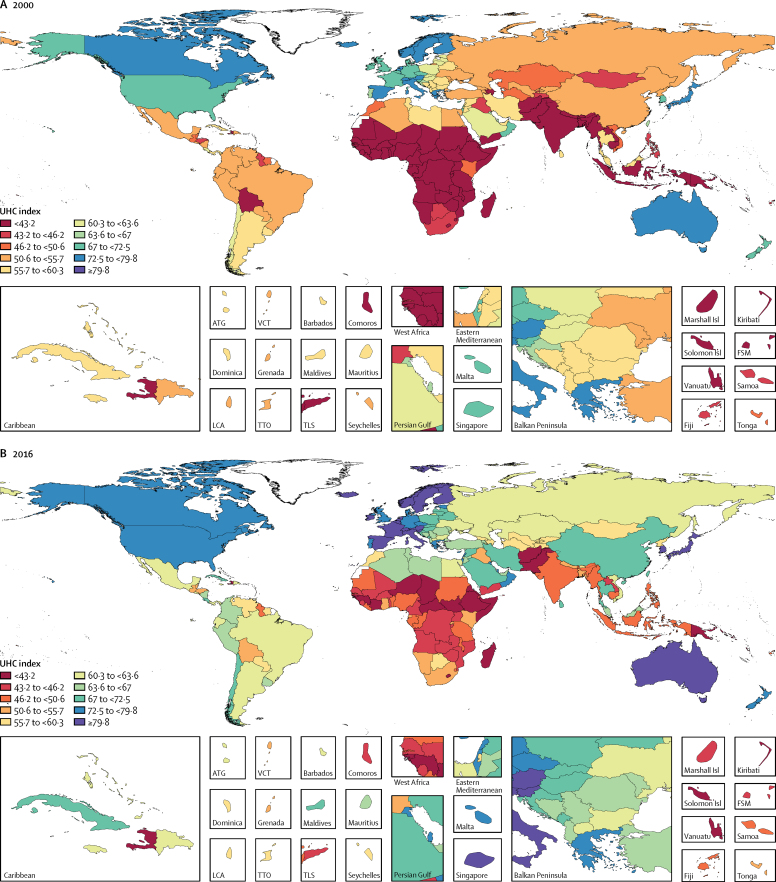
Map of the UHC index, by decile, in 2000 (A) and 2016 (B) Deciles were based on the distribution of UHC index values in 2016 and then were applied for 2000. Deciles ranged from less than 43·2 (first decile) to at least 79·8 (tenth decile) in 2016. UHC=universal health coverage. ATG=Antigua and Barbuda. VCT=Saint Vincent and the Grenadines. LCA=Saint Lucia. TTO=Trinidad and Tobago. Isl=Islands. FSM=Federated States of Micronesia. TLS=Timor-Leste.

Among the health-related indicators refined or added for GBD 2016, the UHC index (SDG indicator 3.8.1) saw the most substantive revision because it was expanded to represent a broader range of essential health services and to capture quality of care. From 2000 to 2016, performance on the UHC index generally improved throughout the world (figure 4 and appendix 2 p 5). Cambodia, Rwanda, Equatorial Guinea, Laos, Turkey, and China recorded the largest improvements since 2000, all recording an increase of 15 or more on the UHC index. Other countries with particularly pronounced progress on the UHC index included Timor-Leste, Bangladesh, Myanmar, and Nepal in Asia and Oceania; Ethiopia and Angola in sub-Saharan Africa; and Lebanon in north Africa and the Middle East. At the same time, a mixture of countries registered minimal progress on the UHC index since 2000. These included lower-SDI countries, such as Lesotho and the Central African Republic, as well as some of the highest-SDI countries, such as the USA and Andorra. In 2016, Switzerland, Iceland, and Finland had the highest UHC index performance, all exceeding 85 on a scale of 0–100, followed by Norway, Sweden, and Japan. Conversely, the lowest levels on the UHC index were in Somalia, the Central African Republic, and Afghanistan, which were all below 35, followed by South Sudan, Chad, and Guinea-Bissau.

### Health-related SDGs in 2030

**Figure 5:**
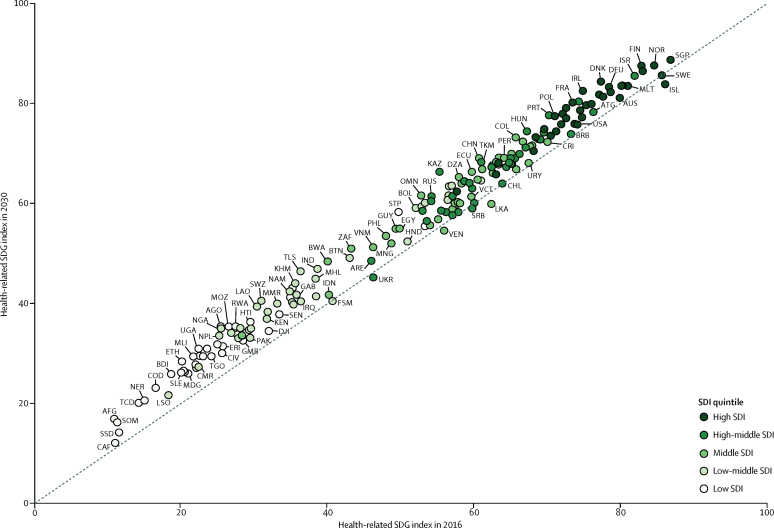
Comparing the health-related SDG index in 2016 and, based on past trends, the projected health-related SDG index in 2030, by country The health-related SDG index is reported on a scale of 0–100, with 0 representing the worst levels from 1990 to 2030 and 100 reflecting the best during that time. The dashed line shows the equivalence line, such that values that fall on this line are equivalent for both the health-related SDG index in 2016 and, based on past trends, the projected health-related SDG index in 2030. Countries are abbreviated according to the ISO3 code. SDI=Socio-demographic Index. SDG=Sustainable Development Goal.

**Figure 6:**
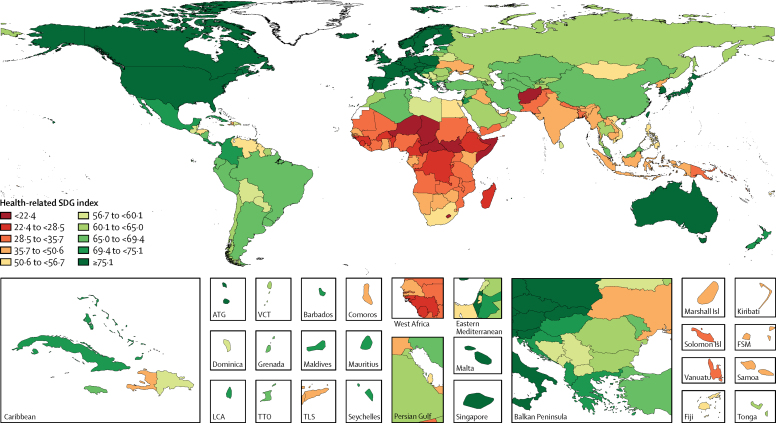
Map of the projected health-related SDG index based on past trends, by decile, in 2030 Deciles were based on the distribution of the health-related SDG index values in 2016 and then were applied for the projected SDG index in 2030. Deciles ranged from less than 22·4 (first decile) to at least 75·1 (tenth decile) in 2016. Projections were based on past trends and rates of change observed from 1990 to 2016. SDG=Sustainable Development Goal. ATG=Antigua and Barbuda. VCT=Saint Vincent and the Grenadines. LCA=Saint Lucia. TTO=Trinidad and Tobago. Isl=Islands. FSM=Federated States of Micronesia. TLS=Timor-Leste.

Based on trends from 1990 to 2016, projections of the health-related SDG index in 2030 generally showed gains (figure 5). Nonetheless, these projections also highlighted the potential for stagnated progress in places, as well as possible worsening of performance by 2030 if current trajectories are not addressed. Kazakhstan, Timor-Leste, Angola, Nigeria, and Swaziland were projected to have the largest improvements by 2030 on the basis of past trends. For most of these countries, projected gains in performance on the UHC index and for a number MDG-related indicators, including MMR, under-5 mortality, neonatal mortality, met need for family planning with modern contraception methods, and skilled birth attendance were shared contributing factors (appendix 2 pp 6–7). Kazakhstan had somewhat different patterns, where projections based on past trends for mortality rates from NCDs, road injuries, and suicide also were among the primary contributing factors for such gains. On the other hand, 2030 projections based on past trends suggest that a subset of countries, including Sri Lanka, Venezuela, Ukraine, and Serbia could experience worsening performance driven by their past trends on indicators including childhood overweight and harmful alcohol use. At the same time several low-middle-SDI countries to low-SDI countries were projected to have marked improvements on the health-related SDG index (eg, Laos, Mozambique, Uganda, Cambodia, Ethiopia, Nepal, and Rwanda). Perhaps most importantly, on the basis of past trends, a subset of low-SDI countries are projected to show minimal progress by 2030 and will continue to have low scores on the health-related SDG index (Figure 5, Figure 6), such as the Central African Republic.

**Projected attainment of defined SDG targets**

**Figure 7:**
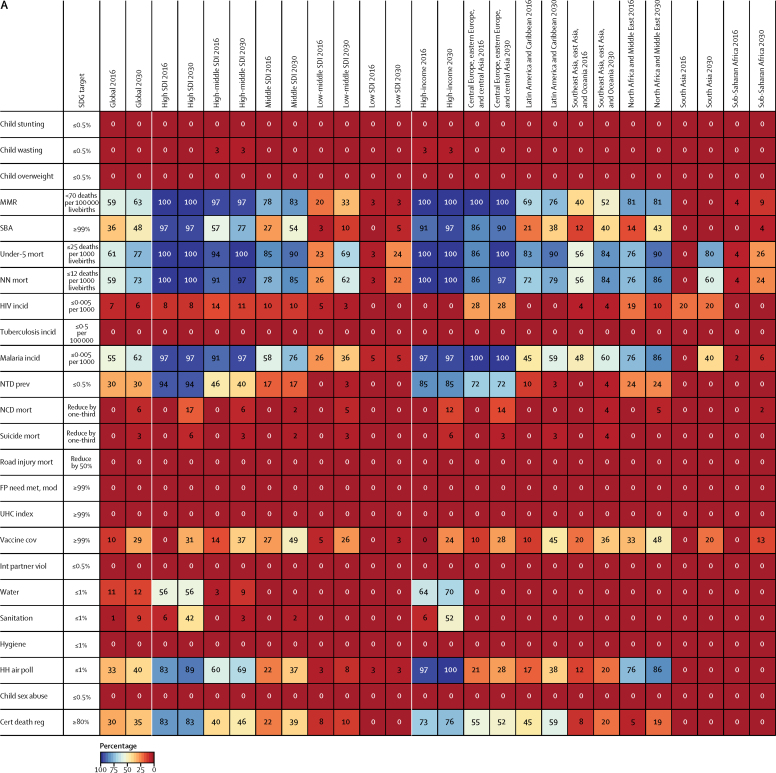

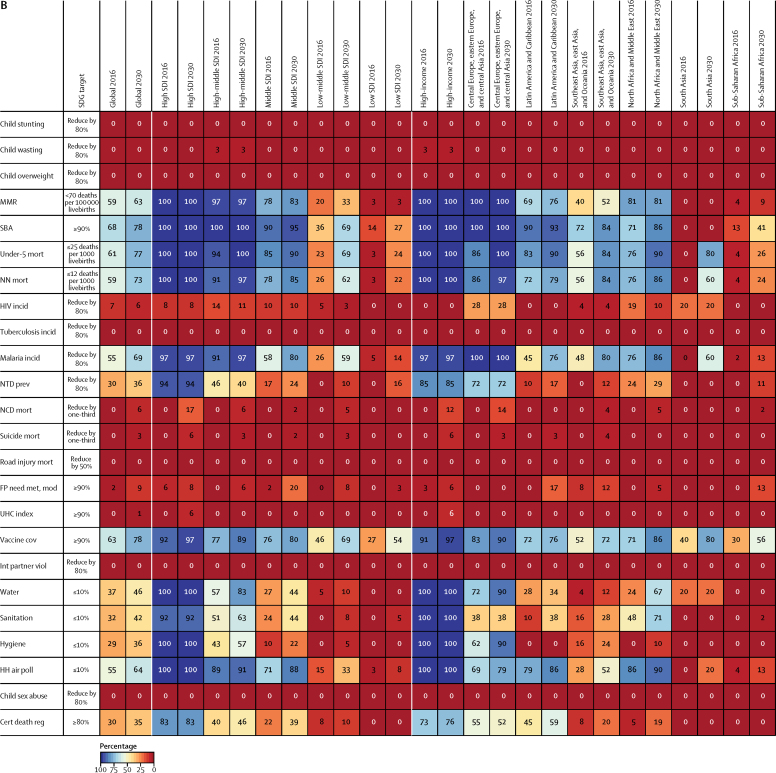
Percentage of countries attaining health-related SDG indicator targets in 2016 and projected to attain in 2030 based on past trends, according to defined SDG targets (A), and defined and conservative SDG targets (B), by indicator, across all countries and by GBD super-region and SDI quintile All projections were based on past trends and rates of change observed from 1990 to 2016. Of the 37 health-related indicators measured in this study, 24 had defined targets linked to each indicator. Definitions of health-related SDG indicators and defined targets associated with them are shown in the table. SDG target 3.6 aims to reduce road injury mortality by 50% between 2015 and 2020, and thus projected attainment for this indicator is based on estimates from 2015 to 2020 rather than 2015 to 2030. For (B), conservative targets were defined as an 80% reduction for elimination targets from 2015 to 2030, and ≥90% by 2030 for universal access or coverage. Under the conservative scenario (B), targets with specific values to meet by 2030 or with specified relative reductions remained as originally defined. SDG=Sustainable Development Goal. SDI=Socio-demographic Index. MMR=maternal mortality ratio. SBA=skilled birth attendance. Under-5 mort=under-5 mortality. NN mort=neonatal mortality. Mort=mortality. Incid=incidence. NTD prev=prevalence of 15 neglected tropical diseases. NCD mort=mortality due to a subset of non-communicable diseases (cardiovascular disease, cancer, diabetes, and chronic respiratory diseases). FP need met, mod=family planning need met with modern contraception methods. UHC index=universal health coverage index. Vaccine cov=vaccine coverage of target populations based on national vaccine schedules. Int partner viol=prevalence of intimate partner violence. HH air poll=prevalence of household air pollution. Child sex abuse=prevalence of childhood sexual abuse. Cert death reg=well-certified death registration.

Based on predictions of past trends for SDG indicators with defined targets, stark differences emerged in terms of projected achievement by 2030 by indicator (figure 7). Globally, more than 60% of countries were projected to attain the SDG targets for under-5 mortality, neonatal mortality, MMR, and malaria by 2030; however, these four indicators all had at least 50% of countries already meeting 2030 targets in 2016. SDG indicators with the next highest levels of projected attainment on the basis of past trends were skilled birth attendance (48% of countries), household air pollution (40%), and well-certified death registration (35%). By contrast, of the 24 SDG indicators with defined targets, 11 indicators had fewer than 5% of countries projected to meet corresponding targets on the basis of past trends. These indicators predominantly involved those calling for eliminating a health challenge (eg, childhood overweight, tuberculosis, and intimate partner violence) or achieving universal coverage or access (eg, met need for family planning and the UHC index). Additionally, on the basis of past trends, few countries were projected to achieve the SDG targets set forth for NCD and suicide mortality (ie, reduce by one-third from 2015 to 2030), with 6% of countries meeting this target for NCD mortality and 3% for suicide. Furthermore, on the basis of past trends, no country was projected to meet the SDG target for road injury mortality, which calls for a 50% reduction from 2015 to 2020.

Overall, based on past trends, projected attainment of defined SDG indicator targets in 2030 was closely associated with SDI, with the highest levels of target achievement occurring among higher-SDI countries. However, among these high-middle and high-SDI countries, the vast majority had already met these targets by 2016, particularly those from the MDG era (ie, MMR, child mortality, malaria, and household air pollution). Some GBD super-regions showed considerable gains for attaining certain SDG targets between 2016 and 2030, including vaccine coverage and household air pollution in Latin America and the Caribbean; skilled birth attendance in southeast Asia, east Asia, and Oceania; and under-5 mortality and neonatal mortality in sub-Saharan Africa. With no south Asian countries attaining the SDG targets for child mortality and malaria in 2016, projections based on past trends pointed to 40% of south Asian countries meeting malaria incidence threshold of 0·005 cases or less per 1000 in 2030, and 80% of countries attaining the SDG target for under-5 mortality and 60% of countries attaining the target for neonatal mortality.

**Figure 8:**
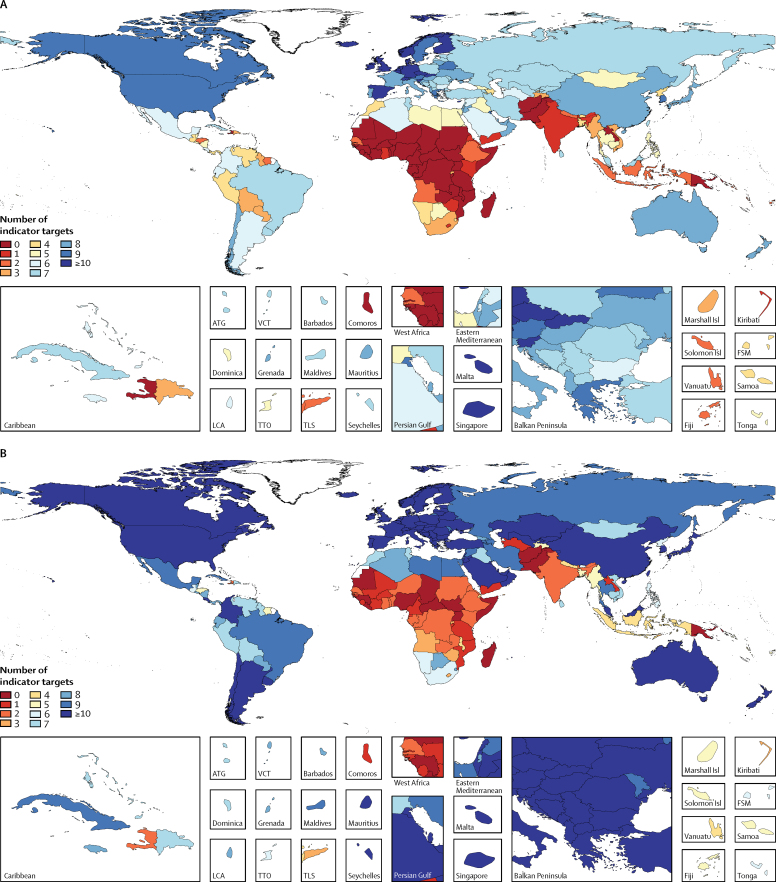
Map of the number of health-related SDG indicator targets, based on past trends, projected to be attained in 2030 according to defined SDG targets (A), and conservative and defined SDG targets (B) All projections were based on past trends and rates of change observed from 1990 to 2016. Of the 37 health-related indicators measured in this study, 24 had defined targets linked to each indicator. Definitions of health-related SDG indicators and defined targets associated with them are shown in the table. SDG target 3.6 aims to reduce road injury mortality by 50% between 2015 and 2020, and thus projected attainment for this indicator is based on estimates from 2015 to 2020 rather than 2015 to 2030. For (B), conservative targets were defined as an 80% reduction for elimination targets from 2015 to 2030, and ≥90% by 2030 for universal access or coverage. For the conservative scenario (B), targets with specific values to meet by 2030 or with specified relative reductions remained as originally defined. SDG=Sustainable Development Goal. ATG=Antigua and Barbuda. VCT=Saint Vincent and the Grenadines. LCA=Saint Lucia. TTO=Trinidad and Tobago. Isl=Islands. FSM=Federated States of Micronesia. TLS=Timor-Leste.

Of the 24 currently measured health-related indicators with defined SDG targets, a median of five (IQR 2–8) indicator targets were projected to be met by 2030, with no country attaining more than 13 (figure 8). On the basis of past trends, 18 countries are projected to meet at least ten indicator targets, including Finland (13 targets), Denmark and Switzerland (11 targets each), and Germany, Ireland, Norway, Singapore, Spain, and the UK each projected to meet ten targets. 31 countries met eight or nine indicator targets in 2030 on the basis of past rates of progress, including Canada, South Korea, Sweden, and the USA (nine targets each), and Australia, Chile, China, and Japan each reaching eight targets. At the other end of the scale, more than 20% of countries (44 of 188) are projected, on the basis of past trends, to meet fewer than two indicator targets in 2030, with most of these countries in sub-Saharan Africa and south Asia. Exceptions to note in sub-Saharan Africa are Botswana and Cape Verde (projected to meet five indicator targets in 2030 on the basis of past trends), Swaziland and Namibia (projected to meet four), and South Africa and Rwanda (projected to meet three).

The use of more conservative target thresholds (ie, 80% reduction from 2015 to 2030 for elimination targets and ≥90% for universal coverage or access targets) resulted in notably higher projected attainment for SDG indicators linked to universal coverage or access targets (figure 7B). This was most pronounced for vaccine coverage, with 78% of countries projected to meet the at least 90% threshold in 2030 on the basis of past trends, compared with 29% of countries meeting at least 99% coverage in 2030. Considerable differences in attainment were also found for skilled birth attendance, water, sanitation, access to hygiene, and household air pollution with at least 90% as the threshold for attainment. Projected attainment based on past trends moderately increased for malaria (69%) and neglected tropical diseases (36%) with the 80% reduction scenario; otherwise, projected attainment for the other SDG indicators with elimination targets did not change with the application of this more conservative target. Globally, the median of indicator targets projected to be met by 2030 increased to eight (IQR 3–11) when more conservative targets were used for elimination and universal coverage or access SDG indicators (figure 8B). More detail on projected attainment of SDG indicator targets based on past trends can be found in appendix 2.

**Figure 9:**
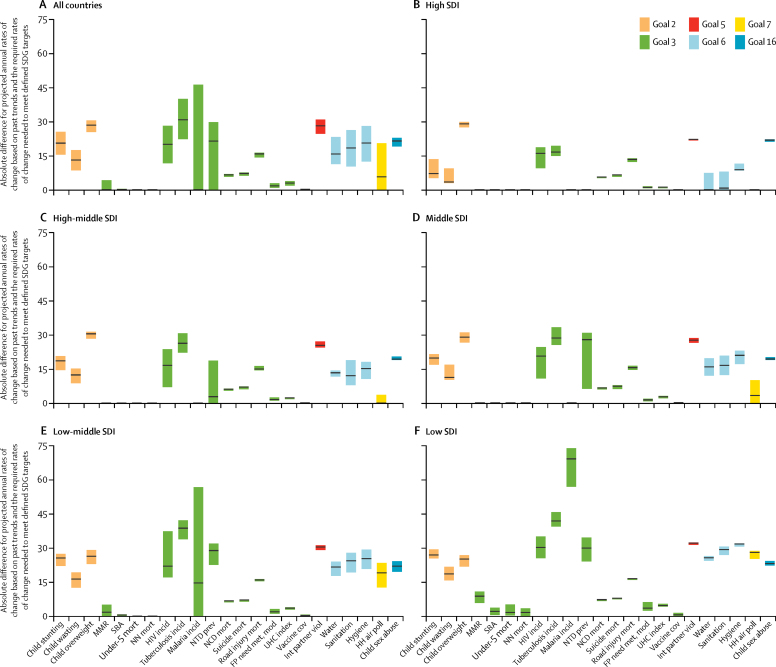
Median and IQR of the absolute difference between projected rates of change from 2016 to 2030 based on past trends and required rate of change needed meet defined SDG targets, by indicator, across all countries (A), high SDI quintile (B), high-middle SDI quintile (C), middle SDI quintile (D), low-middle SDI quintile (E), and low SDI quintile (F) Black stripes represent median absolute change and boxes represent IQR. Health-related indicators are colour-coded according to the health-related goals they represent. All projections were based on past trends and rates of change observed from 1990 to 2016. Of the 37 health-related indicators measured in this study, 24 had defined targets linked to each indicator. Here we present on 23 of these indicators as SDG indicator 17.19.2c, well-certified death registration, had 27 countries with 0% in 2016 and were projected to be the same in 2030. Annualised rates of change observed and required for this indicator were calculated as infinite (and thus implausible). SDG target 3·6 aims to reduce road injury mortality by 50% between 2015 and 2020, and thus annualised rates of change calculated for this indicator are based on estimates from 2015 to 2020 rather than 2015 to 2030. Definitions of health-related SDG indicators and targets associated with them are shown in the table. SDI=Socio-demographic Index. SDG=Sustainable Development Goal. MMR=maternal mortality ratio. SBA=skilled birth attendance. Mort=mortality. Incid=incidence. NN mort=Neonatal mortality. NTD prev=prevalence of 15 neglected tropical diseases. NCD mort=mortality due to a subset of non-communicable diseases (cardiovascular disease, cancer, diabetes, and chronic respiratory diseases). Suicide mort=mortality due to self-harm. FP need met, mod=family planning need met with modern contraception methods. UHC index=universal health coverage index. Vaccine cov=vaccine coverage of target populations based on national vaccine schedules. Int partner viol=intimate partner violence. HH air poll=household air pollution. Child sex abuse=childhood sexual abuse.

Comparing the difference between projected rates of change from 2016 to 2030, on the basis of past progress, and required rates of change that need to be achieved between 2016 and 2030 to meet defined SDG indicator targets can help identify which health areas to prioritise in the SDG era (figure 9). Globally, dramatic acceleration of progress is most needed for indicator targets that call for eliminating health challenges, such as the child malnutrition indicators, especially childhood overweight; infectious diseases, particularly tuberculosis and neglected tropical diseases; and violence indicators. The magnitude by which such gains must be hastened from 2016 to 2030 often differed across the development spectrum, with higher-SDI countries generally requiring far less acceleration than lower-SDI countries. The main exceptions included childhood overweight, road injury mortality, and violence indicators, for which fairly similar rates of acceleration of progress are necessary to reach corresponding SDG targets across levels of SDI.

Further results are in appendix 2, and dynamic visualisations are available online.

## Discussion

### Summary of findings

Formally adopted in 2015, the SDG agenda lays out a series of bold goals and accompanying targets and indicators for attainment by 2030. In the present study, we produced independent and comparable estimates of 37 of the 50 health-related SDG indicators across 188 countries and projected indicators to 2030 on the basis of past trends observed in each country. Our findings show considerable inequality in the health-related SDG index in 2016, spanning from 86·8 in Singapore to 10·9 in Afghanistan. Our revised UHC measure, which incorporates a broader range of tracer indicators including essential health services for NCDs, further highlights geographical and sociodemographic disparities on a key component of the health-related SDGs. Our projections of the health-related SDG indicators point to further entrenchment of these inequalities in the future unless current trajectories are considerably altered. On the basis of past trends, only 21% of health-related SDG indicators with defined targets were projected to be met by 2030, ranging from 38% among high-SDI countries to merely 3% among low-SDI countries. Even when we applied more conservative attainment thresholds, this only increased 30% of health-related SDG indicators with defined targets being met by 2030. Attainment varied considerably across the different indicators, from more than 60% of countries projected to meet 2030 targets on the basis of past trends for under-5 mortality, neonatal mortality, MMR, and malaria to fewer than 5% projected to achieve targets linked to 11 indicator targets, including those for childhood overweight, tuberculosis, and road injury mortality. These projections based on past trends underscore the need for dramatic, if not unprecedented, acceleration of progress to improve health outcomes, reduce risk exposure, and expand essential health services for all countries to achieve the health-related SDGs by 2030. Such action is particularly crucial for countries already showing signs of being left behind, such as the Central African Republic, Afghanistan, Somalia, and South Sudan.

### UHC in the SDG era

Achieving UHC—access to quality essential health services, medicines, and vaccines, and the provision of financial risk protection—is increasingly viewed as imperative to attaining the health-related SDGs.^13^, ^14^, ^15^, ^16^, ^17^, ^18^ Previously, monitoring of progress on the first component of UHC, access to quality essential health services, has been mainly limited to tracking the coverage but not quality of interventions for maternal, reproductive, and child health outcomes and selected communicable diseases. Amid gains in development, many countries' health systems remain unable to fully respond to the rise in NCDs and the demand for more specialised types of medical care.^20^, ^42^ This trend is shown in the divergence by SDI quintile on the more traditional UHC proxy measure and our updated UHC index (appendix 2 p 4), which accounts for an array of NCD outcomes amenable to health care, as well as capturing quality of care.

Although a number of countries saw minimal gains, including low-SDI countries such as Lesotho and the Central African Republic but also high-SDI countries such as the USA, our findings also highlight that progress can be made on expanding UHC. Several countries, including Cambodia, Rwanda, Equatorial Guinea, Laos, Turkey, and China showed substantial improvements on the UHC index between 2000 and 2016. Enacting large-scale health-care reforms and adopting nationwide social health insurance programmes, which enable populations to access essential health services without incurring large financial burdens, are shared characteristics for several countries with notable gains on the UHC index.^43^ Nonetheless, how quickly such government-led initiatives have been formally established, and then how these programmes have been effectively implemented, scaled up, and maintained nationwide vary substantially. This highlights the need for long-term, sustained political commitment to achieving UHC, alongside establishing adequate financing and policies that cover services. For instance, after a change in government in 2002, Turkey introduced its Health Transformation Program in 2003 with the explicit aim of improving public health, providing health insurance for all, and expanding access to care.^44^, ^45^ The phased implementation of UHC-focused reforms in Turkey, alongside continued political support, allowed the country to achieve remarkable strides in achieving UHC and improving care.^44^, ^45^ Yet many country stakeholders and officials fear that the momentum around UHC in Turkey might stall, especially amid increasing regional instability.^44^ China's health-care reforms largely began in the early 2000s, with government-funded insurance schemes increasingly covering rural populations and unemployed urban residents,^46^, ^47^ which was then followed by a more comprehensive health reform in 2009–10 focused on service delivery, essential medicines, public health, insurance, and public hospitals. Strong government commitment to expanding health care to all populations allowed China to make rapid gains in UHC, although concerns about long-term financing and the growth of private insurance are likely to challenge the durability of such political support. Rwanda initiated a pilot programme of its community-based health insurance programme (*Mutuelles de santé* [*Mutuelles*]) in 1999–2000, and then proceeded to formalise and expand the programme nationwide from 2004 to 2008.^48^ Again, strong political commitment to UHC is viewed as a major factor in the rapid expansion of *Mutuelles* in Rwanda;^49^ nonetheless, Rwanda still faces many challenges in terms of UHC financing. In Cambodia, extended health reforms began in the 1990s, gradually rebuilding the country's health system and laying the groundwork for UHC financing arrangements through long-term national health planning.^50^ Cambodia has yet to establish a consolidated national insurance system, though the government recently signalled its commitment to UHC with the March, 2016, Social Health Protection Framework. Overall, our findings on UHC highlight the scope for progress through deliberate, sustained health system investments and political commitment. Further examination of the health system structures, attributes, and financing mechanisms in countries where progress has occurred on the UHC index could offer further insights into how essential health services can be further expanded in health-care settings across the development spectrum.

### Greater investments in health required among the worst off

With its broadened development agenda, the SDGs present substantial financing concerns to higher-income and lower-income countries alike. Projections of government health spending and DAH suggest that absolute levels of overall health spending are likely to remain low among lower-income countries,^38^, ^51^ emphasising the importance of both increased DAH and larger allocations toward health to the extent possible in the SDG era.^52^ Echoing the SDG mantra of “leaving no one behind”, DAH ideally should be targeted toward those with the greatest need. Our analysis shows that a number of countries with the worst performance on the health-related SDG index in 2016 received among the lowest cumulative DAH per capita from 2010 to 2014. The vast majority of these countries were in western and central sub-Saharan Africa, as well as Afghanistan; a number of these countries have experienced protracted conflict or recent surges in civil unrest. Although lower DAH allocations might be justified in settings with limitations in terms of governance or absorptive capacity, in the longer term in the absence of expanded, sustained international support, and increased domestic financing to the extent possible, these countries risk falling further behind in the SDG era.

### Intersectoral action is essential to the health-related SDGs

The confluence of factors leading to poor performance on the SDGs in the worst-off countries underscores how the achievement of several health-related SDG targets will require intersectoral action. Focusing on key sociodemographic factors (eg, improving educational attainment and reducing poverty) might facilitate gains on health-related SDGs.^53^, ^54^ Furthermore, many of the health-related SDGs are not as amenable to traditional DAH-supported programmes.^55^ This is particularly relevant to the health-related SDG indicators for homicide and violence, natural disasters, and conflict; indicators with a strong environmental focus (eg, mean levels of PM_2·5_ [fine particulate matter smaller than 2·5 μm] and mortality attributable to air pollution) or clear links with infrastructure and corresponding laws (eg, road traffic mortality); and broader public health programmes and policies focused on behavioural risk factors (eg, smoking). Ongoing conflict or recent resurgences of violence in the Central African Republic, Afghanistan, Somalia, and South Sudan—countries that were among the worst off in 2016—also risk further entrenching poor health outcomes in the SDG era. War and conflict have widespread, dire ramifications for health systems and related infrastructure, as most recently shown by the 2016–17 cholera outbreak in Yemen as the country's water supply goes untreated.^56^ Several case studies underscore the importance of intersectoral action in making headway on improving SDG indicators, such as legislative successes in combating the tobacco industry in Uruguay^57^ and rebuilding the decimated health system in Timor-Leste after prolonged conflict.^58^

### SDG target setting

Our results showed relatively low levels of projected attainment by 2030 across the health-related SDGs. In GBD 2015, we emphasised the ambitious nature of SDG target 3.3, which calls for ending the epidemics of HIV and tuberculosis; based on our projections of past trends, only 7% of countries were projected to meet the 0·005 cases or less per 1000 threshold for HIV incidence and no country was projected to meet the target for tuberculosis in 2030. A total of 11 indicator targets had fewer than 5% of countries projected to meet them by 2030, including childhood overweight, suicide mortality, and road injury mortality. Although we applied more conservative attainment thresholds (ie, an 80% reduction for elimination targets and ≥90% for universal coverage or access targets), we still found that no country was projected to meet this target for tuberculosis and no additional countries reached this target for HIV. At the same time, coverage measures (eg, vaccine coverage and skilled birth attendance), environmental risks (ie, water, sanitation, access to hygiene, and household air pollution), malaria, and neglected tropical diseases were among the indicators for which using more conservative targets resulted in a larger percentage of countries with projected SDG attainment by 2030. Notably, if the road injury mortality target was extended to 2030 rather than 2020, as the original SDG proposal entailed,^59^ five countries—Austria, Finland, Portugal, Spain, and Slovenia—would have achieved 50% reduction in road injury mortality on the basis of past trends.

For our analysis, we have used the same threshold for SDG targets that call for ending an epidemic or eliminating a health challenge and target universal access or coverage. We recognise, however, that these targets vary considerably in how they are defined quantitatively among stakeholders. For example, in relation to SDG target 3.3, becoming malaria-free involves having no local cases for 3 continuous years and a formal certification process.^60^ By contrast, WHO Global Malaria Technical Strategy for 2016–30 calls for a reduction of incidence of 90% between 2015 and 2030;^61^ the WHO Post-2015 HIV agenda calls for a reduction in HIV incidence by 90% between 2010 and 2030 among adults;^62^ and the Stop TB Global Plan to End TB calls for a reduction in tuberculosis incidence by 80% between 2015 and 2030.^40^ Furthermore, confusion or tension might arise around reconciling ambitious SDG nutrition targets (ie, SDG target 2.2 “By 2030, end all forms of malnutrition, including achieving, by 2025, the internationally agreed targets on stunting and wasting in children”) with the Global Nutrition Targets 2025, which established somewhat different aims (ie, reduce stunting by 40% from 2010 to 2025 and reduce wasting to <5% by 2025).^63^ A delicate balance exists between identification of targets that spur progress but are at the same time achievable. Our projections of SDG achievement might help to identify ambitious yet more feasible targets across these indicators (eg, using the 95th percentile in levels achieved by 2030). They also might be of particular utility to national monitoring agencies as they develop and implement national-level targets to complement the global SDG indicator framework.^5^

### Comparisons with other assessments

To date, a number of other international organisations or collaborations have reported on country-level estimates of SDG indicators, including WHO, SDSN, and the World Bank.^7^, ^10^, ^11^ Of the 50 health-related indicators currently included in the global SDG framework, GBD 2016 reported on 37, whereas WHO included 34 in the 2017 World Health Statistics report, the World Bank covered 28 for the 2017 SDG Atlas, and the 2017 SDSN SDG index included 23. The GBD study offers a number of advantages for monitoring progress on the health-related SDGs, which includes producing comparable, comprehensive indicator estimates for all 188 countries from 1990 to 2016. By contrast, substantial variation was found for country inclusion across the health-related indicators reported by other organisations and collaborations. For instance, WHO provided estimates for 194 of 194 member states for under-5 mortality, neonatal mortality, and tuberculosis, yet only 104 countries had estimates for HIV, 122 for met need for family planning, and 128 for smoking prevalence. Additionally, no other agency measuring the health-related SDGs provides a complete and consistent set of years. Across these organisations, the latest year of reporting ranges from 2013 to 2016, and for several indicators, data from a range of years are combined to represent the most recent year available in countries. Examples include 2005 to 2015 or 2016 for skilled birth attendance and met need for family planning as reported by WHO. Last, proxy or partial measures are currently used by other agencies or collaborations for a subset of health-related SDG indicators, which can be directly measured by the GBD. For instance, the UN definition for SDG indicator 2.2.2 includes childhood overweight, yet the prevalence of adult obesity is reported by SDSN for this indicator. Another example is limiting the measure of met need for family planning to married and in-union women when the IAEG-SDGs metadata indicator definition explicitly includes all women of reproductive age. To properly monitor progress and identify challenges in achieving the health-related SDGs, it is critical to measure indicators' levels and trends in a timely, comparable, and complete manner.

### Future GBD SDG monitoring

With its annual cycle, the GBD study has enabled the incorporation of a number of important revisions and additions to monitor the health-related SDGs. For GBD 2016, the most notable advances include improving UHC index (SDG indicator 3.8.1) to reflect a broader array of conditions covered by essential health services, as well as the addition of two violence indicators and vaccine coverage (SDG indicator 3.b.1). For vaccine coverage, we plan to further improve its measurement in GBD 2017, namely estimating the correlation structure between the coverage of individual vaccines to more precisely measure the UN's definition for SDG indicator 3.b.1 (ie, proportion of the target population covered by all vaccines included in their national programme).

The continued expansion and refinement of health-related indicator measurement is a key priority for GBD 2017 and beyond. Specifically, an assessment of health worker density and distribution (SDG indicator 3.c.1) is presently underway and will be included in GBD 2017. Pending data availability, estimating the proportion of people who feel safe walking alone around the area where they live (SDG indicator 16.1.4) also should be feasible for GBD 2017. Work is underway to estimate the coverage of treatment interventions for substance use disorders (SDG indicator 3.5.1), as well as the prevalence of sexual violence by non-intimate partners for women aged 15 years and older (SDG indicator 5.2.2). To date, however, estimating sexual violence by non-intimate partners has been severely limited by a paucity of data outside of western Europe and the USA. A key area of future work relates to the March, 2017, revisions to SDG indicator 3.8.2,^5^ which was modified to more directly capture financial risk protection by including an indicator of catastrophic household expenditures on health.

In the present study, we used a relatively simple approach for projecting trends for health-related SDG indicators through 2030. This method is based on using the historical rates of change for each country, with more recent trends weighted more heavily. This approach does not explicitly link the likelihood of SDG achievement to underlying investment areas to reach the SDGs; for example, increasing overall or specific types of DAH, enacting socioeconomic policies, implementing health programmes, expanding coverage of currently available interventions, scaling up new interventions or medical technologies, and reducing or preventing exposure to underlying risks. This more structured approach for projecting SDG achievement—including the ability to quantify the potential impact of different SDG investment scenarios—is currently under development as part of the GBD.

### Strengths

A number of strengths, as well as limitations, exist for our study. The extensive GBD collaboration, which currently includes more than 2500 individuals from more than 135 countries and territories, is a core strength. This collective ownership model, in which GBD collaborators actively participate in the production, review, and use of results, addresses many recent critiques regarding the generation of global health estimates.^64^ Specifically, the GBD collaborative network identifies and facilitates access to the latest, locally relevant data sources; works with individuals and institutions in reviewing and generating GBD estimates; and provides both national and subnational avenues for the translation and use of results for decision making.^65^, ^66^, ^67^, ^68^, ^69^ An increasing number of in-depth country engagements with the GBD are also producing subnational assessments of disease burden and maximising policy relevance. For instance, spearheaded by the Indian Council for Medical Research and the Public Health Foundation of India, GBD is presently undertaking state-level disease burden assessments in India, disaggregated by urban and rural areas as part of GBD 2016. This work has been characterised by intensive engagement with the Indian Government and the Indian scientific community in the production of estimates.

Recent changes to the global SDG monitoring framework, as well as the proposal of processes to consider indicator revisions annually and potential additions in 2020 and 2025,^5^ now establish the SDGs as a dynamic development agenda. This is unlike previous international goal-setting efforts, such as the MDGs.^21^ Subsequently, timely efforts to track newly added and revised indicators are central to the ability of the SDG agenda to evolve over time. By the nature of its annual reporting cycle, the extensive range of health indicators currently or potentially measured, and the location of work within academic and scientific organisations, the GBD study is well positioned to quickly respond to indicator revisions and expansion. This is highlighted by the incorporation of March, 2017, indicator refinements into GBD 2016 (eg, vaccine coverage [SDG indicator 3.b.1]), and our ability to report on important violence indicators that no other international agency currently includes. Of the new health-related SDG indicators proposed for consideration by the IAEG-SDGs, most could be incorporated into the GBD measurement cycle with relative ease; these indicators include psychoactive substance abuse, incidence of road traffic injuries, incidence of unintentional poisonings, prevalence of anaemia among women of reproductive age, and illnesses attributable to risk factors (air pollution and unsafe water, sanitation, and hygiene). Other indicators that cover, for example, other dimensions of mental health and NCDs have also been proposed. Continued improvements in indicator measurement are also facilitated through novel extensions and developments from the broader GBD study and related work, which is exemplified by incorporating the HAQ Index into our UHC measure for GBD 2016. Another priority area for GBD expansion is the increased ability to track key health indicators, not only at subnational administrative levels, but also as a continuous geospatial surface. Research on mapping inequalities in child mortality at a 5 km × 5 km resolution in Africa has leveraged the GBD study,^70^ providing an example of the increasing ability to track SDG indicator attainment at levels beyond national averages.

### Limitations

A number of specific limitations for SDG indicators exist, described in the underlying GBD papers as well as in appendix 1. Of note, for sanitation (a key determinant of health) we have used a proxy indicator based on measuring the fraction of the populations that have unimproved sanitation, improved sanitation without a sewer connection, and improved sanitation with a sewer connection. This does not take into account whether waste is safely managed or treated. For the vaccine coverage indicator, we used a proxy indicator based on the geometric mean of vaccine coverage of individual vaccines and did not explicitly account for the correlation structure that exists between individual vaccines.

As noted in more detail above, we have used a relatively simple approach for projecting SDG indicator values. Limitations also exist in terms of the construction of the health-related SDG index. Ideally, we would develop an index that scales indicator values to SDG target values. We have not implemented this in GBD 2016 for several reasons. First, 13 of the health-related SDG indicators do not presently specify a target. Second, a subset of SDG targets are relatively modest for many middle-SDI to high-SDI countries (eg, reducing MMR to <70 deaths per 100 000 livebirths), and the effect of rescaling to these targets is that any differences beyond the target are ignored. In constructing the health-related SDG index, we used the geometric rather than arithmetic mean. The geometric mean allows for partial substitutability (ie, poor performance on one indicator is only partially offset by good performance on another), while the arithmetic mean allows for complete substitutability (ie, poor performance on one indicator can be completely offset by good performance on another indicator). As a result, stagnating progress on some indicators—most notably indicators such as childhood overweight and harmful alcohol use—can have a notable effect on progress and our projections based on past trends of the overall health-related SDG index. As noted in appendix 1, constructing the health-related SDG index using the arithmetic mean suggests somewhat more optimistic but qualitatively similar progress on the health-related SDGs as measured by the index, with no country showing a decline. As part of future iterations of the GBD we will continue to test and refine alternative index construction approaches that can take into account SDG targets.

Our assessment reflects ongoing gaps in data availability and coverage across countries for some indicators and remains a major limitation to any SDG monitoring effort, including GBD. For example, data for the violence indicators are sparse, particularly for non-intimate partner violence, men as victims of sexual violence, and psychological violence. Measurement issues, such as the variability and accuracy of self-report of different types of violence across settings, pose additional challenges. Limitations also exist for the water, sanitation, and hygiene indicators, particularly in view of the relative absence of data to estimate safe sanitation management. A benefit of the GBD study is that it can help identify these data gaps, both over time and by location, and provide an interim solution to data gaps through the use of standardised estimation approaches. Nevertheless, the GBD is not, and should not be, a replacement for investing in high-quality, routine health information systems that are crucial for measuring and evaluating SDG progress at national and subnational levels. Last, any limitations of GBD 2016 relevant to the 37 currently measured health-related SDG indicators apply.^28^, ^29^, ^30^, ^31^, ^32^

### Conclusions

Understanding where countries are, and where they are likely to go on the basis of past trends, is essential to guide strategic and investment decisions to achieve the SDG agenda by 2030. With this updated GBD analysis of the SDGs, we measure 37 of the 50 health-related SDG indicators from 1990 to 2016, and provide projections of SDG attainment by 2030 on the basis of past trends. For a subset of indicators, such as under-5 mortality and neonatal mortality, MMR, and malaria, projected levels of SDG achievement are promising, particularly among higher-SDI countries. However, these more positive projections for SDG attainment appear to be the exception, with most countries, especially countries in western and central sub-Saharan Africa and low-SDI countries, facing a challenging road toward SDG achievement by 2030 on the basis of current trajectories. It is increasingly clear that the health-related SDG agenda hinges upon markedly accelerating progress, particularly among the world's poorest populations. Succeeding in this endeavour is not yet an impossibility—nonetheless, it will demand extraordinary financial and political commitment by national and international agencies alike to ensure that truly no one is left behind in 2030.
