# Department of Error

**DOI:** 10.1016/S0140-6736(17)32441-8

**Published:** 2017-09-30

*GBD 2016 SDG Collaborators. Measuring progress and projecting attainment on the basis of past trends of the health-related Sustainable Development Goals in 188 countries: an analysis from the Global Burden of Disease Study 2016.* Lancet *2017; **390**: 1423–59*—In figure 8B of this Article (published Online First on Sept 12, 2017), the number of indicator targets has been changed from 1 to 9 for Turkmenistan, from 0 to 1 for Afghanistan, and from 1 to 2 for Yemen. Ettore Beghi, Neeraj Bhala, Hélène Carabin, Raimundas Lunevicius, Donald H Silberberg, and Caitlyn Steiner have been added to the list of GBD 2016 SDG Collaborators. Their affiliations, along with the affiliation of Soumya Swaminathan, have been added to the Affiliations section. These corrections have been made to the online version as of Sept 18, 2017, and the printed Article is correct.

