# Department of Error

**DOI:** 10.1016/S0140-6736(17)32650-8

**Published:** 2017-10-28

*GBD 2016 SDG Collaborators. Measuring progress and projecting attainment on the basis of past trends of the health-related Sustainable Development Goals in 188 countries: an analysis from the Global Burden of Disease Study 2016.* Lancet *2017; **390**: 1423–59*—The full-text version of this Article has been updated so that the list of authors is displayed in the correct order, in line with the pdf version, rather than in alphabetical order. This correction has been made to the online version as of Oct 12, 2017.

