# Supplementary material for: Measuring progress and projecting attainment on the basis of past trends of the health-related Sustainable Development Goals in 188 countries: an analysis from the Global Burden of Disease Study 2016
Source: Lancet. 2017 Sep 16;390(10100):1423–59. doi: 10.1016/S0140-6736(17)32336-X (PMC5603800; doi:10.1016/S0140-6736(17)32336-X)

THE LANCET

Supplementary appendix

This appendix formed part of the original submission and has been peer reviewed. We post it as supplied by the authors.

Supplement to: GBD 2016 SDG Collaborators. Measuring progress and projecting attainment on the basis of past trends of the health-related Sustainable Development Goals in 188 countries: an analysis from the Global Burden of Disease Study 2016. *Lancet* 2017; published online Sept 12. http://dx.doi.org/10.1016/S0140-6736(17)32336-X.

Methods appendix to Measuring progress and projecting attainment based on past trends of the health-related Sustainable Development Goals in 188 countries: an analysis from the Global Burden of Disease Study 2016

This appendix provides further methodological detail, supplemental figures, and more detailed results for the health-related Sustainable Development Goals. The appendix is organized into broad sections following the structure of the main paper.

Table of Contents

Author contributions....................i

List of Methods Appendix Figures and Tables....................1

List of Supplementary Results Figures and Tables....................2

Preamble....................3

GATHER Statement....................4

Part 1. Health-related SDG indicators....................7

Section 1. SDGs overview

Section 2. Health-related SDGs

Section 3. SDG indicator write-ups

Part 2. SDG index construction and sensitivity analyses....................323

Part 3. Projections for the health-related SDGs....................324

Section 1. Overall projection modeling strategy

Section 2. Projecting HIV and ART coverage for the health-related SDGs

Part 4. Online tools and glossary of terms....................335

Section 1. Online tools

Section 2. List of abbreviations

Authors' Contributions

Managing the estimation process

Ryan M Barber, Jonathan Brown, Austin Carter, Tahvi Frank, Kyle Foreman, Nancy Fullman, Jun Kim, Stephen S Lim, Shawn Minnig, Christopher JL Murray, Emma Nichols, Anna Torre, and Tianchan Tao.

Writing the first draft of the manuscript

Nancy Fullman, Christopher J L Murray, and Stephen S Lim.

Providing data or critical feedback on data sources

Cristiana Abbafati, Foad Abd-Allah, Semaw Ferede Abera, Habtamu Abera Hareri, Laith J Abu-Raddad, Niveen Abu-Rmeileh, Isaac Adedeji, Olatunji Adetokunboh, Aliasghar Ahmad Kiadaliri, Muktar Ahmed, Amani N Aichour, Ibtihel Aichour, Miloud TE Aichour, Rufus Akinyemi, Nadia Akseer, Deena Al Asfoor, Khurshid Alam, Noore Alam, Kefyalew Addis Alene, Ala'a Alkerwi, François Alla, Peter Allebeck, Ubai Alsharif, Khalid Altirkawi, Dayane Gabriele Alves Silveira, Azmeraw T Amare, Erfan Amini, Walid Ammar, Hossein Ansari, Palwasha Anwari, Paul Argan, Al Artaman, Krishna Kumar Aryal, Hamid Asayesh, Solomon W Asgedom, Reza Assadi, Ashish Awasthi, Umar Bacha, Kalpana Balakrishnan, Aleksandra Barac, Till Bärnighausen, Lope Barrero, Sanjay Basu, Katherine Battle, Bernhard Baune, Justin Beardsley, Neeraj Bedi, Ettore Beghi, Yannick Béjot, Derrick Bennett, Isabela Bensenor, Eduardo Bernabe, Addisu Shunu Beyene, Anil Bhansali, Boris Bikbov, Charles Birungi, Dipan Bose, Ibrahim Bou-Orm, Michael Brauer, Traolach Brugha, Julio Cesar Campuzano, Juan Jesus Carrero, Carlos Castañeda-Orjuela, Ferrán Catalá-López, Hsing-Yi Chang, Jung-Chen Chang, Fiona Charlson, Abdulaal Chitheer, Massimo Cirillo, Michael Criqui, Elizabeth A Cromwell, John Crump, Lalit Dandona, Rakhi Dandona, José das Neves, Louisa Degenhardt, Kebede Deribe, Devasahayam Jesudas Christopher, Gabrielle deVeber, Samath Dharmaratne, Balem Dimtsu, Klara Dokova, E Ray Dorsey, Tim Driscoll, Manisha Dubey, Beth Ebel, Maysaa El Sayed Zaki, Sergey Ermakov, Sharareh Eskandarieh, Alireza Esteghamati, Emerito Jose A Faraon, Carla Farinha, Andre Faro, Farshad Farzadfar, Valery Feigin, Seyed-Mohammad Fereshtehnejad, Joao Fernandes, Irina Filip, Florian Fischer, Richard C Franklin, Joseph Frostad, Nancy Fullman, Thomas Fürst, Joao M Furtado, Emmanuela Gakidou, Johanna M Geleijnse, Ayele Geleto, Sefonias Getachew, Peter Gething, Alireza Ghajar, Katherine Gibney, Melkamu Dedefo Gishu, Philimon Gona, Harish Gugnani, Tanush Gupta, Nima Hafezi-Nejad, Gessessew Hailu, Hilda Harb, Mohammad Sadegh Hassanvand, Hamid Yimam Hassen, Rasmus Havmoeller, Simon Hay, Delia Hendrie, Nathaniel Henry, Ileana B Heredia-Pi, Hans Hoek, Mollie Holmberg, Nobuyuki Horita, H Dean Hosgood, Damian Hoy, Mohamed Hsairi, Aung Soe Htet, Kim Iburg, Manami Inoue, Sheikh Mohammed Shariful Islam, Kathryn H Jacobsen, Nader Jahanmehr, Mihajlo Jakovljevic, Dube Jara, Alejandra Jauregui, Mehdi Javanbakht, Panniyammakal Jeemon, Vivekanand Jha, Catherine O Johnson, Jost B Jonas, Mikk Jürisson, Zubair Kabir, Rajendra Kadel, Ritul Kamal, Chittaranjan Kar, André Karch, Corine Karema, Amir Kasaeian, Nicholas Kassebaum, Srinivasa Vittal Katikireddi, Norito Kawakami, Chandrasekharan Nair Kesavachandran, Yousef Khader, Ibrahim Khalil, Ardeshir Khosravi, Jagdish Khubchandani, Christian Kieling, Yun Jin Kim, Mika Kivimaki, Yoshihiro Kokubo, Soewarta Kosen, Parvaiz Koul, Michael Kravchenko, Kristopher J Krohn, Barthelemy Kuate Defo, Burcu Kucuk Bicer, G Anil Kumar, Basant Kumar Panda, Michael J Kutz, Hmwe H Kyu, Dharmesh Kumar Lal, Van C Lansingh, Quyen Le Nguyen, Misgan Legesse Liben, James Leigh, Bikila Lencha, Ricky Leung, Xiaofeng Liang, Stephen S Lim, Shiwei Liu, Katharine Looker, Alan D Lopez, Stefan Lorkowski, Paulo Lotufo, Rafael Lozano, Marek Majdan, Azeem Majeed, Reza Malekzadeh, Abdullah Mamun, Lorenzo Mantovani, Tsegahun Manyazewal, Guy Marks, Randall Martin, Jose Martinez-Raga,

Manu Mathur, Mohsen Mazidi, Colm McAlinden, John J McGrath, Suresh Mehata, Man Mohan Mehndiratta, Kidanu Meles, Ziad Memish, George A Menash, Walter Mendoza, Melkamu Merid Mengesha, Gert Mensink, Atte Meretoja, Tuomo Meretoja, Renata Micha, Ted Miller, Shiva Raj Mishra, Shafiu Mohammed, Ali H Mokdad, Lorenzo Monasta, Julio Montañez, Maziar Moradi-Lakeh, Ulrich Mueller, GVS Murthy, Kamarul Imran Musa, Mohsen Naghavi, Kovin Naidoo, Lemma Negesa, Minh Nguyen, Marika Nomura, Carla Makhlouf Obermeyer, Felix Ogbo, Olanrewaju Oladimeji, Andrew T Olagunju, Bolajoko Olusanya, Jacob Olusanya, Alberto Ortiz, Mayowa Owolabi, Mahesh PA, Songhomitra Panda-Jonas, Christina Papachristou, George Patton, Norberto Perico, Max Petzold, Huyen Phuc Do, David M Pigott, Farshad Pourmalek, Mostafa Qorbani, Amir Radfar, Anwar Rafay, Vafa Rahimi-Movaghar, Rajesh Kumar Rai, Sasa Rajsic, Usha Ram, Salman Rawaf, Robert C Reiner, Marissa Reitsma, Giuseppe Remuzzi, Andre Renzaho, Luz Myriam Reynales-Shigematsu, David Rojas-Rueda, Mohammad B Rokni, Gholamreza Roshandel, Enrico Rubagotti, Nafis Sadat, Saeid Safiri, Rajesh Sagar, Joshua A Salomon, Abdallah M Samy, Juan Sanabria, Itamar Santos, Milena Santric Milicevic, Maheswar Satpathy, Monika Sawhney, Sonia Saxena, David C Schwebel, Falk Schwendicke, Soraya Seedat, Sadaf Sepanlou, Edson Serván-Mori, Amira Shaheen, Masood Ali Shaikh, Mansour Shamsipour, Morteza Shamsizadeh, Jayendra Sharma, Peilin Shi, Girma Temam Shifa, Girma Temam Shifa, Reza Shirkoohi, Kawkab Shishani, Haitham Shoman, Mark Shrime, Diego Augusto Santos Silva, Jasvinder Singh, Virendra Singh, Eirini Skiadaresi, Erica Leigh Slepak, Eugene Sobngwi, Michael Soljak, Luciano Sposato, Chandrashekhar T Sreeramareddy, Jeffrey D Stanaway, Mark Stokes, Mu'awiyyah Babale Sufiyan, Rizwan Suliankatchi Abdulkader, Patrick Sur, Bryan Sykes, Cassandra Szoeke, Rafael Tabarés-Seisdedos, Cuong Tat Nguyen, Mohammad Tavakkoli, Nuno Taveira, Abdullah Terkawi, Gizachew Tessema, Ruoyan Tobe-Gai, Thomas Truelsen, Kingsley N Ukwaja, Chigozie Uneke, Olalekan A Uthman, Job FM van Boven, Aaron van Donkelaar, Tommi Vasankari, Narayanaswamy Venketasubramanian, Ramesh Vidavalur, Francesco S Violante, Vasiliy Vlassov, Stein Emil Vollset, Theo Vos, Fiseha Wadilo, Tolassa Wakayo, Yuan-Pang Wang, Elisabete Weiderpass, Robert Weintraub, Daniel Weiss, Ronny Westerman, Tissa Wijeratne, Charles Shey Wiysonge, Charles Wolfe, Rachel Woodbrook, Denis Xavier, Gelin Xu, Bach Xuan Tran, Bereket Yakob, Lijing Yan, Pengpeng Ye, Naohiro Yonemoto, Mustafa Younis, Zoubida Zaidi, Luis Zavala-Arciniega, and Xueying Zhang.

Developing methods or computational machinery

Olatunji Adetokunboh, Muktar Ahmed, Christine Allen, Megha Arora, Reza Assadi, Ryan M Barber, Adugnaw Berhane, Stan Biryukov, Michael Brauer, Austin Carter, Daniel C Casey, Kelly Cercy, Elizabeth A Cromwell, Devasahayam Jesudas Christopher, Abraham D Flaxman, Kyle J Foreman, Tahvi Frank, Joseph Frostad, Nancy Fullman, Emmanuela Gakidou, Ayele Geleto, Peter Gething, Hamid Yimam Hassen, Nathaniel Henry, Mollie Holmberg, Sarah C Johnson, Nicholas Kassebaum, Jun Kim, Michael J Kutz, Stephen S Lim, Patrick Y Liu, Rafael Lozano, Reza Malekzadeh, Madeline McGaughey, George A Menash, Ali H Mokdad, Mohsen Naghavi, Lemma Negesa, Grant Nguyen, Carla Makhlouf Obermeyer, David M Pigott, Robert C Reiner, Marissa B Reitsma, Gregory A Roth, Nafis Sadat, Joshua A Salomon, Maheswar Satpathy, David Smith, Vinay Srinivasan, Jeffrey D Stanaway, Patrick Sur, Rachel Updike, Theo Vos, and Gelin Xu.

Applying analytical methods to produce estimates

Muktar Ahmed, Christine Allen, Azmeraw T Amare, Megha Arora, Solomon W Asgedom, Marlena Bannick, Aleksandra Barac, Ryan M Barber, James Bennett, Adugnaw Berhane, Charles Birungi, Stan Biryukov, Austin Carter, Daniel C Casey, Kelly Cercy, Cyrus Cooper, Elizabeth A Cromwell, Devasahayam

Jesudas Christopher, Aman Endries, Mir Sohail Fazeli, Christina Fitzmaurice, Abraham D Flaxman, Tahvi Frank, Joseph Friedman, Joseph Frostad, Nancy Fullman, Emmanuela Gakidou, Ayele Geleto, William W Godwin, Ellen M Goldberg, Max Griswold, Hamid Yimam Hassen, Jiawei He, Nathaniel Henry, Chantal Huynh, Caleb Irvine, Dube Jara, Catherine O Johnson, Nicholas Kassebaum, Jun Kim, Xie Rachel Kulikoff, Hmwe H Kyu, Van C Lansingh, Stephen S Lim, Patrick Y Liu, Rafael Lozano, Emilie Maddison, Helena Manguerra, Ira Martopullo, Mohsen Mazidi, Madeline McGaughey, George A Menash, Anoushka Millear, Ali H Mokdad, Sarah Mollenkopf, Mark W Moses, Cliff Mountjoy-Venning, Mohsen Naghavi, Lemma Negesa, Grant Nguyen, Minh Nguyen, Emma Nichols, Carla Makhlouf Obermeyer, Bolajoko Olusanya, Jacob Olusanya, Kanyin Ong, Katherine Paulson, David M Pigott, Christine Pinho, Mostafa Qorbani, Puja C Rao, Robert C Reiner, Nikolas Reinig, Marissa B Reitsma, Gregory A Roth, Enrico Rubagotti, Nafis Sadat, Joshua A Salomon, Maheswar Satpathy, Matthew T Schneider, Masood Ali Shaikh, Amber Sligar, Alison Smith, Vinay Srinivasan, Jeffrey D Stanaway, Bryan Strub, Patrick Sur, Dillon O Sylte, Tianchan Tao, Christopher Troeger, Derrick Tsoi, Rachel Updike, Theo Vos, Tolassa Wakayo, Ronny Westerman, Tissa Wijeratne, Gelin Xu, and Simon Yadgir.

Providing critical feedback on methods or results

Amanuel Abajobir, Cristiana Abbafati, Kaja Abbas, Foad Abd-Allah, Abdishakur Abdulle, Mubarek Abera, Semaw Ferede Abera, Habtamu Abera Hareri, Victor Aboyans, Laith J Abu-Raddad, Gebre Abyu, Isaac Adedeji, Olatunji Adetokunboh, Mohsen Afarideh, Anurag Agrawal, Aliasghar Ahmad Kiadaliri, Hamid Ahmadieh, Muktar Ahmed, Amani N Aichour, Ibtihel Aichour, Miloud TE Aichour, Rufus Akinyemi, Nadia Akseer, Deena Al Asfoor, Ziyad Al-Aly, Khurshid Alam, Noore Alam, Kefyalew Addis Alene, Reza Alizadeh-Navaei, Ala'a Alkerwi, Peter Allebeck, Rajaa Al-Raddadi, Ubai Alsharif, Khalid Altirkawi, Dayane Gabriele Alves Silveira, Nelson Alvis-Guzman, Azmeraw T Amare, Erfan Amini, Walid Ammar, Hossein Ansari, Carl Abelardo T Antonio, Palwasha Anwari, Paul Argan, Al Artaman, Krishna Kumar Aryal, Hamid Asayesh, Solomon W Asgedom, Tesfay Mehari Atey, Sachin Atre, Leticia Avila-Burgos, Euripide Avokpaho, Ashish Awasthi, Peter Azzopardi, Umar Bacha, Alaa Badawi, Aleksandra Barac, Ryan M Barber, Suzanne Barker-Collo, Till Bärnighausen, Lope Barrero, Sanjay Basu, Bernhard Baune, Justin Beardsley, Neeraj Bedi, Ettore Beghi, Michelle Bell, Derrick Bennett, Isabela Bensenor, Adugnaw Berhane, Derbew Fikadu Berhe, Eduardo Bernabe, Kald Beshir Tuem, Mircea Beuran, Addisu Shunu Beyene, Zulfiqar Bhutta, Boris Bikbov, Arebu Bilal, Charles Birungi, Stan Biryukov, Habtamu Mellie Bizuayehu, Christopher Blosser, Nicholas Breitborde, Traolach Brugha, Zahid Butt, Lucero Cahuana-Hurtado, Rosario Cárdenas, Austin Carter, Carlos Castañeda-Orjuela, Ruben Castro, Ferrán Catalá-López, Kelly Cercy, Jung-Chen Chang, Fiona Charlson, Vesper Chisumpa, Cyrus Cooper, Michael Criqui, Elizabeth A Cromwell, Lalit Dandona, Rakhi Dandona, José das Neves, Julian David, Barbora de Courten, Hans De Steur, Louisa Degenhardt, Devasahayam Jesudas Christopher, Gabrielle deVeber, Samath Dharmaratne, Balem Dimtsu, Klara Dokova, David T Doku, Manisha Dubey, Bruce Duncan, Beth Ebel, Maysaa El Sayed Zaki, Ziad El-Khatib, Ahmadali Enayati, Aman Endries, Sergey Ermakov, Babak Eshrati, Sharareh Eskandarieh, Alireza Esteghamati, Emerito Jose A Faraon, Carla Farinha, Andre Faro, Farshad Farzadfar, Mir Sohail Fazeli, Seyed-Mohammad Fereshtehnejad, Joao Fernandes, Tesfaye R Feyissa, Irina Filip, Florian Fischer, Nataliya Foigt, Kyle J Foreman, Richard C Franklin, Joseph Friedman, Nancy Fullman, Thomas Fürst, Joao M Furtado, Emmanuela Gakidou, Morsaleh Ganji, Tsegaye Gebrehiwot, Ayele Geleto, Sefonias Getachew, Belete Getahun, Katherine Gibney, Paramjit Gill, Ababi Giref, Melkamu Dedefo Gishu, Giorgia Giussani, Ellen M Goldberg, Philimon N Gona, Amador Goodridge, Sameer Gopalani, Yevgeniy Goryakin, Max Griswold, Harish Gugnani, Rajeev Gupta, Tanush Gupta, Vipin Gupta, Nima Hafezi-Nejad, Hassan Haghparast-Bidgoli, Gessessew Hailu, Randah Hamadeh, Mouhanad Hammami, Graeme Hankey, Hilda

Harb, Hamid Yimam Hassen, Rasmus Havmoeller, Simon I Hay, Jiawei He, Delia Hendrie, Ileana B Heredia-Pi, Nobuyuki Horita, H Dean Hosgood, Sorin Hostiuc, Damian Hoy, Hsiang Huang, John Huang, Trang Huyen Nguyen, Chantal Huynh, Kim Iburg, Manami Inoue, Sheikh Mohammed Shariful Islam, Kathryn H Jacobsen, Nader Jahanmehr, Mihajlo Jakovljevic, Dube Jara, Alejandra Jauregui, Mehdi Javanbakht, Panniyammakal Jeemon, Vivekanand Jha, Denny John, Catherine O Johnson, Jost B Jonas, Mikk Jürisson, Zubair Kabir, Rajendra Kadel, Amaha Kahsay, Ritul Kamal, André Karch, Amir Kasaeian, Nicholas Kassebaum, Anshul Kastor, Srinivasa Vittal Katikireddi, Norito Kawakami, Peter Keiyoro, Andre Pascal Kengne, Chandrasekharan Nair Kesavachandran, Yousef Khader, Ibrahim Khalil, Young-Ho Khang, Ardeshir Khosravi, Jagdish Khubchandani, Christian Kieling, Daniel Kim, Jun Kim, Yun Jin Kim, Ruth Kimokoti, Yohannes Kinfu, Adnan Kisa, Katarzyna Kissimova-Skarbek, Mika Kivimaki, Jacek Kopec, Parvaiz Koul, Ai Koyanagi, Kristopher J Krohn, Barthelemy Kuate Defo, Burcu Kucuk Bicer, Xie Rachel Kulikoff, G Anil Kumar, Basant Kumar Panda, Yihunie Lakew, Dharmesh Kumar Lal, Ratilal Lalloo, Van C Lansingh, Anders Larsson, Jeffrey Lazarus, Quyen Le Nguyen, Paul Lee, Misgan Legesse Liben, James Leigh, Bikila Lencha, Miriam Levi, Yongmei Li, Stephen Lim, Shai Linn, Rakesh Lodha, Katharine Looker, Alan Lopez, Stefan Lorkowski, Paulo Lotufo, Rafael Lozano, Erlyn Rachelle Macarayan, Mark Mackay, Hassan Magdy Abd El Razek, Mohammed Magdy Abd El Razek, Marek Majdan, Reza Majdzadeh, Azeem Majeed, Reza Malekzadeh, Rajesh Malhotra, Deborah C Malta, Abdullah Mamun, Lorenzo Mantovani, Tsegahun Manyazewal, Chabila Mapoma, Guy Marks, Jose Martinez-Raga, Francisco Rogerlândio Martins-Melo, Ira Martopullo, Manu Mathur, Mohsen Mazidi, Colm McAlinden, John McGrath, Martin McKee, Suresh Mehata, Toni Meier, Kidanu Meles, George A Menash, Walter Mendoza, Melkamu Merid Mengesha, Gert Mensink, Seid Tiku Mereta, Atte Meretoja, Tuomo Meretoja, Haftay Berhane Mezgebe, Renata Micha, Ted Miller, Vuong Minh Nong, Mojde Mirarefin, Erkin Mirrakhimov, Shiva Raj Mishra, Philip B Mitchell, Kedir Mohammed, Shafiu Mohammed, Murali Mohan, Ali H Mokdad, Lorenzo Monasta, Marcella Montico, Maziar Moradi-Lakeh, Lidia Morawska, Mark W Moses, Ulrich Mueller, Kate Muller, GVS Murthy, Kamarul Imran Musa, Mohsen Naghavi, Aliya Naheed, Kovin Naidoo, Vinay Nangia, Gopalakrishnan Natarajan, Lemma Negesa, Ionut Negoi, Ruxandra Irina Negoi, Grant Nguyen, Jean Jacques Noubiap, Dina Nur Anggraini Ningrum, Carla Makhlouf Obermeyer, Felix Ogbo, Olanrewaju Oladimeji, Andrew T Olagunju, Tinuke Olagunju, Bolajoko Olusanya, Jacob Olusanya, Kanyin Ong, Eyal Oren, Alberto Ortiz, Mayowa Owolabi, Mahesh PA, Adrian Pana, Songhomitra Panda-Jonas, Christina Papachristou, Eun-Kee Park, George Patton, Katherine Paulson, Norberto Perico, Konrad Pesudovs, Michael R Phillips, Huyen Phuc Do, David M Pigott, Christine Pinho, Richie Poulton, Farshad Pourmalek, Mostafa Qorbani, Amir Radfar, Vafa Rahimi-Movaghar, Mahfuzar Rahman, Mohammad Hifz Ur Rahman, Muhammad Aziz Rahman, Rajesh Kumar Rai, Usha Ram, Chhabi Lal Ranabhat, Puja C Rao, Salman Rawaf, Robert C Reiner, Nikolas Reinig, Marissa B Reitsma, Giuseppe Remuzzi, Andre Renzaho, Serge Resnikoff, Satar Rezaei, Kedir Teji Roba, Gholamreza Roshandel, Ambuj Roy, Sare Safi, Saeid Safiri, Rajesh Sagar, Joshua A Salomon, Abdallah M Samy, Juan Sanabria, Itamar Santos, Milena Santric Milicevic, Benn Sartorius, Maheswar Satpathy, Monika Sawhney, Sonia Saxena, Mete Saylan, Maria Inês Schmidt, Ione Schneider, Matthew T Schneider, Ben Schöttker, Aletta E Schutte, David C Schwebel, Falk Schwendicke, Soraya Seedat, Sadaf Sepanlou, Amira Shaheen, Saeid Shahraz, Masood Ali Shaikh, Morteza Shamsizadeh, Jayendra Sharma, Rajesh Sharma, Kenji Shibuya, Chloe Shields, Girma Temam Shifa, Girma Temam Shifa, Mika Shigematsu, Min-Jeong Shin, Rahman Shiri, Reza Shirkoohi, Haitham Shoman, Mark Shrime, Diego Augusto Santos Silva, João Pedro Silva, Jasvinder Singh, Mekonnen Sisay, Eirini Skiadaresi, Amber Sligar, Mari Smith, Badr Sobaih, Eugene Sobngwi, Michael Soljak, Samir Soneji, Reed Sorensen, Luciano Sposato, Chandrashekhar T Sreeramareddy, Jeffrey D Stanaway, Sabine Steinke, Bryan Strub,

Mu'awiyyah Babale Sufiyan, Rizwan Suliankatchi Abdulkader, Bruno Sunguya, Patrick Sur, Bryan Sykes, Dillon O Sylte, Cassandra Szoeke, Rafael Tabarés-Seisdedos, Nikhil Tandon, Cuong Tat Nguyen, Nuno Taveira, Teketo Tegegne, Abdullah Terkawi, Belay Tessema, JS Thakur, Amanda Thrift, Tenaw Yimer Tiruye, Ruoyan Tobe-Gai, Roman Topor-Madry, Miguel Tortajada-Girbés, Thomas Truelsen, Derrick Tsoi, E Murat Tuzcu, Kingsley N Ukwaja, Chigozie Uneke, Olalekan A Uthman, Job FM van Boven, Santosh Varughese, Tommi Vasankari, Narayanaswamy Venketasubramanian, Ramesh Vidavalur, Francesco S Violante, Sergey Vladimirov, Vasiliy Vlassov, Stein Emil Vollset, Theo Vos, Fiseha Wadilo, Tolassa Wakayo, Mitchell Waliln, Scott Weichenthal, Elisabete Weiderpass, Robert Weintraub, Andrea Werdecker, Ronny Westerman, Tissa Wijeratne, Charles Shey Wiysonge, Denis Xavier, Gelin Xu, Bach Xuan Tran, Bereket Yakob, Lijing Yan, Yuichiro Yano, Mehdi Yaseri, Paul SF Yip, Naohiro Yonemoto, Marcel Yotebieng, Mustafa Younis, Zoubida Zaidi, and Sanjay Zodpey.

Drafting the work or revising is critically for important intellectual content

Kalkidan Hassen Abate, Cristiana Abbafati, Foad Abd-Allah, Abdishakur Abdulle, Mubarek Abera, Semaw Ferede Abera, Gebre Abyu, Isaac Adedeji, Olatunji Adetokunboh, Mohsen Afarideh, Sutapa Agrawal, Aliasghar Ahmad Kiadaliri, Muktar Ahmed, Rufus Akinyemi, Nadia Akseer, Khurshid Alam, Noore Alam, Ala'a Alkerwi, Peter Allebeck, Nelson Alvis-Guzman, Azmeraw T Amare, Erfan Amini, Walid Ammar, Hossein Ansari, Carl Abelardo Antonio, Palwasha Anwari, Hamid Asayesh, Solomon W Asgedom, Tesfay Mehari Atey, Leticia Avila-Burgos, Euripide Avokpaho, Ashish Awasthi, Peter Azzopardi, Alaa Badawi, Ryan M Barber, Suzanne Barker-Collo, Till Bärnighausen, Lope Barrero, Sanjay Basu, Bernhard Baune, Neeraj Bedi, Michelle Bell, Isabela Bensenor, Adugnaw Berhane, Eduardo Bernabe, Kald Beshir Tuem, Mircea Beuran, Addisu Shunu Beyene, Charles Birungi, Christopher Blosser, Michael Brauer, Nicholas Breitborde, Traolach Brugha, Zahid Butt, Lucero Cahuana-Hurtado, Juan Jesus Carrero, Carlos Castañeda-Orjuela, Jacqueline Castillo-Rivas, Ruben Castro, Ferrán Catalá-López, Fiona Charlson, Adrienne Chew, Hanne Christensen, Massimo Cirillo, Cyrus Cooper, Michael Criqui, Elizabeth A Cromwell, John Crump, José das Neves, Dragos Davitoiu, Barbora de Courten, Louisa Degenhardt, Selina Deiparine, Devasahayam Jesudas Christopher, Samath Dharmaratne, Balem Dimtsu, Eric Ding, E Ray Dorsey, Tim Driscoll, Manisha Dubey, Bruce Duncan, Hedyeh Ebrahimi, Maysaa El Sayed Zaki, Ziad El-Khatib, Aman Endries, Holly Erskine, Sharareh Eskandarieh, Alireza Esteghamati, Emerito Jose A Faraon, Carla Farinha, Andre Faro, Mir Sohail Fazeli, Andrea Feigl, Seyed-Mohammad Fereshtehnejad, Joao Fernandes, Alize Ferrari, Irina Filip, Florian Fischer, Nataliya Foigt, Kyle J Foreman, Joseph Friedman, Nancy Fullman, Thomas Fürst, Joao M Furtado, Emmanuela Gakidou, Morsaleh Ganji, Alberto L Garcia-Basteiro, Tsegaye Gebrehiwot, Johanna M Geleijnse, Ayele Geleto, Alireza Ghajar, Katherine Gibney, Paramjit Gill, Richard Gillum, Melkamu Dedefo Gishu, Ellen M Goldberg, Philimon N Gona, Amador Goodridge, Sameer Gopalani, Max Griswold, Rajeev Gupta, Tanush Gupta, Nima Hafezi-Nejad, Gessessew Hailu, Randah Hamadeh, Hilda Harb, Hamid Yimam Hassen, Rasmus Havmoeller, Caitlin Hawley, Jiawei He, Ileana B Heredia-Pi, Nobuyuki Horita, H Dean Hosgood, Sorin Hostiuc, Damian Hoy, Hsiang Huang, Trang Huyen Nguyen, Chantal Huynh, Sheikh Mohammed Shariful Islam, Kathryn H Jacobsen, Mihajlo Jakovljevic, Dube Jara, Panniyammakal Jeemon, Vivekanand Jha, Denny John, Jost B Jonas, Mikk Jürisson, Rajendra Kadel, Ritul Kamal, André Karch, Amir Kasaeian, Nicholas Kassebaum, Srinivasa Vittal Katikireddi, Norito Kawakami, Peter Keiyoro, Andre Pascal Kengne, Chandrasekharan Nair Kesavachandran, Yousef Khader, Ejaz Khan, Young-Ho Khang, Jagdish Khubchandani, Christian Kieling, Daniel Kim, Yun Jin Kim, Mika Kivimaki, Yoshihiro Kokubo, Jacek Kopec, Parvaiz Koul, Ai Koyanagi, Kristopher J Krohn, Barthelemy Kuate Defo, Xie Rachel Kulikoff, Santhosh Kumar, Dharmesh Kumar Lal, Anders Larsson, Quyen Le Nguyen, Misgan Legesse Liben, Janni Leung, Miriam Levi, Stephen S Lim, Alan

D Lopez, Rafael Lozano, Hassan Magdy Abd El Razek, Mohammed Magdy Abd El Razek, Marek Majdan, Reza Majdzadeh, Azeem Majeed, Reza Malekzadeh, Abdullah Mamun, Tsegahun Manyazewal, Jose Martinez-Raga, Francisco Rogerlândio Martins-Melo, Ira Martopullo, Mohsen Mazidi, Colm McAlinden, Toni Meier, Kidanu Meles, Ziad Memish, George A Menash, Walter Mendoza, Melkamu Merid Mengesha, Atte Meretoja, Tuomo Meretoja, Haftay Berhane Mezgebe, Ted Miller, Vuong Minh Nong, Shiva Raj Mishra, Karzan Mohammad, Shafiu Mohammed, Ali H Mokdad, Lorenzo Monasta, Maziar Moradi-Lakeh, Paula Moraga, Shane Morrison, Mark W Moses, Ulrich Mueller, Kate Muller, Kamarul Imran Musa, Mohsen Naghavi, Lemma Negesa, Ionut Negoi, Ruxandra Irina Negoi, Ole Norheim, Jean Jacques Noubiap, Carla Makhlouf Obermeyer, Felix Ogbo, In-Hwan Oh, Olanrewaju Oladimeji, Andrew T Olagunju, Pedro Olivares, Bolajoko Olusanya, Jacob Olusanya, Kanyin Ong, Eyal Oren, Alberto Ortiz, Mayowa Owolabi, Mahesh PA, Songhomitra Panda-Jonas, Katherine Paulson, Konrad Pesudovs, Michael R Phillips, Huyen Phuc Do, David M Pigott, Christine Pinho, Michael Piradov, Farhad Pishgar, Richie Poulton, Mostafa Qorbani, Amir Radfar, Anwar Rafay, Vafa Rahimi-Movaghar, Mahfuzar Rahman, Rajesh Kumar Rai, Puja C Rao, Salman Rawaf, Robert C Reiner, Nikolas Reinig, Andre Renzaho, Satar Rezaei, María Jesús Ríos Blancas, David Rojas-Rueda, Gholamreza Roshandel, Ambuj Roy, Mahdi Safdarian, Saeid Safiri, Rajesh Sagar, Joshua A Salomon, Abdallah M Samy, Juan Sanabria, Damian Santomauro, Itamar Santos, João Vasco Santos, Milena Santric Milicevic, Maheswar Satpathy, Monika Sawhney, Sonia Saxena, Maria Inês Schmidt, Matthew T Schneider, Aletta E Schutte, David C Schwebel, Falk Schwendicke, Sadaf Sepanlou, Masood Ali Shaikh, Morteza Shamsizadeh, Jun She, Girma Temam Shifa, Mika Shigematsu, Rahman Shiri, Reza Shirkoohi, Diego Augusto Santos Silva, Jasvinder Singh, Dhirendra Sinha, Eirini Skiadaresi, Amber Sligar, Luciano Sposato, Chandrashekhar T Sreeramareddy, Jeffrey D Stanaway, Dan Stein, Sabine Steinke, Mark Stokes, Bryan Strub, Mu'awiyyah Babale Sufiyan, Bryan Sykes, Dillon O Sylte, Cassandra Szoeke, Cuong Tat Nguyen, Nuno Taveira, Kavumpurathu Thankappan, Amanda Thrift, Roman Topor-Madry, Miguel Tortajada-Girbés, Thomas Truelsen, Derrick Tsoi, Stefanos Tyrovolas, Kingsley N Ukwaja, Olalekan A Uthman, Job FM van Boven, Tommi Vasankari, Narayanaswamy Venketasubramanian, Vasiliy Vlassov, Stein Emil Vollset, Theo Vos, Tolassa Wakayo, Yuan-Pang Wang, Elisabete Weiderpass, Robert Weintraub, Andrea Werdecker, Ronny Westerman, Harvey Whiteford, Tissa Wijeratne, Charles Shey Wiysonge, Charles Wolfe, Denis Xavier, Gelin Xu, Bach Xuan Tran, Bereket Yakob, Naohiro Yonemoto, Seok-Jun Yoon, and Zoubida Zaidi.

Extracting, cleaning, or cataloging data; designing or coding figures and tables

Kalkidan Hassen Abate, Muktar Ahmed, Sneha Aiyar, Christine Allen, Marlena S Bannick, James Bennett, Adugnaw Berhane, Devasahayam Jesudas Christopher, Sergey Ermakov, Joseph Frostad, Nancy Fullman, Ayele Geleto, Scott Glenn, William W Godwin, Chad Ikeda, Dube Jara, Sarah C Johnson, Mikk Jürisson, André Karch, Laura Kemmer, Young-Ho Khang, Jun Kim, Barthelemy Kuate Defo, Michael J Kutz, Misgan Legesse Liben, Patrick Y Liu, Emilie Maddison, Mohsen Mazidi, Haftay Berhane Mezgebe, Anoushka Millear, Shawn Minnig, Cliff Mountjoy-Venning, Mohsen Naghavi, Lemma Negesa, Emma Nichols, Mahesh PA, Marissa B Reitsma, Gregory A Roth, Abdallah M Samy, Maheswar Satpathy, Peilin Shi, Dhirendra Sinha, Erica Leigh Slepak, Alison Smith, Patrick Sur, Tianchan Tao, Mohammad Tavakkoli, Anna Torre, Kingsley N Ukwaja, Rachel Updike, Tissa Wijeratne, Rachel Woodbrook, Simon Yadgir, Luis Zavala-Arciniega, and Ben Zipkin.

Managing the overall research enterprise

Elizabeth A Cromwell, Nancy Fullman, Emmanuela Gakidou, Caitlin Hawley, Simon I Hay, Chantal Huynh, Laura Kemmer, Xiaofeng Liang, Stephen S Lim, Rafael Lozano, George A Menash, Ali H Mokdad, Kate

Muller, Mohsen Naghavi, Carla Makhlouf Obermeyer, Helen Elizabeth Olsen, Puja C Rao, Robert C Reiner, Joseph Salama, Katya A Shackelford, Chloe Shields, Amber Sligar, Jeffrey D Stanaway, and Theo Vos.

Did not provide authorship contribution information

Samir Bhatt, Lemma Negesa Bulto Bulto, Ewan Cameron, Shirin Djalalinia, Timothy Lucas, David M Pereira, and Santosh Kumar Tadakama.

Methods Appendix: Figures and Tables

Methods Appendix Table 1. GATHER checklist of information that should be included in reports of global health estimates, with descriptions of compliance and location of information for SDG Capstone

Methods Appendix Figure 1. Comparison of the health-related SDG index values using the arithmetic mean of targets versus the geometric mean, by country, 2016.

Methods Appendix Figure 2. Comparison of the health-related SDG index ranks using the arithmetic mean of targets versus the geometric mean, by country, 2016.

Methods Appendix Figure 3. Comparison of the health-related SDG index values using geometric mean of the minimums across each target versus the standard geometric mean, by country, 2016.

Methods Appendix Figure 4. Comparison of the health-related SDG index ranks using geometric mean of the minimums across each target versus the standard geometric mean, by country, 2016.

Methods Appendix Figure 5. Comparing the absolute change in the health-related SDG index from 2016 to 2030 using the geometric versus arithmetic mean.

Supplementary Results: Figures and Tables

Supplementary Figure 1. Comparing the UHC index with the geometric mean of nine UHC tracer interventions in 2016, by country.

Supplementary Figure 2. Comparing the UHC index in 2000 and 2016, by country.

Supplementary Figure 3. Projected performance, based on past trends, on the health-related SDG index, MDG index, and non-MDG index, and 37 individual health-related indicators, by country, 2030.

Supplementary Figure 4. Absolute projected change in the health-related SDG index, MDG index, and non-MDG index, and 37 individual health-related indicators, by country from 2016 to 2030.

Supplementary Figure 5. Comparing attainment of defined health-related SDG indicator targets in 2016 and, based on past trends, projected to be attained in 2030, by country.

Supplementary Figure 6. Comparing attainment of defined and conservative health-related SDG indicator targets in 2016 and, based on past trends, projected to be attained in 2030, by country.

Supplementary Table 1. Health-related SDGs excluded in the present analysis, and measurement needs and strategy for future reporting, by SDG target.

Supplementary Table 2. Scaled values for the health-related SDG index, MDG index, and non-MDG index, and 37 individual health-related indicators, by country, in 2000, 2015, 2016, and 2030.

Supplementary Table 3. Unscaled values for the 37 individual health-related indicators, by country, in 2000, 2015, 2016, and 2030.

Preamble

This appendix provides methodological detail, supplemental figures and tables, and more detailed results for the health-related Sustainable Development Goals (SDGs). The appendix is organized into broad sections following the structure of the main paper. This study complies with the Guidelines for Accurate and Transparent Health Estimates Reporting (GATHER) recommendations, and this appendix is more comprehensive and encyclopedic than previous Global Burden of Disease appendices. It includes detailed tables, figures, indicator modeling write-ups and flowcharts, and information on data sourcing in an effort to maximize transparency in our estimation processes and provide a comprehensive account of analytical steps. Components of this document are the same as described in earlier GBD 2016 Capstone appendices but much more of this appendix are new text for the SDG Capstone. We intend this to be a living document, to be updated with each annual iteration of the Global Burden of Disease and in accordance with the 15 year timeline of the SDG cycle until their conclusion in 2030.

GATHER statement

This study complies with the guidelines for Accurate and Transparent Health Estimates Reporting (GATHER) recommendations. We have documented the steps involved in our analytical procedures and detailed the data sources used in compliance with the Guidelines for Accurate and Transparent Health Estimates Reporting (GATHER).

Methods Appendix Table 1. GATHER checklist of information that should be included in reports of global health estimates, with description of compliance and location of information for SDG Capstone

#	GATHER checklist item	Description of compliance	Reference
**Objectives and funding**			
1	Define the indicators, populations, and time periods for which estimates were made.	Narrative provided in paper and appendix describing indicators, definitions, and populations.	Summary; Main text; Appendix Part 1. Sections 1-3; Supplementary Results
2	List the funding sources for the work.	Funding sources listed in paper.	Main text
**Data Inputs**			
For all data inputs from multiple sources that are synthesized as part of the study:			
3	Describe how the data were identified and how the data were accessed.	Narrative description of data seeking methodology provided.	Appendix Part 1. Sections 1-3
4	Specify the inclusion and exclusion criteria. Identify all ad-hoc exclusions.	Narrative about inclusion and exclusion criteria by data type provided.	Appendix Part 1. Sections 1-3
5	Provide information on all included data sources and their main characteristics. For each data source used, report reference information or contact name/institution, population represented, data collection method, year(s) of data collection, sex and age range, diagnostic criteria or measurement method, and sample size, as relevant.	List of all data sources provided in submission materials; interactive, online data source tool that provides metadata for data sources by component, geography, cause, risk, or impairment has been developed.	Appendix Part 3. Section 1.<br><br>http://ghdx.healthdata.org/<br><br>There is a forthcoming custom data source tool with additional information on data sourcing for GBD and SDG capstone publications.
6	Identify and describe any categories of input data that have potentially important biases (e.g., based on characteristics listed in item 5).	Summary of known biases by cause included in methodological appendix.	Appendix Part 1. Section 3
For data inputs that contribute to the analysis but were not synthesized as part of the study:			
7	Describe and give sources for any other data inputs.	Included in list of all data sources provided in submission materials, as well as online data source tool.	http://ghdx.healthdata.org/<br><br>There is a forthcoming custom data source tool with additional information on data sourcing for GBD and SDG capstone publications.
For all data inputs:			
8	Provide all data inputs in a file format from which data can be efficiently extracted (e.g., a spreadsheet as opposed to a PDF), including all relevant meta-data listed in item 5. For any data inputs that cannot be shared due to ethical or legal reasons, such	Downloads of input data will be available through online tools, including data visualization tools and data query tools. Input data not available in tools will be made available upon request.	Online data tools http://www.healthdata.org/results/data-visualizations; http://ghdx.healthdata.org/; http://ghdx.healthdata.org/gbd-data-tool

	as third-party ownership, provide a contact name or the name of the institution that retains the right to the data.		
**Data analysis**			
9	Provide a conceptual overview of the data analysis method. A diagram may be helpful.	Flow diagrams of the overall methodological processes, as well as cause-specific modelling processes have been provided.	Main text; Appendix Part 1. Section 3
10	Provide a detailed description of all steps of the analysis, including mathematical formulae. This description should cover, as relevant, data cleaning, data pre-processing, data adjustments and weighting of data sources, and mathematical or statistical model(s).	Flow diagrams and corresponding methodological write-ups for each cause and modelling processes have been provided.	Appendix Part 1. Section 3
11	Describe how candidate models were evaluated and how the final model(s) were selected.	Provided in the methodological write-ups.	Appendix Part 1. Section 3
12	Provide the results of an evaluation of model performance, if done, as well as the results of any relevant sensitivity analysis.	Provided in the methodological write-ups.	Appendix Part 2
13	Describe methods for calculating uncertainty of the estimates. State which sources of uncertainty were, and were not, accounted for in the uncertainty analysis.	Provided in the methodological write-ups.	Appendix Part 1. Section 3
14	State how analytic or statistical source code used to generate estimates can be accessed.	Access statement provided.	This will be available in an online repository that will be released upon publication of GBD 2016 Capstones.
**Results and Discussion**			
15	Provide published estimates in a file format from which data can be efficiently extracted.	GBD 2016 results will be made available through online data visualization tools, the Global Health Data Exchange, and the online data query tool.	Supplementary Results
16	Report a quantitative measure of the uncertainty of the estimates (e.g. uncertainty intervals).	Uncertainty intervals are provided with all results.	Main text; Supplementary Results
17	Interpret results in light of existing evidence. If updating a previous set of estimates, describe the reasons for changes in estimates.	Discussion of methodological changes between SDG rounds provided in the narrative of the paper and appendix.	N/A
18	Discuss limitations of the estimates. Include a discussion of any modelling assumptions or data limitations that affect interpretation of the estimates.	Discussion of limitations provided in the narrative of the main paper as well as in the methodological write-ups in the appendix.	Main text; Appendix Part 1. Section 3

Part 1. Health-related SDG indicators

Section 1. Sustainable Development Goals overview

In September 2015, the United Nations (UN) General Assembly established the Sustainable Development Goals (SDGs). The SDGs substantially broaden the development agenda beyond the MDGs and are expected to frame UN member state policies through 2030. In March 2017, the global SDG indicator framework was updated, now specifying 17 universal goals, 169 targets, and 232 indicators leading up to 2030. Here we provide an analysis of 37 out of the 50 health-related SDG indicators based on data used and generated by the Global Burden of Diseases, Injuries, and Risk Factors Study 2016 (GBD 2016).

Section 2. Health-related SDGs

Health is a core dimension of the SDGs; the third SDG aims to "ensure healthy lives and promote wellbeing for all at all ages." Health-related indicators are also present among ten of the other 16 goals. Across these 11 goals, there are 29 health-related targets with a total of 50 health-related indicators.

Of the 50 health-related indicators included as part of the SDGs, estimates for 37 indicators, using consistent approaches built on systematic efforts to compile all available data, are included as part of the GBD study. In this paper, while acknowledging the continued debate about the structure and choices of SDG indicators, we use the GBD study to provide an assessment of the current status of these 37 health-related SDG indicators, develop and compute a summary indicator of the health-related SDG indicators, and document historical trends. For GBD 2016, we produce projections based on past trends for the health-related SDGs through 2030 and examine projected attainment for defined SDG targets by 2030.

The GBD study is an annual effort to measure the health of populations at national, and selected sub-national levels, from 1990 to the most recent year (2016 for GBD 2016). The GBD study produces estimates of mortality and morbidity by cause, age and sex as well as that attributable burden to a selected set of major risk factors. Many of the 50 health-related SDG indicators are produced as part of the GBD. Elsewhere in this appendix, we outline the 10 SDGs, corresponding 24 health-related targets, and 37 health-related indicators included in this iteration of the GBD. Part 1. Section 3 of this appendix also further outlines the definition of each indicator used in analysis, as well as the estimation method and data sources.

Direct outputs of the GBD study that are health-related SDG indicators include mortality rates disaggregated by age (under-5 and neonatal) and cause (maternal, cardiovascular diseases, cancers, diabetes, chronic respiratory diseases, road injuries, self-harm, unintentional poisonings, exposure to forces of nature, interpersonal violence, and conflict and terrorism) as well as measures of disease incidence (HIV, malaria, tuberculosis [TB], hepatitis B) and prevalence (neglected tropical diseases [NTDs]). The GBD risk factor analysis includes measurement of exposure prevalence included as health-related SDG indicators (under-5 stunting, wasting and overweight; tobacco smoking; harmful alcohol use; intimate partner violence; unsafe water, sanitation, and hygiene [WaSH]; household air pollution; and ambient particulate matter) as well as deaths or disease burden attributable to risk factors selected as health-related SDG indicators (WaSH, household and ambient air pollution, and occupational risks). In addition, a number of measures of intervention coverage, including skilled birth attendance, antenatal care, in-facility delivery rates, met need for family planning with modern methods, antiretroviral therapy, and coverage of several vaccines are produced within the GBD study.

As noted in Table 1 in the main manuscript, for selected SDG indicators, we made modifications to the definition for clarity and/or based on the definition used in GBD. For example, Indicator 2.2.2 proposes to measure of malnutrition that combined prevalence of wasting and overweight among children under 5. As childhood wasting and overweight have very different determinants, effects on health outcomes, and interventions, we have selected to report them separately. For childhood overweight, we report prevalence among children aged 2 to 4 years, the definition used in GBD based on thresholds set by the International Obesity Task Force (IOTF).

Four indicators have been added for the GBD 2016 analysis: vaccine coverage (Indicator 3.b.1); two violence indicators (prevalence of physical or sexual violence [16.1.3] and childhood sexual abuse [16.2.3]); and well-certified death registration (17.19.2c). Further, we have expanded the measurement of the universal health coverage (UHC) indicator (3.8.1) to represent a broader range of essential health services. This was achieved by including risk-standardized death rates from 32 causes considered amenable to personal healthcare – that is, deaths from these causes should not occur in the presence of high-quality healthcare.

Further details on the estimation used for all indicators, compliant with Guidelines for Accurate and Transparent Health Estimates Reporting (GATHER), are included in Appendix Part 1. Section 3. Indicator-specific estimation.

Section 3. Indicator-specific estimation

The indicator-specific modeling write-ups follow the order of the SDG goals, targets, and indicators established by the UN. In some cases, multiple indicators were addressed in a single write-up, for example mortality due to natural disasters (SDG indicators 1.5.1, 11.5.1, and 13.1.1) are included in a single write-up along with mortality due to conflict and terrorism (16.1.2). In other cases, particular measures may be present in multiple indicators (e.g., mortality due to cardiovascular diseases are included in SDG indicators 3.4.1 and 3.8.1); in these cases, we refer include these model write-ups for one indicator, and reference that indicator write-up as needed elsewhere.

The organization of this section is as follows:

Natural disaster mortality (1.5.1, 11.5.1, 13.1.1), conflict and terrorism mortality (16.1.2)
Child stunting (2.2.1) and child wasting (2.2.2a)
Child overweight (2.2.2b)
Maternal mortality ratio (3.1.1.)
Skilled birth attendance (3.1.2, also in the UHC index [3.8.1])
Under-5 mortality (3.2.1), neonatal mortality (3.2.2)
HIV incidence (3.3.1)
TB incidence (3.3.2)
Malaria incidence (3.3.3)
Hepatitis B incidence (3.3.4)
NTD prevalence (3.3.5) – includes 15 individual NTDs

NCD mortality (3.4.1) - includes cardiovascular diseases, cancers, diabetes, and chronic respiratory diseases
Self-harm mortality (3.4.2), road injury mortality (3.6.1), unintentional poisonings mortality (3.9.3), interpersonal violence mortality (homicide) (16.1.1)
Alcohol use (3.5.2)
Met need for family planning with modern methods (3.7.1, also in the UHC index [3.8.1])
Adolescent birth rate (3.7.2)
Universal health coverage (UHC) index (3.8.1) – includes coverage of three childhood vaccines, antenatal care (1 and 4 visits), in-facility delivery rate, antiretroviral therapy coverage, and risk-standardized death rates from causes amenable to healthcare (3.8.1)
Mortality attributable to household air pollution and ambient air pollution (3.9.1), household air pollution (7.1.2), and mean PM2.5 (11.6.2)
Mortality attributable to WaSH (3.9.2), water (6.1.1), sanitation (6.2.1a), access to handwashing facility (6.2.1b)
Smoking prevalence (3.a.1)
Vaccine coverage (3.b.1)
Prevalence of intimate partner violence (5.2.1)
DALY rates attributable to occupational risks (8.8.1)
Prevalence of physical or sexual violence (16.1.3)
Child sexual abuse (16.2.3)
Well-certified death registration (17.19.2c)

1.5.1, 11.5.1, 13.1.1, and 16.1.2 Fatal Discontinuities SDG Capstone Appendix

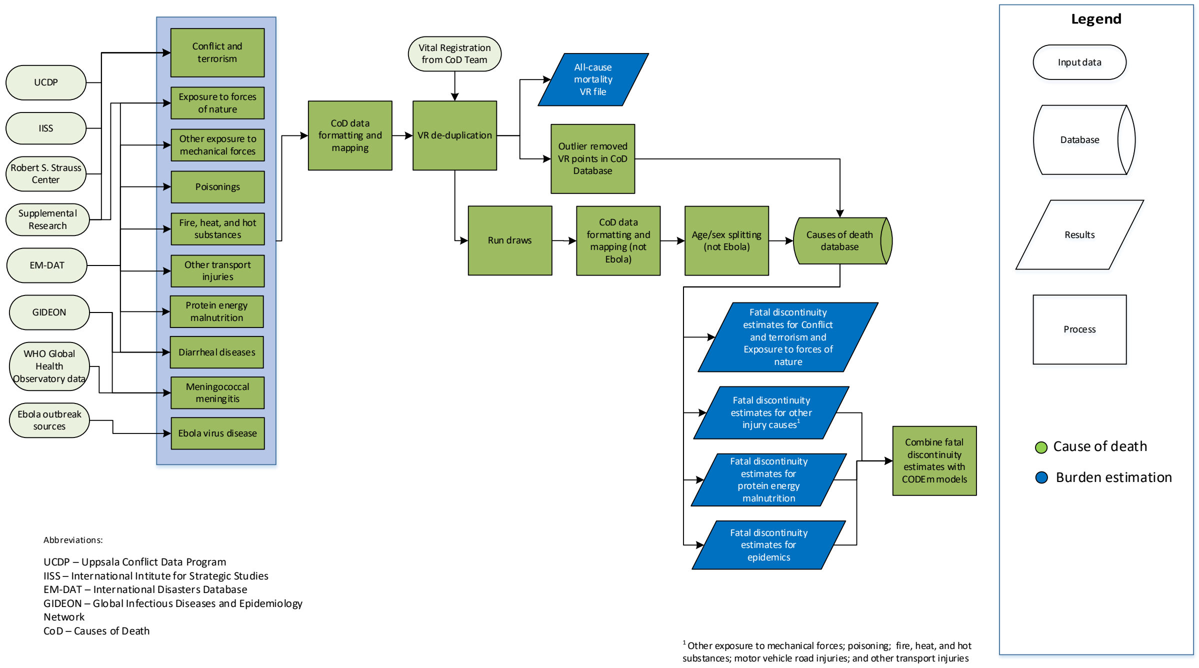

Input data

Indicator definition

This modeling strategy encompasses indicators associated with mortality due to exposure to forces of nature (natural disasters): 1.5.1, 11.5.1, 13.1.1; and mortality due to conflict and terrorism: 16.1.2.

Indicator 1.5.1

As a component of SDG Goal 1. End poverty in all its forms everywhere, SDG Target 1.5., by 2030, build the resilience of the poor and those in vulnerable situations and reduce their exposure and vulnerability to climate-related extreme events and other economic, social and environmental shocks and disasters, is measured using SDG Indicator 1.5.1, deaths due to exposure to forces of nature per 100,000.

Indicator 11.5.1

As a component of SDG Goal 11. Make cities and human settlements inclusive, safe, resilient and sustainable, SDG Target 11.5, by 2030, significantly reduce the number of deaths and the number of people affected and substantially decrease the direct economic losses relative to global gross domestic product caused by disasters, including water-related disasters, with a focus on protecting the poor and people in vulnerable situations, is measured using SDG Indicator 11.5.1, deaths due to exposure to forces of nature per 100,000.

Indicator 13.1.1

As a component of SDG Goal 13. Take urgent action to combat climate change and its impacts, SDG Target 13.1, strengthen resilience and adaptive capacity to climate-related hazards and natural disasters in all countries, is measured using SDG Health Index Indicator 13.1.1, deaths due to exposure to forces of nature per 100,000.

Indicator 16.1.2

As a component of SDG Goal 16. Promote peaceful and inclusive societies for sustainable development, provide access to justice for all and build effective, accountable and inclusive institutions at all levels, SDG Target 16.1, significantly reduce all forms of violence and related death rates everywhere, is measured using SDG Indicator 16.1.2, deaths due to conflict and terrorism per 100,000.

Overall

Input data for fatal discontinuities are compiled a range of sources, including country vital registration (VR) data; international databases that capture several cause-specific fatal discontinuities; and supplemental data in the presence of known issues with data quality or representativeness, or time lags in reporting. A systematic literature review was not used to identify input data for fatal discontinuities, though some literature sources were identified through online supplemental research. Below we provide more detail on the different input data sources by sub-causes of fatal discontinuities.

Subnational locations and population splitting

In locations where we produced estimates at the subnational level for GBD 2016, deaths due to all fatal discontinuity causes were assigned to the relevant subnational location(s) when that information could be obtained either through country data sources (e.g., VR) or through additional online research. If no subnational location could be found, the deaths were split proportionally by population across all subnational locations.

In locations that have experienced boundary changes or split from other locations that we currently estimate (e.g., the former Yugoslavia, Czechoslovakia, the Soviet Union, Sudan and South Sudan), we split deaths due to events that occurred prior to boundary changes proportionally based on the populations residing within the boundaries of present-day locations unless we found documentation that clearly indicated whether the event and corresponding deaths occurred in one of the present-day GBD 2016 locations.

Locations with 4- or 5-star data quality ratings

For countries and territories assigned 4- or 5-star data quality ratings (see Section 2 of the appendix for details), we prioritized data from country-specific vital registration. VR data for fatal discontinuities was exclusively used in 4- and 5-star locations unless there was well-known data quality issues or discrepancies in the cause of death data reporting related to a particular event (e.g., supplemental death data for Louisiana was used for Hurricane Katrina because of established data reporting issues). The process for identification of location-year fatal discontinuities is described more in the Modelling strategy below.

Locations with less than 4-star data quality ratings

For countries and territories assigned data quality ratings below 4 stars, we compared VR with data available from alternative sources for Exposure to forces of nature, taking the highest death estimate

available from all sources. For other fatal discontinuity causes, we disregarded lower quality VR and used well-established databases by type of fatal discontinuity. Whenever specific events were identified that did not have corresponding data points within these databases, we used supplemental data sources, including scientific literature.

Major data sources other than country VR for each fatal discontinuity cause follow.

Conflict and terrorism. Data for conflict and terrorism come from the Uppsala Conflict Data Program (UCDP), International Institute for Strategic Studies, and Robert S. Strauss Center for International Security and Law. The table below provides details about the various datasets we utilized from these sources, the dates they were last accessed, and the years for which we used the data provided.

Data source name	Date accessed	Years of data downloaded	Type of data included
**Uppsala Conflict Data Program**[1]			
Battles	10/6/16	1989-2015	Armed conflict: incompatibility that concerns government and/or territory over which the use of armed force between the military forces of two parties, of which at least one is the government of a state, which resulted in deaths
Non-state	10/6/16	1989-2015	The use of armed force between two organized armed groups, neither of which is the government of a state, which results in deaths
One-sided	10/6/16	1989-2015	The use of armed force by the government of a state or by a formally organized group against civilians which results in deaths
Georeferenced Event Dataset	10/6/16	1989-2015	UCDP battles, non-state, and one-sided conflict deaths with the most disaggregated location information available
PRIO Battles Deaths Dataset	10/6/16	1970-1988	Armed conflict (civil wars, etc.)
**International Institute for Strategic Studies**			
Armed Conflict Dataset	10/6/16	1997-Present	Insurgency, Inter-state, Intra-state conflict deaths
**Robert S. Strauss Center For International Security And Law**			
Armed Conflict Location and Event Dataset (ACLED)	10/6/16	1997-2016	Actions of opposition groups, governments, and militias in selected locations in Africa and Asia, specifying the exact location and date of battle events, transfers of military control, headquarter establishment, civilian violence, and rioting
Social Conflict Analysis Database (SCAD)	10/6/16	1990-2016	Protests, riots, strikes, inter-communal conflict, government violence against civilians, and other forms of social conflict (covers Africa and Latin America)

Supplemental online research was conducted for recent conflicts where the databases above were not up-to-date. In addition, deaths due to conflict and terrorism in Iraq from 2003 to present were estimated using a combination of supplemental sources. The source found with the lowest number of

deaths, Iraq Body Count[2], was used as the lower bound of the uncertainty interval from 2003 to 2016. Estimates from the Iraq Mortality Study by Hagopian et al[3] from 2003 to 2006, the deadliest years of the war, were used to scale deaths to generate the upper uncertainty interval limits using the following formula:

$$deaths_{GBD\ 2016,\ high} = deaths_{IBC} \cdot \overline{\left[\frac{deaths_{IMS}}{deaths_{IBC}}\right]}_{2003-2006}$$

We used the average ratio between IMS and IBC reported deaths between 2003 and 2006, multiplied by the number of deaths reported by the IBC. This high estimate is carried forward through 2016 under the assumption that the Iraq Body Count similarly undercounts the number of deaths due to the ongoing civil war in Iraq. The final, best estimate for conflict and terrorism deaths in Iraq from 2003 to 2016 is the midpoint of the high and low estimates given above.

We identified four major conflicts that were not represented in these databases: 1997 civil conflict in Albania[4]; 1971 genocide in Bangladesh[5]; 1972 genocide in Burundi[6]; and 1993 genocide in Burundi[6]. In these cases, we used literature sources in order to account for these fatal discontinuities.

For country-years where multiple sources provided estimates, we prioritized sources in the following order: (1) country VR data, if death estimates were highest of all sources; (2) UCDP; (3) IISS; (4) country VR if death estimates were not the highest of all sources; (5) Robert Strauss Center; (6) online supplemental research.

Exposure to forces of nature, other injury causes, and protein-energy malnutrition. The Centre for Research on the Epidemiology of Disasters' International Disaster Database (EM-DAT) served as the primary non-VR source of fatal discontinuities due to exposure to forces of nature (i.e., natural disasters); other transport injuries (e.g., plane, train, and boat accidents); poisonings; fire, heat, and hot substances; other exposure to mechanical forces (eg, building collapse); and protein-energy malnutrition (ie, famine or severe drought). Data from EM-DAT were last accessed March 29, 2017. Supplemental online research was conducted for events where EM-DAT was not up-to-date.

For country-years where multiple sources provided estimates, we prioritized sources in the following order: (1) country VR data, if data quality rating is 4 or 5 stars; (2) country VR data if data quality rating is less than 4 stars and death estimates were highest of all sources; (3) EM-DAT; (4) online supplemental research. Exceptions were made where it was clear that VR systems had been compromised by the event being measured.

Meningococcal meningitis and diarrheal diseases. New to GBD 2016, we sought to include fatal discontinuities due to a subset of infectious diseases: meningococcal meningitis (or meningococcal infection) and diarrheal disease caused by cholera. These two infectious diseases were included on the fatal discontinuity cause list for GBD 2016 because (1) their current modelling strategies with the Cause of Death Ensemble model (CODEm) does not optimally capture the potentially highly variable – or epidemic – mortality levels and trends characteristic of these two causes; and (2) they can contribute to significant total fatalities in a given location-year. Other infectious diseases for which the latter is true – high death rates in the presence of an outbreak or epidemic – are currently modelled with alternative cause of death methods (eg, natural history models for measles and yellow fever), which allow for greater variation year-over-year if or when outbreaks occur. In future iterations of the GBD, we plan to revisit the inclusion criteria for infectious diseases as fatal discontinuities and develop more of an

ensemble approach to modelling causes that can be both endemic (and thus result in more uniform levels and trends over time) and epidemic (and subsequently lead to rapid increases – and decreases – in deaths for a given location-year).

The Global Infectious Diseases and Epidemiology Network (GIDEON) served as the primary data source for collating cholera and meningococcal meningitis or meningococcal infection death reports.[7,8] For any year in which cholera or meningococcal meningitis deaths were recorded in a country or territory covered by the GBD, we directly extracted reported deaths from 1970 to 2016. When there were reporting gaps in cholera or meningococcal meningitis deaths over this period of time and the World Health Organization (WHO) annual cholera or meningitis reports had death reports for those years, we used the WHO reports. The primary exception were two major cholera outbreaks in Bangladesh – 1982-1983 and 1991 – which were not captured by either GIDEON or WHO. As result, we used the EM-DAT records for the 1982-1983 outbreak and literature for the 1991 outbreak.[9]

Ebola. Since GBD 2015, outbreaks due to Ebola virus disease have been estimated using the data and methods described in the Ebola write-up of this appendix and included in GBD death estimates in the same way as other fatal discontinuity causes.

Modelling strategy

All input data for fatal discontinuity causes were run through the causes of death data formatting and mapping process.

VR de-duplication

For country-years where deaths due to fatal discontinuity causes were recorded in both VR and other utilized data sources, the higher of the two estimates were taken in the case of deaths due to conflict and terrorism and exposure to forces of nature.

For the other injury causes that also have a CODEm model, a process was established to avoid duplication of fatal discontinuity deaths in the two models. First, location-years with death data from non-VR sources were identified. If these location-cause-years also had VR death estimates that were greater than 40% higher than the immediately surrounding years and could be linked to a specific fatal discontinuity event, these years were marked as outliers in the VR data and the difference between the outlier year and the average of the surrounding years was included in the relevant cause in the fatal discontinuities database. The deaths from the identified events were subtracted from the all-cause VR estimates used in the all-cause mortality estimation process.

Uncertainty analysis for input and draw-level input to age-sex splitting

Uncertainty intervals for deaths due to conflict and terrorism were generated using UCDP high and low death estimates, except in the case of Iraq 2003-2016, as explained above. In cases where low and high estimates were not included in the available data, the regional average uncertainty interval was applied to the available death estimate across all fatal discontinuity causes.

We assumed a normal distribution using the mean deaths and standard deviation based on high and low estimates. The standard deviation was capped at the mean divided by 1.96 in order to ensure that 95% of the 3,000 draws generated were greater than zero. Non-positive draws were dropped, and 1,000

draws were sampled from the remaining set of positive draws. These 1,000 positive draws were used for final calculations of means and uncertainty intervals.

Age-sex splitting

All compiled data were run through the causes of death age-sex splitting process.

Changes from GBD 2015

GBD 2016 saw an effort to systematize the collection of up-to-date fatal discontinuity data through supplemental online research. New tools included expanded use of web scraping and online media tracking. This process resulted in a more comprehensive set of conflict and terrorism data for 2016, as well as large natural disasters not contained in EM-DAT or VR.

In previous rounds of GBD, deaths due to executions and police conflict were included with conflict and terrorism. In GBD 2016, these causes were separated and estimated separately from the overarching war and conflict cause group using a CODEm model, as described in this appendix.

We added two epidemic infectious diseases, cholera and meningococcal meningitis, to the list of fatal discontinuities in an effort to better capture the large variations in mortality that these causes can incur.

We removed the absolute death threshold for fatal discontinuities and limited our inclusion criteria to an event exceeding a mortality rate threshold per location-year. We view this revision as an improvement for estimating the effects of fatal discontinuities in subnational locations and countries with smaller populations, as an absolute threshold of 10, 20, or 50 deaths would ultimately omit events in these places.

References

1 UCDP/PRIO Armed Conflict Dataset Codebook. Uppsala Conflict Data Program (UCDP); Centre for the Study of Civil Wars, International Peace Research Institute, Oslo (PRIO), 2013.

2 Iraq Body Count. https://www.iraqbodycount.org/database/ (accessed May 8, 2017).

3 Hagopian A, Flaxman AD, Takaro TK, *et al.* Mortality in Iraq Associated with the 2003–2011 War and Occupation: Findings from a National Cluster Sample Survey by the University Collaborative Iraq Mortality Study. *PLOS Medicine* 2013; **10**: e1001533.

4 Jarvis C. The Rise and Fall of Albania's Pyramid Schemes. *F&D* 2000; **37**. http://www.imf.org/external/pubs/ft/fandd/2000/03/jarvis.htm.

5 Obermeyer Z, Murray CJL, Gakidou E. Fifty years of violent war deaths from Vietnam to Bosnia: analysis of data from the world health survey programme. *BMJ* 2008; **336**: 1482–6.

6 Milton L. Rwanda, 1994: International incompetence produces genocide. 1994 https://ezproxy.uwc.edu/login?url=http://search.proquest.com/docview/234405747?accountid=42411.

7 Inc GI, Berger DS. Cholera: Global Status: 2017 edition. GIDEON Informatics Inc, 2017.

8 Inc GI, Berger DS. Bacterial Meningitis: Global Status: 2017 edition. GIDEON Informatics Inc, 2017.

9 Siddique A, Zaman K, Baqui A, *et al.* Cholera Epidemics in Bangladesh: 1985-1991. *Journal of Diarrhoeal Diseases Research* 1992; **10**: 79–86.

2.2.1 and 2.2.2a Child Growth Failure SDG Capstone Appendix

Flowchart

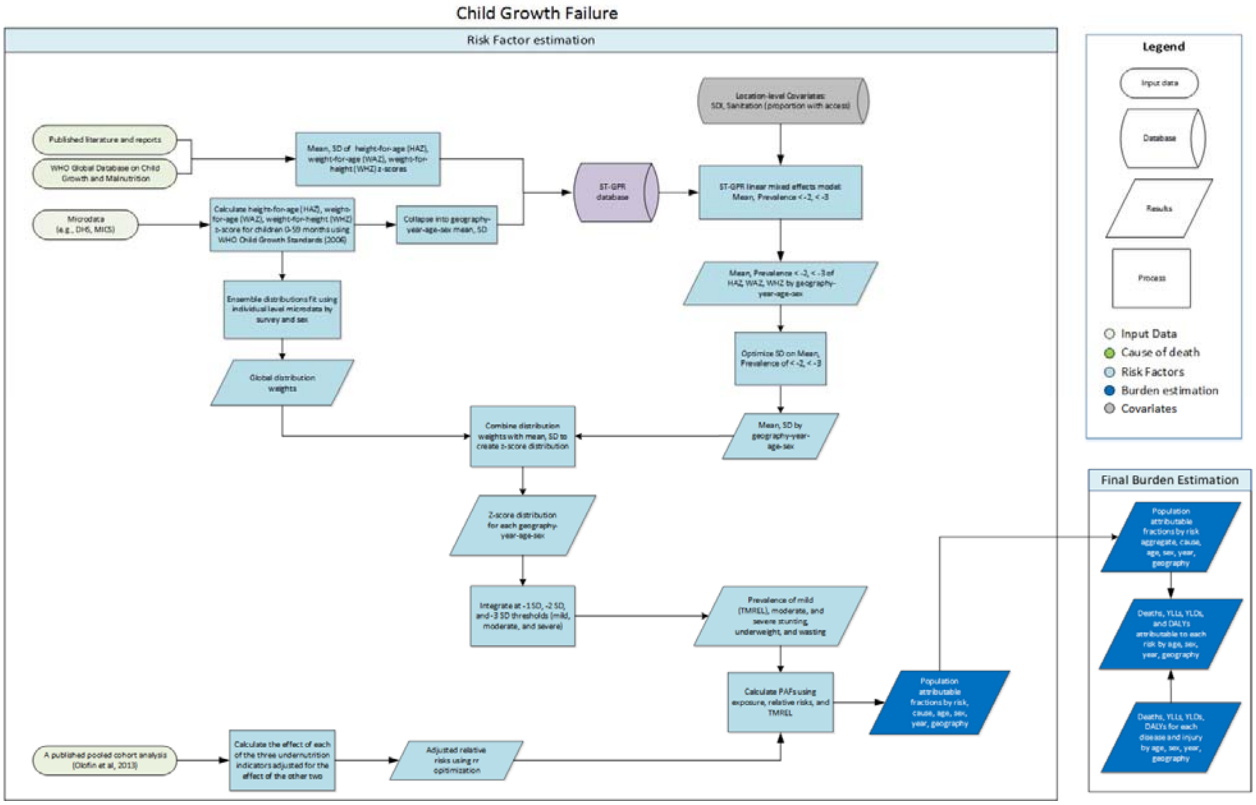

Input Data & Methodological Summary

Indicator definition

This modeling strategy encompasses indicators associated with child undernutrition: 2.2.1 and 2.2.2a.

Indicator 2.2.1

As a component of SDG Goal 2. End hunger, achieve food security, and improved nutrition, SDG Target 2.2, by 2030, end all forms of malnutrition, including achieving, by 2025, the internationally agreed targets on stunting and wasting in children under 5 years of age, and address the nutritional needs of adolescent girls, pregnant and lactating women and older persons, is measured using SDG Indicator 2.2.1, prevalence of stunting among children under 5 (lower than two standard deviations from the median height for age of the reference population).

Indicator 2.2.2a

As a component of SDG Goal 2. End hunger, achieve food security, and improved nutrition, SDG Target 2.2, by 2030, end all forms of malnutrition, including achieving, by 2025, the internationally agreed

targets on stunting and wasting in children under 5 years of age, and address the nutritional needs of adolescent girls, pregnant and lactating women and older persons, is measured using SDG Indicator 2.2.2a, prevalence of wasting among children under 5 (lower than two standard deviations from the median weight for height of the reference population).

Exposure

Case Definition

Child growth failure is estimated using three indicators, stunting, wasting, and underweight, all of which all of which are based on categorical definitions using the WHO 2006 growth standards for children 0-59 months.[1] Definitions are based on Z scores from the growth standards, which were derived from an international reference population. Mild, moderate, and severe categorical prevalences were estimated for each of the three indicators.

Input data

There are two main inputs for the GBD 2016 child growth failure models: microdata from population surveys and tabulated data from reports, literature, and the WHO Global Database on Child Growth and Malnutrition.[2] Population surveys include a variety of multi-country and country-specific survey series such as Multiple Indicator Cluster Surveys (MICS), Demographic and Health Surveys (DHS), Living Standards Measurement Surveys (LSMS), and the China Health and Nutrition Survey (CHNS), as well as other one time country specific surveys such as the Indonesia Family Life Survey and the Brazil National Demographic and Health Survey of Children and Women. These microdata contain information about each individual child's age (from which age in weeks and age in months are calculated), as well as height and/or weight. From that information, a height-for-age z-score (HAZ), weight-for-age z-score (WAZ), and weight-for-height z-score (WHZ) are calculated using the WHO 2006 Child Growth Standards and the LMS method.[3,4]

The second source of data were tabulated data from survey reports, published literature, and the WHO Global Database on Child Growth and Malnutrition that contained the mean z-score and SD for stunting, wasting, and underweight (HAZ, WAZ, WHZ). Any data that was reported using the NCHS 1978 growth standards was given 10% weight in the regression; in future iterations of GBD, we will crosswalk this data to the WHO 2006 Child Growth Standards. All data used in this analysis is catalogued in the Global Health Data Exchange (http://ghdx.healthdata.org). A representative dataset coverage map for moderate stunting is shown below.

Figure 1: Number of data points in moderate stunting (<-2 HAZ) in males, 1990 to 2016

[1] https://www.unicef.org/infobycountry/stats_popup2.html
[2] http://www.who.int/nutgrowthdb/en/
[3] http://www.who.int/childgrowth/standards/en/
[4] http://webnt.calhoun.edu/distance/internet/Business/eco231/downloads/9781441917874-c1.pdf

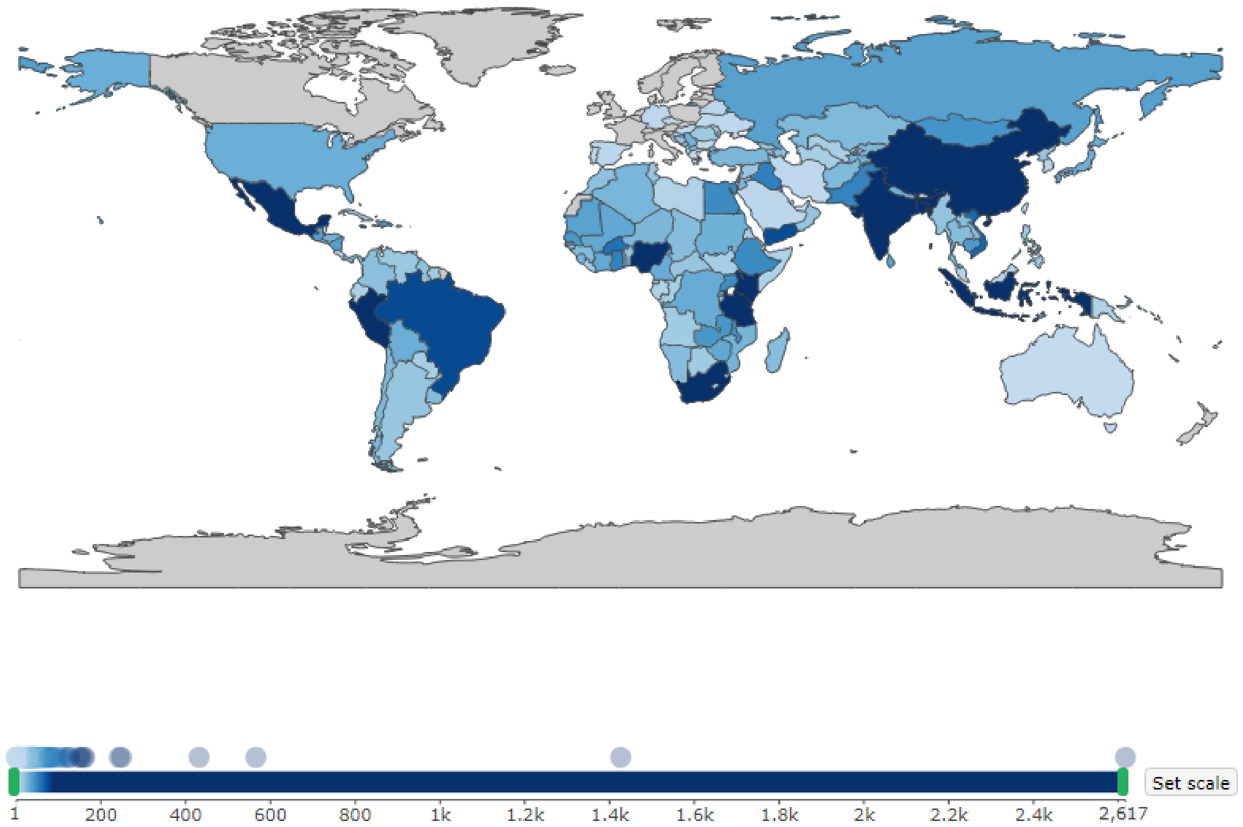

Modeling strategy

Exposure Estimation

The following three-step modeling process was applied to each of stunting, wasting, and underweight.

First, all microdata were fit using an ensemble modeling process, a modeling framework developed for GBD 2016 that is described elsewhere in this appendix. A series of 12 individual distributions (normal, log normal, log logistic, exponential, gamma, mirror gamma, inverse gamma, gumbel, mirror gumbel, Weibull, inverse Weibull, and beta) were fit to the entire set of microdata (approximately 2.5 million individual z-scores) at the individual survey level. A weighting algorithm combined each distribution to find the optimal combination of these distributions for each survey, minimizing the absolute prediction error across the entire distribution. Ensemble weights for each survey were then averaged across all surveys to produce a single set of global weights of the ensemble distributions. Weights were different for each sex, but invariant across geography, time, and age group. All component distributions that were used to derive weights were parameterized using "method of moments," meaning that each corresponding probability density function (PDF) could be described as a function of the mean and variance of the quantity of interest.

Second, models were developed for mean Z scores and prevalence of moderate and severe growth failure. Individual level microdata were collapsed to calculate three metrics: mean z-score, moderate prevalence, and severe prevalence. These data were combined with that derived from literature, GHDx review, and the WHO Global Database on Child Growth and Malnutrition. For those sources where moderate prevalence was reported without a corresponding mean, we calculated a predicted mean using

an ordinary-least square (OLS) regression from those sources where both metrics were present. Each of the three metrics was then modeled using spatiotemporal Gaussian process regression (ST-GPR), a common modeling framework used across GBD 2016 analyses, generating estimates for each age-group, sex, year, and location.

Third, we combined estimates of mean, prevalence (moderate and severe) with ensemble weights in an optimization framework in order to derive the variance that would best correspond to the predicted mean and prevalence. This variance was then paired with the mean and, using the method of moments equation for each of the component distributions of the ensemble, PDF of the distribution of Z-scores were calculated for each location, year, age-group, and sex. PDFs were integrated to determine the prevalence between -1 and -2 Z scores (mild), between -2 and -3 Z scores (moderate), and below -3 Z scores (severe). These were categorical exposures used for subsequent attributable risk analysis.

Differences from GBD 2015

There are several important differences from the GBD 2015 analysis. First, our systematic data searching efforts led to an approximately 30% increase in the number of data sources, including a significant increase in data sources for Oceania, Latin America, and South Asia. Most notable was the increase in data for India through our collaboration with the India Council for Medical Research (ICMR) and Public Health Foundation of India (PHFI). Second, while GBD 2015 also used ST-GPR to model growth failure, models were completed for a single 0-5 age group, followed by application of a pooled uniform age-sex split which resulted in the implicit assumption that the age pattern of growth failure is invariant over time and geography. GBD 2016 estimates, owing to smaller sample sizes in younger age groups, do have wider uncertainty in those age groups. Third, GBD 2015, like all analyses of growth failure before it, assumed that high-income countries had zero prevalence of child growth failure. We have suspended this assumption for GBD 2016 as it is not accurate and instead made explicit estimates of growth failure in all locations. Fourth, GBD 2015 did not use an ensemble approach or estimate the entire distribution of Z scores. Fifth, we have changed the name of this risk factor category changed from childhood undernutrition to child growth failure to more explicitly identify the specific aspects of childhood undernutrition that are covered by the three component indicators.

Theoretical minimum-risk exposure level

Theoretical minimum risk exposure level (TMREL) for underweight, stunting, and wasting was assigned to be greater than or equal to -1 SD of the WHO 2006 standard weight-for-age, height-for-age, and weight-for-height curves respectively. This was unchanged from GBD 2015.

Relative risks

Relative risks (RRs) were derived from a pooled cohort analysis (source and risk-outcome pairs below), which remained the same as GBD 2013 & GBD 2015.[5] The final list of outcomes paired with child growth failure risks included lower respiratory infections (LRI), diarrhea, measles, and protein energy malnutrition (PEM). The RRs were adjusted using an optimization algorithm developed at IHME for GBD 2013 that takes into account covariance between the three child growth failure indicators.

---

[5] Olofin I, McDonald CM, Ezzati M, et al. Associations of suboptimal growth with all-cause and cause-specific mortality in children under five years: a pooled analysis of ten prospective studies. PLoS ONE 2013; 8: e64636

Of historical note, URI and otitis media were included as outcomes in the GBD 2013 risk analysis, based on the “analogy” causal criterion, assuming there is similar pathway as LRI outcome. However, closer review for GBD 2015 did not find sufficient evidence to support their inclusion and they were excluded, a decision that was carried forward into GBD 2016. We also attributed 100% of PEM to childhood wasting and underweight but not stunting. To build on the existing literature base for GBD on risk-outcome pairs, a literature search was conducted for GBD 2016 searching for case-control studies published after January 1st, 1985; this search did not return any sources that were appropriate for this work.

Risk factor	Outcome
Child underweight	Diarrhoeal diseases
Child underweight	Lower respiratory infections
Child underweight	Measles
Child stunting	Diarrhoeal diseases
Child stunting	Lower respiratory infections
Child stunting	Measles
Child wasting	Diarrhoeal diseases
Child wasting	Lower respiratory infections
Child wasting	Measles

2.2.2b High Body Mass index SDG Capstone Appendix

Childhood (Ages 2-19) High Body-Mass Index: Data and Model Flow Chart

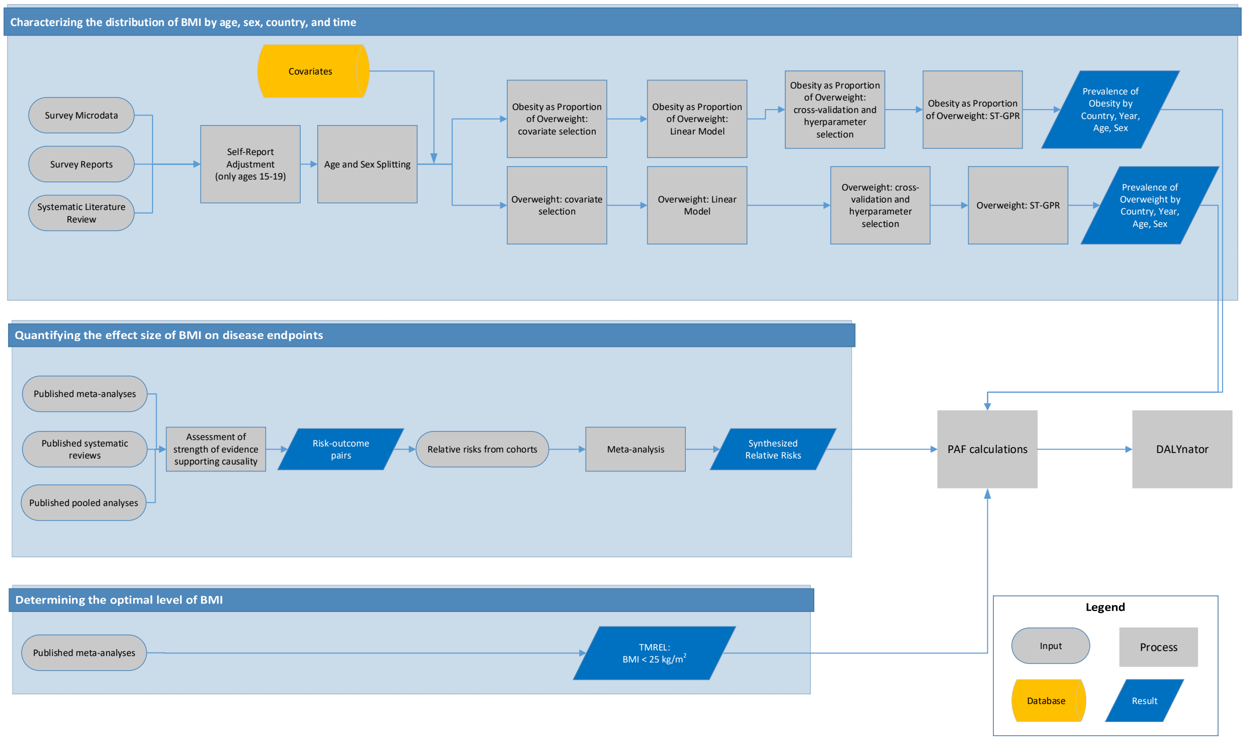

Input data and Methodological Summary

Indicator definition

This modeling strategy encompasses SDG indicator associated with childhood overweight: 2.2.2b.

Indicator 2.2.2b

As a component of SDG Goal 2. End hunger, achieve food security, and improved nutrition, SDG Target 2.2, by 2030, end all forms of malnutrition, including achieving, by 2025, the internationally agreed targets on stunting and wasting in children under 5 years of age, and address the nutritional needs of adolescent girls, pregnant and lactating women and older persons, is measured using SDG indicator 2.2.2b, prevalence of children aged 2 to 4 years with a body-mass index (BMI) exceeding the overweight cut-offs established by the International Obesity Task Force (IOTF) for each sex and by month of age.

Case definition

Exposure to overweight is defined using metrics related to national and subnational estimates of BMI. If a person has a BMI of greater than IOTF cutoff for each sex and age (in month), they are considered overweight.

Data Sources

We systematically searched Medline to identify studies providing nationally or subnationally representative estimates of overweight prevalence, obesity prevalence, or mean body-mass index (BMI) there were published between 1 January 2016 and 31 December 2016 to update the systematic literature search previously performed as part of GBD 2015.

The search for adults was conducted on 4 January 2017 using the following terms:

((("Body Mass Index"[Mesh] OR "Overweight"[Mesh] OR "Obesity"[Mesh]) AND ("Geographic Locations"[Mesh] NOT "United States"[Mesh]) AND ("humans"[Mesh] AND "adult"[MeSH]) AND ("Data Collection"[Mesh] OR "Health Services Research"[Mesh] OR "Population Surveillance"[Mesh] OR "Vital statistics"[Mesh] OR "Population"[Mesh] OR "Epidemiology"[Mesh] OR "surve*"[TiAb]) NOT (Comment[ptyp] OR Case Reports[ptyp] OR "hospital"[TiAb])) AND ("2016/01/01"[Date - Publication] : "2016/12/31"[Date - Publication]))

The search for children was conducted on 4 August 2016 using the following terms:
((("Body Mass Index"[Mesh] OR "Overweight"[Mesh] OR "Obesity"[Mesh]) AND ("Geographic Locations"[Mesh] NOT "United States"[Mesh]) AND ("humans"[Mesh] AND "child"[MeSH]) AND ("Data Collection"[Mesh] OR "Health Services Research"[Mesh] OR "Population Surveillance"[Mesh] OR "Vital statistics"[Mesh] OR "Population"[Mesh] OR "Epidemiology"[Mesh] OR "surve*"[TiAb]) NOT (Comment[ptyp] OR Case Reports[ptyp] OR "hospital"[TiAb])) AND ("2016/01/01"[Date - Publication] : "2016/12/31"[Date - Publication]))

Our search for adult estimates identified 456 abstracts, of which 25 met inclusion criteria and were extracted. The search for child estimates identified 137 articles, of which 4 were extracted. Including sources from the previous GBD systematic literature searches, a total of 11,220 articles were identified, of which 845 were included. Additionally, we searched the Global Health Data Exchange (GHDx) database for individual-level data from major multinational survey series or country-specific surveys and identified 1,038 unique sources meeting the inclusion criteria.

Eligibility Criteria

We included nationally representative studies providing data on mean BMI or prevalence of overweight or obesity among adults or children. For adults, studies were included if they defined overweight as BMI≥25 kg/m^2 and obesity as BMI≥30 kg/m^2, or if estimates using those cutoffs could be back-calculated from reported categories. For children (children ages 2-18), studies were included if they used International Obesity Task Force (IOTF) standards to define overweight and obesity thresholds. We only included studies reporting data collected between 1 January 1980 and 31 December 2016. Studies were excluded if using non-random samples (e.g., case-control studies or convenience samples); conducted among specific subpopulations (e.g., pregnant women, racial or ethnic minorities, immigrants, or individuals with specific diseases); using alternative methods to assess adiposity (e.g., waist-circumference, skin-fold thickness, or hydrodensitometry); having sample sizes of less than 20 per 5-year age-sex group; or providing inadequate information on any of the inclusion criteria. We also excluded review articles and non-English articles.

Data collection process

Where individual-level survey data were available, we computed mean BMI using weight and height and then used BMI to determine the prevalence of overweight and obesity. For individuals aged over 18 years, we considered them to be overweight if their BMI was greater than or equal to 25 kg/m^2, and obese if their BMI was greater than or equal to 30 kg/m^2. For individuals aged 2-18 years, we used monthly IOTF cutoffs[2] to determine overweight and obese status when age in months was available. When only age in years was available, we used the cutoff for the sixth month of that year. Individuals who were obese were also considered to be overweight. We excluded studies using the World Health Organization (WHO) standards or country-specific cutoffs to define childhood overweight and obesity. At the individual level, we considered BMI<10 kg/m^2 and BMI>70 kg/m^2 to be biologically implausible and excluded those observations.

The rationale for choosing to use the IOTF cutoffs over the WHO standards has been described elsewhere. Briefly, the IOTF cutoffs provide consistent child-specific standards for ages 2-18 derived from surveys covering multiple countries. On the other hand, the WHO growth standards apply to children under 5 and the WHO growth reference applies to children ages 5-19. The WHO growth reference for children ages 5-19 was derived from United States' data, which are less representative than the multinational data used by IOTF. Additionally, the switch between references at age 5 can produce artificial discontinuities. Given that we estimate global childhood overweight and obesity for ages 2-19 (with ages 19 using standard adult cutoffs), the IOTF cutoffs were preferable. Additionally, we found that IOTF cutoffs were more commonly used in scientific literature covering childhood obesity.

From report and literature data, we extracted data on mean BMI, prevalence of overweight, and prevalence of obesity, measures of uncertainty for each, and sample size, by the most granular age and sex groups available. Additionally, we extracted the same study-level covariates as were extracted from microdata (measurement, urbanicity, and representativeness), as well as location and year.

In addition to the primary indicators described above, we extracted relevant survey-design variables, including primary sampling unit, strata, and survey weights, which were used to tabulate individual-level microdata and produce accurate measures of uncertainty. We extracted three study-level covariates: 1) whether height and weight data were measured or self-reported, 2) whether the study was predominantly conducted in an urban area, rural area, or both, and 3) the level of representativeness of the study (national or subnational).

Finally, we extracted relevant demographic indicators, including location, year, age, and sex. We estimated the standard error of the mean from individual-level data where available and used the reported standard error of the mean for published data. When multiple data sources were available for the same country, we included all of them in our analysis. If data from the same data source were available in multiple formats such individual-level data and tabulated data, we used individual-level data.

Self-report bias adjustment

We included both measured and self-reported data. Of 68.5 million person-years of data, 16.9 million (25%) were self-reported. We tested for bias in self-report data compared to measured data, which is considered to be the gold-standard. There was no clear direction of bias for children ages 2-14, so for these age groups we only included measured data. For individuals ages 15+, we adjusted self-reported

data for overweight prevalence, obesity prevalence, and mean BMI using the following nested hierarchical mixed-effects regression models, fit using restricted maximum likelihood separately by sex:

$$\mathrm{logit(overweight)}_{c,a,t} = \beta_0 + \beta_1 m + \sum_{k=2}^{19} \beta_k I_{A[a]} + \sum_{l=20}^{55} \beta_l I_{A[a]} I_{M[m]} + \alpha_s + \alpha_s m + \alpha_r + \alpha_r m + \alpha_c + \alpha_c m + \alpha_t + \alpha_t m + \epsilon_{c,a,t}$$

$$\mathrm{logit(obesity)}_{c,a,t} = \beta_0 + \beta_1 m + \sum_{k=2}^{19} \beta_k I_{A[a]} + \sum_{l=20}^{55} \beta_l I_{A[a]} I_{M[m]} + \alpha_s + \alpha_s m + \alpha_r + \alpha_r m + \alpha_c + \alpha_c m + \alpha_t + \alpha_t m + \epsilon_{c,a,t}$$

$$\log(\mathrm{BMI})_{c,a,t} = \beta_0 + \beta_1 m + \sum_{k=2}^{19} \beta_k I_{A[a]} + \sum_{l=20}^{55} \beta_l I_{A[a]} I_{M[m]} + \alpha_s + \alpha_s m + \alpha_r + \alpha_r m + \alpha_c + \alpha_c m + \alpha_t + \alpha_t m + \epsilon_{c,a,t}$$

Where m is a fixed effect on measurement (binary, either measured (1) or self-report (0)), $I_{A[a]}$ is an indicator variable for specific age group A, $I_{A[a]} I_{M[m]}$ is an interaction term between age and measurement, α_s, α_r, and α_c are random effects at the super-region, region, and country, respectively, and α_t is a random effect by time-period (1980–1989, 1990–1999, 2000–2009, 2010–2016). Random effects at the country level and time-period level were used to fit the models, but were taken as noise and were not used in adjustment of self-reported data. We propagated the uncertainty in the self-report adjustment model by adding the variance of each of the regression coefficients used in adjustment to the data variance in delta-transformed space. After adjustment, regressions confirmed that self-reported data were no longer significantly different from measured data. As an additional sensitivity analysis we ran a set of models for overweight and obesity using only measured data and compared the root-mean squared deviation (RMS)E of these models to the RMSE of the full models. RMSE was calculated comparing measured data to the predicted estimates. Differences in RMSE were minimal and are shown in below.

	Measured only	*Measured and self-report*
Overweight	0.063	0.070
Obesity	0.033	0.036

Age and sex splitting

Any report or literature data provided in age groups wider than the standard 5-year age groups or as both sexes combined were split using the approach used by Ng et al (http://www.thelancet.com/journals/lancet/article/PIIS0140-6736(14)60460-8/abstract). Briefly, age-sex patterns were identified using sources with data on multiple age-sex groups and these patterns were applied to split aggregated report and literature data. Uncertainty in the age-sex split was propagated by multiplying the standard error of the data by the square root of the number of splits performed. We did not propagate the uncertainty in the age pattern and sex pattern used to split the data as they seemed to have small effect.

Prevalence estimation for overweight and obesity

After adjusting for self-report bias and splitting aggregated data into 5-year age-sex groups, we used spatiotemporal Gaussian process regression (ST-GPR) to estimate the prevalence of overweight and obesity. This modeling approach has been described in detail elsewhere.

The linear model, which when added to the smoothed residuals forms the mean prior for GPR is as follows:

$$\text{logit}(\text{overweight})_{c,a,t} = \beta_0 + \beta_1\text{energy} + \beta_2\text{SDI} + \beta_3\text{vehicles} + \beta_4\text{urbanicity} + \sum_{k=5}^{22} \beta_k I_{A[a]}$$

$$\text{logit}(\text{obesity/overweight})_{c,a,t} = \beta_0 + \beta_1\text{energy} + \beta_2\text{SDI} + \beta_3\text{vehicles} + \beta_4\text{urbanicity} + \sum_{k=5}^{22} \beta_k I_{A[a]}$$

Estimating mean BMI

To estimate the mean BMI for adults in each country, age, sex, and time period 1980–2016, we first used the following nested hierarchical mixed-effects model, fit using restricted maximum likelihood on data from sources containing estimates of all three indicators (prevalence of overweight, prevalence of obesity, and mean BMI), in order to characterize the relationship between overweight, obesity, and mean BMI:

$$\log(\text{BMI}_{c,a,s,t}) = \beta_0 + \beta_1\text{ow}_{c,a,s,t} + \beta_2\text{ob}_{c,a,s,t} + \beta_3\text{sex} + \sum_{k=4}^{20} \beta_k I_{A[a]} + \alpha_s(1 + \text{ow}_{c,a,s,t} + \text{ob}_{c,a,s,t}) + \alpha_r(1 + \text{ow}_{c,a,s,t} + \text{ob}_{c,a,s,t}) + \alpha_c(1 + \text{ow}_{c,a,s,t} + \text{ob}_{c,a,s,t}) + \epsilon_{c,a,s,t}$$

where $\text{ow}_{c,a,s,t}$ is the prevalence of overweight in country c, age a, sex s, and year t, $\text{ob}_{c,a,s,t}$ is the prevalence of obesity in country c, age a, sex s, and year t, sex is a fixed effect on sex, $I_{A[a]}$ is an indicator variable for age, and α_s, α_r, and α_c are random effects at the super region, region, and country, respectively. The model was run in Stata 13.

We applied 1,000 draws of the regression coefficients to the 1,000 draws of overweight prevalence and obesity prevalence produced through ST-GPR to estimate 1,000 draws of mean BMI for each country, year, age, and sex. This approach ensured that overweight prevalence, obesity prevalence, and mean BMI were correlated at the draw level and uncertainty was propagated.

Estimating BMI distribution

Following the approach described by Ng. et al (https://pophealthmetrics.biomedcentral.com/articles/10.1186/s12963-016-0076-2) and used in GBD 2015, we optimized parameters of a beta distribution to fit estimated mean BMI level, overweight prevalence, and obesity prevalence, by location, year, age, and sex.

3.1.1 Maternal disorders SDG Capstone Appendix

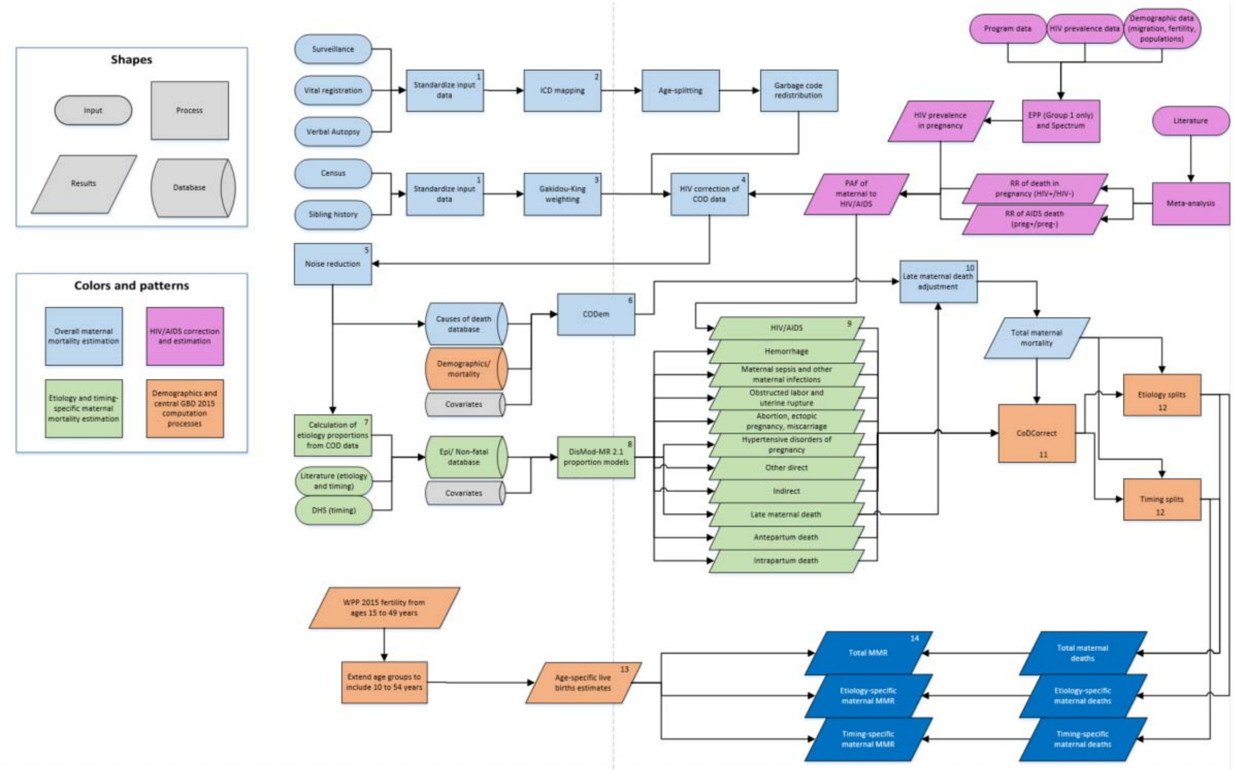

Input Data & Methodological Summary

Indicator definition

This modeling strategy encompassed the indicator associated with the maternal mortality ratio (SDG indicator 3.1.1).

Indicator 3.1.1

As a component of SDG Goal 3. Ensure healthy lives and promote well-being for all at all ages, SDG Target 3.1, by 2030, reduce the global maternal mortality ratio to less than 70 per 100,000 live births, is measured using SDG indicator 3.1.1, maternal mortality ratio (maternal deaths per 100,000 live births).

Input data

CODEm models were informed by centrally prepped data stored in the cause of death (COD) database using standardized processes to adjust for bias due to incompleteness, misclassification, coding to non-specific causes (i.e. garbage codes), stochastic variability, and zero counts. Our GBD 2016 case definition for maternal mortality continues to be all pregnancy-related deaths excluding accidental or incidental causes up to 1 year after the end of the pregnancy.

An updated literature review to inform the relative risk of mortality in pregnancy in HIV-positive versus HIV-negative women produced 23 leads and one usable source. We completed this search on August 30, 2016, using the following search string:

(HIV[Title/Abstract] OR "Acquired Immunodeficiency Syndrome"[Title/Abstract] OR AIDS[Title/Abstract]) AND (“pregnant”[Title/Abstract] OR “pregnancy”[Title/Abstract] OR “postpartum”[Title/Abstract] OR "post partum"[Title/Abstract]) AND (“mortality”[Title/Abstract] OR “death”[Title/Abstract]) NOT "case report" AND "humans"[MeSH Terms] AND (2011/07/06[PDat] : 2016/12/31[PDat])

Correction for incidental HIV deaths was completed during the data preparation phase. Spectrum outputs of HIV prevalence in pregnancy were combined with relative risk of mortality during pregnancy (HIV+ versus HIV-negative) to calculate PAFs. A proportion of these deaths are incidental and a proportion are maternal as determined from two studies that looked at the relative risk of death in HIV positive women who are pregnant versus non-pregnant. All data were corrected using the PAFs. Incidental deaths were removed from sibling history and census data, while maternal HIV deaths were added to VR data. The maternal proportion of the PAF was retained to be combined with estimates of the aetiologic-proportion from other causes as described below.

DisMod-MR 2.1 aetiology proportion models were informed by two sources of data. First, we completed a systematic literature review on August 30, 2016, using the search string below:

(((("maternal mortality"[Title/Abstract] OR "maternal death"[Title/Abstract] OR "MM"[Title/Abstract] OR "confidential enquiry"[Title/Abstract] OR ((obstetric[Title/Abstract] OR pregnancy[Title/Abstract]) AND (etiology[Title/Abstract] OR cause[Title/Abstract] or pattern[Title/Abstract]) AND (death[Title/Abstract] OR mortality[Title/Abstract])))) AND "humans"[MeSH Terms] NOT (fetal[Title/Abstract] OR newborns[Title/Abstract] OR newborn[Title/Abstract] OR neonatal[Title/Abstract] OR “case report”[Title/Abstract] OR “case study”[Title/Abstract] OR pathogenesis[Title/Abstract] OR thromboprophylaxis[Title/Abstract])) OR ((“maternal mortality”[Title/Abstract] OR “maternal death*”[Title/Abstract] OR “MMR”[Title/Abstract]) AND (“Afghanistan”[Title/Abstract] OR “Albania”[Title/Abstract] OR “Algeria”[Title/Abstract] OR “Andorra”[Title/Abstract] OR “Angola”[Title/Abstract] OR “Antigua and Barbuda”[Title/Abstract] OR “Argentina”[Title/Abstract] OR “Armenia”[Title/Abstract] OR “Azerbaijan”[Title/Abstract] OR “Bahrain”[Title/Abstract] OR “Bangladesh”[Title/Abstract] OR “Barbados”[Title/Abstract] OR “Belarus”[Title/Abstract] OR “Belize”[Title/Abstract] OR “Benin”[Title/Abstract] OR “Bhutan”[Title/Abstract] OR “Bolivia”[Title/Abstract] OR “Bosnia and Herzegovina”[Title/Abstract] OR “Botswana”[Title/Abstract] OR “Brazil”[Title/Abstract] OR “Brunei”[Title/Abstract] OR “Bulgaria”[Title/Abstract] OR “Burkina Faso”[Title/Abstract] OR “Burundi”[Title/Abstract] OR “Cambodia”[Title/Abstract] OR “Cameroon”[Title/Abstract] OR “Cape Verde”[Title/Abstract] OR “Central African Republic”[Title/Abstract] OR “Chad”[Title/Abstract] OR “China”[Title/Abstract] OR “Colombia”[Title/Abstract] OR “Comoros”[Title/Abstract] OR “Congo”[Title/Abstract] OR “Costa Rica”[Title/Abstract] OR “Croatia”[Title/Abstract] OR “Cuba”[Title/Abstract] OR “Cyprus”[Title/Abstract] OR “Côte d’Ivoire”[Title/Abstract] OR “Democratic Republic of the Congo”[Title/Abstract] OR “Djibouti”[Title/Abstract] OR “Dominica”[Title/Abstract] OR “Dominican Republic”[Title/Abstract] OR “Ecuador”[Title/Abstract] OR “Egypt”[Title/Abstract] OR “El Salvador”[Title/Abstract] OR “Equatorial Guinea”[Title/Abstract] OR “Eritrea”[Title/Abstract] OR “Ethiopia”[Title/Abstract] OR “Federated States of Micronesia”[Title/Abstract] OR “Fiji”[Title/Abstract] OR “Gabon”[Title/Abstract] OR “Georgia”[Title/Abstract] OR “Ghana”[Title/Abstract] OR “Grenada”[Title/Abstract] OR “Guatemala”[Title/Abstract] OR “Guinea”[Title/Abstract] OR “Guinea-Bissau”[Title/Abstract] OR “Guyana”[Title/Abstract] OR “Haiti”[Title/Abstract] OR “Honduras”[Title/Abstract] OR “India”[Title/Abstract] OR “Indonesia”[Title/Abstract] OR “Iran”[Title/Abstract] OR “Iraq”[Title/Abstract] OR “Jamaica”[Title/Abstract] OR “Jordan”[Title/Abstract] OR “Kazakhstan”[Title/Abstract] OR “Kenya”[Title/Abstract] OR “Kiribati”[Title/Abstract] OR “Kuwait”[Title/Abstract] OR “Kyrgyzstan”[Title/Abstract] OR “Laos”[Title/Abstract] OR “Latvia”[Title/Abstract] OR “Lebanon”[Title/Abstract] OR “Lesotho”[Title/Abstract] OR “Liberia”[Title/Abstract] OR “Libya”[Title/Abstract] OR “Lithuania”[Title/Abstract] OR “Macedonia”[Title/Abstract] OR “Madagascar”[Title/Abstract] OR “Malawi”[Title/Abstract] OR “Malaysia”[Title/Abstract] OR “Maldives”[Title/Abstract] OR “Mali”[Title/Abstract] OR “Malta”[Title/Abstract] OR “Marshall Islands”[Title/Abstract] OR “Mauritania”[Title/Abstract] OR “Mauritius”[Title/Abstract] OR “Moldova”[Title/Abstract] OR “Mongolia”[Title/Abstract] OR “Montenegro”[Title/Abstract] OR “Morocco”[Title/Abstract] OR “Mozambique”[Title/Abstract] OR “Myanmar”[Title/Abstract] OR “Namibia”[Title/Abstract] OR “Nepal”[Title/Abstract] OR “Nicaragua”[Title/Abstract] OR “Niger”[Title/Abstract] OR “Nigeria”[Title/Abstract] OR “North Korea”[Title/Abstract] OR “Oman”[Title/Abstract] OR “Pakistan”[Title/Abstract] OR “Palestine”[Title/Abstract] OR “Panama”[Title/Abstract] OR “Papua New Guinea”[Title/Abstract] OR “Paraguay”[Title/Abstract] OR “Peru”[Title/Abstract] OR “Philippines”[Title/Abstract] OR “Qatar”[Title/Abstract] OR “Romania”[Title/Abstract] OR “Russia”[Title/Abstract] OR “Rwanda”[Title/Abstract] OR “Saint Lucia”[Title/Abstract] OR “Saint Vincent and the Grenadines”[Title/Abstract] OR “Samoa”[Title/Abstract] OR “Saudi

Arabia"[Title/Abstract] OR "Senegal"[Title/Abstract] OR "Serbia"[Title/Abstract] OR "Seychelles"[Title/Abstract] OR "Sierra Leone"[Title/Abstract] OR "Singapore"[Title/Abstract] OR "Solomon Islands"[Title/Abstract] OR "Somalia"[Title/Abstract] OR "South Africa"[Title/Abstract] OR "South Sudan"[Title/Abstract] OR "Sri Lanka"[Title/Abstract] OR "Sudan"[Title/Abstract] OR "Suriname"[Title/Abstract] OR "Swaziland"[Title/Abstract] OR "Syria"[Title/Abstract] OR "São Tomé and Príncipe"[Title/Abstract] OR "Taiwan"[Title/Abstract] OR "Tajikistan"[Title/Abstract] OR "Tanzania"[Title/Abstract] OR "Thailand"[Title/Abstract] OR "The Bahamas"[Title/Abstract] OR "The Gambia"[Title/Abstract] OR "Timor-Leste"[Title/Abstract] OR "Togo"[Title/Abstract] OR "Tonga"[Title/Abstract] OR "Trinidad and Tobago"[Title/Abstract] OR "Tunisia"[Title/Abstract] OR "Turkmenistan"[Title/Abstract] OR "Uganda"[Title/Abstract] OR "Ukraine"[Title/Abstract] OR "United Arab Emirates"[Title/Abstract] OR "Uruguay"[Title/Abstract] OR "Uzbekistan"[Title/Abstract] OR "Vanuatu"[Title/Abstract] OR "Venezuela"[Title/Abstract] OR "Vietnam"[Title/Abstract] OR "Yemen"[Title/Abstract] OR "Zambia"[Title/Abstract] OR "Zimbabwe"[Title/Abstract]) AND "humans"[MeSH] NOT ("demographic and health survey*"[Title/Abstract] OR DHS[Title/Abstract] OR "reproductive health survey*"[Title/Abstract] OR RHS[Title/Abstract]))) AND (2015/04/30[PDat] : 2016/12/31[PDat])) OR ((HIV[Title/Abstract] OR "Acquired Immunodeficiency Syndrome"[Title/Abstract] OR AIDS[Title/Abstract]) AND ("pregnant"[Title/Abstract] OR "pregnancy"[Title/Abstract] OR "postpartum"[Title/Abstract] OR "post partum"[Title/Abstract]) AND ("mortality"[Title/Abstract] OR "death"[Title/Abstract]) NOT "case report" AND "humans"[MeSH Terms] AND (2011/07/06[PDat] : 2016/12/31[PDat]))

A total of 698 sources were reviewed for their title and abstract. Of those selected for full text review, 17 had usable data for aetiology-specific maternal mortality models. All data were prepped as "proportion" of total maternal deaths due to that cause. The second source of data was from the COD database. All aetiology-specific COD data were processed to be "proportion" data by calculating the cause-specific deaths divided by the total maternal deaths for the matching data source, year, age, and location. Owing to the large volume of total COD data and small sample sizes in many locations, COD data were collapsed around each of the five-year periods for which DisMod-MR 2.1 makes distinct estimates (1990, 1995, 2000, 2005, 2010, and 2016). Late maternal death data were only included for the subset of locations where they were reliably coded in raw VR. All data were uploaded to the nonfatal database.

Modelling strategy

Overall maternal mortality was estimated with CODEm. All data from all geographies were reviewed. Outliers were identified as those data where age patterns or temporal patterns were inconsistent with neighbouring age groups or locations or where sparse data were predicting implausible overall temporal or age patterns for a given location.

DisMod-MR 2.1 proportion models for each sub-cause of maternal mortality were all single-parameter meta-regression models. Because many sources do not include the entire cause list, a series of study covariates were used to facilitate crosswalking back to the reference definition. The reference definition ***includes*** "other" direct obstetric complications, indirect maternal deaths, and late maternal death. Country covariates were specific for each model and included abortion legality (for abortion, ectopic pregnancy, and miscarriage), log-transformed lag-distributed income (for sepsis and late maternal death), and logit-transformed in-facility delivery proportion (for haemorrhage, hypertensive disorders of pregnancy, and obstructed labour). The time window was set at +/- 2 years for all models except late maternal death, which was +/- 5 years. The narrower window ensured that any given year of VR data only informed a single estimate.

We corrected the time trend in the CODEm model by identifying the year in which each location began consistently using O95 and O96 codes for late maternal death. These were identified as the earliest year in which the threshold proportion of total maternal deaths coded to late exceeded the lowest reported in the literature (0.5%). After a location was identified as having started using late maternal death codes, we

assumed that practice continued. We adjusted upward results for all years prior to the advent of late maternal death coding using the outputs of the late maternal death proportion DisMod model.

Etiology-specific estimates were derived by multiplying the proportion outputs from DisMod-MR 2.1 by the total maternal deaths for that age-group, location, and year. HIV-related maternal deaths were estimated for all locations using the PAF approach described above for mortality data processing.

ICD10 and ICD9 codes used for maternal disorders

Model	ICD10 code	ICD9 code
Abortion, ectopic pregnancy, miscarriage	O00-O08, O36.4	631, 633-639
Maternal hemorrhage	O20, O43.2, O44-O46, O62.2, O67, O72	640-641, 661.0, 666
Hypertensive disorders of pregnancy	O11-O16	642.3, 642.4, 642.5, 642.6, 642.7, 642.9
Obstructed labor and uterine rupture	O64-O66, O71, O83	659-660, 662, 665, 669.5, 669.6
Maternal sepsis and other infections	O23, O41, O75.2-3, O85, O86, O91	646.5, 646.6, 659.2, 659.3, 670, 672.0, 674.1, 674.2, 674.3, 675
Other maternal disorders	O09-O09.93, O21-O22-O22.93, O26-26.93, O28-O28.9, O29-O29.93, O30-O35.9, O40-O43.93, O47-48.1, O60-O61.9, O63-O63.9, O68-O70.9, O73-O77.9, O80-O84, O87-O90.9, O92-O92.79	646-646.44, 646.7-646.93, 648.1-649.9
Indirect maternal disorders	O24-O25.3, O98-O99.91	647-649.64

DisMod Proportion Models Covariates and Coefficients

Abortion, ectopic pregnancy and miscarriage

Study-level covariate	Parameter	Geography level	beta	Exponentiated beta
Only Maternal Direct Causes	Proportion	Global	0.20 (0.19 — 0.20	1.22 (1.21 — 1.22)

Hospital Inpatient	Proportion	Global	0.30 (0.30 — 0.30)	1.35 (1.35 — 1.35)
Late maternal deaths not included	Proportion	Global	- 0.20 (0.20 — 0.20)	1.22 (1.22 — 1.22)
Country-Level Covariate				
Legality of Abortion	Proportion	Global	0.054 (0.054 — 0.055)	1.06 (1.06 — 1.06)

Maternal hemorrhage

Study-level covariate	Parameter	Geography level	beta	Exponentiated beta
Only Maternal Direct Causes	Proportion	Global	0.20 (0.18 — 0.20)	1.22 (1.20 — 1.22)
Hospital Inpatient	Proportion	Global	0.30 (0.29 — 0.30)	1.35 (1.34 — 1.35)
Late maternal deaths not included	Proportion	Global	0.20 (0.20 — 0.20)	1.22 (1.22 — 1.22)
Country-level covariate				
In-Facility Delivery (proportion)	Proportion	Global	0.100 (0.100 — 0.100)	1.11 (1.10 — 1.11)

Hypertensive disorders of pregnancy

Study-level covariate	Parameter	Geography level	beta	Exponentiated beta
Only Maternal Direct Causes	Proportion	Global	0.20 (0.19 — 0.20)	1.22 (1.21 — 1.22)
Hospital Inpatient	Proportion	Global	0.30 (0.30 — 0.30)	1.35 (1.34 — 1.35)
Late maternal deaths not included	Proportion	Global	0.20 (0.20 — 0.20)	1.22 (1.22 — 1.22)
Country-level covariate				
In-Facility Delivery (proportion)	Proportion	Global	0.100 (0.100 — 0.100)	1.11 (1.11 — 1.11)

Obstructed labor and uterine rupture

Study-level covariate	Parameter	Geography level	beta	Exponentiated beta
Only Maternal Direct Causes	Proportion	Global	0.20 (0.20 — 0.20)	1.22 (1.22 — 1.22)
Hospital Inpatient	Proportion	Global	0.30 (0.30 — 0.30)	1.35 (1.35 — 1.35)

Late maternal deaths not included	Proportion	Global	0.20 (0.20 — 0.20)	1.22 (1.22 — 1.22)
Country-level covariate				
In-Facility Delivery (proportion)	Proportion	Global	0.100 (0.100 — 0.100)	1.11 (1.11 — 1.11)

Maternal sepsis and other infections

Study-level covariate	Parameter	Geography level	beta	Exponentiated beta
Only Maternal Direct Causes	Proportion	Global	0.20 (0.20 — 0.20)	1.22 (1.22 — 1.22)
Hospital Inpatient	Proportion	Global	0.30 (0.30 — 0.30)	1.35 (1.35 — 1.35)
Late maternal deaths not included	Proportion	Global	0.20 (0.20 — 0.20)	1.22 (1.22 — 1.22)
Country-level covariates				
LDI ($ per capita)	Proportion	Global	0.100 (0.100 — 0.100)	1.11 (1.10 — 1.11)

Other Maternal Disorders

Study-level covariate	Parameter	Geography level	beta	Exponentiated beta
Only Maternal Direct Causes	Proportion	Global	0.20 (0.19 — 0.20)	1.22 (1.21 — 1.22)
Hospital Inpatient	Proportion	Global	0.20 (0.19 — 0.20)	1.22 (1.21 — 1.22)
Late maternal deaths not included	Proportion	Global	0.20 (0.20 — 0.20)	1.22 (1.22 — 1.22)
Country-level covariate				
LDI ($ per capita)	Proportion	Global	0.100 (0.100 — 0.100)	1.11 (1.10 — 1.11)

Indirect Maternal Disorders

Study-level covariate	Parameter	Geography level	beta	Exponentiated beta
Hospital Inpatient	Proportion	Global	0.20 (0.20 — 0.30)	1.22 (1.22 — 1.22)
Late maternal deaths not included	Proportion	Global	0.20 (0.20 — 0.20)	1.22 (1.22 — 1.22)
Country-level covariate				

LDI ($ per capita)	Proportion	Global	0.100 (0.100 — 0.100)	1.11 (1.11 — 1.11)

3.1.2, 3.8.1 Skilled Birth Attendance SDG Capstone Appendix

Flowchart

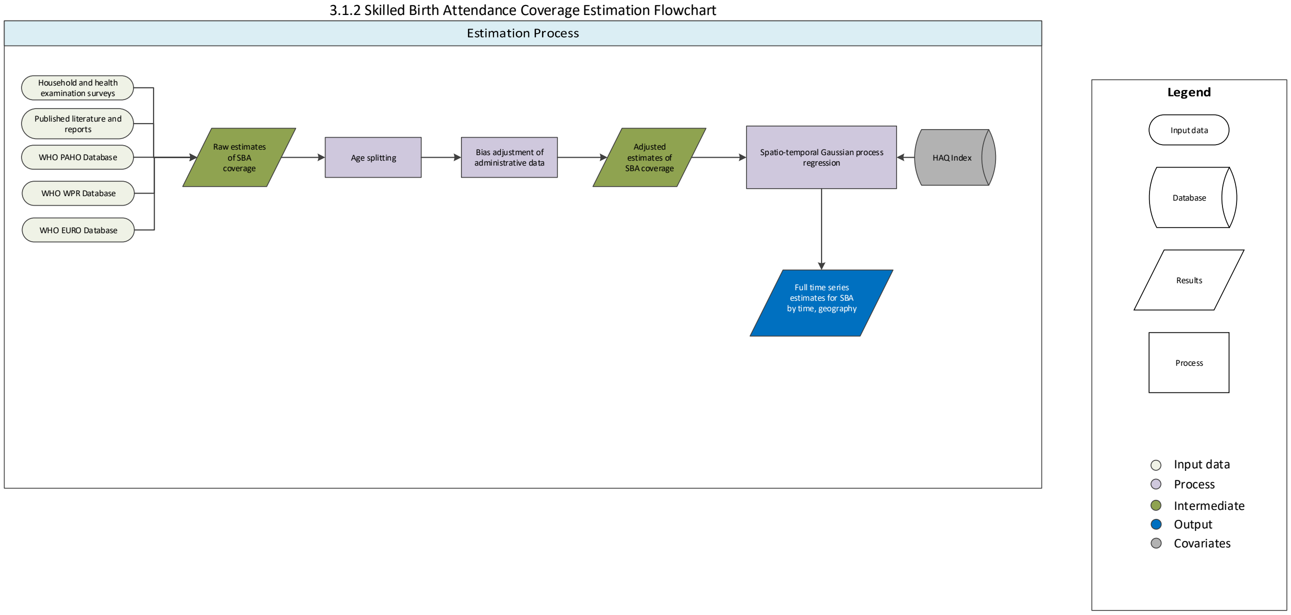

Input data & Methodological summary

Indicator definition

This modeling strategy pertains to the indicator associated with skilled birth attendance (SBA) (SDG indicator 3.1.2), which is also included in the universal health coverage (UHC) index (SDG indicator 3.8.1).

Indicator 3.1.2

As a component of SDG Goal 3. Ensure healthy lives and promote well-being for all at all ages, SDG Target 3.1, by 2030, reduce the global maternal mortality ratio to less than 70 per 100,000 live births, is measured by SDG indicator 3.1.2, proportion of births attended by skilled health personnel (doctors, nurses or midwives). Note that SBA is included in the UHC index (SDG indicator 3.81).

Input data

For the present analysis, we used individual-level microdata from population health surveys and tabulated survey report data on skilled birth attendance (SBA). As defined by the World Health Organization (WHO), SBA reflects the proportion of births in a given year where a doctor, nurse, or midwife was present.[1]

Survey data which provided individual-level data, and specifically among female respondents, were identified and extracted. Major multi-country survey programs included in the analysis include the Demographic and Health Surveys (DHS),[1] Multiple Indicator Cluster Surveys (MICS),[2] Reproductive Health Surveys (RHS),[3] Living Standards Measurement Study (LSMS) surveys,[4] and World Health Surveys (WHS).[5] We also conducted a comprehensive search of the Global Health Data Exchange (GHDx),[6] as well as

targeted internet searches and review of Ministry of Health websites, to identify national surveys and other multi-country survey programs. In addition, we utilized tabulated report data from regional WHO databases, when available, including the PAHO[7], WHO WPR[8], and the WHO European Health for All databases[9].

We excluded all data sources that were not nationally representative or had high levels of missingness. We applied survey weights based on survey sampling frames whenever they were available to generate weighted national estimates of SBA coverage accompanied by estimates of standard error (SE). Estimates of SE, as well as sample sizes, were used to calculate uncertainty, as described below. Any point estimates with sample sizes less than 50 were reviewed to ensure that they were not substantive outliers and would otherwise have an undue influence on our analysis.

Due to potential bias in recall, we limited our analysis to women who gave birth up to five years prior to the time of survey; due to data limitations, we used a limit of up to two years for some surveys. We also had to standardize the definition of "skilled health professional" across countries, which varied by differences in quality of training or health professional roles. For this analysis, doctors, nurses, and midwives were included as our foundational definition for SBA, and we extended this to include country-specific medical staff based on the number of years of training they received and/or their comparable ability to intervene in an emergency situation (eg, clinical officers). Care received during delivery by traditional health personnel was not considered a birth overseen by a skilled attendant.

Modeling strategy

Data processing

Age splitting

Most household surveys collect information on maternal and child health (MCH) indicators for children under 5 and/or mothers who gave birth within five years prior to the time of survey. To maximize data use for our model, we included SBA information for children aged 12 to 59 months at the time of survey. Children younger than 12 months of age were excluded to minimize the influence of potentially censored observations. SBA coverage estimates were assigned to birth-cohort years based on a child's age prior to the time of survey: we used responses recorded for children aged 12 to 23 months for SBA coverage for one year prior to the time of survey, children aged 24 to 35 months for coverage two years prior to the time of survey, and so forth.

Age-specific estimates are easily computed from individual-level microdata, but many published reports and survey summaries present data in broader age aggregates (eg, SBA coverage for children aged 12 to 35 months). To standardize these age groups, we applied an age-splitting model used in the GBD study,[10] as well as analyses that generated smoking and obesity prevalence by age group.[11,12]

Using surveys with microdata as the reference, we used the following model to generate standardized age group-specific estimates for SBA:

$$\tilde{P}_{a,c,t,k} = P_{a,c,t,k}^{a+x} \frac{P_{a,c,t,j}}{P_{a,c,t,j}^{a+x}}$$

where $\tilde{P}_{a,c,k}$ is the adjusted estimate of coverage for target age group a in country c and year t of survey k; and $P_{a,c,k}^{a+x}$ is coverage reported from survey k, for country c in year t for the age group spanning age a to age $(a + x)$. The ratio of coverage between the target age group and broader age group from a survey j with microdata from the same country-year was used to split data from survey k. Surveys to be split were ideally matched with DHS or MICS surveys. If microdata were not available for the same year, ratios within five years of the survey that required age-splitting were applied.

Bias adjustments

Intervention coverage estimates based on administrative sources can be biased, yet the direction and magnitude of such biases are not universal. Some studies show that coverage estimates from administrative data source are systematically higher than those of survey-based estimates,[13] while other studies show that bias directionality is more heterogeneous.[14] Such biases may arise for a number of reasons, including discrepancies in the accurate reporting of services or interventions provided (eg, number of skilled attendants) and target population (eg, number of children born), as well as capturing these data in a timely manner from both public and private sector facilities and healthcare providers.

For SBA, we view individual-level data collected through population health surveys as the most accurate and least biased source of information, particularly for geographies with incomplete health information systems. We thus used SBA coverage estimates from household surveys to calculate country-specific adjustment factors:

$$logit(P_{s,\,c,t}) = \beta_0 + \beta_1 logit(\tilde{P}_{a,\,c,t}) + \sum_{k=2}^{2+B} \beta_k S_k + \varepsilon_{c,t}$$

where $P_{s,\,c,t}$ is the survey-based estimate for SBA coverage (s) in country c for year t; $\tilde{P}_{a,\,c,t}$ is the administrative estimate for coverage in country c in year t; S_k is a spline basis used to capture the secular trend in coverage; β_1 is the estimated adjustment factor used to correct for the administrative bias; and ε is the error term for country c in year t.

To quantify uncertainty for bias-adjusted estimates from the mixed-effects models described above, we calculated prediction error, $\widehat{PE}$, as follows:

$$\widehat{PE} = X^2 var(\hat{\beta})$$

where $var(\hat{\beta})$ is the variance for the estimated fixed-effects coefficient of the adjustment factor and X is the independent variable. Proper estimation of prediction errors is crucial as the data synthesis procedure, Gaussian process regression (GPR) (as described in the subsequent section), accounts for uncertainty from point estimates and bias adjustments when generating fitted values. More weight is given to data with less uncertainty. Prediction errors estimated from the bias adjustment were incorporated into the data variance and propagated through the GPR step to obtain estimates of SBA coverage and uncertainty intervals (UIs).

To assess the accuracy of our estimates in the bias adjustment, we performed cross-validation analyses by randomly holding out 20% of the sample and, if available, the corresponding administrative estimates for the given indicator of the same country and year, 10 separate times. We computed the average root

mean squared errors (RMSE) across each country. Error in the bias adjustments was calculated as the mean difference between the adjusted administrative estimate for a given country, year, and corresponding survey-level estimates (which were considered the "gold-standard").

Trend estimation

We used a spatiotemporal Gaussian process regression (ST-GPR) to synthesize point estimates from multiple data sources and derive a complete time series for SBA coverage. This method has been used extensively in GBD and related studies, and accounts for uncertainty pertaining to each point estimate while borrowing strength across geographic space and time.[10, 11,15,16] Briefly, we assumed the Gaussian process was defined by a mean function *m*(•) and covariance function *Cov*(•).

We estimated the mean function using a two-step approach. Specifically, $m_c(t)$ can be expressed as:

$$m_c(t) = X\beta + h(r_{c,t})$$

where $X\beta$ is a linear model and $h(r_{c,t})$ is a smoothing function for the residuals; and $r_{c,t}$ is derived from the linear model. The following linear model was used for estimating SBA:

$$logit(P_{c,t}) = \beta_0 + \beta_1 \mathrm{HAQ_{c,t}} + \alpha_c + \gamma_{R[c]} + \omega_{\mathrm{SR[c]}} + \varepsilon_{c,t}$$

where $P_{c,t}$ is SBA coverage for country c year t; $HAQ_{c,t}$ is value of the Healthcare Access and Quality Index[16] for country c and year t; α_c, $\gamma_{R[c]}$, and $\omega_{\mathrm{SR[c]}}$ are country, region, and super-region random intercepts, respectively. These estimates were then modeled through ST-GPR.

Random draws of 1,000 samples were obtained from the distributions above for every country for a given vaccine. Ninety-five percent uncertainty intervals were calculated by taking the ordinal 25th and 975th draws from the sample distribution.

To assess the accuracy of our modeled estimates, we performed cross-validation analyses using a knockout structure as previously described[17]. ST-GPR hyperparameters were selected on models that minimized the overall root-mean squared error (RMSE) of the model across a set of 10 knockouts.

References

1 Measure DHS: Demographic and Health Surveys. http://www.measuredhs.com (accessed Aug 11, 2015).

2 UNICEF Stat. Monit. Multiple Indicator Cluster Survey (MICS). http://www.unicef.org/statistics/index_24302.html (accessed Aug 11, 2015).

3 Cent. Dis. Control Prev. Reproductive Health Surveys (RHS). http://www.cdc.gov/reproductivehealth/Global/surveys.htm (accessed Aug 11, 2015).

4 World Bank. Living Standard Measruement Studies (LSMS). http://go.worldbank.org/UK1ETMHBN0 (accessed Aug 11, 2015).

5 WHO Multi-Ctry. Stud. Data Arch. World Health Survey (WHS). http://apps.who.int/healthinfo/systems/surveydata/index.php/catalog/whs/about (accessed Aug 11, 2015).

6 IHME GHDx. Global Helath Data Exchange. http://ghdx.healthdata.org/ (accessed Aug 11, 2015).

7 PAHO/WHO Data - Home. http://www.paho.org/data/index.php/en/ (accessed June 12, 2017).

8 WPR - Health Information & Intelligence Platform > Data & analytics. http://hiip.wpro.who.int/portal/Dataanalytics.aspx (accessed June 12, 2017).

9 European Health for All Family of Databases (HFA-DB). 2017; published online June 12. http://www.euro.who.int/en/data-and-evidence/databases/european-health-for-all-family-of-databases-hfa-db (accessed June 12, 2017).

10 Prospective Studies Collaboration. Age-specific relevance of usual blood pressure to vascular mortality: a meta-analysis of individual data for one million adults in 61 prospective studies. *The Lancet* 2002; **360**: 1903–13.

11 Ng M, Freeman MK, Fleming TD, *et al.* Smoking Prevalence and Cigarette Consumption in 187 Countries, 1980-2012. *JAMA* 2014; **311**: 183.

12 Ng M, Fleming T, Robinson M, *et al.* Global, regional, and national prevalence of overweight and obesity in children and adults during 1980–2013: a systematic analysis for the Global Burden of Disease Study 2013. *The Lancet* 2014; **384**: 766–81.

13 Murray CJ, Shengelia B, Gupta N, Moussavi S, Tandon A, Thieren M. Validity of reported vaccination coverage in 45 countries. *The Lancet* 2003; **362**: 1022–7.

14 Haddad S, Bicaba A, Feletto M, Fournier P, Zunzunegui MV. Heterogeneity in the validity of administrative-based estimates of immunization coverage across health districts in Burkina Faso: implications for measurement, monitoring and planning. *Health Policy Plan* 2010; **25**: 393–405.

15 Wang H, Liddell CA, Coates MM, *et al.* Global, regional, and national levels of neonatal, infant, and under-5 mortality during 1990–2013: a systematic analysis for the Global Burden of Disease Study 2013. *The Lancet* 13; **384**: 957–79.

16 Barber RM, Fullman N, Sorensen RJD, *et al.* Healthcare Access and Quality Index based on mortality from causes amenable to personal health care in 195 countries and territories, 1990?2015: a novel analysis from the Global Burden of Disease Study 2015. *The Lancet* 2017; published online May. DOI:10.1016/S0140-6736(17)30818-8.

17 Foreman KJ, Lozano R, Lopez AD, Murray CJ. Modeling causes of death: an integrated approach using CODEm. *Popul Health Metr* 2012; **10**: 1.

Under-5 Mortality and Neonatal Mortality SDG Capstone Appendix

Flowchart

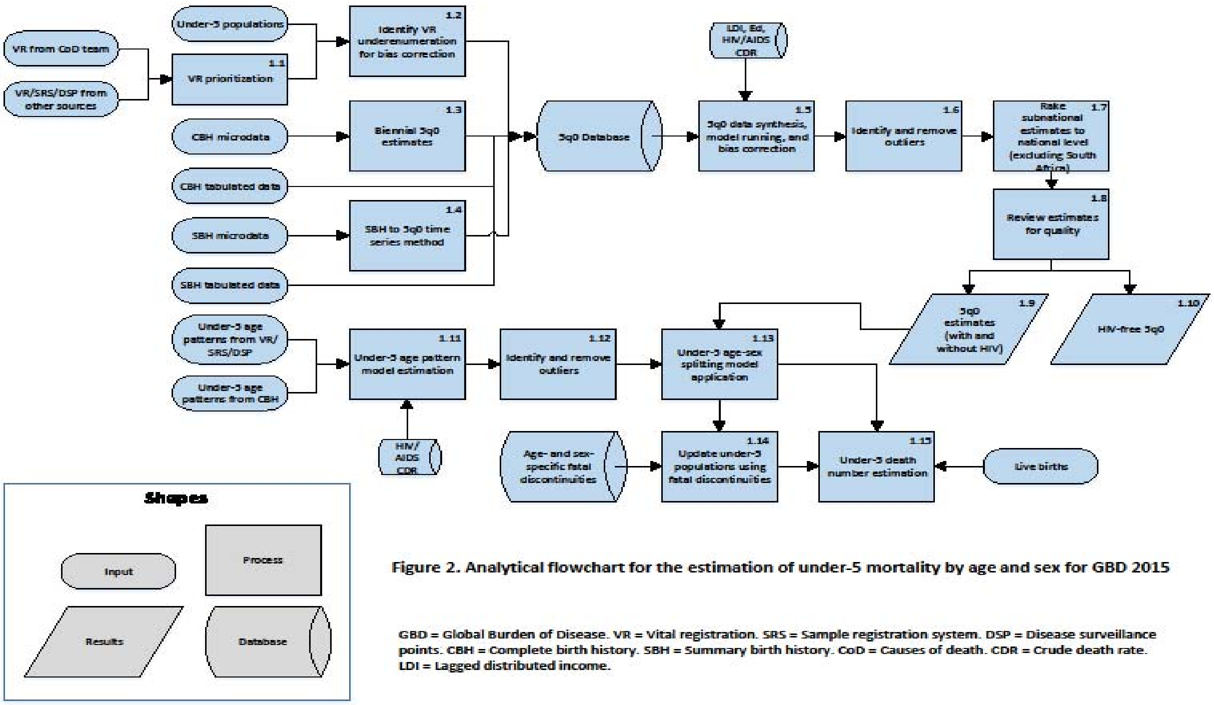

Figure 2. Analytical flowchart for the estimation of under-5 mortality by age and sex for GBD 2015

GBD = Global Burden of Disease. VR = Vital registration. SRS = Sample registration system. DSP = Disease surveillance points. CBH = Complete birth history. SBH = Summary birth history. CoD = Causes of death. CDR = Crude death rate. LDI = Lagged distributed income.

Input Data & Methodological Summary

Indicator definition

This modeling strategy encompassed the indicator associated with under-5 mortality (3.2.1) and neonatal mortality (3.2.2)

Indicator 3.2.1

As a component of SDG Goal 3. Ensure healthy lives and promote well-being for all at all ages, SDG Target 3.2, by 2030, end preventable deaths of newborns and children under 5 years of age, with all countries aiming to reduce neonatal mortality to at least as low as 12 per 1000 live births and under-5 mortality to at least as low as 25 per 1,000 live births, is measured using SDG Indicator 3.2.1, under-5 mortality rate (probability of dying before the age of 5 per 1,000 live births).

Indicator 3.2.2

As a component of SDG Goal 3. Ensure healthy lives and promote well-being for all at all ages, SDG Target 3.2, by 2030, end preventable deaths of newborns and children under 5 years of age, with all countries aiming to reduce neonatal mortality to at least as low as 12 per 1000 live births and under-5 mortality to at least as low as 25 per 1,000 live births, is measured using SDG Indicator 3.2.2, neonatal mortality rate (probability of dying during the first 28 days of life per 1,000 live births).

Input data

Vital registration from Causes of Death team

Approximately 62% of deaths data from vital registration (VR) systems used as input for our all-cause mortality modeling were provided by the GBD causes of death (CoD) research team and were aggregated into total age-sex-specific all-cause mortality for each location-year. This aggregation occurred after the data were adjusted and mapped to the GBD cause list.

Data intended for use in causes of death modeling are assessed for quality with respect to consistency of cause fractions, diagnostic accuracy and missing data, whereas for all-cause mortality modeling it is more important that data are fully representative of the given estimation area and are consistent with other all-cause mortality data sources. Thus, there are cases in which VR data prepared for cause-specific modeling cannot be used in all-cause modeling or must be adjusted based on degree of completeness before being used.

In our vetting of CoD VR data, we dropped points where there was a larger than 1% difference from corresponding points in the WHO database. There were instances where VR data used in cause-specific mortality analysis had been collapsed to Basic Tabulation List (BTL) format rather than in full cause classification list format (e.g., ICD-9). In some of these cases, we elected to use WHO data instead.

Vital registration, sample registration systems, and Disease Surveillance Points from other sources

We endeavored to include all available data from vital registration systems as inputs in our all-cause mortality estimation process. To achieve this, we utilized a number of multi-country vital registration sources, including the WHO Mortality Database, the Human Mortality Database, United Nations Demographic Yearbooks and OECD databases. These multi-country sources are regularly updated in our systems when new data are added. Beyond multi-country sources, for all ongoing national VR systems (for example, the USA National Vital Statistics System), where possible, we cataloged all data sources from each system.

Some countries that do not have a well-performing VR system implement sample registration systems that are incomplete by design. We made use of these data, paying close attention to the proper weighting of sampled data and consistency with other representative sources. We have systematically extracted data from the Sample Registration System Statistical Report series published by the Registrar General of India. For the Disease Surveillance Points system of China, we obtained both national and provincial level DSP data through a data usage agreement with the Chinese Center for Disease Control and Prevention. Census data are systematically extracted from Demographic Yearbook series, Integrated Public Use Microdata Series (IPUMS), and statistical reports from the national statistical bureaus.

Under-5 populations and live births

For most GBD locations, live births come from the World Population Prospects 2015 (WPP 2015). For subnational locations, we often use interpolated census birth numbers scaled to the national estimates. For locations not estimated in the WPP 2015, we use interpolated census birth counts.

Complete birth history microdata

Complete birth histories, the preferred method for data collection on child mortality in the absence of vital registration, rely on administering surveys to mothers. The questionnaires ask about all living and deceased children, including date of birth, survival status, and date of death. These modules are included in many routine survey series, including the World Fertility Surveys (WFS), Demographic and Health Surveys (DHS), Multiple Indicator Cluster Surveys (MICS), and many national survey programs. When available, we download and use microdata that has individual-level survey responses as opposed to using tabulated results.

Complete birth history tabulated data

In some instances, tabulated records from reports become available before survey microdata, and we incorporate these data points into our database of 5q0 data as well. However, as microdata become available, we update with point estimates from our processed microdata rather than the tabulated report estimates.

Summary birth history microdata

Summary birth history questionnaires are a shorter alternative to complete birth histories. Instead of asking in detail about each child, summary birth histories simply ask mothers how many children they have given birth to and how many of the children have died. The questionnaires are shorter and can be more easily attached to other surveys. Often, Censuses and MICS surveys contain summary birth histories. For GBD, we have complied all available SBH data with micro-level data that enables us to apply the updated Summary birth history method that leads to more accurate and timely assessment of U5MR.[1]

Summary birth history tabulated data

In cases where we don't have access to the micro-level data on summary birth history modules from surveys and censuses, we utilize the reported estimates of U5MR from survey or census reports and outlier the first two data points based on mothers in ages 15-19 and 20-24. In cases where tabulated proportions of child died by mother's age group are available, we apply the Maternal Age Cohort method as updated by Rajaratnam and colleagues.[1]

Under-5 age-sex patterns from VR/SRS/DSP

Vital registration systems are the primary source of data for the under-5 age pattern of mortality in developed countries. Often, these data group under-5 deaths into several age groups- Early Neonatal (0-6 days), Late Neonatal (7-27 days), Post Neonatal (28-364 days), and 1 to 4 years. Some country-years of data have other age groupings with less specificity, with the Early and Late Neonatal age groups combined, or all of the under-1 age groups combined. Sample Registration Systems (SRS) also provide data for the age-sex pattern of under-5 mortality in several countries (notably India and Bangladesh), as well as the Disease Surveillance Points (DSP) system in China.

Under-5 age-sex patterns from complete birth history

In many countries without vital registration systems, complete birth history surveys can be used to obtain age-sex patterns of mortality in under-5 age groups. These sources are described above in the "Complete birth history microdata" section. For all complete birth history microdata sources, we apply direct estimation methods to obtain probabilities of death for each of the under-5 age groups. Within each survey, if each observation is a child recalled by a mother, observations are grouped into 5-year groups in time to provide a data point of probability of death for each of the under-5 age-sex groups. Recall is cut off 15 years before the survey, limiting data points estimated from the survey to the 15 years prior. All of these estimates are then in the database of estimates for the age-sex pattern of under-5 mortality.

Modeling Strategy

VR prioritization

Our continual evaluation of VR data sources has led us to develop a general hierarchy of preferred VR sources. When considering which of multiple sources to use for a given location-year, we first prefer to use WHO data from GBD cause-specific mortality estimation, then unadjusted WHO data, then Human Mortality Database (HMD) data, then UN Demographic Yearbook data. There were exceptions to this hierarchy where we had reason to believe that there were quality issues with a certain source. For instance, where available we preferred to use HMD VR over WHO data for Germany, Taiwan, and Spain deaths. Single-country VR sources were evaluated based on consistency with other data sources and also VR system documentation.

Identify VR under-enumeration for bias correction

The approach to estimating the completeness of vital registration systems for deaths under age 5 is the same as that of the previous two GBD studies.

In many countries with vital registration (VR) systems to record deaths, complete and/or summary birth histories are also conducted. By comparing the under-5 death rates from these sources to the levels from VR or sample registration systems, we can assess the completeness of under-5 death registration. Completeness can evolve over time as seen with the likely declines in completeness in Central Asia in the 1990s or the increases in completeness in other settings.[2,3] We estimate VR completeness where VR data are available using a model that allows for completeness to vary over time. This assessment is undertaken in two steps: we first assess whether VR is biased, and then we assess time-varying completeness.

In the first step, a country-level regression of $\log_{10}({}_5q_0)$ on year with a binary indicator variable for ${}_5q_0$ estimates derived from VR systems is used to determine whether or not a VR system is biased (see equation). If the coefficient for the VR indicator variable is statistically significant at the .05 α-level, we deem the VR system to be biased.

$$log_{10}({}_5q_0)_t = \alpha + \beta_1 * t + \beta_2 * I_{VR} + \xi_t$$

Where: t is time (a continuous variable);

I_{VR} is an indicator for ${}_5q_0$ estimates derived from VR systems; and

ξ_t is in error term.

Second, for all countries with biased VR systems, we estimate the bias, allowing for completeness to evolve over time. We first apply Loess regression to all non-VR ${}_5q_0$ estimates in a given country; we then calculate the difference between the Loess predicted $\log_{10}({}_5q_0)$ and the observed $\log_{10}({}_5q_0)$ estimate from VR in a given year. Since we believe that completeness changes relatively slowly over time, the bias in any given year is defined as the mean difference between the predicted $\log_{10}({}_5q_0)$ and the observed $\log_{10}({}_5q_0)$ from VR systems over the adjacent five-year period. This allows for flexibility in the bias correction over time while still maintaining the premise that the completeness of VR systems does not change abruptly. Loess predictions can be unreliable out of sample, so for country-years outside of the range of non-VR data used to generate the predicted ${}_5q_0$, we use the mean bias from the nearest five years of bias estimates from VR points that are within the timespan of the non-VR data. We then correct the VR estimates of ${}_5q_0$ using the bias correction as shown in the equation:

$${}_5q_0^{corr.} = {}_5q_0^{obs.} * 10^{\widehat{bias}}$$

Where: ${}_5q_0^{corr.}$ is the corrected estimate of ${}_5q_0$;
${}_5q_0^{obs.}$ is the observed estimate of ${}_5q_0$; and
$\widehat{bias}$ is the bias estimate described above.

Once the biased estimates have been adjusted, we also approximate the variance of the bias estimate. This variance is approximated using the median absolute deviation (MAD) comparing the biased VR estimates to the Loess-based estimate of $\log_{10}({}_5q_0)$. As with the bias estimation above, the MAD is estimated over a five-year time period. This MAD times 1.4826 is an approximation of standard deviation used to add variance to the biased VR data when included in our final Gaussian Process Regression (GPR) model described in detail later.

In addition to countries where there are both VR estimates and survey estimates of under-5 mortality, there are countries for which only VR data are available, and the VR systems are considered biased. This is a problem particularly in English-speaking Caribbean countries, so for these countries we have adjusted ${}_5q_0$ estimates from VR using the regional average VR bias in a given year for those countries with both VR and survey ${}_5q_0$ estimates. The countries for which VR systems have been adjusted using this method include Antigua and Barbuda, Bahamas, Barbados, Bermuda, Dominica, Grenada, Saint Lucia, and Saint Vincent and the Grenadines. While there is no direct evidence on the level of VR bias in these countries, assuming they are complete when similar countries in the region have under-registration seems unwarranted.

Biennial 5q0 estimates

Complete birth history 5q0 computation

Microdata (individual-level survey data) from complete birth histories yield direct calculation of death numbers and probabilities of death in the under-5 age group. Observations are grouped into two-year intervals such that biennial estimates of 5q0 are obtained from these survey data. In GBD 2015, we have unpooled surveys for our analysis, whereas surveys were pooled by series in GBD 2013. Instead of grouping observations from all DHS complete birth history questionnaires from a country into one full set of observations and all MICS observations from multiple survey years into another full set of observations, we analyzed each survey separately (e.g., DHS 2012, DHS 1996, MICS 2002). This allowed for greater ability to address known data quality issues in specific surveys. To compensate for the decreased sample size and to generate greater stability in the unpooled data points, we created 2-year estimates of under-5 mortality, pooling observations over 2-year periods instead of single years.

Tabular complete birth history processing

In some instances, microdata from surveys were not available. If survey reports could be obtained but the microdata were not available for us to do our own calculations to obtain 5q0, we used report data point estimates. These estimates were added directly to the under-5 mortality database.

Summary birth history time series method

Summary birth history method from microdata

Rajaratnam and colleagues have developed an updated summary birth history method that is able to provide more accurate and timely estimates of U5MR from micro data on SBH from surveys and censuses.[1]

Summary birth history analysis from tabular data

When only tabular data are available for the numbers of children ever born and number of children that have died by mother's age, we apply the Maternal Age Cohort model from the method developed by Rajaratnam and colleagues.[1]

5q0 data synthesis, model running, and bias correction

Data synthesis using ST-GPR and bias correction

We apply the child mortality estimation methodology as reported by Wang et al.[4] Based on the under-five mortality data synthesis model for the Global Burden of Disease Study 2010 and 2013, we have incorporated data bias adjustment into the modeling process. Specifically, we have included a fixed effect for source type across all locations to detect systematic differences in the level of child mortality, controlling for covariates for one source type versus another. The groups of sources to make this adjustment are listed in the table below. In addition, we include a random effect for each country-source. By choosing a reference source country-by-country or using the mean of a set of sources, we can adjust on a country-by-country basis for the problem of compositional bias created by substantial source-specific non-sampling error. Once the systematic difference in sources is removed, we are able to avoid estimating false trends due to partial overlap of sources with different levels of non-sampling variance. We then apply the combination of non-linear mixed effects model, spatial-temporal regression and Gaussian process regression to synthesize raw child mortality data after data bias adjustment to obtain consistent time series estimates of mortality with 95% uncertainty intervals for every country.

Table: Source types used in child mortality bias correction

Data Source Type
Complete Birth History-Demographic and Health Survey
Complete Birth History-AIDS Indicator Survey and Malaria Indicators Survey
Complete Birth History-World Fertility Survey
Complete Birth History-Multiple Indicator Cluster Survey
Complete Birth History-Census
Complete Birth History-Other survey Series
Summary Birth History-Demographic and Health Survey
Summary Birth History-Multiple Indicator Cluster Survey
Summary Birth History-Other survey series
Summary Birth History-AIDS Indicator Survey and Malaria Indicators Survey
Summary Birth History-Census
Summary Birth History-World Fertility Survey
Vital Registration/Sample Registration/Surveillance- complete
Vital Registration/Sample Registration/Surveillance- incomplete
Household Death Recall-Other survey series
Household Death Recall-Census
Household Death Recall – incomplete Vital Registration/Sample Registration/Surveillance

Mixed effect non-linear model and the bias adjustment for raw U5MR sources

In this stage, we used a nonlinear mixed effects regression to estimate data bias and provide first stage predictions.

The nonlinear mixed effects regression model is

$$ {}_5m_{0cys} = \exp[(\beta_1 + \gamma_{1c}) * \log\left(LDI_{cy}\right) + (\beta_2 + \gamma_{2c}) * education_{cy} + \gamma_c + \gamma_{cs} + \alpha_t] + \beta_3 * HIV_{cy} + \varepsilon_{cys} $$

where *c* is country, *y* is year, *s* is source, and *t* is source type; each source was categorized into one of 17 source types across all countries, as listed in the table above.

Additionally,

- ${}_5m_0$ is under five mortality rate
- *LDI* is lagged distributed income per capita
- *education* is mean years of education for women of reproductive age (15-49 years)
- *HIV* is death rate due to HIV in age groups 0-4
- γ is a random effect
- α is a fixed effect on source type across countries
- β_i is a fixed covariate coefficient
- ε is the residual

For each country, we rely on expert opinion to choose a source, or combination of sources, which are believed to be the least biased. If a country has vital registration which we deem to be complete

(described in detail in an earlier section), this is the reference source. If a country does not have complete vital registration, but has DHS estimates from complete birth histories, these were chosen as the reference source. If a country has neither of these types of data or DHS surveys are deemed unreliable, we assigned the surveys conducted after 1980, in combination, as the reference (incomplete vital registration data were not included). Additionally, in many countries we chose other surveys as the reference. For accurate estimation, it is important to have local knowledge on specific data sources' accuracy. All-cause mortality experts draw from their familiarity with data quality to help us to choose the reference category.

Each data source has an associated random effect as well as a source type fixed effect. The values of these random and fixed effects for the reference sources are deemed to be the true deviation from unbiased mortality level. In countries with multiple high-quality sources, the mean of the random and fixed effects from these sources is taken as this true deviation. We adjusted all other sources by including these reference values for the random and fixed effects values instead of those estimated for each individual source, as shown below.

$$adjusted_5m_{0,cys} = \exp[(\beta_1 + \gamma_{1c}) * \log(LDI_{cy}) + (\beta_2 + \gamma_{2c}) * education_{cy} + \gamma_c + \gamma_{ref,c} + \alpha_{ref,c}] + (\beta_3 + \gamma_{3c}) * HIV_{cy} + \varepsilon_{cys}$$

The exception to this correction is incomplete vital registration data, which was adjusted upwards using a five year rolling mean of the difference between incomplete vital regression and a Loess of the already-adjusted survey data, described above in section 1.2.

Spatio-temporal smoothing

The spatio-temporal stage smooths the residuals between the predicted time series of $_5q_0$ and the adjusted raw data over time and across countries in the same GBD region. The predicted time series for this smoother was obtained from the equation below; no random effects or survey type fixed effects are included.

$$predicted_5m_{0,cy} = \exp[\beta_1 * \log(LDI_{cy}) + \beta_2 * education_{cy} + \alpha_{intercept}] + \beta_3 * HIV_{cy}$$

We first found the residuals between the predicted time series, above, and the adjusted points. We then applied a combination of smoothing functions to these residuals. For each country year, we weighted all the data points in this region based on their proximity to this country-year in space and time. We gave 99% of the weight to in-country residuals, and 1% of the weight to out-of-country residuals. Additionally, we used a modified tricubic window, as specified below, to give more weight to points closer in time, and less weight to points further in time.

$$w_t = \left(1 - \left(\frac{|r_t - r_{est}|}{1 + \mathrm{argmax}_t|r_t - r_{est}|}\right)^{\lambda}\right)^3$$

The r_t and r_{est} terms are, respectively, the year of interest and the year of the residual being weighted. The $\mathrm{argmax}_t|r_t - r_{est}|$ term is the maximum distance between the year of interest and a residual within the region. The λ parameter in this weighting function dictates how quickly the weights fall off as the distance in time increases: a larger λ implies that the assigned weights will diminish slowly with time, while a smaller λ allows the weights to diminish more rapidly with time.

For most countries, we chose λ = 0.8. We then created one estimate of the smoothed residuals using a linear fit to this weighted data; this is similar to a Loess fit. Additionally, we created a second estimate of the smoothed residuals by calculating the weighted average of this data.

We then combined these two estimates for a final estimate of the smoothed residuals. In data-dense countries, more weight was given to the local linear fit; in data sparse countries, more weight was given to the weighted average. The equation for this is as follows.

$$final\ smoothed\ residual = k * linear\ estimate + (1-k) * weighted\ average$$

where $k = \frac{number\ of\ in\ country\ data\ points}{number\ of\ in\ country\ data\ points + number\ of\ country\ years\ with\ no\ data}$

Finally, the smoothed residuals were added back to the predictions from above; this smoothed approximation to the adjusted data was used as the prior for the Gaussian process regression, described below.

Third stage: Gaussian process regression (GPR)

The output of the space-time local smoother was used as a prior for the Gaussian process regression, which produced a final time series of point estimates, as well as confidence bounds. Parameters for the GPR were chosen through cross-validation described in section 1.5.E.

The model for the Gaussian process regression is shown below, where μ_t is the true $\log_{10}({}_5q_0)$ at time t, f(t) is the baseline mortality risk, and S_t captures excess mortality due to war and disasters. S_t is estimated independently of f(t). M and C describe the Gaussian process, giving the mean and covariance, respectively.

$$\mu_t = f(t) + S_t$$
$$f(t) \sim GP(M, C)$$

We specified a prior distribution for f(t) from the spatio-temporal regression, and a likelihood function which describes the data generation process; the specified prior distributions and likelihood function are described below. We then used Markov Chain Monte Carlo (MCMC)[5] to approximate the posterior distribution of f(t) which also incorporates information from the observed empirical estimates of adult mortality. An MCMC chain of length 5000 was produced; the first 3000 samples were discarded and the remaining 2000 were thinned by a factor of 2 for a total of 1000 simulations retained. The reported best estimates and confidence intervals were generated from the mean and the 2.5th and 97.5th percentiles of the 1000 samples, respectively.

The prior distribution of f(t) can be described in terms of the mean prior—the prior for M—and the covariance prior—the prior for C. We utilized the second stage predictions as the mean prior and used a Matern covariance function to describe the covariance prior. The parameters of the Matern covariance function were selected through cross-validation and are region-specific.

For cross-validation, data were divided as follows: for each region, a number X between 10 and 20 was sampled and the most recent X years of data in that region were assigned to the testing set. Then a number X between 5 and 10 was sampled, a country from within the region was sampled, and a year where there is data in that country was sampled. All data within X years of the selected year in the selected country were assigned to the testing set. This was repeated as many times as there are countries in the region; because iterations of this procedure were independent, the data selected for the testing set may overlap. Any data that were not selected for the testing set were included in the training set.

For each testing and training division, the second stage model is fit on the training data. Then, the third stage model is also fit on the training data using each combination of scale and squared amplitude values tested for a total of 25 sets of predictions. The testing data are matched to the predictions in the corresponding country and year for each of the 25 sets of predictions. For each match we calculated the absolute relative error of the prediction compared to the empirical estimate in the testing set. We also classified each empirical estimate in the testing set as being covered or not covered by each corresponding prediction. The determination of coverage was made by calculating total variance—the sum of the variance of the empirical estimate (described below) and the variance of the GPR estimate—and then calculating a 95% confidence interval around the prediction based on this total variance and assuming a normal distribution. If the empirical estimate was within this confidence interval, it was classified as covered, and otherwise not.

Once this procedure has been carried out for all 100 testing and training divisions of the data we calculated the mean absolute relative error and the mean coverage for each combination of GPR parameters across all 100 sets of predictions. The ideal set of parameters would produce estimates with low mean absolute relative error and mean coverage close to 0.95. We used the function described in the equation below to calculate a loss metric which incorporates both the coverage and the absolute relative error into a single measure to assess performance. Parameter combinations with lower values of this loss metric are considered preferable.

$$\text{Loss} = \begin{cases} \text{if coverage} \leq 0.95\text{:} & (0.95 - \text{coverage})/5 + (\text{absolute relative error}) \\ \text{if coverage} > 0.95\text{:} & (\text{coverage} - 0.95)/1 + (\text{absolute relative error}) \end{cases}$$

The optimal parameters may differ from country to country. To allow for this, we calculated the loss function described in the equation above separately for each of the 22 GBD geographic regions.

Likelihood

The likelihood describes the probability of observing the data given a particular set of parameters. As shown in the equation below, we used a normal model for describing the probability of observing a particular value of log($_5q_0$) where the mean is given by f(t) and the variance by V_t, the data variance.

$$\log_{10}({}_5q_{0_t}) \sim \text{Normal}(f(t), V_t)$$

Data variance was calculated for each empirical observation of $_5q_0$ and incorporated both sampling and non-sampling variation. The method for calculating the data variance depended on the type of data:

1. For estimates derived from complete vital registration data we assumed that there was no non-sampling variance and included only sampling variance as computed from a binomial model. We set N equal to the national population aged 0 to 5 years and p equal to the mortality rate, $_5m_0$. We calculated the variance of $_5m_0$ from p(1-p)/N and then transformed this to the variance of $\log_{10}({}_5q_0)$ using the delta method.[6]
2. For estimates derived from incomplete vital registration data, we wanted to include not only sampling variance but also the non-sampling variance that arises from uncertainty in the completeness estimate. For these data, the total data variance was given by the sum of the

sampling variance (calculated as for complete vital registration data) and the variance of the completeness estimate;

3. For estimates derived from complete birth histories we generate 1000 simulations of $_5q_0$, convert these estimates into $\log_{10}$ space and calculate the sampling variance from these 1,000 simulations;
4. For estimates derived from summary birth histories we use the standard error from the mean residuals;
5. For estimates not covered under the above 4 calculations the missing data variance is determined as the maximum standard error from non-VR points in the country, if the data variance is still missing it is calculated as the maximum standard error from non-VR data in the GBD region.
6. Finally, for each source type, we calculate the within-source-type variance of the source-specific random effect. This additional non-sampling variance is then converted to $\log_{10}$ space and added to the variance as calculated above for all data points not classified as complete vital registration.

Hyper-parameter selection for under-5 mortality rate ST-GPR

In GBD 2015, we expanded the scope of our parameter selection to include variables used in space-time smoothing in addition to scale and amplitude used in the Gaussian process regression. We have applied rigorous out-of-sample predictive validity testing to select space-time and GPR parameters, and the process is carried out in the following steps:

1. For space-time smoothing, we tested ζ, space weight, values of 0.7, 0.8, 0.9, and 0.99 and λ, time weight, values of 0.1 to 0.9 in increments of 0.1. We test five values of the scale—10, 15, 20, 25, 30 --- and five values of the squared amplitude—1, 1.5, 2, 2.5, and 3 times the mean squared error of the residuals from the second-stage prediction model. Because we tested combinations of both space-time and GPR parameters, this led to a total of 900 combinations tested in each process.

2. We divided the data into testing and training sets 100 times. Data were divided as follows: for each region, a number X between 10 and 20 was sampled and the most recent X years of data in that region were assigned to the testing set. Then a number N between 5 and 10 was sampled, a country from within the region was sampled, and a year where there is data in that country was sampled. All data within N years of the selected year in the selected country were assigned to the testing set. This was repeated as many times as there are countries in the region; because iterations of this procedure were independent, the data selected for the testing set may overlap. Any data that were not selected for the testing set were included in the training set.

3. The space-time smoothing and Gaussian process regression are fit on the training set using each set of parameters and estimates for every location are generated for the entire time period;

4. Within a given iteration we calculate the absolute relative error of the final GPR estimates compared to each empirical estimate in the testing set. We also classify each empirical estimate in the testing set as covered or not covered and calculate the percent of the data covered by the 95% uncertainty interval of the GPR estimates while considering the uncertainty of the data themselves. The determination of coverage is made by calculating total variance—the sum of the data variance and the variance of the GPR

estimate—and then calculating a 95% uncertainty interval around the GPR estimate based on this total variance and assuming a normal distribution. If the empirical estimate is within this uncertainty interval, it is classified as covered, and otherwise not. For each combination of parameters, we calculate the mean absolute relative error and the mean coverage across all iterations from all countries within a particular group. The loss function described below is then calculated for each parameter combination, and the parameter combination with the lowest loss is selected for each group;

if coverage <=.95, loss = absolute relative error + ((1-coverage)-0.05) / 5
if coverage > 0.95, loss = absolute relative error + (0.05 - (1-coverage))/1

5. For U5MR, parameter selection occurs at the location level, i.e. different parameters for each location. While there are data sparse locations, all locations have some data on U5MR for the time period we provide estimates for.

In some cases, we restricted the universe of possible parameters. Most of these restrictions occurred for the λ parameter. For many locations with complete VR, we knew that higher lambda values would result in a larger confidence interval than would be appropriate. We also included a lower limit of λ for some locations with either incomplete VR or no VR, so that confidence intervals would reflect the uncertainty of these data. For example, in Western Europe, High-Income North America, High-Income-Asia-Pacific, and Australasia, we set the condition that λ must be less than 0.5 and the condition that ζ be .99. We also made some other manual exceptions to λ, ζ, and scale where results did not pass the common sense test.

We set the differentiability to 0.8 in countries with only complete VR data, excepting those in the Caribbean, Oceania, and the country of Mauritius, and to 2.0 in other all other locations. We used a lower differentiability in countries with complete VR data because in these countries we want the final estimates to follow the data closely even if the trend described is not smooth. In contrast, in countries where the data are less reliable we don't want the final estimates to be overly influenced by individual data points.

Identify and remove outliers

There are several important quality-control steps in reviewing child mortality data and estimates. First, data points from years in which fatal discontinuities occurred are outliered, unless they are VR data points with sufficient information that the fatal discontinuities can simply be subtracted out of the VR data. The intent is to capture the underlying mortality risk rather than large stochastic variations. These fatal discontinuities are then added on in a later step (see section 5). Secondly, we outlier data sources with quality concerns such as the Afghanistan DHS from 2010. Our extensive collaborator network allows for review of sources, and collaborators can raise concerns over known issues with data sources about which they have expert knowledge.

Rake subnational estimates to national level (excluding South Africa)

The estimation process for 5q0 does not enforce consistency between subnational estimates and national estimates. To ensure consistency throughout the GBD hierarchy, we rescaled the subnational estimates to the national level by population-weighting to get an implied national estimate from the subnational estimates, creating a scalar of the national-level estimate from GPR to the aggregated subnational estimates, and then multiplying all of the subnational estimates by this scalar to obtain the scaled estimates. In most cases, we considered national-level estimates to be more reliable, so we chose this

strategy of subnational scaling. In locations with high-quality vital registration data, this scaling has a minimal effect, but the effect can be greater in locations with more subnational units and variable-quality data. In South Africa, it was essential that the state-specific mortality patterns be consistent with HIV models is essential, since such a large part of the trend is driven by deaths due to HIV/AIDS. In this case, instead of scaling provincial-level estimates to national-level GPR estimates, we aggregated province-level GPR estimates to generate the national-level estimates.

Review estimates for quality

Estimates of U5MR from the ST-GPR process are reviewed with comparison to UNICEF estimates from their 2015 revision and GBD 2013 results. Any change and difference will be traced to either changes in available data or changes induced by the improved parameter selection process. Revision is made after the review process and through expert consultation with country experts and GBD mortality collaborator network.

Under-5 mortality rates with HIV

The U5MR ST-GPR process generates U5MR for all GBD 2015 locations that is inclusive of the impact of all causes of death excluding fatal discontinuities, which are added in a separate step (see section 5).

HIV-free 5q0

As a result of the Non-linear mixed effects model, we are able to generate HIV free 5q0 counterfactuals where the crude death rate due to HIV in age group 0-4 is set to zero. This is a crucial input to the GBD model life table system as described in section 3.

Under-5 age pattern model estimation

The process used to break down under-5 mortality into age- and sex- specific groups has been previously described.[7] The current process is largely similar but has been modified to improve the accuracy of predictions for countries affected by HIV/AIDS. As pointed out by Bradshaw et al., neonatal mortality tends to be overestimated if the all-cause child mortality rate is used as the only predictor.[8] We use a two-stage modeling process to generate sex-specific estimates of early neonatal (days 0 to 6), late neonatal (days 7 to 27), post-neonatal (the remainder of the first year), and childhood (ages 1 to 4) mortality. First, the ratio of male to female under-5 probability of death is estimated, then age- and sex-specific mortality estimates are generated using this ratio. To fit models to obtain estimates, data from vital registration, sample vital registration, and complete birth histories are converted to mortality risks for specific age groups. Sources have differing levels of age specificity and at least include infant (composed of early neonatal, late neonatal, and post-neonatal) and child mortality, but can include all 4 smaller age groups. The two models – first the sex model, then the age-specific and sex specific model – are fit on the data.
The sex model predicts the ratio of male probability of death under age 5 (${}_5q_0$) to female ${}_5q_0$ for each country *i* in region *j* in year *t*. The data are ordered by observed ${}_5q_0$, and categorized into 20 evenly sized bins. Then, the model is fit to the data as described in the equation below.

$$\left(\frac{\text{Male } {}_5q_0}{\text{Female } {}_5q_0}\right)_{\text{jit}} = \beta + \gamma_{{}_5q_0\,\text{bin}} + \gamma_j + \gamma_i + \varepsilon_{\text{jit}}$$

The ratio is predicted by nested location and region random effects γ_i and γ_j, a random effect on the ${}_5q_0$ bin, and an intercept term, β. A Loess regression is then used to smooth the estimated $\gamma_{{}_5q_0\,\text{bin}}$ on ${}_5q_0$,

creating a continuous $\gamma'_{{}_5q_0 \text{ bin}}$. Then, the equation below is used to predict the ratio of male to female ${}_5q_0$:

$$\left(\frac{\text{Male } {}_5q_0}{\text{Female } {}_5q_0}\right)_{\text{jit}} = \hat{\beta} + \gamma'_{{}_5q_0 \text{ bin}}\left({}_5q_{0_{\text{jit}}}\right) + \hat{\gamma}_{\text{j}} + \hat{\gamma}_{\text{i}}$$

The male and female ${}_5q_0$ values are found using the system of equations that includes the model above and equation below, where r_{birth} is the sex-ratio at birth.

$${}_5q_0 = \left(\frac{1}{1+r_{\text{birth}}}\right) * (\text{female}_5q_0) + \left(\frac{r_{\text{birth}}}{1+r_{\text{birth}}}\right) * (\text{male}_5q_0)$$

Age-specific models are then fit for each age group on sex-specific data. A separate model is fit for each age group yielding five models for each sex: early neonatal, late neonatal, postneonatal, infant, and child. The log of the probability that an under-5 death occurs in a given age group is modeled instead of the mortality risk, simplifying the scaling process and restricting risks to be between 0 and 1. Because evidence suggests HIV has differential effects on different under-5 age groups,[8,9] the crude death rates from HIV/AIDS in the under-5 age group were included in the model. We used crude death rate due to HIV from the GBD 2015 model (see section 3). The inclusion of this covariate improves both the fit and prediction of the model in countries with HIV. In addition, in this version of GBD, we added two new covariates to improve model fit. First, we included the maternal education covariate that is also used in the 5q0 first-stage model. Second, we used the completeness of the source-specific 5q0 estimate for the data-point used in the regression. This completeness was calculated by taking the source-specific 5q0 point estimate and dividing by the final 5q0 estimate from GPR. The functional form of the model is below.

$$\log(\Pr(\text{death at age y}|\text{u5 death})_{\text{jit}}) = \beta_1 + \beta_2 * HIV_{it} + \beta_3 * Mat.Ed._{it} + \beta_4 * Completeness_{sit} + \gamma_{{}_5q_0 \text{ bin}} + \gamma_{\text{j}} + \gamma_{\text{i}} + \varepsilon_{\text{jit}}$$

Similar to the sex model, the sex-specific age prediction uses ${}_5q_0$ bins and smooths the random effect on the bin using ${}_5q_0$. The prediction equation for age *y* in country in region *j* at time *t* is seen below, with nested random effects on country ($\hat{\gamma}_{\text{i}}$) and region ($\hat{\gamma}_{\text{j}}$), an intercept term ($\hat{\beta}_1$), a smoothed random effect on ${}_5q_0$ bin ($\hat{\gamma}'_{{}_5q_0 \text{ bin}}({}_5q_{0_{\text{jit}}})$), a coefficient on the under-5 crude death rate from HIV ($\hat{\beta}_2$), a coefficient on maternal education ($\hat{\beta}_3$), and a coefficient on completeness ($\hat{\beta}_4$):

$$\log(\Pr(\text{death at age y}|\text{u5 death})_{\text{jit}}) = \hat{\beta}_1 + \hat{\beta}_2 * HIV_{it} + \hat{\beta}_3 * Mat.Ed._{it} + \hat{\beta}_4 * 1 + \hat{\gamma}'_{{}_5q_0 \text{ bin}}({}_5q_{0_{\text{jit}}}) + \hat{\gamma}_{\text{j}} + \hat{\gamma}_{\text{i}} \quad 13$$

Note that for prediction, the completeness coefficient gets multiplied by 1 instead of a source-specific completeness, as we seek to predict based on a hypothetically complete source.

Once each of these predictions is made by age group, they are rescaled such that the probabilities of death in the Early Neonatal, Late Neonatal, Post Neonatal, and 1-4 year age groups aggregate to the 5q0 estimates from the under-5 model.

Identify and remove outliers

There are several criteria for removing outliers for the under-5 age-sex pattern model. First, sources may be marked as outliers if they contain low population numbers or very few deaths. If data come from vital registration and the under-5 population of the country is less than 20,000 person-years, then the data are outliered. If the total number of deaths in a VR system among both sexes under-5 is less than 200, the data are also outliered. VR data that are considered incomplete are marked as outliers. To be considered incomplete, the 9-year rolling average of the VR data 5q0 value is compared to the 9-year rolling average of the 5q0 estimates. Then, for a given data-year, the value of 5q0 in the raw data are compared to our final 5q0 estimate. A value of 90% would be considered incomplete and outliered, unless the ratio of the 9-year rolling average above is above 90% complete. Any data that are chosen as outliers as part of the 5q0 analysis are also marked as outliers in the age-sex pattern analysis. If the female-to-male ratio of 5q0 in the raw data are less than .5 or greater than 2, the data are outliered because of an implausible sex ratio. If a country has both VR and CBH data, they are typically both used, unless the two conflict, in which case the VR data are used. CBH data points more than 15 years before the survey are outliered. Lastly, some data points are manually outliered. For example, the definition of live birth changed in some Eastern European countries in the 1990s, leading to inconsistencies. For these examples, age group data in ages that would include childbirth deaths (early neonatal, neonatal, and ages 1-4) are outliered if the definition of live birth contains a minimum weight, as it did in some of these locations.

Under-5 age-sex splitting model application

The prediction method from the age-specific model is described above in 1.11. First, the results of the sex model are applied, yielding sex-specific 5q0 estimates. Once age-sex-specific predictions of the log conditional probability of death are made, these are exponentiated and rescaled so that they some to 1. First, the under-1 and 1-4 conditional probabilities are scaled to add to 1. Then, the early neonatal, late neonatal, and post neonatal conditional probabilities are scaled to the under-1 conditional probability. Then, the probabilities of death can be calculated so that they properly aggregate to the final 5q0 prediction. For example, to calculate the probability of death in the early neonatal age group, the rescaled conditional probability of early neonatal death given under-5 death is multiplied by the probability of under-5 death. Then, to obtain the probability of death in the late neonatal age group, the rescaled conditional probability of death in the late neonatal age group given under-5 death is multiplied by the probability of under-5 death and then divided by the probability of survival to the beginning of the age group, and so on. Equations below represent this process, where enn represents early neonatal and lnn represents late neonatal.

$$q_{enn} = \Pr(death\ in\ enn\,|\,u5\ death) * 5q0$$
$$q_{lnn} = \Pr(death\ in\ lnn\,|\,u5\ death) * 5q0/(1 - q_{enn})$$

The rest of the older age groups are also calculated in this manner, yielding probabilities of death in each of the under-5 age-sex groups.

Update under-5 populations using fatal discontinuities

To obtain denominators for vital registration death numbers and to estimate death numbers for age groups under-5, we need to obtain age-specific populations for the under-5 age groups. Taking final probability of death estimates including impacts of fatal discontinuities from the first run of the all-cause mortality process as the mortality risks, we take our input birth numbers and create person-year estimates of population as described in section 1.15. These person-year estimates are then the input as populations for the final run of the estimation process.

Under-5 death number estimation

Assigning under-5 deaths to GBD age-sex groups

To estimate the number of under-5 deaths, we run an estimation process that ages birth cohorts through our estimated probabilities of death. This process separates our yearly birth numbers for each location into week-sized cohorts and ages each of these cohorts through our mortality estimates in week-long steps to estimate the number of person-years and deaths in each of the early neonatal, late neonatal, post neonatal, and 1-4 years age groups.

3.3.1 HIV SDG Capstone Appendix

Flowchart

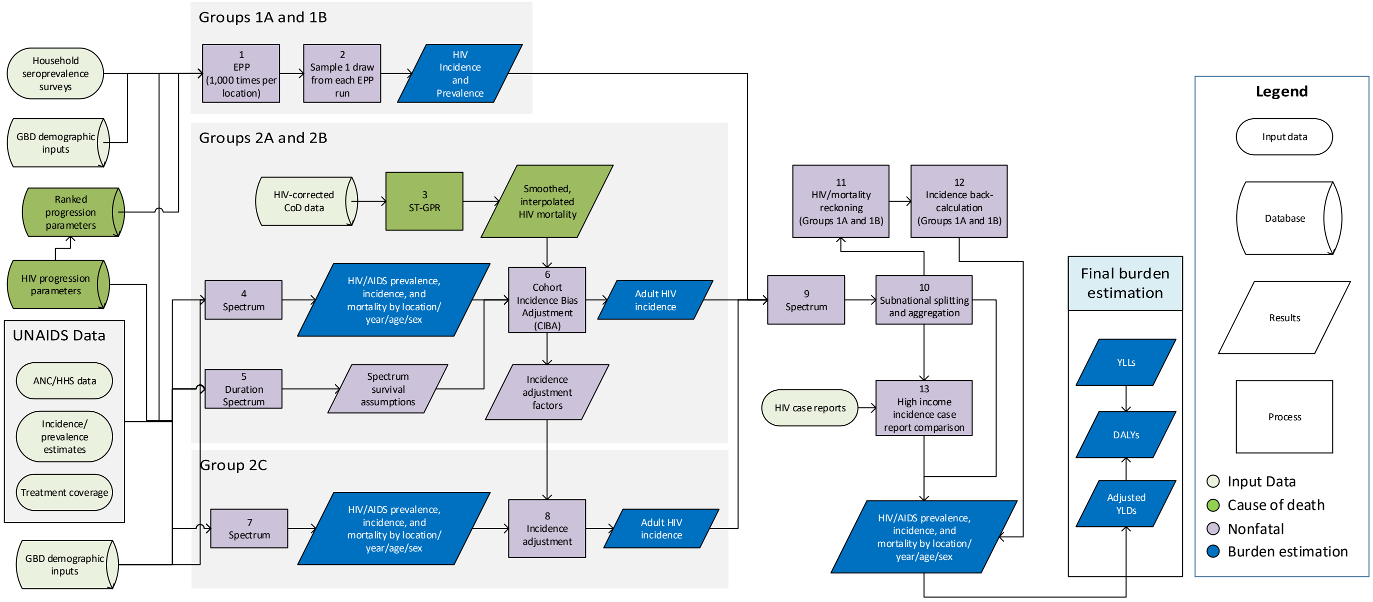

Input data and Methodology

Indicator definition

This modeling strategy encompassed the indicator associated with HIV incidence (3.3.1).

Indicator 3.3.1

As a component of SDG Goal 3. Ensure healthy lives and promote well-being for all at all ages, SDG Target 3.3, by 2030, end the epidemics of AIDS, tuberculosis, malaria and neglected tropical diseases and combat hepatitis, water-borne diseases and other communicable diseases, is measured using SDG Indicator 3.3.1, number of new HIV infections per 1,000.

Case definition

Infection with the human immunodeficiency virus (HIV) causes influenza-like symptoms during the acute period following infection and can lead to acquired immunodeficiency syndrome (AIDS) if untreated. HIV attacks the immune system of its host, leaving infected individuals more susceptible to opportunistic infections like tuberculosis. Although there are two different subtypes of HIV, HIV-1 and HIV-2, no distinction is made in our estimation process or presentation of results. For HIV, ICD 10 codes are B20-B24, C46-C469, D84.9; ICD 9 codes are 042-044, 112-118 (after 1980), 130 (after 1980), 136.3-136.8 (after 1980), 176.0-176.9 (after 1980), 279 (after 1980); and ICD9 BTL codes are B184-B185.

Input data

Model inputs

Household seroprevalence surveys

Geographically representative HIV seroprevalence survey results were used as inputs to the model for countries with generalized HIV epidemics where available.

GBD demographic inputs

Location-specific population, fertility, and HIV-free survival rates from GBD 2016 and migration data from UNAIDS were used as inputs in modeling all locations.

UNAIDS data

The files compiled by UNAIDS for their HIV/AIDS estimation process were our main source of data for producing estimates of HIV burden. These files are typically country-specific and contain both demographic data (population, fertility, migration, and HIV-free survival rates) and HIV-specific information. In all cases except migration, we substituted in our own, internally consistent demographic estimates. The HIV-specific information includes what is needed to run both the Spectrum and Estimation and Projection Package (EPP) models. Spectrum requires data on AIDS mortality among people living with HIV with and without ART, CD4 progression among people living with HIV not on ART, ART coverage among adults and children, coverage of breastfeeding among women living with HIV, prevention of mother-to-child transmission coverage, and CD4 thresholds for treatment eligibility. EPP uses many of the same assumptions as Spectrum but fits a simpler model to HIV prevalence data from surveillance sites and large household surveys. Antenatal care, incidence, prevalence, and treatment coverage data from UNAIDS were used in modeling for all locations. We extracted all of these data from UNAIDS' proprietary formats.

For GBD 2016, we received national-level files for 81 countries and subnational-level files for 6 countries. For many of the GBD locations not covered by these files, we had UNAIDS files from an earlier year of estimation, which we used again. After combining, we were left with a set of 42 countries for which we have never received a UNAIDS file, many of them countries with small populations and/or low HIV prevalence. In those places, we generated regional averages of all needed inputs. This enabled us to run Spectrum for every GBD location.

In several cases, we have modified the structure or data in the UNAIDS files. In South Africa, which we have estimated at the province level since GBD 2015, we split the national-level UNAIDS file into nine provincial datasets. We used GBD 2016 demographic inputs for the provinces. These provinces are already fit as separate subpopulations in EPP, so we extracted the prevalence data for the individual provinces and assumed national rates for all other Spectrum inputs. In some locations that are estimated only at the national level in GBD 2016, we received subnational files from UNAIDS. In these cases, we split GBD 2016 demographic input data using the subnational relative relationships found in the UNAIDS files. Additionally, we identified that the ratio of fertility in HIV-positive women to HIV-negative women was negative in Indonesia. We used linear extrapolation to replace this value.

Vital registration data

We used all available sources of vital registration and sample registration data from the GBD Causes of Death database after garbage code redistribution and HIV/AIDS mis-coding correction, except in Group 1A countries as described below.[1,2] There are two different cause of death data sources for HIV/AIDS in China: the Disease Surveillance Point (DSP) system and the Notifiable Infectious Disease Reporting (NIDR) system. Both systems are administered by the Chinese Center for Disease Control and Prevention, but the reported number of deaths due to HIV is significantly lower in DSP. Therefore, we have used the provincial-level ratio of deaths due to HIV/AIDS from NIDR to those from DSP, choosing the larger ratio between years 2013 and 2014, and scaled the reported deaths in the DSP system, which is in turn used in the Space-Time Gaussian Process Regression (ST-GPR) process.

On-ART literature data

Data were identified by using search terms “HIV,” “mortality,” and “antiretroviral therapy” in PubMed searches across the literature. To be included, studies must include only HIV-positive people who receive antiretroviral therapy (ART) but who were ART-naïve prior to the study. In addition, studies must report either a duration-specific mortality proportion or a hazard ratio across age or sex, and must not include children.

For duration-specific survival data, studies must report uncertainty on mortality estimates or provide stratum-specific sample sizes and must include duration-specific data to allow for calculation of 0-6, 7-12, or 13-24 month conditional mortality. In addition, studies must either report separate mortality and loss-to-follow-up (LTFU) curves, be corrected for LTFU using vital registration data, or be conducted in a high-income setting. Finally, studies must report the percent of participants who are male, the median age of participants, and either data with specific data on the number of CD4 T lymphocytes (CD4 counts) or the median CD4 count used for the data.

Hazard ratio data for ages or sexes can only be used if the hazard ratios are controlled for other variables of interest (age, sex, and CD4 category).

In GBD 2013, we identified 102 papers for extraction. For GBD 2015, we included 13 additional studies informing the duration-specific mortality estimation process and 26 studies informing the age and sex hazard ratio estimation process (some studies were used and counted in both). We also added one study to our LTFU analysis. For GBD 2016, we included 12 additional studies informing the duration-specific mortality estimation process and 11 studies informing the age and sex hazard ratio estimation process (some studies were used and counted in both).

Off-ART literature data

In GBD 2013, to characterize uncertainty in the progression and death rates, we systematically reviewed the literature on mortality without ART. We searched terms related to pre-ART or ART-naive survival since seroconversion.[3] After screening, we identified 13 cohort studies that included the cohorts used by UNAIDS from which we extracted survival at each one-year point after infection. Screening for additional, recently published studies in GBD 2015 and GBD 2016 identified no new cohort studies for inclusion in this analysis.

Severity splits & disability weights

The basis of the GBD disability weight survey assessments are lay descriptions of sequelae highlighting major functional consequences and symptoms. The lay descriptions and disability weights for HIV/AIDS severity levels are shown below.

Severity level	Lay description	DW (95% CI)
Symptomatic HIV	has weight loss, fatigue, and frequent infections.	0.274 (0.184-0.377)
AIDS with antiretroviral treatment	has occasional fevers and infections. The person takes daily medication that sometimes causes diarrhea.	0.078 (0.052-0.111)
AIDS without antiretroviral treatment	has severe weight loss, weakness, fatigue, cough and fever, and frequent infections, skin rashes, and diarrhea.	0.582 (0.406-0.743)

The proportion of people living with HIV/AIDS who are being treated with anti-retroviral therapy is an output of Spectrum, the compartmental model used to make consistent incidence, prevalence, and mortality estimates described below.

Modeling strategy

In GBD 2016, our general modeling strategy for estimating HIV incidence, prevalence, and mortality is very similar to the strategy used in GBD 2015. We continue to use the Spectrum program rewritten in Python for GBD 2013 to facilitate faster and more flexible execution necessary for our more intensive computational needs. We made several changes to Spectrum's assumptions comparing to the Spectrum software used by UNAIDS. We also again ran EPP using an open-source computer program in R written by Jeffrey Eaton.[4] We ran EPP for all Group 1 countries in order to produce incidence and prevalence estimates that were consistent with the demographic and epidemiological assumptions used in GBD 2016.

On-ART

First, we corrected reported probabilities of death for loss to follow-up using an update of the approach developed by Verguet and colleagues.[5] Verguet and colleagues used tracing and follow-up studies to empirically estimate the relationship between death in LTFU and the rate of LTFU.

To create estimates of age-specific hazard ratios, we synthesized hazard ratio data in five broad age groups: 15-25, 25-35, 35-45, 45-55, 55-100, and modeled the data using DisMod-MR 2.0.

To create estimates of sex-specific hazard ratios, we use the *metan* function in Stata to create estimates of relative risks separately by region, using female age groups as the reference group.

The age and sex hazard ratios were applied to the study level mortality rates, accounting for the distribution of ages and sexes in the mortality data. We then subtracted HIV-free mortality from the model life table process to calculate study level age-sex HIV-specific mortality.

We used DisMod-MR 2.0 to synthesize the age-sex split study level data into estimates of conditional probability of death over initial CD4 count.[3] We modeled the data separately by duration, age, and sex and added a fixed effect on whether the study was conducted prior to 2002. We estimated all three regions together using a fixed effect for each region.

Changes for GBD 2016

In GBD 2016, we chose to age-sex split the data at the study level so that we could consider study-specific age-sex distributions, whereas previous GBD iterations relied upon region-specific distributions. Another change was a switch to estimating all regions together with fixed effects for each region. This allowed us to impart a CD4 trend in sub-Saharan Africa and other developing country estimates that led to more realistic estimates in the high CD4 categories where little data was available from those regions.

Off-ART

Following UNAIDS assumptions, no-ART mortality is modeled as shown in the figure below.[3]

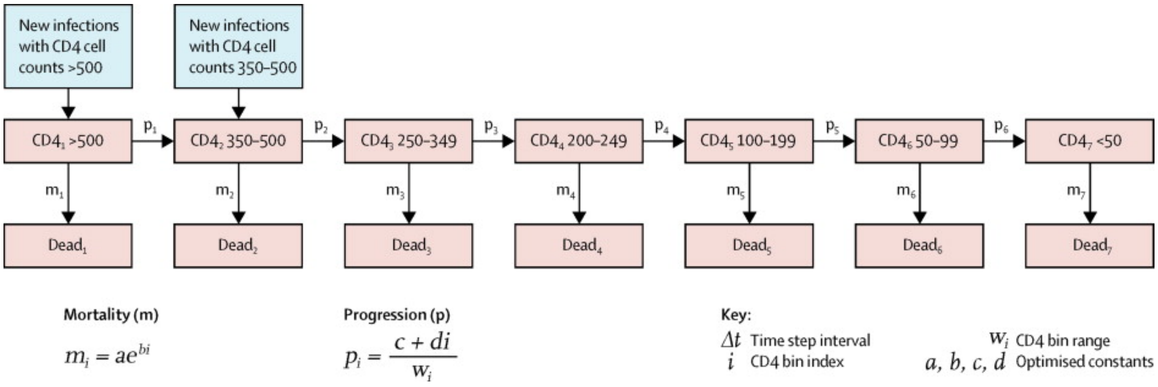

The death and progression rates between CD4 categories vary by age according to four age groups: 15–24 years, 25–34 years, 35–44 years, and 45 years or older. We modeled the logit of the conditional probability of death between years in these studies using the following formula:

$$\text{logit}\,(m_{ijk}) = \beta_0 + \sum_{i=1}^{4} \beta_{1i}\, a_i + \sum_{j=1}^{12} \beta_{2j} t_j + u_k + \varepsilon_{ijk}$$

In the formula, m is conditional probability of death from year t_j to t_{j+1}, a_i is an indicator variable for age group at seroconversion (15–24 years, 25–34 years, 35–44 years, and 45 years or older), t_j is an indicator variable of year since seroconversion, and u_K is a study-level random effect.

By sampling the variance-covariance matrix of the regression coefficients and the study-level random effect, we generated 1,000 survival curves for each age group that capture the systematic variation in survival across the available studies. For each of the 1,000 survival curves, we used a framework modeled after the UNAIDS optimization framework in which we find a set of progression and death rates that minimizes the sum of the squared errors for the fit to the survival curve.[6,7]

Burden estimation overview

UNAIDS uses two key analytical components in their epidemiological estimation. EPP is used to estimate incidence trajectories that are consistent with prevalence surveys and other prevalence measurements such as antenatal clinic serosurveillance. Spectrum is a compartmental HIV progression model used to generate age-specific incidence, prevalence, and death rates from the EPP incidence curves and assumptions about intervention scale-up and local variation in epidemiology.

For GBD 2013, we created an exact replica of Spectrum in Python. This enabled us to run thousands of iterations of the model at once on our computing cluster and allowed for more flexible input data structures. Additionally, in order to generate estimates with more realistic ranges of uncertainty than those in UNAIDS 2012, we adjusted all input data by uniformly sampled factors between 0.9 and 1.1. These changes, along with our new estimation of with- and without-ART mortality and CD4 progression parameters, persist into GBD 2016.

Due to the substantial differences in the quality and types of data available across different countries, we used three different methodologies to produce year-, age-, and sex-specific estimates of HIV incidence, prevalence, and mortality.

Countries with seroprevalence surveys and antenatal clinic data (Groups 1A and 1B)

We identified 50 countries – as well as subnational locations in India, Kenya, and South Africa – with at least 0.5% adult HIV prevalence and at least one geographically representative HIV seroprevalence survey or available antenatal care clinic (ANC) data. In order to ensure that our estimates of incidence and prevalence in these places were consistent with our estimates of HIV progression, we used a version of EPP written in R and C++ by Jeffrey Eaton to create new fits to the available prevalence data. The version of EPP used in GBD 2016 was an updated release from Jeffrey Eaton since completion of GBD 2015. In this new version, an ANC prevalence adjustment was included and incorporated with the 2016 lookup database and an additional parameter to estimate ANC variance inflation was included as well. In the ANC bias adjustment, instead of using the default universal assumption of the prior mean and standard deviation (SD) of the distribution that the adjustment follows, we selected the parameters based on each sub-population (general population and high risk population) in each location. For sub-populations with prevalence survey data, we used the default assumption with mean=0.15 and SD=1. For subpopulations without prevalence survey data, we chose the region/epidemic specific mean and SD based on the median probit difference and probit difference SD in Table 1 of Marsh et al.[8]

India's HIV epidemic is classified as concentrated in specific subpopulations rather than generalized to the full population, and only one prevalence survey, the 2005-2006 National Family Health Survey (NFHS-3), was available, so we used modified parameters for Indian states in EPP. We first calculated the mean of the median probit difference between men and women for "Countries with concentrated epidemics" in Table 1 of March et al as mentioned above, which was 0.245. Then we derived empirical parameters based on the difference between the ANC data and the NFHS-3 survey data in probit space to use for the general population. Specifically, we calculated the probit difference by taking the median of all raw ANC prevalence in years 2004 through 2006 and comparing to the 2005 prevalence survey data in probit space for three states with large HIV epidemics: Andhra Pradesh, Karnataka, and Maharashtra. From this empirical parameter derivation, we got the mean and SD value based on the three states as 0.124 and 0.051, respectively. We then used linear interpolation between the prevalence with a prior of 0.245 and the new prior of 0.124 to recalculate the mean and keep the SD the same as the empirical estimates. The final assumption of the prior mean and SD were 0.182 and 0.051, respectively. We did not make any adjustments for high risk populations.

In the new version of EPP, in addition to the equilibrium prior assumption of the force of infection in projection, a random walk approach is available as an alternative method. For locations with two or more prevalence surveys and a declining trend between the mean of the most recent two surveys, the random walk approach was chosen to project the force of infection. We assumed the change of the log scaled force of infection was following a normal distribution with mean equal to the median of the change of the modeled force of infection among the years having ART implemented or prevalence data, and the SD was equal to the default setting as the mean SD of the change of the modeled force of infections among the years having prevalence data. The projection year was chosen from the most recent year between the year with the lowest model force of infection and the year of the second latest survey data.

For Indian states, we used the equally weighted draw-level estimates of the equilibrium prior and random walk assumptions since we had no further information to support either assumption for each state. Here, the projection year of the random walk was the year with the lowest modeled force of infection because no locations had more than one prevalence survey, and the assumption of increasing ART coverage was supported by the data available to us.

In the new EPP code, an optimization step was added into IMIS function to speed up the parameter sampling step based on Raftery and Bao.[9] Two optimization methods have been introduced. The main algorithm is Broyden–Fletcher–Goldfarb–Shanno (BFGS) optimization. If BFGS fails, Nelder-Mead optimum is used instead. In our 2016 EPP model, by substituting in our own assumptions about HIV progression rates and on/off ART mortality, we were able to ensure that the implied relationship between incidence and mortality/prevalence in EPP is similar to that in Spectrum.

In Group 1 locations, we expect estimates of HIV burden to exhibit substantial uncertainty. To reflect this, we induced a perfect correlation between the previously independent draws of HIV mortality with and without ART and CD4 progression. We paired the draws of the three parameter sets internally and with each other in the following way: we sorted without-ART mortality and CD4 progression internally by age (not CD4), meaning the highest draw of HIV mortality without ART for age a_i and CD4 category c_l will be paired with the highest draw of HIV mortality without ART for age a_k and CD4 category c_l. In the same way, we sorted with-ART mortality internally by age, sex, CD4 count at treatment initiation, and duration on treatment. After this sorting process, the lowest indexed draw of each parameter has the highest values and vice versa. This means that we will use the most extreme possible parameter sets in EPP and Spectrum and should see a commensurate expansion in the range of the uncertainty.

To ensure that this expanded uncertainty is replicated in EPP, we fit the model once for every set of paired draws of the progression parameters for every location. This means that the first iteration of EPP for Uganda sees the highest draws of all three sets of progression parameters. Such a procedure is necessary because EPP currently has no mechanism for incorporating uncertainty in any inputs except prevalence data. This process (Process 1 in the HIV/AIDS Estimation Flowchart), produced 1,000 sets of EPP output for each of the locations that make up the 48 countries in the group. Every set of EPP outputs contains 500 consistent draws of HIV incidence and prevalence in adults aged 15-49. In many cases, the algorithm used to fit EPP, incremental mixture importance sampling, failed, resulting in fewer than 1,000 sets of EPP results.

For every location in the group, we sampled one of the 500 incidence/prevalence draws from each of the sets of EPP results (Process 2 in the HIV/AIDS Estimation Flowchart). By sampling one draw from each set, we ensured that the distribution of progression parameters dictating the relationship between incidence and prevalence was exactly the same as the distribution of the sorted parameters generated in the previous step. In locations where not all 1,000 iterations of EPP fit successfully, we sampled one draw from every iteration that did succeed and then resampled with replacement from that set of draws. To maintain the link between the input progression draws and the resulting incidence and prevalence draws from EPP, we replaced any parameter draw associated with a failed run of EPP with the parameter draw that that failed draw was replaced with. At the end of this process, for every location in the set of 48 countries, we were left with 1,000 linked draws of adult incidence and prevalence and the exact progression parameters that generated those draws.

We then ran these results, along with the previously described demographic and HIV-specific inputs, through Spectrum to produce location-, year-, age-, and sex-specific estimates of HIV incidence, prevalence, and mortality (Process 9 in the HIV/AIDS Estimation Flowchart).

The HIV/mortality reckoning process (Process 11 on the HIV/AIDS Estimation Flowchart) is intended as a method of reconciling separate estimates of HIV mortality (and its resulting effect on estimates of HIV-free and all-cause mortality) in Group 1 countries by averaging estimates of HIV mortality from the model

life table process and EPP-Spectrum. Additional details on the reckoning can be found in the GBD 2016 mortality manuscript.[10]

Since Spectrum produces HIV incidence, prevalence, and deaths that are consistent with one another over time, the reckoning process results in death numbers that are no longer consistent with the incidence and prevalence produced in Spectrum. In order to recreate this consistency, we recalculated incidence for all Group 1 locations using reckoned deaths and prevalence produced by Spectrum (Process 12 on the HIV/AIDS Estimation Flowchart). The updated incidence is calculated by aggregating counts of people living with HIV (PLWH), new infections, and deaths (among PLWH from HIV and other causes) at the year-sex level and calculating the following ratio for each sex:

$$r_t = \frac{[(PLWH_t - PLWH_{t-1}) + (Deaths_{hiv,t} + Deaths_{background,t})]}{NewHIV_t}$$

Age-specific counts of new infections are then scaled by their corresponding sex-year ratios.

Countries with vital registration data (Group 2A and 2B)

Vital registration is one of the highest-quality sources of data on HIV burden in many countries, so generating estimates that are consistent with these data, with necessary adjustment to account for any potential underreporting, is critical. We identified 114 countries – as well as 440 subnational locations from Brazil, China, Japan, Indonesia, Mexico, Sweden, the United Kingdom, and the United States – with at least two usable points of vital registration data, verbal autopsy (VA) data, or sample registration system (SRS) data. In India and Indonesia, we used SRS and VA data, respectively, as input mortality for CIBA. For India we extracted the resulting age-sex distribution of incidence, but scaled the level to match the adult incidence rate estimated from EPP for each state.

We imputed missing years of data to generate a complete time series for HIV from the estimated start year of the epidemic using ST-GPR. We analyzed mortality trends using ST-GPR starting in 1981, the year that HIV was first identified in the United States.[11] For ST-GPR, we adjusted the lambda (time weight) and GPR scale according to the completeness of vital registration data, with 4- and 5-star quality VR using parameters designed to follow the data more closely. We produced separate splines by country/age group, up to the peak year of death rate. We then ran a linear regression with random effects on region, age, and sex. Following this, we ran space-time residual smoothing, in which time, age, and space weights are used to inform smoothing of the residuals between data points and the linear regression estimate. From this process, we generated space-time estimates with the applied weights, along with the median absolute deviation (MAD) of the space-time estimates from the data. The MAD was calculated at various levels of the geographic hierarchy (e.g., subnational and national), and was added into the data variance term. The data variance and space-time estimates were then analyzed using Gaussian Process Regression to return a final estimate of mortality along with uncertainty.

Although Spectrum produces HIV mortality estimates that are within the realm of possibility in most countries using the incidence curves provided in the UNAIDS country files, it is a deterministic model that has not yet been integrated into an optimizable framework. Therefore, in order to “fit” it to vital registration data, we need to adjust input incidence.

To improve the fit of this process, in GBD 2015, we restructured Spectrum to add compartments that identify groups of people living with HIV by year of infection (Process 5 in the HIV/AIDS Estimation Flowchart). With this version of Spectrum we can output, among many other metrics, HIV deaths by year, age, sex, and infection cohort. This enables us to adjust incidence to fit to death much more precisely and without making any rigid assumptions about the time from HIV infection to HIV death.

We have incorporated these improvements into a cohort incidence bias adjustment (CIBA) process. First, we ran Spectrum normally to produce 1,000 draws of incidence, prevalence and mortality (Process 4 in the HIV/AIDS Estimation Flowchart). Then, by year, age, and sex, we took the ratio of VR deaths to Spectrum deaths to quantify the amount of bias in Spectrum. Using draw-level duration data from the new version of Spectrum, for every year-, age-, and sex-specific infection cohort, we calculated the share of all HIV deaths observed over the course of the projection period in that cohort that would occur in each year after the year of infection. For example, projecting from 1970 through 2016, we identified the cohort of men infected in 1992 at the age of 16, calculated the total number of HIV deaths in that cohort in all subsequent years through the end of 2016, and divided the annual number of deaths by that total. This showed us the distribution of deaths among that cohort over the projection period. In the most extreme case (infections in 2015), we could only produce one point of that distribution (2016), so that single value is exactly 1·0; 100% of the deaths observed in that cohort occurred in 2016.

We then used these distributions of death to weigh the ratio of VR deaths to Spectrum deaths, meaning that ratios in the years where we expect the largest share of deaths were weighed most heavily. We then multiplied the initial size of that cohort from the normal run of Spectrum by the sum of the combined ratios to get a new estimate of new cases in that year/age/sex combination.

We can write this method mathematically in the following way:

$$r_t = \frac{VR_t}{D_t}$$

$$\rho_t^{t-i} = \frac{d_t^{t-i}}{\sum_{k=t-i+1}^{n} d_k^{t-i}}$$

$$\alpha^{t-i} = \sum_{k=t-i+1}^{n} r_k * \rho_k^{t-i}$$

$$n_{\text{adjusted}}^{t-i} = \alpha^{t-i} * n^{t-i}$$

VR_t is the number of HIV/AIDS deaths in year t from ST-GPR, and D_t is the number of HIV/AIDS deaths from the first run of Spectrum. In the second equation, d_t^{t-i} is the number of HIV/AIDS deaths among members of infection cohort $t-i$ in year t, with $i \geq 1$, from the new, duration-tracking version of Spectrum, and n is final year of the projection. Therefore, ρ_t^{t-i} is the share of observed deaths in cohort $t-i$ that we expect to occur in year t. It follows that α^{t-i} is the weighted adjustment ratio described above, which we multiply by the estimated initial size of infection cohort $t-i$ as calculated in the first-stage Spectrum run to get the adjusted number of new cases, $n_{\text{adjusted}}^{t-i}$. This process is run separately for every sex, single-age, and draw.

CIBA (Process 6 in the HIV/AIDS Estimation Flowchart) allows ratios in each year after a given infection year to influence the final adjustment to incidence. The size of that influence is determined by the relative importance of that year in the cohort-year's distribution of deaths over time. The result is a new set of 1,000 draws of incidence and a set of 1,000 ratios of post-adjustment incidence to pre-adjustment incidence. We perform this adjustment using mean durations from the new version of Spectrum in order to try to shift the mean of the regular distribution of deaths.

Finally, to produce location-, year-, age-, and sex-specific estimates of HIV incidence, prevalence, and mortality, we ran the new estimates of incidence and all previously input data through Spectrum (Process 9 in the HIV/AIDS Estimation Flowchart).

Countries without survey data and vital registration data (Group 2C)

The remaining 31 countries – as well as 14 subnational locations from China and Saudi Arabia – had neither geographically representative seroprevalence surveys nor reliable vital registration systems. To produce estimates of HIV burden in these countries, we assumed that Spectrum is similarly biased as in other Group 2 countries. This involved running Spectrum (Process 7 in the HIV/AIDS Estimation Flowchart), adjusting incidence using 1,000 adjustment ratios randomly sampled from the entire set of CIBA results (Process 8), and rerunning Spectrum using the new draws of adjusted incidence (Process 9). As above, the estimates of incidence, prevalence, and mortality were incorporated into the rest of the machinery via the reckoning process.

Subnational splitting and aggregation

Spectrum results for India, Kenya, and UK subnational locations are modeled at higher levels of geography than our GBD locations. Spectrum results for India are produced at the state level, while GBD 2016 estimates were produced at the state urban-rural level; Spectrum models Kenya provinces, while we compute Kenyan estimates for 47 counties. Indonesia and the United Kingdom have Spectrum results at the national level, while GBD 2016 estimates Indonesian provinces and Upper Tier Local Authorities in the UK. To split the Spectrum results into more granular results for processing, we assign each GBD subnational unit to a Spectrum modeling unit. From this, we generate age/sex/year-specific proportions for population, HIV-specific death, and HIV-free mortality.

In Cote d'Ivoire, Haiti, Moldova, Mozambique, and Zimbabwe, the country files that we received from UNAIDS contained only subnational data without national-level aggregates. In these locations, we generated GBD 2016 demographic inputs for the provided subnational units using the proportions present in the UNAIDS files and ran the locations through EPP and Spectrum at the subnational level before aggregating to generate final national level GBD 2016 estimates. These aggregation and splitting steps are shown as Process 10 in the HIV/AIDS Estimation Flowchart.

HIV/AIDS resulting in other diseases

There are two Level 4 causes under the HIV/AIDS Level 3 cause in the GBD 2016 cause hierarchy. The modeling process for HIV/AIDS-tuberculosis is detailed in another part of this appendix. We computed the number of people living with HIV resulting in other diseases by subtracting the number of people living with HIV/AIDS-tuberculosis from all people living with HIV/AIDS at the 1,000 draw level.

References

1 Global, regional, and national age–sex specific all-cause and cause-specific mortality for 240 causes of death, 1990–2013: a systematic analysis for the Global Burden of Disease Study 2013. *The Lancet* 2015; **385**: 117–71.

2 Birnbaum JK, Murray CJ, Lozano R. Exposing misclassified HIV/AIDS deaths in South Africa. *Bull World Health Organ* 2011; **89**: 278–85.

3 Murray CJL, Ortblad KF, Guinovart C, *et al.* Global, regional, and national incidence and mortality for HIV, tuberculosis, and malaria during 1990–2013: a systematic analysis for the Global Burden of Disease Study 2013. *The Lancet* 2014; **384**: 1005–70.

4 jeffeaton/epp. GitHub. https://github.com/jeffeaton/epp (accessed April 21, 2016).

5 Verguet S, Lim SS, Murray CJL, Gakidou E, Salomon JA. Incorporating Loss to Follow-up in Estimates of Survival Among HIV-Infected Individuals in Sub-Saharan Africa Enrolled in Antiretroviral Therapy Programs. *J Infect Dis* 2013; **207**: 72–9.

6 Ghys PD, Zaba B, Prins M. Survival and mortality of people infected with HIV in low and middle income countries: results from the extended ALPHA network. *AIDS Lond Engl* 2007; **21 Suppl 6**: S1–4.

7 Hallett TB, Zaba B, Todd J, *et al.*, ALPHA Network. Estimating incidence from prevalence in generalised HIV epidemics: methods and validation. *PLoS Med* 2008; **5**: e80.

8 Marsh K, Mahy M, Salomon JA, Hogan DR. Assessing and adjusting for differences between HIV prevalence estimates derived from national population-based surveys and antenatal care surveillance, with applications for Spectrum 2013. *AIDS Lond Engl* 2014; **28 Suppl 4**: S497–505.

9 Raftery AE, Bao L. Estimating and Projecting Trends in HIV/AIDS Generalized Epidemics Using Incremental Mixture Importance Sampling. *Biometrics* 2010; **66**: 1162–73.

10 Wang H, Murray CJ, Carter A, He F. Global, regional, and national under-5 mortality, adult mortality, age-specific mortality, and life expectancy, 1970-2016: a systematic analysis for the Global Burden of Disease Study 2016. *The Lancet* 2017.

11 CDC. Pneumocystis Pneumonia --- Los Angeles. MMWR Wkly. 1981; published online June 5. http://www.cdc.gov/mmwr/preview/mmwrhtml/june_5.htm (accessed April 21, 2016).

3.3.2 Tuberculosis SDG Capstone Appendix

Flowchart

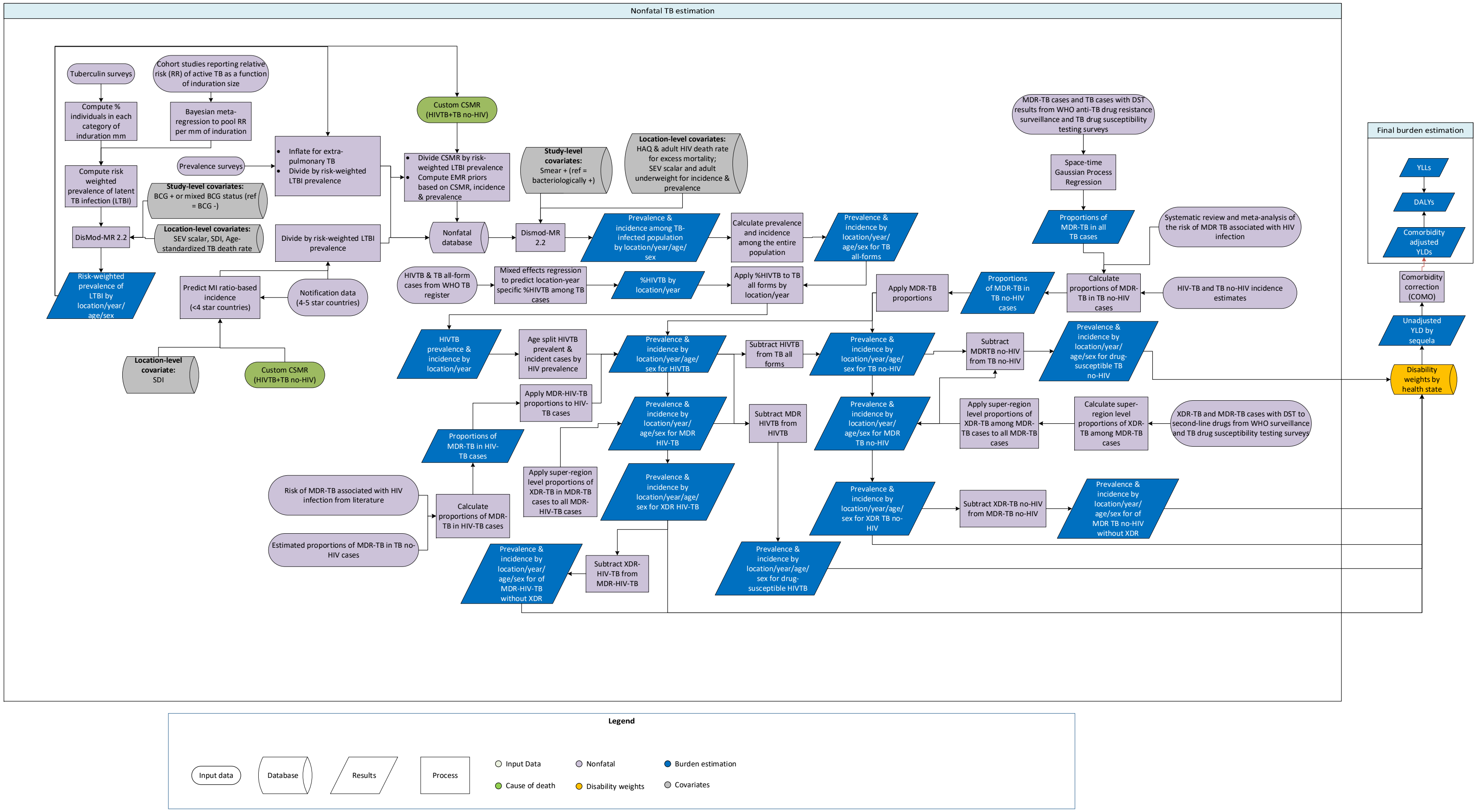

Input Data & Methodological Summary

Indicator definition

This modeling strategy encompassed the indicator associated with tuberculosis incidence (3.3.2).

Indicator 3.3.2

As a component of SDG Goal 3. Ensure healthy lives and promote well-being for all at all ages, SDG Target 3.3, by 2030, end the epidemics of AIDS, tuberculosis, malaria and neglected tropical diseases and combat hepatitis, water-borne diseases and other communicable diseases, is measured using SDG Indicator 3.3.2, number of new and relapsed TB cases per 100,000.

Case Definition

Tuberculosis (TB) is an infectious disease caused by Mycobacterium tuberculosis. The case definition includes all forms of TB including pulmonary TB and extrapulmonary TB which are bacteriologically confirmed or clinically diagnosed. For TB, the ICD 10 codes are A10-A19.9, B90-B90.9, K67.3, K93.0, M49.0, P37.0, and ICD 9 codes are 010-019.9, 137-137.9, 138.0, 138.9, 139.9, 320.4, 730.4-730.6. For HIV-TB, the ICD 10 code is B20.0.

Latent TB infection (a new sequela added for GBD 2016) is defined as an infection with Mycobacterium tuberculosis, without any symptoms or signs of active TB disease.

We have separately estimated the incidence and prevalence of multidrug-resistant tuberculosis and extensively drug-resistant tuberculosis by HIV status in GBD 2016. The case definitions of the new causes are shown below.

(1) Multidrug-resistant TB without extensive drug resistance: a form of TB (among HIV-negative individuals) that is resistant to the two most effective first-line anti-tuberculosis drugs (isoniazid and rifampicin), but is not resistant to any fluoroquinolone and any second-line injectable drugs (amikacin, kanamycin, or capreomycin).

(2) Extensively drug-resistant TB: a form of TB (among HIV-negative individuals) that is resistant to isoniazid and rifampicin, plus any fluoroquinolone and any second-line injectable drugs.

(3) Drug-susceptible TB: TB (among HIV-negative individuals) that is susceptible to isoniazid and rifampicin

(4) Multidrug-resistant HIV-TB without extensive drug resistance: a form of TB (among HIV-positive individuals) that is resistant to the two most effective first-line anti-tuberculosis drugs (isoniazid and rifampicin), but is not resistant to any fluoroquinolone and any second-line injectable drugs (amikacin, kanamycin, or capreomycin).

(5) Extensively drug-resistant HIV-TB: a form of TB (among HIV-positive individuals) that is resistant to isoniazid and rifampicin, plus any fluoroquinolone and any second-line injectable drugs

(6) Drug-susceptible HIV-TB: TB (among HIV-positive individuals) that is susceptible to isoniazid and rifampicin

Input data

Model Inputs

Input data for TB include annual case notifications, data from prevalence surveys, and estimated cause-specific mortality (CSMR) of TB among HIV-positive and HIV-negative individuals. From these inputs, we calculated 'priors' (expected values) on excess mortality to give more guidance to the model. An updated systematic review was done for GBD 2016 (the search terms are shown in the table below).

Input data for latent TB infection (LTBI) include: (1) population-based tuberculin surveys, and (2) cohort studies examining the risk of developing active TB disease as a function of induration size. We searched PubMed and Google Scholar, and also manually searched the reference list of relevant studies to aid identification of additional studies. The search terms, number of studies identified, and number of studies included are shown in the table below.

Outcome	Search Terms	Total number of studies identified	Number of studies included
Tuberculosis*	PubMed search terms: ("tuberculosis"[MeSH] OR tuberculosis[Title/Abstract]) OR TB[Title/Abstract] OR Mycobacterium tuberculosis[Title/Abstract] AND prevalence[Title/Abstract] AND ("2015/01/01"[PDAT] : "2016/11/02"[PDAT]) NOT (animals[MESH] NOT humans[MESH])	1061	3
LTBI (tuberculin surveys)	PubMed search terms: ("tuberculin survey"[tiab] OR (("risk"[MeSH Terms] OR "risk"[tiab] OR "risk of"[tiab]) AND ("tuberculosis"[MeSH Terms] OR "tuberculosis"[tiab] OR "tuberculous"[tiab]) AND ("infection"[MeSH Terms] OR "infection"[tiab])) OR (("risk"[MeSH Terms] OR "risk"[tiab] OR "risk of"[tiab]) AND TB[tiab] AND ("infection"[MeSH Terms] OR "infection"[tiab])) OR "latent tuberculosis infection"[tiab] OR "latent TB infection"[tiab] OR "latent tuberculosis"[MESH]) AND ("survey"[tiab] OR "surveys"[tiab]) NOT (animals[MESH] NOT humans[MESH])<br><br>Google Scholar search terms: ("tuberculin survey") OR (("risk of tuberculous infection" OR "risk of tuberculosis infection" OR "risk of TB infection" OR "latent tuberculosis infection" OR "latent TB infection") AND "survey")	9029	108
LTBI (cohort studies)	PubMed search terms: ("tuberculin"[tiab] OR ("tuberculin"[tiab] AND "positive"[tiab]) OR "Mantoux"[tiab] OR ("Mantoux"[tiab] AND "positive"[tiab]) OR "induration"[tiab]) AND (active[tiab] AND ("tuberculosis"[MeSH] OR "tuberculosis"[tiab])) AND ("risk"[MeSH] OR "risk"[tiab]) AND ("prospective"[tiab] OR "follow up"[tiab] OR "longitudinal"[tiab])	3624	27

	Google Scholar search terms: (("tuberculin" OR "Mantoux" OR “tuberculin reactivity”) AND (“risk of tuberculosis” OR “tuberculosis risk”)) -autopsy -autopsies -nosocomial -qualitative -prison -cancer -malignant -homeless -smoking		

* Updated systematic review, covering the period from 2015/01/01 to 2016/11/02

Input data for multidrug-resistant TB (MDR-TB) and extensively drug-resistant TB (XDR-TB) include: (i) the number of drug-resistant cases by type [MDR-TB, XDR-TB, TB cases with a drug sensitivity testing (DST) result for isoniazid and rifampicin, and MDR-TB cases with DST for second-line drugs] from routine surveillance and surveys reported to the World Health Organization, and (ii) the risk of MDR-TB associated with HIV infection from the literature.[1]

Modeling Strategy

Overview

We made major changes to our modelling of TB. First, we estimated risk-weighted prevalence of LTBI by location, year, age and sex using data from population-based tuberculin surveys and cohort studies reporting on the risk of developing active TB disease as a function of induration size. Next, we divided the inputs on prevalence (from surveys in low and middle income countries), incidence (notification data from countries with a four or five-star rating, and estimated incidence for countries with a less than four-star rating), and CSMR by the risk-weighted LTBI prevalence in order to model TB among those at risk in each country. To generate incidence estimates, we first ran a regression using MI ratios (logit transformed) from locations with a 4 or 5-star rating on causes of death with SDI as a covariate anchoring the lower end of the SDI scale with a data point from the Bangalore study[2] reporting that 49.2% of 126 untreated new pulmonary TB cases were dead at the end of the 5-year follow up period, to predict age-sex specific MI ratios for all locations and years. We then estimated age-sex specific incidence using the predicted MI ratios and CSMR estimates. We used DisMod-MR 2.2, the GBD Bayesian meta-regression tool to generate consistent trends in all parameters. We then multiplied the DisMod-MR 2.2 outputs by the risk-weighted prevalence of LTBI to get population-level estimates of incidence and prevalence. Because the output from DisMod-MR 2.2 are for all forms of TB, we split them into MDR-TB and XDR-TB by HIV status. To do so, we estimated the proportions of TB cases with MDR-TB for all locations and years, using data from notifications and survey data. We then estimated the proportions of MDR-TB among HIV-negative individuals and MDR-TB among HIV-positive individuals based on the risk of MDR-TB associated with HIV infection from a meta-analysis[1]. To split MDR-TB into MDR-TB with and without extensive drug resistance, we pooled the limited notification and survey data on the proportion of MDR-TB cases who are extremely drug resistant by super-region, and applied these proportions to MDR-TB cases among HIV-negative and HIV-positive individuals respectively.

Modeling risk-weighted latent TB infection prevalence

Input data for modeling risk-weighted LTBI prevalence were from two sources: (i) population based tuberculin skin test (TST) surveys, and (ii) cohort studies examining the risk of developing active TB

disease as a function of induration size. First, we extracted the prevalence of tuberculin skin testing results by induration size using the most detailed induration categories reported by studies. Second, from cohort studies reporting on the relative risk of developing active TB disease as a function of induration size, we pooled the risk of developing active TB by induration size in millimeters using the DisMod Ode computational engine. Third, we multiplied the LTBI prevalence by induration in millimeters ranging from 0-20+ with the relative risk of developing active TB at each induration size, and summed them up to derive risk-weighted LTBI prevalence for each age group.

Available evidence[3] suggests that people with very advanced HIV infection (CD4 counts <200 cells/mm^3) may have a false-negative TST (0mm induration) due to profound immune suppression, but still have very high risk for TB. For those who are HIV-positive, but with higher CD4 counts, the risk for active TB increases with greater induration size as in HIV-negative individuals (i.e., the shape of the tuberculin response curve is similar to that for the general population). To take into account the false-negative TST response in HIV cases with profound immune suppression, we first computed the proportion of HIV-positive individuals with CD4 counts <200 cells/mm for the 0mm induration group using our HIV prevalence estimates for that particular category. We then multiplied that proportion by the relative risk of developing active TB disease in the 0mm induration group compared with the 20+ mm induration group among HIV positive individuals. The relative risk was computed using data from a prospective, multicenter cohort study of HIV-positive people in the United States.[3]

Using the risk-weighted LTBI prevalence (adjusting for a false-negative TST among people with advanced HIV infection) as input data, we ran a DisMod MR 2.2 model with three location-level covariates, namely, Socio-demographic Index (SDI), Summary Exposure Variable (SEV) scalar for TB (a summary variable of the exposure levels of TB risk factors weighted by relative risk), and age-standardized TB mortality rate, to generate risk-weighted LTBI prevalence by location, year, age and sex. We included two study covariates (BCG positive, and mixed BCG status) where the reference category is BCG negative. We found no statistically significant difference between studies using different dosages of tuberculin purified protein derivative (PPD). We therefore did not include different PPD dosages as study covariates but added more uncertainty to data points from studies that used dosages larger or smaller than the standard dose of 5 tuberculin units per test dose of 0.1 ml, by entering them as z-covariates in DisMod.

Modeling TB incidence

Incidence inputs were from two different sources: (1) incidence from notification data for countries with a four or five-star rating on their cause of death data[4] as a proxy for the quality of health-related administrative data systems, and (2) estimated incidence for countries with a less than four-star rating. We used the age and sex-specific notifications (all new and relapse cases combined) in our analysis. Prior to 2013, notification data were available by case type (new pulmonary smear-positive, new pulmonary smear-negative, and new extra-pulmonary) and there were missing age data especially for younger age-groups in some countries. We imputed the missing age-groups for the three forms of TB notifications. Smear-positive age-specific notifications were inflated with the proportion smear-unknown and relapsed cases only reported at the country-year level. Some countries reported only pulmonary smear-positive cases for selected years. Missing smear-negative and extra-pulmonary cases were predicted from the adjusted smear-positive cases using a seemingly unrelated regression. All three types of notifications were added together to represent TB-all form incidence for countries with a four or five-star rating.

To generate incidence estimates for locations with a less than four-star rating, we ran a regression using MI ratios (logit transformed) from locations with a 4 or 5-star rating on causes of death as input data with SDI as a covariate anchoring the lower end of the SDI scale with a data point from a cohort study in the 1960s[2] reporting that 49.2% of 126 untreated new pulmonary TB cases were dead at the end of the 5-year follow up period, in order to predict age-sex specific MI ratios for all locations and years. We then used the predicted MI ratios and cause specific mortality estimates to compute age-sex specific incidence estimates for locations with a less than four-star rating. For South Africa, a country with large inequality, we decided that the Health Care Access and Quality (HAQ) index would be a better health-related index than SDI for TB, a health outcome that differentially affects the poor. We therefore used the HAQ index instead, to predict incidence for South Africa. While the MI-ratios predicted using the SDI covariate were within a reasonable range for most countries with a less than four-star rating, there were some outliers with very high MI ratios. We replaced those MI ratios with the MI ratios computed based on notifications and CSMR for 2010. For outliers in other years, we assumed a similar proportional difference between predicted MI ratios and notifications-based MI ratios as in 2010 and adjusted the predicted MI ratios accordingly, which were then used to predict incidence.

We computed the age-sex specific incidence of TB among the latent TB-infected population, using TB incidence as the numerator and our estimated risk-weighted latent TB infection prevalence as the denominator. We included location-level covariates, namely, the age-standardized adult underweight prevalence, and the log-transformed age-standardized SEV scalar for TB to help inform variation over year and geography. We set bounds of 0.75 to 1.25 on the SEV scalar covariate where a value in log space of 1 would reflect perfect agreement with our risk factor estimates.

Modeling TB prevalence

Data from prevalence surveys reporting on pulmonary smear-positive TB and bacteriologically positive TB were included. Because incidence data are for all forms of TB, we adjusted prevalence surveys to account for extra-pulmonary cases. We ran a spatiotemporal Gaussian process regression to predict location-year-age-sex specific proportions of extra-pulmonary TB among all TB cases using data on the three forms of TB from the incidence data above. We then computed the extra-pulmonary inflation factor as *1+(proportion of extrapulmonary TB /(1- proportion of extrapulmonary TB))*, and applied it to data from prevalence surveys. We then computed the prevalence of TB among the TB-infected population, using TB prevalence as the numerator and our estimated risk-weighted LTBI prevalence as the denominator. We included a study covariate indicating whether it was bacteriologically positive TB (reference category) or smear-positive TB. We found no systematic bias between studies that used both symptoms and chest X-ray as screening methods and studies that used only one of the methods. We therefore did not adjust them for systematic bias but added more uncertainty to data points from studies that used only one of the screening methods (by using it as a z-covariate in Dismod). We also added more uncertainty to data points from sub-national surveys. We included the SEV scalar country-level covariate with priors that as the SEV scalar increases, prevalence increases.

Modeling TB excess mortality

We matched each prevalence data point and TB CSMR (TB and HIV-TB combined) by location, year, age, and sex to calculate excess mortality rate (EMR) as *EMR=CSMR/prevalence*. We also matched each incidence data point and TB CSMR by location, year, age, and sex to calculate EMR for countries with a four or five-star rating on their cause of death data. To reflect a gradient in EMR, we added the HAQ index, and adult HIV death rates as country-level covariates.

DisMod-MR 2.1

For each location, we included the following as input in the DisMod model: case notifications for locations with a four or five-star rating, predicted MI-ratio-based incidence for locations with a less than four-star rating, prevalence survey data where available, excess mortality estimates, and CSMR (TB and HIV-TB combined) by age and sex.

The output from the DisMod model was for all forms of TB in TB-infected population including both HIV-negative and HIV-positive individuals. We computed the incidence and prevalence of TB among the entire population, by multiplying the prevalence of LTBI with the DisMod model estimates.

Betas and exponentiated values from the DisMod model are shown in the table below.

Covariate	Parameter	Beta (95% CI)	Exponentiated beta (95% CI)
Smear positive TB	Prevalence	-0.75	0.47 (0.47 — 0.47)
Sex (male)	Prevalence	0.51	1.66 (1.55 — 1.79)
Sex (male)	Incidence	0.13	1.14 (1.14 — 1.14)
Age-standardized proportion adult underweight	Incidence	2.23	9.35 (8.73 — 9.72)
Age-standardized proportion adult underweight	Prevalence	2.95	19.13 (17.32 — 20.07)
Age-standardized SEV scalar (log-transformed)	Prevalence	0.78	2.19 (2.12 — 2.39)
Age-standardized SEV scalar (log-transformed)	Incidence	0.75	2.12 (2.12 — 2.12)
HAQ (log-transformed)	Excess mortality	-1.58	0.21 (0.19 — 0.22)
Adult HIV death rate	Excess mortality	0.96	2.61 (1.04 — 7.02)

HIV-TB incidence and prevalence

To distinguish HIV-TB from all forms of TB, we first estimated the proportions of HIV-TB cases among all TB cases using data on the number of TB cases recorded as HIV-positive and the number of TB cases with an HIV test result recorded in the WHO TB notifications register. We ran a mixed effects regression using

the adult HIV death rate as a covariate to predict location-year specific HIV-TB proportions, which were then applied to TB incident and prevalent cases from DisMod, to generate HIV-TB incident and prevalent cases by location and year. These cases were then age-sex split based on the age-sex pattern of estimated HIV prevalence by location-year to generate location-year-age-sex specific HIV-TB incident and prevalent cases.

Multidrug-resistant TB, extensively drug-resistant TB and drug-susceptible TB

We ran a spatiotemporal Gaussian process regression to predict the proportions of MDR-TB cases among all TB cases for all locations and years. The input data for this regression (i.e., weighted average of the proportions of new and previously treated cases with MDR-TB) were based on the number of MDR-TB cases among new TB cases, MDR-TB cases among previously treated TB cases, and the number of new and previously treated TB cases with drug sensitivity testing for isoniazid and rifampicin from routine surveillance and surveys reported to the World Health Organization. We then used the predicted proportions to MDR-TB cases among all TB cases, along with the HIV-TB and TB no-HIV incidence estimates, and the relative risk of MDR-TB associated with HIV infection from the literature[1] to compute the proportions of MDR-TB cases among HIV negative TB cases $\left(PnoHIV_{c,y,a,s}\right)$ by location, year, age, and sex using the following formula:

$$PnoHIV_{c,y,a,s} = \frac{MDR_{c,y}}{\left(1 + \left(RR\,\frac{HIVTB_{c,y,a,s}}{TBnoHIV_{c,y,a,s}}\right)\right)\,TBnoHIV_{c,y,a,s}}$$

where $MDR_{c,y}$ is the number of all MDR-TB cases among HIV-positive and HIV-negative individuals by location and year, *RR* is the relative risk of MDR-TB associated with HIV infection, $HIVTB_{c,y,a,s}$ is the number of HIV-TB incident cases by location, year, age, and sex, and $TBnoHIV_{c,y,a,s}$ is the number of TB no-HIV incident cases by location, year, age, and sex.

We then applied the predicted proportions of MDR-TB cases among HIV negative TB cases to our predicted HIV-negative TB incident and prevalent cases to generate MDR-TB incident and prevalent cases by location, year, age, and sex. Next, we subtracted MDR-TB cases from all HIV-negative TB cases to generate drug-susceptible TB cases by location, year, age, and sex. To distinguish XDR-TB from MDR-TB, we aggregated the XDR-TB cases and MDR-TB cases (with drug sensitivity testing for second-line drugs) up to the super-region level and calculated the super-region level proportions of XDR-TB among MDR-TB cases, which were then applied to MDR-TB cases in corresponding countries within the super-regions to produce XDR-TB cases by location, year, age, and sex. We linearly extrapolated XDR-TB prevalence and incidence back assuming the rates were zero in 1992, one year before 1993 when XDR-TB was first recorded in USA surveillance data.[5] Finally, we subtracted XDR-TB cases from MDR-TB cases to generate MDR-TB (without XDR) cases by location, year, age, and sex.

Multidrug-resistant HIV-TB, extensively drug-resistant HIV-TB and drug-susceptible HIV-TB

To split HIV-TB into MDR-HIV-TB and drug-susceptible HIV-TB, we first calculated the proportions of MDR-HIV-TB among all HIV-TB cases ($PHIV_{c,y,a,s}$) for each location, year, age, and sex using the following formula:

$$PHIV_{c,y,a,s} = PnoHIV_{c,y,a,s}RR$$

where $PnoHIV_{c,y,a,s}$ is the proportions of MDR-TB among all HIV-negative TB cases for each location, year, age, and sex and RR is the relative risk of MDR-TB associated with HIV infection. We then applied the predicted proportions of MDR-TB cases among HIV-TB cases to all HIV-TB case estimates to generate MDR-HIV-TB cases by location, year, age, and sex. Next, we subtracted MDR-HIV-TB cases from all HIV-TB cases to generate drug-susceptible HIV-TB cases by location, year, age, and sex. To separate out XDR- HIV-TB from MDR-HIV-TB, we applied the super-region level proportions of XDR-TB among MDR-TB cases, to MDR-HIV-TB cases in corresponding countries within the super-regions to produce XDR-HIV-TB cases by location, year, age, and sex. We linearly extrapolated XDR-HIV-TB prevalence and incidence back assuming the rates were zero in 1992, one year before 1993 when XDR-TB was first recorded in USA surveillance data.[5] Finally, we subtracted XDR-HIV-TB cases from MDR-HIV-TB cases to generate MDR-HIV-TB (without extensive drug resistance) cases by location, year, age, and sex.

Disability weights

The lay descriptions and disability weights for severity levels derived from the GBD Disability Weights study are shown below.

Health state Name	Lay description	Disability Weights (95% CI)
Tuberculosis, not HIV infected	has a persistent cough and fever, is short of breath, feels weak, and has lost a lot of weight	0.333 (0.224-0.454)
Tuberculosis, HIV infected	has a persistent cough and fever, shortness of breath, night sweats, weakness and fatigue and severe weight loss	0.408 (0.274-0.549)

For drug-susceptible TB, MDR-TB without extensive drug resistance, and XDR-TB, we used the same disability weight [0.333 (0.224-0.454)] as in non-HIV-infected TB. For drug-susceptible HIV-TB, MDR-HIV-TB without extensive drug resistance, and XDR-HIV-TB, we used the same disability weight [0.408 (0.274-0.549))] as in HIV-infected TB.

References

1. Mesfin YM, Hailemariam D, Biadglign S, Kibret KT. Association between HIV/AIDS and multi-drug resistance tuberculosis: a systematic review and meta-analysis. *PLoS One*. 2014 Jan 8;9(1):e82235.
2. Institute NT. Tuberculosis in a rural population of South India: a five-year epidemiological study. *Bulletin of the World Health Organization*. 1974;51(5):473.

3. Markowitz N, Hansen NI, Hopewell PC, Glassroth J, Kvale PA, Mangura BT, Wilcosky TC, Wallace JM, Rosen MJ, Reichman LB. Incidence of tuberculosis in the United States among HIV-infected persons. *Annals of internal medicine*. 1997 Jan 15;126(2):123-32.
4. GBD 2016 Mortality and Causes of Death Collaborators. Global, regional, and national age-sex specific mortality for 264 causes of death, 1980–2016: a systematic analysis for the Global Burden of Disease Study 2016. *The Lancet* (under review)
5. Centers for Disease Control and Prevention (CDC). Extensively Drug-Resistant Tuberculosis --- United States, 1993—2006. *MMWR*. 2007; 56(11);250-253

3.3.3 Malaria SDG Capstone Appendix

Flowchart

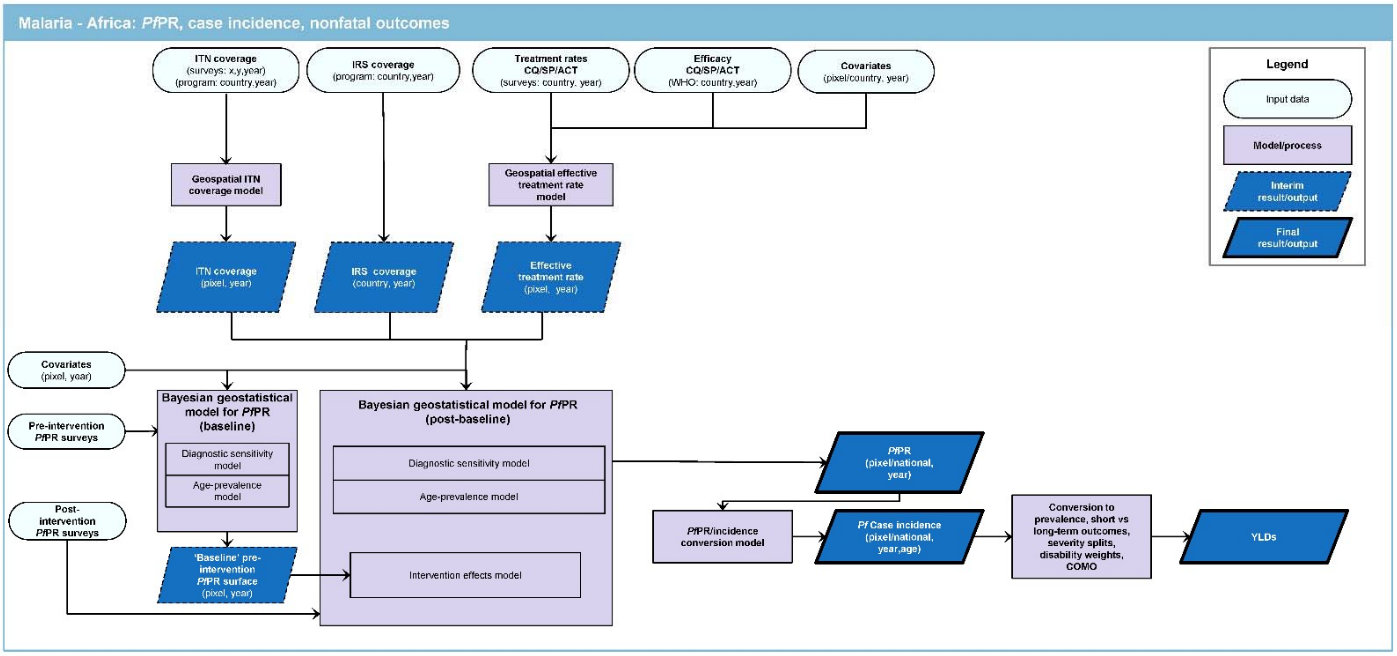

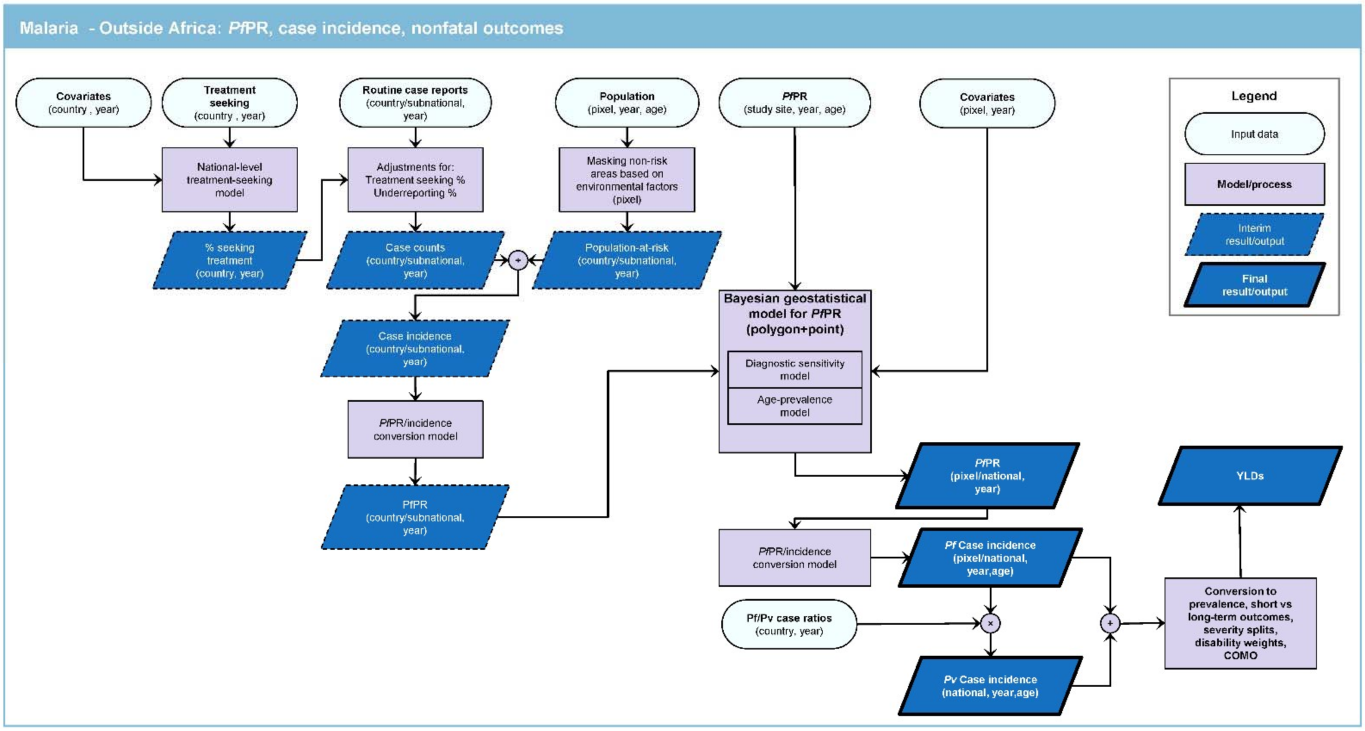

Input Data & Methodological Summary

Indicator definition

This modeling strategy encompassed the indicator associated with malaria incidence (3.3.3).

Indicator 3.3.3

As a component of SDG Goal 3. Ensure healthy lives and promote well-being for all at all ages, SDG Target 3.3, by 2030, end the epidemics of AIDS, tuberculosis, malaria and neglected tropical diseases and combat hepatitis, water-borne diseases and other communicable diseases, is measured using SDG Indicator 3.3.3, malaria cases per 1,000.

Case definition

Malaria is an acute parasitic mosquito-borne disease. An individual with uncomplicated malaria experiences one to two weeks of persistent fever, chills/shivering, sweating, joint pains and headache. The individual will likely be lethargic and feverish, causing loss of daily function during the attack. Individuals with an untreated *P. falciparum* infection may develop severe malaria, which includes the symptoms of uncomplicated malaria plus potentially swelling, difficulty breathing, unconsciousness, and death. Rapid diagnostic test or microscopy are considered the gold-standard diagnostic approaches for the purposes of GBD. The relevant ICD-10 codes are B50-B54.

Data input

Primary data inputs were:

1) Routine malaria case reports from national routine surveillance systems. These were obtained at national level from the WHO World Malaria Report and at the subnational administrative level, wherever possible, via an exhaustive search of published and grey literature sources along with online data portals hosted by national ministries of health. Each retained record consisted of an annual count of malaria cases along with breakdown by whether confirmed/unconfirmed and by malaria parasite species.

2) Cross-sectional geolocated community-representative observations of infection prevalence for Plasmodium falciparum (referred to hereafter as *P. falciparum* parasite rate, *Pf*PR).

These malaria epidemiological metrics were augmented in the modelling by:

3) Malaria Atlas Project (MAP) modelled estimates of malaria control intervention population coverage (ITNs, IRS, antimalarials) resolved to 5 km x 5 km pixel-year level (for Africa) and country-year level (outside Africa).

4) A large suite of environmental, sociodemographic, and economic covariates resolved to 5 km x 5 km pixel-year level (for Africa) and country-year level (outside Africa).

Modelling strategy

The suitability, availability, and quality of *Pf*PR and routine case reporting data, as well as detailed

intervention coverage information, differ markedly inside versus outside Africa. This meant we developed separate modelling strategy for countries inside Africa versus those outside. The exceptions were Algeria, Egypt, Morocco, Comoros, Mauritius, Cape Verde, Sao Tome, Principe, Rwanda, Botswana, Namibia, Eritrea, Djibouti, and South Africa which, despite being part of Africa, have epidemiologies and data availability/quality more akin to non-African settings.

*Pf*PR and case incidence modelling: Africa

Modelling was conducted in the following steps:

1) The large assembly of geolocated *Pf*PR surveys maintained by MAP was used in a Bayesian spatiotemporal geostatistical model to predict *Pf*PR for every pixel-year in sub-Saharan Africa, representing an update to earlier work (Bhatt et al Nature, Gething et al NEJM). The model took into account (i) *Pf*PR survey participant age ranges and diagnostic type; (ii) coverage of ITNs, IRS, and effective antimalarial drug coverage and how these changed through time at each data and prediction location; (iii) environmental conditions at each data and prediction location (including density of vegetation, temperature, humidity, rainfall, elevation, proximity to populated areas). The outcome was a predicted space-time "cube" of *Pf*PR, standardized to the 2-10 age range, for each year 1980–2016.

2) The *Pf*PR cube was then converted into an equivalent cube of the predicted incidence rate of clinical malaria. This conversion was achieved using an established model (Cameron et al Nature Communications) and allowed estimates stratified into three broad age bins (0-5; 5- 15; <15).

*Pf*PR and case incidence modelling: Outside Africa

Malaria endemic countries outside Africa tend to have less *Pf*PR data than those inside, in part because prevalence is generally lower and thus *Pf*PR becomes an inefficient way to measure malaria risk. Conversely, routine surveillance systems outside Africa are generally stronger, meaning that reports of malaria cases from health systems are more reliable and provide some insight into the total malaria burden in the community. Modelling outside Africa was carried out in the following steps:

1) National and subnational case reports were first subject to adjustments to identify and minimize bias. Bias in reported case numbers arises from various sources. First, a fraction of cases in the community will either seek no care or attend only a private or informal health care provider who would not provide a record of that case to the routine surveillance system. We adjusted for this by modelling the fraction of cases seeking care from different provider categories based on data from nationally representative cross-sectional household surveys (primarily from the Demographic and Health Survey (DHS) program and the Multiple Indicator Cluster Survey program). Second, cases reaching formal clinics may not be subject to a confirmatory diagnostic test. We adjusted for this by assuming the fraction of unconfirmed cases that were truly malaria would equal the fraction of positives among all those tested. Third, many routine surveillance systems fail to capture all case reports, with certain facilities/regions missing from the national totals in a given year. We adjusted for this based on reporting completeness statistics published nationally by WHO.

2) These adjusted routine case reports were georeferenced using digitized administrative boundary data using a large library of such boundaries maintained by MAP.

3) Each case report was converted to an estimate of clinical incidence rate by dividing over the estimated population at risk in each unit, with the latter quantity derived by combing high-

resolution gridded population data with MAP models that exclude malaria risk based on aridity or temperature ranges not conducive to transmission.

4) The incidence rate for each unit was then converted to an inferred *Pf*PR value using the same model described earlier (Cameron et al). This allowed us to then combine these data with the true *Pf*PR survey data that existed, albeit sparsely, in many countries outside Africa.

5) The combined *Pf*PR survey point data and (pseudo) *Pf*PR administrative unit data were then used in a Bayesian spatiotemporal geostatistical model to predict *Pf*PR at pixel-year level across all countries. As for the Africa model, *Pf*PR was standardized by age and diagnostic type and informed by a wide suite of covariates. An additional mechanism was developed to allow polygon (ie, administrative unit) and point (ie, survey) data to be used jointly to infer the predicted space-time surfaces.

6) As in Africa, the predicted *Pf*PR cube was then converted into an equivalent cube of the predicted incidence rate of clinical malaria.

Total malaria cases by country, year, sex

The pixel-level predictions of clinical incidence rate (both inside and outside Africa) were combined with high-resolution gridded population data to estimate total cases per pixel-year. These were then aggregated to GBD national/subnational geographies. For countries endemic for *P. vivax* and *P. falciparum*, we calculated the number of cases due to *P. vivax* applying the fraction of *P. vivax* and *P. falciparum* obtained from WHO and literature review. Total cases estimated in the MAP age bins were then redistributed to standard GBD age bins using the age pattern learned during the mortality/CoD estimation process (discussed in more detail in the GBD 2016 CoD paper).

Determining YLDs for malaria

As in GBD 2015, we use a two-step process for determining malaria severity. For acute cases, severity splits for mild, moderate, and severe malaria were produced by analysis of MEPS data. These sequelae and their associated disability weights are presented below.

Table 1. Severity level, lay description, and DW

Severity level	Lay description	DW (95% CI)
Mild	Has a low fever and mild discomfort but no difficulty with daily activities.	0.006 (0.002–0.012)
Moderate	Has a fever and aches and feels weak, which causes some difficulty with daily activities.	0.051 (0.032–0.074)
Severe	Has a high fever and pain and feels very weak, which causes great difficulty with daily activities.	0.133 (0.088–0.19)

To determine long-term neurological burden due to malaria, we use the work by Roca-Felter et al. (2008) that examined the number of uncomplicated cases that led to longer-term impairment. Analytically, this means multiplying incidence estimates (described in the section below) for persons under 20 by 0.00029 (0.000077–0.00057). This subset is then combined with excess mortality rates

derived from all-cause mortality and standardized mortality ratios for neonatal encephalopathy (NE) in a DisMod model to produce prevalence estimates for all estimation years. Implicit in this process is an assumption that the disability and trend of impairment due to severe malaria follow NE. The subsequent severity splitting follows NE as well. Once the incidence estimation procedures were completed, the results were combined and converted to prevalence by matching each draw with a draw of duration. Consistent with GBD 2015, we use a uniform distribution between 14 and 28 days for duration.

References

Bhatt S, Weiss DJ, Cameron E, et al. The effect of malaria control on Plasmodium falciparum in Africa between 2000 and 2015. Nature 2015; 526: 207–11.

Cameron, E., K.E. Battle, S. Bhatt, D.J. Weiss, D. Bisanzio, B. Mappin, U. Dalrymple, S.I. Hay, D.L. Smith, J.T. Griffin, E.A. Wenger, P.A. Eckhoff, T.A. Smith, M.A. Penny, and P.W. Gething. 2015. Defining the relationship between infection prevalence and clinical incidence of Plasmodium falciparum malaria. Nature Communications 6:8170.

Gething, P.W., D.C. Casey, D.J. Weiss, D. Bisanzio, S. Bhatt, E. Cameron, K.E. Battle, U. Dalrymple, J. Rozier, P.C. Rao, M.J. Kutz, R.M. Barber, C. Huynh, K.A. Shackelford, M.M. Coates, G. Nguyen, M.S. Fraser, R. Kulikoff, H. Wang, M. Naghavi, D.L. Smith, C.J.L. Murray, S.I. Hay, and S.S. Lim. 2016. "Mapping Plasmodium falciparum Mortality in Africa between 1990 and 2015." New England Journal of Medicine 375 (25):2435-2445.

3.3.4 Hepatitis B SDG Capstone Appendix

Acute hepatitis B

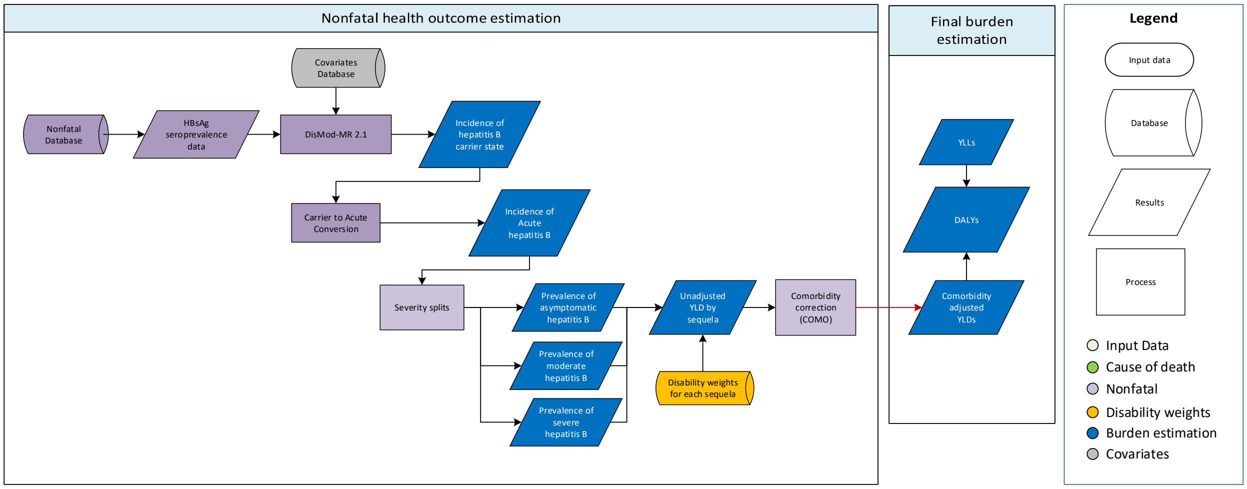

Input data and methodological appendix

Indicator definition

This modeling strategy encompassed the indicator associated with hepatitis B incidence (3.3.4).

Indicator 3.3.4

As a component of SDG Goal 3. Ensure healthy lives and promote well-being for all at all ages, SDG Target 3.3, by 2030, end the epidemics of AIDS, tuberculosis, malaria and neglected tropical diseases and combat hepatitis, water-borne diseases and other communicable diseases, is measured using SDG Indicator 3.3.4, hepatitis B incident cases per 100,000*).*

Case definition

We define acute hepatitis B as the period corresponding to initial infection with the hepatitis B virus, regardless of symptoms. It includes all ICD-10 codes under the heading B16 (Acute hepatitis B). Appendix Table 4 offers additional information on ICD codes.

Input data

Model inputs

We use hepatitis B surface antigen (HBsAg) seroprevalence data from population-based studies and surveys for the incidence model.

Updates to systematic reviews are performed on an ongoing schedule across all GBD causes, and an update for hepatitis A will be performed in the next one to two iterations.

Modelling strategy

We model the incidence of chronic HBsAg carriage using a full DisMod model of HBsAg seroprevalence. We then convert incidence of chronic carriage to total incidence of hepatitis B infection by dividing age-specific estimates of the incidence of chronic carriage by age-specific estimates of the probability of infection resulting in carriage based on Edmunds et al. (1993). The equation is detailed below:

$$P(carrier \mid age \leq 6\ months) = 0.885$$

$$P(carrier \mid 6\ months \leq age < 25\ years) = e^{-0.645 \times age^{0.455}}$$

$$P(carrier \mid age \geq 25\ years) = e^{-0.645 \times 25^{0.455}} = 0.061$$

We then split symptomatic cases into moderate (73%) and severe (27%) severities based on data from McMahon et al. (1985). See references and GHDx source tool for additional information.

Sequela, lay descriptions, and disability weights

The table below illustrates the sequelae, lay descriptions, and DWs associated with acute Hep B.

Sequela	Description	DWs (95% CI)
Moderate	Has a fever and aches, and feels weak, which causes some difficulty with daily activities.	0.051 (0.032–0.074)
Severe	Has a high fever and pain, and feels very weak, which causes great difficulty with daily activities.	0.133 (0.088–0.19)
Asymptomatic	Infection with no apparent illness.	NA

Changes from GBD 2015 to GBD 2016

We have updated the severity splits, but the modelling strategy remains otherwise unchanged from GBD 2013.

3.3.5 Neglected Tropical Diseases (NTDs) SDG Capstone Appendix

African trypanosomiasis, Chagas disease, cystic echinococcosis, cysticerosis, dengue, food-borne trematodiases, intestinal nematode infections, leishmaniosis, leprosy, lymphatic filariasis, onchocerciasis, rabies, schistosomiasis, and trachoma

Indicator definition

This modeling strategy encompassed the indicator associated with neglected tropical disease prevalence (3.3.5).

Indicator 3.3.5

As a component of SDG Goal 3. Ensure healthy lives and promote well-being for all at all ages, SDG Target 3.3, by 2030, end the epidemics of AIDS, tuberculosis, malaria and neglected tropical diseases and combat hepatitis, water-borne diseases and other communicable diseases, is measured using SDG Indicator 3.3.3, prevalence of neglected tropical diseases.

3.3.5 Human African Trypanosomiasis (HAT) SDG Capstone Appendix

Flowchart

African trypanosomiasis

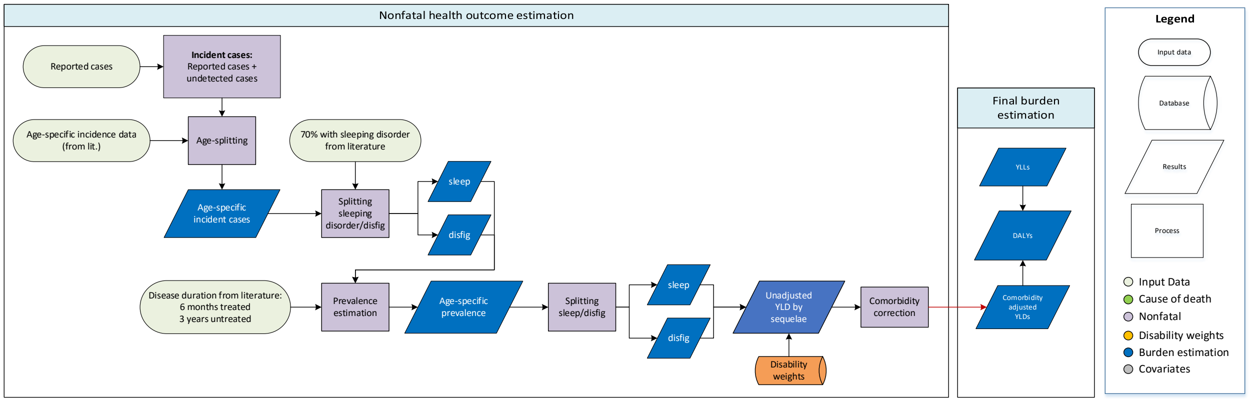

Input Data & Methodological Summary

Case Definition

Human African trypanosomiasis (HAT), also known as sleeping sickness, is a vector-borne disease which is transmitted by the bite of the tsetse fly. It is caused by the parasite *Trypanosoma brucei* with two subspecies, namely *T.b. rhodesience* (makes up less than 5% of total HAT cases) and *T.b. gambiense*. Cases are diagnosed through laboratory methods which rest on finding the parasite in body fluid or tissue by microscopy. In highly endemic or epidemic areas where the likelihood of false positives in serological tests is deemed lower, a seropositive individual is considered affected even in the absence of parasitological confirmation. The ICD-10 codes for HAT are B56.0, B56.1 and B56.9.

Input data

Model inputs

The input data for GBD 2016 included a) population at risk estimates from GBD 2010 ArcGIS analysis using geocoded case notifications for 2000 to 2009 [1] and population Count Grid estimates from Gridded Population of the World 3 [2, 3], b) population screened from 1997 to 2004 [4], c) historical data from GBD 2010 on total number of HAT cases reported [1, 4, 5], and d) cases reported annually to WHO [6] – for Kenya, a study on cases reported subnationally [7] was used to split the national cases into five counties (HomaBay, Migori, Busia, Bungoma, Kakamega). A systematic review of literature was conducted in PubMed on 8/10/2016 using the following search string:

> ((African trypanosomiasis[Title/Abstract] AND incidence[Title/Abstract]) AND (“2009”[Date – Publication] : “2013”[Date – Publication])).

This yielded 72 studies of which only four met the inclusion criteria and were extracted. The inclusion criteria were:

1. Studies representative of the national population
2. Population-based studies
3. Studies with primary data on incidence
4. Studies of human African trypanosomiasis only (excluded studies on animal African trypanosomiasis)

The four studies extracted had national incidence data similar to the ones extracted from WHO [6]. Therefore, three studies with age-specific incidence data from active screening undertaken in the Democratic Republic of Congo [8] and Uganda [9, 10] were used to inform age pattern for incidence and prevalence. Location-years with missing reported cases were excluded and five subnational locations for Kenya were added. The table below shows the number of studies included, and the number of countries or subnational units and GBD world regions represented.

	incidence
Studies	2
Countries/subnationals	34
GBD world regions	4

Severity splits/sequelae

The basis of the GBD disability weight (DW) survey assessments are lay descriptions of sequelae highlighting major functional consequences and symptoms. The lay descriptions and disability weights for sequelae due to HAT are shown below.

Sequela	Lay description	DW (95% CI)
Skin disfigurement, level 1	Has a slight, visible physical deformity that is sometimes sore or itchy. Others notice the deformity, which causes some worry and discomfort.	0.027 (0.015–0.042)
Motor plus cognitive impairments, severe	Cannot move around without help, and cannot lift or hold objects, get dressed or sit upright. The person also has very low intelligence, speaks few words, and needs constant supervision and help with all daily activities	0.542 (0.37–0.702)

Modelling strategy

The non-fatal model for HAT involved estimating prevalence from incidence. First, a multi-level mixed-effects linear regression of natural log-transformed incidence rate (ratio of HAT cases reported to population at risk) on natural log-transformed screening coverage (ratio of number screened for HAT to population at risk), with country random effects, was performed. Gaps were then filled using exponential interpolation between years and extrapolation from 2014 to 2015 for reported cases; for screening coverage only extrapolation from 2014 to 2015 was done. Then 1,000 draws of mortality among treated cases were generated, assuming that 0.7%–6.0% of all treated (reported) cases die [11, 12, 13].

Using the mean and variance-covariance matrix from the regression as parameters, a multivariate normal distribution was used to generate 1,000 draws of case detection rate (CDR), given the expected screening coverage. Undetected deaths were then estimated as the difference between the ratio of reported cases to CDR and reported cases (reported cases/CDR – reported cases). Estimates of incidence were obtained by adding the reported cases to the undetected cases. Without information on sex-specific incidence, equal incidence rates between both sexes was assumed. Finally, an age-pattern was applied to the incidence estimates using the incidence studies from DRC and Uganda [8, 9, 10]. Assuming the same proportion in treated and untreated cases, the incidence estimates were then split into the two sequelae, skin disfigurement and sleeping disorder. This was done by generating 1,000 draws of the splitting proportion for the sequelae (70%–74% with sleeping disorder) based on a study that reported presence of symptoms at admission of patients in treatment centers [14] – draws were generated from a beta distribution with alpha parameter = 1884 and beta parameter = 649.

To compute prevalence of HAT, 1,000 draws of total duration of symptoms in untreated cases was generated from a normal distribution with mean = {ln(3) – 0.5 * sigma^2}, and standard deviation = sigma, where sigma = {ln(4.39)-ln(1.92))/(invnormal(0.975)*2)} – these parameters were based on a study of *T.b. gambiense* [14] which estimated an average duration of three years to untreated cases. An estimated duration of six months was applied to cases that received treatment, based on findings from a paper about *T.b. rhodesiense* in Uganda [12]. Prevalence was then estimated from the incident cases before applying age pattern. Prevalence of treated and untreated cases was summed up, assuming that untreated cases have been prevalent up to their death for a certain duration. For untreated cases, it was assumed that half the duration is spent with sleeping disorder (severe motor and cognitive impairment) and disfigurement [14]. Treated (ie, reported) cases are assumed to have been prevalent for 0.5 years, and for the fraction of treated cases that present with sleeping disorder, it was assumed that this is present for half the total duration and that the rest of the duration is spent suffering from disfiguring skin disease. Treated cases that don't present with sleeping disorder were assigned disfigurement for the entire duration. Lastly, an age-pattern using a cubic spline was applied to the prevalence estimates using the incidence studies from DRC and Uganda [8, 9].

Results from the model were assessed by visualizing time trends of incident and prevalent cases across locations and age (similar trends were applied in both sexes). Maps of the global distribution of HAT and the two sequelae were also generated. In addition, the estimated incident cases were compared with the cases reported to the WHO across time – as expected, the estimates from GBD 2015 were higher than the WHO numbers because we accounted for undetected cases.

Changes from GBD 2013 included: a) inclusion of new data on reported cases from WHO [6] (years 2013 and 2014 for 23 locations), b) inclusion of the following country (years) based on available historical data post-1980: Botswana (1983), Ethiopia (1980–1983), Guinea-Bissau (1980–1983, 1985–1987), Rwanda (1980, 1982–1988), and Sierra Leone (1981–1982), c) adding five subnational locations (out of 49) for Kenya, thus correcting the age-split proportion such that a 0.32/0.68 proportion was used for adults/children – in GBD 2013, this proportion was 0.25/0.75 for adults/children.

Changes from GBD 2015 to GBD 2016

We have made no substantive changes in the modelling strategy from GBD 2015 to GBD 2016.

References

1. Simarro P, Cecchi G, Paone M, Franco J, Diarra A, Ruiz J, Fevre E, Courtin F, Mattioli R, Jannin J. The Atlas of human African Trypanosomiasis: a contribution to global mapping of neglected tropical diseases. International Journal of Health Geographics, 2010. 9:57.
2. Center for International Earth Science Information Network (CIESIN), Columbia University; and Centro Internacional de Agricultura Tropical (CIAT). 2005. Gridded Population of the World, Version 3 (GPWv3): Population Count Grid. Palisades, NY: Socioeconomic Data and Applications Center (SEDAC), Columbia University. Available at http://sedac.ciesin.columbia.edu/gpw.
3. Center for International Earth Science Information Network (CIESIN), Columbia University; United Nations Food and Agriculture Programme (FAO); and Centro Internacional de Agricultura Tropical (CIAT). 2005. Gridded Population of the World, Version 3 (GPWv3): Population Count Grid, Future Estimates. Palisades, NY: Socioeconomic Data and Applications Center (SEDAC), Columbia University. Available at http://sedac.ciesin.columbia.edu/gpw.
4. WHO. Weekly epidemiological record. 2006, February 24. No. 8, 2006, 81. 69-80.
5. WHO Department of Communicable Disease Surveillance and Response (CDS). WHO Report on Global Surveillance of Epidemic-prone Infectious Diseases. 2000. WHO/CDS/CSR/ISR/2000.1.
6. WHO Global Health Observatory Data Repository (http://apps.who.int/gho/data/node.main.A1635?lang=en). Accessed Sept. 2015.
7. Ruto JJ, Karuga JW. Temporal and spatial epidemiology of sleeping sickness and use of geographical information system (GIS) in Kenya. J Vector Borne Dis 2009; 1. 18-25.
8. Lutumba P, Makieya E, Shaw A, Meheus F, Boelaert M. Human African Trypanosomiasis in a Rural Community, Democratic Republic of Congo. Emerging Infectious Diseases, 2007. Vol 13: No.2, 248-254.
9. Fevre E, Odiit M, Coleman P, Woolhouse M, and Welburn S. Estimating the burden of rhodesiense sleeping sickness during an outbreak in Serere, eastern Uganda. BMC Public Health 2008, 8:96.
10. Kato CD, Nanteza A, Mugasa C, Edyelu A, Matovu E, Alibu VP. Clinical profiles, disease outcome and co-morbidities among T. b. rhodesiense sleeping sickness patients in Uganda. *PLoS One*. 2015; **10**: e0118370.
11. Balasegaram M Harris S, Checchi F, Ghorashian S, Hamel C, Karunakara U. Melarsoprol versus eflornithine for treating late-stage Gambian trypanosomiasis in the Republic of Congo. Bulletin of the World Health Organization 2006;84:783-791.
12. Odiit M, Kansiime F, Enyaru JCK. Duration of symptoms and case fatality of sleeping sickness caused by Trypanosoma brucei rhodesiense in Tororo, Uganda. East African Medical Journal. December 1997. 792-795.
13. Priotto G, Kasparian S, Mutombo W, Ngouama D, Ghorashian S, Arnold U, Ghabri S, Baudin E, Buard V, Kazadi-Kyanza S, Ilunga M, Mutangala W, Pohlig G, Schmid C, Karunakara U, Torreele E, Kande V. Nifurtimox-eflornithine combination therapy for second-stage African Trypanosoma brucei gambiense trypanosomiasis: a multicentre, randomised, phase III, non-inferiority trial. Lancet 2009; 374. 56-64.
14. Blum J. Scmid C, Burri C. Clinical aspects of 2541 patients with second stage human African trypanosomiasis. Acta Tropica 97. 2006. 55-64.
15. Checchi F, Filipe J, Haydon D, Chandramohan D, and Chappuis F. Estimates of the duration of early and late stage of gambiense sleeping sickness. BMC Infectious Diseases. 2008. 8:16.

3.3.5 Chagas disease SDG Capstone Appendix

Flowchart

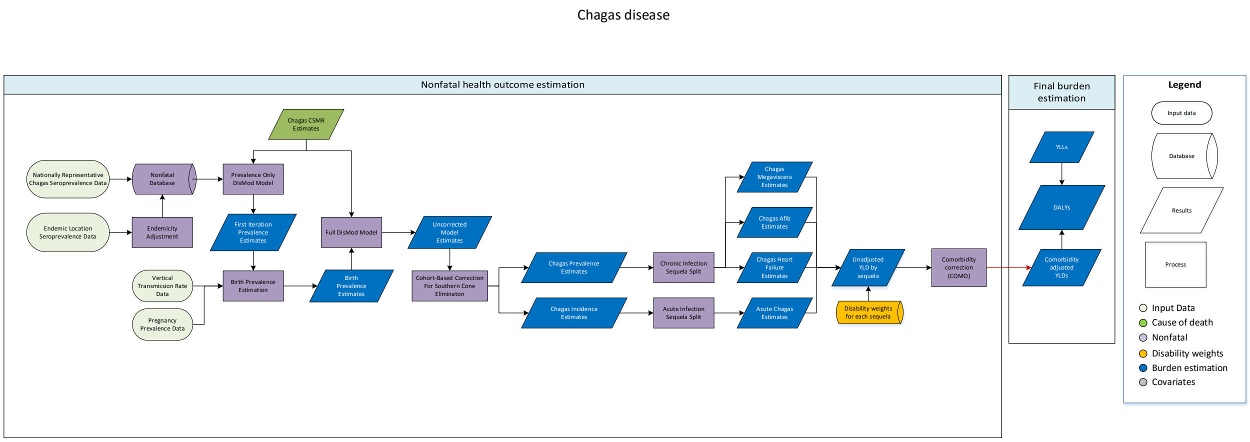

Case definition

Chagas disease is defined by infection with the protozoa *Trypanosoma cruzi,* which is transmitted by *Triatominae* insect vectors (most common), blood transfusion, organ transplant, and congenital transmission. It includes an acute phase corresponding with the time of infection, and is typically asymptomatic. Chronic infection may be latent (ie, asymptomatic), or result in cardiovascular or digestive sequelae. It includes all ICD-10 codes under the heading B57 (Chagas disease), with codes B57.0-B75.1 corresponding to the acute phase, B57.2 corresponding to chronic cardiovascular sequelae, and B57.3 corresponding to chronic digestive sequelae.

Input data

Model inputs

For GBD 2016 estimation, we used seroprevalence data to model Chagas. The table below illustrates the geographic distribution of model input data for the estimation process.

Table 1. Geographies

Level	Prevalence
Data points	407
Studies	56
Locations	20
Regions	4

We also use CSMR estimates in the modeling process, which will be addressed in further detail below.

Modelling strategy

We modeled Chagas disease using a full DisMod-MR 2.1 Bayesian meta-regression model incorporating seroprevalence data, as above, and CSMR estimates. We assume no remission. We eliminate all new infections, except those via vertical transmission, in Chile and Uruguay for years after the interruption of vector-based transmission (Abad-Franch F, Diotaiuti L, Gurgel-Gonçalves R, Gürtler RE. Certifying the interruption of Chagas disease transmission by native vectors: cui bono? Mem Inst Oswaldo Cruz 2013;108:251–4.; Coura JR. Chagas disease: control, elimination and eradication. Is it possible? Mem Inst Oswaldo Cruz 2013;108:962–7.). For non-endemic countries, we estimate the prevalence of imported chronic infections based on migration. For each non-endemic country, we estimate the total number of people infected with Chagas as the sum of the number of immigrants from each endemic country multiplied by the corresponding prevalence of Chagas in that endemic country.

We estimate five sequelae: symptomatic acute infection from incidence; and megaviscera, heart failure, atrial fibrillation, and chronic asymptomatic infection from prevalence. We assume that 5% of acute infections will be symptomatic (Teixeira AR, Nitz N, Guimaro MC, Gomes C, Santos-Buch CA. Chagas disease. Postgrad Med J 2006;82:788–98.). The proportion of chronic infections resulting in a given sequela varies by sex and age: the prevalence of megaviscera among those infected with Chagas ranges from 0% in children to nearly 10% among older adults (Coura JR, Naranjo MA, Willcox HP. Chagas' disease in the Brazilian Amazon: II. A serological survey. Rev Inst Med Trop São Paulo 1995; 37:103–7.); the prevalence of atrial fibrillation attributable to Chagas ranges from 0% among children to approximately 10% in men over 80 years of age (Ribeiro AL, Marcolino MS, Prineas RJ, Lima-Costa MF. Electrocardiographic abnormalities in elderly Chagas disease patients: 10-year follow-up of the Bambuí Cohort Study of Aging. J Am Heart Assoc 2014;3:e000632.); and the prevalence of heart failure attributable to Chagas among those who are infected ranges from 0% among young children, to a maximum of 23% among men over 80 years of age (Sabino EC, Ribeiro AL, Salemi VM, et al., for the National Heart, Lung, and Blood Institute Retrovirus Epidemiology Donor Study-II (REDS-II), International Component. Ten-year incidence of Chagas cardiomyopathy among asymptomatic Trypanosoma cruzi-seropositive former blood donors. Circulation 2013;127:1105–15.).

Severity splits and disability weights

The table below illustrates the sequelae, lay descriptions, and DWs for Chagas disease.

Table 2. Sequelae, lay description and DWs

Sequelae	Description	Disability Weight
Atrial fibrillation and flutter due to Chagas disease	Has periods of rapid and irregular heartbeats and occasional fainting.	0.224 (0.151–0.312)

Mild heart failure due to Chagas disease	Is short of breath and easily tires with moderate physical activity, such as walking uphill or more than a quarter-mile on level ground. The person feels comfortable at rest or during activities requiring less effort.	0.041 (0.026–0.062)
Moderate heart failure due to Chagas disease	Is short of breath and easily tires with minimal physical activity, such as walking only a short distance. The person feels comfortable at rest but avoids moderate activity.	0.072 (0.047–0.103)
Severe heart failure due to Chagas disease	Is short of breath and feels tired when at rest. The person avoids any physical activity, for fear of worsening the breathing problems.	0.179 (0.122–0.251)
Mild chronic digestive disease due to Chagas disease	Has some pain in the belly that causes nausea but does not interfere with daily activities.	0.011 (0.005–0.021)
Moderate chronic digestive disease due to Chagas disease	Has pain in the belly and feels nauseated. The person has difficulties with daily activities.	0.114 (0.078–0.159)
Acute Chagas disease	Has a fever and aches, and feels weak, which causes some difficulty with daily activities.	0.051 (0.032–0.074)
Asymptomatic Chagas disease	Latent Chagas infection (ie, chronic infection with no apparent symptoms)	NA

Changes from GBD 2015 to GBD 2016

We have made no substantive changes in the modelling strategy for endemic countries from GBD 2015 to GBD 2016.

3.3.5 Cystic Echinococcosis SDG Capstone Appendix

Flowchart

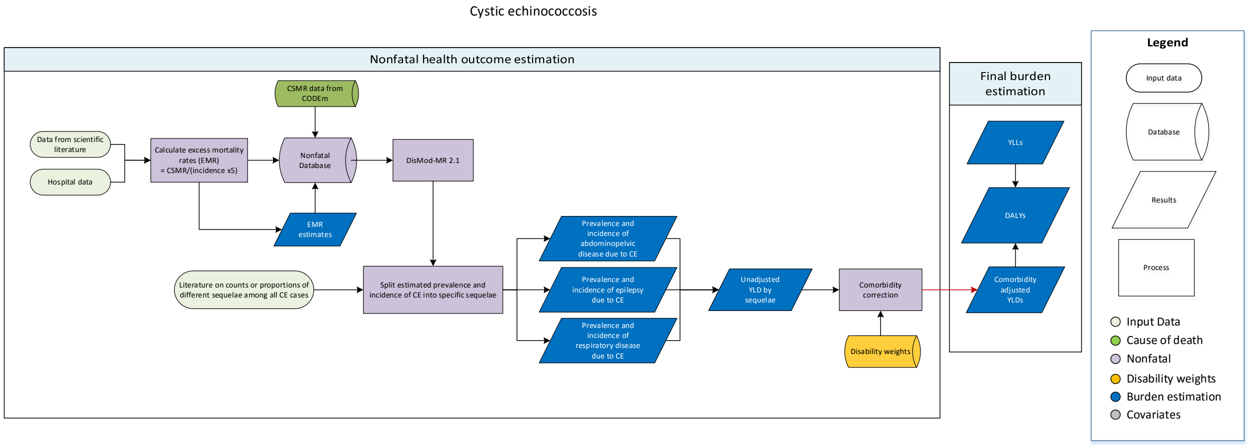

Input Data & Methodological Summary

Case definition

Cystic echinococcosis is a parasitic disease caused by infection with the *Echinococcus granulosis* tapeworm. It is a natural parasite of canines, with sheep being the most common intermediate host in the two-stage lifecycle, but can be spread to humans through ingestion of soil, water, or food contaminated with the fecal matter of an infected dog containing infective eggs. Diagnosis is made by clinical findings, imaging, serology, and tissue pathology. The ICD-9 and ICD-10 codes for echinococcosis are 122.0-122.9 and B67-B67.9, respectively.

Input data

Model inputs

The nonfatal estimation for cystic echinococcosis (CE) focused on estimating incidence and prevalence of CE and its sequelae. A systematic review of literature was conducted in PubMed for GBD 2015 using the following search string:

> ("echinococcosis"[Title/Abstract] OR "hydatid disease"[Title/Abstract] OR "hydatidosis"[Title/Abstract] OR "echinococcal disease"[Title/Abstract] OR "Echinococcus granulosus infection"[Title/Abstract]) AND ("1990"[Date – Publication] : "2015"[Date – Publication]) AND (epidemiology OR incidence OR prevalence).

This yielded 1,619 studies of which 279 were included during the title/abstract screening. Following the full-text screening, 77 studies (32 incidence, 43 prevalence and 2 both) were included and extracted – studies were excluded because of one or more of the following reasons:

1. study not population-based
2. study does not have primary data on prevalence and/or incidence
3. study not in humans

4. study on sub-populations
5. review study

Data from these extracted studies were combined with data from studies extracted during GBD 2013 and hospital data prepared by the GBD team.

Since we were interested in modelling symptomatic CE cases, we only used data on incidence of patients diagnosed by imaging techniques (mainly ultrasonography). Therefore, we excluded prevalence data which were mostly from serological studies.

The table below shows the number of studies finally included, and the number of countries or subnational units and GBD world regions represented.

	prevalence
Studies	84
Countries/subnationals	137
GBD world regions	16

Sequelae due to cystic echinoccocosis

The table below shows the sequelae due to echinococcosis and their associated disability weights.

Sequela	Lay description	DW (95% CI)
Chronic respiratory disease	has cough and shortness of breath after heavy physical activity, but is able to walk long distances and climb stairs.	0.019 (0.011–0.033)
Abdominal problems	has pain in the belly and feels nauseated. The person has difficulties with daily activities.	0.114 (0.078–0.159)
Epilepsy	(Combined DW)	NA

Modelling strategy

DisMod MR was used to model the nonfatal burden of symptomatic cystic echinococcosis (CE) using incidence data. The covariates included were sheep per capita; proportion of the population with access to sanitation; log-transformed lag-distributed income; and clinic or hospital data type.

Mortality estimates from the custom mortality model were used to inform the excess mortality parameter (CODEm estimates used as cause-specific mortality rate data). Estimates of excess mortality rate were obtained and used to estimate prevalence (CSMR/EMR). A remission of 0.15–0.25 per case per year (duration 2–6.7 years, average 5 years) was assumed. The following steps were followed to estimate excess mortality rate: 1) create custom age groups for CE deaths at the 1,000-draw level; 2) calculate CSMR as CSMR=deaths/population at the 1,000-draw level – calculate mean CSMR, uncertainty interval, and standard error; and 3) calculate EMR as EMR=CSMR/(prevalence), where prevalence = (incidence*5) – standard error of EMR was calculated taking into consideration the standard errors of both prevalence and CSMR.

After running DisMod, a thousand draws of proportions for abdominal, respiratory, and epileptic symptoms among echinococcosis cases, that add up to 1, were generated. Uncertainty in the splitting

proportions was captured by drawing them from a Dirichlet distribution, informed by published data on cysts localization [1]. On average, the proportions of abdominal, respiratory, and epileptic symptoms due to echinococcosis were 0.8, 0.19, and 0.01, respectively. These proportions were used to split the prevalence and incidence from DisMod into the three sequelae.

Model evaluation was done by separately assessing the fit of the DisMod MR model and checking the estimates produced after estimating incidence and prevalence of sequelae due to cystic echinococcosis. Plots of time trends of incidence and prevalence across locations and age were used to evaluate the results. In addition, maps of the global distribution of incidence and prevalence were assessed across time.

Changes from GBD 2015 to GBD 2016

We have made no substantive changes in the modelling strategy from GBD 2015 to GBD 2016.

References

1. Eckert J, Deplazes P. Biological, Epidemiological, and Clinical Aspects of Echinococcosis, a Zoonosis of Increasing Concern. *Clin Microbiol Rev*, 2004; 17(1): 107-35

3.3.5 Cysticercosis SDG Capstone Appendix

Flowchart

Cysticercosis

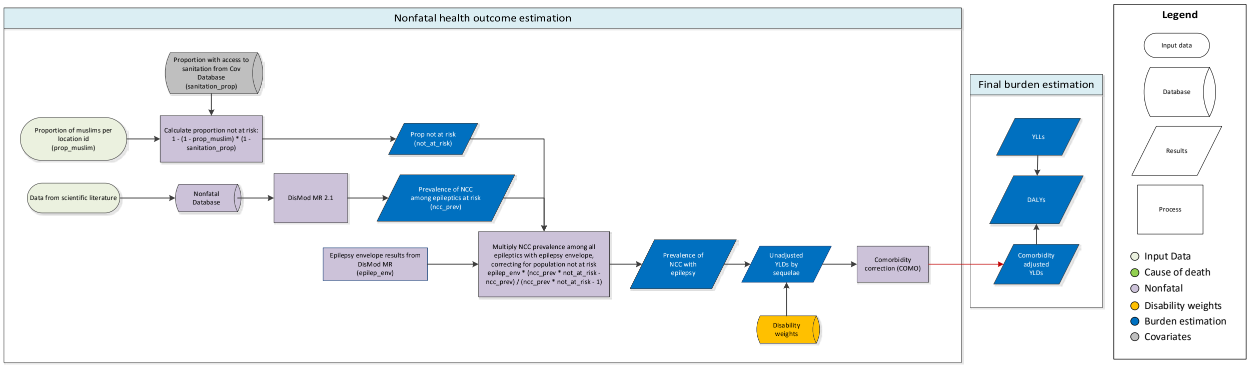

Input Data & Methodological Summary

Case Definition

Cysticercosis, or Neurocysticercosis (NCC), is a parasitic disease caused by the pig tapeworm, *Taenia solium.* It is transmitted via ingestion of eggs or gravid proglottids shed by a human or non-human host with an intestinal infection of the same helminth known as Taeniasis. In rare cases, auto-infection is also possible among people with intestinal infections. Diagnosis is made by magnetic resonance imaging (MRI) or computerized tomography (CT) brain scans to identify cysts. The ICD-10 codes for Cysticercosis are B69-B69.9.

Input data

Model inputs

The nonfatal estimation for cysticercosis focused on estimating prevalence of NCC among epileptics at risk as well as the prevalence of NCC with epilepsy. A systematic review of literature was conducted in PubMed for GBD 2015 using the following search string:

> ("cysticercosis"[Title/Abstract] OR "neurocysticercosis"[Title/Abstract] OR "cysticerciasis"[Title/Abstract] OR "Taenia solium"[Title/Abstract]) AND ("1990"[Date – Publication] : "2015"[Date – Publication]) AND (epidemiology OR prevalence)).

This yielded 1,038 studies of which 166 were included during the title/abstract screening. Following the full-text screening, 17 studies were included and extracted – studies were excluded because of one or more of the following reasons:

1. study not in epileptics
2. study not population-based
3. study does not have primary data on prevalence of NCC among epileptics at risk
4. study not in humans (some studies were on cysticercosis in pigs)

5. study on comorbidities with NCC (other than epilepsy)
6. study on sub-population, eg, patients with neurological disorders
7. review study

We combined the newly extracted studies with studies extracted during GBD 2013. The table below shows the number of studies finally included, and the number of countries or subnational units and GBD world regions represented.

	prevalence
Studies	31
Countries/subnationals	23
GBD world regions	8

A study-level covariate was also created in GBD 2015 to indicate the type of diagnosis for each study, ie, definitive or probable. Of the 77 rows of country-year-age-sex data, there were 15 rows with definitive diagnosis and 62 rows with probable diagnosis.

Three additional data sources that were used included 1) epilepsy envelope prevalence (from the epilepsy DisMod MR model), 2) proportion of the population with access to sanitation (from the GBD covariates database), and 3) proportion of the population that is Muslim (from the PEW Research Center [1].)(http://www.pewforum.org/2011/01/27/table-muslim-population-by-country/).

Modelling strategy

DisMod MR was used to model the prevalence of NCC among epileptics at risk. In the model, pigs per capita, the proportion of the population with access to sanitation and religion (binary, >50% Muslim) were used as country-level covariates. In addition, the prevalence of "definitive diagnosis" was transformed to that of "probable and definitive diagnosis" so as to not underestimate overall prevalence.

After running DisMod, we adjusted the fraction of people with epilepsy attributable to cysticercosis in endemic countries for the population at risk based on the proportion of the population without access to sanitation and the proportion of the population that is Muslim. Predicted NCC prevalence among epileptics at risk was calculated such that $Prevalence=P\times(NM-N)/(NM-1)$, where P = prevalence of all-cause epilepsy in total population, N = proportion of NCC among epileptics at risk (non-Muslims without access to sanitation), and M = proportion of population not at risk of contracting NCC. It was assumed that the prevalence of epilepsy due to causes other than NCC is the same regardless of whether a population is at risk or not. It was also assumed that Muslims and non-Muslims have equal access to sanitation.

Model evaluation was done by separately assessing the fit of the DisMod MR model and checking the estimates produced after estimating prevalence of NCC with epilepsy. Plots of time trends of prevalence across locations and age were used to evaluate the results. In addition, maps of the global distribution of prevalence of NCC among epileptics at risk and prevalence of NCC with epilepsy were also assessed across time.

Other than using additional data extracted from literature, we updated the proportion of population with Muslim data by filling in subnational locations with national proportions – this was done due to lack of data on this covariate at the subnational level.

Changes from GBD 2015 to GBD 2016

We have made no substantive changes in the modelling strategy from GBD 2015 to GBD 2016.

References:

1. "Table: Muslim Population by Country Pew Research Center, Washington, D.C." (March 26, 2014). http://www.pewforum.org/2011/01/27/table-muslim-population-by-country/

3.3.5 Dengue SDG Capstone Appendix

Flowchart

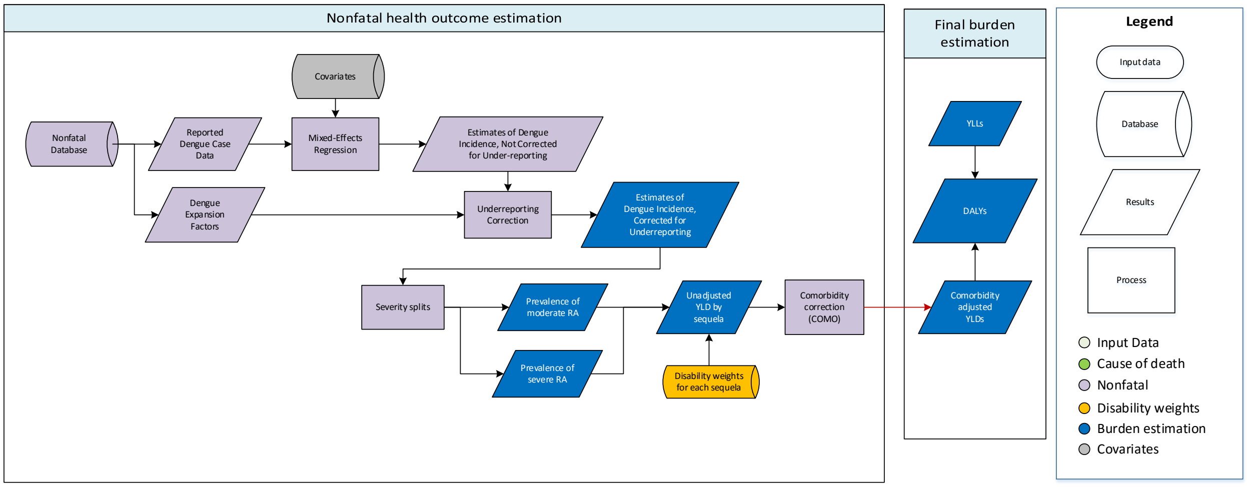

Case definition

Dengue is mosquito-borne viral infection that causes febrile illness and, in severe cases, jaundice, hemorrhage, and death. It includes all ICD-10 codes under the heading A90 (Dengue fever [classical dengue]) and A91 (Dengue hemorrhagic fever).

Input data

Model inputs

For GBD 2016, we modelled dengue incidence based on officially reported cases. The table below illustrates the geographic distribution of data points used in our analysis.

Table 1. Geographies

Level	Incidence
Data points	2,515
Studies	70
Locations	115
Regions	14

Updates to systematic reviews are performed on an ongoing schedule across all GBD causes, and an update for dengue fever will be performed in the next one to two iterations. While no systematic update

was conducted, we did incorporate new expansion factor data that were provided by collaborators and have updated to the latest available case reports for GBD 2016.

Modelling strategy

The methods used to model dengue incidence remain unchanged from GBD 2015, and are an improved variant of the methods used for GBD 2013 that were described by Stanaway et al. Briefly, we derive two dengue-specific covariates: first a variable to define the expected spatial distribution of the disease based on principal components analysis of dengue CSMR estimates and dengue transmission probability and, second, a variable to define the country-specific trends, based on a mixed-effects model of reported cases. We then estimate a mixed-effects negative binomial model with number of reported cases as the dependent variable, fixed effects on the aforementioned spatial and temporal covariates, and random effects on location. These random effects are assumed to correspond to deviations in reporting completeness and, calibrating against published expansion factor data (ie, estimates of the degree of underreporting), they are inflated to adjust for underreporting estimates. The resulting incidence estimates are split into moderate (94.5%) and severe (5.5%) sequelae, based on the proportion of reported cases that were severe. We assume that 8.4% of symptomatic infections will produce post-acute chronic fatigue lasting an average of six months (Teixeira L de AS, Lopes JSM, Martins AG da C, Campos FAB, Miranzi S de SC, Nascentes GAN. Persistence of dengue symptoms in patients in Uberaba, Minas Gerais State, Brazil. *Cad Saúde Pública* 2010; **26**: 624–30.).

Severity splits and disability weights

Table 2. Sequelae, lay descriptions, and DWs

Sequela	Lay description	Disability Weight (DW)
Moderate	Has a fever and aches, and feels weak, which causes some difficulty with daily activities.	0.051 (0.032–0.074)
Severe	Has a high fever and pain, and feels very weak, which causes great difficulty with daily activities.	0.133 (0.088–0.19)
Asymptomatic	Infection with no apparent illness.	NA

Changes from GBD 2015 to GBD 2016

We have made no substantive changes in the modelling strategy from GBD 2015 to GBD 2016.

References

1. Stanaway JD, Shepard DS, Undurraga EA, Halasa YA, Coffeng LE, Brady OJ, et al. The global burden of dengue: an analysis from the Global Burden of Disease Study 2013. *The Lancet Infectious Diseases* [Internet]. 2016 Feb [cited 2016 May 23].

2. Bhatt S, Gething PW, Brady OJ, Messina JP, Farlow AW, Moyes CL, et al. The global distribution and burden of dengue. *Nature*. 2013 Apr 25;496(7446):504–7.

3.3.5 Foodborne Trematodiases SDG Capstone Appendix

Flowcharts

Clonorchiasis

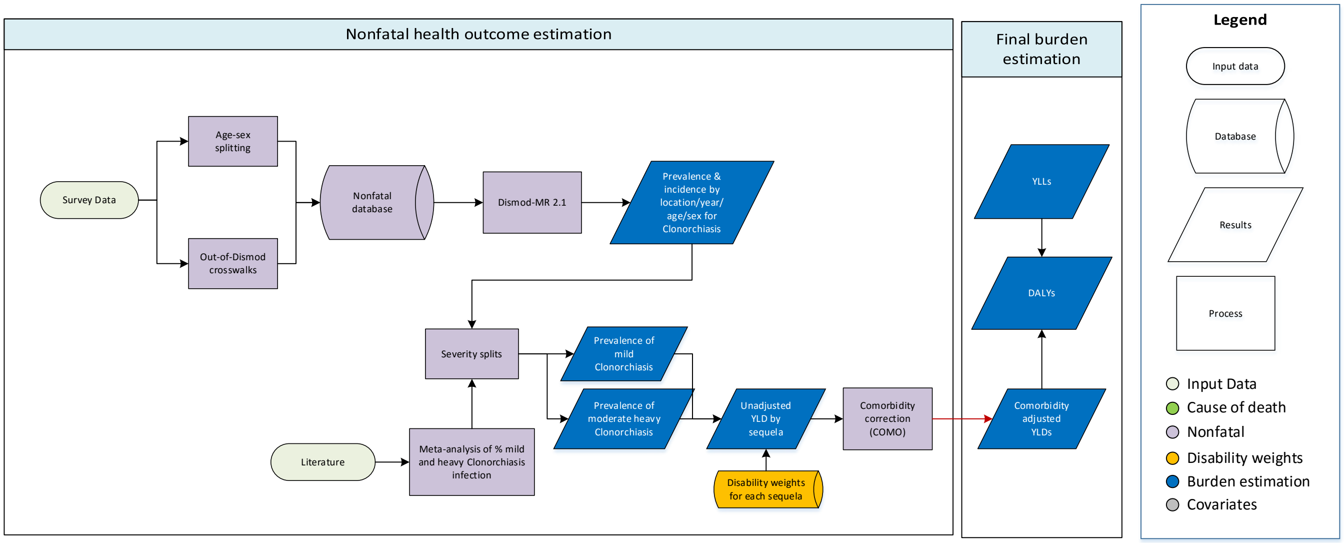

Fascioliasis

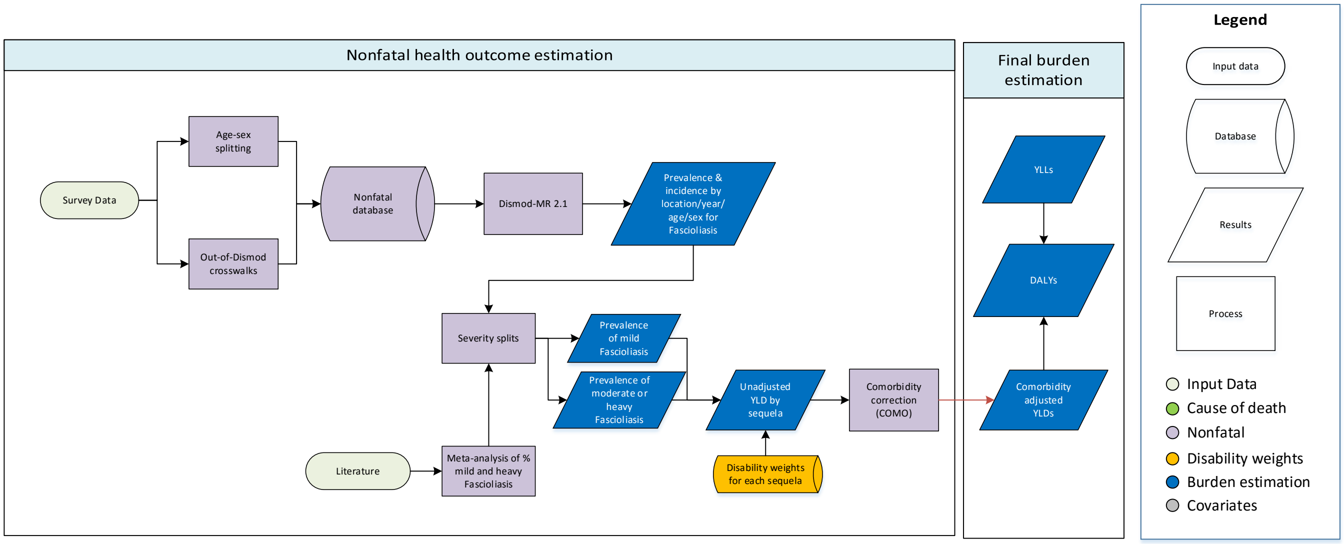

Intestinal fluke

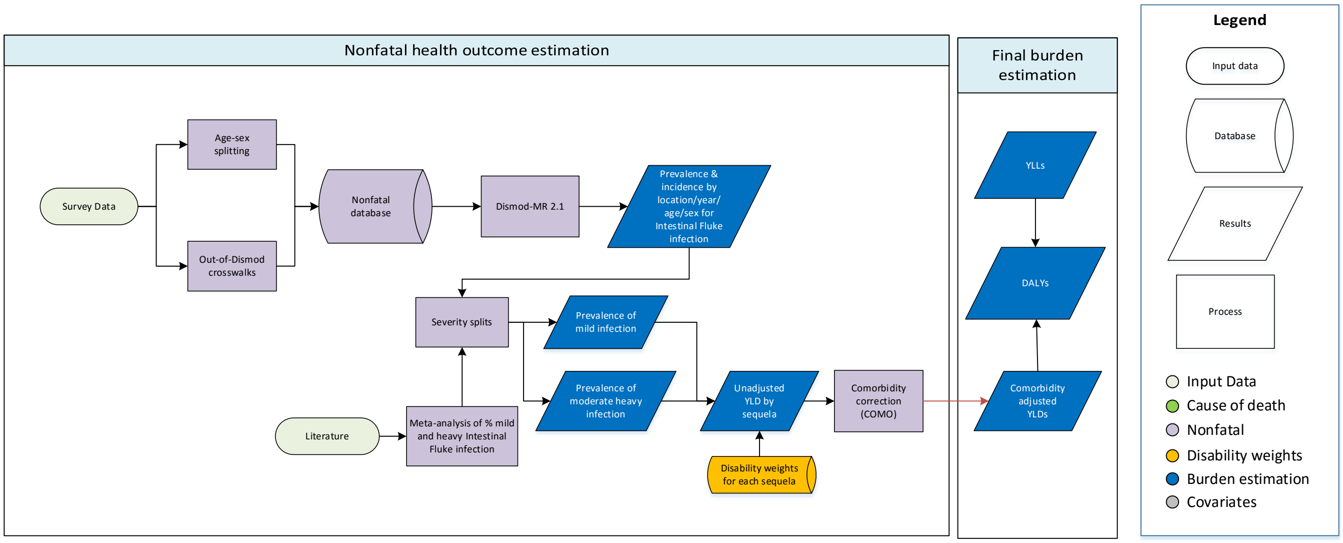

Opisthorchiasis

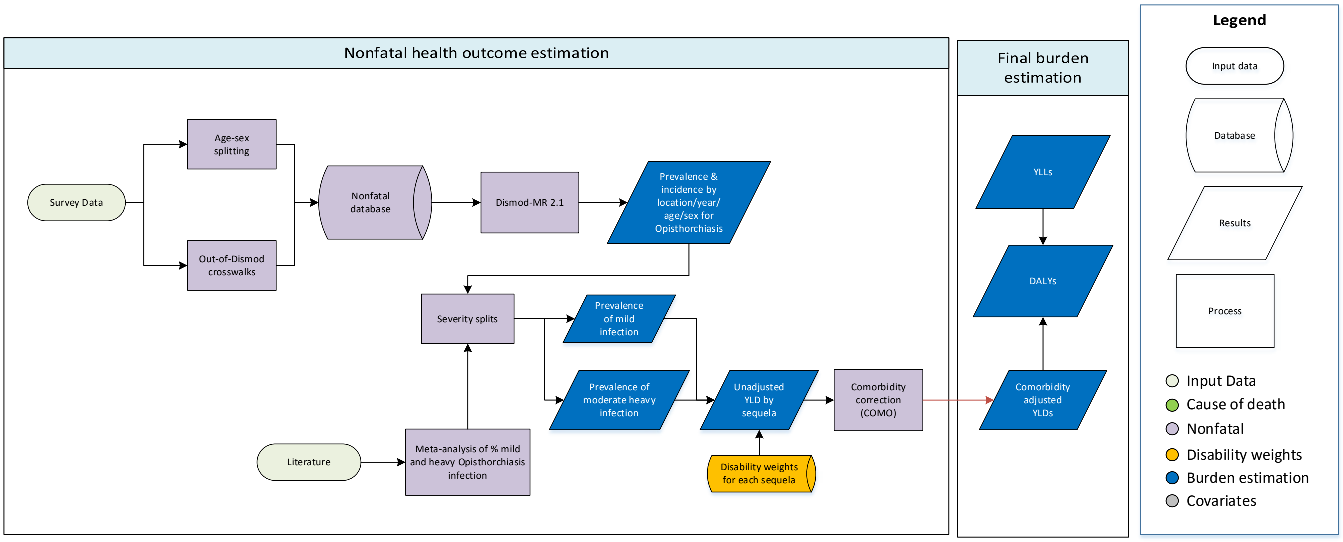

Paragonimiasis

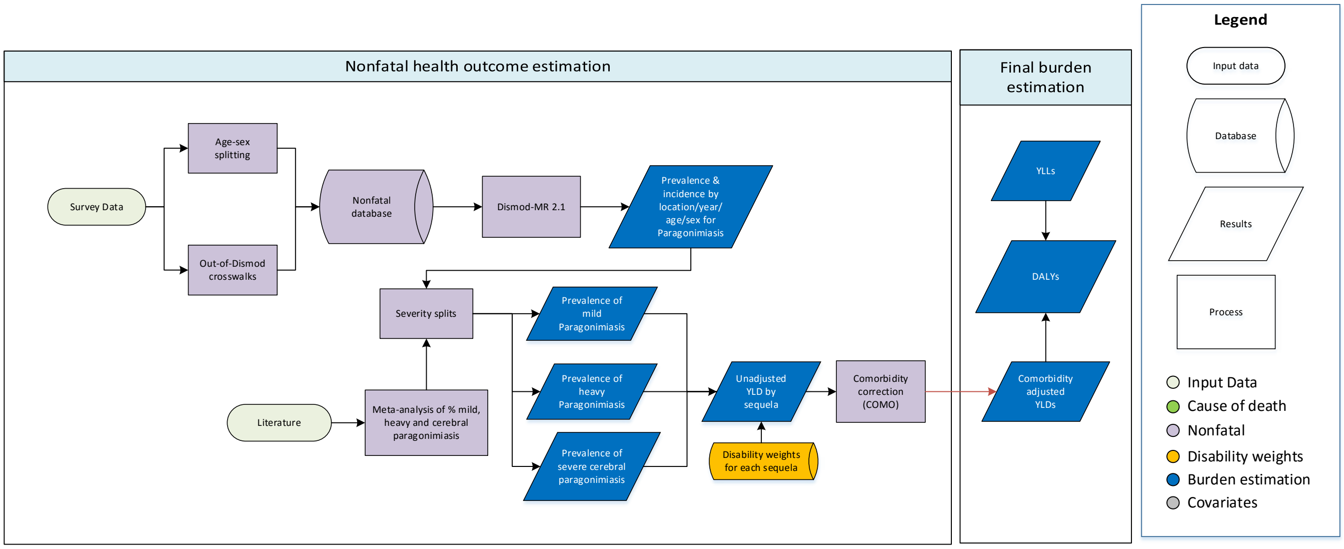

Input Data & Methodological Summary

Case definition

Human foodborne trematodiases (FBT) is defined as the infection with parasitic worms of the class trematoda, which are also known as flukes. Trematodes are transmitted via contaminated food and infection is highly related to food habits. Definitive hosts, including humans, become infected when ingesting viable metacercariae by consuming contaminated aquatic products (eg, watercress). In the ICD-10, FBT are listed under code B66 [1].

FBT is subdivided into six types of FBT (see Table 1):

- Clonorchiasis
- Fascioliasis
- Intestinal fluke
- Opisthorchiasis
- Paragonimiasis (normal and cerebral infections)

Table 1. Subtypes of FBT

	Species of FBT	Also known as:	Carcinogen
1	Chlonorchiasis	(Chinese) Liver fluke	Associated with choliangiocarcinoma
2	Opisthorchiasis<br><br>(*O viverrini* & *O felineus*)	Liver fluke	Associated with choliangiocarcinoma<br><br>(*O viverrini*)
3	Fascioliasis	Liver fluke	No available evidence

4	Intenstinal fluke	Liver fluke	No available evidence
5	Paragonimiasis	Lung fluke	

Thresholds for heavy infection and duration by species of FBT

The majority of people infected with FBTs are asymptomatic. When symptoms do occur they are often non-specific. Among the clinical symptomatic group, severity is associated with worm burden, typically measured by fecal egg counts, and the duration of infection. The thresholds for heavy infection and duration by species of FBT are shown in Table 2. The clinical presentation of FBT depends on the target organs (liver, lung, or intestines). Clonorchiasis and opisthorchiasis patients may suffer from loss of appetite, fullness, indigestion, diarrhoea, pain in the right upper quadrant, lassitude, weight loss, ascites, and oedema.[2, 3] Cholangitis, obstructive jaundice, intra-abdominal mass, cholecystitis, and gallbladder or intrahepatic stones may occur as complications.[3, 4]

Table 2. Thresholds for heavy infection and duration by species of FBT

	Species of FBT	Case thresholds for heavy infection	Duration
1	Chlonorchiasis	10,000 eggs per g of feces	lifelong
2	Opisthorchiasis	10,000 eggs per g of feces	lifelong
3	Fascioliasis	1,000 eggs per g of faces	lifelong
4	Intenstinal fluke	1,000 eggs per g of faces	lifelong
5	Paragonimiasis	100 eggs per 5 ml sputum	lifelong
6	Cerebral paragonimiasis	Any infection of the brain with flukes and/or eggs of *Paragonimus* spp.	lifelong

Input data

Model inputs

For GBD 2010, the data came from the expert group and is the result of their analysis. The expert group analysis used the results of a systematic literature review performed by Furst et al. as a starting point for the analysis.[5] Furst et al. searched PubMed, WHOLIS, FAOBIB, Embase, CAB Abstracts, Literatura Latino Americana e do Caribe em Ciências de Saùde (LILACS), ISI Web of Science, BIOSIS preview, Science Direct, African Journals OnLine (AJOL), and the System for Information on Grey Literature in Europe (SIGLE), period Jan 1, 1980, to

Dec 31, 2008. The initial number of studies identified through the literature review was ~34,000 references. The literature review included extracted data from 181 studies. For GBD 2013 and GBD 2015 the search strategy was replicated to capture epidemiological studies published between 2008 and 2015. Due to the cyclical nature of

systematic review for GBD causes, no data collection was scheduled for GBD 2016. As such, foodborne trematodiases will be a priority for the next iteration of the study.

Input data for the assessment of the total national number of infected people

Only studies that used countrywide surveys to estimate the national prevalence rates were included (or for China, province-wide surveys). Reason for choosing only national studies is that FBT shows a highly focal spatial distribution and local cross-sectional surveys would profoundly under- or overestimate true national prevalences. We decided not to model national and subnational together and get a coefficient on subnational, because there is not a one-fits-all relationship across the world. Infection is highly related to food habits and there are highly varying differences between national and subnational prevalence rates. The final GBD 2016 dataset contained 29 prevalence studies from 17 countries. We used raw data from the selected studies as input for DisMod.

Prevalence intestinal fluke infection

Intestinal fluke is different from the other types of FBT, because there are several pathogens that fall under intestinal fluke infection. It can be caused by pathogens, such as Metagonimus spp., Echinostoma spp., Neodiplostomatidae.[6] When assessing the prevalence of intestinal fluke infection, we added the identified prevalence for each parasite species in order to obtain the overall prevalence of intestinal fluke infections. This approach may lead to a certain overestimation of the true prevalence, because people may be co-infected with more than one intestinal fluke species. There is no sufficient evidence about the proportion of co-infections, but the resulting overestimation of the true prevalence may be more than offset by the assumptions made in our previous modelling approach and the many challenges in generating the underlying epidemiological parameters (eg, diagnostic inaccuracy in the detection of infections with the more than 50 intestinal fluke species). Also of note: the transmission source of intestinal fluke infections are species-specific and therefore vary. For instance, *Fasciolopsis buski* is usually transmitted by eating raw water plants with the infective parasite stage attached to the water plants, whereas Neodiplostomatidae are transmitted by eating undercooked and infested frogs, snakes, and tadpoles. Because of these different transmission pathways, the rate of co-infection might in fact be smaller than expected.

Input data to differentiate between asymptomatic and heavy infections

We estimated the proportion of heavily infected among all infected in all available national and regional cross-sectional surveys. It is expected that heavy infection increases with age and there are data available on heavy infection by age group. We therefore decided to include age-dependent rates of heavy infection for clonorchiasis, opisthorchiasis, and intenstinal fluke infection. For (cerebral) paragonimiasis and fascioliasis there were not sufficient age-dependent data on high intensity FBT infection.

Modelling strategy

We used a three-step process for the disease modelling of FBT. In the first step we used DisMod-MR 2.0 to estimate assess the prevalence of FBT by age, sex, year, and country. In the second we differentiated between asymptomatic and heavy infections. MetaXL (a meta-analysis add in for Microsoft Excel) was used to estimate the proportion of heavy infected among all infected by age group for clonorchiasis, opisthorchiasis, and intenstinal fluke infection (see Table 3 and 4). These proportions were used to estimate the prevalence of heavy FBT infection.

The third step consisted of deselecting countries that have no autochtonous case reports of FBT (input 34,000 references from literature review).

Table 3. Percentage of high intensity infection by age group and type of FBT (based on eight FBT prevalence studies)

Age category	Clonorchiasis			Opisthorchiasis			Intestinal fluke infection		
	Mean	Low	High	Mean	Low	High	Mean	Low	High
0-9	30%	17%	44%	10%	0%	29%	8%	3%	14%
10-19	15%	0%	43%	15%	0%	69%	11%	8%	14%
20-29	18%	10%	29%	16%	0%	52%	18%	15%	21%
30-39	17%	5%	34%	21%	0%	56%	22%	17%	28%
40-49	22%	13%	32%	28%	1%	68%	22%	13%	32%
50-59	18%	0%	49%	29%	0%	75%	17%	9%	28%
60+	32%	18%	47%	25%	0%	64%	15%	8%	23%

Table 4. Percentage of high-intensity infection by type of FBT (based on 4 FBT prevalence studies)

Type of FBT	Mean	Low	High
Paragonimiasis	23%	0%	59%
Fascioliasis	19%	3%	41%

Cerebral paragonimiasis

It was assumed that 0.8% of paragonimiasis cases have cerebral involvement. This proportion was used to estimate the prevalence of cerebral paragonimiasis. This proportion is based on one study. The data are from Oh SJ. The rate of cerebral involvement in paragonimiasis: an epidemiologic study. *Jpn J Parasitol* 1969;18:211-14. The study was performed in Paju, South Korea. This is an area with 6,738 inhabitants and according to the survey, it was estimated that 29.6% of all individuals would react to intradermal test (= an immunological reaction indicating previous or current contact to the parasite). 25% of all "positive reactors" may have eggs in their sputum (= active infection with the parasite currently present in the human host). If these rates are applied to the community as a whole, the number of patients with active paragonimiasis would be at least 498 (=6,738*0.296*0.250). Furthermore, four cases of cerebral paragonimiasis were found in this community. Therefore, four out of 498 individuals with active paragonimus infection suffered from cerebral infection (=0.80%; 95% confidence interval 0.019%-1.587%).

Severity splits and disability weights

For GBD 2016, FBT was not split into health states with different severities. The table below shows the GBD 2016 disability weights that were used to calculate the burden of FBT in YLDs.

Table 5. Disability weights that were used to calculate FBT YLDs

Sequelae	Severity description	Health state name	Disability weight
Asymptomatic clonorchiasis	Clonorchiasis, currently without symptoms	N/A	0.000 (0.000–0.000)

Heavy clonorchiasis	Abdominal pain and nausea reported as moderate	Abdominopelvic problem, moderate	0.114 (0.078–0.159)
Asymptomatic opisthorchiasis	Opisthorchiasis, currently without symptoms	N/A	0.000 (0.000–0.000)
Heavy opisthorchiasis	Abdominal pain and nausea reported as moderate	Abdominopelvic problem, moderate	0.114 (0.078–0.159)
Asymptomatic fascioliasis	Fascioliasis, currently without symptoms	N/A	0.000 (0.000–0.000)
Heavy fascioliasis	Abdominal pain and nausea reported as moderate	Abdominopelvic problem, moderate	0.114 (0.078–0.159)
Asymptomatic intestinal fluke infection	Intestinal fluke infection, currently without symptoms	N/A	0.000 (0.000–0.000)
Heavy intestinal fluke infection	Abdominal pain and nausea reported as moderate	Abdominopelvic problem, moderate	0.114 (0.078–0.159)
Asymptomatic paragonimiasis	Paragonimiasis, currently without symptoms	N/A	0.000 (0.000–0.000)
Heavy paragonimiasis	Cough, fever, and weight loss	Tuberculosis, not HIV-infected	0.333 (0.224–0.454)
Cerebral paragonimiasis	Epilepsy due to cerebral paragonimiasis	Epilepsy, less severe (seizures < once per month)	0.263 (0.173–0.367)
		Epilepsy, severe (seizures >= once per month	0.552 (0.375–0.710)

Note. N/A: not applicable

Changes from GBD 2015 to GBD 2016

We have made no substantive changes in the modelling strategy from GBD 2015 to GBD 2016.

References

1. WHO. *International Statistical Classification of Diseases and Related Health Problems. 10th Revision. Version for 2007.* 2007 [cited 2009 October 14, 2009]; Available from: http://apps.who.int/classifications/apps/icd/icd10online/.
2. Rim, H.J., *Clonorchiasis: an update.* J Helminthol, 2005. **79**(3): p. 269-81.
3. Pungpak, S., et al., *Clinical features in severe opisthorchiasis viverrini.* Southeast Asian J Trop Med Public Health, 1985. **16**(3): p. 405-9.
4. Rim, H.J., *The current pathobiology and chemotherapy of clonorchiasis.* Korean J Parasitol, 1986. **24**(Suppl.): p. 1-141.
5. Furst, T., J. Keiser, and J. Utzinger, *Global burden of human food-borne trematodiasis: a systematic review and meta-analysis.* Lancet Infect Dis, 2012. **12**(3): p. 210-21.

6. Furst, T., et al., *Manifestation, diagnosis, and management of foodborne trematodiasis.* BMJ, 2012. **344**: p. e4093.

3.3.5 Dracunculiasis (Guinea worm) SDG Capstone Appendix

Flowchart

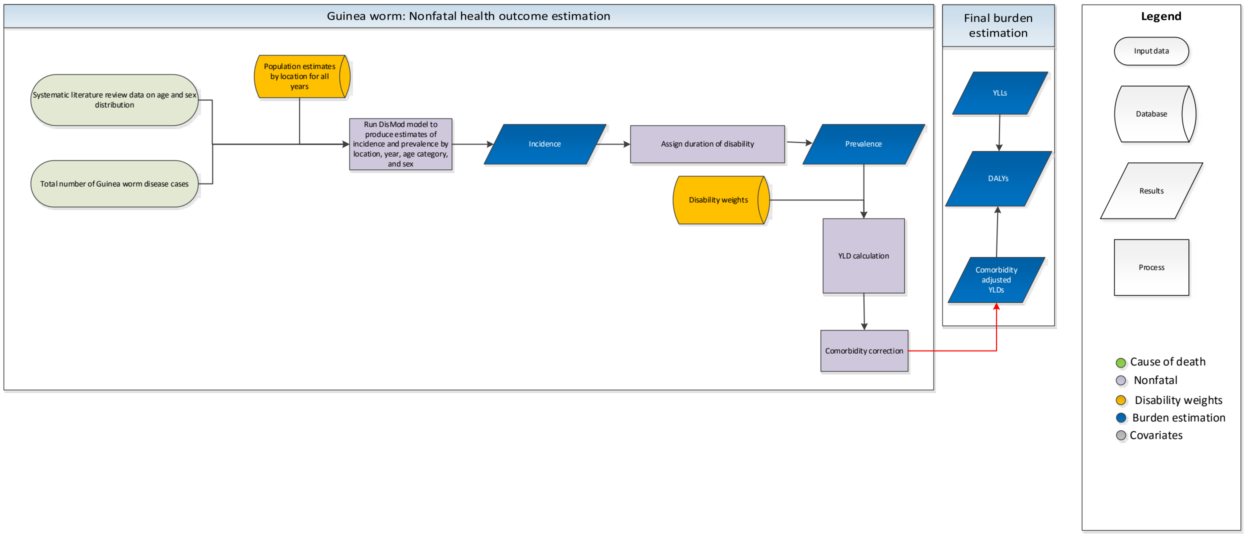

Background

Guinea-worm disease is caused by the parasitic worm *Dracunculus medinensis*. The transmission cycle begins when Guinea worm larvae are released in water (shallow ponds or open wells) where they are ingested by copepod *Cyclops* (water fleas) [1]. When a person consumes contaminated water, the copepod is dissolved by gastric acids and the larvae are released. Larvae then migrate through the intestinal wall; the male and female mate. Shortly thereafter, the male dies and the female worm moves through the victim's subcutaneous tissues. A year post-infection, the adult worm emerges through the skin, usually from the feet or lower limbs. Worm emergence causes an intensely painful edema, blister and an ulcer accompanied by fever, nausea, and vomiting. To relieve the pain associated with the worm's emergence, infected persons immerse the infected part of their body in local water sources such as ponds. Upon entering the water, the female worm will expulse her larvae into drinking water where the cycle can begin again [1,2].

To break the cycle of transmission, ministries of health in endemic countries implement a suite of interventions: case detection and containment; provision of safe water sources; distribution of filter cloths and pipe filters; water source treatment with Abate® (a larvacide); and health education.

By design, the Guinea worm eradication programmatic infrastructure covers the entire at-risk population. Since case containment [3] is a key intervention designed to not only interrupt transmission but also monitor progress toward eradication, incident cases of guinea worm disease are nationally representative. To implement case containment as an intervention, all cases of Guinea worm disease are identified. Containment is defined as detection within 24 hours of the worm's emergence; the patient did not contaminate any water source; the patient received proper wound care and health education on

not entering any water source; and a supervisor verified the case as dracunculiasis within seven days. Case reporting occurs at the village level on a monthly basis; case data are then aggregated within the national Guinea worm eradication program and reported to the World Health Organization. In settings where annual case reports are low (suggesting no transmission) or transmission has been interrupted, cash rewards are promoted to enhance surveillance activities.

Input Data & Methodological Summary

Case Definition

A Guinea worm case is defined as an individual with Guinea worm disease (a single case could have more than one worm emerge at one time). These cases are confirmed through the Guinea worm eradication program infrastructure by clinical exam and verification by local supervisors.

Input data

Model inputs

Geographic restrictions

Only the following countries were identified as guinea-worm endemic as of 1990 (4): Benin, Burkina Faso, Cameroon, Central African Republic, Chad, Côte d'Ivoire, Ethiopia, Ghana, India, Kenya, Mali, Mauritania, Niger, Nigeria, Pakistan, Senegal, Sudan, South Sudan, Togo, Uganda, and Yemen [4]. Any country not reporting Guinea worm in 1990 is not included in the GBD model.

Geographic restrictions by year were also implemented to account for the period post-transmission to reflect the accomplishments of the Guinea worm eradication campaign. Geographic restriction for countries that were endemic in 1990 was defined based on data reported post-interruption of transmission. In the GBD analysis, Guinea worm disease was no longer modelled for the year that followed the last reported case (imported or indigenous) provided that the subsequent years through 2015 also had no case reports. To ensure that cases were attributed to burden in the country in which the case was detected, both indigenous and imported cases were included. For example, if Kenya reported its last case in 2005 (imported), and as no other cases were reported through 2015, the geographic restriction began in 2006. For Ethiopia and Chad, countries that had re-introduction of transmission, no geographic restriction was implemented for the period 1990–2015.

Data sources

1) Case data by geography, by year
2) Literature review of age/sex distribution
3) Literature review for sequelae (type, duration, and proportion)

Case data: Annual case data were reported by WHO in the Weekly Epidemiological Record. For years or geographies for which WER reports were not published, the following sources were also used to extract case counts:

1) CDC's MMWR reports
2) 1990–1999 total country reports from Hopkins *et. al.* [4]: A summary of case totals from 1990–1999, as these are not reported in WER for every year
3) India subnational estimates: India MOH report (1984–1999)

4) The Carter Center's Guinea worm wrap-up: disaggregation of case totals for Sudan and South Sudan pre-2011 (independence) to ensure case totals from 1990–2010 are consistent with current national boundaries.

The number of cases annually was compared to official total numbers to ensure accuracy. WER data were used in the analysis in the event of a discrepancy.

Subnational data

India: Subnational data for India were obtained from the Ministry of Health for the period 1984–1999, cases were reported by year and state: http://www.ncdc.gov.in/index2.asp?slid=329&sublinkid=216.

Kenya: Subnational data from Kenya were requested from the MOH but not obtained. To split cases by subnational unit, the Carter Center Guinea Worm Wrap-Up was reviewed to identify districts with endemic villages. A national survey conducted 1993/1994 found cases in Turkana and West Pokot counties, but case totals were not reported by county. Indigenous transmission was interrupted in 1995, with imported cases reported until 2005. All cases in Kenya are currently analyzed in GBD as occurring in Turkana County as we are unable to disaggregate the data. WER reports from 1999–2006 document that all imported cases from 1998–2005 occurred in Turkana County.

Age/sex distribution

Generally, the risk of Guinea worm infection varies according to sex- or age-specific differences in access to safe drinking water. A study in Ethiopia found women were more likely to experience Guinea worm disease than men; in India, men experienced greater risk of infection [1]. Exposure to unsafe water sources varies largely on mobility patterns and type of water sources: communities in which infested water is carried in for consumption are more likely to see more Guinea worm disease in children and older adults [5]. Communities in which infection results from drinking contaminated water due to human movement patterns (such as cattle grazing, travel) demonstrate a slightly greater risk in adults, particularly those of working age. Once interventions to control the spread of Guinea worm infection are implemented, the age and sex distribution likely changes to reflect variation in access to safe water sources and case-containment practices, but age/sex case data are currently not available.

The evidence base available to describe risk of infection by age is as follows:

1) Studies from Nigeria:
    a. Adeyeba *et al* [6]: Guinea worm disease not common among children <1 year of age; increase in risk by age
    b. Kale *et al* [7]: More boys ages 5-9 years than girls were infected (11.9% v. 6.8%); Women ages 20-29 higher prevalence of infection than men (13.4% v. 4.7%); Overall, the prevalence in both men and women was highest in ages 10-14 and 30 years or older.
2) Other countries:
    a. Sudan [8]: No significant age trend among lower-endemicity villages; higher-endemicity villages (n=4) had higher prevalence in children and older adults. Attributes the difference in age trends to community-level water source.
    b. Ghana [9]: The trend in age of first infection reported was similar for males and females, with more females experiencing first infection between 15 and 19 years and males between 20 and 24 years of age. The proportion of men with guinea worm disease was

much higher than women among ages 25-54 years. Adults (>15 years of age) were more likely to be infected than children.

The evidence base available to describe the risk of infection by gender is as follows:

1) Studies from Nigeria:
    a. Adeyeba *et al* [6]: No difference among males and female
    b. Kale *et al* [7]: No overall gender difference comparing total males infected to total females infected, although gender differences for certain age groups (see notes above).

For the GBD analysis, no difference in incidence based on gender was modelled due to conflicting evidence related to sex-specific risk of infection. While there is limited evidence to suggest that risk varies by sex, it is also modified by age; however, evidence for this modification also suggests that such age- and sex-specific risk may vary by endemic community within a given geography (in some settings, women at higher risk, in others men, but not for all age strata).

To model age-specific variation, we assumed a limited increase in risk among adults aged 15 years and older and assumed no Guinea worm disease in infants less than 1 year of age.

Severity splits/sequelae

Sequelae associated with Guinea worm relate to the wound at the site of the worm's emergence, which can include abscesses and chronic ulcerations. Joint and tissue damage can occur, as well as secondary infection in connective tissues[10]. During the worm's emergence, which takes approximately one month to exit the body, the ulcer is painful and itchy[1]. The wound is subject to secondary infection and scarring. While an individual experiences Guinea worm disease, they are generally unable to work and have limited mobility at the time of emergence and during the period in which they are healing. Although most worms emerge in the feet and lower legs, there are reports of worms exiting at other locations[10], which could cause other disability not accounted for here. A study in Nigeria found that 98% of worms emerged in the lower limbs[11]. Therefore, all disability associated with Guinea worm disease is attributed to lower limb conditions, pain and lack of mobility with no distinction made for number of worms emerging at any single time.

The following evidence base was reviewed to determine the proportion of cases attributed to each sequela, as well as duration of sequelae.

Duration of disability and type of disability:

Studies from Nigeria:

1) Adeyeba *et al* [6]: 93.4% incapacitated for an average of 26 days.
2) Smith *et al* [12]: Average disability duration 12.7 weeks; 58% unable to leave the home for a mean duration of 4.2 weeks; duration of disability greater among those older than 50 years compared to those younger than 50 years.
3) Okoye *et al* [11]: 21% of cases were totally incapacitated due to their infection (not permanently disabled).
4) Kate *et al* [7]: A survey of 17 villages from 1971 to 1975 found that duration of disability was approximately 100 days.

Other countries:

5) Benin [13]: From two villages in highly endemic areas, estimated 39-59 days of disability experienced after worm emergence.
6) Ghana [14]: 28.2% experienced pain 12-18 months post emergence; 5% unable to carry out at least one daily activity, 0.5% permanently impaired.
7) Ghana [9]: Complete disability experienced among males with Guinea worm disease lasted approximately 5 weeks among those untreated. Among cases provided supportive care (wound management), the duration of disability was 2.5 weeks.

We therefore assume that each case of Guinea worm disease identified from 1990 onward likely received some degree of case management through national Guinea worm eradication programs; this assumption likely holds better in the period post-1995 after case-containment was widely adopted. For cases identified prior to 1995, we extend the period of disability due to worm emergence to two months, followed by two months of motor impairment.

For cases reported after 1995, we assume every case experiences pain and disfigurement, Level 3, and motor impairment (severe) for a period of one month, followed by two months of motor impairment (moderate).

For all years, we assume that 30% of all cases will then experience disfigurement level 2 with itch/pain and motor impairment (moderate) for an additional 9 months (approximately a year of disability). A total of 0.5% of all cases will experience permanent disability defined as musculoskeletal problems, lower limbs, severe, experienced after the initial period of pain/disfigurement and motor impairment associated with worm emergence.

Sequela	Lay description	DW (95% CI)
Disfigurement, level 3, with itch/pain	has an obvious physical deformity that is very painful and itchy. The physical deformity makes others uncomfortable, which causes the person to avoid social contact, feel worried, sleep poorly, and think about suicide.	0.405 (0.275–0.546)
Disfigurement, level 2, with itch/pain	has a visible physical deformity that is sore and itchy. Other people stare and comment, which causes the person to worry. The person has trouble sleeping and concentrating.	0.188 (0.125–0.267)
Musculoskeletal problems, lower limbs, severe	has severe pain in the leg, which makes the person limp and causes a lot of difficulty walking, standing, lifting and carrying heavy things, getting up and down, and sleeping.	0.165 (0.112–0.232)
Motor impairment, severe	is unable to move around without help, and is not able to lift or hold objects, get dressed, or sit upright.	0.402 (0.268–0.545)
Motor impairment, moderate	has some difficulty in moving around, and difficulty in lifting and holding objects, dressing and sitting upright, but is able to walk without help.	0.061 (0.04–0.089)

Modelling strategy

The incidence of Guinea worm disease is modeled using DisMod. Duration of Guinea worm disease is used to inform prevalence estimates. Estimates are produced for 1990, 1995, 2000, 2005, 2010, and 2015.

References

1. Cairncross S, Muller R, Zagaria N. Dracunculiasis (Guinea Worm Disease) and the Eradication Initiative. Clin Microbiol Rev. 2002 Apr;15(2):223–46.

2. Biswas G, Sankara DP, Agua-Agum J, Maiga A. Dracunculiasis (guinea worm disease): eradication without a drug or a vaccine. Philos Trans R Soc Lond B Biol Sci. 2013 Aug 5;368(1623):20120146.

3. Dracunculiasis eradication: case definition, surveillance and performance indicators. Releve Epidemiol Hebd. 2003 Sep 12;78(37):323–8.

4. Hopkins DR, Ruiz-Tiben E, Ruebush TK, Diallo N, Agle A, Withers PC. Dracunculiasis eradication: delayed, not denied. Am J Trop Med Hyg. 2000 Feb;62(2):163–8.

5. Watts SJ. The comparative study of patterns of guinea worm prevalence as a guide to control strategies. Soc Sci Med 1982. 1986;23(10):975–82.

6. Adeyeba OA, Kale OO. Epidemiology of dracunculiasis and its socio-economic impact in a village in south-west Nigeria. West Afr J Med. 1991 Dec;10(3–4):208–15.

7. Kale OO. The clinico-epidemiological profile of guinea worm in the Ibadan district of Nigeria. Am J Trop Med Hyg. 1977 Mar;26(2):208–14.

8. Tayeh A, Cairncross S. The impact of dracunculiasis on the nutritional status of children in South Kordofan, Sudan. Ann Trop Paediatr. 1996 Sep;16(3):221–6.

9. Belcher DW, Wurapa FK, Ward WB, Lourie IM. Guinea worm in southern Ghana: its epidemiology and impact on agricultural productivity. Am J Trop Med Hyg. 1975 Mar;24(2):243–9.

10. Muller R. Guinea worm disease: epidemiology, control, and treatment. Bull World Health Organ. 1979;57(5):683–9.

11. Okoye SN, Onwuliri CO, Anosike JC. A survey of predilection sites and degree of disability associated with guineaworm (Dracunculus medinensis). Int J Parasitol. 1995 Sep;25(9):1127–9.

12. Smith GS, Blum D, Huttly SR, Okeke N, Kirkwood BR, Feachem RG. Disability from dracunculiasis: effect on mobility. Ann Trop Med Parasitol. 1989 Apr;83(2):151–8.

13. Chippaux JP, Banzou A, Agbede K. [Social and economic impact of dracunculosis: a longitudinal study carried out in 2 villages in Benin]. Bull World Health Organ. 1992;70(1):73–8.

14. Hours M, Cairncross S. Long-term disability due to guinea worm disease. Trans R Soc Trop Med Hyg. 1994 Oct;88(5):559–60.

3.3.5 Ascariasis SDG Capstone Appendix

Flowchart

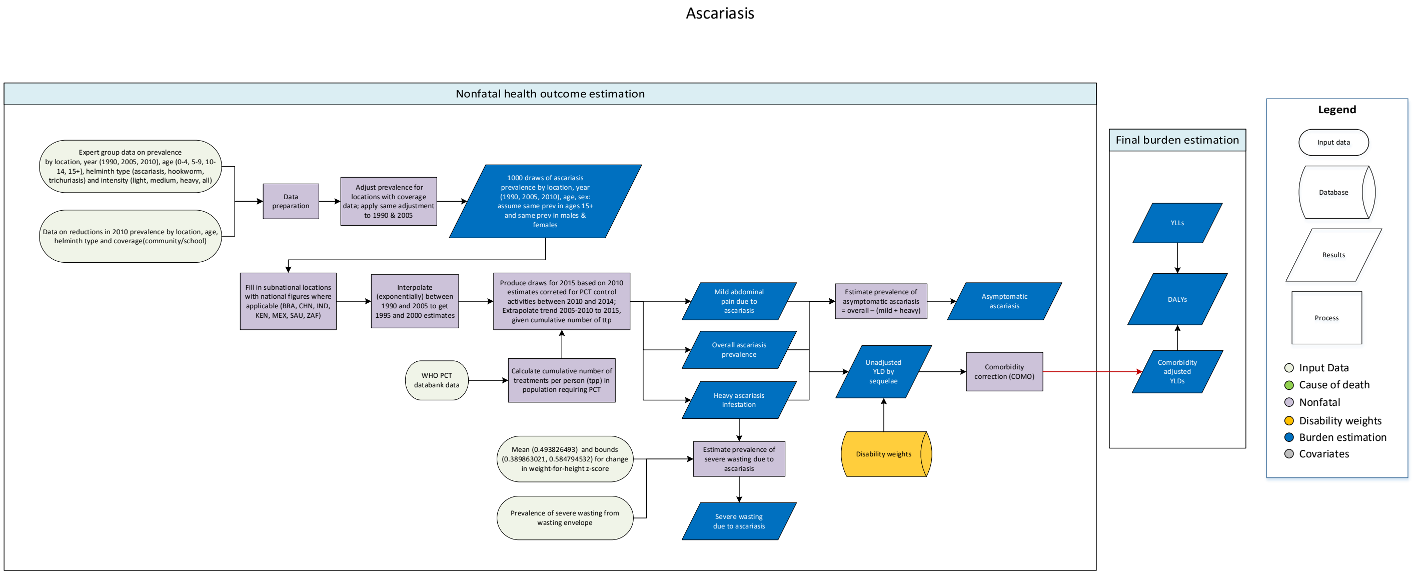

Input data and methodological summary

Case definition

Ascariasis is a helminth diseases caused by the parasitic roundworm *Ascaris lumbricoides*. It is one of the three intestinal nematode infections (INI), or soil-transmitted helminthiasis (STH), that we model in GBD. Diagnosis is made by microscopic exam of stool with or without concentration procedures (recommended as eggs may be difficult to see). The ICD-10 codes for ascariasis are B77-B77.9.

Input data

Model inputs

Four different input data were used in the ascariasis nonfatal model. The first was prevalence data prepared by the expert group (EG) during GBD 2010 [1, 2]. They provided the data (mean, upper, lower) by location, year (1990, 2005, 2010), age (0-4, 5-9, 10-14, 15+ years), helminth type (ascariasis, hookworm disease, trichuriasis) and intensity of infection (light, medium, heavy, all). For the model, light infestation was not attributed any disability. The second data, also from the EG, was on reductions in prevalence in 2010, provided by location, age, helminth type, and coverage (community/school). The table below shows the number of countries or subnational units and GBD world regions represented in the data.

Table 1a. Geographic spread of data

	prevalence
Countries/subnationals	163
GBD world regions	16

The third input data was from the WHO PCT Databank [3]. This data was downloaded from the source website and represented 121 locations and six GBD world regions. The last input data was 1,000 draws of wasting envelope prevalence among children under 5 years – the methods used to generate estimates of wasting prevalence are detailed elsewhere (part of risk factors documentation). The table below shows the number of countries or subnational units and GBD world regions represented in the data.

Table 1b. Geographic spread of data

	prevalence
Countries/subnationals	561
GBD world regions	21

Severity splits/sequelae

The table below shows the list of sequelae due to ascariasis and the associated disability weights (DW). The sequelae were based on prevalence of medium and heavy infestation – medium infestation was assigned mild abdominopelvic problems; heavy infestation was assigned symptomatic worm infection; and light infestation was not attributed any disability.

Table 2. Sequelae, lay descriptions, and disability weights (DWs)

Sequela	Lay description	DW
Mild abdominopelvic problems	has some pain in the belly that causes nausea but does not interfere with daily activities	0.011 (0.005–0.021)
Heavy infestation	has cramping pain and a bloated feeling in the belly	0.027 (0.015–0.043)
Severe wasting	is extremely skinny and has no energy	0.128 (0.082–0.183)
Asymptomatic ascariasis	N/A	N/A

Modelling strategy

In the estimation of morbidity due to ascariasis, the EG data were first prepared by formatting the location names to be consistent with the GBD 2016 location names and applying the 2010 prevalence to 1990 and 2005 for sub-Saharan Africa countries – estimates for these two years were missing. This was followed by using the data on reductions in 2010 prevalence to adjust the prevalence for locations with coverage data. After this adjustment, only data for medium infection, heavy infection, and all infection were retained.

Using the mean prevalence and the upper and lower bounds of the mean provided by the EG, 1,000 draws of prevalence were generated. This was done by multiplying the mean estimates by the exponent of random draws from a normal distribution with mean = 0 and standard deviation = sd, where sd = abs(abs(ln(upper)-ln(lower))/(invnormal(0.975)*2). These draws were created for all GBD age-groups,

assuming the same prevalence in ages 15+ and same prevalence in males and females. Since the draws were only at the national level, subnational locations were filled with national figures where applicable (Brazil, China, India, Indonesia, Kenya, Mexico, Saudi Arabia, and South Africa).

To get 1995 and 2000 estimates, exponential interpolation of estimates between 1990 and 2005 was performed. The draws for 2016 were produced based on 2010 estimates corrected for PCT control activities between 2010 and 2014 – this was done by extrapolating the 2005–2010 trend to 2016, given cumulative number of treatments per person calculated using data from the WHO PCT Databank [3]. The 2005-2010 trend was applied to all intensities of infection. Prevalence was assumed to be zero for the countries with missing input data and also in children younger than 28 days. The resulting estimates were 1,000 draws of ascariasis prevalence by GBD location, year, age, sex, and intensity level (mild, heavy, overall infection). To estimate the prevalence of asymptomatic ascariasis, prevalence of mild and heavy infestation was subtracted from the overall ascariasis prevalence.

The final step in the modelling process was to estimate the prevalence of severe wasting due to ascariasis in age groups 28–364 days and 1–4 years. This was done separately using 1,000 draws of prevalence of heavy infestation due to ascariasis and the wasting envelope prevalence. The initial step in determining prevalence of severe wasting due to ascariasis was generating 1,000 draws of change in weight-for-height z-score per heavy prevalent case from a random normal distribution with mean = 0.493826493 and standard deviation = 0.04972834 (calculated from upper and lower bounds of the mean estimate). The mean, upper, and lower bounds were provided by a GBD collaborator who calculated them based on a published article [4]. The prevalence of severe wasting due to ascariasis was then obtained as a function of change in weight-for-height z-score (z_change) such that prevalence = p_wasting_env – Phi(Phi_inv(p_wasting_env) – z_change*p), where p_wasting_env = wasting envelope prevalence, Phi_inv is the inverse standard normal cumulative distribution function (cdf), and p = prevalence of heavy ascariasis infestation.

Model evaluation was done by plotting prevalence of overall ascariasis and that of each sequela against year for each location and age group. Maps of the global distribution of total ascariasis prevalence and prevalence of sequelae due to ascariasis were also assessed across time and age.

The only change made from GBD 2015 modelling strategy was the incorporation of updated data from the WHO PCT databank [3] in the correction of estimates for MDA activities.

References

1. Brooker S, Pullan R, Smith J, and Hotez P. Chapter: Intestinal nematodes. Cluster D: Communicable Diseases, Neglected Tropical Diseases Group. Global Burden of Diseases, Injuries, and Risk Factors Study. 2011 (4 July). 1-24.
2. Brooker S & Smith JL. Impact of hookworm infection and deworming on anaemia in non-pregnant populations: a systematic review. *Tropical Medicine and International Health*. 2010. 15,7,776-795.
3. WHO PCT Databank. 2016; http://www.who.int/neglected_diseases/preventive_chemotherapy/sth/en/.
4. Hall A, Hewitt G, Tuffrey V, de Silva N. A review and meta-analysis of the impact of intestinal worms on child growth and nutrition. *Maternal and Child Nutrition*. 2008. 4. 118-236.

3.3.5 Cutaneous & Mucocutaneous Leishmaniasis SDG Capstone Appendix

Flowchart

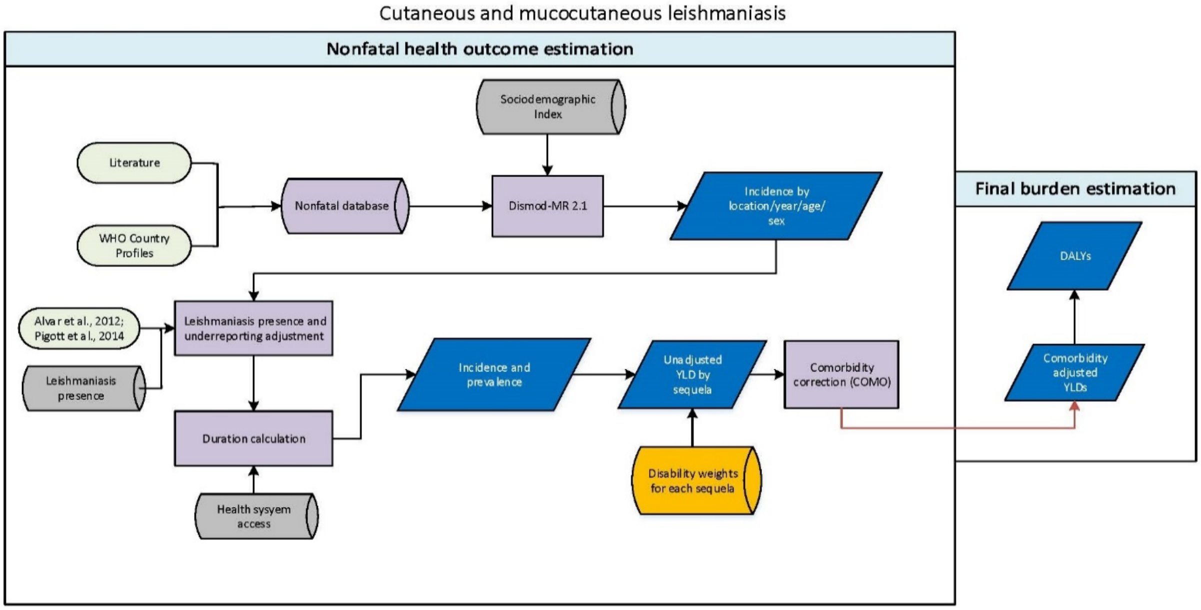

Input Data and Methodological Summary

Case Definition

Cutaneous leishmaniasis (CL) is the most common manifestation of disease caused by the *Leishmania* parasite, transmitted through the bite of phlebotomine sand flies. It causes the appearance of skin lesions, often beginning as papules or nodules and developing into ulcers, on parts of the body exposed to the bite of the sand fly. Mucocutaneous leishmaniasis (MCL) is a much more exceptional – and severe – presentation. Primarily isolated to Latin America, MCL infections can result in degradation of the mucous membranes, typically following an ulcerative sore from CL infection. Transmission varies by geographic region, as approximately 70 animal species have been identified as potential reservoir hosts of the parasite.

Input data

No systematic review of literature in the PubMed database was done for Cutaneous and Mucocutaneous Leishmaniasis for GBD 2016; however WHO country profile datasets were updated from their original 2010 year of reference, and subnational data from India and Brazil were included.

Modelling strategy

In general, there were few updates to the CL modelling strategy. The minimal amount of prevalence data conflicted with incidence where available, and thus was excluded from the model. No study-level

covariates were used. The Sociodemographic Index (SDI) was used as a country-level covariate on the incidence data, with a floor of exp(-1) – as to allow a degree of regional and subnational variation while constraining the predictive power such that predictions in hypo-endemic countries with low SDI values and no data would not be unduly high.

In order to control for DisMod fitting values to locations known to be devoid of CL, we replace estimates in these locations with zeros. Then for locations with confirmed CL presence, we apply an underreporting factor reported in Alvar et al. In order to distinguish prevalence of acute cases and those that endure lifelong disability, we used a normalized version of the health system access (HSA) covariate such that 47.6% of cases with poor access to health care – defined as (cases * (1 - norm(HSA)]) – would progress to the lifelong stage. All acute cases were assumed a six-month duration.

Changes from GBD 2015 to GBD 2016

We have made no substantive changes in the modelling strategy from GBD 2015 to GBD 2016.

3.3.5 Lymphatic Filariasis SDG Capstone Appendix

Flowchart

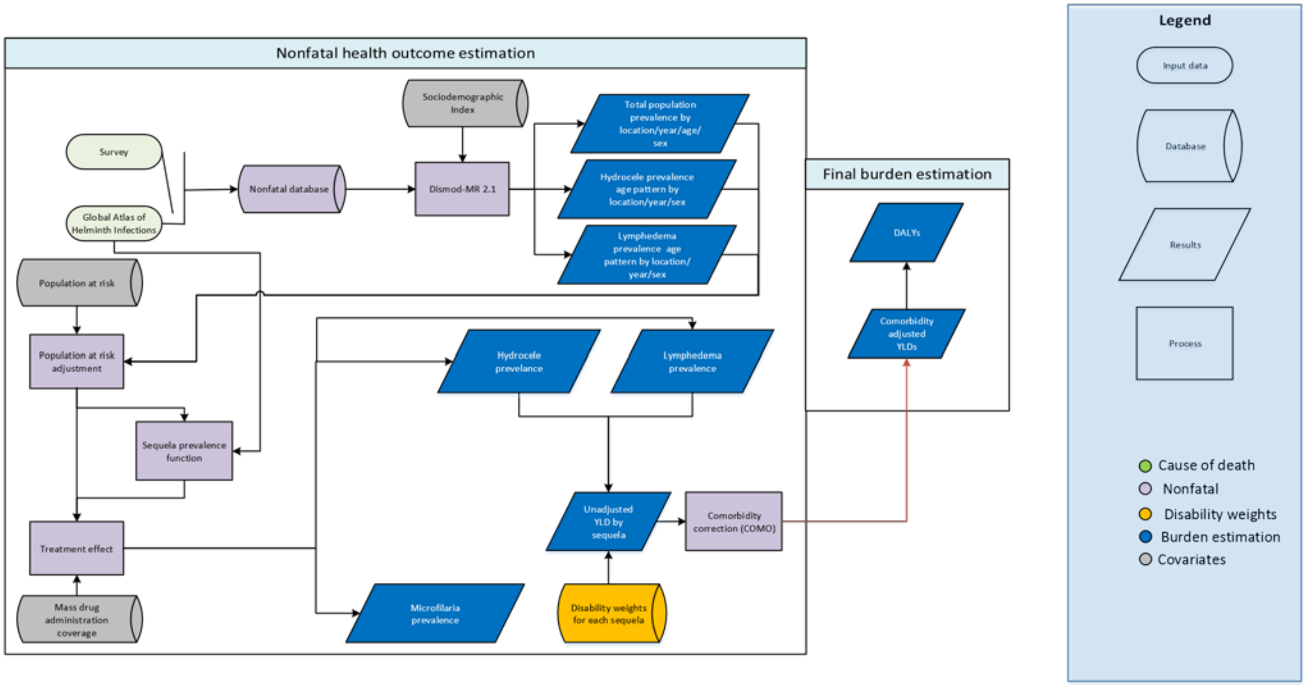

Input Data and Methodological Summary

Case Definition

Lymphatic filariasis (LF) is a neglected tropical disease spread in which threadlike nematodes invade the lymphatic system. The worms responsible – *Wuchereria bancrofti*, *Brugia malayi,* and *Brugia timori* – are spread from human to human via mosquitoes. The most prominent clinical manifestations of LF are lymphedema (a swelling of the legs, also known in its more extreme manifestation as elephantiasis) and hydrocele (a collection of fluid in the sac around the testicles).

Input data

A systematic review of literature for GBD 2016 in the PubMed database was done on October 14, 2016, for prevalence and incidence data using the search (Lymphatic filariasis AND prevalence) OR (Lymphatic filariasis AND (prevalence OR incidence OR "mass drug administration" OR MDA OR coverage)) OR (Lymphedema, hydrocele) OR (Transmission Assessment Survey (TAS)) OR (Lymphatic filariasis AND mapping).

Population at risk and MDA coverage data come from the WHO PCT Databank [1].

Modelling strategy

Data on prevalence of microfilaria is modelled using Dismod-MR 2.1. Due to the focal nature of lymphatic filariasis, we make the assumption that data collected are from endemic locations unless specifically specified in literature or survey methods. If the data are nationally representative, we adjust the data points by multiplying by the inverse of the proportion of the population at risk. Due to the fact that data is collected in endemic locations or we adjust it so that it is within the population at risk, we then scaled the DisMod-MR 2.1 estimates according to at-risk population in order to attain nationally representative values. We developed a new MDA location-level covariate that is used in the DisMod model based off WHO PCT Databank data, informing prevalence estimates.

For lymphedema and hydrocele, we incorporate survey data from the Global LF Atlas in a non-linear error-in-variables regression that determines the prevalence of lymphedema and hydrocele as functions of microfilaria prevalence, which is then applied to the total microfilaria DisMod model in order to attain an envelope of cases by location-year. Separately, all available prevalence data for these conditions is modeled in DisMod in order to determine an age-sex pattern.

In the estimation of lymphedema and hydrocele prevalence, we perform the same population at-risk correction that is done on microfilaria prevalence. For hydrocele prevalence after treatment, we take the value before MDA rollout in 2000 and reduce that by the same treatment efficacy function described for microfilaria prevalence, using dosage-reduction data specific to hydrocele along with the location-year specific MDA coverage. For lymphedema, we assume no new cases appear among treated individuals. As such, we reduce lymphedema prevalence in post-treatment years in accordance with MDA coverage.

Sequela	Data points	Regions	Countries	Subnational units
Prevalence of detectable microfilaria	1,552	10	40	28
Lymphedema due to lymphatic filariasis	511	10	25	15
Hydrocele due to lymphatic filariasis	265	8	22	12

Changes from GBD 2015 to GBD 2016

We conducted a new literature review, and utilized data from recent years and the MDA covariate to predict the time trend rather than last year’s non-linear regression to estimate the reduction of microfilaria as a function of treatments per person.

3.3.5 Visceral Leishmaniasis SDG Capstone Appendix

Flowchart

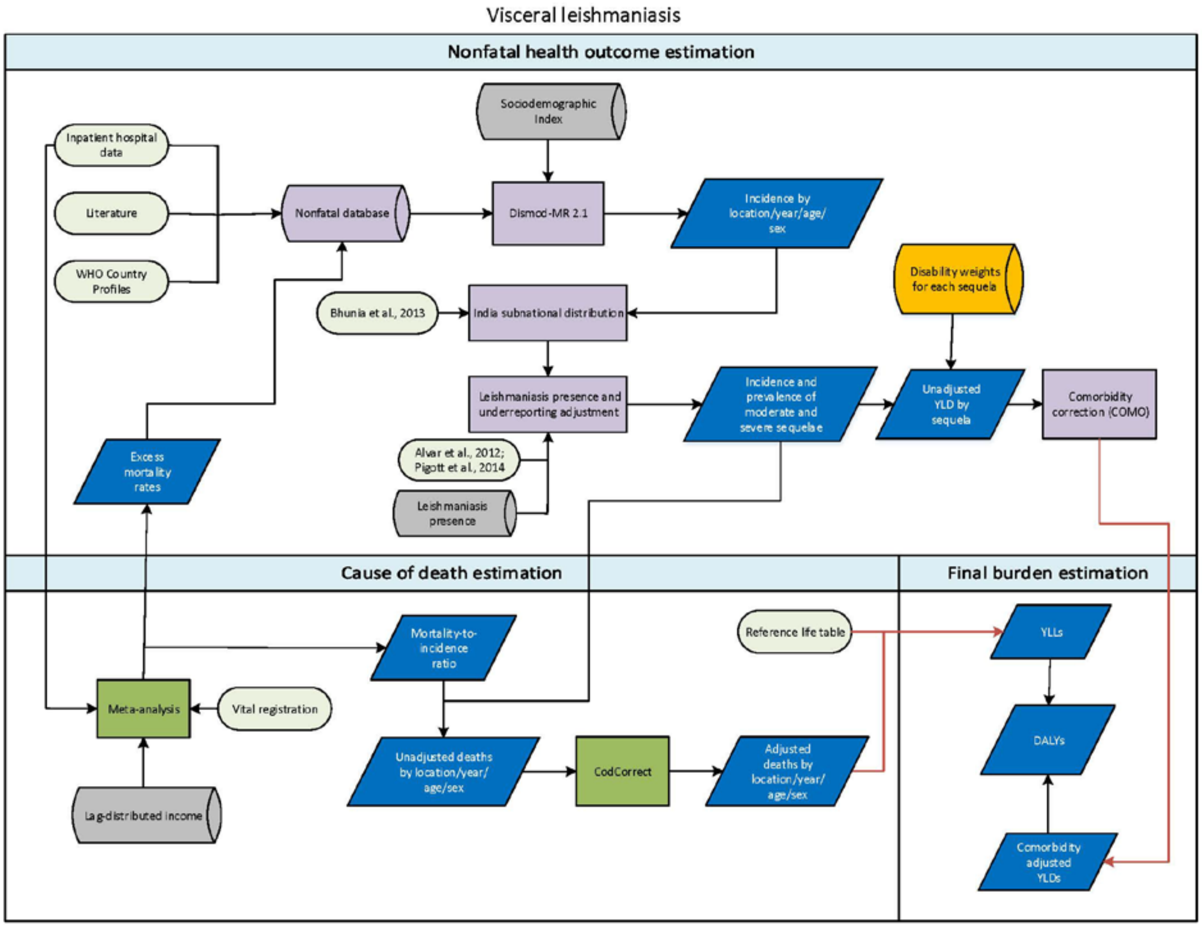

Input data and methodological summary

Case definition

Visceral leishmaniasis (VL) is the most serious manifestation of disease caused by the *Leishmania* parasite, transmitted through the bite of phlebotomine sand flies. Those infected typically present with fever, weight loss, anaemia, leukopenia, thrombocytopenia, and enlargement of the spleen and liver. If left untreated, it can be fatal. Transmission varies by geographic region, as approximately 70 animal species have been identified as potential reservoir hosts of the parasite. The ICD9 code related to visceral leishmaniasis is 085.0, and the ICD10 code is B55.0.

Input data

No systematic review of literature in the PubMed database was undertaken for GBD 2016; however, updates of case notification, primarily from WHO country reports, were included.

We updated estimates of country-year-specific MI ratios by running a linear regression of the logit of the MI ratio on the log of income per capita using vital registration and inpatient hospital data from Brazil and Spain, two countries in which we had both reliable mortality and incidence data at the national level. This ratio was used in two ways; first, in assuming a duration of three months, we were able to derive excess mortality for use in DisMod. Second, the product of the M:I ratio and cases then estimated by DisMod, based upon incidence data, were used as death estimates for CoDCorrect.

Modelling strategy

The minimal amount of prevalence data available conflicted with the relationship between incidence and excess mortality data, as well as the remission prior (set to 4 based on duration assumptions), and thus was excluded from the model. No study-level covariates were used. The Socio-demographic Index (SDI) was used as a country-level covariate on the incidence data, with a floor of exp(-1) – as to allow a degree of regional and subnational variation while constraining the predictive power such that predictions in hypo-endemic countries with low SDI values and no data would not be unduly high.

In order to best represent the documented distribution of VL in India, we used the national fit from the DisMod model and redistributed it among the Indian states based on data from Bhunia, et al. Further, in order to control for DisMod fitting values to locations known to be devoid of VL, we replaced estimates in these locations with zeros. Then for locations with confirmed VL presence, we applied an underreporting factor reported in Alvar et al. Resultant incidence draws are then assumed to have a duration of three months, from which prevalence is calculated. Of those three months, three weeks are assumed to be spend with severe infection, and nine with moderate infection.

Changes from GBD 2015 to GBD 2016

We have made no substantive changes in the modelling strategy for endemic countries from GBD 2015 to GBD 2016.

3.3.5 Leprosy SDG Capstone Appendix

Flowchart

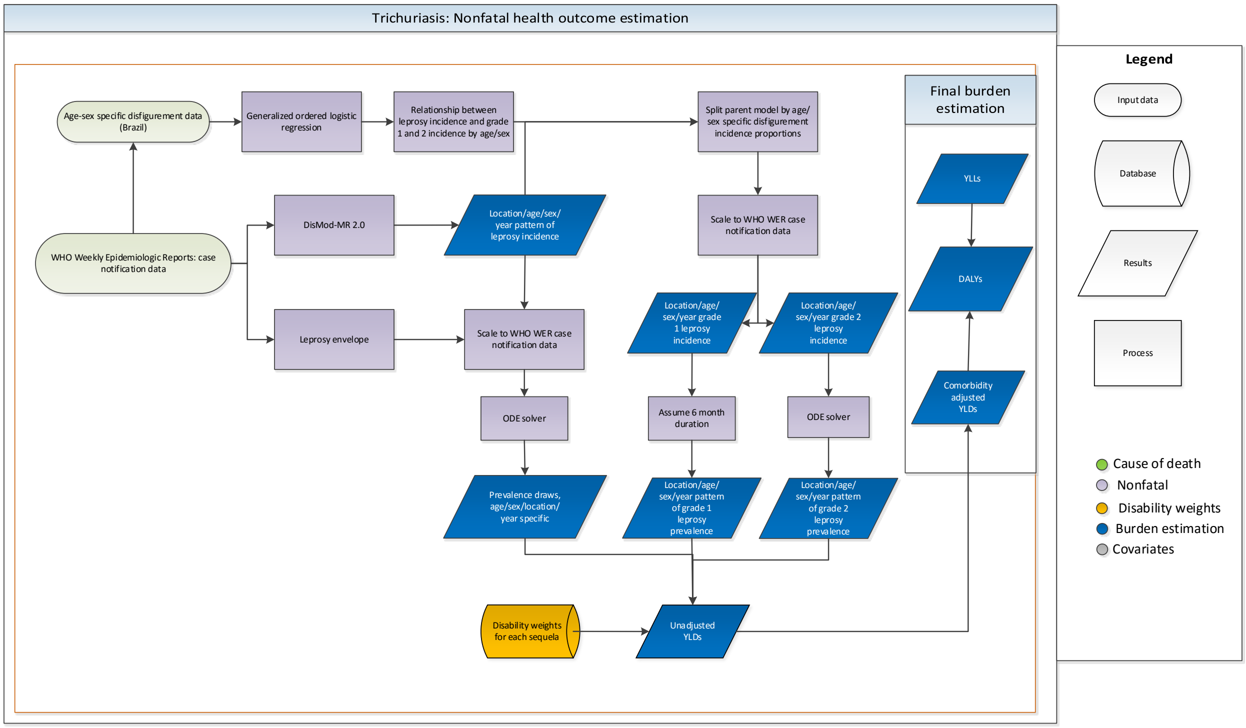

Input Data and Methodological Summary

Case definition

Leprosy is a chronic bacterial infection caused by *Mycobacterium leprae*, primarily affecting the nervous system, skin, respiratory tract, and eyes. Transmission is facilitated through contact with fluid from the nose and mouth of an infected individual. The ICD-10 codes for leprosy are A30.9.

Input data

To model nonfatal outcomes due to leprosy, WHO Weekly Epidemiological Record (WER) case notification data were used from 1987 to 2012 to capture incident cases of leprosy. This is the same database that was used to model GBD 2015 estimates, and due to the cyclical nature of systematic reviews for GBD causes, no data collection was scheduled for GBD 2016. As such, leprosy will be a priority for the next iteration of the study. Stage-specific incidence data for grade 1 and grade 2 leprosy that are used to define age-sex patterns came from Brazil case notification data.

Modelling strategy

We used a multi-step process for the disease modeling of leprosy. In the first step, we ran a single-parameter model using DisMod-MR 2.0 to estimate the leprosy incidence age pattern by age, sex, year, and country. Then, we scaled the incidence outputs to the WHO WER cases, and used the ordinary

differential equations (ODE) solver to calculate prevalence from the scaled DisMod-MR 2.0 incidence outputs.

Severity data were prepared by running a generalized ordered logistic regression using Brazil case notification data to get the relationship between leprosy incidence and grade 1 and grade 2 incidence by age and sex. We then used this relationship to split the parent DisMod-MR 2.0 model, and again scaled to WHO WER severity-specific cases. For disfigurement grade 1, we apply a duration of six months to get prevalence estimates. For disfigurement grade 2, we again use the ODE solver to get prevalence estimates.

Model evaluation was done by separately assessing the fit of the parent DisMod model and checking the final estimates produced after age-sex splits. Plots of time trends of prevalence across locations and age were used to evaluate the results. In addition, maps of the global distribution of leprosy prevalence and prevalence of sequelae due to leprosy were also assessed across time.

Changes from GBD 2015 to GBD 2016

We have made no substantive changes in the modelling strategy from GBD 2015 to GBD 2016.

3.3.5 Onchocerciasis SDG Capstone Appendix

Flowchart

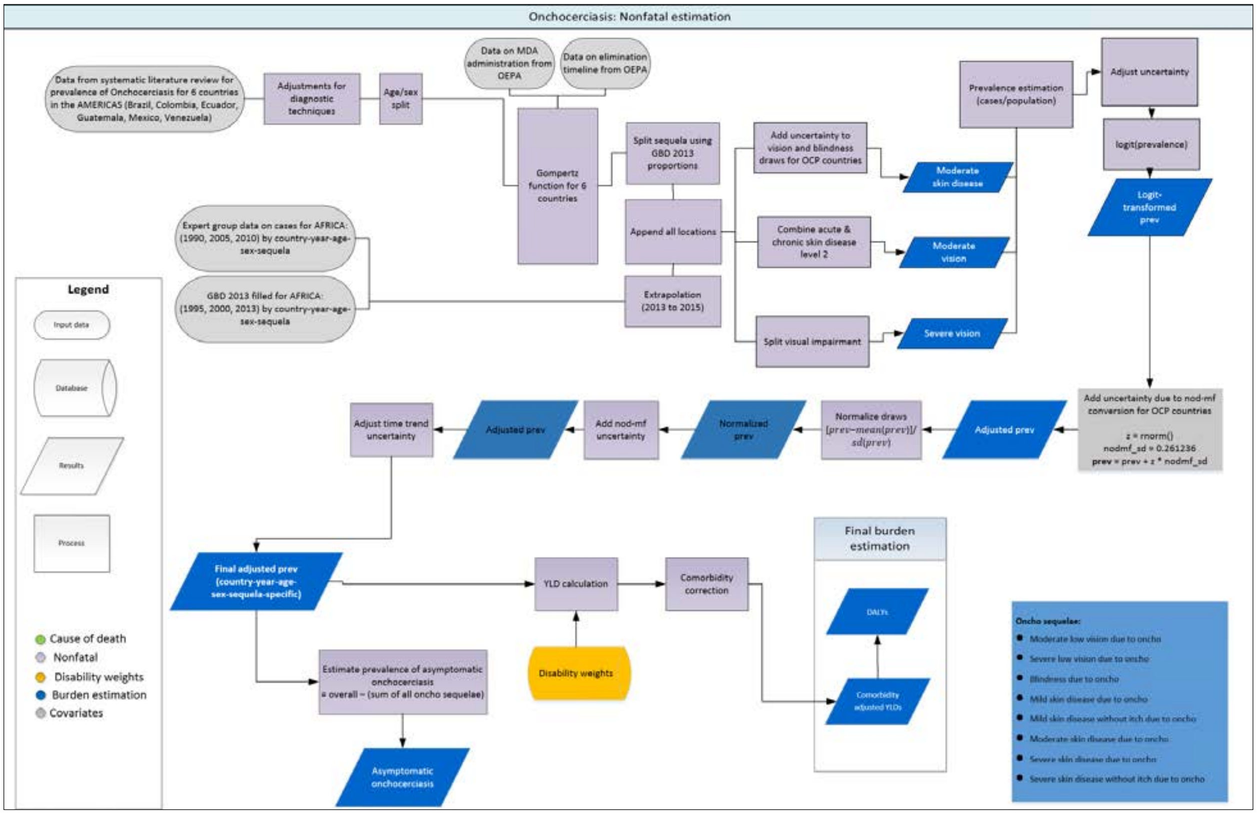

Input data & methodological summary

Case definition

Onchocerciasis, also known as river blindness, is a parasitic disease caused by the helminth *Onchocerca volvulus*. It is transmitted via the bite of one of several species of *Similium* blackflies that have historically bred in fast-moving freshwater rivers and tributaries throughout sub-Saharan Africa, Central America, and South America. Diagnosis can be made by skin snip biopsy to identify larvae, surgical removal of nodules and exam for adult worms, slit lamp exam of anterior part of the eye where larvae or lesions caused by them are visible, and antibody tests (mostly useful to visitors to areas with parasites). The ICD-10 code for onchocerciasis is B73.

Input data

Model inputs

Prevalence data prepared by the GBD 2010 expert group (EG) was used for modelling the nonfatal outcomes resulting from onchocerciasis in Africa. This included 1,000 draws of infection and morbidity (visual impairment, blindness, and skin conditions) cases with confidence intervals categorized by country, age, and sex for years 1990, 1995, 2000, 2005, and 2010. Details about the materials and

methods used by the EG to generate these draws can be found elsewhere [1-5]. These data represented all African countries included in the African Programme for Onchocerciasis Control (APOC) and the Onchocerciasis Control Programme (OCP) for which initial Rapid Epidemiological Mapping of Onchocerciasis (REMO) assessments demonstrated a need for Community-Directed Treatment with Ivermectin (CDTI) (defined as having a prevalence of skin nodules greater than 20%). Four countries – Rwanda, Mozambique, Kenya and Gabon – were designated as hypo-endemic countries after initial REMO assessments and not included due to sparsity of cases and paucity of data. Estimates for Sudan from GBD 2010 were reassigned to South Sudan in GBD 2013 after its independence in 2011 since REMO assessments indicated that the vast majority of cases occurred in that area of the former Sudan. The tables below show the countries included in each program and the number of corresponding GBD locations they represent.

	**APOC Countries**	**OCP Countries**
Countries included	Angola, Burundi, Cameroon, Central African Republic, Chad, Congo, Democratic Republic of Congo, Ethiopia, Equatorial Guinea, Liberia, Malawi, Nigeria, South Sudan, Tanzania, and Uganda	Benin, Burkina Faso, Côte d'Ivoire, Ghana, Guinea Bissau, Guinea, Mali, Niger, Senegal, Sierra Leone, and Togo
Hypo-endemic countries not included	Rwanda, Mozambique, Kenya, Gabon, Sudan	
GBD countries & subnationals	15	11
GBD world regions	3	1

Prevalence data for modelling non-fatal outcomes resulting from onchocerciasis in the Americas was extracted via a systematic literature review. Web of Science, Scopus, and PubMed were searched with the following search strings:

**Database**	**Search string**	**Yield**
PubMed	(oncho*[Title/Abstract] OR "river blindness"[Title/Abstract] OR "O. volvulus"[Title/Abstract] OR "robles disease"[Title/Abstract] OR "blinding filariasis"[Title/Abstract] OR "coast erysipelas"[Title/Abstract] OR “sowda” [Title/Abstract] OR “nodding syndrome”[Title/Abstract]) AND (“1980”[Date – Publication] : “2016”[Date – Publication]) AND (epidemiology[Title/Abstract] OR prevalence[Title/Abstract] OR incidence[Title/Abstract] OR surveillance[Title/Abstract] OR”MDA”[Title/Abstract] OR “Mass Drug Administration”[Title/Abstract] OR “Community-directed treatment with ivermectin”[Title/Abstract] OR “CDTI”[Title/Abstract] OR “mass treatment”[Title/Abstract] OR “multiple ivermectin treatments”[Title/Abstract] OR “monthly doses of ivermectin”[Title/Abstract] OR “large scale treatment”[Title/Abstract] OR REMO[Title/Abstract] OR “Rapid epidemiological mapping of onchocerciasis”[Title/Abstract] OR APOC[Title/Abstract] OR “African Programme for Onchocerciasis Control”[Title/Abstract] OR OCP[Title/Abstract] OR “Onchocerciasis Control Programme”[Title/Abstract]) NOT(Animals[MeSH] NOT Humans[MeSH])	986
Web of Science	TS=(oncho* OR "river blindness" OR "O. volvulus" OR "robles disease" OR "blinding filariasis" OR "coast erysipelas" OR sowda OR “nodding syndrome”) AND TS=(epidemiology OR prevalence OR incidence OR surveillance OR MDA OR “Mass Drug Administration” OR “Community-directed treatment with ivermectin” OR CDTI OR “mass treatment” OR	1,144

	“multiple ivermectin treatments” OR “monthly doses of ivermectin” OR “large scale treatment” OR REMO OR “Rapid epidemiological mapping of onchocerciasis” OR APOC OR “African Programme for Onchocerciasis Control” OR OCP OR “Onchocerciasis Control Programme”) NOT TS=((Animals NOT Humans))	
SCOPUS	(TITLE-ABS-KEY(oncho* OR "river blindness" OR "O. volvulus" OR "robles disease" OR "blinding filariasis" OR "coast erysipelas")) AND TITLE-ABS-KEY(epidemiology OR prevalence OR incidence OR surveillance OR MDA OR "Mass Drug Administration" OR "Community-directed treatment with ivermectin" OR CDTI OR "mass treatment" OR "multiple ivermectin treatments" OR "monthly doses of ivermectin" OR "large scale treatment" OR REMO OR "Rapid epidemiological mapping of onchocerciasis" OR APOC OR "African Programme for Onchocerciasis Control" OR OCP OR "Onchocerciasis Control Programme") AND NOT KEY(Animals NOT Humans) AND PUBYEAR > 1979	2,000

This yielded 4,130 results in total which was reduced to 2,502 after removing duplicates. The title and abstracts were screened for inclusion or exclusion with the following criteria:

Exclusion Criteria:

- Pre-1980
- Non-original source
- Non-representative population
    - Vulnerable populations (eg, slum-dwellers, prisoners, orphans, high-risk jobs, etc.)
    - Hospital-based samples (including saved stool samples)
    - Non-native peoples (eg, migrants, expats, nomads, etc.)
    - Immunosuppression/illness (eg, HIV, TB, CA, RA, asthma, malaria, handicap, etc.)
- Non-human population
- Does not meet case definition
- Case-control study

Sixty-one articles were identified for full text screening and extraction from the historically endemic American countries: Guatemala, Brazil, Ecuador, Venezuela, Mexico, and Colombia.

Severity splits/sequelae

The table below shows the list of common clinical manifestations of onchocerciasis and the sequelae to which they have been mapped along with the lay description and the associated disability weight (DW) of each sequela.

**Clinical manifestation**	**Sequela name**	**Lay description**	**DW**
Uveitis; Punctate keratitis; Optic neuritis; Torpid Iritis; Onchochorioretinitis	Moderate vision impairment	has vision problems that make it difficult to recognize faces or objects across a room	0.031 (0.019–0.049)
Sclerosing keratitis; Optic neuropathy; Optic atrophy; Choroidoretinopathy; Cataracts	Severe vision impairment	has severe vision loss, which causes difficulty in daily activities, some emotional impact (for example worry), and some difficulty going outside the home without assistance	0.184 (0.125–0.258)

Blindness	Blindness	is completely blind, which causes great difficulty in some daily activities, worry and anxiety, and great difficulty going outside the home without assistance	0.187 (0.124–0.260)
Acute papular onchodermatitis; Onchocercomata (subcutaneous nodules)	Mild skin disease	has a slight, visible physical deformity that is sometimes sore or itchy. Others notice the deformity, which causes some worry and discomfort	0.027 (0.015–0.042)
Chronic papular onchodermatitis; Lichenified obchodermatitis ("sowda"); Lymphadenopathy	Mild skin disease without itch	has a slight, visible physical deformity that others notice, which causes some worry and discomfort	0.011 (0.005–0.021)
Skin atrophy; Depigmentation ("leopard skin")	Moderate skin disease	has a visible physical deformity that is sore and itchy. Other people stare and comment, which causes the person to worry. The person has trouble sleeping and concentrating	0.188 (0.124–0.267)
Hanging groin; Lymphoedema	Severe skin disease without itch	has an obvious physical deformity that makes others uncomfortable, which causes the person to avoid social contact, feel worried, sleep poorly, and think about suicide	0.405 (0.275–0.546)
	Asymptomatic onchocerciasis	NA	NA

Modelling strategy

The nonfatal modelling for onchocerciasis included three major steps. In the first step, GBD 2010 prevalence was extrapolated to obtain GBD 2016 estimates. Uncertainty was quantified and provided by the EG for all estimates except those of moderate skin disease. In this case acute skin disease level 2 and chronic skin disease level 2 were summed to create the moderate skin disease sequela, and within each of the OCP draws the number of cases with visual impairment and blindness were multiplied by a random value (the exponent of a normally distributed variable with mean zero and standard deviation 0.1) in order to add uncertainty. Within each draw, the same randomly drawn value was applied to all country-year-age-sex estimates. Visual impairment was split into moderate and severe vision impairment by first multiplying the visual impairment estimates by a random value (from a normal distribution with mean 0.84 and standard deviation 0.0031) to generate moderate vision impairment, and then subtracting the resulting estimates from visual impairment to obtain estimates of severe vision impairment. Prevalence of sequelae was calculated by dividing the cases by the population.

The second step in modelling morbidity due to onchocerciasis was the adjustment of uncertainty in the conversion of nodule prevalence to microfilaria (mf) prevalence and in the effects of mass drug administration (MDA). To adjust for uncertainty in translation of nodule prevalence to mf prevalence, the final OCP draws from the first step were logit transformed uncertainty was added from a random value drawn from a normal distribution to the transformed estimates. The resulting estimates were then

normalized and scaled using estimates published elsewhere [1]. To adjust for uncertainty due to MDA, the year when MDA with Ivermectin started was set according to the table below.

Country	MDA start year
Angola, Burundi, South Sudan	2005
Congo, Ethiopia, DRC	2001
Cameroon, Central African Republic, Equatorial Guinea, Liberia, Nigeria, Uganda	1999
Chad, Niger, Tanzania	1998
Malawi	1997
All others	1990

The uncertainty in the time trend was then multiplied by the normalized prevalence estimates and the final prevalence was obtained by re-expanding the scaled normalized draws and adjusting the scale back from logit scale.

To estimate the prevalence of asymptomatic onchocerciasis, prevalence of morbidity (vision loss, blindness and skin conditions) was subtracted from the overall onchocerciasis prevalence – moderate vision impairment, severe vision impairment and blindness estimates were each multiplied by a factor of 8/33 before subtraction to account for cases that have concurring symptoms.

Model evaluation was done by separately assessing plots of time trends of prevalence across locations and age for each sequela. In addition, maps of the global distribution of total onchocerciasis prevalence and prevalence of sequelae due to onchocerciasis were also assessed across time.

In the final step, estimates for onchocerciasis in the Americas were modelled using Gompertz functions. Uncertainty was obtained by simulating across the deceleration parameter. The proportion of disease manifesting in each of the mapped sequela was derived from the proportion of sequela in the GBD 2010 estimates and uncertainty was added.

Changes from GBD 2015 to GBD 2016

Prevalence of onchocerciasis in foci in the Americas was not previously included but is now being modelled.

References

1. Zouré HG, Noma M, Tekle AH, Amazigo UV, Diggle PJ, Giorgi E, Remme JH. The geographic distribution of onchocerciasis in the 20 participating countries of the African Programme for Onchocerciasis Control: (2) pre-control endemicity levels and estimated number infected. Parasites & Vectors. 2014. 7-326.
2. Coffeng L, Stolk W, Hoerauf A, Habbema D, Bakker R, Hopkins A, de Vlas S. Elimination of African onchocerciasis: modeling the impact of increasing the frequency of ivermectin mass treatment. PLoS One. 2014. 9(12):e115886.
3. Coffeng LE, Stolk WA, Zouré HG, Veerman JL, Agblewonu KB, Murdoch ME, Noma M, Fobi G, Richardus JH, Bundy DA, Habbema D, de Vlas SJ, Amazigo UV. African Programme For Onchocerciasis Control 1995-2015: model-estimated health impact and cost. PLoS Negl Trop Dis. 2013; 7(1): e2032.

4. Murdoch ME, Asuzu MC, Hagan M, Makunde WH, Ngoumou P, Ogbuagu KF, Okello D, Ozoh G, Remme J. Onchocerciasis: the clinical and epidemiological burden of skin disease in Africa. Ann Trop Med Parasitol. 2002; 96(3): 283-296.
5. Brieger WR, Awedoba AK, Eneanya CI, Hagan M, Ogbuagu KF, Okello DO, Ososanya OO, Ovuga EB, Noma M, Kale OO, Burnham GM, Remme JH. The effects of ivermectin on onchocercal skin disease and severe itching: results of a multicentre trial. Trop Med Int Health. 1998; 3(12): 951-61.

3.3.5 Rabies SDG Capstone Appendix

Flowchart

Rabies

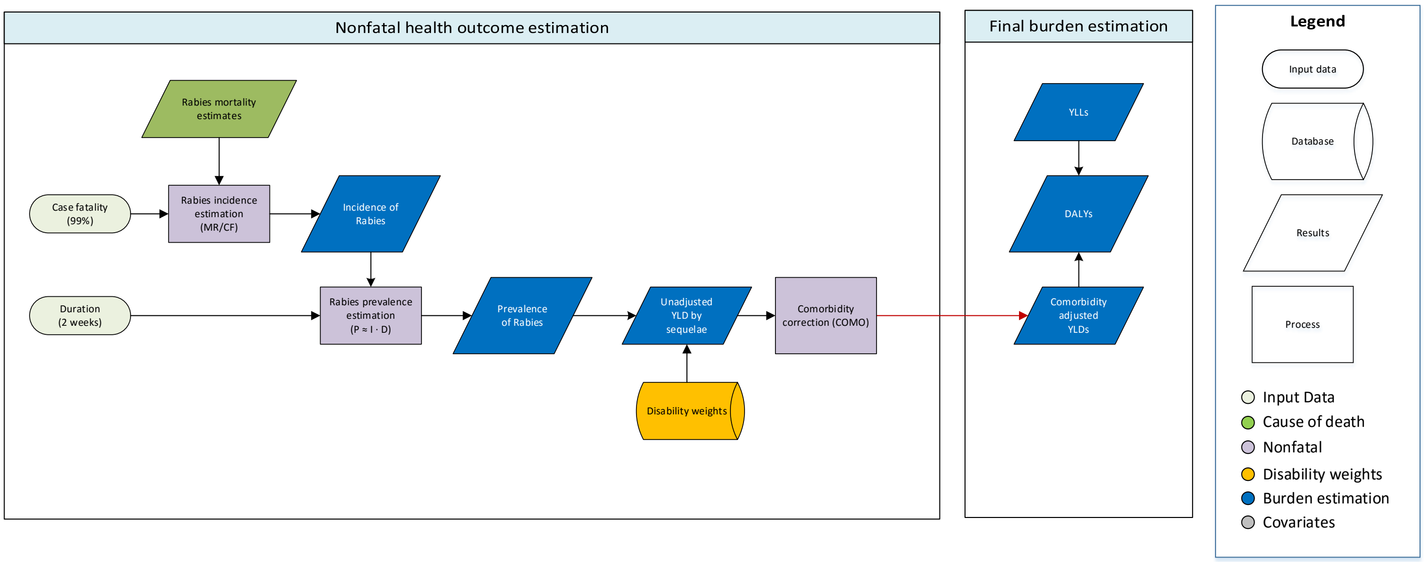

Input data and methodological summary

Case definition

Rabies is a fatal viral infection, transmitted by animal bites. Without prophylactic vaccination the disease is almost universally fatal. The disease has a long incubation period (1-3 months), and early intervention with prophylactic vaccination is nearly 100% effective in preventing symptomatic disease. It is considered a neglected tropical disease (NTD). We model symptomatic infections, not including those infections in which intervention prevented the onset of symptomatic disease, corresponding to the ICD10 code A82.

Input data

Model inputs

As we derive our estimate of cases from our estimate of deaths, no incidence data are used in the model. For GBD 2016, we modelled rabies mortality using all available data in the cause of death database. Data points were outliered if they reported an improbable number of rabies deaths (eg, zero rabies deaths in a hyperendemic country) or if their inclusion in the model yielded distorted trends. In some cases multiple data sources for the same location differed dramatically both in their quality and reported rabies mortality (eg, a verbal autopsy and vital registration source). In these cases the lower-quality data source was outliered.

Modelling strategy

We derive estimates of the number of symptomatic rabies infections (ie, those not averted through prophylactic vaccination) based on rabies mortality estimates, assuming 99% case fatality. All cases are assumed to be severe.

We modelled rabies mortality using a two-model hybrid approach 1) a global CODEm model of all locations, using all data in the CoD database; and 2) a CODEm model restricted to data-rich countries. We have made two substantive changes in the modelling strategy from GBD 2013. First, we have changed from a single global model to the hybrid global/data-rich model approach. Second, we conducted an exploratory analysis to determine the most predictive covariates for rabies and have updated the covariates used in the CODEm model accordingly.

Sequela description and DW

There is only one sequela and associated disability weight for rabies, which is severe. The lay description is included in the table below.

Table 2. Sequela, description, and DW

Sequela	Description	Disability Weight (95% CI)
Severe	Has a high fever and pain, and feels very weak, which causes great difficulty with daily activities.	0.133 (0.088–0.19)

Changes from GBD 2015 to GBD 2016

We have made no substantive changes in the modelling strategy for rabies from GBD 2015.

3.3.5 Schistosomiasis SDG Capstone Appendix

Flowchart

Schistosomiasis

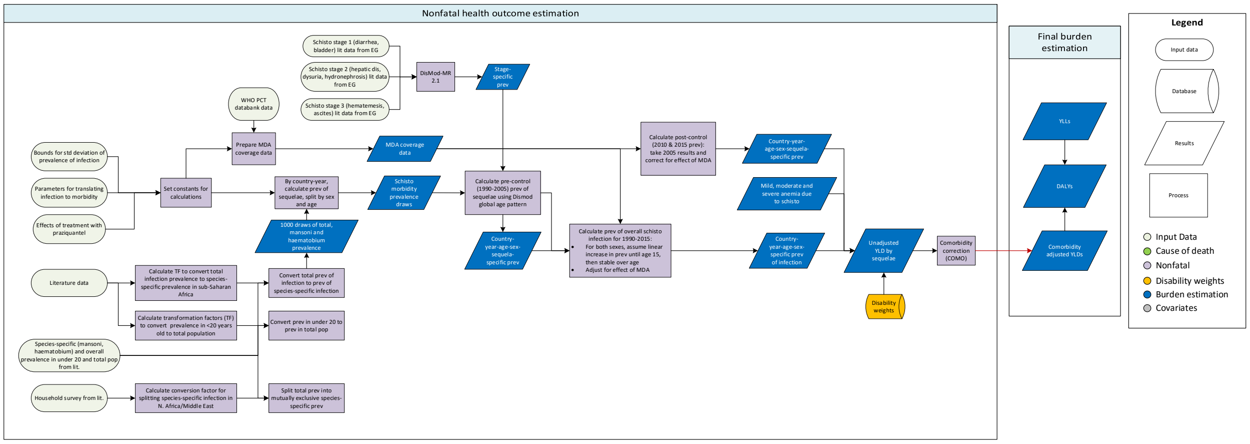

Case definition

Schistosomiasis, also known as bilharzia or “snail fever,” is a helminth disease caused by infection with five species of the parasite *Schistosoma,* namely, *S. mansoni, S. japonicum, S. haematobium, S. mekongi, and S. intercalatuma*. It is considered a neglected tropical disease (NTD). The first three species cause the most infection and the last two rarely cause disease. Diagnosis is made by microscopic exam of stool or urine for parasite eggs. For less advanced infections, serologic techniques are used. The ICD-10 codes for schistosomiasis are B65-B65.9.

Input data

Model inputs

To model nonfatal outcomes due to schistosomiasis, we conducted a systematic literature review, extracting prevalence data from 1980 to 2016 for the five species of schistosomiasis listed above. The search string used in the systematic review is (schistosom*[Title/Abstract] OR bilharzia*[Title/Abstract] OR "snail fever"[Title/Abstract]) AND ("1990"[Date - Publication] : "3000"[Date - Publication]) AND (epidemiolog* OR inciden* OR prevalen* OR seroprevalen*) NOT (animals[mesh] NOT humans[mesh]). Additionally, we used data compiled by the Global Atlas of Helminth Infections (GAHI), which includes grey literature and unpublished data.

Population at risk/mass drug administration data

Population at risk estimates and MDA data were taken from the WHO PCT Databank [1].

Severity splits/sequelae

The table below shows the list of clinical sequelae (including mild, moderate, and severe anaemia) due to schistosomiasis, their lay descriptions, and the associated disease stages and disability weights. Using

literature [1], a list of eight possible clinical sequelae and anaemia sequelae were defined (mild infection, mild diarrhoea, haematemesis (vomiting blood), hepatomegaly, ascites (buildup of fluid in the peritoneal cavity), dysuria (painful urination), bladder pathology, hydronephrosis (swelling of kidney due to buildup of urine in the kidney), mild anaemia, moderate anaemia, and severe anaemia).

Table 2. Clinical sequela, lay descriptions, disease stages, and DWs

Clinical sequela	Lay description	Disease stage	Disability weights (DWs)
Mild infection	has a low fever and mild discomfort , but no difficulty with daily activities	1	0.006 (0.002–0.012)
Mild diarrhoea		1	0.056
Hepatomegaly	has some pain in the belly that causes nausea but does not interfere with daily activities	2	0.011 (0.005–0.021)
Dysuria	has some pain in the belly that causes nausea but does not interfere with daily activities	2	0.011 (0.005–0.021)
Hydronephrosis	has some pain in the belly that causes nausea but does not interfere with daily activities	2	0.011 (0.005–0.021)
Haematemesis	vomits blood and feels nauseated	3	0.325 (0.209–0.463)
Ascites	has pain in the belly and feels nauseated. The person has difficulties with daily activities	3	0.114 (0.078–0.159)
Bladder pathology	has some pain in the belly that causes nausea but does not interfere with daily activities	3	0.011 (0.005–0.021)
Mild anaemia	feels slightly tired and weak at times, but this does not interfere with normal daily activities	NA	0.004 (0.001–0.008)
Moderate anaemia	feels moderate fatigue, weakness, and shortness of breath after exercise, making daily activities more difficult	NA	0.052 (0.034–0.076)
Severe anaemia	feels very weak, tired, and short of breath, and has problems with activities that require physical effort or deep concentration	NA	0.149 (0.101–0.210)

Modelling strategy

The morbidity model for schistosomiasis involved a multi-step process. First, we ran a single-parameter prevalence model in DisMod-MR 2.0 using the prevalence data extracted in the systematic review and from the GAHI database. We make the assumption that all of our data are measured within a population at risk – therefore, the estimates from the DisMod model represent prevalence estimates among the population at risk for schistosomiasis. Additionally, we included the MDA treatment data from the WHO as a country-level covariate in the DisMod model. Second, we then scaled the prevalence estimates to the population at risk estimates from the WHO PCT Databank to get age/sex/location/year all-schistosomiasis prevalence envelopes. 3) We ran a generalized linear model to get species-specific proportional prevalence on data from literature that reported both *S. haematobium* and *S. mansoni* infection, and 4) literature-informed parameters (a, b, c) for translating infection (x) to morbidity (y): y = (a + bx^c)/(1 + bx^c) – a [2-4]. We used the species-specific conversion factors calculated in step (3) to split the all-schistosomiasis envelope into species-specific schistosomiasis. We then used the parameters

determined in step (4) to translate infection into morbidity to get age/sex/year/location-specific prevalence of sequelae. The burden of anemia due to schistosomiasis was estimated (see anaemia documentation for details).

Model evaluation was done by separately assessing the fit of the single-parameter DisMod models and checking the final estimates produced after age-sex splits. Plots of time trends of prevalence across locations and age were used to evaluate the results. In addition, maps of the global distribution of total schistosomiasis prevalence and prevalence of sequelae due to schistosomiasis were also assessed across time.

Changes from GBD 2015 to GBD 2016

The main change made from GBD 2015 was the systematic review and using extracted data in a DisMod model to estimate prevalence within the population at risk. In addition, newly updated data from the WHO PCT databank were downloaded and used in the model, and geographic restrictions were updated.

References

1. World Health Organization (WHO). WHO PCT Databank - Schistosomiasis. Geneva, Switzerland: World Health Organization (WHO).
2. van der Werf MJ, de Vlas SJ, Brooker S, et al. Quantification of clinical morbidity associated with schistosome infection in sub-Saharan Africa. Acta Trop. 2003;86(2-3):125-39
3. van der Werf MJ, de Vlas SJ, Looman CW, Nagelkerke NJ, Habbema JD, Engels D. Associating community prevalence of Schistosoma mansoni infection with prevalence of signs and symptoms. Acta Trop. 2002;82(2):127-37
4. van der Werf MJ, de Vlas SJ. Diagnosis of urinary schistosomiasis: A novel approach to compare bladder pathology measured by ultrasound and three methods for hematuria detection. Am. J. Trop. Med. Hyg. 2004;82:98-106

3.3.5 Vision impairment due to Trachoma SDG Capstone Appendix

Flowcharts

Vision Impairment

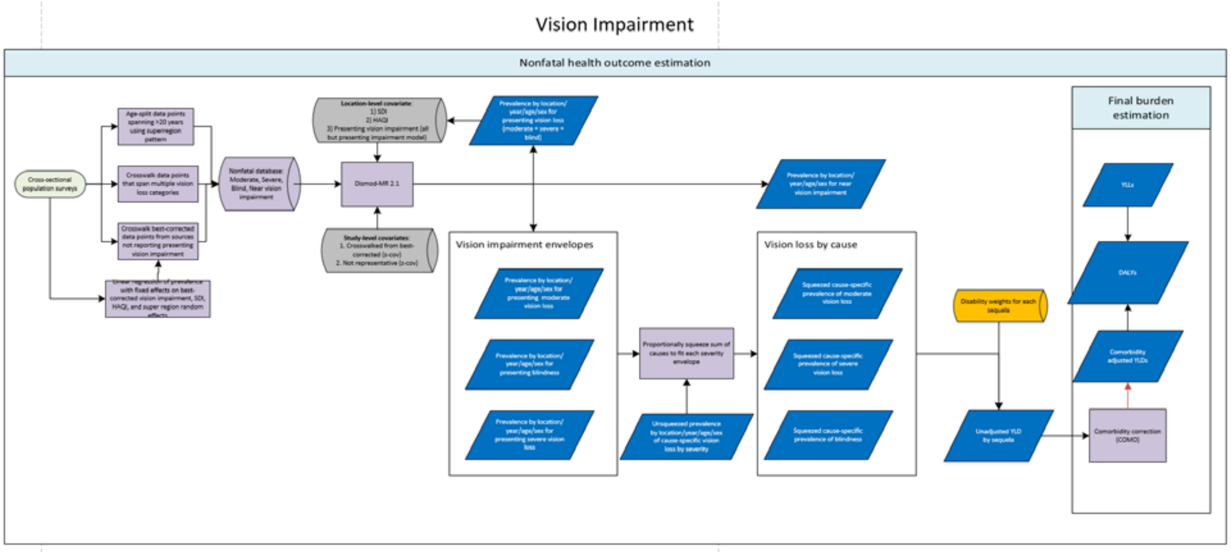

Diabetic Retinopathy

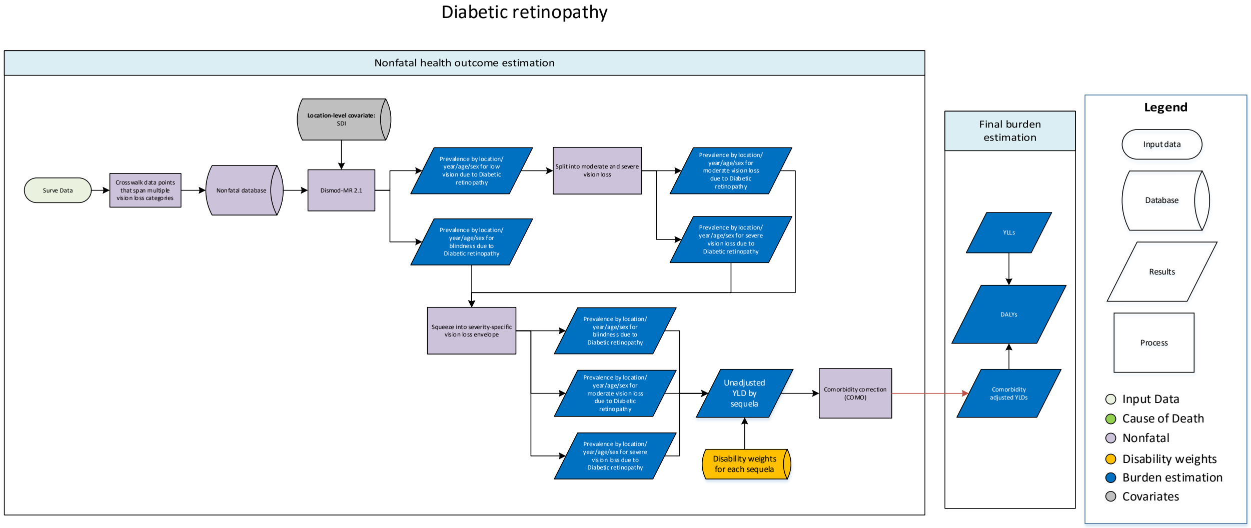

Macular Degeneration

Macular degeneration

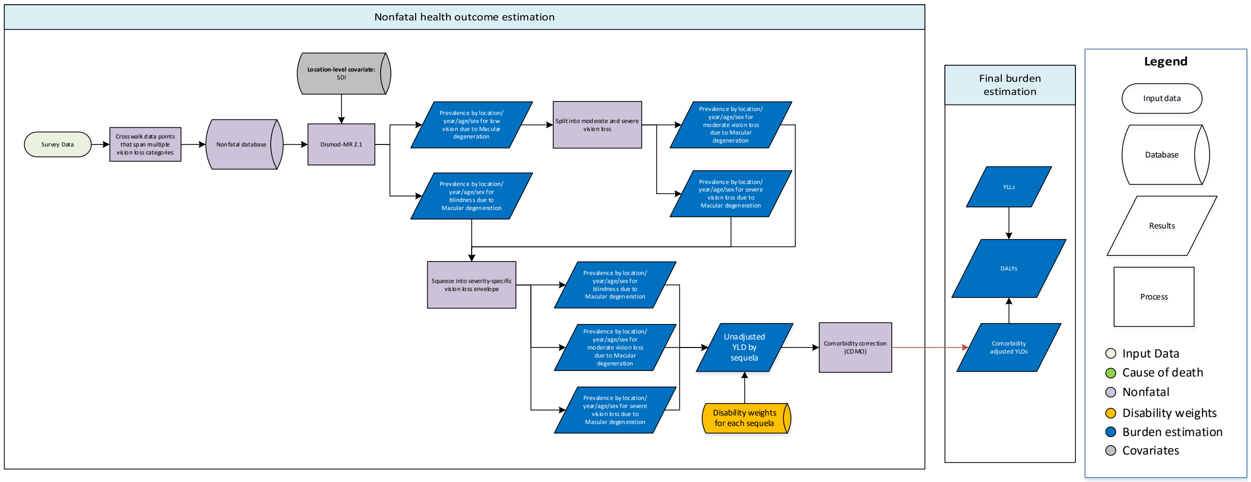

Cataract

Cataract

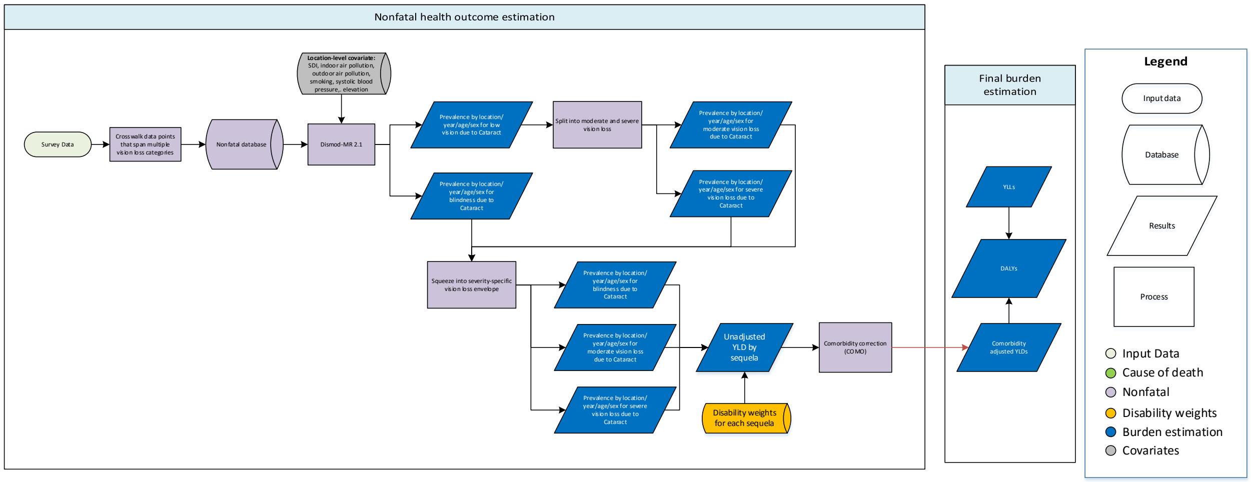

Glaucoma

Glaucoma

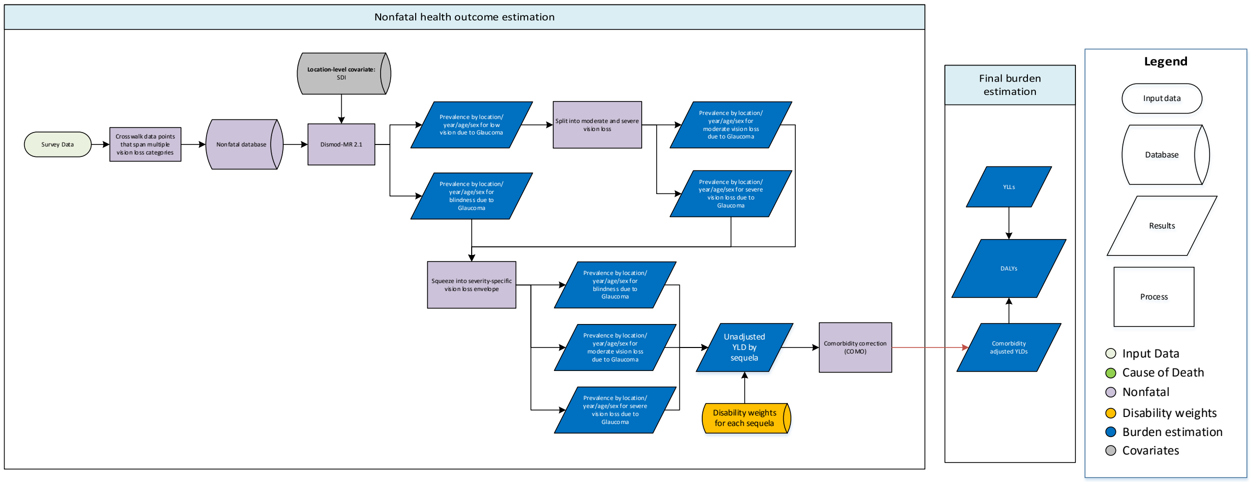

Trachoma

Trachoma

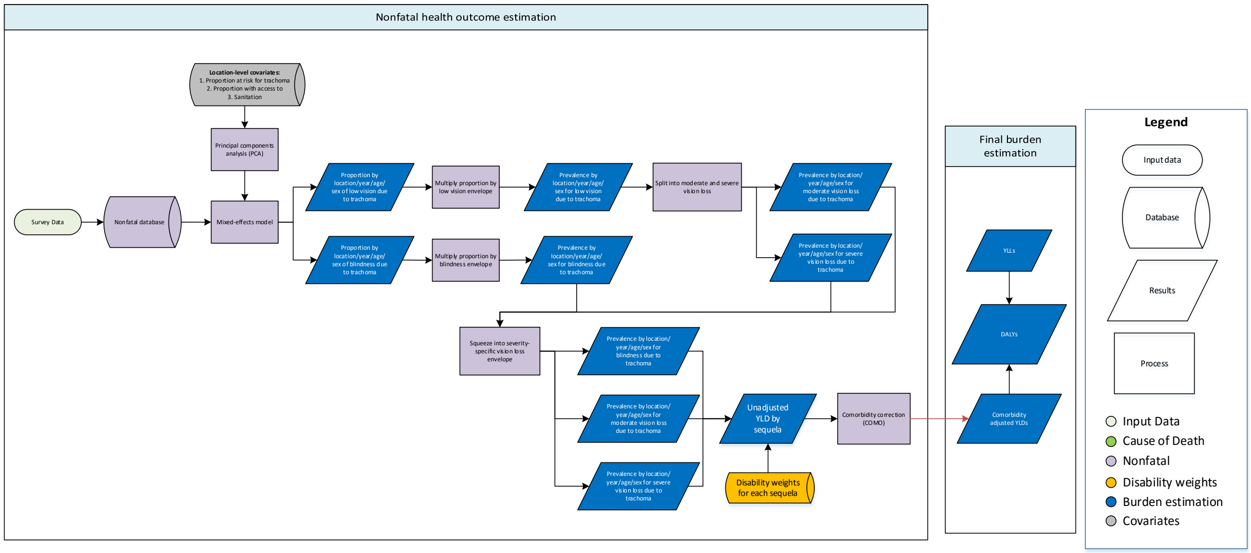

Other vision loss

Other vision loss

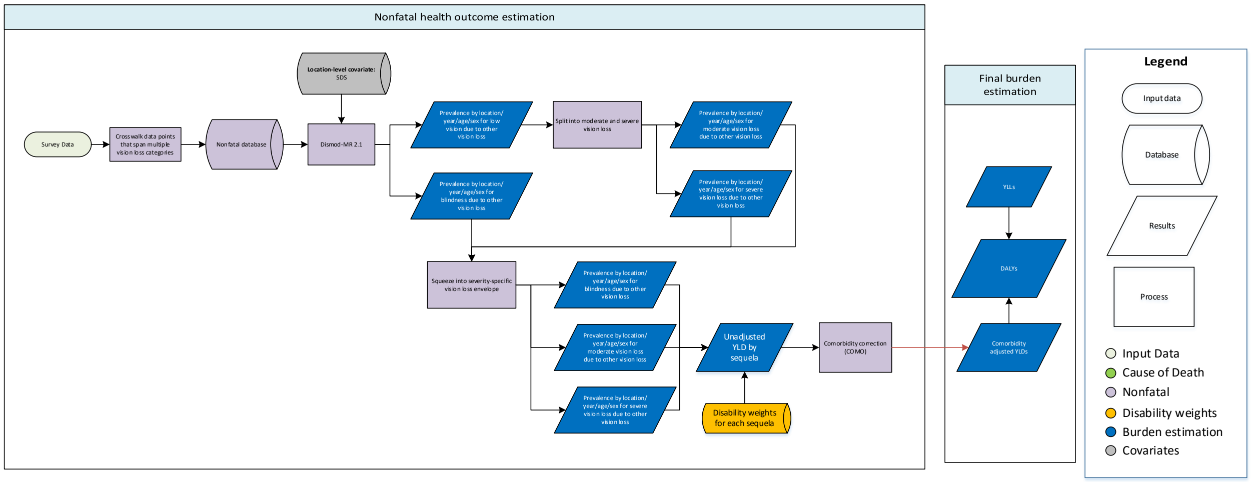

Case definition

We model vision impairment as visual acuity <6/18 according to the Snellen chart. The following impairments are modeled:

Condition	Case definition
Blindness	Visual acuity of <3/60 or <10% visual field around central fixation
Severe vision impairment	≥3/60 and <6/60
Moderate vision impairment	≥6/60 and <6/18
Near vision impairment envelope	Near visual acuity of <6/18 distance equivalent

Near vision impairment describes the progressive inability to focus on near objects as individuals age, and is also called presbyopia. This impairs the ability to read. The majority of presbyopia can be corrected by the use of reading glasses, contact lenses, or refractive surgery.

We model vision impairment due to the following causes: uncorrected refractive error, cataract, glaucoma, macular degeneration, diabetic retinopathy, trachoma, Vitamin A deficiency, retinopathy of prematurity, meningitis, encephalitis, onchocerciasis, and other vision loss. Vision loss due to vitamin A deficiency, retinopathy of prematurity, meningitis, encephalitis, and onchocerciasis are modelled as part of their underlying cause as described in their respective sections.

Refractive error is blurry vision due to the lens's inability to focus. The blurriness caused by refractive error can be addressed through the use of contact lenses, glasses, or refractive surgery. Cataract is

clouding of the lens of the eye due to protein buildup that impairs vision. Glaucoma is a condition with increased intraocular pressure which can lead to damage of the optic nerve. Macular degeneration is a deterioration of the macula, leading to central vision loss. Diabetic retinopathy is damage to the retina caused by damaged blood vessels that can leak blood into the retina and cause scarring of the retina. Trachoma results from a conjunctival bacterial infection (*Chlamydia trachomatis*) that produces inflammation and scarring which leads to an inversion of the eyelids and eyelashes scratching the cornea, which eventually leads to scarring of the cornea and vision impairment or blindness.

Input data

Model inputs

Data on overall vision impairment come from surveys measuring visual acuity in representative population-based studies, either from publications in peer-reviewed and grey literature or surveys for which we had the unit record data. Data were excluded if no test was used of visual acuity that can be converted to the Snellen scale, and if a study did not assess "presenting" or "best-corrected" vision. A subset of these studies that reported vision loss by cause were used to estimate the prevalence of vision loss due to cataract, glaucoma, macular degeneration, diabetic retinopathy, and other causes.

For GBD 2015, we conducted a systematic review for new sources since GBD 2013 (covering 1/1/2013 – 5/20/2015), using the following search string:

((((glaucoma[Title/Abstract] OR cataract[Title/Abstract] OR macular[Title/Abstract] OR 'refractive error'[Title/Abstract] OR presbyopia[Title/Abstract]) OR (('blindness'[MeSH Terms] OR 'blindness'[All Fields]) OR 'vision, low'[MeSH Terms])) AND ('2013'[PDAT] : '3000'[PDAT])) AND 'humans'[MeSH Terms]) AND (prevalence[Title/Abstract] OR incidence[Title/Abstract] OR epidemiology[Title/Abstract])

This yielded 1,169 results, of which we extracted 20 sources. Furthermore, we extracted from the following nationally representative surveys measuring visual acuity: the WHO Studies on Global Ageing and Adult Health (SAGE) and the United States National Health and Examination Surveys (NHANES).

For GBD 2016, we did a comprehensive extraction of the Rapid Assessment of Avoidable Blindness (RAAB) repository (http://raabdata.info/), a database of vision impairment studies in developing settings across the world. There are 266 site-years of data, the majority of which have publicly available reports or publications of the data. A standardized methodology was used by all sources in the repository, allowing inclusion of all available reports. In addition, we added two state-level national surveys from India.

Due to the sparse literature reporting measured near-vision visual acuity, we also extracted data from the following nationally representative studies measuring self-reported near vision loss: SAGE; NHANES; the Surveys of Health, Ageing, and Retirement in Europe (SHARE); the Multi-Country Survey Study on Health and Responsiveness (MCSS); and the World Health Surveys (WHS).

Several adjustments were made to raw data.

1) Where studies reported visual acuity spanning multiple thresholds (eg, <6/60, rather than separate severe and blind estimates), we crosswalked using ratios predicted by a linear regression on age, using data from studies reporting vision loss by each severity.

2) Some studies reported best-corrected vision impairment, but not presenting vision impairment (PVI). We crosswalked these data points using a linear regression of logit-transformed PVI prevalence with fixed effects on best-corrected VI, healthcare quality and access index (HAQI) and Socio-demographic Index (SDI) and super-region random effects. This gave us a predicted PVI data points for these studies not explicitly reporting PVI. These crosswalked data points were flagged with a study-level covariate that increased standard error in DisMod.
3) Where data points spanned more than 20 years of age, we age-split using an algorithm that applies the age-pattern of the super-region to split the data to five-year age groups.

Whereas other vision impairment aetiologies are modelled based on prevalence data, vision impairment due to trachoma is modelled as a proportion of the overall vision impairment envelope, a strategy that was chosen based on the nature of available data.

Health states and disability weights

Health state name	Health state description	Disability weight
Distance vision, severe impairment	This person has severe vision loss, which causes difficulty in daily activities, some emotional impact (for example, worry), and some difficulty going outside the home without assistance.	0.184 (0.125–0.259)
Distance vision, moderate impairment	This person has vision problems that make it difficult to recognize faces or objects across a room.	0.031 (0.019–0.049)
Distance vision blindness	This person is completely blind, which causes great difficulty in some daily activities, worry and anxiety, and great difficulty going outside the home without assistance.	0.187 (0.124–0.26)
Presbyopia	This person has difficulty seeing things that are nearer than 3 feet, but has no difficulty with seeing things at a distance.	0.011 (0.005–0.02)

Modelling strategy

We modelled the prevalence of vision loss in two steps. In the first step, we estimated the total prevalence estimates of presenting vision loss: moderate vision impairment, severe vision impairment, blindness, and near vision impairment (presbyopia). We directly derived prevalence of near vision impairment from this step, whereas the remaining three models that reflect different severity levels of distance vision loss continued to the next step.

1) Estimate severity-specific vision impairment (the "envelopes")

First, we ran five DisMod-MR 2.1 models to estimate the total prevalence estimates of presenting vision loss: moderate vision impairment, severe vision impairment, blindness, near vision impairment (presbyopia), and presenting vision impairment (moderate + severe + blindness). The presenting vision impairment model was used as a covariate in the severity-specific models to improve consistency across severities.

Betas and exponentiated values, which can be interpreted as an odds ratio, are shown in the table below for each covariate. The best-corrected covariate indicates whether the test measures visual acuity with the level of correction the patient presents with (best_corrected = 0) or the ophthalmologist provides additional correction via pinhole (best_corrected = 1). Rapid-assessment corrects for potential biases in cause-specific vision loss from studies using expedited visual acuity measurement. Socio-demographic

Index (SDI) and healthcare access and quality index (HAQI) are used as location covariates as a proxy measure of access to eye care such as cataract surgery. Non-representative studies are those not representative at the level they are used to model (eg, a state-level survey assigned to a country), including a z-cov adjusts for potential bias. Data points that were crosswalked from best-corrected visual acuity are flagged with a z-cov to adjust uncertainty in the crosswalk process. Non-standard severity definition is used to crosswalk between the self-report questionnaire of SHARE (nonstandard) and the other surveys, including SAGE and NHANES, which are crosswalked to examination data using the self-reported covariate.

2) Estimate cause-specific vision impairment

In the second step, we estimated the prevalence of vision loss due to multiple causes: refractive error, cataract, glaucoma, macular degeneration, diabetic retinopathy, retinopathy due to prematurity, trachoma, vitamin A deficiency, onchocerciasis, meningitis, and other causes not classified elsewhere. The vision loss due to retinopathy of prematurity, vitamin A deficiency, onchocerciasis, meningitis, tetanus, and neonatal conditions was modeled as part of these underlying causes. Vision loss due to trachoma is modelled as a proportion of the envelope, with separate proportion models for vision impairment and blindness. For each of cataract, glaucoma, macular degeneration, diabetic retinopathy, and other vision loss, we ran two DisMod-MR 2.1 models: one for the combined category of moderate and severe vision loss due to the cause, and one for blindness due to the cause. Moderate and severe vision loss were modelled together because input data were mostly available for the aggregate. Refractive error was modelled in three models, one for each severity. We used the following age restrictions:

Cause	Minimum age
Cataracts	20
Glaucoma	45
Macular degeneration	45
Diabetic retinopathy	20
Trachoma	15
Other vision loss	0

For the cataract model, we used known risk factors – hypertension, smoking, air pollution, and elevation.

For cataract and refractive error, we used presenting vision impairment as a covariate, as these are the main causes of vision impairment and are treatable and thus should have greater covariance with overall vision impairment than less common causes such as glaucoma or macular degeneration.

We estimated the proportions of low vision and blindness due to trachoma using custom mixed-effects models. For consistency, the two models (blindness and low vision) were parameterized identically and differ only in their input data. Our model included fixed effects on age (using cubic splines with knots at 0, 40, and 100 years of age), sex, and a covariate derived from a principal components analysis of the proportion of the population at risk for trachoma and the proportion of the population with access to sanitation. We included nested random effects on super-region, region, and country. Finally, we applied geographic and age restrictions to ensure that we estimate zero proportions in non-endemic locations and among those younger than 15 year of age (as scarring of the cornea due to trachoma takes decades to develop). The prevalence of trachoma at each severity level was calculated by multiplying the

proportion of vision loss (vision impairment or blindness) due to trachoma by the corresponding best-corrected vision loss envelope.

We split the moderate plus severe vision loss estimates for each cause into moderate and severe using the ratio of best-corrected moderate and severe vision loss envelopes. As exceptions, onchocerciasis and retinopathy of prematurity were modelled for moderate and severe vision loss as part of the estimation process of these causes.

We scaled the cause-specific vision loss prevalence to the total prevalence of the best-corrected vision loss envelopes for each of the three severity levels. The final result is prevalence of vision loss due to each cause by severity.

The following changes have been implemented since GBD 2015:

- DisMod is not designed to handle wide-age data points – by age-splitting the input data we improve model fits.
- In the severity-specific vision impairment models, we use overall presenting vision impairment as a covariate, ensuring greater consistency between severities.
- In GBD 2013 vision impairment models, best-corrected vision data were crosswalked within DisMod using a single beta for all ages and locations. By crosswalking the input data, we allow the ratio between presenting and best-corrected vision impairment to vary with age and location.
- In GBD 2013, we estimated the ratio of vision impairment due to refractive error. In 2016, we are estimating the prevalence of refractive error, as it shows greater covariance with predictors such as SDI and HAQI. This allows the second step (squeezing causes to the envelopes) to include refractive error as an input.

3.4.1 Non-communicable Disease (NCD) Mortality SDG Capstone Appendix

Cardiovascular diseases, cancers, diabetes mellitus, and chronic respiratory diseases

Indicator definition

This modeling strategy encompasses the indicator associated with non-communicable disease mortality (3.4.1).

Indicator 3.4.1

As a component of SDG Goal 3. Ensure healthy lives and promote well-being for all at all ages, SDG Target 3.4, by 2030, reduce by one third premature mortality from NCDs through prevention and treatment and promote mental health and well-being, is measured using SDG Indicator 3.4.1, deaths due to cardiovascular disease, cancer, diabetes, and chronic respiratory disease among populations aged 30 to 70 per 100,000.

3.4.1 Cardiovascular Diseases SDG Capstone Appendix

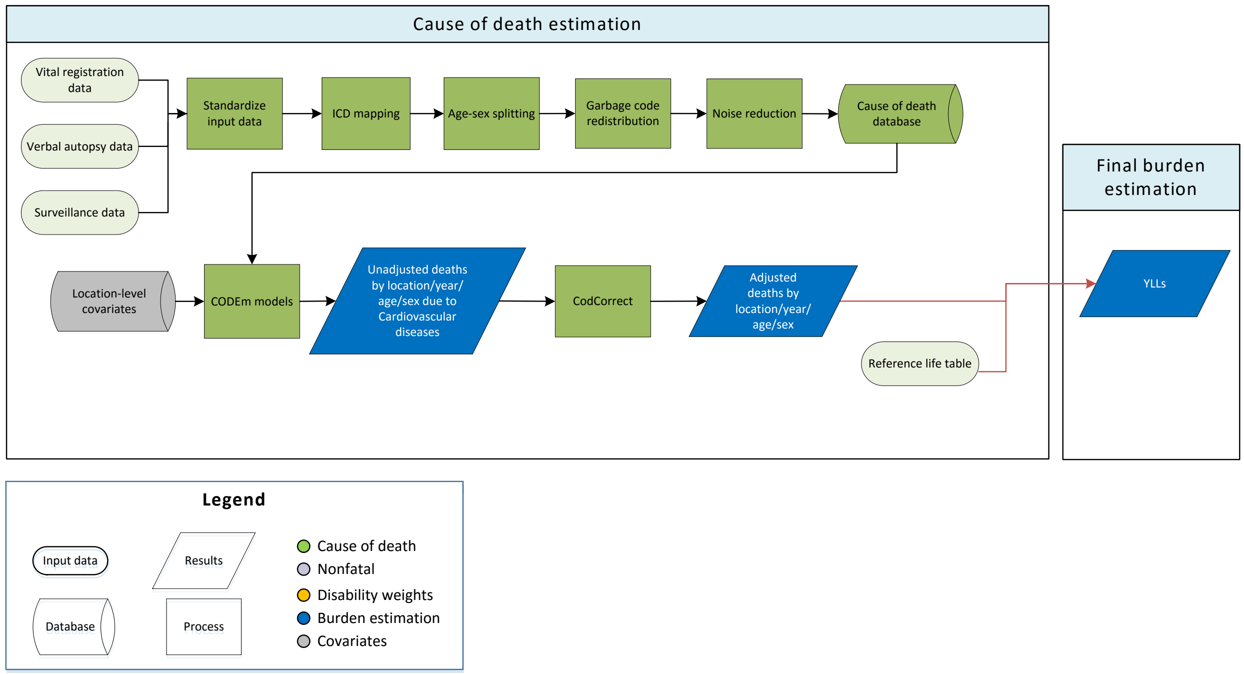

Input data

Vital registration, verbal autopsy, and surveillance data were used to model this cause. We outliered non-representative subnational verbal autopsies from a number of Indian states. We also outliered verbal autopsy data sources that were implausibly low in all age groups and ICD8 and ICD9 BTL data points that were inconsistent with the rest of the data and created implausible time trends.

Modelling strategy

We used a standard CODEm approach to model deaths from cardiovascular diseases. We have updated the covariates included in the ensemble modelling process (see Table). Otherwise, there have been no substantive changes from the approach used in GBD 2015.

Table: Selected covariates for CODEm models, cardiovascular diseases

Covariate	Transformation	Level	Direction
Summary exposure variable	None	1	1
Cholesterol (total, mean per capita)	None	1	1
Smoking prevalence	None	1	1
Systolic blood pressure (mmHg)	None	1	1
Trans fatty acid	None	1	1
Mean BMI	None	2	1
Elevation over 1500m (proportion)	None	2	-1
Fasting plasma glucose (mmol/L)	None	2	1
Outdoor pollution ($PM_{2.5}$)	None	2	1
Indoor air pollution (all fuel types)	None	2	1
Healthcare access and quality index	None	2	-1
Lag distributed income per capita (I$)	Log	3	-1
Socio-demographic Index	None	3	0
Omega-3 (kcal/capita, adjusted)	Log	3	-1
Fruits (kcal/capita, adjusted)	None	3	-1
Vegetables (kcal/capita, adjusted)	None	3	-1
Nuts and seeds (kcal/capita, adjusted)	None	3	-1
Whole grains (kcal/capita, adjusted)	None	3	-1
Pulses/legumes (kcal/capita, adjusted)	None	3	-1
PUFA adjusted (percent)	None	3	-1
Alcohol (litres per capita)	None	3	0

Rheumatic Heart Disease

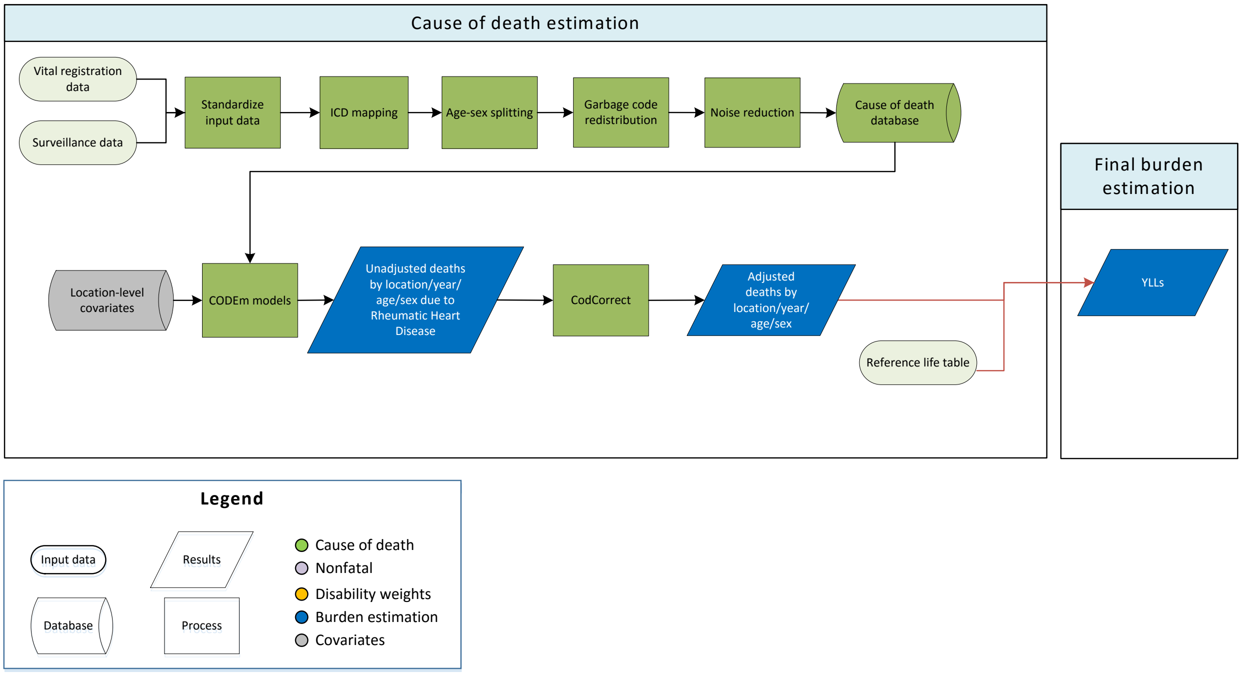

Input data

Vital registration and surveillance data were used to model rheumatic heart disease. We outliered ICD8 and ICD9 BTL data points which were inconsistent with the rest of the data and created implausible time trends. We also outliered data points which were too high after the redistribution process in a number of age groups.

Modelling strategy

We used a standard CODEm approach to model deaths from rheumatic heart disease. We have updated the covariates included in the ensemble modelling process (see Table). Otherwise, there have been no substantive changes from the approach used in GBD 2015.

Table: Selected covariates for CODEm models, rheumatic heart disease

Covariate	Transformation	Level	Direction
SEV	None	1	1
Improved water (proportion)	None	1	-1
Malnutrition	None	1	1
Sanitation (proportion with access)	None	1	-1
Healthcare access and quality index	None	2	-1
LDI	Log	3	-1
SDI	None	3	-1
Education (years per capita)	None	3	-1

Ischemic Heart Disease

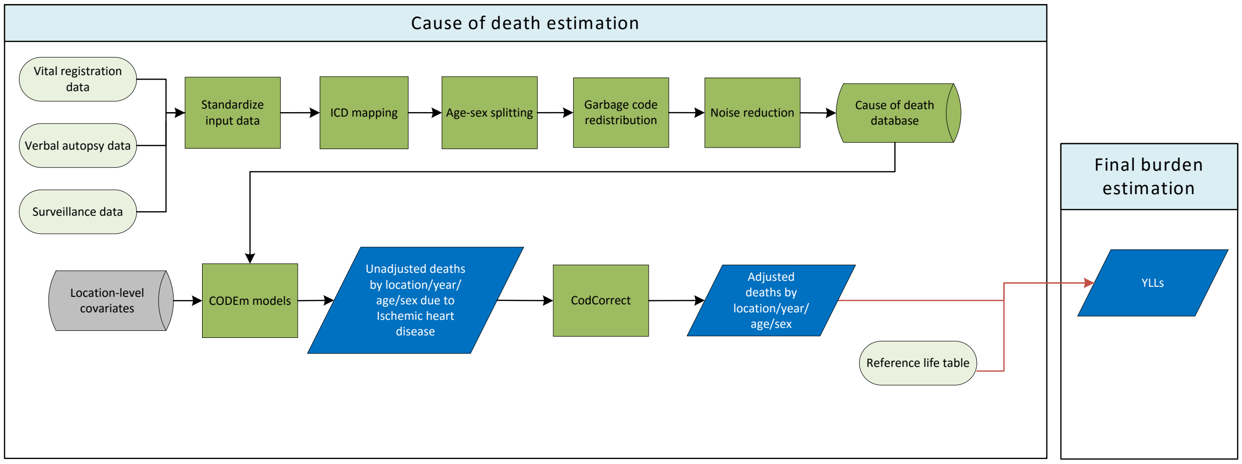

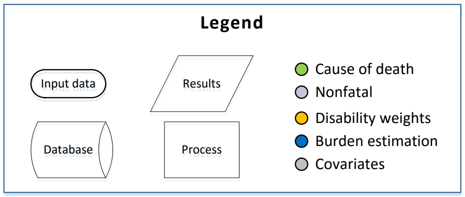

Input data
Vital registration, verbal autopsy, and surveillance data were used to model ischemic heart disease. We outliered verbal autopsy data in countries and subnational locations where high-quality vital registration data were also available. We also outliered non-representative subnational verbal autopsy data points, ICD8 and ICD9 BTL data points which were inconsistent with the rest of the data and created implausible time trends, and data in a number of Indian states identified by experts as poor-quality.

Modelling strategy
We used a standard CODEm approach to model deaths from ischemic heart disease. We have updated the covariates included in the ensemble modelling process (see Table). Otherwise, there have been no substantive changes from the approach used in GBD 2015.

Table: Selected covariates for CODEm models, ischemic heart disease

Covariate	Transformation	Level	Direction
Summary exposure variable	None	1	1
Cholesterol (total, mean per capita)	None	1	1
Smoking prevalence	None	1	1
Systolic blood pressure (mmHg)	None	1	1
Trans fatty acid	None	1	1
Mean BMI	None	2	1
Elevation over 1500m (proportion)	None	2	-1
Fasting plasma glucose	None	2	1
Outdoor pollution ($PM_{2.5}$)	None	2	1
Indoor air pollution	None	2	1
Healthcare access and quality index	None	2	-1
Lag distributed income per capita (I$)	Log	3	-1
Socio-demographic Index	None	3	0
Omega-3 (kcal/capita, adjusted)	Log	3	-1
Fruits (kcal/capita, adjusted)	None	3	-1
Vegetables (kcal/capita, adjusted)	None	3	-1
Nuts and seeds (kcal/capita, adjusted)	None	3	-1
Whole grains (kcal/capita, adjusted)	None	3	-1
Pulses/legumes (kcal/capita, adjusted)	None	3	-1
PUFA adjusted (percent)	None	3	-1
Alcohol (litres per capita)	None	3	0

Cerebrovascular Disease

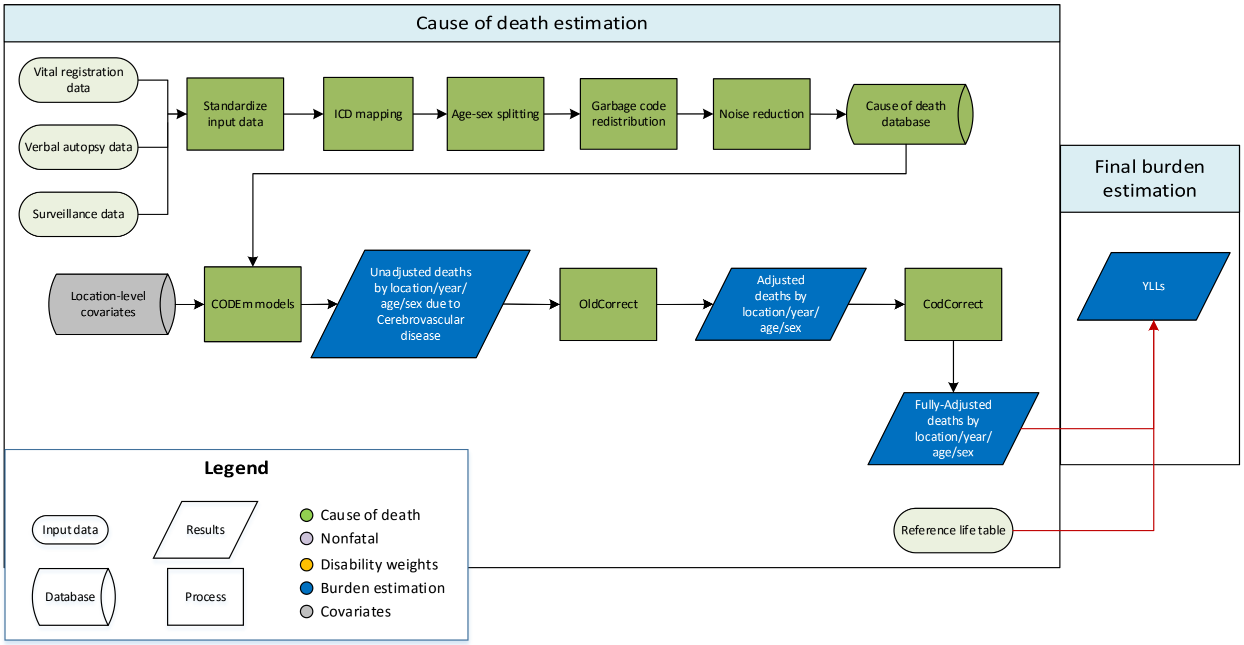

Input data

Verbal autopsy and vital registration data were used to model cerebrovascular disease. We outliered non-representative subnational verbal autopsy data points. We reassigned deaths from verbal autopsy reports for cerebrovascular disease to the parent cardiovascular disease for both sexes for those under 20 years of age. We also outliered ICD8, ICD9 BTL, and ICD10 Tabulated data points which were inconsistent with the rest of the data and created implausible time trends. Data points from sources which were implausibly low in all age groups and data points that were causing the regional estimates to be improbably high were outliered.

Modelling strategy

We used a standard CODEm approach to model deaths from cerebrovascular disease. The most significant update to the cerebrovascular method was the addition of a correction for miscoding of Alzheimer and other dementias and Parkinson disease to the post-CODEm adjustments to generate corrected cause-specific death estimates for final burden estimation. We have also updated the covariates included in the ensemble modelling process (see Table). Otherwise, there have been no substantive changes from the approach used in GBD 2015.

Table: Selected covariates for CODEm models, cerebrovascular disease

Covariate	Transformation	Level	Direction
Summary exposure variable	None	1	1
Cholesterol (total, mean per capita)	None	1	1
Smoking prevalence	None	1	1
Systolic blood pressure (mmHg)	None	1	1
Trans fatty acid	None	1	1
Mean BMI	None	2	1
Elevation over 1500m (proportion)	None	2	-1
Fasting plasma glucose	None	2	1
Outdoor pollution ($PM_{2.5}$)	None	2	1
Indoor air pollution	None	2	1
Healthcare access and quality index	None	2	-1
Lag distributed income per capita (I$)	Log	3	-1
Socio-demographic Index	None	3	0
Omega-3 (kcal/capita, adjusted)	Log	3	-1
Fruits (kcal/capita, adjusted)	None	3	-1
Vegetables (kcal/capita, adjusted)	None	3	-1
Nuts and seeds (kcal/capita, adjusted)	None	3	-1
Whole grains (kcal/capita, adjusted)	None	3	-1
Pulses/legumes (kcal/capita, adjusted)	None	3	-1
PUFA adjusted (percent)	None	3	-1
Alcohol (litres per capita)	None	3	0

Ischemic Stroke

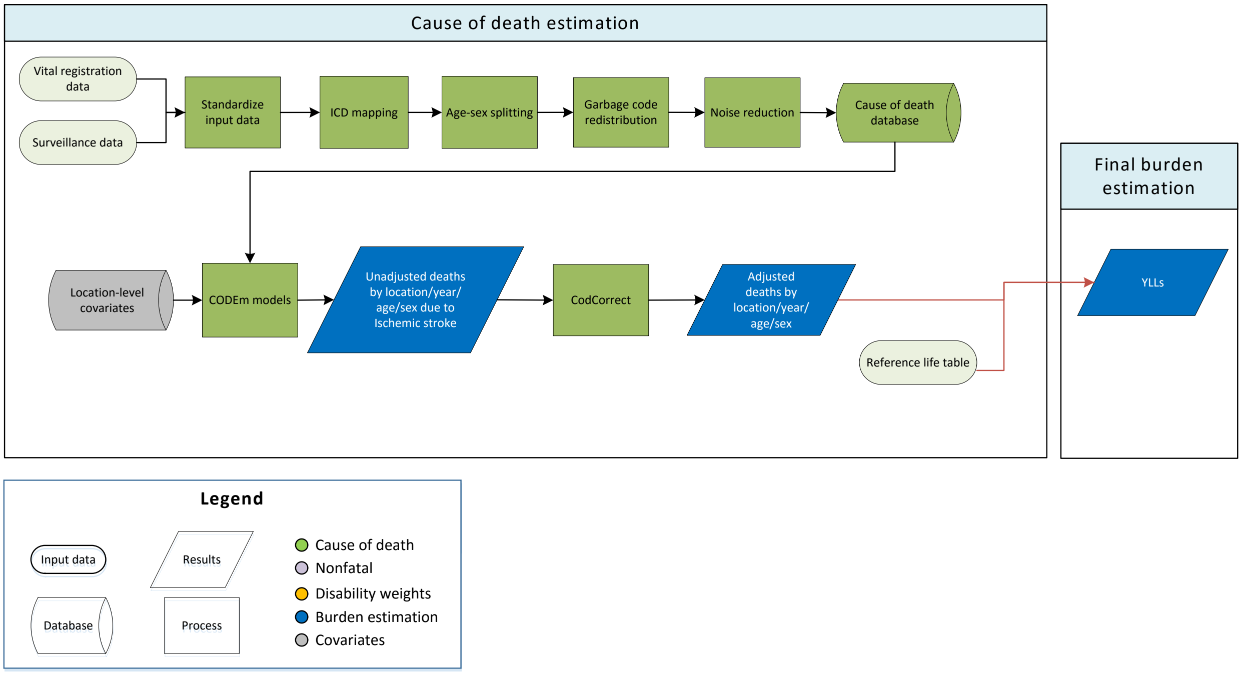

Input data

Vital registration and surveillance data were used to model ischemic stroke. We reassigned deaths from verbal autopsy reports for ischemic stroke to the parent cardiovascular disease for both sexes for those under 20 years of age. We outliered ICD8 data points which were inconsistent with the rest of the data and created implausible time trends.

Modelling strategy

We used a standard CODEm approach to model deaths from ischemic stroke. In locations with limited data on ischemic stroke, the subtype-specific deaths were estimated by squeezing both ischemic and hemorrhagic stroke to the overall cerebrovascular envelope. We have updated the covariates included in the ensemble modelling process (see Table). Otherwise, there have been no substantive changes from the approach used in GBD 2015.

Table: Selected covariates for CODEm models, ischemic stroke

Covariate	Transformation	Level	Direction
Summary exposure variable	None	1	1
Cholesterol (total, mean per capita)	None	1	1
Smoking prevalence	None	1	1
Systolic blood pressure (mmHg)	None	1	1
Trans fatty acid	None	1	1
Mean BMI	None	2	1
Elevation over 1500m (proportion)	None	2	-1
Fasting plasma glucose	None	2	1
Outdoor pollution ($PM_{2.5}$)	None	2	1
Indoor air pollution	None	2	1
Healthcare access and quality index	None	2	-1
Lag distributed income per capita (I$)	Log	3	-1
Socio-demographic Index	None	3	0
Omega-3 (kcal/capita, adjusted)	Log	3	-1
Fruits (kcal/capita, adjusted)	None	3	-1
Vegetables (kcal/capita, adjusted)	None	3	-1
Nuts and seeds (kcal/capita, adjusted)	None	3	-1
Whole grains (kcal/capita, adjusted)	None	3	-1
Pulses/legumes (kcal/capita, adjusted)	None	3	-1
PUFA adjusted (percent)	None	3	-1
Alcohol (litres per capita)	None	3	0

Hemorrhagic Stroke

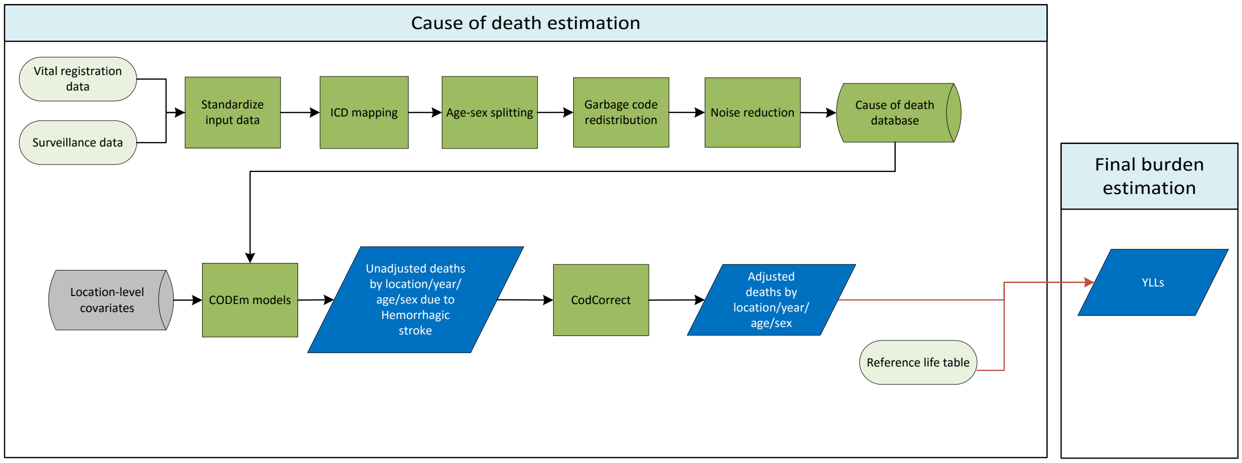

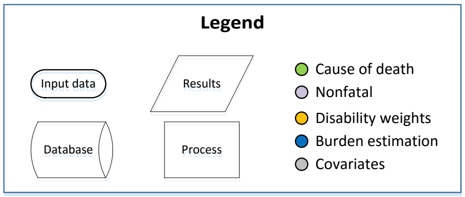

Input data

Vital registration and surveillance data were used to model hemorrhagic stroke. We reassigned deaths from verbal autopsy reports for hemorrhagic stroke to the parent cardiovascular disease for both sexes for those under 20 years of age. We outliered ICD8 data points which were inconsistent with the rest of the data and created implausible time trends.

Modelling strategy

We used a standard CODEm approach to model deaths from hemorrhagic stroke. In locations with limited data on hemorrhagic stroke, the subtype-specific deaths were estimated by squeezing both ischemic and hemorrhagic stroke to the overall cerebrovascular envelope. We have updated the covariates included in the ensemble modelling process (see Table). Otherwise, there have been no substantive changes from the approach used in GBD 2015.

Table: Selected covariates for CODEm models, hemorrhagic stroke

Covariate	Transformation	Level	Direction
Summary exposure variable	None	1	1
Cholesterol (total, mean per capita)	None	1	0
Smoking prevalence	None	1	1
Systolic blood pressure (mmHg)	None	1	1
Trans fatty acid	None	1	1
Mean BMI	None	2	1
Elevation over 1500m (proportion)	None	2	-1
Fasting plasma glucose	None	2	1
Outdoor pollution ($PM_{2.5}$)	None	2	1
Indoor air pollution	None	2	1
Healthcare access and quality index	None	2	-1
Lag distributed income per capita (I$)	Log	3	-1
Socio-demographic index	None	3	0
Omega-3 (kcal/capita, adjusted)	Log	3	-1
Fruits (kcal/capita, adjusted)	None	3	-1
Vegetables (kcal/capita, adjusted)	None	3	-1
Nuts and seeds (kcal/capita, adjusted)	None	3	-1
Whole grains (kcal/capita, adjusted)	None	3	-1
Pulses/legumes (kcal/capita, adjusted)	None	3	-1
PUFA adjusted (percent)	None	3	-1
Alcohol (litres per capita)	None	3	0

Hypertensive Heart Disease

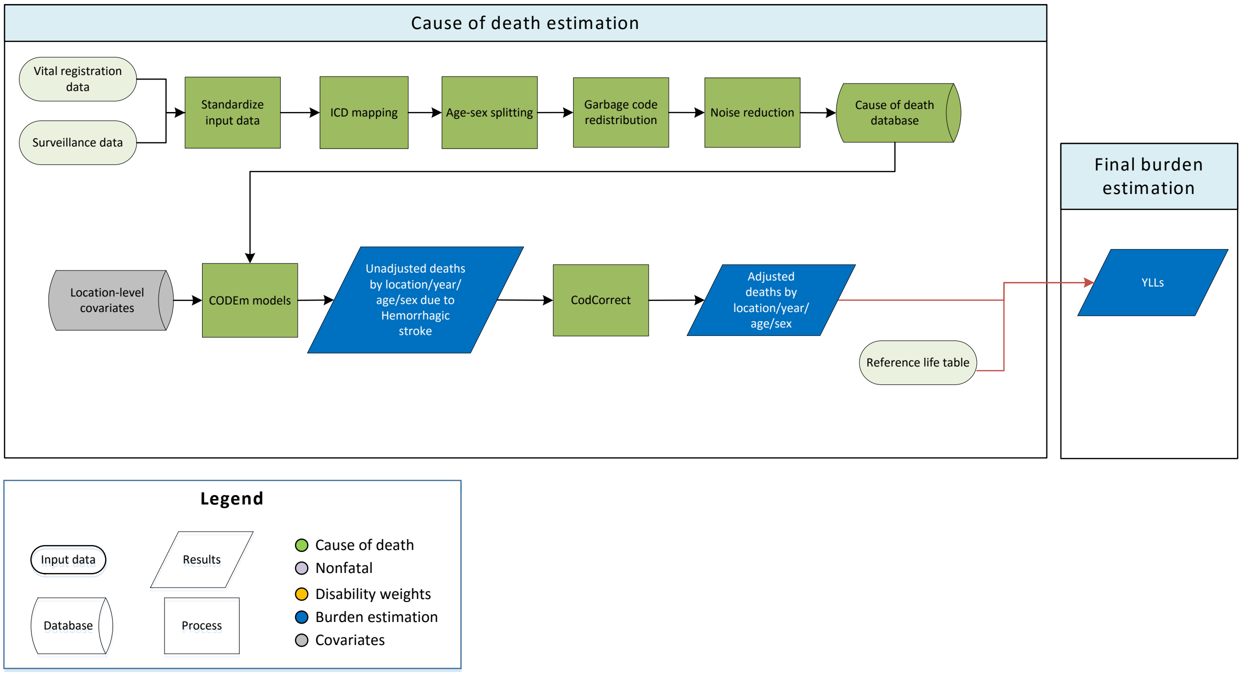

Input data
Vital registration and surveillance data were used to model hypertensive heart disease. We outliered ICD9 BTL data points, which were inconsistent with the rest of the data and created implausible time trends.

Modelling strategy
We used a standard CODEm approach to model deaths from cardiovascular diseases. We have updated the covariates included in the ensemble modelling process (see Table). Otherwise, there have been no substantive changes from the approach used in GBD 2015.

Table: Selected covariates for CODEm models, hypertensive heart disease

Covariate	Transformation	Level	Direction
Cholesterol (total, mean per capita)	None	1	1
Smoking prevalence	None	1	1
Systolic blood pressure (mmHg)	None	1	1
Mean BMI	None	2	1
Healthcare access and quality index	None	2	-1
Lag distributed income per capita (I$)	Log	3	-1
Socio-demographic index	None	3	0

Myocarditis

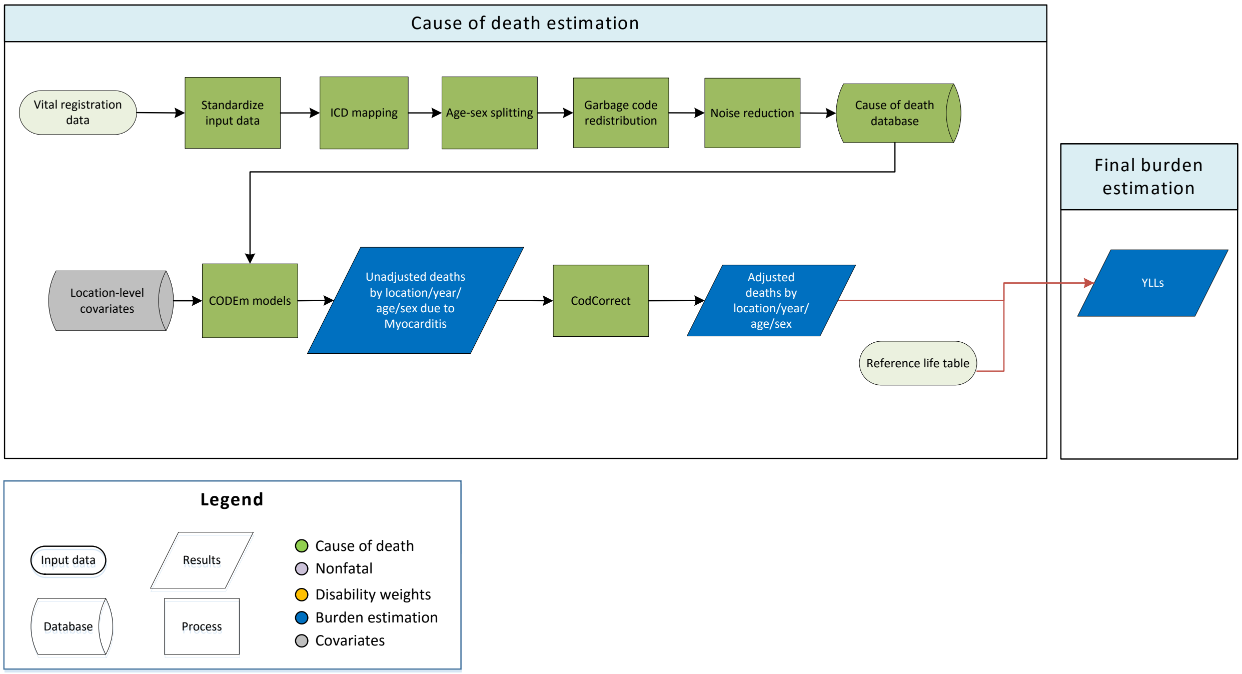

Input data

Vital registration data were used to model deaths due to myocarditis.

Modelling strategy

We used a standard CODEm approach to model deaths from myocarditis. This is one of three new sub-causes under the cardiomyopathy and myocarditis parent cause for GBD 2016. The covariates selected for inclusion in the CODEm modelling process can be found in the table below.

Table: Selected covariates for CODEm models, myocarditis

Covariate	Transformation	Level	Direction
Summary exposure variable, CMP	none	1	1
Systolic blood pressure (mm Hg)	none	1	1
Healthcare access and quality index	none	2	-1
Lag distributed income per capita (I$)	log	3	0
Socio-demographic Index	none	3	0

Alcoholic Cardiomyopathy

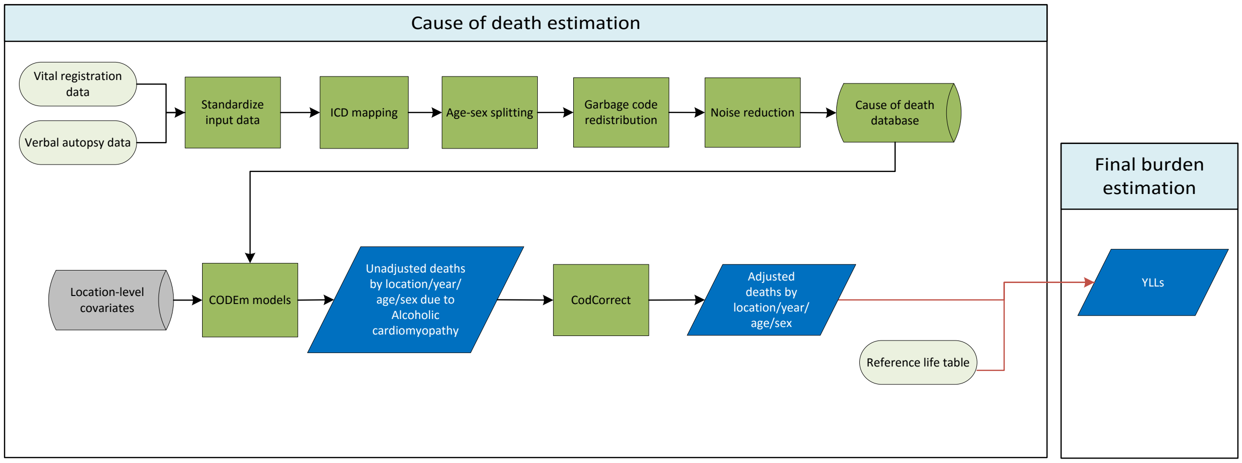

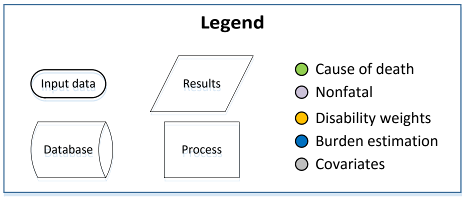

Input data

Vital registration and verbal autopsy data were used to model deaths due to alcoholic cardiomyopathy. We outliered ICD9 data points in Cyprus that were implausibly high and discontinuous with the rest of the time series.

Modelling strategy

We used a standard CODEm approach to model deaths from alcoholic cardiomyopathy. This is one of three new sub-causes under the cardiomyopathy and myocarditis parent cause for GBD 2016. The covariates selected for inclusion in the CODEm modelling process can be found in the table below. As local differences in coding practices may explain some of the geographic variation that we see for deaths due to cardiomyopathy and myocarditis, we plan to explore how this issue may affect the alcoholic cardiomyopathy sub-cause further in future iterations of GBD.

Table: Selected covariates for CODEm models, alcoholic cardiomyopathy

Covariate	Transformation	Level	Direction
Summary exposure variable, CMP	none	1	1
Smoking prevalence	none	1	1
Alcohol (litres per capita)	none	1	1
Healthcare access and quality index	none	2	-1
Lag distributed income per capita (I$)	log	3	0
Socio-demographic Index	none	3	0

Other cardiomyopathy

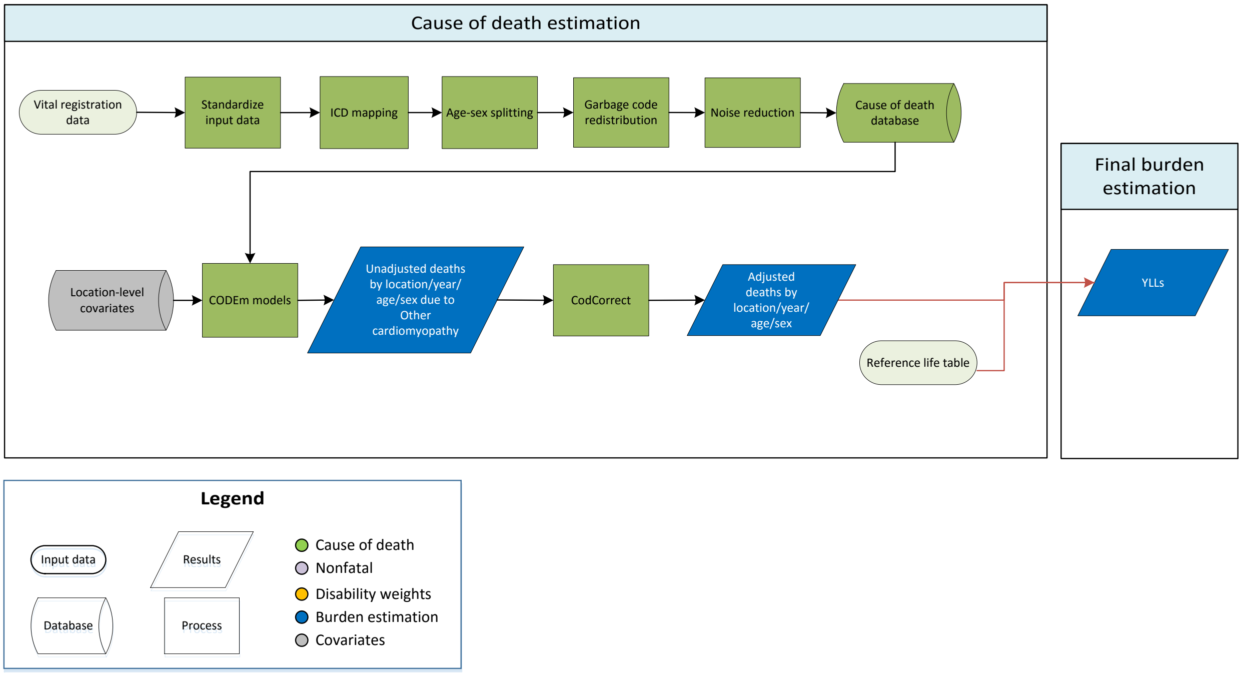

Input data

Vital registration data were used to model deaths due to other cardiomyopathy. We outliered data points in Central Asia and Central and Eastern Europe due to implausibly high values which we attributed to variation in local coding practices after review with experts.

Modelling strategy

We used a standard CODEm approach to model deaths from other cardiomyopathy. This is one of three new sub-causes under the cardiomyopathy and myocarditis parent cause for GBD 2016. The covariates selected for inclusion in the CODEm modelling process can be found in the table below. As local differences in coding practices may explain some of the geographic variation that we see for deaths due to cardiomyopathy and myocarditis, we plan to explore how this issue may affect the other cardiomyopathy sub-cause further in future iterations of GBD.

Table: Selected covariates for CODEm models, other cardiomyopathy

Covariate	Transformation	Level	Direction
Summary exposure variable, CMP	none	1	1
Systolic blood pressure (mmHg)	none	1	1
Smoking prevalence	none	1	1
Body mass index (kg/m^2)	none	2	1
Healthcare access and quality index	none	2	-1
Lag distributed income per capita (I$)	log	3	0
Socio-demographic Index	none	3	0

Atrial Fibrillation and Flutter

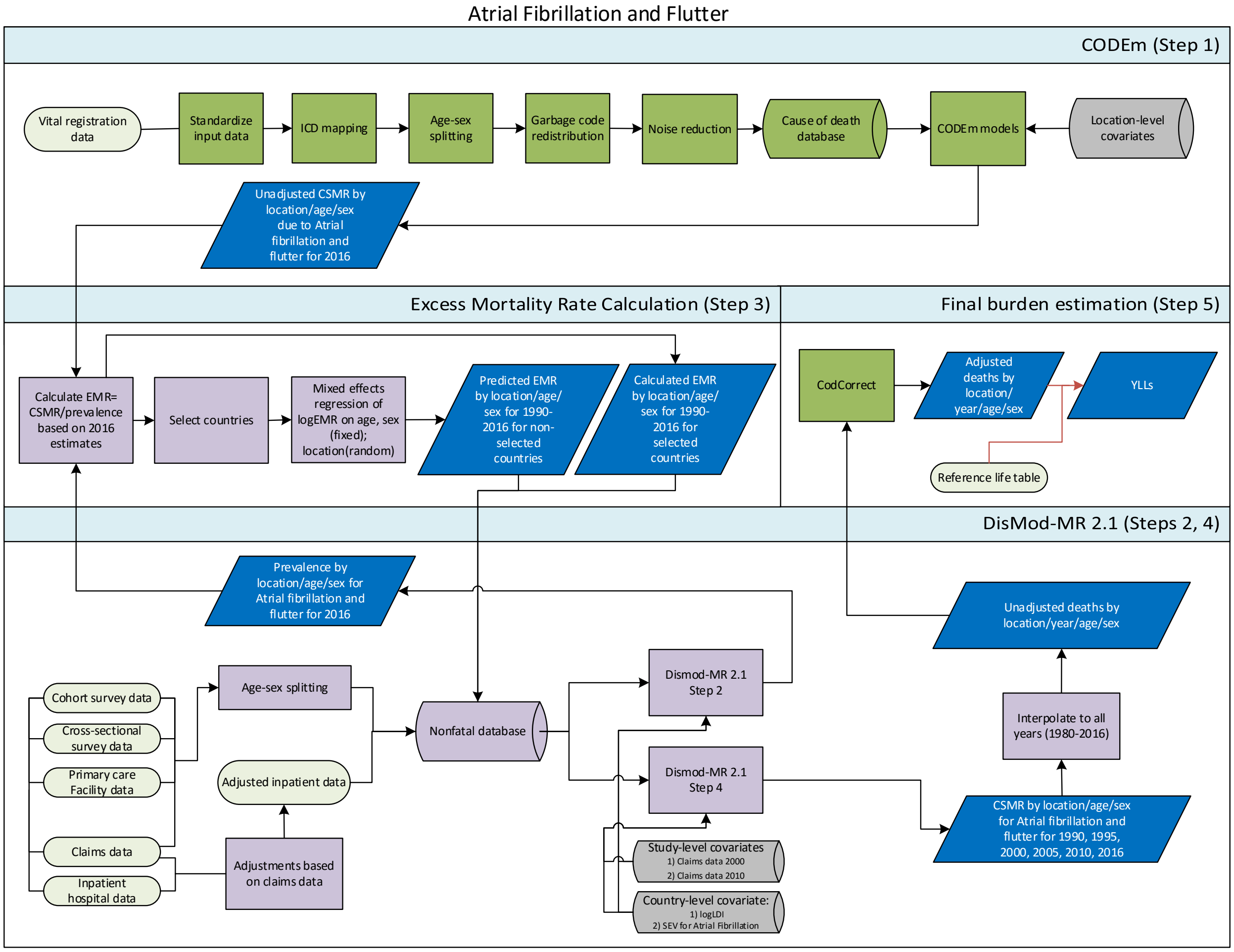

Input data

Vital registration data: We outliered ICD8 and ICD9 data points that were discontinuous from other data in the time series and created an unlikely time trend. We also outliered data points that were implausibly low in multiple age groups.

Modelling strategy

In order to address changes in coding practices for atrial fibrillation, we used an integrated approach that combined DisMod-MR and CODEm models to estimate deaths from atrial fibrillation and flutter. This approach allowed us to adjust estimates to more accurately reflect the number of deaths for which atrial fibrillation was the true underlying cause of death.

The modelling steps are illustrated in the above flowchart. Covariates included in both the DisMod-MR 2.1 and CODEm models can be found in the table below. In Step 1, we estimated deaths for atrial fibrillation using a standard CODEm approach. In Step 2, we estimated prevalence rates in DisMod-MR 2.1 using data from published reports of cross-sectional and cohort surveys, as well as primary care facility data. We also used claims data covering inpatient and outpatient visits for the United States

along with inpatient hospital data from 163 locations in 15 countries. For GBD 2016, inpatient hospital data were adjusted using age- and sex-specific information from US claims data for: 1) readmission within one year; 2) primary diagnosis code to secondary codes; and, 3) the ratio of inpatient to outpatient visits. We set priors of no remission and no excess mortality prior to age 30.

In Step 3, we calculated the excess mortality rate (EMR) for 2016 (defined as the cause-specific mortality rate (CSMR) estimated from CODEm divided by the prevalence rate from DisMod-MR 2.1). We then selected 17 countries based on four conditions: 1) ranking of 4 or 5 stars on the newly developed system for assessing the quality of VR data; 2) prevalence data available from the literature was included in the DisMod-MR 2.1 estimation; 3) prevalence rate ≥ 0.005; and, 4) CSMR ≥ 0.00002. Using information from these countries as input data, we ran a linear mixed-effects regression of logEMR on sex, age, and location. Sex and age were treated as fixed effects for the regression, while location was considered a random effect. We then predicted age- and sex-specific EMR using the results of this regression for all non-selected countries. Countries included in the regression were assigned their directly calculated values. These EMR data points were assigned to the time period 1990–2016 and uploaded into the nonfatal database in order to be used in modelling.

In Step 4, we reran DisMod-MR 2.1 including the EMR estimated in Step 3 as input data using the same priors as in Step 2. The CSMR from the DisMod-MR model in Step 4 was used as the finalized output. As DisMod-MR 2.1 only generates estimates for six years (1990, 1995, 2000, 2005, 2010, 2016), we interpolated the missing years to generate death estimates for all years (1980–2016). These results were then uploaded into the Cause of Death database. Finally, in Step 5, the unadjusted death estimates were run through the CoDCorrect process to generate adjusted deaths, and YLLs were generated by the DALYnator using a standard reference life table.

CODEm Covariates

Covariate	Transformation	Level	Direction
Summary exposure variable	None	1	1
Cholesterol (total, mean per capita)	None	1	1
Smoking prevalence	None	1	1
Systolic blood pressure (mmHg)	None	1	1
Mean BMI	None	2	1
Elevation over 1500m (proportion)	None	2	-1
Fasting plasma glucose	None	2	1
Outdoor pollution ($PM_{2.5}$)	None	2	1
Indoor air pollution	None	2	1
Healthcare Access and Quality Index	None	2	-1
Lag distributed income per capita (I$)	Log	3	-1
Socio-demographic Index	None	3	0
Omega-3 (kcal/capita, adjusted)	Log	3	-1
Fruits (kcal/capita, adjusted)	None	3	-1
Vegetables (kcal/capita, adjusted)	None	3	-1
Nuts and seeds (kcal/capita, adjusted)	None	3	-1
Whole grains (kcal/capita, adjusted)	None	3	-1
Pulses/legumes (kcal/capita, adjusted)	None	3	-1
PUFA adjusted (percent)	None	3	-1
Alcohol (litres per capita)	None	3	0
Trans fatty acid	None	1	1

DisMod Covariates – Step 2

Study covariate	Parameter	Beta	Exponentiated beta
Hospital data	Prevalence	-0.000086 (-0.19 – 0.097)	1.0 (0.82 – 1.10)
All MarketScan, year 2000	Prevalence	-0.47 (-0.5 – -0.44)	0.63 (0.61 – 0.64)
All MarketScan, year 2010	Prevalence	-0.003 (-0.024 – -0.014)	1.0 (0.98 – 1.01)
Log-transformed age-standardized SEV scalar: A Fib	Prevalence	0.75 (0.75 – 0.75)	2.12 (2.12 – 2.12)
LDI (I$ per capita)	Excess mortality rate	-0.48 (-0.5 – -0.43)	0.62 (0.61 – 0.65)

DisMod Covariates – Step 4

Study covariate	Parameter	Beta	Exponentiated beta
All MarketScan, year 2000	Prevalence	-0.46 (-0.49 – -0.43)	0.63 (0.62 – 0.65)
All MarketScan, year 2010	Prevalence	-0.0021 (-0.025 – -0.021)	1.0 (0.98 – 1.02)
Log-transformed age-standardized SEV scalar: A Fib	Prevalence	0.75 (0.75 – 0.75)	2.12 (2.12 – 2.12)
LDI (I$ per capita)	Excess mortality rate	-0.1 (-0.1 – -0.1)	0.9 (0.9 – 0.9)

Aortic Aneurysm

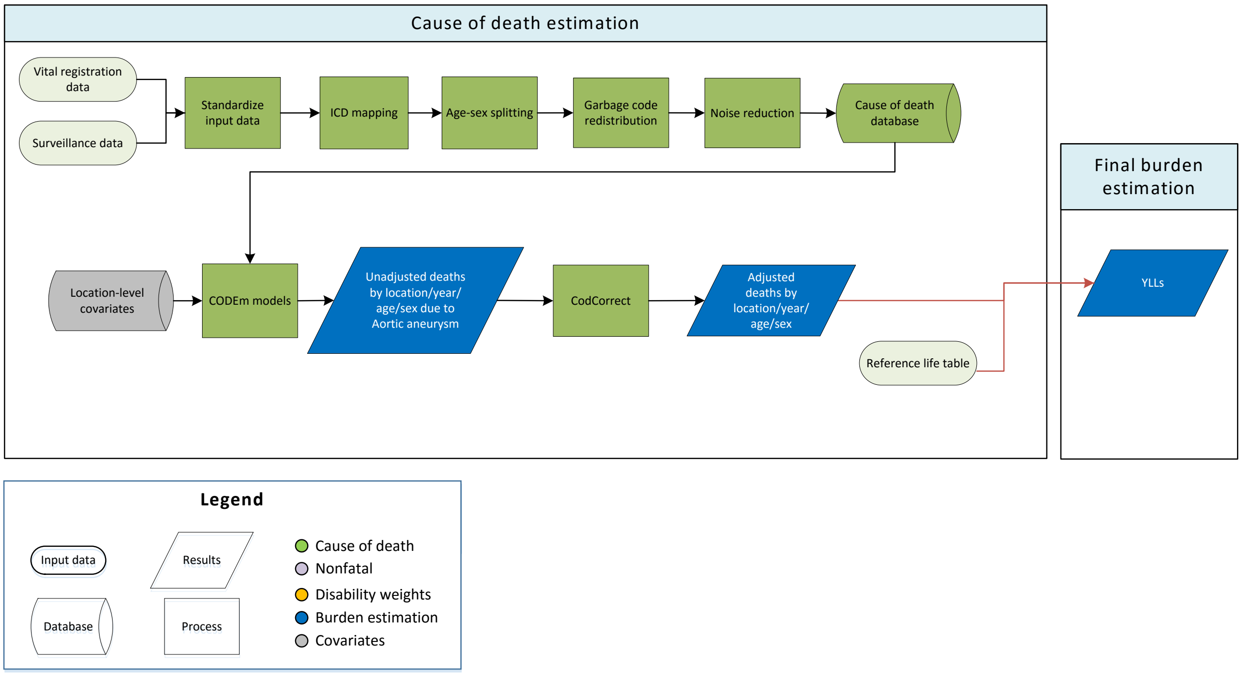

Input data

Vital registration and surveillance data were used to model this cause. We outliered data in Oman as they were improbably high in comparison with the rest of the region. We also outliered ICD8 data that were discontinuous with the rest of the time series and created implausible time trends.

Modelling strategy

We used a standard CODEm approach to model deaths from cardiovascular diseases. We have updated the covariates included in the ensemble modelling process (see Table). Otherwise, there have been no substantive changes from the approach used in GBD 2015.

Table: Selected covariates for CODEm models, cardiovascular diseases

Covariate	Transformation	Level	Direction
Summary exposure variable	None	1	1
Cholesterol (total, mean per capita)	None	1	1
Cumulative cigarettes (10 yrs)	None	1	1
Systolic blood pressure (mmHg)	None	1	1
Trans fatty acid (percent)	None	1	1
Mean BMI	None	2	1
Healthcare access and quality index	None	2	-1
Lag distributed income per capita (I$)	Log	3	-1
Socio-demographic Index	None	3	0
Omega-3 (kcal/capita, adjusted)	Log	3	-1
Fruits (kcal/capita, adjusted)	None	3	-1
Vegetables (kcal/capita, adjusted)	None	3	-1
Nuts and seeds (kcal/capita, adjusted)	None	3	-1
Whole grains (kcal/capita, adjusted)	None	3	-1
Pulses/legumes (kcal/capita, adjusted)	None	3	-1
PUFA adjusted (percent)	None	3	-1
Alcohol (litres per capita)	None	3	0

Peripheral Artery Disease

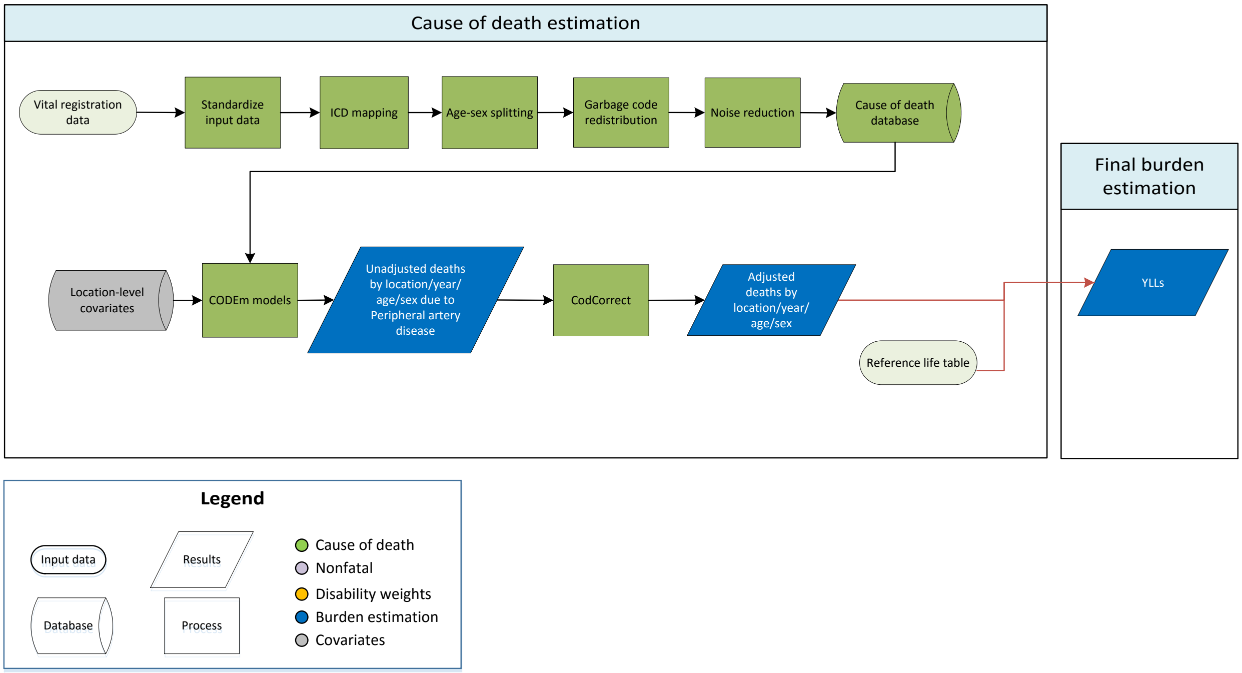

Input data

Vital registration data were used to model peripheral artery disease. We outliered all data points with <1 death in Egypt per expert review.

Modelling strategy

We used a standard CODEm approach to model deaths from peripheral artery disease. We have updated the covariates included in the ensemble modelling process (see Table). Otherwise, there have been no substantive changes from the approach used in GBD 2015.

Table: Selected covariates for CODEm models, peripheral artery disease

Covariate	Transformation	Level	Direction
Summary exposure variable	None	1	1
Systolic blood pressure (mmHg)	None	1	1
Cholesterol (total, mean per capita)	None	1	1
Smoking prevalence	None	1	1
Mean body mass index (kg/m^2)	None	2	1
Healthcare access and quality index	None	2	-1
Lag distributed income per capita (I$)	Log	3	-1
Socio-demographic Index	None	3	0
Omega-3 (kcal/capita, adjusted)	Log	3	-1
Fruits (kcal/capita, adjusted)	None	3	-1
Vegetables (kcal/capita, adjusted)	None	3	-1
Nuts and seeds (kcal/capita, adjusted)	None	3	-1
Whole grains (kcal/capita, adjusted)	None	3	-1
Pulses/legumes (kcal/capita, adjusted)	None	3	-1
PUFA adjusted (percent)	None	3	-1
Trans fatty acid (percent)	None	3	1
Alcohol (litres per capita)	None	3	0

Endocarditis

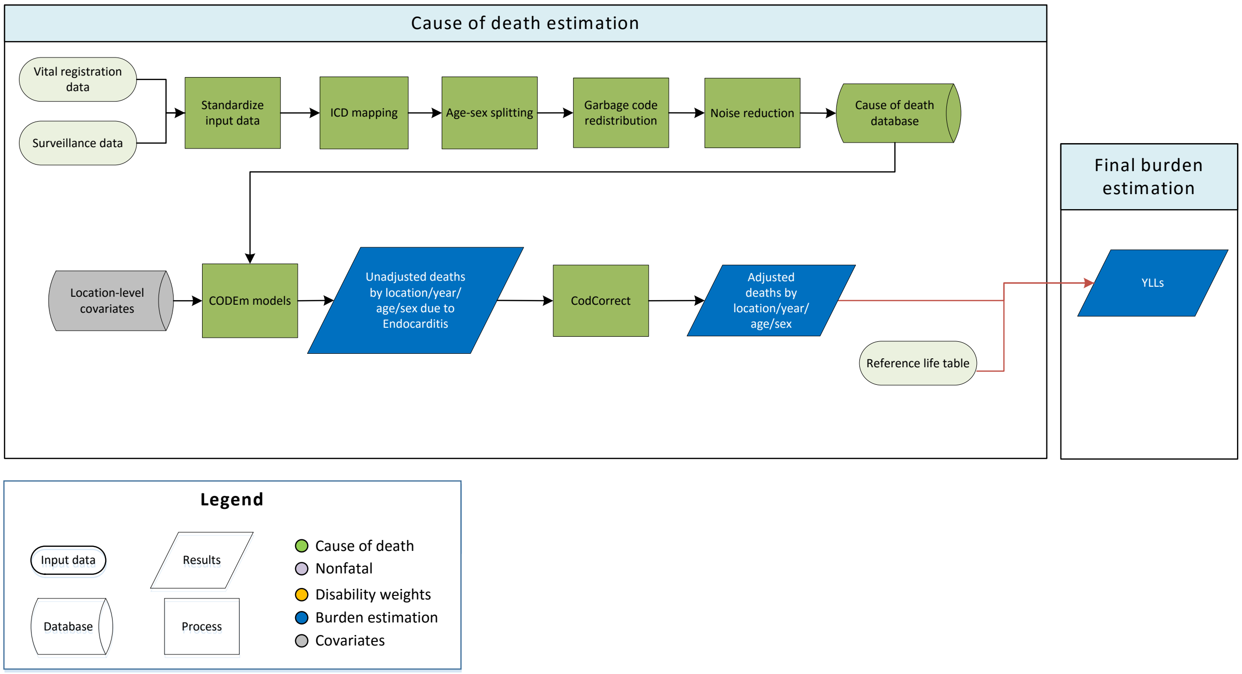

Input data

Vital registration and surveillance data were used to model endocarditis. We outliered vital registration data in Mozambique as these were non-representative for sub-Saharan Africa and were causing regional estimates to be implausibly low. We also outliered ICD8 data that were discontinuous from the rest of the data series and created an implausible time trend.

Modelling strategy

We used a standard CODEm approach to model deaths from endocarditis. We have updated the covariates included in the ensemble modelling process (see Table). Otherwise, there have been no substantive changes from the approach used in GBD 2015.

Table: Selected covariates for CODEm models, endocarditis

Covariate	Transformation	Level	Direction
Summary exposure variable	None	1	1
Improved water (proportion)	None	1	-1
Sanitation (proportion with access)	None	1	-1
Healthcare access and quality index	None	1	-1
Lag distributed income per capita (I$)	Log	3	-1
Socio-demographic Index	None	3	0

Other Cardiovascular and Circulatory Diseases

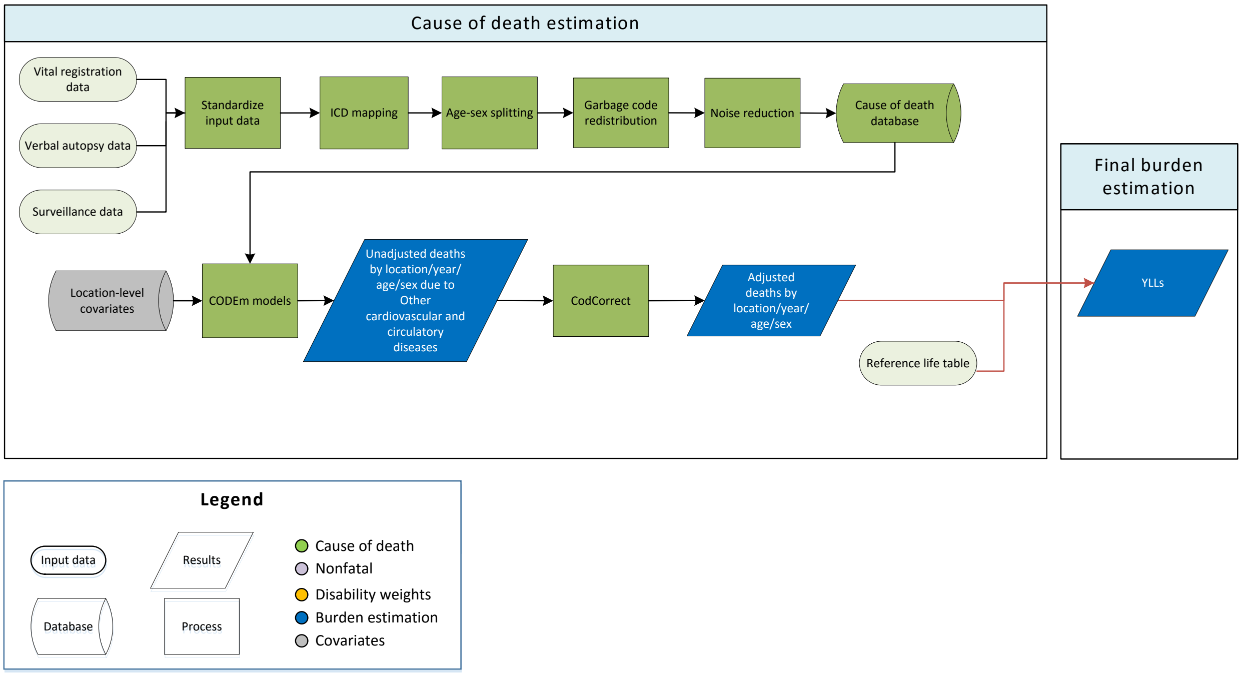

Input data

Vital registration, verbal autopsy, and surveillance data were used to model other cardiovascular and circulatory diseases. We outliered ICD8 and ICD9 BTL data points that were inconsistent with the rest of the data and created implausible time trends. We also outliered ICD8 data points which were not nationally representative.

Modelling strategy

We used a standard CODEm approach to model deaths from other circulatory and cardiovascular diseases. We have updated the covariates included in the ensemble modelling process (see Table). Otherwise, there have been no substantive changes from the approach used in GBD 2015.

Table: Selected covariates for CODEm models, cardiovascular diseases

Covariate	Transformation	Level	Direction
Summary exposure variable	None	1	1
Cholesterol (total, mean per capita)	None	1	1
Smoking prevalence	None	1	1
Systolic blood pressure (mmHg)	None	1	1
Trans fatty acid (percent)	None	1	1
Mean BMI	None	2	1
Elevation over 1500m (proportion)	None	2	-1
Fasting plasma glucose (mmol/L)	None	2	1
Indoor air pollution (all fuel types)	None	2	1
Outdoor air pollution ($PM_{2.5}$)	None	2	1
Healthcare access and quality index	None	2	-1
Lag distributed income per capita (I$)	Log	3	-1
Socio-demographic Index	None	3	0
Omega-3 (kcal/capita, adjusted)	Log	3	-1
Fruits (kcal/capita, adjusted)	None	3	-1
Vegetables (kcal/capita, adjusted)	None	3	-1
Nuts and seeds (kcal/capita, adjusted)	None	3	-1
Whole grains (kcal/capita, adjusted)	None	3	-1
Pulses/legumes (kcal/capita, adjusted)	None	3	-1
PUFA adjusted (percent)	None	3	-1
Alcohol (litres per capita)	None	3	0

3.4.1 Cancers SDG Capstone Appendix

Input data and methodological summary for all cancers except for non-melanoma skin cancer

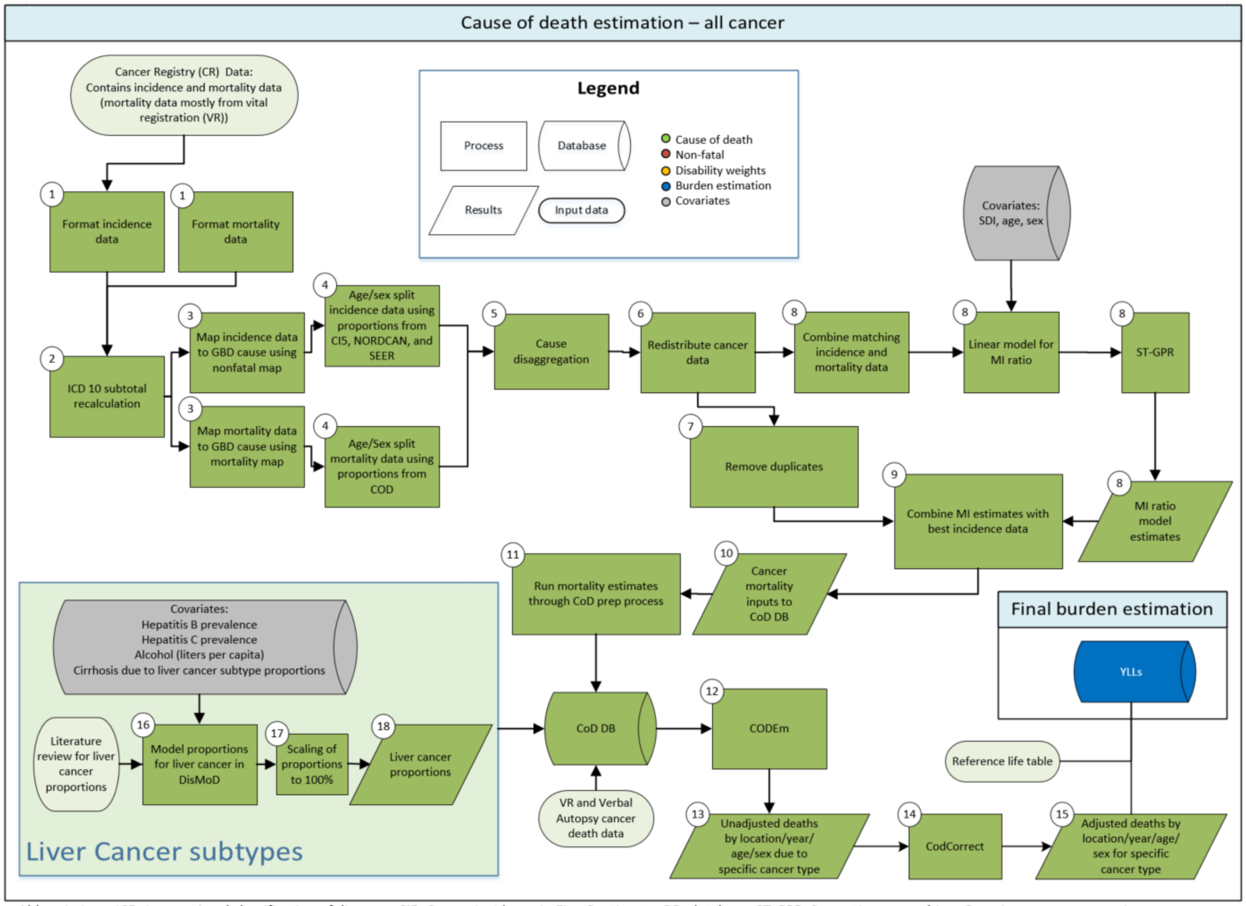

Abbreviations: ICD: International classification of diseases; CI5: Cancer Incidence in Five Continents, DB: database, ST-GPR: Space-time smoothing, Gaussian process regression

Data

The cause of death (COD) database contains multiple sources of cancer mortality data. These sources include vital registration, verbal autopsy, and cancer registry data. The cancer registry mortality estimates that are uploaded into the COD database stem from cancer registry incidence data that have been transformed to mortality estimates through the use of mortality-to-incidence ratios (MIR).

Data seeking processes

Cancer mortality data in the cause of death database other than cancer registry data

Sources for cancer mortality data other than cancer registry data are described in the COD database description (Section 2).

Cancer registry data

Cancer registry data were used from publicly available sources or provided by collaborators. We attempted to collect data from all registries that are members of the International Association of Cancer Registries (IACR) by either downloading publicly available data or contacting the registries. We also used cancer registry databases like Cancer Incidence in Five Continents (CI5), EUREG, and NORDCAN.[1–9]

Most cancer registries only report cancer incidence. However, if a cancer registry also reported cancer mortality, mortality data were also extracted from the source to be used in the MIR estimation.

Inclusion and exclusion criteria

Only population-based cancer registries were included, and only those that included all cancers (no specialty registries), data for all age groups, and data for both sexes. Pathology-based cancer registries were included if they had a defined population. Hospital-based cancer registries were excluded.
Cancer registry data were excluded from either the final incidence data input or the MI model input if a more detailed source (e.g., providing more detailed age or diagnostic groups) was available for the same population. Preference was given to registries with national coverage over those with only local coverage, except those from countries where the GBD study provides subnational estimates.
Data were excluded if the coverage population was unknown.

Bias of categories of input data

Cancer registry data can be biased in multiple ways. A high proportion of ill-defined cancer cases in the registry data requires redistribution of these cases to other cancers, which introduces a potential for bias. Changes between coding systems can lead to artificial differences in disease estimates; however, we adjust for this bias by mapping the different coding systems to the GBD causes. Underreporting of cancers that require advanced diagnostic techniques (e.g., leukemia and brain, pancreatic, and liver cancer) can be an issue in cancer registries from low-income countries. On the other hand, misclassification of metastatic sites as primary cancer can lead to overestimation of cancer sites that are common sites for metastases, like brain or liver. Since many cancer registries are located in urban areas, the representativeness of the registry for the general population can also be problematic. The accuracy of mortality data reported in cancer registries usually depends on the quality of the vital registration system. If the vital registration system is incomplete or of poor quality, the mortality-to-incidence ratio can be biased to lower ratios.

Data for liver cancer etiology splits

To find the proportion of liver cancer cases due to the four etiology groups included in GBD (1. Liver cancer due to hepatitis B, 2. Liver cancer due to hepatitis C, 3. Liver cancer due to alcohol, 4. Liver cancer due to other causes), a systematic literature search was performed in PubMed. Studies were included if the study population was representative of liver cancer population for the respective location. For each study the proportions of liver cancer due to the three specific risk factors were calculated. Remaining risk factors were included under a combined “other” group. Cryptogenic cases were only included if other etiologies like viral hepatitis or alcoholic cirrhosis had been excluded. If multiple risk factors were reported for an individual patient these were apportioned proportionally to the individual risk factors.

Methods

Steps of analysis and data transformation processes

Cancer registry data went through multiple processing steps before integration with the COD database. First, the original data were transformed into standardized files, which included standardization of format, categorization, and registry names (#1 in flowchart).

Second, some cancer registries report individual codes as well as aggregated totals (e.g., C18, C19, and C20 are reported individually but the aggregated group of C18-C20 [colorectal cancer] is also reported in

the registry data). The data processing step “subtotal recalculation” (#2 in flowchart) verifies these totals and subtracts the values of any individual codes from the aggregates.

In the third step (#3 in the flowchart), cancer registry incidence data and cancer registry mortality data are mapped to GBD causes. A different map is used for incidence and for mortality data because of the assumption that there are no deaths for certain cancers. One example is basal cell carcinoma of the skin. In the cancer registry incidence data, basal cell carcinoma is mapped to non-melanoma skin cancer (basal cell carcinoma). However, if basal cell skin cancer is recorded in the cancer registry mortality data, the deaths are instead mapped to non-melanoma skin cancer (squamous cell carcinoma) under the assumption that they were indeed misclassified squamous cell skin cancers. Other examples are benign or in situ neoplasms. Benign or in situ neoplasms found in the cancer registry incidence dataset were simply dropped from that dataset. The same neoplasms reported in a cancer registry mortality dataset were mapped to the respective invasive cancer (e.g., melanoma in situ in the cancer registry incidence dataset was dropped from the dataset; melanoma in situ in the cancer registry mortality dataset was mapped to melanoma).

In the fourth data processing step (#4 in the flowchart) cancer registry data were standardized to the GBD age groups. Age-specific incidence rates were generated using CI5, SEER, and NORDCAN data, while age-specific mortality rates were generated from the CoD data through a method described in Part 2. Age-specific proportions were then generated by applying the age-specific rates to a given registry population that required age-splitting to produce the expected number of cases/deaths for that registry by age. The expected number of cases/deaths for each sex, age, and cancer were then normalized to 1, creating final, age-specific proportions. These proportions were then applied to the total number of cases/deaths by sex and cancer to get the age-specific number of cases/deaths.

In the rare case that the cancer registry only contained data for both sexes combined, the now-age-specific cases/deaths were split and re-assigned to separate sexes using the same weights that are used for the age-splitting process. Starting from the expected number of deaths, proportions were generated by sex for each age (e.g., if for ages 15 to 19 years old there are six expected deaths for males and four expected deaths for females, then 60% of the combined-sex deaths for ages 15-19 years would be assigned to males and the remaining 40% would be assigned to females).

In the fifth step (#5 in the flowchart) data for cause entries that are aggregates of GBD causes were redistributed. Examples of these aggregated causes include some registries reporting ICD10 codes C00-C14 together as, “lip, oral cavity, and pharyngeal cancer.” These groups were broken down into sub-causes that could be mapped to single GBD causes. In this example, those include lip and oral cavity cancer (C00-C08), nasopharyngeal cancer (C11), cancer of other parts of the pharynx (C09-C10, C12-C13), and “Malignant neoplasm of other and ill-defined sites in the lip, oral cavity, and pharynx” (C14). To redistribute the data, weights were created using the same “rate-applied-to-population” method employed in age-sex splitting (see step four above). For the undefined code (C14 in the example) an “average all cancer” weight was used, which was generated by adding all cases from SEER/NORDCAN/CI5 and dividing the total by the combined population. Then, proportions were generated by sub-cause for each aggregate cause as in the sex-splitting example above (see step four). The total number of cases from the aggregated group (C00-C14) was then recalculated for each

subgroup and the undefined code (C14). C14 was then redistributed as a “garbage code” in step six. Distinct proportions were used for C44 (non-melanoma skin cancer) and C46 (Kaposi’s sarcoma). Non-melanoma skin cancer processing is described under section “Input data and methodological summary for non-melanoma skin cancer (squamous-cell carcinoma).” C46 entries were redistributed as “other cancer,” HIV, and C80 (other and unknown cancers) using proportions described in Part 2.
In the sixth step (#6 in the flowchart) unspecified codes (“garbage codes”) were redistributed. Redistribution of cancer registry incidence and mortality data mirrored the process of the redistribution used in the cause of death database (Part 2).

In the seventh step (#7 in the flowchart) duplicate or redundant sources were removed from the processed cancer registry dataset. Duplicate sources were present if, for example, the cancer registry was part of the CI5 database but we also had data from the registry directly. Redundancies occurred and were removed as described in “Inclusion and Exclusion Criteria,” where more detailed data were available, or when national registry data could replace regionally representative data. From here, two parallel selection processes were run to generate input data for the MI models and to generate incidence for final mortality estimation. Higher priority was given to registry data from the most standardized source when creating the final incidence input, whereas for the MI model input only sources that reported incidence and mortality were used. This is different to GBD 2015 where mortality and incidence could come from different sources as long as they covered the same population.
In the eighth step (#8 in the flowchart) the processed incidence and mortality data from cancer registries were matched by cancer, age, sex, year, and location to generate MI ratios. These MI ratios were used as input for a three-step modelling approach using the general GBD ST-GPR approach with SDI as a covariate in the linear step mixed effects model using a logit link function. Predictions were made without the random effects. The ST-GPR model has three main hyper-parameters that control for smoothing across time, age, and geography. The time adjustment parameter (λ) was set to 2, which aims to borrow strength from neighboring time points (i.e. the exposure in this year is highly correlated with exposure in the previous year but less so further back in time). The age adjustment parameter ω was set to 0.5, which borrows strength from data in neighboring age groups. The space adjustment parameter ξ was set to 0.95 in locations with data and to 0.5 in locations without data (the higher ξ was applied when at least one age-sex group in the country of estimation had at least five unique data points. The lower ξ was applied when estimating data-scarce countries). Zeta aims to borrow strength across the hierarchy of geographical locations.[10] For the amplitude parameter in the Gaussian process regression we used 2 and for the scale we used a value of 15.

As in GBD 2015 we have modified the approach to estimate MI ratios. Since for GBD 2015 MI ratio predictions for some cancers yielded similar predictions for low-SDI countries without data as for high-SDI countries we refined the estimation process. Inclusion criteria for the MI ratio input data were changed to only include mortality and incidence data if they were reported by the same source. We excluded MI ratios reported in the CI5[1,1–7] since mortality data used for the calculation of these MI ratios by definition has to be independent from the cancer registry. We also revised the outliering process and excluded data based on the SDI quintile categorization rather than on development status. For each cancer, MI ratios from locations in SDI quintiles 1-4 (low to high-middle SDI) were dropped if they were below the median of MI ratios from locations in SDI quintile 5 (high SDI). We also dropped MI ratios from locations in SDI quintiles 1-4 if the MI ratios were above the third quartile + 1.5 * IQR (inter-quartile range). We dropped all MIR that were based on less than 25 cases to avoid noise due to small

numbers except for mesothelioma and acute lymphoid leukemia where we dropped MIR that were based on less than 10 cases because of lower data availability for these two cancers. We also aggregated incidence and mortality to the youngest 5-year age bin where we had at least 50 data points to avoid MIR predictions in young age groups that were based on few data points. The MIR in the age-bin that was used to aggregate MIR to, was used to backfill the MIR for younger age groups.

Since MI ratios can be above 1, especially in older age groups and cancers with low cure rates, we used the 95th percentile of the cleaned dataset that only included MIR that were based on 50 or more cases, to cap the MIR input data. This "upper cap" was used to allow MIR over 1 but to constrain the MIR to a maximum level. To run the logit model, the input data was divided by the upper caps and model predictions after ST-GPR was rescaled by multiplying them by the upper caps.
Upper caps used for GBD 2016 were the following:

Age group	Maximum MIR
0-4	0.57
5-9	0.69
10-14	0.81
15-19	0.84
20-24	0.72
25-29	0.62
30-34	0.69
35-39	0.78
40-44	0.86
45-49	0.89
50-54	0.92
55-59	0.95
60-64	0.99
65-69	1.04
70-74	1.10
75-79	1.17
80+	1.32

To constrain the model at the lower end, we used the 5th percentile of the cancer specific cleaned MIR input data to replace all model predictions with this lower cap.

Final MI ratios were matched with the cancer registry incidence dataset in the ninth step (#9 in the flowchart) to generate mortality estimates (Incidence * Mortality/Incidence = Mortality) (#10 in the flowchart). The final mortality estimates were then uploaded into the COD database (#11 in the flowchart). Cancer-specific mortality modelling then followed the general CODEm process.

Liver cancer etiology split models

The proportion data found through the systematic literature review were used as input for four separate DisMod-MR 2.1 models to determine the proportion of liver cancers due to the four subgroups for all locations, both sexes, and all age groups (step #16 in the flowchart). A study covariate was used for

publications that only assessed liver cancer in a cirrhotic population. The reference or "gold standard" that was used for crosswalking was the compilation of all studies that assessed the etiology of liver cancer in a general population. For liver cancer due to hepatitis C and hepatitis B, a prior value of 0 was set between age 0 and 0.01. For liver cancer due to alcohol a prior value of 0 was set for ages 0 to 5 years. For liver cancer due to hepatitis C, hepatitis C (IgG) seroprevalence was used as a covariate as well as a covariate for alcohol (liters per capita) and hepatitis B prevalence (HBsAg seroprevalence), forcing a negative relationship between the alcohol and hepatitis B covariate and the outcome of liver cancer due to hepatitis C proportion. For liver cancer due to hepatitis B, seroprevalence of HBsAg was used as a covariate as well as a covariate for alcohol and hepatitis C IgG seroprevalence, forcing a negative relationship between the alcohol and hepatitis C covariate and the outcome of liver cancer due to hepatitis B proportion. For liver cancer due to alcohol, alcohol (liters per capita) was used as a covariate as well as a covariate for proportion of alcohol abstainers, hepatitis B and hepatitis C seroprevalence, forcing a negative relationship between the proportion of alcohol abstainers, hepatitis B and hepatitis C covariates and the outcome of liver cancer due to alcohol proportion. All covariates used were modelled independently. To ensure consistency between cirrhosis and liver cancer estimates and to take advantage of the data for the respective other related cause (e.g. liver cancer due to hepatitis C and the related cause cirrhosis due to hepatitis C), we generated covariates from the liver cancer proportion models that we used in the cirrhosis etiology proportion models. We then created covariates from the cirrhosis etiology proportion models and used those in the liver cancer etiology models.

Since the proportion models are run independently of each other, the final proportion models were scaled to sum to 100% within each age, sex, year, and location, by dividing each proportion by the sum of the four (step # 17). For the liver cancer subtype mortality estimates, we multiplied the parent cause "liver cancer" by the corresponding scaled proportions (step # 18). Single cause estimates were adjusted to fit into the separately modelled all-cause mortality in the process CoDCorrect.

Results

Interpretation of results

Cancer mortality estimates for GBD 2016 can differ from the GBD 2015 results for multiple reasons. Updated cancer mortality data were added from vital registration system data, verbal autopsy studies, as well as cancer registry incidence data. Mapping of cancer ICD codes to the GBD cancer causes was updated slightly based on collaborator comments. Mapping for the ICD10 code D46 (myelodysplastic syndrome) was changed back to "other cancer" as it had been in GBD 2013 based on collaborator comments and the consideration of adding myelodysplastic syndrome as a separate cause for future GBD iterations. To improve estimation of the leukemia sub-causes, a new cause, "leukemia other" was added since not all leukemia subtypes can be mapped the four most common types (acute and chronic lymphoid and myeloid leukemia). The mortality-to-incidence ratio estimation has changed compared to GBD 2015. Covariate inputs for the CODEm models were changed based on recommendations from collaborators. Covariates used in CODEm models were updated for GBD 2016.
The other group producing country-level cancer mortality estimates is the International Agency for Research on Cancer (IARC) with their GLOBOCAN database. Significantly different methods between the GBD study and GLOBOCAN can lead to differences in results. Whereas estimates in GLOBOCAN are based on the assumption that there are "In theory, […] as many methods as countries,"[11] the cancer estimation process for the GBD study follows a coherent, well-documented method for all cancers,

which allows cross-validation of models as well as determination of uncertainty. Another major difference is the ability in the GBD study to adjust single cause estimates to the all-cause mortality, which is being determined independently. This also allows us to adjust individual causes of death to the all-cause mortality envelope which permits us to correct for the underdiagnosis of cancer in countries with inadequate diagnostic resources. Redistribution of a fraction of undefined causes of death to certain cancers is another methodical advantage the GBD study has over GLOBOCAN, and estimates for cancer mortality can therefore differ substantially in countries with a large proportion of undefined causes of deaths in their vital registration data or a large proportion of undefined cancer cases in their cancer registry data.

Limitations

There are certain limitations to consider when interpreting the GBD mortality cancer estimates. First, even though every effort is made to include the most recently available data for each country, data-seeking resources are not limitless and new data cannot always be accessed as soon as they are made available. It is therefore possible that the GBD study does not include all available data sources for cancer incidence or cancer mortality. Second, different redistribution methods can potentially change the cancer estimates substantially if the data sources used for the estimated location contain a large number of undefined causes; however, neglecting to account for these undefined deaths would likely introduce an even greater bias in the disease estimates. Third, using mortality-to-incidence ratios to transform cancer registry incidence data to mortality estimates requires accurate MIR. For GBD 2016 we have made further changes to the MIR estimation, but the method remains sensitive to underdiagnosis of cancer cases or underascertainment of cancer deaths. However, given that the majority of data used for the cancer mortality estimation come from vital registration data and not cancer registry data this is not a major limitation.

Non-melanoma skin cancer (squamous-cell carcinoma)

Data

Data seeking processes

The input data were identified and processed using the same methods as all other cancers described above.

Inclusion and exclusion criteria

Inclusion and exclusion criteria followed the same methods as described for other cancers (see above).

Bias of categories of input data

The potential biases of the input data are the same as for other cancers (see above).

Methods

Overall methodological process

The GBD produces estimates for non-melanoma skin cancer via two subgroups: non-melanoma skin cancer (basal cell carcinoma) and non-melanoma skin cancer (squamous cell carcinoma). While some cancer registries report non-melanoma skin cancer at the four- or five- digit level required to distinguish between the subtypes (eg, “C44.01” versus “C44.02”, “173.01” versus “173.02), most registries report these cancers at the three-digit level as “C44” or “173” (“Other and unspecified malignant neoplasm of skin”). Because of this, those incident cases that were reported at this three-digit level were split to

"basal cell carcinoma" and "squamous cell carcinoma" based on proportions reported by Karagas et al during the cause disaggregation step (step #5 in the flowchart).[12] Since mortality estimates are produced for squamous cell carcinoma under the assumption that basal cell carcinoma causes almost no deaths, all mortalities reported as "C44" or "173" were mapped to the "squamous cell carcinoma" GBD cause. Apart from this additional step for some incident cases, the remainder of the cancer registry processing was the same as for other cancers as described above.

Steps of analysis and data transformation processes

Non-melanoma skin cancer (squamous cell carcinoma) mortality estimation followed the same steps as the other cancers (see flowchart and description above) except for step #5 in the flowchart as described above.

Model selection

The modelling strategy for non-melanoma skin cancer (squamous cell carcinoma) followed the general CODEm process.

Model performance and sensitivity

The modelling performance and sensitivity for non-melanoma skin cancer (squamous cell carcinoma) mirrored that of the general CODEm process.

Uncertainty intervals

Uncertainty was determined using standard CODEm methodology.

Results

Interpretation of results

Non-melanoma skin cancer mortality estimates are not available from other sources. GLOBOCAN, for example, does not report deaths due to non-melanoma skin cancer. Even though the data availability for non-melanoma skin cancer is poor, the fact that it is the most common incident cancer with rates expected to rise makes it a necessity to include the disease in the GBD framework.

Limitations

Cancer registry data for non-melanoma skin cancer incidence have to be interpreted with caution due to a substantial amount of underreporting or rules that only the first non-melanoma skin cancer has to be registered. Many cancer registries therefore do not include non-melanoma skin cancers at all. For vital registration data we make the assumption that there are no deaths due to non-melanoma skin cancer (basal cell carcinoma), therefore all deaths attributed to basal cell carcinoma were included instead as squamous cell carcinoma.

References

1 Waterhouse J, Muir C, Shanmugaratnam K, Powell J. Cancer Incidence in Five Continents IV. Lyon: IARC, 1982.

2 Curado M, Edwards B, Shin H, *et al.* Cancer Incidence in Five Continents IX. Lyon: IARC, 2007 http://www.iarc.fr/en/publications/pdfs-online/epi/sp160/CI5vol9-A.pdf.

3 Muir C, Mack T, Powell J, Whelan S. Cancer Incidence in Five Continents V. Lyon: IARC, 1987.

4 Parkin D, Muir C, Whelan S, Gao Y, Ferlay J, Powell J. Cancer Incidence in Five Continents VI. Lyon: IARC, 1992.

5 Parkin D, Whelan S, Ferlay J, Raymond L, Young J. Cancer Incidence in Five Continents VII. Lyon: IARC, 1997.

6 Parkin D, Whelan S, Ferlay J, Teppo L, Thomas D. Cancer Incidence in Five Continents VIII. Lyon: IARC, 2002.

7 Forman D, Bray F, Brewster D, *et al.* Cancer Incidence in Five Continents X. 2013. http://ci5.iarc.fr.

8 Engholm G, Ferlay J, Christensen N, *et al.* NORDCAN: Cancer Incidence, Mortality, Prevalence and Survival in the Nordic Countries, Version 7.3 Association of the Nordic Cancer Registries. Danish Cancer Society. 2016; published online Aug 7. http://www.ancr.nu.

9 Steliarova-Foucher E, O'Callaghan M, Ferlay J, Masuyer E, Forman D, Comber H, Bray F. European Cancer Observatory: Cancer Incidence, Mortality, Prevalence and Survival in Europe. Version 1.0 European Network of Cancer Registries, International Agency for Research on Cancer. 2012; published online Sept. http://eco.iarc.fr.

10 GBD 2015 Risk Factors Collaborators. Global, regional, and national comparative risk assessment of 79 behavioural, environmental and occupational, and metabolic risks or clusters of risks, 1990–2015: a systematic analysis for the Global Burden of Disease Study 2015. *The Lancet* 2016; **388**: 1659–724.

11 International Agency for Research on Cancer, World Health Organization. GLOBOCAN estimated cancer incidence, mortality, and prevalence worldwide in 2012. Lyon, France: IARC, 2014 http://globocan.iarc.fr/Default.aspx (accessed April 19, 2016).

12 Karagas MR, Greenberg ER, Spencer SK, Stukel TA, Mott LA. Increase in incidence rates of basal cell and squamous cell skin cancer in New Hampshire, USA. New Hampshire Skin Cancer Study Group. *Int J Cancer* 1999; **81**: 555–9.

3.4.1 Diabetes Mellitus SDG Capstone Appendix

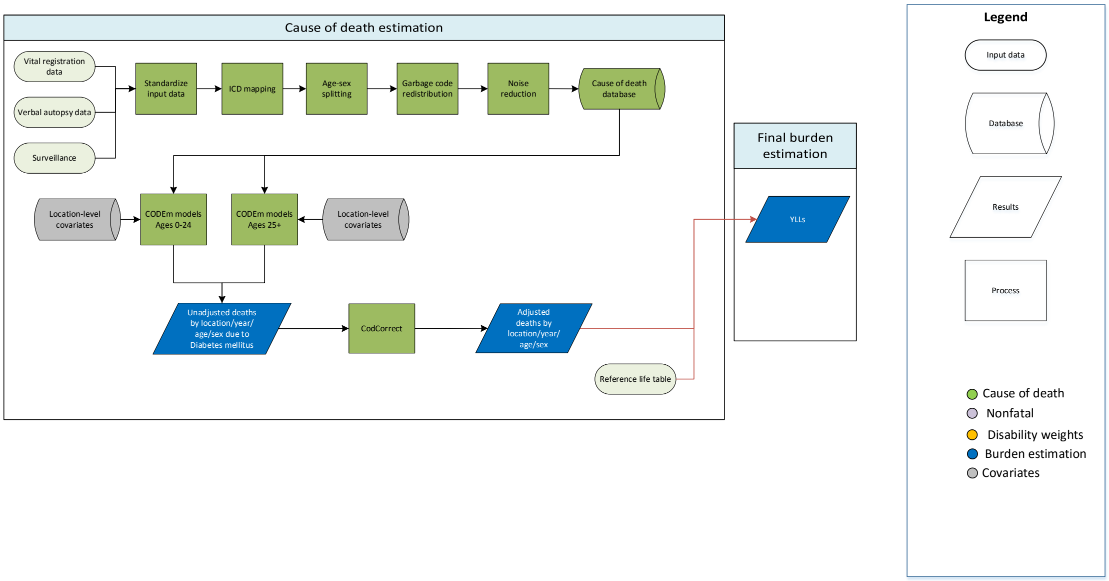

Input data

Verbal Autopsy Data: We outliered VA data points in urban Indian states where high-quality vital registration data were also available. We also outliered data points where the VA data were implausible in all age groups as we determined that these data sources were unreliable.

Vital Registration Data: We outliered all data in four urban Indian states where the source of the data was unreliable according to expert opinion. We also outliered ICD9BTL data points which were inconsistent with the rest of the data series and created unlikely time trends.

Modeling strategy

We used a slight variation on the standard CODEm approach to model deaths from diabetes mellitus. Since deaths in younger age groups are almost exclusively due to Type 1 diabetes while deaths in older ages are primarily due to Type 2, we used two models to estimate overall diabetes deaths. We reviewed the cause-fraction of deaths due to Type 1 and Type 2 diabetes at the global, super region, and regional level. We selected a conservative estimate of 25 years; one model is for deaths in 0-25 year olds and the second model is for deaths in 25+ year olds.

The following list are the covariates included in the model.

- Education years per-capita
- A composite score that approximates access to and quality of personal healthcare (Healthcare Access and Quality Index)
- Lag distributed GDP per capita in base 2010 international dollars
- Estimated national availability of animal fat expressed as kilocalories per capita
- Mean diabetes fasting plasma glucose (mmol/L) by age group
- Age-standardized prevalence of diabetes

- Age-standardized mean body mass index for adults ages 20+ (separate by sex)
- Mean serum total cholesterol (mmol/L) for individuals above age 25
- Mean systolic blood pressure (mmHg) for individuals above age 25
- Estimated energy adjusted national availability of fruits expressed in grams per person per day
- Estimated energy adjusted national availability of vegetables expressed in grams per person per day
- Estimated energy adjusted national availability of whole grains expressed in grams per person per day
- Estimated national availability of dietary energy expressed in kilocalories per person per day

3.4.1 Chronic Respiratory Diseases SDG Capstone Appendix

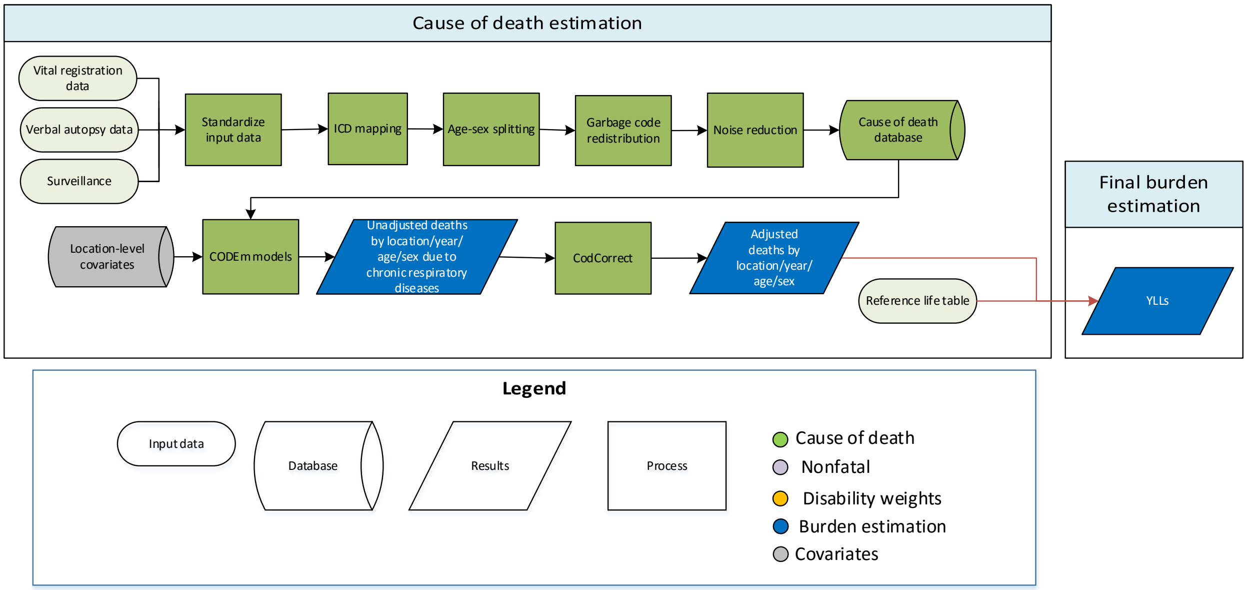

Input data

Sources used to estimate chronic respiratory disease mortality included vital registration, verbal autopsy, and surveillance data from China. Our outlier criteria excluded data points that (1) were implausibly high or low, (2) substantially conflicted with established age or temporal patterns, or (3) significantly conflicted with other data sources conducted from the same locations or locations with similar characteristics (ie, Socio-demographic Index).

Modelling strategy

The standard CODEm modelling approach was applied to estimate deaths due to chronic respiratory diseases. Chronic respiratory diseases served as the parent cause to chronic obstructive pulmonary disease, pneumoconiosis (including silicosis, asbestosis, coal worker's pneumoconiosis, other pneumoconiosis), asthma, interstitial lung disease and pulmonary sarcoidosis, and other chronic respiratory diseases. Functionally, this means the death estimates for Chronic Respiratory Diseases serve as a "parent" envelope into which the "child" causes are squeezed by the CodCorrect algorithm. This approach allows us to use a broader range of data – specifically verbal autopsy data – which cannot be accurately mapped to specific respiratory diseases.

Separate models were conducted for male and female mortality, and the age range for both models was 0 to 95+ years. The same covariates from GBD 2015 were used, with the exception of indoor air pollution, which was changed from cooking-fuel-specific covariates to a generic all cooking fuel covariate.

Level	Covariate	Direction
1	log-transformed SEV scalar: chronic respiratory diseases	+

	cumulative cigarettes (10 years)	+
	cumulative cigarettes (5 years)	+
	health care quality and access index	-
2	smoking prevalence	+
	indoor air pollution (all cooking fuels)	+
	outdoor air pollution ($PM_{2.5}$)	+
	population above 1500m elevation (proportion)	+
3	log LDI (I$ per capita)	-
	education (years per capita)	-
	Socio-demographic Index	-
	population between 500 and 1,500m elevation (proportion)	+
	population density over 1,000 people/square meter (proportion)	+

Beyond changes in the underlying covariates, there were no substantial deviations from the GBD 2015 approach.

Chronic Obstructive Pulmonary Disease

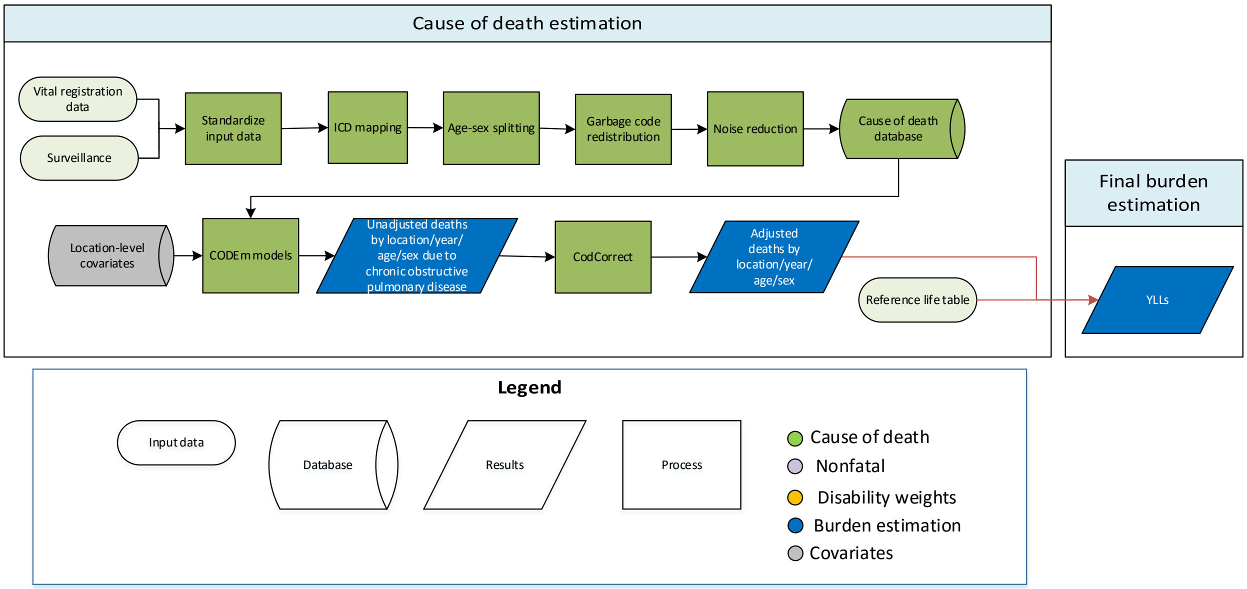

Input data

Data used to estimate chronic obstructive pulmonary disease (COPD) mortality included vital registration and surveillance data from the cause of death (COD) database. Our outlier criteria excluded data points that (1) were implausibly high or low, (2) substantially conflicted with established age or temporal patterns, or (3) significantly conflicted with other data sources conducted from the same locations or locations with similar characteristics (ie, Socio-demographic Index).

Modelling strategy

The standard CODEm modelling approach was applied to estimate deaths due to COPD. Separate models were conducted for male and female mortality, and the age range for both models was 1-95+ years. The mortality estimates from the COPD models were ultimately fit into the chronic respiratory diseases envelope.

The same covariates from GBD 2015 were used, with the exception of indoor air pollution, which was changed from cooking-fuel-specific covariates to a generic all cooking fuel covariate, and the health care access and quality index covariate, which was used in place of health systems access.

Level	Covariate	Direction
1	log-transformed SEV scalar: COPD	+
	cumulative cigarettes (10 years)	+
	cumulative cigarettes (5 years)	+
	elevation over 1,500m (proportion)	+

2	smoking prevalence	+
	indoor air pollution (all cooking fuels)	+
	outdoor air pollution ($PM_{2.5}$)	+
	health care access and quality index	-
3	Socio-demographic Index	-
	log LDI (I$ per capita)	-
	education (years per capita)	-

Pneumoconiosis diseases: Silicosis, asbestosis, coal worker's pneumoconiosis, and other pneumoconiosis

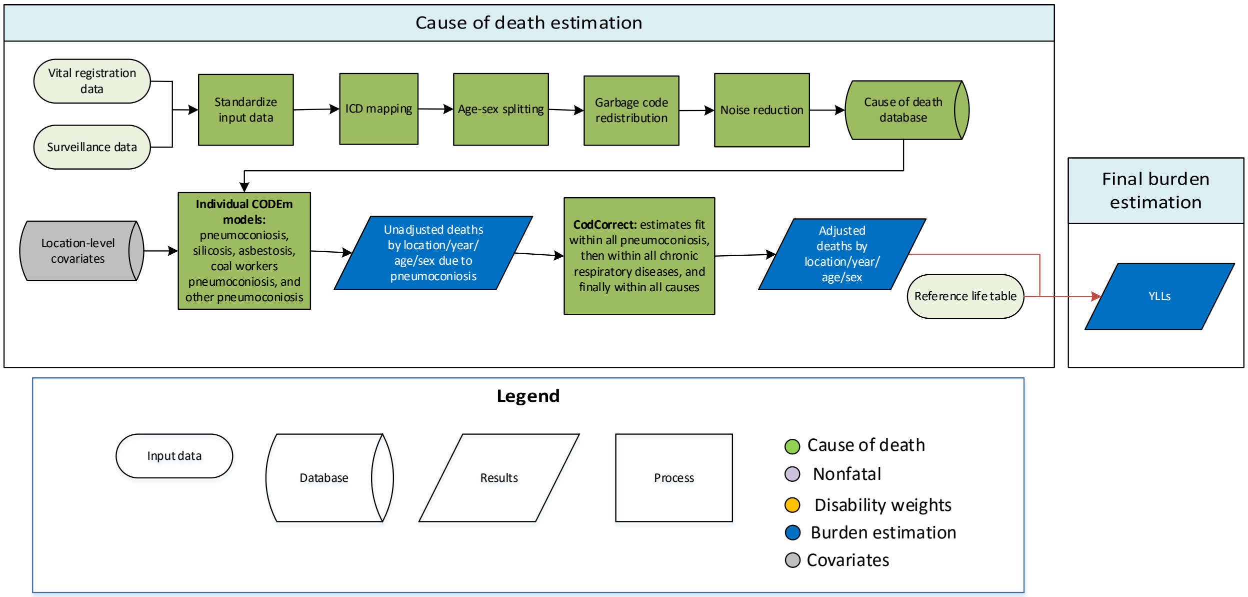

Input data

Data used to estimate pneumoconiosis diseases mortality included vital registration and China mortality surveillance data from the cause of death (COD) database. Our outlier criteria excluded data points that (1) were implausibly high or low, (2) substantially conflicted with established age or temporal patterns, or (3) significantly conflicted with other data sources conducted from the same locations or locations with similar characteristics (i.e., socio-demographic index).

Modelling strategy

The standard CODEm modelling approach was applied to estimate deaths due to pneumoconiosis diseases. Separate models were conducted for male and female mortality, and the age range for both models was 1–95+ years. The mortality estimates from pneumoconiosis disease models were ultimately fit into the chronic respiratory envelope, which is the parent cause for pneumoconiosis disease. The pneumoconiosis model serves as an envelope for silicosis, asbestosis, coal worker's pneumoconiosis, and other pneumoconiosis. In CoDCorrect, estimates are first fit within all pneumoconiosis, then within all chronic respiratory disease, before being fit to the all-cause mortality envelope.

For the most part, the same covariates from GBD 2015 were used. Indoor air pollution was changed from cooking-fuel specific covariates to a generic all cooking fuel covariate. Adjustments were also made to the coal and asbestos covariates.

The coal production covariate was improved to include subnational data for the United States and India. United States state-level data for 2001-2015 came from the U.S. Energy Information Administration. India state-level data for 2005-2014 came from the Ministry of Coal in India. We scaled these figures to the national estimates from the BP Statistical Review of World Energy 2016. For years with missing

state-level data we split the national-level data according to the proportions by state in the closest year for which we did have state-level data.

We also created a covariate for asbestos consumption per capita with a 30-year lag, and used that instead of the GBD 2015 asbestos production covariate. This change is based on the idea that asbestos production may be too limited in scope, given that asbestosis may occur in locations where asbestos is used and handled but not necessarily mined. To create the asbestos consumption covariate we used data from the United States Geological Survey to run a model in DisMod 2.1. A 30-year lag was placed on this model to account for the delay between asbestos consumption and occurrence of disease.

The following table indicates covariates used in the pneumoconiosis models, their level, and direction:

Level	Covariate	Direction
1	log-transformed SEV scalar: pneumoconiosis	+
	asbestos consumption per capita*	+
	coal production per capita*	+
	gold production per capita*	+
2	smoking prevalence	+
	indoor air pollution (all cooking fuels)	+
	cumulative cigarettes (5 years)	+
	elevation over 1,500m (proportion)	+
	elevation 500 to 1,500m (proportion)	+
	health care access and quality index	-
3	log LDI (I$ per capita)	-
	education (years per capita)	-
	Socio-demographic Index	-

* asbestos, coal, and gold covariates are each only used in a subset of the pneumoconiosis models, as follows: all three are included in the parent all pneumoconiosis model, asbestos consumption is included in the asbestosis model, coal production is included in the coal worker’s pneumoconiosis model, and gold production is included in the silicosis model.

Asthma

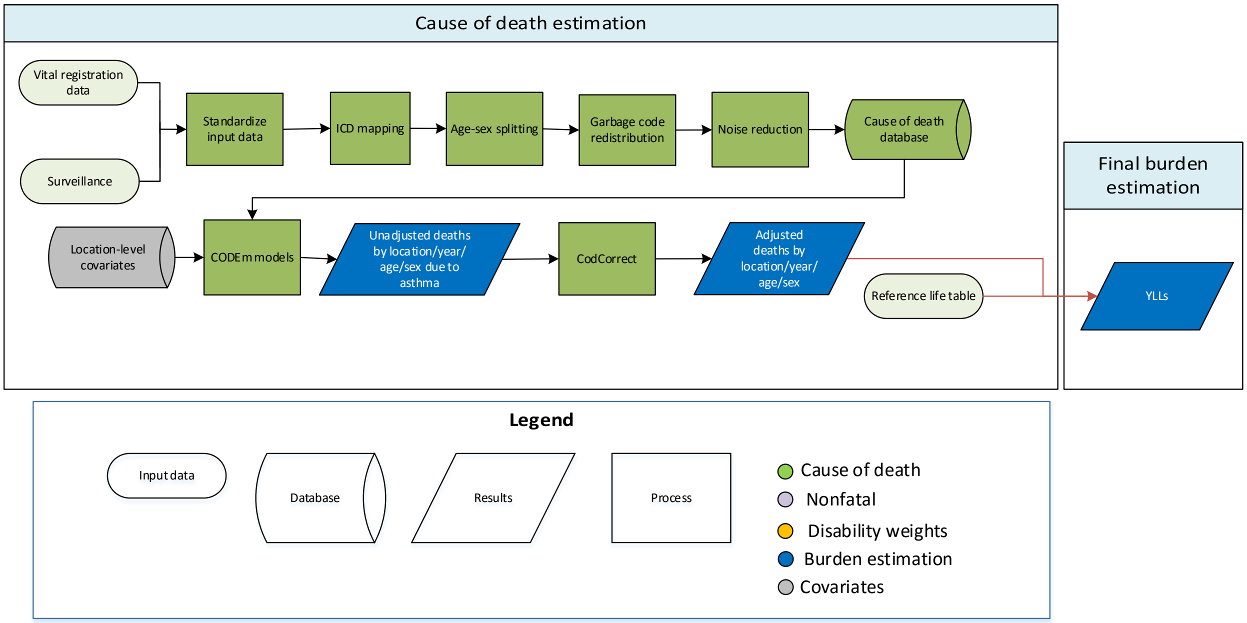

Input data

Data used to estimate asthma mortality included vital registration and surveillance data from the cause of death (COD) database. Verbal autopsy data were not included and were instead mapped to the parent model (Chronic Respiratory Diseases). Our outlier criteria excluded data points that (1) were implausibly high or low relative to global or regional patterns, (2) substantially conflicted with established age or temporal patterns, or (3) significantly conflicted with other data sources conducted from the same locations or locations with similar characteristics (ie, Socio-demographic Index).

Modelling strategy

The standard CODEm modelling approach was applied to estimate deaths due to asthma. Separate models were conducted for male and female mortality, and the age range for both models was 1–95+ years. The mortality estimates from the asthma models were ultimately fit into the chronic respiratory diseases envelope.

The same covariates from GBD 2015 were used, with the exception of indoor air pollution, which was changed from cooking-fuel-specific covariates to a generic all cooking fuel covariate.

Level	Covariate	Direction
1	log-transformed SEV scalar: asthma	+
	cumulative cigarettes (10 years)	+
	cumulative cigarettes (5 years)	+

	health care access and quality index	-
2	smoking prevalence	+
	indoor air pollution (all cooking fuels)	+
	outdoor air pollution ($PM_{2.5}$)	+
3	log LDI (I$ per capita)	-
	education (years per capita)	-
	Socio-demographic Index	-

Interstitial lung disease and pulmonary sarcoidosis

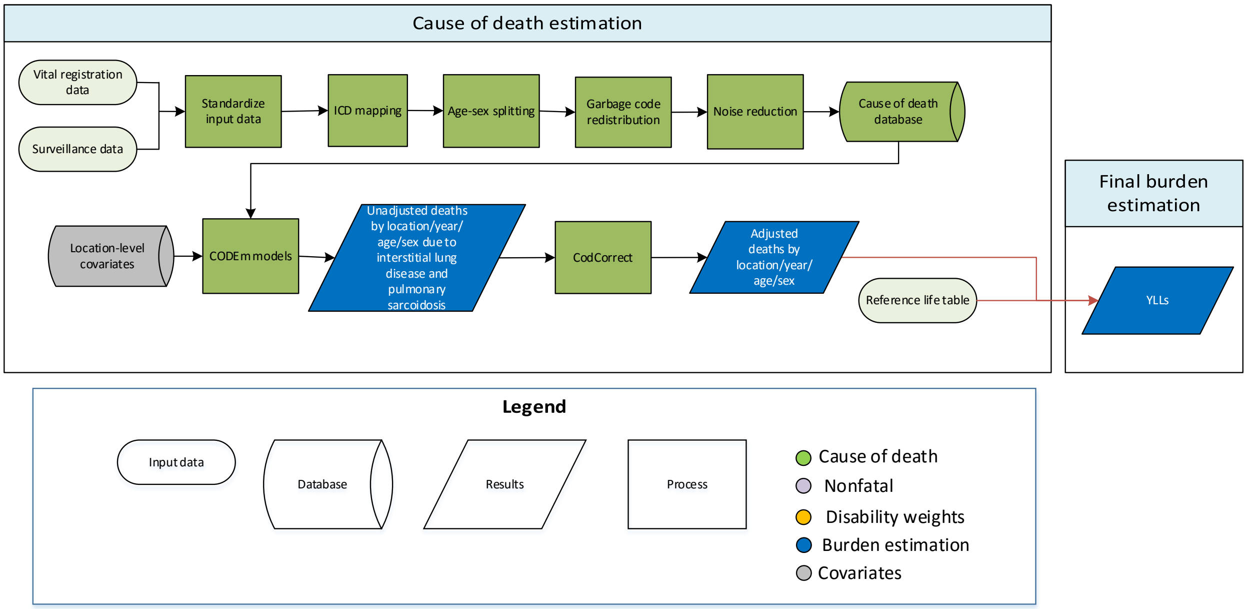

Input data

Data used to estimate interstitial lung disease and pulmonary sarcoidosis mortality included vital registration and surveillance data from the cause of death (COD) database. Our outlier criteria excluded data points that (1) were implausibly high or low, (2) substantially conflicted with established age or temporal patterns, or (3) significantly conflicted with other data sources conducted from the same locations or locations with similar characteristics (ie, Socio-demographic Index).

Modelling strategy

The standard CODEm modelling approach was applied to estimate deaths due to interstitial lung disease and pulmonary sarcoidosis. Separate models were conducted for male and female mortality, and the age range for both models was 1–95+ years. The mortality estimates from the interstitial lung disease and pulmonary sarcoidosis models were ultimately fit into the chronic respiratory envelope.

The same covariates from GBD 2015 were used, with the exception of indoor air pollution, which was changed from cooking-fuel-specific covariates to a generic all cooking fuel covariate.

Level	Covariate	Direction
1	log-transformed SEV scalar: interstitial lung disease	+
	smoking prevalence	+
	cumulative cigarettes (5 years)	+
2	elevation over 1,500m (proportion)	+

	elevation between 500 and 1,500m (proportion)	+
	population density over 1,000 ppl/sqkm (proportion)	+
	indoor air pollution (all cooking fuels)	+
	outdoor air pollution ($PM_{2.5}$)	+
	health care access and quality index	-
3	log LDI (I$ per capita)	-
	education (years per capita)	-
	Socio-demographic Index	-

Other chronic respiratory diseases

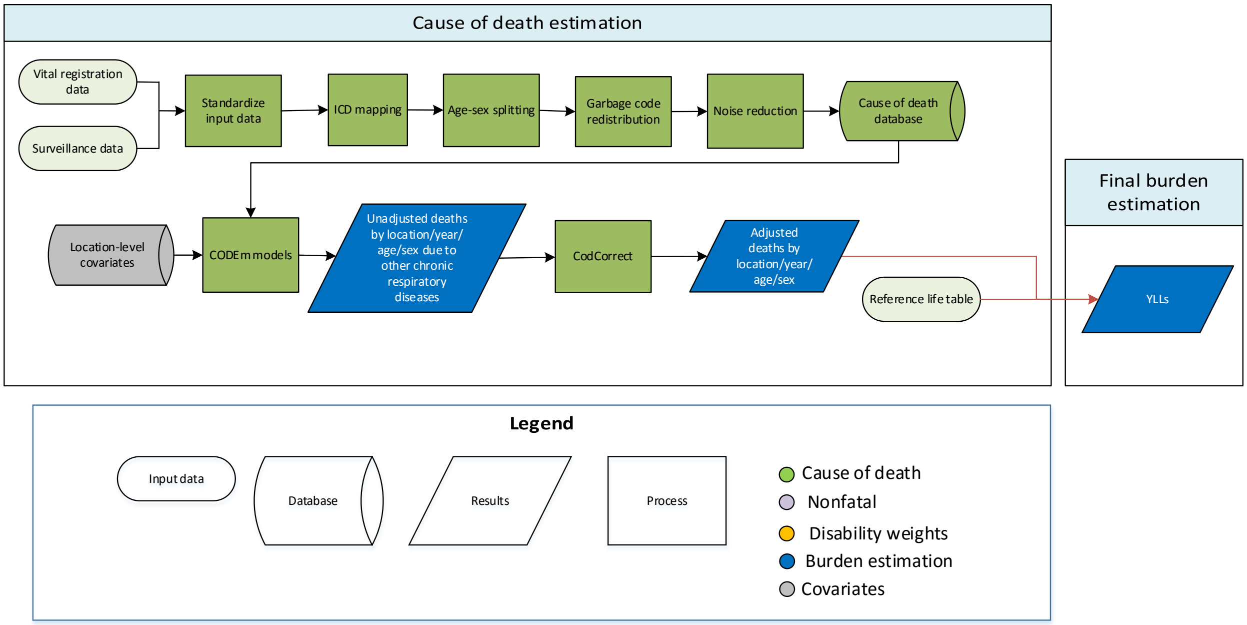

Input data

Data used to estimate other chronic respiratory diseases included vital registration and surveillance data from the cause of death (COD) database. Our outlier criteria excluded data points that (1) were implausibly high or low, (2) substantially conflicted with established age or temporal patterns, or (3) significantly conflicted with other data sources conducted from the same locations or locations with similar characteristics (ie, Socio-demographic Index).

Modelling strategy

The standard CODEm modelling approach was applied to estimate deaths due to other chronic respiratory diseases. Separate models were conducted for male and female mortality, and the age range for both models was 0 days to 95+ years. Like other respiratory causes, the mortality estimates from other chronic respiratory diseases were ultimately fit into the chronic respiratory envelope.

The same covariates from GBD 2015 were used, with the exception of indoor air pollution, which was changed from cooking-fuel-specific covariates to a generic all cooking fuel covariate.

Level	Covariate	Direction
1	log-transformed SEV scalar: other chronic respiratory diseases	+
	smoking prevalence	+
	cumulative cigarettes (5 years)	+
	indoor air pollution (all cooking fuels)	+

	outdoor air pollution ($PM_{2.5}$)	+
2	elevation over 1,500m (proportion)	+
	elevation between 500 and 1,500m (proportion)	+
	population density over 1,000 ppl/sqkm (proportion)	+
	health care access and quality index	-
3	log LDI (I$ per capita)	-
	education (years per capita)	-
	Socio-demographic Index	-

3.4.2, 3.6.1, 3.9.3, 16.1.1 Injuries SDG Capstone Appendix

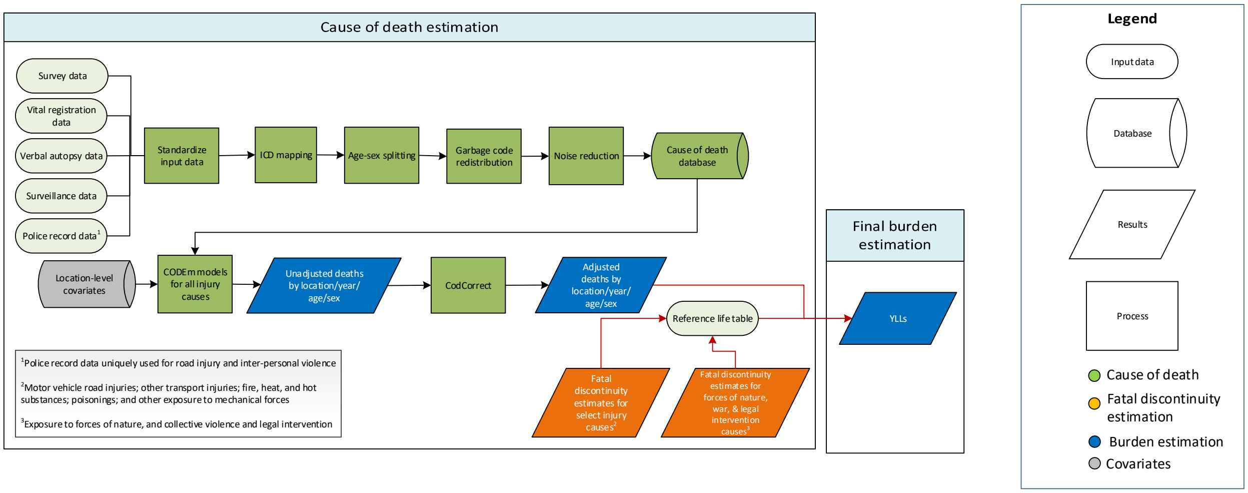

Indicator definition

This modeling strategy encompassed the indicator associated with mortality due to self-harm (3.4.2), road injuries (3.6.1), unintentional poisonings (3.9.3), and interpersonal violence (16.1.1).

Indicator 3.4.2

As a component of SDG Goal 3. Ensure healthy lives and promote well-being for all at all ages, SDG Target 3.4, reduce by one third premature mortality from NCDs through prevention and treatment and promote mental health and well-being, is measured using SDG Indicator 3.4.2, deaths due to self-harm per 100,000.

Indicator 3.6.1

As a component of SDG Goal 3. Ensure healthy lives and promote well-being for all at all ages, SDG Target 3.6, by 2030, halve the number of global deaths and injuries from road traffic accidents, is measured using SDG Indicator 3.6.1, deaths due to road injuries per 100,000.

Indicator 3.9.3

As a component of SDG Goal 3. Ensure healthy lives and promote well-being for all at all ages, SDG Target 3.9, by 2030, substantially reduce the number of deaths and illnesses from hazardous chemicals and air, water and soil pollution and contamination, is measured using SDG Indicator 3.9.3, deaths due to unintentional poisoning per 100,000.

Indicator 16.1.1

As a component of SDG Goal 16. Promote peaceful and inclusive societies for sustainable development, provide access to justice for all and build effective, accountable and inclusive institutions at all levels, SDG Target 16.1, by 2030, significantly reduce all forms of violence and related death rates everywhere, is measured using SDG Indicator 16.1.1, deaths due to interpersonal violence per 100,000.

Input data

In GBD 2016, we estimated injury mortality from vital registration, verbal autopsy, mortality surveillance, censuses, surveys, and police record data. Police and crime reports were data sources uniquely used for the estimation of deaths from road traffic injury and interpersonal violence. The police data were collected from published studies, national agencies, and institutional surveys such as the United Nations Crime Trends Survey and the WHO Global Status Report on Road Safety Survey. For countries with vital registration data we did not use police records, except if the recorded number of road injury and interpersonal violence deaths from police records exceeded that in the vital registration.

Infrequently, data points were marked as outliers. Outlier criteria excluded data points that (1) were implausibly high or low relative to global or regional patterns, (2) substantially conflicted with established age or temporal patterns, or (3) significantly conflicted with other data sources conducted from the same locations or locations with similar characteristics (ie, Socio-demographic Index).

Modelling strategy

Overview

In GBD 2016, the standard CODEm modelling approach was applied to estimate deaths due to all causes of injury, excluding "Exposure to forces of nature," "Military operations and terrorism," and "State actor violence," which fall under the aggregate cause "Forces of nature, military operations and terrorism, and state actor violence." These causes were modelled solely outside of the CODEm process as fatal discontinuities estimation; this process is detailed further in the section on fatal discontinuities estimation in the appendix.

Fatal discontinuity was estimated for five injury causes also modeled in CODEm. These causes included "Motor vehicle road injuries," "Other transport injuries," "Fire, heat, and hot substances," "Poisonings," and "Other exposure to mechanical forces." Final fatal discontinuity estimations for these causes were merged with CODEm results post-CoDCorrect to produce final cause of death results.

Refer to the Table at the end of this section for a complete list of the cause-of-injury categories, modelling strategies, and covariate changes from GBD 2015.

GBD injury codes and categories

The International Classification of Diseases (ICD) was used to classify injuries. In GBD, injury incidence and death are defined as ICD-9 codes E000-E999 and ICD-10 chapters V to Y. There is one exception: deaths and cases of alcohol poisoning and drug overdoses are classified under drug and alcohol use disorders. In GBD 2016, injury causes were organized into 26 mutually exclusive and collectively exhaustive external cause-of-injury categories. For GBD 2016, "Self-harm" was distinguished into "Self-harm by firearm," and "Self-harm by other specified means."

Preparation of data

The preparation of cause of death data includes age splitting, age-sex splitting, smoothing, and outlier detection. These steps are described in detail by Naghavi et al and Lozano et al.[1,2,3] The concept of "garbage codes" and redistribution of these codes was proposed in GBD 1990.[4] Garbage codes are causes of death that should not be identified as specific underlying causes of death but have been entered as the underlying cause of death on death certificates. A classic example of these types of codes in injuries chapters are "Exposure to unspecified factor" (X59 in ICD-10 and E887 in ICD-9) and all undetermined

intent codes (Y10-Y34 in ICD-10 and E980-E988 in ICD-9). Other examples of garbage codes in injuries are the coding of an injury death to intermediate codes like septicemia or peritonitis or as an ill-defined and unknown cause of mortality (R99). Approximately 2% of total deaths in countries with vital registration data are assigned to these three injury garbage code categories.

Splitting into sublevel causes

In countries with non-detail ICD code data, cause-of-injury categories were proportionally split into sublevel cause-of-injury categories. The sublevel cause-of-injury causes were created in the CoDCorrect process. One of the countries with non-detail ICD code data is South Africa, and in GBD 2013 the proportions of sublevel cause-of-injury were based on vital registration data. For GBD iterations of 2015 and 2016 the proportions were based on post-mortem investigation of injury deaths as described in the paper by Matzopoulos et al. 2015.[5]

Limitations and model assumptions

We added police data for road injuries and interpersonal violence to help predict level and age patterns in countries with sparse or absent cause of death data even though we know from countries with near-complete vital registration data that police records tend to underestimate the true level of deaths. However, we applied police data estimates in instances where reported deaths were higher than vital registration numbers.

For the cause-of-injury category “Unintentional suffocation” we suspect that varying practices in coding deaths to sudden infant death syndrome (which end up in “Unintentional suffocation”) can explain some of the differences we see and we plan to explore that further in the next iteration of GBD.

Table – Injury Cause List			
ID	Cause	Modelling Strategy	Covariate changes from GBD 2015
1	Transport injuries	CODEm	
1.1	Road injuries	CODEm	
1.1.a	Pedestrian road injuries	CODEm	
1.1.b	Cyclist road injuries	CODEm	
1.1.c	Motorcyclist road injuries	CODEm	
1.1.d	Motor vehicle road injuries	CODEm and fatal discontinuity estimation	
1.1.e	Other road injuries	CODEm	
1.2	Other transport injuries	CODEm and fatal discontinuity estimation	
2	Unintentional injuries	CODEm	
2.1	Falls	CODEm	
2.2	Drowning	CODEm	
2.3	Fire, heat, and hot substances	CODEm and fatal discontinuity estimation	
2.4	Poisonings	CODEm and fatal discontinuity estimation	
2.5	Exposure to mechanical forces	CODEm	
2.5.a	Unintentional firearm injuries	CODEm	
2.5.b	Unintentional suffocation	CODEm	
2.5.c	Other exposure to mechanical forces	CODEm and fatal discontinuity estimation	
2.6	Adverse effects of medical treatment	CODEm	
2.7	Animal contact	CODEm	
2.7.a	Venomous animal contact	CODEm	
2.7.b	Non-venomous animal contact	CODEm	
2.8	Foreign body	CODEm	
2.8.a	Pulmonary aspiration and foreign body in airway	CODEm	
2.8.b	Foreign body in other body part	CODEm	
2.9	Environmental exposure to heat and cold	CODEm	
2.10	Other unintentional injuries	CODEm	
3	Self-harm and interpersonal violence	CODEm	
3.1	Self-harm	CODEm	
3.1.1	Self-harm by firearm	CODEm	Same covariates used as self-harm from GBD 2015
3.1.2	Self-harm by other specified means	CODEm	Same covariates used as self-harm from GBD 2015
3.2	Interpersonal violence	CODEm	
3.2.a	Assault by firearm	CODEm	
3.2.b	Assault by sharp object	CODEm	
3.2.c	Assault by other means	CODEm	
4	Forces of nature, military operations and terrorism, and state actor violence		

Table – Injury Cause List			
ID	Cause	Modelling Strategy	Covariate changes from GBD 2015
4.1	Exposure to forces of nature	Fatal discontinuity estimation for disaster (appended post-CoDCorrect)	N/A
4.2	State actor violence	Fatal discontinuity estimation for state actor violence (appended post-CoDCorrect)	N/A
4.3	Military operations and terrorism	Fatal discontinuity estimation for state actor violence (appended post-CoDCorrect)	N/A

References

1 Lozano R, Naghavi M, Foreman K, *et al.* Global and regional mortality from 235 causes of death for 20 age groups in 1990 and 2010: a systematic analysis for the Global Burden of Disease Study 2010. *The Lancet* 2012; **380**: 2095–128.

2 Global, regional, and national age–sex specific all-cause and cause-specific mortality for 240 causes of death, 1990–2013: a systematic analysis for the Global Burden of Disease Study 2013. *The Lancet* 2015; **385**: 117–71.

3 Global, regional, and national life expectancy, all-cause mortality, and cause-specific mortality for 249 causes of death, 1980-2015: a systematic analysis for the Global Burden of Disease Study 2015. *The Lancet* 2016; **388**: 1459-1544.

4 Murray CJL, Lopez AD, Harvard School of Public Health, World Health Organization, World Bank. The global burden of disease: a comprehensive assessment of mortality and disability from diseases, injuries, and risk factors in 1990 and projected to 2020. Cambridge, MA: Published by the Harvard School of Public Health on behalf of the World Health Organization and the World Bank : Distributed by Harvard University Press, 1996.

5 Matzopoulos R, Prinsloo M, Wyk VP, Gwebushe N, Mathews S, et al. Injury-related mortality in South Africa: a retrospective descriptive study of postmortem investigations. *Bull World Health Organ* 2015; **93**: 303–13.

3.5.2 Alcohol use SDG capstone appendix

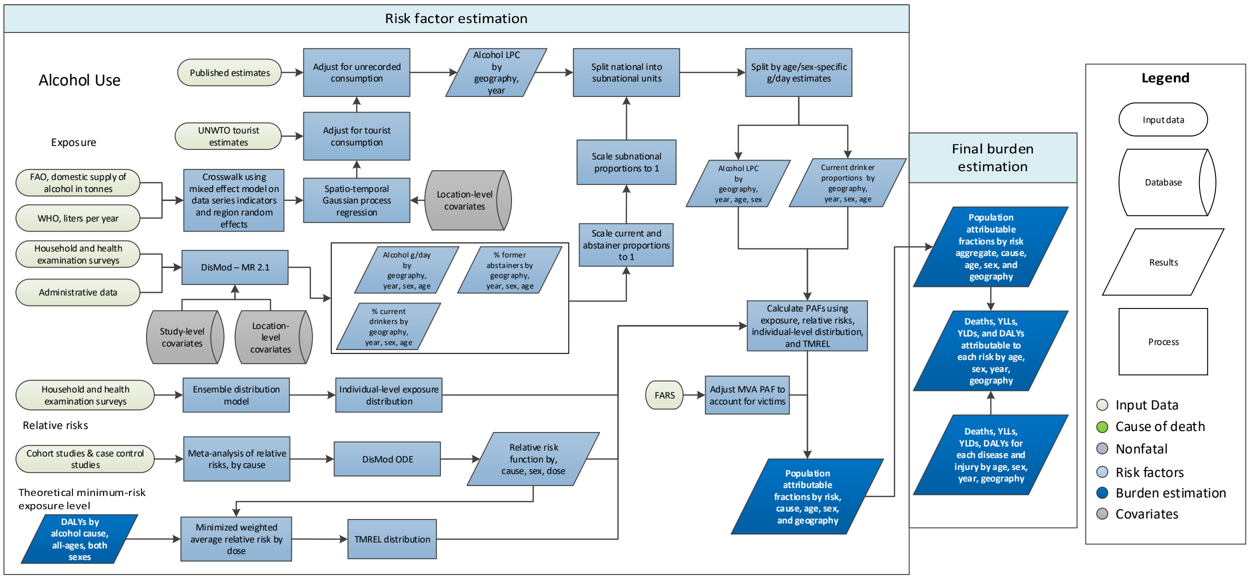

Indicator definition

This modeling strategy encompassed the indicator associated with alcohol use (3.5.2).

Indicator 3.5.2

As a component of SDG Goal 3. Ensure healthy lives and promote well-being for all at all ages, SDG Target 3.5, by 2030, strengthen the prevention and treatment of substance abuse, including narcotic drug abuse and harmful use of alcohol, is measured using SDG Indicator 3.5.2, risk-weighted prevalence of alcohol use.

Exposure

Definitions

We defined exposure as the grams per day of pure alcohol consumed amongst drinkers. We constructed this exposure using the indicators outlined below:

1. Current drinkers, defined as the proportion of individuals who have consumed at least one alcoholic beverage (or some approximation) in a 12-month period.
2. Lifetime abstainers, defined as the proportion of individuals who have never consumed an alcoholic beverage.
3. Alcohol consumption (in grams per day), defined as grams of alcohol consumed by current drinkers, per day, over a 12-month period.
4. Alcohol liters per capita stock, defined in liters per capita of pure alcohol, over a 12-month period.

We also used three additional indicators to adjust alcohol exposure estimates to account for different types of bias:

1. Number of tourists within a location, defined as the total amount of visitors to a location within a 12 month period.
2. Tourists' duration of stay, defined as the number of days resided in a hosting country.
3. Unrecorded alcohol stock, defined as a percentage of the total alcohol stock produced outside established markets.

Input data

A systematic review of the literature was performed to extract data on our primary indicators. The Global Health Exchange (GHDx), IHME's online database of health-related data, was searched for population survey data containing participant-level information from which we could formulate the required alcohol use indicators on current drinkers, lifetime abstainers, alcohol consumption, and binge drinkers. Data-sources were included if they captured a sample representative of the geographic location under study. We documented relevant survey variables from each data-source in a spreadsheet and extracted using STATA 13.1 and R 3.3 . A total of 2,821 potential data-sources were available in the GHDx across countries with subnational locations, out of which 191 data-sources (corresponding 88,734 tabulated data-points by location/year/sex/age) were included across the four indicators mentioned above.

To generate estimates of alcohol consumption in liters per capita (LPC), we obtained data from FAOSTAT, and WHO GISAH database [1-2]. To provide more stable time trends in the model, we transformed FAO sales data (which calculates stock based on primary inputs) to a lagged five-year average. Given WHO uses FAO data in locations where WHO could not find data using their own methods, we removed FAO data in the locations where WHO used FAO data in place of their own. To correct for bias in the underlying data sources, we adjusted the input data (crosswalked), by running a mixed effect model on the log average of the data with dummy variables for the data series, as well as random effects on super region, region, country, and time. We adjusted the data points using the following equation:

$$Log\ Average\ Data = D + (Super\ Region\ |D, Region\ |\ D, Country\ |D, Year|\ D)$$

$$Transformed\ data = \ data\ *\ e^{\widehat{\beta_1} + \widehat{\beta_3}}$$

where:

$D\ is\ a\ dummy\ variable\ for\ a\ data\ source$

None of the data sources on liters per capita provided estimates of uncertainty, which is a component required for our eventual modeling strategy. To generate uncertainty, we ran a Loess model on the adjusted data points and the standard deviation between the difference of the Loess smoothed model and the adjusted data points across a five-year span was used as the standard deviation of the data. (i.e., if the total stock changes more variably in a narrow time frame, we believe the data to be more uncertain).

We obtained data on the number of tourists and their duration of stay from the UNWTO [4]. We applied a crosswalk across different tourist categories, similar to the one used for the liters per capita data, to arrive at a consistent definition (i.e. visitors to a country).

We obtained estimates on unrecorded alcohol stock from six published papers [4-9], consisting of 166 locations.

Modeling Strategy

While population-based surveys provide accurate estimates of the prevalence of lifetime abstainers and current drinkers, they typically underestimate real alcohol consumption levels [10-12]. As a result, we considered the liter per capita input to be a better estimate of overall volume of consumption. Per capita consumption, however, does not provide age- and sex-specific consumption estimates needed to compute alcohol-attributable burden of disease. Therefore, we use the age-sex pattern of consumption among drinkers modeled from the population survey data and the overall volume of consumption from FAO and GISAH to determine the total amount of alcohol consumed within a location. In the paragraphs we outline how we estimated each primary input in the alcohol exposure model, as well as how we combined these inputs to arrive at our final estimate of grams per day of pure alcohol. We estimated all models below using 1000 draws.

For data obtained through surveys, we used DisMod-MR 2.1 to construct estimates for each country/year/age/sex. We chose to use DisMod due to its ability to leverage information across the heterogeneous age groups reported in the surveys, through age-integration, as well as the model's ability to leverage information available from data in nearby locations or time-periods [13].

We modeled the alcohol liters per capita data, as well as the total number of tourists, using a spatio-temporal Gaussian process regression (ST-GPR). We chose parameters, as well as our final model, using out-of-sample 10-fold cross validation.

Given the heterogeneous nature of the estimates on unrecorded consumption, as well as the wide variation across countries and time-periods, we took 1000 draws from the uniform distribution of the lowest and highest estimates available for a given country. We did this to incorporate the diffuse uncertainty within the unrecorded estimates reported. We used these 1000 draws in the above equation. We adjusted LPC only for countries where estimates were available.

We adjusted the alcohol LPC for unrecorded consumption using the following equation:

$$Alcohol\ LPC = \frac{Alcohol\ LPC}{(1 - \%\ Unrecorded)}$$

We then adjusted the estimates for alcohol LPC for tourist consumption by adding in the per capita rate of consumption abroad and subtracting the per capita rate of tourist consumption domestically.

$$Alcohol\ LPC_d = Unadjusted\ Alcohol\ LPC_d + Alcohol\ LPC_{Domestic\ consumption\ abroad} - Alcohol\ LPC_{Tourist\ consumption\ domestically}$$

$Alcohol\ LPC_{i} =$
$$\frac{\sum_{l} Tourist\ Population_{l} * Proportion\ of\ tourists_{i,l} * Unadjusted\ Alcohol\ LPC_{l} * \frac{Average\ length\ of\ stay_{i,l}}{365} *}{Population_{d}}$$

where:

$l\ is\ the\ set\ of\ all\ locations, i\ is\ either$ Domestic consumption abroad or Tourist consumption domestically, $and\ d\ is\ a\ domestic\ location$

After adjusting alcohol LPC by tourist consumption and unrecorded consumption for all location/years reported, sex-specific and age-specific estimates were generated by incorporating estimates modeled in DisMod for percentage of current drinkers within a location/year/sex/age, as well as consumption trends modeled in the DisMod g/day model. We do this by first making sure the sum of percent current drinkers and percent abstainers sum to one for a given location/year/age/sex. We then calculate the proportion of total consumption for a given location/year by age and sex, using the estimates of alcohol consumed per day, the population size, and the percentage of current drinkers. Lastly, we then multiply this proportion of total stock for a given location/year/sex/age by the total stock for a given location/year to calculate the consumption in terms of liter per capita for a given location/year/sex/age. We then convert these estimates to be in terms of grams/per day. The following equations describe these calculations:

$$\%\ Current\ drinkers_{l,y,s,a} = \frac{\%\ Current\ drinkers_{l,y,s,a}}{\%\ Current\ drinkers_{l,y,s,a} + \%\ Abstainers_{l,y,s,a}}$$

$$Proportion\ of\ total\ consumption_{l,y,s,a} = \frac{Alcohol\ \ g/day_{l,y,s,a} * Population_{l,y,s,a} * \%\ Current\ drinkers_{l,y,s,a}}{\sum_{s,a} Alcohol\ \ g/day_{l,y,s,a} * Population_{l,y,s,a} * \%\ Current\ drinkers_{l,y,s,a}}$$

$$Alcohol\ LPC_{l,y,s,a} = \frac{Alcohol\ LPC_{l,y} * Population_{l,y} * Proportion\ of\ total\ consumption_{l,y,s,a}}{\%\ Current\ drinkers_{l,y,s,a} * Population_{l,y,s,a}}$$

$$Alcohol\ g/day_{l,y,s,a} = Alcohol\ LPC_{l,y,s,a} * \frac{1000}{365}$$

where:

$l\ is\ a\ location, y\ is\ a\ year, s\ is\ a\ sex, and\ a\ is\ an\ age\ group.$

We then used the gamma distribution to estimate individual level variation within location, year, sex, age drinking populations, following the recommendations of other published alcohol studies [7-8]. We chose parameters of the gamma distribution based on the mean and standard deviation of the 1000 draws of alcohol g/day exposure for a given population.

Theoretical minimum-risk exposure level

We calculated TMREL by first calculating the overall risk attributable to alcohol. We did this by weighting each relative risk curve by the share of overall DALYs for a given cause. We then took the minimum of this overall-risk curve as the TMREL of alcohol-use. More formally,

$$TMREL = argmin\ average\ overall\ risk_{\omega}(g/day)$$

$$Average\ overall\ risk_{\omega}(g/day) = \sum_{i}^{\omega} RR_i(g/day) * \frac{DALY_i}{\sum_i^{\omega} DALY_i}$$

Where:

ω *is the set of causes associated with alcohol*, i *is a given cause from that set*, *DALY is the global DALY rate in* 2010, *and RR is the dose response curve for a given cause and exposure level in grams per day*.

In other words, we chose TMREL as being the exposure that minimizes your risk of suffering burden from any given cause related to alcohol. We weight the risk for a particular cause in our aggregation by the proportion of DALYs due to that cause. (e.g. since more observed people die from IHD, we weight the risk for IHD more in the above calculation of average risk compared to, say, diabetes, even if both have the same relative risk for a given level of consumption)

Relative risks

For GBD2016, we performed a systematic literature review of all cohort and case-control studies reporting a relative risk, hazard ratio, or odds ratio for any risk-outcome pairs studied in GBD 2016. Studies were included if they reported a categorical or continuous dose for alcohol consumption, as well as uncertainty measures for their outcomes, and the population under study was representative.

We then used these studies to calculate a dose-response, modeled using DisMod ODE. We chose DisMod ODE rather than a conventional mixed effect meta-regression because of its ability to estimate nonparametric splines over doses (i.e. for most alcohol causes, there is a non-linear relationship with different doses) and incorporate heterogeneous doses through dose-integration (i.e. most studies report doses categorically in wide ranges. DisMod ODE estimates specific doses when categories overlap across studies, through an integration step.) We used the results of the meta-regression to estimate a non-parametric curve for all doses between 0-150 g/day and their corresponding relative risks. For all causes, we assumed the relative risk was the same for all-ages and sexes, with the exception of ischemic heart disease, ischemic stroke, hemorrhagic stroke, and diabetes, which we estimated by sex.

Regarding injuries outcomes, we constructed relative risks based on chronic exposure rather than acute, which has a weaker relationship to the outcome, though still significant [15-16, 18-21]. We decided to use chronic exposure given the lack of available data on acute exposure, as well as, the lack of cohort studies using acute exposure as a metric. Further, using chronic exposure allowed us to construct relative risks curves for unintentional injuries, interpersonal violence, motor vehicle accidents, and self-harm using the same method as reported above.

In the case of motor vehicle accidents, we adjusted the PAF to account for victims of drunk drivers that are involved in accidents. Using data from the Fatality Analysis Reporting System in the US [17], we calculated the average number of fatalities in a car crash involving alcohol, as well as the percentage of those fatalities distributed by age and sex (figures 1 and 2). We aggregated FARS data across the years 1985-2015, given there was little variation in the data temporally and the number of cases in old age groups had too much variance when constructing estimates by year. To adjust PAFs, we multiplied attributable deaths by the average number of fatalities from FARS and redistributed the PAF amongst each population, based on the probability of being a victim to a certain drunk driver by age and sex, based on the FARS data. The following equation describes this process:

$$Adjusted\ PAF_i = \frac{\sum_d PAF_d * DALY_d * Avg\ Fatalities_d * \ P(i\ is\ a\ victim)_d}{DALY_i}$$

where:

$i\ is\ a\ population\ by\ location, year, age, sex\ and$
$d\ is\ the\ set\ of\ all\ age\ and\ sex\ exposed\ groups\ within\ that\ location\ and\ year.$

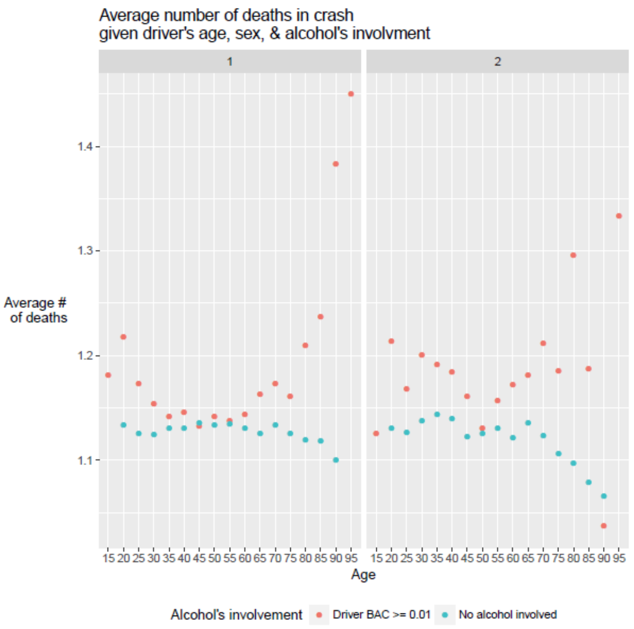

Figure 1

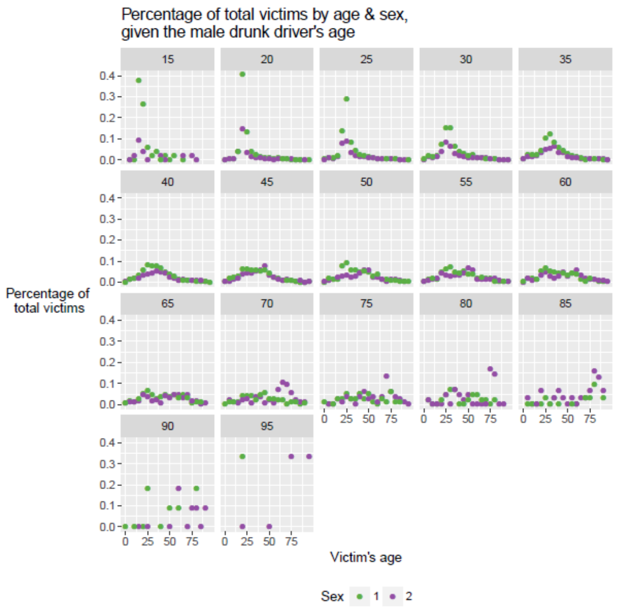

Figure 2

PAF

For all causes, we defined PAF as:

$$PAF(x) = \frac{P_A + \int_0^{150} P(x) * RR_C(x)\, dx - 1}{P_A + \int_0^{150} P(x) * RR_C(x)\, dx} \qquad P(x) = P_C * \Gamma(\boldsymbol{p})$$

where:

P_C *is the prevalence of current drinkers*, P_a *is the prevalence of abstainers*, $RR_c(x)$ *is the relative risk function for current drinkers, and* $\boldsymbol{p}$ *are parameters determined by the mean and sd of exposure*

We performed the above equation for 1000 draws of the exposure and relative risk models. We then used the estimated PAF draws to calculate YLL, YLDs, and DALYs, as per the other risk factors.

References

1. Food and Agriculture Organization of the United Nations (FAO). FAOSTAT Food Balance Sheets, October 2014. Rome, Italy: Food and Agriculture Organization of the United Nations (FAO).
2. World Health Organization (WHO). WHO Global Health Observatory - Recorded adult per capita alcohol consumption, Total per country. Geneva, Switzerland: World Health Organization (WHO).
3. UN World Tourism Organization (UNWTO). UN World Tourism Organization Compendium of Tourism Statistics 2015 [Electronic]. Madrid, Spain: UN World Tourism Organization (UNWTO), 2016.
4. Norstrom, Thor. "Estimating changes in unrecorded alcohol consumption in Norway using indicators of harm." *Addiction* 93.10 (1998): 1531-1538.
5. Macdonald, Scott. "Unrecorded alcohol consumption in Ontario, Canada: estimation procedures and research implications." *Drug and Alcohol Review* 18.1 (1999): 21-29.
6. Meier, Petra Sylvia, et al. "Adjusting for unrecorded consumption in survey and per capita sales data: quantification of impact on gender-and age-specific alcohol-attributable fractions for oral and pharyngeal cancers in Great Britain." *Alcohol and Alcoholism* 48.2 (2013): 241-249.
7. Hao, Wei, Hanhui Chen, and Zhonghua Su. "China: alcohol today." *Addiction* 100.6 (2005): 737-741.
8. Rehm, Jürgen, and Vladimir Poznyak. "On monitoring unrecorded alcohol consumption." *Alcoholism and Drug Addiction* 28.2 (2015): 79-89.
9. Probst et al. "Unrecorded Alcohol Use: A global modeling study based on Delphi assessments and survey data". Toronto, Canada: CAMH.
10. Ramstedt, Mats. "How much alcohol do you buy? A comparison of self-reported alcohol purchases with actual sales." *Addiction* 105.4 (2010): 649-654.
11. Stockwell, Tim, et al. "Under-reporting of alcohol consumption in household surveys: a comparison of quantity–frequency, graduated–frequency and recent recall." *Addiction* 99.8 (2004): 1024-1033.
12. Kerr, William C., and Thomas K. Greenfield. "Distribution of alcohol consumption and expenditures and the impact of improved measurement on coverage of alcohol sales in the 2000 National Alcohol Survey." *Alcoholism: Clinical and Experimental Research* 31.10 (2007): 1714-1722.
13. An Integrative Metaregression Framework for Descriptive Epidemiology. Abraham D. Flaxman, Theo Vos, Christopher J. L. Murray. Seattle: University of Washington Press, [2015]
14. Taylor, Bruce, et al. "The more you drink, the harder you fall: a systematic review and meta-analysis of how acute alcohol consumption and injury or collision risk increase together." *Drug and alcohol dependence* 110.1 (2010): 108-116.
15. Vinson, Daniel C., Guilherme Borges, and Cheryl J. Cherpitel. "The risk of intentional injury with acute and chronic alcohol exposures: a case-control and case-crossover study." *Journal of studies on alcohol* 64.3 (2003): 350-357.

16. Vinson, Daniel C., et al. "A population-based case-crossover and case-control study of alcohol and the risk of injury." *Journal of studies on alcohol*64.3 (2003): 358-366.
17. Fatal Accident Reporting System (FARS). National Highway Traffic Safety Administration, National Center for Statistics and Analysis Data Reporting and Information Division (NVS-424); 1985, 1990, 1995, 2000, 2005, 2010, 2015
18. Chen, Li-Hui, Susan P. Baker, and Guohua Li. "Drinking history and risk of fatal injury: comparison among specific injury causes." *Accident Analysis & Prevention* 37.2 (2005): 245-251.
19. Bell, Nicole S., et al. "Self-reported risk-taking behaviors and hospitalization for motor vehicle injury among active duty army personnel." *American journal of preventive medicine* 18.3 (2000): 85-95.
20. Margolis, Karen L., et al. "Risk factors for motor vehicle crashes in older women." *The Journals of Gerontology Series A: Biological Sciences and Medical Sciences* 57.3 (2002): M186-M191.
21. Sorock, Gary S., et al. "Alcohol-drinking history and fatal injury in older adults." *Alcohol* 40.3 (2006): 193-199.

3.7.1 Met Need for Family Planning with Modern Methods
SDG Capstone Appendix

Flowcharts

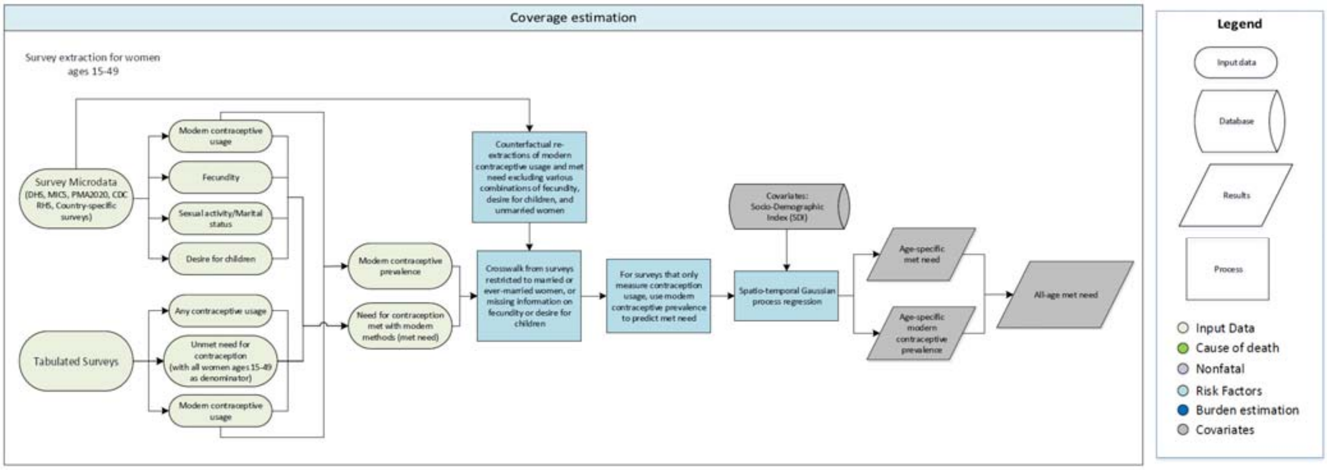

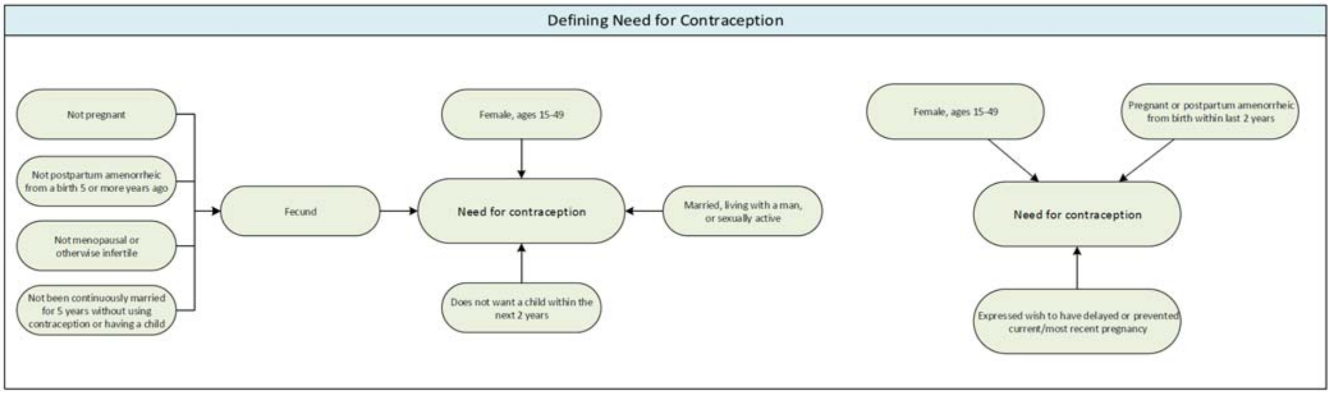

Input Data & Methodological Summary

Indicator definition

This modeling strategy encompassed the indicator associated with the proportion of women aged 15 to 49 years with their family planning needs met with modern contraception methods (3.7.1). This indicator also is an individual component of Indicator 3.8.1, which the indicator for universal health coverage (UHC) index.

Indicator 3.7.1

As a component of SDG Goal 3. Ensure healthy lives and promote well-being for all at all ages, SDG Target 3.7, by 2030, ensure universal access to sexual and reproductive health-care services, including for family planning, information and education, and the integration of reproductive health into national strategies and programmes, is measured using SDG Indicator 3.7.1 proportion of women of reproductive age (15 to 49 years) who are sexually active and have their need for family planning satisfied with modern methods (ie, female and male sterilization, oral hormonal pills, intra-uterine devices (IUD), male condoms, injectables, implants [including Norplant], vaginal barrier methods, female condoms, and emergency contraception)

Input data

We defined modern contraception methods as the current use of male or female sterilization, male or female condoms, spermicide foam/jelly, oral contraceptive, diaphragms, implants, injections, emergency contraceptives, or use of an IUD. Traditional contraception methods were defined as the current use of alternative methods including but not limited to withdrawal, periodic abstinence, the rhythm method, and lactational amenorrhea method (LAM).

Women between the ages of 15 and 49 were defined as having need for family planning if they were using any method of contraception, or if they were fecund, sexually active, and did not wish to become pregnant within the next two years. We defined met need with modern methods as the proportion of women who have a need for contraception that are using modern methods. Women were assumed to be fecund unless they met one or more of the following criteria: (1) were pregnant (2) were postpartum amenorrheic from a birth that occurred 5 or more years ago (3) had not menstruated within the last 6 months (unless postpartum amenorrheic for less than 5 years) (4) had been continuously married/in a union for 5 or more years without having a child and without ever having used any method of contraceptive (modern or traditional) (5) otherwise indicated that they were infertile (ex. mentioned having had a hysterectomy). Women who were pregnant or postpartum amenorrheic from a birth within the last 2 years were considered separately, and were determined to have a need for contraception if they indicated a desire to have delayed or prevented their current or most recent pregnancy.

The present study used two primary types of input data in order to ultimately generate a time series of met need for family planning with modern methods: (1) individual-level microdata from which met need for family planning with modern methods could be directly estimated; and (2) tabulated data from which met need with modern methods could be indirectly calculated based on reported estimates of modern contraception coverage, any contraception coverage, and unmet need for family planning.

Our primary data sources for met need with modern methods included multi-country survey series such as Demographic and Health Surveys (DHS), Multiple Indicator Cluster Surveys (MICS), and Centers for Disease Control and Prevention Reproductive Health Surveys (CDC RHS). In addition, we extracted data from the Performance Monitoring and Accountability 2020 (PMA2020) surveys, to which we were granted access. We originally sought a wider universe of population surveys, but our search was somewhat restricted to the survey series for which information on contraception use by method and marital status was readily available for all women of reproductive age. Notably, relatively few microdata sources were available for higher-income countries; subsequently, we heavily relied on tabulated data for these geographies.

The below table shows the number of studies included in the 2016 SDG Capstone paper.

Surveys	Contraception Methods	Met Need
DHS	277	256
MICS	149	136
CDC RHS	50	30
PMA2020	34	34
Country-specific	207	29

Among the surveys for which we had access to microdata, we applied survey weights based on survey sampling frames to generate weighted national estimates of met need accompanied by estimates of standard error (SE). In the absence of microdata or survey sampling information, we used survey sample sizes as a mechanism for informing uncertainty estimation.

A number of our survey report data sources did not include tabulated estimates of met need with modern methods; instead, such sources would include information on prevalence of modern contraception use, prevalence of any contraception use, and prevalence of unmet need for family planning among women of reproductive age. Following the recommended analytic approach from DHS and Inter-agency Expert Group on the SDG Indicators (IAEG-SDGs)[1,2], we estimated met need with modern methods based on this formula:

$$Prev_{MetMod} = \frac{Prev_{Mod}}{Prev_{Any} + Prev_{Unmet}}$$

where $Prev_{MetMod}$ is the prevalence of met need with modern methods among women aged 15 to 49 years; $Prev_{Mod}$ is the prevalence of current modern contraception use among women aged 15 to 49 years; $Prev_{Any}$ is the prevalence of any contraception use among women aged 15 to 49 years; and $Prev_{Unmet}$ is the prevalence of women who have need for family planning but are not currently using any method of contraception. In future iterations of this analysis, we will prioritize gaining access to microdata to these surveys, so that we can directly estimate met need with modern methods from individual-level data.

For a subset of surveys, contraception use and met need was only reported for women who were currently or had ever been married. To predict the prevalence of modern contraceptive use and met need for all women, we re-extracted those microdata surveys for which met need could be calculated for all women, this time restricting our re-analysis to just currently or ever married women. We used the average difference between the original extractions and the counterfactual re-extractions to crosswalk modern contraceptive use and met need for those surveys which only sampled current or ever-married women. These crosswalks were performed separately within each 5-year age group to account for the potential influence of age on the effect of the restricted sampling. Additionally, some surveys did not ask questions related to fecundity or the desire for children within the next two years, creating a potential bias in our met need estimates. We repeated this prediction method with the additional factors related to met need for family planning, re-extracting microdata to inform age-specific crosswalks. This allowed us to predict met need for women in countries where information on fecundity and desire for children were unavailable. When multiple issues applied to a survey (for example, when a survey was restricted to married women and also lacked questions related to fecundity), we crosswalked only once, using a counterfactual re-extraction that matched the issues in the survey, in order to account for potential interaction effects between multiple survey issues.

After accounting for differences between survey sampling frames and question types, we leveraged the relationship between modern contraception use and met need with modern contraption to predict met need where only modern contraceptive prevalence data was available. To do this, we ran a regression of met need observations against modern contraceptive prevalence by age group. To account for geographical differences in the relationship between modern contraceptive prevalence and met need, the regression included an interaction term between modern contraceptive use and super region.

Modelling strategy

For the present analysis, we used Spatiotemporal Gaussian process regression (ST-GPR), a model used widely within the GBD study to synthesize coherent trends and uncertainty from multiple sources of data. The first stage included fitting a mixed-effect linear model with fixed effects on age and the Socio-Demographic Index, with random effects for countries, GBD regions, and GBD super-regions. Then smoothing over space time based on the residuals from the first-stage linear model took place, followed by GPR to generate a cohesive time series of met need with modern contraception and uncertainty for all 188 countries and from 1990 to 2016.

After generating age-specific estimates of met need and modern contraceptive prevalence, we could calculate age-specific estimates of need for contraception using the formula:

$$Prev_{Need} = \frac{Prev_{Mod}}{Prev_{MetMod}}$$

where $Prev_{Need}$ is the age-specific prevalence of need for contraception among women aged 15 to 49. Using $Prev_{Need}$ we could calculate the weights needed to aggregate age-specific met need for contraception into total met need among women aged 15 to 49, which is the SDG indicator reported in this paper.

References

1. Bradley, S. E. K., Croft, T. N. & Fishel, J. D. Revising Unmet Need for Family Planning: DHS Analytical Studies No. 25. 63 (2012).

2. United Nations Department of Economics and Social Affairs. *Goal 3: Ensure healthy lives and promote well-being for all at all ages*. (2015). at <https://sustainabledevelopment.un.org/sdg3>

3.7.2 Adolescent Birth Rate SDG Capstone Appendix

Flowchart

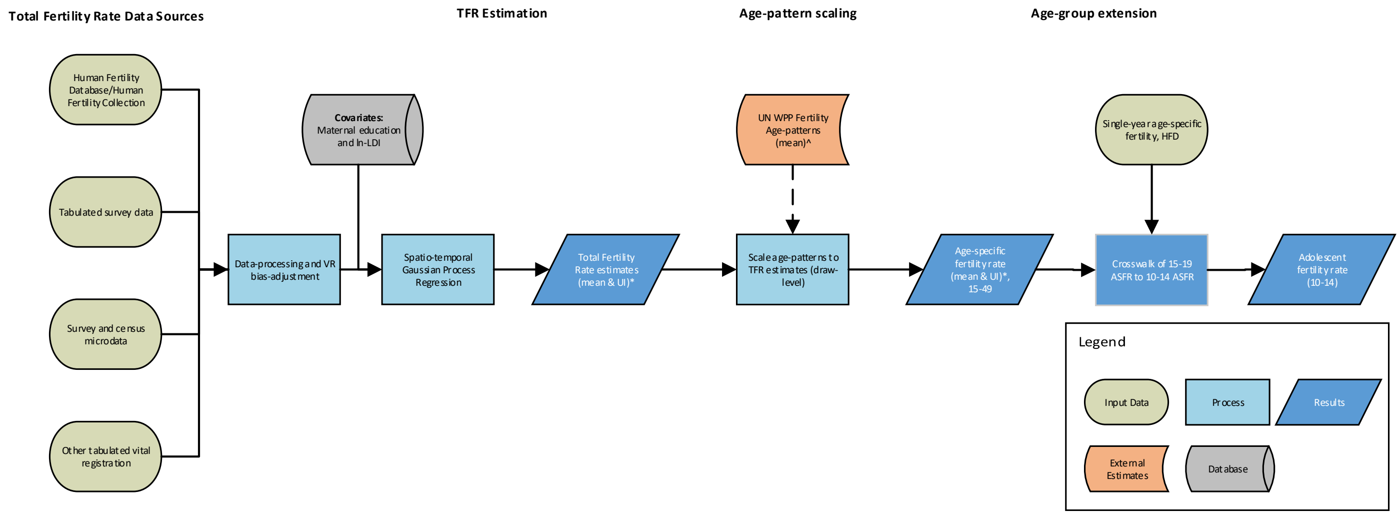

Input data & Methodological summary

Indicator definition

This modeling strategy encompassed the indicator associated with adolescent birth rates (3.7.2).

Indicator 3.7.2

As a component of SDG Goal 3. Ensure healthy lives and promote well-being for all at all ages, SDG Target 3.7, by 2030, ensure universal access to sexual and reproductive health-care services, including for family planning, information and education, and the integration of reproductive health into national strategies and programmes, is measured using SDG Indicator 3.7.2, adolescent birth rates (number of livebirths per 1,000 women) for women aged 10 to 14 years and women aged 15 to 19 years.

Input data

For developed countries with complete vital registration systems, we primarily utilized tabulated TFRs provided by the Human Fertility Database (HFD), a repository of rigorously-vetted empirical fertility data curated by the Max Planck Institute for Demographic Research (MPIDR). Also managed by MPIDR, the Human Fertility Collection (HFC) provided a plethora of additional data for developing countries and countries that lack complete vital registration systems. Though they are also vetted prior to inclusion in the database, not all the data from the HFC meet the complete quality standards of those from the HFD, nor are they all empirical in nature. Accordingly, from the HFC we excluded all research estimates applying methods beyond standard demographic techniques used to compute TFR from raw empirical data. We additionally maintained all HFC data from vital statistics, tabulated surveys, and official government statistical reports.

At the national level we extracted all surveys in the GHDx that fully met complete birth history requirements, applying standard direct estimation methods to generate age-specific fertility rates (ASFR),

which we collapsed to TFR. Where microdata were unavailable, we extracted tabulated TFRs from the corresponding survey reports. At the subnational level, we re-utilized surveys that had been extracted for GBD 2015, which included both complete and summary birth histories. Additional data sources included provisions from international collaborators as well as tabulated reports from national statistics offices. In total, 20, 260 location years of data were included in our model over the 1950-2016 estimation period. For GBD 2017 we hope to expand our extractions to all available summary birth histories and registrations of live births, as well as hope to receive more data directly from collaborators.

Modeling strategy

Overview

In previous iterations of GBD, fertility estimates from UN WPP were used for all national locations for which they were available. For subnational locations and miscellaneous countries not covered by UN WPP we modeled total fertility rate (TFR) using spatio-temporal Gaussian process regression (ST-GPR).

To improve upon estimates used in the past and ensure consistency within our processes, we expanded the use of ST-GPR to model TFR for all 755 national and subnational locations included in GBD 2016. UN WPP age patterns were then scaled to our estimates of TFR to produce age-specific fertility rates, including those of the 15-19 age group. Lastly, to produce estimates for the 10-14 age group, we developed and implemented a crosswalk to predict fertility rates for that age group from those of the 15-19 age group.

Trend estimation – Total Fertility Rate

We used a spatiotemporal Gaussian process regression (ST-GPR) to synthesize point estimates from multiple data sources and derive a complete time series for total fertility rate. This method has been used extensively in GBD and related studies, and accounts for uncertainty pertaining to each point estimate while borrowing strength across geographic space and time.[1-3] Briefly, we assumed the Gaussian process was defined by a mean function *m*(•) and covariance function *Cov*(•).

We estimated the mean function using a two-step approach. Specifically, $m_c(t)$ can be expressed as:

$$m_c(t) = X\beta + h(r_{c,t})$$

where $X\beta$ is a linear model and $h(r_{c,t})$ is a smoothing function for the residuals; and $r_{c,t}$ is derived from the linear model. The following linear model was used for the estimation of TFR:

$$logit\left(tfr_{c,t}/9.5\right) = \beta_0 + (\beta_1 + \omega_{1\mathrm{R[c]}})medu_{\mathrm{c,t}} + (\beta_2 + \omega_{2\mathrm{R[c]}})lnLDI_{\mathrm{c,t}} + \alpha_c + \gamma_{R[c]} + \varepsilon_{c,t}$$

where $tfr_{c,t}$ is TFR for country c year t; $medu_{c,t}$ and $lnLDI_{c,t}$ are the level of maternal education and natural log of per capita lag-distributed income for country c and year t; α_c and $\gamma_{R[c]}$ are country and region random intercepts; and $\omega_{\mathrm{iR[c]}}$ are region random slopes for the aforementioned covariates. These estimates were then modeled through ST-GPR.

The upper bound on TFR of 9.5 was informed by data from the 1981 Jordanian demographic survey, from which a TFR of 9.1 was calculated for 1972. This represented the maximum observed TFR from all available location-years of data.

Hyperparameters for the spatiotemporal smoothing and Gaussian-process regression stages were dichotomized and selected based on the extent of vital registration completeness in a given country, as determined previously assessed for CODEm. Accordingly, countries were categorized as either data rich or not data rich. Data rich countries were assigned hyper-parameters that drew relatively less strength over space and time and yielded less variability in the mean function of the Gaussian process, such that our predictions would be more driven by high-quality, in-country data. The converse applied to countries not deemed data rich.

Random draws of 1,000 samples were obtained from the distributions above for every country. Ninety-five percent uncertainty intervals were calculated by taking the ordinal 25 and 975th draws from the sample distribution.

Lastly, we scaled the outputs of the Gaussian process regression for subnational locations to national-level estimates, except in the cases of China (where Hong Kong and Macao do not exhibit trends in fertility reflective of the mainland) and the UK (where much of our high-quality data spanning the complete estimation period was at the level of the four member states). In these two cases, the national estimates were instead population-weighted aggregates of the first tier of subnational locations. Estimates for secondary, and, in the case of the UK, tertiary, tiers of subnational locations were still scaled to those of their respective geographic parents.

Trend estimation – Age-specific fertility rate (15-19)

For all locations reported by UN WPP, we scaled interpolated UN WPP age-specific fertility rates (15-49) to GBD 2016 estimates of Total Fertility Rate (TFR). Interpolation was necessary, as UN WPP only produces estimates for fertility indicators in five-year intervals.

Trend estimation – Age-specific fertility rate (10-19)

Since the youngest age group for which UN WPP produces age-specific fertility is 15-19, we developed a crosswalk to predict fertility rates in the 10-14 age group. We utilized tabulated age-specific fertility rates from the Human Fertility Database (HFD). HFD reports single year age-specific fertility from 1950-2016, with the exception of an aggregate group for 12 and under. We collapsed the single-year data into five year age groups—10-14 and 15-19—and fit a Loess of degree 1 to characterize the secular trend. The loess was then used to generate estimates of 10-14 fertility corresponding to all estimates of 15-19 fertility produced for GBD 2016. One clear limitation in this method is that HFD only covers developed countries with complete vital registration systems, and we lacked additional data sources for the 10-14 age group specific to any country not included in HFD.

For future iterations of GBD, we intend to model the 10-14 age group directly for all countries as we expand data-seeking and extraction efforts.

References

1 Ng M, Freeman MK, Fleming TD, *et al.* Smoking Prevalence and Cigarette Consumption in 187 Countries, 1980-2012. *JAMA* 2014; **311**: 183.

2 Ng M, Fleming T, Robinson M, *et al.* Global, regional, and national prevalence of overweight and obesity in children and adults during 1980–2013: a systematic analysis for the Global Burden of Disease Study 2013. *The Lancet* 2014; **384**: 766–81.

3 Wang H, Liddell CA, Coates MM, *et al.* Global, regional, and national levels of neonatal, infant, and under-5 mortality during 1990–2013: a systematic analysis for the Global Burden of Disease Study 2013. *The Lancet* 13; **384**: 957–79.

3.8.1 Universal Health Coverage (UHC) Index SDG Capstone Appendix

Input data & Methodological summary

Indicator definition

This modeling strategy involves the construction of a composite measure for the universal health coverage (UHC) index (SDG Indicator 3.8.1), which includes nine measures of coverage for a subset of interventions for communicable diseases and maternal and child health and the 32 causes that comprise the Healthcare Access and Quality (HAQ) Index, a summary measure of personal healthcare access and quality based on risk-standardised death rates from causes amenable to healthcare.[1] Our UHC measurement approach used for GBD 2016 represents a substantial improvement since GBD 2015,[2] considerably expanding the representation of essential health services pertaining to reproductive, maternal, newborn, and child health (RMNCH); infectious diseases; non-communicable diseases; and service capacity and access. Each component of the UHC index was scaled on a scale of 0 to 100, with 0 being the worst observed from 1990 to 2016 and 100 being the best observed during this time, and then the arithmetic mean was taken of each component. We then projected the UHC index, based on past trends, as a composite indicator from 2017 to 2030.

The measures of intervention coverage are as follows: three doses of diphtheria-pertussis-tetanus (DPT3), measles vaccine, three doses of the oral polio vaccine or inactivated polio vaccine; met need for family planning with modern methods; antenatal care (ANC) coverage (one ANC visit [ANC1] and four ANC visits [ANC4]); skilled birth attendance (SBA); in-facility delivery rates; and coverage of antiretroviral therapy (ART) among people living with HIV.

The causes that comprise the HAQ Index are as follows: tuberculosis, diarrheal diseases, lower respiratory infections, upper respiratory infections, diphtheria, whooping cough, tetanus, measles, maternal disorders, neonatal disorders, colon and rectum cancer, non-melanoma cancer, breast cancer, cervical cancer, uterine cancer, testicular cancer, Hodgkin's lymphoma, leukemia, rheumatic heart disease, ischemic heart disease, cerebrovascular disease, hypertensive heart disease, peptic ulcer disease, appendicitis, hernia, gallbladder and biliary diseases, epilepsy, diabetes, chronic kidney disease, congenital heart anomalies, and adverse effects of medical treatment.

Indicator 3.8.1

As a component of SDG Goal 3. Ensure healthy lives and promote well-being for all at all ages, SDG Target 3.8, achieve universal health coverage, including financial risk protection, access to quality essential health-care services and access to safe, effective, quality and affordable essential medicines and vaccines for all, is measured using SDG Indicator 3.8.1.

The UN definition of Indicator 3.8.1 is "Coverage of essential health services (defined as the average coverage of essential services based on tracer interventions that include reproductive, maternal, newborn and child health, infectious diseases, non-communicable diseases and service capacity and access)," with which we sought to more closely align by combining measures of RMNCH and ART intervention coverage and component parts of the HAQ Index.

UHC indicator input data

Individual UHC index components serve as the input data for the overall UHC index, and unless otherwise specified, their write-ups are included in this portion of the appendix. Exceptions were interventions or causes covered by other indicator write-ups, such as skilled birth attendance (SDG indicator 3.1.2); maternal disorders (SDG indicator 3.1.1); and ardiovascular diseases, cancers, diabetes, and chronic respiratory diseases considered amenable to healthcare (SDG indicator 3.4.1).

UHC index component	Appendix content
Vaccine coverage (diphtheria-pertussis-tetanus vaccination, three doses [DPT3], measles, and polio vaccination, 3 doses)	Under 3.8.1
Antenatal care (ANC), 1 visit (ANC1) and 4 visits (ANC4)	Under 3.8.1
Skilled birth attendance (SBA)	Indicator 3.1.2
In-facility delivery rate (IFD)	Under 3.8.1
Met need for family planning with modern contraception methods	Indicator 3.7.1
Antiretroviral therapy (ART) coverage among people living with HIV	Under 3.8.1
Causes of death included in the Healthcare Access and Quality (HAQ) Index: tuberculosis, diarrheal diseases, lower respiratory infections, upper respiratory infections, diphtheria, whooping cough, tetanus, measles, maternal disorders, neonatal disorders, colon and rectum cancer, non-melanoma cancer, breast cancer, cervical cancer, uterine cancer, testicular cancer, Hodgkin's lymphoma, leukemia, rheumatic heart disease, ischemic heart disease, cerebrovascular disease, hypertensive heart disease, chronic respiratory diseases, peptic ulcer disease, appendicitis, hernia, gallbladder and biliary diseases, epilepsy, diabetes, chronic kidney disease, congenital heart anomalies, and adverse effects of medical treatment.	Maternal disorders covered by Indicator 3.1.1; cancers, cardiovascular diseases, diabetes, and chronic respiratory diseases are covered under Indicator 3.4.1

In sum, each component is estimated within the broader GBD study, with many of the measures of intervention coverage used as covariates to inform cause-specific models. Most of the individual interventions use population health survey microdata, or tabulated report data when microdata are not publicly available, as their primary input data sources. For vaccination, administrative data sources are also used to supplement survey-based estimates.

Each cause of death considered amenable to healthcare is estimated as part of the GBD cause of death analysis. Risk-standardization of cause-specific death rates is based on the joint population attributable fraction (PAF) of environmental or occupational and behavioral risks as quantified by the GBD comparative risk assessment.[3] Additional information risk-standardization is provided in the next section.

UHC Index modeling strategy

Summary

To construct the composite UHC Index, we first risk-standardized cause-specific death rates (as described below) and used draw-level estimates for both intervention coverage and risk-standardized cause-specific death rates computed as part of GBD 2016. For each input, 1,000 draws were used in order to estimate uncertainty. We then scaled each UHC Index component on a scale of 0 to 100 from 1990 to 2016, and took the arithmetic mean across components.

Risk standardization and rescaling

We estimated a joint-risk (PAF) for each cause, using all risks except metabolic risks, which are so directly linked to personal healthcare, and thus would not be appropriate for risk-standardisation steps. Assumptions about how one risk factor is mediated through other risk factors are needed in order to estimate the joint-risk factor burden for combinations of behavioural or environmental risks. To accomplish this, for every two risk factors for an outcome, we used published studies to estimate the fraction of risk that was mediated through the other risk. This resulted in a matrix of parameters containing each possible pairing of risk factors included in the GBD 2015.[3] Using this matrix, we computed the aggregated burden of disease at each level of the GBD 2015 hierarchy and for all risk factors using the following formula:

$$PAF_{J,o,a,s,g,t} = 1 - \prod_{j=1}^{J}\left(1 - PAF_{j,o,a,s,g,t}\prod_{i=1}^{J}(1 - MF_{j,i,o})\right)$$

where J is a set of risk factors for the aggregation; $PAF_{j,o,a,s,g,t}$ is the PAF for risk j for cause o, age group a, sex s, geography g, and year t; and $MF_{j,i,o}$ is the mediation factor for risk j mediated through i for cause o.

The risk standardisation process is a key innovation of this work on amenable mortality. The aim of this process is to eliminate the residual effects of localised risk exposure that would otherwise act as a confounding element in our ability to draw inferences about healthcare from mortality due to amenable causes. By imposing a global level of exposure on all geographies, we de-contextualise them and create a measure of mortality that is a more appropriate proxy for healthcare access and quality. Death rates are risk-standardised using the formula:

$$RSD_{l,y,a,s} = D_{l,y,a,s} \times \left(1 - PAF_{l,y,a,s}\right) \times \frac{1}{1 - GPAF_{a,s}}$$

where $RSD_{l,y,a,s}$ is the risk-standardised death rate in location l, year y, age group a, and sex s; $D_{l,y,a,s}$ is the death rate for those specifications; $PAF_{l,y,a,s}$ is the PAF for those specifications; and $GPAF_{a,s}$ is the global PAF over all six estimation years for age group a, and sex s. If for a given cause no risk attribution is present or all deaths are attributed to risks (ie, PAF of 0 or 1), the observed deaths are used. Also, for diarrhoeal diseases, observed deaths are used due to the fact that no PAF is estimated for water and sanitation in high income countries.

If any cause has a maximum observed mean joint-risk PAF greater than 0.9 but less than 1 for a given age and sex, PAFs across all location years are scaled such that the maximum is scaled down to 0.9. Deaths outside of the ages defined for each amenable cause were eliminated,[1] as only deaths in those ages were deemed highly amenable to healthcare. We then aggregated cause-specific mortality rates by sex to both sexes. Using the GBD population age standard, we compiled age-standardised risk-standardised mortality rates for both sexes by location, year, and amenable cause:

$$RSASD_{l,y} = \sum_{a=1}^{n} RSD_{l,y,a} \times PAS_a$$

here $RSASD_{l,y,d}$ is the age-standardised risk-standardised death rate for location l, and year y; $RSD_{l,y,a}$ is the risk-standardised death rate in location l, year y, and age group a; and $PAS_a$is the population age standard for age group a.

In order to have a standardised score of amenable mortality by cause across location-years, we then take the age-standardized risk-standardised death rates and convert them to a 0 to 100 index value:

$$I_{l,y} = \frac{\log(RSASD_{l,y}) - \min(\log(RSASD'_{CY}))}{\max(\log(RSASD'_{CY})) - \min(\log(RSASD'_{CY}))}$$

where $I_{l,y}$ and $RSASD_{l,y}$ are the cause-specific index values and age-standardised risk-standardised death rate for location l and year y, respectively. $RSASD'_{CY}$ is the matrix age-standardised risk-standardised death rates for all countries with populations over one million for each of the six years. We apply the limitation of population size for the purpose of eliminating elongated tails in the location-year distribution, which leads to clustering of the indexed values nearer to the center. In order to eliminate zeroes while maintaining the observed range, we added one death per 1 million population to all values before log transformation.

Creating the UHC index and projections based on past trends

Using the above methodology, we created 1,000 draws of location-year index values for 32 causes of death amenable to healthcare. In combination with the 1,000 draws for each of the nine intervention coverage indicators, we created a composite measure – the UHC Index – based on these 41 components.

To do this, we first log-transformed each of the amenable causes and then scaled them on a scale of 0 to 100, with 0 being the worst observed from 1990 to 2016 and 100 being the best observed during this time. No transformation was needed for the intervention coverage measures, so we applied the same scaling approach to them as well. We then took the arithmetic mean of each of the 41 components to construct the UHC Index, by country, from 1990 to 2016. We then projected the UHC index from 2017 to 2030 on the basis of past trends; additional detail on the projection methodologies used in the present study can be found in Section 1 Part 3 in the Methods Appendix.

References

1 Barber RM, Fullman N, Sorensen RJD, *et al.* Healthcare Access and Quality Index based on mortality from causes amenable to personal health care in 195 countries and territories, 1990–2015: a novel analysis from the Global Burden of Disease Study 2015. *The Lancet* 2017; 0. DOI:10.1016/S0140-6736(17)30818-8.

2 Lim SS, Allen K, Bhutta ZA, *et al.* Measuring the health-related Sustainable Development Goals in 188 countries: a baseline analysis from the Global Burden of Disease Study 2015. *The Lancet* 2016; **388**: 1813–50.

3 Forouzanfar MH, Alexander L, Anderson HR, *et al.* Global, regional, and national comparative risk assessment of 79 behavioural, environmental and occupational, and metabolic risks or clusters of risks in 188 countries, 1990–2013: a systematic analysis for the Global Burden of Disease Study 2013. *The Lancet* 2015; **386**: 2287–323.

3.8.1 UHC Index – Immunizations SDG Capstone Appendix

Flowchart

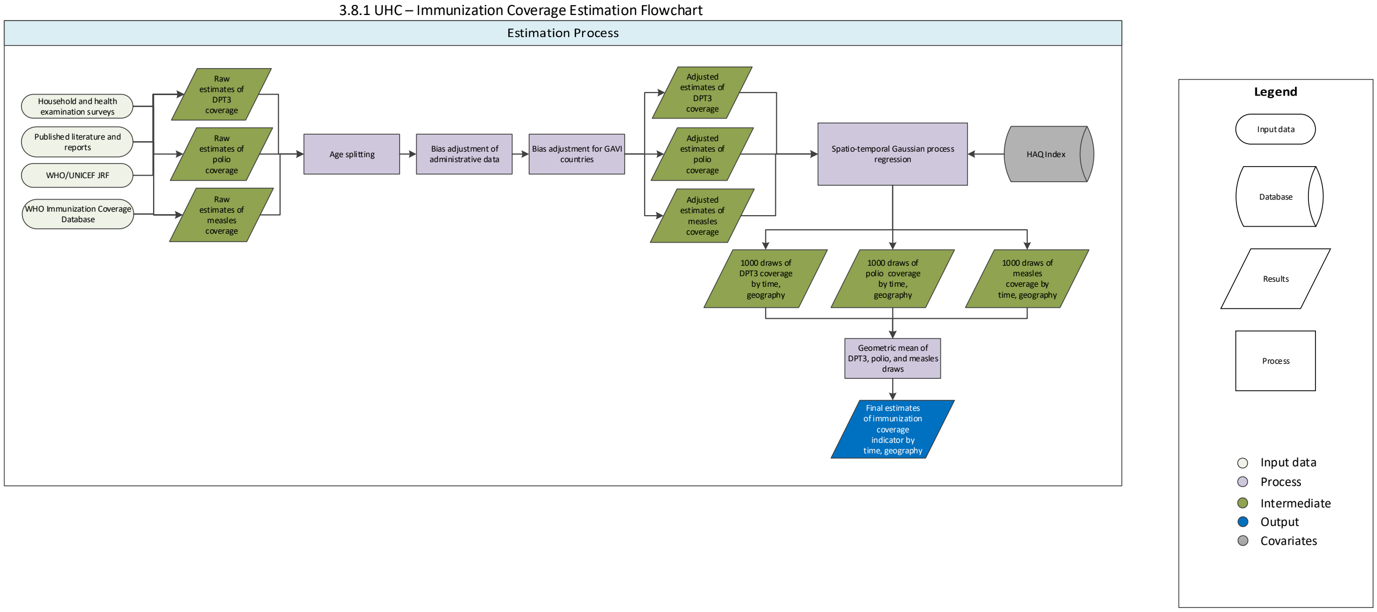

Input data & Methodological summary

Indicator definition

This modeling strategy pertains to the composite universal health coverage (UHC) index (Indicator 3.8.1) and specifically the estimation of immunization coverage for diphtheria-pertussis-tetanus (DPT3), measles vaccine, and three doses of the oral polio vaccine or inactivated polio vaccine (OPV3 or IPV3).

Indicator 3.8.1

As a component of SDG Goal 3. Ensure healthy lives and promote well-being for all at all ages, SDG Target 3.8, achieve universal health coverage, including financial risk protection, access to quality essential health-care services and access to safe, effective, quality and affordable essential medicines and vaccines for all, is measured using SDG Indicator 3.8.1, three measures of immunization coverage: DPT3, measles, and polio (OPV3 and/or IPV3) among children aged 12 to 23 months.

Input data

The present study used data from household-level surveys as well as administrative reports of immunization coverage. Survey data which provided person-level information on immunization were identified and extracted. Major multi-country survey programs included in the analysis include the Demographic and Health Surveys (DHS),[1] Multiple Indicator Cluster Surveys (MICS),[2] Reproductive Health Surveys (RHS),[3] Living Standards Measurement Study (LSMS) surveys,[4] and World Health Surveys (WHS).[5] We also conducted a comprehensive search of the Global Health Data Exchange (GHDx),[6] as well as

targeted internet searches and review of Ministry of Health websites, to identify national surveys and other multi-country survey programs.

Administrative estimates of immunization coverage were obtained from the Joint Reporting Process,[7] through which the World Health Organization (WHO) and UNICEF collates annual estimates of immunization coverage reported UN member states. These immunization coverage estimates are separate from those synthesized by WHO, and are calculated by dividing the number of doses of a given vaccine delivered to the target population (i.e., children aged 12 to 23) by the number of individuals in that target population.

We excluded all data sources that were not nationally representative or had high levels of missingness. We applied survey weights based on survey sampling frames whenever they were available to generate weighted national estimates of vaccination coverage accompanied by estimates of standard error (SE). Estimates of SE, as well as sample sizes, were used to calculate uncertainty, as described below. Any point estimates with sample sizes less than 50 were reviewed to ensure that they were not substantive outliers and would otherwise have an undue influence on our analysis.

Modeling strategy

Data processing

Age splitting

Most household surveys collect information on maternal and child health (MCH) indicators for children under 5 and/or mothers who gave birth within five years prior to the time of survey. To maximize data use for our model, we included immunization data for children aged 12 to 59 at the time of survey. Children younger than 12 months of age were excluded to minimize the influence of potentially censored observations. For each vaccine, coverage estimates were assigned to birth-cohort years based on a child's age prior to the time of survey: we used responses recorded for children aged 12 to 23 months for immunization coverage for one year prior to the time of survey, children aged 24 to 35 months for coverage two years prior to the time of survey, and so forth.

Age-specific estimates are easily computed from individual-level microdata, but many published reports and survey summaries present data in broader age aggregates (e.g., DPT3 coverage for children aged 12 to 35 months). To standardize these age groups, we applied an age-splitting model used in the GBD study,[8] as well as analyses that generated smoking and obesity prevalence by age group.[9,10]

Using surveys with microdata as the reference, we used the following model to generate standardized age group-specific estimates of immunization coverage:

$$\tilde{P}_{a,c,t,k} = P^{a+x}_{a,c,t,k} \frac{P_{a,c,t,j}}{P^{a+x}_{a,c,t,j}}$$

where $\tilde{P}_{a,c,k}$ is the adjusted estimate of coverage for target age group a in country c and year t of survey k; and $P^{a+x}_{a,c,k}$ is coverage reported from survey k, for country c in year t for the age group spanning age a to age $(a + x)$. The ratio of coverage between the target age group and broader age group from a survey j with microdata from the same country-year was used to split data from survey k. Surveys to be split

were ideally matched with DHS or MICS surveys. If microdata were not available for the same year, ratios within five years of the survey that required age-splitting were applied.

Bias adjustments

Intervention coverage estimates based on administrative sources can be biased, yet the direction and magnitude of such biases are not universal. Some studies show that immunization coverage estimates from administrative data sources are systematically higher than those of survey-based estimates,[11] while other studies show that bias directionality is more heterogeneous.[12] Such biases may arise for a number of reasons, including discrepancies in the accurate reporting of services or interventions provided (e.g., number of vaccine doses administered) and target population (e.g., number of children in need of vaccines), as well as capturing these data in a timely manner from both public and private-sector facilities and health care providers.

For immunization coverage, we view individual-level data collected through population health surveys as the most accurate and least biased source of information of vaccination coverage, particularly for geographies with incomplete health information systems. We thus used vaccination coverage estimates from household surveys to calculate country-specific adjustment factors:

$$logit(P_{s,\,c,t}) = \beta_0 + \beta_1 logit(\tilde{P}_{a,\,c,t}) + \sum_{k=2}^{2+B} \beta_k S_k + \varepsilon_{c,t}$$

where $P_{s,\,c,t}$ is the survey-based estimate for immunization coverage (s) in country c for year t; $\tilde{P}_{a,\,c,t}$ is the administrative estimate for coverage in country c in year t; S_k is a spline basis used to capture the secular trend in coverage; β_1 is the estimated adjustment factor used to correct for the administrative bias; and ε is the error term for country c in year t.

Administrative estimates of immunization also may be subject to an additional bias from participation in performance-based health system support programs, such as the Gavi Immunization Services Support Program (Gavi ISS). It has previously been demonstrated that administrative estimates from participant countries are biased linearly with the number of year enrolled in the program.[13] To correct for this bias, we performed an additional bias adjustment on immunization coverage:

$$logit(P_{s,c,t}) = \beta_0 + \beta_1 logit(P_{a,c,t}) + \beta_2 T_{c,t}^{g} + \alpha_c + \varepsilon_{c,t}$$

where $P_{s,t}$is the survey-based estimate for immunization coverage (s) for country c in year t; $P_{a,t}$is the corresponding administrative coverage, T_t^g is the number of years of enrollment in the Gavi ISS program by year t; α_c is the country-specific random intercept to capture country-specific variation; β_2 is the estimated adjustment factor used to correct for the Gavi bias by the number of years of participation; and ε is the error term for country c in year t.

To quantify uncertainty for bias-adjusted estimates from the mixed-effects models described above, we calculated prediction error, $\widehat{PE}$, as follows:

$$\widehat{PE} = X^2 var(\hat{\beta})$$

where $var(\hat{\beta})$ is the variance for the estimated fixed-effects coefficient of the adjustment factor and X is the independent variable. Proper estimation of prediction errors is crucial as the data synthesis procedure, Gaussian process regression (GPR) (as described in the subsequent section), accounts for uncertainty from point estimates and bias adjustments when generating fitted values. More weight is given to data with less uncertainty. Prediction errors estimated from the bias adjustment were incorporated into the data variance and propagated through the GPR step to obtain estimates of coverage and uncertainty intervals (UIs).

To assess the accuracy of our estimates in the bias adjustment, we performed cross-validation analyses by randomly holding out 20% of the sample and, if available, the corresponding administrative estimates for the given indicator of the same country and year, 10 separate times. We computed the average root mean squared errors (RMSE) across each country. Error in the bias adjustments was calculated as the mean difference between the adjusted administrative estimate for a given country, year, and corresponding survey-level estimates (which were considered the “gold-standard”).

Trend estimation

We used a spatiotemporal Gaussian process regression (ST-GPR) to synthesize point estimates from multiple data sources and derive a complete time series for each vaccine. This method has been used extensively in GBD and related studies, and accounts for uncertainty pertaining to each point estimate while borrowing strength across geographic space and time.[10, 11,15,16] Briefly, we assumed the Gaussian process was defined by a mean function *m*(•) and covariance function *Cov*(•).

We estimated the mean function using a two-step approach. Specifically, $m_c(t)$ can be expressed as:

$$m_c(t) = X\beta + h(r_{c,t})$$

where $X\beta$ is a linear model and $h(r_{c,t})$ is a smoothing function for the residuals; and $r_{c,t}$ is derived from the linear model. The following linear model was used to model DPT3, measles, and polio coverage:

$$logit(P_{c,t}) = \beta_0 + \beta_1 \mathrm{HAQ_{c,t}} + \alpha_c + \gamma_{R[c]} + \omega_{\mathrm{SR[c]}} + \varepsilon_{c,t}$$

where $P_{c,t}$ is vaccination coverage for country c year t; $HAQ_{c,t}$ is value of the Healthcare Access and Quality Index[15] for country c and year t; α_c, $\gamma_{R[c]}$, and $\omega_{\mathrm{SR[c]}}$ are country, region, and super-region random intercepts, respectively. These estimates were then modeled through ST-GPR.

Random draws of 1,000 samples were obtained from the distributions above for every country for a given vaccine. Ninety-five percent uncertainty intervals were calculated by taking the ordinal 25th and 975th draws from the sample distribution.

To assess the accuracy of our modeled estimates, we performed cross-validation analyses using a knockout structure as previously described[16]. ST-GPR hyperparameters were selected on models that minimized the overall root-mean squared error (RMSE) of the model across a set of 10 knockouts.

References

1 Measure DHS: Demographic and Health Surveys. http://www.measuredhs.com (accessed Aug 11, 2015).

2 UNICEF Stat. Monit. Multiple Indicator Cluster Survey (MICS). http://www.unicef.org/statistics/index_24302.html (accessed Aug 11, 2015).

3 Cent. Dis. Control Prev. Reproductive Health Surveys (RHS). http://www.cdc.gov/reproductivehealth/Global/surveys.htm (accessed Aug 11, 2015).

4 World Bank. Living Standard Measruement Studies (LSMS). http://go.worldbank.org/UK1ETMHBN0 (accessed Aug 11, 2015).

5 WHO Multi-Ctry. Stud. Data Arch. World Health Survey (WHS). http://apps.who.int/healthinfo/systems/surveydata/index.php/catalog/whs/about (accessed Aug 11, 2015).

6 IHME GHDx. Global Helath Data Exchange. http://ghdx.healthdata.org/ (accessed Aug 11, 2015).

7 WHO | WHO/UNICEF Joint Reporting Process. WHO. http://www.who.int/immunization/monitoring_surveillance/routine/reporting/reporting/en/ (accessed Aug 17, 2015).

8 Prospective Studies Collaboration. Age-specific relevance of usual blood pressure to vascular mortality: a meta-analysis of individual data for one million adults in 61 prospective studies. *The Lancet* 2002; **360**: 1903–13.

9 Ng M, Freeman MK, Fleming TD, *et al.* Smoking Prevalence and Cigarette Consumption in 187 Countries, 1980-2012. *JAMA* 2014; **311**: 183.

10 Ng M, Fleming T, Robinson M, *et al.* Global, regional, and national prevalence of overweight and obesity in children and adults during 1980–2013: a systematic analysis for the Global Burden of Disease Study 2013. *The Lancet* 2014; **384**: 766–81.

11 Murray CJ, Shengelia B, Gupta N, Moussavi S, Tandon A, Thieren M. Validity of reported vaccination coverage in 45 countries. *The Lancet* 2003; **362**: 1022–7.

12 Haddad S, Bicaba A, Feletto M, Fournier P, Zunzunegui MV. Heterogeneity in the validity of administrative-based estimates of immunization coverage across health districts in Burkina Faso: implications for measurement, monitoring and planning. *Health Policy Plan* 2010; **25**: 393–405.

13 Lim SS, Stein DB, Charrow A, Murray CJ. Tracking progress towards universal childhood immunisation and the impact of global initiatives: a systematic analysis of three-dose diphtheria, tetanus, and pertussis immunisation coverage. *The Lancet* 2008; **372**: 2031–46.

14 Wang H, Liddell CA, Coates MM, *et al.* Global, regional, and national levels of neonatal, infant, and under-5 mortality during 1990–2013: a systematic analysis for the Global Burden of Disease Study 2013. *The Lancet* 13; **384**: 957–79.

15 Barber RM, Fullman N, Sorensen RJD, *et al.* Healthcare Access and Quality Index based on mortality from causes amenable to personal health care in 195 countries and territories, 1990?2015: a novel analysis from the Global Burden of Disease Study 2015. *The Lancet* 2017; published online May. DOI:10.1016/S0140-6736(17)30818-8.

16 Foreman KJ, Lozano R, Lopez AD, Murray CJ. Modeling causes of death: an integrated approach using CODEm. *Popul Health Metr* 2012; **10**: 1.

3.8.1 UHC Index – Antenatal Care SDG Capstone Appendix

Flowchart

3.8.1 UHC – ANC1/ANC4 Coverage Estimation Flowchart

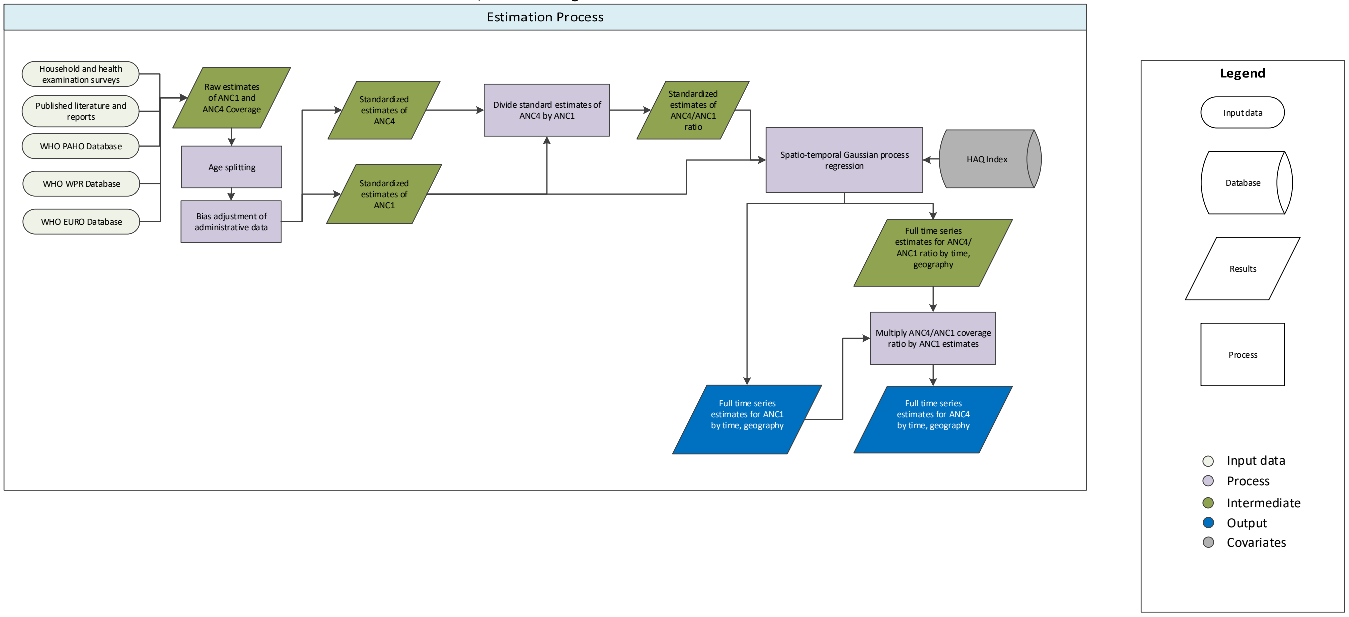

Input data & Methodological summary

Indicator definition

This modeling strategy pertains to the composite universal health coverage (UHC) index (Indicator 3.8.1) and specifically the estimation of antenatal care (ANC), as defined by the proportion of women who attended at least one ANC visit (ANC1) and/or at least four ANC visits (ANC4) for previous births, as provided by a skilled attendant.

Indicator 3.8.1

As a component of SDG Goal 3. Ensure healthy lives and promote well-being for all at all ages, SDG Target 3.8, achieve universal health coverage, including financial risk protection, access to quality essential health-care services and access to safe, effective, quality and affordable essential medicines and vaccines for all, is measured using SDG Indicator 3.8.1, ANC1 and ANC4 coverage.

Input data

For the present analysis, we used individual-level microdata from population health surveys and tabulated survey report data on skilled ANC1 and ANC4. As defined by the World Health Organization (WHO), ANC is considered attended by a skilled health professional when a doctor, nurse, or trained midwife are in attendance.[1]

Survey data which provided individual-level data, and specifically among female respondents, were identified and extracted. Major multi-country survey programs included in the analysis include the Demographic and Health Surveys (DHS),[1] Multiple Indicator Cluster Surveys (MICS),[2] Reproductive Health

Surveys (RHS),[3] Living Standards Measurement Study (LSMS) surveys,[4] and World Health Surveys (WHS).[5] We also conducted a comprehensive search of the Global Health Data Exchange (GHDx),[6] as well as targeted internet searches and review of Ministry of Health websites, to identify national surveys and other multi-country survey programs. In addition, we utilized tabulated report data from regional WHO databases, when available, including the PAHO[7], WHO WPR[8], and the WHO European Health for All databases[9].

We excluded all data sources that were not nationally representative or had high levels of missingness. We applied survey weights based on survey sampling frames whenever they were available to generate weighted national estimates of ANC1 and ANC4 coverage accompanied by estimates of standard error (SE). Estimates of SE, as well as sample sizes, were used to calculate uncertainty, as described below. Any point estimates with sample sizes less than 50 were reviewed to ensure that were not substantive outliers and would otherwise have an undue influence on our analysis.

Due to potential bias in recall, we limited our analysis to women who gave birth up to five years prior to the time of survey; due to data limitations, we used a limit of up to two years for some surveys. We also had to standardize the definition of "skilled health professional" across countries, which varied by differences in quality of training or health professional roles. For this analysis, doctors, nurses, and midwives were included as our foundational definition for skilled ANC, and we extended this to include country-specific medical staff based on the number of years of training they received and/or their comparable ability to intervene in an emergency situation (e.g., clinical officers). Care received during an ANC visit by traditional health personnel was not considered a skilled ANC visit.

Modeling strategy

Data processing

Age splitting

Most household surveys collect information on maternal and child health (MCH) indicators for children under 5 and/or mothers who gave birth within five years prior to the time of survey. To maximize data use for our model, we included ANC information for children aged 12 to 59 at the time of survey. Children younger than 12 months of age were excluded to minimize the influence of potentially censored observations. ANC coverage estimates were assigned to birth-cohort years based on a child's age prior to the time of survey: we used responses recorded for children aged 12 to 23 months for ANC coverage for one year prior to the time of survey, children aged 24 to 35 months for coverage two years prior to the time of survey, and so forth.

Age-specific estimates are easily computed from individual-level microdata, but many published reports and survey summaries present data in broader age aggregates (e.g., ANC coverage for children aged 12 to 35 months). To standardize these age groups, we applied an age-splitting model used in the GBD study,[10] as well as analyses that generated smoking and obesity prevalence by age group.[11,12]

Using surveys with microdata as the reference, we used the following model to generate standardized age-group-specific estimates for ANC:

$$\tilde{P}_{a,c,t,k} = P^{a+x}_{a,c,t,k} \frac{P_{a,c,t,j}}{P^{a+x}_{a,c,t,j}}$$

where $\tilde{P}_{a,c,k}$ is the adjusted estimate of coverage for target age group a in country c and year t of survey k; and $P^{a+x}_{a,c,k}$ is coverage reported from survey k, for country c in year t for the age group spanning age a to age $(a + x)$. The ratio of coverage between the target age group and broader age group from a survey j with microdata from the same country-year was used to split data from survey k. Surveys to be split were ideally matched with DHS or MICS surveys. If microdata were not available for the same year, ratios within five years of the survey that required age-splitting were applied.

Bias adjustments

Intervention coverage estimates based on administrative sources can be biased, yet the direction and magnitude of such biases are not universal. Some studies show that coverage estimates from administrative data sources are systematically higher than those of survey-based estimates,[13] while other studies show that bias directionality is more heterogeneous.[14] Such biases may arise for a number of reasons, including discrepancies in the accurate reporting of services or interventions provided (e.g., number of ANC visits) and target population (e.g., number of children born), as well as capturing these data in a timely manner from both public and private-sector facilities and health care providers.

For ANC, we view individual-level data collected through population health surveys as the most accurate and least biased source of information, particularly for geographies with incomplete health information systems. We thus used ANC coverage estimates from household surveys to calculate country-specific adjustment factors:

$$logit(P_{s,\,c,t}) = \beta_0 + \beta_1 logit(\tilde{P}_{a,\,c,t}) + \sum_{k=2}^{2+B} \beta_k S_k + \varepsilon_{c,t}$$

where $P_{s,\,c,t}$ is the survey-based estimate for ANC coverage (s) in country c for year t; $\tilde{P}_{a,\,c,t}$ is the administrative estimate for coverage in country c in year t; S_k is a spline basis used to capture the secular trend in coverage; β_1 is the estimated adjustment factor used to correct for the administrative bias; and ε is the error term for country c in year t.

To quantify uncertainty for bias-adjusted estimates from the mixed-effects models described above, we calculated prediction error, $\widehat{PE}$, as follows:

$$\widehat{PE} = X^2 var(\hat{\beta})$$

where $var(\hat{\beta})$ is the variance for the estimated fixed-effects coefficient of the adjustment factor and X is the independent variable. Proper estimation of prediction errors is crucial as the data synthesis procedure, Gaussian process regression (GPR) (as described in the subsequent section), accounts for uncertainty from point estimates and bias adjustments when generating fitted values. More weight is given to data with less uncertainty. Prediction errors estimated from the bias adjustment were incorporated into the data variance and propagated through the GPR step to obtain estimates of ANC coverage and uncertainty intervals (UIs).

To assess the accuracy of our estimates in the bias adjustment, we performed cross-validation analyses by randomly holding out 20% of the sample and, if available, the corresponding administrative estimates for the given indicator of the same country and year, 10 separate times. We computed the average root mean squared errors (RMSE) across each country. Error in the bias adjustments was calculated as the mean difference between the adjusted administrative estimate for a given country, year, and corresponding survey-level estimates (which were considered the "gold-standard").

Trend estimation

We used a spatiotemporal Gaussian process regression (ST-GPR) to synthesize point estimates from multiple data sources and derive a complete time series for ANC coverage. This method has been used extensively in GBD and related studies, and accounts for uncertainty pertaining to each point estimate while borrowing strength across geographic space and time.[10, 11,15,16] Briefly, we assumed the Gaussian process was defined by a mean function *m*(•) and covariance function *Cov*(•).

We estimated the mean function using a two-step approach. Specifically, $m_c(t)$ can be expressed as:

$$m_c(t) = X\beta + h(r_{c,t})$$

where $X\beta$ is a linear model and $h(r_{c,t})$ is a smoothing function for the residuals; and $r_{c,t}$ is derived from the linear model. The following linear model was used for the estimation of ANC indicators:

$$logit(P_{c,t}) = \beta_0 + \beta_1 \mathrm{HAQ_{c,t}} + \alpha_c + \gamma_{R[c]} + \omega_{\mathrm{SR[c]}} + \varepsilon_{c,t}$$

where $P_{c,t}$ is ANC coverage for country c year t; $HAQ_{c,t}$ is value of the Healthcare Access and Quality Index[16] for country c and year t; α_c, $\gamma_{R[c]}$, and $\omega_{\mathrm{SR[c]}}$ are country, region, and super-region random intercepts, respectively. These estimates were then modeled through ST-GPR.

By definition, point estimates from a given survey-year for ANC4 cannot exceed ANC1. To ensure definitional consistency for levels of ANC1 and ANC4 coverage, we estimated the coverage of ANC4 by first calculating the ratio of ANC4/ANC1 by survey-year, modeling the ratio of ANC4/ANC1 through ST-GPR, and subsequently multiplying out by the final estimated coverage of ANC4.

Random draws of 1,000 samples were obtained from the distributions above for every country for a given vaccine. Ninety-five percent uncertainty intervals were calculated by taking the ordinal 25th and 975th draws from the sample distribution.

To assess the accuracy of our modeled estimates, we performed cross-validation analyses using a knockout structure as previously described[17]. ST-GPR hyperparameters were selected on models that minimized the overall root-mean squared error (RMSE) of the model across a set of 10 knockouts.

References

1 WHO Indicator and Measurement Registry (WHO IMR). WHO IMR. http://apps.who.int/gho/indicatorregistry/App_Main/indicator_registry.aspx (accessed Aug 11, 2015).

2 Measure DHS: Demographic and Health Surveys. http://www.measuredhs.com (accessed Aug 11, 2015).

3 UNICEF Stat. Monit. Multiple Indicator Cluster Survey (MICS). http://www.unicef.org/statistics/index_24302.html (accessed Aug 11, 2015).

4 Cent. Dis. Control Prev. Reproductive Health Surveys (RHS). http://www.cdc.gov/reproductivehealth/Global/surveys.htm (accessed Aug 11, 2015).

5 World Bank. Living Standard Measruement Studies (LSMS). http://go.worldbank.org/UK1ETMHBN0 (accessed Aug 11, 2015).

6 **WHO Multi-Ctry. Stud. Data Arch. World Health Survey (WHS). http://apps.who.int/healthinfo/systems/surveydata/index.php/catalog/whs/about (accessed Aug 11, 2015).**1 Measure DHS: Demographic and Health Surveys. http://www.measuredhs.com (accessed Aug 11, 2015).

2 UNICEF Stat. Monit. Multiple Indicator Cluster Survey (MICS). http://www.unicef.org/statistics/index_24302.html (accessed Aug 11, 2015).

3 Cent. Dis. Control Prev. Reproductive Health Surveys (RHS). http://www.cdc.gov/reproductivehealth/Global/surveys.htm (accessed Aug 11, 2015).

4 World Bank. Living Standard Measruement Studies (LSMS). http://go.worldbank.org/UK1ETMHBN0 (accessed Aug 11, 2015).

5 WHO Multi-Ctry. Stud. Data Arch. World Health Survey (WHS). http://apps.who.int/healthinfo/systems/surveydata/index.php/catalog/whs/about (accessed Aug 11, 2015).

6 IHME GHDx. Global Helath Data Exchange. http://ghdx.healthdata.org/ (accessed Aug 11, 2015).

7 PAHO/WHO Data - Home. http://www.paho.org/data/index.php/en/ (accessed June 12, 2017).

8 WPR - Health Information & Intelligence Platform > Data & analytics. http://hiip.wpro.who.int/portal/Dataanalytics.aspx (accessed June 12, 2017).

9 European Health for All Family of Databases (HFA-DB). 2017; published online June 12. http://www.euro.who.int/en/data-and-evidence/databases/european-health-for-all-family-of-databases-hfa-db (accessed June 12, 2017).

10 Prospective Studies Collaboration. Age-specific relevance of usual blood pressure to vascular mortality: a meta-analysis of individual data for one million adults in 61 prospective studies. *The Lancet* 2002; 360: 1903–13.

11 Ng M, Freeman MK, Fleming TD, *et al.* Smoking Prevalence and Cigarette Consumption in 187 Countries, 1980-2012. *JAMA* 2014; **311**: 183.

12 Ng M, Fleming T, Robinson M, *et al.* Global, regional, and national prevalence of overweight and obesity in children and adults during 1980–2013: a systematic analysis for the Global Burden of Disease Study 2013. *The Lancet* 2014; **384**: 766–81.

13 Murray CJ, Shengelia B, Gupta N, Moussavi S, Tandon A, Thieren M. Validity of reported vaccination coverage in 45 countries. *The Lancet* 2003; **362**: 1022–7.

14 Haddad S, Bicaba A, Feletto M, Fournier P, Zunzunegui MV. Heterogeneity in the validity of administrative-based estimates of immunization coverage across health districts in Burkina Faso: implications for measurement, monitoring and planning. *Health Policy Plan* 2010; **25**: 393–405.

15 Wang H, Liddell CA, Coates MM, *et al.* Global, regional, and national levels of neonatal, infant, and under-5 mortality during 1990–2013: a systematic analysis for the Global Burden of Disease Study 2013. *The Lancet* 13; **384**: 957–79.

16 Barber RM, Fullman N, Sorensen RJD, *et al.* Healthcare Access and Quality Index based on mortality from causes amenable to personal health care in 195 countries and territories, 1990?2015: a novel analysis from the Global Burden of Disease Study 2015. *The Lancet* 2017; published online May. DOI:10.1016/S0140-6736(17)30818-8.

17 Foreman KJ, Lozano R, Lopez AD, Murray CJ. Modeling causes of death: an integrated approach using CODEm. *Popul Health Metr* 2012; **10**: 1.

3.8.1 UHC – In-Facility Birth Rate (IFD) SDG Capstone Appendix

Input data & Methodological summary

3.8.1 UHC – In Facility Birth Rate Coverage Estimation Flowchart

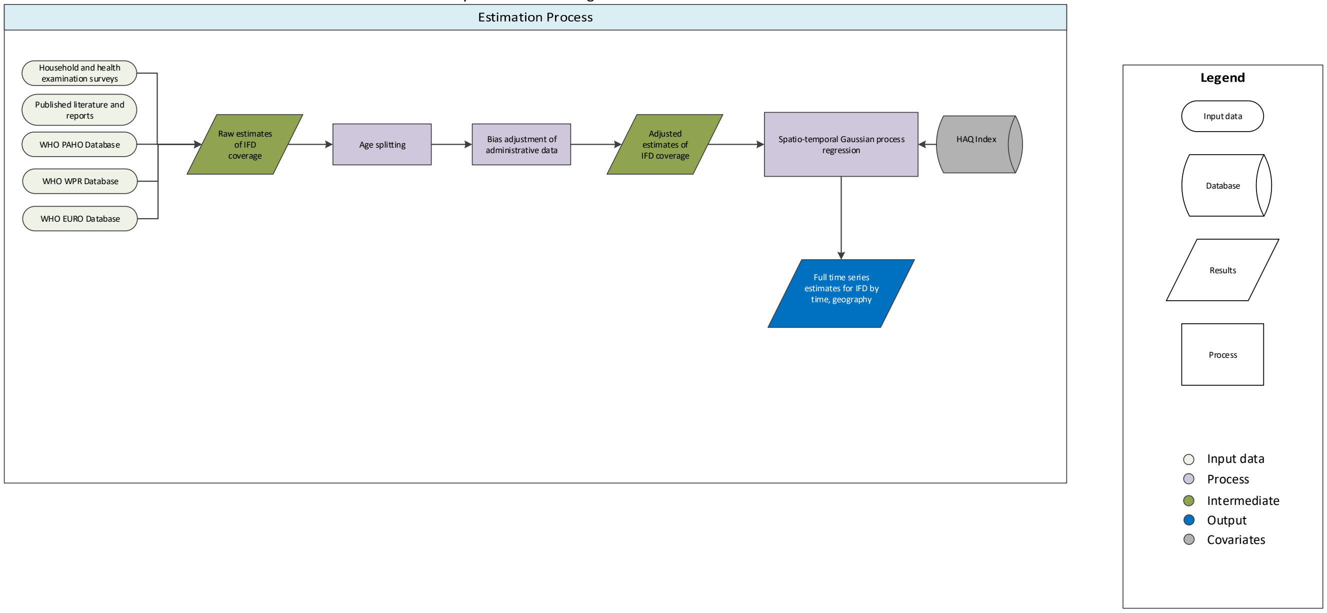

Indicator definition

This modeling strategy pertains to the composite universal health coverage (UHC) index (Indicator 3.8.1) and specifically the estimation of in-facility delivery (IFD), as defined by the proportion of births that were delivered in a health facility.

Indicator 3.8.1

As a component of SDG Goal 3. Ensure healthy lives and promote well-being for all at all ages, SDG Target 3.8, achieve universal health coverage, including financial risk protection, access to quality essential health-care services and access to safe, effective, quality and affordable essential medicines and vaccines for all, is measured using SDG Indicator 3.8.1, in-facility birth rate (IFD).

Input data

Our study included data from household-level surveys as well as administrative reports of in-facility delivery (IFD), defined by WHO as the proportion of births in a given year delivered in a health facility.[1] Survey data which provided person-level information were identified and extracted. Major multi-country survey programs included in the analysis include the Demographic and Health Surveys (DHS)[1], the Multiple Indicator Cluster Surveys (MICS)[2], the Reproductive Health Surveys (RHS)[3], the Living Standards Measurement Studies[4], and the World Health Surveys (WHS)[5]. In additional, a comprehensive search was performed on the Global Health Data Exchange (GHDx)[6], as well as a targeted Google search and a search on the websites of national ministries of health, to identify national surveys and smaller multi-country

surveys. In addition we utilized administrative estimates from regional WHO databases, when available, including the PAHO[7], WHO WPR[8], and the WHO European Health for All databases[9].

We excluded all data sources that were not nationally representative or had high levels of missingness. We applied survey weights based on survey sampling frames whenever they were available to generate weighted national estimates of IFD coverage accompanied by estimates of standard error (SE). Estimates of SE, as well as sample sizes, were used to calculate uncertainty, as described below. Any point estimates with sample sizes less than 50 were reviewed to ensure that there were not substantive outliers and would otherwise have an undue influence on our analysis.

Modeling strategy

Data processing

Age Splitting

Household-level surveys typically collect information about MCH indicators for children under 5 years of age or mothers who have given birth at most 5 years prior to the time of survey. For the sake of utilizing as much data as available, we incorporated estimates for births 0–59 months prior to the survey for analysis. For each indicator, estimates were assigned to a given birth cohort year based on the birth age prior to the time of interview – we used the responses recorded for children aged 12–23 months to estimate coverage 1 year prior to the survey, 24–35 months to estimate coverage 2 years prior to the survey, and so forth.

While information aggregated to these specific age ranges was easily extracted from surveys with person-level data, many published reports and summaries of surveys presented data in broader age groups. We disaggregated these data into the age grouping of interest in this study by applying a splitting model previously used in the Global Burden of Diseases, Injuries, and Risk Factors Study (GBD)[10], as well as in a studies estimating global smoking[11] and obesity prevalence[12].

Using surveys that provided person-level data as references, the following model was applied on estimates with the broader age groups. Specifically, let $\tilde{P}_{a,c,k}$ be the adjusted estimate of coverage for a given indicator for the target age group a in country c and year t of survey k. To disaggregate data that were reported in a broader age group, the following formula was used:

$$\tilde{P}_{a,c,t,k} = P_{a,c,t,k}^{a+x} \frac{P_{a,c,t,j}}{P_{a,c,t,j}^{a+x}}$$

Where $P_{a,c,k}^{a+x}$ denotes the coverage reported from survey k, for country c in year t, but of the age group spanning age a to age $(a + x)$. The ratio of coverage between the age group of interest and the broader age group from a survey j with person-level data from the same country and year was used to split data from survey k. Surveys to be split were ideally matched with DHS or MICS surveys. If person-level data were not available for the same year, data within five years to be split were used.

Bias adjustments

Administrative estimates of IFD are most typically produced using data gathered from supply-side registries. The quality and accuracy of the data therefore depends on the completeness of the nation's health information system.[13] Previous studies have reported that administrative reports of MCH coverage indicators tend to be biased.[8,12,13]

To reduce the impact of these biases on the final results, we performed adjustments on administrative data to account for overall systematic error. Using mixed effects models, we compared administrative data and survey data to derive appropriate adjustment ratios:

$$logit(P_{s,\,c,t}) = \beta_0 + \beta_1 logit(\tilde{P}_{a,\,c,t}) + \varepsilon_{c,t}$$

where $P_{s,\,c,t}$ is the survey-based coverage for a specific indicator for country c in year t, $\tilde{P}_{a,\,c,t}$ is the administrative coverage for country *c* in year *t*, β_1 is the estimated adjustment factor used to correct for the administrative bias.

To assess the accuracy of our estimates in the bias adjustment, we performed cross-validation analyses by randomly holding out 20% of the sample and, if available, the corresponding administrative estimates for the given indicator of the same country and year, 10 separate times. We computed the average root mean squared errors (RMSE) across each country. Error in the bias adjustments was calculated as the mean difference between the adjusted administrative estimate for a given country, year, and corresponding survey-level estimates (which were considered the "gold-standard").

Trend estimation

We used a spatiotemporal Gaussian process regression (ST-GPR) to synthesize information from the various data sources in order to derive a complete time series for each indicator for all countries. This method has been used extensively in other studies to combine information from different sources, taking into account uncertainty for each data point as well as to interpolate nonlinear trends by borrowing strength across geographic space and time.[9-11] Briefly, we assumed the Gaussian process was defined by a mean function *m*(•) and covariance function *Cov*(•).

We estimated the mean function using a two-step approach. Specifically, $m_c(t)$ can be expressed as:

$$m_c(t) = X\beta + h(r_{c,t})$$

where $X\beta$ is a linear model and $h(r_{c,t})$ is a smoothing function for the residuals; and $r_{c,t}$ is derived from the linear model. The following linear model was used for the estimation of ANC indicators:

$$logit(P_{c,t}) = \beta_0 + \beta_1 \mathrm{HAQ_{c,t}} + \alpha_c + \gamma_{R[c]} + \omega_{\mathrm{SR[c]}} + \varepsilon_{c,t}$$

where $P_{c,t}$ is IFD coverage for country c year t; $HAQ_{c,t}$ is value of the Healthcare Access and Quality Index[15] for country c and year t; α_c, $\gamma_{R[c]}$, and $\omega_{\mathrm{SR[c]}}$ are country, region, and super-region random intercepts, respectively. These estimates were then modeled through ST-GPR.

Random draws of 1,000 samples were obtained from the distributions above for every country for a given vaccine. Ninety-five percent uncertainty intervals were calculated by taking the ordinal 25th and 975th draws from the sample distribution.

To assess the accuracy of our modeled estimates, we performed cross-validation analyses using a knockout structure as previously described[16]. ST-GPR hyperparameters were selected on models that minimized the overall root-mean squared error (RMSE) of the model across a set of 10 knockouts.

References

1 Measure DHS: Demographic and Health Surveys. http://www.measuredhs.com (accessed Aug 11, 2015).

2 UNICEF Stat. Monit. Multiple Indicator Cluster Survey (MICS). http://www.unicef.org/statistics/index_24302.html (accessed Aug 11, 2015).

3 Cent. Dis. Control Prev. Reproductive Health Surveys (RHS). http://www.cdc.gov/reproductivehealth/Global/surveys.htm (accessed Aug 11, 2015).

4 World Bank. Living Standard Measruement Studies (LSMS). http://go.worldbank.org/UK1ETMHBN0 (accessed Aug 11, 2015).

5 WHO Multi-Ctry. Stud. Data Arch. World Health Survey (WHS). http://apps.who.int/healthinfo/systems/surveydata/index.php/catalog/whs/about (accessed Aug 11, 2015).

6 IHME GHDx. Global Helath Data Exchange. http://ghdx.healthdata.org/ (accessed Aug 11, 2015).

7 PAHO/WHO Data - Home. http://www.paho.org/data/index.php/en/ (accessed June 12, 2017).

8 WPR - Health Information & Intelligence Platform > Data & analytics. http://hiip.wpro.who.int/portal/Dataanalytics.aspx (accessed June 12, 2017).

9 European Health for All Family of Databases (HFA-DB). 2017; published online June 12. http://www.euro.who.int/en/data-and-evidence/databases/european-health-for-all-family-of-databases-hfa-db (accessed June 12, 2017).

10 Prospective Studies Collaboration. Age-specific relevance of usual blood pressure to vascular mortality: a meta-analysis of individual data for one million adults in 61 prospective studies. *The Lancet* 2002; **360**: 1903–13.

11 Ng M, Freeman MK, Fleming TD, *et al.* Smoking Prevalence and Cigarette Consumption in 187 Countries, 1980-2012. *JAMA* 2014; **311**: 183.

12 Ng M, Fleming T, Robinson M, *et al.* Global, regional, and national prevalence of overweight and obesity in children and adults during 1980–2013: a systematic analysis for the Global Burden of Disease Study 2013. *The Lancet* 2014; **384**: 766–81.

13 Murray CJ, Shengelia B, Gupta N, Moussavi S, Tandon A, Thieren M. Validity of reported vaccination coverage in 45 countries. *The Lancet* 2003; **362**: 1022–1027.

14 Haddad S, Bicaba A, Feletto M, Fournier P, Zunzunegui MV. Heterogeneity in the validity of administrative-based estimates of immunization coverage across health districts in Burkina Faso: implications for measurement, monitoring and planning. *Health Policy Plan* 2010; **25**: 393–405.

15 Barber RM, Fullman N, Sorensen RJD, *et al.* Healthcare Access and Quality Index based on mortality from causes amenable to personal health care in 195 countries and territories, 1990?2015: a novel analysis from the Global Burden of Disease Study 2015. *The Lancet* 2017; published online May. DOI:10.1016/S0140-6736(17)30818-8.

16 Foreman KJ, Lozano R, Lopez AD, Murray CJ. Modeling causes of death: an integrated approach using CODEm. *Popul Health Metr* 2012; **10**: 1.

UHC – ART SDG Capstone Appendix

Indicator definition

This modeling strategy encompassed the indicator associated with the universal health coverage index (3.8.1), specifically antiretroviral therapy (ART) coverage.

Indicator 3.8.1

As a component of SDG Goal 3. Ensure healthy lives and promote well-being for all ages, SDG Target 3.8, achieve universal health coverage, including financial risk protection, access to quality essential health-care services and access to safe, effective, quality and affordable essential medicines and vaccines for all, is measured using SDG Indicator 3.8.1, ART Coverage.

Input data

ART Coverage Data

We define ART coverage as the percentage of individuals living with HIV who are receiving ART. This can be broken into two components: the numerator is the number of people receiving ART and the denominator is the number of people living with HIV. Location-, year-, and sex-specific data on the number of individuals receiving ART or the percentage of HIV positive individuals receiving ART were extracted from UNAIDS country files. Whether or not coverage is reported as a count or a percentage varies by year for a given location and sex. This data was estimated by UNAIDS using facility data reported to the WHO by ministries of health as well as data reported by non-profit organizations, private companies, and insurance companies.

Modelling strategy

Full details of the modelling strategy can be found in the GBD 2015 HIV paper published in Lancet HIV. Spectrum, the compartmental model used for estimation of HIV burden, takes ART coverage as an input which informs the initiation of treatment by sub-group within Spectrum. We report the number of individuals found to be on treatment after running Spectrum so that our estimates of the number people receiving treatment are consistent with our estimates of the number of people living with HIV

Tuberculosis

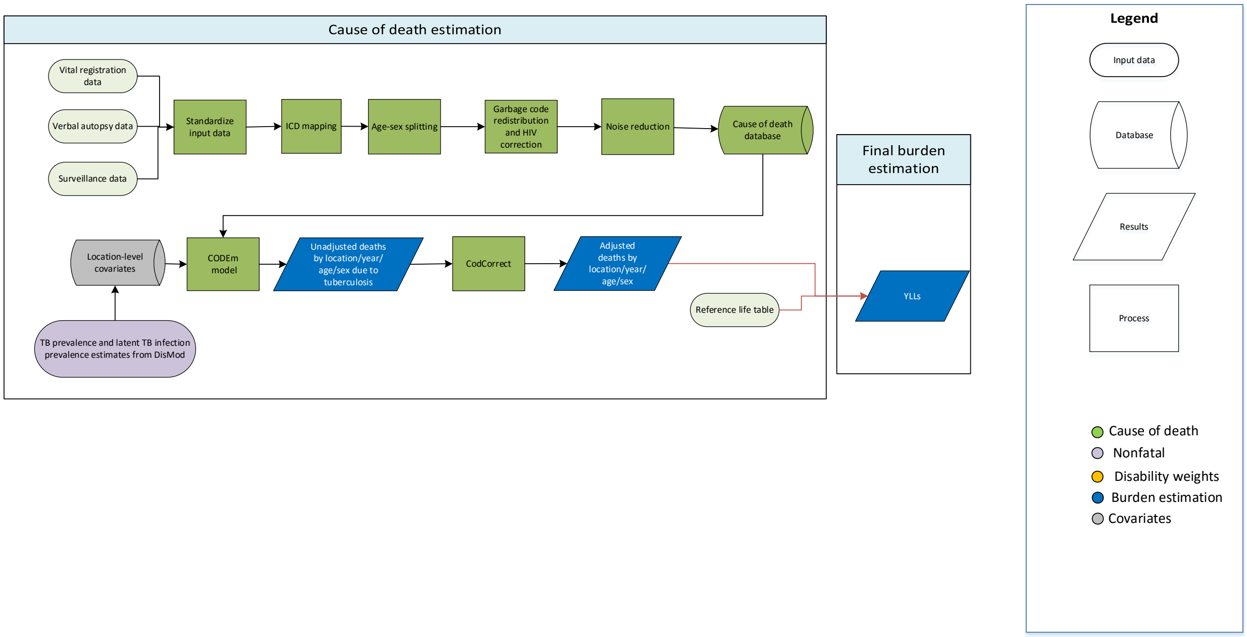

Input data

Input data for modeling tuberculosis mortality among HIV-negative individuals include vital registration, verbal autopsy, and surveillance data. Vital registration data were adjusted for garbage coding (including ill-defined codes, and the use of intermediate causes) following GBD algorithms and misclassified HIV deaths (i.e., HIV deaths being assigned to other underlying causes of death such as tuberculosis or diarrhea because of stigma or misdiagnosis). This correction was done based on examining changes in the age pattern of diseases over time.

Verbal autopsy data in countries with age-standardized HIV prevalence greater than 5% were removed because of a high probability of misclassification, as verbal autopsy studies have poor validity in distinguishing HIV deaths from HIV-TB deaths.

Modeling strategy

We changed the modeling strategy of tuberculosis in GBD2016 by first modeling prevalence of disease and prevalence of latent infection which were then used as covariates in the CODEm model. We dropped the health system access covariate and replaced it by the newly developed Healthcare Access and Quality Index covariate. We also added the adult underweight proportion covariate. Other location-level covariates included in the CODEm model were the same as in GBD 2015: alcohol (liters per capita), diabetes (fasting plasma glucose mmol/L), education (years per capita), lag-distributed income, indoor air pollution, outdoor air pollution, population density, smoking prevalence, sociodemographic status, and a summary exposure variable reflecting the average exposure to all of the risk factors.

Multidrug-resistant tuberculosis, extensively drug-resistant tuberculosis, and drug-sensitive tuberculosis

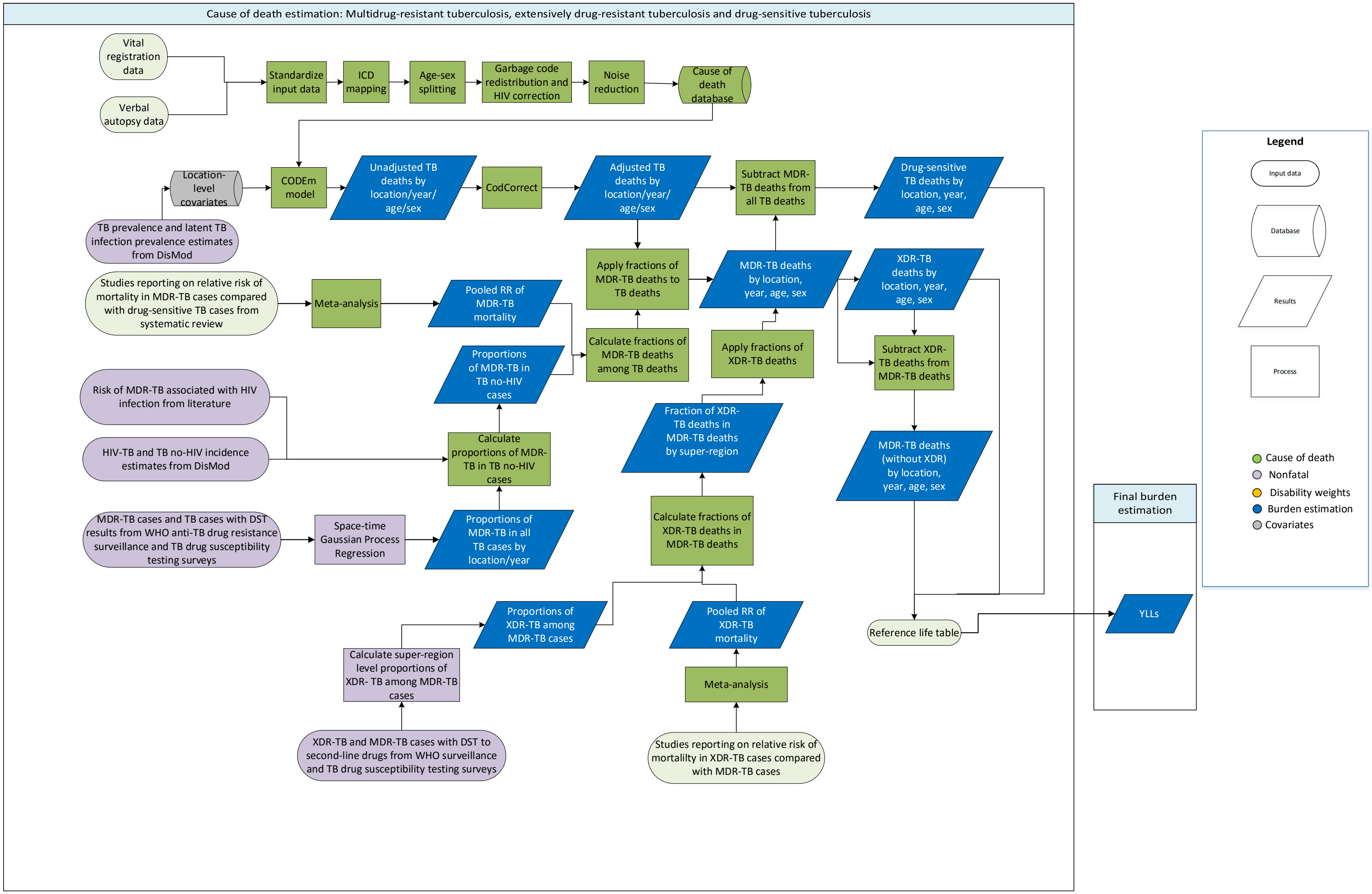

Input data

Input data include: (i) the number of drug-resistant cases by type (multidrug-resistant tuberculosis [MDR-TB], extensively drug-resistant tuberculosis [XDR-TB], all TB cases with a drug sensitivity testing [DST] result for isoniazid and rifampicin, and MDR-TB cases with DST for second-line drugs) from routine surveillance and surveys reported to the World Health Organization, (ii) data from studies (identified through our systematic review) reporting on the relative risk of death in MDR-TB cases compared with non-MDR TB (drug-sensitive TB) cases, and the relative risk of death in XDR-TB cases compared with MDR-TB cases, and (iii) the risk of MDR-TB associated with HIV infection from the literature.[1]

Modelling strategy

We conducted a systematic review and meta-analysis of studies reporting the relative risk of death in MDR-TB cases compared with drug-sensitive TB cases. We also ran a spatiotemporal Gaussian process regression to predict the proportions of MDR-TB cases among all TB cases for all locations and years. We computed the weighted average of the proportions of new and previously treated cases with MDR-TB, and used these as the input data for this regression. We then used the predicted proportions of MDR-TB, along with the HIV-TB and TB no-HIV incidence estimates (from our modeling of non-fatal TB), and the relative risk of MDR-TB associated with HIV infection from the literature[1] to compute the proportions of MDR-TB cases among HIV negative TB cases $\left(P_{MDRnoHIVc,y,a,s}\right)$ by location, year, age, and sex using the following formula:

$$P_{MDRnoHIVc,y,a,s} = \frac{MDR_{c,y}}{\left(1 + \left(RR_{HIV}\frac{HIVTB_{c,y,a,s}}{TBnoHIV_{c,y,a,s}}\right)\right) TBnoHIV_{c,y,a,s}}$$

where $MDR_{c,y}$ is the number of all MDR-TB cases among HIV-positive and HIV-negative individuals by location and year, RR_{HIV} is the relative risk of MDR-TB associated with HIV infection, $HIVTB_{c,y,a,s}$ is the number of HIV-TB incident cases by location, year, age, and sex, and $TBnoHIV_{c,y,a,s}$ is the number of TB no-HIV incident cases by location, year, age, and sex.

We then computed the fraction of MDR-TB deaths among all HIV-negative TB deaths ($D_{MDRnoHIVc,y,a,s}$) using the following formula:

$$D_{MDRnoHIVc,y,a,s} = \frac{P_{MDRnoHIVc,y,a,s}RR_{MDR}}{P_{MDRnoHIVc,y,a,s}RR_{MDR} + 1 - P_{MDRnoHIVc,y,a,s}}$$

where RR_{MDR} is the relative risk of death in MDR-TB cases compared with drug-sensitive TB cases. We then applied the predicted fractions of MDR-TB deaths among HIV-negative TB deaths to our CODEm TB death estimates to generate MDR-TB deaths by location, year, age, and sex. Next, we subtracted MDR-TB deaths from all TB deaths to generate drug-sensitive TB deaths by location, year, age, and sex.

To separate out XDR-TB from MDR-TB, we aggregated the XDR-TB cases and MDR-TB cases (with DST for second-line drugs) up to the super-region level and calculated the super-region level proportions of XDR-TB among MDR-TB cases. Next, we computed the super-region-specific fractions of XDR-TB deaths among all MDR-TB deaths (D_{XDRsr}) using the following formula:

$$D_{XDRsr} = \frac{P_{XDRsr}RR_{XDR}}{P_{XDRsr}RR_{XDR} + 1 - P_{XDRsr}}$$

where P_{XDRsr} is the proportion of XDR-TB among MDR-TB cases for each super-region, and RR_{XDR} is the pooled relative risk of mortality in XDR-TB cases compared with MDR-TB cases. These fractions were then applied to MDR-TB deaths in corresponding countries within the super-regions to produce XDR-TB deaths by location, age, and sex for the most recent year of estimation. We linearly extrapolated XDR-TB mortality rates back assuming the mortality rates were zero in 1992, one year before 1993 when XDR-TB was first recorded in USA surveillance data.[2] Finally, we subtracted XDR-TB deaths from MDR-TB deaths to generate MDR-TB (without extensive drug resistance) deaths by location, year, age, and sex.

Reference

1. Mesfin YM, Hailemariam D, Biadglign S, Kibret KT. Association between HIV/AIDS and multi-drug resistance tuberculosis: a systematic review and meta-analysis. PLoS One. 2014;9(1):e82235.
2. Centers for Disease Control and Prevention (CDC). Extensively Drug-Resistant Tuberculosis --- United States, 1993—2006. MMWR. 2007; 56(11);250-253

Diarrheal diseases

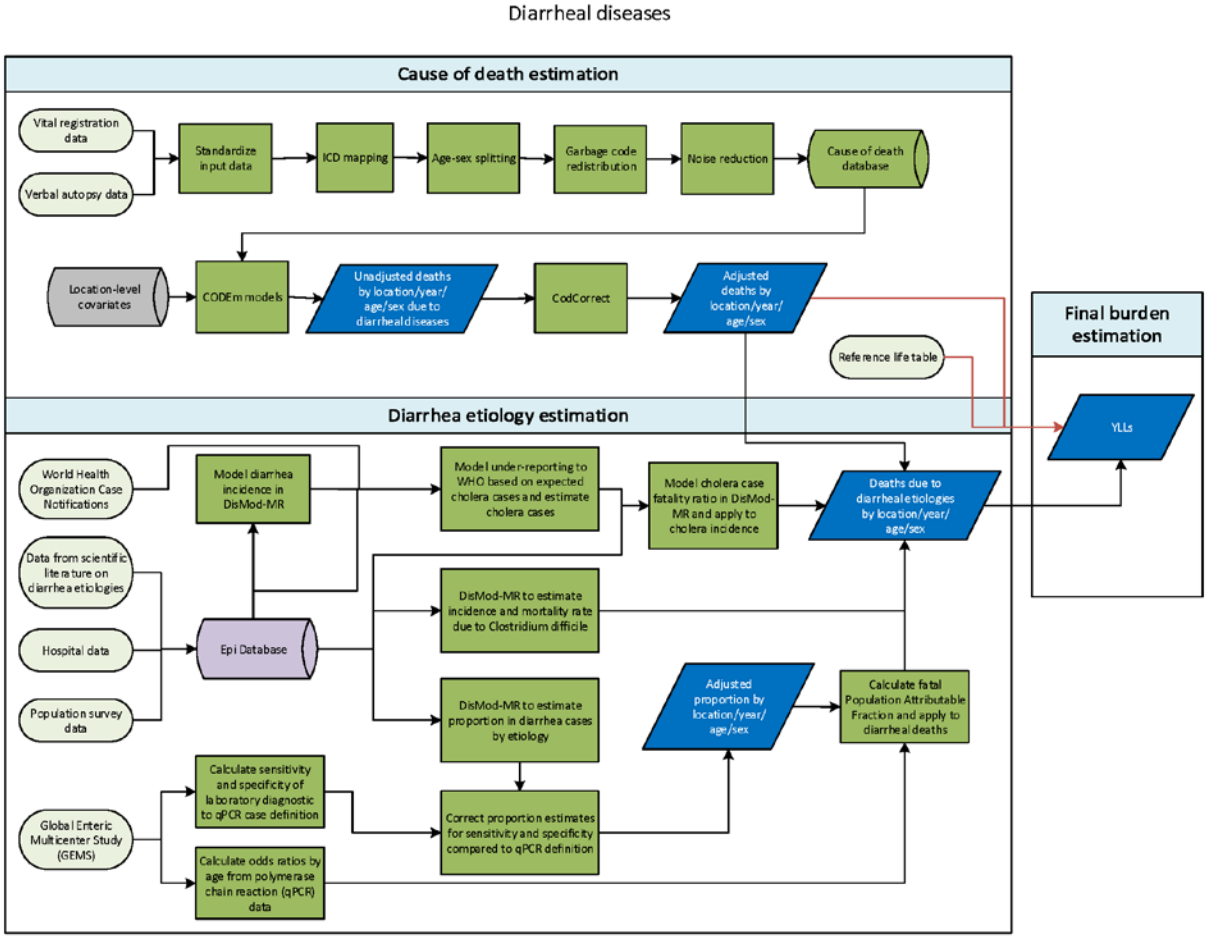

Input data

Cause of death. Diarrheal disease mortality was estimated in CODEm. We estimated diarrhea mortality separately for males and females and for children under 5 years and older than 5 years. We used all available data from vital registration systems, surveillance systems and verbal autopsy (**Table 1**). We checked for and excluded outliers from our data by country or region. We also excluded early neonatal mortality data in the Philippines (1994–1998) and India Civil Registration System data in all states (1986–1995).

Etiologies. We conducted a systematic literature review for the proportion of diarrhea cases that tested positive for each etiology. We updated our review of literature to include studies published between May 2015 and May 2016. Inclusion criteria included diarrhea as the case definition, studies with a sample size of at least 100, and studies with at least one year of follow-up. We excluded studies that reported on diarrheal outbreaks exclusively and those that used acute gastroenteritis with or without diarrhea. We identified 442 studies, of which 36 met our criteria of inclusion and were included. We extracted data points for location, sex, year, and age. We assigned an age range based on the prevalence-weighted mean age of diarrhea in the appropriate year/sex/location if the age of the study participants was not reported.

We used the Global Enteric Multicenter Study (GEMS), a seven-site, case-control study of moderate-to-severe diarrhea in children under 5 years,[1] to calculate odds ratios for the diarrheal pathogens. We analyzed raw data for a systematic reanalysis, representative of the distribution of cases and controls by age and site, of roughly half of the 22,000 original GEMS samples that were tested for the presence of pathogen using quantitative polymerase chain reaction (qPCR).[2]

Modeling strategy

Cause of death. We used country-level covariates to inform our CODEm models. We included covariates for years of education per capita, income per capita, prevalence of undernutrition (weight-for-age, weight-for-height, and height-for-age), population density above 1,000 or below 150 people per square kilometer, sanitation access, safe water access, Socio-Demographic Index, and rotavirus vaccine coverage. We evaluated our diarrheal disease cause of death models using in and out of sample predictive performance.

Etiologies. We estimated diarrheal disease etiologies separately from overall diarrhea mortality using a counterfactual strategy for enteric adenovirus, *Aeromonas*, *Entamoeba histolytica* (amoebiasis), *Campylobacter enteritis*, *Cryptosporidium*, typical enteropathogenic *Escherichia coli* (t-EPEC), enterotoxigenic *Escherichia coli* (ETEC), norovirus, non-typhoidal salmonella infections, rotavirus, and *Shigella*. *Vibrio cholerae* and *Clostridium difficile* were modeled separately.

Diarrheal etiologies are attributed to diarrheal deaths using a counter-factual approach. We calculated a population attributable fraction (PAF) from the proportion of severe diarrhea cases that are positive for each etiology. The PAF represents the relative reduction in diarrhea mortality if there was no exposure to a given etiology. As diarrhea can be caused by multiple pathogens and the pathogens may co-infect, PAFs can overlap and add up to more than 100%. We calculated the PAF from the proportion of severe diarrhea cases that are positive for each etiology. We assumed that hospitalized diarrhea cases are a proxy of severe and fatal cases. We used the following formula to estimate PAF:[4]

$$PAF = Proportion * (1 - \frac{1}{OR})$$

Where *Proportion* is the proportion of diarrhea cases positive for an etiology and *OR* is the odds ratio of diarrhea given the presence of the pathogen.

We dichotomized the continuous qPCR test result using the value of the cycle threshold (Ct) that most accurately discriminated between cases and controls. The Ct values range from 0 to 35 cycles representing the relative concentration of the target gene in the stool sample. A low value indicates a higher concentration of the pathogen while a value of 35 indicates the absence of the target in the sample. We used the lower Ct value when we had multiple Ct values for the cutpoint. The case definition for each pathogen is a Ct value that is below the established cutoff point.

We used a mixed effects conditional logistic regression model to calculate the odds ratio for under 1 year and 1-4 years old for each of our pathogens. The odds ratio for 1-4 years was applied to all GBD age groups over 5 years. There were three pathogen-age odds ratios that were not statistically significant: Aeromonas and Amoebiasis in under 1 year and Campylobacter in 1-4 years. The mean value of the odds ratio was above 1 in all three cases so we transformed the odds ratios for these three exceptions only in log-space such that exponentiated values could not be below 1. The transformation was:

$$Odds\ ratio = exp(log(or) - 1)) + 1$$

We modeled the proportion data using the meta-regression tool DisMod-MR to estimate the proportion of positive diarrhea cases for each separate etiology by location/year/age/sex and to adjust for the covariates.

We used the estimated sensitivity and specificity of the laboratory diagnostic technique used in the GEMS study compared to the qPCR case definition to adjust our proportion before we computed the PAF:[5]

$$Proportion_{True} = \frac{(Proportion_{Observed} + Specificity - 1)}{(Sensitivity + Specificity - 1)}$$

We used this correction to account for the fact that the proportions we used are based on a new test that is not consistent with the laboratory-based case definition (qPCR versus GEMS conventional laboratory testing for pathogens).[15]

Our literature review extracted the proportion of any enteropathogenic *Escherichia coli* (EPEC) without differentiating between typical (tEPEC) and atypical (aEPEC). In order to be consistent with the odds ratios that we obtained, we adjusted our proportion estimates of any EPEC to typical EPEC only. This adjustment was informed by a subset of our literature review that reported both atypical and typical EPEC. We estimated a ratio by super-region of tEPEC to any EPEC and adjusted our proportion estimates accordingly. We found that the majority of EPEC diarrhea cases were positive for atypical EPEC, consistent with other published work.[3]

For *Vibrio cholerae* (cholera), we used the literature review to estimate expected number of cholera cases for each country-year using the incidence of diarrhoea, estimated using DisMod-MR, and the proportion of diarrhoea cases that are positive for cholera. We assigned cholera PAF using odds ratios from the qPCR results to estimate a number of cholera-attributable cases. We compared this expected number of cholera cases to the number reported to the World Health Organization at the country-year level.[6] We modeled the underreporting fraction to correct the cholera case notification data for all countries using health system access and the diarrhoea SEV scalar to predict total cholera cases. We used the age-specific proportion of positive cholera samples in DisMod and our incidence estimates to predict the number of cholera cases for each age/sex/year/location. Finally, we modeled the case fatality ratio of cholera using DisMod-MR and to estimate the number of cholera deaths.

For *C. difficile*, we modeled incidence and mortality in DisMod-MR for each age, sex, year, location. DisMod-MR is a Bayesian meta-regression tool that uses spatio-temporal information as priors to estimate prevalence, incidence, remission, and mortality for *C. difficile* infection. DisMod-MR uses a compartmental model to relate prevalence, incidence, remission, and mortality. We set remission in our model to 1 month.

Table 1. Cause-specific mortality input data.

Type of data	Input data
**Total data sources**	**16,980 site-years**
Vital registration data	15,087 site-years
Surveillance data	877 site-years
Verbal autopsy data	1,016 site-years

References

1 Kotloff KL, Nataro JP, Blackwelder WC, *et al.* Burden and aetiology of diarrhoeal disease in infants and young children in developing countries (the Global Enteric Multicenter Study, GEMS): a prospective, case-control study. *Lancet Lond Engl* 2013; **382**: 209–22.

2 Liu J, Gratz J, Amour C, *et al.* A laboratory-developed TaqMan Array Card for simultaneous detection of 19 enteropathogens. *J Clin Microbiol* 2013; **51**: 472–80.

3 Ochoa TJ, Barletta F, Contreras C, Mercado E. New insights into the epidemiology of enteropathogenic Escherichia coli infection. *Trans R Soc Trop Med Hyg* 2008; **102**: 852–6.

4 Miettinen OS. Proportion of disease caused or prevented by a given exposure, trait or intervention. *Am J Epidemiol* 1974; **99**: 325–32.

5 Reiczigel J, Földi J, Ozsvári L. Exact confidence limits for prevalence of a disease with an imperfect diagnostic test. *Epidemiol Infect* 2010; **138**: 1674–8.

6 World Health Organization. Global Health Observatory data repository: Cholera. 2016. http://apps.who.int/gho/data/node.main.174?lang=en (accessed Aug 25, 2016).

Lower Respiratory Infections

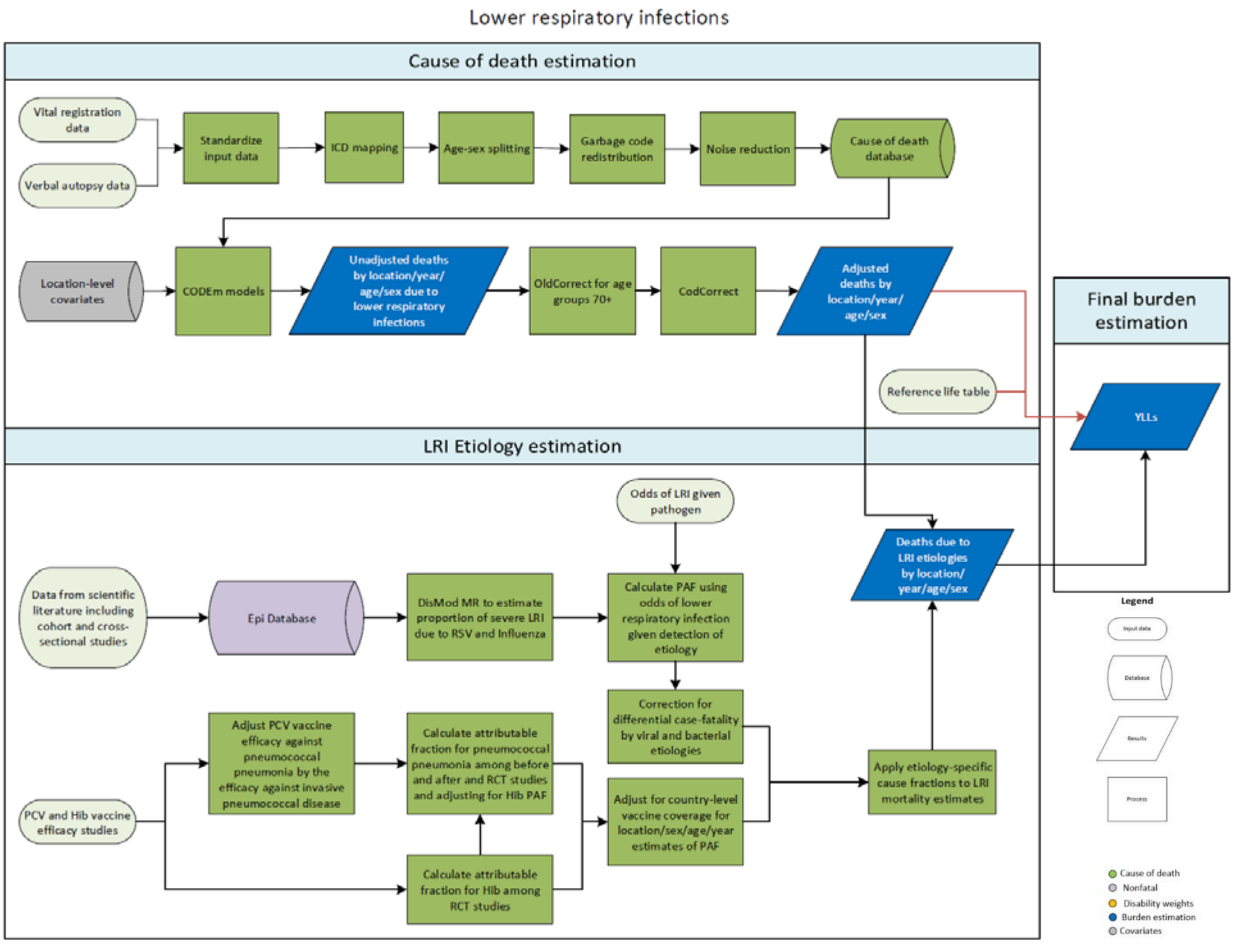

Input data

Cause of deaths. Lower respiratory infection (LRI) mortality was estimated in CODEm. We estimated LRI mortality separately for males and females and for children under 5 years and older than 5 years. We used all available data from vital registration systems, surveillance systems, and verbal autopsy (**Table 1**). We checked for and excluded outliers from our data by country or region. We also excluded ICD9-coded mortality data in Sri Lanka (1982, 1987–1992), ICD9-coded neonatal mortality data in Guatemala (1980, 1981, 1984, 2000–2004), and Civil Registration System data in many Indian states (1986–1995).

Etiologies. We updated our systematic review of scientific literature for the proportion of LRI that tested positive for influenza and respiratory syncytial virus (RSV) to include all data from GBD 2015 and from studies published between May 2015 and May 2016. Inclusion criteria were studies that had a sample size of at least 100, studies that were at least one year in duration, and studies describing lower respiratory infections, pneumonia, or bronchiolitis as the case definition. During our literature review we identified 209 studies, of which 7 met our inclusion criteria and were extracted. We excluded studies that described pandemic H1N1 influenza solely and studies that used influenza-like illness as the case definition. We assigned an age range based on the prevalence-weighted mean age of LRI in the appropriate year/sex/location if the ages of the study participants were not reported.

We also conducted a systematic literature review of studies on the Hib vaccine and PCV effectiveness studies against X-ray-confirmed pneumonia and against pneumococcal and Hib disease until May 2016. For PCV studies, we extracted, if available, the distribution of pneumococcal pneumonia serotypes and the serotypes included in the PCV used in the study. No new studies were identified for GBD 2016. We excluded observational and case-control studies due to implausibly high vaccine efficacy estimates. Hib trial data were exclusively from children <5 years so we did not include the effect of Hib on ages over 5 years of age. PCV trial data are also frequently limited to younger age populations. To understand the contribution of pneumococcal pneumonia in older populations, we also included PCV efficacy studies that used before-after approaches.

Modeling strategy

Cause of death. We used country-level covariates to inform our CODEm models. We included the following covariates in our LRI models: diphtheria-tetanus-pertussis vaccine coverage, years of education per capita, health system access, income per capita, prevalence of children malnutrition (<2 standard deviations below global mean of weight for age), prevalence of exposure to indoor air pollution (solid fuel use), outdoor air pollution level of $PM_{2.5}$, smoking prevalence, pneumococcal conjugate vaccine (PCV) coverage, *Haemophilus influenzae* type B (Hib) vaccine coverage, access to improved water, access to improved sanitation, and Socio-Demographic Index. We evaluated our LRI cause of death models using in and out of sample predictive performance.

Like all models of mortality in GBD, LRI mortality models are single-cause, requiring in effect that the sum of all mortality models must be equal to the all-cause mortality envelope. We correct LRI mortality estimates, and other causes of mortality, by re-scaling them according to the uncertainty around the cause-specific mortality rate. This process is called CoDCorrect and is essential to ensure internal consistency among causes of death. Before CoDCorrect, we also adjust LRI mortality for unreliable estimates due to improper death certification and ICD coding among elderly adults where the underlying cause of death should be Alzheimer's or Parkinson's diseases. This process scales LRI mortality among adult age groups 70+ years into a new envelope without Alzheimer's and Parkinson's. Further details can be found in section 4 of the appendix.

Etiologies. We estimated LRI etiologies separately from overall LRI mortality using two distinct counterfactual modeling strategies to estimate population attributable fractions (PAFs), described in detail below. The PAF represents the relative reduction in LRI mortality if there was no exposure to a given etiology. As LRIs can be caused by multiple pathogens and the pathogens may co-infect, PAFs can overlap and add up to more than 100%. Separate strategies were used for viral- influenza and respiratory syncytial virus (RSV)- and bacterial- *Streptococcus pneumoniae* and *Haemophilus influenzae* type B- etiologies. We did not attribute etiologies to neonatal pneumonia deaths due to a dearth of reliable data in this age group. We calculated uncertainty of our PAF estimates from 1,000 draws of each parameter using normal distributions in log space.

Influenza and RSV. We calculated the population attributable fraction (PAF) from the proportion of severe LRI cases positive for influenza and RSV. We assumed that hospitalized LRI cases are a proxy of severe cases. We used the following formula to estimate PAF:[1]

$$PAF = Proportion * (1-1/OR)$$

Where *Proportion* is the proportion of LRI cases that test positive for influenza or RSV and *OR* is the odds ratio of LRI given the presence of the pathogen. We used an odds ratio of 5.1 (3.19 – 8.14) for influenza and 9.79 (4.98 – 19.27) for RSV from a recently published meta-analysis.[2] These odds ratios are marginally different from those used in GBD 2013.

We modeled the proportion data using the meta-regression tool DisMod-MR to estimate the proportion of LRI cases that are positive for influenza and RSV, separately, by location/year/age/sex. We accounted for study-level covariates in our models such as PCR as the diagnostic technique, studies that investigated RSV or influenza exclusively, and studies from inpatient populations.

As the case-fatality of viral causes of pneumonia is lower than for bacterial causes, we adjusted for differential case-fatality by determining the etiological fractions for mortality attributable to RSV and influenza (**Table 2**). We measured the etiologic fractions by applying a relative case-fatality adjustment based on in-hospital case-fatality, which we coded to specific pneumonia etiologies. Hospital admissions data of this type were limited to data from the USA, Austria, Brazil, and Mexico. We generated the pooled estimate of the case-fatality differential between bacterial (pneumococcus, Hib) and viral etiologies (RSV, influenza) using DisMod-MR.

Pneumococcal pneumonia and Hib. For *Streptococcus pneumoniae* (pneumococcal pneumonia) and *Haemophilus influenzae* type B (Hib), we calculated the population attributable fraction using a vaccine probe design.[3,4] The ratio of vaccine effectiveness against nonspecific pneumonia to pathogen-specific disease represents the fraction of pneumonia cases attributable to each pathogen.

To estimate the PAF for Hib and pneumococcal pneumonia, we calculated the ratio of vaccine effectiveness against nonspecific pneumonia to pathogen-specific pneumonia (Equations 1 and 3). We estimated a study-level estimate of PAF from a meta-analysis of these ratios. To estimate the PAF for Hib, we only used randomized controlled trials because of implausibly high values of vaccine efficacy in case-control studies. To estimate the PAF for pneumococcal pneumonia, we included RCTs and before and after vaccine introduction longitudinal studies.

We adjusted the study-level PAF estimate by vaccine coverage and expected vaccine performance to estimate country- and year-specific PAF values. For pneumococcal pneumonia, we adjusted the PAF by the final Hib PAF estimate and by vaccine serotype coverage. Finally, we used an age distribution of PAF modeled in DisMod to determine the PAF by age. Because of an absence of data describing vaccine efficacy against Hib in children older than two years, we did not attribute Hib to episodes of LRI in ages five years and older.

We used a vaccine probe design to estimate the PAF for pneumococcal pneumonia and (Hib) by first calculating the ratio of vaccine effectiveness against nonspecific pneumonia to pathogen-specific pneumonia at the study level (Equations 1 and 2).[3–5] We then adjusted this estimate by vaccine coverage and expected vaccine performance to estimate country- and year-specific PAF values (Equations 3 and 4).

$$1)\quad HibPAF_{Base} = 1 - \frac{VE_{Pneumonia}}{VE_{Hib}}$$

$$2)\quad PneumoPAF_{Base} = 1 - \frac{VE_{Pneumonia} * (1 - PAF_{Hib} * VE_{Hib\ Optimal})}{VE_{Streptococcus} * Cov_{Serotype}}$$

3) $PAF_{Hib} = PAF_{Base} * \frac{(1-Cov_{Hib}*VE_{Hib\ Optimal})}{(1-PAF_{Base}*Cov_{Hib}*VE_{Hib\ Optimal})}$

4) $PAF_{Pneumo} = \frac{PAF_{Base}*(1-Cov_{PCV}*VE_{PCV\ Optimal})}{(1-PAF_{Hib}*Cov_{Hib}*VE_{Hib\ Optimal})*\left(1-\frac{PAF_{Base}Cov_{PCV}*VE_{PCV\ Optimal}}{(1-PAF_{Hib}*Cov_{Hib}*VE_{Hib\ Optimal})}\right)}$

Where $VE_{Pneumonia}$ is the vaccine efficacy against nonspecific pneumonia, VE_{Hib} is the vaccine efficacy against invasive Hib disease, $VE_{Streptococcus}$ is the vaccine efficacy against serotype-specific pneumococcal pneumonia, $Cov_{serotype}$ is the serotype-specific vaccine coverage for PCV,[6] $VE_{Hib\ Optimal}$ is the Hib effectiveness in the community (0.8)[7], PAF_{Hib} is the final PAF for Hib, Cov_{PCV} is the PCV coverage, Cov_{Hib} is the Hib coverage by country, and $VE_{PCV\ Optimal}$ is the vaccine effectiveness in the community (0.8).[8]

For Hib, we assumed that the vaccine efficacy against invasive Hib disease is the same against Hib pneumonia. For pneumococcal pneumonia, a recent study in adults [9] found that the vaccine efficacy against invasive pneumococcal disease may be significantly higher than against pneumococcal pneumonia. We used this ratio to adjust estimates of vaccine efficacy against invasive pneumococcal disease from other studies. However, recognizing that the study is unique in that it uses a urine antigen test among adults, we added uncertainty around our adjustment using a wide uniform distribution (median 0.65, 0.3-1.0). This has increased the estimates of pneumococcal pneumonia mortality in a meaningful way.

There are no major changes to the cause of death estimation strategy for LRI or its etiologies from GBD 2015 to GBD 2016.

Table 1. Summary of cause-specific mortality modeling input data.

**Type of data**	**Input data**
**Total data sources**	**12,155 site-years**
Vital registration data	10,312 site-years
Surveillance data	928 site-years
Verbal autopsy data	915 site-years

Table 2: **The median values for the ratio of viral to bacterial pneumonia case fatality ratio by age is shown.** These estimates are modeled using hospital-based, ICD-coded admissions and mortality for etiology-specified pneumonia. Values in parentheses represent 95% Uncertainty Interval.

**Age Group**	**Ratio**
Early Neonatal	0.34 (0.19-0.58)
Late Neonatal	0.34 (0.19-0.58)
Post Neonatal	0.34 (0.19-0.58)
1 to 4	0.28 (0.16-0.44)
5 to 9	0.31 (0.15-0.56)
10 to 14	0.33 (0.19-0.53)
15 to 19	0.37 (0.2-0.64)
20 to 24	0.46 (0.12-1.16)
25 to 29	0.44 (0.17-0.93)
30 to 34	0.46 (0.22-0.83)
35 to 39	0.5 (0.22-1)
40 to 44	0.61 (0.13-1.75)
45 to 49	0.5 (0.21-0.99)
50 to 54	0.44 (0.23-0.74)
55 to 59	0.42 (0.21-0.75)
60 to 64	0.42 (0.15-0.95)
65 to 69	0.39 (0.19-0.7)
70 to 74	0.38 (0.21-0.61)
75 to 79	0.37 (0.2-0.62)
80 to 84	0.37 (0.17-0.71)
85 to 89	0.34 (0.19-0.59)
90 to 94	0.33 (0.16-0.61)
95 to 99	0.34 (0.13-0.8)

References

1 Miettinen OS. Proportion of disease caused or prevented by a given exposure, trait or intervention. *Am J Epidemiol* 1974; **99**: 325–32.

2 Shi T, McLean K, Campbell H, Nair H. Aetiological role of common respiratory viruses in acute lower respiratory infections in children under five years: A systematic review and meta-analysis. *J Glob Health* 2015; **5**: 10408.

3 Feikin DR, Scott JAG, Gessner BD. Use of vaccines as probes to define disease burden. *Lancet Lond Engl* 2014; **383**: 1762–70.

4 O’Brien KL, Wolfson LJ, Watt JP, *et al.* Burden of disease caused by Streptococcus pneumoniae in children younger than 5 years: global estimates. *Lancet Lond Engl* 2009; **374**: 893–902.

5 Watt JP, Wolfson LJ, O’Brien KL, *et al.* Burden of disease caused by Haemophilus influenzae type b in children younger than 5 years: global estimates. *Lancet Lond Engl* 2009; **374**: 903–11.

6 Johnson HL, Deloria-Knoll M, Levine OS, *et al.* Systematic evaluation of serotypes causing invasive pneumococcal disease among children under five: the pneumococcal global serotype project. *PLoS Med* 2010; **7**. DOI:10.1371/journal.pmed.1000348.

7 Swingler G, Fransman D, Hussey G. Conjugate vaccines for preventing Haemophilus influenzae type B infections. *Cochrane Database Syst Rev* 2007; : CD001729.

8 Lucero MG, Dulalia VE, Nillos LT, *et al.* Pneumococcal conjugate vaccines for preventing vaccine-type invasive pneumococcal disease and X-ray defined pneumonia in children less than two years of age. *Cochrane Database Syst Rev* 2009; : CD004977.

9 Bonten MJM, Huijts SM, Bolkenbaas M, *et al.* Polysaccharide conjugate vaccine against pneumococcal pneumonia in adults. *N Engl J Med* 2015; **372**: 1114–25.

Upper respiratory infections

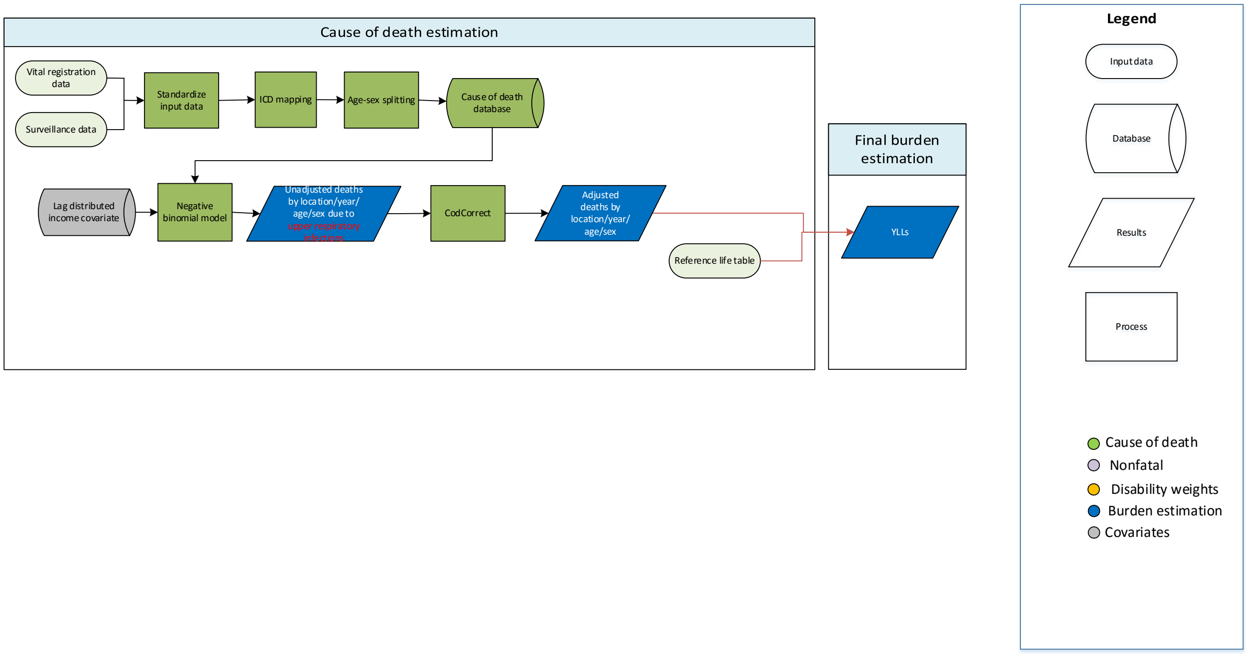

Input data

Vital registration and surveillance data from the cause of death database were used. Data with very high cause fractions (those greater than the 99th percentile values) were excluded in the regression.

Modeling strategy

Due to a small number of deaths, mortality from upper respiratory infections was modeled using a negative binomial regression, which is more appropriate than a Poisson count model as it accounts for greater variance (over-dispersion) in the data. By utilizing the exposure option in Stata, we model cause fractions with a negative binomial model. We tested both rate- and cause fraction-based models but selected a cause fraction model due to better model performance. Using the input data mentioned above, we modeled mortality from upper respiratory infections using the lag distributed income covariate and age dummy variables and the exposure set to the total number of deaths in the study. Uncertainty was estimated by taking 1,000 iterations of the predictions based on the variance covariance matrix and a random sample from a gamma distribution. The fit of the model was evaluated using diagnostic plots of predicted versus observed values.

Diphtheria

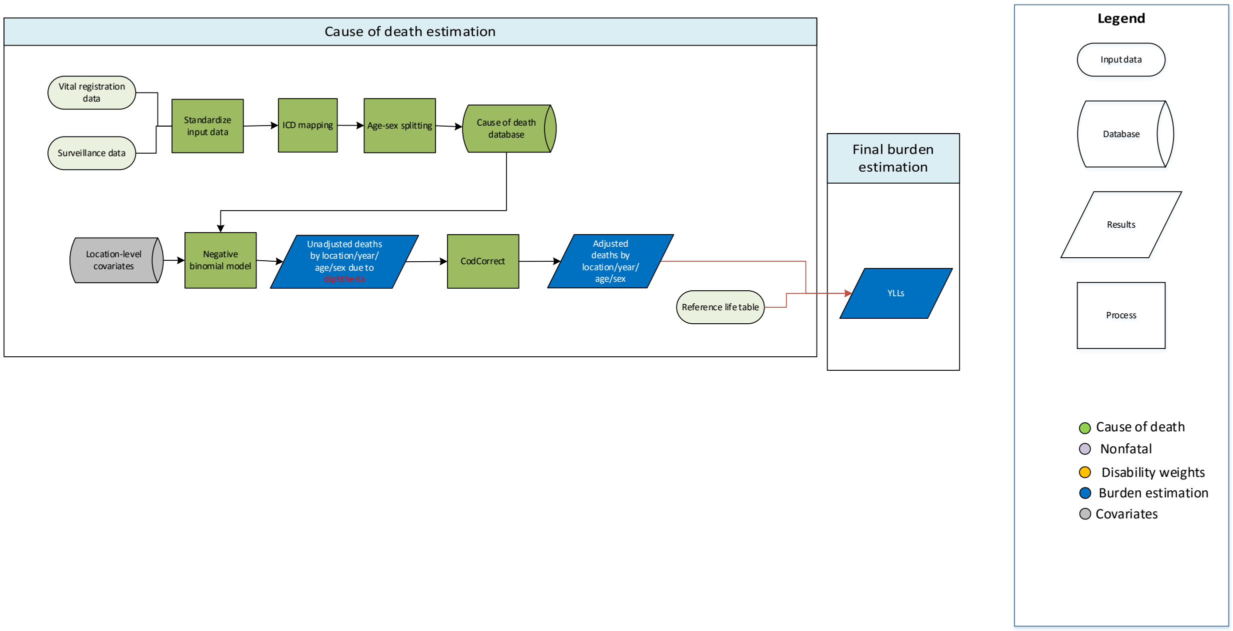

Input data

Vital registration and surveillance data from the cause of death database were used. Data with very high cause fractions (those greater than the 99th percentile values) were excluded in the regression.

Modeling strategy

Due to the small number of deaths, diphtheria mortality was modeled using a negative binomial regression, which is more appropriate than a Poisson count model as it accounts for greater variance (over-dispersion) in the data. Using the input data mentioned above, we modeled mortality due to diphtheria with the diphtheria-pertussis-tetanus third-dose (DPT3) vaccine coverage covariate and age dummy variables, with the offset as the total number of deaths in the study. Uncertainty was estimated by taking 1,000 iterations of the predictions based on the variance-covariance matrix and a random sample of the dispersion parameter from a gamma distribution.

Pertussis (whooping cough)

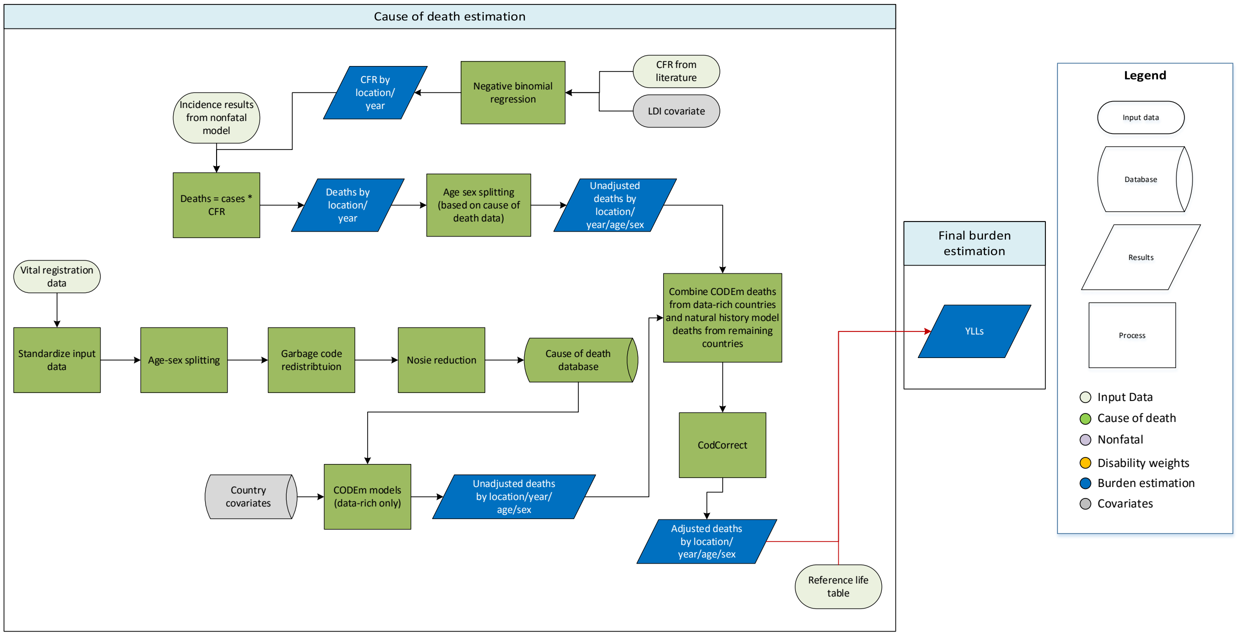

Input data

Vital registration data from the cause of death database were used for data-rich countries. To inform the natural history model, we used data from the following sources: World Health Organization (WHO) case notifications; historical case notifications for the United Kingdom back to 1940; case fatality data identified by collaborators; and case fatality data identified through systematic literature reviews for GBD 2010, GBD 2013, and GBD 2016. The PubMed search query for GBD 2016 was: (whooping cough [Title/Abstract]) OR (pertussis [Title/Abstract]) AND (case fatality [Title/Abstract]) AND ("2013"[Date - Publication]: "2016"[Date - Publication]). Studies were included if they reported case fatality rate, number of deaths, and number of cases. Studies were excluded if they included non-representative samples only.

Modeling strategy – data-rich countries

Mortality was modeled separately for data-rich and other countries. For data-rich countries (i.e., countries with vital registration more than 95% complete for more than 25 years), we used a general CODEm strategy with DPT3 vaccination coverage, lagged distributed income, and education as country-level covariates. We made estimations for the age range post-neonatal to 59 years.

Modeling strategy – other countries

For the remaining countries, we used a natural history-based model because CODEm does not predict well for those countries. First, we modeled log-transformed incidence with a mixed-effects linear regression of case notifications from the WHO (1985-2015) on diphtheria-tetanus-pertussis dose 3 (DTP3) vaccination coverage. Historical data of United Kingdom (UK) pertussis cases and UK DTP3 coverage rates (both back to 1940) were also used to inform the incidence model. The random effect by

country allowed for registration completeness to vary by country. The results of this model were then used to predict incidence as a function of vaccine coverage. To correct for underreporting in case notifications, we used a value of the random effect that matched the highest random effect in a high income region—Switzerland (which has a pertussis monitoring system which captures a high percentage of cases)—to get an implied attack rate assumed to be the same for all unvaccinated populations. Uncertainty was estimated by taking 1,000 iterations of the predictions based on the variance-covariance matrix.

Second, we modeled the pertussis case fatality rate using a negative binomial model with the health system access and lagged-distributed income covariates. Uncertainty was estimated by taking 1,000 iterations of the predictions based on the variance-covariance matrix and a random sample from a gamma distribution of the dispersion parameter. Finally, whooping cough deaths were calculated at the 1,000-draw level as

$$deaths = incidence * CFR\ .$$

We estimated overall number of deaths and then assigned an age-sex distribution based on the age- and sex-specific patterns found in the cause of death data. We made estimations for the age range post-neonatal to 59 years.

Tetanus

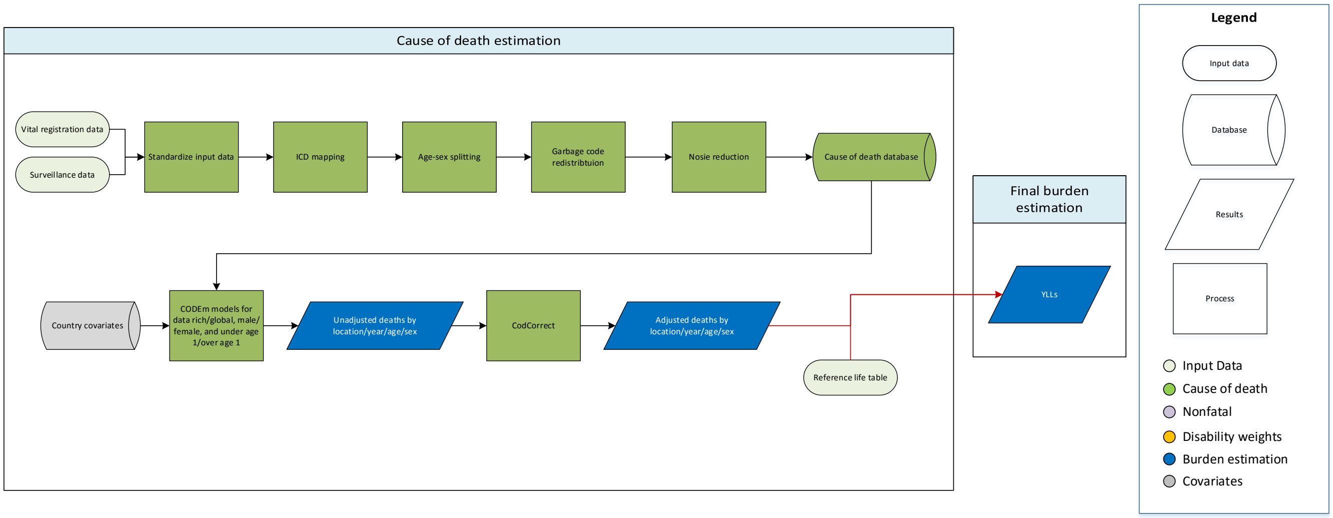

Input data

Mortality data from vital registration, verbal autopsy, and surveillance sources were used. Data were outliered if they largely conflicted with the majority of data from other studies conducted either in the same or different countries with similar sociodemographic characteristics in the same region.

Modeling strategy

A general CODEm modeling strategy was used. We ran separate models for under 1 year and 1 to 95+ years. There were no substantive changes in modeling strategy from GBD 2015.

Measles

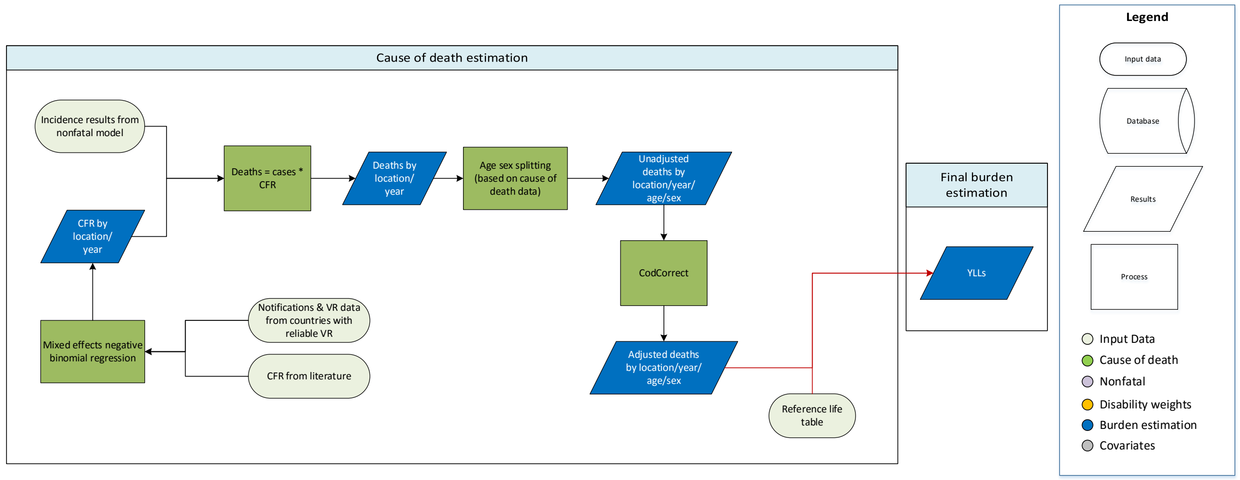

Input data

Vital registration data from the cause of death database were used for data-rich countries. To inform the natural history model, we used data from the following sources: World Health Organization (WHO) case notifications from 1995 to 2015; case notifications identified by collaborators; vital registration (VR) data in countries in the following three super-regions: high-income, Central Europe/Eastern Europe/Central Asia, and Latin America and Caribbean; and case fatality data identified through systematic literature reviews for GBD 2010, GBD 2013, and GBD 2016. The PubMed search query for GBD 2016 was: (measles [Title/Abstract]) AND (case fatality [Title/Abstract]) AND ("2013"[Date - Publication]: "2016"[Date - Publication]). Studies were included if they reported case fatality rate, number of deaths, and number of cases. Studies were excluded if they included non-representative samples only.

Modeling strategy – data-rich countries

Mortality was modeled separately for data-rich and other countries. For data-rich countries (i.e., countries with vital registration more than 95% complete for more than 25 years), we used a general CODEm strategy to model VR data with measles-containing vaccination dose one (MCV1) coverage, childhood malnutrition, lagged distributed income the healthcare access and quality index, and education as country-level covariates. We made estimations for the age range post-neonatal to 59 years.

Modeling strategy – other countries

Measles mortality in the remaining countries was modeled using a natural-history-based model. First, we modeled measles incidence with a mixed-effects linear regression of case notifications from the WHO (1995-2015) on routine measles vaccination rates and supplementary immunization activities (SIAs). More precisely, log-transformed incidence rates were regressed on the log of the proportion unvaccinated with first- and second-dose measles-containing vaccine, and additional SIA coverage lagged by one, two, three, four, and five years, with super-region, region, and country-level random effects. The results of this mixed effects regression model were then used to predict location-year-

specific incidence as a function of routine vaccine coverage and SIAs. To correct for underreporting in case notifications, we added the effect of a 95% attack rate, assumed to be the same across all unvaccinated populations. Uncertainty was estimated by taking 1,000 iterations of the predictions based on the variance-covariance matrix. For locations in three super-regions—high-income, Central Europe/Eastern Europe/Central Asia and Latin America and Caribbean—we used reported measles cases as incident cases.

Second, the case fatality rate was modeled using a mixed effects negative binomial regression with the child malnutrition covariate and study-level indicators (hospital-based or not; outbreak or not; and rural or urban/mixed), with country random effects. Uncertainty was estimated by taking 1,000 iterations of the predictions based on the variance-covariance matrix and uncertainty in country random effects. The fit of the model was evaluated using diagnostic plots of predicted versus observed values. Finally, estimated deaths were calculated at the 1,000-draw level as

$$deaths = incidence * CFR \ .$$

We estimated overall number of deaths and then assigned an age-sex distribution based on the age- and sex-specific patterns found in the cause of death data. We made estimations for the age range post-neonatal to 59 years.

Neonatal disorders

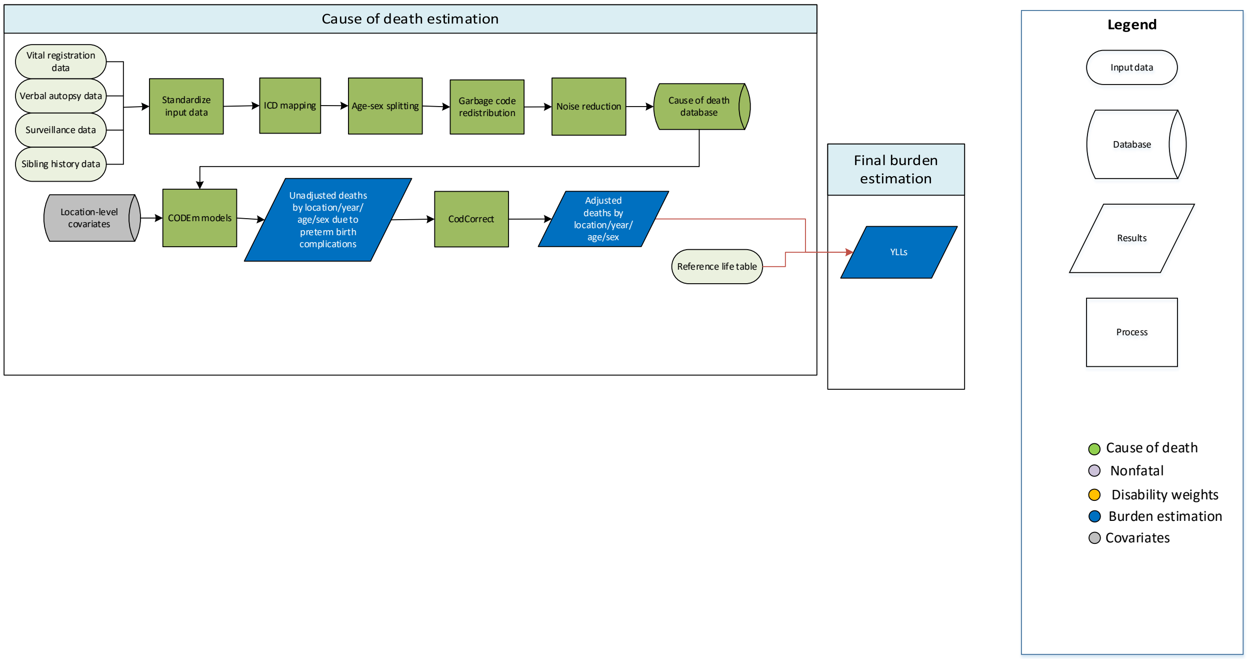

Input data

For the neonatal disorders envelope, preterm birth complications, and neonatal encephalopathy, vital registration, verbal autopsy, surveillance, and sibling history data were used for GBD 2016 to estimate number of deaths from each condition. For sepsis and other neonatal infections, vital registration, surveillance, and sibling history data were used. And for neonatal hemolytic disease and other neonatal conditions, vital registration and surveillance data were used. For all neonatal causes of death, vital registration was by far the most common data type. We only modelled deaths among males and females under age 5. Data points were selected as outliers if they were implausibly high, low, or significantly conflicted with established age or temporal patterns. Addition of significant new data from the Sample Registration System (SRS) in India had a significant effect on the estimates of mortality due to neonatal conditions at the global level.

Modelling strategy

For GBD 2016, an ensemble modelling approach was used via CODEm to model each of the different neonatal conditions. The same was done for GBD 2013 and 2015.

Varying levels of data quality and coding issues may still have affected our results. Validation studies suggest that verbal autopsy methods tend to be less accurate for cause of death ascertainment in the neonatal age groups.[1–4] This implies that in regions such as sub-Saharan Africa or South Asia, where the data primarily come from verbal autopsy studies, the distribution of sub-causes within all neonatal conditions may be less accurate. Furthermore, validation studies suggest that verbal autopsy methods tend to be particularly poor at ascertaining deaths from neonatal sepsis. Thus, for GBD 2016, all verbal autopsy data were excluded for neonatal sepsis and neonatal hemolytic disease.

Selected Covariates

Covariate	Transformation	Level	Direction
Education (years per capita)	None	3	-1
Health System Access	None	2	-1
In-Facility Delivery	None	2	-1
LDI (I$ per capita)	Log	3	-1
Underweight (proportion <2SD weight for age, <5 years)	None	2	1
Live Births 35+	None	2	1
Indoor Air Pollution (All cooking fuels)	None	1	1
Smoking prevalence (Reproductive Age-Standardized)	None	1	1
Total Fertility Rate	Log	3	1
SDI	None	3	-1
HAQI	None	2	-1
Skilled Birth Attendance	None	2	-1
Antenatal Care (4 visit)	None	2	-1

References

1 Anker M, Black RE, Coldham C, *et al.* A Standard Verbal Autopsy Method for Investigating Causes of Death in Infants and Children. Geneva, Switzerland: World Health Organization Department of Communicable Disease Surveillance and Response; The Johns Hopkins School of Hygiene and Public Health; The London School of Hygiene and Tropical Medicine, 1999.

2 Kalter HD, Gray RH, Black RE, Gultiano SA. Validation of postmortem interviews to ascertain selected causes of death in children. *Int J Epidemiol* 1990; **19**: 380–6.

3 Quigley MA, Armstrong Schellenberg JR, Snow RW. Algorithms for verbal autopsies: a validation study in Kenyan children. *Bull World Health Organ* 1996; **74**: 147–54.

4 Snow RW, Armstrong JR, Forster D, *et al.* Childhood deaths in Africa: uses and limitations of verbal autopsies. *The Lancet* 1992; **340**: 351–5.

Peptic ulcer disease

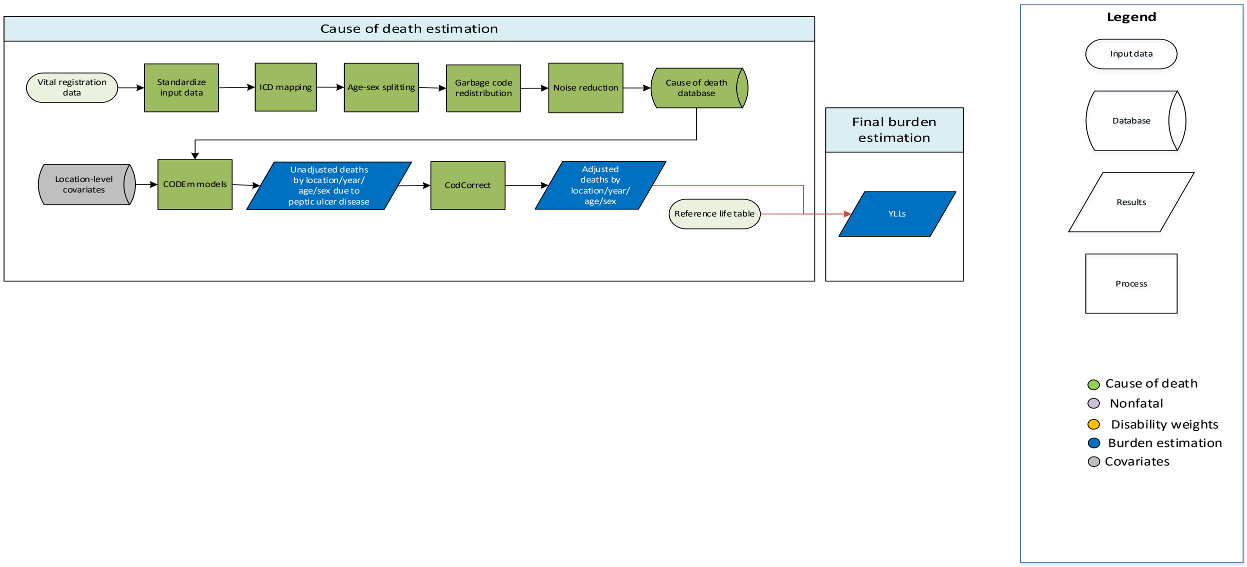

Input data

Data used to estimate mortality of peptic ulcer disease consisted of vital registration data from the cause of death (COD) database. We outliered data in instances where garbage code redistribution and noise reduction, in combination with small sample sizes, resulted in unreasonable cause fractions, and data that violated well-established time or age trends.

Modelling strategy

We modelled deaths due to peptic ulcer disease with a standard CODEm model using the cause of death database and location-level covariates as inputs. The model followed standard parameters, with the exception that the start age of the model was 1 year old instead of 0. We hybridized separate global and data-rich models to acquire unadjusted results, which we finalized and adjusted using CodCorrect to reach final years of life lost (YLLs) due to peptic ulcer disease. The covariate changes from GBD 2015 to GBD 2016 include changing the directionality of vegetables adjusted (grams per person availability) from -1 to 0, the addition of the summary exposure variable unsafe water, and the addition of the healthcare access and quality index (HAQI) covariate.

Covariate	Level	Direction
Alcohol (liters per capita)	1	1
Cumulative cigarettes (10 years)	1	1
Cumulative cigarettes (5 years)	1	1
Lag distributed income (per capita)	3	-1
Sanitation (proportion with access)	2	-1
Smoking (prevalence)	1	1
Maternal education (years per capita)	3	-1
Improved water source (proportion with access)	2	1
Sociodemographic index	3	-1
Vegetables (grams adjusted)	2	0
Health access and quality index	2	-1

Appendicitis

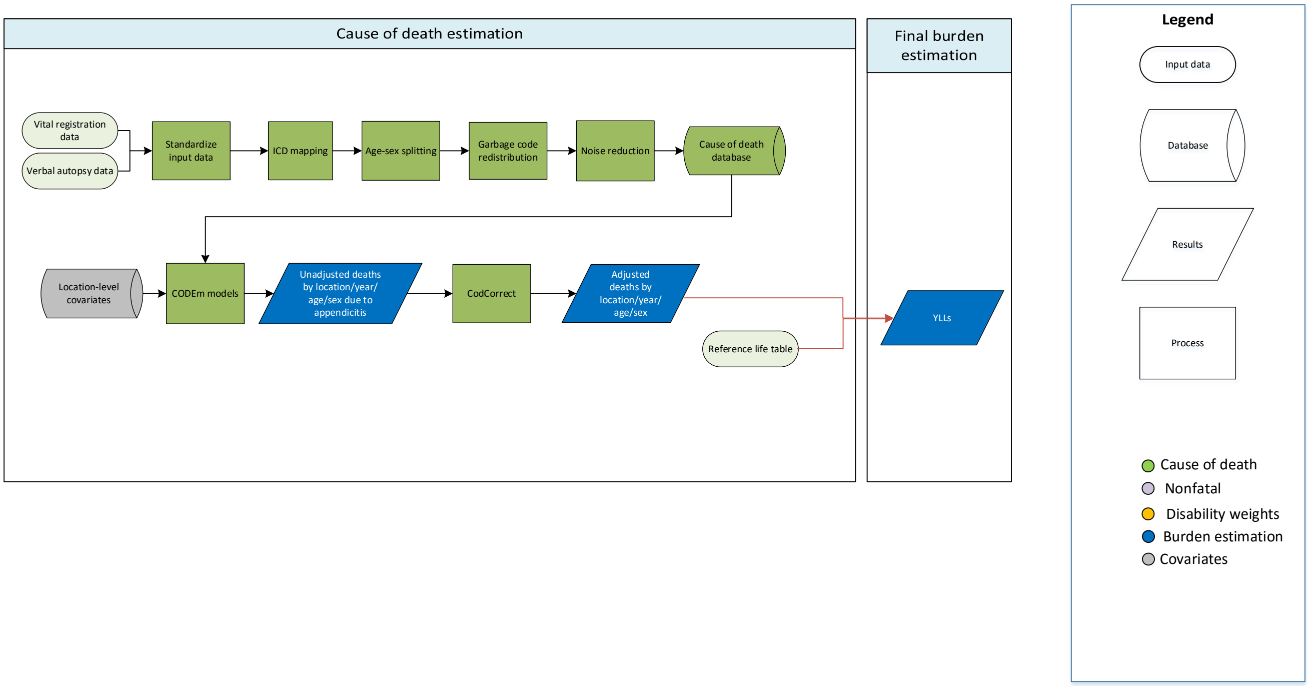

Input data

Data used to estimate appendicitis mortality consisted of vital registration and verbal autopsy data from the cause of death (COD) database. We outliered data in instances where garbage code redistribution and noise reduction, in combination with small sample sizes, resulted in unreasonable cause fractions; and data that violated well-established time or age trends.

Modelling strategy

We modelled deaths due to appendicitis with a standard CODEm model using the cause of death database and location-level covariates as inputs. The model followed standard parameters, with the exception that the start age of the model was 1 year old instead of 0 and the linear floor rate was lowered to 0.0001 in order to better capture low data. We hybridized separate global and data-rich models to acquire unadjusted results, which we finalized and adjusted using CodCorrect to reach final YLLs due to appendicitis.

There were no significant changes in the modelling process between GBD 2015 and GBD 2016.

Covariate	Level	Direction
Education (years per capita)	3	-1
Log LDI (I$ per capita)	3	-1
Health system access (capped)	3	-1
Socio-demographic Index	3	-1
Fruits adjusted (g)	2	-1
Vegetables adjusted (g)	2	-1
Healthcare access and quality index	2	-1

Inguinal, Femoral, and Abdominal Hernias

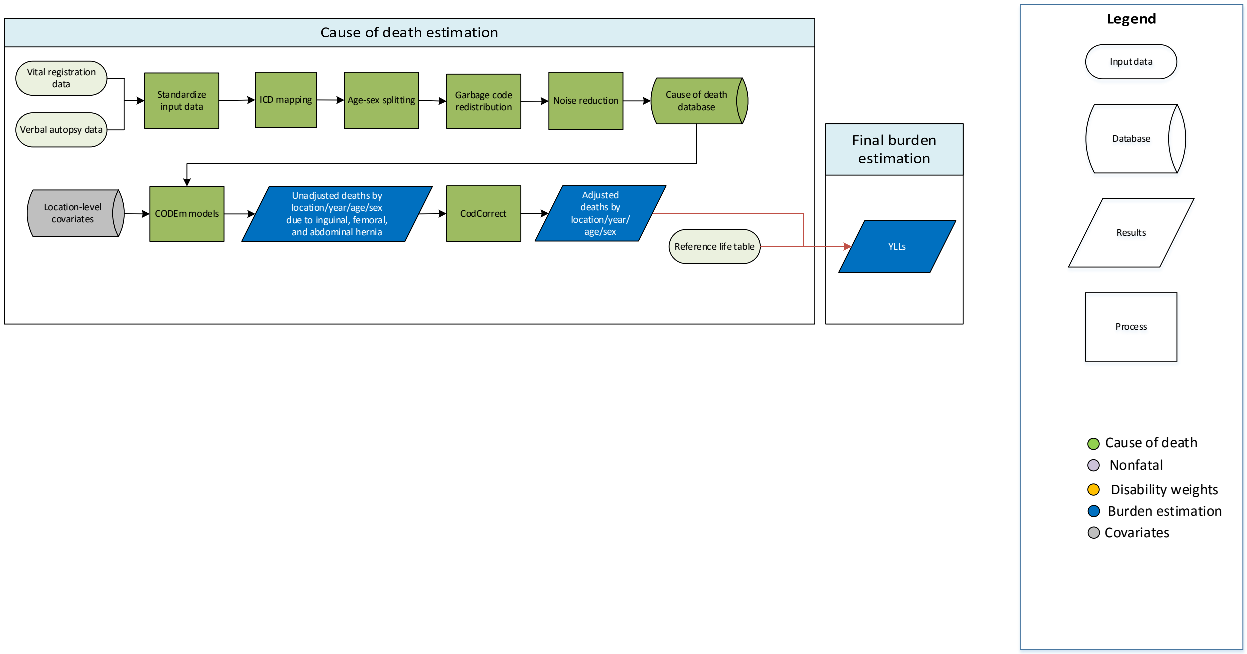

Input data

Vital registration and verbal autopsy data were used to model this cause. We outliered data in instances where garbage code redistribution and noise reduction, in combination with small sample sizes, resulted in unreasonable cause fractions; and data that violated well-established time or age trends. Outliering methods were consistent across both vital registration and verbal autopsy data.

Modelling strategy

We modelled deaths due to inguinal, femoral, and abdominal hernias with a standard CODEm model using the cause of death database and location-level covariates as inputs. The model followed standard parameters, with the exception that the start age of the model was 1 year old instead of 0 and the linear floor rate was lowered to 0.0001 in order to better capture low data. We hybridized separate global and data-rich models to acquire unadjusted results, which we finalized and adjusted using CodCorrect to reach final years of life lost (YLLs) due to inguinal, femoral, and abdominal hernias. In GBD 2016 we added the healthcare access and quality index (HAQI) covariate to the model.

Covariate	Level	Direction
Education (years per capita)	3	-1
Lag distributed income (per capita)	3	-1
Sociodemographic index	3	0
Health access and quality index	2	-1

Gallbladder and biliary diseases

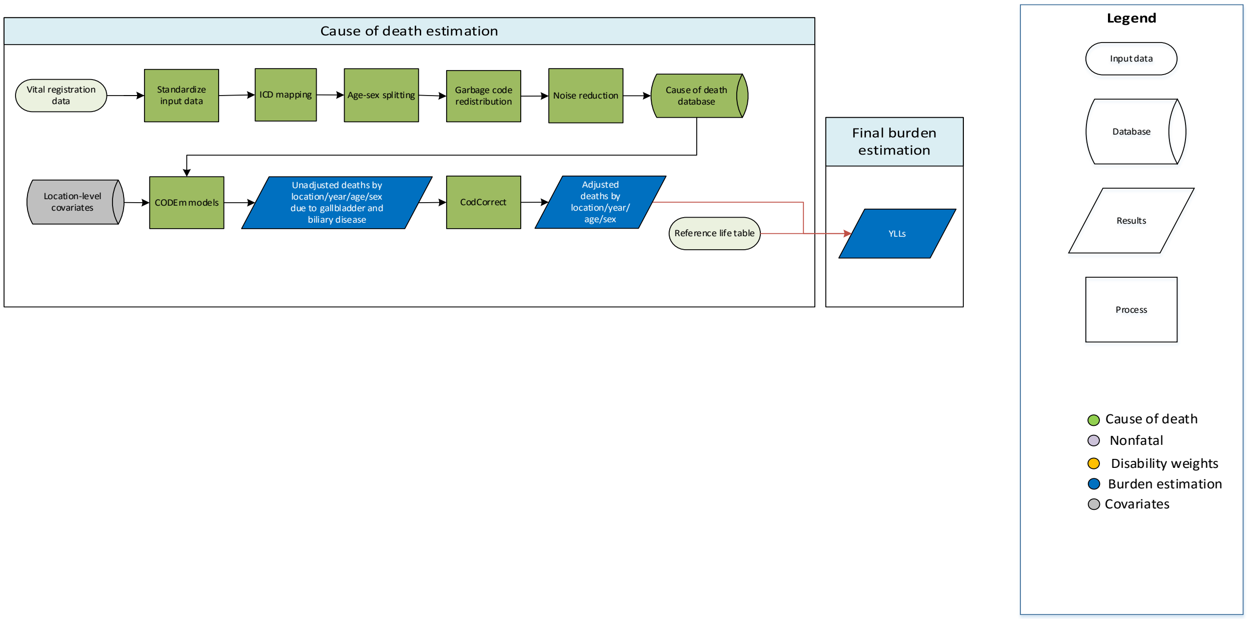

Input data

Data used to estimate mortality of gallbladder and biliary diseases consisted of vital registration data from the cause of death (COD) database. We outliered data in instances where garbage code redistribution and noise reduction, in combination with small sample sizes, resulted in unreasonable cause fractions; and data that violated well-established time or age trends.

Modelling strategy

We modelled deaths due to gallbladder and biliary diseases with a standard CODEm model using the cause of death database and location-level covariates as inputs. The model followed standard parameters, with the exception that the start age of the model was 1 year old instead of 0 and the linear floor rate was lowered to 0.0001 in order to better capture low data. We hybridized separate global and data-rich models to acquire unadjusted results, which we finalized and adjusted using CodCorrect to reach final years of life lost (YLLs) due to gallbladder and biliary diseases. In GBD 2016 we added the healthcare access and quality index (HAQI) covariate and replaced the animal fats (kcal per capita) covariate with an updated saturated fats (adjusted percent).

Covariate	Level	Direction
Alcohol (liters per capita)	2	1
Education (years per capita)	3	0
Lag distributed income (per capita)	3	0
Body mass index (mean)	1	1
Population over 65 (proportion)	2	1
Sociodemographic index	3	0
Red meats (grams adjusted)	2	1
Saturated fats (adjusted percent)	1	1
Health access and quality index	2	-1

Epilepsy

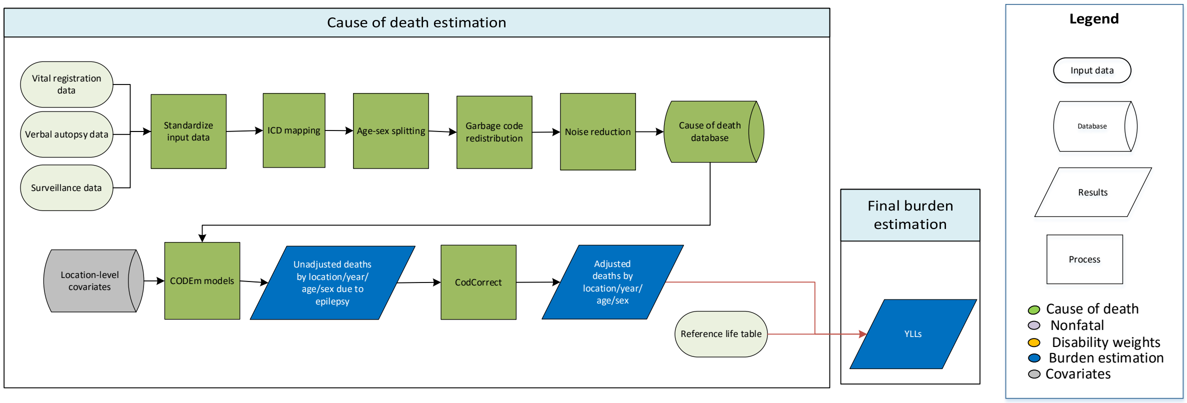

Input data

Data used to estimate epilepsy mortality included vital registration (VR), verbal autopsy, and China mortality surveillance data from the cause of death (COD) database. Our outlier criteria were to exclude data points that were (1) implausibly high or low relative to global or regional patterns, (2) substantially conflicted with established age or temporal patterns, or (3) significantly conflicted with other data sources based from the same locations or locations with similar characteristics (i.e., socio-demographic index).

Based on these criteria, we excluded ICD-9 BTL data for Sri Lanka, Fiji, and Kiribati as the estimates varied from year to year between zero and high values. We also excluded the Survey of Causes of Death Data and Medical Certification of Cause of Death Data for India, as these data types were not consistent with the Sample Registration System Data and would have led to discontinuities in our estimates over time.

Modelling strategy

The standard CODEm modelling approach was applied to estimate deaths due to epilepsy. Separate models were conducted for male and female mortality, and the age range for both models was 28 days–95+ years. For GBD 2016, the health systems access covariate was replaced with the health access and quality index covariate. There were no other substantial changes for GBD 2016. The covariates used are displayed below.

Level	Covariate	Direction
1	pig meat consumption (kcal per capita)	+
	pigs (per capita)	+
	SEV scalar: epilepsy	+
	mean systolic blood pressure (mmHg)	+
2	health access and quality index	-
	mean body mass index	+
	mean serum total cholesterol (mmol/L)	+

3	cumulative cigarettes (10 years)	+
	cumulative cigarettes (5 years)	+
	education (years per capita)	-
	log LDI (per capita)	-
	Socio-demographic Index	-

Chronic Kidney Disease

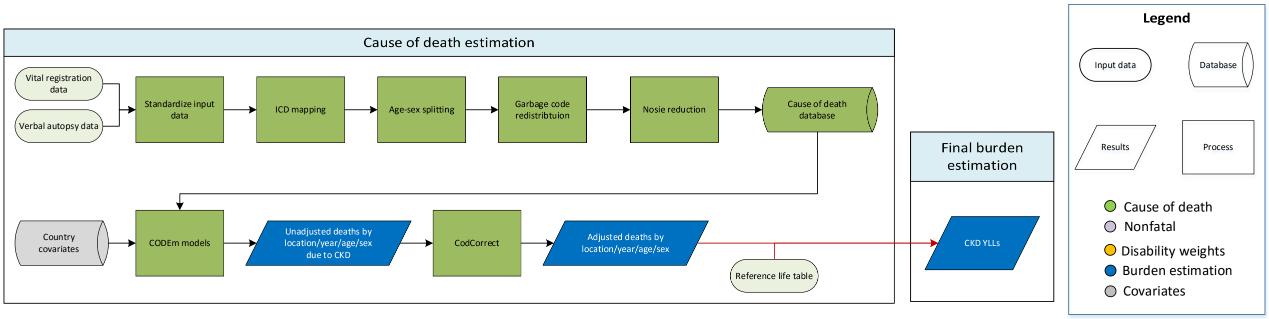

Input data

Vital registration and verbal autopsy data were used to model mortality due to urolithiasis. Outliers were identified by systematic examination of data points for all location-years. Data were standardised and mapped according to the GBD causes of death ICD mapping method. These data were then age-sex split, and appropriate redistribution of garbage code data was performed. Data points that violated well-established age or time trends or that resulted in extremely high or low cause fractions were determined to be outliers. For GBD 2016, deaths due to congenital kidney anomalies (cystic kidney disease and reflux hydronephrosis) were attributed to chronic kidney disease, marking a change from GBD 2015 when these deaths were assigned to congenital anomalies.

Modelling strategy

The estimation strategy used for fatal chronic kidney disease is largely similar to methods used in GBD 2015. A standard CODEm model with location-level was used to model deaths due to chronic kidney disease. Iterations of models were assessed at the location/year/age-group/sex level to determine whether data points merited exclusion via outliering. Unadjusted death estimates were adjusted using CoDCorrect to produce final estimates of YLLs. The covariates used are displayed below.

Level	Covariate	Direction
1	Diabetes fasting plasma glucose (mmol/L)	+
	Diabetes age-standardized prevalence (proportion)	+
	Mean systolic blood pressure (mmHg)	+
	Mean BMI	+
	Health care access and quality index	–
2	Mean cholesterol	+
	Total calories (kcal per capita)	–
	Red meat (kcal per capita)	0
	Whole grains (kcal per capita)	0
	Animal fat (kcal per capita)	0
3	Socio-demographic Index	0
	Education (years per capita)	–
	Log LDI ($I per capita)	–

Chronic Kidney Disease subtypes

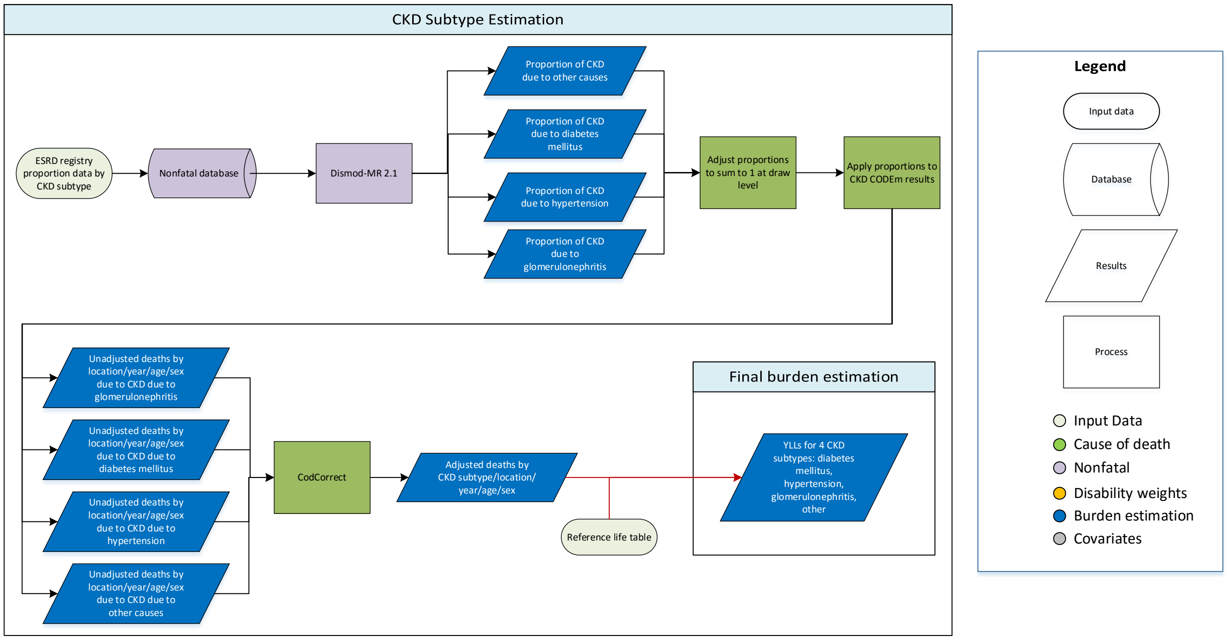

Input data

The estimation strategy for CKD subtypes of 1) diabetes mellitus, 2) hypertension, 3) glomerulonephritis, and 4) "other" has changed significantly from the GBD 2015 analysis to achieve consistency of method among the four subtypes. This improved method is detailed below.

Data from end-stage renal disease registries were used to inform estimates of proportion of CKD mortality attributable to each CKD subtype. These data were age-split using the age pattern obtained from the Australia & New Zealand Dialysis and Transplant Registry (ANZDATA) which provides age and subtype-specific data. The age-pattern was determined by calculating the number of cases of CKD by etiology over the total number of cases for all etiologies for each 5-year age group. Then, aggregate-age proportions were split using the age-specific prevalence proportions and rescaled to sum to 1 within each 5-year age group.

Vital registration (VR) data were excluded from estimates as etiology coding in VR sources was considered highly variable and inconsistent between countries.

Modelling strategy

We ran DisMod-MR 2.1 models including diabetes prevalence and mean systolic blood pressure as country-level covariates to obtain estimates of proportions for each subtype by location, year, age, and sex. The results from these models were adjusted so that estimates across the subtypes equaled 1 at each of 1,000 draws. These adjusted proportions were applied to the parent CKD CODEm model.

Model	Covariate	Value	Exponentiated
CKD proportion due to diabetes mellitus	Diabetes age-standardized prevalence	0.36<br>(0.29 – 0.42)	1.43<br>(1.34 – 1.53)
CKD proportion due to hypertension	Mean systolic blood pressure	0.013<br>(0.00036 – 0.043)	1.01<br>(1.00 – 1.04)

Congenital Birth Defects: *Neural tube defects, Congenital heart anomalies, Orofacial clefts, Down Syndrome, Turner syndrome, Klinefelter syndrome, Other chromosomal disorders, Congenital musculoskeletal anomalies, Urogenital congenital anomalies, Digestive congenital anomalies, and Other congenital birth defects.*

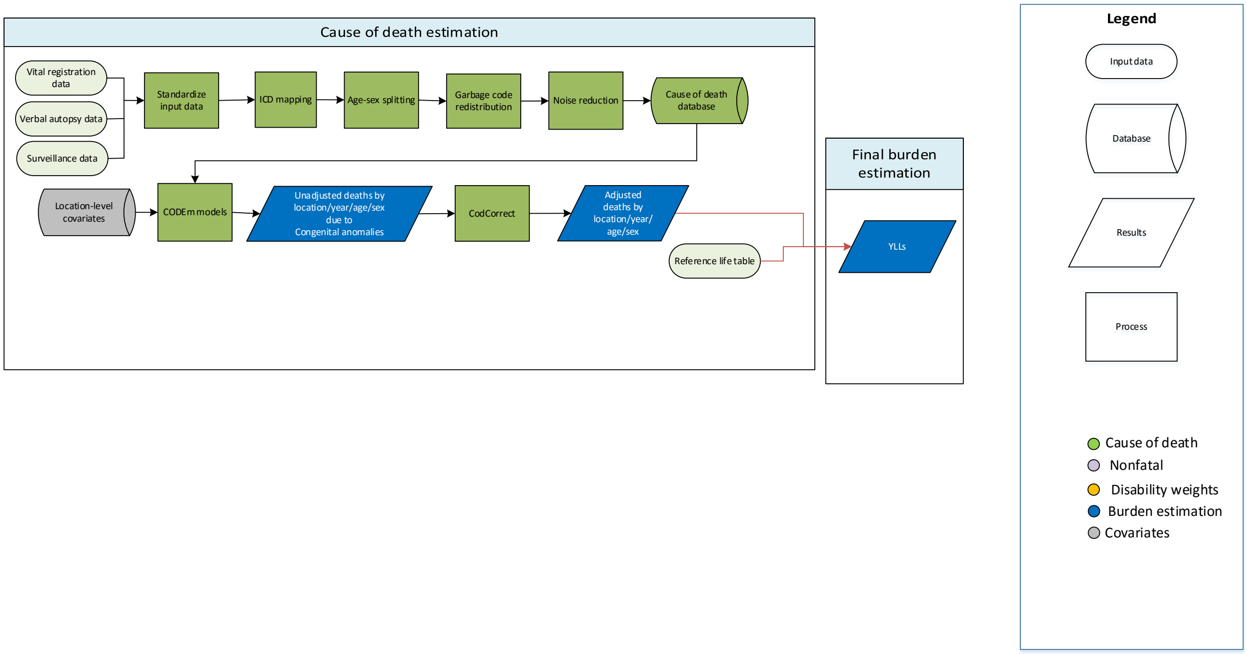

Input data

For GBD 2016, input data for estimating mortality due to congenital anomalies was centrally extracted, processed, and stored in causes of death (CoD) database. Vital registration (VR) was the dominant data type, followed by verbal autopsy (VA) and surveillance. Those CoD data sources that specified the sub-cause of birth defect were included in estimation of both the parent congenital anomalies model as well as in sub-type-specific models.

For GBD 2016, data exclusions were limited. We outliered all VA data in those over 5 years old as the age patterns were unreliable and led to poor model performance in the under-5 age groups. We also excluded some data sources from the parent model where only a subset of sub-causes were specified (eg, congenital heart disease, neural tube defects, and other congenital anomalies) and the sum of the sub-causes clearly represented systematic underreporting of one of the sub-causes. Systematic underreporting was suspected when sex- and age-specific rates were more than an order of magnitude lower than neighboring or comparable locations. Data sources for those locations were still included by default for sub-cause specific models because under-reporting of the total was not assumed to necessarily be associated with under-reporting of all of the component conditions.

Modeling strategy

All types of congenital anomalies were estimated using cause of death ensemble modeling (CODEm) for GBD 2016, as was done for previous iterations of the GBD study. Specific causes included neural tube defects, congenital heart anomalies, orofacial clefts, Down Syndrome, other chromosomal anomalies, congenital musculoskeletal anomalies, urogenital congenital anomalies, digestive congenital anomalies, and other congenital birth defects. We assumed no mortality from either Klinefelter syndrome or Turner syndrome, for which we model nonfatal outcomes only. For GBD 2016, we modeled congenital anomalies as a cause of death for ages 0-69 years only, assuming that all mortality from congenital conditions occurs before age 70 years of age.

For GBD 2016, we added three new causes to the congenital anomalies: congenital musculoskeletal and limb anomalies; urogenital congenital anomalies; and digestive congenital anomalies.

Covariates selected for CODEm model of overall congenital birth defects

Covariate	Transformation	Level	Direction
Maternal alcohol consumption during pregnancy (proportion)	None	1	Positive
In-Facility Delivery (proportion)	None	1	Negative
Live Births 35+ (proportion)	None	1	Positive
Folic acid unadjusted (ug)	None	1	Negative
Legality of Abortion	None	2	Negative
Antenatal Care (1 visit) Coverage (proportion)	None	2	Not specified
Smoking Prevalence (Reproductive Age Standardized)	None	2	Positive
Antenatal Care (4 visits) Coverage (proportion)	None	2	Negative
Healthcare access and quality index	None	2	Negative
Education (years per capita)	None	2	Negative
Alcohol (liters per capita)	None	3	Positive
fruits unadjusted(g)	None	3	Positive
Outdoor Air Pollution (PM2.5)	None	3	Positive
Indoor Air Pollution (All Cooking Fuels)	None	3	Positive
Socio-demographic Index	None	3	Negative
vegetables unadjusted(g)	None	3	Positive

Covariates selected for CODEm model of neural tube defects

Covariate	Transformation	Level	Direction
Health System Access (capped)	None	1	Negative
fruits adjusted(g)	None	2	Negative
vegetables adjusted(g)	None	2	Negative
Healthcare access and quality index	None	2	Negative
Education (years per capita)	None	3	Negative
LDI (I$ per capita)	Log	3	Negative

Socio-demographic Index	None	3	Negative

Covariates selected for CODEm model of congenital heart anomalies

Covariate	Transformation	Level	Direction
Maternal alcohol consumption during pregnancy (proportion)	None	1	Positive
Socio-demographic Index	Log	2	Negative
Smoking Prevalence (Reproductive Age Standardized)	None	2	Positive
Diabetes Age-Standardized Prevalence (proportion)	None	2	Positive
Healthcare access and quality index	None	2	Negative
Legality of Abortion	None	2	Negative
Antenatal Care (1 visit) Coverage (proportion)	None	2	Negative
In-Facility Delivery (proportion)	None	2	Negative
Education (years per capita)	None	2	Negative
Alcohol (liters per capita)	None	3	Positive
Antenatal Care (4 visits) Coverage (proportion)	None	3	Negative
Skilled Birth Attendance (proportion)	None	3	Negative
Live Births 35+ (proportion)	None	3	Positive

Covariates selected for CODEm model of cleft lip and cleft palate

Covariate	Transformation	Level	Direction
Indoor Air Pollution (All Cooking Fuels)	None	1	Positive
Diabetes Age-Standardized Prevalence (proportion)	None	2	Positive
Maternal alcohol consumption during pregnancy (proportion)	None	2	Positive
Healthcare access and quality index	None	2	Negative
Outdoor Air Pollution (PM2.5)	None	2	Positive
Legality of Abortion	None	2	Negative
Skilled Birth Attendance (proportion)	None	2	Negative
Smoking Prevalence (Reproductive Age Standardized)	None	2	Positive
vegetables unadjusted(g)	None	3	Not specified
Alcohol (liters per capita)	None	3	Positive
Antenatal Care (4 visits) Coverage (proportion)	None	3	Negative
Education (years per capita)	None	3	Negative
fruits unadjusted(g)	None	3	Not specified
Antenatal Care (1 visit) Coverage (proportion)	None	3	Negative

Covariates selected for CODEm model of Down Syndrome

Covariate	Transformation	Level	Direction
Live Births 35+ (proportion)	None	1	Positive
Legality of Abortion	None	1	Negative
Live Births 40+ (proportion)	None	1	Positive
Socio-demographic Index	None	2	Negative
LDI (I$ per capita)	Log	2	Negative
In-Facility Delivery (proportion)	None	2	Negative
Healthcare access and quality index	None	2	Negative
Maternal alcohol consumption during pregnancy (proportion)	None	3	Positive
Antenatal Care (1 visit) Coverage (proportion)	None	3	Negative
Education (years per capita)	None	3	Negative
Indoor Air Pollution (All Cooking Fuels)	None	3	Positive
Antenatal Care (4 visits) Coverage (proportion)	None	3	Negative
vegetables unadjusted(g)	None	3	Negative
Smoking Prevalence (Reproductive Age Standardized)	None	3	Positive

Covariates selected for CODEm model of other chromosomal abnormalities

Covariate	Transformation	Level	Direction
Live Births 35+ (proportion)	None	1	Positive
Live Births 40+ (proportion)	None	1	Positive
Legality of Abortion	None	1	Negative
LDI (I$ per capita)	Log	2	Negative
Healthcare access and quality index	None	2	Negative
Antenatal Care (4 visits) Coverage (proportion)	None	2	Negative
Antenatal Care (1 visit) Coverage (proportion)	None	2	Negative
In-Facility Delivery (proportion)	None	2	Negative
Maternal alcohol consumption during pregnancy (proportion)	None	2	Positive
Socio-demographic Index	None	3	Not specified
Alcohol (liters per capita)	None	3	Positive
Smoking Prevalence (Reproductive Age Standardized)	None	3	Positive
Education (years per capita)	None	3	Negative
Skilled Birth Attendance (proportion)	None	3	Negative

Covariates selected for CODEm model of congenital musculoskeletal and limb anomalies

Covariate	Transformation	Level	Direction
Maternal alcohol consumption during pregnancy (proportion)	None	1	Positive
Legality of Abortion	None	1	Negative
In-Facility Delivery (proportion)	None	2	Negative
Diabetes Age-Standardized Prevalence (proportion)	None	2	Positive
Socio-demographic Index	None	2	Negative
Healthcare access and quality index	None	2	Negative
Indoor Air Pollution (All Cooking Fuels)	None	2	Positive

Smoking Prevalence (Reproductive Age Standardized)	None	2	Positive
Antenatal Care (4 visits) Coverage (proportion)	None	3	Negative
Alcohol (liters per capita)	None	3	Positive
vegetables unadjusted(g)	None	3	Not specified
fruits unadjusted(g)	None	3	Not specified
Education (years per capita)	None	3	Negative
Antenatal Care (1 visit) Coverage (proportion)	None	3	Negative

Covariates selected for CODEm model of urogenital congenital anomalies

Covariate	Transformation	Level	Direction
Smoking Prevalence (Reproductive Age Standardized)	None	1	Positive
Maternal alcohol consumption during pregnancy (proportion)	None	1	Positive
Healthcare access and quality index	None	2	Negative
Diabetes Age-Standardized Prevalence (proportion)	None	2	Positive
Socio-demographic Index	None	2	Negative
Outdoor Air Pollution (PM2.5)	None	2	Positive
In-Facility Delivery (proportion)	None	2	Negative
Indoor Air Pollution (All Cooking Fuels)	None	2	Positive
Antenatal Care (1 visit) Coverage (proportion)	None	3	Negative
Alcohol (liters per capita)	None	3	Positive
Education (years per capita)	None	3	Negative
LDI (I$ per capita)	Log	3	Negative
Antenatal Care (4 visits) Coverage (proportion)	None	3	Negative

Covariates selected for CODEm model of digestive congenital anomalies

Covariate	Transformation	Level	Direction
Maternal alcohol consumption during pregnancy (proportion)	None	1	Positive
Smoking Prevalence (Reproductive Age Standardized)	None	1	Positive
Indoor Air Pollution (All Cooking Fuels)	None	2	Positive
Diabetes Age-Standardized Prevalence (proportion)	None	2	Positive
Socio-demographic Index	None	2	Negative
Prevalence of obesity (age-standardized)	None	2	Positive
In-Facility Delivery (proportion)	None	2	Negative
Healthcare access and quality index	None	2	Negative
Alcohol (liters per capita)	None	3	Positive
Health System Access (capped)	None	3	Negative
Education (years per capita)	None	3	Negative
vegetables unadjusted(g)	None	3	Not specified
Antenatal Care (1 visit) Coverage (proportion)	None	3	Negative
Antenatal Care (4 visits) Coverage (proportion)	None	3	Negative
fruits unadjusted(g)	None	3	Not specified
LDI (I$ per capita)	Log	3	Negative

Covariates selected for CODEm model of other congenital birth defects

Covariate	Transformation	Level	Direction
Maternal alcohol consumption during pregnancy (proportion)	None	1	Positive
Live Births 35+ (proportion)	None	1	Positive
Education (years per capita)	None	2	Negative
Smoking Prevalence (Reproductive Age Standardized)	None	2	Positive
Legality of Abortion	None	2	Negative
In-Facility Delivery (proportion)	None	2	Negative
Indoor Air Pollution (All Cooking Fuels)	None	2	Positive
Healthcare access and quality index	None	2	Negative
Antenatal Care (1 visit) Coverage (proportion)	None	3	Negative
Diabetes Age-Standardized Prevalence (proportion)	None	3	Positive
LDI (I$ per capita)	Log	3	Negative
Socio-demographic Index	None	3	Negative
Antenatal Care (4 visits) Coverage (proportion)	None	3	Negative
Alcohol (liters per capita)	None	3	Positive

3.9.1 and 7.1.2 Household Air Pollution SDG Capstone Appendix

Flowchart

Household Air Pollution from Solid Fuels

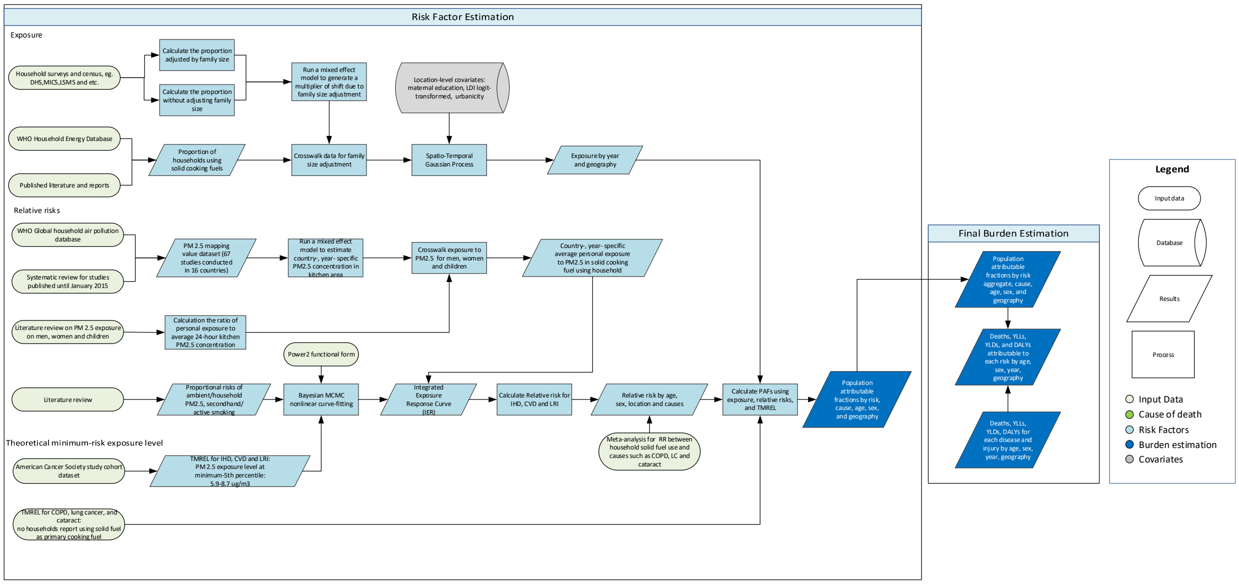

Input Data & Methodological Summary

Indicator definition

This modeling strategy encompassed the indicator associated with deaths attributable to household air pollution (3.9.1).

Indicator 3.9.1

As a component of SDG Goal 3. Ensure healthy lives and promote well-being for all at all ages, SDG Target 3.9, by 2030, substantially reduce the number of deaths and illnesses from hazardous chemicals and air, water and soil pollution and contamination, is measured using SDG Indicator 3.9.1, deaths attributable to household air pollution and ambient air pollution per 100,000.

Indicator 7.1.2

As a component of SDG Goal 7. Ensure access to affordable, reliable, sustainable, and modern energy for all, SDG Target 7.1, by 2030, ensure universal access to affordable, reliable and modern energy services, is measured using SDG Indicator 7.1.2, risk weighted prevalence of population using unsafe cooking fuel, which comes from household air pollution (HAP).

Exposure

Case Definition

Exposure to household air pollution from solid fuels (HAP) is defined as the proportion of households using solid cooking fuels. The definition of solid fuel in our analysis includes coal, wood, charcoal, dung, and agricultural residues.

Input data
Data were extracted from the standard multi-country survey series such as Demographic and Health Surveys (DHS), Living Standards Measurement Surveys (LSMS), Multiple Indicator Cluster Surveys (MICS), and World Health Surveys (WHS), as well as country-specific survey series such as Kenya Welfare Monitoring Survey and South Africa General Household Survey. To fill the gaps of data in surveys and censuses, we also downloaded and updated HAP estimates from WHO Energy Database and extracted from literature through systematic review. Each nationally or subnationally representative data point provided an estimate for the percentage of households using solid cooking fuels. Estimates for the usage of solid fuels for non-cooking purpose were excluded, i.e., primary fuels for lighting. The database, with estimates from 1980 to 2016, contained about 680 studies from 150 countries. As updates to systematic reviews are performed on an ongoing schedule across all GBD causes and risk factors, an update for household air pollution will be performed in the next 1-2 iterations.

Modeling strategy
Household air pollution was modeled at household level using a three-step modeling strategy that uses linear regression, spatiotemporal regression and Gaussian process regression (GPR). The first step is a mixed-effect linear regression of logit-transformed proportion of households using solid cooking fuels. The linear model contains maternal education, proportion of population living in urban areas, and lagged-distributed income as covariates and has nested random effect by GBD region, and GBD super-region, respectively. The full ST-GPR process is specified in Section 2 of this appendix.

No substantial modeling changes were in this round compared to GBD 2015. A variety of combinations of socioeconomic and environmental covariates in different transformation format were tested by running mixed-effect models with exposure data. The final list of covariates included in the exposure model are maternal education, proportion of population living in urban area, and lagged-distributed income since they proved to be the strongest predictors.

Theoretical minimum-risk exposure level
For outcomes where we extracted relative risks (RR) based on direct epidemiological evidence, i.e., chronic obstructive pulmonary disease (COPD), lung cancer, and cataract, TMREL was defined such that no households would report using solid fuel as their primary cooking fuel. For outcomes that utilize evidence based on the Integrated Exposure Response (IER), the TMREL is defined as uniform distribution between 2.4 and 5.9 $\mu g/m^3$. TMREL for household air pollution.

Relative risks
The disease-outcomes paired with household air pollution have not changed since GBD 2015. These outcomes include lower respiratory infections (LRI), stroke, ischemic heart disease (IHD), COPD, lung cancer, and cataract. The relative risks of all outcomes, with the exception of cataracts, were generated by using the integrated exposure-response functions (IER). The relative risks for cataracts were extracted from a meta-analysis paper (1). The IER curves are updated to reflect the newly updated data and utilization of a new method that specified elsewhere.

PM2.5 mapping value
The relative risk estimates describing the association of HAP with outcomes including ischemic heart disease (IHD), cardiovascular disease (CVD), and lower respiratory infections (LRI) were derived from the IER curves. This is done by first estimating the crosswalk values that map household use of solid fuel to

$PM_{2.5}$ exposure because the IER curve measures exposure using $PM_{2.5}$. For GBD 2015, this step of the analysis relied on 67 studies conducted in 16 countries to generate the $PM_{2.5}$ mapping values. In this round, we have extracted $PM_{2.5}$ data from about 20 additional studies to add to bring the total study sum of the database to almost 90 studies. The addition of more studies has provided more stability in the model and allowed us to use Socio-demographic Index as a covariate to predict exposure for all location-years. The $PM_{2.5}$ exposures were then crosswalked to men, women, and children by generating the ratio of personal exposure to average 24-hour kitchen $PM_{2.5}$ concentration based on a study after the literature review in GBD 2013.

References

1. Smith KR, Bruce N, Balakrishnan K, Adair-Rohani H, Balmes J, Chafe Z, et al. Millions Dead: How Do We Know and What Does It Mean? Methods Used in the Comparative Risk Assessment of Household Air Pollution. Annu Rev Public Health. 2014;35(1):185–206.

3.9.1 and 11.6.2 Ambient Particulate Matter Pollution SDG Capstone Appendix

Ambient PM2.5

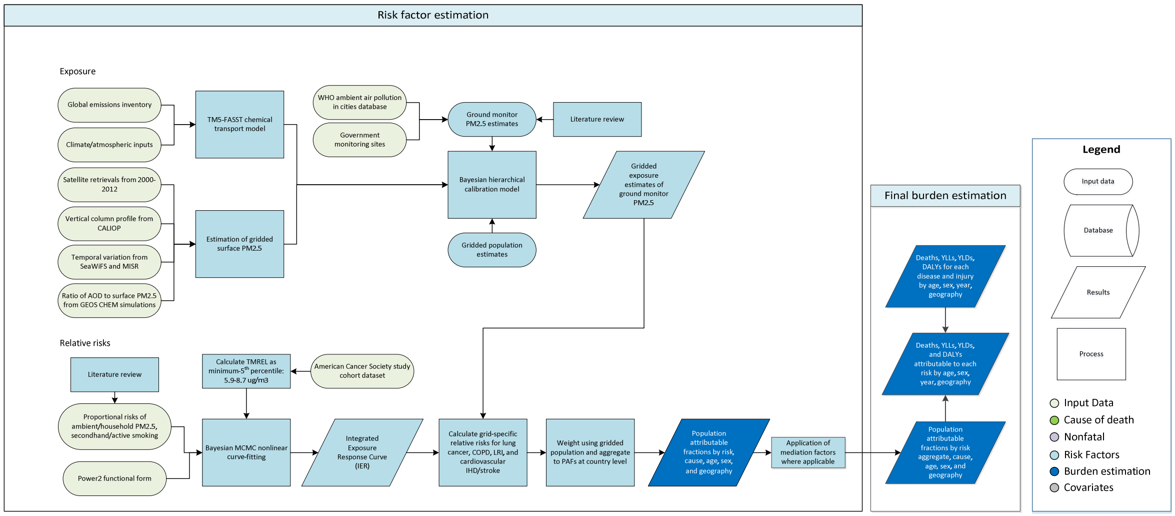

Input data and modeling strategy

Indicator definition

This modeling strategy encompassed the indicator associated with deaths attributable to ambient air pollution (3.9.1) as well as mean PM2.5 (11.6.2)

Indicator 3.9.1

As a component of SDG Goal 3. Ensure healthy lives and promote well-being for all at all ages, SDG Target 3.9, by 2030, substantially reduce the number of deaths and illnesses from hazardous chemicals and air, water and soil pollution and contamination, is measured using SDG Indicator 3.9.1, deaths attributable to household air pollution and ambient air pollution per 100,000

This modeling strategy encompassed the indicator associated with population-weighted PM2.5 (fine particulate matter) (11.6.2)

Indicator 11.6.2

As a component of SDG Goal 11. Make cities and human settlements inclusive, safe, resilient and sustainable, SDG Target 11.6, by 2030, reduce the adverse per capita environmental impact of cities, including by paying special attention to air quality and municipal and other waste management, is measured using SDG Indicator 11.6.2, Population-weighted mean levels of fine particulate matter (PM2.5).

Exposure

Definition

Exposure to ambient air pollution is defined as the population-weighted annual average mass concentration of particles with an aerodynamic diameter less than 2.5 micrometers ($PM_{2.5}$) in a cubic meter of air. This measurement is reported in µg/m^3.

Input Data

The data used to estimate exposure to ambient air pollution is drawn from multiple sources, including satellite observations of aerosols in the atmosphere, ground measurements, chemical transport model simulations, population estimates and land-use data.

The following details the updates in methodology and input data used in GBD2015 and GBD2016 from that used in GBD2013.

$PM_{2.5}$ ground measurement database

Updates of ground measurements used for GBD2015 and GBD2016 include using more recent data than that used in GBD2013 and the addition of data from locations where measurement data have become available. These updates were made in collaboration with the WHO and are included within the May 2016 update of the WHO Air Pollution in Cities database. Monitor-specific measurements (rather than city averages as reported in the WHO database) were used, resulting in measurements of concentrations of PM_{10} and $PM_{2.5}$ from 6,003 ground monitors from 117 countries. The majority of measurements were recorded in 2014 (as there is a lag in reporting measurements, little data from 2015 were available). Where data were not available for 2014 (2760 monitors), data was used from 2015 (18 monitors), 2013 (2155), 2012 (564), 2011 (60), 2010 (375), 2009 (49), 2008 (21) and 2006 (1). For locations measuring only PM_{10}, $PM_{2.5}$ measurements were estimated from PM_{10}. This was performed using a locally derived conversion factor ($PM_{2.5}$/PM_{10} ratio, for stations where measurements are available for the same year) that was estimated using population-weighted averages of location-specific conversion factors for the country. If country-level conversion factors were not available, the average of country-level conversion factors within a region were used. As in the GBD2013 database, additional information related to the ground measurements was also included where available, including monitor geo coordinates and monitor site type.

Satellite-based estimates

The updated satellite-based estimates for years 2000-2015 are described in detail in van Donkelaar et al. 2016[1]. These estimates were available at 0.1°×0.1° resolution (~11 x 11 km resolution at the equator) and combine aerosol optical depth retrievals from multiple satellites with the GEOS Chem chemical transport model and land use information.

Population data

A comprehensive set of population data on a high-resolution grid was obtained from the Gridded Population of the World (GPW) database. These data are provided on a 0.0417°×0.0417° resolution. Aggregation to each 0.1°×0.1° grid cell comprised of summing the central 3 × 3 population cells. As this resulted in a resolution higher than necessary, it was repeated four times, each offset by one cell in a North, South, East and West direction. The average of the resulting five quantities was used as the estimated population for each grid cell. Population estimates for 2000, 2005, 2010, 2015 and 2020 were availalble from GPW version 4. Populations for 2015 and 2016 were obtained by

interpolation using natural splines with knots placed at 2000, 2005, 2010, 2015 and 2020. This was performed for each grid cell.

Chemical transport model simulations

Estimates of the sum of particulate sulfate, nitrate, ammonium and organic carbon and the compositional concentrations of mineral dust simulated using the GEOS Chem chemical transport model, and a measure combining elevation and the distance to the nearest urban land surface (as described in van Donkelaar et al. 2016[1]) were available for 2000 to 2015 for each 0.1°×0.1° grid cell. These were not included within the GBD2013 analysis.

Modelling Strategy

Significant advances have been made in the methodology used to estimate exposure to ambient particulate matter pollution since GBD2013. The following is a summary of the modelling approach, known as the Data Integration Model for Air Quality (DIMAQ) used in GBD2015 and 2016; further details can be found in Shaddick *et al.* (2017)[2]

In GBD2010 and GBD2013 exposure estimates were obtained using a single global function to calibrate available ground measurements to a 'fused' estimate of $PM_{2.5}$; the mean of satellite-based estimates and those from the TM5 chemical transport model, calculated for each 0.1°×0.1° grid cell. This was recognized to represent a trade-off between accuracy and computationally efficiency when utilising all the available data sources. In particular, the GBD2013 exposure estimates were known to underestimate ground measurements in specific locations (see discussion in Brauer et al., 2016[3]). This underestimation was largely due to the use of a single, global, calibration function, whereas in reality the relationship between ground measurements and other variables will vary spatially.

In GBD2015 and GBD2016, coefficients in the calibration model were estimated for each country. Where data were insufficient within a country, information can be `borrowed' from a higher aggregation (region) and if enough information is still not available from an even higher level (super-region). Individual country level estimates were therefore based on a combination of information from the country, its region and super-region. This was implemented within a Bayesian Hierarchical modelling (BHM) framework. BHMs provide an extremely useful and flexible framework in which to model complex relationships and dependencies in data. Uncertainty can also be propagated through the model allowing uncertainty arising from different components, both data sources and models, to be incorporated within estimates of uncertainty associated with the final estimates. The results of the modelling comprise a posterior distribution for each grid cell, rather than just a single point estimate, allowing a variety of summaries to be calculated. The primary outputs here are the median and 95% credible intervals for each grid cell. Based on the availability of ground measurement data, modeling and evaluation was focused on the year 2014.

Due to both the complexity of the models and the size of the data, notably the number of spatial predictions that are required, recently developed techniques that perform 'approximate' Bayesian inference based on integrated nested Laplace approximations (INLA) were used[4]. Computation was performed using the R interface to the INLA computational engine (R-INLA). Fitting the models and performing predictions for each of the ca. 1.4 million grid cells required the use of a high performance computing cluster (HPC) making use of high memory nodes.

Model Evaluation

Model development and comparison was performed using within- and out-of-sample assessment. In the evaluation, cross validation was performed using 25 combinations of training (80%) and validation (20%) datasets. Validation sets were obtained by taking a stratified random sample, using sampling probabilities based on the cross-tabulation of $PM_{2.5}$ categories (0-24.9, 25-49.9, 50-74.9, 75-99.9, 100+ µg/m^3) and super-regions, resulting in them having the same distribution of $PM_{2.5}$ concentrations and super-regions as the overall set of sites. The following metrics were calculated for each training/evaluation set combination: for model fit - R^2 and deviance information criteria (DIC, a measure of model fit for Bayesian models); for predictive accuracy - root mean squared error (RMSE) and population weighted root mean squared error (PwRMSE).

All modelling was performed on the log-scale. The choice of which variables were included in the model was made based on their contribution to model fit and predictive ability. The following is a list variables and model structures that were considered in developing the model.

Continuous explanatory variables:

- (SAT) Estimate of $PM_{2.5}$ (in μgm^{-3}) for 2014 from satellite remote sensing on the log-scale.
- (CTM) Estimate of $PM_{2.5}$ (in μgm^{-3}) for 2010 from the TM5 chemical transport model on the log-scale.
- (POP) Estimate of population for 2014 on the log-scale.
- (SNAOC) Estimate of the sum of sulfate, nitrate, ammonium and organic carbon simulated using the GEOS Chem chemical transport model.
- (DST) Estimate of compositional concentrations of mineral dust simulated using the GEOS Chem chemical transport model.
- (EDxDU) The log of the elevation difference between the elevation at the ground measurement location and the mean elevation within the GEOS Chem simulation grid cell multiplied by the inverse distance to the nearest urban land surface.

Discrete explanatory variables:

- (LOC) Binary variable indicating whether exact location of ground measurement is known.
- (TYPE) Binary variable indicating whether exact type of ground monitor is known.
- (CONV) Binary variable indicating whether ground measurement is $PM_{2.5}$ or converted from PM_{10}.

Random Effects:

- Grid cell random effects on the intercept to allow for multiple ground monitors in a grid cell.
- Country-region-super-region hierarchical random effects for the intercept.
- Country-region-super-region hierarchical random effects for the coefficient associated with SAT .
- Country-region-super-region hierarchical random effects for the coefficient associated with the difference between estimates from CTM and SAT.
- Country-region-super-region hierarchical random effects for the coefficient associated with POP.
- Country level random effects for population uses a neighbourhood structure allowing specific borrowing of information from neighbouring countries.

- Within a region, country level effects of SAT and the difference between SAT AND CTM are assumed to be independent and identically distributed.
- Within a super-region, region level random effects are assumed to be independent and identically distributed.
- Super-region random effects are assumed to be independent and identically distributed.

Interactions:

- Interactions between the binary variables and the effects of SAT and CTM.

Results

The final model contained the following variables: SAT, POP, SNAOC, DST, EDxDU, LOC, TYPE, and CONV, together with interactions between SAT and each of LOC, TYPE and CONV. The model structure contained grid cell random effects on the intercept to allow for multiple ground monitors in a grid cell, country-region-super-region hierarchical random effects for intercepts and SAT and country level random effects for population using a neighbourhood structure allowing specific borrowing of information from neighbouring countries together with region-super-region hierarchical random effects for POP. Notably, based on the evaluation of candidate models, including estimates from the TM5 chemical transport model (CTM) used in GBD2013 did not improve the predictive ability of the model and was therefore not included.

Compared to the model used in GBD2013, DIMAQ showed improved predictions of ground measurements in all super regions (Table 1). Using this model resulted in an improvement in both within-sample fit; with an increase in R^2 from 0.64 (reported in GBD 2013[1]) to 0.91, and out-of-sample predictive ability; with a global population-weighted RMSE of 12.1 μg/m^3 compared to 23.1 μg/m^3 when using the GBD 2013 approach.

	GBD2013	GBD2015/16
Global	23.1	12.1
High income	*6.4*	*2.7*
Central Europe, Eastern Europe and Central Asia	*9.7*	*6.0*
Latin America and Caribbean	*13.9*	*7.1*
Southeast Asia, East Asia and Oceania	*20.1*	*10.8*
North Africa / Middle East	*23.6*	*14.3*
Sub-Saharan Africa	*38.8*	*32.3*
South Asia	*44.8*	*22.0*

Table 1: Summary measures of predictive ability, globally and by super-region. Results are the median values of population weighted root mean squared error (μg/m^3), from 25 validation sets.

Estimates for other years

Satellite estimates, populations and quantities estimated using the GEOS-Chem model were available for 1990, 1995, 2000, 2005, 2010, 2011, 2012, 2013, 2014 and 2015. Population estimates for 2000, 2005, 2010, 2015 and 2020 were availalble from GPW version 4. For 1990 and 1995 data were extracted from GPW version 3, as in GBD2013[2]. As with populations for 2015, values for each cell for 2011, 2012, 2012, 2013 and 2014 were obtained by interpolation using natural splines with knots placed at 2000, 2005, 2010, 2015 and 2020.

These were used as inputs to DIMAQ, enabling estimates of exposures to be obtained for each of these years respectively. For 2016, estimates of exposures were obtained from predictions from locally-varying regression models[4]. For each cell a model was fit to the values within that cell over time, with a constraint placed on the rate of change between 2015 and 2016 to avoid unrealistic and/or unjustified extrapolation of trends. Measures of uncertainty were obtained by repeating the procedure for the limits of the 95% credible intervals, again on a cell-by-cell basis.

References

1. van Donkelaar, A.; Martin, R. V; Brauer, M.; Hsu, N. C.; Kahn, R. A.; Levy, R. C.; Lyapustin, A.; Sayer, A. M.; Winker, D. M. Global Estimates of Fine Particulate Matter using a Combined Geophysical-Statistical Method with Information from Satellites, Models, and Monitors. Environ. Sci. Technol. 2016, 50 (7), 3762–3772
2. Shaddick, G., Thomas, M.L., Jobling, A., Brauer, M., van Donkelaar, A., Burnett, R., Chang, H., Cohen, A., Van Dingenen, R., Dora, C. and Gumy, S., 2016. Data Integration Model for Air Quality: A Hierarchical Approach to the Global Estimation of Exposures to Ambient Air Pollution. Journal of Royal Statistical Society Series C (Applied Statistics).2017. DOI: 10.1111/rssc.12227
3. Brauer, M.; Freedman, G.; Frostad, J.; van Donkelaar, A.; Martin, R. V; Dentener, F.; Van Dingenen, R.; Estep, K.; Amini, H.; Apte, J. S.; et al. Ambient Air Pollution Exposure Estimation for the Global Burden of Disease 2013. Environ. Sci. Technol. 2015, 50 (1), 79–88.
4. Rue, H.; Martino, S.; Chopin, N.; Approximate Bayesian inference for latent Gaussian models by using integrated nested Laplace approximations. Journal of the royal statistical society: Series b (statistical methodology). 2009;71(2):319-92.
5. Cleveland, W.S. and Devlin, S.J., 1988. Locally weighted regression: an approach to regression analysis by local fitting. Journal of the American statistical association, 83(403), pp.596-610.

3.9.2, 6.1.1, 6.2.1 WaSH SDG Capstone Appendix

Input data & Methodological Summary

Indicator definition

This modeling strategy encompassed the indicator associated with deaths attributable to unsafe water, sanitation, and hygiene (WaSH) (3.9.2).

For GBD 2016, the WaSH category is an aggregate of the risk estimates for water (6.1.1), hygiene (6.2.1b) and sanitation (6.2.1a). These are modeled independently and then aggregated together to generate the overall risk estimates for deaths attributable to WaSH.

Indicator 3.9.2

As a component of SDG Goal 3. Ensure healthy lives and promote well-being for all at all ages, SDG Target 3.9 by 2030, substantially reduce the number of deaths and illnesses from hazardous chemicals and air, water and soil pollution and contamination, is measured using SDG Indicator 3.9.2, deaths attributable to unsafe WaSH per 100,000.

Indicator 6.1.1

As a component of SDG Goal 6. Ensure availability and sustainable management of water and sanitation for all, SDG Target 6.1, by 2030, achieve universal and equitable access to safe and affordable drinking water for all, is measured using SDG Indicator 6.1.1, risk-weighted prevalence of population using unsafe/unimproved water sources.

Indicator 6.2.1a

As a component of SDG Goal 6. Ensure availability and sustainable management of water and sanitation for all, SDG Target 6.2, by 2030, achieve access to adequate and equitable sanitation and hygiene for all and end open defecation, paying special attention to the needs of women and girls and those in vulnerable situations, is measured using SDG Indicator 6.2.1a, risk-weighted prevalence of population using unsafe sanitation practices.

Indicator 6.2.1b

As a component of SDG Goal 6. Ensure availability and sustainable management of water and sanitation for all, SDG Target 6.2, by 2030, achieve access to adequate and equitable sanitation and hygiene for all and end open defecation, paying special attention to the needs of women and girls and those in vulnerable situations, is measured using SDG Indicator 6.2.1b, risk-weighted prevalence of population with no access to a handwashing facility.

3.9.2 and 6.1.1 Unsafe Water SDG Capstone Appendix

Flowchart

Unsafe Drinking Water

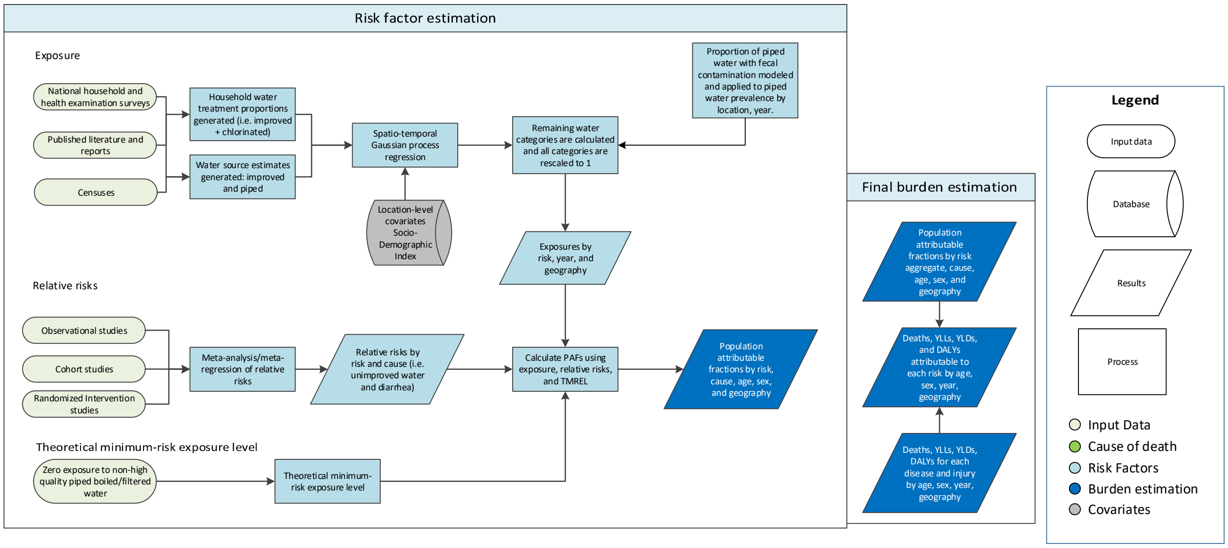

Input Data & Methodological Summary

Indicator definition

In addition to the indicator associated with deaths attributable to WaSH (3.9.2), this modeling strategy encompassed the indicator associated with water (6.1.1).

Indicator 6.1.1

As a component of SDG Goal 6. Ensure availability and sustainable management of water and sanitation for all, SDG Target 6.1, by 2030, achieve universal and equitable access to safe and affordable drinking water for all, is measured using SDG Indicator 6.1.1, risk-weighted prevalence of population using unsafe/unimproved water sources.

Exposure

Case Definition

For GBD 2016, exposure to unsafe water is defined based on reported primary water source used by the household and use of household water treatment (HWT) to improve the quality of drinking water before consumption. Water sources were defined as “improved” based on the JMP designation (WHO), which includes piped water as improved water, and households with access to piped water connection to the house, yard, or plot were defined as having access to piped water supply. One exception to this classification is that bottled water is considered “unimproved” by the JMP, however we treat it as an “improved” source. Solar treatment, chlorine treatment, boiling, or the use of filters were all assumed to be effective point-of-use household water treatments, and based on effect sizes published by Wolf et al. (2014), boiling or filtering was the most effective form of water treatment.

Input Data

The search for usable household surveys and censuses was conducted using the Global Health Data Exchange (GHDx) database. All surveys through December 2016 that provide household-level micro-data on water source were added. Tabulated and report data were lower priority and were only updated when time permitted. HWT input data were limited to two large survey series (DHS and MICS) due to time constraints. An update to HWT input data is a top priority for estimating exposure to unsafe water in future iterations.

Modeling

Water source data are modeled in two distinct categories: household prevalence of improved water (excluding piped) and household prevalence of piped water. HWT is modeled in six distinct categories based on the three water treatment categories (filtered/boiled, solar/chlorine, or untreated) and two water source categories (piped or improved). One modeling change made for GBD 2016 was to model prevalence of piped water independent of the improved water envelope, as was done in GBD 2015. By year and location, each of the above categories are modeled using a three-step modeling scheme of mixed effect linear regression followed by spatiotemporal Gaussian process regression (ST-GPR), which outputs full time series estimates for each GBD 2016 location. Socio-demographic Index (SDI), an index metric that includes a measure of education and income level, was used as a fixed effect in the linear regression since it proved to have significant coefficients. Random effects were placed at GBD 2016 region and super-region levels.

The process of vetting and validating models was accomplished primarily through an examination of ST-GPR scatter plots by GBD 2016 location from 1990 to 2016. Any unfitting data points were re-inspected for error at the level of extraction and survey implementation, and subsequently excluded from analysis if deemed appropriate. In addition to SDI, a number of different potential fixed effects were considered, including lag-distributed income and urbanicity, but SDI proved to be the strongest predictor of unsafe water. Uncertainty in the estimates was initially formed based on standard deviation by survey, then propagated through ST-GPR modeling by means of confidence intervals around each data point that reflect the point-estimate specific variance.

Once models are vetted, full time series outputs from ST-GPR modeling are then converted from proportion to prevalence by year and geography and then rescaled to form nine mutually exclusive categories that sum up to 1. The table below provides the final result of this rescaling.

Category	*Definition*
Unimproved, no HWT	Proportion of households that use unimproved source, and *do not* use any HWT to purify their drinking water.
Unimproved, chlorine/solar	Proportion of households that use unimproved source, and solar or chlorine treatment to purify their drinking water.
Unimproved, boil/filter	Proportion of households that use unimproved source, and boil or filter to purify their drinking water.

Improved water except piped, no HWT	Proportion of households that use improved sources other than piped water supply, and *do not* use any HWT to purify their drinking water.
Improved water except piped, chlorine/solar	Proportion of households that use improved sources other than piped water supply, and use solar or chlorine treatment to purify their drinking water.
Improved water except piped, boil/filter	Proportion of households that use improved sources other than piped water supply, and boil/filter their drinking water.
Piped water, no boil/filter	Proportion of households that use piped water supply, and *do not* use any HWT to purify their drinking water
Piped water, chlorine/solar	Proportion of households that use piped water supply, and *use* solar or chlorine water treatment to purify their drinking water.
Piped water, boil/filter	Proportion of households that use piped water supply, and boil or filter to purify their drinking water

In previous GBD iterations, high-income countries were assumed to have no risk of unsafe water. For GBD 2016, we estimated the risk of unsafe water in high-income countries as well. Additionally, we modeled the microbiological quality of piped water sources primarily using data a review by Bain et al. (2014) that measured proportion of piped water sources contaminated with fecal indicators. We use the value generated from this model to split the prevalence of piped water into basic piped water and high quality piped water by location, year, age, and sex.

A substantial limitation in our analysis is the paucity of data on HWT and piped water quality. The inclusion of more location-specific data on water treatment utilization at the household level can greatly improve our estimates in future iterations.

Theoretical minimum-risk exposure level

The theoretical minimum-risk exposure level for unsafe water is defined as all households have access to high quality piped water that has been boiled or filtered before drinking.

Relative risks

Notable updates were made to the relative risks for unsafe water from GBD 2015. For GBD 2016, there is only one adverse health outcome paired with unsafe water, which is diarrheal disease. Note that previously typhoid fever and paratyphoid fever were also included as outcomes but were excluded this round due to the lack of direct evidence. A meta-analysis by Wolf et al. (2014) provided the bulk of the relative risk evidence for the relationship between unsafe water and diarrheal diseases. This meta-analysis was updated through a literature review that searched for related intervention studies post-2014 conducted in PubMed. Search terms used were identical to those provided by Wolf et al. (2014). Relative risk values for water-source interventions and point-of-use treatment interventions were calculated separately so the combined effect of a source intervention and point-of-use intervention was assumed to be multiplicative in order to match GBD 2016 exposure definitions. Please refer to appendix tables for more information on relative risk values and citations.

References

1. "Improved and Unimproved Water Sources and Sanitation Facilities."*WHO / UNICEF Joint Monitoring Programme: Wat/san Categories*. The WHO/UNICEF, n.d. Web. 08 June 2016
2. Wolf, Jennyfer, Annette Prüss-Ustün, Oliver Cumming, Jamie Bartram, Sophie Bonjour, Sandy Cairncross, Thomas Clasen, John M. Colford, Valerie Curtis, Jennifer De France, Lorna Fewtrell, Matthew C. Freeman, Bruce Gordon, Paul R. Hunter, Aurelie Jeandron, Richard B. Johnston, Daniel Mäusezahl, Colin Mathers, Maria Neira, and Julian P. T. Higgins. "Systematic Review: Assessing the Impact of Drinking Water and Sanitation on Diarrhoeal Disease in Low- and Middle-income Settings: Systematic Review and Meta-regression." Trop Med Int Health Tropical Medicine & International Health 19.8 (2014): 928-42. Web.
3. Bain, R., Cronk, R., Wright, J., Yang, H., Slaymaker, T., & Bartram, J. (2014). Fecal Contamination of Drinking-Water in Low- and Middle-Income Countries: A Systematic Review and Meta-Analysis. PLoS Medicine, 11(5). doi:10.1371/journal.pmed.1001644

3.9.2 and 6.2.1a Unsafe Sanitation SDG Capstone Appendix

Flowchart

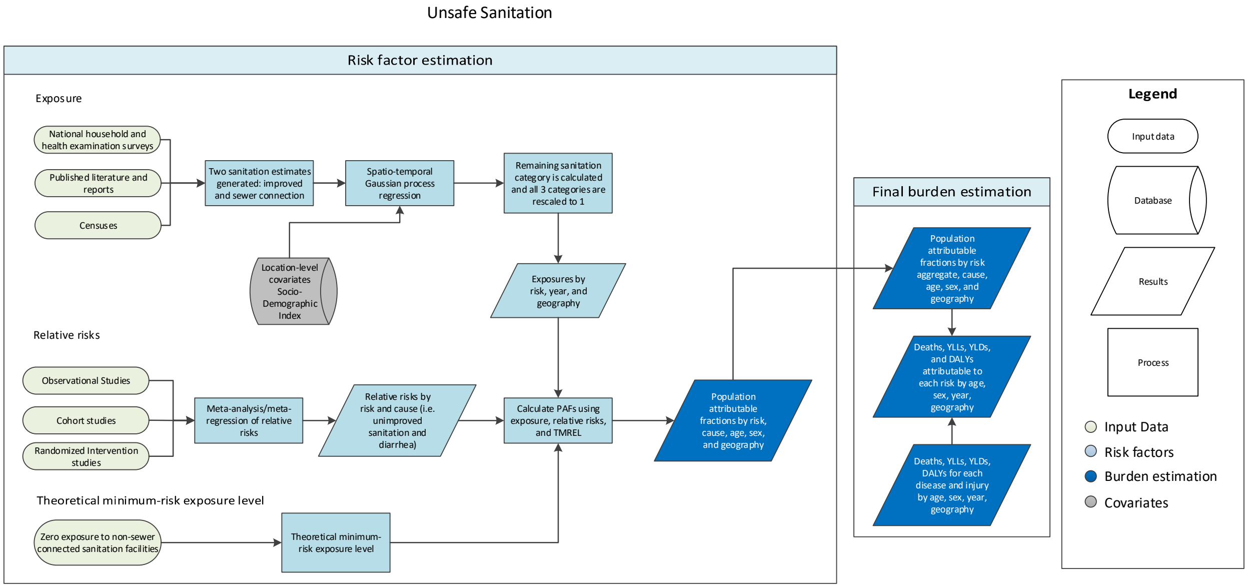

Input Data & Methodological Summary

Indicator definition

In addition to the indicator associated with deaths attributable to WaSH (3.9.2), this modeling strategy encompassed the indicator associated with sanitation (6.2.1a).

Indicator 6.2.1a

As a component of SDG Goal 6. Ensure availability and sustainable management of water and sanitation for all, SDG Target 6.2, by 2030, achieve access to adequate and equitable sanitation and hygiene for all and end open defecation, paying special attention to the needs of women and girls and those in vulnerable situations, is measured using SDG Indicator 6.2.1a, risk-weighted prevalence of population using unsafe sanitation practices.

Exposure

Case Definition

Exposure to unsafe sanitation was defined based on the primary toilet type used by households. Improved facilities are defined as such based on JMP designation (WHO). Sewer connection toilets included flush toilets or any toilet with connection to the sewer or septic tank.

Input Data

The search for usable household surveys and censuses was conducted using the Global Health Data Exchange (GHDx) database. Searches were conducted from October 2016 to December 2016, with the final search of household-level micro-data on toilet type conducted December 2016. Due to the organized nature of the GHDx, the only search term used was "unsafe sanitation," which yielded just under 1,400 results, of which 795 were extracted and used as inputs for modeling. Tabulated and report data were lower priority and were only updated when time permitted.

Modeling

One modeling change made in GBD 2016 was that proportion of households with sewer connection is modeled independently, instead of within the "improved" sanitation envelope. Two distinct models were produced from sanitation data: prevalence of households with improved sanitation and the prevalence of households with a sewer connection. By each location-year, both models were generated using a three-step modeling scheme of mixed effect linear regression followed by spatiotemporal Gaussian process regression (ST-GPR), which outputs full time series estimates for each GBD 2016 location. Socio-demographic Index (SDI), an index metric that includes measure of education, income level, and fertility, was used as a fixed effect in the linear regression since it proved to have significant coefficients. Random effects were placed at GBD 2016 region and super-region levels.

The process of vetting and validating models was accomplished primarily through an examination of ST-GPR scatter plots by GBD 2016 location from 1990 to 2016. Any unfitting data points were re-inspected for error at the level of extraction and survey implementation, and subsequently excluded from analysis if deemed appropriate. In addition to SDI, a number of different potential fixed effects were considered, including lag-distributed income and urbanicity, but SDI proved to be the strongest predictor of unsafe sanitation. Uncertainty in the estimates was initially formed based on standard deviation by survey, then propagated through ST-GPR modeling by means of confidence intervals around each data point that reflect the point-estimate specific variance.

Once models were fully vetted, full time series outputs from ST-GPR modeling were rescaled to form three mutually exclusive categories that sum up to 1. The table below provides the final result of this rescaling.

Category	*Definition*
Unimproved sanitation	Proportion of households that use unimproved sanitation facilities.
Improved sanitation, excluding sewer	Proportion of households that use improved sanitation facilities except those with sewer connection.
Sanitation facilities with sewer connection	Proportion of households that use toilet facilities with sewer connection.

In previous GBD iterations, high-income countries were assumed to have no risk of unsafe sanitation. For GBD 2016, we estimate the risk of unsafe sanitation in high-income countries as well. One limitation that extends to the other two risk factors that comprise WaSH (unsafe water and unsafe hygiene) and can be improved upon in future iterations is taking into account covariance of access to water, sanitation, and handwashing facilities. Currently, all three components of WaSH were modeled independently, which may lead to an overestimation of the burden of WaSH risk factors.

Theoretical minimum-risk exposure level

The theoretical minimum-risk exposure level for unsafe sanitation was defined as all households have access to a sanitation facility with sewer connection. Since it was assumed that all households in high-income countries have access to sewer-connected sanitation, this counterfactual exposure level is applied to all households in high-income countries.

Relative risks

Notable updates were made to the relative risks for unsafe sanitation from GBD 2015. For GBD 2016, there was only one adverse health outcome paired with unsafe sanitation, which was diarrheal disease. Note that previously typhoid fever and paratyphoid fever were also included as outcomes but were excluded this round due to the lack of direct evidence. A meta-analysis by Wolf et al. 2014 provides the bulk of the relative risk evidence for the relationship between unsafe sanitation and diarrheal diseases. This meta-analysis was updated through a literature review that searched for related intervention studies post-2014 conducted in PubMed. Search terms used were identical to those provided by Wolf et al. 2014. Please refer to appendix tables for more information on relative risk values and citations.

References

1. "Improved and Unimproved Water Sources and Sanitation Facilities." *WHO / UNICEF Joint Monitoring Programme: Wat/san Categories*. The WHO/UNICEF, n.d. Web. 08 June 2016
2. Wolf, Jennyfer, Annette Prüss-Ustün, Oliver Cumming, Jamie Bartram, Sophie Bonjour, Sandy Cairncross, Thomas Clasen, John M. Colford, Valerie Curtis, Jennifer De France, Lorna Fewtrell, Matthew C. Freeman, Bruce Gordon, Paul R. Hunter, Aurelie Jeandron, Richard B. Johnston, Daniel Mäusezahl, Colin Mathers, Maria Neira, and Julian P. T. Higgins. "Systematic Review: Assessing the Impact of Drinking Water and Sanitation on Diarrhoeal Disease in Low- and Middle-income Settings: Systematic Review and Meta-regression." Trop Med Int Health Tropical Medicine & International Health 19.8 (2014): 928-42. Web.

3.9.2 and 6.2.1b Access to Hygiene SDG Capstone Appendix

Flowchart

Unsafe Handwashing

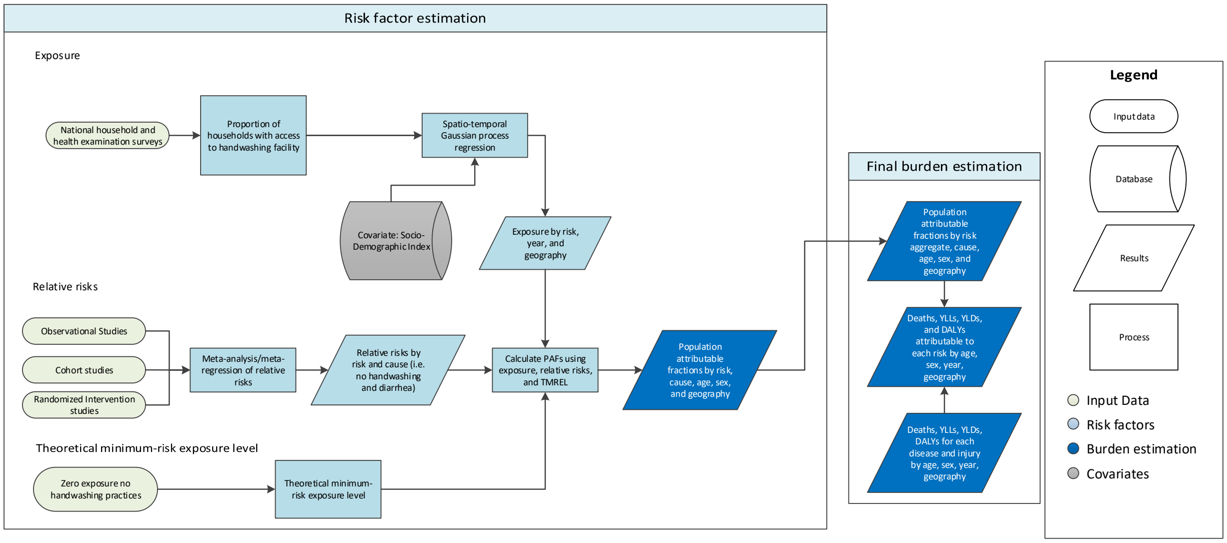

Input Data & Methodological Summary

Indicator definition

In addition to the indicator associate with Deaths attributable to WaSH (3.9.2), this modeling strategy encompassed the indicator associated with access to a handwashing facility (6.2.1b).

Indicator 6.2.1b

As a component of SDG Goal 6. Ensure availability and sustainable management of water and sanitation for all, SDG Target 6.2, by 2030, achieve access to adequate and equitable sanitation and hygiene for all and end open defecation, paying special attention to the needs of women and girls and those in vulnerable situations, is measured using SDG Indicator 6.2.1b, risk-weighted prevalence of population with no access to a handwashing facility.

Exposure

Case Definition

Lack of access to handwashing facility is defined as no access to a handwashing station with available soap and water. We estimated the burden of unsafe handwashing in both developed and developing settings.

Input Data

Since water and soap availability data were very limited, only country-specific Demographic Health Surveys (DHS) and Malaria Indicator Survey Series (MICS) conducted after 2006 were able to be used as input data.

Modeling Strategy

By year and location, proportion of households with a handwashing facility is modeled using a three-step modeling scheme of mixed effect linear regression followed by spatiotemporal Gaussian process regression (ST-GPR), which outputs full time series estimates for each GBD 2016 location. Socio-demographic Index (SDI), an index metric that includes a measure of education and income level, was used as a fixed effect in the linear regression since it proved to have significant coefficients. Random effects were placed at GBD 2016 region and super-region levels.

The process of vetting and validating models was accomplished primarily through an examination of ST-GPR scatter plots by GBD 2016 location from 1990 to 2016. Any unreasonable data points were re-inspected for error at the level of extraction and survey implementation, and subsequently excluded from analysis if deemed appropriate. In addition to SDI, a number of different potential fixed effects were considered, including lag-distributed income and urbanicity; however, SDI proved to be the strongest predictor.

A considerable limitation for when estimating handwashing practices for over 190 independent locations around the world was data sparseness. Even when data were published on handwashing prevalence, the definition was often altered from the GBD 2016 standard definition or it may only have pertained to certain populations (such as hospital patients) and lacked representativeness at the geographic scale we required. The incorporation of questions about soap and water availability in DHS and MICS added much-needed information, but there remains a large data gap that must be filled if we are to become more certain in handwashing access estimates.

Theoretical minimum-risk exposure level

The theoretical minimum-risk exposure level for unsafe hygiene is defined as all households engaging in handwashing with soap practices after any contact with excreta, including children's excreta.

Relative risks

Notable updates were made to the relative risks for unsafe water from GBD 2015. For GBD 2016, there were two adverse health outcomes paired with unsafe water: diarrheal disease and lower respiratory infection. Note that previously typhoid fever and paratyphoid fever were also included as outcomes but were excluded this round due to the lack of direct evidence. A meta-analysis by Cairncross et al. (2010) provided relative risk evidence for the relationship between lack of facility access and diarrheal diseases. A meta-analysis by Rabie and Curtis (2006) provided relative risk evidence for the relationship between lack of facility access and lower respiratory infection. Please refer to appendix tables for more information on relative risk values and citations.

References

1. Cairncross, S., Hunt, C., Boisson, S., Bostoen, K., Curtis, V., Fung, I. C., & Schmidt, W. P. (2010). Water, sanitation and hygiene for the prevention of diarrhoea. International Journal of Epidemiology, 39(Supplement 1), I193-I205. doi:10.1093/ije/dyq035
2. Rabie, T., & Curtis, V. (2006). Handwashing and risk of respiratory infections: a quantitative systematic review. Tropical Medicine and International Health, 11(3), 258-267. doi:10.1111/j.1365-3156.2006.01568.x

3.a.1 Smoking Prevalence SDG Capstone Appendix

Flowchart

Smoking

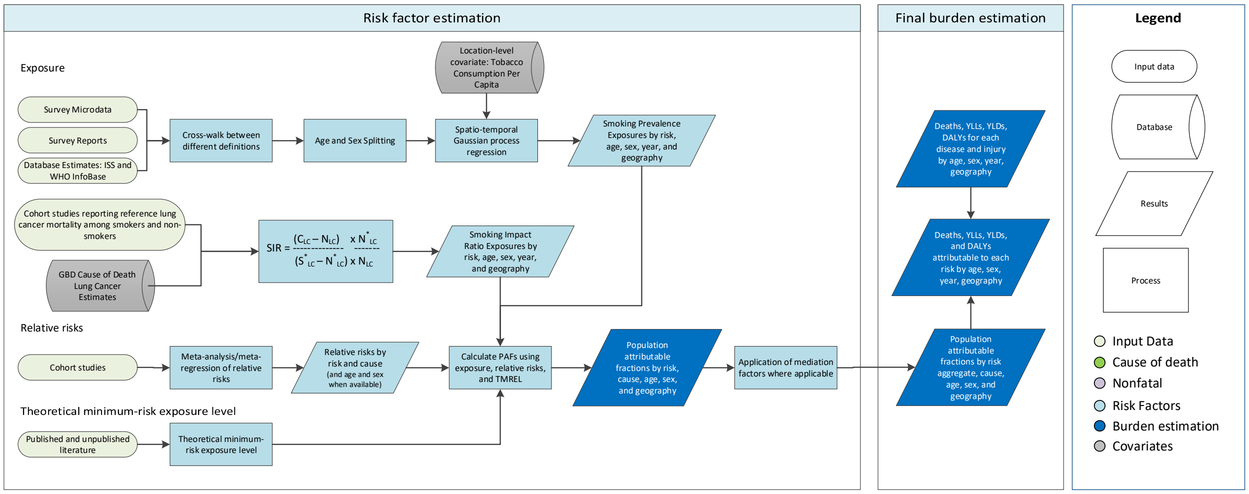

Input Data & Methodological Summary

Indicator definition

This modeling strategy encompassed the indicator associated with daily smoking prevalence (SDG indicator 3.a.1).

Indicator 3.a.1

As a component of SDG Goal 3. Ensure healthy lives and promote well-being for all at all ages, SDG Target 3.a, Strengthen the implementation of the World Health Organization Framework Convention on Tobacco Control in all countries, as appropriate, is measured using SDG indicator 3.a.1, daily smoking prevalence.

Inclusion criteria

We included nationally representative survey data sources that captured information on primary tobacco use among individuals over age 10. We included only self-reported smoking data and excluded data from questions asking about others' smoking behaviors. We included data that were collected between 1 January 1980 and 31 December 2016.

Data sources

A complete list of sources is available from the GBD 2016 Data Input Sources Tool.

Prevalence

We searched the Global Health Data Exchange (GHDx) database for primary data sources with the keyword "Tobacco Use" on 1 January 2017 to ensure all available data sources were captured. Of the 3,318 sources identified in the GHDx, 2,224 sources met inclusion criteria and were included.

In addition to the primary data sources identified through the GHDx, we supplemented with secondary database estimates from the WHO InfoBase Database and International Smoking Statistics Database for sources for which primary data are unavailable. We included 281 sources from the WHO InfoBase and 313 sources from the International Smoking Statistics Database.

Smoking Impact Ratio

The Smoking Impact Ratio (SIR) is computed using four estimates: 1) lung cancer mortality rates in a reference population of smokers, 2) lung cancer mortality rates in a reference population of never-smokers, 3) lung cancer mortality rates among never smokers in a population of interest, and 4) observed lung cancer mortality rates in a population of interest. We used available prospective cohort studies to estimate values 1, 2, and 3. A list of included prospective cohorts is available in the GBD 2016 Data Input Sources Tool. We used lung cancer mortality rate estimates from GBD 2016 for value 4.

Relative risk

Relative risk estimates were derived from prospective cohort studies. Sources used in relative risk estimation are reported in Appendix Table 6.

Smoking prevalence data preparation

Data extraction

We extracted primary data from individual-level microdata and survey report tabulations. We extracted data on current smoked tobacco use reported as any combination of frequency of use (daily, occasional, and current, which includes both daily and occasional smokers), type of smoked tobacco used (all smoked tobacco, cigarettes, hookah, and other smoked tobacco products such as cigars or pipes), and whether the data included only current smokers, only former smokers, or both current and former smokers, resulting in 36 possible combinations.

For microdata, we extracted relevant demographic information, including age, sex, location, and year, as well as survey metadata, including survey weights, primary sampling units, and strata. This information allowed us to tabulate individual-level data in the standard GBD five-year age-sex groups and produce accurate estimates of uncertainty. For survey report tabulations, we extracted data at the most granular age-sex group provided.

Crosswalking

Our case-definition for smoking prevalence is current daily use of any smoked tobacco products. All other data points were adjusted to be consistent with this definition. Some sources contained information on more than one indicator, and these sources were used to develop the adjustment coefficient to transform that alternative definitions to the GBD standard case-definition of daily use of smoked tobacco. The adjustment coefficient was the beta value derived from the following model:

$$p_{daily-smoked,k} = \beta p_{i,k} + \epsilon_k$$

where $p_{daily-smoked,k}$ is the prevalence of daily smoking reported in survey k and $p_{i,k}$ is the prevalence of an alternative frequency-type combination i also reported in survey k. Models with adjusted R-squared values > 0.8 were used in order of their R-squared value.

We propagated uncertainty at the survey (k) level from the crosswalk using the following equation:

$$PE_k = \sigma_\epsilon^2 + X_k^2 \text{var}(\hat{\beta})$$

where PE_k is the crosswalk prediction error that is added to the sampling variance of the data point, σ_ϵ^2 is the variance of the error, X_k^2 is the squared value of the data being adjusted, and $var(\hat{\beta})$ is the variance of the adjustment coefficient.

Age and sex splitting

We split data reported in broader age groups than the GBD five-year age groups or as both sexes combined by adapting the method reported in Ng et al. (http://jamanetwork.com/journals/jama/fullarticle/1812960) to split using a sex- geography- time-specific reference age pattern. We separated the data into two sets: a training dataset, with data already falling into GBD sex-specific five-year age groups, and a split dataset, which reported data in aggregated age or sex groups. Each source reporting aggregated data was temporarily duplicated into correct GBD categorizations covering their form of aggregation. These duplicated age groups were iteratively matched to the closest 200 sources of the same age and sex in the training dataset by geography and time. The mean of these 200 sources was used to generate an estimate for each duplicated estimate. Finally, we multiplied the original aggregated estimate by the population-weighted ratio of the mean estimates generated from the training data.

We defined the "closest" sources in space by assigning space weights based on GBD regions. If a training source was from the same country or subnational unit as the source to be split, it received a full space weight of 1. If from a different country but the same region, it received a space weight of 0.66. If the sources only shared a super-region, it received a space weight of 0.33. The time weights were generated using the equation:

$$\text{Time weight} = 1 - \text{abs}(\text{year}_{\text{train}} - \text{year}_{\text{split}}) * .05$$

Essentially, sources from the training dataset published in the same year as the source to be split would receive a full time weight of 1, with diminishing weight as the difference in publication years increased. The time weight and space weight each made up 50% of a combined total weight. The 200 training sources with the highest total weights were then used to estimate the mean prevalence pattern for each source in need of splitting.

Smoking prevalence modeling

We used ST-GPR to model smoking prevalence given the abundance of age and sex-specific data. Full details on the ST-GPR method are available elsewhere. Briefly, the mean function input to GPR is a complete time series of estimates generated from a mixed effects hierarchical linear model plus weighted residuals smoothed across time, space, and age. The linear model formula, fit separately by sex using restricted maximum likelihood in R, is:

$$\text{logit}(p_{g,a,t}) = \beta_0 + \beta_1 CPC_{g,t} + \sum_{k=2}^{19} \beta_k I_{A[a]} + \alpha_s + \alpha_r + \alpha_g + \epsilon_{g,a,t}$$

where $CPC_{c,t}$ is the tobacco consumption covariate, by geography g and time t, described above, $I_{A[a]}$ is a dummy variable indicating specific age group A that the prevalence point $p_{g,a,t}$ captures, and α_s, α_r, and α_g are super-region, region, and geography random intercepts, respectively. Random effects were used in model fitting but were not used in prediction.

We used out-of-sample cross validation for hyperparameter selection for the space (zeta), age (omega), and time (lambda) weights used in spatiotemporal smoothing along with the scale used in Gaussian process regression (details on the effects of different parameters have been previously published). We used a space weight of 0.95 in data-dense countries (at least five years covered in a geography-age-sex group) and space weight of 0.7 in data-sparse countries. The other parameters were consistent across data-density levels: age weight = 1, time weight = 1, and scale = 10.

Smoking Impact Ratio calculation

We calculated SIR for each geography, year, age group, and sex included in attributable burden analysis using the following formula:

$$SIR = \frac{C_{LC} - N_{LC}}{S_{LC}^{*} - N_{LC}^{*}} \times \frac{N_{LC}^{*}}{N_{LC}}$$

where C_{LC} is the lung cancer mortality rate specific to the age-sex-geography-year of interest, N_{LC} is the age-sex-geography-year-specific lung cancer mortality rate of never-smokers in the population of interest, S_{LC}^{*} is the lung cancer mortality rate in a reference population of smokers, N_{LC}^{*} is the lung cancer mortality rate in a reference population of never-smokers. Additional details on SIR calculation can be found elsewhere.

3.b.1 Vaccine Coverage SDG Capstone Appendix

Flowchart

3.b.1 Full Immunization Coverage Estimation Flowchart

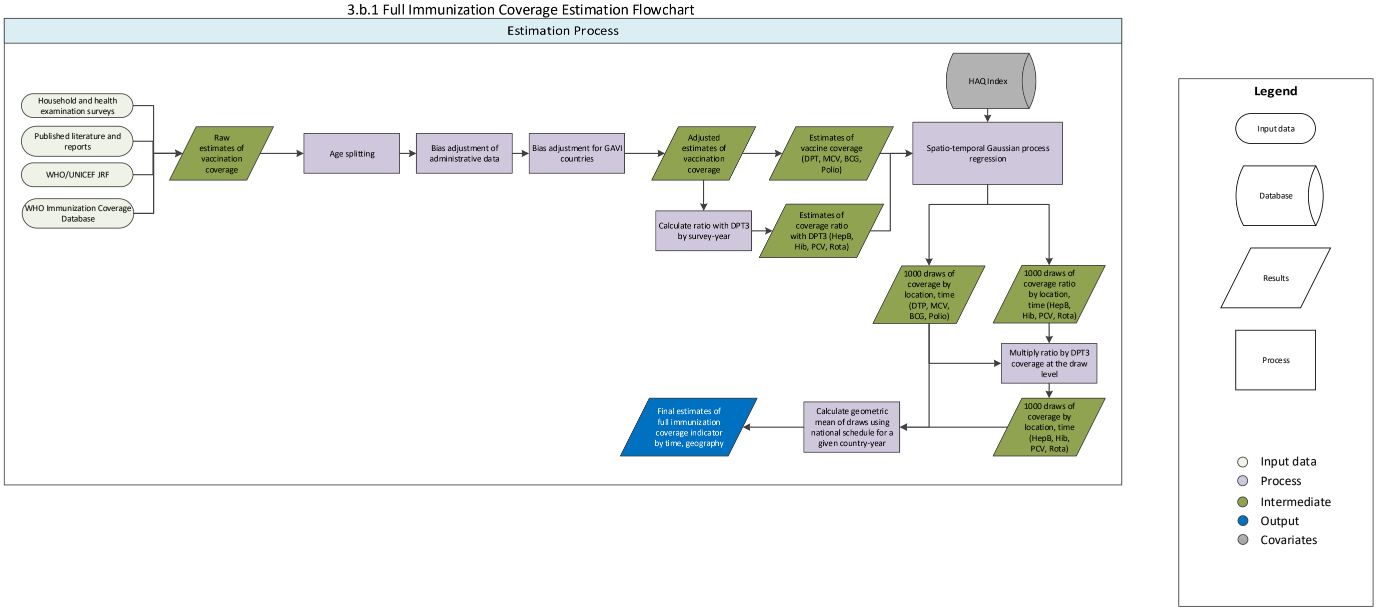

Input data & Methodological summary

Indicator definition

This modeling strategy pertains to the vaccine coverage measure (Indicator 3.b.1), the proportion of the target population covered by all vaccines included in the national program, including DPT (three doses), measles (one dose), BCG, polio vaccine (three doses), hepatitis B (three doses), *Haemophilus influenzae* type b (Hib, three doses), pneumococcal conjugate vaccine (PCV, three doses), and rotavirus vaccine (two or three doses). We use the geometric mean of coverage of these eight vaccines, based on their inclusion in the national vaccine schedule, to compute overall vaccine coverage of target populations

Indicator 3.b.1

As a component of Goal 3: Ensure healthy lives and promote well-being for all at all ages, Target 3.b: Support the research and development of vaccines and medicines for the communicable and non-communicable diseases that primarily affect developing countries, provide access to affordable essential medicines and vaccines, in accordance with the Doha Declaration on the TRIPS Agreement and Public Health, which affirms the right of developing countries to use to the full the provisions in the Agreement on Trade-Related Aspects of Intellectual Property Rights regarding flexibilities to protect public health, and, in particular, provide access to medicines for all., is measured using SGD Indicator 3.b.1: proportion of the target population covered by all vaccines included in their national programme.

Input data

The present study used data from household-level surveys as well as administrative reports of immunization coverage. Survey data which provided person-level information on immunization were identified and extracted. Major multi-country survey programs included in the analysis include the Demographic and Health Surveys (DHS),[1] Multiple Indicator Cluster Surveys (MICS),[2] Reproductive Health Surveys (RHS),[3] Living Standards Measurement Study (LSMS) surveys,[4] and World Health Surveys (WHS).[5] We also conducted a comprehensive search of the Global Health Data Exchange (GHDx),[6] as well as targeted internet searches and review of Ministry of Health websites, to identify national surveys and other multi-country survey programs.

Administrative estimates of immunization coverage were obtained from the Joint Reporting Process (JRF),[7] through which the World Health Organization (WHO) and UNICEF collate annual estimates of immunization coverage reported by UN member states. These immunization coverage estimates are separate from those synthesized by WHO, and are calculated by dividing the number of doses of a given vaccine delivered to the target population (i.e., children aged 12 to 23) by the number of individuals in that target population.

We excluded all data sources that were not nationally representative or had high levels of missingness. We applied survey weights based on survey sampling frames whenever they were available to generate weighted national estimates of vaccination coverage accompanied by estimates of standard error (SE). Estimates of SE, as well as sample sizes, were used to calculate uncertainty, as described below. Any point estimates with sample sizes less than 50 were reviewed to ensure that were not substantive outliers and would otherwise have an undue influence on our analysis.

Modeling strategy

Data processing

Age splitting

Most household surveys collect information on maternal and child health (MCH) indicators for children under 5 and/or mothers who gave birth within five years prior to the time of survey. To maximize data use for our model, we included immunization data for children aged 12 to 59 at the time of survey. Children younger than 12 months of age were excluded to minimize the influence of potentially censored observations. For each vaccine, coverage estimates were assigned to birth-cohort years based on a child's age prior to the time of survey: we used responses recorded for children aged 12 to 23 months for immunization coverage for one year prior to the time of survey, children aged 24 to 35 months for coverage two years prior to the time of survey, and so forth.

Age-specific estimates are easily computed from individual-level microdata, but many published reports and survey summaries present data in broader age aggregates (e.g., DPT3 coverage for children aged 12 to 35 months). To standardize these age groups, we applied an age-splitting model used in the GBD study,[8] as well as analyses that generated smoking and obesity prevalence by age group.[9,10]

Using surveys with microdata as the reference, we used the following model to generate standardized age group-specific estimates of immunization coverage:

$$\tilde{P}_{a,c,t,k} = P^{a+x}_{a,c,t,k} \frac{P_{a,c,t,j}}{P^{a+x}_{a,c,t,j}}$$

where $\tilde{P}_{a,c,k}$ is the adjusted estimate of coverage for target age group a in country c and year t of survey k; and $P^{a+x}_{a,c,k}$ is coverage reported from survey k, for country c in year t for the age group spanning age a to age $(a+x)$. The ratio of coverage between the target age group and broader age group from a survey j with microdata from the same country-year was used to split data from survey k. Surveys to be split were ideally matched with DHS or MICS surveys. If microdata were not available for the same year, ratios within five years of the survey that required age-splitting were applied.

Bias adjustments

Intervention coverage estimates based on administrative sources can be biased, yet the direction and magnitude of such biases are not universal. Some studies show that immunization coverage estimates from administrative data source are systematically higher than those of survey-based estimates,[11] while other studies show that bias directionality is more heterogeneous.[12] Such biases may arise for a number of reasons, including discrepancies in the accurate reporting of services or interventions provided (e.g., number of vaccine doses administered) and target population (e.g., number of children in need of vaccines), as well as capturing these data in a timely manner from both public and private sector facilities and healthcare providers.

For immunization coverage, we view individual-level data collected through population health surveys as the most accurate and least biased source of information of vaccination coverage, particularly for geographies with incomplete health information systems. We thus used vaccination coverage estimates from household surveys to calculate country-specific adjustment factors:

$$logit(P_{s,\,c,t}) = \beta_0 + \beta_1 logit(\tilde{P}_{a,\,c,t}) + \sum_{k=2}^{2+B} \beta_k S_k + \varepsilon_{c,t}$$

where $P_{s,\,c,t}$ is the survey-based estimate for immunization coverage (s) in country c for year t; $\tilde{P}_{a,\,c,t}$ is the administrative estimate for coverage in country c in year t; S_k is a spline basis used to capture the secular trend in coverage; β_1 is the estimated adjustment factor used to correct for the administrative bias; and ε is the error term for country c in year t.

Administrative estimates of immunization also may be subject to an additional bias from participation in performance-based health system support programs, such as the Gavi Immunization Services Support Program (Gavi ISS). It has previously been demonstrated that administrative estimates from participant countries are biased linearly with the number of year enrolled in the program.[13] To correct for this bias, we performed an additional bias adjustment on immunization coverage:

$$logit(P_{s,c,t}) = \beta_0 + \beta_1 logit(P_{a,c,t}) + \beta_2 T^{g}_{c,t} + \alpha_c + \varepsilon_{c,t}$$

where $P_{s,t}$is the survey-based estimate for immunization coverage (s) for country c in year t; $P_{a,t}$is the corresponding administrative coverage, T^{g}_{t} is the number of years of enrollment in the Gavi ISS program by year t; α_c is the country-specific random intercept to capture country-specific variation; β_2 is the

estimated adjustment factor used to correct for the Gavi bias by the number of years of participation; and ε is the error term for country c in year t.

To quantify uncertainty for bias-adjusted estimates from the mixed-effects models described above, we calculated prediction error, $\widehat{PE}$, as follows:

$$\widehat{PE} = X^2 var(\hat{\beta})$$

where $var(\hat{\beta})$ is the variance for the estimated fixed-effects coefficient of the adjustment factor and X is the independent variable. Proper estimation of prediction errors is crucial as the data synthesis procedure, Gaussian process regression (GPR) (as described in the subsequent section), accounts for uncertainty from point estimates and bias adjustments when generating fitted values. More weight is given to data with less uncertainty. Prediction errors estimated from the bias adjustment were incorporated into the data variance and propagated through the GPR step to obtain estimates of coverage and uncertainty intervals (UIs).

To assess the accuracy of our estimates in the bias adjustment, we performed cross-validation analyses by randomly holding out 20% of the sample and, if available, the corresponding administrative estimates for the given indicator of the same country and year, 10 separate times. We computed the average root mean squared errors (RMSE) across each country. Error in the bias adjustments was calculated as the mean difference between the adjusted administrative estimate for a given country, year, and corresponding survey-level estimates (which were considered the "gold-standard").

Trend estimation

We used a spatiotemporal Gaussian process regression (ST-GPR) to synthesize point estimates from multiple data sources and derive a complete time series for each vaccine. This method has been used extensively GBD and related studies, and accounts for uncertainty pertaining to each point estimate while borrowing strength across geographic space and time.[10, 11,15,16] Briefly, we assumed the Gaussian process was defined by a mean function *m*(•) and covariance function *Cov*(•).

We estimated the mean function using a two-step approach. Specifically, $m_c(t)$ can be expressed as:

$$m_c(t) = X\beta + h(r_{c,t})$$

where $X\beta$ is a linear model and $h(r_{c,t})$ is a smoothing function for the residuals; and $r_{c,t}$ is derived from the linear model. The following linear model was used to model DPT3, measles, BCG, polio coverage:

$$logit(P_{c,t}) = \beta_0 + \beta_1 \mathrm{HAQ_{c,t}} + \alpha_c + \gamma_{R[c]} + \omega_{\mathrm{SR[c]}} + \varepsilon_{c,t}$$

where $P_{c,t}$ is vaccination coverage for country c year t; $HAQ_{c,t}$ is value of the Healthcare Access and Quality Index[15] for country c and year t; α_c, $\gamma_{R[c]}$, and $\omega_{\mathrm{SR[c]}}$ are country, region, and super-region random intercepts, respectively. These estimates were then modeled through ST-GPR.

Given their recent introduction, there is limited coverage data for HepB, Hib, PCV, and rotavirus vaccines. To leverage the relatively data-rich DPT3 estimates, we modeled the ramp-up of each vaccine by modeling their ratio with DPT3 coverage. We first calculated the ratio of each particular vaccine with DPT3 by survey-year. We then modeled the full time series of the ratio using ST-GPR and ultimately

obtained estimates of coverage by multiplying the modeled ratio by the final estimated DPT3 coverage by location-year. The following linear model was used as the mean function for the HepB, Hib, PCV, and Rota ratio with DPT3:

$$logit(P_{c,i}) = \beta_0 + \beta_1 \mathrm{HAQ_{c,i}} + \alpha_c + \gamma_{R[c]} + \omega_{\mathrm{SR[c]}} + \varepsilon_{c,i}$$

where $P_{c,i}$ is the coverage ratio for country c time since introduction i; $HAQ_{c,i}$ is value of the Healthcare Access and Quality Index[15] for country c and time since introduction i; α_c, $\gamma_{R[c]}$, and $\omega_{\mathrm{SR[c]}}$ are country, region, and super-region random intercepts, respectively.

Random draws of 1,000 samples were obtained from the distributions above for every country for a given vaccine. Ninety-five percent uncertainty intervals were calculated by taking the ordinal 25th and 975th draws from the sample distribution.

To assess the accuracy of our modeled estimates, we performed cross-validation analyses using a knockout structure as previously described[16]. ST-GPR hyperparameters were selected on models that minimized the overall root-mean squared error (RMSE) of the model across a set of 10 knockouts.

Full Coverage Indicator

To synthesize the full vaccination coverage indicator (SDG indicator 3.b.1), we calculated the geometric mean of the eight vaccines based on their inclusion in the national vaccine schedule for a given year. Newer-generation vaccinations such as PCV and Rota are introduced in the country-year's calculation only after the vaccine has been introduced into the national schedule. Vaccines that are removed from the national schedule (e.g., BCG in several European countries, Australia, New Zealand) are included in the country's calculation up until the year that is is removed from the schedule.

National vaccine schedules and vaccine introduction dates were used as reported from WHO[17] or from the country's Ministry of Health website where otherwise unavailable. Dates of policy changes for the BCG vaccine were used as reported by the BCG Atlas[18] or directly form the country's ministry of health website.

References

1 Measure DHS: Demographic and Health Surveys. http://www.measuredhs.com (accessed Aug 11, 2015).

2 UNICEF Stat. Monit. Multiple Indicator Cluster Survey (MICS). http://www.unicef.org/statistics/index_24302.html (accessed Aug 11, 2015).

3 Cent. Dis. Control Prev. Reproductive Health Surveys (RHS). http://www.cdc.gov/reproductivehealth/Global/surveys.htm (accessed Aug 11, 2015).

4 World Bank. Living Standard Measruement Studies (LSMS). http://go.worldbank.org/UK1ETMHBN0 (accessed Aug 11, 2015).

5 WHO Multi-Ctry. Stud. Data Arch. World Health Survey (WHS). http://apps.who.int/healthinfo/systems/surveydata/index.php/catalog/whs/about (accessed Aug 11, 2015).

6 IHME GHDx. Global Helath Data Exchange. http://ghdx.healthdata.org/ (accessed Aug 11, 2015).

7 WHO | WHO/UNICEF Joint Reporting Process. WHO. http://www.who.int/immunization/monitoring_surveillance/routine/reporting/reporting/en/ (accessed Aug 17, 2015).

8 Prospective Studies Collaboration. Age-specific relevance of usual blood pressure to vascular mortality: a meta-analysis of individual data for one million adults in 61 prospective studies. *The Lancet* 2002; **360**: 1903–13.

9 Ng M, Freeman MK, Fleming TD, *et al.* Smoking Prevalence and Cigarette Consumption in 187 Countries, 1980-2012. *JAMA* 2014; **311**: 183.

10 Ng M, Fleming T, Robinson M, *et al.* Global, regional, and national prevalence of overweight and obesity in children and adults during 1980–2013: a systematic analysis for the Global Burden of Disease Study 2013. *The Lancet* 2014; **384**: 766–81.

11 Murray CJ, Shengelia B, Gupta N, Moussavi S, Tandon A, Thieren M. Validity of reported vaccination coverage in 45 countries. *The Lancet* 2003; **362**: 1022–7.

12 Haddad S, Bicaba A, Feletto M, Fournier P, Zunzunegui MV. Heterogeneity in the validity of administrative-based estimates of immunization coverage across health districts in Burkina Faso: implications for measurement, monitoring and planning. *Health Policy Plan* 2010; **25**: 393–405.

13 Lim SS, Stein DB, Charrow A, Murray CJ. Tracking progress towards universal childhood immunisation and the impact of global initiatives: a systematic analysis of three-dose diphtheria, tetanus, and pertussis immunisation coverage. *The Lancet* 2008; **372**: 2031–46.

14 Wang H, Liddell CA, Coates MM, *et al.* Global, regional, and national levels of neonatal, infant, and under-5 mortality during 1990–2013: a systematic analysis for the Global Burden of Disease Study 2013. *The Lancet* 13; **384**: 957–79.

15 Barber RM, Fullman N, Sorensen RJD, *et al.* Healthcare Access and Quality Index based on mortality from causes amenable to personal health care in 195 countries and territories, 1990?2015: a novel analysis from the Global Burden of Disease Study 2015. *The Lancet* 2017; published online May. DOI:10.1016/S0140-6736(17)30818-8.

16 Foreman KJ, Lozano R, Lopez AD, Murray CJ. Modeling causes of death: an integrated approach using CODEm. *Popul Health Metr* 2012; **10**: 1.

17 WHO | Data, statistics and graphics. WHO. http://www.who.int/immunization/monitoring_surveillance/data/en/ (accessed June 12, 2017).

18 The BCG World Atlas: A Database of Global BCG Vaccination Policies and Practices. http://journals.plos.org/plosmedicine/article?id=10.1371/journal.pmed.1001012 (accessed June 12, 2017).

5.2.1 Intimate Partner Violence SDG Capstone Appendix

Flowchart

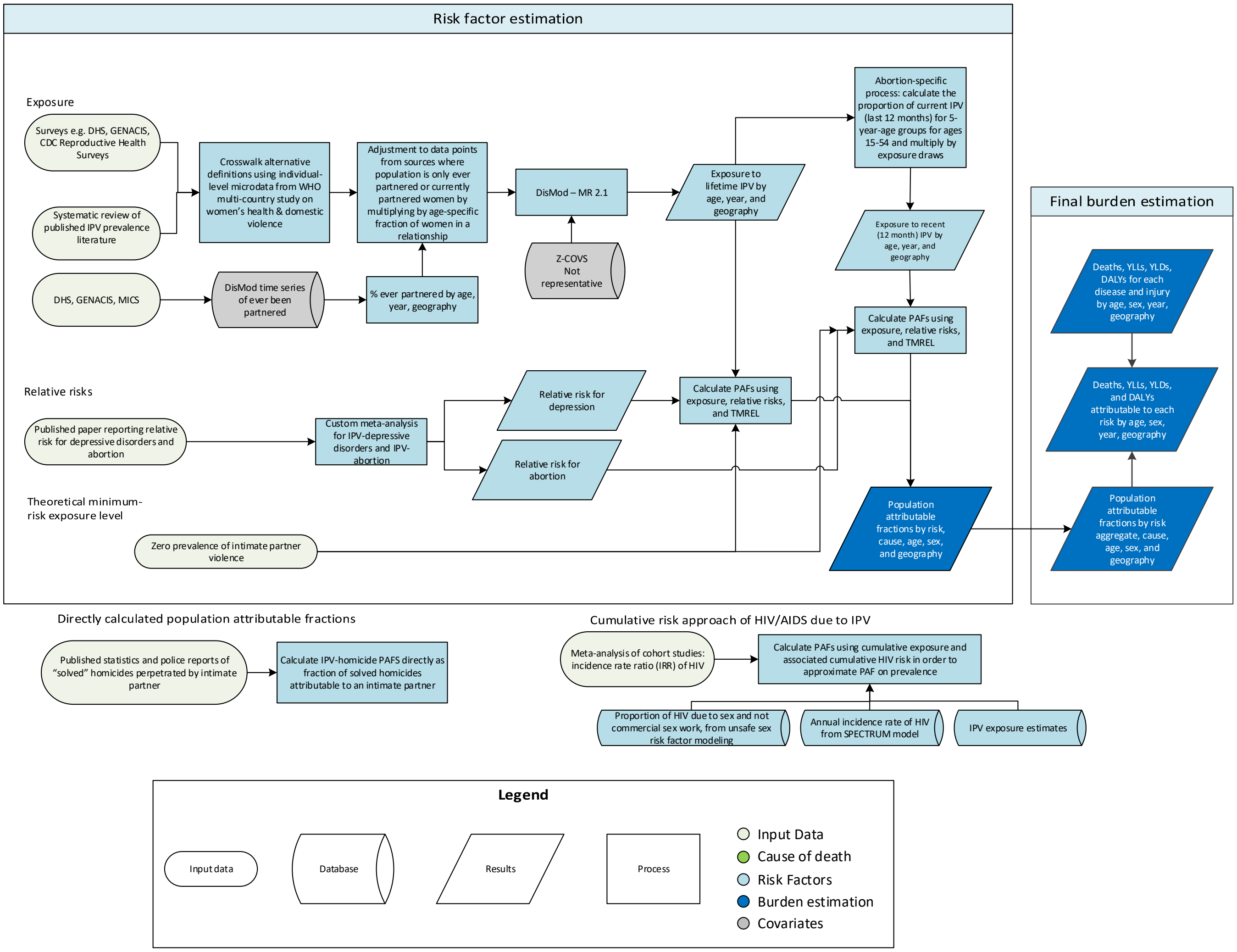

Input Data & Methodological Summary

Indicator definition

This modeling strategy encompassed the SDG indicator associated with prevalence of intimate partner violence (5.2.1).

Indicator 5.2.1

As a component of SDG Goal 5. Eliminate all forms of violence against all women and girls in the public and private spheres, including trafficking and sexual and other types of exploitation, SDG Target 5.2 eliminate all forms of violence against all women and girls in the public and private spheres, including trafficking and sexual and other types of exploitation, is measured using SDG Indicator 5.2.1, Prevalence of women aged 15 years and older who experienced intimate partner violence.

Exposure

Case Definition

The case definition for intimate partner violence (IPV) is ever experienced one or more acts of physical and/or sexual violence by a current or former intimate partner since the age of 15 years. Estimated in females only because evidence of risk-outcomes for males does not meet our criteria.

- Physical violence is defined as: "being slapped or having something thrown at you that could hurt you, being pushed or shoved, being hit with a fist or something else that could hurt, being kicked, dragged, or beaten up, being choked or burnt on purpose, and/or being threatened with or actually having a gun, knife, or other weapon used on you."
- Sexual violence is defined as: "being physically forced to have intercourse when you did not want to, having sexual intercourse because you were afraid of what your partner might do, and/or being forced to do something that you found humiliating or degrading" (the definition of humiliating and degrading may vary across studies depending on the regional and cultural setting).
- Intimate partner is defined as: "a partner to whom you are married or with whom you cohabit." In countries where people date, dating partners will also be considered (a partner with whom you have an intimate (sexual) relationship with but are not married to or cohabiting).

Input data

A systematic review of the intimate partner violence prevalence literature was conducted in PubMed for anything published between January 2016 and January 2017. The following search terms were used to conduct the systematic review:

((("health surveys"[MeSH Terms] AND prevalence[Title/Abstract]) OR ("sentinel surveillance"[MeSH Terms] AND prevalence[Title/Abstract]) OR ("prevalence"[Title/Abstract] AND cross sectional studies[MeSH Terms])) AND (abuse, sexual[MeSH Terms] OR domestic violence[MeSH Terms] OR abuse, partner[MeSH Terms] OR abuse, spousal[MeSH Terms] OR rape[MeSH Terms]) NOT ("comment"[Publication Type] OR "letter"[Publication Type] OR "editorial"[Publication Type]))

We get the proportion of solved homicides that were perpetrated by an intimate partner from crime statistics and police reports.

In GBD 2015, an updated systematic review was done for IPV homicide sources in PubMed through April 2016. The query used for this Pubmed search was:

((IPV[All Fields] OR ("intimate partner violence"[MeSH Terms] OR ("intimate"[All Fields] AND "partner"[All Fields] AND "violence"[All Fields]) OR "intimate partner violence"[All Fields])) AND (("homicide"[MeSH Terms] OR "homicide"[All Fields]) OR femicide[All Fields])) AND ("2013/01/01"[PDAT] : "3000/12/31"[PDAT])

These literature sources were supplemented with sources from the GHDx that were tagged with Intimate partner violence AND Homicide.

8.8.1 DALYs due to Occupational Risk Factors – SDG Appendix

Input Data and Methodological Summary

Indicator definition

This modeling strategy encompassed the indicator associated with DALY rates attributable to occupational risks (8.8.1).

Indicator 8.8.1

As a component of SDG Goal 8. Promote sustained, inclusive and sustainable economic growth, full and productive employment and decent work for all, SDG Target 8.8, protect labour rights and promote safe and secure working environments for all workers, including migrant workers, in particular women migrants, and those in precarious employment, is measured using SDG Indicator 8.8.1, age-standardised all-cause DALY rates (per 100,000) attributable to occupational risks.

Exposure

Definition

The following definitions were used for occupational risk factor exposures. All exposures were estimated only for ages 15+

Occupational Asbestos	Cumulative exposure to occupational asbestos using mesothelioma death rate as an analogue.
Occupational Asthmagens	Proportion of working population exposed to asthmagens based on distribution of the population in nine occupational groups
Occupational Carcinogens (arsenic, acid, benzene, beryllium, cadmium, chromium, diesel, formaldehyde, nickel, polycyclic aromatic hydrocarbons, secondhand smoke, silica, trichloroethylene)	Proportion of working population ever exposed to carcinogens in high or low exposures groups, based on distribution of the population in 17 economic activity groups
Occupational Injuries	Proportion of fatal injuries attributed to occupational work in seventeen economic activities, based on fatal injury rates in those economic activities.

Occupational Ergonomic Factors	Proportion of working population exposed to lower back pain, based on distribution of the population in nine occupational groups.
Occupational Noise	Proportion of working population exposed to 85+ decibels of noise, based on distribution in 17 economic activities.
Occupational Particulates	Proportion of working population exposed based on distribution in 17 economic activities

Estimates of the proportion of population involved in economic activities and occupations were coded into the following categories:

Economic Activities	Occupations
Agriculture, hunting, forestry	Legislators, senior officials, and managers
Fishing	Professionals
Mining and quarrying	Technicians and associate professionals
Manufacturing	Clerks
Electricity, gas, and water	Service workers and shop/market sales workers
Construction	Skilled agricultural and fishery workers
Wholesale and retail trade/repair	Plant and machine operators and assemblers
Hospitality	Craft and related workers
Transport, storage, and communication	Elementary occupations
Financial intermediation	
Real estate/renting	
Public administration/defense; compulsory social security	
Education	
Health and social work	
Other community/social/personal service activities	
Private households	
Extra-territorial organizations/bodies	

Input data

Primary inputs were obtained from the ILO [1-4], using raw data on economic activity proportions, occupation proportions, fatal injury rates, and employment to population ratio estimates. A systematic web review was conducted in order to collect the underlying microdata from the ILO's estimates to aid in re-extraction at lower levels of granularity. Where freely available, survey datasets were downloaded from the survey organizations in question. Other datasets were obtained through submission of requests to the agencies and through the GBD collaborator network. Microdata were tabulated in order to create survey-weighted estimates of economic activity and occupation for the GBD geographies and years. Various classification systems were crosswalked to ISIC Rev.3 (for economic activities) and ISCO 1988 (for occupations). Subnational estimates for UK and China were added to the datasets for economic activities and occupations [5-6].

For occupational asbestos, primary inputs were obtained through GBD 2016 cause of death estimates and published studies [7, 13-14].

Uncertainty for inputs where microdata were not available was generated by fitting a Loess curve to the data and determining the standard deviation of the data from the fitted curve.

Modeling strategies

A spatiotemporal Gaussian process regression was used to generate estimates for all year/locations for the primary inputs (see app section 2). Study-level covariates included for the prior model were education years per capita, geological covariates (for mining models), proportion of population living with access to coastline (for fishing models), the GBD Socio-demographic Index (SDI), mean temperature/latitude (for agriculture models), and proportion of population in urban areas. Space-time parameters were chosen by maximizing out-of-sample cross-validation and minimizing RMSE. For economic activity and occupation proportions, estimates from ST-GPR were then re-scaled to sum to 1 across categories by dividing each estimate by the sum of all the estimates.

The following sections describe the modeling approaches for each occupational risk's prevalence exposure.

<u>Occupational carcinogens, occupational noise, occupational particulates</u>

Prevalence of exposure to these risks was determined using the following equation:

$$Prevalence\ of\ Exposure_{c,y,s,a,r,l} = \sum_{EA} Proportion_{EA,c,y} * EAP_{c,y,s,a} * Exposure\ rate_{EA,r,l,d}$$

where:

EAP = Economically active population	c = country	r = risk
EA = economic activity	d = duration	s = sex
a = age	l = level of exposure	y =year

Exposure rate was provided by expert group recommendations and literature [8-11] (see table 1). The CAREX database was used in order to quantify the association between exposure by industry/carcinogen to SDI across all the countries in the database. This effect was used to predict exposure in countries that were not included in CAREX. Duration was considered for occupational carcinogens through application of occupational turnover factors [12] and for occupational noise and particulates by calculating cumulative exposure as the average exposure over the lifetime (past 50 years) for each age/sex cohort.

<u>Occupational ergonomic factors and asthmagens</u>

Prevalence of exposure to these risks was determined using the following equation:

$$Prevalence\ of\ Exposure_{c,y,s,a,r} = \sum_{EA} Proportion_{OCC,c,y} * EAP_{c,y,s,a}$$

where:

EAP = Economically active population	c = country	r = risk
OCC = occupation	a = age	s = sex
		y = year

<u>Occupational injuries</u>

Occupational injury counts were estimated using the following equation:

$$Occupational\ fatal\ injuries_{c,y,a,s} = \sum_{EA} Injury\ rate_{EA,c,y,s} * Population_{c,y,a,s} * EAP_{c,y,s,a} * Proportion_{EA,c,y}$$

where:

EAP = Economically active population	c = country	y = year
EA = economic activity	a = age	s = sex

<u>Occupational asbestos</u>

Prevalence of exposure to asbestos was estimated using the asbestos impact ratio (AIR), which is equivalent to the excess deaths due to mesothelioma observed in a population divided by excess deaths due to mesothelioma in a population heavily exposed to asbestos. Formally, this is defined using the following equation:

$$AIR = \frac{Mort_{c,y,s} - N_{c,y,s}}{Mort^{*}_{c,ys,} - N_{c,y,s}}$$

where:

Mort = Mortality rate due to mesothelioma	c = country
Mort* = Mortality rate due to mesothelioma in population highly exposed to asbestos	y = year
	s = sex
N = Mortality rate due to mesothelioma in population not exposed to asbestos	

Mortality rate due to mesothelioma was estimated from GBD 2015 causes of death [7]. Mortality rate due to mesothelioma in the population not exposed to asbestos was calculated using the model in Lin et al. [13], while the mortality rate due to high exposure to asbestos was estimated in Goodman et al. [14] Asbestos exposure prevalence created using the AIR was used to estimate PAFs for all associated causes except for mesothelioma. Custom PAFs were calculated for mesothelioma by using the ratio of excess mortality compared to the unexposed population (Mort – N) to the mortality rate in the population in question (Mort). This calculation assumes that all mesothelioma is a product of occupational asbestos exposure and could potentially overestimate burden due to occupational asbestos exposure in populations with high non-occupational asbestos exposure.

Theoretical minimum-risk exposure level

For all occupational risks, with the exception of occupational asbestos, the theoretical minimum-risk exposure level was assumed to be no exposure to that risk.

Relative risk

Relative risks were obtained for all occupational risks by conducting a systematic review of published meta-analysis. The estimates used, as well as the associated studies, are reported by category group in appendix table 5.

PAF

For all occupational risks, with the exception of injuries (outlined below) and mesothelioma (outlined above), PAFs were calculated using the prevalences estimated above, using the PAF formula in appendix section 2.

Occupational injuries PAF

The PAF for occupational injuries was calculated using the following formula:

$$PAF_{c,y,a,s} = \frac{Occupational\ fatal\ injuries_{c,y,a,s} - TMREL}{Fatal\ injuries_{c,y,a,s}}$$

where:

c = country	a = age
y = year	s = sex

Fatal injuries total was obtained from GBD 2016 causes of death [7].

References

[1] International Labour Organization (ILO). International Labour Organization Database (ILOSTAT) - Employment by Sex and Economic Activity. International Labour Organization (ILO).

[2] International Labour Organization (ILO). International Labour Organization Database (ILOSTAT) - Employment by Sex and Occupation. International Labour Organization (ILO).

[3] International Labour Organization (ILO). International Labour Organization Database (ILOSTAT) - Fatal Injuries by Sex and Economic Activity. International Labour Organization (ILO).

[4] International Labour Organization (ILO). International Labour Organization LABORSTA Economically Active Population, Estimates and Projections, October 2011. International Labour Organization (ILO), 2011.

[5] Office for National Statistics (United Kingdom). Nomis Official Labor Market Statistics - Annual Population Survey. Newport, United Kingdom: Office for National Statistics (United Kingdom).

[6] National Bureau of Statistics of China. China 1% National Population Sample Survey 1995. Ann Arbor, United States: China Data Center, University of Michigan.

[7] GBD 2015 Mortality and Causes of Death Collaborators. Global, regional, and national life expectancy, all-cause and cause-specific mortality for 249 causes of death, 1980–2015: a systematic analysis for the Global Burden of Disease Study 2015. *Lancet*.

[8] Wilson DH, Walsh PG, Sanchez L, *et al.* The epidemiology of hearing impairment in an Australian adult population. *Int J Epidemiol* 1999; 28: 247–52

[9] Kauppinen T, Toikkanen J, Pederson D, Young R, Kogevinas M, Ahrens W, et al. Occupational Exposure to Carcinogens in the European Union in 1990-93. Helsinki, Finland: Finnish Institute of Occupational Health; 1998.

[10] Kauppinen T, Toikkanen J, Pedersen D, Young R, Ahrens W, Boffetta P, et al. Occupational exposure to carcinogens in the European Union. *Occup Environ Med* 2000; 57(1): 10–18.

[11] Driscoll T, et al. The global burden of non-malignant respiratory disease due to occupational airborne exposures. *American Journal of Industrial Medicine 2005; 48*(6): 432-445.

[12] Nelson, D. I., Concha-Barrientos, M., Driscoll, T., Steenland, K., Fingerhut, M., Punnett, L. & Corvalan, C. (2005). The global burden of selected occupational diseases and injury risks: Methodology and summary. *American journal of industrial medicine*, *48*(6), 400-418

[13] Lin R-T, Takahashi K, Karjalainen A, *et al.* Ecological association between asbestos-related diseases and historical asbestos consumption: an international analysis. *Lancet* 2007; 369: 844–9.

[14] Goodman M, Morgan RW, Ray R, Malloy CD, Zhao K. Cancer in asbestos-exposed occupational cohorts: a meta-analysis. *Cancer Causes Control* 1999; 10: 453–65

16.1.3 Physical and Sexual Violence SDG Capstone Appendix

Input Data & Methodological Summary

Indicator definition

This modeling strategy encompassed the SDG health-related indicator associated with prevalence of physical or sexual violence (16.1.3)

Indicator 16.1.3

As a component of SDG Goal 16. Promote peaceful and inclusive societies for sustainable development, provide access to justice for all and build effective, accountable and inclusive institutions at all levels, SDG Target 16.1, by 2030, significantly reduce all forms of violence and related death rates everywhere, is measured using SDG Indicator 16.1.3, the proportion of the population subjected to physical, psychological, or sexual violence in the previous 12 months. For GBD 2016, we have excluded psychological violence from the measurement of this indicator due to data availability issues.

Input data

Input data come from three sources, listed below:

Demographic and Health Surveys (DHS)

In the Global Health Data Exchange (GHDx), we identified DHS that had variables related to sexual violence. The vast majority of the DHS only contain questions relating to intimate partner violence (SDG Indicator 5.2.1) and prevalence of sexual violence or physical violence (SDG Indicator 16.1.3)

The European Union Violence against Women Study

This violence against women study for the European Union provided data for many countries in the European Union. Questions related to sexual violence covered both intimate partner violence and non-partner sexual violence.

The United States Behavioral Risk Factor Surveillance System (BRFSS)

The BRFSS study has US state-level data. Not all states choose to expand their BRFSS survey to include sexual and physical violence questions, so the data included are only from a select number of states. The surveys included are from 2005, 2006, and 2007.

The figures below shows the input data density of unique source by location for indicator 16.1.3: prevalence of sexual or physical violence in the last 12 months for females (Figure 1) and males (Figure 2).

Figure 1. Input data density of unique source by location for indicator 16.1.3: prevalence of sexual or physical violence in the last 12 months (males).

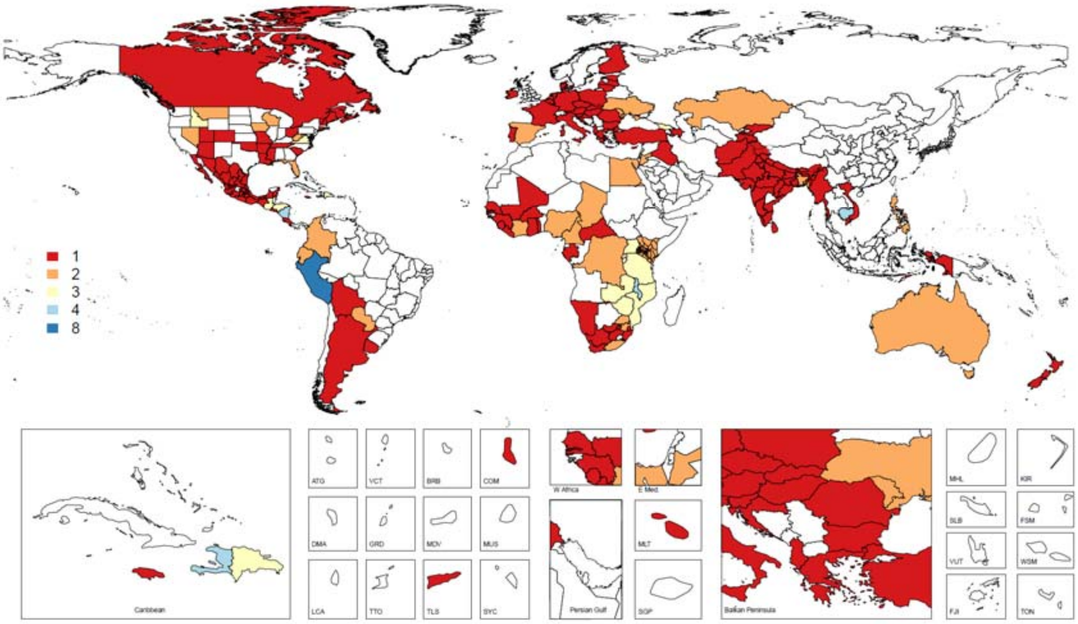

Figure 2. Input data density of unique source by location for indicator 16.1.3: prevalence of sexual or physical violence in the last 12 months (males).

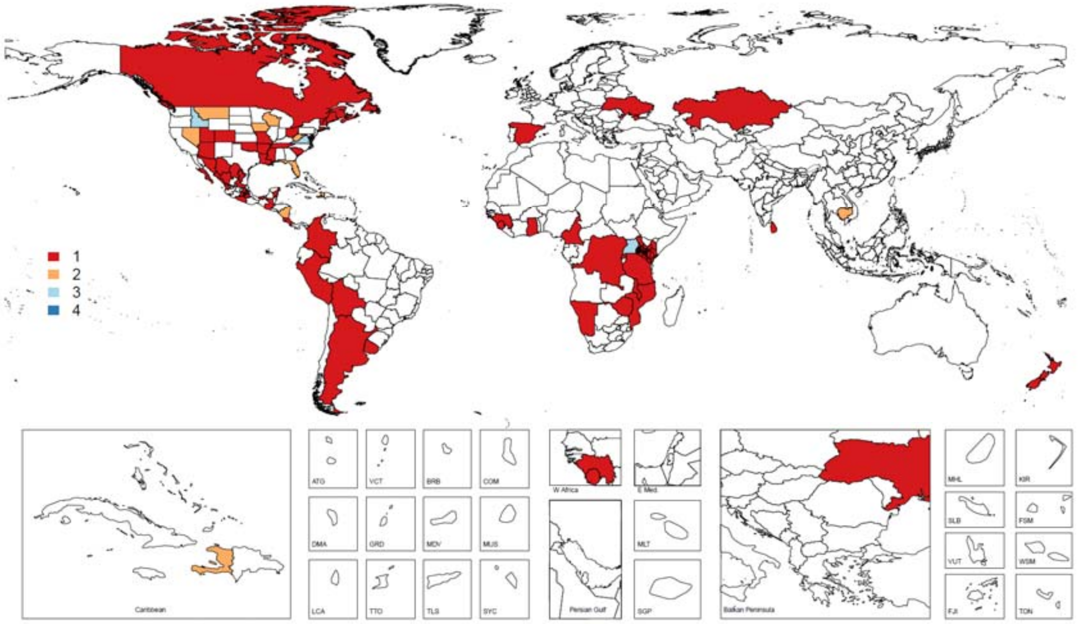

Modeling Strategy

Overall Modeling Strategy

In order to model the prevalence of this indicator with a 12-month recall, we used DisMod-MR 2.0, a descriptive epidemiological meta-regression tool. We used a single-parameter modeling approach within DisMod-MR 2.0 to fit the data for prevalence rather than modeling all of the estimates of disease simultaneously including incidence, prevalence, and remission like we did for overall sexual violence in the Global Burden of Disease framework.

The three main data sources ask questions in different ways, so we have included study-level covariates in order to adjust these data sources to the "gold standard" measure of prevalence in the last 12 months. These covariates are described in Table 1 for indicator models (intimate partner violence and prevalence of physical or sexual violence). We also present the exponentiated coefficient representing the magnitude of difference between the data points deviating from the gold standard from each of the indicator models (interpreted as a ratio: larger ratios indicate that the gold standard data points have lower estimates than those marked with the covariate, and smaller ratios indicate that the gold standard data points have higher estimates than those marked with the covariate). When appropriate, we pre-specified logical bounds for study-level covariates.

To inform estimates in areas where we do not have data, we included country-level covariates in the indicator models. The exponentiated coefficients for these country-level covariates are also included in Table 1. Where appropriate, we have also included the estimate of the sex covariate (where values < 1 indicate that the indicator is more prevalent in females).

Table 1. Study- and country-level covariates for DisMod-MR 2.0 yearly recall prevalence models for SDG indicators 5.2.1 and 16.1.3.

Covariate	Indicator 5.2.1: intimate partner physical or sexual violence among women	Indicator 16.1.3: physical or sexual violence
Study-level covariates		
Does not include sexual violence	0.70 (0.62 – 0.78)	0.72 (0.65 – 0.79)
Does not include physical violence	0.41 (0.28 – 0.64)	0.26 (0.22 – 0.31)
Ever-partnered people only (all people for Indicator 5.2.1)	0.87 (0.81 – 0.94)	1.11 (1.05 – 1.17)
Only includes partner sexual violence		0.99 (0.97 – 1.00)
Sex (male)		0.67 (0.63 – 0.71)
Country-level covariates		
Socio-demographic Index	0.30 (0.24 – 0.36)	0.31 (0.24 – 0.38)
Age-standardized, sex-specific homicide rate	1.13 (1.07 – 1.19)	1.16 (1.11 – 1.20)

16.2.3 Childhood Sexual Abuse SDG Capstone Appendix

Flowchart

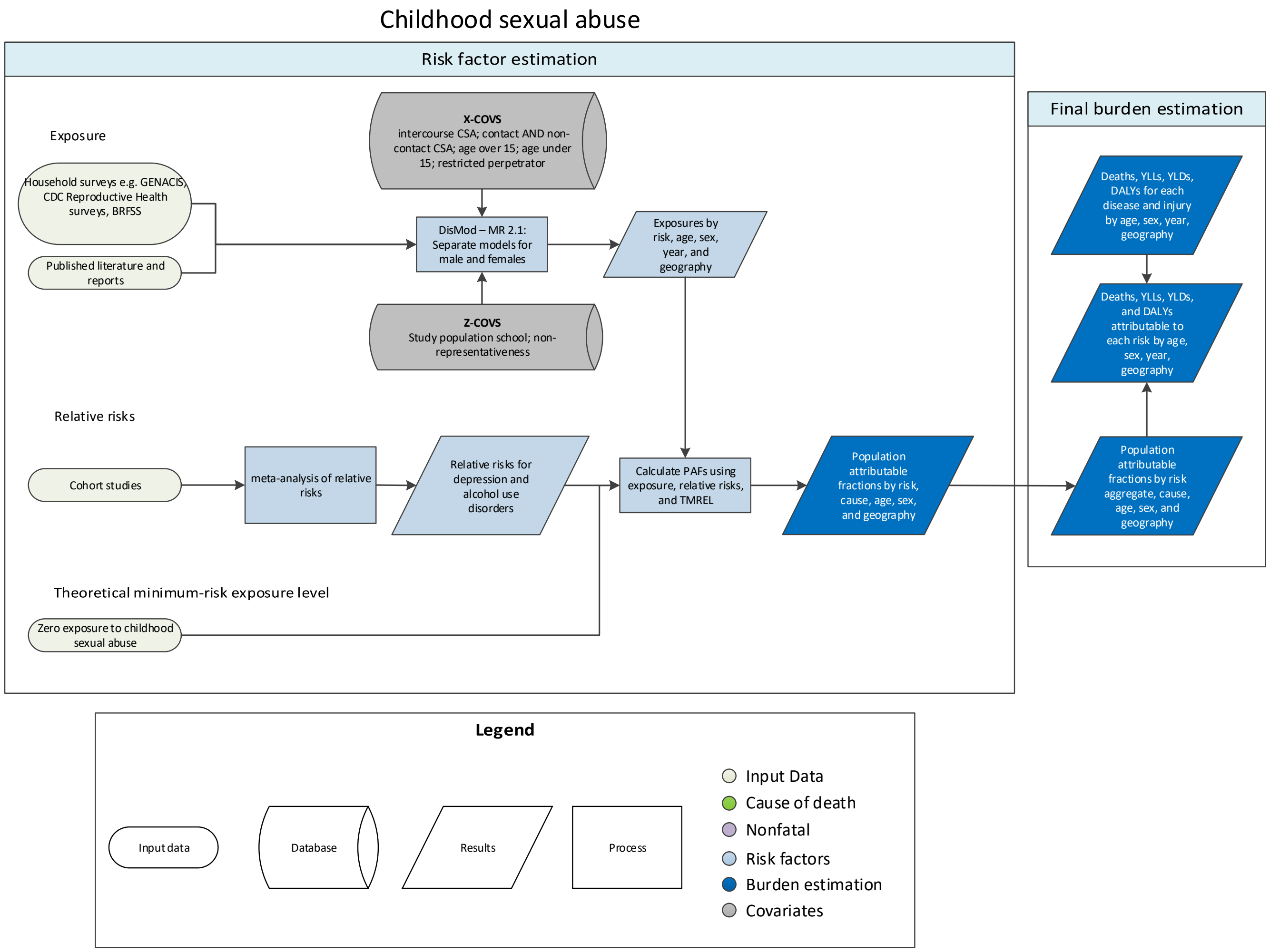

Input Data & Methodological Summary

Indicator definition

This modeling strategy encompassed the SDG health-related indicator associated with prevalence of childhood sexual abuse (16.2.3).

Indicator 16.2.3

As a component of SDG Goal 16. Promote peaceful and inclusive societies for sustainable development, provide access to justice for all and build effective, accountable and inclusive institutions at all levels, SDG Target 16.2, end abuse, exploitations, trafficking and all forms of violence against and torture of children, is measured using SDG Indicator 16.2.3, proportion of young women and men aged 18-29 years old who experienced sexual violence by age 18.

Exposure

Case Definition

The case definition for childhood sexual abuse (CSA) is ever having had the experience of intercourse or other contact abuse (i.e. fondling and other sexual touching) when aged 15 years or younger, and the perpetrator or partner was greater than five years older than the victim.

Input data

Currently, we use self-reported survey data to measure CSA prevalence, not data from Child Protection Services (CPS) or other crime data. The reliability and comprehensiveness of CPS and crime statistics varies too much geographically to warrant including it.

An updated systematic review of CSA prevalence literature was conducted for sources published between August 2015 and January 2017. The following search terms were used:

(((("health surveys"[MeSH Terms] AND prevalence[Title/Abstract]) OR ("sentinel surveillance"[MeSH Terms] AND prevalence[Title/Abstract]) OR ("prevalence"[Title/Abstract] AND cross sectional studies[MeSH Terms])) AND (("child abuse"[MeSH Terms] OR "child abuse, sexual"[MeSH Terms]) OR ("sex offenses"[MeSH Terms] OR "child abuse, sexual"[MeSH Terms]) OR (child*[Title/Abstract] AND sexual[Title/Abstract] AND abuse[Title/Abstract]))) NOT ("comment"[Publication Type] OR "letter"[Publication Type] OR "editorial"[Publication Type]))

We supplemented with data from relevant national health surveys and violence-specific surveys. Several survey series used include the United States Behavioral Risk Factor Surveillance System, the CDC Reproductive Health Surveys, Brazil National Alcohol and Drug Survey, and the Gender, Alcohol, and Culture International Study (GENACIS).

A number of study level covariates were also extracted that were used in the modelling process to adjust for heterogeneous definitions across sources. All crosswalks and adjustments were done in DisMod-MR 2.1.

Modeling strategy

CSA prevalence was modeled as a single parameter prevalence model in DisMod-MR. CSA exposure is modeled separately for males and females because we observe little correlation between the prevalence of child abuse among females and males, and modeling both sexes together causes unreasonable estimates in countries where we only have data for one sex.

Three study-level covariates were used for alternate definitions of the violence.

- Study asked only about intercourse CSA
- Study asked about contact and non-contact CSA
- Study placed restrictions on the relationship between the perpetrator and the victim (e.g. only asked about CSA committed by a father)

We also included study-level fixed effects for varying age thresholds across studies.

- Study asked about recall for events before ages above 15 years (versus reference age threshold of 15)
- Study asked about recall for events before ages less than 15 years (versus reference age threshold of 15)

Two study-level covariate fixed effects on variance (z-cov) were also included in both the male and female models, including an indicator that the survey was not nationally representative, as well as whether the survey was administered in schools. These study-level covariates were tested as x-covs first, but we did not find coefficients which would indicate systematic bias. We have not included any national-level covariates to date due to lack of knowledge about a covariate (for which we have a time series for all GBD locations) that predicts CSA prevalence.

References

1. Brown J, Cohen P, Johnson JG., and Smailes EM. Childhood abuse and neglect: specificity of effects on adolescent and young adult depression and suicidality. Journal of the American Academy of Child & Adolescent Psychiatry. 1999; 38(12): 1490-1496.
2. Chapman DP, Whitfield CL, Felitti VJ, Dube SR, Edwards VJ and Anda RF. Adverse childhood experiences and the risk of depressive disorders in adulthood. Journal of affective disorders. 2004; 82(2): 217-225.
3. Cheasty M, Clare AW and Collins C. Relation between sexual abuse in childhood and adult depression: case-control study. Bmj. 1998; 316(7126): 198-201.
4. Dinwiddie S, Heath AC, Dunne MP, Bucholz KK, Madden PA, Slutske WS, Bierut LJ, Statham DB, Martin NG. Early sexual abuse and lifetime psychopathology: a co-twin-control study. *Psychol Med*. 2000; 30(1): 41–52.
5. Dube SR, Anda RF, Whitfield CL, Brown DW, Felitti VJ, Dong M and Giles WH. Long-term consequences of childhood sexual abuse by gender of victim. American journal of preventive medicine. 2005; 28(5): 430-438.
6. Ernst C, Angst J, Földényi M. The Zurich Study. XVII. Sexual abuse in childhood. Frequency and relevance for adult morbidity data of a longitudinal epidemiological study. *Eur Arch Psychiatry Clin Neurosci*. 1993; 242(5): 293–300.
7. Fleming J, Mullen PE, Sibthorpe B, Attewell R and Bammer G. The relationship between childhood sexual abuse and alcohol abuse in women-a case-control study. Addiction. 1998; 93(12): 1787-1798.
8. Jaffee SR, Moffitt TE, Caspi A, Fombonne E, Poulton R, Martin J. Differences in early childhood risk factors for juvenile-onset and adult-onset depression. *Arch Gen Psychiatry*. 2002; 59(3): 215-22.
9. Kendler KS, Bulik CM, Silberg J, Hettema JM, Myers J, Prescott CA. Childhood sexual abuse and adult psychiatric and substance use disorders in women: an epidemiological and cotwin control analysis. *Arch Gen Psychiatry*. 2000; 57(10): 953–9.
10. Molnar BE, Buka SL and Kessler, RC. Child sexual abuse and subsequent psychopathology: results from the National Comorbidity Survey. American journal of public health. 2001; 91(5): 753.
11. Nelson EC, Heath AC, Madden PA, Cooper ML, Dinwiddie SH, Bucholz KK, Glowinski A, McLaughlin T, Dunne MP, Statham DJ, Martin NG. Association between self-reported sexual abuse and adverse psychosocial outcomes: results from a twin study. Arch Gen Psychiatry. 2002; 59(2): 139-45.

12. Peleikis DE, Mykletun A and Dahl AA. The relative influence of childhood sexual abuse and other family background risk factors on adult adversities in female outpatients treated for anxiety disorders and depression. Child Abuse & Neglect. 2004; 28(1): 61-76.
13. Sartor CE, Lynskey MT, Bucholz KK, McCutcheon VV, Nelson EC, Waldron M, Heath AC. Childhood sexual abuse and the course of alcohol dependence development: findings from a female twin sample. *Drug Alcohol Depend*. 2007; 89(2-3): 139–44.
14. Silverman AB, Reinherz HZ and Giaconia RM. The long-term sequelae of child and adolescent abuse: A longitudinal community study. Child abuse & neglect. 1996; 20(8): 709-723.
15. Widom CS, DuMont K and Czaja SJ. A prospective investigation of major depressive disorder and comorbidity in abused and neglected children grown up. Archives of general psychiatry. 2007; 64(1): 49-56.

17.19.2c Well-certified deaths SDG Capstone Appendix

GBD estimates are most accurate when computed with a full time series of complete vital registration with a low percentage of garbage codes. For GBD 2016, we developed a simple star-rating system from 0 to 5 to give a picture of the quality of data available in a given country over the full time series used in GBD estimates. Countries improve in the star rating as they increase availability, completeness, and detail of their mortality data and reduce the percentage of deaths coded to ill-defined garbage codes or highly aggregated causes.

To assign stars, we measure the proportion of deaths registered to a well-defined cause from 1980 to 2016. We call this proportion "percent well-certified". We measure this proportion for each location-year of vital registration and each verbal autopsy study separately, and then combine the yearly measurements into a percent well-certified for the full time series.

For each year of vital registration, percent well-certified is:

$$pct_{wellcertified} = completeness * (1 - pct_{majgarbage})$$

Where:

$$completeness = \frac{registered\ deaths}{GBD\ mortality\ envelope}$$

$$pct_{majgarbage} = \frac{deaths\ coded\ to\ level\ 1\ or\ 2\ garbage\ or\ highly\ aggregated\ cause}{registered\ deaths}$$

Simplifying this equation, one can see that in this case "percent well-certified" is simply the number of deaths that are registered to a well-defined cause (those codes which are not Level 1 or 2 garbage or highly aggregated) divided by the GBD mortality envelope.

ICD10 and ICD9 codes assigned to Level 1 or 2 garbage can be found in Appendix Table 3.

For each verbal autopsy data source, percent well-certified is:

$$pct_{wellcertified} = VerbalAutopsyAdjustment * (1 - pct_{majgarbage})$$

Where:

$$VerbalAutopsyAdjustment = SubAdj * RegAdj * AgeSexCoverage$$

And:

SubAdj:

10% for subnationally representative studies, 100% for nationally representative studies. This adjustment, while arbitrary in its specific value, reflects the bias that can be associated with studies that only cover a potentially non-representative sample of a country's population.

RegAdj:

64% for all verbal autopsy data sources. This accounts for the inaccuracy of verbal autopsy in assigning cause of death compared to medically verified vital registration.

The specific multiplier 0·64 is based on the chance-corrected concordance of Physician Certified Verbal Autopsy (PCVA) versus medical certification by the Population Health Metrics Research Consortium.[15]

Age-Sex Coverage:

The number of deaths estimated in the GBD mortality envelope for the ages and sexes in the study for the country and year divided by the number of deaths estimated in the GBD mortality envelope for the country and year. Studies that only cover children under 5 or maternal mortality, for example, will be highly discounted by this multiplier.

In the case of verbal autopsy, all garbage codes are considered ill-defined, as redistribution for verbal autopsy is highly imprecise. Causes such as "Injuries" or "Cancer" will also be included in major garbage percentage, as this percentage includes use of highly aggregated causes.

Once percent well-certified is calculated for each location-year of vital registration and each verbal autopsy study-year, we then combine these into one measurement for each five-year time interval and the full time series 1980–2016. For each five-year time interval, we take the maximum percent well-certified. Then for 1980–2016, we take the average of the maximum percentages well-certified for the seven five-year time intervals, including any five-year time interval where no data were available as a zero.

Once these values are calculated, we assign stars as follows:
5 stars: 85%–100% well-certified
4 stars: 65%–84% well-certified
3 stars: 35%–64% well-certified
2 stars: 10%–34% well-certified
1 star: >0%–9% well-certified
0 stars: No vital registration or verbal autopsy data available from 1980–2016

While stars are calculated for each five-year time interval as well as the full time series from 1980 to 2016, stars in the main text are presented for the full time series only.

Appendix Table 17 in the GBD 2016 Cause of Death Capstone shows the percent well-certified, stars, data source, and underlying values for percent well certified used for each country and time interval.

Part 2. SDG index construction and sensitivity analyses

In this analysis we have constructed indices that represent overall performance on: the health-related SDG indicators (referred to as the SDG index); the indicators that were previous MDG indicators (MDG index); and indicators that are newly added compared to the MDGs (non-MDG index).

For rate-space indicators, we first transformed the values to natural log space; proportion indicators were not transformed. The resultant indicator distributions were then rescaled to a 0 to 100 scale with 0 being the 2.5th quantile and 100 being the 97.5th quantile of the distribution of indicator values over the time period 1990 to 2030. The health-related SDG index was then computed by first determining the geometric mean of each rescaled health-related SDG indicator for a given target and then taking the geometric mean of the resulting values across the targets. This approach weights each of the health-related SDG targets equally and assumes partial substitutability with high values on one target partly compensating for low values on another target.

As a sensitivity analysis, for a second approach, we first take the arithmetic mean of indicator values for a given target and then the arithmetic mean of the resulting values across the targets. In contrast to the approach using the geometric mean, this approach assumes complete substitutability whereby poor performance on a target is linearly compensated for by better performance on another target. The resulting index using this approach was highly correlated with the approach using the geometric mean both in terms of 2016 values of the health-related SDG index (Methods Appendix Figure 1; Pearson correlation coefficient = 0.99, $p<0.0001$) as well as the corresponding country ranks (Methods Appendix Figure 2; Spearman's rank correlation coefficient=0.97, $p<0.0001$).

For the third approach, we first take the geometric mean of indicator values for a given target then determine the minimum of the resulting values across the health-related targets. This approach adopts what is called zero substitutability, such that better performance on one target in no way compensates for poor performance on another target. The resulting index using this approach was also well correlated with the approach using the geometric mean both in terms of 2016 values of the health-related SDG index (Methods Appendix Figure 3; Pearson correlation coefficient = 0.78, $p<0.0001$) and country rank (Methods Appendix Figure 4; Spearman's rank correlation coefficient=0.79 $p<0.0001$), although not as highly as the approach taking the arithmetic mean.

Part 3. Projections for the health-related SDGs

Section 1. Overall projection modeling strategy

We projected the health-related Sustainable Development Goal (SDG) indicators based on past trends. We first calculated for each country the change in each year from 1990 to 2016 in natural-log space or, for indicators bounded between 0 and 1 (eg, intervention coverage, percentage of population) in logit-space.

We then calculated the median annualised rate of change for each country, with monotonically increasing temporal weights to favor more recent trends (with t being the years between the start and end of the time-series [1990 to 2007 for predictive validity, 1990 to 2016 when calculating final projections]):

$$weight_{year} = \frac{(year - 1990)^{\omega}}{\sum_{t=1990}^{T}(year - 1990)^{\omega}}$$

To determine the indicator specific value of ω, we undertook an out-of-sample validity test by holding out data from 2008-2016 and using data from 1990-2007 to predict values for the 2008-2016 period. We tested ω values ranging from 0 to 2, in increments of 0.2. We chose the value of ω specific to each indicator that minimized the root mean squared error (RMSE) for the time period 2008-2016; the weights selected by this process are summarized below.

We then use that weight function to determine the median annualised rate of change for each of the 1,000 draws of a given indicator. In addition, for each of the draws we allow for year-to-year deviation from the median rate of change based on the variance across all draws. This resulted in 1,000 rate of change draws by country for each year from 2017-2030, which are then applied in a stepwise manner to produce the projected time series. Lastly, we scaled the 1,000 draws such that the mean of the draws for each country-year was equal to the value projected using the mean alone.

The exception to the above was for natural disasters given the stochastic nature of the cause we used the long-term average over the 1990 to 2016 period to project the rate to 2030.

Table 1. Weights selected with out-of-sample validity testing for projecting the health-related SDGs. Disaster mort=Mortality due to exposure to forces of nature. MMR=Maternal mortality ratio. SBA=Skilled birth attendance. Under-5 mort=Under-5 mortality. Neonatal mort=Neonatal mortality. HIV incid=HIV incidence. Tuberculosis incid=Tuberculosis incidence. Malaria incid=Malaria incidence. Hep B incid=Hepatitis B incidence. NTD prev=Prevalence of 15 neglected tropical diseases. NCD mort=Mortality due to a subset of non-communicable diseases (cardiovascular disease, cancer, diabetes, and chronic respiratory diseases). Suicide mort=Mortality due to self-harm. Road injury mort=Mortality due to road injuries. FP need met, mod=Met need for family planning with modern contraception methods. Adol birth rate=Adolescent birth rate. UHC index=universal health coverage index. Air poll mort=Mortality attributable to household air pollution and ambient air pollution. WaSH mort=Mortality attributable to unsafe water, sanitation, and hygiene. Poisoning mort=mortality due to unintentional poisonings. Smoking prev=prevalence of daily smoking. Vaccine cov=Vaccine coverage of target populations based on national vaccine schedules. Int partner viol=Intimate partner violence. HH air poll=Household air pollution. Occ burden=Disease burden attributable to occupational risks. Mean PM2.5=Mean particulate matter smaller than 2.5 microns in diameter. Homicide=Mortality due to interpersonal violence. Conflict mort=Mortality due to conflict and terrorism. Violence prev=Prevalence of physical or sexual violence. Child sex abuse=Childhood sexual abuse. Cert death reg=Well-certified death registration.

SDG indicator	Weight function	SDG indicator	Weight function
Disaster mort	N/A	UHC index	2.0
Child stunting	0.4	Air Poll mort	1.6
Child wasting	0.4	WaSH mort	1.6
Child overweight	0.4	Poisoning mort	2.0
MMR	2.0	Smoking prev	0.4
SBA	2.0	Vaccine cov	2.0
Under-5 mort	2.0	Int partner viol	1.6
Neonatal mort	2.0	Water	1.8
HIV incid	N/A	Sanitation	1.4
Tuberculosis Incid	1.6	Hygiene	0.4
Malaria incid	0.4	HH air poll	1.8
Hep B incid	1.6	Occ burden	2.0
NTD prev	2.0	Mean PM2.5	0.0
NCD mort	1.8	Homicide	1.8
Suicide mort	2.0	Conflict mort	0.8
Alcohol use	2.0	Violence prev	2.0
Road injury mort	2.0	Child sex abuse	0.6
FP need met, mod	0.6	Cert death reg	0.4
Adol birth rate	1.4		

Section 2. Projecting HIV and ART coverage for the health-related SDGs

Producing ART Coverage Caps

In recent years, we have seen a massive scale up of ART treatment among low-income nations, who through large internal investments and substantial development assistance have been able to scale up ART access considerably. For that reason, if the past trends in ART coverage for each country are simply scaled up in projections using a logistic curve, all countries would be projected to achieve 100% coverage by 2040. Given limitations on coverage by health system capacity, and due to the cost of treatment, we bound ART projections with a frontier by income level to reflect resource availability.

Cross-walking Cross-Sectional and Spectrum CD4 Definitions

In order to model the relationship between income and ART coverage, we must also consider CD4 count as a major stratifying variable. As CD4 count defines eligibility for ART treatment in many locations, and individuals who are sicker (with lower ART counts) are more likely to have received a diagnosis and receive treatment, taking CD4 count into account is essential. Survey data provides cross-sectional CD4 count information. However, the Spectrum modeling framework tracks individuals by categorical CD4 count at the initiation of treatment, for individuals who are currently receiving ART. Therefore, in order to model the relationship between ART coverage and income in a CD4 specific fashion usable in Spectrum, we developed a method to crosswalk cross-sectional CD4 values to CD4 at treatment initiation, using information regarding the years each individual has been on treatment, and cohort information about the average CD4 progression pattern on treatment.

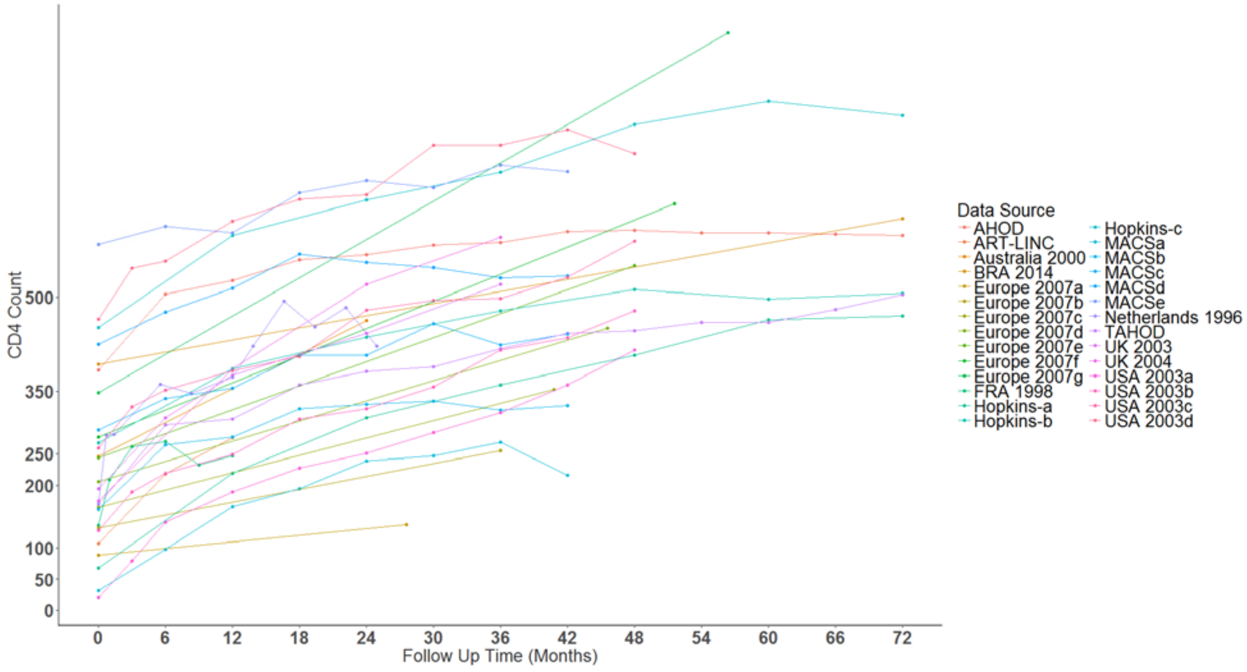

Figure 1. Cohort data used to fit the CD4 count progression model

We extracted information on the average CD4 progression over time after the initiation of ART treatment from a number of cohort studies (figure 1). We used a natural spline model to find the average

progression rate over time. Our outcome variable $Y_{g,ts}$was the difference in the average CD4 count for a cohort *I* at time *t* from the value at the beginning of treatment, time *s:*

$$Y_{i,t} = CD4_{i,t} - CD4_{i,s}$$

We model this change over time using the following model:

$$Y_{g,ts} = (S_1 Time * CD4_{i,s}) + S_2 Time$$

Where $S_2 Time$ is a natural spline on the number of months since treatment initiation, and $(S_1 Time * CD4_{i,s})$ is a natural spline on the number of months interacted with the starting average CD4 count of the cohort. Both spline bases use knots at 3,12,24, and 36 months. The natural spline term captures the overall growth over time in CD4 count with ART treatment, while the interaction term captures the slow convergence effect that occurs over time as all individuals tend to stabilize at similar values regardless of starting CD4 counts. Figure 2 shows the model fit, for each of the CD4 cutpoints used to define compartmental categories in the Spectrum modeling framework (0-49,50-99,100-199,200-249,250-349, 350-500, and 500+) alongside the model input data.

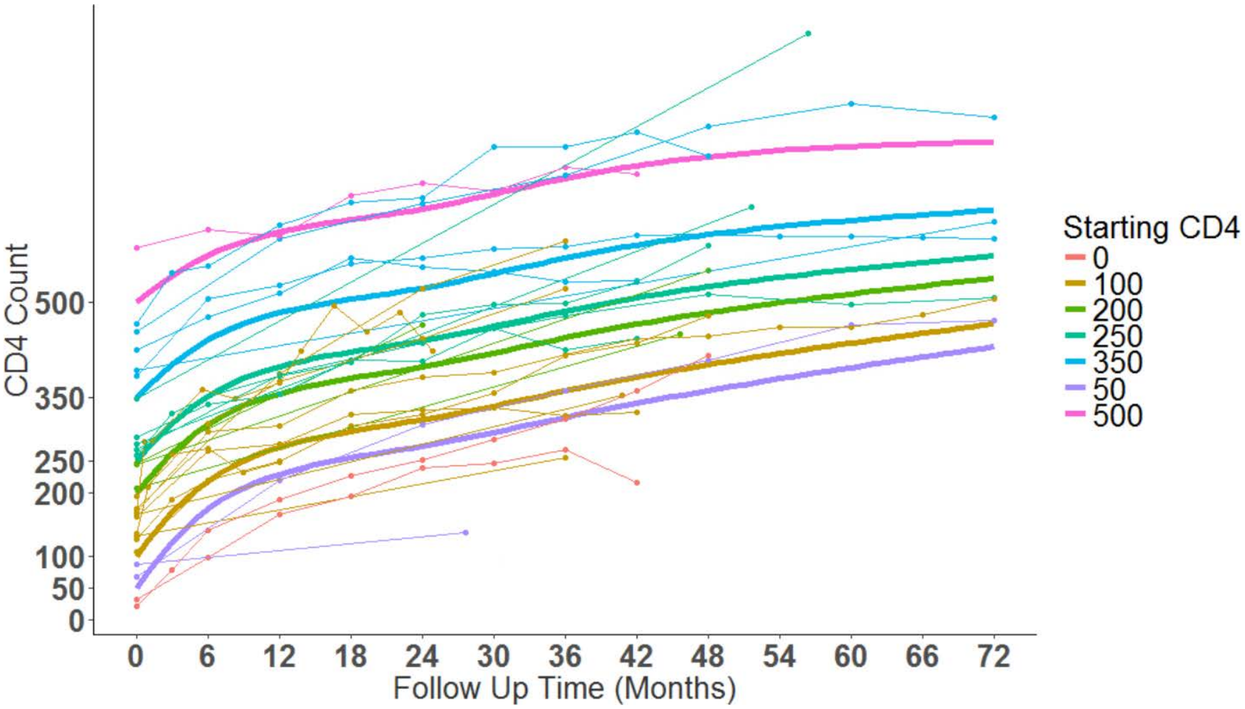

Figure 2. Model fit for each categorical cutpoint used in the Spectrum modeling framework.

We then use the progression curves from this model to categorically backcast each individual observed in our cross-sectional survey data sources to one of the aforementioned categories (figure 3).

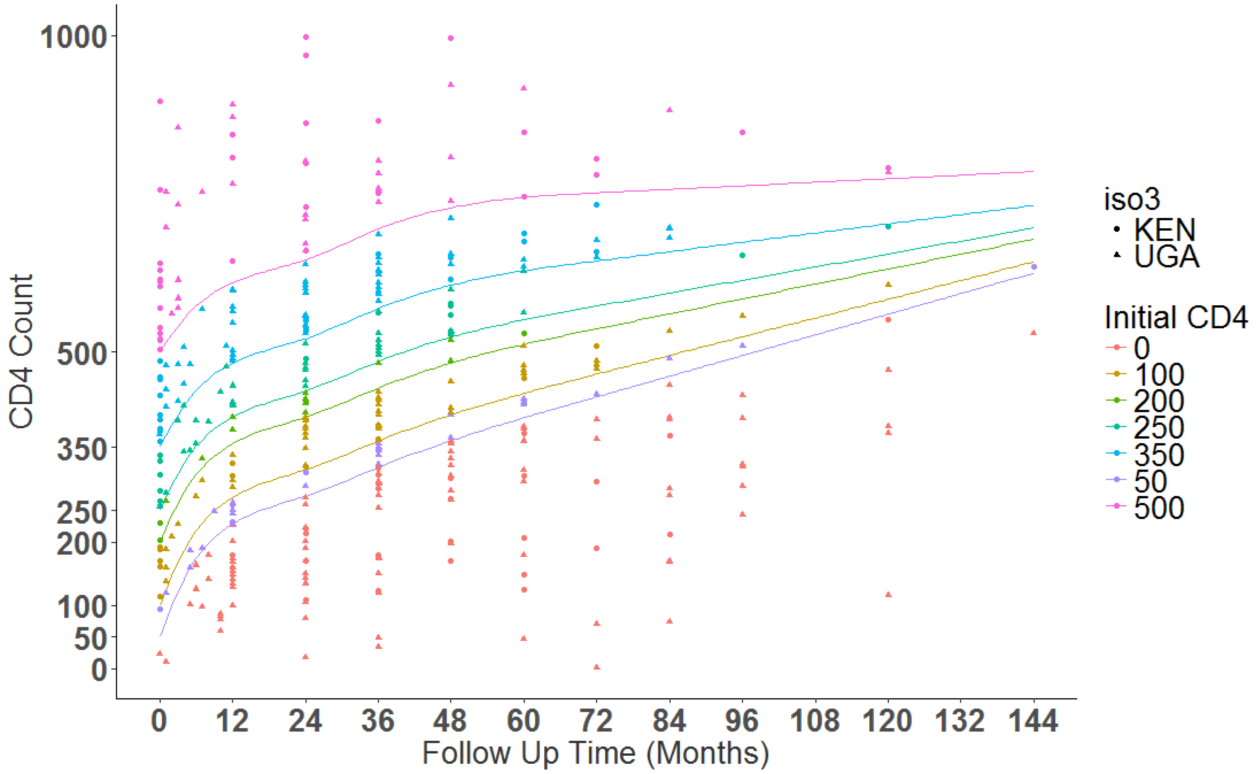

Figure 3. Categorical backcast of survey microdata using modelled progression curves.

Modeling Coverage Frontier as a Function of Income and CD4 Count

We identified two publically available survey datasets that provide person-level information regarding the distribution of ART coverage by CD4 count. We used data from the 2011 Uganda and 2012 Kenya Aids Indicator Surveys, subsetting the analysis to only individuals who were determined to be HIV positive from laboratory tests. ART coverage is defined as a binary variable, representing if the surveyed individual was currently taking ART medication at the time of the survey. CD4 information for each participant was obtained from laboratory test values, and crosswalked to the Spectrum definition as described in the previous section. As a proxy for income, we used a household asset index based on assets present in the respondent's home. This asset-based index is converted to international dollars income (cite Nick's work). A logistic curve describing the relationship between ART coverage probability and income is then fit, controlling for CD4 count, age and sex, using a logistic regression:

$$P_{coverage} = \beta_1 Income + \beta_2 CD4 + \beta_3 Age + \beta_4 Female$$

The predicted probabilities from this model are the used to fit a stochastic frontier analysis, which estimates the maximum possible coverage for a given degree of income and CD4 count. The probability of coverage values from the first model are logit transformed to ensure the final frontier predictions cannot exceed one. An offset is added to each value to bring each logit-transformed value above zero, and then the values are logged for the stochastic frontier analysis. Formally, we estimate:

$$\log(logit(P_{coverage}) + offset) = \beta_1 Income + \beta_2 CD4$$

The stochastic frontier analysis assumes that inefficiencies are distributed according to a truncated normal distribution. Figure 4 shows the predicted probabilities and model fit for each CD4 group from the stochastic frontier analysis, after the frontiers have been transformed back to probability space using the reverse of the aforementioned transformation.

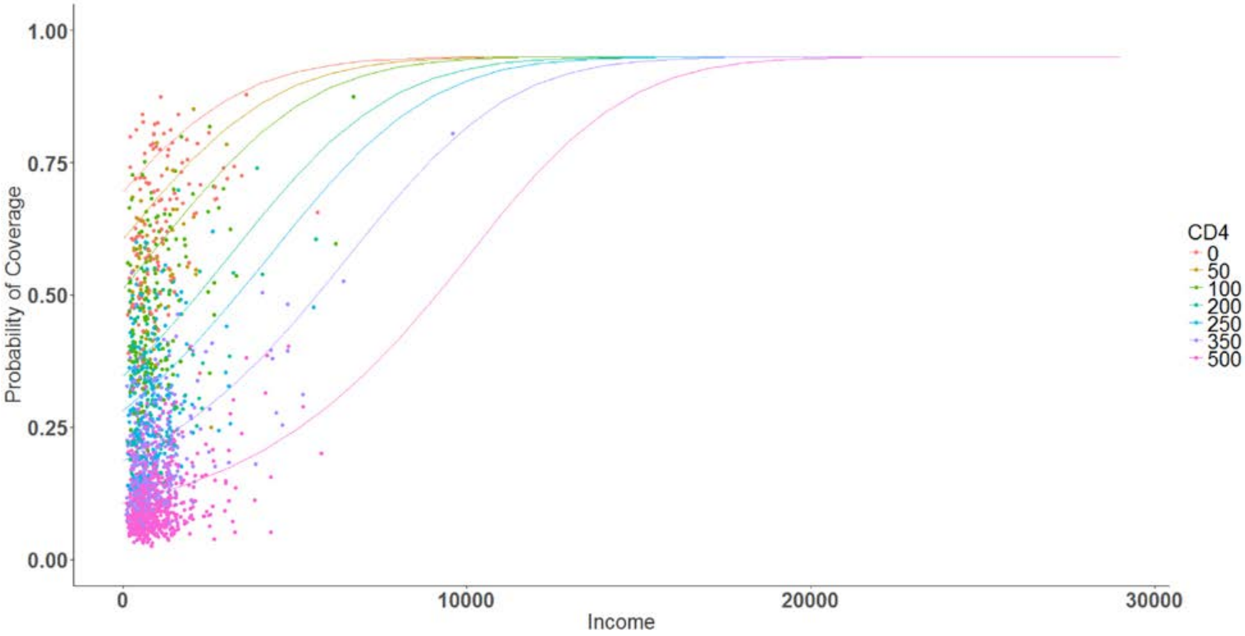

Figure 4. Predicted probabilities of coverage for each individual shown as points. Frontier of coverage as a function of income shown with lines. Color indicates categorical CD4 count.

Projecting ART Prices

In order to project ART coverage, an understanding of the cost of ART treatment over time is necessary. We created estimates and projections of the average cost of ART treatment using data from the Global Price Reporting Mechanism (GPRM)[1]. From the GPRM we obtained 1,175 country-years of data representing the average cost of ART in dollars per person per year, covering 130 countries and spanning 2004-2016. We used a stochastic frontier analysis and Gaussian process regression modelling framework to complete the timeseries and project the estimates through 2040

Stochastic Frontier Analysis

In order to bound the future minimum cost plausibly, we use a stochastic frontier analysis to model the minimum ART price possible over time. In this case, the secular trend should be viewed as representing the inherent decrease in cost of ART with improved technology. First we create our outcome variable by transforming cost, by rescaling to an inverse zero to one scale, where 0 is the lowest observed cost and 1 is the highest. This is necessary as the stochastic frontier analysis function is used to find a maximum value, and therefore the outcome must be rescaled to find a minimum cost frontier. We then take the logit of this transformed cost variable, which creates our outcome variable:

$$Y_{c,t} = logit\left(\frac{Cost_{i,t} - \min(Cost_{i,t})}{range(Cost_{i,t})} + offset\right)$$

We use an offset value of .000001 to prevent true zero values, which cannot be logit transformed. We then fit a stochastic frontier analysis, with time as the independent variable, assuming a truncated normal distribution for the inefficiencies:

$$Y_{c,t} = \beta_1 Year$$

Figure 5. illustrates the transformation used to create the outcome variable, as well as the stochastic frontier analysis fit.

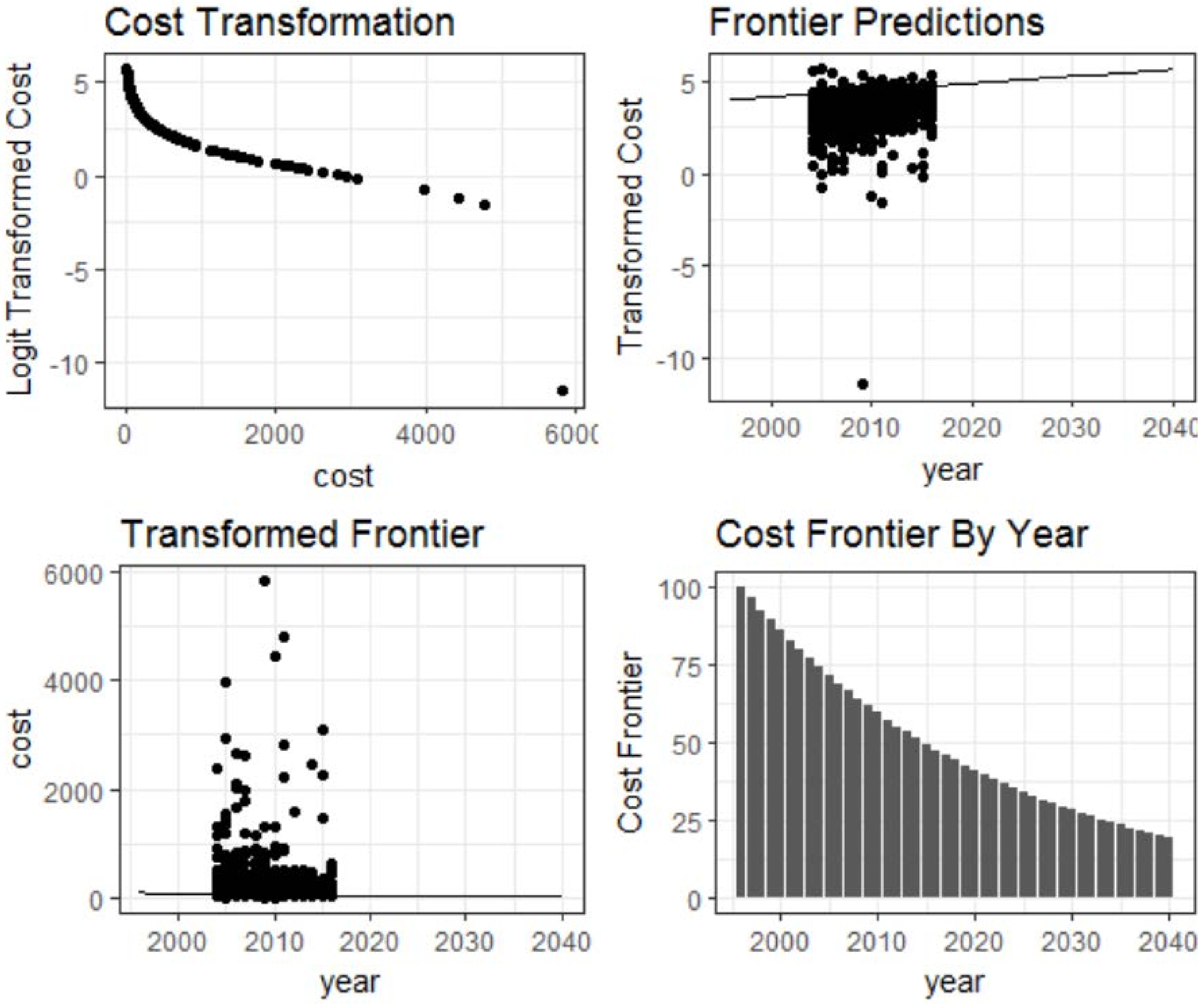

Figure 5. Illustrations of the transformed cost of ART prices, and the frontier model fit and final estimates.

Gaussian Process Regression

We used Gaussian process regression (GPR) to complete the timeseries and make projections through the year 2040, ensuring that the estimates fit the data well. GPR has been used extensively in the Global Burden of Disease estimation framework as a data synthesis tool [2,3]. GPR uses a covariance function to smooth the residuals from a linear model prior. GPR also synthesizes both data and model uncertainty, in order to produce estimate uncertainty intervals. GPR assumes that the trend in the underlying data

follows a Gaussian process, which is defined using a mean function $m(\cdot)$ and a covariance function $Cov(\cdot)$. The mean function is a linear model which models the log of the difference between the cost frontier and the current cost, as a function of lag-distributed GDP per capita (LDI) [4] and super-region secular trends:

$$\log\left(Cost_{c,t} - frontier_t\right) = \beta_1 LDI + \beta_r Year$$

Consistent with prior implementations of GPR, A matern covariance function was used to smooth the residuals from the first stage mean function, and produce complete time series with uncertainty[3]. Figure 6 shows the median and IQR of ART cost globally, as well as the cost frontier.

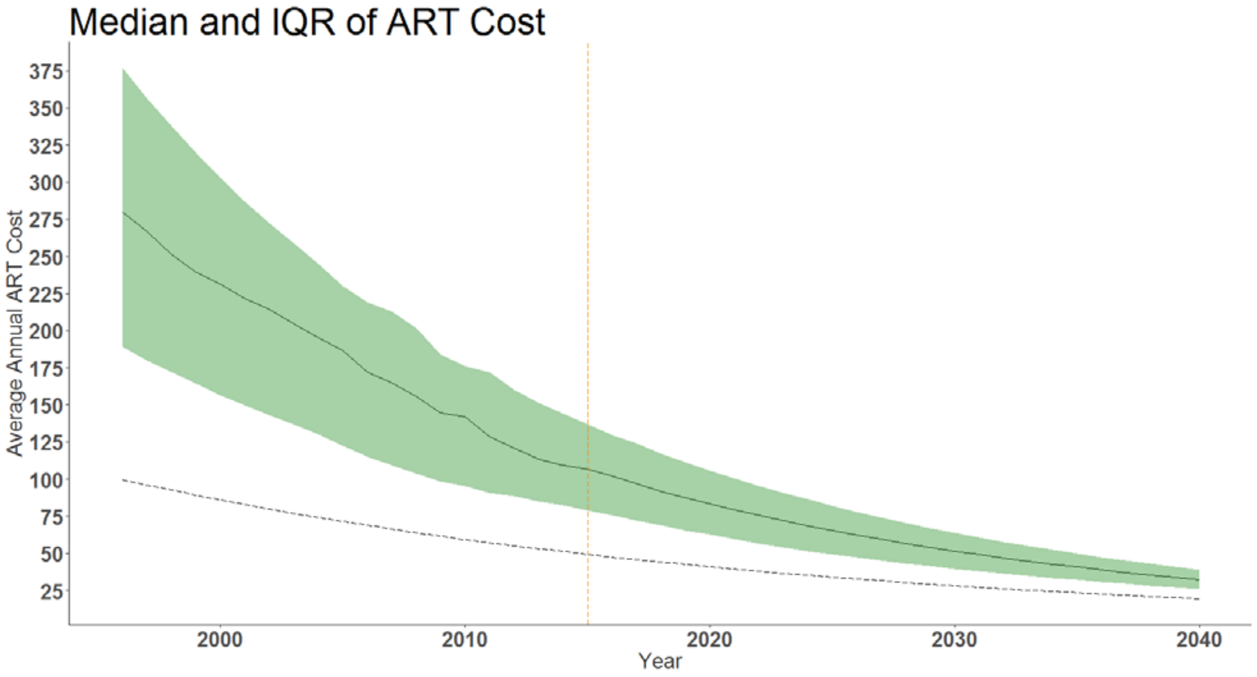

Figure 6. Median and IQR of ART price over time globally, alongside the cost frontier as a dashed line. All series are shown in USD.

Projecting Scenarios of Exogenous Inputs

A number of inputs to the ART projecting, incidence hazard projecting, and Spectrum HIV modeling systems are treated as exogenous inputs. Projection scenarios for these inputs were created using a rate of change approach, consistent with that used across the projecting platform. These inputs include:

- ART Price
- Lag Distributed GDP per capita
- HIV-specific development assistance for health
- Government Health Expenditure per capita
- Child ART coverage
- Cotrimoxazole coverage among children

- Coverage of medication used to prevent mother-to-child transmission of HIV (PMTCT) prenatally and postnatally

For each indicator, the distribution of the rate of change across countries was calculated. The timeseries in each indicator was projected for three scenarios. The 'reference' scenario assumed each country grows in the future at the 50th percentile of the rate of change across countries, the 'worse' scenario assumes growth at the 15th percentile, and the 'better' scenario assumed growth at the 85th percentile. For ART price, the 'better' and 'worse' scenarios are flipped, since decreases in price should be considered 'better' for health outcomes. Therefore, the 'better' scenario uses the 15th percentile of the rate of change and the 'worse' scenario uses the 85th.

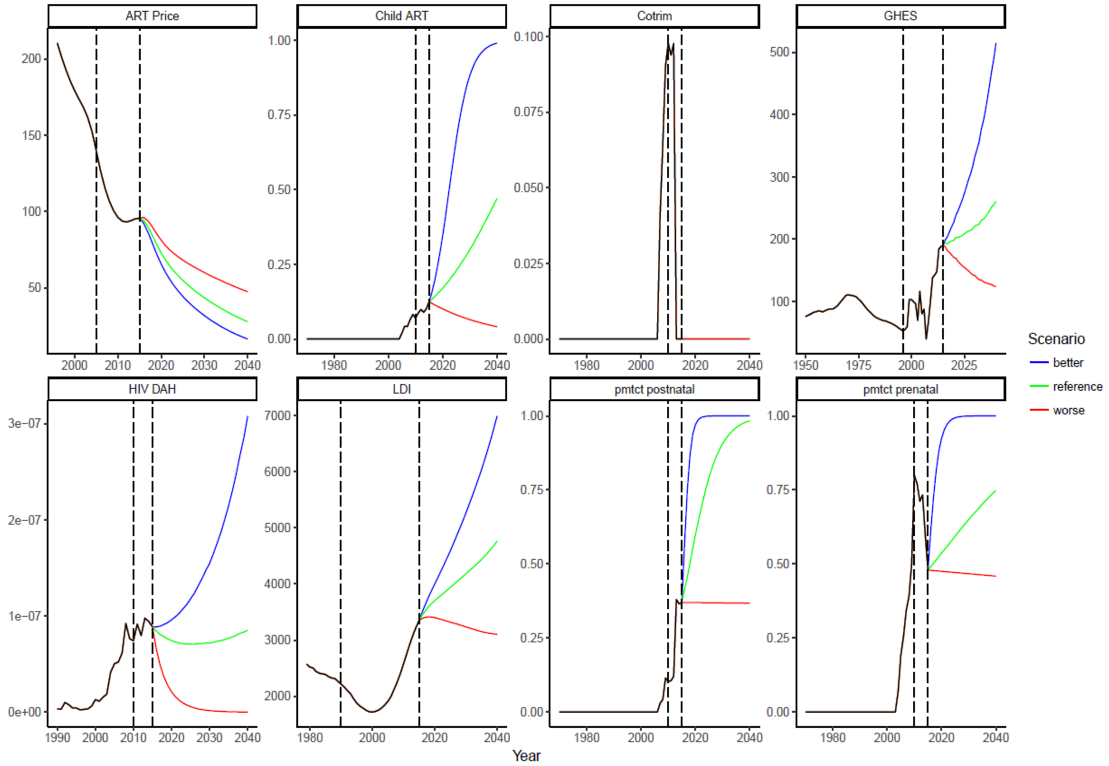

Figure 7. Scenarios of exogenous input projections for Zambia

Some of the series had previously existing 'reference' projections, such as ART price (described above), or LDI (published separately). For these indicators, the projections for each scenario were scaled so that the original 'reference' scenario projections were used, and the 'better' and 'worse' projections fall on either side of the 'reference.' This was accomplished by simply calculating a single country-specific scaling factor for each year from 2016 through 2040 to adjust the rate of change based 'reference' scenario to be identical to the previously existing 'reference' scenario, and using this factor for all 3 scenarios. Inputs that represent a coverage indicator, including PMTCT, Cotrimoxazole, and ART, were projected in logit space, while the remaining indicators were modeled in log space. Figure 7 shows an example of projected exogenous inputs for Zambia.

Projecting ART Coverage

ART coverage is projected using the ART caps described above, as well as the HIV-specific development assistance for health (DAH) and Government Health Expenditure per capita (GHES) exogenous inputs, which are projected as described in the previous section. In order to account for the changing costs of ART over time, the DAH and GHES covariates are rescaled to "dose equivalents," by dividing by ART cost. The relationship between country-year specific ART coverage is then modelled with a slope on dose-equivalents of GHES, a slope on dose-equivalents of DAH (using an indicator variable to remove the countries that are never recipients of DAH), and fixed intercepts for each CD4 group.

$$logit(ART_{c,t}) = \beta_1 GHESdoses + (\beta_2 DAHdoses * I_{DAHrecipient}) + (\beta_3 CD4_{0-49} * I_{0-49}) \ldots (\beta_9 CD4_{500+} * I_{500+})$$

Projected ART values are capped using the frontiers estimated as described above, or the largest value observed in the past for the timeseries in question, whichever is larger. Scenarios of ART coverage are created by using scenario specific ART caps, as well as scenario specific DAH, GHES, and ART price series. ART caps are estimated as a function of income, and we use scenarios of LDI as a proxy for income at the national level. DAH, GHES, LDI, and ART price scenarios are created as detailed in the previous section. We then project ART coverage at the granularity it is used in Spectrum, specific to single-year age and sex groups, as well as draws used in Spectrum to propagate uncertainty:

$$logit(ART_{c,a,s,t,d} = \beta_1 GHESdoses + (\beta_2 DAHdoses * I_{DAHrecipient}) + (\beta_3 CD4_{0-49} * I_{0-49}) \ldots (\beta_9 CD4_{500+} * I_{500+}) + \emptyset_{c,a,s,t,d}$$

where $\emptyset_{c,a,s,t,d}$ is a country-year-age-sex-draw specific intercept shift term, used to ensure no disjunctions in the first year of the projects by removing the difference from year 2015 to year 2016 from all projected estimates for each timeseries. The intercept shift term also preserves draw-level uncertainty from past ART coverage estimates from the GBD.

Project Incidence Hazard

Incidence hazard is a key input to the Spectrum modeling process, which is projected using ART projections, as well as a rate of change approach, similar to those described above, with respect to the trend in the counterfactual incidence hazard. First the hazard counterfactual is calculated as:

$$Hazard\ Counterfactual_{c,a,s,t,i} = \frac{Hazard_{c,a,s,t,i}}{1-(ART_{c,a,s,t,i} * Viral\ Suppression_{c,a,s,t,i})}$$

Where: ART is the proportion of HIV+ individuals receiving ART, hazard is the number of new HIV infections over population at risk, hazard counterfactual is the estimated hazard if ART coverage were zero and viral Suppression is the proportion of individuals taking ART who achieve viral suppression, which is assumed to be uniformly distributed with a mean of 70%:

$$Viral\ Suppression \sim U(.6,.8)$$

Consistent with the approach taken to project the independent drivers, projections scenarios for the secular trend in the counterfactual hazard are created by calculating the rate of change across countries in the past, and applying the 15th,50th,and 85th percentile to each country to create the 'worse,' 'reference,' and 'better' scenarios respectively. The final projected hazard rates therefore decreases in response to improvements in ART coverage, as well as change due to the underlying

secular trend in the counterfactual hazard. Scenarios of ART coverage and the secular trend are both reflected in the final incidence hazard scenarios.

References

1 WHO | Global Price Reporting Mechanism for HIV, tuberculosis and malaria. WHO. http://www.who.int/hiv/amds/gprm/en/ (accessed Nov 5, 2016).

2 Ng M, Fleming T, Robinson M, *et al.* Global, regional, and national prevalence of overweight and obesity in children and adults during 1980–2013: a systematic analysis for the Global Burden of Disease Study 2013. *The Lancet* 2014; **384**: 766–81.

3 Ng M, Freeman MK, Fleming TD, *et al.* Smoking Prevalence and Cigarette Consumption in 187 Countries, 1980-2012. *JAMA* 2014; **311**: 183–92.

4 James SL, Gubbins P, Murray CJ, Gakidou E. Developing a comprehensive time series of GDP per capita for 210 countries from 1950 to 2015. *Popul Health Metr* 2012; **10**: 12.

Part 4. Online tools and abbreviations

Part 1. Online tools

Further results are presented as dynamic visualizations at https://vizhub.healthdata.org/sdg/

GBD data sources are available at http://ghdx.healthdata.org/gbd-data-tool

Part 2. List of abbreviations

Adol: adolescent

Air poll mort: mortality attributable to air pollution

ANC: Antenatal care

ART: antiretroviral therapy

DAH: development assistance for health

DALY: disability-adjusted life-year

DHS: Demographic and Health Survey

DPT: diphtheria-pertussis-tetanus

GATHER: Guidelines for Accurate and Transparent Health Estimates Reporting

GBD: Global Burden of Diseases, Injuries, and Risk Factors Study

GPRM: Global Price Reporting Mechanism

HAQ: Healthcare Access and Quality

HH air poll: household air pollution

Hib: Haemophilus influenzae type B

IAEG-SDGs: Inter-Agency and Expert Group on Sustainable Development Goal Indicators

IHR: International Health Regulations

IOTF: International Obesity Task Force

ISIC: International Standard Industrial Classification

JMP: Joint Monitoring Programme

MDG: Millennium Development Goal

MMR: maternal mortality ratio

Mort: mortality

NCDs: non-communicable diseases

NTDs: neglected tropical diseases

OECD: Organisation for Economic Co-operation and Development

Occ burden: disease burden attributable to occupational risks

ODA: Official development assistance

PCV3: Three-dose pneumococcal conjugate vaccine

PM2.5: particulate matter $<2.5\mu m$ in diameter

SBA: skilled birth attendance

SD: standard deviation

SDG: Sustainable Development Goal

SDI: Socio-demographic Index

SDSN: Sustainable Development Solutions Network

SEV: summary exposure value

TB: tuberculosis

TRIPS: Agreement on Trade-Related Aspects of Intellectual Property Rights

UHC: universal health coverage

UIs: uncertainty intervals

UN: United Nations

UNICEF: United Nations Children’s Fund

VR: vital registration

WaSH: water, sanitation, and hygiene

WHO: World Health Organization

Methods Appendix Figure 1. Comparison of health−related SDG index values using the arithmetic mean of targets versus the geometric mean, by country, 2016. The black line shows the equivalence line, such that values that fall on this line are the same for each construction of the health−related SDG index. Countries are abbreviated according to the ISO3 code. SDG=Sustainable Development Goal.

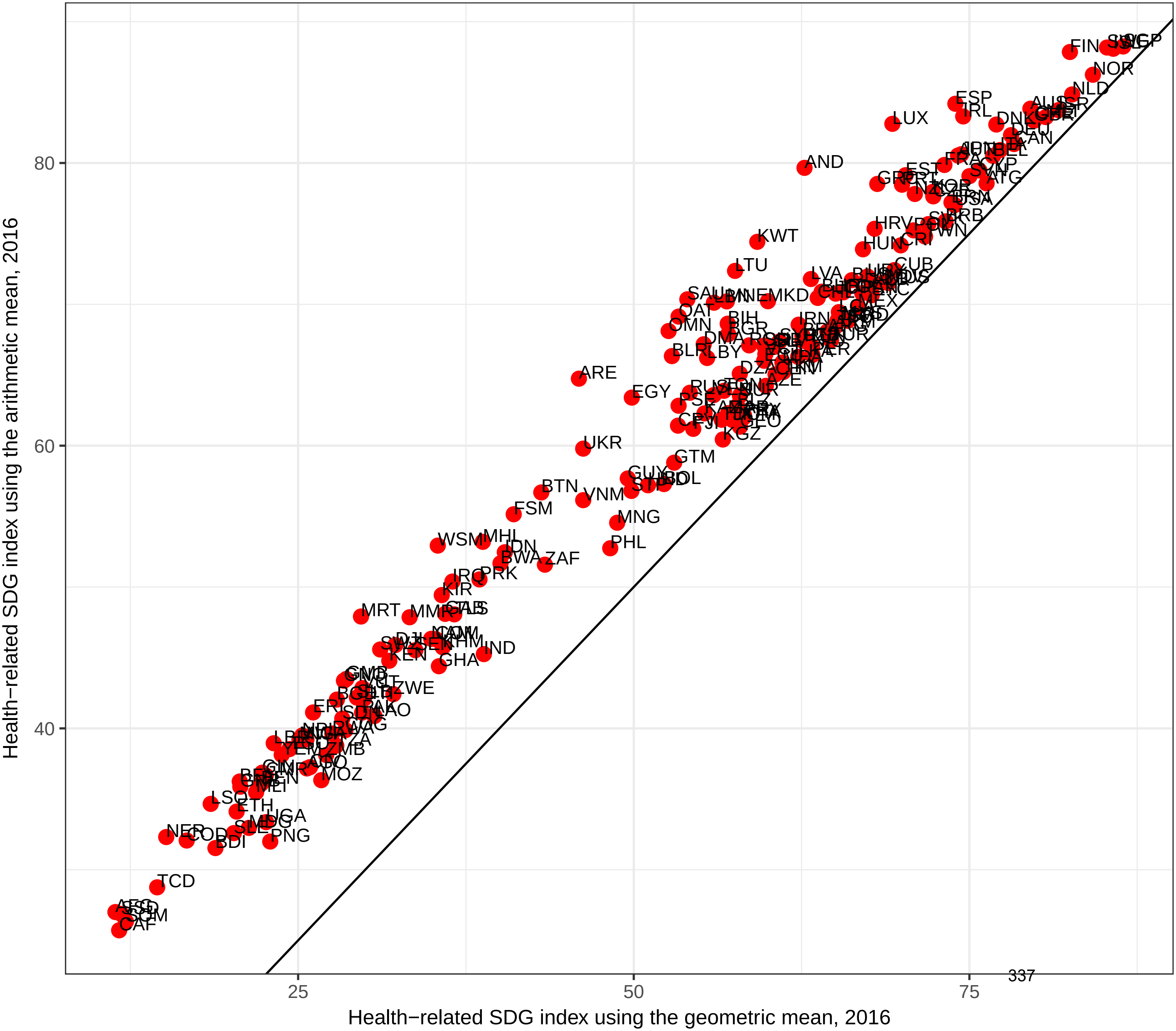

Methods Appendix Figure 2. Comparison of health−related SDG index ranks using the arithmetic mean of targets versus the geometric mean, by country, 2016. The black line shows the equivalence line, such that values that fall on this line are the same for each construction of the health−related SDG index. Countries are abbreviated according to the ISO3 code. SDG=Sustainable Development Goal.

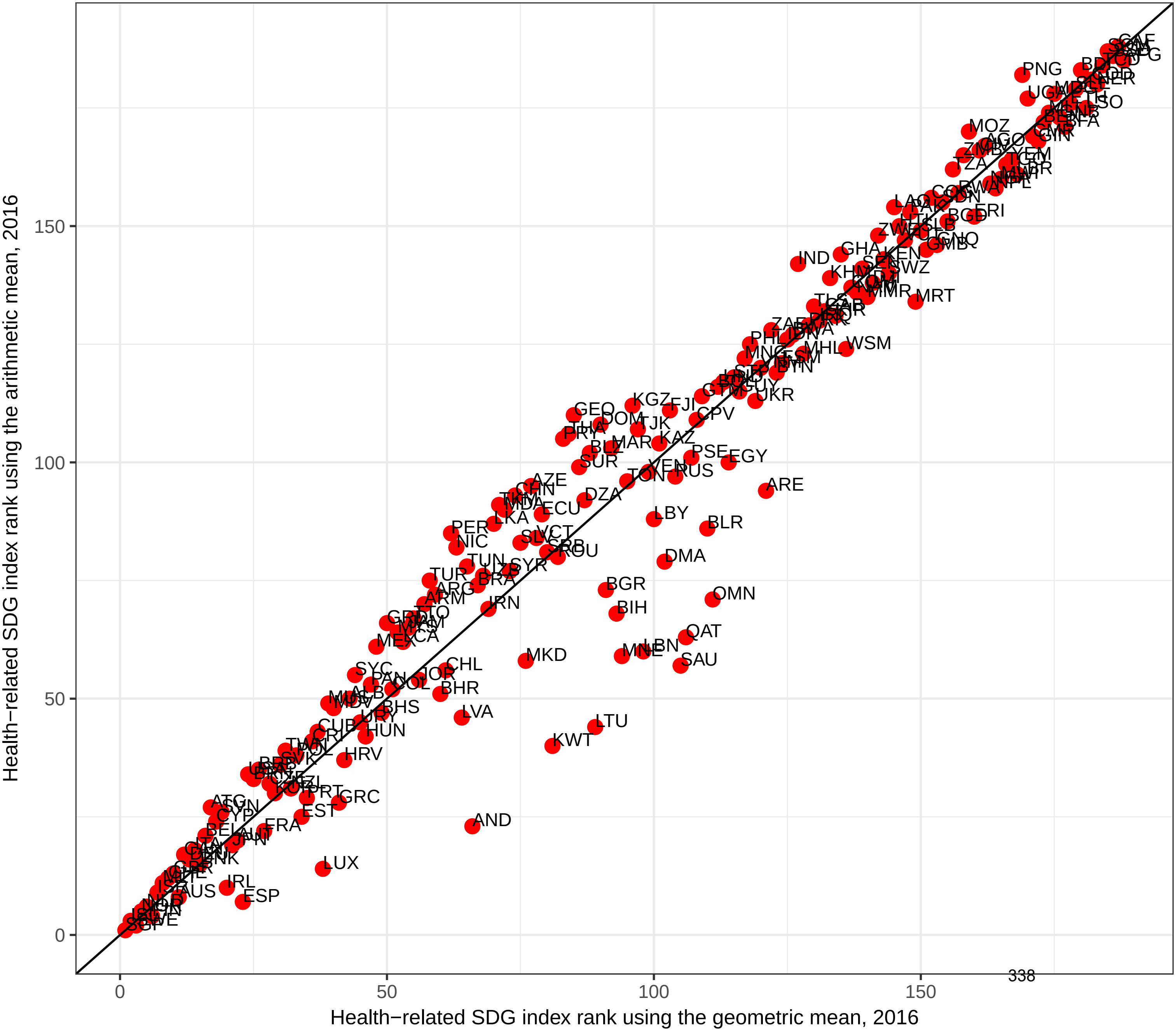

Methods Appendix Figure 3. Comparison of health−related SDG index values using geometric mean of the minimums across each target versus the standard geometric mean, by country, 2016. The black line shows the equivalence line, such that values that fall on this line are the same for each construction of the health−related SDG index. Countries are abbreviated according to ISO3 code. SDG=Sustainable Development Goal.

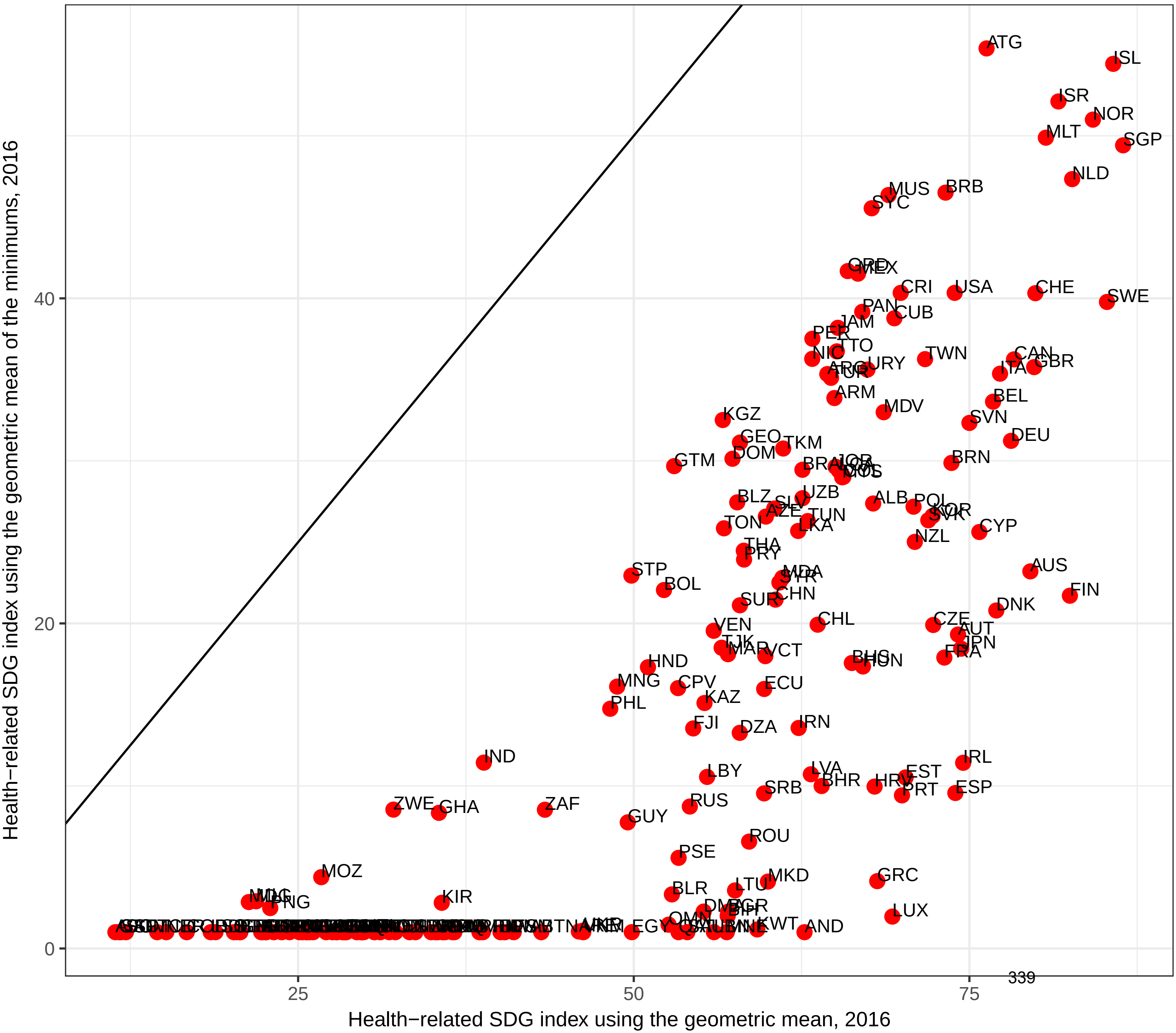

Methods Appendix Figure 4. Comparison of health−related SDG index ranks using geometric mean of the minimums across each target versus the standard geometric mean, by country, 2016. The black line shows the equivalence line, such that values that fall on this line are the same for each construction of the health−related SDG index. Countries are abbreviated according to ISO3 code. SDG=Sustainable Development Goal.

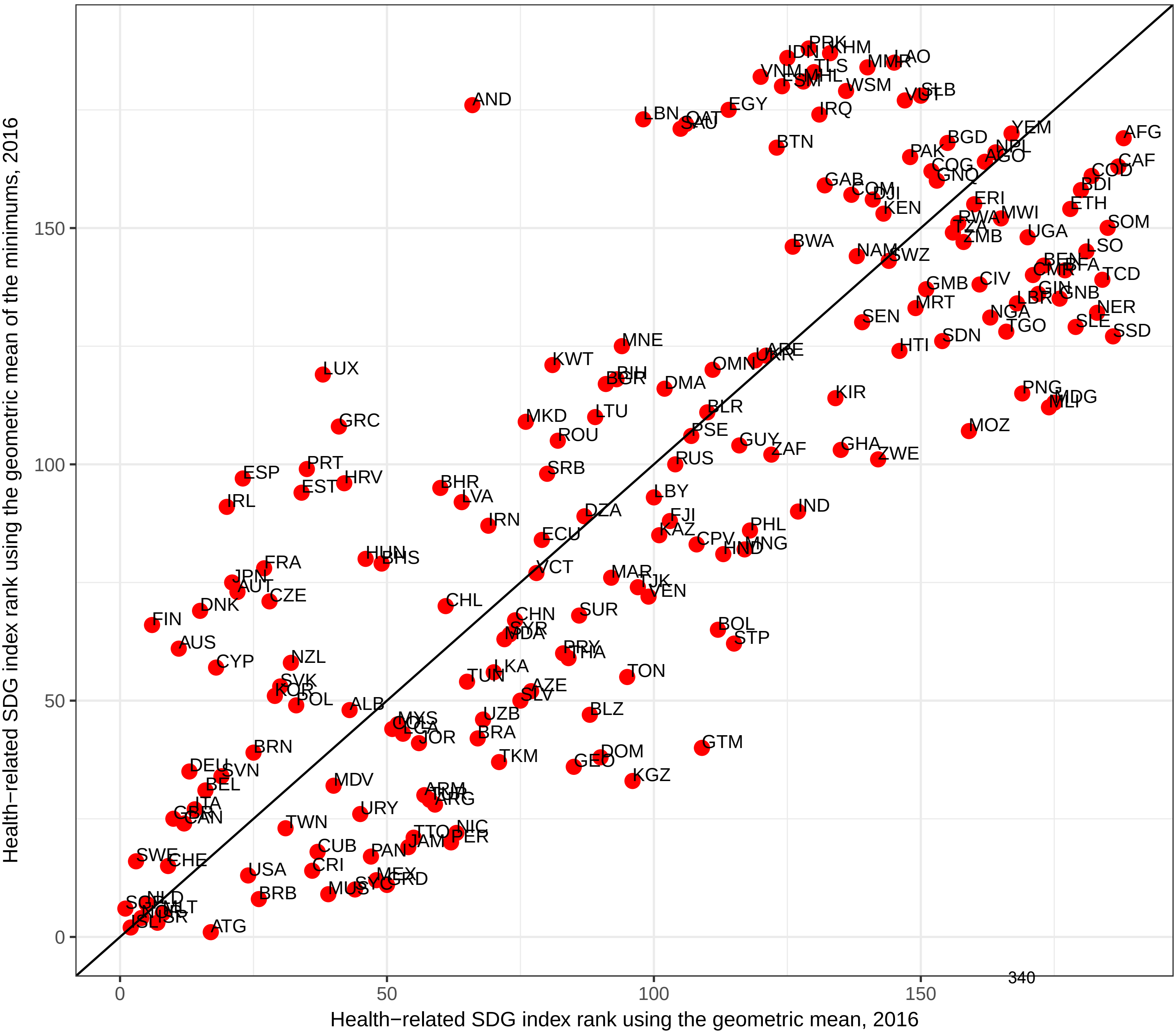

Methods Appendix Figure 5. Comparing the absolute change in the health-related SDG index from 2016 to 2030 using the geometric versus arithmetic mean

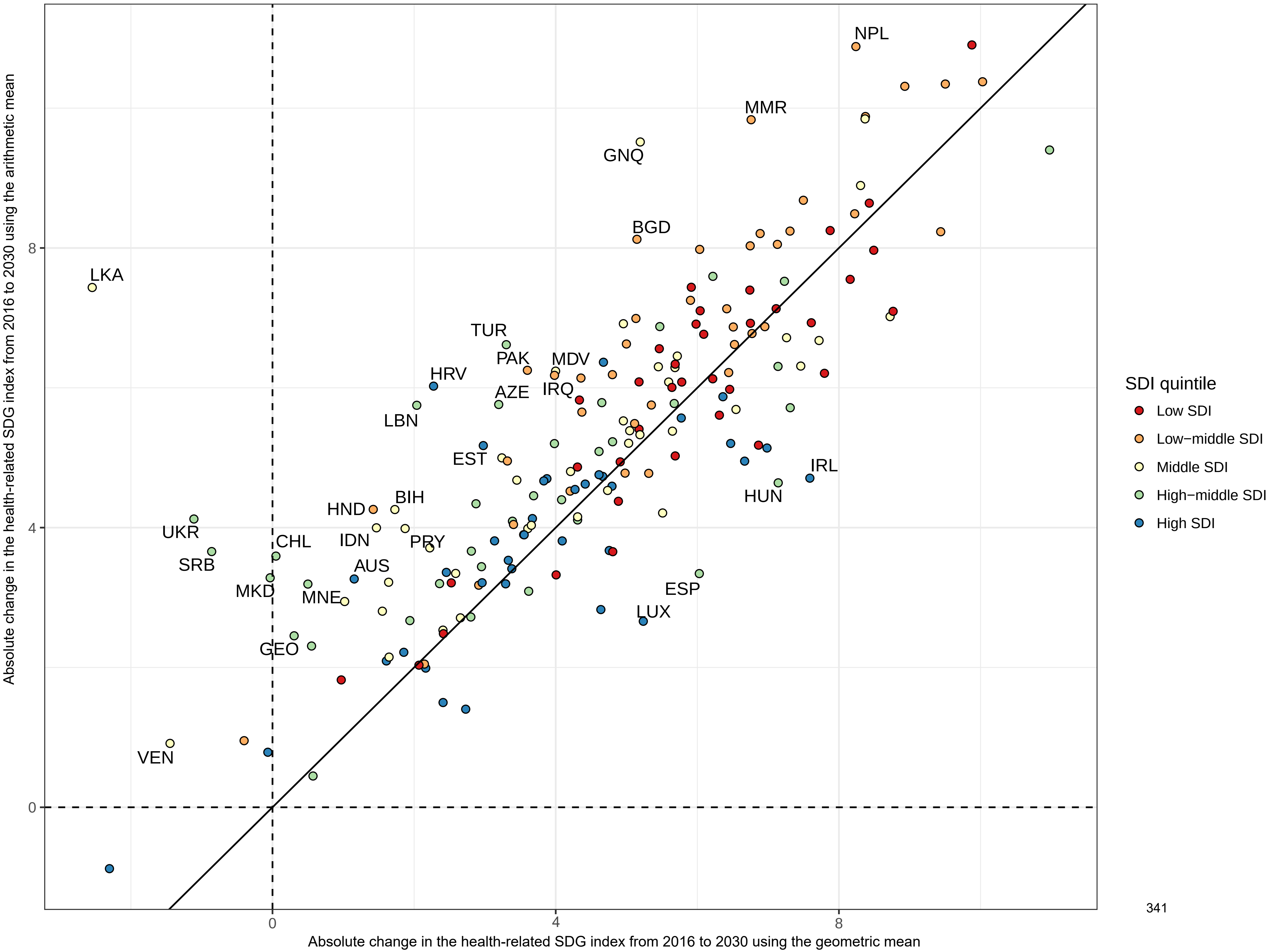

Supplement: Supplementary appendix 1 [file mmc1.pdf]
