# Supplementary material for: Measuring progress and projecting attainment on the basis of past trends of the health-related Sustainable Development Goals in 188 countries: an analysis from the Global Burden of Disease Study 2016
Source: Lancet. 2017 Sep 16;390(10100):1423–59. doi: 10.1016/S0140-6736(17)32336-X (PMC5603800; doi:10.1016/S0140-6736(17)32336-X)
Supplement: Supplementary appendix 2 [file mmc2.pdf]

THE LANCET

Supplementary appendix

This appendix formed part of the original submission and has been peer reviewed. We post it as supplied by the authors.

Supplement to: GBD 2016 SDG Collaborators. Measuring progress and projecting attainment on the basis of past trends of the health-related Sustainable Development Goals in 188 countries: an analysis from the Global Burden of Disease Study 2016. *Lancet* 2017; published online Sept 12. http://dx.doi.org/10.1016/S0140-6736(17)32336-X.

Supplementary results to Measuring progress and projecting attainment based on past trends of the health-related Sustainable Development Goals in 188 countries: an analysis from the Global Burden of Disease Study 2016

The supplementary results appendix offers additional figures and tables.

List of Supplementary Results: Figures and Tables

Supplementary Figure 1. Comparing the UHC index with the geometric mean of nine UHC tracer interventions in 2016, by country.

Supplementary Figure 2. Comparing the UHC index in 2000 and 2016, by country.

Supplementary Figure 3. Projected performance, based on past trends, on the health-related SDG index, MDG index, and non-MDG index, and 37 individual health-related indicators, by country, 2030.

Supplementary Figure 4. Absolute projected change in the health-related SDG index, MDG index, and non-MDG index, and 37 individual health-related indicators, by country from 2016 to 2030.

Supplementary Figure 5. Comparing attainment of defined health-related SDG indicator targets in 2016 and, based on past trends, projected to be attained in 2030, by country.

Supplementary Figure 6. Comparing attainment of defined and conservative health-related SDG indicator targets in 2016 and, based on past trends, projected to be attained in 2030, by country.

Supplementary Table 1. Health-related SDGs excluded in the present analysis, and measurement needs and strategy for future reporting, by SDG target.

Supplementary Table 2. Scaled values for the health-related SDG index, MDG index, and non-MDG index, and 37 individual health-related indicators, by country, in 2000, 2015, 2016, and 2030.

Supplementary Table 3. Unscaled values for the 37 individual health-related indicators, by country, in 2000, 2015, 2016, and 2030.

Supplementary Figure 1. Comparing the UHC index with the geometric mean of nine UHC tracer interventions in 2016, by country. The blue line shows the equivalence line, such that values that fall on this line are equivalent for both the UHC index and the geometric mean of nine UHC tracer interventions (coverage of three doses of the diphtheria-pertussis-tetanus vaccine, measles vaccine, and three doses of the oral polio vaccine or inactivated polio vaccine; met need for modern contraception; antenatal care coverage, 1 and 4 visits; skilled birth attendance; coverage; in-facility delivery rates; and coverage of antiretroviral therapy among people living with HIV). Countries are abbreviated according to the ISO3 code. UHC=universal health coverage. SDI=Socio-demographic Index. SDG=Sustainable Development Goal.

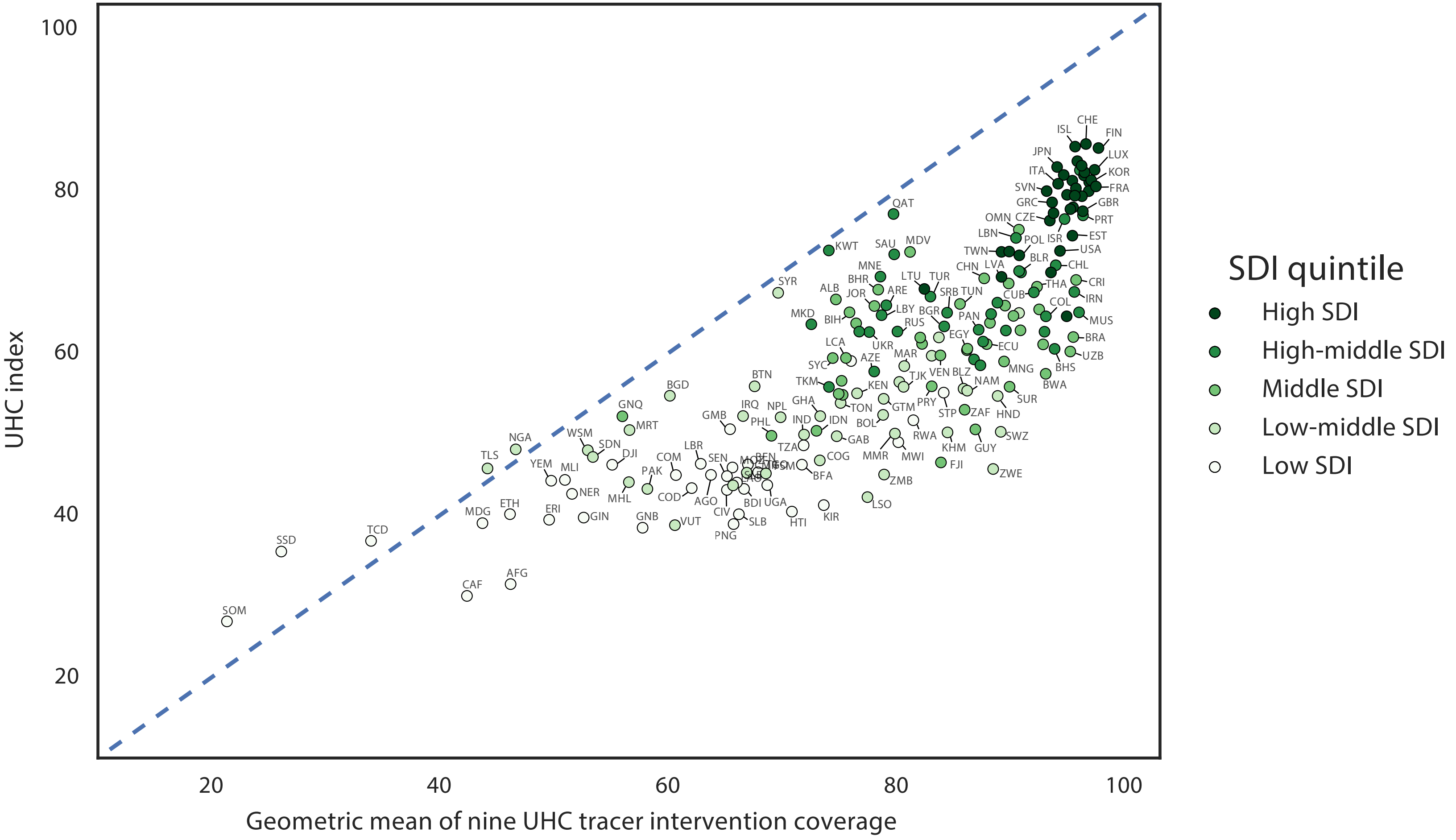

Supplementary Figure 2. Comparing the UHC index in 2000 and 2016, by country.
The blue line shows the equivalence line, such that values that fall on this line are
the same for the UHC index in 2000 and 2016. Countries are abbreviated according to the ISO3 code.
UHC=universal health coverage. SDI=Socio-demographic Index.

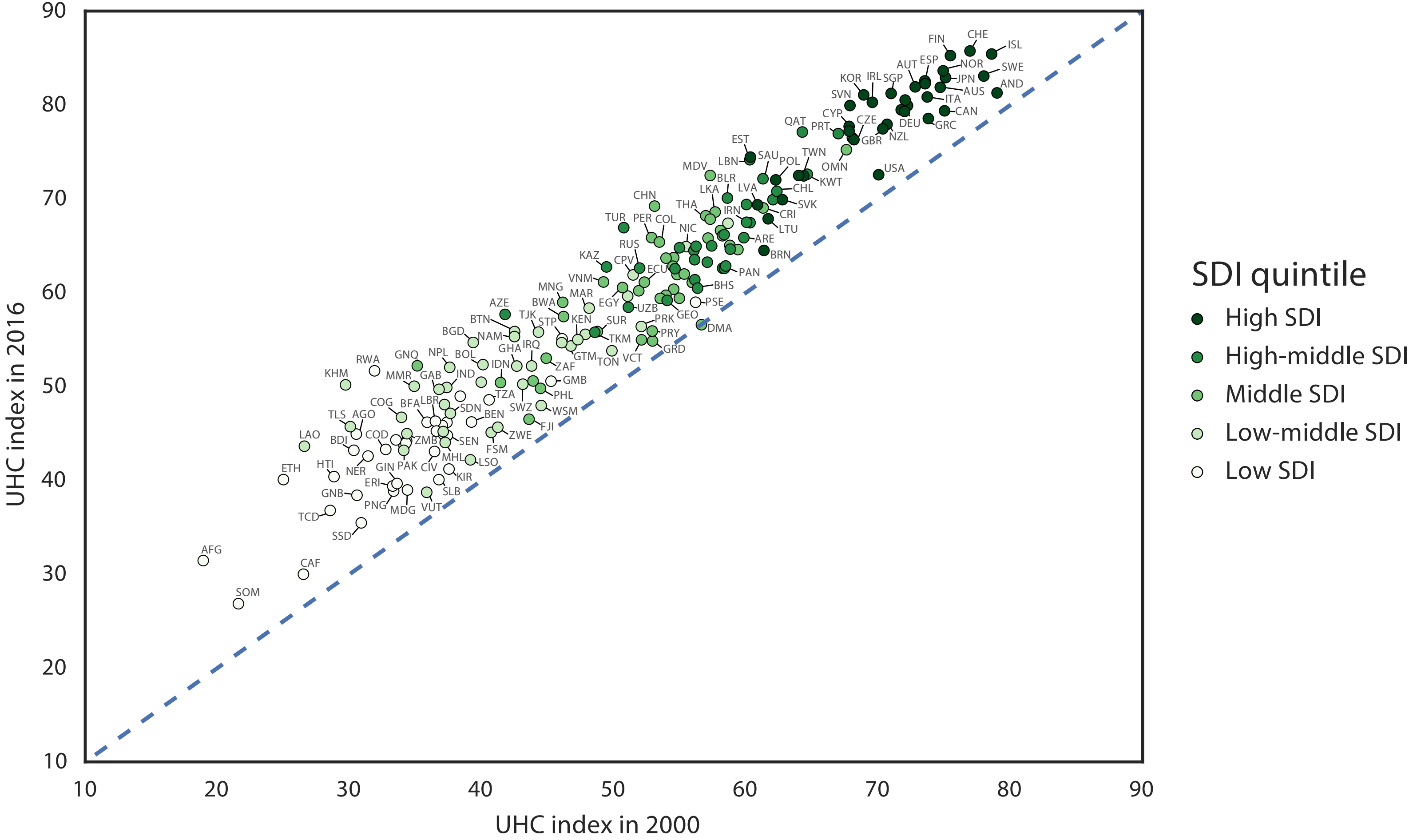

Supplementary Figure 3. Projected performance, based on past trends, on the health-related SDG index, MDG index, and non-MDG index, and 37 individual health-related indicators, by country, 2030. All projections were based on past trends and rates of change observed from 1990 to 2016. Countries are ranked by their health-related SDG index from highest to lowest in 2030, which was projected based on past trends. Indices and individual indicators are reported on a scale of 0 to 100, with 0 representing the worst levels from 1990 to 2030 and 100 reflecting the best during that time. Definitions of health-related SDG indicators are shown in table 1 in the main manuscript. SDG=Sustainable Development Goal. MDG=Millennium Development Goal. Disaster mort=Mortality due to exposure to forces of nature. MMR=Maternal mortality ratio. SBA=Skilled birth attendance. Under-5 mort=Under-5 mortality. Neonatal mort=Neonatal mortality. HIV incid=HIV incidence. Tuberculosis incid=tuberculosis incidence. Malaria incid=malaria incidence. Hep B incid=hepatitis B incidence NTD pev=prevalence of 15 neglected tropical diseases. NCD mort=mortality due to a subset of non-communicable diseases (cardiovascular disease, cancer, diabetes, and chronic respiratory diseases). Suicide mort=mortality due to self-harm. Road injury mort=mortality due to road injuries. FP need met, mod=met need for family planning with modern contraception methods. Adol birth rate=adolescent birth rate. UHC index=universal health coverage index. Air poll mort=mortality attributable to household air pollution and ambient air pollution. WaSH mort=mortality attributable to unsafe water, sanitation, and hygiene. Poisoning mort=Mortality due to unintentional poisonings. Smoking prev=prevalence of daily smoking. Vaccine cov=vaccine coverage of target populations based on national vaccine schedules. Int partner viol=prevalence of intimate partner violence. HH air poll=prevalence of household air pollution. Occ burden=disease burden attributable to occupational risks. Mean PM2.5=mean particulate matter smaller than 2.5 microns in diameter. Homicide mort=mortality due to interpersonal violence. Conflict mort=mortality due to conflict and terrorism. Violence prev=prevalence of physical or sexual violence. Child sex abuse=prevalence of childhood sexual abuse. Cert death reg=well-certified death registration.

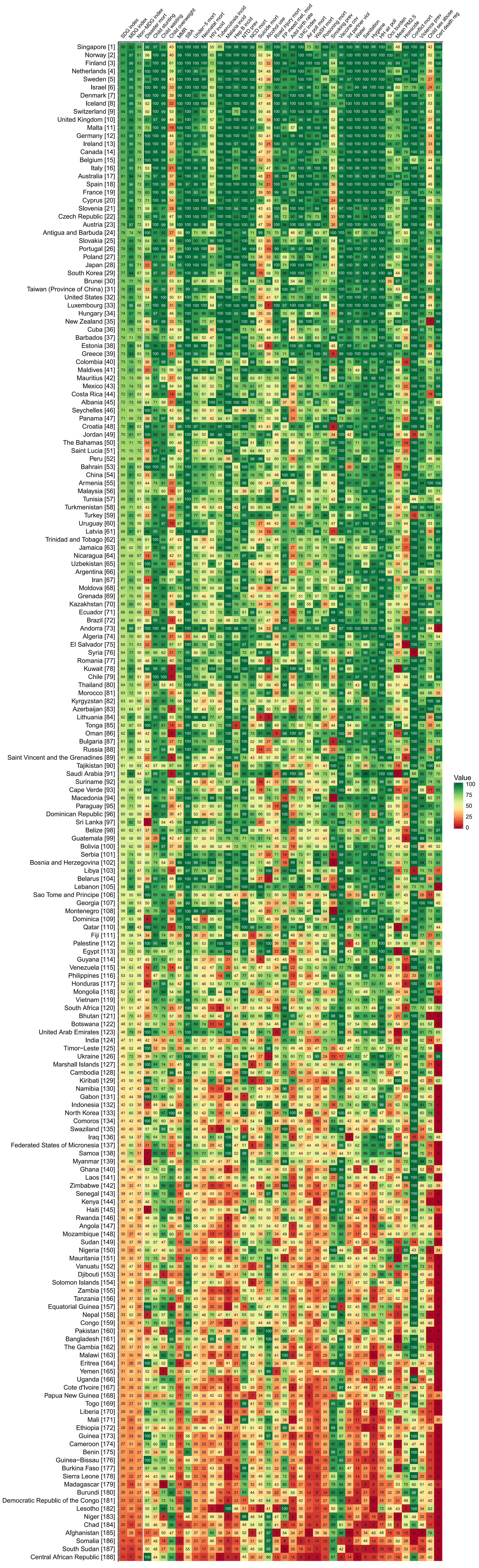

Supplementary Figure 4. Absolute projected change in the health-related SDG index, MDG index, and non-MDG index, and 37 individual health-related indicators, by country from 2016 to 2030. All projections were based on past trends and rates of change observed from 1990 to 2016. Countries are ranked by the difference in their projected health-related SDG index in 2030 from the health-related SDG index in 2016. Positive values represent projected improvements based on past trends for overall indices and scaled indicators from 2016 to 2030, while negative values represented projected worsening based on past trends for overall indices and scaled indicators. Definitions of health-related SDG indicators are shown in table 1 in the main manuscript. SDG=Sustainable Development Goal. MDG=Millennium Development Goal. Disaster mort=Mortality due to exposure to forces of nature. MMR=Maternal mortality ratio. SBA=Skilled birth attendance. Under-5 mort=Under-5 mortality. Neonatal mort=Neonatal mortality. HIV incid=HIV incidence. Tuberculosis incid=tuberculosis incidence. Malaria incid=malaria incidence. Hep B incid=hepatitis B incidence NTD pev=prevalence of 15 neglected tropical diseases. NCD mort=mortality due to a subset of non-communicable diseases (cardiovascular disease, cancer, diabetes, and chronic respiratory diseases). Suicide mort=mortality due to self-harm. Road injury mort=mortality due to road injuries. FP need met, mod=met need for family planning with modern contraception methods. Adol birth rate=adolescent birth rate. UHC index=universal health coverage index. Air poll mort=mortality attributable to household air pollution and ambient air pollution. WaSH mort=mortality attributable to unsafe water, sanitation, and hygiene. Poisoning mort=mortality due to unintentional poisonings. Smoking prev=prevalence of daily smoking. Vaccine cov=vaccine coverage of target populations based on national vaccine schedules. Int partner viol=prevalence of intimate partner violence. HH air poll=prevalence of household air pollution. Occ burden=disease burden attributable to occupational risks. Mean PM2.5=mean particulate matter smaller than 2.5 microns in diameter. Homicide=mortality due to interpersonal violence. Conflict mort=mortality due to conflict and terrorism. Violence prev=prevalence of physical or sexual violence. Child sex abuse=prevalence of childhood sexual abuse. Cert death reg=well-certified death registration.

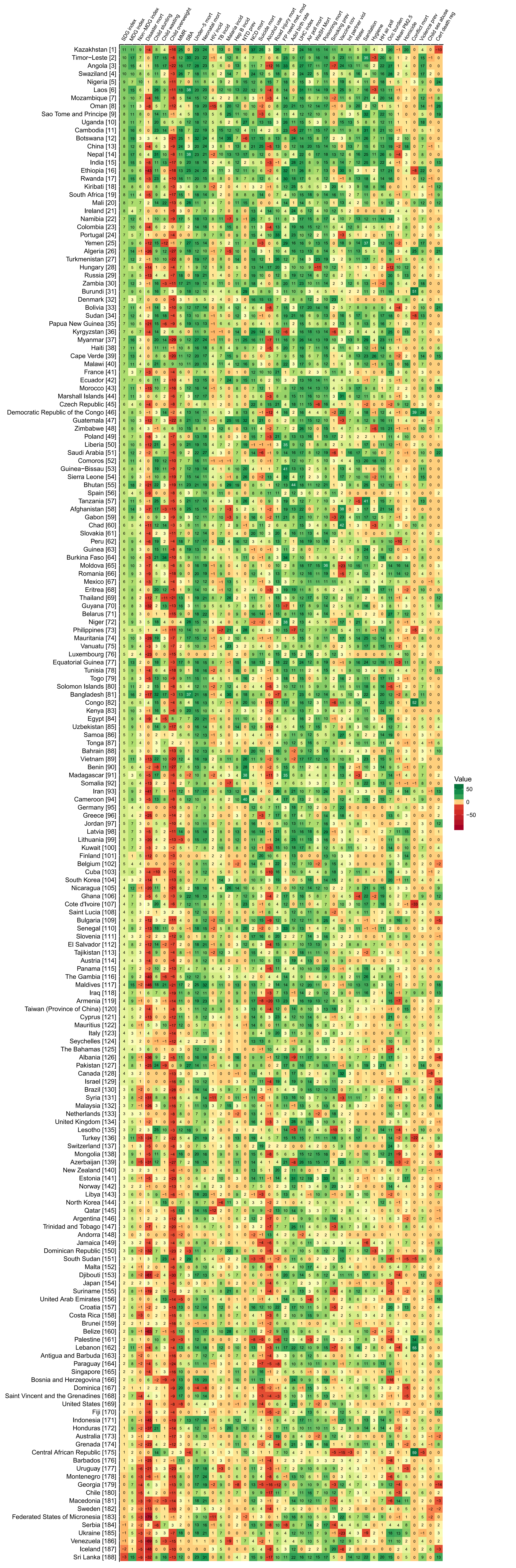

Supplementary Figure 5. Comparing attainment of defined health-related SDG indicator targets in 2016 and, based on past trends, projected to be attained in 2030, by country. Countries are ranked by the number of SDG indicator targets they were projected to attain in 2030. All projections were based on past trends and rates of change observed from 1990 to 2016. Of the 37 health-related indicators measured in this study, 24 had defined targets linked to each indicator. SDG target 3.6 aims to reduce road injury mortality by 50% between 2015 and 2020, and thus projected attainment for this indicator is based on estimates from 2015 to 2020 rather than 2015 to 2030. Definitions of health-related SDG indicators and targets associated with them, as well as the specific target thresholds applied, are shown in table 1 in the main manuscript. SDG=Sustainable Development Goal. MMR=Maternal mortality ratio. SBA=Skilled birth attendance. Under-5 mort=Under-5 mortality. Neonatal mort=Neonatal mortality. HIV incid=HIV incidence. Tuberculosis incid=Tuberculosis incidence. Malaria incid=Malaria incidence. NTD prev=prevalence of 15 neglected tropical diseases. NCD mort=death rate due to a subset of non-communicable diseases (cardiovascular disease, cancer, diabetes, and chronic respiratory diseases). Suicide mort=mortality due to self-harm. Road injury mort=Mortality rate due to road injuries. FP need met, mod=met need for family planning with modern contraception methods. UHC index=Universal health coverage index. Vaccine cov=vaccine coverage of target populations based on national vaccine schedules. Int partner viol=Intimate partner violence. HH air poll=Household air pollution. Child sex abuse=Childhood sexual abuse. Cert death reg=Well-certified death registration.

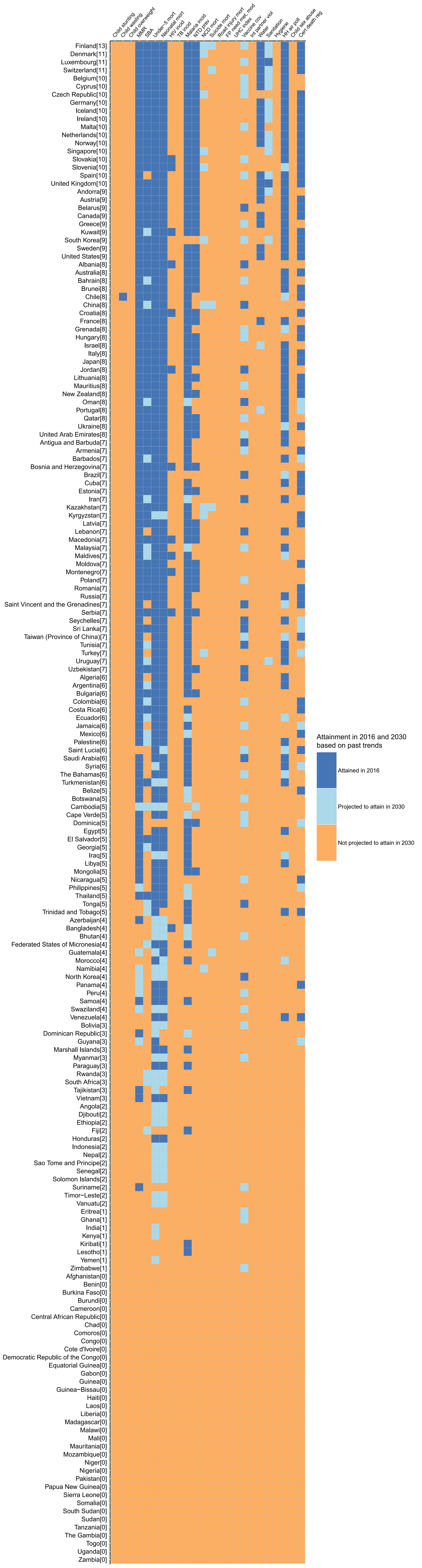

Supplementary Figure 6. Comparing attainment of defined and conservative health-related SDG indicator targets in 2016 and, based on past trends, projected to be attained in 2030, by country. Conservative targets were defined as an 80% reduction for elimination targets from 2015 to 2030, and ≥90% by 2030 for universal access or coverage. Targets with specific values to meet by 2030 or with specified relative reductions remained as originally defined, as found in table 1 in the main manuscript. Countries are ranked by the number of SDG indicator targets they were projected to attain in 2030. All projections were based on past trends and rates of change observed from 1990 to 2016. Of the 37 health-related indicators measured in this study, 24 had defined targets linked to each indicator. SDG target 3.6 aims to reduce road injury mortality by 50% between 2015 and 2020, and thus projected attainment for this indicator is based on estimates from 2015 to 2020 rather than 2015 to 2030. Definitions of health-related SDG indicators and targets associated with them are shown in table 1 in the main manuscript. SDG=Sustainable Development Goal. MMR=Maternal mortality ratio. SBA=Skilled birth attendance. Under-5 mort=Under-5 mortality. Neonatal mort=Neonatal mortality. HIV incid=HIV incidence. Tuberculosis incid=Tuberculosis incidence. Malaria incid=Malaria incidence. NTD prev=prevalence of 15 neglected tropical diseases. NCD mort=death rate due to a subset of non-communicable diseases (cardiovascular disease, cancer, diabetes, and chronic respiratory diseases). Suicide mort=mortality due to self-harm. Road injury mort=Mortality rate due to road injuries. FP need met, mod=met need for family planning with modern contraception methods. UHC index=Universal health coverage index. Vaccine cov=vaccine coverage of target populations based on national vaccine schedules. Int partner viol=Intimate partner violence. HH air poll=Household air pollution. Child sex abuse=Childhood sexual abuse. Cert death reg=Well-certified death registration.

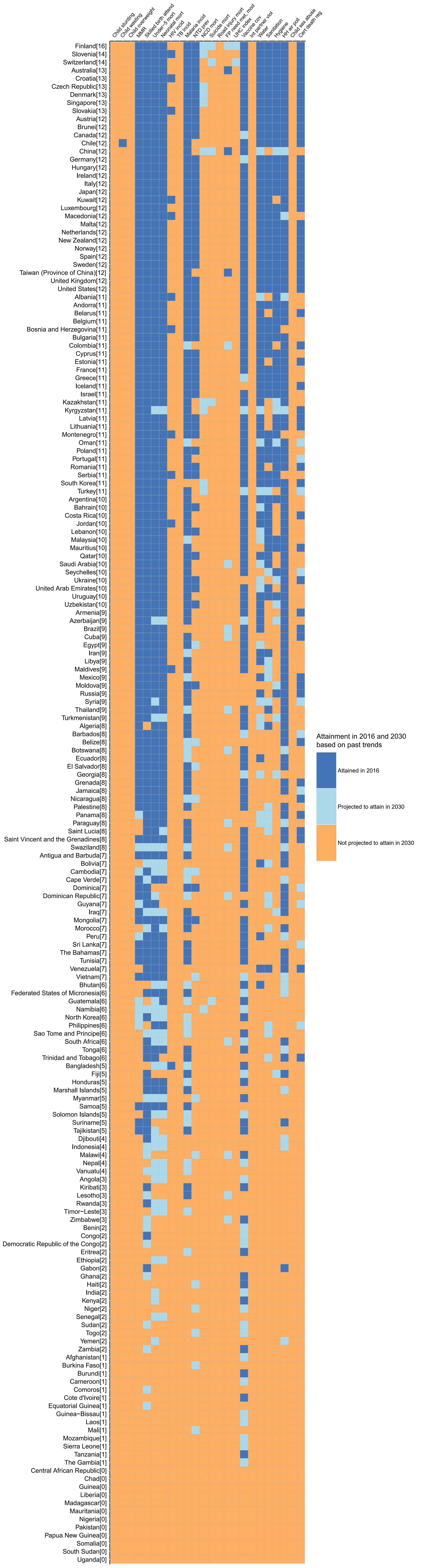

***Supplementary Table 1.* Health-related SDGs excluded in the present analysis, and measurement needs and strategy for future reporting, by SDG target.** Definitions and descriptions of health-related SDG indicators beyond the specific indicators originate from the IAEG-SDGs compilation of metadata for each SDG (as provided by each indicator's custodial agency). DAH=development assistance for health. DHS=Demographic and Health Survey. GBD=Global Burden of Disease. IAEG-SDGs=Inter-agency and Expert Group on SDG Indicators. IHR=International Health Regulations. ISIC=International Standard Industrial Classification. JEE=Joint External Evaluation. NCD=non-communicable diseases. SDG=Sustainable Development Goal. TRIPS=Agreement on Trade-Related Aspects of Intellectual Property Rights. UHC=universal health coverage. UN=United Nations. WHO=World Health Organization.

SDG target	Health-related SDG indicator	Measurement needs and strategy
**Goal 3: Ensure healthy lives and promote well-being for all at all ages.**		
Target 3.5: Strengthen the prevention and treatment of substance abuse, including narcotic drug abuse and harmful use of alcohol.	Indicator 3.5.1: Coverage of treatment interventions (pharmacological, psychosocial, and rehabilitation and aftercare services) for substance use disorders.	Prevalence of specific substance use disorders (opioid use disorders, cocaine use disorders, amphetamine use disorders, and cannabis use disorders), as well as alcohol use disorders, are presently estimated as part of the Global Burden of Disease (GBD) study. Systematic reviews on coverage of specific interventions (eg, opioid substitution therapy) are currently in progress as part of the GBD.
Target 3.8: Achieve universal health coverage, including financial risk protection, access to quality essential health-care services and access to safe, effective, quality, and affordable essential medicines and vaccines for all	Indicator 3.8.2: Proportion of population with large household expenditures on health as a share of total household expenditure or income.	Comprehensive and comparable datasets on household expenditures on health as a fraction of total household expenditure or income are not currently available across all locations and over time. Appropriately measuring what constitutes total household expenditure or income while accounting for non-monetary household types of wealth which contribute to overall household income will be necessary for populations on the lower end of the sociodemographic spectrum.
Target 3.b: Support the research and development of vaccines and medicines for the communicable and non-communicable diseases that primarily affect developing countries, provide access to affordable essential medicines and vaccines, in accordance with the Doha Declaration on the TRIPS Agreement and Public Health, which affirms the right of developing countries to use to the full the provisions in the Agreement on Trade-Related Aspects of Intellectual Property Rights regarding flexibilities to protect public health, and, in particular, provide access to medicines for all.	Indicator 3.b.2: Total net official development assistance to the medical research and basic health sectors.	Development assistance for health (DAH) is currently assessed within a comprehensive, comparable analytic framework by source, channel, recipient country, and health focus area from 1990 to 2016; however, funding specifically for medical research (eg, research and development of vaccines and medicines, as described in Target 3.b), is not systematically available across source and recipient countries. In addition, the appropriate assessment of country-level performance remains unclear (eg, whether countries that receive high levels of DAH for medical research are equivalent, in terms of indicator performance, to countries that disburse high levels of DAH for medical research).
Target 3.b (same as above)	Indicator 3.b.3: Proportion of health facilities that have a core set of relevant essential medicines available and affordable on a sustainable basis.	Across all locations and over time, comparable data on the stocking and stock-out rates of essential medicines for all facility types (hospitals, primary care facilities, pharmacies, and other health care outlets) and facility ownership (public, private, informal) are not currently available. In the absence of robust measures of stock-outs in both the public and private sectors across countries and over time, the measurement strategy for producing comparable results for this indicator is unclear. Further, what should constitute "a core set of relevant essential medicines" is likely to vary by location based on its epidemiological profile, and thus work is needed to more precisely define what these core sets of relevant essential medicines should be given known disease burden, risk factor profiles, and health risks across countries. Last, the proposed indicator stipulates measurement of not simply access to a core set of essential medicines but also access to affordable medicines. No comprehensive and comparable datasets on the status of essential medicine affordability, in addition to their stocks, presently exist.
Target 3.c: Substantially increase health financing and the recruitment, development, training and retention of the health workforce in developing countries, especially in least developed countries and small island developing States.	Indicator 3.c.1: Health worker density and distribution, as measured by number of health workers per 1,000 population by cadre. Cadres include generalist medical practitioners, specialist medical practitioners (surgeons, anaesthetists, obstetricians, emergency medicine specialists, cardiologists, paediatricians, psychiatrists, ophthalmologists, gynaecologists, etc.), nursing and midwifery professionals, traditional and complementary medicine professionals, among others.	A systematic analysis of population census data and Labour Force Surveys and censuses is presently under way as part of the GBD study.

SDG target	Health-related SDG indicator	Measurement needs and strategy
**Goal 3: Ensure healthy lives and promote well-being for all at all ages.**		
Target 3.d: Strengthen the capacity of all countries, in particular developing countries, for early warning, risk reduction and management of national and global health risks.	Indicator 3.d.1: International Health Regulations (IHR) capacity and health emergency preparedness. The WHO-recommended measure is the percentage of 13 core capacities that have been attained at a specific time (IHR core capacity index). The 13 core capacities are (1) national legislation, policy, and financing; (2) coordination and national focal point communications; (3) surveillance; (4) response; (5) preparedness; (6) risk communication; (7) human resources; (8) laboratory; (9) points of entry; (10) zoonotic events; (11) food safety; (12) chemical events; and (13) radionuclear emergencies.	Comprehensive and comparable data for all components of the IHR core capacity index, for all geographies and over time, are not currently openly available. Self-evaluations have been undertaken by some member states, with a subset followed up with independent assessments via the Joint External Evaluation process (JEEs). To date 23 countries have completed this process and made reports fully available out of a total of 43 completed JEEs. An additional 30 countries are scheduled for assessment by end of 2018. As these data become more openly available it may be possible to model regional and temporal trends to estimate outstanding countries, but will likely necessitate creating bespoke covariates relating to policy status and types of surveillance system that are not currently reported in the GBD study.
**Goal 5. Achieve gender equality and empower all women and girls**		
Target 5.2: Eliminate all forms of violence against all women and girls in the public and private spheres, including trafficking and sexual and other types of exploitation.	Indicator 5.2.2: Proportion of women and girls aged 15 years and older subjected to sexual violence by persons other than an intimate partner in the previous 12 months, by age and place of occurrence. UN Women defines sexual violence to include acts of abusive sexual contact, forced engagement in sexual acts, attempted or completed sexual acts without consent, incest, sexual harassment, etc. It is noted that survey data on sexual violence against women by non-partners is often limited to information on whether a woman was forced into sexual intercourse when she did not want to and whether a woman experienced an attempt at being forced to perform a sexual act or sexual intercourse against her will.	Prevalence of intimate partner violence among women and girls aged 15 and older is currently estimated as part of the GBD study. An updated systematic review of the literature, data re-extraction, and analysis to specifically quantify prevalence of sexual violence (separately and/or in addition to physical violence) and by persons other than an intimate partner is currently ongoing as part of the GBD. Data availability on the latter, sexual violence by persons other than intimate partners, is very minimal in many parts of the world (eg, sub-Saharan Africa). Currently identified data sources are limited to higher-income countries (ie, Western Europe and the USA).
Target 5.6: Ensure universal access to sexual and reproductive health and reproductive rights as agreed in accordance with the Programme of Action of the International Conference on Population and Development and the Beijing Platform for Action and the outcome documents of their review conferences.	Indicator 5.6.1: Proportion of women aged 15-49 years who make their own informed decisions regarding sexual relations, contraceptive use, and reproductive health care.	The proportion of women who make their own informed decisions regarding all three dimensions of this indicator – sexual relations, contraceptive use, and reproductive health care – are included in the Demographic and Health Survey (DHS) series. Data availablility for non-DHS countries is unclear. The feasibility of measuring this indicator as part of future iterations of the GBD study is presently under review.
	Indicator 5.6.2: Number of countries with laws and regulations that guarantee full and equal access to women and men aged 15 years and older to sexual and reproductive health care, information and education.	Across all locations and over time, comprehensive and comparable data documenting the status of laws and regulations regarding access to sexual and reproductive healthcare, information, and education currently do not exist. Compiling the past and current status of such laws and regulations might be be possible; however, systematically assessing their depth or intensity, enforcement, and effectiveness in guaranteeing access to reproductive health care, information, and education may be challenging across locations and over time.
**Goal 6: Ensure availability and sustainable management of water and sanitation for all.**		
Target 6.3: By 2030, improve water quality by reducing pollution, eliminating dumping and minimizing release of hazardous chemicals and materials, halving the proportion of untreated wastewater and substantially increasing recycling and safe reuse globally.	Indicator 6.3.1: Proportion of wastewater safely treated. UN Water defines this indicator as the proportion of wastewater generated by both households (sewage and faecal sludge), as well as economic activities (based on International Standard Industrial Classification [ISIC] categories) safely treated compared to total wastewater generated both through households and economic activities. While the definition conceptually includes wastewater generated from all economic activities, monitoring will focus on wastewater generated from hazardous industries (as defined by relevant ISIC categories).	Across all locations and over time, comprehensive and comparable data containing information on total wastewater, as generated by both households and non-household entities (however they are to be defined), and wasterwater treatment status do not currently exist. UN Water suggests there will be sufficient data to generate estimates of global and regional levels of safely treated wastewater by 2018; however, in the absence of more country-level data, it is difficult to determine the representativeness of such global and regional estimates.
**Goal 16: Promote peaceful and inclusive societies for sustainable development, provide access to justice for all and build effective, accountable and inclusive institutions at all levels**		
Target 16.1: Significantly reduce all forms of violence and related death rates everywhere.	Indicator 16.1.4: Proportion of people that feel safe walking alone around the area they live.	The Gallup World Poll, which is currently active in more than 140 countries, includes questions about reported safety while walking alone near one's residence. A systematic analysis of these data are planned as part of GBD 2017.

SDG target	Health-related SDG indicator	Measurement needs and strategy
**Goal 16: Promote peaceful and inclusive societies for sustainable development, provide access to justice for all and build effective, accountable and inclusive institutions at all levels**		
Target 16.9 By 2030, provide legal identity for all, including birth registration.	Indicator 16.9.1: Proportion of children under 5 years of age whose births have been registered with a civil authority, by age. *(Considered a duplicate of SDG indicator 17.19.2b)*	Currently, birth registration data reported to WHO do not fully cover all locations or years under analysis, and supplementary data sources, such as household survey data, are often required to estimate births and birth rates outside of high-income regions. Substative data collation efforts would be required for birth registration by location and over time.
**Goal 17: Strengthen the means of implementation and revitalize the global partnership for sustainable development.**		
Target 17.19: By 2030, build on existing initiatives to develop measurements of progress on sustainable development that complement gross domestic product, and support statistical capacity-building in developing countries.	Indicator 17.19.2a: Proportion of countries that have conducted at least one population and housing census in the last 10 years.	A comprehensive assesment of the availability and timing of population and housing censuses across all locations is planned as part of future iterations of the GBD study.
Target 17.19 (as above)	Indicator 17.19.2b: Proportion of countries that have achieved 100 percent birth registration. *(Considered a duplicate of SDG indicator 16.9.1)*	Currently, birth registration data reported to WHO do not fully cover all locations or years under analysis, and supplementary data sources, such as household survey data, are often required to estimate births and birth rates outside of high-income regions. Substative data collation efforts would be required for birth registration by location and over time.

Supplementary Table 2. Scaled values for the health-related SDG index, MDG index, and non-MDG index, and 37 individual health-related indicators, by country, in 2000, 2015, 2016, and 2030. Projected estimates for 2030 were produced based on past trends and rates of change observed from 1990 to 2016. Indices and individual indicators are reported on a scale of 0 to 100, with 0 representing the worst levels from 1990 to 2030 and 100 reflecting the best during that time. SDG=Sustainable Development Goal. MDG=Millennium Development Goal.

Location	SDG Index: Geometric mean of all health-related SDG indicators (scale of 0 to 100)				MDG Index: Geometric mean of all health-related SDG indicators with corresponding MDG indicators (scale of 0 to 100)				Non-MDG Index: Geometric mean of all health-related indicators without corresponding MDG indicators (scale of 0 to 100)				Indicator 1.5.1: Death rate due to exposure to forces of nature (per 100,000 population)			
	2000	2015	2016	2030	2000	2015	2016	2030	2000	2015	2016	2030	2000	2015	2016	2030
**High-income North America**																
Canada	72·9 (71·4-74·2)	78·0 (75·4-80·1)	78·6 (76·0-80·6)	82·2 (78·0-84·8)	86·5 (85·4-87·5)	89·0 (87·9-90·2)	89·2 (88·2-90·4)	92·0 (90·6-93·7)	68·5 (66·5-70·3)	73·4 (70·0-76·4)	74·0 (70·6-76·8)	75·7 (62·2-80·9)	100·0 (100·0-100·0)	100·0 (100·0-100·0)	100·0 (100·0-100·0)	100·0 (100·0-100·0)
United States	69·3 (68·2-70·3)	73·7 (71·9-75·2)	74·1 (72·1-75·6)	75·7 (71·2-78·2)	78·9 (78·0-79·7)	82·2 (81·2-83·4)	82·7 (81·7-83·8)	84·7 (83·0-86·3)	67·0 (65·6-68·3)	71·6 (69·1-73·7)	71·8 (69·0-73·9)	72·5 (66·1-76·3)	54·7 (48·1-61·3)	55·6 (49·0-62·4)	58·1 (51·5-64·9)	53·9 (47·2-61·5)
**Australasia**																
Australia	76·2 (74·1-77·8)	79·7 (72·5-82·9)	79·9 (70·1-83·2)	81·0 (71·2-87·4)	86·6 (85·6-87·5)	91·1 (90·2-91·9)	91·4 (90·6-92·2)	94·1 (93·0-95·3)	72·9 (70·2-75·0)	75·0 (65·9-79·2)	75·0 (63·2-79·4)	74·2 (61·9-82·9)	75·6 (68·7-84·8)	74·8 (67·8-84·3)	76·8 (69·9-86·0)	78·6 (71·9-87·4)
New Zealand	69·0 (67·0-71·2)	70·8 (66·5-73·9)	71·2 (66·6-74·5)	74·3 (64·1-78·4)	83·0 (82·0-83·9)	86·2 (85·3-87·2)	86·5 (85·6-87·5)	89·7 (87·9-91·4)	64·3 (61·7-67·2)	65·3 (60·0-69·3)	65·5 (59·8-69·7)	67·7 (55·5-73·4)	67·2 (59·9-77·8)	34·7 (28·3-44·5)	37·2 (30·7-47·2)	39·9 (33·3-50·0)
**High-income Asia Pacific**																
Brunei	69·3 (62·2-72·9)	73·4 (69·6-77·2)	73·7 (69·7-77·8)	75·8 (69·4-81·7)	72·4 (70·9-73·6)	75·5 (74·0-76·9)	75·6 (74·0-77·1)	77·4 (74·3-80·2)	69·2 (59·9-73·9)	73·7 (69·3-78·5)	74·1 (69·4-79·3)	75·3 (67·0-83·0)	100·0 (100·0-100·0)	63·6 (54·9-80·2)	66·4 (57·8-82·4)	68·0 (59·5-83·6)
Japan	71·3 (70·0-73·0)	74·0 (71·0-77·3)	74·7 (72·2-77·7)	77·1 (74·5-80·2)	87·8 (86·6-88·8)	92·6 (91·4-93·7)	92·9 (91·8-94·0)	95·0 (93·0-96·7)	66·7 (65·1-68·8)	68·6 (64·9-72·7)	69·3 (66·2-73·0)	71·3 (67·9-75·1)	33·2 (23·9-52·6)	16·5 (6·7-36·7)	18·9 (9·2-39·1)	22·0 (12·4-42·0)
Singapore	79·0 (77·3-80·6)	86·6 (84·6-88·5)	86·8 (84·6-88·9)	88·6 (84·5-91·3)	83·4 (82·0-84·6)	90·4 (89·1-91·9)	90·8 (89·5-92·3)	93·3 (91·8-94·6)	77·5 (75·3-79·6)	83·8 (81·1-86·4)	83·9 (80·9-86·9)	83·7 (67·9-89·0)	77·3 (69·7-90·2)	100·0 (100·0-100·0)	100·0 (100·0-100·0)	96·5 (94·1-99·1)
South Korea	60·0 (57·9-61·9)	72·1 (67·0-75·9)	72·5 (67·2-76·6)	76·9 (68·6-83·0)	81·1 (79·6-82·3)	89·8 (88·2-91·5)	90·2 (88·3-91·9)	93·6 (91·3-95·3)	53·8 (50·9-56·2)	64·8 (58·7-69·7)	65·2 (58·8-70·2)	67·3 (53·0-76·9)	41·2 (32·1-60·0)	70·0 (61·6-85·2)	73·2 (65·1-87·4)	59·3 (50·4-76·5)
**Western Europe**																
Andorra	53·6 (51·9-58·4)	62·7 (54·8-66·9)	63·0 (54·9-66·9)	65·7 (58·6-69·2)	92·7 (90·9-94·4)	94·2 (92·1-95·9)	94·2 (92·2-95·9)	94·7 (92·3-96·7)	44·4 (42·4-49·7)	54·1 (45·3-58·9)	54·3 (45·4-58·8)	56·9 (47·9-61·4)	100·0 (100·0-100·0)	100·0 (100·0-100·0)	100·0 (100·0-100·0)	100·0 (100·0-100·0)
Austria	65·9 (60·9-68·5)	73·8 (64·2-77·4)	74·4 (64·6-78·2)	78·5 (67·4-83·1)	85·3 (84·0-86·4)	92·3 (91·3-93·3)	92·9 (91·9-93·8)	96·7 (95·3-97·8)	60·9 (54·8-64·2)	68·1 (56·7-72·6)	68·7 (57·0-73·3)	72·0 (58·7-77·7)	52·7 (45·7-63·1)	51·0 (44·2-61·5)	54·3 (47·3-64·8)	50·6 (43·8-61·0)
Belgium	69·9 (68·5-71·1)	77·3 (73·4-79·5)	77·0 (72·9-79·5)	81·7 (69·4-85·6)	86·3 (85·2-87·3)	90·8 (89·8-91·9)	91·1 (90·1-92·2)	94·8 (93·0-96·2)	65·5 (63·7-67·1)	73·1 (68·3-75·9)	72·7 (67·5-75·7)	76·8 (62·5-81·8)	100·0 (100·0-100·0)	100·0 (100·0-100·0)	100·0 (100·0-100·0)	100·0 (100·0-100·0)
Cyprus	66·1 (64·4-67·6)	75·3 (72·9-77·1)	76·0 (73·7-77·8)	79·8 (75·5-82·8)	82·5 (81·3-83·7)	91·0 (89·7-92·2)	91·2 (90·0-92·5)	95·9 (94·4-97·1)	61·7 (59·5-63·6)	69·3 (66·1-71·8)	70·0 (66·8-72·6)	71·7 (62·4-76·9)	100·0 (100·0-100·0)	77·1 (70·9-84·3)	84·8 (77·7-94·8)	71·4 (65·4-77·5)
Denmark	63·2 (57·3-68·1)	76·9 (67·4-81·5)	77·3 (67·6-81·9)	84·3 (72·2-88·4)	87·9 (86·8-89·0)	92·6 (91·4-93·8)	92·9 (91·8-94·1)	95·7 (93·9-97·2)	56·2 (49·2-62·3)	70·7 (59·2-76·6)	71·1 (59·5-77·0)	77·9 (63·8-84·1)	100·0 (100·0-100·0)	100·0 (100·0-100·0)	100·0 (100·0-100·0)	100·0 (100·0-100·0)
Finland	72·2 (68·8-74·5)	82·5 (72·2-86·5)	82·8 (72·4-86·8)	87·4 (76·3-91·2)	90·0 (88·9-91·0)	96·5 (95·6-97·4)	96·7 (95·9-97·5)	98·0 (97·2-98·9)	67·2 (63·0-70·1)	77·3 (64·8-82·2)	77·6 (65·0-82·6)	82·7 (69·1-87·8)	54·7 (47·7-65·1)	100·0 (100·0-100·0)	100·0 (100·0-100·0)	88·2 (82·8-94·1)
France	59·9 (55·1-65·7)	72·4 (63·5-76·8)	73·4 (64·4-78·0)	80·1 (68·8-84·6)	86·3 (85·2-87·3)	91·6 (90·7-92·4)	91·8 (90·9-92·7)	94·7 (92·9-96·2)	53·4 (47·8-60·3)	66·8 (56·2-72·5)	67·9 (57·0-73·6)	74·8 (61·2-80·8)	63·2 (56·2-73·4)	80·2 (73·7-88·3)	82·4 (76·0-90·0)	79·6 (73·0-87·9)
Germany	71·0 (68·2-73·0)	78·3 (73·5-80·7)	78·4 (73·6-80·8)	83·2 (76·9-86·3)	86·4 (85·2-87·4)	91·5 (90·3-92·6)	91·9 (90·6-93·1)	95·9 (94·0-97·3)	66·7 (63·0-69·2)	73·5 (67·6-76·6)	73·4 (67·5-76·6)	76·9 (67·2-81·6)	100·0 (100·0-100·0)	100·0 (100·0-100·0)	100·0 (100·0-100·0)	100·0 (100·0-100·0)
Greece	58·7 (56·6-60·3)	66·0 (60·6-70·9)	68·4 (62·4-72·6)	73·1 (63·4-78·8)	84·7 (83·4-86·0)	89·2 (88·0-90·2)	89·4 (88·2-90·5)	93·0 (90·6-95·2)	51·8 (49·5-53·8)	58·0 (51·7-63·9)	60·7 (53·2-66·0)	62·9 (48·8-71·9)	43·9 (37·2-54·1)	100·0 (100·0-100·0)	100·0 (100·0-100·0)	74·7 (67·8-84·0)
Iceland	73·4 (71·9-74·9)	85·4 (83·4-86·9)	86·0 (84·1-87·6)	83·7 (78·8-86·8)	89·1 (88·0-90·0)	95·7 (94·9-96·8)	96·0 (95·2-97·1)	97·5 (96·5-98·6)	67·6 (65·3-69·8)	78·6 (74·7-81·6)	79·3 (75·3-82·3)	73·9 (61·3-80·7)	18·5 (13·4-26·2)	85·7 (78·8-95·2)	100·0 (100·0-100·0)	52·2 (47·1-58·7)
Ireland	62·5 (59·7-68·9)	74·2 (67·8-81·4)	74·8 (67·9-81·8)	82·4 (71·1-87·9)	84·2 (83·0-85·4)	90·9 (89·9-92·1)	91·3 (90·2-92·6)	95·7 (93·4-97·5)	56·5 (52·9-64·1)	68·1 (60·1-77·0)	68·6 (60·1-77·3)	75·3 (60·8-83·2)	100·0 (100·0-100·0)	100·0 (100·0-100·0)	100·0 (100·0-100·0)	100·0 (100·0-100·0)
Israel	75·7 (74·7-76·7)	81·2 (79·6-82·6)	81·9 (80·1-83·5)	85·4 (81·7-88·3)	83·2 (82·0-84·5)	90·0 (88·6-91·4)	90·4 (89·1-91·9)	95·4 (93·5-96·9)	73·6 (72·0-75·0)	77·2 (74·9-79·2)	77·8 (75·5-80·1)	78·4 (65·1-83·6)	100·0 (100·0-100·0)	100·0 (100·0-100·0)	100·0 (100·0-100·0)	100·0 (100·0-100·0)
Italy	70·2 (64·4-73·0)	78·0 (74·1-80·6)	77·6 (73·6-80·2)	81·2 (75·4-85·0)	86·4 (85·2-87·6)	92·0 (91·0-92·9)	92·3 (91·3-93·3)	95·4 (93·7-96·7)	64·1 (56·9-67·7)	71·2 (65·6-75·0)	70·5 (65·2-74·3)	71·2 (58·4-78·8)	66·5 (59·5-76·5)	59·8 (52·7-70·1)	49·0 (41·9-57·4)	53·0 (46·3-60·6)
Luxembourg	68·2 (61·3-71·0)	69·2 (67·5-76·6)	69·5 (67·6-77·4)	74·8 (68·4-83·6)	87·6 (86·4-88·7)	93·6 (92·6-94·5)	93·8 (92·9-94·8)	96·0 (94·7-97·2)	62·6 (54·4-66·0)	61·3 (58·6-70·2)	61·6 (58·7-71·0)	65·5 (54·4-77·0)	55·1 (48·9-63·3)	100·0 (100·0-100·0)	100·0 (100·0-100·0)	77·1 (71·6-82·4)
Malta	75·0 (73·5-76·5)	80·6 (78·5-82·4)	80·9 (78·7-82·9)	83·4 (78·6-86·9)	81·6 (80·4-82·8)	84·9 (83·7-86·3)	85·3 (84·0-86·8)	89·6 (87·2-91·5)	71·4 (67·6-74·2)	76·4 (71·7-80·0)	76·6 (71·9-80·2)	75·9 (63·7-83·9)	65·8 (56·5-82·4)	68·5 (59·3-84·5)	70·3 (61·3-85·9)	72·7 (63·9-87·6)
Netherlands	74·2 (73·1-75·4)	82·8 (81·0-84·2)	83·0 (80·9-84·6)	86·4 (82·1-88·7)	86·4 (85·3-87·5)	93·8 (92·8-94·8)	94·0 (93·1-95·0)	96·7 (95·5-97·9)	71·3 (69·8-72·8)	78·9 (76·5-80·9)	79·0 (76·4-81·1)	81·6 (75·2-85·2)	100·0 (100·0-100·0)	100·0 (100·0-100·0)	100·0 (100·0-100·0)	100·0 (100·0-100·0)
Norway	75·9 (74·7-77·1)	84·2 (82·2-85·8)	84·5 (82·5-86·2)	87·5 (84·6-89·7)	88·8 (87·7-89·9)	94·7 (93·7-95·6)	94·9 (94·0-95·8)	97·1 (96·0-98·1)	72·0 (70·5-73·6)	79·5 (76·7-81·8)	79·8 (76·9-82·2)	81·3 (75·2-85·4)	61·6 (55·2-69·9)	53·9 (46·9-64·6)	56·3 (49·2-66·9)	55·5 (49·2-64·2)
Portugal	58·6 (56·8-64·1)	69·4 (63·8-76·1)	70·2 (64·2-76·7)	77·5 (66·0-83·9)	77·0 (75·3-78·6)	86·4 (85·0-87·9)	86·9 (85·5-88·4)	92·3 (90·8-94·0)	53·0 (50·5-60·0)	63·1 (56·0-71·3)	63·9 (56·4-72·0)	70·4 (56·0-79·1)	74·8 (67·7-84·5)	65·7 (58·6-75·8)	68·4 (61·3-78·3)	69·4 (62·3-79·3)
Spain	62·5 (59·2-68·4)	73·6 (67·5-80·7)	74·3 (67·9-81·3)	80·3 (70·5-86·7)	85·6 (84·5-86·6)	91·3 (90·4-92·2)	91·6 (90·7-92·5)	95·5 (93·7-96·9)	55·4 (51·0-62·8)	66·5 (58·6-75·3)	67·1 (59·0-75·9)	71·8 (58·2-81·5)	63·9 (56·9-74·0)	100·0 (100·0-100·0)	100·0 (100·0-100·0)	90·9 (86·3-95·7)

Location	Indicator 2.2.1: Prevalence of stunting in children under 5 (%)				Indicator 2.2.2a: Prevalence of wasting in children under 5 (%)				Indicator 2.2.2b: Prevalence of overweight in children aged 2-4 (%)				Indicator 3.1.1: Maternal mortality ratio (maternal deaths per 100,000 livebirths) in women aged 10-54 years			
	**2000**	**2015**	**2016**	**2030**	**2000**	**2015**	**2016**	**2030**	**2000**	**2015**	**2016**	**2030**	**2000**	**2015**	**2016**	**2030**
**High-income North America**																
Canada	97·4 (96·6-98·1)	98·0 (97·4-98·6)	98·1 (97·4-98·6)	98·5 (97·9-99·0)	99·1 (95·6-100·0)	99·3 (96·0-100·0)	99·3 (96·0-100·0)	99·3 (95·7-100·0)	61·4 (47·9-72·3)	47·0 (30·4-61·8)	46·6 (29·6-61·2)	33·4 (0·0-62·0)	86·4 (83·4-89·2)	83·4 (80·5-86·2)	83·8 (80·8-86·6)	87·2 (78·3-95·3)
United States	97·4 (97·2-97·6)	98·4 (98·2-98·6)	98·4 (98·2-98·6)	98·9 (98·7-99·1)	99·9 (99·3-100·0)	100·0 (99·6-100·0)	100·0 (99·6-100·0)	100·0 (99·9-100·0)	65·4 (56·7-73·2)	58·9 (46·8-69·9)	58·0 (45·1-69·3)	50·4 (22·7-72·8)	71·3 (69·5-73·1)	59·2 (57·1-61·1)	59·6 (57·5-61·7)	51·2 (47·7-54·8)
**Australasia**																
Australia	95·6 (94·1-97·0)	96·4 (95·2-97·6)	96·5 (95·2-97·7)	97·3 (96·1-98·4)	97·0 (93·5-99·6)	98·4 (94·9-100·0)	98·5 (95·0-100·0)	99·1 (95·7-100·0)	69·0 (62·5-74·8)	54·8 (44·5-64·3)	54·1 (43·6-63·8)	36·8 (16·1-57·5)	85·6 (82·4-88·3)	91·4 (88·0-94·3)	91·6 (88·1-94·7)	94·2 (83·7-100·0)
New Zealand	95·2 (93·8-96·6)	96·0 (94·7-97·3)	96·1 (94·8-97·3)	97·0 (95·8-98·2)	96·8 (93·2-99·3)	98·3 (94·8-100·0)	98·3 (94·9-100·0)	99·2 (96·1-100·0)	42·3 (30·1-53·7)	39·5 (29·2-49·6)	39·0 (28·3-49·3)	32·8 (12·3-53·0)	79·7 (76·6-82·7)	76·4 (72·9-79·8)	76·3 (72·1-80·5)	75·4 (62·7-89·5)
**High-income Asia Pacific**																
Brunei	83·3 (80·1-86·2)	86·0 (83·2-88·7)	86·3 (83·6-89·0)	89·6 (87·0-91·8)	82·7 (74·1-89·3)	86·4 (80·1-91·6)	86·6 (80·1-91·8)	88·9 (84·4-93·5)	82·9 (70·0-92·4)	69·6 (53·5-83·2)	69·1 (53·3-82·7)	53·4 (16·3-82·0)	53·5 (48·6-58·6)	55·6 (49·3-61·3)	55·8 (48·1-62·3)	60·8 (40·3-78·8)
Japan	96·5 (96·3-96·8)	94·9 (94·6-95·3)	95·0 (94·6-95·3)	95·5 (95·2-95·9)	92·5 (91·5-93·4)	94·6 (93·6-95·5)	94·6 (93·6-95·5)	95·3 (94·2-96·3)	88·8 (81·5-94·6)	81·0 (67·3-90·7)	80·6 (66·9-90·4)	74·5 (48·2-92·7)	82·0 (80·0-83·9)	89·5 (87·3-91·6)	89·7 (87·3-91·9)	95·1 (90·0-100·0)
Singapore	95·7 (94·5-97·0)	96·3 (95·0-97·6)	96·3 (95·1-97·6)	97·1 (96·0-98·2)	80·9 (74·9-85·6)	84·5 (76·1-89·8)	84·7 (76·4-90·0)	87·6 (79·7-92·8)	72·7 (55·7-85·1)	59·5 (40·1-74·9)	58·8 (39·6-74·6)	42·7 (0·1-75·7)	74·2 (69·7-78·4)	86·3 (81·7-91·3)	89·3 (83·1-96·1)	99·6 (94·3-100·0)
South Korea	97·4 (96·1-98·4)	96·0 (94·7-97·3)	96·1 (94·8-97·3)	96·8 (95·2-98·1)	94·6 (90·4-98·0)	94·0 (89·1-97·6)	94·1 (89·2-97·7)	94·8 (90·1-98·5)	56·1 (36·9-72·6)	40·7 (19·0-61·0)	39·9 (17·6-59·6)	26·8 (0·0-62·1)	72·0 (67·7-76·3)	77·9 (70·0-85·5)	78·4 (69·1-87·2)	86·4 (64·8-100·0)
**Western Europe**																
Andorra	99·7 (98·9-100·0)	99·8 (98·8-100·0)	99·8 (98·8-100·0)	99·8 (98·9-100·0)	97·5 (94·2-99·5)	98·5 (95·1-100·0)	98·6 (95·2-100·0)	98·8 (95·5-100·0)	56·4 (42·1-68·9)	52·5 (37·3-65·9)	52·5 (37·2-66·0)	46·2 (15·7-71·2)	96·9 (89·8-100·0)	96·1 (88·6-100·0)	96·1 (88·1-100·0)	94·2 (77·5-100·0)
Austria	99·6 (98·7-100·0)	99·7 (98·8-100·0)	99·7 (98·8-100·0)	99·8 (98·8-100·0)	97·3 (93·7-99·6)	98·4 (94·7-100·0)	98·5 (94·8-100·0)	98·8 (95·3-100·0)	66·3 (54·4-77·4)	64·0 (50·9-75·6)	63·6 (49·7-75·1)	58·0 (30·2-80·1)	87·8 (84·4-91·2)	96·8 (93·4-100·0)	97·8 (94·4-100·0)	100·0 (100·0-100·0)
Belgium	99·6 (98·8-100·0)	99·7 (98·9-100·0)	99·8 (98·9-100·0)	99·8 (99·0-100·0)	97·4 (94·1-99·4)	98·5 (94·9-100·0)	98·5 (94·9-100·0)	99·0 (95·4-100·0)	69·5 (58·0-79·7)	69·0 (56·1-79·4)	68·9 (56·4-79·2)	67·4 (44·2-85·5)	81·3 (78·4-84·3)	86·3 (83·0-89·7)	86·7 (82·9-90·7)	91·6 (78·8-100·0)
Cyprus	99·4 (98·7-99·8)	99·6 (98·8-100·0)	99·7 (98·8-100·0)	99·8 (99·0-100·0)	97·0 (93·6-99·2)	98·4 (95·0-100·0)	98·4 (95·1-100·0)	98·9 (95·5-100·0)	55·7 (41·8-67·5)	45·7 (30·4-60·2)	45·4 (30·1-59·7)	33·7 (3·6-59·7)	64·9 (58·2-71·4)	79·8 (74·3-84·9)	80·0 (74·5-85·3)	91·3 (79·7-100·0)
Denmark	99·8 (99·0-100·0)	99·8 (98·9-100·0)	99·8 (98·9-100·0)	99·8 (98·9-100·0)	97·6 (94·5-99·7)	98·6 (95·4-100·0)	98·7 (95·4-100·0)	99·0 (95·9-100·0)	48·2 (33·2-62·3)	43·9 (27·8-58·0)	44·2 (28·2-58·2)	39·0 (9·0-63·9)	94·3 (91·0-97·8)	95·3 (91·6-99·2)	96·1 (92·0-100·0)	99·2 (91·2-100·0)
Finland	99·6 (98·8-100·0)	99·8 (98·8-100·0)	99·8 (98·8-100·0)	99·8 (98·9-100·0)	97·4 (94·2-99·5)	98·5 (95·1-100·0)	98·5 (95·1-100·0)	98·9 (95·6-100·0)	66·8 (53·7-77·8)	63·9 (50·9-74·7)	63·5 (50·1-74·5)	60·9 (35·3-80·2)	85·3 (82·3-88·1)	98·9 (96·4-100·0)	99·2 (96·6-100·0)	99·7 (96·4-100·0)
France	99·5 (98·8-99·9)	99·6 (98·9-100·0)	99·6 (98·9-100·0)	99·8 (99·0-100·0)	97·2 (94·0-99·4)	98·3 (95·0-100·0)	98·3 (95·0-100·0)	98·7 (95·5-100·0)	66·9 (54·0-77·2)	63·9 (50·3-75·4)	63·9 (50·7-75·6)	60·2 (34·7-80·3)	78·5 (75·7-81·3)	84·9 (81·9-88·0)	85·1 (81·9-88·5)	91·0 (80·9-100·0)
Germany	99·9 (99·0-100·0)	99·9 (99·0-100·0)	99·9 (99·0-100·0)	99·9 (99·0-100·0)	98·9 (95·3-100·0)	99·0 (95·8-100·0)	99·0 (95·8-100·0)	99·1 (95·8-100·0)	62·5 (49·9-73·6)	54·4 (38·7-67·5)	53·8 (38·3-67·2)	43·8 (13·5-68·3)	77·7 (74·7-80·6)	83·0 (79·7-86·6)	83·4 (79·8-87·2)	88·5 (79·6-98·6)
Greece	99·3 (98·7-99·7)	99·6 (98·7-100·0)	99·6 (98·7-100·0)	99·8 (98·7-100·0)	97·0 (93·6-99·2)	98·3 (94·8-100·0)	98·3 (94·8-100·0)	98·8 (95·3-100·0)	49·6 (36·5-62·0)	34·3 (17·7-49·5)	34·6 (18·7-50·0)	21·1 (0·0-47·3)	87·8 (84·7-90·9)	81·9 (78·4-85·5)	81·9 (78·2-85·7)	83·6 (71·2-98·4)
Iceland	99·7 (98·8-100·0)	99·8 (98·9-100·0)	99·8 (98·9-100·0)	99·8 (99·0-100·0)	97·6 (94·2-99·6)	98·6 (94·9-100·0)	98·6 (95·0-100·0)	98·9 (95·2-100·0)	43·7 (28·6-57·4)	32·1 (14·4-47·7)	31·8 (14·0-47·1)	21·7 (0·0-49·0)	100·0 (100·0-100·0)	100·0 (100·0-100·0)	100·0 (100·0-100·0)	100·0 (100·0-100·0)
Ireland	99·5 (98·8-99·9)	99·7 (98·8-100·0)	99·7 (98·8-100·0)	99·9 (99·0-100·0)	97·1 (93·8-99·2)	98·5 (94·9-100·0)	98·5 (94·9-100·0)	99·0 (95·6-100·0)	50·7 (37·5-62·9)	46·3 (30·2-59·9)	45·7 (29·6-59·0)	37·1 (5·3-62·9)	98·1 (94·7-100·0)	91·9 (87·8-95·9)	92·2 (87·2-96·6)	95·2 (80·1-100·0)
Israel	99·1 (98·5-99·5)	99·2 (98·6-99·7)	99·2 (98·6-99·7)	99·4 (98·9-99·9)	96·7 (93·3-98·9)	98·0 (94·5-100·0)	98·1 (94·5-100·0)	98·6 (95·2-100·0)	61·7 (48·4-73·0)	48·5 (32·7-62·9)	48·2 (32·0-63·2)	32·7 (1·5-60·3)	86·2 (81·9-90·2)	89·1 (83·7-94·2)	89·5 (83·4-95·2)	98·8 (87·7-100·0)
Italy	99·5 (98·8-99·9)	99·6 (98·9-100·0)	99·6 (98·9-100·0)	99·7 (99·0-100·0)	97·2 (93·9-99·2)	98·4 (95·0-100·0)	98·4 (95·1-100·0)	98·7 (95·3-100·0)	43·0 (27·6-58·0)	34·6 (18·9-50·5)	34·7 (18·2-51·1)	20·6 (0·0-49·7)	88·8 (85·7-91·7)	97·2 (93·9-100·0)	97·4 (93·9-100·0)	97·9 (90·2-100·0)
Luxembourg	99·8 (98·9-100·0)	99·8 (98·8-100·0)	99·8 (98·8-100·0)	99·8 (98·7-100·0)	97·6 (94·4-99·7)	98·7 (95·3-100·0)	98·7 (95·3-100·0)	98·8 (95·5-100·0)	59·2 (46·2-71·3)	45·4 (29·7-58·9)	45·2 (29·8-58·6)	30·6 (0·8-57·1)	83·4 (80·1-86·4)	79·9 (76·4-83·7)	80·3 (76·6-84·2)	85·6 (73·8-98·2)
Malta	99·3 (98·7-99·8)	99·6 (98·8-100·0)	99·6 (98·8-100·0)	99·7 (98·9-100·0)	97·1 (93·7-99·1)	98·4 (95·3-100·0)	98·4 (95·4-100·0)	98·9 (96·0-100·0)	30·2 (13·3-46·1)	25·7 (8·9-42·3)	24·8 (8·3-42·1)	19·0 (0·0-49·5)	85·7 (81·6-89·9)	90·9 (85·7-96·2)	91·4 (85·7-97·6)	99·9 (99·6-100·0)
Netherlands	99·7 (98·7-100·0)	99·8 (98·9-100·0)	99·8 (98·9-100·0)	99·9 (99·1-100·0)	97·6 (94·1-99·7)	98·6 (95·1-100·0)	98·6 (95·2-100·0)	99·0 (95·8-100·0)	74·6 (64·5-83·6)	67·3 (54·9-78·2)	67·1 (54·5-78·2)	58·9 (32·9-79·6)	74·3 (71·3-77·3)	89·3 (86·1-92·4)	89·1 (85·2-92·9)	96·8 (87·1-100·0)
Norway	99·7 (98·8-100·0)	99·8 (98·8-100·0)	99·8 (98·8-100·0)	99·8 (98·7-100·0)	97·5 (94·2-99·6)	98·6 (94·9-100·0)	98·6 (94·9-100·0)	99·1 (95·7-100·0)	65·9 (54·1-76·3)	53·1 (38·7-66·6)	52·6 (37·7-66·4)	39·7 (11·6-65·4)	91·8 (88·4-95·2)	98·4 (95·2-100·0)	98·2 (94·8-100·0)	99·4 (92·3-100·0)
Portugal	98·8 (98·2-99·3)	99·2 (98·6-99·6)	99·2 (98·6-99·7)	99·5 (98·9-99·9)	96·4 (93·3-98·6)	97·9 (94·4-100·0)	97·9 (94·4-100·0)	98·4 (94·9-100·0)	46·8 (32·6-60·4)	41·6 (25·2-57·0)	41·4 (24·8-56·3)	37·1 (7·0-62·5)	77·8 (75·0-80·5)	78·5 (75·3-81·6)	79·1 (75·7-82·5)	79·6 (71·6-88·1)
Spain	99·7 (99·0-100·0)	99·9 (99·1-100·0)	99·9 (99·1-100·0)	99·9 (99·2-100·0)	95·4 (91·7-97·6)	96·0 (92·4-98·3)	96·0 (92·4-98·4)	96·4 (92·6-98·8)	41·9 (25·7-57·3)	36·0 (19·9-51·3)	35·7 (19·6-51·2)	28·1 (0·0-55·7)	87·3 (84·4-90·1)	92·6 (89·5-95·4)	92·5 (89·3-95·5)	98·8 (93·4-100·0)

Location	Indicator 3.1.2: Proportion of births attended by skilled health personnel (%)				Indicator 3.2.1: Under-5 mortality rate (probability of dying before the age of 5 per 1,000 livebirths)				Indicator 3.2.2: Neonatal mortality rate (probability of dying during the first 28 days of life per 1,000 livebirths)				Indicator 3.3.1: Age-standardised rate of new HIV infections (per 1,000 population)			
	**2000**	**2015**	**2016**	**2030**	**2000**	**2015**	**2016**	**2030**	**2000**	**2015**	**2016**	**2030**	**2000**	**2015**	**2016**	**2030**
**High-income North America**																
Canada	98·8 (98·3-99·2)	99·2 (98·8-99·5)	99·2 (98·8-99·5)	99·5 (98·9-99·9)	77·3 (73·7-80·4)	80·3 (76·8-83·5)	80·5 (77·7-83·2)	83·9 (81·0-86·9)	69·3 (66·1-72·2)	72·0 (68·7-75·1)	72·3 (69·6-74·8)	75·1 (71·6-78·6)	55·4 (47·6-64·5)	56·5 (45·8-74·8)	56·6 (45·9-74·9)	57·9 (47·1-77·3)
United States	97·9 (97·1-98·5)	99·0 (98·5-99·3)	99·0 (98·5-99·3)	99·5 (98·7-99·9)	70·7 (67·6-73·7)	74·5 (71·2-77·7)	74·9 (72·2-77·4)	79·3 (76·2-82·0)	62·3 (59·3-65·2)	66·2 (62·9-69·2)	66·6 (63·9-69·1)	71·0 (66·5-75·4)	44·7 (39·8-50·3)	50·8 (41·8-65·8)	50·9 (41·8-65·8)	54·3 (45·0-70·8)
**Australasia**																
Australia	98·9 (98·2-99·3)	99·4 (98·9-99·7)	99·4 (99·0-99·7)	99·7 (99·1-100·0)	76·6 (73·1-79·8)	87·4 (84·5-90·0)	88·0 (85·8-90·1)	96·1 (91·9-100·0)	69·0 (65·9-71·8)	80·8 (78·2-83·0)	81·5 (79·4-83·6)	91·9 (86·3-96·9)	62·7 (54·9-71·8)	63·0 (55·7-71·7)	63·0 (55·7-71·7)	64·4 (56·9-73·4)
New Zealand	98·8 (98·1-99·3)	99·4 (99·1-99·7)	99·5 (99·1-99·8)	99·8 (99·2-100·0)	72·6 (69·5-75·6)	79·0 (76·5-81·1)	79·7 (77·6-81·7)	88·3 (85·0-91·4)	69·3 (66·1-72·2)	71·6 (69·2-73·6)	72·7 (70·6-74·6)	72·8 (67·1-78·0)	64·8 (57·3-73·6)	66·1 (57·0-76·6)	66·1 (57·0-76·7)	67·3 (57·7-78·5)
**High-income Asia Pacific**																
Brunei	98·6 (97·6-99·3)	98·9 (98·0-99·5)	98·9 (98·0-99·5)	99·1 (97·1-100·0)	69·0 (65·9-72·0)	68·0 (66·8-69·1)	68·1 (66·7-69·2)	66·9 (61·9-71·1)	65·9 (62·6-68·8)	63·9 (62·3-65·2)	64·0 (62·2-65·3)	63·0 (57·3-67·9)	47·5 (39·1-56·4)	49·3 (41·1-58·8)	49·4 (41·2-58·8)	49·8 (41·8-59·4)
Japan	98·7 (97·8-99·4)	99·2 (98·5-99·7)	99·2 (98·5-99·7)	99·5 (98·1-100·0)	84·5 (80·8-88·1)	96·3 (93·3-99·0)	97·0 (94·5-99·3)	100·0 (100·0-100·0)	87·4 (83·4-91·4)	100·0 (100·0-100·0)	100·0 (100·0-100·0)	100·0 (100·0-100·0)	78·8 (72·3-85·3)	78·1 (70·6-87·1)	78·0 (70·5-87·0)	79·5 (72·0-88·1)
Singapore	99·1 (98·3-99·6)	99·6 (99·1-99·8)	99·6 (99·1-99·8)	99·8 (99·0-100·0)	88·3 (84·4-91·9)	99·2 (96·7-100·0)	99·7 (98·2-100·0)	100·0 (100·0-100·0)	88·5 (84·5-92·2)	99·9 (98·9-100·0)	100·0 (100·0-100·0)	100·0 (100·0-100·0)	54·5 (45·0-65·1)	54·0 (45·5-65·1)	54·0 (45·7-65·2)	55·2 (46·7-66·7)
South Korea	98·8 (97·8-99·4)	99·5 (99·0-99·8)	99·5 (99·0-99·8)	99·8 (99·0-100·0)	74·2 (70·9-77·5)	90·1 (84·7-95·4)	90·4 (84·5-96·8)	97·5 (88·7-100·0)	71·6 (68·0-75·3)	91·7 (85·6-98·3)	92·1 (85·4-99·6)	98·9 (91·2-100·0)	59·5 (44·6-71·4)	74·2 (63·1-92·6)	74·2 (63·1-92·5)	75·4 (63·8-96·6)
**Western Europe**																
Andorra	99·5 (99·3-99·6)	99·7 (99·6-99·8)	99·7 (99·6-99·8)	99·7 (99·5-99·9)	92·9 (87·2-98·4)	91·8 (85·3-97·5)	91·9 (85·4-97·6)	93·3 (83·0-100·0)	99·4 (95·4-100·0)	98·3 (93·6-100·0)	98·4 (93·7-100·0)	98·1 (89·9-100·0)	65·1 (45·5-87·3)	65·3 (43·5-87·8)	65·5 (43·8-88·0)	66·1 (44·5-88·6)
Austria	99·3 (99·1-99·5)	99·7 (99·6-99·8)	99·8 (99·6-99·9)	99·9 (99·8-100·0)	79·1 (75·4-82·7)	90·4 (88·3-92·6)	91·4 (89·6-92·8)	99·4 (93·8-100·0)	72·4 (68·6-76·3)	84·9 (82·7-87·2)	86·0 (83·1-88·2)	97·8 (87·1-100·0)	53·0 (44·0-62·7)	53·9 (45·1-64·4)	53·9 (45·1-64·4)	55·2 (46·0-66·3)
Belgium	98·3 (97·8-98·7)	99·2 (99·0-99·4)	99·3 (99·0-99·5)	99·6 (99·3-99·9)	78·5 (75·0-82·0)	87·1 (84·6-89·3)	87·6 (85·6-89·6)	95·6 (91·2-98·7)	73·2 (69·8-76·2)	83·7 (81·1-86·2)	84·4 (81·8-86·8)	95·4 (89·9-100·0)	54·6 (44·9-65·0)	54·1 (46·1-66·0)	54·2 (46·1-66·1)	55·7 (47·3-67·7)
Cyprus	96·9 (96·1-97·6)	98·9 (98·6-99·2)	99·0 (98·7-99·2)	99·6 (99·3-99·8)	71·0 (67·6-74·0)	91·5 (87·1-95·8)	92·0 (87·5-96·5)	99·9 (98·7-100·0)	61·4 (58·3-64·4)	87·6 (82·5-93·1)	88·2 (83·1-94·2)	99·9 (99·4-100·0)	61·0 (53·2-70·0)	55·1 (47·9-63·8)	55·1 (48·0-63·9)	58·3 (50·9-67·0)
Denmark	97·8 (97·2-98·3)	99·0 (98·7-99·3)	99·1 (98·8-99·3)	99·6 (99·2-99·8)	79·5 (75·5-83·0)	86·5 (84·5-88·4)	87·0 (85·5-88·4)	92·3 (83·9-98·8)	71·4 (67·8-74·8)	78·1 (76·0-80·3)	78·7 (76·2-80·7)	84·2 (74·1-92·0)	57·2 (48·7-66·1)	61·0 (51·0-75·2)	61·0 (51·0-75·2)	62·6 (52·4-76·8)
Finland	99·2 (98·9-99·4)	99·7 (99·6-99·8)	99·7 (99·6-99·8)	100·0 (99·9-100·0)	86·0 (81·4-90·0)	99·8 (97·9-100·0)	99·9 (99·3-100·0)	100·0 (100·0-100·0)	79·5 (75·1-83·7)	98·2 (94·6-100·0)	98·5 (95·4-100·0)	100·0 (100·0-100·0)	67·3 (58·7-75·9)	70·5 (60·7-84·4)	70·6 (60·8-84·5)	72·1 (62·4-86·5)
France	99·1 (98·8-99·3)	99·7 (99·5-99·8)	99·7 (99·6-99·8)	99·9 (99·8-100·0)	80·4 (76·8-83·9)	88·0 (85·6-89·8)	88·5 (86·6-89·8)	95·0 (86·2-100·0)	75·6 (72·1-78·9)	83·2 (81·1-84·6)	83·6 (80·7-85·7)	90·0 (79·5-99·0)	54·7 (44·2-64·4)	64·0 (54·4-74·7)	64·0 (54·5-74·7)	64·5 (54·8-75·4)
Germany	98·1 (97·6-98·5)	99·1 (98·8-99·3)	99·1 (98·8-99·3)	99·5 (99·2-99·8)	80·5 (76·9-83·9)	89·0 (84·4-93·0)	89·8 (84·1-94·9)	97·1 (87·3-100·0)	75·2 (71·8-78·5)	84·7 (80·4-88·5)	85·7 (80·3-90·2)	94·7 (82·9-100·0)	62·3 (54·9-70·9)	59·8 (52·7-68·7)	59·8 (52·7-68·8)	60·5 (53·1-69·4)
Greece	98·3 (97·8-98·7)	99·1 (98·9-99·4)	99·2 (99·0-99·4)	99·6 (99·3-99·8)	76·7 (73·0-80·0)	87·4 (84·7-89·8)	87·9 (85·9-89·8)	95·7 (89·8-100·0)	66·9 (63·5-69·9)	81·0 (78·4-83·2)	81·6 (79·6-83·5)	90·7 (82·9-97·3)	65·5 (57·8-74·4)	62·0 (54·0-71·6)	61·9 (54·0-71·5)	63·8 (55·4-73·1)
Iceland	99·1 (98·8-99·4)	99·7 (99·5-99·8)	99·7 (99·5-99·8)	99·9 (99·7-100·0)	87·3 (83·2-91·0)	99·6 (98·1-100·0)	100·0 (99·6-100·0)	100·0 (100·0-100·0)	85·2 (80·9-89·0)	100·0 (99·2-100·0)	100·0 (100·0-100·0)	100·0 (100·0-100·0)	57·5 (48·0-68·2)	59·9 (50·8-76·1)	59·9 (50·8-76·1)	62·7 (53·3-79·4)
Ireland	98·4 (97·9-98·7)	99·5 (99·3-99·6)	99·5 (99·3-99·6)	99·8 (99·7-100·0)	73·6 (69·9-76·9)	87·4 (85·2-89·5)	87·9 (86·4-89·3)	95·4 (87·2-100·0)	65·4 (62·1-68·6)	80·7 (78·4-83·0)	81·3 (78·9-83·3)	90·3 (80·1-99·5)	56·9 (46·7-68·8)	54·3 (45·5-67·9)	54·2 (45·5-67·8)	56·0 (47·4-68·8)
Israel	97·2 (96·5-97·8)	98·9 (98·6-99·2)	99·0 (98·6-99·2)	99·6 (99·2-99·8)	74·1 (70·7-77·3)	88·7 (86·2-90·8)	89·9 (88·3-91·4)	100·0 (100·0-100·0)	67·9 (64·6-71·0)	86·5 (84·0-88·6)	87·7 (85·7-89·5)	99·9 (99·5-100·0)	60·0 (50·9-70·5)	58·5 (50·9-70·3)	58·4 (50·8-70·2)	59·7 (51·9-71·6)
Italy	98·5 (98·0-98·8)	99·3 (99·1-99·5)	99·3 (99·1-99·5)	99·6 (99·3-99·8)	80·6 (77·0-84·1)	92·0 (90·4-93·5)	92·6 (89·7-94·4)	99·3 (93·7-100·0)	70·3 (67·0-73·6)	85·2 (83·1-87·1)	86·1 (82·1-88·6)	97·5 (87·6-100·0)	53·5 (45·9-62·7)	53·8 (46·2-63·1)	54·0 (46·5-63·3)	55·1 (47·4-63·9)
Luxembourg	98·6 (98·2-98·9)	99·4 (99·2-99·6)	99·4 (99·3-99·6)	99·8 (99·6-99·9)	83·7 (79·9-87·3)	99·8 (98·7-100·0)	100·0 (100·0-100·0)	100·0 (100·0-100·0)	79·4 (75·7-83·0)	98·9 (96·4-100·0)	99·7 (97·8-100·0)	100·0 (100·0-100·0)	51·7 (42·4-62·1)	51·5 (43·5-63·4)	51·5 (43·4-63·4)	53·7 (45·3-65·8)
Malta	98·1 (97·6-98·6)	99·1 (98·8-99·3)	99·2 (98·9-99·4)	99·6 (99·4-99·8)	72·0 (68·7-75·1)	76·4 (74·9-77·6)	76·9 (75·4-78·1)	84·8 (75·8-94·1)	58·6 (55·8-61·6)	62·5 (60·3-64·3)	63·1 (60·6-65·0)	73·0 (62·7-82·7)	59·8 (49·7-70·8)	48·5 (40·1-61·1)	48·6 (40·2-61·2)	52·3 (43·5-65·1)
Netherlands	98·8 (98·5-99·1)	99·6 (99·4-99·7)	99·6 (99·4-99·7)	99·9 (99·6-100·0)	76·7 (73·1-80·0)	88·0 (84·8-91·0)	88·5 (86·6-90·5)	96·0 (91·1-100·0)	67·0 (63·7-70·1)	79·5 (76·3-82·4)	80·2 (78·1-82·1)	89·6 (83·9-95·0)	58·1 (49·5-67·4)	65·5 (57·4-75·9)	65·6 (57·4-75·9)	66·9 (58·4-78·0)
Norway	98·5 (98·1-98·9)	99·5 (99·3-99·6)	99·5 (99·3-99·7)	99·9 (99·7-100·0)	82·5 (78·4-86·2)	95·7 (93·6-97·6)	96·4 (94·8-97·9)	99·9 (99·1-100·0)	76·3 (72·5-79·8)	90·9 (88·7-93·0)	91·8 (89·5-93·6)	99·6 (95·3-100·0)	60·3 (51·1-70·1)	63·2 (55·0-73·6)	63·2 (55·1-73·7)	65·2 (56·6-76·1)
Portugal	98·4 (98·0-98·7)	99·5 (99·4-99·7)	99·6 (99·4-99·7)	99·9 (99·8-100·0)	74·3 (71·1-77·4)	92·1 (89·2-94·7)	93·1 (90·4-95·6)	100·0 (100·0-100·0)	68·3 (65·4-71·2)	89·9 (86·8-92·5)	91·1 (88·1-93·7)	100·0 (100·0-100·0)	25·6 (16·5-35·6)	32·3 (24·2-44·7)	32·2 (24·2-44·7)	39·0 (30·5-52·1)
Spain	85·4 (82·5-88·0)	93·7 (92·2-95·1)	93·9 (92·3-95·3)	97·2 (95·6-98·3)	80·8 (77·2-84·4)	91·7 (89·1-93·9)	92·1 (90·4-93·6)	99·2 (95·1-100·0)	74·8 (71·5-78·1)	88·0 (85·8-89·8)	88·4 (86·5-90·2)	98·9 (93·8-100·0)	49·9 (43·0-58·0)	51·0 (44·4-58·7)	51·0 (44·3-58·7)	57·4 (50·1-65·6)

Location	Indicator 3.3.2: Age-standardised rate of tuberculosis cases (per 100,000 population)				Indicator 3.3.3: Age-standardised rate of malaria cases (per 1,000 population)				Indicator 3.3.4: Age-standardised rate of hepatitis B incidence (per 100,000 population)				Indicator 3.3.5: Age-standardised prevalence* of the sum of 15 neglected tropical diseases (NTDs) (%) *Prevalence estimates reported here may exceed 100% as they reflect the sum of prevalent cases of 15 NTDs.*			
	**2000**	**2015**	**2016**	**2030**	**2000**	**2015**	**2016**	**2030**	**2000**	**2015**	**2016**	**2030**	**2000**	**2015**	**2016**	**2030**
**High-income North America**																
Canada	98·0 (95·5-100·0)	100·0 (99·3-100·0)	99·9 (99·0-100·0)	100·0 (100·0-100·0)	100·0 (100·0-100·0)	100·0 (100·0-100·0)	100·0 (100·0-100·0)	100·0 (100·0-100·0)	65·5 (61·8-69·0)	81·8 (79·3-84·1)	81·8 (79·3-84·1)	81·1 (78·5-83·6)	100·0 (100·0-100·0)	100·0 (100·0-100·0)	100·0 (100·0-100·0)	100·0 (100·0-100·0)
United States	98·5 (96·0-100·0)	100·0 (100·0-100·0)	100·0 (100·0-100·0)	100·0 (100·0-100·0)	100·0 (100·0-100·0)	100·0 (100·0-100·0)	100·0 (100·0-100·0)	100·0 (100·0-100·0)	54·8 (50·0-59·7)	88·1 (85·6-90·5)	88·0 (85·6-90·5)	87·4 (85·0-89·9)	99·9 (99·9-99·9)	99·9 (99·9-99·9)	99·9 (99·9-99·9)	99·9 (99·9-99·9)
**Australasia**																
Australia	97·7 (95·8-99·4)	99·6 (98·1-100·0)	99·8 (98·5-100·0)	100·0 (100·0-100·0)	100·0 (100·0-100·0)	100·0 (100·0-100·0)	100·0 (100·0-100·0)	100·0 (100·0-100·0)	63·9 (60·5-67·0)	72·7 (69·3-75·4)	73·6 (70·3-76·2)	81·1 (77·6-84·1)	100·0 (100·0-100·0)	100·0 (100·0-100·0)	100·0 (100·0-100·0)	100·0 (100·0-100·0)
New Zealand	89·3 (87·7-90·8)	95·2 (93·5-96·7)	95·4 (93·8-97·0)	99·7 (98·3-100·0)	100·0 (100·0-100·0)	100·0 (100·0-100·0)	100·0 (100·0-100·0)	100·0 (100·0-100·0)	77·7 (75·2-80·2)	84·9 (82·5-87·6)	85·5 (83·0-88·1)	93·5 (91·1-96·1)	100·0 (100·0-100·0)	100·0 (100·0-100·0)	100·0 (100·0-100·0)	100·0 (100·0-100·0)
**High-income Asia Pacific**																
Brunei	44·4 (42·5-46·3)	52·9 (50·8-54·8)	53·3 (51·2-55·2)	60·3 (58·2-62·4)	100·0 (100·0-100·0)	100·0 (100·0-100·0)	100·0 (100·0-100·0)	100·0 (100·0-100·0)	32·8 (29·5-36·0)	66·5 (64·3-68·7)	66·8 (64·6-69·0)	71·0 (68·8-73·2)	99·9 (99·9-100·0)	99·8 (99·7-99·8)	99·8 (99·7-99·8)	99·8 (99·6-99·9)
Japan	70·2 (67·6-72·6)	82·3 (79·9-84·7)	82·8 (80·4-85·2)	90·4 (87·9-93·0)	100·0 (100·0-100·0)	100·0 (100·0-100·0)	100·0 (100·0-100·0)	100·0 (100·0-100·0)	47·8 (43·2-52·4)	75·2 (73·4-76·7)	75·3 (73·6-76·9)	77·6 (75·9-79·3)	99·9 (99·9-99·9)	99·9 (99·9-99·9)	99·9 (99·9-99·9)	99·9 (99·9-99·9)
Singapore	56·8 (54·6-59·0)	63·5 (61·6-65·5)	63·3 (61·4-65·3)	65·4 (63·5-67·4)	100·0 (100·0-100·0)	100·0 (100·0-100·0)	100·0 (100·0-100·0)	100·0 (100·0-100·0)	67·2 (63·3-70·6)	86·0 (82·7-88·9)	86·7 (83·5-89·6)	98·1 (95·0-100·0)	100·0 (99·9-100·0)	99·9 (99·8-99·9)	99·9 (99·8-99·9)	99·9 (99·8-99·9)
South Korea	34·7 (33·1-36·4)	49·7 (47·8-51·6)	50·1 (48·2-52·0)	63·8 (61·4-66·1)	65·3 (61·2-68·0)	77·4 (72·8-80·4)	79·6 (74·9-82·6)	82·9 (78·3-85·9)	59·9 (55·9-63·5)	73·9 (70·2-77·4)	74·2 (70·6-77·7)	79·8 (76·1-83·3)	97·4 (97·1-97·6)	97·6 (97·3-97·8)	97·6 (97·3-97·8)	97·6 (97·3-97·9)
**Western Europe**																
Andorra	79·1 (76·3-81·8)	86·2 (83·7-88·5)	86·5 (83·9-88·7)	91·0 (88·1-93·7)	100·0 (100·0-100·0)	100·0 (100·0-100·0)	100·0 (100·0-100·0)	100·0 (100·0-100·0)	93·8 (91·2-95·9)	97·9 (95·6-99·7)	98·0 (95·7-99·7)	99·3 (96·9-100·0)	100·0 (100·0-100·0)	100·0 (99·9-100·0)	100·0 (99·9-100·0)	100·0 (99·9-100·0)
Austria	83·5 (81·4-85·6)	92·2 (90·3-94·1)	92·3 (90·4-94·3)	100·0 (100·0-100·0)	100·0 (100·0-100·0)	100·0 (100·0-100·0)	100·0 (100·0-100·0)	100·0 (100·0-100·0)	85·8 (82·6-88·7)	100·0 (100·0-100·0)	100·0 (100·0-100·0)	100·0 (100·0-100·0)	100·0 (100·0-100·0)	100·0 (100·0-100·0)	100·0 (100·0-100·0)	100·0 (100·0-100·0)
Belgium	84·4 (82·5-86·2)	89·3 (87·4-91·1)	89·6 (87·6-91·3)	93·8 (91·7-95·8)	100·0 (100·0-100·0)	100·0 (100·0-100·0)	100·0 (100·0-100·0)	100·0 (100·0-100·0)	79·9 (76·4-83·7)	92·8 (90·1-95·4)	92·6 (89·9-95·3)	90·3 (87·0-93·6)	100·0 (100·0-100·0)	100·0 (100·0-100·0)	100·0 (100·0-100·0)	100·0 (100·0-100·0)
Cyprus	79·7 (77·3-81·8)	87·7 (85·5-89·8)	88·0 (85·8-90·1)	92·4 (90·0-94·6)	100·0 (100·0-100·0)	100·0 (100·0-100·0)	100·0 (100·0-100·0)	100·0 (100·0-100·0)	78·8 (76·4-81·5)	91·2 (89·2-93·0)	91·5 (89·6-93·4)	96·8 (95·0-98·6)	100·0 (100·0-100·0)	100·0 (100·0-100·0)	100·0 (100·0-100·0)	100·0 (100·0-100·0)
Denmark	86·3 (84·4-88·3)	95·0 (93·0-97·2)	95·2 (93·2-97·4)	99·2 (96·8-100·0)	100·0 (100·0-100·0)	100·0 (100·0-100·0)	100·0 (100·0-100·0)	100·0 (100·0-100·0)	94·7 (92·6-96·9)	96·6 (94·8-98·5)	97·0 (95·2-98·8)	100·0 (100·0-100·0)	100·0 (100·0-100·0)	100·0 (100·0-100·0)	100·0 (100·0-100·0)	100·0 (100·0-100·0)
Finland	90·6 (88·6-92·4)	97·5 (95·7-99·1)	97·7 (95·9-99·4)	100·0 (99·4-100·0)	100·0 (100·0-100·0)	100·0 (100·0-100·0)	100·0 (100·0-100·0)	100·0 (100·0-100·0)	92·4 (89·7-94·5)	100·0 (100·0-100·0)	100·0 (100·0-100·0)	100·0 (100·0-100·0)	100·0 (100·0-100·0)	100·0 (100·0-100·0)	100·0 (100·0-100·0)	100·0 (100·0-100·0)
France	83·1 (81·2-85·2)	91·5 (89·7-93·4)	91·7 (89·8-93·6)	98·8 (97·3-100·0)	100·0 (100·0-100·0)	100·0 (100·0-100·0)	100·0 (100·0-100·0)	100·0 (100·0-100·0)	79·9 (77·2-82·6)	98·9 (97·5-100·0)	99·0 (97·7-100·0)	99·9 (99·1-100·0)	100·0 (100·0-100·0)	100·0 (100·0-100·0)	100·0 (100·0-100·0)	100·0 (100·0-100·0)
Germany	87·1 (84·7-89·4)	95·0 (93·0-96·8)	94·1 (92·0-96·0)	100·0 (99·8-100·0)	100·0 (100·0-100·0)	100·0 (100·0-100·0)	100·0 (100·0-100·0)	100·0 (100·0-100·0)	80·8 (76·6-86·7)	97·8 (94·8-100·0)	98·5 (95·7-100·0)	100·0 (100·0-100·0)	100·0 (100·0-100·0)	100·0 (100·0-100·0)	100·0 (100·0-100·0)	100·0 (100·0-100·0)
Greece	95·5 (93·6-97·4)	99·5 (98·0-100·0)	99·6 (98·1-100·0)	100·0 (100·0-100·0)	100·0 (100·0-100·0)	100·0 (100·0-100·0)	100·0 (100·0-100·0)	100·0 (100·0-100·0)	74·0 (71·0-77·1)	67·9 (64·2-71·6)	68·5 (64·8-72·1)	71·7 (67·9-75·4)	100·0 (100·0-100·0)	100·0 (100·0-100·0)	100·0 (100·0-100·0)	100·0 (100·0-100·0)
Iceland	79·7 (77·4-82·1)	87·9 (85·4-90·2)	88·2 (85·8-90·6)	93·5 (90·9-96·0)	100·0 (100·0-100·0)	100·0 (100·0-100·0)	100·0 (100·0-100·0)	100·0 (100·0-100·0)	99·0 (96·8-100·0)	100·0 (100·0-100·0)	100·0 (100·0-100·0)	100·0 (100·0-100·0)	100·0 (100·0-100·0)	100·0 (100·0-100·0)	100·0 (100·0-100·0)	100·0 (100·0-100·0)
Ireland	85·7 (84·0-87·5)	92·4 (90·6-94·3)	92·9 (91·2-94·8)	99·9 (98·8-100·0)	100·0 (100·0-100·0)	100·0 (100·0-100·0)	100·0 (100·0-100·0)	100·0 (100·0-100·0)	77·2 (74·3-80·0)	88·0 (85·5-90·2)	88·6 (86·1-90·7)	96·7 (94·8-98·6)	100·0 (100·0-100·0)	100·0 (100·0-100·0)	100·0 (100·0-100·0)	100·0 (100·0-100·0)
Israel	90·0 (88·2-91·9)	99·9 (98·9-100·0)	100·0 (99·3-100·0)	100·0 (100·0-100·0)	100·0 (100·0-100·0)	100·0 (100·0-100·0)	100·0 (100·0-100·0)	100·0 (100·0-100·0)	74·8 (71·6-77·6)	91·7 (89·5-93·6)	92·0 (89·7-93·9)	95·3 (93·1-97·1)	100·0 (99·9-100·0)	100·0 (100·0-100·0)	100·0 (100·0-100·0)	100·0 (100·0-100·0)
Italy	92·9 (91·4-94·3)	95·5 (94·0-97·0)	96·2 (94·6-97·6)	100·0 (100·0-100·0)	100·0 (100·0-100·0)	100·0 (100·0-100·0)	100·0 (100·0-100·0)	100·0 (100·0-100·0)	56·9 (52·5-61·5)	79·3 (76·4-82·4)	79·4 (76·6-82·4)	80·6 (77·6-83·7)	100·0 (100·0-100·0)	100·0 (100·0-100·0)	100·0 (100·0-100·0)	100·0 (100·0-100·0)
Luxembourg	83·0 (80·6-85·4)	91·6 (89·1-94·0)	91·9 (89·4-94·3)	96·7 (94·1-99·3)	100·0 (100·0-100·0)	100·0 (100·0-100·0)	100·0 (100·0-100·0)	100·0 (100·0-100·0)	81·8 (78·5-84·7)	97·7 (95·9-99·3)	98·1 (96·2-99·6)	100·0 (100·0-100·0)	100·0 (100·0-100·0)	100·0 (100·0-100·0)	100·0 (100·0-100·0)	100·0 (100·0-100·0)
Malta	88·9 (86·1-91·4)	94·9 (92·2-97·3)	95·2 (92·4-97·5)	98·5 (95·9-100·0)	100·0 (100·0-100·0)	100·0 (100·0-100·0)	100·0 (100·0-100·0)	100·0 (100·0-100·0)	81·6 (78·7-84·1)	96·5 (94·8-98·3)	97·0 (95·3-98·8)	100·0 (100·0-100·0)	100·0 (100·0-100·0)	100·0 (100·0-100·0)	100·0 (100·0-100·0)	100·0 (100·0-100·0)
Netherlands	88·5 (86·6-90·4)	97·1 (95·2-99·0)	97·5 (95·7-99·5)	100·0 (100·0-100·0)	100·0 (100·0-100·0)	100·0 (100·0-100·0)	100·0 (100·0-100·0)	100·0 (100·0-100·0)	94·6 (92·3-96·5)	98·2 (95·9-100·0)	98·0 (95·7-99·8)	96·2 (93·6-98·2)	100·0 (100·0-100·0)	100·0 (100·0-100·0)	100·0 (100·0-100·0)	100·0 (100·0-100·0)
Norway	93·9 (92·2-95·6)	95·9 (93·8-97·8)	96·2 (94·1-98·1)	99·2 (97·0-100·0)	100·0 (100·0-100·0)	100·0 (100·0-100·0)	100·0 (100·0-100·0)	100·0 (100·0-100·0)	90·5 (87·5-92·8)	99·6 (97·8-100·0)	99·8 (98·3-100·0)	100·0 (100·0-100·0)	100·0 (100·0-100·0)	100·0 (100·0-100·0)	100·0 (100·0-100·0)	100·0 (100·0-100·0)
Portugal	62·9 (60·4-65·5)	75·0 (72·8-77·2)	75·5 (73·3-77·7)	84·3 (82·0-86·6)	100·0 (100·0-100·0)	100·0 (100·0-100·0)	100·0 (100·0-100·0)	100·0 (100·0-100·0)	53·0 (49·0-56·8)	83·2 (80·6-85·8)	83·8 (81·3-86·4)	93·2 (90·9-95·6)	99·2 (98·9-99·4)	99·3 (99·1-99·5)	99·3 (99·1-99·5)	99·3 (99·1-99·5)
Spain	76·6 (73·7-79·5)	88·1 (85·8-90·1)	89·1 (86·8-91·1)	100·0 (99·9-100·0)	100·0 (100·0-100·0)	100·0 (100·0-100·0)	100·0 (100·0-100·0)	100·0 (100·0-100·0)	75·2 (70·7-79·5)	93·7 (90·3-96·9)	94·2 (90·9-97·3)	99·9 (98·4-100·0)	99·8 (99·8-99·9)	99·8 (99·7-99·8)	99·8 (99·7-99·8)	99·8 (99·8-99·9)

Location	Indicator 3.4.1: Age-standardised death rate due to cardiovascular disease, cancer, diabetes, and chronic respiratory disease in populations aged 30-70 (per 100,000 population)				Indicator 3.4.2: Age-standardised death rate due to self-harm (per 100,000 population)				Indicator 3.5.2: Risk-weighted prevalence of alcohol consumption, as measured by the summary exposure value (SEV) for alcohol use (%)				Indicator 3.6.1: Age-standardised death rate due to road injuries (per 100,000 population)			
	**2000**	**2015**	**2016**	**2030**	**2000**	**2015**	**2016**	**2030**	**2000**	**2015**	**2016**	**2030**	**2000**	**2015**	**2016**	**2030**
**High-income North America**																
Canada	70·8 (68·7-73·1)	86·7 (84·3-89·0)	87·2 (84·5-89·9)	99·8 (97·3-100·0)	37·7 (34·5-45·6)	42·2 (37·5-48·9)	42·6 (37·4-49·3)	46·7 (37·4-55·4)	43·8 (33·9-54·2)	36·3 (19·6-52·0)	36·3 (19·6-52·1)	37·0 (18·2-54·5)	59·0 (56·3-61·5)	73·3 (70·3-75·9)	73·8 (70·5-76·5)	81·8 (74·4-89·3)
United States	57·7 (56·0-59·6)	69·9 (68·0-71·8)	69·7 (67·7-71·7)	78·9 (74·8-82·7)	39·7 (32·7-42·5)	35·5 (31·4-40·6)	35·6 (31·4-40·8)	32·0 (27·5-40·8)	45·9 (36·3-54·8)	41·1 (23·6-56·8)	41·1 (22·8-57·5)	40·9 (11·2-66·5)	41·7 (39·6-44·1)	51·4 (49·4-53·9)	51·3 (49·3-53·8)	57·4 (53·8-60·7)
**Australasia**																
Australia	76·6 (74·4-78·8)	93·9 (90·7-97·3)	93·9 (89·9-98·0)	100·0 (100·0-100·0)	36·7 (33·4-41·4)	44·0 (38·5-50·5)	44·1 (38·3-50·7)	48·1 (38·1-59·4)	29·5 (16·6-42·7)	23·4 (2·2-43·8)	23·2 (1·2-44·3)	21·1 (0·0-54·1)	58·4 (55·8-61·3)	78·7 (75·9-81·5)	78·6 (75·3-82·0)	97·6 (92·3-100·0)
New Zealand	65·7 (63·4-68·0)	85·8 (82·0-89·5)	85·8 (79·9-91·5)	99·2 (93·3-100·0)	35·6 (32·2-41·2)	41·7 (36·1-48·8)	41·6 (35·0-49·0)	46·9 (34·7-60·9)	34·8 (24·3-46·7)	28·5 (7·2-48·3)	28·6 (6·7-48·7)	29·5 (0·6-54·4)	50·8 (48·0-53·5)	71·7 (68·1-75·0)	71·6 (67·0-76·2)	92·1 (85·1-100·0)
**High-income Asia Pacific**																
Brunei	51·8 (46·9-57·1)	58·5 (50·3-67·5)	59·0 (48·9-70·7)	63·7 (38·4-88·3)	79·9 (73·1-86·1)	76·3 (67·5-85·0)	76·4 (65·2-87·8)	75·4 (46·6-100·0)	99·3 (97·5-100·0)	99·4 (97·2-100·0)	99·3 (97·1-100·0)	97·8 (76·7-100·0)	35·1 (30·6-39·8)	46·5 (39·8-54·1)	46·9 (38·3-56·0)	52·6 (28·6-74·6)
Japan	82·9 (81·1-84·8)	99·6 (97·7-100·0)	99·8 (98·6-100·0)	100·0 (100·0-100·0)	19·4 (17·0-23·9)	23·3 (19·3-29·3)	23·8 (19·4-29·5)	30·0 (21·2-37·0)	59·9 (51·3-67·5)	61·4 (49·1-72·2)	61·2 (48·6-72·2)	57·6 (39·8-72·1)	67·5 (65·3-69·8)	93·7 (90·7-95·8)	94·2 (91·2-96·3)	100·0 (100·0-100·0)
Singapore	64·5 (58·1-70·7)	88·9 (81·6-96·0)	90·0 (79·0-100·0)	98·6 (85·3-100·0)	37·3 (30·6-42·7)	53·5 (39·9-61·1)	53·4 (40·5-63·6)	69·3 (43·1-94·2)	86·5 (81·3-90·8)	87·5 (81·8-92·3)	87·5 (81·6-92·4)	86·9 (79·1-92·8)	78·3 (72·8-83·9)	98·4 (93·7-100·0)	98·0 (91·4-100·0)	99·7 (95·6-100·0)
South Korea	60·1 (54·9-65·7)	89·3 (76·8-100·0)	89·0 (74·5-100·0)	98·7 (83·4-100·0)	16·4 (9·5-23·1)	8·8 (0·0-24·4)	9·3 (0·0-25·6)	18·5 (0·0-53·9)	51·7 (40·8-62·6)	54·7 (37·3-69·2)	54·9 (37·2-69·5)	58·2 (35·7-74·8)	23·9 (19·8-28·5)	50·7 (42·1-60·3)	50·8 (40·4-61·6)	70·0 (45·7-96·9)
**Western Europe**																
Andorra	86·1 (74·7-96·9)	91·3 (79·8-100·0)	91·2 (79·5-100·0)	89·4 (64·8-100·0)	52·1 (38·1-65·7)	54·2 (41·8-67·5)	54·0 (41·2-67·2)	53·5 (30·4-76·7)	0·5 (0·0-5·6)	19·7 (0·0-44·8)	20·6 (0·0-45·9)	33·2 (3·6-59·6)	80·5 (70·9-91·5)	90·5 (81·4-99·8)	90·7 (81·2-100·0)	95·2 (79·3-100·0)
Austria	67·4 (65·2-69·5)	85·7 (82·6-88·7)	86·5 (82·9-89·9)	97·7 (89·5-100·0)	23·7 (14·2-27·1)	36·6 (21·5-41·7)	36·8 (21·5-42·5)	47·1 (27·8-58·9)	16·6 (3·0-30·4)	23·5 (0·0-46·4)	23·9 (0·0-47·3)	29·4 (0·0-58·0)	56·8 (54·2-60·4)	86·8 (83·8-89·8)	87·0 (83·6-90·4)	100·0 (100·0-100·0)
Belgium	66·2 (63·3-68·9)	82·1 (77·7-86·3)	82·4 (76·8-88·2)	96·1 (85·7-100·0)	18·1 (12·8-21·4)	27·0 (17·5-32·1)	26·9 (17·3-32·9)	32·5 (17·5-46·6)	46·5 (35·2-56·9)	34·4 (10·8-53·4)	34·5 (9·7-53·9)	35·3 (0·0-62·9)	43·0 (40·3-46·7)	69·6 (65·8-73·0)	69·5 (65·0-73·7)	92·0 (83·1-100·0)
Cyprus	65·6 (62·9-68·3)	83·3 (79·9-86·6)	83·3 (79·9-86·7)	96·9 (90·9-100·0)	67·0 (58·4-73·6)	77·1 (69·3-82·1)	76·8 (69·0-81·9)	84·5 (74·5-93·9)	42·0 (31·0-53·6)	45·6 (25·6-63·0)	45·8 (25·3-63·7)	48·8 (21·4-71·4)	29·7 (26·3-35·0)	58·4 (54·9-62·3)	58·0 (54·3-62·0)	80·8 (73·5-88·4)
Denmark	57·1 (53·7-60·8)	81·8 (76·2-87·3)	82·4 (75·9-89·0)	98·7 (89·7-100·0)	29·7 (19·8-34·1)	46·2 (28·8-53·5)	46·3 (29·0-54·5)	61·7 (36·5-78·8)	9·1 (0·0-25·8)	19·8 (0·0-44·9)	20·7 (0·0-46·2)	33·9 (0·0-63·8)	61·4 (58·3-64·8)	93·0 (88·8-97·5)	92·7 (87·8-97·6)	100·0 (100·0-100·0)
Finland	69·6 (67·0-71·9)	90·2 (87·4-93·2)	91·6 (87·0-96·5)	100·0 (100·0-100·0)	13·4 (9·5-16·2)	31·9 (18·6-37·4)	32·4 (18·6-39·0)	52·3 (28·9-67·7)	19·5 (7·2-33·0)	21·0 (0·0-41·6)	21·7 (0·0-42·6)	31·5 (1·5-56·2)	66·4 (63·5-69·2)	93·1 (89·9-96·0)	93·8 (89·9-97·9)	100·0 (100·0-100·0)
France	71·2 (68·9-73·6)	85·2 (82·3-88·1)	85·9 (82·4-89·1)	97·4 (91·3-100·0)	18·8 (10·4-22·5)	28·7 (16·7-33·5)	28·8 (16·8-34·0)	38·9 (22·7-48·5)	6·8 (0·0-25·6)	19·6 (0·0-45·3)	20·3 (0·0-46·5)	30·0 (0·0-61·2)	50·1 (47·2-55·1)	78·3 (74·9-82·9)	78·5 (74·8-83·1)	99·2 (94·3-100·0)
Germany	64·1 (61·1-67·1)	80·6 (76·3-84·8)	81·3 (76·6-86·0)	92·9 (81·4-100·0)	34·0 (25·6-37·9)	43·9 (31·2-49·5)	44·0 (30·9-50·0)	49·6 (32·2-62·3)	23·7 (9·8-36·8)	33·5 (8·6-53·4)	34·0 (8·6-54·3)	41·0 (7·0-65·0)	60·9 (58·1-64·7)	88·9 (85·7-92·2)	89·2 (85·6-92·8)	100·0 (100·0-100·0)
Greece	69·8 (67·2-72·2)	79·4 (75·8-83·0)	79·5 (74·6-84·0)	86·6 (77·1-96·2)	90·7 (73·5-96·6)	86·0 (75·4-92·0)	86·1 (75·4-93·1)	82·7 (69·9-96·6)	27·4 (13·3-40·5)	32·0 (7·8-53·5)	32·5 (7·9-54·3)	39·2 (7·4-65·8)	38·7 (36·3-42·1)	57·1 (54·0-60·3)	57·0 (53·1-60·9)	74·1 (67·3-81·6)
Iceland	76·6 (73·4-79·7)	93·7 (89·6-97·8)	93·5 (88·5-98·4)	98·1 (87·7-100·0)	36·1 (27·1-40·2)	42·6 (32·2-47·5)	42·8 (32·6-48·3)	46·7 (33·8-58·2)	67·9 (59·8-75·8)	56·9 (38·6-71·4)	56·3 (37·6-71·2)	48·1 (18·4-69·2)	71·6 (68·6-75·4)	96·7 (93·3-100·0)	94·8 (90·9-99·3)	100·0 (100·0-100·0)
Ireland	58·7 (55·3-62·1)	85·3 (79·7-90·4)	85·5 (78·2-92·5)	98·7 (90·0-100·0)	36·7 (31·7-46·9)	46·3 (39·1-59·0)	46·5 (38·3-59·2)	50·1 (31·6-69·3)	2·8 (0·0-14·8)	10·2 (0·0-35·5)	11·2 (0·0-37·0)	24·9 (0·0-57·1)	56·9 (52·4-60·5)	90·1 (84·9-94·5)	90·1 (84·0-95·4)	100·0 (100·0-100·0)
Israel	69·4 (63·5-75·3)	92·1 (83·5-100·0)	92·2 (81·5-100·0)	99·2 (89·7-100·0)	46·4 (40·0-52·2)	56·7 (43·0-65·5)	56·9 (42·7-67·5)	67·4 (41·9-92·3)	88·6 (85·0-91·9)	84·1 (77·6-89·3)	83·8 (77·0-89·2)	79·4 (68·3-87·6)	49·6 (45·0-54·7)	70·0 (63·8-75·5)	70·1 (62·9-76·9)	89·5 (71·4-100·0)
Italy	74·0 (71·9-76·3)	91·1 (87·8-94·5)	91·4 (87·4-95·3)	99·5 (95·2-100·0)	60·8 (49·4-65·0)	69·2 (54·6-75·8)	69·1 (54·4-76·3)	73·6 (56·7-86·7)	22·0 (3·8-39·6)	37·9 (12·2-60·9)	38·5 (12·2-62·0)	47·3 (13·2-74·3)	46·8 (44·7-50·1)	69·0 (65·8-72·0)	69·1 (65·6-72·7)	89·3 (83·3-94·9)
Luxembourg	67·7 (65·0-70·8)	89·1 (84·0-94·3)	89·8 (84·1-95·6)	99·3 (91·6-100·0)	34·1 (25·4-37·9)	48·7 (32·6-55·1)	48·8 (32·2-55·9)	59·7 (35·0-76·6)	16·7 (1·5-31·2)	0·9 (0·0-10·6)	1·1 (0·0-12·0)	7·1 (0·0-31·4)	53·5 (50·7-57·1)	85·1 (81·2-88·8)	84·9 (80·3-89·0)	99·9 (98·5-100·0)
Malta	64·4 (59·5-69·0)	88·0 (79·9-95·6)	88·3 (78·8-97·9)	97·4 (81·5-100·0)	70·3 (58·9-76·4)	76·8 (62·4-85·8)	77·0 (62·7-87·4)	83·3 (57·9-100·0)	70·6 (62·6-78·2)	65·0 (49·9-77·6)	64·5 (48·6-77·5)	56·4 (27·6-77·5)	79·3 (74·8-84·4)	99·5 (95·9-100·0)	99·4 (94·9-100·0)	99·9 (100·0-100·0)
Netherlands	63·5 (60·6-66·3)	85·8 (82·6-88·9)	85·8 (80·7-90·5)	98·8 (91·8-100·0)	43·8 (35·6-47·6)	48·9 (40·6-54·3)	49·3 (41·1-55·2)	53·6 (40·7-67·1)	46·9 (36·6-56·8)	48·9 (31·1-64·0)	48·8 (30·2-64·4)	47·7 (17·0-70·6)	67·3 (64·7-70·7)	94·8 (92·2-97·4)	94·5 (90·8-98·2)	100·0 (100·0-100·0)
Norway	71·3 (68·5-73·9)	92·3 (88·0-96·8)	92·5 (87·0-98·6)	99·9 (99·8-100·0)	36·3 (31·0-39·8)	46·9 (36·6-52·6)	46·7 (35·7-53·1)	51·6 (35·5-66·6)	58·9 (48·8-68·0)	53·7 (34·4-68·5)	53·7 (34·0-68·7)	53·8 (29·3-71·3)	69·1 (65·8-72·8)	98·4 (95·1-100·0)	98·2 (93·8-100·0)	100·0 (100·0-100·0)
Portugal	64·1 (61·8-66·4)	84·0 (80·6-87·6)	84·5 (80·6-88·9)	99·5 (94·7-100·0)	41·2 (31·5-45·4)	47·1 (37·9-52·3)	47·3 (37·9-53·2)	51·2 (38·0-64·2)	1·4 (0·0-9·5)	8·4 (0·0-29·0)	9·1 (0·0-30·3)	19·4 (0·0-48·6)	33·2 (30·5-37·7)	66·4 (63·1-69·8)	66·6 (62·9-70·7)	99·3 (94·9-100·0)
Spain	73·9 (71·9-76·1)	91·5 (88·8-94·4)	92·3 (89·1-95·5)	100·0 (100·0-100·0)	58·9 (48·3-62·8)	66·6 (54·9-72·0)	66·8 (55·0-72·6)	74·3 (58·8-86·5)	3·6 (0·0-15·8)	9·3 (0·0-33·3)	10·1 (0·0-34·9)	20·9 (0·0-54·6)	48·7 (46·4-52·4)	89·4 (87·0-91·8)	89·0 (86·3-91·8)	100·0 (100·0-100·0)

Location	Indicator 3.7.1: Proportion of women of reproductive age (15-49 years) who have their need for family planning satisfied with modern contraception methods (%)				Indicator 3.7.2: Number of livebirths per 1,000 women aged 10-14 years and women aged 15-19 years				Indicator 3.8.1: Coverage of essential health services, as defined by the UHC index comprised of the coverage of 9 tracer interventions and risk-standardised death rates from 32 causes amenable to personal healthcare (scale of 0 to 100)				Indicator 3.9.1: Age-standardised death rate attributable to household air pollution and ambient air pollution (per 100,000 population)			
	**2000**	**2015**	**2016**	**2030**	**2000**	**2015**	**2016**	**2030**	**2000**	**2015**	**2016**	**2030**	**2000**	**2015**	**2016**	**2030**
**High-income North America**																
Canada	94·1 (89·3-98·5)	94·0 (88·4-98·6)	94·1 (88·6-98·7)	95·1 (83·9-100·0)	55·6 (53·5-57·7)	66·4 (64·2-68·4)	67·8 (64·9-70·2)	84·8 (75·7-91·4)	83·9 (82·3-85·6)	91·4 (89·4-93·6)	91·8 (89·7-94·0)	96·8 (93·8-99·5)	84·8 (82·4-87·2)	97·9 (95·7-99·8)	98·1 (95·9-100·0)	100·0 (100·0-100·0)
United States	91·3 (87·7-94·4)	82·3 (75·0-88·6)	82·6 (74·7-89·2)	86·2 (63·8-100·0)	29·3 (28·0-30·7)	50·9 (49·5-52·1)	54·3 (52·9-55·5)	75·1 (71·5-78·6)	74·8 (73·6-76·1)	79·4 (78·1-80·7)	79·4 (78·0-80·7)	79·2 (77·1-81·1)	69·4 (67·1-71·8)	81·8 (79·4-84·0)	81·7 (79·4-83·9)	88·9 (81·9-92·8)
**Australasia**																
Australia	99·3 (96·0-100·0)	99·9 (98·1-100·0)	99·9 (98·1-100·0)	99·8 (96·5-100·0)	53·9 (51·9-55·9)	60·9 (59·6-62·0)	61·8 (60·4-63·1)	70·1 (67·2-72·9)	83·3 (81·7-84·9)	95·7 (93·5-97·9)	96·3 (94·1-98·5)	100·0 (100·0-100·0)	92·0 (88·5-95·7)	100·0 (100·0-100·0)	100·0 (100·0-100·0)	100·0 (100·0-100·0)
New Zealand	92·2 (86·3-97·6)	95·0 (89·6-99·5)	95·1 (90·1-99·5)	97·0 (87·5-100·0)	42·6 (40·8-44·3)	47·4 (46·3-48·3)	47·9 (45·6-49·7)	59·3 (53·1-65·2)	76·0 (74·2-77·7)	88·5 (85·5-91·7)	89·2 (86·0-92·5)	97·6 (92·8-100·0)	91·5 (86·6-96·5)	100·0 (100·0-100·0)	100·0 (100·0-100·0)	100·0 (100·0-100·0)
**High-income Asia Pacific**																
Brunei	76·4 (66·8-84·6)	83·1 (74·7-90·5)	83·3 (75·1-90·2)	89·7 (75·1-100·0)	43·2 (41·5-44·9)	49·2 (47·4-51·0)	49·3 (47·5-51·1)	53·9 (51·5-56·3)	59·1 (55·9-62·1)	64·7 (59·5-70·5)	64·8 (59·4-71·0)	65·7 (55·2-77·0)	90·7 (82·5-100·0)	89·7 (81·6-99·4)	89·7 (81·5-99·4)	89·2 (80·5-99·4)
Japan	65·4 (57·8-72·8)	65·6 (55·6-75·4)	66·0 (56·2-75·8)	71·3 (50·1-91·7)	82·6 (79·6-85·4)	91·7 (89·8-93·4)	93·4 (91·5-95·2)	100·0 (99·3-100·0)	84·0 (82·6-85·2)	97·3 (95·8-98·7)	98·2 (96·6-99·7)	100·0 (100·0-100·0)	85·5 (82·3-88·6)	96·2 (92·9-99·5)	96·7 (93·4-100·0)	100·0 (100·0-100·0)
Singapore	71·4 (62·1-80·0)	79·5 (70·5-87·1)	79·8 (71·1-87·2)	86·0 (70·2-97·0)	75·0 (72·2-77·7)	92·9 (91·5-93·9)	93·5 (90·8-95·6)	98·8 (92·5-100·0)	76·5 (73·3-79·9)	94·2 (89·3-98·9)	95·1 (89·9-100·0)	99·9 (98·6-100·0)	62·5 (54·6-70·8)	74·7 (65·7-83·9)	74·5 (65·4-83·7)	73·2 (63·3-82·1)
South Korea	84·7 (78·1-90·8)	90·8 (84·3-96·3)	91·0 (84·5-96·7)	94·3 (83·1-100·0)	99·1 (96·1-100·0)	100·0 (100·0-100·0)	100·0 (100·0-100·0)	100·0 (100·0-100·0)	72·8 (69·3-75·9)	93·8 (87·1-100·0)	94·7 (87·5-100·0)	99·5 (93·6-100·0)	61·7 (57·9-65·6)	76·7 (69·5-84·6)	76·8 (69·3-84·9)	93·1 (80·7-100·0)
**Western Europe**																
Andorra	91·8 (85·7-97·5)	93·6 (88·4-98·5)	93·7 (88·3-98·5)	95·9 (85·7-100·0)	72·4 (69·3-75·4)	79·2 (76·2-82·2)	79·4 (76·3-82·6)	85·1 (80·2-90·4)	91·1 (85·3-97·1)	95·3 (89·2-100·0)	95·2 (89·0-100·0)	92·9 (84·6-100·0)	86·4 (79·0-93·5)	90·9 (83·3-98·7)	90·9 (83·3-98·8)	94·9 (86·3-100·0)
Austria	81·0 (73·0-87·9)	91·8 (85·6-96·8)	91·9 (85·7-96·9)	94·4 (83·4-100·0)	60·0 (57·7-62·2)	77·8 (76·2-79·1)	80·5 (78·9-82·1)	99·5 (96·8-100·0)	79·8 (78·2-81·5)	95·4 (93·2-97·8)	96·4 (94·1-98·9)	100·0 (100·0-100·0)	68·0 (65·6-70·8)	82·7 (79·8-85·8)	83·4 (80·5-86·5)	96·3 (92·0-100·0)
Belgium	89·1 (82·4-95·0)	91·2 (84·8-96·4)	91·3 (85·1-96·5)	93·8 (82·0-100·0)	66·2 (63·7-68·6)	74·0 (73·0-74·9)	74·9 (73·3-76·3)	86·9 (78·9-92·9)	78·8 (77·0-80·5)	92·0 (89·1-95·0)	92·8 (89·8-95·8)	99·6 (97·0-100·0)	65·9 (63·7-68·1)	78·6 (75·3-81·7)	79·2 (75·9-82·3)	89·3 (85·2-93·6)
Cyprus	85·9 (78·6-92·2)	90·2 (84·2-96·1)	90·3 (84·4-95·9)	93·8 (82·0-100·0)	71·2 (68·7-73·8)	85·6 (81·7-89·1)	86·0 (81·9-89·9)	97·6 (88·6-100·0)	70·8 (68·0-73·6)	87·8 (85·0-90·7)	88·8 (85·9-91·8)	98·4 (93·9-100·0)	62·9 (60·0-65·9)	74·8 (71·7-77·8)	74·9 (71·8-77·8)	88·5 (78·6-96·3)
Denmark	86·7 (79·3-93·0)	89·5 (83·1-95·2)	89·6 (83·2-95·2)	92·0 (79·9-100·0)	77·5 (74·7-80·2)	91·1 (89·0-92·9)	91·8 (89·9-93·6)	99·8 (98·0-100·0)	77·9 (75·7-80·2)	91·0 (87·6-94·5)	92·0 (88·4-95·6)	99·7 (96·9-100·0)	69·8 (66·7-72·9)	85·4 (81·3-89·2)	86·3 (82·1-90·1)	98·1 (93·8-100·0)
Finland	97·0 (91·9-100·0)	98·4 (94·1-100·0)	98·4 (94·1-100·0)	98·9 (92·0-100·0)	67·6 (65·0-70·0)	79·4 (77·8-80·9)	80·5 (78·9-82·0)	93·7 (89·1-98·2)	84·7 (82·9-86·3)	99·9 (98·5-100·0)	100·0 (99·9-100·0)	100·0 (100·0-100·0)	80·8 (77·5-84·1)	99·8 (97·9-100·0)	100·0 (99·6-100·0)	100·0 (100·0-100·0)
France	91·0 (85·0-96·1)	95·2 (89·9-99·6)	95·3 (90·0-99·6)	96·2 (85·3-100·0)	66·3 (63·9-68·6)	71·6 (70·1-72·9)	72·2 (70·7-73·6)	78·7 (74·9-81·5)	78·5 (76·6-80·7)	93·2 (91·0-95·4)	93·9 (91·7-96·1)	100·0 (99·7-100·0)	83·6 (81·6-85·5)	96·9 (94·2-99·3)	97·5 (94·9-100·0)	100·0 (100·0-100·0)
Germany	86·7 (78·9-92·9)	90·7 (84·5-96·1)	90·8 (84·9-96·3)	93·5 (82·2-100·0)	62·0 (59·8-64·3)	78·0 (75·3-80·4)	80·4 (77·6-82·8)	98·5 (94·3-100·0)	78·4 (76·4-80·4)	90·9 (88·4-93·5)	91·7 (89·0-94·3)	99·1 (96·1-100·0)	68·3 (66·0-70·8)	80·9 (78·2-83·8)	81·5 (78·8-84·4)	91·8 (88·2-96·1)
Greece	56·2 (46·2-65·4)	65·1 (55·7-74·3)	65·3 (56·0-74·3)	71·1 (53·5-87·4)	64·0 (61·6-66·3)	76·9 (75·0-78·6)	78·2 (75·2-80·7)	95·7 (84·1-100·0)	81·7 (80·0-83·3)	89·8 (86·9-92·7)	90·3 (87·3-93·2)	94·5 (90·6-98·5)	67·6 (65·2-70·4)	75·9 (73·0-79·0)	76·5 (73·5-79·5)	83·8 (80·7-87·2)
Iceland	90·9 (84·8-96·3)	93·8 (88·2-98·8)	93·9 (88·2-98·8)	96·4 (85·5-100·0)	51·1 (49·1-53·0)	84·3 (83·3-85·3)	87·8 (86·1-89·5)	100·0 (100·0-100·0)	90·3 (88·5-92·1)	100·0 (99·4-100·0)	100·0 (99·9-100·0)	100·0 (100·0-100·0)	83·0 (79·6-86·5)	97·8 (94·3-100·0)	98·4 (95·1-100·0)	100·0 (100·0-100·0)
Ireland	86·9 (79·8-92·8)	91·2 (85·0-96·6)	91·4 (85·6-97·0)	95·3 (84·2-100·0)	50·9 (48·9-52·8)	71·1 (69·0-72·8)	72·9 (69·3-75·9)	96·7 (84·4-100·0)	74·0 (71·7-76·0)	92·7 (89·1-96·2)	93·4 (89·7-97·1)	99·7 (96·9-100·0)	65·2 (62·3-68·0)	86·4 (82·7-89·8)	87·0 (83·2-90·3)	98·8 (93·7-100·0)
Israel	81·6 (73·9-88·8)	83·8 (75·9-90·4)	84·0 (76·1-90·5)	87·6 (73·1-98·1)	54·3 (52·2-56·3)	70·4 (67·9-72·6)	72·1 (69·5-74·4)	91·0 (87·5-94·1)	71·2 (68·0-74·2)	85·8 (80·4-90·8)	86·6 (80·8-91·7)	95·5 (86·1-100·0)	71·0 (67·6-74·3)	84·1 (79·1-89·2)	84·3 (79·2-89·5)	98·3 (91·8-100·0)
Italy	70·3 (61·7-78·7)	81·1 (73·1-88·2)	81·3 (73·2-88·5)	84·2 (68·5-95·9)	77·1 (74·3-79·8)	83·1 (81·7-84·4)	84·1 (82·0-85·8)	95·0 (88·3-99·9)	81·5 (79·9-83·1)	93·8 (91·4-96·4)	94·5 (91·9-97·2)	99·8 (98·1-100·0)	75·4 (73·1-78·1)	86·4 (83·4-89·4)	86·7 (83·7-89·7)	96·0 (90·2-100·0)
Luxembourg	92·8 (86·6-97·9)	95·0 (89·6-99·5)	95·0 (89·4-99·5)	96·8 (86·5-100·0)	65·7 (63·1-68·1)	83·1 (81·7-84·3)	84·6 (82·8-86·2)	99·9 (98·0-100·0)	81·2 (79·4-83·0)	97·0 (93·9-100·0)	97·5 (94·5-100·0)	100·0 (100·0-100·0)	67·7 (65·2-70·4)	82·4 (78·7-85·7)	83·0 (79·3-86·3)	97·8 (92·8-100·0)
Malta	70·1 (60·4-78·8)	75·8 (66·4-83·7)	76·1 (66·9-83·7)	81·3 (64·5-93·5)	52·9 (50·7-54·8)	52·6 (51·5-53·5)	53·7 (51·3-55·6)	57·2 (49·8-62·2)	70·8 (68·1-73·7)	86·8 (82·0-91·7)	87·9 (82·9-92·9)	98·3 (92·3-100·0)	61·9 (57·6-66·0)	79·9 (74·3-85·4)	81·2 (75·4-86·7)	97·8 (91·0-100·0)
Netherlands	82·4 (74·8-89·1)	91·6 (85·2-96·9)	91·7 (85·4-97·0)	93·8 (81·5-100·0)	78·1 (75·3-80·8)	93·6 (92·6-94·5)	94·8 (92·7-96·7)	100·0 (100·0-100·0)	81·2 (79·4-82·9)	96·4 (93·7-99·1)	97·0 (94·3-99·8)	100·0 (100·0-100·0)	65·4 (62·8-67·9)	80·9 (77·7-83·9)	81·5 (78·2-84·5)	89·8 (86·2-93·1)
Norway	86·3 (79·7-92·5)	89·7 (83·2-95·3)	89·7 (83·2-95·2)	92·7 (80·0-100·0)	66·7 (64·2-69·0)	81·4 (79·6-83·0)	81·5 (79·8-83·0)	93·2 (89·2-97·7)	83·7 (81·8-85·4)	98·4 (95·6-100·0)	99·1 (96·5-100·0)	100·0 (100·0-100·0)	76·6 (74·4-78·9)	95·8 (92·6-98·9)	97·1 (93·8-100·0)	100·0 (100·0-100·0)
Portugal	81·7 (73·8-88·9)	86·0 (78·2-92·7)	86·2 (78·6-93·0)	89·9 (75·7-99·9)	47·2 (45·3-49·0)	71·0 (69·7-72·4)	73·0 (71·5-74·5)	95·8 (90·4-100·0)	69·3 (67·3-71·2)	86·5 (84·0-88·8)	87·4 (84·9-89·7)	97·2 (94·0-100·0)	67·6 (65·1-70·3)	87·0 (84·0-89·9)	87·9 (84·9-90·8)	100·0 (100·0-100·0)
Spain	86·0 (79·5-92·1)	86·8 (78·9-93·2)	87·0 (79·4-93·3)	89·4 (73·9-100·0)	67·8 (65·3-70·2)	73·7 (72·2-74·9)	74·9 (71·3-77·9)	87·0 (68·0-100·0)	81·2 (79·6-82·8)	96·7 (94·4-99·1)	97·5 (95·2-100·0)	100·0 (100·0-100·0)	77·7 (75·2-80·0)	93·5 (90·9-96·1)	94·2 (91·6-96·8)	100·0 (100·0-100·0)

Location	Indicator 3.9.2: Age-standardised death rate attributable to unsafe water, sanitation, and hygiene (WaSH) (per 100,000 population)				Indicator 3.9.3: Age-standardised death rate due to unintentional poisonings (per 100,000 population)				Indicator 3.a.1: Age-standardised prevalence of daily smoking in populations aged 10 and older (%)				Indicator 3.b.1: Geometric mean of the coverage of eight vaccines, conditional on inclusion in national vaccine schedules, in target populations (%)			
	**2000**	**2015**	**2016**	**2030**	**2000**	**2015**	**2016**	**2030**	**2000**	**2015**	**2016**	**2030**	**2000**	**2015**	**2016**	**2030**
**High-income North America**																
Canada	89·2 (85·5-92·6)	82·4 (78·1-86·7)	82·8 (78·4-87·1)	83·8 (70·1-92·5)	69·5 (63·5-72·5)	74·4 (70·0-78·5)	74·8 (70·3-79·1)	78·3 (71·2-86·8)	44·5 (42·6-46·5)	64·6 (62·1-66·8)	64·8 (62·4-67·0)	73·8 (69·7-78·1)	32·4 (23·3-41·3)	54·2 (38·3-68·7)	60·2 (44·8-72·9)	91·7 (81·5-97·8)
United States	87·4 (84·7-89·7)	83·6 (81·1-86·0)	83·7 (81·2-86·2)	81·5 (77·0-86·8)	57·5 (48·2-60·4)	55·5 (50·6-58·7)	55·7 (50·7-58·8)	56·1 (51·2-61·0)	44·7 (43·5-45·8)	67·4 (66·5-68·3)	67·8 (66·9-68·6)	77·5 (76·4-78·6)	86·2 (83·7-88·2)	86·9 (82·3-90·6)	87·9 (82·9-91·6)	97·3 (91·0-100·0)
**Australasia**																
Australia	91·9 (89·9-94·0)	89·8 (86·4-92·6)	89·6 (86·2-92·5)	88·0 (83·8-91·8)	78·7 (72·4-81·7)	84·9 (81·4-88·7)	85·0 (81·3-88·8)	93·2 (85·4-100·0)	49·7 (47·3-52·1)	63·9 (60·3-67·3)	64·2 (60·2-67·8)	76·3 (71·0-81·2)	88·1 (84·5-91·1)	93·1 (89·5-95·3)	93·5 (89·7-95·9)	97·9 (92·3-100·0)
New Zealand	92·4 (90·4-94·5)	86·1 (82·0-89·9)	86·2 (82·1-90·0)	87·1 (82·4-91·5)	77·1 (72·2-80·4)	81·7 (77·7-87·8)	81·8 (77·3-88·1)	83·9 (72·5-97·4)	37·6 (34·8-40·4)	53·6 (48·3-58·4)	53·6 (48·2-58·6)	61·7 (51·8-70·4)	78·7 (73·3-83·2)	90·2 (85·4-93·8)	90·8 (85·5-94·3)	96·1 (88·5-99·4)
**High-income Asia Pacific**																
Brunei	76·9 (73·4-79·6)	77·3 (74·0-80·2)	77·3 (74·0-80·1)	76·9 (73·0-80·3)	57·9 (51·6-62·0)	62·3 (54·5-68·5)	62·6 (54·6-69·7)	66·6 (48·8-82·2)	59·8 (56·7-62·9)	67·1 (63·5-70·5)	67·5 (63·9-70·9)	73·8 (66·9-79·8)	94·8 (91·6-97·0)	96·5 (92·1-98·6)	96·7 (91·5-98·7)	98·0 (85·7-100·0)
Japan	79·6 (77·8-81·4)	82·3 (80·4-84·0)	82·5 (80·6-84·2)	85·3 (83·1-87·2)	68·3 (60·8-71·0)	73·2 (67·6-76·0)	73·6 (68·1-76·6)	81·1 (73·1-87·7)	21·9 (20·0-23·6)	31·7 (30·0-33·3)	31·5 (29·8-33·2)	40·9 (38·3-43·4)	93·2 (89·3-95·9)	95·9 (92·6-98·0)	95·7 (90·9-98·1)	96·4 (83·4-100·0)
Singapore	73·2 (70·2-76·4)	78·2 (74·8-81·6)	78·4 (74·9-81·9)	81·8 (77·5-86·0)	90·6 (82·4-95·2)	99·9 (98·0-100·0)	99·9 (98·5-100·0)	100·0 (100·0-100·0)	81·4 (79·0-83·7)	85·6 (82·9-87·8)	85·9 (83·3-88·1)	90·9 (86·6-94·6)	95·3 (93·4-96·8)	94·5 (92·0-95·9)	94·9 (92·2-96·4)	98·2 (93·5-100·0)
South Korea	77·0 (71·4-81·5)	80·0 (72·7-86·1)	80·1 (72·6-86·3)	81·0 (72·0-89·3)	39·2 (32·3-43·4)	59·2 (47·4-67·1)	59·6 (47·6-68·3)	73·1 (49·9-92·8)	20·5 (16·2-24·3)	45·4 (41·6-49·0)	46·2 (42·1-49·8)	61·0 (53·1-68·1)	82·1 (74·3-87·8)	98·0 (94·4-99·3)	98·1 (94·9-99·3)	99·7 (97·6-100·0)
**Western Europe**																
Andorra	92·1 (87·4-98·5)	93·5 (88·5-99·7)	93·5 (88·5-99·6)	95·3 (89·6-100·0)	83·0 (71·2-94·6)	89·4 (76·5-100·0)	89·5 (76·5-100·0)	91·4 (73·6-100·0)	24·4 (19·6-29·3)	35·9 (30·4-41·0)	36·2 (30·7-41·4)	42·3 (32·5-51·4)	86·3 (83·2-88·9)	94·8 (92·0-96·7)	94·9 (91·9-96·8)	97·3 (91·9-99·5)
Austria	100·0 (100·0-100·0)	100·0 (100·0-100·0)	100·0 (100·0-100·0)	100·0 (100·0-100·0)	76·1 (66·0-79·7)	82·0 (76·3-85·2)	82·5 (76·6-86·0)	91·2 (81·3-99·7)	17·1 (13·1-20·9)	24·1 (19·8-28·4)	24·6 (20·2-29·2)	30·1 (21·4-38·6)	71·2 (65·5-76·3)	87·0 (77·7-92·9)	88·3 (79·0-93·9)	97·4 (90·2-100·0)
Belgium	85·7 (82·8-88·3)	82·8 (78·5-86·4)	83·0 (78·7-86·7)	81·0 (72·3-87·8)	68·3 (55·9-72·4)	82·9 (65·3-87·8)	83·1 (65·9-88·1)	91·7 (73·9-100·0)	25·5 (21·6-29·2)	46·0 (42·9-49·0)	46·2 (42·8-49·4)	60·8 (55·1-66·4)	71·3 (66·6-75·1)	95·8 (94·5-96·7)	96·4 (95·1-97·3)	100·0 (99·7-100·0)
Cyprus	81·4 (75·0-86·7)	86·9 (80·9-92·2)	87·0 (80·9-92·4)	93·1 (85·0-100·0)	81·6 (76·4-87·6)	96·2 (91·7-100·0)	96·3 (91·7-100·0)	100·0 (100·0-100·0)	16·9 (11·5-22·1)	24·3 (19·3-28·8)	25·7 (20·8-30·4)	24·5 (14·7-34·0)	90·4 (87·0-92·8)	93·5 (90·7-95·6)	94·1 (91·0-96·3)	99·2 (96·7-100·0)
Denmark	89·2 (86·3-91·7)	83·2 (79·0-87·0)	83·5 (79·2-87·3)	85·5 (80·3-90·3)	72·1 (68·3-81·2)	89·8 (85·4-94·2)	89·7 (85·0-94·6)	99·9 (97·7-100·0)	8·4 (5·3-11·4)	50·8 (47·6-53·8)	50·8 (47·4-54·0)	73·6 (68·1-78·8)	92·2 (89·1-94·3)	90·4 (85·9-93·6)	91·3 (86·9-94·5)	95·8 (84·9-100·0)
Finland	88·7 (86·2-90·9)	100·0 (100·0-100·0)	100·0 (100·0-100·0)	100·0 (100·0-100·0)	65·2 (58·8-68·9)	84·8 (74·2-88·4)	85·2 (74·6-89·4)	97·8 (84·7-100·0)	37·4 (35·4-39·4)	53·6 (50·5-56·7)	53·4 (50·1-56·7)	63·5 (57·7-69·3)	96·1 (93·4-97·6)	95·4 (93·8-96·5)	96·1 (94·5-97·2)	99·1 (97·1-100·0)
France	85·3 (81·7-88·7)	86·8 (82·5-90·7)	87·0 (82·7-91·0)	90·2 (85·3-94·5)	69·4 (63·5-72·2)	82·6 (75·0-85·7)	82·9 (75·1-86·3)	88·4 (80·3-96·7)	11·7 (8·0-15·2)	27·3 (22·9-31·4)	28·2 (23·5-32·4)	39·1 (31·5-46·5)	59·0 (55·2-62·5)	89·2 (86·6-91·0)	90·2 (87·6-91·9)	97·8 (95·8-98·9)
Germany	97·0 (94·1-99·5)	86·9 (82·1-91·3)	87·0 (82·2-91·4)	89·0 (82·9-94·5)	93·9 (90·0-98·0)	100·0 (100·0-100·0)	100·0 (100·0-100·0)	100·0 (100·0-100·0)	27·2 (24·2-30·0)	37·1 (33·3-40·8)	37·9 (33·6-41·8)	44·7 (37·0-51·9)	69·6 (63·7-74·4)	78·3 (70·9-84·5)	74·1 (68·1-79·1)	95·9 (92·1-98·4)
Greece	99·9 (98·2-100·0)	100·0 (100·0-100·0)	100·0 (100·0-100·0)	100·0 (100·0-100·0)	67·5 (63·9-75·1)	81·3 (76·9-85·0)	81·1 (76·6-85·3)	95·1 (84·5-100·0)	0·0 (0·0-0·0)	2·0 (0·0-5·9)	4·1 (0·0-8·7)	8·3 (0·0-18·0)	90·5 (84·5-94·6)	76·2 (74·0-77·7)	80·8 (78·7-82·2)	98·6 (96·7-99·8)
Iceland	92·3 (90·0-94·6)	92·6 (90·2-94·8)	92·7 (90·3-95·0)	94·9 (92·1-97·5)	69·3 (64·8-73·1)	83·2 (76·2-86·7)	85·3 (76·6-89·1)	93·0 (81·9-100·0)	37·4 (34·0-41·1)	63·1 (60·4-65·7)	62·9 (60·0-65·7)	83·2 (79·9-86·1)	94·5 (91·0-96·8)	90·1 (85·9-93·7)	90·3 (85·8-93·9)	84·1 (63·0-95·1)
Ireland	85·7 (82·4-89·0)	93·3 (90·4-95·9)	93·6 (90·7-96·2)	97·8 (94·6-100·0)	59·8 (54·3-67·5)	77·1 (72·0-81·2)	77·4 (71·7-82·1)	87·3 (73·8-100·0)	26·3 (23·0-29·5)	42·8 (38·7-46·5)	43·7 (39·4-47·5)	55·3 (49·2-61·0)	72·0 (65·7-77·1)	89·6 (85·6-92·7)	90·1 (84·9-93·5)	95·5 (85·6-99·3)
Israel	82·4 (78·4-86·1)	80·4 (75·6-84·9)	80·5 (75·7-85·1)	82·9 (75·9-88·5)	79·8 (72·9-83·9)	88·2 (83·2-92·8)	88·6 (82·7-93·8)	94·0 (82·1-100·0)	42·6 (39·2-45·8)	52·3 (48·2-56·4)	52·7 (48·5-56·8)	63·8 (57·1-69·9)	92·4 (90·1-94·2)	92·0 (87·7-94·7)	92·7 (87·9-95·7)	95·1 (85·6-99·1)
Italy	100·0 (100·0-100·0)	99·8 (98·1-100·0)	99·9 (98·4-100·0)	100·0 (100·0-100·0)	70·2 (66·4-76·3)	95·9 (81·8-100·0)	95·9 (82·2-100·0)	99·3 (89·3-100·0)	37·6 (34·9-40·2)	42·1 (38·4-45·8)	43·2 (39·3-46·9)	51·1 (42·8-58·6)	76·9 (47·2-83·6)	92·5 (89·2-95·0)	92·7 (89·4-95·3)	95·2 (86·1-98·8)
Luxembourg	91·7 (89·0-94·2)	89·0 (84·6-92·7)	89·1 (84·7-92·8)	91·5 (85·0-96·0)	76·4 (68·3-80·0)	94·3 (81·6-98·6)	94·5 (81·7-99·1)	99·0 (88·5-100·0)	24·6 (20·3-29·0)	42·6 (37·7-47·0)	43·1 (38·1-47·5)	54·6 (46·0-62·2)	95·7 (92·8-97·4)	96·1 (94·8-97·0)	96·7 (95·4-97·6)	99·9 (99·4-100·0)
Malta	90·7 (87·4-94·1)	95·6 (92·4-98·4)	95·9 (92·7-98·8)	99·6 (97·1-100·0)	83·2 (76·6-86·7)	96·7 (85·0-100·0)	96·8 (84·7-100·0)	99·2 (89·5-100·0)	44·9 (41·2-48·5)	50·0 (46·0-54·0)	50·7 (46·5-54·9)	59·6 (51·0-67·3)	72·8 (62·4-80·9)	92·5 (86·6-95·8)	93·2 (87·1-96·5)	98·6 (93·2-100·0)
Netherlands	88·3 (85·9-90·4)	87·0 (84·1-89·6)	86·8 (83·9-89·5)	84·9 (80·8-88·3)	94·5 (85·7-98·2)	99·9 (98·4-100·0)	99·9 (98·3-100·0)	100·0 (100·0-100·0)	25·3 (22·8-27·7)	53·5 (50·4-56·4)	53·8 (50·5-57·0)	72·0 (67·6-76·1)	93·8 (91·6-95·5)	85·1 (82·0-87·4)	85·8 (82·3-88·3)	88·3 (76·0-95·3)
Norway	86·3 (83·4-88·9)	83·1 (78·6-87·2)	83·4 (78·9-87·6)	87·3 (80·0-92·5)	81·0 (73·1-84·6)	86·5 (82·6-91·6)	86·8 (82·3-92·3)	95·5 (85·9-100·0)	22·8 (18·8-27·0)	61·3 (57·9-64·6)	61·2 (57·5-64·4)	81·5 (76·3-86·2)	87·1 (83·3-90·1)	92·4 (89·0-94·9)	92·9 (89·3-95·5)	96·2 (90·8-99·1)
Portugal	86·2 (83·6-88·7)	85·7 (82·8-88·2)	85·9 (83·0-88·4)	87·9 (84·7-90·8)	61·4 (55·5-64·7)	78·8 (69·5-82·3)	79·2 (69·8-83·0)	89·9 (78·7-98·7)	44·5 (41·8-47·3)	46·5 (42·6-50·1)	47·2 (43·2-51·2)	49·8 (41·8-56·7)	88·3 (80·9-92·5)	92·0 (72·6-98·4)	92·4 (72·5-98·6)	89·4 (3·7-100·0)
Spain	94·7 (92·4-96·6)	96·4 (93·6-98·7)	96·6 (93·7-99·0)	98·9 (95·3-100·0)	76·1 (69·3-79·2)	93·5 (82·4-96·7)	93·7 (82·7-97·0)	99·7 (96·9-100·0)	14·7 (12·0-17·4)	23·2 (18·6-27·6)	24·9 (20·2-29·5)	35·8 (27·3-44·1)	91·2 (88·9-93·3)	97·0 (94·8-98·4)	97·1 (94·8-98·5)	98·8 (94·9-100·0)

Location	Indicator 5.2.1: Age-standardised prevalence of women aged 15 years and older who experienced physical or sexual violence by an intimate partner in the last 12 months (%)				Indicator 6.1.1: Risk-weighted prevalence of populations using unsafe or unimproved water sources, as measured by the summary exposure value (SEV) for unsafe water (%)				Indicator 6.2.1a: Risk-weighted prevalence of populations using unsafe or unimproved sanitation, as measured by the summary exposure value (SEV) for unsafe sanitation (%)				Indicator 6.2.1b: Risk-weighted prevalence of populations without access to a handwashing facility, as measured by the summary exposure value (SEV) for unsafe hygiene (%)			
	**2000**	**2015**	**2016**	**2030**	**2000**	**2015**	**2016**	**2030**	**2000**	**2015**	**2016**	**2030**	**2000**	**2015**	**2016**	**2030**
**High-income North America**																
Canada	93·8 (92·6-95·1)	96·7 (95·6-97·6)	96·9 (95·9-97·8)	99·6 (98·6-100·0)	99·4 (98·2-99·9)	99·7 (99·5-100·0)	99·7 (99·4-100·0)	99·8 (99·5-100·0)	95·8 (91·3-98·7)	97·1 (93·4-99·3)	97·1 (93·5-99·3)	97·9 (94·6-99·6)	99·5 (99·3-99·8)	99·8 (99·5-100·0)	99·8 (99·5-100·0)	99·9 (99·6-100·0)
United States	92·8 (91·8-93·6)	96·3 (95·6-97·1)	96·4 (95·6-97·1)	99·5 (98·6-100·0)	98·9 (98·6-99·2)	100·0 (100·0-100·0)	100·0 (100·0-100·0)	100·0 (100·0-100·0)	94·3 (93·2-95·3)	95·7 (94·7-96·5)	95·8 (94·8-96·6)	96·7 (95·8-97·3)	99·8 (99·6-100·0)	100·0 (100·0-100·0)	100·0 (100·0-100·0)	100·0 (100·0-100·0)
**Australasia**																
Australia	94·1 (92·7-95·4)	97·4 (96·2-98·5)	97·5 (96·5-98·6)	99·8 (98·9-100·0)	97·8 (96·8-98·6)	96·5 (94·1-98·3)	96·4 (94·1-98·3)	96·0 (93·6-98·3)	97·4 (94·2-99·4)	98·9 (97·2-100·0)	99·0 (97·3-100·0)	99·7 (98·9-100·0)	96·8 (96·5-97·2)	97·2 (96·9-97·6)	97·2 (96·9-97·6)	97·6 (97·2-98·0)
New Zealand	87·4 (85·7-89·0)	91·9 (90·5-93·0)	92·3 (90·9-93·4)	97·0 (95·6-98·1)	97·2 (96·2-98·0)	96·1 (93·7-98·0)	96·1 (93·6-98·0)	95·7 (92·4-97·9)	96·6 (92·4-99·0)	98·4 (95·7-99·9)	98·5 (95·9-99·9)	99·4 (98·0-100·0)	96·6 (96·2-97·0)	97·0 (96·6-97·4)	97·0 (96·6-97·4)	97·4 (97·0-97·9)
**High-income Asia Pacific**																
Brunei	80·5 (76·6-83·8)	87·4 (84·8-89·4)	87·9 (85·4-89·8)	94·2 (92·6-95·5)	96·3 (95·1-97·2)	96·2 (93·5-98·0)	96·1 (93·4-98·0)	95·5 (92·0-97·9)	95·4 (90·2-98·6)	98·5 (95·8-99·9)	98·5 (95·9-99·9)	99·7 (98·3-100·0)	96·3 (95·9-96·7)	97·0 (96·7-97·4)	97·1 (96·7-97·4)	97·6 (97·1-98·3)
Japan	96·2 (95·5-96·8)	100·0 (100·0-100·0)	100·0 (100·0-100·0)	100·0 (100·0-100·0)	97·8 (97·5-98·1)	96·3 (95·8-96·8)	96·3 (95·8-96·8)	95·6 (95·0-96·2)	97·6 (97·0-98·1)	98·8 (98·4-99·1)	98·8 (98·4-99·2)	99·4 (99·1-99·8)	96·9 (96·7-97·0)	97·2 (97·0-97·3)	97·2 (97·0-97·3)	97·4 (97·1-97·7)
Singapore	89·1 (87·1-91·0)	98·3 (97·3-99·2)	98·6 (97·6-99·5)	100·0 (100·0-100·0)	96·7 (95·6-97·6)	96·4 (94·2-98·1)	96·3 (94·1-98·1)	95·9 (93·8-97·9)	96·0 (91·4-98·7)	98·8 (96·6-100·0)	98·9 (96·8-100·0)	99·9 (99·1-100·0)	96·4 (96·0-96·8)	97·2 (96·8-97·5)	97·2 (96·8-97·5)	97·8 (97·4-98·4)
South Korea	84·2 (81·6-86·6)	93·6 (92·3-94·8)	94·1 (92·9-95·3)	100·0 (99·9-100·0)	96·8 (95·8-97·7)	96·3 (93·8-98·0)	96·3 (93·7-98·0)	95·8 (92·3-98·0)	96·0 (91·6-98·8)	98·7 (96·5-100·0)	98·8 (96·7-100·0)	99·8 (98·7-100·0)	96·4 (96·0-96·8)	97·1 (96·8-97·5)	97·2 (96·8-97·5)	97·6 (97·2-98·2)
**Western Europe**																
Andorra	98·1 (97·1-99·1)	100·0 (100·0-100·0)	100·0 (100·0-100·0)	100·0 (100·0-100·0)	100·0 (99·7-100·0)	99·9 (99·7-100·0)	99·9 (99·7-100·0)	99·9 (99·7-100·0)	99·1 (97·5-100·0)	99·7 (98·5-100·0)	99·7 (98·5-100·0)	99·9 (99·0-100·0)	99·7 (99·4-99·9)	99·8 (99·5-100·0)	99·8 (99·5-100·0)	99·9 (99·6-100·0)
Austria	96·3 (95·0-97·4)	99·9 (99·4-100·0)	100·0 (99·6-100·0)	100·0 (100·0-100·0)	98·9 (98·2-99·4)	99·6 (99·1-99·9)	99·6 (99·1-100·0)	99·9 (99·6-100·0)	96·0 (92·4-98·3)	97·5 (94·8-99·4)	97·6 (94·9-99·5)	98·5 (96·3-100·0)	99·4 (99·1-99·7)	99·6 (99·4-99·9)	99·7 (99·4-99·9)	99·8 (99·6-100·0)
Belgium	91·3 (89·9-92·6)	95·4 (94·4-96·3)	95·6 (94·6-96·4)	98·8 (97·8-99·5)	99·8 (99·5-100·0)	99·8 (99·5-100·0)	99·8 (99·6-100·0)	99·9 (99·6-100·0)	98·8 (96·9-99·9)	99·5 (98·1-100·0)	99·5 (98·2-100·0)	99·8 (98·8-100·0)	99·6 (99·3-99·8)	99·7 (99·5-100·0)	99·8 (99·5-100·0)	99·9 (99·6-100·0)
Cyprus	90·7 (88·9-92·1)	95·2 (93·7-96·4)	95·4 (93·9-96·6)	99·0 (97·1-100·0)	98·7 (98·2-99·2)	99·5 (99·2-99·8)	99·5 (99·3-99·8)	99·6 (99·4-99·8)	97·5 (94·2-99·4)	99·1 (97·2-100·0)	99·1 (97·2-100·0)	99·8 (98·6-100·0)	99·4 (99·1-99·6)	99·6 (99·4-99·9)	99·6 (99·4-99·9)	99·9 (99·6-100·0)
Denmark	94·4 (93·3-95·4)	98·3 (97·6-99·1)	98·5 (97·8-99·2)	100·0 (100·0-100·0)	100·0 (99·8-100·0)	99·9 (99·6-100·0)	99·9 (99·6-100·0)	99·9 (99·7-100·0)	99·2 (97·7-100·0)	99·7 (98·7-100·0)	99·7 (98·7-100·0)	99·9 (99·3-100·0)	99·7 (99·5-100·0)	99·8 (99·6-100·0)	99·9 (99·6-100·0)	99·9 (99·7-100·0)
Finland	90·9 (89·4-92·0)	96·5 (95·5-97·2)	96·8 (95·9-97·5)	100·0 (100·0-100·0)	99·9 (99·6-100·0)	99·9 (99·7-100·0)	99·9 (99·7-100·0)	100·0 (99·8-100·0)	98·7 (96·6-100·0)	99·5 (98·2-100·0)	99·6 (98·3-100·0)	99·9 (99·3-100·0)	99·6 (99·3-99·8)	99·8 (99·5-100·0)	99·8 (99·5-100·0)	99·9 (99·7-100·0)
France	92·4 (91·2-93·6)	97·1 (96·3-97·8)	97·3 (96·6-98·1)	100·0 (100·0-100·0)	99·7 (99·3-100·0)	99·8 (99·5-100·0)	99·8 (99·5-100·0)	99·8 (99·5-100·0)	98·0 (95·4-99·6)	99·0 (97·1-100·0)	99·1 (97·2-100·0)	99·6 (98·1-100·0)	99·5 (99·2-99·7)	99·6 (99·4-99·9)	99·7 (99·4-99·9)	99·8 (99·5-100·0)
Germany	95·7 (94·4-96·7)	100·0 (99·6-100·0)	100·0 (99·9-100·0)	100·0 (100·0-100·0)	99·6 (99·1-100·0)	99·9 (99·7-100·0)	100·0 (99·7-100·0)	100·0 (100·0-100·0)	98·5 (96·4-99·8)	99·3 (97·9-100·0)	99·4 (97·9-100·0)	99·7 (98·7-100·0)	100·0 (99·8-100·0)	100·0 (99·9-100·0)	100·0 (99·9-100·0)	100·0 (100·0-100·0)
Greece	91·1 (89·7-92·3)	96·1 (95·2-97·0)	96·3 (95·4-97·1)	99·8 (98·9-100·0)	99·7 (99·5-99·9)	99·7 (99·4-99·9)	99·7 (99·4-99·9)	99·6 (99·3-99·9)	97·7 (94·9-99·4)	99·0 (97·0-100·0)	99·0 (97·1-100·0)	99·7 (98·6-100·0)	99·5 (99·2-99·8)	99·7 (99·4-100·0)	99·7 (99·4-100·0)	99·9 (99·6-100·0)
Iceland	93·4 (91·8-94·8)	97·8 (96·6-98·7)	98·1 (96·9-99·0)	100·0 (100·0-100·0)	99·9 (99·6-100·0)	99·9 (99·7-100·0)	99·9 (99·7-100·0)	100·0 (99·8-100·0)	98·9 (97·0-100·0)	99·7 (98·6-100·0)	99·7 (98·7-100·0)	100·0 (99·6-100·0)	99·6 (99·4-99·9)	99·8 (99·6-100·0)	99·8 (99·6-100·0)	100·0 (99·8-100·0)
Ireland	91·8 (90·2-93·2)	97·6 (96·6-98·5)	98·0 (97·1-99·0)	100·0 (100·0-100·0)	99·5 (99·0-99·8)	99·9 (99·6-100·0)	99·9 (99·6-100·0)	99·9 (99·7-100·0)	97·9 (95·4-99·6)	99·3 (97·8-100·0)	99·3 (97·9-100·0)	99·8 (98·8-100·0)	99·4 (99·2-99·7)	99·7 (99·4-99·9)	99·7 (99·4-99·9)	99·8 (99·6-100·0)
Israel	82·2 (79·0-84·7)	86·9 (84·4-88·9)	87·1 (84·6-89·0)	89·5 (87·4-91·2)	98·3 (97·6-98·9)	99·0 (98·4-99·5)	99·0 (98·5-99·5)	99·4 (98·9-99·8)	95·9 (91·2-98·6)	97·5 (94·2-99·5)	97·6 (94·4-99·5)	98·6 (96·4-100·0)	99·1 (98·8-99·4)	99·3 (99·0-99·6)	99·3 (99·0-99·6)	99·5 (99·2-99·7)
Italy	92·5 (91·0-93·6)	97·4 (96·5-98·2)	97·5 (96·7-98·4)	100·0 (100·0-100·0)	96·6 (95·6-97·5)	97·7 (96·9-98·4)	97·8 (97·0-98·5)	98·3 (97·5-98·9)	98·3 (95·9-99·7)	99·0 (97·1-100·0)	99·0 (97·2-100·0)	99·5 (98·0-100·0)	99·1 (98·8-99·4)	99·4 (99·1-99·6)	99·4 (99·1-99·6)	99·6 (99·3-99·9)
Luxembourg	95·8 (94·5-96·9)	99·0 (98·1-99·7)	99·1 (98·2-99·8)	100·0 (100·0-100·0)	100·0 (99·8-100·0)	99·9 (99·7-100·0)	99·9 (99·7-100·0)	99·9 (99·7-100·0)	99·3 (97·6-100·0)	99·8 (98·9-100·0)	99·8 (99·0-100·0)	100·0 (99·5-100·0)	99·8 (99·5-100·0)	99·9 (99·6-100·0)	99·9 (99·6-100·0)	100·0 (99·7-100·0)
Malta	90·1 (88·4-91·5)	95·7 (94·6-96·6)	96·0 (94·9-96·9)	99·9 (99·4-100·0)	99·2 (98·6-99·7)	99·8 (99·5-100·0)	99·8 (99·5-100·0)	100·0 (99·7-100·0)	97·4 (94·1-99·4)	98·8 (96·7-100·0)	98·9 (96·8-100·0)	99·5 (98·3-100·0)	99·3 (99·1-99·6)	99·6 (99·3-99·8)	99·6 (99·3-99·8)	99·7 (99·5-100·0)
Netherlands	92·3 (90·7-93·6)	97·6 (96·8-98·3)	97·8 (97·1-98·6)	100·0 (100·0-100·0)	100·0 (99·7-100·0)	100·0 (99·8-100·0)	100·0 (99·8-100·0)	100·0 (99·8-100·0)	99·0 (97·1-100·0)	99·7 (98·6-100·0)	99·7 (98·7-100·0)	99·9 (99·3-100·0)	99·6 (99·4-99·9)	99·8 (99·6-100·0)	99·8 (99·6-100·0)	99·9 (99·7-100·0)
Norway	95·0 (93·6-96·2)	98·0 (97·0-98·9)	98·2 (97·1-99·1)	99·9 (99·4-100·0)	99·9 (99·7-100·0)	99·9 (99·7-100·0)	99·9 (99·7-100·0)	100·0 (99·8-100·0)	99·1 (97·6-100·0)	99·7 (98·8-100·0)	99·7 (98·9-100·0)	100·0 (99·6-100·0)	99·7 (99·4-99·9)	99·8 (99·6-100·0)	99·8 (99·6-100·0)	99·9 (99·7-100·0)
Portugal	86·8 (84·8-88·5)	94·4 (93·3-95·4)	94·8 (93·7-95·7)	99·8 (98·7-100·0)	97·2 (96·4-98·0)	98·8 (98·2-99·4)	98·8 (98·2-99·4)	99·5 (98·9-99·9)	90·2 (83·8-94·8)	94·7 (89·2-98·0)	94·8 (89·5-98·1)	97·1 (93·1-99·5)	98·8 (98·5-99·1)	99·3 (99·0-99·5)	99·3 (99·0-99·5)	99·6 (99·2-100·0)
Spain	94·4 (92·9-95·8)	99·6 (98·8-100·0)	99·8 (99·2-100·0)	100·0 (100·0-100·0)	99·7 (99·4-99·9)	99·7 (99·4-99·9)	99·7 (99·4-99·9)	99·7 (99·4-99·9)	98·0 (95·7-99·4)	99·6 (98·5-100·0)	99·7 (98·6-100·0)	100·0 (99·6-100·0)	99·4 (99·2-99·7)	99·7 (99·4-99·9)	99·7 (99·4-99·9)	99·8 (99·6-100·0)

Location	Indicator 7.1.2: Risk-weighted prevalence of household air pollution, as measured by the summary exposure value (SEV) for household air pollution (%)				Indicator 8.8.1: Age-standardised all-cause disability-adjusted life year (DALY) rates attributable to occupational risks (per 100,000 population)				Indicator 11.6.2: Population-weighted mean levels of fine particulate matter smaller than 2.5 microns in diameter (PM2.5)				Indicator 16.1.1: Age-standardised death rate due to interpersonal violence (per 100,000 population)			
	**2000**	**2015**	**2016**	**2030**	**2000**	**2015**	**2016**	**2030**	**2000**	**2015**	**2016**	**2030**	**2000**	**2015**	**2016**	**2030**
**High-income North America**																
Canada	99·9 (99·9-99·9)	100·0 (99·9-100·0)	100·0 (99·9-100·0)	100·0 (100·0-100·0)	52·7 (47·7-58·3)	61·0 (56·0-66·1)	61·2 (56·1-66·3)	63·9 (58·5-69·5)	88·4 (87·1-89·9)	89·5 (88·1-90·9)	89·5 (88·0-91·0)	90·3 (87·9-92·9)	75·3 (64·1-80·3)	75·1 (68·6-79·9)	75·5 (68·9-80·3)	79·2 (70·6-86·7)
United States	99·9 (99·9-99·9)	99·9 (99·9-99·9)	99·9 (99·9-99·9)	99·9 (99·9-100·0)	61·1 (55·7-67·0)	76·6 (71·3-81·7)	76·7 (71·4-81·8)	78·0 (72·5-83·1)	77·6 (76·5-78·8)	82·8 (81·7-84·0)	82·7 (81·6-83·9)	85·2 (84·0-86·6)	40·0 (36·3-46·6)	42·7 (37·4-48·5)	42·7 (37·3-48·5)	44·9 (37·1-51·7)
**Australasia**																
Australia	99·8 (99·6-99·9)	99·9 (99·8-99·9)	99·9 (99·8-99·9)	99·9 (99·9-100·0)	72·4 (66·5-77·8)	77·9 (70·7-84·5)	78·5 (71·3-85·3)	87·7 (79·1-95·7)	92·9 (91·3-94·5)	96·5 (94·9-98·2)	96·4 (94·9-98·0)	96·1 (93·5-99·0)	72·4 (66·4-77·1)	80·1 (74·3-84·3)	80·1 (74·2-84·5)	85·3 (78·3-91·6)
New Zealand	99·7 (99·6-99·8)	99·8 (99·7-99·9)	99·8 (99·7-99·9)	99·9 (99·8-100·0)	56·7 (48·7-63·7)	63·0 (54·9-70·8)	63·3 (55·2-71·1)	67·4 (58·2-76·1)	95·8 (93·8-97·7)	99·5 (97·8-100·0)	99·7 (98·0-100·0)	99·6 (97·9-100·0)	73·2 (66·7-77·3)	79·6 (73·4-84·1)	79·7 (73·3-84·6)	86·7 (78·4-95·1)
**High-income Asia Pacific**																
Brunei	99·8 (99·8-99·9)	100·0 (100·0-100·0)	100·0 (100·0-100·0)	100·0 (100·0-100·0)	15·7 (1·0-35·6)	29·8 (12·1-57·6)	30·2 (12·5-58·1)	35·1 (16·3-65·4)	98·2 (93·0-100·0)	95·2 (88·6-100·0)	94·9 (88·4-100·0)	96·6 (89·6-100·0)	76·0 (70·3-83·6)	80·2 (73·6-86·4)	80·3 (72·8-87·0)	83·0 (68·3-97·8)
Japan	100·0 (100·0-100·0)	100·0 (100·0-100·0)	100·0 (100·0-100·0)	100·0 (100·0-100·0)	65·4 (60·3-69·7)	69·1 (62·5-74·7)	68·8 (62·0-74·6)	65·6 (56·1-73·5)	72·5 (71·3-73·7)	70·6 (69·4-71·8)	70·6 (69·4-71·8)	71·3 (70·0-72·6)	89·2 (83·9-93·4)	98·9 (91·9-100·0)	99·0 (92·3-100·0)	99·9 (98·1-100·0)
Singapore	99·9 (99·9-99·9)	100·0 (100·0-100·0)	100·0 (100·0-100·0)	100·0 (100·0-100·0)	52·3 (40·5-66·3)	73·2 (60·6-90·8)	74·0 (61·2-92·1)	85·2 (70·5-100·0)	58·5 (47·1-70·7)	49·6 (37·6-61·4)	49·5 (37·7-61·5)	48·5 (36·7-61·1)	85·1 (80·5-93·1)	99·2 (91·7-100·0)	99·1 (91·5-100·0)	99·8 (98·1-100·0)
South Korea	100·0 (100·0-100·0)	100·0 (100·0-100·0)	100·0 (100·0-100·0)	100·0 (100·0-100·0)	34·4 (22·3-44·9)	77·4 (67·4-86·8)	79·1 (69·1-88·7)	99·1 (92·2-100·0)	47·9 (46·1-49·6)	44·3 (42·7-45·9)	44·3 (42·7-45·8)	43·8 (42·2-45·4)	67·6 (62·1-78·7)	77·0 (69·3-91·2)	77·1 (68·5-91·4)	88·2 (71·1-100·0)
**Western Europe**																
Andorra	99·3 (99·1-99·5)	99·7 (99·6-99·7)	99·7 (99·6-99·8)	99·8 (99·8-99·9)	63·4 (49·6-77·8)	63·4 (49·7-76·4)	63·5 (49·9-76·5)	64·0 (50·9-77·6)	76·8 (66·5-86·1)	77·3 (68·1-86·2)	77·4 (68·4-86·9)	78·5 (69·2-88·0)	93·3 (78·3-100·0)	95·2 (80·2-100·0)	95·2 (80·1-100·0)	94·9 (79·8-100·0)
Austria	99·0 (98·7-99·2)	99·5 (99·4-99·6)	99·5 (99·4-99·6)	99·8 (99·7-99·8)	75·9 (67·0-84·0)	79·3 (69·3-87·6)	79·5 (69·5-88·0)	82·9 (70·4-94·3)	65·6 (64·3-67·0)	66·1 (64·6-67·6)	66·3 (64·7-67·8)	67·9 (65·3-72·0)	85·0 (76·3-89·2)	93·3 (85·6-96·5)	93·7 (85·6-97·0)	99·0 (92·4-100·0)
Belgium	99·7 (99·6-99·8)	99·8 (99·8-99·9)	99·8 (99·8-99·9)	99·9 (99·9-99·9)	59·0 (50·2-67·0)	70·5 (61·2-79·3)	71·1 (61·7-80·0)	80·3 (69·1-91·1)	62·2 (60·9-63·5)	64·0 (62·5-65·3)	63·9 (62·5-65·3)	67·3 (65·5-69·9)	69·1 (65·2-76·2)	79·6 (75·2-85·1)	79·8 (75·1-85·5)	88·2 (80·6-96·5)
Cyprus	99·2 (99·0-99·4)	99·7 (99·6-99·8)	99·7 (99·6-99·8)	99·9 (99·8-99·9)	60·9 (52·5-69·9)	70·7 (58·5-80·3)	72·1 (60·6-81·4)	92·9 (82·8-100·0)	65·8 (62·5-69·0)	60·2 (57·0-63·3)	60·3 (57·1-63·5)	60·2 (56·3-65·1)	61·0 (55·2-66·6)	71·0 (65·3-75·8)	70·9 (65·3-75·7)	77·4 (69·9-83·9)
Denmark	99·8 (99·7-99·8)	99·9 (99·8-99·9)	99·9 (99·8-99·9)	99·9 (99·9-99·9)	50·4 (43·5-57·7)	54·1 (44·4-62·3)	54·4 (44·6-63·0)	59·3 (48·1-69·6)	76·8 (75·0-78·6)	78·7 (76·7-80·7)	78·8 (76·9-80·6)	84·4 (82·4-86·5)	77·0 (72·9-84·4)	90·9 (85·1-96·2)	91·1 (85·0-96·4)	99·1 (92·3-100·0)
Finland	99·6 (99·4-99·7)	99·8 (99·7-99·8)	99·8 (99·7-99·8)	99·9 (99·8-99·9)	82·6 (75·9-89·1)	91·1 (82·8-98·1)	91·4 (82·9-98·5)	96·1 (85·8-100·0)	91·5 (90·0-92·9)	95·6 (93·9-97·3)	95·9 (94·2-97·4)	98·7 (97·0-100·0)	62·8 (58·0-70·5)	76·3 (69·4-81·6)	76·7 (69·6-82·4)	90·6 (80·8-97·8)
France	99·5 (99·4-99·6)	99·8 (99·7-99·8)	99·8 (99·7-99·8)	99·9 (99·9-99·9)	65·6 (55·7-75·4)	62·2 (48·9-73·5)	63·3 (49·9-74·8)	78·6 (62·8-92·6)	72·1 (70·9-73·2)	74·0 (72·8-75·2)	74·1 (72·9-75·3)	76·7 (75·3-78·7)	78·7 (70·8-82·3)	88·0 (80·4-92·8)	88·4 (80·4-93·6)	94·6 (85·5-100·0)
Germany	99·2 (98·9-99·4)	99·5 (99·4-99·6)	99·5 (99·4-99·7)	99·8 (99·7-99·8)	66·0 (58·8-72·5)	70·9 (62·6-78·3)	71·0 (62·5-78·4)	72·0 (62·3-80·8)	68·3 (67·3-69·4)	69·8 (68·7-70·9)	69·8 (68·8-70·9)	74·6 (73·2-76·5)	82·0 (77·4-87·1)	92·4 (85·5-95·8)	92·6 (85·8-96·1)	98·8 (91·3-100·0)
Greece	99·2 (99·0-99·4)	99·7 (99·6-99·8)	99·7 (99·7-99·8)	99·9 (99·8-99·9)	67·8 (59·0-75·5)	81·3 (71·6-89·3)	82·3 (72·5-90·3)	95·1 (84·3-100·0)	73·7 (71·4-76·0)	75·5 (73·2-77·9)	75·8 (73·5-78·0)	81·4 (78·0-85·5)	84·6 (78·1-91·2)	85·8 (79·9-95·5)	86·3 (79·5-96·2)	89·3 (77·0-100·0)
Iceland	99·6 (99·5-99·7)	99·8 (99·7-99·8)	99·8 (99·7-99·9)	99·9 (99·9-99·9)	56·0 (47·2-63·2)	68·3 (59·1-76·8)	68·3 (59·0-76·9)	68·5 (56·9-78·6)	87·4 (83·9-90·7)	90·4 (86·8-94·0)	90·6 (86·9-93·8)	92·2 (88·5-95·5)	85·7 (80·3-91·4)	94·1 (87·7-98·3)	94·9 (87·7-98·7)	99·1 (91·1-100·0)
Ireland	99·3 (99·1-99·5)	99·7 (99·6-99·8)	99·7 (99·6-99·8)	99·8 (99·8-99·9)	68·1 (60·8-74·9)	86·0 (76·2-94·8)	86·1 (76·0-95·1)	87·9 (74·6-99·9)	80·4 (78·7-82·3)	82·8 (81·1-84·4)	82·8 (81·1-84·4)	87·0 (85·2-88·8)	84·9 (79·4-92·5)	92·8 (85·6-100·0)	93·1 (85·6-100·0)	96·8 (84·7-100·0)
Israel	99·4 (99·3-99·5)	99·6 (99·6-99·7)	99·7 (99·6-99·7)	99·8 (99·8-99·9)	91·3 (84·4-97·6)	97·3 (89·4-100·0)	97·2 (89·2-100·0)	96·4 (86·0-100·0)	62·0 (60·0-64·0)	58·6 (56·7-60·7)	58·8 (56·7-60·8)	59·6 (57·0-62·9)	57·8 (49·5-72·1)	67·5 (57·4-81·3)	67·6 (56·8-82·0)	77·2 (57·8-96·8)
Italy	99·2 (99·0-99·4)	99·6 (99·5-99·7)	99·6 (99·6-99·7)	99·8 (99·8-99·9)	66·2 (57·4-74·4)	76·2 (64·0-86·5)	76·2 (63·7-86·7)	76·5 (59·6-90·1)	65·2 (64·1-66·3)	64·8 (63·8-66·0)	65·1 (64·0-66·3)	72·9 (71·7-74·1)	81·9 (77·1-90·4)	91·6 (86·8-99·9)	91·7 (86·8-99·9)	98·5 (92·6-100·0)
Luxembourg	99·5 (99·4-99·6)	99·8 (99·7-99·8)	99·8 (99·7-99·8)	99·9 (99·9-99·9)	72·6 (64·2-81·1)	83·3 (71·9-92·7)	83·6 (72·1-93·1)	89·4 (75·9-100·0)	62·0 (58·7-65·3)	63·8 (60·4-67·1)	63·8 (60·4-67·0)	68·2 (64·4-72·0)	75·9 (71·3-80·8)	86·6 (81·8-91·4)	86·7 (81·7-91·5)	96·5 (89·2-100·0)
Malta	99·0 (98·7-99·2)	99·5 (99·4-99·6)	99·6 (99·4-99·7)	99·8 (99·8-99·9)	63·4 (54·4-71·0)	75·6 (64·9-85·5)	75·9 (65·1-86·0)	80·5 (67·0-92·8)	70·3 (63·2-77·3)	73·0 (65·9-79·7)	73·5 (66·3-80·2)	77·8 (70·3-84·7)	75·1 (69·1-80·6)	82·2 (75·7-87·0)	82·6 (75·4-88·0)	89·8 (77·3-100·0)
Netherlands	99·7 (99·6-99·8)	99·9 (99·8-99·9)	99·9 (99·8-99·9)	99·9 (99·9-100·0)	53·6 (46·3-61·0)	69·3 (61·8-76·5)	70·0 (62·4-77·2)	80·2 (71·2-88·6)	64·5 (63·1-65·7)	65·7 (64·4-67·1)	65·7 (64·4-67·0)	68·9 (67·3-71·4)	77·8 (73·5-86·4)	90·2 (86·0-96·2)	89·9 (85·3-96·4)	98·3 (92·0-100·0)
Norway	99·4 (99·2-99·5)	99·7 (99·6-99·8)	99·7 (99·6-99·8)	99·9 (99·8-99·9)	74·5 (68·3-80·6)	84·7 (77·1-92·1)	86·1 (78·5-93·7)	99·8 (97·2-100·0)	81·3 (79·9-82·8)	87·8 (86·3-89·3)	88·0 (86·4-89·4)	92·4 (90·4-94·4)	82·9 (76·3-87·8)	93·0 (87·1-97·7)	93·2 (86·9-97·8)	97·2 (88·3-100·0)
Portugal	96·8 (96·1-97·4)	99·1 (98·9-99·3)	99·1 (98·9-99·3)	99·7 (99·7-99·8)	59·7 (53·8-64·4)	76·7 (67·4-85·3)	77·5 (68·0-86·2)	88·1 (76·6-98·8)	80·8 (78·8-82·7)	81·6 (79·6-83·6)	81·7 (79·5-83·5)	82·5 (80·1-84·6)	66·2 (60·4-72·7)	78·8 (73·4-84·9)	79·2 (73·2-85·6)	85·9 (76·4-95·4)
Spain	98·6 (98·3-98·9)	99·4 (99·3-99·6)	99·5 (99·3-99·6)	99·7 (99·6-99·8)	76·3 (67·9-83·4)	97·5 (89·6-100·0)	97·9 (90·3-100·0)	99·9 (98·8-100·0)	79·6 (78·3-80·9)	80·0 (78·8-81·3)	80·0 (78·8-81·3)	81·3 (79·9-82·7)	86·8 (82·2-94·3)	96·7 (92·9-100·0)	96·8 (92·7-100·0)	100·0 (100·0-100·0)

Location	Indicator 16.1.2: Death rate due to conflict and terrorism (per 100,000 population)				Indicator 16.1.3: Age-standardised prevalence of physical or sexual violence experienced by populations in the last 12 months (%)			
	2000	2015	2016	2030	2000	2015	2016	2030
**High-income North America**								
Canada	100·0 (100·0-100·0)	100·0 (100·0-100·0)	100·0 (100·0-100·0)	100·0 (100·0-100·0)	92·8 (90·9-94·5)	93·5 (91·5-95·2)	93·6 (91·6-95·3)	95·1 (93·2-96·7)
United States	96·5 (94·5-97·4)	100·0 (100·0-100·0)	100·0 (100·0-100·0)	100·0 (100·0-100·0)	100·0 (100·0-100·0)	100·0 (100·0-100·0)	100·0 (100·0-100·0)	100·0 (100·0-100·0)
**Australasia**								
Australia	100·0 (100·0-100·0)	100·0 (100·0-100·0)	100·0 (100·0-100·0)	100·0 (100·0-100·0)	91·0 (89·5-92·3)	93·3 (92·0-94·5)	93·5 (92·2-94·6)	96·0 (94·8-97·1)
New Zealand	100·0 (100·0-100·0)	100·0 (100·0-100·0)	100·0 (100·0-100·0)	100·0 (100·0-100·0)	69·1 (66·2-71·8)	72·8 (70·2-75·2)	73·4 (70·8-75·7)	80·6 (78·5-82·6)
**High-income Asia Pacific**								
Brunei	100·0 (100·0-100·0)	100·0 (100·0-100·0)	100·0 (100·0-100·0)	100·0 (100·0-100·0)	87·8 (85·7-89·5)	91·3 (89·4-92·8)	91·4 (89·6-93·0)	93·5 (91·9-95·0)
Japan	100·0 (100·0-100·0)	100·0 (100·0-100·0)	100·0 (100·0-100·0)	100·0 (100·0-100·0)	92·2 (91·0-93·2)	94·7 (93·7-95·7)	94·9 (93·8-95·8)	96·8 (95·8-97·7)
Singapore	100·0 (100·0-100·0)	100·0 (100·0-100·0)	100·0 (100·0-100·0)	100·0 (100·0-100·0)	90·1 (88·3-91·8)	96·4 (95·0-97·8)	96·6 (95·2-98·0)	99·2 (98·0-100·0)
South Korea	100·0 (100·0-100·0)	100·0 (100·0-100·0)	100·0 (100·0-100·0)	100·0 (100·0-100·0)	86·3 (84·2-88·1)	91·1 (89·3-92·6)	91·4 (89·5-92·9)	95·3 (93·5-96·7)
**Western Europe**								
Andorra	100·0 (100·0-100·0)	100·0 (100·0-100·0)	100·0 (100·0-100·0)	100·0 (100·0-100·0)	94·2 (91·1-96·9)	95·0 (92·1-97·8)	95·1 (92·1-97·8)	95·9 (92·6-98·6)
Austria	100·0 (100·0-100·0)	100·0 (100·0-100·0)	100·0 (100·0-100·0)	100·0 (100·0-100·0)	97·5 (96·4-98·4)	99·8 (98·9-100·0)	99·8 (99·0-100·0)	100·0 (100·0-100·0)
Belgium	100·0 (100·0-100·0)	100·0 (100·0-100·0)	62·4 (55·9-70·0)	62·4 (55·9-70·0)	78·4 (76·0-80·5)	82·4 (80·3-84·3)	82·6 (80·5-84·4)	84·9 (82·9-86·7)
Cyprus	100·0 (100·0-100·0)	100·0 (100·0-100·0)	100·0 (100·0-100·0)	100·0 (100·0-100·0)	95·2 (93·9-96·2)	98·5 (97·5-99·3)	98·6 (97·6-99·4)	99·9 (99·4-100·0)
Denmark	100·0 (100·0-100·0)	100·0 (100·0-100·0)	100·0 (100·0-100·0)	100·0 (100·0-100·0)	79·7 (77·6-81·7)	83·1 (81·2-84·9)	83·4 (81·4-85·1)	87·0 (85·1-88·6)
Finland	100·0 (100·0-100·0)	100·0 (100·0-100·0)	100·0 (100·0-100·0)	100·0 (100·0-100·0)	78·1 (75·7-80·1)	83·4 (81·3-85·1)	83·8 (81·8-85·5)	89·1 (87·5-90·6)
France	100·0 (100·0-100·0)	59·0 (52·4-66·6)	100·0 (100·0-100·0)	100·0 (100·0-100·0)	78·5 (76·1-80·6)	81·9 (79·9-83·7)	82·1 (80·1-83·8)	84·7 (82·8-86·3)
Germany	100·0 (100·0-100·0)	100·0 (100·0-100·0)	100·0 (100·0-100·0)	100·0 (100·0-100·0)	86·9 (85·0-88·6)	90·5 (88·8-91·9)	90·7 (89·1-92·1)	93·8 (92·3-95·1)
Greece	100·0 (100·0-100·0)	100·0 (100·0-100·0)	100·0 (100·0-100·0)	100·0 (100·0-100·0)	89·3 (87·7-90·8)	91·3 (89·8-92·7)	91·4 (89·8-92·7)	91·7 (90·1-93·1)
Iceland	100·0 (100·0-100·0)	100·0 (100·0-100·0)	100·0 (100·0-100·0)	100·0 (100·0-100·0)	91·7 (88·5-94·6)	94·4 (91·4-97·0)	94·5 (91·6-97·2)	96·8 (94·2-99·1)
Ireland	100·0 (100·0-100·0)	100·0 (100·0-100·0)	100·0 (100·0-100·0)	100·0 (100·0-100·0)	89·6 (88·0-91·0)	93·0 (91·6-94·2)	93·2 (91·8-94·4)	95·9 (94·6-96·9)
Israel	43·2 (39·0-46·5)	44·3 (37·8-52·6)	72·0 (65·6-79·4)	78·5 (49·2-96·7)	79·6 (75·2-83·5)	83·3 (79·2-87·0)	83·5 (79·4-87·2)	86·0 (81·9-89·3)
Italy	100·0 (100·0-100·0)	100·0 (100·0-100·0)	100·0 (100·0-100·0)	100·0 (100·0-100·0)	86·2 (84·2-87·7)	89·8 (88·2-91·2)	89·9 (88·3-91·3)	91·2 (89·7-92·6)
Luxembourg	100·0 (100·0-100·0)	100·0 (100·0-100·0)	100·0 (100·0-100·0)	100·0 (100·0-100·0)	92·9 (91·7-94·2)	96·3 (95·3-97·4)	96·4 (95·4-97·5)	98·3 (97·4-99·3)
Malta	100·0 (100·0-100·0)	100·0 (100·0-100·0)	100·0 (100·0-100·0)	100·0 (100·0-100·0)	95·9 (94·8-96·8)	98·3 (97·3-99·2)	98·5 (97·5-99·3)	100·0 (99·6-100·0)
Netherlands	100·0 (100·0-100·0)	100·0 (100·0-100·0)	100·0 (100·0-100·0)	100·0 (100·0-100·0)	78·1 (75·5-80·2)	81·9 (79·7-83·7)	82·1 (79·9-83·9)	84·5 (82·4-86·5)
Norway	100·0 (100·0-100·0)	100·0 (100·0-100·0)	100·0 (100·0-100·0)	100·0 (100·0-100·0)	91·1 (87·8-93·9)	93·9 (91·0-96·6)	94·1 (91·2-96·8)	97·0 (94·4-99·3)
Portugal	100·0 (100·0-100·0)	100·0 (100·0-100·0)	100·0 (100·0-100·0)	100·0 (100·0-100·0)	87·3 (85·5-88·9)	91·8 (90·2-93·0)	91·9 (90·4-93·2)	94·2 (92·8-95·4)
Spain	100·0 (100·0-100·0)	100·0 (100·0-100·0)	100·0 (100·0-100·0)	100·0 (100·0-100·0)	98·6 (97·5-99·5)	100·0 (100·0-100·0)	100·0 (100·0-100·0)	100·0 (100·0-100·0)

Location	Indicator 16.2.3: Age-standardised prevalence of women and men aged 18-29 years who experienced sexual violence by age 18 (%)				Indicator 17.19.2c: Percentage of well-certified deaths by a vital registration (VR) system among a country's total population (%)			
	2000	2015	2016	2030	2000	2015	2016	2030
**High-income North America**								
Canada	46·6 (35·4-56·6)	41·8 (29·8-52·0)	41·3 (29·2-51·5)	33·4 (19·3-44·5)	95·1 (93·5-96·6)	96·3 (94·3-98·0)	96·4 (94·4-98·1)	97·6 (94·4-100·0)
United States	36·6 (25·1-48·1)	36·9 (25·4-48·4)	37·0 (25·5-48·6)	37·7 (26·2-49·0)	94·3 (93·6-95·0)	93·7 (93·0-94·4)	93·8 (93·0-94·5)	93·5 (92·3-94·6)
**Australasia**								
Australia	50·0 (39·0-60·5)	47·1 (34·5-59·8)	47·0 (33·8-59·9)	45·7 (29·7-60·7)	97·4 (94·6-99·9)	96·1 (91·1-100·0)	96·2 (89·6-100·0)	94·4 (80·2-100·0)
New Zealand	4·6 (0·0-18·0)	2·7 (0·0-14·6)	2·5 (0·0-14·3)	1·2 (0·0-11·3)	100·0 (99·7-100·0)	99·9 (98·2-100·0)	99·8 (97·7-100·0)	99·3 (93·0-100·0)
**High-income Asia Pacific**								
Brunei	42·5 (31·6-53·9)	42·1 (31·1-53·5)	42·1 (31·2-53·5)	42·1 (31·1-53·5)	87·1 (83·7-90·1)	85·8 (81·2-90·0)	86·1 (81·2-90·6)	90·6 (81·0-98·0)
Japan	38·7 (27·6-51·1)	38·7 (27·4-51·2)	38·7 (27·4-51·1)	38·7 (27·2-50·8)	90·9 (90·1-91·7)	87·8 (86·7-88·8)	88·1 (87·0-89·2)	85·9 (83·4-88·3)
Singapore	42·4 (31·6-54·0)	42·3 (31·5-53·5)	42·2 (31·4-53·5)	42·2 (31·1-53·6)	99·8 (98·7-100·0)	100·0 (100·0-100·0)	100·0 (100·0-100·0)	100·0 (100·0-100·0)
South Korea	42·1 (31·1-53·4)	41·9 (31·0-53·4)	41·9 (31·0-53·4)	41·8 (30·9-53·4)	84·4 (81·1-87·4)	87·3 (82·8-91·3)	87·7 (82·5-91·8)	92·1 (81·0-99·1)
**Western Europe**								
Andorra	44·5 (34·8-54·7)	44·4 (34·7-55·0)	44·4 (34·6-55·0)	44·3 (34·5-54·9)	0·0 (0·0-0·0)	0·0 (0·0-0·0)	0·0 (0·0-0·0)	0·0 (0·0-0·0)
Austria	44·6 (34·9-54·8)	44·4 (34·6-55·0)	44·4 (34·6-54·9)	44·2 (34·3-54·7)	94·1 (91·4-96·7)	95·2 (91·5-98·5)	95·4 (91·3-98·9)	95·1 (85·9-100·0)
Belgium	44·5 (34·8-54·8)	44·4 (34·7-55·0)	44·4 (34·8-55·0)	44·4 (34·8-54·8)	88·3 (85·2-91·4)	85·2 (79·8-90·1)	85·4 (79·4-90·8)	83·5 (71·7-93·7)
Cyprus	44·6 (34·8-54·8)	44·5 (34·9-54·9)	44·5 (34·9-54·9)	44·6 (34·8-54·7)	37·9 (31·6-44·8)	70·3 (63·6-77·2)	70·5 (62·9-78·4)	75·0 (58·4-86·9)
Denmark	51·3 (41·2-62·6)	51·3 (40·3-63·2)	50·9 (39·6-62·9)	50·7 (39·1-63·3)	90·2 (86·9-93·1)	89·6 (84·9-93·8)	89·7 (84·5-94·3)	90·7 (79·9-98·1)
Finland	50·6 (41·6-60·5)	50·3 (40·6-60·6)	50·3 (40·5-60·6)	50·2 (40·2-60·6)	100·0 (100·0-100·0)	100·0 (99·5-100·0)	100·0 (99·5-100·0)	99·9 (98·3-100·0)
France	74·4 (67·5-82·0)	73·9 (66·7-81·8)	73·9 (66·7-81·7)	74·0 (66·8-82·0)	83·8 (80·4-86·9)	83·4 (77·4-88·7)	83·7 (76·9-89·3)	83·6 (70·9-92·7)
Germany	33·7 (22·2-46·1)	33·7 (22·2-46·1)	33·8 (22·3-46·0)	33·9 (22·4-45·8)	88·9 (85·9-91·8)	89·7 (85·1-93·7)	89·9 (84·4-94·3)	91·6 (82·2-98·9)
Greece	44·2 (34·4-54·8)	44·3 (34·3-54·8)	44·3 (34·3-54·8)	44·4 (34·9-54·8)	76·0 (72·4-79·4)	77·1 (71·2-82·6)	77·3 (70·6-83·2)	79·3 (67·5-89·2)
Iceland	41·4 (29·6-52·7)	46·2 (34·7-57·7)	46·7 (35·2-58·2)	53·1 (41·5-64·4)	98·4 (96·3-100·0)	94·8 (91·7-97·5)	94·9 (91·6-97·8)	92·1 (82·7-98·4)
Ireland	38·8 (27·4-49·2)	34·3 (22·0-46·1)	33·2 (20·4-45·6)	33·4 (20·3-45·7)	96·3 (93·9-98·4)	97·9 (94·7-100·0)	98·1 (94·5-100·0)	98·6 (92·6-100·0)
Israel	24·4 (15·0-33·1)	24·2 (14·4-33·1)	24·0 (14·2-33·0)	23·9 (13·6-33·5)	87·1 (83·9-90·1)	83·5 (78·2-88·1)	83·8 (77·5-89·0)	80·7 (68·6-90·4)
Italy	44·6 (34·7-54·8)	44·4 (34·6-54·9)	44·3 (34·5-54·8)	44·1 (33·6-54·5)	92·9 (90·0-95·4)	93·9 (88·9-97·6)	94·0 (88·7-97·8)	94·7 (84·9-100·0)
Luxembourg	44·6 (34·8-54·8)	44·5 (34·8-54·7)	44·5 (34·8-54·7)	44·4 (34·5-54·6)	87·0 (83·6-90·2)	86·1 (80·9-90·8)	86·2 (80·2-91·5)	87·1 (74·3-97·1)
Malta	44·2 (34·2-54·7)	44·1 (34·0-54·8)	44·3 (34·4-54·9)	44·2 (34·4-54·9)	94·2 (91·5-96·9)	95·4 (91·8-98·4)	95·7 (91·6-98·9)	97·7 (90·7-100·0)
Netherlands	38·9 (25·7-49·8)	38·8 (25·4-49·6)	38·8 (25·3-49·6)	38·7 (25·0-49·6)	88·2 (85·1-91·1)	88·8 (84·6-92·3)	88·9 (83·7-93·0)	89·4 (79·2-96·9)
Norway	51·5 (39·2-65·0)	51·5 (39·1-64·9)	51·6 (39·0-64·9)	51·7 (38·6-65·5)	92·5 (89·7-95·1)	88·2 (83·1-92·4)	88·3 (82·4-93·2)	86·1 (73·1-95·9)
Portugal	44·4 (34·7-54·9)	44·5 (34·5-54·9)	44·4 (34·5-54·8)	44·4 (34·6-54·8)	79·3 (75·5-83·0)	84·6 (79·0-89·3)	84·8 (78·5-90·0)	87·5 (76·1-95·8)
Spain	50·2 (41·8-59·0)	49·3 (40·2-59·3)	48·9 (39·7-59·0)	49·2 (38·8-59·6)	88·4 (85·5-91·1)	90·8 (86·5-94·7)	90·9 (86·3-95·0)	93·2 (85·3-99·5)

Location	SDG Index: Geometric mean of all health-related SDG indicators (scale of 0 to 100)				MDG Index: Geometric mean of all health-related SDG indicators with corresponding MDG indicators (scale of 0 to 100)				Non-MDG Index: Geometric mean of all health-related indicators without corresponding MDG indicators (scale of 0 to 100)				Indicator 1.5.1: Death rate due to exposure to forces of nature (per 100,000 population)			
	**2000**	**2015**	**2016**	**2030**	**2000**	**2015**	**2016**	**2030**	**2000**	**2015**	**2016**	**2030**	**2000**	**2015**	**2016**	**2030**
Sweden	81·0 (79·2-82·5)	85·6 (82·0-87·6)	85·6 (81·8-87·8)	85·5 (75·5-88·9)	91·4 (90·3-92·5)	94·6 (93·7-95·6)	94·7 (93·8-95·7)	96·3 (94·9-97·5)	77·3 (75·1-79·3)	81·2 (76·7-84·1)	81·1 (76·4-84·1)	79·6 (66·2-84·7)	90·0 (85·4-94·4)	100·0 (100·0-100·0)	100·0 (100·0-100·0)	87·5 (82·6-92·2)
Switzerland	69·9 (68·1-71·5)	79·8 (77·0-81·8)	80·2 (77·3-82·4)	83·5 (79·3-86·4)	89·1 (88·0-90·1)	93·6 (92·7-94·6)	93·9 (92·9-94·9)	95·2 (93·1-96·6)	64·2 (62·0-66·3)	74·4 (71·1-77·0)	74·8 (71·1-77·5)	77·9 (72·1-82·2)	42·9 (36·4-53·0)	51·7 (45·1-61·5)	54·8 (48·0-64·8)	49·7 (43·1-59·5)
United Kingdom	68·5 (59·2-73·2)	79·9 (75·3-82·2)	80·1 (75·3-82·4)	83·4 (77·1-86·4)	82·8 (81·9-83·7)	88·6 (87·7-89·3)	88·9 (88·1-89·5)	93·7 (92·7-94·7)	64·6 (53·3-70·4)	76·5 (70·6-79·6)	76·6 (70·5-79·7)	78·0 (69·9-82·5)	74·3 (69·0-80·2)	88·1 (83·9-92·0)	89·4 (85·3-92·9)	87·3 (82·9-91·3)
**Southern Latin America**																
Argentina	57·3 (55·5-58·9)	64·0 (61·1-66·2)	64·4 (61·2-66·6)	67·2 (57·5-71·1)	60·7 (59·3-62·0)	68·9 (67·5-70·1)	69·2 (67·8-70·4)	73·9 (71·6-75·9)	59·2 (56·7-61·3)	64·8 (60·8-67·6)	65·1 (60·8-68·1)	66·4 (54·4-72·0)	66·2 (57·6-82·2)	73·4 (65·3-87·6)	74·9 (67·0-88·6)	77·4 (69·8-90·2)
Chile	57·3 (54·1-61·2)	63·4 (59·7-66·7)	63·8 (59·6-67·4)	63·9 (55·1-71·0)	71·2 (69·8-72·5)	77·3 (75·6-78·7)	77·9 (76·0-79·5)	82·5 (79·4-85·0)	53·6 (49·1-58·6)	56·1 (47·3-62·1)	56·2 (47·2-62·5)	50·9 (41·1-63·6)	56·5 (47·5-74·1)	33·0 (23·7-52·5)	35·4 (26·1-54·7)	38·9 (29·9-57·9)
Uruguay	59·9 (57·9-61·5)	67·1 (65·1-68·8)	67·4 (65·2-69·2)	68·0 (63·7-71·8)	67·0 (65·6-68·2)	73·1 (71·9-74·4)	73·5 (72·0-74·9)	78·9 (75·1-82·1)	59·8 (57·1-61·9)	65·0 (60·8-67·8)	65·1 (60·6-68·0)	58·8 (51·1-68·5)	59·3 (50·4-76·5)	100·0 (100·0-100·0)	100·0 (100·0-100·0)	79·0 (71·7-91·3)
**Eastern Europe**																
Belarus	47·2 (42·6-50·4)	52·3 (48·4-58·9)	52·9 (48·4-59·8)	58·4 (51·9-66·9)	66·1 (64·0-68·0)	78·5 (76·3-80·5)	79·2 (76·8-81·2)	86·8 (83·5-89·3)	43·8 (38·1-47·5)	46·0 (41·5-53·7)	46·5 (41·4-54·5)	49·9 (39·0-60·4)	100·0 (100·0-100·0)	31·7 (24·1-42·1)	34·7 (26·9-45·1)	35·1 (27·5-44·9)
Estonia	57·4 (55·6-59·5)	70·2 (63·2-75·7)	70·4 (63·3-76·1)	73·4 (64·7-82·1)	71·0 (69·0-72·7)	84·4 (81·4-86·3)	85·0 (82·1-87·1)	90·5 (86·6-93·0)	55·2 (52·9-57·9)	65·3 (56·6-72·3)	65·3 (56·7-72·4)	64·2 (48·3-77·2)	74·7 (66·1-89·0)	100·0 (100·0-100·0)	100·0 (100·0-100·0)	95·2 (91·8-98·8)
Latvia	51·9 (50·1-54·3)	63·0 (56·4-66·5)	63·3 (56·4-67·1)	68·0 (58·2-74·8)	69·3 (67·1-71·1)	78·9 (75·7-81·2)	79·5 (76·1-81·9)	86·7 (82·5-89·5)	48·7 (46·5-51·6)	58·5 (50·5-62·8)	58·7 (50·4-63·3)	61·3 (49·0-70·3)	73·0 (63·9-84·9)	100·0 (100·0-100·0)	100·0 (100·0-100·0)	94·5 (90·2-98·1)
Lithuania	55·1 (53·8-56·4)	57·1 (53·3-63·2)	57·6 (53·5-64·0)	62·3 (56·0-71·2)	70·8 (66·8-72·9)	79·6 (76·4-81·6)	80·7 (77·6-82·7)	87·3 (84·0-89·7)	52·4 (50·8-53·9)	51·6 (47·0-58·8)	51·8 (47·0-59·4)	54·7 (44·4-65·6)	51·9 (43·5-62·5)	100·0 (100·0-100·0)	100·0 (100·0-100·0)	80·4 (74·2-86·4)
Moldova	45·0 (39·9-49·0)	60·4 (52·2-63·9)	61·1 (52·7-64·8)	66·8 (56·8-72·9)	49·8 (46·9-52·8)	61·9 (57·3-66·0)	63·1 (58·1-67·2)	73·0 (66·4-78·6)	45·9 (39·5-51·1)	62·0 (51·2-66·7)	62·5 (51·5-67·3)	65·8 (53·3-73·1)	47·6 (40·8-56·3)	62·0 (54·2-70·5)	64·9 (57·0-73·4)	58·3 (51·8-65·5)
Russia	37·9 (36·0-39·9)	53·4 (47·8-58·4)	54·2 (48·0-60·1)	61·3 (49·9-70·7)	59·8 (57·2-62·1)	70·6 (66·4-73·6)	71·2 (66·8-74·3)	79·5 (72·3-83·7)	33·9 (31·7-36·2)	49·3 (42·7-55·1)	50·0 (42·8-57·3)	55·4 (41·8-66·5)	32·6 (23·7-47·8)	61·1 (52·0-72·8)	64·9 (55·6-77·6)	52·4 (44·7-62·3)
Ukraine	42·6 (39·1-47·0)	45·4 (42·2-48·0)	46·2 (42·2-49·5)	45·1 (36·1-54·9)	57·7 (55·5-59·7)	66·6 (62·9-69·5)	67·1 (63·3-70·3)	71·7 (56·8-79·3)	40·7 (36·4-46·4)	41·1 (37·6-43·9)	41·9 (37·6-45·6)	38·9 (29·0-50·5)	100·0 (100·0-100·0)	37·1 (29·6-46·9)	40·7 (32·8-50·4)	39·0 (31·3-48·4)
**Central Europe**																
Albania	57·4 (55·7-59·0)	67·4 (64·9-69·6)	67·9 (65·2-70·2)	71·5 (66·2-76·1)	53·5 (50·8-55·8)	63·9 (60·4-67·0)	64·6 (61·1-67·8)	73·1 (66·4-79·2)	59·9 (56·1-62·7)	68·6 (62·5-72·2)	68·8 (61·6-72·7)	68·0 (56·6-76·5)	36·4 (29·5-44·6)	100·0 (100·0-100·0)	100·0 (100·0-100·0)	63·6 (57·2-69·5)
Bosnia and Herzegovina	58·2 (55·4-60·4)	56·1 (53·8-60·0)	57·0 (54·2-61·2)	58·7 (55·3-62·4)	65·8 (63·3-67·8)	72·5 (69·8-74·8)	73·1 (70·4-75·5)	78·0 (73·0-82·3)	57·8 (54·1-60·5)	52·0 (48·9-57·0)	52·9 (49·2-58·3)	52·5 (42·7-58·0)	100·0 (100·0-100·0)	52·8 (42·6-69·8)	54·4 (44·2-71·2)	60·5 (50·4-76·8)
Bulgaria	49·5 (48·5-50·4)	55·9 (53·2-60·1)	57·0 (53·5-61·8)	61·3 (52·4-69·0)	66·4 (64·8-67·7)	73·9 (71·9-75·5)	74·5 (72·2-76·4)	79·9 (75·3-83·8)	46·1 (44·3-47·8)	51·1 (47·2-56·3)	52·2 (47·3-58·1)	54·0 (41·5-64·8)	39·3 (32·4-47·2)	62·7 (53·5-74·2)	66·2 (56·6-78·2)	54·4 (47·1-61·6)
Croatia	61·1 (58·6-63·1)	67·4 (63·0-70·5)	68·1 (63·9-71·3)	70·4 (63·1-77·9)	75·3 (73·4-76·8)	82·6 (80·7-84·4)	83·0 (81·1-84·9)	88·9 (85·3-91·8)	58·6 (55·5-61·0)	62·8 (57·8-66·9)	63·5 (57·9-67·5)	62·7 (49·5-73·1)	81·7 (73·2-92·5)	100·0 (100·0-100·0)	100·0 (100·0-100·0)	98·0 (96·1-99·5)
Czech Republic	63·5 (60·6-65·7)	71·8 (66·8-74·8)	72·5 (68·1-75·4)	79·0 (75·0-82·0)	82·6 (81·3-83·8)	88·5 (86·9-89·9)	89·0 (87·5-90·4)	93·0 (90·2-95·0)	59·0 (55·6-61·9)	66·6 (60·3-70·4)	67·3 (61·5-71·0)	73·0 (65·7-77·5)	68·4 (58·5-83·6)	83·4 (74·2-94·0)	86·0 (77·4-95·3)	82·5 (74·0-91·6)
Hungary	54·1 (48·7-57·5)	66·8 (62·8-69·5)	67·2 (63·4-70·0)	74·3 (70·3-77·8)	75·1 (73·3-76·7)	81·6 (79·4-83·1)	82·1 (79·8-83·7)	87·2 (84·3-89·8)	49·7 (43·2-53·8)	62·8 (58·1-66·4)	63·1 (58·2-66·8)	69·6 (60·3-74·6)	59·3 (48·8-75·5)	100·0 (100·0-100·0)	100·0 (100·0-100·0)	86·1 (78·0-95·1)
Macedonia	57·9 (55·4-59·8)	58·6 (55·3-62·9)	60·0 (56·0-64·1)	60·0 (56·1-65·4)	62·7 (59·3-65·5)	68·5 (65·4-71·2)	69·4 (66·2-72·3)	74·6 (67·3-80·0)	59·2 (56·2-61·5)	57·3 (53·0-62·8)	58·6 (53·3-64·0)	55·2 (44·6-63·4)	38·1 (32·8-43·7)	58·8 (52·0-67·2)	63·9 (56·5-73·0)	55·4 (49·3-61·6)
Montenegro	60·8 (57·3-63·3)	56·8 (53·2-58·9)	57·0 (53·4-59·2)	57·5 (50·3-61·7)	68·6 (66·0-70·8)	78·5 (76·4-80·4)	78·8 (76·7-80·8)	84·7 (80·8-87·8)	59·9 (55·5-63·1)	50·6 (46·2-53·1)	50·7 (46·1-53·3)	47·5 (38·3-54·4)	100·0 (100·0-100·0)	76·9 (68·5-90·4)	82·1 (74·5-93·5)	75·9 (67·4-89·8)
Poland	55·9 (53·1-60·0)	70·5 (68·2-72·3)	71·0 (68·5-73·1)	77·4 (72·8-80·8)	72·7 (70·3-74·6)	81·6 (79·7-83·2)	82·2 (80·0-84·0)	89·0 (86·2-91·4)	53·0 (49·4-58·0)	67·9 (65·1-70·3)	68·2 (65·3-70·8)	73·1 (65·7-77·9)	65·6 (58·0-74·5)	100·0 (100·0-100·0)	100·0 (100·0-100·0)	92·1 (87·6-95·9)
Romania	49·3 (45·2-52·8)	57·9 (53·4-63·0)	58·6 (53·9-63·9)	64·3 (57·1-70·7)	57·9 (55·4-60·1)	69·2 (64·9-71·9)	69·9 (65·7-72·7)	78·7 (73·0-82·4)	49·0 (43·9-53·7)	55·7 (50·0-62·1)	56·2 (50·4-62·8)	59·0 (48·9-67·4)	40·1 (33·6-47·3)	53·5 (45·0-63·9)	57·2 (48·7-67·4)	48·3 (41·0-56·3)
Serbia	53·5 (49·7-56·2)	59·4 (54·2-62·7)	59·7 (54·6-62·9)	58·9 (49·9-66·3)	66·0 (63·3-68·4)	71·2 (68·9-73·2)	71·5 (69·1-73·6)	73·6 (67·7-77·9)	51·8 (47·0-55·3)	57·9 (51·4-62·0)	58·1 (51·5-62·3)	56·5 (45·6-65·8)	72·3 (63·0-86·5)	54·2 (43·0-71·9)	56·0 (44·8-73·5)	57·8 (48·5-70·0)
Slovakia	62·7 (60·2-64·7)	71·6 (67·7-74·2)	72·1 (68·1-74·7)	77·8 (71·6-81·7)	76·6 (74·9-78·0)	82·1 (80·7-83·5)	82·7 (81·0-84·1)	88·6 (85·0-91·5)	60·3 (57·1-62·9)	69·2 (64·5-72·5)	69·5 (64·4-72·9)	73·6 (63·8-78·9)	46·7 (37·0-60·7)	65·4 (57·7-74·9)	67·8 (59·8-77·7)	63·8 (56·8-70·4)
Slovenia	64·6 (62·0-66·8)	74·7 (71·9-77·0)	75·3 (72·3-77·7)	79·5 (74·3-83·2)	81·8 (79·7-83·3)	90·1 (88·2-91·6)	90·7 (88·8-92·2)	93·6 (91·1-95·6)	60·2 (56·8-63·0)	68·6 (64·7-71·8)	69·0 (64·9-72·3)	71·1 (58·1-78·1)	58·6 (49·0-76·5)	56·0 (49·3-63·5)	59·4 (52·6-67·2)	57·8 (51·3-64·8)
**Central Asia**																
Armenia	59·6 (57·8-61·2)	63·4 (61·4-65·3)	65·0 (62·8-66·9)	68·9 (63·8-72·8)	55·7 (51·8-58·3)	68·2 (64·0-71·0)	69·1 (64·6-71·9)	78·1 (70·6-82·8)	63·8 (61·9-65·6)	61·8 (59·2-64·2)	63·3 (60·7-66·0)	62·8 (51·6-69·0)	100·0 (100·0-100·0)	40·2 (33·7-48·5)	43·6 (37·1-52·2)	43·9 (37·5-52·5)

Location	Indicator 2.2.1: Prevalence of stunting in children under 5 (%)				Indicator 2.2.2a: Prevalence of wasting in children under 5 (%)				Indicator 2.2.2b: Prevalence of overweight in children aged 2-4 (%)				Indicator 3.1.1: Maternal mortality ratio (maternal deaths per 100,000 livebirths) in women aged 10-54 years			
	**2000**	**2015**	**2016**	**2030**	**2000**	**2015**	**2016**	**2030**	**2000**	**2015**	**2016**	**2030**	**2000**	**2015**	**2016**	**2030**
Sweden	99·6<br>(99·0-100·0)	99·7<br>(99·0-100·0)	99·7<br>(99·0-100·0)	99·8<br>(99·0-100·0)	97·3<br>(94·5-99·3)	98·5<br>(95·6-100·0)	98·5<br>(95·7-100·0)	99·1<br>(96·8-100·0)	61·9<br>(48·2-73·0)	53·5<br>(39·5-65·9)	52·8<br>(38·7-65·2)	42·6<br>(13·8-66·9)	88·3<br>(85·7-90·7)	99·4<br>(97·1-100·0)	97·7<br>(93·9-100·0)	99·9<br>(99·4-100·0)
Switzerland	99·7<br>(98·9-100·0)	99·8<br>(98·9-100·0)	99·8<br>(98·9-100·0)	99·8<br>(98·8-100·0)	97·5<br>(94·3-99·6)	98·5<br>(95·2-100·0)	98·5<br>(95·2-100·0)	98·9<br>(95·7-100·0)	70·5<br>(58·5-79·8)	61·4<br>(48·0-73·3)	61·0<br>(48·0-73·2)	53·0<br>(24·1-74·8)	83·4<br>(79·8-86·6)	91·4<br>(86·3-96·1)	91·6<br>(83·7-98·5)	94·2<br>(70·5-100·0)
United Kingdom	96·0<br>(95·8-96·3)	96·5<br>(96·3-96·7)	96·6<br>(96·4-96·8)	97·1<br>(96·9-97·3)	98·3<br>(97·6-99·0)	98·8<br>(98·2-99·5)	98·8<br>(98·2-99·5)	99·0<br>(98·4-99·7)	59·0<br>(51·4-66·2)	53·5<br>(41·6-63·8)	52·7<br>(40·3-63·5)	43·6<br>(19·3-63·9)	79·0<br>(76·8-80·8)	86·7<br>(84·6-88·5)	86·2<br>(84·2-88·0)	90·4<br>(87·1-93·8)
**Southern Latin America**																
Argentina	83·6<br>(78·2-88·1)	89·2<br>(85·0-92·7)	89·4<br>(85·2-92·8)	91·7<br>(88·2-94·8)	82·2<br>(75·5-87·7)	90·3<br>(82·7-95·9)	90·5<br>(82·9-96·1)	93·8<br>(86·1-99·4)	83·2<br>(71·4-91·7)	72·7<br>(56·9-85·0)	72·2<br>(55·7-84·6)	60·5<br>(23·7-85·7)	47·1<br>(44·9-49·4)	47·9<br>(45·0-50·7)	48·3<br>(45·1-51·4)	52·3<br>(43·6-60·7)
Chile	97·1<br>(96·1-98·1)	97·7<br>(96·3-99·0)	97·9<br>(96·6-99·1)	99·9<br>(99·3-100·0)	99·6<br>(96·8-100·0)	99·7<br>(97·2-100·0)	99·7<br>(97·3-100·0)	99·8<br>(98·1-100·0)	40·7<br>(18·1-59·1)	15·0<br>(0·0-35·2)	13·9<br>(0·0-35·1)	2·6<br>(0·0-23·9)	63·2<br>(60·2-66·3)	65·4<br>(59·6-71·0)	66·3<br>(58·2-74·2)	69·9<br>(50·5-85·9)
Uruguay	80·9<br>(75·6-85·8)	84·8<br>(79·3-89·3)	85·0<br>(79·7-89·5)	88·4<br>(83·4-92·3)	88·7<br>(83·9-92·7)	93·6<br>(87·9-97·8)	93·8<br>(88·1-98·1)	96·8<br>(90·9-100·0)	60·8<br>(42·3-74·6)	33·7<br>(11·7-52·3)	32·6<br>(10·5-51·2)	10·0<br>(0·0-41·7)	59·4<br>(56·6-62·4)	62·3<br>(59·1-65·6)	63·0<br>(59·4-66·4)	67·4<br>(55·8-79·1)
**Eastern Europe**																
Belarus	90·4<br>(83·7-95·1)	93·3<br>(87·9-96·9)	93·5<br>(88·0-97·0)	94·9<br>(89·3-97·9)	87·5<br>(83·4-91·2)	89·8<br>(85·9-93·2)	89·9<br>(86·0-93·3)	91·0<br>(87·3-94·6)	75·5<br>(56·9-88·5)	63·5<br>(41·9-78·8)	62·6<br>(41·2-78·6)	47·8<br>(0·0-82·6)	52·2<br>(49·5-54·8)	90·0<br>(85·9-94·0)	90·2<br>(84·2-96·1)	99·4<br>(92·8-100·0)
Estonia	82·6<br>(71·4-90·7)	90·9<br>(83·7-95·6)	91·1<br>(84·0-95·8)	94·3<br>(89·2-97·8)	86·8<br>(82·0-91·0)	90·9<br>(86·9-94·7)	91·0<br>(87·0-94·8)	93·0<br>(89·0-96·6)	75·6<br>(60·6-87·8)	52·3<br>(29·9-71·5)	51·6<br>(29·4-70·9)	28·9<br>(0·0-68·9)	60·7<br>(58·1-63·7)	91·0<br>(87·5-94·8)	91·6<br>(87·3-96·3)	96·3<br>(81·0-100·0)
Latvia	80·1<br>(68·1-89·3)	89·9<br>(81·9-95·2)	90·0<br>(82·1-95·3)	94·7<br>(89·2-97·9)	85·3<br>(80·0-89·8)	90·1<br>(85·8-93·6)	90·2<br>(86·0-93·7)	91·9<br>(88·3-95·2)	79·8<br>(64·7-90·5)	63·3<br>(42·9-79·4)	62·6<br>(42·0-78·8)	51·9<br>(9·6-81·9)	60·8<br>(57·9-63·6)	77·9<br>(73·6-81·8)	78·6<br>(73·4-83·4)	92·9<br>(78·3-100·0)
Lithuania	81·1<br>(70·0-89·8)	90·5<br>(83·3-95·6)	90·7<br>(83·6-95·7)	95·1<br>(90·8-98·0)	85·8<br>(80·8-90·3)	90·4<br>(86·2-94·0)	90·5<br>(86·4-94·1)	92·5<br>(89·2-95·9)	84·8<br>(73·7-93·1)	66·5<br>(45·3-81·7)	65·6<br>(44·2-80·7)	52·7<br>(1·3-83·4)	72·2<br>(69·4-75·1)	75·7<br>(72·5-78·9)	78·4<br>(74·7-81·9)	75·7<br>(63·5-86·4)
Moldova	76·3<br>(64·0-86·4)	87·3<br>(80·0-93·0)	87·6<br>(80·1-93·3)	91·3<br>(82·4-96·8)	69·9<br>(61·9-77·0)	87·2<br>(82·9-91·1)	87·6<br>(83·3-91·5)	92·7<br>(88·6-96·4)	92·7<br>(82·5-99·6)	87·9<br>(77·1-95·8)	87·4<br>(74·8-95·6)	81·3<br>(51·2-98·7)	56·1<br>(52·9-59·5)	63·8<br>(59·7-67·6)	66·2<br>(61·7-70·5)	73·9<br>(61·6-85·5)
Russia	75·7<br>(67·1-82·6)	82·7<br>(71·5-90·5)	82·9<br>(71·8-90·7)	86·6<br>(75·4-94·5)	78·6<br>(73·9-82·9)	90·8<br>(86·1-94·4)	91·2<br>(86·4-94·7)	95·2<br>(90·7-98·2)	61·1<br>(42·8-76·3)	56·8<br>(34·9-75·4)	56·3<br>(33·1-75·0)	49·4<br>(0·5-81·8)	49·4<br>(45·9-52·8)	69·7<br>(62·2-77·2)	69·4<br>(58·0-80·3)	87·7<br>(67·0-100·0)
Ukraine	59·6<br>(52·6-65·9)	70·7<br>(56·7-81·9)	70·9<br>(56·9-82·2)	74·0<br>(58·5-85·3)	67·9<br>(60·6-74·6)	74·8<br>(66·4-81·8)	75·1<br>(66·8-82·0)	78·5<br>(70·5-85·3)	86·3<br>(73·8-94·9)	73·8<br>(55·4-86·5)	73·8<br>(55·6-86·7)	67·0<br>(28·6-91·1)	46·9<br>(43·9-50·0)	64·7<br>(58·9-70·6)	64·5<br>(55·9-74·2)	82·7<br>(62·0-100·0)
**Central Europe**																
Albania	34·5<br>(28·0-40·4)	60·4<br>(50·8-68·9)	61·1<br>(51·2-69·9)	70·6<br>(57·0-82·6)	50·0<br>(41·7-57·1)	56·2<br>(39·7-68·6)	56·5<br>(38·8-69·6)	58·1<br>(24·1-79·1)	29·5<br>(9·8-48·0)	27·2<br>(6·4-47·0)	26·3<br>(4·9-46·5)	21·0<br>(0·0-55·9)	70·1<br>(65·7-74·2)	80·8<br>(75·0-86·9)	81·4<br>(75·4-87·7)	92·7<br>(79·1-100·0)
Bosnia and Herzegovina	80·0<br>(76·0-84·0)	75·4<br>(68·0-82·4)	75·9<br>(68·3-83·0)	74·1<br>(65·5-83·5)	67·2<br>(58·7-74·0)	63·8<br>(50·9-73·6)	63·5<br>(49·8-73·7)	54·3<br>(37·7-69·7)	57·5<br>(40·6-71·2)	42·3<br>(22·4-61·3)	41·6<br>(20·7-60·1)	33·0<br>(0·0-66·1)	65·0<br>(58·1-71·9)	86·3<br>(79·8-93·1)	86·0<br>(79·5-92·7)	99·1<br>(90·1-100·0)
Bulgaria	79·4<br>(71·7-85·5)	84·4<br>(78·1-89·2)	84·6<br>(78·4-89·4)	87·3<br>(82·2-91·4)	80·3<br>(68·9-88·6)	83·2<br>(71·2-91·3)	83·3<br>(71·1-91·4)	84·8<br>(72·9-93·0)	49·5<br>(28·2-65·9)	38·3<br>(16·5-56·3)	37·2<br>(14·9-55·2)	26·6<br>(0·0-60·4)	52·3<br>(49·7-54·8)	66·9<br>(62·4-71·2)	67·1<br>(61·3-72·4)	71·6<br>(52·1-91·2)
Croatia	83·0<br>(76·1-88·5)	87·5<br>(81·9-91·7)	87·6<br>(82·0-91·7)	89·2<br>(84·1-93·0)	79·1<br>(65·4-88·1)	83·9<br>(74·0-91·2)	84·1<br>(74·3-91·3)	86·9<br>(77·6-93·5)	65·9<br>(50·2-77·0)	46·7<br>(26·8-62·8)	46·4<br>(26·3-62·5)	31·8<br>(0·0-62·9)	71·0<br>(67·9-74·2)	82·8<br>(78·8-86·8)	82·4<br>(77·9-87·2)	95·7<br>(84·5-100·0)
Czech Republic	96·5<br>(94·7-98·2)	97·1<br>(95·2-98·6)	97·1<br>(95·3-98·6)	97·5<br>(95·7-99·1)	87·0<br>(81·9-91·1)	86·8<br>(77·4-93·2)	86·8<br>(77·2-93·4)	86·5<br>(73·5-95·0)	63·4<br>(46·6-76·4)	55·2<br>(36·8-70·0)	54·6<br>(36·1-69·4)	47·0<br>(11·5-74·2)	78·9<br>(76·0-81·6)	90·8<br>(87·8-93·7)	90·3<br>(87·0-93·3)	97·7<br>(89·3-100·0)
Hungary	94·7<br>(91·8-97·0)	95·9<br>(93·4-97·8)	95·9<br>(93·5-97·9)	96·6<br>(94·5-98·3)	92·4<br>(85·6-97·5)	93·0<br>(86·3-98·0)	93·1<br>(86·4-98·0)	93·5<br>(86·9-98·5)	58·0<br>(39·3-72·1)	51·4<br>(30·7-68·1)	50·8<br>(29·3-67·7)	46·4<br>(5·7-73·6)	76·9<br>(74·2-79·8)	74·0<br>(70·8-77·3)	75·5<br>(71·4-79·9)	83·0<br>(71·8-94·4)
Macedonia	87·5<br>(82·4-91·6)	86·9<br>(81·4-91·1)	87·4<br>(81·8-91·7)	85·8<br>(80·1-89·9)	86·4<br>(79·7-91·9)	81·2<br>(73·1-87·3)	81·6<br>(73·1-87·9)	79·0<br>(73·1-83·8)	60·8<br>(43·8-74·3)	48·1<br>(28·9-64·0)	47·3<br>(28·1-63·7)	33·4<br>(0·0-65·4)	73·7<br>(70·1-77·4)	84·3<br>(80·2-88·4)	84·5<br>(80·3-88·3)	87·2<br>(78·4-96·1)
Montenegro	82·0<br>(74·6-87·6)	76·2<br>(68·1-82·5)	76·2<br>(68·0-82·6)	75·6<br>(67·1-81·2)	75·4<br>(63·5-83·6)	84·0<br>(77·7-89·2)	84·3<br>(77·9-89·5)	88·1<br>(84·5-92·7)	53·8<br>(35·8-68·2)	34·2<br>(20·2-46·7)	33·6<br>(18·1-47·6)	18·7<br>(0·0-49·0)	73·1<br>(67·6-78·7)	89·3<br>(83·7-95·1)	89·5<br>(83·5-95·5)	97·4<br>(86·0-100·0)
Poland	82·9<br>(75·9-88·7)	88·2<br>(82·8-92·5)	88·4<br>(83·0-92·7)	90·9<br>(86·5-94·6)	78·4<br>(65·2-87·7)	84·0<br>(73·2-91·6)	84·3<br>(73·7-91·8)	87·9<br>(79·0-94·3)	69·1<br>(52·9-81·2)	61·6<br>(43·9-75·3)	60·9<br>(43·1-74·6)	54·1<br>(20·7-80·1)	74·2<br>(71·4-76·9)	93·9<br>(90·5-97·2)	94·3<br>(90·6-97·9)	99·2<br>(93·6-100·0)
Romania	69·8<br>(66·8-72·7)	78·7<br>(70·5-85·3)	79·0<br>(70·8-85·6)	83·6<br>(74·9-89·7)	80·8<br>(77·1-84·0)	81·7<br>(69·5-90·0)	81·9<br>(69·4-90·3)	84·5<br>(63·3-94·0)	73·7<br>(59·4-83·9)	58·6<br>(40·4-73·3)	58·0<br>(40·0-72·5)	39·8<br>(2·4-70·7)	53·3<br>(50·1-56·3)	66·3<br>(63·0-69·9)	66·8<br>(62·9-71·1)	75·7<br>(63·6-88·9)
Serbia	88·3<br>(82·8-92·5)	83·6<br>(78·5-87·8)	83·0<br>(77·7-87·3)	76·9<br>(70·8-81·8)	70·2<br>(56·0-79·4)	79·5<br>(73·3-84·5)	79·5<br>(73·1-84·6)	86·1<br>(83·5-88·5)	57·2<br>(37·8-71·9)	60·1<br>(50·6-68·0)	59·6<br>(48·9-68·9)	62·9<br>(40·5-80·2)	69·9<br>(66·0-73·7)	69·6<br>(65·6-73·5)	69·0<br>(64·8-73·1)	70·5<br>(60·9-78·7)
Slovakia	84·4<br>(77·8-89·7)	88·6<br>(83·0-92·8)	88·7<br>(83·2-92·9)	90·5<br>(85·6-94·4)	79·5<br>(66·9-88·9)	84·6<br>(74·8-91·9)	84·8<br>(75·1-92·1)	87·8<br>(79·5-94·0)	70·6<br>(54·5-81·8)	61·2<br>(45·0-73·4)	60·4<br>(44·5-72·8)	48·9<br>(16·5-75·0)	74·3<br>(71·3-77·3)	85·3<br>(80·9-89·6)	85·7<br>(80·7-90·5)	93·0<br>(81·4-100·0)
Slovenia	84·7<br>(78·4-89·8)	88·5<br>(83·7-92·6)	88·7<br>(83·9-92·8)	90·2<br>(85·9-94·0)	79·1<br>(65·3-88·0)	83·9<br>(73·4-91·6)	84·1<br>(73·5-91·8)	86·7<br>(75·3-94·1)	57·2<br>(37·6-72·1)	41·0<br>(21·1-58·9)	40·3<br>(19·7-58·3)	27·8<br>(0·0-62·3)	76·9<br>(73·3-80·4)	86·6<br>(82·3-90·8)	88·3<br>(83·7-92·8)	97·7<br>(86·3-100·0)
**Central Asia**																
Armenia	67·7<br>(61·7-73·2)	69·6<br>(57·9-79·8)	70·8<br>(58·1-81·4)	74·1<br>(61·6-85·0)	87·0<br>(82·2-91·0)	81·9<br>(74·3-87·6)	82·4<br>(74·6-88·2)	81·1<br>(70·7-88·2)	56·7<br>(47·5-65·4)	37·6<br>(23·2-49·7)	36·7<br>(22·6-49·3)	22·2<br>(0·0-47·8)	47·8<br>(44·9-50·8)	69·0<br>(65·5-72·5)	68·6<br>(64·6-72·8)	79·5<br>(67·1-93·5)

Location	Indicator 3.1.2: Proportion of births attended by skilled health personnel (%)				Indicator 3.2.1: Under-5 mortality rate (probability of dying before the age of 5 per 1,000 livebirths)				Indicator 3.2.2: Neonatal mortality rate (probability of dying during the first 28 days of life per 1,000 livebirths)				Indicator 3.3.1: Age-standardised rate of new HIV infections (per 1,000 population)			
	**2000**	**2015**	**2016**	**2030**	**2000**	**2015**	**2016**	**2030**	**2000**	**2015**	**2016**	**2030**	**2000**	**2015**	**2016**	**2030**
Sweden	98·9 (98·6-99·2)	99·5 (99·3-99·6)	99·5 (99·3-99·6)	99·7 (99·5-99·9)	87·7 (83·6-91·4)	96·4 (94·5-98·2)	97·0 (95·1-98·5)	99·7 (95·7-100·0)	82·7 (78·3-86·9)	93·8 (91·6-96·0)	94·6 (91·2-97·4)	99·1 (91·8-100·0)	63·0 (55·1-72·4)	62·3 (54·0-74·8)	62·3 (54·0-74·8)	64·9 (56·5-77·9)
Switzerland	98·9 (98·6-99·2)	99·6 (99·4-99·7)	99·6 (99·4-99·7)	99·8 (99·6-100·0)	78·7 (75·1-82·0)	86·7 (85·0-88·4)	87·7 (85·9-89·0)	94·4 (87·5-99·4)	69·3 (65·7-73·0)	76·0 (74·2-77·8)	77·2 (75·1-78·9)	82·1 (75·3-88·3)	56·9 (48·8-66·8)	56·2 (48·2-69·1)	56·2 (48·2-69·0)	57·1 (48·5-71·1)
United Kingdom	98·5 (98·1-98·8)	99·3 (99·1-99·5)	99·3 (99·1-99·5)	99·7 (99·4-99·9)	75·6 (72·3-78·7)	84·1 (81·5-86·3)	84·3 (82·3-86·2)	90·9 (87·9-93·6)	67·7 (64·6-70·5)	77·3 (75·1-79·2)	77·5 (75·6-79·4)	85·9 (81·7-89·6)	50·6 (43·6-58·2)	54·8 (47·8-62·5)	54·8 (47·8-62·5)	56·6 (49·7-64·2)
**Southern Latin America**																
Argentina	97·8 (96·9-98·5)	98·7 (98·3-99·1)	98·8 (98·3-99·2)	99·4 (98·6-99·9)	49·5 (47·3-51·7)	61·0 (58·9-63·1)	61·5 (59·6-63·4)	70·2 (68·4-72·2)	37·8 (35·5-40·0)	50·8 (48·7-52·9)	51·4 (49·6-53·4)	60·4 (57·0-64·1)	47·5 (41·3-53·7)	46·8 (40·1-54·0)	47·0 (40·3-54·2)	49·8 (42·9-56·8)
Chile	98·8 (98·2-99·2)	99·3 (98·8-99·6)	99·3 (98·9-99·6)	99·7 (99·0-100·0)	63·8 (61·0-66·5)	70·9 (68·7-73·1)	71·7 (69·7-73·7)	78·8 (76·7-80·8)	56·6 (53·9-59·2)	59·9 (57·7-62·1)	60·8 (58·7-62·9)	65·5 (61·7-69·6)	52·6 (46·1-58·9)	59·7 (50·7-67·7)	62·5 (53·4-70·8)	62·6 (53·5-71·3)
Uruguay	95·7 (93·5-97·2)	98·6 (97·8-99·1)	98·6 (97·9-99·2)	99·3 (98·2-99·9)	55·2 (52·4-57·8)	68·8 (66·8-70·3)	69·3 (65·4-72·1)	86·6 (71·3-99·6)	47·7 (44·9-50·4)	62·4 (59·9-64·4)	62·9 (58·5-66·5)	81·2 (63·8-96·2)	45·1 (38·7-51·3)	44·9 (39·0-51·4)	45·6 (39·7-52·2)	50·1 (43·6-56·8)
**Eastern Europe**																
Belarus	98·6 (97·4-99·3)	99·5 (98·9-99·9)	99·5 (98·9-99·9)	99·8 (98·9-100·0)	55·2 (50·6-60·0)	78·9 (71·9-85·3)	80·1 (72·6-86·6)	98·2 (88·1-100·0)	46·0 (40·8-51·7)	75·2 (69·1-80·3)	76·5 (70·1-81·8)	98·7 (89·6-100·0)	57·7 (50·4-64·3)	46·9 (39·1-55·1)	45·9 (38·3-54·0)	47·4 (39·6-55·4)
Estonia	98·9 (97·8-99·5)	99·8 (99·4-100·0)	99·8 (99·4-100·0)	100·0 (99·7-100·0)	63·3 (59·9-66·8)	92·0 (86·1-97·6)	93·1 (87·0-99·3)	98·6 (84·5-100·0)	57·7 (54·2-61·4)	94·3 (88·0-100·0)	95·4 (89·1-100·0)	99·5 (91·1-100·0)	61·9 (55·6-68·7)	59·3 (53·0-65·7)	59·4 (53·1-65·7)	61·0 (54·7-67·2)
Latvia	99·0 (98·1-99·5)	99·6 (99·0-99·9)	99·6 (99·0-100·0)	99·9 (99·2-100·0)	59·8 (56·3-62·9)	82·8 (76·8-89·2)	83·4 (75·3-91·4)	98·0 (86·4-100·0)	51·6 (48·1-55·0)	78·2 (72·3-84·6)	78·9 (70·9-87·3)	97·1 (83·3-100·0)	61·5 (56·8-66·6)	57·0 (52·0-62·4)	57·0 (52·0-62·4)	58·8 (54·1-63·7)
Lithuania	99·0 (98·1-99·5)	99·5 (98·8-99·9)	99·5 (98·8-99·9)	99·8 (98·8-100·0)	63·4 (60·4-66·4)	80·8 (77·0-84·4)	83·8 (79·8-87·8)	98·3 (90·5-100·0)	58·8 (55·3-62·1)	78·2 (74·6-82·0)	82·0 (77·9-86·3)	99·4 (94·0-100·0)	67·8 (61·3-73·4)	75·2 (65·9-91·3)	75·3 (66·0-91·4)	77·1 (67·7-95·0)
Moldova	98·9 (98·1-99·5)	99·5 (98·9-99·9)	99·6 (99·1-99·9)	99·9 (99·3-100·0)	39·7 (34·6-45·0)	62·0 (53·7-70·6)	63·0 (54·2-72·7)	79·4 (55·0-100·0)	24·8 (19·2-30·8)	47·7 (38·5-57·3)	48·8 (38·7-59·8)	67·7 (38·8-98·0)	54·7 (49·4-59·9)	58·8 (52·2-65·7)	60·0 (53·1-66·9)	59·2 (53·5-65·4)
Russia	98·7 (97·7-99·4)	99·7 (99·1-99·9)	99·7 (99·1-100·0)	99·9 (99·3-100·0)	50·2 (48·0-52·5)	69·0 (66·0-71·8)	70·2 (67·1-73·1)	89·5 (85·0-93·5)	40·5 (37·9-43·2)	62·9 (59·7-66·0)	64·2 (61·2-67·2)	85·6 (81·5-89·3)	47·5 (41·1-53·4)	44·4 (36·2-51·8)	44·1 (35·6-51·4)	44·7 (36·9-51·9)
Ukraine	98·8 (98·0-99·5)	99·4 (98·7-99·8)	99·3 (98·6-99·7)	99·6 (98·2-100·0)	49·2 (42·9-55·0)	68·0 (58·7-77·3)	68·7 (58·4-78·9)	86·3 (59·6-100·0)	34·1 (27·3-40·7)	58·7 (49·9-68·0)	59·3 (49·5-69·4)	80·3 (51·8-100·0)	37·2 (31·1-43·2)	39·4 (33·9-44·9)	38·3 (32·8-43·9)	39·0 (33·8-44·3)
**Central Europe**																
Albania	97·3 (96·4-98·1)	99·3 (98·6-99·8)	99·3 (98·7-99·8)	99·8 (99·0-100·0)	42·8 (37·9-47·5)	58·3 (51·1-64·7)	59·1 (51·9-65·5)	75·0 (53·7-95·4)	38·5 (33·0-43·7)	49·4 (41·0-56·7)	50·5 (42·1-57·6)	68·4 (44·3-91·8)	100·0 (100·0-100·0)	100·0 (100·0-100·0)	100·0 (100·0-100·0)	100·0 (100·0-100·0)
Bosnia and Herzegovina	98·9 (98·1-99·5)	99·5 (99·0-99·8)	99·5 (99·0-99·8)	99·8 (99·0-100·0)	64·0 (60·8-67·1)	80·8 (76·1-85·1)	81·4 (76·6-85·9)	96·5 (88·1-100·0)	49·4 (46·8-52·0)	68·6 (63·6-73·4)	69·4 (63·9-74·7)	89·1 (77·6-100·0)	100·0 (100·0-100·0)	99·1 (96·2-100·0)	99·1 (96·2-100·0)	99·8 (98·3-100·0)
Bulgaria	98·8 (97·9-99·4)	99·4 (98·6-99·8)	99·4 (98·8-99·8)	99·8 (98·7-100·0)	54·5 (51·8-57·2)	70·1 (65·6-74·1)	70·6 (64·3-76·0)	78·8 (60·0-96·3)	48·4 (45·8-51·1)	65·7 (59·9-71·0)	66·3 (58·1-72·8)	77·9 (55·1-98·3)	78·0 (72·3-83·2)	79·6 (71·9-87·3)	79·6 (71·9-87·3)	77·2 (69·6-85·2)
Croatia	98·8 (97·8-99·4)	99·4 (98·8-99·8)	99·5 (98·9-99·8)	99·8 (98·9-100·0)	68·2 (64·8-71·6)	84·0 (78·8-88·4)	84·6 (79·4-89·5)	96·8 (88·2-100·0)	56·4 (53·4-59·5)	75·0 (69·9-79·5)	75·7 (70·3-80·7)	90·0 (78·4-100·0)	92·2 (86·1-96·6)	91·3 (85·2-98·0)	91·3 (85·2-98·0)	92·6 (86·5-99·6)
Czech Republic	98·8 (98·0-99·4)	99·5 (98·8-99·8)	99·5 (99·0-99·9)	99·8 (99·1-100·0)	80·5 (76·5-84·4)	94·3 (90·8-97·5)	95·5 (93·8-97·0)	100·0 (100·0-100·0)	75·6 (71·8-79·1)	92·9 (89·1-96·3)	94·3 (92·0-96·3)	100·0 (100·0-100·0)	89·6 (83·8-94·9)	85·2 (78·7-93·1)	85·2 (78·7-93·2)	86·6 (80·4-94·2)
Hungary	97·3 (95·2-98·6)	99·0 (97·9-99·6)	99·1 (98·1-99·7)	99·8 (98·7-100·0)	64·8 (61·7-67·8)	79·5 (76·8-81·9)	79·7 (76·9-81·5)	88·6 (80·7-97·0)	54·5 (51·3-57·7)	73·8 (70·5-76·9)	73·9 (70·2-76·5)	86·4 (74·5-96·8)	86·0 (81·9-90·1)	88·5 (84·2-92·5)	88·5 (84·1-92·5)	89·5 (85·3-93·2)
Macedonia	96·4 (94·8-97·6)	98·9 (98·1-99·5)	98·9 (98·0-99·5)	99·6 (98·1-100·0)	57·6 (54·2-60·7)	64·8 (55·7-73·4)	66·5 (56·0-76·8)	84·2 (55·0-100·0)	44·7 (41·6-47·6)	51·4 (41·4-61·3)	53·5 (41·6-64·8)	74·9 (41·4-100·0)	99·9 (98·7-100·0)	98·6 (95·4-100·0)	98·6 (95·4-100·0)	99·0 (96·2-100·0)
Montenegro	98·3 (96·9-99·2)	99·3 (98·6-99·7)	99·3 (98·6-99·7)	99·6 (98·5-100·0)	53·9 (50·8-57·2)	81·4 (75·3-87·2)	81·9 (75·1-88·4)	98·6 (87·4-100·0)	39·9 (36·6-43·1)	73·0 (66·6-79·4)	73·6 (66·3-80·9)	97·4 (82·1-100·0)	95·6 (92·1-98·1)	92·6 (87·9-96·5)	92·6 (88·0-96·6)	93·6 (89·1-97·2)
Poland	99·0 (98·2-99·5)	99·6 (99·1-99·9)	99·7 (99·2-99·9)	99·9 (99·4-100·0)	67·1 (64·0-70·1)	83·8 (81·3-85·6)	85·0 (78·7-89·9)	98·3 (90·7-100·0)	55·7 (52·8-58·3)	75·9 (72·9-78·3)	77·3 (69·5-83·7)	95·8 (83·5-100·0)	78·4 (71·2-86·1)	79·9 (73·2-90·1)	79·9 (73·1-90·1)	81·1 (74·2-91·4)
Romania	98·3 (97·1-99·1)	99·3 (98·6-99·8)	99·4 (98·7-99·8)	99·8 (99·0-100·0)	46·4 (44·3-48·6)	66·4 (63·4-69·2)	67·4 (63·6-71·2)	87·5 (79·3-95·7)	41·7 (39·0-44·4)	61·5 (58·0-65·0)	62·6 (58·2-67·5)	81·6 (71·4-92·7)	79·2 (72·6-86·1)	68·5 (62·0-77·8)	68·4 (61·9-77·7)	67·8 (61·0-76·9)
Serbia	98·8 (97·8-99·4)	99·6 (99·2-99·9)	99·6 (99·2-99·9)	99·9 (99·3-100·0)	56·6 (51·8-61·7)	71·2 (66·7-75·7)	71·7 (66·3-77·1)	84·4 (72·0-98·1)	45·9 (40·8-51·2)	62·4 (57·9-67·0)	62·9 (57·4-68·6)	78·8 (64·9-93·8)	90·2 (72·0-100·0)	92·0 (85·3-96·4)	92·0 (85·3-96·4)	90·7 (83·8-95·8)
Slovakia	98·8 (98·0-99·4)	99·5 (98·9-99·9)	99·6 (99·0-99·9)	99·9 (99·3-100·0)	66·3 (63·0-69·6)	77·0 (74·4-79·1)	77·9 (73·8-80·8)	89·8 (74·3-100·0)	59·6 (56·4-62·9)	72·5 (69·5-74·8)	73·5 (68·1-77·6)	87·1 (68·7-100·0)	99·0 (96·0-100·0)	95·2 (90·2-98·8)	95·2 (90·2-98·9)	95·7 (90·8-99·1)
Slovenia	98·9 (98·1-99·4)	99·6 (99·2-99·9)	99·7 (99·2-99·9)	99·9 (99·5-100·0)	80·3 (76·0-84·3)	98·2 (93·3-100·0)	98·6 (93·9-100·0)	100·0 (100·0-100·0)	72·1 (68·0-76·0)	91·1 (86·3-95·4)	91·9 (87·0-96·2)	99·9 (98·8-100·0)	93·8 (89·1-97·1)	93·3 (87·8-97·5)	93·3 (87·8-97·5)	93·6 (88·3-97·8)
**Central Asia**																
Armenia	97·5 (96·2-98·4)	99·4 (97·8-100·0)	99·4 (97·6-100·0)	99·4 (94·8-100·0)	40·2 (35·9-44·3)	60·6 (53·9-67·6)	61·9 (55·1-68·9)	81·2 (63·5-99·7)	29·9 (25·3-34·5)	53·3 (45·9-61·0)	54·8 (47·1-62·4)	77·6 (57·2-99·1)	86·5 (74·1-95·1)	73·2 (65·1-81·2)	71·9 (63·4-80·3)	72·6 (64·2-80·7)

Location	**Indicator 3.3.2:** Age-standardised rate of tuberculosis cases (per 100,000 population)				**Indicator 3.3.3:** Age-standardised rate of malaria cases (per 1,000 population)				**Indicator 3.3.4:** Age-standardised rate of hepatitis B incidence (per 100,000 population)				**Indicator 3.3.5:** Age-standardised prevalence* of the sum of 15 neglected tropical diseases (NTDs) (%) **Prevalence estimates reported here may exceed 100% as they reflect the sum of prevalent cases of 15 NTDs.*			
	**2000**	**2015**	**2016**	**2030**	**2000**	**2015**	**2016**	**2030**	**2000**	**2015**	**2016**	**2030**	**2000**	**2015**	**2016**	**2030**
Sweden	96·7 (94·8-98·3)	92·7 (90·8-94·5)	92·9 (91·0-94·7)	95·3 (93·0-97·6)	100·0 (100·0-100·0)	100·0 (100·0-100·0)	100·0 (100·0-100·0)	100·0 (100·0-100·0)	96·1 (94·1-98·2)	100·0 (100·0-100·0)	100·0 (100·0-100·0)	100·0 (100·0-100·0)	100·0 (100·0-100·0)	100·0 (100·0-100·0)	100·0 (100·0-100·0)	100·0 (100·0-100·0)
Switzerland	89·5 (87·5-91·6)	95·0 (93·1-96·9)	95·0 (93·1-96·9)	97·7 (95·6-99·6)	100·0 (100·0-100·0)	100·0 (100·0-100·0)	100·0 (100·0-100·0)	100·0 (100·0-100·0)	63·6 (60·4-67·0)	69·7 (66·5-73·3)	70·0 (66·8-73·6)	74·6 (71·2-78·3)	100·0 (100·0-100·0)	100·0 (100·0-100·0)	100·0 (100·0-100·0)	100·0 (100·0-100·0)
United Kingdom	82·1 (80·7-83·5)	84·1 (82·4-85·6)	84·5 (82·8-86·0)	90·1 (88·3-91·8)	100·0 (100·0-100·0)	100·0 (100·0-100·0)	100·0 (100·0-100·0)	100·0 (100·0-100·0)	80·8 (78·9-82·6)	95·4 (94·2-96·4)	96·2 (95·1-97·2)	100·0 (100·0-100·0)	100·0 (100·0-100·0)	100·0 (100·0-100·0)	100·0 (100·0-100·0)	100·0 (100·0-100·0)
**Southern Latin America**																
Argentina	65·1 (63·5-66·7)	72·3 (70·5-74·0)	72·0 (70·2-73·7)	73·1 (70·1-75·9)	78·2 (73·5-81·1)	100·0 (100·0-100·0)	100·0 (100·0-100·0)	100·0 (100·0-100·0)	68·0 (65·2-70·1)	78·0 (75·6-79·9)	78·4 (76·0-80·3)	84·4 (82·1-86·4)	84·5 (83·3-85·7)	87·1 (85·9-88·2)	87·2 (86·1-88·3)	89·4 (88·4-90·4)
Chile	71·3 (69·5-73·1)	78·8 (76·9-80·6)	79·0 (77·1-80·9)	84·6 (82·7-86·6)	100·0 (100·0-100·0)	100·0 (100·0-100·0)	100·0 (100·0-100·0)	100·0 (100·0-100·0)	80·5 (78·4-82·6)	92·0 (90·2-93·9)	92·1 (90·2-94·0)	98·9 (96·9-100·0)	78·0 (73·9-81·7)	78·5 (74·0-82·3)	78·6 (74·0-82·4)	79·2 (74·7-83·0)
Uruguay	72·9 (71·4-74·5)	73·9 (72·4-75·5)	73·7 (72·2-75·3)	70·7 (69·0-72·3)	100·0 (100·0-100·0)	100·0 (100·0-100·0)	100·0 (100·0-100·0)	100·0 (100·0-100·0)	78·2 (76·4-79·9)	83·7 (81·9-85·4)	84·1 (82·3-85·8)	89·7 (87·6-91·7)	98·8 (98·6-99·0)	99·3 (99·1-99·4)	99·3 (99·1-99·4)	99·6 (99·5-99·7)
**Eastern Europe**																
Belarus	56·8 (54·6-58·8)	62·2 (60·1-64·2)	63·0 (60·9-65·1)	70·3 (65·9-73·9)	100·0 (100·0-100·0)	100·0 (100·0-100·0)	100·0 (100·0-100·0)	100·0 (100·0-100·0)	57·2 (54·5-59·7)	66·3 (64·1-68·4)	67·1 (64·9-69·2)	76·5 (74·1-79·3)	100·0 (99·9-100·0)	100·0 (100·0-100·0)	100·0 (100·0-100·0)	100·0 (100·0-100·0)
Estonia	58·2 (56·2-60·4)	74·0 (72·0-76·3)	74·7 (72·6-76·9)	84·4 (82·1-86·8)	100·0 (100·0-100·0)	100·0 (100·0-100·0)	100·0 (100·0-100·0)	100·0 (100·0-100·0)	70·4 (67·8-73·2)	81·2 (78·6-83·8)	81·4 (78·8-84·0)	84·2 (81·7-86·7)	100·0 (100·0-100·0)	100·0 (100·0-100·0)	100·0 (100·0-100·0)	100·0 (100·0-100·0)
Latvia	51·4 (49·6-53·2)	63·6 (61·7-65·5)	64·1 (62·2-66·1)	72·0 (69·8-74·1)	100·0 (100·0-100·0)	100·0 (100·0-100·0)	100·0 (100·0-100·0)	100·0 (100·0-100·0)	62·6 (59·8-65·5)	60·4 (58·5-62·3)	61·3 (59·4-63·2)	73·9 (71·7-76·0)	100·0 (99·9-100·0)	100·0 (100·0-100·0)	100·0 (100·0-100·0)	100·0 (100·0-100·0)
Lithuania	52·0 (50·0-53·9)	58·7 (56·7-60·6)	59·2 (57·2-61·1)	66·8 (64·6-68·9)	100·0 (100·0-100·0)	100·0 (100·0-100·0)	100·0 (100·0-100·0)	100·0 (100·0-100·0)	66·5 (64·6-68·5)	69·1 (67·1-71·0)	69·9 (68·0-71·8)	81·7 (79·5-83·7)	100·0 (100·0-100·0)	100·0 (100·0-100·0)	100·0 (100·0-100·0)	100·0 (100·0-100·0)
Moldova	50·7 (48·9-52·7)	50·7 (48·8-52·7)	51·3 (49·5-53·4)	59·4 (57·1-61·7)	100·0 (100·0-100·0)	100·0 (100·0-100·0)	100·0 (100·0-100·0)	100·0 (100·0-100·0)	45·6 (42·1-49·0)	69·9 (67·3-72·8)	70·1 (67·6-73·1)	74·0 (71·4-77·0)	99·9 (99·7-99·9)	99·9 (99·9-100·0)	99·9 (99·9-100·0)	100·0 (100·0-100·0)
Russia	47·8 (45·8-49·8)	52·4 (50·4-54·4)	52·9 (50·9-54·9)	56·6 (54·3-58·6)	100·0 (100·0-100·0)	100·0 (100·0-100·0)	100·0 (100·0-100·0)	100·0 (100·0-100·0)	58·0 (55·1-61·3)	69·7 (67·6-72·1)	70·2 (68·1-72·6)	77·6 (75·3-79·7)	98·9 (98·8-99·1)	99·0 (98·8-99·1)	99·0 (98·8-99·1)	99·0 (98·8-99·1)
Ukraine	52·1 (50·2-54·0)	53·5 (51·5-55·4)	54·0 (52·0-55·9)	61·3 (59·1-63·6)	100·0 (100·0-100·0)	100·0 (100·0-100·0)	100·0 (100·0-100·0)	100·0 (100·0-100·0)	59·4 (57·0-62·3)	62·6 (60·6-64·8)	62·6 (60·7-64·8)	63·1 (60·7-65·4)	99·7 (99·6-99·8)	99·7 (99·6-99·8)	99·7 (99·6-99·8)	99·7 (99·6-99·8)
**Central Europe**																
Albania	72·8 (70·7-74·9)	81·4 (79·3-83·5)	81·7 (79·6-83·8)	87·9 (85·5-90·2)	100·0 (100·0-100·0)	100·0 (100·0-100·0)	100·0 (100·0-100·0)	100·0 (100·0-100·0)	67·6 (65·3-70·1)	83·8 (81·9-85·9)	84·0 (82·0-86·0)	100·0 (99·7-100·0)	100·0 (99·9-100·0)	100·0 (100·0-100·0)	100·0 (100·0-100·0)	100·0 (100·0-100·0)
Bosnia and Herzegovina	63·1 (61·1-65·1)	68·9 (66·9-70·9)	69·4 (67·4-71·4)	75·8 (73·8-77·9)	100·0 (100·0-100·0)	100·0 (100·0-100·0)	100·0 (100·0-100·0)	100·0 (100·0-100·0)	74·7 (72·5-76·8)	78·3 (76·1-80·5)	79·0 (76·9-81·2)	88·8 (86·8-90·8)	99·9 (99·7-100·0)	100·0 (99·9-100·0)	100·0 (99·9-100·0)	100·0 (100·0-100·0)
Bulgaria	63·9 (61·9-65·9)	72·6 (70·6-74·8)	73·6 (71·5-75·7)	83·3 (80·7-85·9)	100·0 (100·0-100·0)	100·0 (100·0-100·0)	100·0 (100·0-100·0)	100·0 (100·0-100·0)	52·3 (49·9-55·4)	64·5 (62·0-67·6)	65·0 (62·5-68·1)	73·0 (70·4-75·7)	100·0 (99·9-100·0)	100·0 (99·9-100·0)	100·0 (100·0-100·0)	100·0 (100·0-100·0)
Croatia	69·8 (67·9-71·7)	84·2 (82·2-86·3)	85·1 (83·0-87·2)	96·8 (94·5-99·0)	100·0 (100·0-100·0)	100·0 (100·0-100·0)	100·0 (100·0-100·0)	100·0 (100·0-100·0)	83·5 (81·8-85·3)	88·2 (86·5-89·9)	88·5 (86·8-90·2)	92·9 (91·1-94·8)	100·0 (100·0-100·0)	100·0 (100·0-100·0)	100·0 (100·0-100·0)	100·0 (100·0-100·0)
Czech Republic	84·6 (82·4-86·8)	97·8 (95·5-100·0)	98·2 (96·0-100·0)	100·0 (100·0-100·0)	100·0 (100·0-100·0)	100·0 (100·0-100·0)	100·0 (100·0-100·0)	100·0 (100·0-100·0)	87·5 (85·6-89·2)	88·0 (86·1-89·8)	88·3 (86·4-90·1)	93·3 (91·2-95·2)	100·0 (100·0-100·0)	100·0 (100·0-100·0)	100·0 (100·0-100·0)	100·0 (100·0-100·0)
Hungary	73·0 (70·8-75·2)	89·4 (87·2-91·7)	90·6 (88·4-92·8)	100·0 (100·0-100·0)	100·0 (100·0-100·0)	100·0 (100·0-100·0)	100·0 (100·0-100·0)	100·0 (100·0-100·0)	84·6 (82·6-86·6)	90·1 (88·1-92·0)	90·2 (88·3-92·1)	95·2 (93·1-97·3)	100·0 (99·9-100·0)	100·0 (100·0-100·0)	100·0 (100·0-100·0)	100·0 (100·0-100·0)
Macedonia	58·7 (56·9-60·6)	72·4 (70·5-74·4)	72·7 (70·8-74·7)	77·8 (75·7-79·8)	100·0 (100·0-100·0)	100·0 (100·0-100·0)	100·0 (100·0-100·0)	100·0 (100·0-100·0)	68·2 (66·1-70·2)	78·7 (76·8-80·6)	78·8 (76·9-80·6)	79·6 (77·4-81·6)	100·0 (99·9-100·0)	100·0 (100·0-100·0)	100·0 (100·0-100·0)	100·0 (100·0-100·0)
Montenegro	72·5 (70·5-74·6)	78·6 (76·4-80·6)	78·9 (76·7-81·0)	83·5 (81·2-85·6)	100·0 (100·0-100·0)	100·0 (100·0-100·0)	100·0 (100·0-100·0)	100·0 (100·0-100·0)	64·4 (62·2-66·3)	75·0 (73·0-76·9)	75·4 (73·4-77·3)	81·2 (79·1-83·1)	100·0 (99·9-100·0)	100·0 (100·0-100·0)	100·0 (100·0-100·0)	100·0 (100·0-100·0)
Poland	71·2 (69·0-73·3)	81·0 (78·7-83·3)	81·5 (79·1-83·8)	87·9 (85·4-90·4)	100·0 (100·0-100·0)	100·0 (100·0-100·0)	100·0 (100·0-100·0)	100·0 (100·0-100·0)	83·3 (79·9-86·0)	97·2 (95·2-99·1)	97·3 (95·2-99·2)	100·0 (100·0-100·0)	100·0 (99·9-100·0)	100·0 (100·0-100·0)	100·0 (100·0-100·0)	100·0 (100·0-100·0)
Romania	43·8 (42·0-45·7)	52·0 (50·1-53·9)	52·6 (50·8-54·5)	61·9 (60·0-63·9)	100·0 (100·0-100·0)	100·0 (100·0-100·0)	100·0 (100·0-100·0)	100·0 (100·0-100·0)	68·5 (66·2-71·1)	64·3 (62·0-66·3)	64·4 (62·1-66·4)	65·3 (62·1-68·1)	99·9 (99·9-100·0)	100·0 (99·9-100·0)	100·0 (99·9-100·0)	100·0 (100·0-100·0)
Serbia	64·0 (62·0-66·0)	73·8 (71·7-75·9)	74·2 (72·1-76·3)	80·2 (78·1-82·3)	100·0 (100·0-100·0)	100·0 (100·0-100·0)	100·0 (100·0-100·0)	100·0 (100·0-100·0)	76·8 (74·6-79·2)	86·6 (84·7-88·9)	86·7 (84·8-89·1)	88·6 (86·6-91·2)	99·9 (99·9-99·9)	100·0 (100·0-100·0)	100·0 (100·0-100·0)	100·0 (100·0-100·0)
Slovakia	82·4 (80·3-84·5)	91·9 (89·7-94·0)	92·6 (90·4-94·7)	100·0 (99·9-100·0)	100·0 (100·0-100·0)	100·0 (100·0-100·0)	100·0 (100·0-100·0)	100·0 (100·0-100·0)	81·9 (79·9-83·6)	89·1 (87·3-90·8)	89·4 (87·6-91·0)	93·4 (91·6-95·0)	100·0 (100·0-100·0)	100·0 (100·0-100·0)	100·0 (100·0-100·0)	100·0 (100·0-100·0)
Slovenia	79·0 (76·9-81·1)	92·1 (89·9-94·2)	92·6 (90·5-94·8)	99·9 (98·8-100·0)	100·0 (100·0-100·0)	100·0 (100·0-100·0)	100·0 (100·0-100·0)	100·0 (100·0-100·0)	86·5 (84·8-88·4)	92·5 (90·6-94·5)	92·8 (90·9-94·8)	96·4 (94·1-98·7)	100·0 (100·0-100·0)	100·0 (100·0-100·0)	100·0 (100·0-100·0)	100·0 (100·0-100·0)
**Central Asia**																
Armenia	57·6 (55·4-59·6)	62·1 (60·0-64·3)	62·9 (60·6-65·1)	71·7 (66·5-75·8)	100·0 (100·0-100·0)	100·0 (100·0-100·0)	100·0 (100·0-100·0)	100·0 (100·0-100·0)	59·4 (57·0-62·0)	61·9 (59·8-64·4)	63·4 (61·4-65·9)	72·0 (69·3-75·0)	99·3 (99·1-99·5)	99·4 (99·2-99·5)	99·4 (99·2-99·5)	99·4 (99·2-99·5)

Location	Indicator 3.4.1: Age-standardised death rate due to cardiovascular disease, cancer, diabetes, and chronic respiratory disease in populations aged 30-70 (per 100,000 population)				Indicator 3.4.2: Age-standardised death rate due to self-harm (per 100,000 population)				Indicator 3.5.2: Risk-weighted prevalence of alcohol consumption, as measured by the summary exposure value (SEV) for alcohol use (%)				Indicator 3.6.1: Age-standardised death rate due to road injuries (per 100,000 population)			
	**2000**	**2015**	**2016**	**2030**	**2000**	**2015**	**2016**	**2030**	**2000**	**2015**	**2016**	**2030**	**2000**	**2015**	**2016**	**2030**
Sweden	76·2 (73·8-78·5)	93·7 (89·0-98·4)	93·5 (87·0-100·0)	99·7 (95·9-100·0)	33·3 (29·4-39·0)	38·1 (32·0-46·6)	38·4 (31·2-47·1)	42·7 (28·4-57·7)	37·2 (23·6-49·8)	40·2 (16·2-58·9)	39·9 (15·4-59·0)	36·1 (2·7-61·8)	77·8 (75·3-80·8)	99·3 (96·4-100·0)	98·8 (94·9-100·0)	100·0 (100·0-100·0)
Switzerland	80·3 (76·4-84·4)	98·3 (91·9-100·0)	97·6 (86·7-100·0)	99·6 (94·4-100·0)	21·7 (13·5-25·7)	39·7 (23·7-48·4)	39·7 (22·4-52·1)	58·9 (29·1-85·1)	38·8 (26·4-50·3)	44·0 (23·7-60·1)	44·2 (23·3-60·5)	46·4 (16·2-67·2)	69·4 (66·0-73·0)	98·1 (92·7-100·0)	97·6 (89·0-100·0)	100·0 (100·0-100·0)
United Kingdom	62·7 (60·9-64·4)	82·3 (80·2-84·3)	82·5 (80·4-84·5)	98·9 (95·7-100·0)	49·9 (47·1-54·4)	55·4 (52·0-61·2)	55·7 (52·3-61·3)	59·6 (54·1-66·0)	42·3 (14·5-64·2)	39·2 (10·3-62·9)	39·0 (10·1-62·8)	36·4 (6·3-61·4)	73·6 (70·4-76·2)	97·3 (94·0-99·5)	96·9 (93·5-99·1)	100·0 (100·0-100·0)
**Southern Latin America**																
Argentina	49·2 (46·5-51·7)	61·7 (57·9-65·7)	62·1 (57·7-66·7)	73·9 (64·5-84·3)	36·9 (33·5-43·8)	40·8 (36·0-46·9)	41·4 (36·2-47·9)	43·2 (31·4-54·4)	36·1 (21·6-50·1)	40·7 (16·4-61·0)	40·1 (15·2-61·0)	32·9 (0·0-61·3)	44·5 (41·7-47·6)	45·6 (42·0-49·5)	46·8 (43·0-51·2)	47·0 (39·2-54·9)
Chile	70·5 (66·9-74·0)	83·9 (75·9-92·0)	84·4 (71·3-97·3)	93·1 (71·5-100·0)	34·3 (29·2-39·7)	42·9 (32·3-51·4)	42·9 (29·8-55·0)	52·1 (27·1-81·0)	53·5 (43·9-63·1)	38·4 (16·8-56·2)	37·2 (14·8-55·6)	19·9 (0·0-47·3)	44·0 (40·6-47·5)	54·2 (47·8-61·1)	54·8 (44·7-64·4)	66·1 (46·2-87·6)
Uruguay	47·6 (45·2-50·0)	59·9 (56·7-63·2)	60·5 (56·7-64·3)	71·6 (62·8-80·5)	25·5 (21·8-32·9)	24·4 (20·2-32·4)	24·9 (20·3-32·6)	27·0 (16·4-38·3)	41·7 (31·3-52·1)	49·2 (29·1-65·6)	48·9 (28·4-65·7)	45·5 (17·5-67·6)	42·5 (40·0-45·4)	41·7 (38·4-45·4)	42·8 (39·1-46·6)	41·7 (34·3-49·7)
**Eastern Europe**																
Belarus	16·4 (12·6-20·0)	37·1 (30·9-43·4)	38·0 (28·9-47·7)	53·6 (31·5-80·9)	0·1 (0·0-1·5)	12·5 (3·6-19·9)	12·5 (2·1-22·2)	26·8 (4·9-53·8)	9·9 (0·0-23·4)	3·0 (0·0-18·4)	2·9 (0·0-18·3)	2·1 (0·0-17·5)	28·4 (24·6-32·0)	49·3 (44·2-54·2)	49·6 (42·3-57·0)	64·1 (50·8-83·2)
Estonia	31·1 (28·1-34·0)	60·1 (55·3-65·2)	60·9 (54·4-67·8)	74·5 (57·1-100·0)	1·6 (0·0-5·2)	28·3 (10·9-36·3)	28·5 (11·1-38·1)	39·6 (11·9-67·5)	41·1 (31·4-51·3)	10·5 (0·0-29·9)	10·4 (0·0-30·2)	8·9 (0·0-32·7)	37·2 (33·5-40·5)	76·5 (72·1-80·8)	77·0 (71·2-82·5)	91·3 (77·9-100·0)
Latvia	28·6 (25·1-32·1)	43·5 (38·2-49·0)	44·2 (37·6-51·2)	60·2 (43·5-81·2)	1·2 (0·0-5·1)	19·3 (6·6-26·4)	19·3 (6·8-27·8)	33·6 (9·3-54·3)	36·1 (25·6-46·9)	20·2 (1·8-40·6)	20·1 (1·3-40·8)	18·9 (0·0-45·4)	24·8 (21·6-28·0)	60·0 (55·2-65·0)	60·0 (54·5-66·0)	80·5 (66·4-97·6)
Lithuania	39·0 (35·8-42·0)	47·8 (44·3-51·3)	48·6 (44·4-53·0)	59·2 (45·6-73·2)	0·0 (0·0-0·0)	0·8 (0·0-5·8)	1·2 (0·0-6·5)	8·9 (0·0-22·8)	31·3 (21·9-40·5)	4·4 (0·0-17·8)	4·2 (0·0-17·7)	2·9 (0·0-17·8)	29·1 (26·0-32·0)	55·6 (52·4-59·0)	55·7 (51·9-59·8)	80·2 (67·5-93·8)
Moldova	23·3 (18·9-27·6)	36·8 (31·0-42·4)	37·3 (31·0-43·6)	45·8 (26·7-62·6)	21·1 (16·1-26·9)	29·6 (22·6-36·9)	29·4 (21·7-37·1)	30·0 (13·1-47·0)	14·4 (0·0-39·9)	26·3 (0·0-53·4)	26·3 (0·0-53·6)	26·1 (0·0-57·7)	39·8 (36·0-43·5)	56·8 (51·8-61·7)	57·3 (51·6-62·9)	67·0 (52·5-81·9)
Russia	11·4 (6·2-17·0)	32·0 (20·9-42·8)	32·4 (15·7-49·0)	51·6 (14·4-90·0)	0·0 (0·0-0·0)	2·7 (0·0-11·3)	3·5 (0·0-16·1)	14·0 (0·0-47·2)	38·7 (26·6-51·1)	22·6 (3·2-42·0)	22·6 (2·9-42·3)	23·0 (0·0-47·0)	21·9 (16·7-26·9)	38·7 (30·2-47·2)	39·3 (27·6-51·9)	51·7 (23·4-83·6)
Ukraine	16·4 (12·6-20·3)	34·9 (26·3-43·1)	35·1 (21·5-48·0)	41·1 (7·7-77·8)	0·4 (0·0-3·7)	18·1 (6·5-27·2)	18·0 (4·0-32·1)	26·9 (0·0-59·7)	5·7 (0·0-20·4)	0·1 (0·0-0·4)	0·2 (0·0-1·5)	1·2 (0·0-14·3)	35·0 (31·2-38·6)	50·6 (44·0-57·7)	50·4 (40·0-61·1)	56·4 (26·8-90·4)
**Central Europe**																
Albania	56·5 (54·2-58·9)	63·9 (57·7-70·3)	64·4 (57·7-71·3)	73·2 (57·6-89·9)	67·0 (61·3-72·1)	68·5 (60·1-78·6)	69·1 (60·4-79·1)	78·5 (59·5-94·7)	66·9 (58·0-75·1)	55·1 (38·2-68·9)	55·1 (37·6-69·0)	54·3 (29·9-72·1)	61·9 (57·2-65·4)	69·7 (64·0-75·5)	70·5 (64·1-76·6)	82·2 (67·0-97·9)
Bosnia and Herzegovina	41·6 (35·1-47·6)	52·0 (44·3-60·4)	53·0 (44·7-61·8)	64·2 (46·9-82·4)	46·1 (36·9-54·2)	58·8 (48·0-68·3)	58·8 (48·0-68·8)	70·7 (48·4-93·3)	40·9 (30·6-49·5)	45·0 (34·4-54·1)	45·0 (34·4-54·2)	46·0 (35·2-55·2)	78·0 (71·5-84·2)	75·7 (68·5-82·7)	75·8 (68·3-82·8)	77·1 (62·4-91·6)
Bulgaria	29·2 (26·5-31·8)	41·7 (35·2-48·5)	42·0 (33·2-51·0)	52·4 (36·0-72·1)	26·1 (22·2-29·7)	43·8 (29·0-52·4)	43·6 (28·1-53·9)	58·2 (31·4-78·3)	43·1 (34·6-52·7)	36·1 (19·5-51·4)	35·5 (18·2-51·2)	26·6 (0·9-49·5)	52·8 (49·9-55·9)	67·0 (60·7-73·1)	67·5 (60·1-74·6)	79·9 (60·9-100·0)
Croatia	40·8 (36·8-44·7)	58·9 (52·9-65·0)	58·9 (51·9-66·7)	75·3 (59·1-92·6)	18·8 (14·1-23·2)	31·7 (20·6-38·2)	31·5 (20·1-39·2)	43·4 (23·9-60·5)	16·1 (7·4-24·5)	30·0 (15·3-44·6)	30·8 (15·8-45·6)	40·9 (21·2-59·2)	43·2 (39·9-46·5)	63·7 (59·2-68·4)	63·8 (58·5-69·5)	86·2 (73·7-99·9)
Czech Republic	44·7 (42·4-47·1)	67·6 (65·0-70·0)	68·4 (64·8-71·7)	90·6 (84·1-96·9)	29·9 (24·4-33·5)	39·7 (29·6-47·6)	40·1 (29·7-48·0)	48·0 (32·1-61·2)	13·9 (5·4-23·7)	19·6 (3·9-36·1)	20·8 (4·9-37·3)	35·7 (17·4-54·1)	49·1 (46·8-51·3)	73·0 (70·4-75·6)	73·9 (70·8-77·1)	95·3 (86·6-100·0)
Hungary	27·7 (24·0-31·2)	42·4 (37·9-46·7)	42·6 (36·7-49·4)	56·1 (43·0-71·1)	7·4 (2·2-11·2)	21·8 (9·6-27·9)	21·2 (8·5-28·7)	35·3 (14·0-50·6)	9·9 (0·9-18·3)	21·1 (6·0-36·7)	22·3 (6·9-38·2)	38·9 (21·1-56·3)	48·2 (45·2-51·4)	73·2 (69·5-77·0)	72·9 (68·0-78·4)	93·0 (82·7-100·0)
Macedonia	30·2 (26·6-33·8)	42·2 (38·5-46·2)	43·1 (39·2-47·2)	54·6 (47·0-62·4)	47·8 (40·9-53·0)	52·9 (46·0-58·9)	53·0 (46·2-59·0)	56·4 (44·2-67·2)	75·0 (68·4-81·0)	80·8 (72·6-87·2)	80·7 (72·4-87·2)	79·4 (68·8-87·1)	68·1 (64·0-71·7)	70·1 (65·8-74·4)	70·2 (65·9-74·7)	71·8 (63·0-81·3)
Montenegro	39·4 (35·6-42·8)	50·8 (45·4-55·7)	51·3 (45·8-56·6)	60·6 (48·9-71·6)	41·7 (35·0-46·9)	42·7 (36·3-48·8)	41·6 (34·8-48·0)	46·1 (32·7-57·4)	39·5 (22·7-57·4)	27·8 (7·0-48·3)	27·3 (6·3-48·0)	19·6 (0·0-43·5)	57·2 (52·9-61·3)	64·3 (59·4-69·9)	64·7 (59·7-70·1)	70·8 (60·0-82·5)
Poland	38·1 (35·1-40·7)	56·7 (52·7-60·7)	57·8 (52·7-63·0)	73·2 (60·3-87·8)	24·8 (20·3-35·1)	30·4 (24·5-42·7)	30·9 (23·9-43·1)	37·9 (22·9-57·0)	44·4 (35·4-53·2)	35·6 (19·0-51·7)	35·4 (18·4-51·9)	32·8 (9·5-54·3)	37·2 (33·9-40·4)	60·4 (56·1-64·0)	61·2 (56·1-65·7)	81·7 (72·9-91·4)
Romania	32·6 (29·2-35·8)	47·0 (43·0-50·9)	47·6 (41·8-52·9)	59·3 (44·3-72·2)	41·7 (37·9-49·0)	45·7 (40·5-55·7)	46·0 (39·2-56·7)	49·9 (33·8-67·2)	6·3 (0·0-16·9)	6·1 (0·0-21·0)	6·3 (0·0-21·4)	9·1 (0·0-28·1)	46·2 (43·0-49·3)	59·9 (56·3-63·5)	60·0 (55·4-64·8)	73·0 (61·9-85·7)
Serbia	30·4 (26·6-34·6)	48·3 (44·8-52·1)	48·9 (45·2-52·7)	62·1 (54·7-70·6)	18·7 (12·8-23·6)	28·9 (21·8-33·8)	28·1 (20·9-33·3)	36·8 (25·6-46·4)	43·9 (30·6-56·9)	28·2 (9·9-47·6)	27·6 (9·1-47·2)	19·5 (0·0-42·7)	53·3 (49·4-57·4)	62·0 (57·8-66·3)	60·3 (56·0-64·9)	70·6 (61·7-79·5)
Slovakia	36·6 (33·8-39·7)	56·1 (51·0-60·8)	56·5 (49·9-63·2)	76·3 (62·4-92·2)	33·2 (28·2-40·1)	43·4 (36·8-50·2)	43·6 (35·8-51·4)	53·1 (35·0-70·1)	18·2 (8·5-29·4)	26·3 (8·3-42·8)	26·5 (8·0-43·5)	29·6 (5·0-51·8)	44·4 (41·4-48·1)	69·6 (64·6-74·9)	69·6 (63·5-75·8)	89·5 (78·7-100·0)
Slovenia	55·5 (50·6-60·4)	81·2 (74·7-88·2)	82·2 (74·8-90·1)	98·8 (90·5-100·0)	6·3 (1·7-12·0)	26·7 (13·8-34·3)	26·8 (13·8-35·4)	42·9 (18·5-60·8)	26·9 (16·8-37·6)	37·0 (19·6-53·0)	37·4 (19·5-53·7)	43·0 (19·6-63·3)	43·9 (40·0-47·9)	79·8 (74·9-85·0)	79·8 (74·3-85·7)	99·7 (96·0-100·0)
**Central Asia**																
Armenia	32·1 (28·3-35·8)	48·3 (44·1-52·4)	49·5 (43·7-55·7)	66·5 (49·6-86·9)	79·2 (67·2-84·8)	57·7 (50·7-75·4)	58·7 (50·2-77·1)	50·2 (25·9-84·4)	73·5 (66·8-80·0)	62·2 (49·6-73·8)	61·0 (47·9-73·1)	41·1 (18·7-61·3)	54·3 (50·8-57·5)	67·2 (63·3-71·4)	67·5 (61·9-73·2)	80·5 (67·7-94·2)

Location	Indicator 3.7.1: Proportion of women of reproductive age (15-49 years) who have their need for family planning satisfied with modern contraception methods (%)				Indicator 3.7.2: Number of livebirths per 1,000 women aged 10-14 years and women aged 15-19 years				Indicator 3.8.1: Coverage of essential health services, as defined by the UHC index comprised of the coverage of 9 tracer interventions and risk-standardised death rates from 32 causes amenable to personal healthcare (scale of 0 to 100)				Indicator 3.9.1: Age-standardised death rate attributable to household air pollution and ambient air pollution (per 100,000 population)			
	**2000**	**2015**	**2016**	**2030**	**2000**	**2015**	**2016**	**2030**	**2000**	**2015**	**2016**	**2030**	**2000**	**2015**	**2016**	**2030**
Sweden	86·9 (79·7-93·0)	87·4 (80·6-93·6)	87·6 (80·6-93·5)	89·4 (75·6-99·9)	80·8 (77·8-83·6)	83·2 (81·7-84·4)	83·5 (81·7-85·1)	88·5 (81·9-95·0)	89·3 (87·7-90·7)	97·9 (95·0-100·0)	98·3 (95·3-100·0)	99·9 (99·3-100·0)	92·5 (88·0-97·3)	100·0 (100·0-100·0)	100·0 (100·0-100·0)	100·0 (100·0-100·0)
Switzerland	91·1 (84·7-96·9)	92·7 (87·2-97·5)	92·9 (87·2-97·9)	94·6 (83·9-100·0)	83·4 (80·4-86·2)	99·3 (97·6-100·0)	100·0 (100·0-100·0)	100·0 (100·0-100·0)	87·4 (85·1-89·7)	99·7 (96·9-100·0)	99·8 (97·2-100·0)	100·0 (100·0-100·0)	79·4 (77·1-81·9)	94·8 (89·0-100·0)	95·5 (89·7-100·0)	100·0 (100·0-100·0)
United Kingdom	95·7 (91·3-99·5)	97·8 (93·3-100·0)	97·9 (93·4-100·0)	98·4 (91·1-100·0)	43·5 (41·8-45·2)	60·3 (58·6-62·0)	62·5 (61·1-63·8)	90·1 (83·2-94·5)	75·4 (73·7-77·0)	87·7 (86·1-89·2)	88·3 (86·8-89·9)	96·5 (94·5-98·4)	65·3 (63·2-67·5)	81·6 (79·5-83·9)	82·0 (79·9-84·3)	98·1 (90·9-100·0)
**Southern Latin America**																
Argentina	70·9 (61·6-79·7)	79·4 (70·8-87·3)	79·7 (70·9-87·7)	86·6 (71·5-98·9)	21·7 (20·3-23·3)	22·7 (20·3-24·9)	22·6 (20·0-25·0)	23·5 (16·6-29·9)	49·6 (47·2-51·7)	58·5 (55·3-61·5)	59·1 (56·0-62·2)	67·8 (63·3-72·1)	57·2 (54·5-60·1)	67·0 (63·5-70·9)	67·7 (64·1-71·5)	76·8 (72·9-81·0)
Chile	73·7 (65·0-81·7)	82·3 (74·5-89·7)	82·6 (74·8-89·9)	89·3 (76·3-99·8)	25·2 (23·9-26·5)	27·4 (24·3-30·3)	27·8 (24·7-30·7)	32·5 (25·2-39·4)	60·8 (58·5-63·1)	75·4 (68·8-81·7)	76·2 (69·1-83·0)	87·4 (75·2-98·8)	59·7 (57·1-62·2)	76·0 (69·5-82·5)	76·7 (70·0-83·4)	93·0 (81·7-100·0)
Uruguay	69·6 (59·7-78·2)	78·0 (69·6-85·3)	78·4 (70·1-85·8)	85·0 (68·7-96·4)	21·6 (19·9-23·7)	24·3 (21·2-27·1)	24·5 (21·5-27·4)	26·2 (18·4-33·5)	55·5 (53·2-57·7)	64·3 (61·2-67·1)	65·0 (61·8-67·9)	74·4 (69·7-79·2)	64·4 (61·9-67·1)	76·9 (74·0-80·0)	77·7 (74·7-80·7)	88·1 (84·8-91·7)
**Eastern Europe**																
Belarus	76·7 (68·5-84·4)	76·5 (71·0-81·7)	77·1 (70·8-82·6)	84·8 (67·5-97·5)	42·0 (40·1-43·8)	53·0 (50·8-55·0)	55·7 (53·5-57·8)	66·8 (61·5-72·6)	54·1 (51·5-56·3)	73·4 (68·7-78·3)	74·9 (70·0-80·1)	93·4 (86·5-100·0)	33·0 (29·3-36·7)	45·2 (39·6-50·8)	46·5 (40·8-52·2)	56·4 (44·9-65·4)
Estonia	76·9 (68·5-84·6)	81·6 (73·5-89·2)	81·8 (73·5-89·3)	86·3 (70·2-98·7)	44·4 (42·6-46·1)	65·5 (63·2-67·5)	69·0 (66·3-71·4)	100·0 (100·0-100·0)	57·2 (55·0-59·3)	81·9 (78·6-85·5)	82·8 (79·2-86·5)	94·6 (89·1-100·0)	46·7 (39·7-53·9)	77·3 (60·6-89·4)	79·7 (62·3-92·3)	98·9 (85·8-100·0)
Latvia	69·3 (60·0-77·5)	78·3 (69·4-86·1)	78·4 (69·6-86·4)	83·0 (66·9-95·6)	52·6 (50·6-54·6)	60·1 (58·0-62·0)	62·6 (60·4-64·5)	78·0 (74·5-81·4)	58·2 (55·8-60·3)	72·3 (68·2-76·1)	73·6 (69·4-77·6)	89·4 (82·7-95·6)	39·2 (35·0-43·4)	53·8 (43·7-60·4)	55·1 (44·7-61·8)	73·4 (59·3-82·0)
Lithuania	68·8 (58·9-77·5)	79·2 (69·9-86·8)	79·6 (70·4-87·0)	85·9 (67·6-96·9)	45·8 (44·0-47·6)	65·1 (62·8-67·2)	68·5 (65·9-70·8)	93·7 (89·1-97·9)	59·7 (57·5-61·6)	70·1 (67·4-72·7)	70·9 (68·1-73·6)	82·3 (78·3-86·5)	48·8 (44·4-52·4)	56·9 (52·8-60·9)	58·0 (53·9-62·0)	73·3 (68·5-77·8)
Moldova	64·1 (59·0-68·8)	61·2 (55·3-67·3)	62·0 (55·2-69·2)	63·6 (49·3-77·2)	35·6 (33·8-37·4)	44·9 (42·9-46·9)	46·7 (44·6-48·8)	54·4 (49·9-59·2)	46·7 (43·8-49·8)	61·9 (58·0-65·5)	63·4 (59·5-67·2)	81·2 (76·0-86·7)	17·4 (14·3-20·7)	41·6 (36·2-46·5)	42·8 (37·4-47·8)	60·2 (53·4-66·4)
Russia	68·9 (60·8-76·4)	74·2 (64·6-82·5)	74·6 (65·0-83·0)	79·7 (62·2-93·4)	41·1 (39·4-42·9)	47·4 (45·5-49·1)	50·1 (47·8-52·6)	69·4 (65·0-73·5)	42·0 (38·9-45·0)	60·8 (53·0-68·3)	61·4 (52·7-69·5)	73·1 (55·1-88·5)	37·6 (33·4-41·5)	49·0 (40·8-56·9)	50·0 (41·5-58·1)	63·7 (51·3-74·9)
Ukraine	60·7 (54·9-66·2)	70·9 (64·8-76·5)	71·3 (64·2-77·5)	75·7 (58·5-91·9)	37·9 (35·8-39·9)	46·2 (44·0-48·3)	48·7 (46·4-51·2)	60·4 (53·1-67·4)	46·9 (44·1-49·4)	60·5 (53·4-67·0)	61·2 (53·6-68·2)	70·9 (55·4-84·3)	29·7 (26·4-33·1)	41·3 (33·5-47·9)	42·1 (34·1-48·7)	52·6 (41·4-61·6)
**Central Europe**																
Albania	12·2 (8·0-17·2)	16·1 (9·3-23·6)	17·3 (10·2-25·2)	36·7 (14·7-62·2)	52·8 (50·0-55·4)	45·7 (41·3-50·3)	45·0 (40·4-49·7)	36·1 (25·0-46·8)	53·1 (50·6-55·5)	67·9 (63·5-72·2)	68·6 (64·2-73·2)	79·9 (73·9-86·1)	29·3 (26·6-32·3)	47·2 (42·1-52·2)	48·4 (43·2-53·6)	65·2 (58·1-72·6)
Bosnia and Herzegovina	24·4 (16·6-32·6)	18·1 (12·1-24·4)	18·8 (12·4-25·9)	18·9 (5·8-34·6)	52·0 (50·0-54·1)	71·8 (68·8-74·7)	75·2 (71·4-78·9)	99·2 (93·1-100·0)	54·4 (50·7-57·8)	65·1 (60·7-69·6)	65·8 (61·2-70·3)	74·5 (68·3-80·3)	25·4 (21·6-29·4)	39·6 (34·5-44·9)	40·3 (35·1-45·7)	49·7 (43·0-57·9)
Bulgaria	53·8 (44·0-63·8)	57·2 (46·8-67·6)	57·6 (47·4-68·2)	62·5 (42·2-82·0)	28·9 (27·6-30·4)	36·4 (34·7-38·0)	38·7 (35·9-41·2)	51·2 (43·9-56·7)	51·3 (49·1-53·4)	61·5 (57·3-66·0)	62·5 (57·9-67·3)	74·1 (64·8-82·9)	29·6 (26·3-32·9)	41·5 (36·9-46·1)	42·2 (37·6-46·9)	53·0 (47·1-58·7)
Croatia	55·7 (45·5-66·4)	51·6 (41·6-62·1)	51·8 (41·5-62·3)	53·4 (32·6-74·3)	56·4 (54·3-58·5)	70·0 (67·5-72·3)	72·5 (69·7-74·9)	89·6 (84·6-94·9)	63·8 (61·3-66·3)	78·5 (75·4-82·3)	79·3 (76·0-83·3)	89·7 (85·0-95·7)	43·6 (40·8-46·8)	55·8 (51·7-60·2)	56·3 (52·2-60·7)	67·0 (61·5-74·1)
Czech Republic	64·0 (54·7-72·9)	68·0 (57·8-77·7)	68·2 (58·2-77·8)	72·1 (51·8-88·9)	60·7 (58·3-62·8)	68·8 (67·6-69·7)	71·6 (69·0-73·7)	89·9 (82·3-95·0)	71·4 (69·5-73·2)	85·2 (83·0-87·6)	86·2 (84·0-88·7)	97·7 (94·4-100·0)	48·7 (46·2-51·5)	63·4 (60·7-66·3)	64·4 (61·7-67·4)	79·1 (76·1-82·5)
Hungary	66·9 (57·4-75·6)	66·4 (56·0-75·8)	66·7 (56·4-76·6)	70·0 (47·8-88·2)	46·5 (44·6-48·3)	52·1 (51·1-52·9)	54·0 (52·1-55·6)	63·7 (58·4-67·6)	60·3 (58·2-62·5)	74·1 (70·9-77·7)	74·6 (71·3-78·3)	84·6 (78·8-90·2)	38·1 (34·4-41·8)	48·4 (44·3-52·4)	48·9 (44·8-52·9)	58·2 (53·3-63·5)
Macedonia	11·3 (5·0-18·9)	11·6 (6·4-17·2)	12·7 (7·0-19·1)	13·5 (3·0-25·9)	40·5 (38·9-42·2)	54·7 (52·3-57·0)	56·1 (53·4-58·7)	69·7 (63·1-75·6)	49·5 (46·9-52·3)	62·4 (58·9-65·8)	63·0 (59·3-66·6)	72·6 (66·7-78·5)	25·0 (20·7-28·3)	38·3 (33·6-42·6)	39·5 (34·7-43·7)	51·0 (44·4-56·8)
Montenegro	43·3 (34·1-52·7)	36·7 (28·2-45·6)	37·0 (28·1-46·3)	36·5 (18·6-55·9)	52·1 (50·2-54·1)	62·5 (60·1-64·8)	63·4 (60·8-65·8)	76·1 (69·9-81·7)	56·7 (53·8-59·3)	73·0 (69·8-76·2)	73·7 (70·3-76·9)	83·5 (78·7-88·2)	34·6 (31·2-38·0)	41·3 (36·9-45·7)	41·7 (37·3-46·1)	47·3 (42·4-52·3)
Poland	44·3 (34·2-54·9)	49·3 (39·5-59·6)	49·8 (39·5-60·2)	58·2 (37·5-77·0)	53·6 (51·4-55·6)	60·9 (59·7-61·8)	62·5 (59·9-64·5)	75·5 (67·5-81·2)	60·7 (58·6-62·8)	77·4 (73·9-80·7)	78·4 (74·9-82·0)	91·8 (85·9-97·3)	42·1 (39·1-44·9)	56·8 (53·6-60·1)	58·0 (54·7-61·3)	73·9 (69·9-77·8)
Romania	45·1 (35·4-54·9)	51·7 (40·7-62·1)	52·1 (41·5-62·5)	58·8 (37·9-78·5)	35·1 (33·4-36·7)	37·0 (34·2-39·8)	38·7 (34·1-42·8)	47·8 (35·6-56·2)	53·6 (51·2-55·9)	66·9 (63·6-70·0)	67·8 (64·5-71·0)	80·0 (75·4-84·6)	29·1 (26·5-31·6)	46·7 (42·9-50·6)	47·9 (44·0-51·8)	64·2 (59·5-69·2)
Serbia	45·0 (36·3-54·4)	21·6 (15·4-28·3)	22·1 (15·6-29·5)	14·1 (3·8-26·2)	46·7 (44·7-48·8)	54·1 (52·6-55·5)	55·2 (53·8-56·4)	63·7 (59·6-67·3)	49·8 (46·7-52·5)	65·1 (62·6-67·9)	65·6 (63·0-68·3)	72·3 (68·6-76·0)	28·1 (24·3-31·5)	46·3 (41·6-49·9)	47·2 (42·6-50·9)	60·8 (55·9-64·8)
Slovakia	70·9 (61·9-79·3)	71·2 (61·2-79·9)	71·5 (61·4-80·2)	75·2 (56·3-90·9)	46·6 (44·9-48·3)	49·5 (48·4-50·3)	51·2 (49·3-52·7)	67·1 (58·3-72·5)	61·6 (58·8-64·4)	73·8 (70·0-77·6)	74·6 (70·8-78·7)	85·7 (79·9-92·0)	42·2 (39·4-45·3)	55·4 (51·6-59·1)	56·3 (52·5-60·1)	69·1 (64·9-73·4)
Slovenia	68·0 (58·8-76·3)	66·4 (57·1-75·4)	66·8 (57·1-76·1)	68·8 (49·0-85·3)	75·0 (72·3-77·6)	94·6 (91·0-97·7)	97·7 (93·8-100·0)	100·0 (100·0-100·0)	70·9 (68·3-73·7)	91·8 (88·1-95·4)	92·8 (88·9-96·7)	100·0 (100·0-100·0)	56·6 (53·3-59·8)	75·9 (71·7-80·0)	76·6 (72·5-80·8)	91·1 (84·9-100·0)
**Central Asia**																
Armenia	20·2 (17·4-23·0)	39·2 (29·6-49·7)	40·4 (30·1-51·3)	63·0 (38·1-86·4)	37·3 (35·3-39·3)	43·1 (40·6-45·6)	45·6 (42·8-48·3)	46·5 (40·7-52·3)	47·5 (44·6-50·4)	64·2 (60·3-67·8)	65·3 (61·4-69·1)	81·5 (76·1-86·9)	32·3 (28·4-36·3)	44·2 (35·8-50·8)	45·6 (36·9-52·3)	64·2 (52·0-73·5)

Location	Indicator 3.9.2: Age-standardised death rate attributable to unsafe water, sanitation, and hygiene (WaSH) (per 100,000 population)				Indicator 3.9.3: Age-standardised death rate due to unintentional poisonings (per 100,000 population)				Indicator 3.a.1: Age-standardised prevalence of daily smoking in populations aged 10 and older (%)				Indicator 3.b.1: Geometric mean of the coverage of eight vaccines, conditional on inclusion in national vaccine schedules, in target populations (%)			
	**2000**	**2015**	**2016**	**2030**	**2000**	**2015**	**2016**	**2030**	**2000**	**2015**	**2016**	**2030**	**2000**	**2015**	**2016**	**2030**
Sweden	92·2 (89·5-94·6)	84·6 (80·4-88·6)	84·7 (80·5-88·7)	86·2 (79·7-90·7)	65·4 (60·9-69·0)	67·1 (63·0-75·1)	67·8 (63·3-75·7)	67·7 (56·7-82·3)	55·4 (52·7-58·0)	76·8 (75·1-78·4)	76·9 (75·0-78·5)	87·9 (85·3-90·4)	95·1 (93·5-96·5)	96·0 (93·4-97·6)	96·2 (93·4-97·9)	98·1 (93·0-100·0)
Switzerland	96·6 (94·2-98·6)	95·3 (91·6-98·7)	95·5 (91·7-98·9)	98·0 (93·4-100·0)	92·7 (86·7-96·3)	99·9 (98·8-100·0)	99·8 (97·6-100·0)	99·9 (99·5-100·0)	16·5 (13·7-19·0)	44·2 (40·0-48·4)	44·4 (39·9-48·7)	49·3 (37·4-59·0)	82·6 (79·0-85·7)	85·1 (79·9-88·9)	85·3 (79·6-89·4)	94·8 (86·8-98·8)
United Kingdom	84·1 (82·3-85·7)	85·8 (83·4-87·6)	86·0 (83·6-87·8)	88·9 (87·2-90·4)	64·8 (61·3-75·9)	84·4 (79·9-86·4)	84·4 (80·1-86·7)	93·9 (87·6-98·0)	33·8 (31·2-36·4)	43·1 (39·5-46·6)	43·6 (40·0-47·1)	52·3 (46·1-58·7)	30·4 (0·0-70·8)	94·5 (91·8-96·5)	92·6 (89·9-94·6)	98·3 (94·4-100·0)
**Southern Latin America**																
Argentina	60·1 (57·7-62·3)	66·8 (64·2-69·4)	67·2 (64·6-69·9)	73·6 (70·5-76·7)	44·9 (38·8-48·7)	54·1 (48·1-57·8)	54·8 (48·7-58·6)	64·2 (55·5-71·5)	30·9 (22·8-38·9)	47·5 (43·0-52·0)	47·4 (42·4-52·5)	61·2 (52·5-69·6)	80·9 (67·6-87·3)	90·6 (82·7-94·2)	91·8 (84·8-95·3)	97·0 (87·2-100·0)
Chile	64·0 (61·4-66·5)	73·5 (69·2-77·6)	73·8 (69·3-78·0)	80·7 (73·2-87·2)	65·8 (61·3-75·7)	82·3 (77·2-87·8)	82·6 (75·1-89·9)	92·2 (76·9-100·0)	3·0 (0·0-9·1)	19·8 (11·4-27·5)	20·5 (11·7-28·5)	32·2 (16·5-45·2)	91·0 (88·3-93·4)	90·7 (86·7-93·7)	91·6 (87·2-94·6)	92·2 (81·7-97·4)
Uruguay	64·5 (61·3-67·7)	69·4 (65·2-73·3)	69·8 (65·5-73·8)	75·5 (69·8-80·2)	54·3 (49·4-59·8)	65·3 (60·6-69·4)	65·9 (61·0-70·2)	74·2 (64·5-83·1)	14·6 (8·5-20·5)	37·8 (30·0-45·0)	37·7 (29·4-45·5)	46·7 (33·1-59·8)	92·1 (89·0-94·3)	95·4 (92·0-97·5)	95·6 (91·8-97·6)	97·9 (91·0-100·0)
**Eastern Europe**																
Belarus	75·6 (71·5-79·3)	95·6 (90·3-100·0)	96·2 (91·0-100·0)	100·0 (100·0-100·0)	9·4 (3·0-16·0)	22·7 (14·5-32·0)	23·4 (14·5-32·9)	37·0 (20·2-53·8)	19·8 (12·9-26·3)	19·2 (11·7-27·0)	19·9 (12·0-27·6)	20·9 (6·7-34·0)	95·9 (89·2-98·5)	98·3 (88·5-100·0)	98·6 (90·4-100·0)	99·3 (97·2-100·0)
Estonia	84·0 (79·1-88·2)	96·3 (91·6-100·0)	96·7 (91·9-100·0)	99·8 (97·8-100·0)	21·7 (14·9-31·3)	59·0 (41·1-65·0)	58·9 (41·8-65·5)	91·8 (58·9-100·0)	17·9 (14·2-21·4)	36·9 (32·1-41·5)	37·0 (31·5-42·4)	44·9 (32·5-57·2)	91·0 (88·2-93·3)	92·6 (86·2-95·7)	92·6 (86·6-95·9)	96·6 (82·8-100·0)
Latvia	82·1 (77·4-86·3)	91·4 (85·8-96·2)	91·9 (86·2-96·7)	97·9 (92·3-100·0)	12·6 (6·8-27·2)	43·9 (32·0-49·7)	43·6 (32·5-50·2)	72·2 (42·6-86·9)	11·2 (8·0-14·6)	11·5 (6·6-16·5)	11·9 (6·4-17·6)	10·6 (0·0-21·0)	87·3 (80·9-91·7)	90·8 (85·7-94·1)	91·8 (86·9-95·0)	94·4 (79·5-100·0)
Lithuania	86·2 (81·3-90·5)	88·6 (83·9-92·8)	88·8 (84·1-93·0)	92·1 (87·2-96·3)	20·7 (14·7-30·6)	50·4 (33·6-55·5)	51·2 (34·0-56·5)	66·8 (42·5-79·7)	26·8 (23·7-30·1)	27·5 (21·1-33·8)	27·6 (21·1-34·4)	27·2 (12·6-40·8)	94·2 (92·5-95·7)	92·2 (85·5-95·6)	92·3 (86·7-95·8)	94·9 (80·0-100·0)
Moldova	59·3 (54·6-63·9)	74·1 (69·1-78·9)	75·1 (70·1-80·0)	90·2 (83·3-96·7)	0·4 (0·0-6·6)	37·7 (11·3-44·7)	37·1 (11·9-44·4)	72·9 (26·1-90·5)	39·4 (33·8-45·2)	46·2 (42·0-50·5)	46·5 (41·8-51·2)	52·3 (42·7-60·7)	92·6 (89·3-95·0)	84·5 (70·8-91·3)	85·8 (74·6-91·4)	62·7 (21·2-85·6)
Russia	68·3 (64·7-72·0)	81·5 (75·8-86·8)	81·9 (76·1-87·4)	88·4 (80·4-96·1)	14·4 (5·6-19·2)	27·8 (18·7-34·1)	28·5 (18·2-36·8)	40·2 (20·8-58·4)	0·3 (0·0-2·2)	15·9 (11·6-20·0)	16·8 (11·5-21·5)	23·2 (11·0-35·2)	75·9 (71·3-80·2)	74·6 (59·8-82·6)	74·2 (58·1-83·1)	75·9 (44·2-92·0)
Ukraine	74·0 (69·5-78·5)	87·4 (81·3-92·7)	87·9 (81·6-93·3)	95·3 (86·2-100·0)	8·0 (0·8-14·0)	20·3 (12·7-27·3)	20·4 (11·3-29·7)	29·1 (9·6-50·9)	11·1 (7·0-15·0)	13·5 (6·7-20·5)	13·6 (6·1-21·3)	13·0 (0·0-30·9)	94·4 (91·7-96·3)	41·6 (19·1-58·9)	39·4 (16·1-58·7)	16·6 (0·0-72·5)
**Central Europe**																
Albania	67·9 (63·0-72·2)	85·4 (79·8-90·9)	86·1 (80·4-91·6)	95·1 (88·0-100·0)	39·1 (34·8-47·9)	63·2 (51·9-69·6)	63·7 (52·1-70·1)	73·1 (51·9-87·6)	52·2 (49·1-55·2)	52·2 (47·8-56·7)	52·5 (48·2-57·1)	53·9 (44·3-63·3)	97·2 (95·4-98·3)	99·8 (99·3-100·0)	99·8 (99·4-100·0)	100·0 (99·9-100·0)
Bosnia and Herzegovina	87·2 (83·0-91·1)	95·0 (90·7-99·4)	95·3 (90·9-99·8)	99·8 (97·7-100·0)	33·9 (28·7-38·8)	60·5 (41·9-68·3)	60·8 (42·1-68·6)	80·2 (53·6-97·7)	7·4 (3·1-11·6)	0·8 (0·0-5·0)	1·5 (0·0-6·5)	0·2 (0·0-3·8)	90·2 (87·9-92·1)	93·6 (90·0-96·2)	94·1 (90·2-96·7)	97·8 (91·6-100·0)
Bulgaria	79·8 (75·4-84·0)	88·3 (83·3-92·8)	88·9 (83·8-93·5)	96·5 (90·4-100·0)	31·0 (26·6-38·1)	57·2 (43·7-62·2)	57·3 (43·9-63·1)	82·0 (56·4-92·5)	0·0 (0·0-0·0)	0·9 (0·0-5·3)	1·8 (0·0-7·3)	5·3 (0·0-16·9)	91·5 (88·6-93·9)	91·9 (87·6-94·9)	93·0 (88·7-95·9)	92·8 (82·0-97·9)
Croatia	86·5 (81·6-90·8)	92·2 (88·4-96·6)	92·4 (88·5-96·8)	97·8 (93·7-100·0)	74·1 (62·9-78·1)	90·8 (72·8-95·9)	90·8 (72·9-96·3)	98·5 (80·0-100·0)	16·9 (12·2-21·4)	8·8 (2·7-15·0)	10·0 (3·5-16·7)	5·1 (0·0-18·4)	91·9 (89·7-93·8)	94·6 (91·7-96·5)	95·0 (92·2-97·0)	97·0 (90·2-99·7)
Czech Republic	92·9 (87·7-97·8)	80·0 (76·0-85·9)	79·5 (75·5-85·5)	73·4 (68·2-80·8)	63·5 (54·0-67·0)	78·6 (65·0-82·5)	78·6 (65·6-82·8)	94·5 (74·3-100·0)	17·6 (13·3-22·0)	28·1 (22·3-33·6)	29·0 (22·7-34·9)	33·0 (20·6-44·5)	96·2 (94·5-97·5)	98·2 (96·0-99·3)	98·4 (96·3-99·5)	99·5 (96·7-100·0)
Hungary	95·3 (91·9-98·8)	79·7 (75·3-86·3)	78·9 (74·4-85·7)	67·7 (61·6-76·5)	68·9 (55·6-73·2)	77·9 (68·2-82·1)	78·2 (67·6-83·0)	88·7 (76·8-99·4)	7·6 (1·7-13·0)	18·8 (13·0-24·7)	19·7 (13·6-25·9)	31·4 (19·3-43·0)	98·3 (97·1-99·1)	97·9 (96·7-98·8)	98·3 (97·3-99·1)	99·2 (97·1-100·0)
Macedonia	69·3 (64·7-74·8)	88·7 (82·6-94·5)	89·0 (82·7-94·9)	99·5 (94·7-100·0)	54·4 (48·1-58·4)	62·2 (56·7-72·0)	62·5 (57·0-72·2)	69·9 (60·2-81·8)	14·2 (6·2-21·5)	2·9 (0·0-8·9)	3·9 (0·0-10·6)	0·6 (0·0-6·6)	88·4 (85·7-90·7)	89·6 (85·1-92·8)	89·4 (84·2-93·0)	93·6 (79·6-99·9)
Montenegro	87·8 (83·6-91·7)	98·7 (94·6-100·0)	98·9 (95·0-100·0)	100·0 (100·0-100·0)	36·0 (32·1-40·7)	52·9 (44·3-59·1)	53·0 (43·2-59·4)	64·5 (48·3-76·9)	11·9 (3·8-20·3)	0·0 (0·0-0·0)	0·0 (0·0-0·0)	0·0 (0·0-0·0)	87·8 (81·1-92·8)	91·7 (87·8-94·3)	92·0 (87·5-94·8)	94·7 (82·9-99·6)
Poland	88·4 (82·9-93·3)	89·4 (84·7-93·4)	89·5 (84·8-93·5)	91·0 (86·2-95·4)	65·8 (60·5-73·6)	82·8 (78·4-86·3)	83·3 (78·7-87·2)	98·7 (90·8-100·0)	2·6 (0·0-8·7)	27·5 (22·4-32·5)	28·3 (22·5-33·7)	44·8 (34·7-54·0)	94·8 (92·8-96·4)	95·8 (93·8-97·3)	96·2 (94·2-97·6)	98·6 (94·9-100·0)
Romania	63·6 (59·9-66·9)	80·4 (75·6-84·6)	81·1 (76·2-85·3)	91·7 (84·8-98·0)	41·6 (36·4-51·7)	66·0 (57·2-69·6)	66·2 (57·3-70·4)	85·5 (68·8-94·4)	22·6 (17·2-27·8)	29·3 (23·9-34·3)	30·1 (24·4-35·7)	30·9 (18·2-42·5)	97·1 (95·3-98·4)	92·6 (87·6-95·8)	93·0 (87·8-96·2)	87·6 (64·2-98·6)
Serbia	87·9 (83·0-92·0)	92·0 (85·0-97·6)	92·1 (84·7-97·8)	94·0 (81·4-100·0)	50·0 (45·7-58·0)	68·2 (60·8-72·6)	67·8 (60·5-72·4)	84·4 (69·0-93·7)	4·7 (0·1-9·4)	9·0 (1·1-17·2)	9·7 (1·7-18·1)	5·8 (0·0-21·5)	86·4 (81·3-90·4)	93·6 (90·0-96·2)	93·7 (89·8-96·5)	97·8 (90·6-100·0)
Slovakia	83·0 (78·2-87·7)	87·3 (82·2-92·2)	87·6 (82·4-92·5)	91·8 (85·9-97·7)	48·3 (44·6-54·3)	67·6 (57·2-72·7)	68·0 (57·1-73·8)	81·6 (61·5-96·2)	34·2 (28·4-39·5)	43·4 (38·4-48·1)	43·7 (38·4-48·8)	53·5 (43·7-62·3)	96·6 (94·5-97·9)	97·7 (95·6-99·0)	97·9 (95·9-99·1)	99·0 (95·5-100·0)
Slovenia	87·7 (82·5-91·9)	95·8 (91·0-100·0)	96·0 (91·2-100·0)	99·0 (95·1-100·0)	53·0 (47·9-63·5)	76·5 (69·6-80·8)	76·8 (69·9-81·4)	92·4 (76·9-100·0)	34·7 (28·9-40·7)	34·6 (29·1-39·9)	35·3 (29·3-40·9)	40·8 (27·2-52·4)	91·2 (88·4-93·3)	94·6 (91·6-96·8)	94·7 (91·5-97·0)	96·7 (89·5-99·8)
**Central Asia**																
Armenia	53·6 (49·7-57·5)	77·3 (72·6-81·6)	78·2 (73·5-82·5)	91·6 (85·9-98·8)	33·7 (26·0-38·2)	47·2 (38·2-51·4)	47·5 (38·6-52·7)	59·1 (45·1-71·1)	23·7 (18·0-29·4)	33·1 (26·4-40·0)	34·1 (27·1-41·3)	41·9 (30·5-53·2)	84·8 (80·0-88·4)	93·2 (89·0-96·0)	94·1 (90·4-96·7)	99·1 (96·5-100·0)

Location	Indicator 5.2.1:<br>Age-standardised prevalence of women aged 15 years and older who experienced physical or sexual violence by an intimate partner in the last 12 months (%)				Indicator 6.1.1:<br>Risk-weighted prevalence of populations using unsafe or unimproved water sources, as measured by the summary exposure value (SEV) for unsafe water (%)				Indicator 6.2.1a:<br>Risk-weighted prevalence of populations using unsafe or unimproved sanitation, as measured by the summary exposure value (SEV) for unsafe sanitation (%)				Indicator 6.2.1b:<br>Risk-weighted prevalence of populations without access to a handwashing facility, as measured by the summary exposure value (SEV) for unsafe hygiene (%)			
	**2000**	**2015**	**2016**	**2030**	**2000**	**2015**	**2016**	**2030**	**2000**	**2015**	**2016**	**2030**	**2000**	**2015**	**2016**	**2030**
Sweden	93·5<br>(92·5-94·4)	95·1<br>(94·2-95·9)	95·2<br>(94·3-96·0)	96·8<br>(95·7-97·9)	99·7<br>(99·3-100·0)	99·8<br>(99·6-100·0)	99·8<br>(99·6-100·0)	99·9<br>(99·7-100·0)	98·5<br>(96·4-99·9)	99·2<br>(97·7-100·0)	99·2<br>(97·8-100·0)	99·6<br>(98·7-100·0)	99·5<br>(99·3-99·8)	99·6<br>(99·4-99·8)	99·6<br>(99·4-99·8)	99·8<br>(99·5-100·0)
Switzerland	95·0<br>(93·5-96·2)	98·7<br>(97·9-99·5)	98·9<br>(98·1-99·6)	100·0<br>(100·0-100·0)	99·9<br>(99·6-100·0)	99·9<br>(99·6-100·0)	99·9<br>(99·6-100·0)	99·8<br>(98·6-100·0)	98·9<br>(96·9-100·0)	99·5<br>(98·3-100·0)	99·6<br>(98·4-100·0)	99·9<br>(99·2-100·0)	99·6<br>(99·3-99·9)	99·8<br>(99·5-100·0)	99·8<br>(99·5-100·0)	99·9<br>(99·6-100·0)
United Kingdom	94·3<br>(93·6-94·9)	97·2<br>(96·7-97·7)	97·4<br>(96·8-97·9)	99·8<br>(99·0-100·0)	99·2<br>(99·0-99·3)	99·8<br>(99·6-99·9)	99·8<br>(99·7-99·9)	100·0<br>(100·0-100·0)	100·0<br>(100·0-100·0)	100·0<br>(100·0-100·0)	100·0<br>(100·0-100·0)	100·0<br>(100·0-100·0)	99·4<br>(99·2-99·5)	99·6<br>(99·4-99·7)	99·6<br>(99·4-99·7)	99·8<br>(99·6-99·9)
**Southern Latin America**																
Argentina	72·4<br>(68·0-75·5)	78·0<br>(74·4-80·7)	78·4<br>(74·9-81·1)	83·6<br>(80·7-85·9)	77·1<br>(71·4-83·0)	89·9<br>(84·3-94·0)	90·3<br>(84·7-94·4)	94·8<br>(89·4-97·9)	81·1<br>(72·6-87·6)	94·9<br>(90·0-98·0)	95·3<br>(90·6-98·2)	99·3<br>(97·4-100·0)	90·3<br>(89·7-91·0)	94·4<br>(93·9-94·9)	94·6<br>(94·1-95·1)	97·1<br>(96·4-98·2)
Chile	76·2<br>(72·3-79·6)	84·4<br>(81·8-86·8)	84·9<br>(82·4-87·2)	91·7<br>(89·9-93·3)	88·9<br>(82·8-93·5)	94·0<br>(89·5-96·8)	94·3<br>(89·9-96·9)	97·1<br>(94·7-98·5)	84·2<br>(75·2-90·8)	93·8<br>(87·3-97·6)	94·1<br>(87·9-97·7)	97·8<br>(94·4-99·8)	94·0<br>(93·5-94·5)	95·7<br>(95·3-96·2)	95·8<br>(95·4-96·2)	97·0<br>(96·5-97·7)
Uruguay	78·5<br>(75·5-81·0)	83·0<br>(80·4-85·1)	83·4<br>(80·8-85·4)	87·7<br>(85·7-89·4)	90·2<br>(86·3-93·4)	94·0<br>(90·9-95·9)	94·3<br>(91·1-96·2)	97·2<br>(94·4-98·4)	92·3<br>(87·2-96·0)	98·3<br>(96·0-99·8)	98·5<br>(96·3-99·9)	99·9<br>(99·3-100·0)	94·0<br>(93·4-94·5)	95·4<br>(94·9-95·8)	95·5<br>(95·0-95·9)	96·9<br>(96·5-97·3)
**Eastern Europe**																
Belarus	75·7<br>(73·3-78·0)	84·6<br>(82·4-86·4)	85·3<br>(83·0-87·1)	93·5<br>(90·6-95·7)	77·0<br>(73·6-81·3)	87·5<br>(85·0-90·0)	87·6<br>(85·1-90·2)	89·1<br>(86·3-92·2)	70·5<br>(55·9-82·5)	80·2<br>(65·6-90·7)	80·3<br>(65·2-91·0)	82·5<br>(60·7-94·0)	90·5<br>(82·9-96·1)	95·7<br>(90·4-99·1)	95·7<br>(90·4-99·2)	98·1<br>(93·5-100·0)
Estonia	84·2<br>(82·4-85·7)	93·0<br>(91·6-94·2)	93·4<br>(92·0-94·6)	99·0<br>(97·4-100·0)	81·8<br>(78·4-85·4)	85·0<br>(81·9-88·4)	85·2<br>(82·1-88·5)	86·8<br>(83·8-90·0)	84·5<br>(69·6-93·7)	82·6<br>(62·7-93·7)	82·5<br>(62·3-93·7)	81·9<br>(57·8-94·4)	94·9<br>(89·6-98·5)	96·7<br>(92·4-99·6)	96·7<br>(92·5-99·7)	97·8<br>(94·3-100·0)
Latvia	79·5<br>(77·6-81·3)	88·5<br>(86·9-89·8)	89·0<br>(87·3-90·3)	95·1<br>(93·1-97·0)	80·2<br>(76·8-83·9)	84·0<br>(81·0-87·3)	84·1<br>(81·1-87·4)	85·2<br>(82·4-88·5)	78·4<br>(61·1-89·9)	80·1<br>(57·8-93·0)	80·0<br>(57·3-93·0)	80·1<br>(55·0-94·3)	94·0<br>(87·6-98·2)	96·1<br>(91·7-99·4)	96·1<br>(91·8-99·4)	97·5<br>(93·4-100·0)
Lithuania	82·6<br>(80·6-84·2)	90·7<br>(89·4-92·0)	91·2<br>(89·9-92·5)	97·6<br>(96·2-99·0)	80·8<br>(77·6-84·2)	84·4<br>(81·1-87·7)	84·5<br>(81·2-87·9)	86·2<br>(83·1-89·9)	77·7<br>(47·8-93·1)	80·5<br>(58·9-93·1)	80·6<br>(59·1-93·1)	82·7<br>(63·8-93·8)	94·0<br>(88·2-97·9)	96·0<br>(91·2-99·5)	96·1<br>(91·3-99·6)	97·5<br>(93·5-100·0)
Moldova	64·1<br>(61·2-66·7)	73·1<br>(70·3-75·5)	73·9<br>(71·2-76·4)	83·9<br>(79·8-87·2)	46·9<br>(42·7-52·3)	60·6<br>(54·9-66·7)	61·8<br>(56·1-67·8)	76·4<br>(70·3-81·1)	33·6<br>(21·4-48·8)	45·5<br>(23·5-67·7)	46·4<br>(24·1-69·1)	57·4<br>(30·9-84·8)	76·8<br>(65·5-85·9)	85·6<br>(79·1-91·0)	86·3<br>(79·8-91·5)	93·3<br>(85·4-96·9)
Russia	71·5<br>(68·7-73·9)	80·7<br>(78·0-82·8)	81·3<br>(78·5-83·4)	88·2<br>(84·4-91·3)	79·3<br>(75·9-82·9)	82·9<br>(79·7-86·6)	83·0<br>(79·8-86·7)	84·4<br>(81·0-88·4)	63·1<br>(46·4-79·7)	72·4<br>(44·9-90·0)	72·6<br>(44·4-90·2)	76·5<br>(36·7-93·5)	92·7<br>(86·5-97·6)	95·0<br>(90·0-98·7)	95·1<br>(90·1-98·7)	96·1<br>(91·4-99·4)
Ukraine	72·1<br>(69·8-74·2)	79·8<br>(77·4-81·7)	80·1<br>(77·6-82·0)	83·8<br>(80·0-86·6)	76·0<br>(72·7-79·5)	78·3<br>(74·7-81·7)	78·6<br>(74·8-82·0)	81·9<br>(77·5-85·2)	71·1<br>(56·7-83·5)	66·3<br>(45·2-82·6)	66·7<br>(44·7-83·4)	57·4<br>(8·9-88·7)	89·9<br>(81·7-95·5)	91·5<br>(84·7-96·2)	91·6<br>(84·9-96·2)	93·4<br>(87·0-97·0)
**Central Europe**																
Albania	71·1<br>(68·2-73·7)	84·3<br>(82·2-86·3)	84·7<br>(82·6-86·7)	91·0<br>(88·3-93·3)	61·3<br>(49·4-69·7)	72·2<br>(58·7-80·1)	72·8<br>(59·2-80·6)	80·2<br>(57·0-93·2)	66·5<br>(54·2-77·4)	82·3<br>(63·5-94·5)	82·9<br>(63·1-95·3)	90·1<br>(62·9-100·0)	90·8<br>(82·9-96·4)	94·7<br>(89·1-98·6)	94·9<br>(89·4-98·7)	97·0<br>(93·1-99·9)
Bosnia and Herzegovina	83·9<br>(81·6-86·0)	86·0<br>(83·8-87·8)	86·6<br>(84·4-88·3)	93·4<br>(91·0-95·2)	80·2<br>(64·2-84·2)	84·2<br>(75·2-86·8)	84·4<br>(75·6-87·0)	86·4<br>(80·6-88·7)	81·2<br>(74·5-86·5)	92·6<br>(81·7-98·0)	92·9<br>(81·4-98·2)	97·6<br>(87·2-100·0)	97·8<br>(94·4-100·0)	98·9<br>(96·8-100·0)	98·9<br>(96·8-100·0)	99·5<br>(97·8-100·0)
Bulgaria	81·8<br>(80·0-83·2)	89·7<br>(88·3-90·9)	90·1<br>(88·7-91·3)	95·6<br>(93·8-97·1)	82·9<br>(75·2-86·2)	85·9<br>(83·8-87·6)	86·0<br>(83·9-87·7)	87·5<br>(85·6-89·1)	96·5<br>(89·3-99·6)	95·6<br>(87·3-99·5)	95·6<br>(87·1-99·5)	94·8<br>(84·4-99·5)	97·2<br>(93·3-100·0)	98·4<br>(95·4-100·0)	98·5<br>(95·5-100·0)	99·2<br>(96·9-100·0)
Croatia	90·2<br>(88·8-91·5)	96·1<br>(95·0-97·1)	96·4<br>(95·3-97·3)	99·9<br>(99·2-100·0)	84·5<br>(78·9-86·7)	86·1<br>(82·0-88·0)	86·2<br>(82·0-88·1)	86·8<br>(81·6-88·8)	94·0<br>(82·1-98·9)	94·6<br>(85·0-99·0)	94·6<br>(85·2-99·0)	94·6<br>(86·5-98·9)	97·8<br>(94·1-100·0)	98·7<br>(95·8-100·0)	98·7<br>(95·9-100·0)	99·2<br>(97·0-100·0)
Czech Republic	89·2<br>(87·7-90·5)	94·3<br>(93·0-95·5)	94·7<br>(93·3-95·8)	99·3<br>(97·7-100·0)	86·8<br>(85·0-88·4)	87·8<br>(86·4-89·3)	87·8<br>(86·4-89·3)	88·4<br>(87·0-89·9)	97·5<br>(92·3-99·9)	96·0<br>(88·1-99·6)	95·9<br>(87·8-99·6)	93·7<br>(80·0-99·4)	98·9<br>(96·0-100·0)	99·3<br>(96·7-100·0)	99·3<br>(96·7-100·0)	99·5<br>(97·4-100·0)
Hungary	83·8<br>(81·9-85·2)	90·5<br>(89·1-91·8)	90·9<br>(89·5-92·1)	95·9<br>(94·4-97·3)	85·1<br>(81·8-87·0)	86·9<br>(85·4-88·5)	87·0<br>(85·4-88·5)	87·8<br>(86·1-89·3)	86·2<br>(74·4-94·4)	90·9<br>(74·3-98·3)	91·0<br>(74·4-98·3)	93·7<br>(79·1-99·7)	98·5<br>(95·5-100·0)	99·1<br>(96·5-100·0)	99·1<br>(96·5-100·0)	99·3<br>(96·9-100·0)
Macedonia	78·9<br>(76·1-81·3)	85·9<br>(83·7-87·8)	86·3<br>(84·2-88·2)	91·8<br>(89·9-93·5)	86·2<br>(75·1-90·3)	88·1<br>(80·7-90·4)	88·2<br>(80·9-90·5)	89·5<br>(84·4-91·3)	85·9<br>(66·7-96·3)	90·3<br>(78·6-97·1)	90·4<br>(78·3-97·2)	93·6<br>(80·9-99·1)	97·6<br>(93·6-100·0)	98·3<br>(95·0-100·0)	98·3<br>(95·1-100·0)	98·8<br>(96·1-100·0)
Montenegro	78·2<br>(75·7-80·4)	84·8<br>(82·8-86·4)	85·1<br>(83·2-86·8)	90·0<br>(88·2-91·6)	76·2<br>(62·5-84·9)	83·5<br>(75·0-87·0)	83·8<br>(75·3-87·1)	87·6<br>(81·1-90·1)	88·6<br>(72·9-97·3)	95·0<br>(85·5-99·3)	95·2<br>(85·7-99·4)	97·1<br>(89·0-100·0)	95·4<br>(90·4-99·1)	97·5<br>(93·9-100·0)	97·6<br>(94·1-100·0)	98·8<br>(96·3-100·0)
Poland	87·3<br>(85·5-88·8)	94·8<br>(93·3-96·0)	95·2<br>(93·8-96·5)	100·0<br>(99·5-100·0)	85·0<br>(82·5-86·9)	87·0<br>(85·5-88·8)	87·1<br>(85·6-88·9)	88·6<br>(87·2-90·3)	90·2<br>(72·0-98·4)	93·5<br>(81·4-98·8)	93·6<br>(81·7-98·8)	95·7<br>(88·1-99·3)	97·8<br>(94·2-100·0)	98·7<br>(95·6-100·0)	98·8<br>(95·6-100·0)	99·3<br>(96·7-100·0)
Romania	80·0<br>(78·0-81·7)	89·2<br>(87·6-90·6)	89·7<br>(88·2-91·1)	96·8<br>(95·1-98·5)	71·1<br>(60·9-79·4)	83·4<br>(74·9-86·9)	83·8<br>(75·3-87·0)	88·1<br>(74·1-96·3)	50·3<br>(37·1-63·0)	74·4<br>(45·5-93·4)	75·5<br>(46·0-94·2)	87·9<br>(55·9-99·7)	93·7<br>(87·7-97·7)	97·3<br>(93·1-100·0)	97·4<br>(93·3-100·0)	99·3<br>(96·7-100·0)
Serbia	80·1<br>(77·5-82·3)	86·5<br>(84·7-88·2)	86·7<br>(84·9-88·4)	90·4<br>(88·6-91·9)	67·5<br>(54·3-78·0)	80·6<br>(71·2-84·5)	81·0<br>(71·9-84·8)	86·9<br>(79·6-90·1)	85·8<br>(69·2-95·5)	93·1<br>(83·2-98·0)	93·3<br>(83·0-98·1)	96·8<br>(86·7-99·9)	97·3<br>(94·0-99·8)	99·4<br>(97·5-100·0)	99·4<br>(97·5-100·0)	99·9<br>(98·7-100·0)
Slovakia	81·9<br>(79·7-83·6)	88·4<br>(86·6-89·8)	88·8<br>(87·1-90·3)	95·1<br>(93·2-96·7)	85·6<br>(81·3-87·7)	87·4<br>(86·0-89·0)	87·5<br>(86·0-89·0)	88·4<br>(86·8-90·0)	95·4<br>(86·4-99·4)	95·3<br>(86·4-99·4)	95·4<br>(86·5-99·4)	95·4<br>(86·0-99·7)	98·7<br>(95·7-100·0)	99·2<br>(96·7-100·0)	99·3<br>(96·7-100·0)	99·6<br>(97·5-100·0)
Slovenia	91·6<br>(90·2-92·8)	97·9<br>(96·8-98·9)	98·3<br>(97·2-99·2)	100·0<br>(100·0-100·0)	85·8<br>(79·2-88·2)	87·6<br>(85·9-89·0)	87·6<br>(85·9-89·1)	88·2<br>(76·7-95·1)	94·4<br>(84·1-99·1)	95·0<br>(86·7-99·1)	95·0<br>(86·7-99·1)	95·2<br>(87·0-99·3)	99·1<br>(96·5-100·0)	99·4<br>(97·3-100·0)	99·4<br>(97·3-100·0)	99·6<br>(97·7-100·0)
**Central Asia**																
Armenia	75·8<br>(73·0-78·2)	83·7<br>(81·4-85·7)	84·5<br>(82·2-86·5)	90·6<br>(88·5-92·9)	76·8<br>(44·0-87·9)	86·9<br>(61·3-93·9)	87·2<br>(61·3-94·1)	91·4<br>(62·3-96·9)	66·0<br>(52·6-80·2)	76·5<br>(54·9-91·1)	76·8<br>(54·2-91·7)	81·6<br>(46·9-97·2)	85·5<br>(80·0-90·3)	89·1<br>(83·1-93·7)	89·3<br>(83·2-94·0)	91·7<br>(84·4-96·4)

Location	Indicator 7.1.2: Risk-weighted prevalence of household air pollution, as measured by the summary exposure value (SEV) for household air pollution (%)				Indicator 8.8.1: Age-standardised all-cause disability-adjusted life year (DALY) rates attributable to occupational risks (per 100,000 population)				Indicator 11.6.2: Population-weighted mean levels of fine particulate matter smaller than 2.5 microns in diameter (PM2.5)				Indicator 16.1.1: Age-standardised death rate due to interpersonal violence (per 100,000 population)			
	**2000**	**2015**	**2016**	**2030**	**2000**	**2015**	**2016**	**2030**	**2000**	**2015**	**2016**	**2030**	**2000**	**2015**	**2016**	**2030**
Sweden	99·1 (98·9-99·3)	99·3 (99·2-99·5)	99·3 (99·2-99·5)	99·5 (99·4-99·6)	83·3 (77·3-88·6)	88·1 (80·8-94·7)	88·4 (81·1-95·0)	92·5 (84·2-99·8)	97·6 (96·1-99·2)	100·0 (100·0-100·0)	100·0 (100·0-100·0)	100·0 (100·0-100·0)	79·7 (75·6-87·9)	83·9 (78·5-92·6)	84·3 (78·6-92·6)	89·0 (79·4-99·4)
Switzerland	99·0 (98·8-99·3)	99·4 (99·3-99·6)	99·5 (99·3-99·6)	99·8 (99·7-99·8)	54·7 (45·7-62·6)	63·9 (54·0-73·9)	65·6 (55·6-76·0)	89·8 (76·7-100·0)	76·0 (74·5-77·5)	77·9 (76·3-79·5)	78·2 (76·7-79·7)	80·7 (78·7-83·9)	80·6 (75·6-85·7)	93·6 (86·3-98·4)	93·6 (85·9-99·9)	99·0 (90·6-100·0)
United Kingdom	99·2 (99·1-99·3)	99·7 (99·6-99·7)	99·7 (99·7-99·7)	99·9 (99·8-99·9)	62·9 (57·1-68·5)	72·9 (67·3-77·8)	73·0 (67·4-77·9)	74·8 (68·8-79·9)	71·8 (70·6-73·0)	75·0 (73·8-76·3)	75·1 (74·0-76·3)	80·0 (78·8-81·4)	89·6 (85·8-97·5)	97·0 (93·2-100·0)	97·0 (93·0-100·0)	99·9 (98·8-100·0)
**Southern Latin America**																
Argentina	96·8 (95·9-97·5)	98·7 (98·4-99·1)	98·8 (98·5-99·1)	99·5 (99·4-99·7)	37·0 (27·3-46·4)	43·5 (30·9-54·8)	43·9 (31·3-55·3)	50·0 (34·9-62·3)	64·9 (63·2-66·5)	68·2 (66·6-69·8)	68·2 (66·6-69·7)	70·9 (69·0-72·9)	40·3 (33·6-46·1)	41·7 (35·0-48·4)	42·3 (35·5-49·0)	40·4 (30·7-51·1)
Chile	92·2 (90·2-93·9)	97·5 (96·8-98·2)	97·7 (97·0-98·3)	99·2 (98·9-99·4)	77·8 (68·5-87·5)	75·9 (64·8-86·0)	76·3 (65·1-86·5)	81·8 (69·2-93·7)	53·3 (51·2-55·3)	53·3 (51·5-55·2)	53·3 (51·4-55·1)	50·5 (48·5-52·5)	46·3 (37·5-51·9)	50·9 (41·1-58·3)	51·2 (40·4-60·1)	55·6 (37·7-72·0)
Uruguay	98·2 (97·7-98·6)	98·9 (98·6-99·2)	99·0 (98·7-99·3)	99·6 (99·4-99·7)	61·0 (51·1-69·8)	55·5 (45·8-63·4)	55·9 (46·2-63·9)	62·4 (52·0-70·6)	70·2 (67·3-72·9)	75·1 (72·4-77·8)	75·1 (72·4-77·9)	76·0 (72·8-79·0)	47·7 (42·5-55·3)	45·9 (39·3-56·5)	46·2 (39·7-56·7)	43·6 (31·7-58·3)
**Eastern Europe**																
Belarus	97·8 (93·6-99·5)	99·4 (98·0-99·9)	99·4 (98·1-99·9)	99·8 (99·1-100·0)	44·7 (37·3-51·7)	64·6 (54·3-73·7)	66·5 (56·1-75·7)	93·2 (80·7-100·0)	56·0 (51·2-59·6)	57·0 (52·1-61·0)	56·9 (51·4-61·2)	63·6 (56·6-71·6)	26·1 (21·4-34·9)	41·0 (30·5-46·3)	41·0 (30·2-47·5)	53·4 (33·6-66·9)
Estonia	90·2 (81·7-95·6)	93·4 (80·3-99·0)	93·7 (80·6-99·1)	96·5 (84·5-99·9)	68·0 (61·6-73·8)	88·8 (80·1-96·2)	89·3 (80·4-96·8)	95·4 (85·2-100·0)	87·2 (84·4-89·9)	96·9 (94·0-100·0)	97·5 (94·7-100·0)	100·0 (100·0-100·0)	23·8 (19·1-32·7)	48·0 (35·0-54·2)	47·9 (35·1-54·8)	64·7 (39·2-83·3)
Latvia	93·4 (88·3-96·8)	95·2 (83·5-99·4)	95·4 (83·2-99·4)	96·9 (82·1-99·9)	59·8 (51·9-67·4)	70·9 (59·4-81·0)	71·4 (59·7-81·6)	78·0 (63·9-90·5)	59·6 (57·3-61·8)	66·6 (63·9-69·1)	67·1 (64·7-69·5)	78·4 (70·4-87·1)	26·9 (22·4-34·7)	40·6 (31·5-45·8)	40·8 (31·5-46·6)	56·1 (38·5-69·0)
Lithuania	99·2 (96·7-99·9)	99·0 (95·8-99·9)	99·0 (95·9-99·9)	99·3 (96·8-99·9)	60·5 (53·2-67·3)	77·0 (67·6-85·1)	77·7 (68·2-86·0)	88·3 (76·2-98·6)	59·1 (57·6-60·6)	62·4 (60·8-64·0)	62·5 (60·9-64·1)	67·9 (62·7-76·1)	31·5 (26·2-38·2)	41·2 (28·8-45·9)	41·2 (28·9-46·2)	49·4 (30·9-61·1)
Moldova	74·1 (68·6-79·4)	96·7 (93·0-98·8)	96·8 (93·0-98·9)	98·5 (93·6-99·9)	54·8 (47·3-62·1)	80·8 (70·8-90·0)	81·7 (71·6-91·0)	95·3 (83·6-100·0)	53·5 (48·8-57·6)	56·5 (52·1-60·6)	56·6 (51·9-60·6)	72·2 (67·2-76·7)	27·3 (22·4-34·3)	42·9 (30·0-49·8)	43·4 (29·7-51·4)	57·0 (33·5-74·9)
Russia	99·6 (98·8-99·9)	99·7 (98·8-100·0)	99·7 (98·9-100·0)	100·0 (99·8-100·0)	33·7 (26·3-41·1)	56·6 (45·6-67·1)	58·0 (46·8-68·9)	78·1 (63·0-92·9)	65·5 (64·3-66·7)	65·0 (63·8-66·2)	65·1 (63·8-66·3)	70·4 (66·5-74·2)	4·3 (0·0-14·2)	20·7 (10·3-30·5)	20·7 (9·0-31·1)	33·6 (8·6-55·5)
Ukraine	95·6 (92·8-97·6)	97·8 (94·9-99·2)	97·8 (94·8-99·3)	98·8 (94·6-99·9)	45·9 (39·9-52·4)	67·7 (57·4-78·8)	68·9 (58·4-80·2)	87·0 (73·0-100·0)	56·4 (54·6-58·0)	57·8 (56·1-59·5)	57·8 (56·1-59·5)	70·6 (68·7-72·7)	22·1 (17·5-30·5)	38·0 (26·8-45·5)	37·6 (26·0-46·3)	46·5 (24·3-65·6)
**Central Europe**																
Albania	57·8 (51·1-64·7)	82·6 (76·9-87·5)	83·3 (77·6-88·1)	90·8 (85·8-94·4)	49·9 (37·9-61·3)	58·1 (46·1-69·5)	59·3 (47·2-70·9)	76·1 (61·5-90·3)	67·8 (64·9-70·5)	66·8 (62·6-70·4)	67·1 (63·1-70·9)	72·1 (64·6-81·2)	34·2 (27·1-55·7)	65·3 (53·8-71·2)	65·3 (54·0-71·5)	68·0 (53·5-81·1)
Bosnia and Herzegovina	61·7 (55·0-68·4)	77·7 (70·7-84·1)	78·4 (71·5-84·7)	86·2 (80·0-91·3)	80·6 (66·1-94·9)	83·1 (71·0-94·1)	81·9 (69·7-93·1)	66·4 (52·6-78·5)	34·7 (32·5-36·8)	33·4 (31·2-35·5)	33·7 (31·5-35·6)	34·8 (29·6-40·9)	56·1 (49·6-64·4)	64·8 (58·2-70·9)	64·7 (58·1-70·9)	70·9 (59·2-81·9)
Bulgaria	91·7 (87·5-94·8)	94·8 (92·0-97·0)	94·9 (92·3-97·1)	96·9 (95·2-98·3)	69·6 (62·6-76·2)	65·9 (56·5-74·3)	66·5 (56·8-75·0)	74·0 (62·2-84·5)	43·7 (42·5-44·9)	48·0 (46·8-49·3)	48·1 (46·9-49·3)	63·9 (62·4-66·2)	54·4 (50·3-62·6)	65·6 (54·8-71·4)	66·0 (54·3-72·6)	75·3 (56·8-89·3)
Croatia	91·3 (88·1-93·7)	95·2 (92·6-97·0)	95·4 (92·8-97·1)	97·3 (95·3-98·6)	68·3 (60·0-76·5)	69·4 (60·6-78·5)	70·9 (61·8-80·2)	90·8 (79·1-100·0)	59·9 (58·3-61·4)	56·7 (55·0-58·5)	56·8 (55·2-58·6)	59·3 (57·1-63·4)	65·0 (60·5-71·4)	79·5 (68·8-84·5)	79·7 (69·0-85·2)	93·0 (76·1-100·0)
Czech Republic	98·5 (97·7-99·1)	99·1 (98·6-99·5)	99·2 (98·6-99·5)	99·5 (99·1-99·7)	60·5 (51·7-68·2)	63·4 (51·5-73·3)	63·2 (51·1-73·1)	61·6 (45·8-74·6)	54·8 (53·7-55·9)	57·7 (56·6-58·9)	57·9 (56·6-59·0)	67·0 (63·3-71·7)	71·0 (66·0-76·1)	82·8 (74·7-85·9)	83·2 (74·8-86·7)	94·8 (82·3-100·0)
Hungary	91·0 (86·4-94·6)	94·2 (91·0-96·6)	94·4 (91·3-96·8)	96·4 (94·3-98·0)	64·5 (57·8-71·2)	68·5 (60·8-75·8)	67·6 (59·8-75·0)	56·0 (46·0-66·3)	49·9 (48·6-51·2)	49·7 (48·3-51·1)	49·5 (48·1-50·9)	59·7 (57·8-62·6)	60·9 (52·5-64·4)	73·5 (62·4-78·3)	73·5 (61·8-79·2)	85·1 (69·7-94·8)
Macedonia	72·7 (66·8-78·3)	84·7 (80·6-88·6)	85·4 (81·4-89·3)	92·5 (89·8-95·3)	86·9 (76·8-95·8)	87·9 (78·0-97·2)	87·5 (77·5-96·8)	81·9 (70·8-92·3)	37·0 (35·2-38·8)	40·3 (38·5-42·3)	40·7 (38·7-42·5)	54·9 (52·3-58·4)	57·2 (51·8-69·5)	69·2 (63·6-74·8)	69·1 (63·6-75·0)	79·7 (68·6-88·1)
Montenegro	79·7 (74·8-84·3)	79·9 (74·6-84·4)	80·0 (74·7-84·6)	82·4 (76·8-87·2)	84·8 (74·4-95·2)	89·2 (79·5-98·1)	88·8 (78·9-97·8)	82·7 (70·8-93·5)	59·1 (57·2-61·0)	55·7 (53·7-57·7)	55·9 (53·8-57·8)	62·3 (60·1-64·4)	44·3 (38·1-48·9)	52·2 (44·8-56·9)	52·4 (45·0-57·1)	57·1 (46·5-65·9)
Poland	94·6 (91·6-96·7)	97·1 (95·5-98·3)	97·2 (95·7-98·4)	98·5 (97·7-99·2)	59·6 (52·6-66·6)	70·2 (61·7-77·7)	71·0 (62·3-78·6)	82·0 (70·9-92·0)	47·2 (46·3-48·2)	48·2 (47·2-49·1)	48·1 (47·2-49·1)	52·4 (50·8-55·3)	61·3 (56·5-68·9)	73·2 (61·9-78·9)	73·6 (61·9-79·6)	83·7 (66·3-93·8)
Romania	82·8 (78·6-86·3)	92·2 (88·5-95·1)	92·6 (89·0-95·4)	96·5 (94·0-98·2)	27·8 (20·1-34·9)	53·8 (46·1-61·5)	54·6 (46·7-62·6)	66·0 (55·0-77·0)	54·6 (53·4-55·7)	58·2 (57·0-59·4)	58·1 (56·8-59·3)	72·3 (70·8-74·3)	56·9 (51·9-62·9)	68·9 (56·3-73·6)	68·8 (56·3-74·1)	80·6 (62·2-91·6)
Serbia	70·7 (65·1-76·0)	83·3 (79·5-86·7)	83·4 (79·5-86·7)	84·5 (80·4-88·0)	59·1 (48·6-69·7)	67·1 (58·7-74·8)	67·4 (59·0-75·1)	71·9 (62·5-80·6)	58·0 (56·5-59·4)	58·3 (56·9-59·7)	58·5 (57·0-59·9)	62·6 (58·0-69·2)	51·9 (47·4-59·8)	61·5 (54·9-65·4)	60·8 (54·5-64·7)	69·3 (59·6-76·6)
Slovakia	97·9 (96·8-98·7)	98·6 (97·8-99·2)	98·7 (97·8-99·2)	99·1 (98·6-99·5)	73·6 (67·5-79·3)	80·8 (73·4-87·8)	81·2 (73·6-88·3)	86·5 (77·0-95·1)	53·7 (52·5-55·0)	55·9 (54·7-57·1)	55·9 (54·7-57·2)	63·1 (61·0-66·9)	57·2 (50·3-60·9)	65·6 (57·3-70·0)	65·6 (57·2-70·7)	72·0 (60·2-83·6)
Slovenia	94·1 (91·8-96·0)	97·3 (95·7-98·4)	97·4 (95·9-98·5)	98·7 (97·6-99·4)	56·0 (48·1-63·9)	68·0 (58·1-77·3)	68·6 (58·6-78·1)	77·1 (63·8-89·9)	62·4 (60·7-64·2)	60·2 (58·4-62·1)	60·4 (58·6-62·0)	65·0 (62·3-70·0)	71·8 (64·7-76·4)	87·3 (77·2-92·1)	87·4 (77·3-92·7)	96·0 (84·7-100·0)
**Central Asia**																
Armenia	81·4 (77·0-85·2)	91·0 (79·1-97·7)	91·3 (78·6-98·0)	95·1 (77·2-99·9)	50·3 (42·7-57·2)	52·4 (45·6-59·0)	53·5 (46·6-60·3)	68·9 (60·6-76·9)	59·4 (48·8-67·9)	46·5 (35·3-55·0)	46·7 (35·4-55·1)	39·3 (26·2-49·6)	49·2 (43·5-60·3)	55·5 (48·5-62·3)	55·3 (48·2-62·7)	63·7 (49·9-76·6)

Location	Indicator 16.1.2: Death rate due to conflict and terrorism (per 100,000 population)				Indicator 16.1.3: Age-standardised prevalence of physical or sexual violence experienced by populations in the last 12 months (%)				Indicator 16.2.3: Age-standardised prevalence of women and men aged 18-29 years who experienced sexual violence by age 18 (%)				Indicator 17.19.2c: Percentage of well-certified deaths by a vital registration (VR) system among a country's total population (%)			
	2000	2015	2016	2030	2000	2015	2016	2030	2000	2015	2016	2030	2000	2015	2016	2030
Sweden	100·0 (100·0-100·0)	100·0 (100·0-100·0)	100·0 (100·0-100·0)	100·0 (100·0-100·0)	75·8 (73·6-78·0)	74·9 (72·7-77·2)	75·0 (72·8-77·3)	75·9 (73·9-78·0)	42·2 (30·3-54·3)	29·3 (15·3-42·8)	29·4 (15·4-42·9)	26·9 (12·0-41·1)	91·6 (89·1-93·8)	90·1 (86·9-92·8)	90·2 (86·7-93·2)	88·4 (81·4-94·5)
Switzerland	100·0 (100·0-100·0)	100·0 (100·0-100·0)	100·0 (100·0-100·0)	100·0 (100·0-100·0)	90·0 (86·5-93·2)	93·3 (90·1-96·2)	93·5 (90·3-96·3)	95·7 (92·4-98·4)	44·2 (33·9-53·8)	45·0 (32·4-56·4)	43·4 (29·6-55·5)	43·2 (29·7-55·5)	89·7 (86·0-93·0)	91·9 (86·8-96·4)	92·1 (86·7-96·8)	94·8 (85·1-100·0)
United Kingdom	100·0 (100·0-100·0)	100·0 (100·0-100·0)	100·0 (100·0-100·0)	100·0 (100·0-100·0)	89·8 (88·7-90·8)	90·6 (89·5-91·5)	90·6 (89·5-91·5)	91·0 (89·9-91·9)	26·5 (16·2-37·0)	34·2 (24·1-44·4)	35·6 (25·3-45·8)	37·4 (27·5-47·7)	96·5 (95·8-97·2)	96·0 (95·3-96·8)	96·1 (95·4-96·9)	95·6 (94·6-96·6)
**Southern Latin America**																
Argentina	100·0 (100·0-100·0)	100·0 (100·0-100·0)	100·0 (100·0-100·0)	100·0 (100·0-100·0)	50·7 (46·2-54·8)	57·5 (53·3-61·5)	58·0 (53·9-62·0)	65·3 (61·5-69·1)	53·1 (43·3-63·2)	53·0 (43·2-63·4)	52·9 (43·2-63·5)	52·8 (42·5-63·8)	70·9 (66·9-74·7)	72·8 (66·4-79·0)	73·4 (66·2-80·8)	72·9 (60·5-83·2)
Chile	100·0 (100·0-100·0)	100·0 (100·0-100·0)	100·0 (100·0-100·0)	100·0 (100·0-100·0)	70·4 (62·5-76·6)	76·7 (69·6-82·2)	77·1 (70·1-82·6)	82·9 (76·7-87·8)	53·3 (44·3-62·6)	53·0 (44·0-62·5)	53·0 (43·9-62·5)	52·8 (43·6-62·3)	92·3 (89·3-94·8)	95·9 (92·4-98·8)	96·1 (92·4-99·2)	98·4 (92·1-100·0)
Uruguay	100·0 (100·0-100·0)	100·0 (100·0-100·0)	100·0 (100·0-100·0)	100·0 (100·0-100·0)	81·6 (78·7-84·2)	84·7 (82·1-87·0)	85·0 (82·4-87·2)	89·1 (87·0-91·2)	53·8 (45·5-62·2)	53·1 (44·5-61·7)	53·0 (44·4-61·5)	52·8 (44·0-61·4)	83·6 (80·0-86·8)	82·0 (76·5-86·7)	82·7 (76·5-87·9)	83·6 (71·5-92·5)
**Eastern Europe**																
Belarus	100·0 (100·0-100·0)	100·0 (100·0-100·0)	100·0 (100·0-100·0)	100·0 (100·0-100·0)	83·7 (82·5-84·9)	90·4 (89·3-91·5)	90·8 (89·7-91·8)	95·5 (94·5-96·6)	29·1 (18·9-39·3)	29·6 (19·4-40·0)	29·6 (19·3-40·1)	30·1 (19·5-40·7)	84·7 (81·0-88·2)	87·5 (82·1-92·0)	87·9 (81·9-92·8)	89·7 (78·7-97·4)
Estonia	100·0 (100·0-100·0)	100·0 (100·0-100·0)	100·0 (100·0-100·0)	100·0 (100·0-100·0)	90·4 (89·2-91·3)	97·7 (96·8-98·5)	97·9 (97·0-98·7)	100·0 (100·0-100·0)	29·7 (19·6-39·6)	30·0 (20·0-40·2)	30·0 (20·0-40·3)	30·3 (20·2-40·6)	97·2 (94·7-99·5)	98·7 (95·7-100·0)	98·8 (95·4-100·0)	99·3 (94·7-100·0)
Latvia	100·0 (100·0-100·0)	100·0 (100·0-100·0)	100·0 (100·0-100·0)	100·0 (100·0-100·0)	83·9 (82·7-85·2)	91·4 (90·3-92·4)	91·6 (90·6-92·6)	94·7 (93·6-95·7)	29·3 (19·0-39·4)	29·8 (19·7-39·9)	29·8 (19·7-40·1)	29·9 (19·0-40·6)	96·1 (93·4-98·6)	98·2 (94·9-100·0)	98·3 (94·5-100·0)	99·4 (95·2-100·0)
Lithuania	100·0 (100·0-100·0)	100·0 (100·0-100·0)	100·0 (100·0-100·0)	100·0 (100·0-100·0)	90·2 (89·1-91·2)	95·6 (94·7-96·5)	95·9 (95·0-96·8)	99·7 (98·9-100·0)	28·9 (18·6-39·1)	29·8 (19·9-39·6)	29·8 (19·9-39·6)	30·0 (20·1-39·7)	98·3 (95·9-100·0)	99·7 (97·9-100·0)	99·7 (98·0-100·0)	99·8 (97·8-100·0)
Moldova	100·0 (100·0-100·0)	100·0 (100·0-100·0)	100·0 (100·0-100·0)	100·0 (100·0-100·0)	74·6 (72·6-76·4)	82·2 (80·5-83·9)	82·7 (80·9-84·3)	88·4 (86·0-90·5)	29·2 (18·9-39·4)	29·3 (19·0-39·8)	29·2 (18·9-40·0)	29·6 (18·7-40·1)	90·5 (87·5-93·4)	95·9 (92·5-98·9)	96·1 (92·3-99·4)	98·7 (93·5-100·0)
Russia	32·7 (24·4-44·6)	100·0 (100·0-100·0)	100·0 (100·0-100·0)	100·0 (100·0-100·0)	79·4 (77·9-80·8)	86·5 (85·2-87·8)	86·8 (85·5-88·0)	90·3 (89·1-91·6)	28·7 (18·6-38·9)	29·1 (18·7-40·0)	29·1 (18·4-39·8)	29·2 (16·9-40·9)	90·5 (87·3-93·5)	92·6 (88·3-96·5)	92·9 (88·1-97·2)	94·8 (84·0-100·0)
Ukraine	100·0 (100·0-100·0)	32·4 (28·4-35·5)	100·0 (100·0-100·0)	100·0 (100·0-100·0)	80·9 (79·5-82·5)	86·9 (85·7-88·3)	86·9 (85·6-88·3)	86·2 (84·5-87·9)	29·1 (18·8-39·2)	29·5 (19·1-40·2)	29·5 (19·1-40·2)	29·5 (17·5-40·6)	88·4 (84·9-91·6)	95·7 (91·9-99·0)	95·7 (91·3-99·2)	98·7 (93·3-100·0)
**Central Europe**																
Albania	100·0 (100·0-100·0)	100·0 (100·0-100·0)	100·0 (100·0-100·0)	100·0 (100·0-100·0)	87·5 (85·9-89·0)	94·5 (93·1-95·9)	94·6 (93·3-96·1)	96·8 (95·3-98·3)	80·8 (74·5-87·1)	79·0 (72·6-85·5)	79·0 (72·6-85·4)	78·7 (72·1-85·1)	69·4 (64·9-73·9)	61·4 (51·8-70·7)	62·4 (52·8-72·0)	54·7 (38·8-69·1)
Bosnia and Herzegovina	100·0 (100·0-100·0)	100·0 (100·0-100·0)	100·0 (100·0-100·0)	100·0 (100·0-100·0)	91·1 (89·2-92·8)	89·8 (87·8-91·5)	90·1 (88·1-91·8)	94·0 (92·2-95·6)	73·4 (68·0-78·3)	73·2 (67·6-78·2)	73·3 (67·8-78·2)	73·2 (67·2-78·5)	76·5 (67·2-84·6)	75·1 (67·8-81·3)	75·4 (67·9-81·9)	81·7 (66·6-93·5)
Bulgaria	100·0 (100·0-100·0)	100·0 (100·0-100·0)	100·0 (100·0-100·0)	100·0 (100·0-100·0)	82·9 (81·6-84·0)	89·7 (88·6-90·6)	90·0 (88·9-90·9)	93·7 (92·7-94·6)	73·5 (68·3-78·4)	73·4 (68·1-78·2)	73·4 (68·1-78·2)	73·4 (67·8-78·2)	76·2 (72·2-79·8)	75·2 (68·3-81·1)	75·9 (68·5-82·3)	71·4 (57·0-82·3)
Croatia	73·6 (63·5-88·4)	100·0 (100·0-100·0)	100·0 (100·0-100·0)	100·0 (100·0-100·0)	92·3 (91·4-93·3)	97·1 (96·2-97·9)	97·3 (96·4-98·1)	99·4 (98·5-100·0)	73·6 (68·3-78·5)	73·4 (68·2-78·5)	73·4 (68·2-78·4)	73·4 (67·8-78·3)	85·4 (82·2-88·7)	93·0 (89·1-96·4)	93·0 (88·8-96·6)	96·8 (89·9-100·0)
Czech Republic	100·0 (100·0-100·0)	100·0 (100·0-100·0)	100·0 (100·0-100·0)	100·0 (100·0-100·0)	88·7 (87·5-89·8)	92·7 (91·7-93·7)	92·9 (91·9-93·9)	95·9 (94·8-96·9)	69·7 (61·9-76·0)	69·9 (62·2-76·3)	69·9 (62·0-76·7)	70·0 (61·5-77·0)	89·1 (86·2-91·9)	92·0 (88·3-95·3)	92·2 (88·0-95·7)	94·1 (86·3-100·0)
Hungary	100·0 (100·0-100·0)	100·0 (100·0-100·0)	100·0 (100·0-100·0)	100·0 (100·0-100·0)	81·4 (80·0-82·7)	87·7 (86·5-88·9)	88·0 (86·8-89·2)	91·6 (90·3-92·8)	73·6 (68·4-78·4)	73·4 (68·1-78·3)	73·4 (68·1-78·3)	73·4 (68·2-78·3)	96·9 (94·7-99·0)	99·1 (97·0-100·0)	99·2 (96·9-100·0)	99·7 (97·1-100·0)
Macedonia	100·0 (100·0-100·0)	100·0 (100·0-100·0)	100·0 (100·0-100·0)	100·0 (100·0-100·0)	86·7 (84·4-88·7)	90·9 (88·8-92·6)	91·0 (89·0-92·7)	93·5 (91·5-95·1)	63·4 (54·6-71·4)	62·1 (53·7-70·2)	62·1 (53·7-70·2)	62·0 (53·5-70·1)	84·6 (80·9-88·0)	79·8 (73·5-85·4)	80·4 (73·7-86·3)	77·4 (62·9-88·4)
Montenegro	100·0 (100·0-100·0)	100·0 (100·0-100·0)	100·0 (100·0-100·0)	100·0 (100·0-100·0)	83·1 (80·8-85·2)	88·1 (86·0-89·9)	88·3 (86·3-90·1)	91·5 (89·7-93·2)	73·9 (68·7-78·7)	73·4 (68·1-78·2)	73·4 (68·1-78·2)	73·3 (67·8-78·1)	71·8 (66·8-76·6)	79·5 (71·5-86·8)	79·9 (71·7-87·4)	85·4 (70·9-96·2)
Poland	100·0 (100·0-100·0)	100·0 (100·0-100·0)	100·0 (100·0-100·0)	100·0 (100·0-100·0)	96·1 (95·2-97·0)	100·0 (100·0-100·0)	100·0 (100·0-100·0)	100·0 (100·0-100·0)	71·6 (65·9-77·6)	70·5 (64·9-76·5)	70·3 (64·7-76·3)	69·8 (63·5-76·1)	76·9 (72·8-80·8)	76·9 (71·5-82·0)	77·6 (71·5-83·3)	79·1 (66·5-89·4)
Romania	100·0 (100·0-100·0)	100·0 (100·0-100·0)	100·0 (100·0-100·0)	100·0 (100·0-100·0)	87·3 (86·1-88·5)	93·1 (92·1-94·1)	93·4 (92·4-94·3)	97·0 (96·0-98·0)	73·6 (68·4-78·3)	73·2 (67·5-78·2)	73·2 (67·5-78·2)	72·9 (66·6-78·5)	89·8 (87·1-92·5)	90·4 (86·3-93·6)	90·9 (86·8-94·2)	92·2 (84·5-97·9)
Serbia	25·3 (15·2-43·7)	100·0 (100·0-100·0)	100·0 (100·0-100·0)	100·0 (100·0-100·0)	83·9 (81·5-86·0)	88·6 (86·4-90·5)	88·8 (86·6-90·6)	91·0 (88·9-92·9)	70·0 (63·0-76·8)	68·3 (61·5-74·8)	68·3 (61·5-74·8)	67·8 (61·0-74·3)	78·2 (74·4-82·0)	80·6 (76·0-84·6)	81·0 (75·7-85·6)	83·2 (72·6-91·9)
Slovakia	100·0 (100·0-100·0)	100·0 (100·0-100·0)	100·0 (100·0-100·0)	100·0 (100·0-100·0)	81·3 (79·8-82·7)	86·5 (85·3-87·7)	86·8 (85·6-88·0)	91·0 (89·8-92·2)	73·7 (68·5-78·4)	73·5 (68·2-78·4)	73·5 (68·2-78·4)	73·4 (67·9-78·4)	88·8 (85·6-91·6)	96·7 (93·4-99·5)	96·8 (93·3-99·6)	98·5 (93·9-100·0)
Slovenia	100·0 (100·0-100·0)	100·0 (100·0-100·0)	100·0 (100·0-100·0)	100·0 (100·0-100·0)	99·9 (99·4-100·0)	100·0 (100·0-100·0)	100·0 (100·0-100·0)	100·0 (100·0-100·0)	73·4 (68·2-78·2)	73·2 (67·6-78·2)	73·3 (67·6-78·2)	73·1 (67·3-78·3)	92·4 (89·7-95·0)	91·5 (88·0-94·6)	91·9 (88·0-95·2)	91·4 (83·0-97·9)
**Central Asia**																
Armenia	100·0 (100·0-100·0)	40·4 (32·4-53·9)	100·0 (100·0-100·0)	100·0 (100·0-100·0)	85·0 (82·5-87·4)	90·7 (88·6-92·7)	91·0 (88·8-92·9)	94·0 (91·9-96·0)	99·9 (99·6-100·0)	99·7 (99·3-100·0)	99·6 (99·1-100·0)	99·9 (99·4-100·0)	88·5 (84·5-92·1)	97·6 (93·9-100·0)	97·7 (93·3-100·0)	99·5 (95·3-100·0)

Location	**SDG Index:** Geometric mean of all health-related SDG indicators (scale of 0 to 100)				**MDG Index:** Geometric mean of all health-related SDG indicators with corresponding MDG indicators (scale of 0 to 100)				**Non-MDG Index:** Geometric mean of all health-related indicators without corresponding MDG indicators (scale of 0 to 100)				**Indicator 1.5.1:** Death rate due to exposure to forces of nature (per 100,000 population)			
	**2000**	**2015**	**2016**	**2030**	**2000**	**2015**	**2016**	**2030**	**2000**	**2015**	**2016**	**2030**	**2000**	**2015**	**2016**	**2030**
Azerbaijan	48·4 (46·4-50·1)	59·0 (56·2-61·4)	59·7 (56·9-62·3)	62·9 (57·1-68·3)	40·0 (36·8-43·0)	54·6 (49·8-58·5)	55·2 (50·3-59·0)	63·7 (54·9-70·7)	55·4 (53·2-57·5)	61·4 (57·8-64·5)	62·0 (58·3-65·4)	56·8 (49·1-66·9)	51·3 (44·9-60·7)	100·0 (100·0-100·0)	100·0 (100·0-100·0)	69·4 (62·8-78·0)
Georgia	59·3 (57·4-60·9)	57·2 (54·3-59·7)	57·8 (54·5-60·6)	58·1 (51·2-63·5)	53·6 (48·9-56·8)	60·7 (55·7-64·1)	61·5 (56·6-64·9)	68·3 (62·3-73·2)	64·1 (62·1-65·9)	56·6 (53·4-59·7)	57·0 (53·3-60·4)	53·2 (43·0-60·5)	100·0 (100·0-100·0)	43·0 (35·0-53·8)	44·7 (36·5-55·8)	50·5 (42·7-60·6)
Kazakhstan	42·4 (39·2-45·3)	54·6 (47·8-58·2)	55·2 (48·5-59·6)	66·2 (57·0-72·2)	54·4 (51·6-57·2)	67·6 (63·3-70·9)	68·4 (64·1-71·7)	79·8 (75·2-83·9)	40·3 (36·5-43·7)	50·9 (43·0-55·4)	51·5 (43·5-56·8)	60·1 (48·8-67·8)	59·0 (50·6-70·2)	40·2 (34·2-47·5)	43·9 (37·7-51·3)	40·0 (34·0-47·2)
Kyrgyzstan	48·1 (46·3-49·9)	55·7 (52·4-57·7)	56·5 (53·1-58·7)	63·3 (59·3-66·7)	46·5 (41·7-50·1)	53·1 (36·9-58·2)	53·6 (37·1-58·9)	59·8 (40·4-65·8)	50·1 (48·0-52·1)	58·1 (56·1-60·0)	58·9 (56·7-61·0)	65·7 (61·9-69·1)	35·8 (29·0-46·8)	36·0 (30·4-43·7)	39·2 (33·6-47·1)	35·0 (29·4-43·1)
Mongolia	36·7 (34·3-39·1)	48·2 (44·8-51·3)	48·7 (45·2-51·9)	51·9 (45·0-58·0)	42·3 (38·7-46·3)	57·0 (50·0-62·5)	57·7 (50·5-63·2)	67·1 (56·5-74·2)	35·1 (32·1-38·0)	45·9 (42·1-49·4)	46·3 (42·3-50·0)	47·4 (39·8-54·7)	36·9 (30·5-45·3)	41·6 (35·7-50·0)	45·8 (39·8-54·3)	40·5 (34·4-49·3)
Tajikistan	40·9 (38·4-43·2)	55·9 (52·1-58·7)	56·4 (52·6-59·1)	60·6 (54·5-65·8)	36·5 (29·6-40·5)	49·9 (36·3-55·3)	50·3 (36·9-56·2)	55·3 (38·4-64·4)	44·2 (41·5-46·9)	59·3 (56·1-62·2)	59·7 (56·4-62·7)	62·9 (56·4-68·1)	18·0 (11·7-28·0)	43·8 (36·9-55·0)	47·4 (40·3-58·8)	41·6 (34·7-52·6)
Turkmenistan	49·9 (48·0-51·8)	60·1 (58·2-62·0)	60·9 (59·0-62·8)	68·2 (64·1-71·3)	42·6 (37·8-47·0)	57·9 (53·3-61·9)	59·0 (54·4-63·0)	71·2 (65·5-75·9)	53·6 (51·7-55·4)	60·6 (58·2-62·7)	61·1 (58·6-63·2)	63·2 (52·5-68·9)	64·6 (57·3-75·7)	41·5 (35·8-49·3)	44·4 (38·6-52·2)	43·3 (37·5-51·0)
Uzbekistan	52·9 (50·5-54·7)	62·1 (59·3-64·5)	62·5 (59·7-65·0)	67·5 (62·1-72·2)	50·4 (40·7-55·4)	62·9 (53·6-68·1)	63·6 (54·3-68·9)	72·7 (63·6-79·8)	53·4 (51·2-55·5)	60·9 (58·2-63·3)	61·1 (58·2-63·6)	62·2 (50·8-68·7)	100·0 (100·0-100·0)	100·0 (100·0-100·0)	100·0 (100·0-100·0)	100·0 (100·0-100·0)
**Central Latin America**																
Colombia	51·7 (50·2-52·9)	64·5 (62·7-66·3)	65·6 (63·8-67·6)	73·1 (69·7-76·0)	53·0 (51·6-54·4)	67·1 (65·3-68·4)	67·9 (66·0-69·3)	78·1 (74·9-80·3)	53·7 (51·8-55·1)	65·0 (62·8-67·3)	66·2 (63·8-68·7)	71·8 (67·6-75·3)	27·6 (22·6-34·3)	37·1 (31·7-44·2)	40·1 (34·7-47·3)	36·3 (31·0-43·4)
Costa Rica	62·9 (60·9-64·2)	69·8 (67·0-71·6)	70·0 (67·2-72·0)	72·2 (67·9-76·2)	66·6 (64·7-68·1)	74·7 (72·8-76·3)	75·3 (73·3-76·8)	81·0 (77·8-83·7)	62·1 (59·0-64·2)	66·1 (59·5-69·8)	66·1 (59·1-69·9)	63·0 (54·0-72·8)	37·5 (32·2-44·3)	46·3 (40·7-53·5)	46·0 (40·4-53·2)	46·4 (40·8-53·6)
El Salvador	49·2 (47·7-50·6)	59·3 (57·5-60·8)	60·4 (58·7-62·0)	64·7 (61·8-67·3)	49·8 (47·5-52·2)	62·5 (59·8-65·0)	63·2 (60·2-65·8)	71·2 (66·2-75·8)	51·4 (49·7-53·2)	59·9 (58·0-61·7)	61·2 (59·2-63·1)	63·4 (59·7-66·5)	18·1 (13·5-24·2)	30·7 (25·4-37·9)	34·3 (28·8-41·6)	22·1 (17·1-28·6)
Guatemala	38·8 (36·8-40·6)	51·8 (49·2-54·6)	52·8 (49·9-56·0)	59·3 (52·7-65·2)	31·9 (28·8-35·6)	47·2 (42·1-50·7)	47·9 (42·9-51·5)	58·2 (51·5-63·3)	41·4 (39·3-43·6)	54·9 (52·2-58·1)	56·1 (52·9-59·8)	59·6 (47·1-67·5)	27·3 (22·4-33·9)	46·7 (41·2-53·9)	50·2 (44·5-57·5)	37·7 (32·4-44·6)
Honduras	35·4 (33·2-38·0)	49·9 (46·6-53·6)	50·9 (47·6-54·8)	52·3 (47·2-57·2)	39·0 (36·7-41·3)	53·1 (49·7-56·4)	53·7 (50·1-57·1)	62·9 (56·9-67·7)	36·7 (34·2-39·8)	50·9 (47·3-55·6)	52·1 (48·3-56·8)	50·5 (44·5-56·2)	0·0 (0·0-0·0)	40·1 (34·6-47·3)	43·4 (37·8-50·6)	6·5 (2·4-11·9)
Mexico	57·4 (56·5-58·3)	65·5 (64·5-66·4)	66·6 (65·6-67·6)	72·3 (70·0-74·1)	57·3 (56·4-58·3)	66·7 (65·8-67·5)	67·3 (66·3-68·1)	73·9 (72·1-75·6)	59·1 (57·0-60·5)	66·4 (64·7-68·0)	67·6 (65·8-69·4)	71·8 (65·4-75·3)	36·1 (31·4-41·9)	51·4 (46·1-57·8)	52·4 (47·1-58·6)	49·1 (43·9-55·3)
Nicaragua	42·9 (42·2-43·8)	62·4 (60·3-64·3)	63·3 (60·9-65·2)	67·7 (63·3-71·1)	39·7 (38·1-41·5)	55·3 (49·4-59·6)	56·0 (49·7-60·4)	67·8 (59·2-72·7)	46·3 (45·3-47·2)	66·6 (64·3-69·0)	67·5 (64·9-69·9)	67·0 (53·8-72·6)	0·0 (0·0-0·0)	34·3 (28·9-41·6)	39·0 (33·5-46·4)	19·4 (14·7-25·6)
Panama	61·5 (60·4-62·6)	66·1 (64·4-67·8)	67·0 (65·1-68·8)	71·1 (67·1-74·4)	53·8 (52·1-55·4)	61·5 (59·3-63·6)	62·4 (59·9-64·6)	69·3 (65·0-73·0)	67·0 (65·6-68·5)	70·0 (67·8-72·1)	70·7 (68·3-73·1)	72·6 (66·3-77·2)	40·7 (35·4-47·6)	36·9 (31·6-44·1)	39·8 (34·3-47·1)	37·7 (32·3-44·9)
Venezuela	40·8 (37·1-43·7)	55·4 (52·4-58·7)	55·9 (52·6-59·5)	54·4 (47·7-60·6)	57·5 (56·1-58·9)	61·0 (58·9-62·7)	61·1 (59·0-62·9)	63·0 (59·6-65·8)	37·1 (32·5-40·5)	52·6 (46·6-57·3)	53·0 (46·7-57·9)	47·8 (38·3-58·2)	0·0 (0·0-0·0)	77·9 (71·9-84·6)	82·9 (77·3-88·8)	13·8 (9·3-19·7)
**Andean Latin America**																
Bolivia	38·9 (37·1-40·8)	51·3 (47·6-54·8)	52·0 (48·2-55·5)	59·0 (52·7-64·3)	35·1 (32·3-37·4)	50·6 (47·0-53·5)	51·4 (47·8-54·3)	62·3 (57·6-66·2)	43·7 (41·3-46·1)	53·6 (49·2-57·9)	54·2 (49·7-58·7)	58·2 (49·3-65·1)	31·9 (22·5-51·4)	31·4 (21·9-50·9)	33·7 (24·4-53·1)	33·2 (23·9-52·6)
Ecuador	53·2 (51·8-54·8)	62·4 (60·8-63·9)	59·7 (57·0-62·3)	66·2 (63·2-69·1)	50·3 (48·4-52·0)	61·4 (59·0-63·4)	61·9 (59·5-63·9)	68·7 (65·3-71·4)	55·9 (54·0-58·2)	63·8 (61·7-65·9)	60·0 (56·3-63·6)	65·7 (62·1-69·8)	33·3 (24·0-52·8)	46·2 (37·2-64·8)	16·7 (6·9-36·9)	22·4 (12·8-42·4)
Peru	51·6 (49·9-53·5)	62·5 (60·2-64·7)	63·3 (60·9-65·6)	69·1 (65·1-72·4)	47·2 (44·7-49·4)	59·9 (56·4-62·4)	60·5 (57·0-63·3)	69·2 (63·2-73·0)	54·7 (52·5-57·0)	64·4 (61·5-67·1)	65·2 (62·0-68·0)	69·2 (64·6-73·1)	28·8 (19·4-48·6)	37·7 (28·6-56·8)	41·7 (32·7-60·6)	36·1 (26·9-55·3)
**Caribbean**																
Antigua and Barbuda	72·5 (70·6-74·2)	76·0 (73·7-78·0)	76·2 (74·1-78·3)	78·2 (73·1-82·6)	63·1 (60·5-65·8)	68·9 (65·3-71·7)	69·4 (65·5-72·2)	73·9 (66·8-79·4)	77·8 (75·1-80·1)	78·8 (74·9-81·8)	78·8 (74·8-82·0)	76·5 (63·2-85·0)	100·0 (100·0-100·0)	100·0 (100·0-100·0)	100·0 (100·0-100·0)	100·0 (100·0-100·0)
The Bahamas	63·8 (62·0-65·7)	65·6 (62·4-68·8)	66·2 (63·0-69·1)	69·8 (63·9-74·7)	62·9 (60·6-65·1)	67·2 (63·5-70·3)	67·6 (63·8-70·8)	71·1 (60·6-77·9)	65·8 (63·1-68·4)	66·5 (62·4-70·6)	67·1 (63·1-70·9)	70·2 (64·1-75·0)	38·4 (29·4-57·5)	16·2 (6·3-36·4)	17·7 (7·9-37·9)	23·9 (14·4-43·8)
Barbados	68·8 (66·6-71·1)	73·1 (70·6-75·3)	73·2 (70·7-75·4)	73·8 (68·7-77·7)	63·3 (59·2-66·9)	67·2 (62·4-70·6)	67·6 (62·5-71·1)	71·0 (61·3-77·6)	72·4 (69·3-75·1)	76·8 (74·0-79·4)	76·7 (73·9-79·2)	75·7 (70·7-79·9)	58·6 (49·6-75·9)	100·0 (100·0-100·0)	100·0 (100·0-100·0)	75·4 (67·5-88·9)
Belize	45·9 (43·7-48·2)	56·8 (54·0-59·1)	57·5 (54·7-60·1)	59·7 (54·2-64·4)	41·3 (39·1-43·7)	53·0 (49·6-56·0)	53·6 (49·8-56·7)	62·4 (54·8-68·4)	50·9 (47·8-54·1)	61·1 (57·6-64·0)	61·9 (58·3-65·1)	60·7 (53·8-66·3)	22·4 (12·8-42·5)	85·5 (79·3-94·8)	100·0 (100·0-100·0)	36·6 (27·5-55·8)
Cuba	59·4 (57·2-61·6)	68·8 (66·4-71·1)	69·5 (67·2-71·7)	74·1 (69·3-77·9)	65·5 (61·4-68·5)	71·9 (69·6-74·1)	72·4 (69·9-74·6)	78·2 (69·4-82·6)	59·1 (56·2-61·9)	68·3 (65·3-71·3)	68·9 (65·8-71·9)	71·6 (65·4-76·3)	33·9 (24·6-53·3)	36·6 (27·5-55·8)	39·7 (30·7-58·6)	36·0 (26·8-55·2)
Dominica	65·2 (63·2-67·1)	54·6 (51·9-61·8)	55·1 (52·1-62·3)	56·7 (49·9-64·8)	59·3 (53·6-63·7)	61·5 (55·2-65·4)	62·2 (55·7-65·9)	63·1 (50·2-69·8)	68·8 (66·0-71·1)	53·8 (50·6-62·8)	54·1 (50·7-63·5)	55·5 (45·9-65·4)	100·0 (100·0-100·0)	1·2 (0·0-15·6)	1·5 (0·0-17·1)	3·7 (0·0-22·8)
Dominican Republic	48·4 (46·3-50·5)	57·2 (54·9-59·4)	57·3 (54·9-59·8)	59·9 (55·3-63·9)	43·0 (40·9-45·8)	51·2 (48·6-53·5)	51·9 (49·2-54·2)	60·0 (55·5-63·9)	53·1 (50·6-55·6)	61·2 (58·0-63·9)	61·0 (57·7-64·1)	58·9 (47·3-64·8)	28·1 (18·6-47·9)	59·7 (50·8-76·9)	68·3 (59·8-83·9)	35·8 (26·6-55·1)
Grenada	62·3 (60·2-64·3)	65·2 (62·7-67·9)	65·7 (62·8-68·5)	66·7 (59·8-72·4)	56·9 (54·2-60·1)	64·9 (59·9-68·6)	65·4 (60·1-69·2)	70·5 (58·0-78·5)	66·2 (63·2-68·7)	66·5 (63·3-69·6)	66·8 (63·5-70·2)	65·2 (52·8-71·7)	100·0 (100·0-100·0)	49·3 (40·3-67·7)	51·1 (42·1-69·3)	28·1 (18·6-47·9)

Location	Indicator 2.2.1: Prevalence of stunting in children under 5 (%)				Indicator 2.2.2a: Prevalence of wasting in children under 5 (%)				Indicator 2.2.2b: Prevalence of overweight in children aged 2-4 (%)				Indicator 3.1.1: Maternal mortality ratio (maternal deaths per 100,000 livebirths) in women aged 10-54 years			
	**2000**	**2015**	**2016**	**2030**	**2000**	**2015**	**2016**	**2030**	**2000**	**2015**	**2016**	**2030**	**2000**	**2015**	**2016**	**2030**
Azerbaijan	58·1 (53·6-62·3)	74·7 (63·7-84·0)	75·9 (64·3-85·4)	88·1 (71·4-97·1)	75·8 (71·9-79·5)	73·9 (65·7-80·5)	74·0 (65·2-81·0)	75·1 (59·7-81·7)	75·6 (67·3-82·5)	33·4 (19·7-46·5)	32·3 (18·1-45·4)	5·7 (0·0-27·6)	49·4 (45·8-53·2)	62·8 (56·5-69·0)	62·8 (56·2-69·4)	70·1 (54·2-85·5)
Georgia	76·8 (73·2-80·5)	82·2 (73·2-89·5)	82·4 (72·7-90·1)	85·1 (65·0-96·2)	87·1 (84·3-89·4)	89·6 (85·1-93·7)	89·7 (85·0-93·8)	90·8 (84·5-96·3)	56·7 (45·0-67·3)	39·6 (26·7-51·7)	38·3 (24·5-50·7)	25·4 (0·0-49·5)	63·6 (60·2-66·9)	56·4 (51·9-60·5)	57·0 (51·3-62·2)	66·5 (44·6-83·8)
Kazakhstan	73·8 (66·2-80·6)	85·0 (79·4-89·6)	86·3 (80·9-90·8)	93·8 (89·9-96·5)	80·5 (74·6-85·3)	80·3 (74·9-85·0)	80·7 (75·2-85·5)	84·6 (78·8-88·7)	70·6 (62·4-78·0)	51·9 (39·7-64·0)	50·9 (38·3-62·8)	34·7 (6·3-58·3)	43·6 (40·1-47·2)	71·6 (67·6-75·6)	70·2 (64·6-75·8)	95·3 (81·8-100·0)
Kyrgyzstan	56·2 (44·0-68·4)	75·5 (66·8-83·4)	77·0 (67·8-85·2)	91·2 (81·2-95·0)	87·0 (82·7-91·1)	88·1 (84·4-91·4)	87·9 (84·1-91·3)	89·8 (87·0-92·0)	65·5 (55·5-73·8)	73·1 (65·9-79·2)	72·3 (64·5-78·8)	78·3 (64·7-89·0)	46·2 (43·5-48·8)	50·4 (47·6-53·4)	50·6 (46·8-54·3)	56·3 (45·6-67·4)
Mongolia	38·1 (30·0-44·8)	74·3 (64·4-83·0)	75·2 (64·7-84·3)	86·7 (81·6-95·0)	70·4 (65·2-75·3)	88·9 (84·3-93·2)	89·4 (84·6-93·7)	94·0 (88·6-98·5)	63·0 (51·9-72·7)	59·2 (48·3-68·4)	57·8 (45·6-67·5)	53·6 (30·7-71·5)	27·1 (23·4-30·5)	49·6 (44·5-55·0)	49·5 (43·7-55·6)	65·8 (49·8-81·6)
Tajikistan	26·1 (15·3-35·5)	47·9 (33·1-61·7)	48·4 (32·4-63·1)	56·9 (39·3-79·9)	44·5 (38·1-50·8)	55·8 (44·1-66·2)	55·8 (43·4-66·8)	55·7 (39·9-72·8)	76·8 (68·7-83·9)	71·5 (62·2-79·3)	70·7 (60·6-78·5)	66·7 (46·9-81·8)	41·5 (37·3-46·3)	55·5 (48·9-62·8)	55·8 (48·9-63·2)	64·1 (47·7-81·9)
Turkmenistan	54·1 (45·8-60·5)	80·1 (74·1-85·9)	81·1 (75·8-86·3)	91·4 (89·8-92·7)	64·2 (60·3-67·8)	78·1 (72·8-82·4)	79·2 (74·1-83·4)	89·0 (85·0-93·1)	65·8 (54·9-75·6)	43·6 (29·0-56·2)	42·0 (27·5-55·1)	20·2 (0·0-44·1)	64·4 (61·4-67·2)	77·0 (73·8-80·0)	77·5 (74·2-80·6)	85·0 (76·0-94·3)
Uzbekistan	42·9 (31·7-53·1)	71·4 (57·6-82·5)	72·1 (58·2-83·0)	85·9 (68·5-95·4)	56·6 (46·1-65·9)	75·6 (64·7-84·1)	75·9 (64·8-84·6)	85·3 (73·6-93·1)	50·5 (38·2-60·6)	42·3 (28·0-54·7)	40·6 (26·1-53·7)	23·8 (0·0-51·4)	48·3 (45·5-51·1)	59·7 (55·4-63·7)	60·0 (55·3-64·8)	66·2 (48·7-82·6)
**Central Latin America**																
Colombia	67·6 (63·7-71·5)	81·0 (75·2-86·4)	81·5 (75·4-87·0)	87·4 (80·2-93·8)	94·0 (90·8-96·6)	96·1 (92·5-98·8)	96·2 (92·4-98·9)	97·7 (94·2-100·0)	84·2 (76·7-89·9)	83·8 (72·6-92·9)	83·0 (70·5-92·6)	83·0 (61·6-97·9)	38·7 (35·6-41·3)	46·7 (43·3-50·0)	47·2 (43·4-50·9)	54·2 (45·5-63·0)
Costa Rica	87·0 (81·8-91·0)	90·2 (85·9-93·4)	90·3 (86·2-93·6)	92·7 (89·7-95·3)	87·7 (81·7-92·9)	89·8 (84·3-94·3)	89·8 (84·5-94·4)	91·2 (86·5-95·3)	49·4 (27·1-68·0)	29·7 (5·2-52·4)	28·9 (5·4-51·8)	14·1 (0·0-52·9)	54·0 (51·2-56·7)	58·9 (55·2-62·4)	59·3 (55·3-63·2)	65·7 (56·7-75·9)
El Salvador	42·2 (33·8-49·7)	74·6 (67·4-81·2)	75·9 (68·8-82·4)	89·7 (85·7-93·7)	92·8 (89·2-95·9)	93·0 (88·9-96·4)	92·9 (88·8-96·3)	91·2 (87·0-94·9)	83·6 (75·7-90·1)	74·9 (59·9-87·1)	74·0 (58·2-86·6)	67·3 (34·6-90·0)	50·0 (46·3-53·6)	54·3 (49·1-59·5)	54·5 (48·9-60·3)	56·7 (43·6-70·9)
Guatemala	4·0 (0·0-12·7)	18·1 (3·3-29·4)	18·6 (3·7-29·9)	25·6 (8·5-36·9)	84·1 (80·5-87·2)	96·3 (92·7-99·1)	96·6 (92·9-99·4)	99·3 (96·4-100·0)	70·8 (64·3-76·8)	65·8 (48·4-80·6)	64·4 (45·9-80·0)	42·1 (0·0-79·2)	34·2 (29·8-38·7)	37·9 (31·7-44·3)	38·4 (30·7-46·6)	46·7 (25·9-68·3)
Honduras	26·0 (19·2-32·6)	59·1 (50·4-68·9)	59·8 (50·9-69·8)	80·6 (75·5-84·7)	93·5 (90·8-95·4)	95·1 (91·5-97·8)	95·1 (91·5-97·9)	95·9 (91·7-99·4)	88·0 (81·3-93·7)	79·2 (68·7-88·2)	78·4 (66·8-88·4)	64·4 (35·9-88·0)	29·3 (22·0-35·8)	36·1 (27·0-46·0)	36·4 (27·3-46·5)	41·7 (23·7-61·9)
Mexico	68·5 (66·6-70·2)	78·0 (76·3-79·4)	78·8 (77·1-80·2)	85·6 (83·9-87·1)	87·4 (86·1-88·7)	90·9 (89·7-92·0)	91·2 (90·0-92·3)	94·9 (93·9-95·8)	53·7 (32·6-71·8)	59·4 (41·3-74·1)	58·1 (39·1-73·8)	53·8 (11·7-83·3)	47·6 (46·0-49·1)	50·3 (48·7-51·8)	50·5 (48·8-52·1)	53·5 (48·8-57·8)
Nicaragua	54·0 (48·4-59·3)	70·9 (63·3-77·4)	71·6 (64·0-78·1)	82·6 (74·6-87·3)	88·9 (85·7-91·7)	92·6 (88·9-95·9)	92·7 (89·0-96·0)	94·0 (89·6-97·8)	73·7 (66·6-79·8)	65·1 (45·5-80·3)	63·9 (42·3-79·7)	42·5 (0·0-76·9)	37·5 (34·4-40·7)	44·3 (38·8-49·8)	44·9 (38·6-51·3)	50·6 (35·2-65·1)
Panama	54·8 (47·6-61·6)	71·6 (64·1-78·5)	72·5 (64·7-79·5)	82·5 (72·2-90·3)	93·9 (89·7-97·2)	95·0 (90·6-98·4)	95·1 (90·7-98·6)	96·7 (92·1-100·0)	84·5 (71·7-93·9)	72·7 (57·2-85·9)	71·0 (55·5-84·8)	58·0 (20·0-86·7)	45·1 (41·6-48·4)	41·7 (37·4-46·0)	42·7 (37·7-47·8)	49·8 (34·7-64·0)
Venezuela	71·5 (66·1-76·2)	81·5 (75·0-87·0)	81·9 (75·4-87·3)	87·4 (81·0-92·1)	81·8 (77·7-85·6)	76·3 (66·1-84·2)	76·2 (65·5-84·5)	73·5 (55·7-84·8)	52·1 (31·8-71·0)	29·7 (4·9-52·5)	28·8 (4·4-52·4)	14·0 (0·0-52·5)	46·0 (42·8-49·0)	43·1 (37·7-48·4)	43·1 (37·1-49·2)	43·9 (28·8-58·3)
**Andean Latin America**																
Bolivia	37·9 (21·2-51·2)	68·0 (53·7-79·1)	69·4 (54·7-80·5)	83·8 (68·3-93·8)	88·0 (83·2-92·2)	93·2 (88·1-97·4)	93·4 (88·2-97·6)	96·6 (91·8-100·0)	51·5 (43·9-58·7)	48·6 (35·1-60·3)	46·9 (33·1-58·9)	36·6 (8·2-61·9)	21·3 (18·3-24·7)	28·3 (21·2-35·6)	28·8 (21·4-36·3)	38·0 (21·9-53·4)
Ecuador	45·5 (33·4-55·6)	63·2 (49·8-74·6)	64·1 (50·5-75·6)	74·9 (62·5-87·0)	88·8 (85·4-91·8)	92·7 (87·9-96·7)	92·8 (88·0-96·8)	94·5 (88·4-98·9)	73·1 (64·3-81·0)	65·9 (56·0-74·1)	64·7 (54·7-73·7)	54·9 (32·5-73·0)	36·6 (34·3-38·9)	45·7 (42·7-48·6)	46·2 (43·1-49·2)	50·2 (42·3-58·6)
Peru	44·5 (32·5-55·7)	66·0 (55·7-75·3)	67·9 (57·1-77·6)	86·9 (76·6-91·4)	93·9 (89·2-97·3)	96·5 (91·5-99·6)	96·7 (91·7-99·8)	98·8 (94·6-100·0)	51·9 (44·4-59·2)	63·9 (54·2-71·9)	62·3 (51·8-70·9)	58·2 (32·8-77·9)	25·4 (22·1-28·8)	41·3 (36·2-46·7)	42·6 (36·8-49·1)	60·5 (45·4-76·4)
**Caribbean**																
Antigua and Barbuda	87·8 (83·0-92·1)	89·1 (84·6-93·0)	89·2 (84·7-93·1)	90·6 (86·2-94·2)	89·4 (85·1-93·0)	91·2 (87·6-94·6)	91·3 (87·7-94·6)	92·3 (89·1-95·5)	59·6 (37·8-75·9)	44·6 (21·0-65·2)	43·5 (20·2-64·1)	27·1 (0·0-65·6)	55·2 (51·4-59·0)	53·7 (48·8-58·3)	54·6 (49·4-59·2)	54·7 (43·9-65·0)
The Bahamas	89·3 (84·6-93·0)	89·2 (84·6-93·0)	89·2 (84·6-93·0)	89·3 (84·6-93·2)	89·9 (85·4-93·6)	91·2 (87·2-94·6)	91·3 (87·2-94·7)	92·1 (88·1-95·2)	54·9 (34·5-72·3)	60·1 (38·8-76·7)	59·8 (38·5-76·5)	59·8 (20·5-85·8)	45·3 (41·9-48·6)	45·5 (41·6-49·4)	45·3 (41·1-49·8)	47·9 (35·0-60·2)
Barbados	87·8 (82·6-91·9)	88·7 (84·2-92·5)	88·8 (84·2-92·6)	89·7 (85·5-93·6)	72·4 (64·4-78·9)	74·7 (68·1-80·5)	74·9 (68·2-80·6)	76·9 (71·0-82·1)	56·2 (36·5-73·0)	63·1 (46·1-76·1)	62·5 (44·7-76·4)	61·9 (26·8-87·8)	48·9 (45·4-52·5)	45·7 (41·7-49·7)	46·3 (42·0-50·6)	56·8 (44·2-70·0)
Belize	68·1 (58·4-76·3)	74·8 (72·1-77·4)	76·1 (73·8-78·4)	82·9 (81·1-84·7)	90·2 (86·6-93·5)	88·6 (85·7-91·2)	88·6 (85·7-91·3)	87·9 (85·5-90·4)	69·2 (51·3-82·6)	69·5 (52·6-82·4)	69·2 (51·9-82·6)	64·4 (28·4-88·6)	46·6 (42·7-50·0)	68·6 (63·2-73·9)	68·5 (62·6-74·3)	78·5 (62·9-95·6)
Cuba	91·2 (88·6-93·6)	86·8 (82·2-90·6)	86·2 (81·6-90·1)	75·8 (71·5-80·3)	89·9 (87·1-92·2)	91·5 (87·4-94·7)	91·4 (87·2-94·8)	91·0 (86·0-95·1)	71·8 (55·0-84·1)	56·9 (46·6-67·0)	55·9 (44·0-67·2)	44·3 (15·1-69·2)	49·3 (46·6-52·1)	51·8 (48·4-55·3)	52·2 (48·4-55·8)	57·7 (47·5-67·8)
Dominica	84·3 (78·1-89·6)	86·4 (80·9-91·1)	86·5 (81·1-91·1)	88·4 (83·7-92·5)	87·2 (81·9-91·3)	89·7 (85·4-93·2)	89·7 (85·5-93·2)	91·2 (87·5-94·4)	56·9 (36·9-74·5)	48·6 (27·2-68·5)	48·1 (26·3-68·1)	39·1 (0·0-73·7)	64·3 (60·4-68·1)	69·3 (64·4-73·7)	68·2 (63·0-73·1)	88·3 (71·4-100·0)
Dominican Republic	83·0 (79·5-85·9)	85·9 (81·1-89·9)	86·6 (81·8-90·5)	93·4 (89·3-96·5)	90·7 (87·9-93·0)	90·4 (87·4-93·0)	90·4 (87·4-93·1)	91·1 (87·2-93·8)	78·2 (70·2-85·4)	60·4 (39·4-75·7)	58·9 (38·7-74·4)	36·1 (0·0-69·0)	45·8 (43·1-48·7)	43·8 (38·2-49·2)	44·2 (38·6-50·0)	46·6 (33·3-60·3)
Grenada	80·9 (73·9-87·1)	85·7 (80·0-90·3)	85·9 (80·3-90·4)	89·8 (85·3-92·9)	86·8 (82·1-90·9)	90·1 (86·2-93·4)	90·2 (86·3-93·5)	91·7 (88·2-94·7)	64·2 (45·6-79·0)	54·2 (32·4-72·2)	53·3 (31·2-71·5)	40·9 (0·0-75·0)	59·0 (54·3-63·8)	59·8 (53·8-65·6)	56·7 (50·4-62·8)	60·1 (46·2-75·3)

Location	Indicator 3.1.2: Proportion of births attended by skilled health personnel (%)				Indicator 3.2.1: Under-5 mortality rate (probability of dying before the age of 5 per 1,000 livebirths)				Indicator 3.2.2: Neonatal mortality rate (probability of dying during the first 28 days of life per 1,000 livebirths)				Indicator 3.3.1: Age-standardised rate of new HIV infections (per 1,000 population)			
	**2000**	**2015**	**2016**	**2030**	**2000**	**2015**	**2016**	**2030**	**2000**	**2015**	**2016**	**2030**	**2000**	**2015**	**2016**	**2030**
Azerbaijan	85·1 (81·9-88·2)	92·6 (75·5-99·0)	92·9 (75·5-99·2)	95·4 (55·7-100·0)	23·5 (19·2-27·7)	40·0 (32·8-47·6)	40·9 (33·5-48·3)	56·7 (37·6-76·2)	12·9 (7·4-18·0)	27·1 (19·6-34·8)	28·0 (20·6-35·6)	42·6 (21·2-64·5)	75·2 (68·3-81·7)	71·3 (63·6-79·5)	71·1 (63·4-79·6)	72·5 (65·4-80·6)
Georgia	96·9 (95·6-97·9)	98·9 (97·5-99·7)	98·9 (97·1-99·8)	98·9 (91·9-100·0)	35·2 (32·3-38·5)	55·2 (48·9-61·5)	56·3 (49·7-62·7)	73·5 (59·4-86·9)	21·7 (18·4-25·2)	41·5 (34·2-49·0)	42·7 (35·1-50·3)	62·1 (46·6-77·9)	91·2 (85·7-95·6)	71·8 (65·0-78·1)	71·1 (64·3-77·7)	69·3 (62·8-75·4)
Kazakhstan	99·1 (98·2-99·7)	99·5 (98·1-100·0)	99·4 (97·8-100·0)	99·2 (92·5-100·0)	35·9 (31·8-40·3)	58·5 (52·1-64·9)	59·4 (52·7-66·0)	82·3 (65·0-100·0)	30·9 (24·9-37·2)	51·4 (45·2-57·9)	52·6 (45·6-59·8)	76·3 (55·2-99·0)	63·8 (58·1-69·7)	58·6 (51·1-66·4)	57·2 (49·5-65·3)	58·0 (50·4-65·9)
Kyrgyzstan	97·5 (95·0-99·1)	99·5 (98·8-99·9)	99·5 (98·7-99·9)	99·7 (97·4-100·0)	28·9 (26·8-30·9)	40·7 (37·1-44·3)	41·5 (37·5-45·4)	52·5 (43·7-61·0)	17·3 (15·7-19·0)	27·0 (23·3-31·1)	27·9 (23·8-32·3)	37·4 (28·5-46·3)	61·6 (56·2-67·2)	55·1 (48·2-62·3)	53·6 (45·9-61·3)	53·0 (46·0-60·4)
Mongolia	98·0 (97·3-98·6)	98·8 (97·2-99·6)	98·8 (96·9-99·7)	98·6 (89·2-100·0)	25·5 (23·2-27·9)	47·7 (43·0-52·4)	48·8 (43·6-54·0)	68·9 (56·5-81·4)	20·1 (16·3-23·9)	37·4 (33·9-41·3)	38·6 (34·8-42·8)	54·9 (44·7-66·6)	99·9 (98·3-100·0)	89·7 (72·8-100·0)	90·1 (73·3-100·0)	90·8 (74·6-100·0)
Tajikistan	76·8 (57·8-89·5)	91·4 (75·9-98·2)	91·5 (73·1-98·5)	90·9 (28·4-100·0)	19·5 (15·9-22·9)	34·9 (28·2-42·0)	35·7 (28·5-42·8)	46·9 (27·7-65·5)	14·5 (10·0-18·8)	27·0 (20·3-34·1)	27·7 (20·7-34·9)	37·3 (16·8-57·4)	63·1 (57·0-68·8)	70·8 (65·3-77·1)	71·0 (65·1-77·5)	68·9 (62·8-75·8)
Turkmenistan	97·1 (95·7-98·2)	99·4 (97·5-100·0)	99·4 (97·3-100·0)	99·4 (93·5-100·0)	17·8 (12·8-22·9)	34·8 (27·0-43·1)	36·1 (27·8-44·5)	55·5 (35·4-75·2)	11·9 (6·4-17·7)	26·4 (16·9-36·0)	27·7 (18·3-37·3)	44·7 (22·0-67·8)	54·0 (48·1-59·2)	63·0 (56·5-69·1)	62·3 (55·8-68·6)	62·8 (56·8-68·6)
Uzbekistan	98·0 (96·0-99·1)	99·8 (98·8-100·0)	99·8 (98·9-100·0)	99·7 (96·3-100·0)	32·6 (28·2-37·0)	45·6 (38·2-52·9)	46·6 (39·2-53·9)	62·4 (41·9-82·0)	25·7 (20·7-30·7)	36·0 (27·7-44·3)	37·0 (28·6-45·5)	50·6 (26·7-73·1)	60·3 (54·3-66·0)	88·1 (78·1-100·0)	89·0 (79·2-100·0)	90·0 (80·2-100·0)
**Central Latin America**																
Colombia	85·0 (83·1-86·6)	96·8 (94·4-98·3)	97·0 (94·7-98·5)	99·1 (96·3-100·0)	43·4 (40·4-46·3)	55·1 (52·3-57·4)	56·0 (52·9-58·2)	69·8 (60·1-78·3)	32·0 (28·5-35·7)	43·3 (39·9-46·1)	44·4 (40·8-47·3)	60·3 (49·7-70·0)	58·6 (53·0-65·0)	53·9 (47·4-60·8)	54·2 (47·7-61·0)	54·9 (48·9-61·4)
Costa Rica	97·8 (96·1-99·0)	99·0 (97·9-99·6)	99·1 (98·0-99·7)	99·6 (97·9-100·0)	56·2 (51·9-59·7)	64·2 (60·3-66·9)	64·9 (60·9-68·0)	73·4 (63·0-82·5)	44·7 (40·3-48·8)	51·8 (47·4-55·5)	52·8 (48·0-56·9)	61·4 (48·9-72·4)	59·8 (55·5-64·9)	56·4 (50·8-62·7)	56·7 (51·1-62·9)	57·7 (52·7-63·3)
El Salvador	99·0 (98·5-99·4)	99·5 (98·8-99·9)	99·5 (98·9-99·9)	99·8 (98·8-100·0)	41·2 (37·6-44·8)	60·8 (57·4-63·5)	62·2 (59·0-64·4)	80·2 (68·9-90·3)	35·6 (31·6-39·5)	56·3 (51·8-60·1)	57·9 (53·4-61·3)	78·5 (64·8-90·2)	45·7 (41·4-50·3)	58·4 (53·2-64·0)	60·7 (55·2-66·6)	60·2 (54·7-66·1)
Guatemala	36·2 (32·4-40·0)	56·5 (39·0-72·0)	58·4 (39·7-74·6)	79·7 (43·7-100·0)	28·3 (26·8-29·8)	42·3 (40·0-43·9)	43·3 (40·9-45·1)	56·7 (51·8-61·1)	28·6 (26·6-30·8)	38·5 (34·7-42·1)	39·5 (35·5-43·2)	49·5 (39·9-58·3)	46·7 (40·8-52·6)	54·5 (44·7-63·6)	54·0 (43·5-63·9)	53·2 (42·9-63·4)
Honduras	60·2 (50·9-68·8)	94·4 (90·5-97·1)	94·3 (89·9-97·2)	98·7 (93·2-100·0)	35·9 (33·9-38·1)	47·6 (43·8-51·2)	48·6 (44·5-52·2)	60·6 (53·3-66·7)	27·7 (25·2-30·3)	36·7 (32·0-41·0)	37·6 (32·7-42·1)	46·0 (37·1-53·7)	48·6 (44·4-55·2)	52·8 (47·3-59·2)	53·9 (48·0-60·8)	52·7 (46·5-59·9)
Mexico	90·6 (88·1-92·8)	97·6 (95·4-98·9)	97·7 (95·7-99·0)	98·9 (95·4-100·0)	42·5 (39·7-45·4)	55·2 (51·6-58·1)	56·1 (52·2-59·1)	68·2 (58·8-76·3)	37·3 (34·3-40·4)	48·9 (44·4-52·7)	49·8 (45·0-53·7)	61·7 (51·3-71·0)	58·3 (54·7-62·5)	51·9 (46·7-57·8)	51·9 (46·7-57·8)	52·3 (47·5-58·0)
Nicaragua	89·8 (86·8-92·2)	95·6 (91·8-97·9)	95·7 (92·2-98·1)	97·7 (91·6-99·9)	36·3 (34·3-38·4)	55·4 (51·0-59·4)	56·7 (52·1-60·8)	74·7 (66·3-82·2)	32·1 (29·5-35·0)	48·2 (43·7-52·1)	49·7 (45·0-53·9)	65·3 (55·9-73·6)	64·9 (59·3-70·6)	54·3 (47·3-61·9)	54·0 (46·3-62·1)	54·5 (46·8-62·0)
Panama	88·3 (85·7-90·5)	92·1 (88·1-95·0)	92·7 (88·5-95·6)	96·4 (89·1-99·9)	46·7 (43·2-49·6)	53·7 (50·3-56·4)	54·4 (50·7-57·4)	61·3 (52·4-69·1)	41·6 (38·0-44·8)	49·8 (45·1-53·5)	50·5 (45·4-54·6)	58·8 (47·0-68·9)	43·5 (40·0-47·6)	37·4 (31·5-43·6)	37·4 (31·8-43·6)	41·6 (35·7-48·2)
Venezuela	96·9 (94·9-98·3)	96·7 (93·6-98·5)	96·8 (93·6-98·6)	96·9 (87·3-100·0)	48·4 (46·3-50·6)	54·0 (52·3-55·6)	54·0 (52·6-55·5)	55·0 (52·4-57·7)	38·3 (36·2-40·6)	41·4 (39·9-42·9)	41·4 (40·0-42·9)	42·0 (37·8-46·2)	49·5 (44·4-54·8)	47·4 (42·3-53·0)	47·6 (42·5-53·3)	47·2 (42·2-53·1)
**Andean Latin America**																
Bolivia	53·9 (50·9-57·0)	77·9 (71·4-82·9)	79·0 (72·6-84·1)	91·1 (82·9-96·0)	20·1 (18·5-21·7)	36·9 (32·6-40·6)	38·0 (33·5-41·9)	54·6 (45·6-63·0)	15·2 (12·9-17·6)	29·3 (24·1-34·1)	30·4 (24·9-35·6)	44·6 (33·2-55·6)	59·1 (43·4-76·3)	55·9 (38·4-74·2)	56·5 (38·9-74·8)	56·2 (38·8-74·7)
Ecuador	95·5 (93·9-96·7)	98·6 (97·9-99·1)	98·7 (98·0-99·2)	99·7 (98·9-100·0)	38·6 (36·5-40·4)	53·0 (50·6-54·8)	53·5 (50·8-55·4)	67·0 (60·9-72·2)	29·8 (27·3-32·4)	45·9 (42·6-48·5)	46·9 (43·6-49·5)	62·4 (55·8-68·3)	56·2 (50·0-62·0)	51·1 (46·1-56·5)	52·3 (47·3-57·8)	52·8 (48·0-58·6)
Peru	59·5 (53·8-64·5)	89·7 (86·8-92·1)	90·5 (87·7-93·0)	97·4 (95·2-98·8)	35·0 (32·8-37·2)	53·4 (50·4-55·7)	54·4 (50·8-57·2)	71·0 (59·6-80·2)	29·9 (26·8-33·2)	46·8 (42·5-50·4)	48·1 (43·1-52·3)	64·9 (51·2-76·7)	59·1 (54·9-63·5)	62·8 (56·3-69·7)	63·2 (56·2-70·2)	63·1 (56·4-70·3)
**Caribbean**																
Antigua and Barbuda	98·7 (97·2-99·5)	99·0 (97·4-99·8)	99·1 (97·2-99·8)	99·1 (93·8-100·0)	54·9 (49·2-60·5)	63·5 (56·4-69·5)	63·9 (56·8-70·1)	70·7 (54·6-85·0)	42·3 (34·8-49·8)	56·5 (47·0-64·6)	57·0 (47·1-65·5)	68·9 (48·3-87·7)	47·0 (43·7-50·8)	52·7 (46·5-58·3)	52·9 (46·7-58·7)	60·3 (54·1-66·1)
The Bahamas	98·7 (97·0-99·6)	98·7 (97·0-99·7)	98·8 (97·0-99·7)	98·6 (91·5-100·0)	58·7 (50·2-68·1)	63·9 (50·2-75·8)	65·0 (49·8-77·9)	76·6 (44·6-100·0)	53·5 (43·8-64·0)	59·0 (43·4-73·1)	59·6 (42·8-74·7)	70·8 (33·7-100·0)	30·2 (27·6-33·1)	36·9 (30·9-42·3)	37·4 (31·2-42·7)	46·2 (40·8-51·8)
Barbados	97·5 (94·8-99·1)	98·7 (96·6-99·7)	98·7 (96·7-99·7)	98·9 (92·5-100·0)	53·3 (42·5-64·1)	57·9 (46·9-68·1)	58·0 (46·3-68·7)	65·9 (36·3-92·9)	37·8 (26·2-49·7)	42·5 (29·3-54·7)	42·8 (28·7-55·8)	51·5 (16·1-83·0)	43·3 (39·0-47·5)	45·5 (37·9-53·4)	45·9 (37·5-54·4)	47·3 (38·6-55·9)
Belize	91·3 (87·2-94·3)	95·8 (92·1-98·0)	95·9 (91·8-98·2)	97·3 (84·8-100·0)	42·3 (36·8-48·6)	57·0 (48·0-65·4)	57·3 (47·3-66·4)	71·9 (42·9-100·0)	32·4 (26·3-39·3)	46·8 (35·8-57·2)	47·2 (34·6-58·0)	64·2 (31·2-96·9)	27·2 (19·9-48·6)	36·0 (30·9-41·1)	35·8 (30·8-41·1)	41·3 (37·2-46·0)
Cuba	98·7 (97·2-99·5)	99·1 (98·2-99·7)	99·1 (98·2-99·7)	99·4 (95·9-100·0)	69·4 (66·4-72·3)	80·2 (76·7-83·5)	80·8 (77·1-84·0)	89·1 (83·8-93·9)	64·5 (61·2-67·5)	78·9 (75·3-81·9)	79·5 (76·0-82·5)	91·4 (84·8-97·3)	66·0 (60·2-73·1)	40·5 (34·4-48·4)	38·9 (32·8-46·7)	39·7 (33·5-47·8)
Dominica	98·1 (96·0-99·3)	98·3 (95·3-99·6)	98·3 (95·6-99·6)	98·2 (87·7-100·0)	51·9 (45·1-58·8)	42·9 (33·1-50·7)	45·4 (36·5-53·5)	41·8 (20·5-60·4)	36·2 (29·1-43·6)	25·0 (14·9-34·2)	26·0 (15·6-35·7)	18·1 (0·0-39·7)	50·8 (47·4-54·7)	54·3 (48·6-59·6)	54·5 (48·5-60·1)	54·8 (49·3-60·2)
Dominican Republic	97·8 (96·8-98·6)	96·0 (92·8-97·9)	95·9 (92·1-98·1)	93·0 (67·2-100·0)	35·5 (32·8-38·3)	41·8 (38·2-45·2)	42·8 (38·8-46·2)	53·3 (40·5-64·8)	21·6 (18·7-24·6)	25·5 (20·5-30·5)	26·5 (20·9-31·5)	34·4 (19·0-48·5)	24·2 (20·7-28·8)	32·9 (29·0-37·0)	33·1 (28·9-37·6)	40·3 (35·6-45·3)
Grenada	98·7 (97·3-99·5)	98·9 (97·1-99·7)	98·9 (96·9-99·8)	98·9 (90·8-100·0)	56·5 (47·5-66·3)	55·7 (42·6-67·6)	57·1 (42·5-69·5)	60·6 (26·4-93·1)	44·2 (33·1-55·8)	41·5 (27·2-54·7)	43·0 (27·7-56·7)	45·0 (6·3-82·2)	49·5 (45·9-53·7)	54·3 (48·3-59·8)	54·7 (48·6-60·4)	55·9 (50·9-60·9)

Location	Indicator 3.3.2: Age-standardised rate of tuberculosis cases (per 100,000 population)				Indicator 3.3.3: Age-standardised rate of malaria cases (per 1,000 population)				Indicator 3.3.4: Age-standardised rate of hepatitis B incidence (per 100,000 population)				Indicator 3.3.5: Age-standardised prevalence* of the sum of 15 neglected tropical diseases (NTDs) (%) *Prevalence estimates reported here may exceed 100% as they reflect the sum of prevalent cases of 15 NTDs.			
	**2000**	**2015**	**2016**	**2030**	**2000**	**2015**	**2016**	**2030**	**2000**	**2015**	**2016**	**2030**	**2000**	**2015**	**2016**	**2030**
Azerbaijan	30·7 (28·7-32·6)	39·8 (37·5-42·1)	40·2 (37·8-42·4)	46·6 (43·9-49·0)	72·5 (68·0-75·4)	100·0 (100·0-100·0)	100·0 (100·0-100·0)	100·0 (100·0-100·0)	42·0 (38·4-45·4)	46·9 (44·4-49·2)	47·7 (45·2-49·9)	59·0 (55·5-61·9)	96·8 (95·4-97·7)	97·1 (96·0-97·9)	97·1 (96·0-97·9)	97·2 (96·1-98·0)
Georgia	46·6 (44·7-48·4)	49·0 (47·1-51·0)	49·7 (47·7-51·7)	59·0 (56·0-61·6)	81·1 (76·5-84·1)	100·0 (100·0-100·0)	100·0 (100·0-100·0)	100·0 (100·0-100·0)	60·3 (57·2-63·6)	48·5 (46·1-51·3)	47·9 (45·5-50·6)	40·1 (36·5-43·6)	99·4 (99·3-99·6)	99·5 (99·3-99·6)	99·5 (99·3-99·6)	99·5 (99·3-99·6)
Kazakhstan	33·4 (31·4-35·4)	45·5 (43·7-47·2)	46·8 (45·0-48·5)	59·6 (57·3-61·9)	100·0 (100·0-100·0)	100·0 (100·0-100·0)	100·0 (100·0-100·0)	100·0 (100·0-100·0)	30·5 (28·1-33·3)	59·1 (56·8-61·9)	60·4 (58·1-63·2)	79·0 (76·4-82·0)	79·9 (73·7-84·4)	79·8 (72·7-84·9)	79·8 (72·7-84·9)	79·8 (72·7-84·9)
Kyrgyzstan	37·2 (35·4-38·9)	41·2 (39·5-42·9)	41·0 (39·3-42·7)	39·9 (37·1-42·8)	66·9 (62·7-69·6)	100·0 (100·0-100·0)	100·0 (100·0-100·0)	100·0 (100·0-100·0)	22·4 (19·2-25·6)	38·8 (36·1-41·7)	39·6 (36·8-42·5)	53·3 (49·7-56·6)	79·3 (72·7-84·4)	79·1 (72·4-84·5)	79·1 (72·4-84·5)	79·2 (72·4-84·6)
Mongolia	27·3 (25·5-29·1)	34·4 (32·2-36·3)	35·1 (32·9-37·0)	43·9 (41·4-46·1)	100·0 (100·0-100·0)	100·0 (100·0-100·0)	100·0 (100·0-100·0)	100·0 (100·0-100·0)	5·5 (1·7-9·2)	32·3 (28·2-36·5)	32·9 (28·8-37·2)	41·8 (36·2-48·7)	99·5 (99·2-99·7)	99·7 (99·5-99·7)	99·7 (99·6-99·7)	99·8 (99·7-99·8)
Tajikistan	37·6 (35·9-39·3)	47·3 (45·3-49·7)	48·2 (46·1-50·5)	59·9 (57·3-63·4)	28·5 (24·4-36·5)	95·4 (92·4-96·9)	97·2 (95·0-98·2)	100·0 (100·0-100·0)	12·3 (8·8-15·7)	26·3 (23·6-28·9)	26·6 (23·9-29·2)	32·7 (30·2-35·5)	91·3 (88·4-93·5)	91·5 (88·4-93·6)	91·5 (88·4-93·6)	91·6 (88·5-93·8)
Turkmenistan	27·3 (25·4-29·1)	34·6 (32·6-36·8)	35·2 (33·2-37·3)	42·8 (40·4-45·5)	97·5 (95·5-98·4)	100·0 (100·0-100·0)	100·0 (100·0-100·0)	100·0 (100·0-100·0)	10·1 (7·1-13·3)	27·4 (25·1-30·0)	28·3 (26·0-30·9)	41·8 (38·4-45·6)	97·3 (96·2-98·0)	97·4 (96·4-98·1)	97·4 (96·4-98·1)	97·4 (96·5-98·1)
Uzbekistan	45·0 (43·1-46·8)	50·5 (48·6-52·5)	50·9 (49·0-52·8)	57·1 (54·2-60·3)	96·8 (94·4-97·9)	100·0 (100·0-100·0)	100·0 (100·0-100·0)	100·0 (100·0-100·0)	16·2 (12·5-19·8)	33·2 (30·0-36·0)	34·1 (30·8-37·0)	47·8 (43·7-51·7)	99·6 (99·3-99·8)	99·7 (99·6-99·8)	99·7 (99·6-99·8)	99·8 (99·7-99·9)
**Central Latin America**																
Colombia	64·3 (62·4-66·2)	71·1 (69·2-73·1)	71·5 (69·6-73·5)	77·0 (74·9-79·0)	27·1 (25·1-29·9)	43·3 (40·2-45·5)	44·8 (41·6-47·0)	59·4 (54·5-62·5)	51·0 (47·4-54·5)	72·7 (69·2-76·4)	73·3 (69·9-76·9)	81·5 (78·7-84·6)	51·3 (46·0-56·0)	51·3 (45·4-56·6)	51·3 (45·4-56·6)	51·6 (45·7-56·9)
Costa Rica	75·9 (74·0-77·8)	86·0 (84·1-87·9)	86·5 (84·6-88·4)	94·0 (92·0-96·0)	85·5 (80·8-88·3)	100·0 (100·0-100·0)	100·0 (100·0-100·0)	100·0 (100·0-100·0)	60·9 (58·4-62·9)	70·5 (68·2-72·4)	71·0 (68·6-72·9)	78·0 (75·3-80·2)	64·6 (56·1-71·2)	64·5 (56·0-71·0)	64·5 (56·0-71·0)	64·7 (56·3-71·1)
El Salvador	60·6 (58·8-62·4)	69·9 (68·1-71·7)	70·2 (68·4-72·0)	78·5 (76·0-81·0)	82·3 (77·6-85·2)	100·0 (100·0-100·0)	100·0 (100·0-100·0)	100·0 (100·0-100·0)	83·6 (81·0-86·0)	85·0 (82·6-87·2)	85·2 (82·7-87·4)	88·1 (85·3-90·7)	61·3 (56·5-65·5)	83·2 (79·3-86·3)	85·2 (81·7-87·9)	97·4 (96·7-97·9)
Guatemala	62·2 (60·4-64·0)	68·7 (66·8-70·6)	69·1 (67·2-71·0)	75·5 (73·6-77·5)	22·0 (18·7-25·5)	50·0 (46·9-52·1)	51·6 (48·3-53·8)	76·2 (68·3-81·0)	78·7 (76·2-81·5)	62·2 (60·0-64·1)	63·3 (61·1-65·2)	78·2 (76·0-80·0)	0·3 (0·0-3·8)	35·7 (25·9-44·6)	39·1 (29·8-47·6)	71·4 (67·3-75·2)
Honduras	49·2 (47·6-51·0)	56·4 (54·7-58·3)	56·8 (55·0-58·7)	62·0 (60·2-63·9)	28·6 (25·4-31·5)	49·6 (46·3-51·7)	51·1 (47·9-53·4)	70·9 (66·6-73·9)	49·9 (45·4-54·2)	60·1 (56·9-63·1)	60·7 (57·7-63·6)	70·0 (68·0-71·9)	38·1 (25·6-48·7)	48·7 (39·3-56·9)	49·7 (40·4-57·8)	61·6 (54·5-67·8)
Mexico	65·1 (63·2-67·2)	73·5 (71·5-75·5)	73·2 (71·3-75·2)	72·2 (69·5-74·5)	59·0 (55·3-61·6)	82·5 (77·8-85·4)	83·8 (79·1-86·7)	97·3 (95·1-98·2)	52·1 (48·8-55·3)	70·5 (67·9-73·0)	70·8 (68·3-73·3)	75·9 (73·8-78·3)	77·1 (73·3-80·4)	77·6 (75·3-79·5)	77·6 (75·4-79·6)	78·6 (76·4-80·5)
Nicaragua	51·2 (49·4-52·9)	60·7 (58·8-62·5)	61·0 (59·1-62·8)	65·2 (63·2-67·0)	27·6 (25·5-29·9)	53·4 (50·0-55·6)	52·3 (49·0-54·5)	78·5 (71·1-84·7)	44·8 (40·6-49·4)	62·6 (59·9-65·5)	63·5 (60·9-66·2)	77·0 (74·7-79·0)	70·5 (66·8-73·9)	86·3 (82·7-89·2)	87·8 (84·5-90·4)	97·4 (96·8-97·9)
Panama	57·4 (55·7-59·0)	59·9 (58·3-61·5)	60·1 (58·6-61·7)	63·8 (62·2-65·5)	47·4 (43·7-50·8)	59·8 (55·8-62·3)	59·6 (55·8-62·1)	61·4 (48·1-73·3)	58·6 (56·2-60·8)	72·7 (70·6-74·7)	73·2 (71·0-75·1)	79·8 (77·6-81·8)	86·5 (83·8-88·8)	89·3 (86·4-91·5)	89·3 (86·5-91·6)	90·3 (87·6-92·4)
Venezuela	65·4 (63·6-67·2)	70·1 (68·2-72·0)	70·1 (68·3-72·0)	75·2 (72·7-77·6)	39·5 (35·5-42·8)	30·2 (27·0-33·2)	28·6 (25·4-31·8)	19·8 (17·8-22·0)	51·9 (49·3-54·1)	61·9 (59·6-64·0)	62·0 (59·7-64·1)	63·1 (60·6-65·4)	36·2 (30·1-41·6)	39·1 (27·2-48·9)	39·6 (27·8-49·3)	46·5 (35·7-55·2)
**Andean Latin America**																
Bolivia	32·0 (30·2-33·8)	42·9 (41·0-44·8)	43·4 (41·6-45·4)	52·5 (50·5-54·5)	29·4 (26·0-31·3)	44·8 (40·3-47·1)	44·9 (40·7-47·4)	48·5 (44·0-52·4)	44·8 (42·6-47·0)	58·5 (56·7-60·3)	59·4 (57·5-61·1)	71·5 (69·0-73·5)	71·0 (67·2-74·1)	71·5 (67·4-74·8)	71·8 (67·7-75·1)	75·4 (71·4-78·5)
Ecuador	50·6 (48·8-52·6)	62·8 (60·9-64·8)	63·0 (61·1-64·9)	69·8 (67·2-72·1)	24·0 (22·1-26·3)	67·3 (63·9-70·6)	69·1 (65·6-72·5)	93·3 (90·2-95·4)	62·4 (60·4-64·4)	72·1 (70·3-74·0)	72·7 (70·9-74·6)	81·9 (80·0-83·8)	32·1 (23·3-39·9)	44·8 (34·4-54·0)	46·1 (35·9-55·0)	60·9 (53·4-67·4)
Peru	36·6 (34·7-38·6)	52·5 (50·6-54·6)	53·4 (51·5-55·5)	66·3 (64·2-68·6)	31·7 (28·8-34·3)	36·1 (33·1-38·7)	35·5 (32·4-38·1)	39·1 (36·1-41·3)	53·8 (51·5-55·8)	73·2 (71·2-75·0)	74·3 (72·3-76·0)	89·9 (87·8-91·9)	59·6 (51·5-66·0)	70·8 (63·1-76·9)	72·0 (64·6-77·8)	84·2 (80·2-87·4)
**Caribbean**																
Antigua and Barbuda	76·5 (74·6-78·4)	82·5 (80·7-84·3)	82·7 (80·9-84·5)	86·3 (84·3-88·1)	100·0 (100·0-100·0)	100·0 (100·0-100·0)	100·0 (100·0-100·0)	100·0 (100·0-100·0)	73·7 (71·4-75·8)	83·0 (81·1-84·9)	83·6 (81·7-85·4)	90·9 (89·2-92·6)	97·0 (95·1-99·7)	96·9 (95·1-99·7)	96·8 (95·0-99·6)	96·4 (94·3-99·5)
The Bahamas	53·1 (51·4-54·7)	60·0 (58·4-61·4)	60·0 (58·4-61·5)	61·8 (59·9-63·5)	100·0 (100·0-100·0)	100·0 (100·0-100·0)	100·0 (100·0-100·0)	100·0 (100·0-100·0)	73·9 (70·9-76·5)	78·7 (76·0-81·3)	78·7 (76·0-81·3)	79·2 (76·1-82·0)	99·8 (99·8-99·8)	99·7 (99·6-99·7)	99·7 (99·6-99·7)	99·5 (99·3-99·6)
Barbados	78·5 (76·6-80·2)	83·1 (81·4-84·8)	83·4 (81·6-85·0)	86·5 (84·7-88·3)	100·0 (100·0-100·0)	100·0 (100·0-100·0)	100·0 (100·0-100·0)	100·0 (100·0-100·0)	73·8 (70·8-76·3)	80·0 (77·4-82·4)	80·0 (77·5-82·4)	80·5 (77·9-82·8)	99·7 (99·6-99·8)	99·6 (99·5-99·7)	99·6 (99·5-99·7)	99·5 (99·2-99·6)
Belize	51·8 (50·2-53·4)	59·4 (57·8-61·0)	60·3 (58·6-61·9)	70·3 (68·5-72·1)	21·9 (16·8-27·9)	67·7 (63·5-70·4)	71·7 (67·2-74·5)	99·8 (99·6-99·9)	58·2 (55·7-60·4)	69·4 (66·9-71·6)	69·8 (67·3-72·1)	76·3 (73·6-78·7)	77·3 (72·5-81·4)	91·0 (88·9-92·9)	92·1 (90·2-93·8)	98·7 (98·4-99·0)
Cuba	86·4 (84·7-88·0)	95·0 (93·1-96·7)	95·4 (93·5-97·1)	99·9 (99·4-100·0)	100·0 (100·0-100·0)	100·0 (100·0-100·0)	100·0 (100·0-100·0)	100·0 (100·0-100·0)	69·9 (67·0-72·3)	89·5 (87·6-91·4)	89·8 (87·9-91·7)	94·4 (92·5-96·4)	73·3 (71·3-75·1)	73·2 (70·9-75·2)	73·2 (70·9-75·2)	73·2 (70·9-75·2)
Dominica	54·9 (53·2-56·5)	59·6 (58·0-61·1)	59·8 (58·2-61·3)	62·1 (60·5-63·6)	100·0 (100·0-100·0)	100·0 (100·0-100·0)	100·0 (100·0-100·0)	100·0 (100·0-100·0)	77·3 (75·2-79·3)	83·3 (81·3-85·2)	83·4 (81·4-85·2)	87·8 (85·7-89·5)	99·7 (99·7-99·8)	99·8 (99·6-99·9)	99·8 (99·6-99·9)	99·7 (99·5-99·8)
Dominican Republic	47·3 (45·9-48·8)	54·7 (53·2-56·2)	55·0 (53·5-56·6)	62·1 (60·1-63·8)	54·5 (51·2-56·9)	64·4 (60·5-67·0)	66·2 (62·0-68·9)	88·5 (83·5-91·3)	44·4 (41·6-47·1)	61·0 (58·8-63·2)	61·5 (59·2-63·7)	69·1 (66·5-71·9)	98·3 (98·0-98·5)	98·7 (98·5-98·9)	98·8 (98·5-98·9)	99·0 (98·8-99·2)
Grenada	81·7 (79·8-83·5)	85·2 (83·3-87·0)	85·5 (83·6-87·4)	89·9 (87·8-91·8)	100·0 (100·0-100·0)	100·0 (100·0-100·0)	100·0 (100·0-100·0)	100·0 (100·0-100·0)	67·4 (64·8-69·9)	78·2 (76·0-80·3)	79·0 (76·8-81·0)	90·3 (88·2-92·4)	74·9 (71·9-77·5)	72·2 (66·2-77·1)	72·2 (66·2-77·1)	72·4 (66·4-77·4)

Location	Indicator 3.4.1: Age-standardised death rate due to cardiovascular disease, cancer, diabetes, and chronic respiratory disease in populations aged 30-70 (per 100,000 population)				Indicator 3.4.2: Age-standardised death rate due to self-harm (per 100,000 population)				Indicator 3.5.2: Risk-weighted prevalence of alcohol consumption, as measured by the summary exposure value (SEV) for alcohol use (%)				Indicator 3.6.1: Age-standardised death rate due to road injuries (per 100,000 population)			
	**2000**	**2015**	**2016**	**2030**	**2000**	**2015**	**2016**	**2030**	**2000**	**2015**	**2016**	**2030**	**2000**	**2015**	**2016**	**2030**
Azerbaijan	23·1 (18·4-27·9)	37·7 (29·4-46·6)	38·6 (28·4-49·0)	52·8 (27·3-80·0)	95·3 (87·4-100·0)	81·9 (70·0-94·1)	82·2 (69·6-94·2)	86·2 (55·4-100·0)	58·8 (45·5-70·3)	48·8 (30·3-67·3)	48·9 (30·0-67·7)	49·6 (26·2-72·3)	63·3 (58·9-67·3)	68·6 (60·8-76·3)	68·8 (59·8-77·2)	73·0 (50·1-95·6)
Georgia	37·4 (32·7-42·6)	39·9 (31·5-48·1)	41·1 (30·4-51·5)	54·3 (27·7-79·6)	65·6 (59·3-73·7)	61·7 (53·5-70·4)	62·5 (52·5-73·2)	57·7 (32·9-80·1)	66·0 (57·7-73·9)	50·1 (35·4-64·6)	49·1 (34·1-64·0)	33·3 (10·5-57·4)	62·4 (58·5-66·9)	42·2 (35·4-49·6)	43·4 (35·2-52·1)	31·9 (13·4-49·3)
Kazakhstan	5·0 (0·0-10·5)	35·8 (28·7-41·9)	35·6 (26·1-44·8)	72·6 (46·9-97·6)	0·1 (0·0-0·3)	9·1 (2·0-16·5)	9·1 (0·0-18·0)	33·9 (6·0-58·9)	32·1 (16·3-47·4)	24·9 (0·0-47·4)	25·1 (0·0-48·0)	28·1 (0·0-55·4)	36·2 (31·3-41·2)	40·0 (33·9-45·4)	40·1 (32·7-47·6)	66·4 (41·8-89·0)
Kyrgyzstan	20·1 (16·8-23·3)	38·9 (35·4-42·0)	39·2 (34·1-44·3)	67·8 (52·8-81·9)	22·8 (18·2-28·4)	37·4 (24·0-43·1)	37·5 (24·5-44·4)	51·7 (27·3-68·4)	50·0 (39·9-60·4)	51·1 (37·2-65·5)	51·6 (37·6-66·1)	58·0 (42·0-74·0)	42·6 (39·5-45·6)	38·5 (35·1-42·2)	39·9 (35·3-45·0)	51·4 (36·2-64·8)
Mongolia	4·6 (0·0-8·8)	16·3 (9·8-23·2)	17·2 (9·6-24·9)	31·9 (9·7-54·8)	13·3 (7·0-21·3)	18·1 (9·5-27·1)	17·7 (8·1-27·4)	24·2 (4·1-43·8)	72·2 (63·7-79·9)	56·5 (40·4-71·5)	56·0 (39·4-71·3)	48·2 (26·6-68·1)	40·9 (35·9-45·6)	32·4 (26·6-38·7)	32·3 (25·5-39·4)	37·5 (19·0-55·8)
Tajikistan	25·8 (20·3-31·8)	44·6 (35·9-51·7)	45·3 (36·7-52·7)	60·4 (40·7-80·0)	66·0 (57·0-72·3)	71·3 (62·1-79·6)	71·5 (62·3-79·7)	75·3 (56·2-94·0)	82·9 (77·3-87·9)	87·5 (82·5-91·8)	87·6 (82·6-91·9)	89·3 (84·3-93·4)	59·2 (54·0-64·3)	68·4 (61·5-76·1)	68·7 (61·7-76·4)	76·3 (59·2-93·5)
Turkmenistan	13·4 (10·4-16·2)	30·1 (26·7-33·4)	31·5 (27·9-35·1)	49·9 (42·2-58·1)	37·9 (33·1-51·4)	53·3 (46·9-59·6)	53·2 (46·4-59·8)	65·3 (50·4-80·6)	62·2 (49·0-74·1)	62·4 (45·7-75·9)	62·4 (45·8-76·0)	63·7 (44·7-78·2)	47·7 (44·5-50·9)	67·6 (63·6-71·1)	67·6 (63·5-71·4)	93·5 (82·6-100·0)
Uzbekistan	15·9 (11·4-19·8)	28·3 (22·6-33·9)	29·1 (22·6-35·7)	41·5 (22·5-59·4)	36·7 (31·1-48·0)	42·7 (36·0-51·3)	43·1 (35·7-52·1)	47·8 (31·7-65·0)	69·8 (59·5-78·7)	57·9 (40·2-72·6)	57·1 (39·1-72·3)	45·3 (20·3-66·5)	42·5 (38·6-46·1)	42·8 (37·0-48·5)	43·5 (37·4-49·8)	48·0 (34·8-63·4)
**Central Latin America**																
Colombia	61·8 (59·1-64·6)	87·7 (83·1-92·1)	88·2 (82·7-93·7)	99·0 (92·6-100·0)	56·3 (46·3-62·4)	69·0 (60·2-77·3)	69·1 (60·1-78·4)	72·1 (53·3-95·3)	51·4 (40·2-62·8)	50·0 (33·5-65·0)	49·8 (33·0-65·0)	46·3 (24·2-65·2)	26·7 (19·6-31·3)	45·6 (39·7-50·3)	46·4 (39·9-51·8)	58·9 (45·7-71·9)
Costa Rica	80·1 (77·3-82·8)	93·4 (89·3-97·5)	94·0 (89·2-98·6)	98·0 (88·1-100·0)	60·4 (55·1-72·7)	61·9 (55·0-73·0)	62·0 (54·7-73·1)	63·4 (49·0-78·9)	53·1 (43·6-62·3)	56·1 (41·1-69·0)	55·9 (40·6-68·9)	53·2 (34·5-68·6)	33·6 (30·5-36·4)	45·9 (41·8-50·2)	46·4 (42·0-51·1)	54·6 (44·1-65·2)
El Salvador	74·4 (70·3-77·8)	78·1 (71·9-84·1)	79·1 (72·3-85·8)	84·7 (71·8-97·5)	43·6 (35·5-48·8)	48·3 (39·2-55·2)	48·4 (39·4-56·2)	51·0 (35·6-66·9)	70·2 (63·1-76·9)	62·9 (51·1-73·9)	62·7 (50·6-73·7)	58·8 (44·2-72·1)	12·2 (8·3-15·8)	28·2 (22·4-33·4)	28·5 (22·4-34·2)	43·5 (31·4-54·5)
Guatemala	68·5 (60·9-77·2)	70·3 (58·3-82·2)	70·7 (56·8-84·8)	76·3 (42·1-100·0)	34·8 (24·5-53·7)	61·2 (44·8-72·8)	60·3 (43·1-74·5)	80·9 (40·9-100·0)	73·3 (69·0-77·3)	73·5 (68·7-78·2)	73·5 (68·7-78·2)	73·8 (68·8-78·5)	42·9 (35·9-49·8)	39·2 (30·1-49·0)	39·6 (28·7-51·1)	44·6 (16·5-70·9)
Honduras	46·8 (34·7-60·5)	56·5 (41·7-70·6)	57·0 (42·2-71·0)	65·6 (41·0-90·1)	76·0 (62·2-91·1)	78·0 (61·0-93·2)	78·1 (61·5-93·1)	81·0 (53·0-100·0)	75·3 (71·8-78·7)	73·5 (68·8-78·2)	73·5 (68·8-78·2)	73·7 (68·7-78·8)	34·3 (24·7-44·9)	40·4 (28·9-51·5)	40·9 (29·5-51·8)	47·9 (27·4-66·4)
Mexico	66·8 (64·8-68·7)	73·7 (71·5-75·8)	74·1 (71·8-76·4)	80·7 (73·6-87·4)	74·8 (71·1-82·9)	67·9 (63·6-76·6)	68·0 (63·5-76·8)	67·4 (60·3-77·8)	48·1 (41·9-54·2)	53·7 (44·1-63·3)	53·8 (44·1-63·6)	56·4 (43·2-68·9)	32·8 (30·7-35·3)	41·3 (38·2-44·3)	41·7 (38·6-44·8)	54·9 (49·8-59·5)
Nicaragua	78·3 (75·1-81·3)	83·1 (76·2-90·3)	84·1 (75·1-93·1)	89·8 (71·1-100·0)	50·1 (44·1-59·1)	58·3 (50·4-66·8)	58·7 (48·8-67·9)	63·5 (42·3-83·6)	67·9 (62·9-72·7)	68·1 (60·7-74·6)	68·1 (60·6-74·7)	68·5 (59·6-75·8)	41·3 (37·6-44·6)	49·5 (43·0-56·1)	50·2 (42·7-57·6)	57·5 (39·2-74·0)
Panama	77·8 (73·3-81·9)	84·2 (78·0-90·7)	85·4 (77·9-92·9)	92·6 (74·7-100·0)	62·8 (56·7-75·1)	67·5 (57·1-76·5)	67·7 (56·8-77·9)	71·4 (47·7-96·2)	59·8 (55·3-63·9)	55·6 (47·6-63·1)	55·2 (47·2-62·8)	49·9 (40·4-58·6)	32·9 (28·6-37·1)	43·8 (36·8-50·3)	44·7 (37·0-52·0)	55·2 (37·0-75·6)
Venezuela	59·1 (55·4-63·1)	65·8 (57·5-73·4)	66·1 (56·3-75·0)	69·7 (45·8-93·2)	47·8 (40·3-55·4)	49·3 (33·2-61·4)	49·2 (32·2-62·6)	47·1 (13·1-76·2)	49·2 (41·5-56·1)	51·5 (41·0-61·0)	51·6 (41·1-61·1)	52·8 (41·6-62·6)	24·7 (18·7-29·4)	27·4 (16·8-36·3)	27·6 (16·0-37·8)	34·2 (9·0-57·6)
**Andean Latin America**																
Bolivia	54·5 (50·2-59·0)	64·4 (52·8-75·9)	64·7 (52·6-76·5)	70·7 (45·9-94·2)	53·3 (46·4-62·7)	59·2 (49·6-70·5)	59·4 (49·4-70·8)	63·7 (44·0-84·3)	76·6 (69·3-83·4)	72·3 (60·7-81·7)	71·8 (59·8-81·6)	64·2 (44·2-79·0)	18·0 (13·6-22·9)	30·0 (22·0-38·7)	30·5 (22·0-39·6)	38·0 (21·5-54·6)
Ecuador	72·2 (69·2-75·2)	86·6 (82·4-90·7)	87·1 (82·8-91·5)	97·7 (89·4-100·0)	55·3 (50·5-62·0)	55·4 (49·2-66·5)	55·8 (49·3-66·9)	61·4 (48·1-77·2)	60·8 (53·5-67·2)	62·1 (54·1-68·7)	62·3 (54·3-68·8)	64·4 (56·8-70·6)	20·9 (17·3-24·4)	24·9 (21·1-29·0)	25·9 (21·9-30·0)	35·9 (25·4-46·9)
Peru	81·0 (76·5-86·0)	95·2 (86·3-100·0)	95·7 (85·3-100·0)	98·4 (83·7-100·0)	78·0 (71·7-86·0)	93·6 (85·2-100·0)	94·2 (84·7-100·0)	98·2 (84·4-100·0)	40·0 (26·2-53·4)	46·9 (27·1-62·8)	47·3 (27·2-63·2)	52·0 (29·8-69·2)	38·1 (34·3-42·2)	46·1 (39·5-52·9)	47·4 (39·8-55·8)	60·4 (40·4-82·1)
**Caribbean**																
Antigua and Barbuda	53·3 (49·0-57·6)	64·5 (58·4-70·7)	65·0 (58·4-71·6)	71·3 (55·8-85·9)	99·9 (100·0-100·0)	99·9 (98·1-100·0)	99·8 (97·9-100·0)	99·2 (90·4-100·0)	85·7 (80·1-90·8)	78·8 (64·6-89·9)	78·6 (63·9-90·0)	74·6 (51·0-90·9)	65·1 (60·8-69·4)	66·4 (59·3-73·5)	66·8 (59·1-74·5)	70·3 (54·1-87·7)
The Bahamas	45·0 (41·9-48·1)	51·9 (46·3-57·2)	51·3 (45·0-57·2)	51·3 (35·5-65·5)	98·4 (92·9-100·0)	93·9 (84·9-100·0)	93·6 (84·4-100·0)	92·2 (75·5-100·0)	48·1 (39·1-57·7)	59·6 (41·1-77·4)	60·4 (41·6-78·3)	70·6 (50·3-88·6)	35·5 (30·9-40·7)	43·6 (37·8-49·5)	43·9 (37·8-50·2)	48·1 (32·8-62·9)
Barbados	57·9 (53·6-62·3)	63·7 (58·7-68·6)	63·5 (58·1-68·7)	68·6 (55·8-79·9)	78·1 (72·1-84·7)	75·2 (68·5-82·8)	75·2 (68·0-82·9)	74·2 (59·4-88·4)	60·1 (54·4-66·1)	53·2 (40·6-66·4)	52·9 (40·2-66·3)	48·9 (33·1-64·7)	56·7 (52·4-61·2)	59·9 (54·3-65·2)	60·0 (54·1-65·5)	61·3 (48·6-73·2)
Belize	35·2 (29·8-39·8)	47·3 (40·4-54·6)	47·3 (39·7-55·5)	58·3 (40·0-78·4)	55·4 (49·5-66·0)	55·9 (47·2-65·6)	56·0 (46·2-66·3)	57·7 (36·3-78·3)	54·5 (45·3-64·3)	44·0 (30·6-58·3)	43·8 (30·2-58·3)	41·4 (23·2-60·6)	19·5 (15·6-24·4)	28·7 (22·0-35·2)	29·1 (21·6-36·3)	37·9 (19·5-57·0)
Cuba	54·8 (52·1-57·6)	64·7 (60·5-69·2)	65·1 (60·5-70·0)	73·6 (62·6-84·7)	25·1 (21·1-28·6)	38·7 (29·9-44·2)	38·7 (29·8-44·7)	43·6 (29·4-57·0)	68·9 (62·7-75·2)	58·9 (45·4-70·5)	58·2 (44·4-70·1)	48·2 (28·9-65·3)	45·0 (42·4-48·1)	69·2 (64·9-73·6)	70·7 (64·8-76·6)	86·7 (71·9-100·0)
Dominica	52·0 (47·9-56·2)	50·9 (44·7-57·0)	51·2 (44·2-58·2)	52·6 (34·5-68·6)	86·0 (79·1-94·2)	77·6 (68·7-89·2)	77·6 (67·8-89·9)	72·2 (50·7-94·3)	46·2 (38·6-53·6)	48·2 (32·5-62·3)	48·1 (32·2-62·3)	46·5 (28·0-63·5)	43·8 (39·9-47·7)	39·8 (33·9-46·1)	40·0 (33·4-47·0)	38·9 (24·0-54·4)
Dominican Republic	67·1 (61·1-75·3)	74·8 (65·5-86·2)	74·9 (65·1-86·0)	79·8 (58·8-100·0)	60·3 (52·2-70·8)	66·9 (57·0-77·0)	66·7 (56·7-77·2)	66·4 (43·7-87·6)	63·1 (55·9-70·6)	59·7 (46·6-72·0)	59·3 (45·6-72·0)	54·6 (35·0-71·7)	27·2 (22·9-32·1)	34·1 (27·8-41·7)	34·4 (27·7-42·1)	38·3 (23·7-53·2)
Grenada	35·5 (30·1-40·5)	36·7 (29·8-43·8)	37·3 (29·9-45·0)	41·6 (23·3-61·1)	62·1 (54·8-71·2)	60·2 (51·3-69·3)	60·0 (50·5-69·8)	57·9 (36·6-77·8)	43·7 (36·1-50·9)	51·0 (38·8-63·8)	51·2 (38·8-64·1)	54·8 (41·0-68·5)	48·4 (43·0-54·2)	42·1 (34·8-49·6)	44·0 (36·3-51·8)	39·6 (21·4-58·4)

Location	Indicator 3.7.1: Proportion of women of reproductive age (15-49 years) who have their need for family planning satisfied with modern contraception methods (%)				Indicator 3.7.2: Number of livebirths per 1,000 women aged 10-14 years and women aged 15-19 years				Indicator 3.8.1: Coverage of essential health services, as defined by the UHC index comprised of the coverage of 9 tracer interventions and risk-standardised death rates from 32 causes amenable to personal healthcare (scale of 0 to 100)				Indicator 3.9.1: Age-standardised death rate attributable to household air pollution and ambient air pollution (per 100,000 population)			
	2000	2015	2016	2030	2000	2015	2016	2030	2000	2015	2016	2030	2000	2015	2016	2030
Azerbaijan	24·5 (17·5-31·8)	34·0 (24·9-43·5)	35·5 (26·3-45·2)	56·7 (31·8-82·2)	40·1 (38·3-42·1)	20·7 (19·3-22·3)	20·1 (18·5-21·9)	12·0 (6·2-18·2)	23·5 (20·2-26·7)	51·0 (45·3-56·6)	52·4 (46·3-58·2)	78·9 (69·0-87·5)	18·2 (11·9-23·3)	31·6 (21·9-39·0)	32·6 (22·9-40·1)	48·0 (36·0-56·8)
Georgia	43·6 (38·5-48·9)	56·2 (46·2-66·2)	56·7 (46·9-66·7)	66·1 (45·2-84·7)	29·5 (27·2-31·9)	25·8 (22·9-28·5)	28·5 (25·3-31·6)	28·6 (18·6-38·3)	45·8 (42·6-48·9)	54·5 (49·4-59·3)	55·1 (49·8-60·2)	64·4 (55·5-73·3)	24·1 (19·5-29·0)	32·3 (23·6-42·2)	33·5 (24·3-43·7)	43·7 (28·2-61·1)
Kazakhstan	70·3 (67·0-73·7)	85·1 (82·6-87·8)	85·4 (82·2-88·5)	92·5 (88·1-96·8)	39·5 (37·4-41·8)	41·8 (39·6-44·0)	44·2 (41·9-46·7)	46·4 (40·4-52·5)	37·5 (33·9-40·9)	60·0 (55·4-64·7)	61·6 (56·6-66·6)	85·4 (79·2-91·2)	24·0 (17·8-29·5)	37·9 (28·7-45·9)	39·0 (29·6-47·2)	55·0 (42·0-66·1)
Kyrgyzstan	69·6 (63·5-75·4)	65·8 (61·4-69·8)	66·2 (60·9-71·1)	60·7 (49·8-70·8)	34·5 (32·6-36·8)	32·4 (30·0-35·1)	33·5 (30·9-36·4)	37·4 (29·9-44·8)	40·4 (38·1-42·8)	54·6 (51·2-57·8)	55·9 (52·4-59·4)	72·0 (67·1-76·8)	15·3 (9·6-21·7)	28·1 (22·4-34·1)	29·5 (23·6-35·6)	47·2 (39·0-55·4)
Mongolia	69·8 (61·3-77·4)	70·5 (66·0-74·5)	71·0 (65·7-76·1)	76·8 (62·6-88·3)	46·8 (43·9-49·6)	54·5 (51·1-58·3)	57·3 (53·2-61·5)	62·0 (54·4-71·1)	31·4 (28·4-34·6)	54·0 (49·0-59·0)	54·8 (49·7-59·8)	69·5 (62·7-76·2)	7·0 (3·7-10·2)	19·3 (14·0-25·1)	20·1 (14·8-26·0)	31·6 (24·8-39·7)
Tajikistan	47·6 (39·2-56·0)	53·1 (46·1-60·3)	53·5 (46·0-61·8)	58·6 (41·0-75·4)	37·7 (35·4-39·8)	34·9 (32·0-37·9)	35·1 (32·0-38·3)	35·3 (27·3-44·6)	28·1 (24·4-32·0)	47·8 (42·9-52·8)	49·0 (43·9-53·9)	67·3 (60·5-74·1)	2·6 (0·0-6·2)	19·7 (13·8-25·5)	20·5 (14·4-26·4)	31·1 (22·9-39·2)
Turkmenistan	60·4 (50·7-69·5)	76·9 (73·7-80·2)	77·6 (74·2-81·1)	90·0 (82·3-97·1)	48·8 (46·1-51·9)	55·1 (51·6-58·6)	56·2 (52·7-59·7)	63·2 (56·1-70·5)	35·8 (32·9-38·7)	47·4 (43·4-51·1)	49·0 (44·9-52·7)	62·9 (57·4-67·5)	23·2 (19·6-26·7)	34·8 (31·0-38·4)	35·0 (31·2-38·6)	38·2 (34·4-41·9)
Uzbekistan	82·8 (78·2-87·3)	86·8 (79·3-92·6)	87·1 (79·6-93·0)	91·9 (78·3-100·0)	44·9 (42·1-47·9)	52·7 (46·7-58·8)	53·3 (47·4-59·5)	57·1 (42·7-72·9)	41·9 (38·9-44·9)	56·1 (51·4-60·2)	57·0 (52·2-61·3)	72·3 (66·3-77·5)	16·2 (10·8-20·8)	27·4 (19·1-33·0)	27·9 (19·4-33·5)	34·6 (23·9-41·3)
**Central Latin America**																
Colombia	79·0 (77·4-80·7)	96·4 (95·1-97·6)	96·5 (95·0-98·0)	100·0 (100·0-100·0)	14·2 (12·8-15·7)	30·3 (28·4-32·4)	32·1 (29·9-34·4)	51·0 (46·5-55·9)	44·7 (42·6-46·9)	65·6 (62·2-68·7)	66·4 (62·9-69·7)	82·9 (77·7-87·3)	49·2 (46·4-52·3)	63·6 (58·3-68·6)	65·1 (59·6-70·2)	80·3 (70·8-88·4)
Costa Rica	91·1 (85·2-96·3)	96·1 (91·0-100·0)	96·4 (91·5-100·0)	98·1 (90·3-100·0)	18·1 (16·0-20·3)	27·9 (24·3-31·2)	28·7 (25·0-32·0)	38·4 (29·4-47·0)	59·0 (56·6-61·2)	72·4 (69·0-75·5)	73·0 (69·6-76·2)	81·7 (76·7-86·0)	61·9 (59·2-64·6)	81·8 (77·9-85·4)	81·8 (77·9-85·4)	97·0 (85·7-100·0)
El Salvador	81·9 (79·7-84·2)	88·9 (86·5-91·3)	89·3 (86·4-92·2)	96·9 (91·5-100·0)	13·0 (11·1-15·2)	20·1 (16·7-23·3)	20·4 (17·0-23·7)	27·9 (20·4-35·1)	46·6 (43·5-49·5)	61·0 (56·5-65·4)	61·7 (57·1-66·1)	71·9 (65·4-78·5)	38·6 (35·2-42·7)	56·3 (50·8-61·5)	57·0 (51·4-62·3)	70·5 (61·7-80·2)
Guatemala	59·9 (56·3-63·2)	70·9 (67·7-74·0)	71·4 (67·1-75·4)	73·4 (62·0-85·1)	8·4 (6·7-10·4)	18·0 (15·8-20·4)	18·0 (15·7-20·7)	26·4 (20·7-32·6)	32·6 (28·1-37·0)	45·6 (38·9-52·7)	46·3 (39·3-53·8)	57·6 (46·5-69·3)	22·8 (19·1-26·8)	41·1 (33·5-49·1)	42·1 (34·1-50·6)	57·2 (43·4-70·7)
Honduras	75·7 (73·1-78·5)	84·2 (79·3-88·7)	84·6 (78·7-89·5)	95·8 (87·1-100·0)	12·2 (8·9-15·8)	17·5 (14·3-21·3)	17·7 (14·3-21·9)	22·6 (13·0-32·1)	31·3 (25·2-37·8)	46·3 (39·1-53·5)	46·9 (39·7-54·1)	57·0 (48·6-64·8)	24·1 (17·7-30·4)	34·9 (27·8-42·3)	35·7 (28·6-43·1)	47·2 (39·4-54·9)
Mexico	83·5 (76·4-90·0)	86·1 (83·4-88·4)	86·6 (83·8-89·4)	89·9 (80·7-96·8)	13·9 (12·4-15·5)	21·9 (20·2-23·8)	22·4 (20·6-24·5)	29·6 (25·4-34·0)	46·7 (45·0-48·3)	56·3 (53·9-58·5)	57·3 (54·9-59·4)	66·1 (62·8-69·2)	56·9 (54·5-59·4)	68·0 (65·4-70·9)	68·8 (66·1-71·7)	79·7 (76·3-83·0)
Nicaragua	82·8 (80·5-85·0)	96·9 (92·3-100·0)	97·0 (92·2-100·0)	97·4 (88·5-100·0)	4·6 (3·8-5·6)	13·5 (12·6-14·4)	14·0 (13·2-14·9)	24·1 (22·6-25·6)	48·4 (46·0-50·9)	64·7 (60·1-69·4)	65·5 (60·8-70·4)	77·8 (69·7-85·3)	43·6 (40·5-46·7)	55·4 (48·7-62·5)	56·3 (49·4-63·8)	70·3 (59·5-82·7)
Panama	86·3 (78·8-92·8)	93·3 (87·2-98·0)	93·9 (88·0-98·5)	97·8 (89·0-100·0)	14·4 (12·3-16·7)	19·8 (16·5-22·7)	20·8 (17·3-24·2)	24·4 (15·6-32·6)	53·8 (51·3-56·7)	60·8 (56·2-65·2)	61·8 (57·0-66·4)	71·7 (64·1-79·3)	61·1 (57·4-64·8)	74·8 (68·3-80·4)	75·4 (68·8-81·1)	84·9 (76·6-92·2)
Venezuela	82·2 (74·4-88·9)	90·3 (84·0-95·6)	90·7 (84·3-96·0)	94·6 (84·0-100·0)	14·2 (11·5-16·9)	17·3 (14·7-19·9)	17·7 (14·8-20·5)	21·7 (15·8-26·8)	45·6 (42·8-48·5)	55·7 (50·5-60·4)	56·1 (50·7-61·1)	62·4 (53·3-70·6)	54·3 (51·4-57·4)	62·9 (57·5-68·1)	62·8 (57·3-68·0)	73·2 (63·4-86·8)
**Andean Latin America**																
Bolivia	36·0 (33·2-38·8)	46·9 (37·8-55·9)	47·7 (37·8-57·6)	62·3 (42·5-81·8)	12·4 (11·0-14·1)	16·0 (14·5-18·0)	16·0 (14·3-17·9)	19·1 (14·8-23·3)	20·5 (17·5-23·6)	41·6 (35·3-47·8)	42·7 (36·4-49·2)	60·2 (50·4-70·1)	27·3 (23·9-30·2)	44·6 (38·2-51·7)	46·1 (39·4-53·4)	65·7 (55·2-76·7)
Ecuador	76·9 (73·9-79·8)	86·4 (79·3-92·4)	86·6 (79·2-92·8)	89·9 (76·3-99·7)	13·9 (11·3-16·8)	14·3 (11·3-17·1)	14·4 (11·4-17·2)	15·9 (9·9-22·3)	42·7 (40·2-44·9)	57·7 (54·8-60·7)	58·7 (55·7-61·7)	71·8 (67·9-75·7)	58·5 (55·4-61·5)	80·1 (75·3-84·6)	81·3 (76·4-85·8)	97·5 (91·9-100·0)
Peru	60·8 (58·7-62·9)	65·8 (60·7-70·5)	66·2 (59·8-71·8)	73·3 (61·8-85·1)	23·1 (21·5-24·7)	24·0 (22·6-25·6)	24·4 (22·9-26·1)	25·6 (21·9-29·6)	43·6 (40·6-47·0)	66·4 (61·0-71·7)	67·3 (61·7-73·0)	83·0 (74·1-91·0)	39·1 (35·3-43·0)	54·6 (47·6-61·9)	56·0 (48·7-63·5)	75·3 (63·8-88·0)
**Caribbean**																
Antigua and Barbuda	86·2 (78·4-92·6)	91·1 (84·7-96·2)	91·3 (85·0-96·5)	94·6 (82·7-100·0)	28·4 (24·0-33·4)	35·6 (30·7-40·7)	36·8 (31·6-42·1)	46·2 (33·4-59·1)	53·4 (50·1-56·6)	60·6 (56·7-64·8)	61·4 (57·2-65·6)	67·4 (62·2-72·7)	66·8 (58·1-76·4)	77·2 (67·8-88·0)	77·1 (67·7-87·8)	89·2 (76·5-100·0)
The Bahamas	88·4 (81·7-94·2)	91·6 (85·5-96·5)	91·8 (85·9-96·7)	93·5 (81·6-100·0)	25·7 (23·8-27·8)	35·6 (31·9-39·4)	37·6 (33·5-41·4)	46·1 (38·3-54·4)	50·0 (46·8-53·3)	57·6 (52·9-62·0)	57·5 (52·6-62·0)	61·3 (52·2-69·0)	60·1 (53·2-68·3)	72·2 (63·7-81·1)	72·0 (63·4-81·0)	80·8 (66·4-97·0)
Barbados	85·7 (78·7-92·3)	89·4 (82·3-94·9)	89·5 (82·5-94·9)	91·5 (78·2-100·0)	31·4 (28·2-34·7)	37·9 (34·6-40·8)	39·9 (36·5-42·9)	42·6 (33·7-51·7)	53·7 (50·1-57·1)	61·3 (56·6-65·5)	61·3 (56·5-65·7)	63·8 (55·7-70·3)	69·9 (59·3-81·8)	78·0 (66·9-90·9)	77·2 (66·2-90·0)	83·1 (62·7-100·0)
Belize	75·9 (67·5-83·0)	78·0 (71·2-84·5)	78·8 (71·8-85·4)	91·8 (76·2-100·0)	10·3 (7·2-13·9)	16·3 (11·7-21·0)	16·5 (11·8-21·2)	21·3 (11·1-31·4)	34·5 (31·6-37·5)	48·0 (42·8-52·7)	48·6 (43·2-53·4)	57·5 (48·2-65·4)	34·0 (30·8-37·7)	48·3 (43·4-53·5)	48·7 (43·7-54·1)	54·5 (47·7-62·0)
Cuba	94·2 (89·2-98·5)	97·8 (95·5-99·9)	98·4 (95·9-100·0)	99·2 (94·2-100·0)	25·6 (24·3-27·1)	29·6 (25·0-34·4)	31·1 (25·0-37·2)	41·3 (27·6-53·0)	57·2 (55·1-59·6)	69·5 (66·6-72·4)	70·2 (67·2-73·2)	79·3 (75·0-83·5)	53·5 (50·7-56·2)	67·3 (64·0-70·6)	67·6 (64·3-70·9)	71·3 (67·1-78·8)
Dominica	80·1 (72·5-87·4)	87·3 (80·2-93·1)	87·7 (80·5-93·5)	91·7 (78·6-100·0)	42·5 (40·0-45·3)	50·4 (47·6-53·1)	51·3 (48·1-54·4)	59·4 (51·6-68·3)	50·5 (47·5-53·4)	50·1 (45·5-54·0)	50·4 (45·7-54·5)	49·8 (42·5-56·4)	54·0 (48·0-60·6)	66·0 (58·1-74·2)	66·1 (58·2-74·3)	81·4 (69·5-98·0)
Dominican Republic	85·9 (83·7-87·9)	91·1 (88·9-93·2)	91·7 (89·1-94·4)	99·5 (95·5-100·0)	8·5 (5·6-11·7)	14·7 (8·8-19·9)	15·2 (8·7-21·0)	22·5 (8·9-35·5)	47·1 (43·4-51·0)	59·3 (54·7-64·8)	60·1 (55·4-65·7)	70·2 (64·3-77·3)	50·1 (46·2-54·4)	58·5 (53·2-63·9)	58·8 (53·5-64·2)	63·7 (57·8-70·0)
Grenada	75·1 (66·4-83·2)	86·8 (79·9-93·0)	87·4 (80·3-93·3)	93·8 (82·4-100·0)	25·0 (22·2-28·0)	44·3 (41·1-47·0)	46·3 (43·1-49·1)	67·2 (58·9-75·1)	43·8 (40·2-47·2)	46·7 (41·8-51·2)	47·3 (42·3-52·0)	50·7 (44·1-57·4)	47·3 (39·7-57·0)	57·6 (48·1-69·3)	57·2 (47·7-68·9)	74·9 (57·8-99·1)

Location	Indicator 3.9.2: Age-standardised death rate attributable to unsafe water, sanitation, and hygiene (WaSH) (per 100,000 population)				Indicator 3.9.3: Age-standardised death rate due to unintentional poisonings (per 100,000 population)				Indicator 3.a.1: Age-standardised prevalence of daily smoking in populations aged 10 and older (%)				Indicator 3.b.1: Geometric mean of the coverage of eight vaccines, conditional on inclusion in national vaccine schedules, in target populations (%)			
	**2000**	**2015**	**2016**	**2030**	**2000**	**2015**	**2016**	**2030**	**2000**	**2015**	**2016**	**2030**	**2000**	**2015**	**2016**	**2030**
Azerbaijan	40·1<br>(36·4-44·2)	61·4<br>(54·9-67·7)	62·4<br>(55·7-68·9)	77·1<br>(67·3-85·6)	34·0<br>(25·5-38·5)	45·5<br>(29·1-54·2)	46·6<br>(30·1-55·7)	63·9<br>(33·5-85·7)	46·1<br>(38·3-53·2)	46·3<br>(39·0-53·1)	46·7<br>(38·9-54·1)	47·7<br>(32·5-60·5)	22·4<br>(15·5-28·8)	73·3<br>(60·6-82·8)	69·0<br>(55·2-78·5)	93·7<br>(81·4-98·9)
Georgia	57·6<br>(53·6-61·3)	79·5<br>(74·8-84·0)	80·0<br>(75·3-84·7)	88·7<br>(83·0-95·5)	43·5<br>(31·3-47·9)	44·8<br>(36·6-50·5)	45·6<br>(36·7-52·0)	51·2<br>(32·1-70·0)	37·3<br>(30·7-43·7)	31·3<br>(23·8-39·2)	31·4<br>(22·9-39·7)	28·7<br>(11·1-45·4)	68·2<br>(62·7-73·2)	79·8<br>(73·7-85·3)	79·6<br>(72·1-85·7)	91·6<br>(78·3-98·2)
Kazakhstan	50·6<br>(46·1-54·9)	75·6<br>(70·5-80·5)	76·6<br>(71·3-81·5)	91·9<br>(84·5-98·9)	2·9<br>(0·0-9·1)	15·3<br>(8·7-23·6)	15·8<br>(8·7-24·5)	30·1<br>(13·0-47·2)	43·3<br>(37·5-49·3)	43·4<br>(37·4-49·3)	44·2<br>(37·5-50·8)	55·2<br>(41·4-67·5)	91·6<br>(88·3-94·1)	88·6<br>(83·3-92·1)	90·3<br>(84·1-94·3)	98·3<br>(92·0-100·0)
Kyrgyzstan	42·8<br>(39·0-46·6)	58·3<br>(54·4-61·8)	59·2<br>(55·2-62·7)	71·9<br>(66·4-76·4)	25·9<br>(19·7-31·4)	44·5<br>(31·7-49·0)	45·2<br>(32·1-50·4)	59·6<br>(40·6-70·3)	54·7<br>(50·7-58·9)	44·7<br>(38·9-51·2)	45·2<br>(38·5-52·1)	39·2<br>(23·1-53·7)	76·7<br>(72·1-80·6)	81·6<br>(75·0-87·1)	81·9<br>(74·4-87·8)	86·5<br>(63·4-97·4)
Mongolia	49·8<br>(44·6-55·1)	68·9<br>(63·0-74·8)	69·8<br>(63·8-75·8)	84·5<br>(76·7-92·0)	0·0<br>(0·0-0·0)	16·1<br>(0·0-23·3)	16·7<br>(0·0-24·6)	32·3<br>(3·0-51·5)	29·5<br>(24·5-34·5)	31·2<br>(22·5-39·5)	30·7<br>(21·2-39·7)	30·8<br>(14·2-47·3)	84·2<br>(80·6-87·4)	94·0<br>(90·4-96·4)	94·4<br>(90·7-96·9)	96·8<br>(87·1-100·0)
Tajikistan	23·5<br>(19·7-27·1)	39·4<br>(34·1-44·4)	40·1<br>(34·6-45·3)	51·0<br>(42·1-58·5)	16·8<br>(11·1-24·8)	30·6<br>(17·7-41·8)	31·1<br>(17·6-42·5)	41·4<br>(15·8-61·5)	97·3<br>(93·8-100·0)	99·8<br>(98·3-100·0)	99·8<br>(98·1-100·0)	99·9<br>(99·1-100·0)	78·4<br>(74·0-82·0)	87·9<br>(82·4-91·8)	88·4<br>(82·6-92·7)	94·5<br>(85·0-99·0)
Turkmenistan	31·6<br>(26·9-36·5)	59·2<br>(53·4-65·1)	60·9<br>(54·9-66·7)	84·3<br>(76·8-91·3)	30·5<br>(22·1-34·8)	45·2<br>(37·7-50·2)	46·0<br>(38·4-51·2)	64·5<br>(52·3-74·7)	81·5<br>(74·9-87·4)	85·1<br>(79·3-90·5)	85·3<br>(79·4-90·7)	88·1<br>(77·8-95·9)	88·9<br>(86·2-91·2)	86·9<br>(80·7-91·4)	88·0<br>(81·4-92·6)	90·1<br>(75·9-97·0)
Uzbekistan	50·1<br>(45·3-54·8)	67·7<br>(60·9-74·2)	68·8<br>(61·9-75·3)	84·1<br>(75·1-91·9)	28·8<br>(22·0-33·2)	33·4<br>(27·1-42·1)	34·3<br>(27·2-43·2)	39·1<br>(26·8-56·2)	71·2<br>(66·5-76·1)	83·4<br>(80·3-86·4)	83·2<br>(79·5-86·7)	90·0<br>(83·8-95·3)	66·8<br>(50·0-80·6)	99·4<br>(98·7-99·9)	99·5<br>(98·8-99·9)	100·0<br>(100·0-100·0)
**Central Latin America**																
Colombia	47·3<br>(44·3-50·4)	63·3<br>(60·5-66·2)	64·1<br>(61·3-67·1)	76·2<br>(72·9-80·0)	61·5<br>(48·8-66·2)	79·1<br>(64·8-84·2)	79·8<br>(65·3-85·3)	91·2<br>(76·1-100·0)	64·9<br>(58·3-70·8)	77·1<br>(72·5-81·7)	77·3<br>(72·3-82·0)	83·7<br>(75·5-91·1)	74·3<br>(70·5-77·8)	93·6<br>(90·9-95·6)	94·5<br>(92·0-96·3)	98·9<br>(97·6-99·6)
Costa Rica	56·1<br>(52·8-59·7)	67·1<br>(63·4-71·0)	67·3<br>(63·6-71·3)	76·4<br>(69·2-84·8)	65·9<br>(61·3-75·0)	76·8<br>(72·2-84·4)	77·4<br>(72·3-85·0)	85·7<br>(73·4-98·6)	71·0<br>(67·2-74·3)	86·5<br>(82·3-90·3)	86·7<br>(82·5-90·6)	91·0<br>(81·7-98·5)	92·7<br>(90·4-94·5)	95·6<br>(93·6-97·1)	96·1<br>(94·0-97·5)	98·5<br>(96·5-99·5)
El Salvador	34·3<br>(32·2-37·0)	47·8<br>(43·2-52·1)	48·3<br>(43·7-52·6)	61·7<br>(54·5-71·9)	63·2<br>(58·6-74·4)	82·1<br>(76·1-89·0)	82·7<br>(76·1-90·0)	91·5<br>(77·3-100·0)	84·0<br>(79·3-88·1)	87·6<br>(83·6-91·3)	87·7<br>(83·6-91·5)	90·4<br>(83·2-96·0)	91·2<br>(88·7-93·2)	89·1<br>(84·7-92·8)	89·8<br>(85·2-93·6)	92·2<br>(83·0-97·7)
Guatemala	18·3<br>(14·9-22·0)	30·9<br>(27·0-34·9)	31·6<br>(27·8-35·7)	43·4<br>(38·7-48·4)	29·2<br>(20·1-34·9)	30·0<br>(22·1-42·0)	30·7<br>(21·6-42·8)	40·8<br>(19·8-64·0)	93·5<br>(90·8-95·9)	93·2<br>(89·9-96·2)	93·3<br>(90·0-96·3)	94·6<br>(88·0-100·0)	92·6<br>(90·3-94·4)	90·7<br>(86·9-93·4)	92·5<br>(89·0-95·0)	89·3<br>(77·3-95·8)
Honduras	24·9<br>(20·4-28·8)	36·4<br>(29·5-42·6)	37·0<br>(30·0-43·3)	47·0<br>(37·7-56·0)	35·9<br>(23·9-49·8)	46·4<br>(28·9-63·3)	47·0<br>(29·3-63·9)	57·8<br>(32·5-80·6)	71·5<br>(67·3-75·2)	79·0<br>(74·6-82·9)	79·2<br>(74·4-83·1)	84·2<br>(75·9-90·6)	91·2<br>(89·4-92·8)	92·8<br>(90·0-94·9)	93·1<br>(90·1-95·2)	95·2<br>(89·4-98·6)
Mexico	45·2<br>(42·5-48·1)	57·6<br>(54·8-60·6)	58·0<br>(55·2-61·1)	69·5<br>(64·1-73·8)	46·1<br>(37·7-49·0)	59·4<br>(48·9-62·8)	60·0<br>(49·5-63·8)	71·0<br>(58·5-78·3)	55·1<br>(53·3-56·9)	71·7<br>(70·1-73·1)	72·8<br>(71·3-74·2)	83·5<br>(81·0-85·7)	81·4<br>(77·1-85·0)	74·7<br>(66·9-81·6)	76·1<br>(67·7-83·4)	82·2<br>(62·2-95·0)
Nicaragua	35·0<br>(32·5-37·6)	51·0<br>(46·3-55·3)	51·7<br>(47·0-56·1)	63·6<br>(56·8-73·3)	46·9<br>(42·6-53·1)	61·6<br>(54·2-68·7)	62·3<br>(54·2-69·8)	72·3<br>(52·1-88·3)	75·3<br>(69·9-80·5)	77·2<br>(71·5-82·3)	77·3<br>(71·7-82·3)	79·1<br>(68·4-87·4)	90·1<br>(86·6-92·7)	98·0<br>(96·3-99·1)	98·2<br>(96·4-99·2)	99·8<br>(98·7-100·0)
Panama	49·8<br>(46·8-52·6)	52·8<br>(49·0-56·7)	53·5<br>(49·7-57·4)	63·2<br>(57·6-68·7)	49·6<br>(42·7-53·3)	61·5<br>(50·8-67·0)	62·0<br>(51·7-67·9)	84·4<br>(62·0-100·0)	87·7<br>(84·5-90·5)	100·0<br>(99·6-100·0)	99·9<br>(99·2-100·0)	100·0<br>(100·0-100·0)	82·3<br>(79·2-85·1)	71·2<br>(62·5-78·8)	74·0<br>(64·9-81·3)	72·7<br>(52·0-87·1)
Venezuela	45·2<br>(41·5-49·3)	55·8<br>(51·9-60·4)	56·2<br>(52·2-60·8)	61·6<br>(55·7-68·2)	58·6<br>(51·6-63·4)	66·9<br>(60·3-73·3)	67·3<br>(60·2-74·1)	76·0<br>(60·6-91·7)	58·7<br>(52·2-65·0)	74·4<br>(68·0-79·6)	75·0<br>(68·8-80·3)	83·5<br>(73·9-91·2)	60·0<br>(55·6-64·0)	63·4<br>(55·6-70·0)	64·3<br>(56·0-71·8)	76·7<br>(60·6-88·7)
**Andean Latin America**																
Bolivia	28·0<br>(24·7-30·9)	43·2<br>(39·4-46·6)	44·1<br>(40·3-47·6)	58·0<br>(53·7-62·2)	20·8<br>(11·0-30·2)	38·6<br>(29·8-46·0)	39·4<br>(30·6-47·1)	55·1<br>(36·0-71·1)	79·2<br>(75·8-82·7)	81·8<br>(78·4-84·7)	81·9<br>(78·4-84·9)	83·6<br>(77·3-88·7)	86·1<br>(83·4-88·4)	93·5<br>(90·3-95·9)	94·2<br>(91·1-96·5)	99·0<br>(96·6-100·0)
Ecuador	41·6<br>(38·8-44·5)	56·4<br>(53·5-59·5)	57·1<br>(54·1-60·2)	70·9<br>(66·2-76·4)	40·7<br>(34·2-45·4)	54·6<br>(49·6-58·6)	55·4<br>(49·9-59·7)	66·6<br>(55·9-75·6)	86·1<br>(84·5-87·7)	95·8<br>(94·1-97·3)	95·8<br>(94·0-97·3)	99·3<br>(96·5-100·0)	84·9<br>(79·4-88·9)	91·6<br>(87·2-94·9)	92·3<br>(87·9-95·6)	96·4<br>(88·9-99·9)
Peru	38·1<br>(35·6-40·4)	48·5<br>(44·8-52·0)	49·2<br>(45·3-52·7)	59·2<br>(54·1-63·7)	32·6<br>(27·5-36·6)	50·5<br>(42·6-55·9)	51·8<br>(43·0-57·8)	69·5<br>(51·8-84·5)	85·1<br>(82·4-87·8)	87·7<br>(85·1-90·1)	87·8<br>(85·1-90·2)	89·5<br>(84·8-93·8)	88·5<br>(86·0-90·6)	92·7<br>(89·5-95·1)	93·9<br>(91·1-96·0)	99·6<br>(98·4-100·0)
**Caribbean**																
Antigua and Barbuda	52·9<br>(49·9-56·0)	58·4<br>(55·1-61·6)	58·6<br>(55·2-61·7)	63·1<br>(58·8-67·6)	54·4<br>(44·8-58·9)	62·9<br>(52·8-68·1)	63·2<br>(53·2-68·5)	67·3<br>(54·8-77·7)	100·0<br>(100·0-100·0)	100·0<br>(100·0-100·0)	100·0<br>(100·0-100·0)	100·0<br>(100·0-100·0)	98·5<br>(97·1-99·2)	99·5<br>(98·9-99·9)	99·6<br>(99·1-99·9)	100·0<br>(99·8-100·0)
The Bahamas	56·6<br>(53·3-59·4)	60·2<br>(56·6-63·8)	60·1<br>(56·4-63·8)	63·0<br>(56·7-68·9)	67·7<br>(58·7-71·5)	74·1<br>(66·6-78·6)	74·1<br>(66·3-78·8)	77·4<br>(64·6-89·0)	87·2<br>(83·6-90·6)	93·9<br>(91·1-96·5)	93·9<br>(91·2-96·7)	96·2<br>(91·5-100·0)	88·5<br>(85·3-91·2)	94·7<br>(92·0-96·6)	95·7<br>(93·6-97·3)	99·5<br>(98·5-100·0)
Barbados	56·7<br>(53·4-60·2)	59·3<br>(56·1-63·1)	59·3<br>(56·0-63·1)	62·8<br>(58·0-68·8)	71·2<br>(61·9-75·6)	78·0<br>(64·9-82·8)	78·1<br>(65·1-83·2)	81·5<br>(66·5-93·6)	92·7<br>(89·4-95·7)	96·8<br>(94·3-98·9)	96·9<br>(94·3-99·1)	98·8<br>(95·0-100·0)	87·1<br>(82·2-90·5)	82·4<br>(75·0-87·7)	83·0<br>(75·1-88·7)	85·6<br>(64·9-96·1)
Belize	40·0<br>(36·9-43·4)	45·9<br>(42·5-49·3)	46·2<br>(42·8-49·6)	50·7<br>(46·3-54·8)	31·7<br>(22·0-36·0)	43·2<br>(30·2-49·1)	43·5<br>(30·2-49·9)	47·9<br>(28·0-63·8)	87·0<br>(84·0-89·9)	88·6<br>(85·0-91·6)	88·7<br>(85·0-91·6)	89·5<br>(83·2-94·8)	64·4<br>(59·8-68·9)	66·3<br>(57·7-74·2)	67·0<br>(57·5-75·6)	73·0<br>(46·3-89·8)
Cuba	52·4<br>(49·7-55·1)	59·9<br>(57·0-62·7)	60·2<br>(57·3-63·0)	64·6<br>(61·3-68·2)	64·3<br>(60·2-72·2)	78·0<br>(74·0-85·6)	78·7<br>(74·6-86·3)	87·0<br>(77·3-99·7)	27·8<br>(19·4-36·1)	57·9<br>(51·6-63·4)	58·7<br>(52·5-64·2)	76·8<br>(67·8-84·5)	90·0<br>(87·2-92·3)	94·6<br>(91·7-96·7)	94·9<br>(91·8-97·0)	97·7<br>(92·1-100·0)
Dominica	50·2<br>(46·8-53·5)	55·4<br>(52·1-58·8)	55·6<br>(52·3-59·1)	59·0<br>(55·2-62·8)	22·6<br>(10·0-27·4)	30·0<br>(15·4-36·6)	30·4<br>(15·3-37·2)	35·6<br>(15·5-51·9)	94·1<br>(91·0-96·8)	97·1<br>(94·3-99·3)	97·2<br>(94·4-99·3)	98·3<br>(93·7-100·0)	98·8<br>(97·8-99·4)	98·1<br>(96·8-99·0)	98·3<br>(97·1-99·1)	99·4<br>(96·7-100·0)
Dominican Republic	37·7<br>(34·6-40·9)	46·0<br>(42·4-49·5)	46·5<br>(42·9-50·0)	54·2<br>(50·2-58·4)	59·7<br>(48·5-65·8)	73·8<br>(55·4-82·7)	74·3<br>(56·2-83·3)	86·1<br>(60·3-100·0)	60·8<br>(57·2-64·2)	82·3<br>(78·0-85·6)	82·4<br>(77·9-85·7)	89·7<br>(80·5-96·9)	53·8<br>(48·3-59·3)	68·4<br>(61·7-74·5)	56·7<br>(40·4-69·6)	72·8<br>(51·9-86·9)
Grenada	48·0<br>(44·4-51·9)	52·4<br>(48·8-56·2)	52·5<br>(48·8-56·3)	56·8<br>(52·0-61·9)	39·2<br>(31·8-44·2)	54·1<br>(41·0-60·4)	53·7<br>(42·2-60·2)	70·0<br>(48·9-83·7)	84·2<br>(80·2-88·0)	85·2<br>(81·5-88·8)	85·3<br>(81·6-88·9)	86·6<br>(79·9-92·8)	93·0<br>(89·8-95·4)	97·5<br>(95·9-98·7)	97·7<br>(96·1-98·8)	99·1<br>(97·3-99·9)

Location	Indicator 5.2.1: Age-standardised prevalence of women aged 15 years and older who experienced physical or sexual violence by an intimate partner in the last 12 months (%)				Indicator 6.1.1: Risk-weighted prevalence of populations using unsafe or unimproved water sources, as measured by the summary exposure value (SEV) for unsafe water (%)				Indicator 6.2.1a: Risk-weighted prevalence of populations using unsafe or unimproved sanitation, as measured by the summary exposure value (SEV) for unsafe sanitation (%)				Indicator 6.2.1b: Risk-weighted prevalence of populations without access to a handwashing facility, as measured by the summary exposure value (SEV) for unsafe hygiene (%)			
	**2000**	**2015**	**2016**	**2030**	**2000**	**2015**	**2016**	**2030**	**2000**	**2015**	**2016**	**2030**	**2000**	**2015**	**2016**	**2030**
Azerbaijan	69·8 (66·6-72·6)	79·1 (76·6-81·3)	79·7 (77·2-81·9)	87·9 (84·6-90·9)	69·4 (56·6-86·5)	80·3 (70·2-92·1)	80·9 (71·2-92·4)	87·9 (74·5-96·9)	32·5 (19·6-49·0)	61·8 (31·6-83·4)	62·6 (31·3-84·3)	72·9 (34·4-93·3)	76·9 (64·9-87·2)	88·6 (80·2-94·4)	89·0 (80·8-94·6)	94·3 (88·3-97·7)
Georgia	84·0 (82·2-85·6)	87·5 (85·9-88·9)	87·9 (86·3-89·3)	91·6 (89·7-93·3)	62·7 (37·9-73·8)	73·9 (48·8-83·0)	74·5 (49·4-83·4)	81·7 (59·3-89·2)	55·2 (33·8-76·9)	57·4 (32·8-76·1)	57·6 (33·0-76·6)	61·2 (36·0-82·4)	84·7 (74·7-92·6)	92·0 (85·3-97·1)	92·2 (85·6-97·2)	95·1 (90·1-99·2)
Kazakhstan	70·8 (67·9-73·1)	77·0 (74·5-79·1)	77·7 (75·2-79·8)	86·0 (83·3-88·4)	78·0 (66·8-90·7)	82·0 (70·6-93·0)	82·5 (71·2-93·3)	87·8 (78·0-96·0)	50·5 (34·8-67·8)	61·6 (37·1-80·7)	62·3 (37·7-81·4)	71·5 (48·6-89·0)	84·2 (74·9-91·9)	89·6 (81·3-95·2)	89·9 (81·7-95·3)	93·8 (87·7-97·8)
Kyrgyzstan	55·5 (51·7-59·1)	58·4 (55·0-61·5)	59·0 (55·5-62·1)	61·4 (58·0-64·6)	59·8 (44·3-73·6)	72·2 (55·2-84·6)	72·8 (56·1-85·0)	80·7 (65·4-90·6)	35·1 (13·5-55·4)	34·1 (1·4-59·4)	34·3 (1·4-59·8)	38·1 (1·1-64·6)	80·7 (70·7-88·7)	88·8 (84·0-92·8)	89·1 (84·3-93·1)	93·3 (89·4-96·3)
Mongolia	57·4 (52·4-61·6)	64·9 (60·7-68·5)	65·7 (61·5-69·2)	73·0 (68·5-76·9)	68·9 (56·8-100·0)	59·3 (47·3-90·1)	59·3 (47·3-89·8)	59·6 (19·9-92·1)	22·6 (11·4-38·9)	37·7 (11·6-61·5)	38·2 (12·4-62·3)	48·6 (23·9-74·2)	62·5 (49·0-74·3)	69·4 (61·4-77·0)	70·1 (61·9-77·7)	74·8 (67·2-83·7)
Tajikistan	48·9 (44·1-53·0)	58·5 (54·3-61·8)	58·8 (54·8-62·1)	63·9 (59·8-67·7)	67·1 (54·4-97·8)	74·9 (64·3-100·0)	75·1 (64·3-100·0)	77·5 (64·3-100·0)	34·3 (6·1-59·0)	37·3 (3·2-63·7)	37·2 (3·0-63·9)	35·5 (1·9-66·4)	67·6 (55·4-78·3)	75·2 (65·8-83·3)	75·4 (66·0-83·5)	82·0 (72·2-88·9)
Turkmenistan	63·8 (59·6-67·8)	74·0 (70·6-77·2)	74·8 (71·5-78·0)	83·7 (80·7-86·4)	60·1 (45·7-75·6)	74·1 (61·0-84·9)	75·1 (62·2-85·6)	86·0 (76·2-92·9)	28·6 (11·6-54·0)	45·6 (25·6-67·9)	46·9 (26·6-69·1)	63·6 (40·9-83·9)	74·0 (59·2-84·7)	86·8 (78·0-93·6)	87·4 (78·9-94·0)	94·4 (89·6-98·6)
Uzbekistan	65·0 (60·7-68·8)	73·2 (69·9-76·5)	73·9 (70·6-77·1)	81·6 (78·7-84·5)	86·1 (75·0-100·0)	89·8 (79·7-100·0)	90·1 (80·2-100·0)	93·2 (81·0-100·0)	33·6 (6·1-58·0)	44·4 (12·6-68·8)	45·3 (13·3-69·6)	55·1 (22·0-78·2)	76·1 (64·2-87·1)	86·6 (77·4-93·5)	87·1 (78·2-93·9)	93·6 (87·4-98·0)
**Central Latin America**																
Colombia	45·9 (41·7-49·7)	61·5 (58·8-64·1)	62·4 (59·7-65·0)	74·7 (72·1-77·1)	70·1 (63·8-77·4)	79·6 (74·9-84·5)	80·4 (75·8-85·0)	88·9 (85·6-91·5)	74·8 (64·1-83·8)	91·8 (76·2-98·3)	92·1 (75·4-98·6)	95·8 (72·8-100·0)	73·2 (68·4-78·3)	78·7 (74·0-83·1)	79·2 (74·6-83·6)	84·2 (80·0-88·0)
Costa Rica	67·5 (64·3-70·3)	74·2 (71·8-76·5)	74·9 (72·6-77·2)	80·2 (78·0-82·4)	77·9 (70·9-84·7)	87·5 (82·4-91·7)	88·0 (83·0-92·0)	93·2 (89·7-95·8)	89·7 (74·2-97·9)	95·7 (85·5-99·9)	95·8 (85·9-99·9)	98·5 (93·7-100·0)	74·9 (69·9-79·8)	79·7 (75·4-83·4)	80·0 (75·7-83·6)	83·6 (80·1-86·7)
El Salvador	55·2 (51·9-58·5)	65·4 (62·4-68·3)	66·0 (63·0-68·9)	74·4 (71·4-77·2)	56·0 (49·1-63·6)	66·6 (60·2-72·5)	67·2 (60·9-73·0)	75·1 (69·7-80·2)	44·2 (31·4-57·3)	51·8 (38·0-66·1)	52·0 (37·4-67·1)	57·8 (34·4-81·2)	64·3 (58·0-70·2)	74·0 (68·8-79·1)	74·5 (69·4-79·6)	81·7 (77·3-86·9)
Guatemala	48·8 (45·2-52·6)	56·2 (52·6-60·2)	56·9 (53·3-60·8)	63·5 (59·6-67·7)	55·0 (46·4-77·4)	67·3 (60·7-82·8)	68·0 (61·4-83·1)	76·3 (53·8-90·3)	43·1 (36·8-49·5)	54·5 (41·4-67·0)	55·4 (41·7-68·3)	67·3 (44·9-84·6)	56·2 (49·4-62·7)	66·8 (60·9-72·5)	67·4 (61·5-73·0)	76·4 (71·4-80·9)
Honduras	46·2 (42·1-50·0)	56·1 (52·2-60·0)	56·9 (52·9-60·7)	66·0 (62·0-69·6)	52·7 (45·6-60·5)	60·5 (54·6-66·6)	61·2 (55·4-67·2)	70·0 (65·0-74·8)	41·3 (29·5-54·1)	62·4 (39·8-84·4)	63·3 (38·9-86·5)	76·9 (38·5-99·3)	73·3 (68·2-78·0)	77·6 (73·3-81·5)	78·0 (73·7-81·9)	81·8 (77·9-85·9)
Mexico	77·2 (74·5-79·4)	81·8 (79·9-83·4)	82·2 (80·3-83·8)	86·7 (85·2-87·9)	79·2 (77·1-84·1)	87·4 (85·8-91·4)	87·8 (86·2-91·7)	92·2 (91·0-95·0)	75·3 (71·4-78·3)	88·0 (84·2-90·8)	88·1 (84·3-91·0)	93·5 (89·8-96·3)	80·7 (79·7-81·7)	84·6 (83·7-85·5)	84·8 (83·8-85·7)	87·6 (86·2-89·4)
Nicaragua	55·5 (51·2-58·9)	66·3 (62·9-69·2)	66·9 (63·4-69·7)	73·9 (70·5-76·9)	46·2 (40·6-52·0)	55·4 (50·3-60·5)	56·1 (50·9-61·2)	64·4 (59·9-69·0)	23·3 (17·6-29·7)	46·8 (18·1-78·2)	48·2 (17·5-80·9)	69·4 (22·2-98·6)	61·9 (55·6-67·9)	73·3 (68·0-78·5)	74·0 (68·8-79·1)	82·7 (78·6-86·9)
Panama	69·7 (66·0-72·9)	75·4 (72·5-78·0)	76·2 (73·3-78·7)	80·3 (77·8-82·5)	73·2 (66·6-79·4)	78·8 (73·7-83·5)	79·3 (74·3-83·8)	85·1 (81·0-88·7)	51·9 (44·5-60·3)	74·5 (61·9-86·1)	76·0 (62·5-88·0)	90·5 (72·8-99·6)	80·4 (75·8-84·3)	84·5 (80·6-88·0)	84·8 (80·9-88·3)	88·3 (85·1-91·5)
Venezuela	62·2 (58·0-65·9)	69·2 (65·8-72·2)	69·8 (66·6-72·8)	78·6 (75·6-81·0)	79·1 (72·6-86·0)	87·0 (82·5-91·6)	87·4 (83·1-92·0)	92·7 (90·0-95·9)	87·7 (78·1-94·1)	90·3 (72·2-98·0)	90·5 (72·4-98·1)	92·9 (75·5-99·0)	79·1 (74·5-83·4)	82·9 (79·0-86·8)	83·2 (79·2-87·0)	86·2 (82·6-89·6)
**Andean Latin America**																
Bolivia	43·8 (39·2-48·0)	54·1 (50·1-57·8)	54·6 (50·6-58·2)	61·6 (58·1-65·2)	66·0 (56·0-81·5)	82·1 (72·5-93·0)	82·9 (73·4-93·4)	91·3 (74·6-99·3)	64·4 (35·3-87·4)	81·4 (50·2-96·5)	82·3 (51·5-96·8)	91·7 (69·5-99·0)	62·9 (57·0-68·7)	73·5 (68·5-78·5)	74·0 (69·1-79·0)	81·9 (78·2-86·2)
Ecuador	60·0 (56·7-63·2)	66·8 (63·6-69·8)	67·5 (64·3-70·5)	73·4 (70·5-76·3)	75·1 (68·5-87·9)	85·9 (78·0-93·9)	86·3 (78·3-94·1)	91·1 (83·0-96·6)	76·2 (62·4-86·9)	77·1 (58·3-90·8)	77·0 (58·1-90·8)	76·2 (56·2-91·0)	66·2 (60·5-71·7)	75·5 (70·4-80·5)	75·8 (70·7-80·8)	82·7 (77·9-87·9)
Peru	64·5 (61·5-67·1)	72·8 (70·6-74·8)	73·3 (71·2-75·3)	80·5 (78·5-82·3)	79·6 (72·2-97·4)	86·9 (80·9-98·9)	87·3 (81·3-99·0)	92·5 (86·5-100·0)	58·5 (38·8-74·4)	61·6 (37·5-78·3)	61·6 (36·8-78·5)	62·2 (29·0-80·6)	63·8 (58·3-69·2)	73·6 (68·1-78·4)	74·2 (68·7-78·9)	81·8 (76·9-85·5)
**Caribbean**																
Antigua and Barbuda	80·3 (77·2-82·9)	83·2 (80·8-85·1)	83·6 (81·2-85·5)	88·5 (85·9-90·8)	57·7 (49·9-70·4)	62·8 (54·7-73·3)	63·1 (54·9-73·5)	67·2 (34·8-87·1)	74·8 (62·4-85·9)	79·8 (55·0-94·0)	79·9 (54·9-94·1)	82·4 (55·9-96·5)	72·1 (66·5-77·2)	76·4 (71·6-80·9)	76·6 (72·0-81·1)	80·0 (75·9-84·6)
The Bahamas	72·3 (69·0-75·2)	75·6 (72·8-78·0)	76·1 (73·4-78·5)	81·5 (79·1-83·4)	66·6 (58·3-78·7)	68·8 (60·7-79·3)	68·9 (60·8-79·4)	70·7 (27·7-93·4)	85·4 (70·5-94·2)	83·6 (62·5-95·0)	83·6 (62·3-95·0)	82·6 (59·0-95·3)	80·1 (75·3-83·9)	80·6 (75·6-84·7)	80·7 (75·6-84·7)	81·1 (76·0-85·4)
Barbados	81·5 (79·1-83·7)	84·9 (83·0-86·6)	85·2 (83·4-86·9)	89·6 (88·0-91·0)	71·4 (62·0-82·5)	75·2 (66·3-85·8)	75·3 (66·5-85·9)	77·7 (55·3-92·6)	77·3 (46·9-94·4)	81·0 (51·3-96·3)	81·2 (51·7-96·3)	83·7 (56·6-97·3)	76·5 (71·7-80·8)	77·5 (73·4-81·6)	77·5 (73·4-81·6)	78·6 (74·9-82·5)
Belize	52·0 (46·5-56·6)	58·0 (53·4-62·1)	58·5 (53·9-62·5)	64·7 (60·2-68·2)	27·8 (23·0-33·6)	29·6 (24·6-35·0)	30·0 (25·0-35·4)	35·5 (28·7-42·3)	55·4 (44·1-69·1)	71·1 (52·0-86·4)	71·6 (51·7-87·5)	81·3 (54·3-97·6)	68·6 (63·5-73·7)	67·8 (62·7-72·5)	68·2 (63·1-72·9)	66·4 (60·4-76·2)
Cuba	77·8 (74·7-80·4)	86·0 (84·1-87·6)	86·5 (84·7-88·1)	93·2 (91·0-95·1)	51·0 (41·8-64·6)	65·9 (57·4-78·2)	66·8 (58·4-78·9)	77·8 (34·9-97·7)	69·1 (41·9-88·1)	79·1 (65·9-89·9)	79·8 (66·3-90·7)	88·2 (73·5-97·7)	75·3 (70·4-79·5)	81·1 (77·3-84·6)	81·5 (77·7-84·9)	85·9 (82·6-88·9)
Dominica	73·8 (70·4-76·8)	76·9 (74·1-79·4)	77·2 (74·3-79·6)	81·1 (78·4-83·2)	49·5 (42·1-61·4)	56·5 (48·7-67·8)	57·0 (49·2-68·3)	62·9 (55·8-74·3)	57·3 (24·7-85·4)	70·4 (31·9-91·8)	71·0 (32·5-92·0)	80·8 (43·4-95·7)	64·6 (58·5-70·3)	71·2 (65·7-76·4)	71·6 (66·2-76·7)	76·7 (71·5-81·7)
Dominican Republic	58·6 (54·8-61·8)	66·3 (63·4-68·9)	66·8 (63·9-69·4)	73·6 (71·0-76·0)	27·3 (20·4-38·7)	31·4 (25·2-38·5)	31·8 (25·7-38·6)	37·1 (31·8-42·4)	54·4 (44·7-66·1)	78·1 (63·5-88·8)	79·0 (64·7-89·4)	91·4 (82·6-96·8)	36·8 (30·8-42·8)	35·4 (30·2-40·5)	35·6 (30·4-40·8)	32·2 (26·7-37·8)
Grenada	66·8 (62·4-70·7)	73·1 (69·5-76·5)	73·6 (70·1-77·0)	79·6 (75·5-83·4)	46·5 (37·4-61·8)	60·9 (52·1-76·2)	61·6 (52·7-76·3)	69·7 (41·6-90·1)	54·1 (43·2-66·6)	72·9 (43·1-91·4)	73·4 (42·5-91·9)	84·6 (49·4-98·3)	68·8 (63·2-74·0)	77·3 (72·3-81·8)	77·6 (72·6-82·1)	83·8 (79·2-89·1)

Location	Indicator 7.1.2: Risk-weighted prevalence of household air pollution, as measured by the summary exposure value (SEV) for household air pollution (%)				Indicator 8.8.1: Age-standardised all-cause disability-adjusted life year (DALY) rates attributable to occupational risks (per 100,000 population)				Indicator 11.6.2: Population-weighted mean levels of fine particulate matter smaller than 2.5 microns in diameter (PM2.5)				Indicator 16.1.1: Age-standardised death rate due to interpersonal violence (per 100,000 population)			
	**2000**	**2015**	**2016**	**2030**	**2000**	**2015**	**2016**	**2030**	**2000**	**2015**	**2016**	**2030**	**2000**	**2015**	**2016**	**2030**
Azerbaijan	81·8 (70·5-90·0)	91·7 (76·1-98·5)	91·8 (75·8-98·6)	93·5 (71·6-99·5)	49·1 (42·7-55·4)	52·4 (45·3-60·1)	53·6 (46·4-61·3)	69·9 (61·3-79·0)	50·6 (45·2-54·6)	40·0 (35·2-43·9)	39·6 (34·0-43·6)	36·4 (27·4-46·8)	42·7 (36·5-48·8)	52·4 (39·7-59·8)	52·1 (40·1-59·9)	50·4 (30·5-69·0)
Georgia	68·8 (55·3-80·5)	73·6 (53·5-91·5)	74·4 (53·8-92·4)	83·5 (58·8-98·6)	50·3 (43·2-56·5)	48·4 (41·2-55·9)	49·3 (42·0-56·8)	61·6 (52·9-70·1)	64·6 (61·4-67·4)	55·4 (51·9-58·6)	55·3 (51·8-58·6)	51·2 (47·1-55·0)	54·5 (48·4-65·4)	53·9 (44·5-61·1)	53·9 (44·5-61·9)	54·4 (34·7-71·7)
Kazakhstan	87·1 (78·1-93·5)	91·9 (79·2-98·0)	92·2 (79·0-98·3)	95·4 (79·4-99·8)	11·7 (4·1-18·9)	34·5 (26·3-42·1)	35·9 (27·6-43·5)	56·2 (47·0-64·7)	62·8 (60·1-65·2)	57·1 (53·9-59·8)	57·1 (53·6-59·7)	56·1 (51·8-62·5)	13·3 (5·9-19·2)	30·0 (16·4-36·5)	29·4 (15·2-37·4)	49·0 (25·9-66·8)
Kyrgyzstan	69·1 (51·2-85·1)	81·6 (69·8-90·4)	82·4 (70·5-91·0)	90·8 (80·1-96·9)	34·0 (28·4-39·9)	57·6 (51·7-63·7)	59·3 (53·4-65·6)	84·8 (77·2-92·6)	66·3 (63·5-68·8)	60·4 (57·3-62·8)	60·2 (57·3-62·9)	59·7 (56·7-62·6)	31·3 (24·2-37·2)	42·9 (33·1-47·5)	43·0 (32·8-48·3)	55·5 (42·1-66·3)
Mongolia	49·2 (38·7-59·8)	63·4 (49·8-77·0)	64·4 (50·4-78·2)	76·9 (58·9-91·4)	42·9 (35·5-50·0)	57·5 (50·3-64·5)	59·0 (51·7-66·0)	79·8 (71·4-87·7)	57·9 (55·8-59·9)	43·7 (40·8-46·2)	43·1 (40·1-45·7)	34·2 (29·7-41·2)	24·9 (18·5-30·5)	30·4 (23·0-36·4)	30·3 (22·8-36·6)	33·6 (18·4-47·4)
Tajikistan	51·0 (38·0-63·5)	75·4 (58·9-89·1)	75·6 (58·2-89·7)	78·3 (50·7-96·2)	41·8 (33·8-49·6)	65·2 (56·3-73·2)	65·4 (56·5-73·5)	70·0 (59·9-79·0)	18·0 (10·5-22·6)	19·1 (11·5-23·8)	18·7 (11·4-23·5)	18·3 (5·7-30·9)	37·8 (31·9-47·4)	60·3 (43·7-68·1)	60·0 (43·5-68·3)	65·0 (42·3-84·2)
Turkmenistan	99·2 (96·7-99·9)	99·8 (99·1-100·0)	99·8 (99·2-100·0)	100·0 (99·8-100·0)	29·7 (21·4-37·6)	46·8 (38·9-54·9)	47·5 (39·5-55·4)	56·4 (48·1-64·7)	37·6 (33·0-40·6)	35·5 (29·8-38·9)	35·1 (29·9-38·4)	34·5 (26·3-43·0)	40·3 (31·2-45·1)	46·5 (33·9-53·2)	46·5 (34·1-53·4)	51·4 (35·9-63·6)
Uzbekistan	84·6 (71·5-93·7)	91·4 (74·7-98·3)	91·7 (74·6-98·5)	95·2 (75·9-99·8)	40·2 (32·8-47·7)	57·3 (50·0-65·0)	58·2 (50·9-65·8)	70·5 (63·3-77·9)	32·9 (28·8-35·6)	28·2 (24·5-30·9)	27·8 (23·9-30·5)	34·5 (29·3-39·7)	49·7 (41·8-54·7)	55·3 (41·8-62·1)	55·3 (41·7-62·6)	59·7 (41·0-74·3)
**Central Latin America**																
Colombia	86·0 (82·3-89·1)	91·0 (85·1-95·1)	91·7 (85·7-95·6)	97·2 (92·5-99·3)	78·2 (73·0-83·1)	97·8 (93·0-100·0)	98·9 (94·7-100·0)	100·0 (100·0-100·0)	55·8 (54·5-57·1)	61·7 (60·4-63·0)	61·7 (60·4-63·0)	63·7 (60·6-66·2)	0·0 (0·0-0·0)	1·3 (0·0-4·8)	1·5 (0·0-5·2)	9·7 (0·8-18·3)
Costa Rica	91·1 (89·7-92·4)	96·4 (94·3-97·7)	96·6 (94·4-97·9)	98·4 (95·9-99·5)	79·5 (73·8-85·0)	92·9 (87·5-98·2)	93·4 (87·9-98·8)	99·0 (94·7-100·0)	53·2 (50·4-55·7)	59·0 (56·4-61·4)	59·1 (56·5-61·5)	60·2 (56·8-64·3)	40·5 (32·9-45·8)	36·9 (30·9-47·3)	37·4 (31·2-47·7)	37·8 (25·9-52·3)
El Salvador	68·5 (61·4-75·2)	89·2 (81·3-94·4)	89·9 (82·0-95·0)	96·4 (90·5-99·1)	100·0 (99·4-100·0)	100·0 (100·0-100·0)	100·0 (100·0-100·0)	100·0 (100·0-100·0)	30·8 (27·9-33·9)	38·9 (36·0-41·6)	39·2 (36·4-42·1)	40·2 (32·6-48·9)	0·0 (0·0-0·0)	0·0 (0·0-0·0)	0·0 (0·0-0·0)	0·1 (0·0-2·7)
Guatemala	47·6 (40·6-54·2)	66·5 (52·4-80·3)	68·1 (53·9-81·6)	84·5 (72·1-94·2)	16·8 (8·5-25·0)	37·7 (30·0-45·1)	38·5 (30·8-45·9)	49·7 (41·7-57·6)	34·2 (32·8-35·7)	44·3 (42·9-45·6)	44·5 (43·1-45·9)	45·1 (41·3-48·7)	0·3 (0·0-3·1)	0·6 (0·0-4·8)	0·8 (0·0-5·8)	4·6 (0·0-21·3)
Honduras	49·2 (42·5-56·0)	65·5 (56·3-74·0)	66·8 (57·2-75·3)	80·7 (69·2-88·9)	42·3 (31·0-64·3)	41·3 (28·4-65·9)	41·5 (28·6-66·2)	45·2 (31·4-72·4)	35·4 (33·2-37·4)	43·0 (40·6-45·3)	43·3 (41·0-45·7)	41·3 (37·5-44·4)	1·2 (0·0-7·1)	1·3 (0·0-7·7)	1·5 (0·0-7·9)	5·6 (0·0-19·4)
Mexico	87·0 (85·4-88·5)	93·2 (92·0-94·3)	93·7 (92·6-94·8)	97·9 (97·4-98·3)	61·1 (56·5-65·2)	73·1 (68·1-77·6)	73·6 (68·6-78·1)	81·0 (75·6-86·0)	44·0 (42·6-45·5)	58·4 (57·1-59·6)	58·5 (57·3-59·7)	63·1 (55·0-69·7)	20·6 (13·4-25·0)	17·4 (12·4-24·1)	17·8 (12·5-24·2)	22·4 (15·3-28·2)
Nicaragua	47·9 (41·1-54·9)	69·1 (55·8-81·5)	70·6 (56·9-82·9)	86·0 (72·9-95·2)	89·1 (83·2-94·9)	90·8 (84·9-96·2)	91·2 (85·3-96·7)	96·3 (89·9-100·0)	38·3 (31·0-43·3)	51·9 (45·5-56·5)	52·3 (46·2-56·8)	55·3 (46·9-62·6)	26·0 (20·0-29·9)	29·0 (21·2-34·4)	29·2 (20·7-35·4)	32·3 (16·3-45·4)
Panama	85·7 (82·0-89·0)	92·4 (87·2-96·1)	92·9 (87·6-96·5)	97·4 (93·1-99·4)	78·5 (73·6-83·4)	89·9 (84·5-94·9)	91·2 (85·8-96·4)	100·0 (100·0-100·0)	63·5 (58·4-67·4)	68·6 (63·6-72·4)	68·3 (63·4-72·2)	70·8 (64·4-80·3)	29·0 (22·7-35·1)	19·1 (11·4-30·6)	19·8 (11·7-31·3)	22·1 (5·6-40·4)
Venezuela	98·0 (96·1-99·1)	99·4 (98·7-99·8)	99·5 (98·8-99·8)	99·8 (99·6-100·0)	9·4 (0·0-27·1)	20·0 (9·7-37·8)	20·8 (10·3-38·9)	32·0 (18·0-58·4)	40·0 (37·3-42·3)	47·5 (45·3-49·5)	47·2 (44·4-49·8)	57·6 (54·3-61·0)	5·1 (0·4-12·2)	1·6 (0·0-11·0)	1·7 (0·0-11·3)	3·3 (0·0-18·0)
**Andean Latin America**																
Bolivia	70·7 (66·8-74·5)	85·8 (78·0-91·7)	86·8 (78·7-92·7)	95·3 (88·2-98·8)	47·2 (39·7-53·8)	53·0 (44·7-61·5)	53·3 (44·9-61·9)	56·8 (47·8-66·1)	42·5 (40·9-44·1)	52·8 (51·2-54·4)	53·2 (51·7-54·7)	49·3 (46·9-51·3)	37·5 (30·8-43·4)	40·5 (32·5-48·9)	40·6 (32·5-49·1)	42·9 (28·1-58·9)
Ecuador	89·3 (85·9-92·0)	96·8 (93·9-98·5)	97·0 (94·3-98·7)	99·0 (97·5-99·8)	50·7 (38·6-61·2)	57·5 (45·7-68·4)	57·8 (45·9-68·9)	62·6 (48·3-75·4)	60·1 (58·7-61·5)	70·7 (69·3-72·0)	70·6 (69·2-72·0)	70·0 (68·0-71·7)	13·9 (9·2-21·4)	21·9 (15·4-28·2)	22·1 (15·6-28·8)	28·5 (19·3-37·6)
Peru	72·0 (67·7-76·2)	82·6 (77·1-87·3)	83·4 (78·0-88·2)	92·3 (87·4-95·7)	62·1 (54·4-69·4)	69·7 (60·8-78·2)	70·4 (61·5-79·1)	80·6 (69·8-91·2)	37·2 (34·6-39·2)	47·5 (45·2-49·9)	47·5 (45·1-49·8)	37·1 (34·1-39·7)	50·1 (44·1-54·6)	56·0 (48·5-63·1)	56·5 (48·5-63·8)	63·1 (44·7-78·7)
**Caribbean**																
Antigua and Barbuda	97·9 (96·2-99·0)	99·0 (98·2-99·6)	99·1 (98·3-99·6)	99·6 (99·2-99·9)	74·2 (63·1-83·5)	86·7 (77·1-95·9)	87·2 (77·6-96·4)	94·9 (85·1-100·0)	62·9 (43·4-81·8)	66·4 (47·1-84·9)	66·1 (45·5-84·4)	67·0 (45·4-86·4)	49·7 (41·7-54·6)	45·8 (38·6-55·4)	46·2 (39·0-55·7)	50·3 (36·7-63·3)
The Bahamas	96·9 (93·9-98·7)	98·2 (96·3-99·2)	98·2 (96·5-99·2)	99·0 (98·0-99·6)	87·0 (78·0-94·7)	94·2 (85·0-100·0)	93·7 (84·5-100·0)	87·2 (77·4-95·8)	60·7 (43·6-74·8)	72·1 (56·6-87·1)	72·5 (55·7-87·1)	76·9 (60·0-91·8)	18·2 (11·9-22·7)	13·5 (6·4-26·0)	13·9 (6·4-26·5)	14·5 (0·0-35·8)
Barbados	99·9 (99·8-100·0)	100·0 (99·9-100·0)	100·0 (99·9-100·0)	100·0 (100·0-100·0)	94·2 (80·9-100·0)	93·6 (80·3-100·0)	93·0 (79·7-100·0)	84·9 (72·4-94·3)	57·4 (34·3-79·7)	61·5 (40·4-81·7)	61·1 (40·2-84·3)	62·4 (36·7-88·6)	34·4 (28·2-39·5)	30·9 (24·3-41·4)	31·1 (24·3-41·7)	34·6 (21·8-48·6)
Belize	86·4 (82·2-89·8)	90·8 (86·3-94·1)	91·1 (86·5-94·5)	94·7 (88·6-98·0)	45·4 (22·3-62·1)	54·0 (30·1-70·3)	53·8 (30·1-70·1)	51·3 (32·8-67·4)	43·4 (39·9-46·9)	55·9 (52·6-59·2)	56·2 (52·8-59·5)	59·3 (51·2-68·0)	13·5 (6·7-19·1)	11·6 (4·1-22·6)	12·0 (3·8-23·0)	16·0 (0·0-34·2)
Cuba	96·5 (94·0-98·2)	98·7 (97·7-99·4)	98·8 (97·7-99·4)	99·5 (98·7-99·9)	40·7 (23·3-62·8)	58·6 (36·6-85·1)	59·8 (37·6-85·9)	77·4 (53·0-99·4)	53·7 (50·5-56·3)	62·6 (59·6-65·2)	62·7 (59·5-65·4)	67·0 (63·8-69·8)	43·3 (35·6-47·4)	45·5 (40·0-52·4)	45·6 (40·1-52·3)	48·3 (39·0-58·1)
Dominica	87·7 (82·3-91·7)	95·7 (92·0-98·1)	96·0 (92·4-98·2)	98·5 (96·5-99·5)	86·8 (76·1-95·3)	91·8 (81·7-100·0)	92·0 (81·9-100·0)	93·8 (83·9-100·0)	61·0 (45·9-75·5)	63·2 (47·2-77·4)	63·0 (47·5-76·8)	65·5 (49·9-81·6)	45·1 (33·6-51·1)	30·3 (22·6-42·8)	30·3 (22·0-42·7)	25·5 (10·0-43·5)
Dominican Republic	88·2 (85·2-90·9)	93·5 (90·2-95·9)	93·8 (90·6-96·2)	97·0 (94·4-98·6)	61·5 (34·8-80·1)	65·4 (41·1-84·7)	65·9 (41·6-85·4)	73·1 (46·4-93·4)	43·3 (34·9-49·4)	51·9 (44·1-57·6)	50·9 (43·0-57·0)	50·7 (40·2-59·2)	25·3 (16·7-30·2)	21·9 (15·0-29·9)	22·1 (14·8-30·3)	23·6 (10·1-38·1)
Grenada	93·6 (90·3-96·1)	98·3 (96·6-99·2)	98·4 (96·7-99·3)	99·4 (98·6-99·8)	71·5 (60·6-80·8)	79·5 (68·9-90·2)	80·4 (69·9-91·2)	93·2 (82·6-100·0)	58·3 (37·6-79·1)	61·8 (41·2-83·1)	60·9 (38·5-82·0)	62·0 (36·2-87·7)	42·9 (35·5-51·3)	38·8 (31·4-48·3)	39·2 (31·7-49·0)	37·3 (21·4-54·4)

Location	Indicator 16.1.2: Death rate due to conflict and terrorism (per 100,000 population)				Indicator 16.1.3: Age-standardised prevalence of physical or sexual violence experienced by populations in the last 12 months (%)				Indicator 16.2.3: Age-standardised prevalence of women and men aged 18-29 years who experienced sexual violence by age 18 (%)				Indicator 17.19.2c: Percentage of well-certified deaths by a vital registration (VR) system among a country's total population (%)			
	**2000**	**2015**	**2016**	**2030**	**2000**	**2015**	**2016**	**2030**	**2000**	**2015**	**2016**	**2030**	**2000**	**2015**	**2016**	**2030**
Azerbaijan	100·0 (100·0-100·0)	52·8 (48·3-56·5)	100·0 (100·0-100·0)	100·0 (100·0-100·0)	84·3 (82·6-86·2)	91·3 (89·9-92·7)	91·4 (90·0-92·8)	93·1 (91·0-95·0)	99·9 (99·8-100·0)	99·9 (99·7-100·0)	99·9 (99·6-100·0)	99·8 (99·4-100·0)	74·7 (69·0-80·3)	72·5 (52·6-88·5)	73·7 (53·9-89·7)	79·2 (41·0-100·0)
Georgia	58·9 (50·9-70·2)	100·0 (100·0-100·0)	100·0 (100·0-100·0)	100·0 (100·0-100·0)	96·4 (95·3-97·5)	98·8 (97·8-99·8)	99·0 (98·0-100·0)	100·0 (100·0-100·0)	100·0 (100·0-100·0)	100·0 (100·0-100·0)	100·0 (100·0-100·0)	100·0 (100·0-100·0)	78·7 (71·1-85·4)	58·5 (47·7-69·3)	59·9 (47·4-72·6)	45·4 (24·7-68·1)
Kazakhstan	100·0 (100·0-100·0)	100·0 (100·0-100·0)	100·0 (100·0-100·0)	100·0 (100·0-100·0)	89·9 (88·4-91·2)	95·0 (93·9-96·1)	95·4 (94·2-96·4)	99·7 (98·7-100·0)	96·6 (94·8-98·4)	96·1 (94·3-97·9)	95·9 (94·1-97·8)	95·7 (93·9-97·6)	86·2 (81·6-90·3)	86·8 (79·8-92·4)	87·3 (78·6-93·6)	76·8 (62·8-88·2)
Kyrgyzstan	57·5 (50·5-66·7)	100·0 (100·0-100·0)	100·0 (100·0-100·0)	100·0 (100·0-100·0)	66·4 (63·8-68·7)	71·7 (69·5-73·6)	72·0 (69·9-74·0)	76·8 (74·8-78·6)	100·0 (99·9-100·0)	100·0 (99·9-100·0)	99·9 (99·8-100·0)	100·0 (99·9-100·0)	83·4 (79·0-87·4)	95·0 (90·2-98·7)	95·1 (89·2-99·3)	98·4 (91·3-100·0)
Mongolia	100·0 (100·0-100·0)	100·0 (100·0-100·0)	100·0 (100·0-100·0)	100·0 (100·0-100·0)	78·6 (75·9-81·2)	83·2 (80·8-85·5)	83·4 (81·1-85·8)	87·3 (85·0-89·3)	100·0 (100·0-100·0)	99·9 (99·6-100·0)	99·8 (99·4-100·0)	99·8 (99·1-100·0)	10·4 (4·8-20·0)	25·3 (12·1-43·7)	24·7 (11·6-42·1)	15·3 (1·5-48·7)
Tajikistan	51·0 (44·2-59·9)	100·0 (100·0-100·0)	100·0 (100·0-100·0)	100·0 (100·0-100·0)	63·9 (61·3-66·3)	75·1 (73·1-76·9)	75·2 (73·2-77·0)	77·8 (75·9-79·6)	100·0 (99·9-100·0)	100·0 (100·0-100·0)	100·0 (99·9-100·0)	100·0 (99·9-100·0)	44·3 (38·7-50·4)	56·4 (35·1-77·6)	56·7 (34·6-78·3)	62·7 (21·2-98·5)
Turkmenistan	100·0 (100·0-100·0)	100·0 (100·0-100·0)	100·0 (100·0-100·0)	100·0 (100·0-100·0)	82·5 (79·8-84·6)	89·5 (87·4-91·4)	90·0 (87·9-91·8)	95·9 (94·1-97·5)	100·0 (99·9-100·0)	100·0 (100·0-100·0)	100·0 (99·9-100·0)	100·0 (99·9-100·0)	67·1 (61·2-72·5)	76·0 (67·0-83·4)	77·0 (66·4-86·0)	81·4 (60·7-95·6)
Uzbekistan	44·9 (37·7-54·8)	100·0 (100·0-100·0)	100·0 (100·0-100·0)	100·0 (100·0-100·0)	83·4 (81·1-85·7)	89·1 (87·0-91·0)	89·5 (87·4-91·3)	94·2 (92·4-95·8)	100·0 (99·9-100·0)	100·0 (99·9-100·0)	100·0 (99·9-100·0)	99·9 (99·7-100·0)	63·9 (58·2-69·7)	70·2 (60·8-78·9)	70·9 (59·5-81·4)	75·4 (46·9-97·2)
**Central Latin America**																
Colombia	27·3 (23·3-31·0)	63·6 (59·0-66·8)	100·0 (100·0-100·0)	100·0 (100·0-100·0)	56·5 (53·1-60·0)	66·5 (63·7-69·4)	66·7 (64·0-69·5)	70·0 (67·1-73·1)	65·8 (57·3-73·0)	65·5 (56·9-72·9)	65·5 (56·9-72·9)	65·3 (56·2-72·7)	89·0 (86·3-91·5)	93·5 (90·6-95·9)	93·9 (90·9-96·5)	98·7 (94·0-100·0)
Costa Rica	100·0 (100·0-100·0)	100·0 (100·0-100·0)	100·0 (100·0-100·0)	100·0 (100·0-100·0)	78·2 (76·4-79·8)	81·7 (80·0-83·2)	82·0 (80·4-83·5)	87·0 (85·4-88·3)	18·1 (0·7-32·8)	16·4 (0·0-32·0)	16·0 (0·0-32·3)	15·6 (0·0-32·9)	94·6 (92·3-96·7)	97·1 (95·0-99·2)	97·4 (95·1-99·5)	99·7 (96·9-100·0)
El Salvador	100·0 (100·0-100·0)	53·8 (48·4-59·1)	100·0 (100·0-100·0)	100·0 (100·0-100·0)	81·3 (79·3-83·4)	86·9 (85·1-88·6)	87·1 (85·3-88·9)	90·7 (88·9-92·5)	71·7 (66·6-76·9)	71·7 (66·5-76·8)	71·5 (66·4-76·7)	71·5 (66·2-76·7)	67·6 (63·7-71·3)	70·7 (65·2-75·6)	71·8 (66·1-77·1)	76·7 (67·1-85·1)
Guatemala	72·6 (67·8-76·6)	71·7 (66·8-75·9)	100·0 (100·0-100·0)	100·0 (100·0-100·0)	89·8 (88·2-91·5)	94·5 (92·9-96·0)	94·8 (93·3-96·3)	99·3 (97·9-100·0)	76·9 (72·2-81·5)	75·7 (70·9-80·4)	75·5 (70·8-80·3)	74·5 (69·4-79·3)	71·8 (68·3-75·3)	79·4 (74·9-83·5)	80·0 (75·4-84·3)	84·7 (76·4-91·4)
Honduras	100·0 (100·0-100·0)	59·6 (54·0-64·9)	100·0 (100·0-100·0)	100·0 (100·0-100·0)	82·8 (80·5-85·2)	88·5 (86·5-90·5)	88·9 (86·9-90·9)	94·1 (92·3-95·9)	65·9 (60·1-71·5)	63·9 (57·8-69·9)	63·6 (57·4-69·5)	63·3 (57·1-69·3)	14·2 (10·8-18·2)	16·8 (13·7-20·3)	17·3 (14·1-21·2)	23·8 (14·2-36·9)
Mexico	91·4 (88·6-93·5)	43·5 (34·1-59·2)	100·0 (100·0-100·0)	100·0 (100·0-100·0)	100·0 (100·0-100·0)	100·0 (100·0-100·0)	100·0 (100·0-100·0)	100·0 (100·0-100·0)	79·8 (75·3-84·4)	79·2 (74·5-84·0)	79·1 (74·4-83·9)	78·6 (73·7-83·5)	82·4 (81·6-83·2)	92·1 (91·2-93·0)	92·3 (91·4-93·2)	97·8 (96·8-98·8)
Nicaragua	57·5 (52·1-62·5)	100·0 (100·0-100·0)	100·0 (100·0-100·0)	100·0 (100·0-100·0)	86·5 (84·6-88·4)	92·4 (90·7-94·0)	92·4 (90·7-94·0)	92·5 (89·9-94·8)	76·9 (71·3-81·1)	77·2 (72·1-81·5)	76·9 (71·7-81·2)	76·6 (71·3-81·1)	69·2 (65·5-72·7)	89·0 (85·3-92·3)	89·4 (85·5-92·8)	98·6 (94·5-100·0)
Panama	100·0 (100·0-100·0)	100·0 (100·0-100·0)	100·0 (100·0-100·0)	100·0 (100·0-100·0)	92·0 (88·6-94·8)	93·8 (90·6-96·5)	94·1 (90·9-96·8)	98·4 (95·6-100·0)	65·6 (57·2-72·8)	65·5 (57·0-72·9)	65·6 (57·1-72·9)	65·5 (56·8-72·9)	83·9 (80·9-86·9)	90·9 (87·8-93·6)	91·5 (88·4-94·4)	96·9 (91·3-100·0)
Venezuela	76·7 (66·9-90·6)	100·0 (100·0-100·0)	100·0 (100·0-100·0)	100·0 (100·0-100·0)	87·1 (83·5-90·4)	89·6 (86·3-92·7)	89·9 (86·5-92·9)	93·4 (90·3-96·2)	65·8 (57·3-72·9)	65·5 (57·0-72·9)	65·5 (56·9-72·9)	65·3 (56·3-72·8)	94·1 (91·8-96·1)	96·3 (93·9-98·4)	96·5 (94·1-98·6)	98·8 (95·3-100·0)
**Andean Latin America**																
Bolivia	54·9 (44·8-73·4)	100·0 (100·0-100·0)	100·0 (100·0-100·0)	100·0 (100·0-100·0)	14·0 (8·5-19·9)	27·8 (23·0-33·1)	28·4 (23·6-33·7)	38·0 (31·6-43·9)	46·4 (37·6-55·6)	46·5 (37·6-55·4)	46·5 (37·6-55·4)	46·5 (37·6-55·5)	12·4 (6·2-21·5)	29·3 (7·8-64·6)	30·3 (7·8-65·8)	51·5 (5·1-100·0)
Ecuador	100·0 (100·0-100·0)	100·0 (100·0-100·0)	100·0 (100·0-100·0)	100·0 (100·0-100·0)	69·0 (66·9-71·5)	73·6 (71·6-75·8)	74·1 (72·1-76·3)	80·5 (78·7-82·4)	46·4 (37·5-55·4)	46·5 (37·6-55·4)	46·5 (37·6-55·4)	46·5 (37·6-55·5)	64·8 (58·6-70·5)	73·3 (60·3-84·8)	73·6 (55·5-87·7)	78·0 (50·0-97·9)
Peru	100·0 (100·0-100·0)	100·0 (100·0-100·0)	100·0 (100·0-100·0)	100·0 (100·0-100·0)	74·7 (71·1-77·9)	81·6 (78·6-84·2)	82·0 (79·0-84·5)	87·3 (84·7-89·5)	48·0 (38·0-56·0)	46·7 (34·7-56·7)	46·4 (33·6-57·0)	46·5 (33·5-57·1)	53·5 (44·8-61·9)	63·1 (47·7-76·3)	63·4 (44·1-79·6)	69·1 (40·2-88·3)
**Caribbean**																
Antigua and Barbuda	100·0 (100·0-100·0)	100·0 (100·0-100·0)	100·0 (100·0-100·0)	100·0 (100·0-100·0)	90·5 (88·4-92·4)	92·0 (89·8-93·9)	92·2 (90·1-94·1)	95·1 (93·0-96·9)	74·4 (70·3-78·7)	75·0 (71·6-78·6)	75·0 (71·5-78·5)	75·3 (71·8-78·8)	79·4 (74·8-83·5)	78·5 (71·5-84·8)	79·2 (71·3-86·2)	82·0 (67·8-93·4)
The Bahamas	100·0 (100·0-100·0)	100·0 (100·0-100·0)	100·0 (100·0-100·0)	100·0 (100·0-100·0)	85·5 (82·7-87·7)	85·8 (83·1-88·1)	86·1 (83·4-88·3)	89·0 (86·5-91·1)	74·3 (70·7-78·3)	74·6 (71·1-78·2)	74·5 (71·0-78·2)	74·5 (70·9-78·2)	78·0 (73·2-82·3)	79·5 (72·3-86·2)	79·8 (71·6-86·5)	83·2 (67·7-94·4)
Barbados	100·0 (100·0-100·0)	100·0 (100·0-100·0)	100·0 (100·0-100·0)	100·0 (100·0-100·0)	89·9 (87·6-92·0)	89·4 (87·2-91·6)	89·5 (87·3-91·6)	90·8 (88·8-93·0)	74·3 (70·6-78·3)	74·6 (71·0-78·3)	74·6 (71·0-78·3)	74·8 (71·3-78·4)	74·3 (68·6-79·5)	85·1 (78·2-90·8)	85·2 (77·4-91·1)	88·0 (73·7-97·4)
Belize	100·0 (100·0-100·0)	100·0 (100·0-100·0)	100·0 (100·0-100·0)	100·0 (100·0-100·0)	75·4 (71·9-78·4)	77·7 (74·4-80·7)	78·0 (74·8-80·9)	82·0 (79·2-84·8)	80·0 (75·0-84·6)	79·8 (74·9-84·7)	79·8 (74·8-84·7)	79·5 (74·4-84·7)	72·8 (67·4-77·9)	87·5 (81·6-92·9)	87·7 (80·9-93·4)	96·7 (89·8-100·0)
Cuba	100·0 (100·0-100·0)	100·0 (100·0-100·0)	100·0 (100·0-100·0)	100·0 (100·0-100·0)	88·9 (86·4-91·1)	92·3 (90·0-94·3)	92·5 (90·3-94·5)	95·1 (93·1-96·9)	73·6 (69·4-78·2)	74·0 (70·4-78·0)	74·0 (70·3-78·0)	73·3 (69·1-78·0)	92·9 (90·0-95·5)	97·9 (94·8-100·0)	98·1 (94·7-100·0)	99·6 (96·5-100·0)
Dominica	100·0 (100·0-100·0)	100·0 (100·0-100·0)	100·0 (100·0-100·0)	100·0 (100·0-100·0)	87·4 (84·9-89·6)	88·0 (85·6-90·2)	88·2 (85·8-90·3)	90·5 (88·3-92·4)	74·5 (70·9-78·3)	74·5 (71·0-78·2)	74·5 (70·9-78·2)	74·2 (70·5-78·1)	65·0 (60·3-69·8)	83·5 (77·8-89·1)	83·9 (77·4-89·9)	89·0 (77·5-97·5)
Dominican Republic	100·0 (100·0-100·0)	100·0 (100·0-100·0)	100·0 (100·0-100·0)	100·0 (100·0-100·0)	73·8 (70·7-77·0)	78·1 (75·6-80·7)	78·3 (75·8-81·0)	81·6 (78·5-84·4)	74·8 (71·3-78·3)	74·6 (71·2-78·2)	74·6 (71·1-78·2)	74·5 (71·0-78·1)	55·6 (50·6-60·4)	69·6 (60·1-78·1)	70·3 (60·1-79·5)	82·0 (66·0-94·2)
Grenada	100·0 (100·0-100·0)	100·0 (100·0-100·0)	100·0 (100·0-100·0)	100·0 (100·0-100·0)	83·7 (81·0-86·1)	88·2 (85·7-90·3)	88·4 (86·0-90·6)	91·9 (89·6-93·9)	74·8 (71·4-78·3)	74·2 (70·5-78·2)	74·1 (70·5-78·1)	73·5 (69·2-78·2)	69·1 (62·8-74·8)	86·5 (81·3-91·1)	86·9 (81·3-92·0)	95·1 (88·2-100·0)

Location	SDG Index: Geometric mean of all health-related SDG indicators (scale of 0 to 100)				MDG Index: Geometric mean of all health-related SDG indicators with corresponding MDG indicators (scale of 0 to 100)				Non-MDG Index: Geometric mean of all health-related indicators without corresponding MDG indicators (scale of 0 to 100)				Indicator 1.5.1: Death rate due to exposure to forces of nature (per 100,000 population)			
	**2000**	**2015**	**2016**	**2030**	**2000**	**2015**	**2016**	**2030**	**2000**	**2015**	**2016**	**2030**	**2000**	**2015**	**2016**	**2030**
Guyana	44·0 (41·9-45·9)	47·9 (45·9-51·0)	49·3 (46·8-52·6)	54·8 (50·0-59·5)	34·8 (32·7-37·1)	42·3 (40·1-44·5)	43·6 (41·3-45·8)	51·7 (47·1-55·3)	50·9 (48·2-53·3)	52·8 (50·1-56·8)	54·0 (51·0-58·6)	57·3 (50·7-63·5)	57·7 (48·7-75·2)	75·6 (67·8-89·1)	77·3 (69·7-90·2)	45·2 (36·2-63·9)
Haiti	21·2 (18·5-24·1)	28·8 (25·9-31·6)	29·4 (26·5-32·1)	36·2 (31·6-40·1)	12·0 (8·9-15·5)	27·1 (20·9-32·1)	27·8 (21·7-32·8)	39·2 (30·5-46·4)	30·6 (27·1-33·9)	32·2 (29·3-34·8)	32·7 (29·9-35·3)	37·0 (32·5-40·7)	16·9 (7·1-37·1)	0·0 (0·0-0·0)	0·0 (0·0-0·0)	0·0 (0·0-0·0)
Jamaica	62·0 (60·0-63·7)	64·7 (62·0-67·1)	65·1 (62·3-67·7)	67·8 (61·5-72·8)	53·4 (49·7-56·3)	59·0 (54·5-63·1)	59·3 (54·5-63·6)	62·0 (48·9-70·6)	68·1 (66·0-69·9)	68·4 (65·2-71·2)	68·8 (65·5-71·8)	70·5 (63·8-75·5)	79·9 (72·7-91·8)	57·8 (48·8-75·3)	61·3 (52·5-78·3)	57·3 (48·4-74·9)
Saint Lucia	60·3 (58·5-62·1)	64·6 (62·4-67·0)	65·2 (63·0-67·6)	69·5 (64·9-73·0)	55·8 (52·8-59·0)	63·2 (59·8-66·4)	63·8 (60·1-67·1)	69·9 (60·0-75·7)	64·8 (62·9-66·9)	67·9 (65·5-70·7)	68·4 (66·1-71·1)	71·6 (68·2-75·1)	54·2 (45·3-72·1)	27·2 (17·7-47·0)	30·2 (20·7-49·8)	31·8 (22·4-51·3)
Saint Vincent and the Grenadines	62·3 (60·7-64·0)	59·1 (55·7-62·4)	59·6 (56·2-62·8)	61·2 (57·1-65·9)	51·8 (49·1-54·9)	58·7 (52·9-62·1)	59·2 (53·3-62·7)	65·8 (58·8-70·5)	68·0 (64·5-70·7)	56·7 (48·1-63·3)	57·0 (48·6-63·7)	53·3 (47·8-64·4)	100·0 (100·0-100·0)	16·7 (6·9-37·0)	18·5 (8·8-38·7)	22·1 (12·5-42·2)
Suriname	52·0 (50·1-53·7)	57·1 (54·6-59·3)	57·7 (55·0-60·1)	60·1 (55·1-64·2)	43·3 (41·4-45·2)	53·7 (50·8-56·3)	54·5 (51·4-57·1)	62·6 (57·2-66·8)	59·6 (56·9-61·7)	61·6 (58·2-64·5)	62·2 (58·8-65·0)	61·6 (55·1-66·6)	65·1 (56·4-81·3)	100·0 (100·0-100·0)	100·0 (100·0-100·0)	81·3 (74·2-92·6)
Trinidad and Tobago	61·1 (59·9-62·4)	64·5 (62·5-66·4)	65·0 (62·9-66·9)	67·8 (61·8-72·1)	54·1 (52·1-56·2)	64·0 (59·7-67·4)	64·3 (59·6-67·8)	70·4 (58·9-77·9)	67·7 (66·2-69·1)	67·1 (65·0-69·2)	67·6 (65·4-69·8)	67·5 (58·5-72·5)	100·0 (100·0-100·0)	64·4 (55·7-80·8)	67·6 (59·1-83·3)	60·8 (52·0-77·8)
**Tropical Latin America**																
Brazil	54·0 (52·8-54·9)	62·0 (60·6-63·1)	62·5 (61·1-63·6)	66·0 (62·8-68·4)	52·8 (51·5-54·0)	61·2 (59·6-62·3)	61·6 (60·1-62·7)	68·0 (65·1-70·2)	55·8 (54·3-57·1)	62·0 (60·1-63·6)	62·5 (60·6-64·2)	60·4 (52·1-66·2)	52·0 (47·2-55·8)	47·7 (43·1-51·5)	50·5 (45·8-54·3)	50·1 (45·4-53·9)
Paraguay	50·4 (49·1-51·6)	57·4 (55·4-59·3)	58·0 (56·0-60·1)	59·9 (53·1-64·6)	51·3 (49·8-53·1)	63·2 (60·6-65·1)	63·8 (61·1-65·7)	71·3 (67·4-74·3)	52·7 (50·9-54·2)	56·8 (54·4-59·1)	57·4 (54·9-59·9)	55·5 (47·1-61·2)	36·2 (32·2-39·4)	44·2 (39·8-47·7)	47·2 (42·6-50·9)	43·6 (39·2-47·1)
**East Asia**																
China	45·7 (44·5-46·8)	59·5 (58·3-60·9)	60·6 (59·5-62·0)	68·9 (66·8-70·9)	55·0 (53·2-57·1)	74·6 (73·2-76·7)	75·4 (74·0-77·5)	87·2 (85·1-89·3)	43·9 (42·4-45·3)	55·0 (53·3-56·9)	56·1 (54·4-57·9)	62·0 (55·6-65·5)	44·4 (35·4-63·0)	32·9 (23·6-52·3)	37·3 (28·3-56·3)	33·4 (24·1-52·7)
North Korea	34·7 (32·1-36·9)	38·6 (36·5-40·6)	38·4 (36·2-40·4)	41·3 (37·8-44·2)	40·8 (34·6-46·2)	54·9 (51·6-58·5)	55·3 (51·9-59·0)	59·5 (52·7-65·1)	34·8 (32·5-36·8)	35·9 (33·5-38·0)	35·5 (33·1-37·8)	38·0 (34·7-40·9)	46·3 (37·3-64·9)	41·0 (31·9-59·8)	31·3 (21·9-50·9)	32·0 (22·6-51·5)
Taiwan (Province of China)	58·6 (55·6-61·5)	72·3 (70·3-74·2)	71·9 (69·6-74·0)	75·7 (71·1-79·4)	77·5 (74·8-79·4)	85·6 (83·2-87·5)	85·8 (83·4-87·7)	91·1 (87·0-93·9)	52·9 (49·2-56·3)	66·4 (63·1-69·1)	65·7 (62·3-68·6)	67·5 (54·6-73·5)	14·7 (4·9-35·0)	43·1 (34·0-61·8)	37·2 (28·1-56·3)	33·0 (23·7-52·5)
**Southeast Asia**																
Cambodia	16·4 (15·2-18·0)	34·8 (33·0-36·4)	35·6 (33·7-37·2)	43·9 (39·4-46·8)	13·0 (10·9-15·5)	42·0 (39·1-45·4)	43·3 (40·2-46·8)	58·9 (46·6-65·0)	19·8 (18·3-21·4)	34·4 (32·3-36·2)	35·0 (33·0-36·9)	41·5 (39·3-43·6)	22·0 (17·2-28·2)	33·5 (27·6-42·2)	35·6 (29·6-44·4)	35·9 (31·2-42·2)
Indonesia	33·8 (32·8-35·0)	39·7 (38·5-41·2)	40·2 (38·8-41·6)	41·6 (37·1-44·2)	33·4 (31·9-35·4)	44·1 (42·5-47·0)	45·1 (43·4-48·0)	52·8 (39·9-57·7)	36·6 (35·4-37·9)	40·3 (38·7-41·7)	40·5 (38·8-42·0)	39·1 (31·1-42·1)	68·4 (62·3-75·5)	52·3 (46·6-59·6)	54·5 (49·0-61·2)	9·5 (6·2-13·6)
Laos	13·5 (12·0-15·1)	29·5 (26·3-31·8)	30·3 (27·2-32·6)	39·3 (31·4-43·7)	8·7 (7·4-9·9)	30·8 (25·6-35·4)	32·0 (26·8-36·8)	47·0 (28·2-57·7)	19·5 (16·9-22·6)	31·8 (27·7-34·5)	32·5 (28·8-35·1)	38·9 (33·0-42·3)	44·0 (38·4-51·3)	47·3 (40·7-57·3)	49·8 (43·2-59·8)	51·2 (45·1-60·1)
Malaysia	56·0 (54·6-57·3)	64·7 (61·5-66·4)	65·5 (62·2-67·2)	68·9 (65·0-72·5)	60·9 (58·7-63·0)	71·1 (69·1-73·1)	71·8 (69·9-73·6)	78·5 (74·7-81·2)	54·2 (51·5-56·3)	60·0 (49·0-63·7)	60·6 (49·6-64·5)	59·2 (51·2-68·6)	44·1 (38·3-52·0)	81·9 (75·9-88·4)	90·3 (85·8-94·6)	64·4 (57·9-72·5)
Maldives	50·1 (47·9-52·3)	67·9 (65·6-70·2)	68·8 (66·3-71·2)	72·8 (68·3-76·6)	36·8 (32·1-41·0)	66·0 (62·6-69·3)	67·0 (63·4-70·4)	82·0 (78·1-85·9)	59·9 (57·6-62·2)	70·2 (67·6-72·7)	70·9 (68·1-73·4)	68·8 (55·1-74·5)	100·0 (100·0-100·0)	62·9 (57·7-66·9)	66·5 (61·2-70·6)	20·1 (15·3-26·2)
Mauritius	63·7 (62·3-65·1)	68·6 (66·4-70·7)	69·0 (66·5-71·2)	72·7 (67·7-77·3)	60·8 (57·2-64·0)	66·9 (63·2-70·1)	67·4 (63·6-70·5)	73·2 (69·2-77·2)	67·2 (64·3-69·1)	69·6 (64·4-73·0)	69·9 (64·3-73·4)	68·9 (56·8-78·5)	77·6 (71·3-84·8)	50·9 (44·7-59·0)	52·6 (46·5-60·8)	58·0 (51·6-66·3)
Myanmar	25·9 (23·3-28·1)	32·1 (30·7-33·5)	33·1 (31·7-34·4)	39·9 (35·8-42·4)	21·4 (15·5-25·1)	43·1 (39·6-46·5)	44·6 (41·1-48·2)	60·3 (48·0-67·5)	30·0 (26·8-32·3)	30·7 (29·2-32·0)	31·5 (30·0-32·9)	34·7 (27·5-37·5)	68·3 (61·8-76·3)	0·0 (0·0-0·0)	0·0 (0·0-0·0)	0·0 (0·0-0·0)
Philippines	41·1 (39·9-42·5)	47·3 (45·3-49·4)	47·9 (45·9-50·0)	53·4 (49·4-56·9)	40·2 (38·1-42·6)	46·6 (44·0-49·1)	47·2 (44·5-49·8)	52·7 (48·6-56·4)	41·5 (40·0-43·0)	50·4 (47·9-52·8)	51·1 (48·7-53·5)	56·0 (50·6-60·4)	22·5 (17·5-29·4)	12·2 (7·2-19·0)	14·8 (9·8-21·7)	16·1 (11·1-23·0)
Sri Lanka	46·1 (44·6-47·8)	61·6 (57·5-64·8)	62·3 (58·0-65·8)	59·8 (52·4-66·0)	45·9 (42·2-50·0)	66·8 (62·4-71·3)	68·0 (63·6-72·5)	82·5 (72·4-88·3)	47·3 (45·7-49·1)	60·5 (55·2-64·4)	60·9 (55·4-65·0)	52·0 (40·7-60·1)	29·0 (23·8-36·6)	32·0 (26·3-40·1)	32·2 (26·6-40·5)	0·3 (0·0-4·3)
Seychelles	61·4 (59·6-63·2)	67·2 (65·0-69·4)	67·6 (65·3-69·9)	71·3 (66·1-75·6)	62·7 (60·1-65·3)	65·6 (63·0-68·1)	66·3 (63·8-68·9)	69·1 (64·1-72·8)	62·0 (59·1-64·4)	67·9 (63·9-71·1)	68·0 (63·9-71·3)	69·9 (55·7-76·8)	100·0 (100·0-100·0)	100·0 (100·0-100·0)	100·0 (100·0-100·0)	100·0 (100·0-100·0)
Thailand	44·2 (41·0-46·4)	57·6 (55·9-59·3)	58·3 (56·4-60·1)	63·9 (59·7-67·1)	53·8 (51·9-56·1)	63·7 (61·4-66·4)	64·3 (61·9-67·2)	72·5 (68·5-76·5)	43·6 (39·6-46·4)	56·8 (54·7-58·7)	57·3 (54·9-59·5)	59·8 (47·7-65·0)	37·4 (31·3-46·6)	35·9 (29·9-44·8)	38·8 (32·7-47·8)	26·6 (21·0-35·0)
Timor-Leste	15·5 (13·7-18·4)	35·5 (32·8-38·8)	36·3 (33·3-39·6)	46·3 (37·5-51·6)	8·5 (6·4-11·7)	33·1 (27·4-38·5)	34·7 (28·9-40·1)	52·0 (31·1-61·2)	23·5 (21·3-26·5)	39·2 (36·0-42·3)	39·6 (36·2-42·9)	46·3 (40·5-50·8)	100·0 (100·0-100·0)	100·0 (100·0-100·0)	100·0 (100·0-100·0)	100·0 (100·0-100·0)
Vietnam	35·9 (34·3-37·5)	45·5 (43·4-47·3)	46·2 (44·2-48·1)	51·1 (47·2-54·4)	41·4 (38·9-44·4)	60·1 (57·2-63·7)	61·1 (58·3-64·7)	72·1 (64·1-77·1)	34·6 (32·9-36·3)	42·3 (40·0-44·6)	43·0 (40·7-45·3)	45·8 (40·5-49·5)	19·0 (14·6-24·7)	47·4 (41·0-56·9)	52·1 (45·6-61·8)	39·6 (34·6-46·2)
**Oceania**																
Federated States of Micronesia	36·1 (32·1-39·5)	40·0 (35·0-44·9)	40·6 (35·4-45·4)	40·2 (33·7-46·6)	44·9 (40·8-48·9)	54·0 (48·3-59·0)	54·8 (49·6-59·7)	60·2 (51·8-67·2)	34·1 (27·1-39·1)	35·2 (27·6-41·9)	35·7 (27·9-42·6)	33·0 (25·0-42·1)	100·0 (100·0-100·0)	100·0 (100·0-100·0)	100·0 (100·0-100·0)	20·7 (17·4-23·7)
Fiji	46·5 (43·6-49·2)	53·6 (48·5-59·1)	54·0 (48·6-59·7)	55·5 (45·0-65·0)	54·5 (51·7-57·0)	56·9 (52·5-60·9)	56·9 (52·2-60·9)	58·2 (45·3-66·5)	45·2 (35·8-49·2)	53·1 (41·9-60·6)	53·4 (41·7-61·2)	53·9 (39·0-66·9)	24·4 (21·0-27·4)	39·5 (35·4-43·2)	42·4 (38·2-46·3)	33·9 (30·1-37·4)

Location	Indicator 2.2.1: Prevalence of stunting in children under 5 (%)				Indicator 2.2.2a: Prevalence of wasting in children under 5 (%)				Indicator 2.2.2b: Prevalence of overweight in children aged 2-4 (%)				Indicator 3.1.1: Maternal mortality ratio (maternal deaths per 100,000 livebirths) in women aged 10-54 years			
	**2000**	**2015**	**2016**	**2030**	**2000**	**2015**	**2016**	**2030**	**2000**	**2015**	**2016**	**2030**	**2000**	**2015**	**2016**	**2030**
Guyana	75·3 (71·6-79·0)	77·3 (71·6-82·2)	78·7 (73·1-83·6)	80·9 (78·5-82·5)	42·3 (36·2-48·6)	68·8 (62·5-75·0)	69·2 (62·7-75·5)	82·0 (78·4-85·3)	83·2 (72·1-91·8)	75·3 (60·1-86·0)	74·3 (59·1-85·8)	61·2 (22·5-87·4)	35·2 (31·7-38·6)	31·2 (27·0-35·5)	34·0 (29·5-38·9)	50·4 (38·1-64·0)
Haiti	47·3 (39·9-54·4)	61·2 (52·7-69·0)	62·1 (53·6-69·9)	73·3 (65·7-79·3)	62·9 (57·7-67·7)	76·3 (70·9-81·0)	77·4 (72·0-82·0)	88·6 (84·5-92·3)	93·9 (88·6-97·9)	91·9 (84·7-97·3)	91·6 (83·9-97·5)	90·7 (74·3-100·0)	5·5 (1·0-10·0)	11·6 (3·9-19·5)	12·2 (4·1-20·5)	22·5 (6·3-38·6)
Jamaica	86·1 (83·1-88·6)	89·5 (85·3-92·8)	89·8 (85·5-93·2)	91·9 (87·2-95·7)	86·9 (84·1-89·7)	91·6 (87·9-94·7)	91·8 (88·1-94·8)	95·0 (91·0-98·1)	76·4 (59·7-88·8)	70·7 (53·4-83·9)	70·5 (53·3-83·7)	66·3 (32·1-89·9)	45·3 (40·6-50·3)	50·3 (42·8-57·1)	50·8 (42·8-58·2)	56·7 (37·6-75·5)
Saint Lucia	94·5 (91·3-96·9)	96·1 (93·2-98·1)	96·1 (93·2-98·1)	97·4 (95·2-99·0)	83·7 (77·3-88·8)	87·9 (83·3-91·7)	88·0 (83·5-91·8)	90·8 (87·1-93·8)	82·2 (69·3-91·7)	84·5 (73·7-92·5)	84·1 (72·5-92·8)	85·6 (65·1-99·7)	35·5 (32·5-38·3)	38·2 (34·9-41·4)	39·1 (35·7-42·4)	42·3 (33·4-51·2)
Saint Vincent and the Grenadines	80·8 (73·6-87·0)	83·9 (77·7-89·3)	84·1 (77·8-89·4)	86·6 (81·5-91·5)	86·3 (80·7-90·8)	89·2 (84·6-93·0)	89·3 (84·6-93·1)	90·8 (86·6-94·3)	41·9 (17·9-61·7)	13·6 (0·0-36·1)	12·7 (0·0-35·9)	4·2 (0·0-30·5)	53·0 (49·2-56·7)	50·9 (46·7-55·0)	51·3 (46·9-55·8)	67·9 (48·7-85·7)
Suriname	75·5 (71·1-79·3)	85·9 (80·4-90·2)	86·1 (80·3-90·5)	88·5 (84·2-94·3)	71·9 (65·6-78·1)	82·3 (76·6-87·4)	82·8 (77·0-87·7)	88·2 (82·1-92·5)	92·3 (83·1-98·5)	82·6 (69·7-92·4)	82·0 (68·9-91·7)	70·0 (35·0-91·1)	43·0 (39·1-47·0)	45·2 (40·2-50·4)	45·4 (40·4-50·9)	48·5 (36·4-60·7)
Trinidad and Tobago	97·2 (95·6-98·5)	99·1 (97·7-100·0)	99·1 (97·7-100·0)	99·8 (98·9-100·0)	77·8 (71·7-83·4)	82·0 (75·2-88·2)	82·2 (75·4-88·4)	84·3 (76·7-91·0)	82·7 (70·6-92·3)	67·8 (50·4-81·5)	67·4 (49·8-80·6)	47·0 (4·9-78·6)	43·7 (40·4-46·8)	43·7 (38·8-48·0)	43·6 (38·4-48·1)	42·9 (29·7-53·7)
**Tropical Latin America**																
Brazil	79·9 (78·3-81·3)	90·5 (89·6-91·4)	90·8 (89·9-91·6)	95·4 (94·8-96·0)	82·8 (80·7-84·9)	90·0 (88·2-91·5)	90·1 (88·4-91·6)	91·7 (90·0-93·3)	65·9 (59·0-72·3)	40·2 (30·0-49·2)	39·4 (28·5-48·8)	11·5 (0·0-30·4)	44·3 (42·8-45·8)	42·6 (40·9-44·2)	43·3 (41·5-45·0)	43·1 (40·0-46·3)
Paraguay	74·3 (66·5-80·9)	88·3 (83·6-91·8)	88·8 (84·3-92·2)	94·1 (91·4-96·3)	95·3 (90·4-98·4)	95·0 (89·5-98·4)	95·0 (89·6-98·5)	94·6 (88·7-99·1)	70·9 (64·3-76·5)	51·3 (42·8-59·3)	50·0 (40·9-58·0)	26·3 (8·1-42·1)	35·2 (32·1-38·1)	36·1 (30·7-40·8)	36·5 (30·6-41·6)	41·0 (27·0-54·1)
**East Asia**																
China	64·9 (62·8-67·1)	79·8 (78·4-81·2)	80·3 (78·9-81·7)	86·8 (85·6-87·9)	81·6 (79·5-83·5)	88·9 (87·4-90·2)	89·1 (87·6-90·5)	92·1 (90·8-93·2)	72·5 (57·0-84·4)	58·5 (38·6-74·8)	57·8 (37·7-74·7)	49·2 (8·6-81·2)	38·2 (36·4-40·1)	65·2 (63·1-67·2)	65·7 (63·5-67·9)	89·4 (84·1-94·1)
North Korea	8·1 (0·0-18·9)	44·3 (33·6-54·2)	44·7 (33·5-55·0)	50·0 (36·2-64·7)	37·5 (30·0-44·9)	71·6 (63·5-78·4)	71·9 (63·5-78·9)	87·0 (81·8-89·8)	100·0 (100·0-100·0)	100·0 (100·0-100·0)	100·0 (100·0-100·0)	100·0 (100·0-100·0)	39·9 (34·5-45·6)	39·9 (34·9-45·7)	40·4 (35·3-46·3)	47·7 (34·7-61·4)
Taiwan (Province of China)	90·6 (86·8-93·7)	93·1 (90·0-95·6)	93·2 (90·1-95·7)	94·7 (91·9-96·7)	91·1 (86·5-94·8)	93·4 (89·3-96·7)	93·5 (89·5-96·8)	95·0 (91·2-98·0)	53·1 (32·1-72·3)	45·8 (22·7-65·9)	44·9 (22·2-65·5)	36·9 (0·0-73·7)	68·2 (63·2-73·4)	73·0 (68·2-77·5)	73·1 (67·7-78·2)	78·4 (65·5-90·1)
**Southeast Asia**																
Cambodia	10·7 (3·0-17·9)	36·7 (18·6-50·6)	40·0 (21·0-54·5)	63·0 (42·2-76·8)	36·0 (29·5-43·7)	51·8 (42·5-60·1)	52·9 (42·9-61·7)	66·6 (52·5-75·1)	98·3 (94·1-100·0)	99·5 (95·9-100·0)	99·3 (94·8-100·0)	98·1 (86·6-100·0)	7·9 (4·7-11·2)	30·2 (24·8-35·6)	30·7 (24·9-36·4)	48·5 (35·2-61·5)
Indonesia	31·1 (26·9-34·5)	40·8 (37·6-43·8)	43·1 (39·7-46·1)	44·1 (42·0-46·3)	34·1 (29·3-38·6)	32·5 (28·1-36·9)	33·4 (28·9-37·8)	33·9 (30·2-37·5)	89·0 (79·4-95·9)	74·1 (51·1-90·9)	73·0 (48·8-90·7)	54·4 (0·0-91·4)	9·1 (7·4-11·0)	16·1 (14·0-18·2)	16·5 (14·3-18·6)	23·2 (19·2-27·1)
Laos	0·0 (0·0-0·0)	25·1 (2·7-45·0)	27·0 (3·6-47·9)	52·9 (18·9-77·7)	40·8 (30·3-51·2)	67·4 (55·2-78·2)	68·0 (55·4-78·6)	76·6 (68·5-85·9)	97·6 (88·1-100·0)	93·7 (79·9-100·0)	93·0 (78·9-100·0)	81·6 (39·5-100·0)	7·6 (4·5-10·9)	24·4 (18·7-30·3)	25·5 (19·5-31·7)	43·2 (30·8-56·7)
Malaysia	66·4 (60·0-72·6)	69·5 (64·2-74·7)	69·1 (65·0-73·2)	71·9 (69·4-75·2)	30·7 (20·2-40·5)	50·4 (41·4-58·4)	51·0 (42·5-58·3)	59·6 (54·1-65·3)	58·1 (25·3-82·5)	34·9 (0·0-67·5)	33·7 (0·0-65·7)	17·6 (0·0-68·3)	42·9 (39·4-46·6)	48·8 (44·1-53·5)	49·5 (44·8-54·3)	57·0 (46·6-67·7)
Maldives	28·4 (18·9-37·0)	64·0 (52·7-75·1)	64·4 (52·2-76·1)	82·8 (79·9-87·8)	6·3 (0·0-15·9)	49·9 (34·5-62·4)	50·4 (33·9-63·7)	71·5 (60·3-81·9)	93·5 (80·1-100·0)	79·2 (57·0-94·6)	77·9 (55·3-94·4)	56·4 (0·0-95·4)	25·9 (21·4-31·1)	49·1 (41·7-56·4)	48·6 (39·8-56·6)	65·6 (45·4-85·1)
Mauritius	78·7 (68·7-86·8)	83·1 (74·0-90·1)	83·4 (74·4-90·2)	86·8 (78·8-92·7)	29·2 (12·9-42·8)	42·4 (20·0-60·9)	42·9 (20·1-61·4)	52·8 (28·5-73·0)	61·2 (28·6-84·0)	45·1 (10·6-74·0)	44·4 (11·9-73·6)	27·2 (0·0-80·7)	53·4 (50·8-56·0)	47·1 (43·5-50·7)	47·8 (42·9-52·6)	59·5 (39·0-79·5)
Myanmar	19·0 (6·0-29·7)	47·0 (37·4-55·2)	49·0 (40·0-57·1)	69·1 (61·3-77·8)	42·5 (34·3-49·9)	65·6 (58·2-71·8)	66·2 (58·8-72·4)	80·1 (76·2-82·9)	96·4 (85·2-100·0)	79·0 (57·2-95·4)	77·7 (54·4-94·8)	49·2 (0·0-93·1)	10·0 (6·2-13·8)	18·8 (14·7-23·2)	20·0 (15·9-24·6)	29·3 (18·6-40·5)
Philippines	32·0 (19·2-42·7)	41·6 (21·6-57·4)	43·2 (21·8-59·8)	47·3 (23·9-65·0)	62·8 (55·2-69·8)	66·3 (55·3-75·8)	66·2 (54·6-76·1)	65·0 (50·4-79·7)	97·2 (88·0-100·0)	89·0 (71·1-99·8)	88·5 (70·2-99·8)	77·5 (29·9-100·0)	39·3 (36·7-41·8)	40·7 (36·2-45·4)	41·3 (36·3-46·5)	51·3 (37·4-66·4)
Sri Lanka	54·6 (51·2-58·1)	75·2 (65·5-84·0)	75·8 (65·8-84·8)	83·8 (73·7-93·4)	13·9 (7·7-20·0)	37·6 (18·9-54·0)	38·1 (18·2-55·4)	54·0 (30·0-73·3)	59·1 (44·5-72·3)	60·2 (29·0-83·4)	58·7 (28·3-82·6)	45·3 (0·0-90·1)	45·5 (42·1-49·1)	55·3 (48·6-62·4)	56·2 (48·9-64·0)	68·0 (49·2-88·0)
Seychelles	86·1 (78·6-91·8)	85·3 (78·3-90·7)	85·2 (77·7-90·9)	84·0 (71·1-91·9)	83·0 (75·6-88·4)	78·6 (72·2-83·6)	78·6 (72·1-83·7)	77·9 (69·8-83·0)	68·4 (38·3-88·9)	56·2 (23·8-81·1)	54·8 (21·7-79·1)	42·5 (0·0-87·8)	52·9 (48·0-57·6)	59·0 (52·8-64·4)	59·0 (51·9-65·1)	63·5 (46·0-80·5)
Thailand	73·2 (62·5-82·7)	76·6 (67·4-84·6)	77·1 (67·7-85·3)	83·7 (70·5-93·2)	72·4 (65·4-78·2)	65·2 (56·3-72·4)	64·5 (54·6-72·3)	53·4 (32·2-70·4)	83·2 (63·8-96·4)	67·1 (48·4-82·5)	65·8 (44·1-83·5)	44·8 (0·0-84·5)	41·9 (35·3-48·4)	59·2 (52·7-66·0)	59·7 (53·0-67·0)	72·5 (55·8-89·1)
Timor-Leste	0·1 (0·0-2·1)	12·7 (0·0-26·9)	15·0 (0·0-30·2)	31·9 (14·1-42·6)	14·3 (1·3-25·8)	20·9 (8·1-32·5)	23·8 (10·0-36·1)	39·2 (28·1-49·0)	84·5 (71·1-94·5)	84·8 (67·0-97·1)	84·3 (65·5-97·4)	76·3 (30·3-100·0)	0·6 (0·0-6·7)	17·4 (9·1-32·4)	17·3 (8·8-32·2)	29·1 (12·3-52·4)
Vietnam	21·8 (10·6-30·5)	62·5 (51·3-73·5)	64·7 (52·7-76·4)	86·8 (72·6-94·7)	47·0 (41·9-51·9)	72·3 (65·3-78·1)	73·1 (65·5-79·3)	82·8 (74·1-91·1)	99·1 (94·0-100·0)	87·8 (69·0-99·7)	87·2 (67·0-99·4)	67·0 (12·8-98·9)	55·1 (47·6-62·4)	67·6 (59·3-74·8)	68·1 (59·4-75·2)	80·2 (62·2-96·8)
**Oceania**																
Federated States of Micronesia	74·2 (65·3-82·2)	79·7 (72·3-86·4)	80·1 (72·8-86·7)	85·3 (78·8-90·7)	75·9 (64·7-84·6)	74·2 (62·9-83·3)	74·3 (62·9-83·3)	74·8 (62·7-83·5)	32·2 (0·0-76·2)	23·6 (0·0-67·5)	23·1 (0·0-67·5)	22·2 (0·0-85·5)	29·1 (22·1-36·9)	31·2 (22·2-40·7)	31·9 (22·9-42·7)	34·3 (18·4-52·6)
Fiji	89·2 (85·0-92·7)	90·4 (86·1-93·7)	90·6 (86·4-93·8)	93·2 (89·5-96·0)	65·2 (55·5-73·4)	71·6 (59·4-80·9)	71·7 (59·5-81·0)	74·1 (60·6-84·7)	49·6 (0·0-87·6)	43·7 (0·0-84·6)	43·1 (0·0-84·3)	38·9 (0·0-97·7)	29·7 (23·6-36·0)	31·0 (21·7-39·9)	30·7 (21·1-39·8)	30·5 (6·3-54·6)

Location	Indicator 3.1.2: Proportion of births attended by skilled health personnel (%)				Indicator 3.2.1: Under-5 mortality rate (probability of dying before the age of 5 per 1,000 livebirths)				Indicator 3.2.2: Neonatal mortality rate (probability of dying during the first 28 days of life per 1,000 livebirths)				Indicator 3.3.1: Age-standardised rate of new HIV infections (per 1,000 population)			
	2000	2015	2016	2030	2000	2015	2016	2030	2000	2015	2016	2030	2000	2015	2016	2030
Guyana	85·1 (80·7-88·9)	89·7 (83·5-93·6)	90·2 (83·4-94·5)	93·5 (77·5-99·2)	34·5 (32·1-36·8)	42·7 (39·0-45·5)	45·1 (41·2-48·3)	55·6 (45·9-64·8)	18·3 (16·1-20·6)	25·9 (21·0-30·4)	28·4 (23·1-33·2)	37·1 (25·4-48·1)	18·8 (12·0-25·1)	24·0 (14·6-30·2)	26·1 (19·8-32·2)	31·0 (23·9-38·8)
Haiti	19·3 (6·9-32·8)	74·1 (54·2-88·1)	74·4 (52·6-89·2)	82·2 (31·2-97·8)	12·8 (9·6-16·3)	25·8 (17·3-33·0)	26·7 (18·9-34·0)	42·8 (19·8-62·4)	10·1 (6·3-14·2)	19·5 (9·3-28·5)	20·7 (11·2-29·6)	38·6 (13·9-61·7)	10·2 (7·6-13·6)	50·2 (40·6-59·3)	44·9 (33·5-55·3)	51·0 (38·8-63·3)
Jamaica	97·6 (96·5-98·4)	98·4 (96·3-99·5)	98·5 (96·2-99·6)	98·7 (91·1-100·0)	45·4 (38·1-52·7)	50·2 (41·0-58·8)	50·5 (41·2-59·1)	58·9 (31·2-85·2)	33·7 (25·8-41·5)	38·1 (27·8-48·0)	38·4 (27·9-48·1)	47·0 (16·8-76·4)	39·7 (36·4-43·3)	41·4 (36·2-46·9)	43·4 (38·0-49·2)	43·6 (39·0-48·8)
Saint Lucia	98·3 (96·2-99·4)	98·4 (95·9-99·7)	98·4 (95·8-99·7)	98·3 (86·9-100·0)	52·9 (42·9-63·0)	53·4 (42·0-63·5)	54·2 (41·7-65·6)	65·8 (31·8-100·0)	36·8 (25·7-48·1)	34·5 (21·2-46·4)	35·4 (21·3-48·8)	45·3 (7·9-82·6)	56·4 (52·6-60·8)	60·9 (55·1-66·6)	61·2 (55·3-66·8)	60·9 (55·2-66·3)
Saint Vincent and the Grenadines	98·4 (96·6-99·4)	98·6 (96·8-99·6)	98·6 (96·7-99·7)	98·5 (89·2-100·0)	46·8 (39·6-54·3)	54·3 (43·8-63·6)	54·9 (43·4-64·7)	65·2 (42·5-88·0)	32·0 (23·5-40·5)	39·4 (28·8-48·8)	40·4 (28·6-50·4)	54·1 (29·8-79·3)	40·0 (36·5-43·5)	44·4 (38·1-49·5)	44·7 (38·7-50·1)	44·3 (38·6-49·5)
Suriname	89·8 (84·2-93·8)	95·3 (89·5-98·4)	95·6 (89·6-98·7)	97·9 (85·7-100·0)	31·1 (27·8-34·3)	36·9 (32·3-40·9)	37·3 (32·6-41·5)	43·4 (34·2-51·6)	17·3 (13·5-21·0)	22·3 (16·4-27·6)	22·7 (16·6-28·2)	28·1 (17·3-38·6)	32·0 (27·1-36·3)	36·2 (30·5-41·7)	36·7 (30·8-42·6)	42·4 (36·7-48·2)
Trinidad and Tobago	98·2 (96·5-99·3)	98·6 (96·5-99·7)	98·6 (96·5-99·7)	98·9 (93·0-100·0)	42·8 (38·3-47·3)	49·4 (39·8-58·1)	49·6 (39·4-59·0)	55·1 (31·5-76·0)	22·2 (17·3-27·4)	29·6 (17·9-40·4)	29·9 (17·5-41·4)	36·3 (8·8-61·5)	35·8 (31·0-40·0)	44·9 (39·0-51·2)	46·2 (40·2-52·7)	52·9 (47·3-58·9)
**Tropical Latin America**																
Brazil	96·2 (94·9-97·3)	98·9 (98·1-99·5)	99·0 (98·2-99·5)	99·7 (98·7-100·0)	39·4 (35·5-43·5)	53·4 (48·7-57·2)	54·1 (49·3-57·9)	67·8 (52·6-81·9)	31·9 (27·6-36·5)	44·5 (38·8-49·7)	45·4 (39·5-50·5)	59·2 (41·1-76·3)	49·3 (44·7-54·0)	49·0 (43·4-54·8)	49·0 (43·4-54·7)	51·5 (46·3-56·8)
Paraguay	71·1 (66·5-75·2)	91·1 (86·9-94·0)	91·4 (87·1-94·3)	96·3 (91·4-98·9)	41·6 (38·3-45·1)	50·7 (46·3-54·5)	51·5 (46·9-55·4)	62·6 (50·2-73·7)	30·2 (26·7-34·0)	39·0 (33·1-44·3)	40·0 (34·0-45·2)	50·7 (35·7-64·8)	58·2 (52·0-64·5)	60·4 (55·1-66·3)	61·2 (55·9-67·0)	60·3 (55·3-65·7)
**East Asia**																
China	81·7 (60·4-93·7)	96·1 (90·4-98·9)	96·3 (90·6-99·0)	99·1 (94·5-100·0)	37·1 (34·2-40·3)	61·0 (56·9-64·6)	62·4 (58·4-66·0)	82·2 (73·2-90·7)	29·0 (25·8-32·6)	53·9 (50·1-57·3)	55·6 (51·9-58·8)	79·6 (70·3-89·4)	69·6 (61·8-77·1)	70·3 (61·0-81·1)	70·3 (61·0-81·1)	73·2 (64·2-84·7)
North Korea	76·3 (49·3-91·6)	89·7 (74·6-96·8)	89·8 (75·1-96·9)	94·3 (68·4-100·0)	17·0 (4·0-32·9)	43·1 (37·0-48·6)	43·9 (37·6-49·4)	52·1 (38·7-64·7)	29·8 (24·2-35·5)	33·9 (28·0-39·6)	34·9 (28·6-40·4)	41·8 (27·5-55·6)	77·1 (45·8-98·5)	76·1 (45·8-99·9)	76·1 (45·7-99·7)	75·8 (45·5-99·8)
Taiwan (Province of China)	93·6 (84·2-98·2)	98·0 (94·7-99·5)	97·9 (94·4-99·5)	98·7 (90·9-100·0)	69·5 (66·3-72·6)	83·5 (79·4-87·0)	83·6 (79·2-87·4)	94·7 (87·1-100·0)	65·3 (62·0-68·5)	81·4 (77·7-84·4)	81·7 (78·0-84·7)	94·0 (85·9-100·0)	81·1 (73·7-87·5)	77·1 (67·9-87·3)	77·1 (67·8-87·3)	84·8 (76·0-93·8)
**Southeast Asia**																
Cambodia	16·4 (13·4-19·3)	91·8 (90·0-93·4)	92·9 (90·9-94·6)	99·8 (99·4-100·0)	14·0 (12·3-15·8)	38·0 (33·7-42·2)	39·3 (35·0-43·7)	61·3 (50·3-71·6)	7·4 (5·3-9·6)	28·2 (23·1-33·4)	29·5 (24·0-35·0)	48·5 (35·2-61·3)	35·3 (23·3-54·2)	63·8 (48·6-82·1)	66·1 (50·5-85·5)	70·7 (54·5-90·5)
Indonesia	46·9 (43·6-50·1)	79·4 (73·5-84·4)	79·8 (72·7-85·6)	93·0 (84·1-97·9)	27·0 (25·1-28·6)	43·6 (40·4-45·9)	44·7 (41·3-47·4)	61·3 (52·9-69·5)	18·1 (16·4-19·8)	32·3 (28·2-35·9)	33·5 (29·0-37·3)	47·4 (36·8-56·9)	73·1 (66·8-79·4)	57·8 (50·6-65·1)	59·2 (51·9-66·4)	58·8 (51·7-66·0)
Laos	6·9 (0·9-13·1)	41·6 (26·4-57·3)	44·5 (28·3-60·6)	82·2 (53·3-98·5)	4·8 (1·9-7·6)	24·3 (16·8-31·4)	25·6 (17·9-33·2)	45·9 (27·7-64·0)	0·1 (0·0-0·9)	15·2 (7·1-23·5)	16·5 (7·7-25·2)	36·1 (15·6-56·6)	60·4 (27·6-85·6)	64·7 (34·8-88·7)	65·3 (35·4-89·0)	64·8 (34·5-89·2)
Malaysia	94·8 (92·6-96·5)	98·7 (97·6-99·5)	98·9 (97·8-99·5)	99·7 (98·6-100·0)	67·7 (64·6-70·7)	76·6 (74·9-78·2)	78·0 (76·5-79·2)	89·4 (83·0-96·2)	63·6 (60·6-66·3)	73·0 (71·5-74·5)	74·6 (73·3-75·7)	87·3 (79·9-95·3)	52·8 (49·4-57·0)	63·2 (58·4-68·4)	62·9 (58·1-68·1)	65·4 (60·3-70·7)
Maldives	71·6 (59·8-80·8)	98·3 (97·0-99·2)	98·3 (97·0-99·2)	99·9 (99·3-100·0)	39·6 (37·7-41·5)	71·0 (67·2-74·5)	72·8 (68·8-76·4)	97·7 (90·4-100·0)	22·4 (20·8-24·1)	60·0 (56·0-63·7)	62·1 (57·8-65·9)	93·6 (83·8-100·0)	92·6 (89·0-96·0)	93·1 (89·2-96·3)	93·1 (89·2-96·3)	99·4 (97·3-100·0)
Mauritius	98·4 (97·2-99·2)	99·2 (98·1-99·7)	99·2 (98·3-99·7)	99·6 (98·2-100·0)	52·4 (50·1-54·7)	57·8 (56·7-58·9)	58·4 (57·3-59·5)	63·7 (58·7-67·9)	36·8 (34·7-39·0)	44·3 (42·5-46·1)	45·0 (43·1-46·7)	51·6 (45·5-56·7)	57·4 (50·7-63·9)	60·8 (53·2-68·0)	62·1 (53·8-69·8)	62·4 (54·3-69·9)
Myanmar	60·9 (54·0-68·0)	78·2 (67·1-86·3)	79·4 (68·2-87·7)	91·4 (74·2-99·3)	17·0 (12·4-21·9)	40·8 (34·0-47·1)	42·7 (35·6-48·8)	69·6 (53·3-86·0)	8·3 (3·2-13·6)	29·0 (21·3-35·8)	30·9 (23·2-37·8)	54·7 (37·7-71·9)	29·4 (26·7-32·1)	48·4 (43·9-53·2)	48·3 (43·8-53·1)	47·3 (42·8-52·1)
Philippines	50·8 (47·7-53·8)	65·0 (51·4-76·3)	66·0 (51·9-77·7)	79·7 (53·5-96·5)	34·7 (32·9-36·5)	44·8 (42·2-46·6)	45·7 (43·1-47·6)	55·8 (49·5-61·6)	28·5 (26·7-30·3)	36·5 (33·3-39·2)	37·6 (34·1-40·4)	46·4 (38·7-53·7)	59·5 (48·6-67·3)	45·7 (38·3-54·1)	44·3 (36·9-52·8)	43·9 (36·8-52·3)
Sri Lanka	99·3 (98·8-99·7)	100·0 (99·9-100·0)	100·0 (99·9-100·0)	100·0 (100·0-100·0)	53·9 (51·4-56·4)	73·2 (69·4-76·3)	75·2 (71·0-78·4)	98·6 (86·3-100·0)	42·2 (40·0-44·6)	63·5 (58·5-67·7)	65·9 (60·5-70·2)	97·0 (80·0-100·0)	88·6 (79·5-93·2)	83·4 (75·6-90·0)	82·5 (74·4-89·5)	82·3 (74·0-89·5)
Seychelles	91·4 (83·9-95·9)	96·8 (93·6-98·6)	96·9 (93·8-98·7)	98·4 (95·0-99·9)	59·4 (56·2-62·6)	61·7 (59·2-63·3)	63·2 (60·3-65·1)	65·7 (58·2-73·5)	43·5 (40·6-46·6)	46·6 (43·2-49·1)	48·3 (44·5-51·2)	50·4 (41·6-58·8)	59·0 (54·7-63·8)	49·7 (43·6-55·8)	50·9 (44·8-56·9)	51·2 (45·3-57·0)
Thailand	94·9 (92·4-97·0)	99·0 (98·4-99·5)	99·1 (98·4-99·5)	99·8 (99·0-100·0)	55·8 (51·7-59·1)	75·2 (72·5-77·0)	76·5 (73·5-78·5)	95·3 (86·7-100·0)	46·4 (42·9-49·6)	69·6 (65·5-72·6)	71·2 (66·9-74·3)	92·6 (81·9-100·0)	45·8 (35·5-61·8)	41·1 (33·1-48·0)	42·3 (34·5-49·2)	50·4 (43·2-57·5)
Timor-Leste	0·0 (0·0-0·0)	34·9 (19·7-50·9)	35·4 (19·4-52·0)	65·8 (29·8-90·0)	12·8 (8·8-16·9)	36·5 (25·5-46·9)	37·6 (26·6-48·5)	60·9 (33·2-88·1)	11·3 (5·3-17·4)	30·5 (15·7-45·3)	31·5 (16·8-46·7)	53·1 (16·9-88·6)	54·4 (21·6-79·3)	57·5 (27·6-82·1)	58·7 (29·0-83·4)	57·3 (27·6-81·9)
Vietnam	82·7 (78·8-86·2)	94·0 (90·9-96·2)	94·4 (91·2-96·6)	98·2 (94·2-99·9)	42·5 (39·9-45·0)	58·7 (54·0-63·0)	59·9 (54·9-64·2)	75·5 (66·7-83·2)	32·1 (29·8-34·4)	49·7 (45·1-53·7)	51·1 (46·3-55·5)	69·8 (60·3-78·4)	46·3 (40·0-52·4)	46·9 (40·9-53·8)	48·6 (42·6-55·4)	50·4 (44·7-56·7)
**Oceania**																
Federated States of Micronesia	89·7 (83·2-94·2)	97·5 (95·0-99·0)	97·7 (95·3-99·1)	99·1 (96·0-100·0)	37·4 (28·3-45·6)	52·6 (44·3-59·6)	53·8 (45·8-61·0)	63·3 (42·2-82·8)	31·9 (25·0-39·0)	43·4 (31·4-53·6)	44·7 (32·9-54·9)	54·7 (31·0-76·8)	73·1 (41·6-98·3)	39·6 (6·8-66·1)	40·1 (9·4-67·2)	29·1 (0·0-59·9)
Fiji	98·6 (97·1-99·4)	98·8 (97·6-99·6)	98·9 (97·6-99·6)	99·2 (96·1-100·0)	40·3 (35·1-45·5)	35·8 (26·1-44·6)	35·9 (25·5-45·5)	36·5 (11·0-61·3)	32·4 (28·5-36·7)	27·7 (17·2-37·8)	27·9 (16·4-38·5)	30·7 (2·3-58·8)	69·8 (64·9-75·7)	63·2 (55·4-70·7)	61·7 (53·5-69·4)	60·5 (52·5-68·3)

Location	Indicator 3.3.2: Age-standardised rate of tuberculosis cases (per 100,000 population)				Indicator 3.3.3: Age-standardised rate of malaria cases (per 1,000 population)				Indicator 3.3.4: Age-standardised rate of hepatitis B incidence (per 100,000 population)				Indicator 3.3.5: Age-standardised prevalence* of the sum of 15 neglected tropical diseases (NTDs) (%) *Prevalence estimates reported here may exceed 100% as they reflect the sum of prevalent cases of 15 NTDs.*			
	**2000**	**2015**	**2016**	**2030**	**2000**	**2015**	**2016**	**2030**	**2000**	**2015**	**2016**	**2030**	**2000**	**2015**	**2016**	**2030**
Guyana	48·8 (47·3-50·3)	48·7 (47·3-50·2)	49·3 (47·8-50·8)	55·0 (52·7-57·6)	12·2 (10·1-14·6)	16·6 (14·3-18·8)	16·2 (14·2-18·1)	21·3 (19·4-24·3)	44·6 (41·3-47·9)	53·2 (50·0-56·4)	53·6 (50·6-56·8)	61·0 (57·4-64·6)	72·0 (66·3-76·4)	76·3 (71·5-80·1)	76·5 (71·7-80·3)	79·3 (74·7-83·0)
Haiti	34·1 (32·4-35·7)	42·4 (40·7-44·2)	43·1 (41·3-44·9)	50·6 (48·7-52·7)	25·9 (22·7-29·3)	31·8 (27·8-35·7)	33·9 (29·9-37·8)	38·3 (25·3-43·1)	39·8 (37·6-41·9)	49·4 (47·1-51·6)	50·0 (47·6-52·2)	58·8 (55·9-61·6)	52·6 (47·8-56·8)	88·0 (86·0-89·7)	89·3 (87·4-90·8)	97·8 (97·3-98·2)
Jamaica	77·5 (75·9-79·0)	86·7 (84·8-88·5)	86·8 (84·9-88·6)	88·9 (86·8-91·0)	100·0 (100·0-100·0)	100·0 (100·0-100·0)	100·0 (100·0-100·0)	100·0 (100·0-100·0)	76·1 (73·4-81·1)	78·0 (75·3-82·5)	78·1 (75·4-82·6)	79·5 (76·5-84·3)	70·6 (67·9-73·2)	66·8 (63·0-70·1)	66·9 (63·1-70·2)	68·2 (64·5-71·4)
Saint Lucia	59·6 (57·9-61·3)	66·6 (65·0-68·2)	67·0 (65·5-68·7)	73·7 (72·1-75·4)	100·0 (100·0-100·0)	100·0 (100·0-100·0)	100·0 (100·0-100·0)	100·0 (100·0-100·0)	75·9 (73·1-78·5)	84·8 (82·5-87·2)	85·2 (82·9-87·5)	90·5 (88·2-92·5)	54·0 (49·9-58·0)	57·7 (50·0-64·3)	57·7 (50·0-64·3)	57·9 (50·0-64·5)
Saint Vincent and the Grenadines	58·6 (56·9-60·3)	64·8 (63·3-66·4)	65·0 (63·5-66·6)	68·1 (66·5-69·8)	100·0 (100·0-100·0)	100·0 (100·0-100·0)	100·0 (100·0-100·0)	100·0 (100·0-100·0)	81·7 (79·6-83·6)	90·0 (88·2-91·7)	90·4 (88·6-92·1)	96·2 (94·3-98·3)	79·6 (75·5-83·2)	74·2 (69·3-78·6)	74·2 (69·3-78·6)	74·3 (69·4-78·7)
Suriname	59·2 (57·7-60·7)	64·9 (63·3-66·5)	65·6 (64·0-67·2)	73·7 (71·6-75·8)	16·1 (12·9-21·9)	43·0 (37·5-48·4)	46·0 (39·9-51·3)	66·7 (60·4-76·2)	60·8 (58·3-62·7)	71·1 (68·8-72·9)	71·7 (69·4-73·5)	79·9 (77·6-81·8)	80·0 (77·7-82·1)	80·5 (78·0-82·7)	80·5 (78·0-82·7)	80·9 (78·5-83·1)
Trinidad and Tobago	72·4 (70·5-74·0)	77·8 (76·1-79·4)	78·1 (76·4-79·7)	82·7 (80·9-84·6)	100·0 (100·0-100·0)	100·0 (100·0-100·0)	100·0 (100·0-100·0)	100·0 (100·0-100·0)	71·1 (68·2-73·8)	75·0 (72·1-77·7)	75·1 (72·3-77·9)	77·8 (74·8-80·5)	96·2 (95·6-96·7)	96·2 (95·6-96·7)	96·2 (95·6-96·7)	96·0 (95·4-96·5)
**Tropical Latin America**																
Brazil	57·4 (55·7-59·2)	62·6 (60·7-64·6)	63·0 (61·1-65·0)	68·0 (66·0-70·0)	23·8 (21·6-25·9)	38·8 (35·5-41·6)	39·4 (35·8-42·2)	48·0 (42·6-51·5)	47·1 (45·5-48·8)	55·1 (53·8-56·5)	55·6 (54·3-57·0)	62·7 (61·3-64·0)	71·2 (63·7-77·3)	73·1 (68·4-77·0)	73·4 (68·7-77·3)	77·6 (73·2-81·5)
Paraguay	56·8 (55·3-58·4)	58·1 (56·5-59·7)	58·3 (56·7-59·9)	61·1 (59·3-62·9)	100·0 (100·0-100·0)	100·0 (100·0-100·0)	100·0 (100·0-100·0)	100·0 (100·0-100·0)	69·9 (67·5-72·4)	67·4 (65·3-69·7)	67·6 (65·6-70·0)	71·3 (69·4-73·4)	78·3 (75·1-81·1)	78·4 (75·0-81·2)	78·5 (75·0-81·2)	78·9 (75·5-81·7)
**East Asia**																
China	39·0 (36·8-41·4)	47·7 (45·4-50·2)	47·7 (45·4-50·2)	55·2 (52·8-57·8)	65·2 (61·1-68·0)	98·4 (92·5-100·0)	98·9 (94·4-100·0)	99·9 (99·6-100·0)	33·2 (29·5-37·0)	56·1 (52·7-59·4)	56·6 (53·2-59·9)	69·2 (65·4-72·7)	68·1 (60·0-74·1)	81·6 (78·4-84·0)	82·1 (79·0-84·3)	87·7 (86·3-89·0)
North Korea	25·4 (23·8-26·9)	24·8 (23·3-26·5)	24·4 (22·8-26·0)	18·4 (16·7-20·3)	35·7 (33·3-37·4)	55·8 (52·1-58·2)	58·5 (54·7-61·0)	69·1 (64·9-72·0)	37·2 (35·1-39·2)	40·0 (37·9-42·1)	40·3 (38·2-42·4)	43·5 (41·2-45·8)	76·6 (70·1-81·9)	87·7 (83·5-91·0)	88·4 (84·3-91·5)	94·4 (92·5-95·9)
Taiwan (Province of China)	61·2 (58·8-63·7)	70·4 (68·1-72·8)	70·6 (68·3-73·0)	79·4 (77·1-81·9)	100·0 (100·0-100·0)	100·0 (100·0-100·0)	100·0 (100·0-100·0)	100·0 (100·0-100·0)	47·1 (44·5-49·8)	51·2 (48·5-54·0)	51·2 (48·4-54·0)	56·2 (53·0-59·6)	64·8 (61·8-67·4)	76·4 (73·4-79·3)	76·6 (73·6-79·4)	79·1 (76·4-81·7)
**Southeast Asia**																
Cambodia	11·3 (9·6-13·1)	24·8 (23·1-26·9)	25·9 (24·2-28·0)	40·5 (38·0-43·1)	17·6 (12·6-20·6)	36·1 (33·3-38·4)	36·9 (34·0-39·4)	49·3 (45·5-53·1)	0·5 (0·0-2·7)	12·9 (8·9-16·5)	13·7 (9·7-17·4)	25·3 (20·1-29·8)	23·9 (17·3-30·0)	95·1 (94·6-95·5)	95·9 (95·5-96·2)	99·7 (99·7-99·8)
Indonesia	13·8 (12·4-15·6)	20·2 (18·7-21·9)	20·5 (19·0-22·2)	25·4 (23·8-27·1)	30·2 (27·8-32·7)	33·6 (30·0-37·2)	34·7 (30·7-38·6)	40·2 (37·1-43·3)	16·3 (13·2-19·1)	29·8 (26·7-32·6)	30·6 (27·5-33·5)	42·6 (38·5-46·0)	65·3 (61·1-69·4)	74·8 (71·6-77·8)	75·1 (71·9-78·0)	79·5 (76·8-82·1)
Laos	21·8 (20·4-23·2)	33·5 (32·0-34·9)	34·4 (32·9-35·8)	46·8 (45·1-48·7)	19·5 (15·2-23·0)	27·5 (24·3-32·9)	26·6 (23·2-32·5)	36·3 (32·5-39·3)	28·2 (23·7-32·3)	41·0 (36·8-44·7)	42·1 (37·8-45·7)	55·2 (50·8-59·0)	25·7 (15·1-34·6)	55·6 (48·0-62·1)	57·9 (50·8-64·1)	80·3 (76·8-83·5)
Malaysia	46·0 (43·6-48·2)	52·7 (50·4-54·8)	53·3 (51·0-55·4)	60·6 (58·3-62·8)	41·4 (37·0-46·5)	77·2 (72·5-80·2)	81·6 (76·9-84·5)	96·6 (93·3-98·6)	51·6 (46·9-56·1)	52·0 (47·2-56·6)	52·2 (47·5-56·8)	55·4 (51·1-59·6)	30·0 (22·3-37·4)	33·2 (24·6-41·2)	33·6 (25·0-41·6)	38·8 (30·5-46·8)
Maldives	43·6 (41·0-46·0)	55·5 (52·9-57·8)	55·7 (53·2-58·1)	66·3 (63·6-68·8)	100·0 (100·0-100·0)	100·0 (100·0-100·0)	100·0 (100·0-100·0)	100·0 (100·0-100·0)	50·6 (48·6-52·6)	64·2 (62·2-66·1)	64·6 (62·7-66·6)	77·2 (75·0-79·1)	99·7 (99·6-99·8)	99·7 (99·6-99·7)	99·5 (99·4-99·6)	99·5 (99·4-99·6)
Mauritius	70·7 (68·8-72·7)	72·8 (70·7-75·2)	72·9 (70·7-75·2)	73·8 (71·5-76·2)	100·0 (100·0-100·0)	100·0 (100·0-100·0)	100·0 (100·0-100·0)	100·0 (100·0-100·0)	73·1 (71·2-75·1)	58·2 (55·4-60·6)	58·5 (55·8-60·9)	62·5 (60·1-64·6)	95·1 (93·4-96·5)	94·3 (92·1-96·0)	94·2 (92·0-96·0)	93·2 (90·5-95·3)
Myanmar	17·9 (15·5-19·9)	29·4 (27·3-31·5)	30·3 (28·2-32·4)	41·0 (38·7-43·4)	24·9 (22·4-27·5)	34·7 (31·8-37·7)	37·2 (34·1-40·5)	37·0 (34·1-39·4)	29·7 (27·0-32·2)	42·5 (39·7-45·1)	43·2 (40·5-45·8)	54·1 (51·1-57·0)	14·7 (2·0-25·3)	64·9 (57·4-71·1)	68·9 (62·3-74·4)	94·3 (93·0-95·3)
Philippines	23·4 (21·8-25·0)	26·8 (25·1-28·6)	26·1 (24·4-28·0)	19·4 (17·4-21·5)	46·2 (41·3-82·0)	65·4 (63·0-68·2)	67·6 (63·4-70·4)	88·8 (67·1-93·9)	28·9 (26·9-30·9)	43·3 (40·8-45·8)	43·6 (41·1-46·1)	47·4 (44·5-50·1)	0·1 (0·0-1·7)	45·9 (38·2-52·4)	48·9 (41·5-55·0)	76·9 (73·5-79·5)
Sri Lanka	49·4 (47·8-51·0)	59·9 (58·2-61·5)	60·5 (58·7-62·1)	68·7 (66·9-70·5)	43·6 (17·6-77·2)	100·0 (100·0-100·0)	100·0 (100·0-100·0)	100·0 (100·0-100·0)	73·0 (70·8-75·0)	82·7 (80·7-84·4)	82·9 (81·0-84·7)	87·0 (85·1-88·8)	78·7 (75·4-81·6)	85·1 (82·2-87·3)	85·2 (82·4-87·5)	87·5 (85·0-89·7)
Seychelles	58·3 (56·0-60·6)	64·2 (61·8-66·6)	64·4 (61·9-66·8)	67·6 (64·7-70·4)	100·0 (100·0-100·0)	100·0 (100·0-100·0)	100·0 (100·0-100·0)	100·0 (100·0-100·0)	42·3 (39·2-45·4)	49·7 (46·9-52·3)	49·6 (46·8-52·3)	50·4 (47·3-53·4)	39·1 (27·2-49·2)	39·1 (27·2-49·3)	39·1 (27·2-49·3)	39·6 (27·8-49·6)
Thailand	33·8 (31·7-36·1)	47·3 (45·0-49·5)	47·8 (45·4-50·0)	55·0 (52·5-57·2)	37·4 (33·6-41·0)	60·6 (56·7-63·2)	63·5 (59·5-66·1)	89·1 (83·7-93·0)	55·8 (53·0-58·5)	62·8 (60·1-65·3)	62·9 (60·3-65·4)	65·2 (62·6-67·8)	71·0 (67·0-74·8)	75·7 (71·8-79·3)	75·7 (71·8-79·3)	76·2 (72·6-79·7)
Timor-Leste	32·0 (29·6-34·3)	41·6 (39·1-43·9)	41·8 (39·3-44·1)	46·3 (43·7-48·6)	16·8 (13·9-19·9)	54·7 (50·1-58·6)	63·4 (58·3-67·6)	75·0 (69·8-78·8)	40·1 (38·1-41·9)	51·9 (49·5-53·7)	52·3 (49·9-54·2)	60·1 (57·1-62·5)	59·3 (52·8-64·9)	64·2 (57·8-69·6)	64·5 (58·0-69·9)	68·6 (62·1-73·7)
Vietnam	29·9 (28·3-31·7)	37·5 (35·7-39·3)	38·1 (36·3-39·9)	46·3 (43·9-48·4)	40·8 (33·6-87·5)	56·3 (53·9-61·1)	56·3 (52·8-58·7)	67·0 (58·2-73·8)	42·9 (39·8-45·8)	51·9 (48·7-54·9)	52·4 (49·2-55·4)	60·6 (56·9-63·9)	17·4 (11·0-23·7)	64·1 (58·4-69·9)	68·2 (63·1-73·3)	94·1 (93·2-95·1)
**Oceania**																
Federated States of Micronesia	32·5 (30·7-34·5)	36·9 (34·8-39·0)	36·6 (34·4-38·7)	41·6 (39·2-44·0)	100·0 (100·0-100·0)	100·0 (100·0-100·0)	100·0 (100·0-100·0)	100·0 (100·0-100·0)	41·8 (39·0-44·5)	44·2 (41·2-47·1)	44·4 (41·4-47·3)	46·8 (43·5-50·0)	52·1 (43·6-59·6)	52·9 (41·7-62·1)	52·6 (41·4-61·8)	50·9 (38·7-60·3)
Fiji	51·2 (49·2-53·3)	53·7 (51·7-55·9)	53·7 (51·7-55·9)	54·3 (52·2-56·5)	100·0 (100·0-100·0)	100·0 (100·0-100·0)	100·0 (100·0-100·0)	100·0 (100·0-100·0)	42·9 (40·2-45·6)	44·8 (42·5-47·0)	44·7 (42·4-46·8)	45·5 (42·8-47·9)	44·6 (36·4-51·6)	46·1 (36·3-53·8)	46·3 (36·5-54·0)	50·5 (40·9-58·1)

Location	Indicator 3.4.1: Age-standardised death rate due to cardiovascular disease, cancer, diabetes, and chronic respiratory disease in populations aged 30-70 (per 100,000 population)				Indicator 3.4.2: Age-standardised death rate due to self-harm (per 100,000 population)				Indicator 3.5.2: Risk-weighted prevalence of alcohol consumption, as measured by the summary exposure value (SEV) for alcohol use (%)				Indicator 3.6.1: Age-standardised death rate due to road injuries (per 100,000 population)			
	**2000**	**2015**	**2016**	**2030**	**2000**	**2015**	**2016**	**2030**	**2000**	**2015**	**2016**	**2030**	**2000**	**2015**	**2016**	**2030**
Guyana	17·3 (13·6-21·6)	25·5 (20·4-31·4)	28·4 (22·3-35·0)	36·1 (23·3-49·4)	9·3 (3·6-14·1)	1·1 (0·0-8·2)	2·0 (0·0-9·9)	7·5 (0·0-21·7)	43·8 (36·2-51·7)	54·7 (44·6-65·2)	54·7 (44·2-65·7)	55·0 (38·7-71·5)	32·6 (28·8-36·5)	28·6 (22·8-34·4)	30·6 (24·5-37·1)	43·3 (29·8-56·5)
Haiti	13·0 (6·3-20·1)	21·4 (9·7-33·5)	21·6 (10·1-32·9)	29·1 (8·5-50·3)	45·8 (34·0-59·6)	47·1 (33·3-60·9)	47·0 (33·4-60·6)	48·9 (27·1-69·5)	64·6 (58·6-71·0)	60·1 (48·1-71·4)	59·8 (47·6-71·3)	55·0 (39·5-69·7)	14·9 (7·3-21·5)	21·5 (11·1-30·4)	21·8 (11·1-30·7)	27·3 (9·6-43·5)
Jamaica	49·6 (45·0-54·4)	53·7 (45·9-62·4)	53·7 (45·3-63·6)	54·1 (32·1-80·3)	99·9 (98·4-100·0)	95·4 (86·3-100·0)	95·3 (85·8-100·0)	91·0 (68·2-100·0)	67·0 (59·8-74·6)	58·7 (46·4-71·5)	58·3 (45·7-71·2)	52·0 (36·9-67·9)	76·5 (70·7-81·6)	62·4 (54·7-69·7)	62·7 (54·9-70·9)	67·4 (46·1-89·6)
Saint Lucia	47·7 (44·7-50·6)	58·3 (54·4-62·2)	58·7 (54·8-62·7)	64·2 (56·3-72·7)	63·5 (57·3-71·1)	61·4 (55·0-68·6)	60·4 (50·5-70·1)	59·6 (43·6-75·7)	34·8 (27·2-42·1)	48·5 (34·1-62·1)	49·1 (34·5-62·7)	57·1 (41·4-71·6)	42·9 (38·9-47·0)	46·8 (41·9-51·6)	45·6 (39·5-51·8)	50·1 (39·0-61·2)
Saint Vincent and the Grenadines	35·9 (31·8-39·6)	39·3 (34·5-44·0)	39·7 (34·8-44·4)	42·6 (31·7-52·4)	61·3 (55·7-69·8)	57·9 (50·9-64·8)	57·2 (50·0-64·9)	54·6 (39·7-69·7)	68·4 (61·1-75·5)	60·7 (47·3-73·7)	60·5 (46·8-73·7)	57·0 (39·2-73·3)	57·6 (53·9-61·2)	51·3 (46·3-56·3)	52·9 (47·7-58·3)	49·7 (38·2-61·5)
Suriname	44·5 (41·7-47·3)	48·1 (44·1-52·5)	48·8 (44·3-53·4)	56·2 (46·3-66·2)	15·5 (9·5-21·5)	8·2 (2·3-16·5)	9·6 (3·1-17·7)	10·1 (0·0-24·5)	59·3 (49·9-68·0)	45·6 (30·7-59·8)	45·3 (30·2-59·7)	41·1 (24·0-57·5)	34·7 (31·6-37·8)	31·9 (27·7-36·6)	32·0 (27·4-37·1)	32·4 (22·0-44·8)
Trinidad and Tobago	28·2 (25·1-31·2)	45·9 (41·3-50·6)	46·1 (41·3-51·0)	49·5 (38·7-60·6)	32·9 (28·0-42·8)	38·6 (31·6-46·0)	38·7 (31·5-46·3)	40·8 (27·3-54·5)	78·1 (73·5-82·7)	71·9 (62·2-80·2)	71·4 (61·4-80·0)	64·2 (49·8-76·6)	39·5 (36·1-42·7)	40·5 (34·9-45·5)	40·9 (35·4-46·3)	47·6 (37·7-58·9)
**Tropical Latin America**																
Brazil	47·4 (45·4-49·5)	63·5 (61·2-66·0)	63·8 (61·2-66·3)	77·6 (72·8-81·9)	62·1 (55·0-66·0)	64·1 (56·6-69·2)	64·3 (56·9-70·0)	67·1 (56·5-77·9)	47·5 (39·9-54·6)	48·3 (37·3-58·6)	48·2 (37·0-58·6)	46·7 (33·0-59·1)	23·4 (20·1-26·5)	29·1 (24·5-32·5)	29·5 (25·0-33·2)	34·6 (27·6-40·3)
Paraguay	58·5 (55·6-61·6)	56·6 (51·5-61·3)	57·0 (51·5-62·6)	58·5 (45·8-72·6)	73·1 (67·5-80·6)	62·8 (55·3-75·6)	63·2 (54·8-76·0)	56·4 (39·1-76·4)	32·2 (20·7-45·2)	43·6 (26·2-59·9)	43·3 (25·5-59·9)	38·6 (15·6-61·3)	36·0 (32·7-39·6)	23·1 (18·2-28·1)	24·0 (18·5-29·5)	16·5 (2·0-30·8)
**East Asia**																
China	32·8 (30·5-34·9)	54·8 (52·8-57·0)	56·7 (54·5-58·8)	77·5 (73·3-81·7)	22·6 (18·0-29·1)	51·4 (46·8-54·5)	52·2 (47·6-55·4)	77·7 (68·2-83·1)	69·9 (65·6-74·3)	65·0 (61·1-68·8)	64·7 (60·6-68·5)	59·3 (53·9-64·3)	22·7 (20·5-25·1)	32·7 (30·5-35·6)	33·6 (31·4-36·5)	46·2 (42·0-50·9)
North Korea	31·0 (26·1-36·5)	30·1 (25·8-34·8)	30·4 (26·1-34·9)	32·9 (24·4-42·4)	34·8 (24·7-43·2)	37·8 (25·4-49·6)	38·2 (25·7-49·8)	41·1 (24·2-57·1)	76·1 (71·2-81·1)	80·4 (72·0-87·1)	80·3 (71·6-87·1)	78·3 (65·0-87·5)	25·7 (19·3-33·0)	22·0 (15·1-30·6)	22·1 (15·3-30·7)	23·9 (10·8-39·5)
Taiwan (Province of China)	57·6 (54·3-60·7)	73·7 (68·4-79·5)	74·0 (66·5-82·4)	86·6 (71·0-100·0)	32·5 (26·7-37·8)	33·5 (27·1-40·6)	33·5 (26·0-41·1)	39·7 (23·0-56·8)	68·4 (53·1-82·9)	66·2 (50·1-81·3)	65·9 (49·7-81·1)	62·3 (45·2-79·0)	29·3 (26·1-32·4)	46·2 (41·5-50·7)	46·0 (40·2-52·0)	58·2 (46·4-71·4)
**Southeast Asia**																
Cambodia	29·1 (24·6-33·5)	42·8 (37·8-47·8)	43·4 (38·2-48·5)	54·0 (43·9-63·9)	56·6 (50·0-63·6)	62·3 (56·4-68·7)	62·6 (56·9-69·3)	66·6 (55·1-79·8)	59·2 (49·6-67·4)	56·3 (46·1-65·6)	56·4 (46·3-65·8)	58·5 (48·9-67·7)	16·8 (11·4-21·5)	22·1 (16·7-27·7)	22·4 (17·1-28·3)	27·4 (18·0-38·4)
Indonesia	39·7 (35·8-43·0)	42·4 (39·1-45·5)	43·2 (39·8-46·5)	49·0 (42·2-56·0)	83·0 (76·7-87·5)	87·8 (81·6-91·3)	88·0 (82·1-91·5)	91·9 (83·8-98·2)	97·9 (97·6-98·2)	97·5 (96·8-98·2)	97·4 (96·7-98·1)	96·8 (95·7-97·7)	20·3 (16·9-24·2)	37·0 (33·2-40·6)	37·4 (33·5-41·1)	46·2 (40·1-51·9)
Laos	19·3 (14·7-26·3)	32·2 (28·4-37·5)	33·1 (29·0-38·5)	45·5 (38·7-53·8)	31·3 (21·0-48·7)	43·6 (32·3-58·5)	43·9 (32·5-58·6)	52·7 (37·9-72·4)	70·6 (63·5-77·3)	69·9 (56·2-80·7)	69·6 (55·3-80·8)	65·5 (41·6-82·3)	18·7 (12·8-24·3)	25·5 (19·7-31·9)	26·0 (20·3-32·4)	34·1 (24·3-45·0)
Malaysia	47·3 (43·3-50·2)	57·5 (53·0-61·3)	58·4 (53·7-62·5)	68·0 (60·1-75·6)	47·4 (41·4-52·1)	52·5 (45·1-58·5)	52·9 (45·5-58·9)	56·7 (44·4-68·7)	85·9 (83·1-88·5)	86·1 (83·1-89·1)	86·0 (82·9-89·1)	85·2 (82·0-88·4)	21·3 (17·9-25·1)	29·0 (24·6-34·2)	29·9 (25·3-35·1)	38·1 (28·7-47·8)
Maldives	44·9 (40·2-49·6)	77·5 (67·2-87·1)	77·8 (65·9-88·6)	90·6 (63·2-100·0)	64·9 (55·4-75·1)	84·4 (73·1-95·1)	84·6 (72·7-96·3)	92·0 (70·6-100·0)	90·9 (87·0-94·4)	91·5 (84·5-96·6)	91·7 (84·6-96·8)	93·8 (85·8-98·6)	43·2 (37·9-48·4)	63·8 (55·0-72·2)	64·4 (54·8-73·6)	78·2 (57·1-100·0)
Mauritius	29·7 (26·8-32·6)	48·7 (43·3-54·3)	49·1 (41·7-56·2)	63·1 (48·9-79·9)	40·3 (36·3-48·5)	53·3 (46·5-60·1)	53·5 (45·0-61·9)	61·3 (43·2-82·4)	66·8 (58·2-75·1)	71·7 (58·6-82·7)	71·7 (58·0-83·0)	72·0 (52·0-86·5)	45·4 (42·4-48·5)	56·8 (52·5-61·5)	57·6 (51·7-63·7)	68·9 (53·9-85·0)
Myanmar	24·5 (16·6-30·5)	41·5 (34·0-46·7)	42·5 (36·2-47·5)	58·2 (49·1-66·5)	53·4 (41·1-64·9)	66·6 (57·1-73·4)	67·3 (58·4-73·9)	78·4 (66·4-90·1)	91·3 (87·3-94·8)	87·3 (80·8-93·1)	86·9 (80·2-92·9)	80·0 (69·4-90·1)	11·5 (4·3-17·4)	21·5 (15·2-26·8)	22·3 (16·3-27·3)	33·2 (24·3-42·4)
Philippines	37·3 (33·7-41·0)	37·7 (30·7-44·8)	38·4 (30·8-46·2)	44·6 (26·0-64·3)	63·9 (46·8-70·3)	64·5 (45·9-73·7)	64·8 (45·8-74·3)	68·7 (45·6-90·8)	66·1 (59·8-72·8)	69·9 (57·9-80·0)	70·2 (57·8-80·4)	73·2 (56·9-85·6)	51·6 (48·2-54·7)	55·2 (49·6-61·1)	55·9 (49·9-62·3)	65·8 (51·0-80·9)
Sri Lanka	51·9 (47·8-56·3)	68·5 (58·1-79·8)	69·5 (58·3-81·3)	84·3 (60·5-100·0)	0·0 (0·0-0·0)	11·0 (0·1-21·0)	11·3 (0·0-21·9)	20·2 (0·0-46·0)	90·7 (87·0-93·7)	72·9 (62·7-81·8)	71·9 (61·5-81·1)	55·0 (39·9-70·3)	37·3 (33·6-41·2)	46·2 (37·9-55·1)	46·9 (37·7-56·7)	57·0 (36·4-78·9)
Seychelles	35·8 (32·0-39·6)	50·2 (43·6-56·5)	50·6 (42·4-57·9)	63·2 (44·2-80·8)	41·0 (33·8-46·9)	53·5 (43·1-60·5)	53·4 (42·6-61·2)	66·3 (46·4-83·2)	62·9 (56·7-68·7)	67·8 (57·5-78·6)	68·5 (58·1-79·3)	76·4 (66·2-86·8)	36·7 (32·5-41·6)	47·2 (41·7-52·9)	47·5 (41·0-54·2)	54·1 (39·3-68·7)
Thailand	54·4 (50·6-57·9)	72·9 (67·0-78·2)	73·4 (66·8-79·7)	83·9 (68·2-100·0)	26·9 (20·4-33·7)	42·3 (35·9-48·8)	42·5 (35·8-49·6)	49·7 (34·2-66·0)	59·3 (53·8-64·9)	58·6 (48·4-68·0)	58·6 (48·2-68·2)	59·2 (46·7-70·1)	4·8 (0·7-9·1)	24·2 (19·6-29·3)	24·5 (19·1-30·2)	39·7 (26·8-52·2)
Timor-Leste	44·6 (35·2-58·5)	56·9 (44·8-73·0)	56·5 (43·9-72·5)	63·7 (39·8-90·9)	46·1 (35·3-64·2)	58·6 (44·1-80·8)	58·0 (43·6-79·5)	65·4 (40·4-97·8)	98·6 (97·8-99·3)	98·3 (96·8-99·3)	98·3 (96·8-99·3)	98·0 (95·9-99·3)	46·8 (37·3-58·1)	53·0 (42·2-67·0)	53·1 (42·2-66·7)	59·0 (41·6-78·8)
Vietnam	40·6 (34·6-47·5)	50·7 (43·3-58·3)	51·4 (44·2-58·4)	62·1 (48·1-77·7)	43·6 (35·3-51·9)	53·5 (46·1-60·9)	53·9 (46·6-61·5)	62·4 (46·6-77·8)	67·3 (55·0-78·7)	34·3 (19·9-51·1)	34·2 (19·6-51·1)	31·8 (15·5-51·4)	20·5 (14·9-27·0)	24·3 (17·3-31·5)	25·0 (18·2-32·0)	34·3 (20·5-49·8)
**Oceania**																
Federated States of Micronesia	9·1 (0·0-19·0)	5·8 (0·0-19·1)	6·7 (0·0-20·6)	7·8 (0·0-29·3)	17·8 (8·0-29·5)	18·9 (6·3-34·1)	19·5 (6·8-35·3)	22·5 (1·0-46·4)	87·2 (83·5-90·8)	90·2 (84·6-94·2)	90·2 (84·4-94·3)	90·7 (82·5-95·6)	33·9 (26·0-42·0)	33·8 (22·8-45·2)	34·6 (23·6-46·5)	35·9 (18·1-54·3)
Fiji	0·6 (0·0-4·9)	5·6 (0·0-18·7)	5·8 (0·0-19·4)	13·0 (0·0-44·3)	32·7 (24·8-40·1)	37·2 (25·8-49·4)	36·8 (24·9-50·3)	36·0 (3·5-68·9)	81·4 (77·2-86·0)	74·3 (65·5-82·5)	73·9 (65·0-82·4)	68·6 (56·5-80·1)	42·4 (36·7-48·1)	51·0 (42·1-60·3)	50·5 (41·4-60·8)	48·9 (23·4-73·5)

Location	Indicator 3.7.1: Proportion of women of reproductive age (15-49 years) who have their need for family planning satisfied with modern contraception methods (%)				Indicator 3.7.2: Number of livebirths per 1,000 women aged 10-14 years and women aged 15-19 years				Indicator 3.8.1: Coverage of essential health services, as defined by the UHC index comprised of the coverage of 9 tracer interventions and risk-standardised death rates from 32 causes amenable to personal healthcare (scale of 0 to 100)				Indicator 3.9.1: Age-standardised death rate attributable to household air pollution and ambient air pollution (per 100,000 population)			
	**2000**	**2015**	**2016**	**2030**	**2000**	**2015**	**2016**	**2030**	**2000**	**2015**	**2016**	**2030**	**2000**	**2015**	**2016**	**2030**
Guyana	61·7 (52·5-70·9)	50·6 (45·8-55·5)	52·0 (46·3-57·7)	45·0 (31·4-59·4)	11·3 (7·4-15·5)	15·1 (10·4-20·2)	14·7 (9·9-19·9)	15·4 (3·7-27·2)	27·4 (24·0-30·4)	37·8 (33·8-41·9)	39·5 (35·6-43·6)	56·1 (50·3-62·3)	35·9 (31·5-40·3)	43·9 (38·5-50·0)	44·5 (39·0-50·6)	52·6 (46·7-59·1)
Haiti	26·5 (22·6-30·4)	44·6 (38·1-50·9)	45·2 (37·8-52·2)	65·0 (52·1-78·3)	23·2 (18·7-29·0)	29·3 (22·5-37·8)	29·5 (22·5-38·0)	36·1 (19·6-54·3)	0·9 (0·0-4·6)	19·9 (13·9-26·4)	21·1 (15·1-27·7)	40·4 (33·2-48·0)	2·9 (0·0-6·6)	10·9 (6·0-16·3)	11·3 (6·3-16·8)	17·9 (12·2-24·4)
Jamaica	79·9 (75·5-84·1)	84·9 (76·8-91·4)	85·2 (77·3-91·9)	90·3 (75·5-100·0)	10·8 (3·8-17·6)	18·2 (10·0-26·0)	18·9 (10·4-26·7)	26·7 (8·9-44·6)	49·2 (45·2-53·2)	58·6 (53·1-64·6)	58·6 (53·0-64·8)	64·8 (57·3-73·1)	54·8 (51·5-58·2)	65·6 (60·4-71·2)	65·8 (60·5-71·5)	76·8 (68·4-88·5)
Saint Lucia	76·5 (68·1-84·2)	85·9 (78·7-92·0)	86·2 (79·0-92·3)	91·2 (78·0-100·0)	23·1 (20·4-25·8)	34·7 (27·6-41·4)	35·2 (27·9-41·7)	47·6 (32·6-62·2)	47·5 (44·4-50·3)	54·8 (50·9-58·6)	55·5 (51·6-59·4)	61·2 (55·8-66·5)	60·0 (54·4-65·9)	74·7 (68·1-82·1)	74·6 (68·0-82·0)	85·2 (72·5-98·2)
Saint Vincent and the Grenadines	71·3 (62·4-80·0)	81·5 (73·1-88·8)	81·9 (73·7-89·0)	87·1 (72·1-98·5)	18·6 (14·9-22·6)	25·5 (21·4-30·0)	26·4 (21·7-31·6)	36·6 (24·9-48·0)	42·3 (39·2-45·2)	46·9 (43·2-50·5)	47·5 (43·7-51·1)	50·3 (45·4-55·3)	41·8 (36·2-48·1)	57·9 (50·6-66·4)	57·8 (50·5-66·3)	68·7 (54·6-86·3)
Suriname	66·7 (59·0-74·7)	71·3 (63·9-78·0)	72·5 (64·7-79·8)	76·7 (62·4-88·0)	23·1 (18·2-28·3)	31·2 (25·3-36·7)	31·4 (25·5-37·2)	39·4 (25·2-52·9)	36·2 (33·0-39·3)	48·0 (44·0-51·9)	49·1 (45·2-53·0)	62·0 (56·9-67·1)	39·0 (33·5-45·1)	51·3 (44·6-58·5)	51·3 (44·7-58·6)	54·7 (47·2-62·7)
Trinidad and Tobago	61·2 (53·0-69·1)	75·3 (66·5-83·4)	75·6 (66·3-84·0)	82·7 (66·7-94·5)	31·3 (26·8-36·4)	50·2 (41·3-58·7)	51·9 (42·7-60·6)	76·9 (53·6-99·6)	40·5 (37·5-43·2)	53·2 (48·0-57·7)	53·8 (48·5-58·5)	64·6 (56·8-71·5)	51·9 (48·1-55·6)	66·6 (62·1-71·0)	66·0 (61·6-70·3)	70·3 (58·1-79·1)
**Tropical Latin America**																
Brazil	88·7 (87·2-90·2)	95·4 (93·8-96·9)	95·7 (94·2-97·2)	99·8 (98·4-100·0)	15·5 (14·4-16·6)	19·6 (18·5-20·7)	19·7 (18·6-20·9)	23·0 (21·4-24·8)	48·1 (46·5-49·7)	59·8 (57·9-61·6)	60·2 (58·3-61·9)	69·4 (67·1-71·5)	44·7 (42·6-46·8)	67·1 (64·8-69·8)	67·7 (65·4-70·4)	82·6 (75·3-89·4)
Paraguay	77·1 (72·2-81·7)	94·1 (89·4-98·3)	94·3 (89·5-98·6)	99·7 (96·7-100·0)	15·1 (13·6-16·9)	26·4 (24·5-28·6)	27·0 (25·0-29·2)	37·3 (32·3-42·1)	43·7 (40·9-46·4)	48·6 (44·7-52·7)	49·2 (45·2-53·4)	57·2 (50·6-63·2)	36·1 (33·4-38·7)	44·9 (40·0-49·6)	45·7 (40·5-50·4)	55·8 (49·0-62·5)
**East Asia**																
China	95·8 (90·8-100·0)	99·8 (97·7-100·0)	99·9 (98·2-100·0)	100·0 (99·8-100·0)	76·7 (72·8-81·0)	85·4 (79·3-91·5)	86·8 (80·0-93·2)	98·5 (87·6-100·0)	44·1 (41·8-46·3)	72·2 (70·2-74·2)	73·4 (71·3-75·3)	91·5 (88·6-94·2)	8·8 (6·4-11·2)	29·0 (26·6-31·4)	30·5 (28·1-32·9)	50·6 (47·6-53·6)
North Korea	77·9 (69·6-85·6)	78·4 (70·0-85·7)	78·7 (70·5-86·0)	79·2 (61·3-92·4)	100·0 (100·0-100·0)	100·0 (100·0-100·0)	100·0 (100·0-100·0)	100·0 (100·0-100·0)	42·2 (38·0-46·2)	49·8 (45·3-54·0)	50·1 (45·6-54·3)	54·6 (48·8-59·9)	6·0 (2·0-10·1)	7·5 (3·8-11·3)	7·8 (4·0-11·5)	10·3 (6·2-14·5)
Taiwan (Province of China)	98·7 (94·9-100·0)	99·8 (97·7-100·0)	99·8 (97·9-100·0)	99·8 (97·2-100·0)	56·0 (53·8-58·1)	80·8 (78·9-82·6)	81·8 (80·2-83·4)	95·8 (81·4-100·0)	64·5 (62·3-66·8)	78·9 (75·0-83·0)	79·2 (75·1-83·4)	87·3 (80·8-93·4)	56·4 (52·2-60·1)	66·7 (62·3-71·5)	67·3 (62·8-72·2)	77·2 (71·3-83·3)
**Southeast Asia**																
Cambodia	25·7 (23·2-28·4)	58·3 (55·3-61·4)	59·1 (55·0-63·2)	82·4 (73·6-89·8)	28·6 (26·6-30·8)	24·7 (22·5-27·1)	24·4 (22·2-27·1)	19·8 (12·8-27·3)	1·7 (0·0-4·5)	37·3 (34·2-40·4)	38·8 (35·7-41·9)	65·8 (60·4-70·2)	4·3 (1·9-6·8)	16·4 (13·9-19·1)	17·1 (14·6-19·9)	28·0 (25·0-31·2)
Indonesia	82·8 (81·2-84·4)	85·1 (83·8-86·3)	85·7 (84·3-87·0)	89·9 (86·8-92·9)	25·6 (22·9-28·3)	30·8 (26·8-35·0)	31·2 (27·1-35·7)	37·2 (28·1-46·2)	22·9 (20·8-24·9)	38·0 (35·4-40·3)	39·2 (36·6-41·6)	55·8 (51·4-60·0)	29·0 (26·7-30·9)	39·3 (36·6-41·8)	40·9 (38·1-43·5)	51·9 (44·0-61·4)
Laos	45·6 (36·4-54·7)	63·7 (53·4-73·2)	64·5 (54·3-74·0)	78·9 (62·0-92·3)	15·2 (11·2-20·2)	12·7 (6·8-20·0)	12·9 (6·9-19·8)	13·3 (0·0-28·6)	0·0 (0·0-0·0)	24·9 (19·6-30·1)	26·9 (21·6-32·2)	58·4 (51·3-65·3)	1·1 (0·0-3·6)	11·8 (8·8-14·8)	12·6 (9·6-15·6)	23·6 (20·3-26·9)
Malaysia	66·9 (57·5-76·0)	83·3 (74·2-90·6)	83·8 (75·0-90·8)	94·7 (82·4-100·0)	59·3 (57·0-61·4)	59·6 (57·0-62·1)	59·1 (56·3-61·9)	57·8 (51·0-64·4)	49·5 (47·4-51·5)	63·4 (60·8-66·2)	64·8 (62·1-67·5)	77·9 (74·5-81·2)	45·1 (40·7-49·0)	51·6 (47·7-55·3)	51·9 (48·1-55·6)	56·4 (53·0-59·9)
Maldives	57·0 (47·4-66·0)	77·4 (69·0-85·0)	78·1 (70·0-85·6)	92·0 (80·6-100·0)	46·4 (44·1-48·5)	87·2 (82·5-91·9)	92·2 (87·4-97·0)	100·0 (100·0-100·0)	51·7 (48·4-55·4)	78·5 (73·2-83·5)	79·3 (73·6-84·6)	91·6 (82·3-99·7)	23·4 (19·2-27·7)	55·4 (48·0-63·3)	56·4 (48·7-64·4)	76·2 (62·8-92·5)
Mauritius	70·4 (61·1-79·4)	81·5 (73·0-88·5)	81·7 (73·5-88·7)	89·4 (76·1-100·0)	35·4 (33·8-37·0)	42·6 (41·3-43·8)	42·7 (40·1-44·7)	48·7 (43·5-53·8)	51·9 (49·7-53·9)	64·9 (60·9-68·9)	65·7 (61·4-69·8)	76·1 (69·6-82·8)	40·5 (36·1-44·6)	66·5 (61·3-71·9)	67·1 (61·8-72·5)	83·0 (74·6-92·3)
Myanmar	53·3 (44·1-62·5)	82·2 (79·5-84·8)	83·0 (79·7-85·9)	98·8 (94·1-100·0)	45·7 (41·0-50·8)	57·1 (51·4-62·5)	57·6 (51·9-63·0)	66·4 (55·4-77·7)	11·1 (6·4-14·8)	36·6 (32·2-40·5)	38·5 (34·2-42·4)	64·8 (60·6-68·8)	9·4 (4·9-12·9)	22·5 (18·7-26·7)	23·5 (19·6-27·6)	37·0 (33·1-41·7)
Philippines	42·1 (38·7-45·2)	53·1 (48·8-57·5)	54·1 (48·3-59·7)	69·3 (55·6-81·5)	29·3 (27·7-31·0)	21·2 (19·9-22·7)	20·4 (19·1-21·9)	13·1 (9·3-16·8)	28·4 (25·7-30·9)	36·7 (32·5-41·0)	38·1 (33·6-42·5)	49·8 (42·3-57·6)	16·9 (14·0-20·0)	16·8 (12·2-21·6)	17·4 (12·6-22·5)	24·0 (14·8-32·6)
Sri Lanka	70·3 (61·5-78·4)	83·3 (75·3-90·2)	83·9 (76·2-90·7)	94·5 (83·0-100·0)	40·7 (39·0-42·3)	64·2 (62·2-65·7)	66·9 (64·5-68·8)	88·7 (82·9-95·8)	52·4 (49·7-54·9)	71·0 (65·0-76·9)	72·2 (65·9-78·5)	88·7 (78·4-97·6)	29·7 (26·2-32·9)	44·3 (37·3-51·9)	45·8 (38·4-53·8)	57·6 (42·8-73·8)
Seychelles	81·2 (72·9-88·0)	86·5 (79·2-92·8)	86·9 (79·6-93·2)	91·9 (79·1-100·0)	25·4 (24·1-26·7)	24·1 (22·8-25·5)	25·4 (23·7-27·3)	23·9 (21·4-26·3)	44·8 (42·1-47·6)	54·9 (50·5-59·0)	55·5 (51·0-59·6)	63·6 (56·0-69·7)	52·5 (41·8-66·3)	66·1 (52·4-83·0)	66·6 (52·7-83·7)	74·5 (58·1-95·2)
Thailand	94·1 (89·1-98·6)	95·3 (92·0-98·0)	95·6 (91·9-98·9)	98·6 (91·3-100·0)	27·6 (22·4-33·6)	35·1 (29·2-41·3)	35·6 (29·0-42·8)	44·1 (29·4-59·4)	51·1 (48·1-53·9)	70·7 (67·0-74·6)	71·5 (67·7-75·5)	83·1 (78·2-88·2)	40·1 (36·7-43·4)	58·6 (52·6-64·6)	59·9 (53·7-66·0)	77·1 (68·9-85·2)
Timor-Leste	24·9 (16·5-34·2)	46·3 (37·6-55·2)	47·4 (38·4-56·9)	70·4 (49·9-88·5)	34·9 (29·8-40·7)	38·9 (33·7-45·2)	40·0 (34·6-46·5)	48·6 (35·0-65·1)	2·8 (0·0-10·4)	29·9 (21·8-39·6)	30·7 (22·7-40·2)	48·0 (37·7-58·4)	13·5 (9·5-17·9)	26·1 (20·7-32·4)	26·7 (21·2-33·2)	35·3 (28·8-43·3)
Vietnam	73·1 (69·6-76·2)	77·0 (72·5-81·2)	78·0 (72·7-83·0)	87·3 (75·8-97·0)	42·2 (39·1-45·2)	34·6 (30·0-39·2)	34·6 (30·2-39·1)	32·7 (22·5-42·3)	37·0 (33·0-41·2)	57·4 (53·1-61·8)	58·7 (54·3-63·1)	75·6 (70·4-80·6)	22·1 (18·5-26·1)	38·0 (33·3-42·9)	39·2 (34·4-44·3)	56·4 (49·9-62·5)
**Oceania**																
Federated States of Micronesia	62·9 (52·6-72·7)	77·7 (68·7-85·8)	77·9 (69·1-85·9)	87·7 (71·5-98·9)	24·5 (22·2-27·0)	47·1 (41·0-52·9)	48·2 (41·8-54·0)	64·5 (50·9-77·9)	21·6 (15·4-27·3)	28·8 (21·1-37·3)	29·6 (21·8-38·2)	37·2 (27·0-48·8)	21·9 (14·1-30·2)	33·8 (23·4-45·8)	34·4 (23·9-46·6)	44·0 (31·2-59·0)
Fiji	78·7 (70·1-86·3)	89·3 (82·3-94·9)	89·6 (82·6-95·0)	95·8 (84·5-100·0)	40·9 (39·2-42·5)	44·4 (42·7-46·1)	44·3 (42·6-46·0)	46·1 (44·3-47·8)	26·8 (22·4-31·0)	32·0 (25·8-39·4)	32·1 (25·7-39·8)	36·0 (26·4-47·8)	31·2 (24·2-38·8)	50·7 (40·6-62·2)	51·6 (41·3-63·3)	64·7 (51·2-79·9)

Location	Indicator 3.9.2: Age-standardised death rate attributable to unsafe water, sanitation, and hygiene (WaSH) (per 100,000 population)				Indicator 3.9.3: Age-standardised death rate due to unintentional poisonings (per 100,000 population)				Indicator 3.a.1: Age-standardised prevalence of daily smoking in populations aged 10 and older (%)				Indicator 3.b.1: Geometric mean of the coverage of eight vaccines, conditional on inclusion in national vaccine schedules, in target populations (%)			
	**2000**	**2015**	**2016**	**2030**	**2000**	**2015**	**2016**	**2030**	**2000**	**2015**	**2016**	**2030**	**2000**	**2015**	**2016**	**2030**
Guyana	28·7 (25·6-31·9)	37·0 (33·4-40·6)	37·6 (33·9-41·2)	46·2 (41·4-50·7)	38·3 (29·4-42·5)	48·9 (38·8-54·3)	50·5 (40·3-56·1)	64·3 (49·2-78·1)	69·8 (64·8-74·2)	73·0 (68·0-77·8)	73·0 (68·1-77·8)	75·5 (67·2-83·2)	84·0 (80·6-87·1)	92·5 (89·3-94·7)	93·3 (90·1-95·5)	98·4 (95·4-99·9)
Haiti	14·9 (11·8-18·4)	19·3 (15·3-23·5)	20·5 (16·4-24·8)	31·8 (21·6-40·4)	12·0 (0·0-25·0)	25·7 (12·8-37·9)	26·7 (14·3-38·5)	41·8 (22·6-60·4)	84·7 (81·3-87·8)	94·1 (92·1-96·0)	94·1 (92·0-96·1)	98·3 (94·2-100·0)	54·2 (48·4-59·4)	83·9 (78·4-88·2)	85·5 (80·0-89·8)	96·8 (93·1-99·1)
Jamaica	49·6 (45·7-53·3)	54·6 (48·8-59·5)	54·7 (48·9-59·8)	57·9 (51·4-64·1)	64·2 (59·0-68·5)	69·4 (63·4-75·7)	69·7 (63·5-76·1)	74·0 (61·0-87·4)	69·9 (66·0-73·7)	77·2 (72·9-81·3)	77·5 (73·1-81·6)	80·2 (71·6-87·2)	87·4 (84·4-90·1)	96·0 (93·7-97·6)	96·3 (94·0-97·8)	98·9 (96·6-99·9)
Saint Lucia	53·3 (50·2-56·3)	61·1 (57·8-64·1)	61·3 (57·9-64·4)	68·8 (63·6-74·5)	73·4 (64·8-77·1)	83·6 (73·6-88·0)	84·0 (74·2-88·7)	91·9 (80·0-100·0)	79·6 (75·0-83·9)	78·5 (73·6-82·5)	78·8 (74·1-82·8)	77·1 (68·2-85·3)	89·3 (86·1-92·1)	98·4 (97·2-99·1)	98·4 (97·2-99·2)	99·9 (99·3-100·0)
Saint Vincent and the Grenadines	44·5 (41·6-47·5)	50·4 (47·3-53·5)	50·5 (47·3-53·6)	55·6 (50·4-61·2)	66·4 (58·9-70·0)	79·1 (67·2-83·9)	78·5 (67·5-83·5)	91·3 (73·1-100·0)	79·3 (74·8-83·4)	83·0 (79·2-86·7)	83·1 (79·3-86·8)	84·7 (77·5-91·0)	97·5 (95·3-98·9)	99·3 (98·4-99·8)	99·4 (98·6-99·8)	100·0 (99·6-100·0)
Suriname	34·6 (32·1-37·2)	42·0 (38·3-45·4)	42·5 (38·7-45·9)	48·6 (43·7-52·9)	50·7 (41·1-55·3)	59·7 (47·0-65·8)	60·3 (47·4-66·7)	69·0 (51·5-81·4)	63·8 (58·0-69·4)	55·2 (50·7-59·6)	56·1 (51·2-60·7)	48·2 (35·9-58·3)	82·5 (78·5-85·9)	94·4 (91·4-96·5)	95·1 (92·2-97·0)	99·3 (97·4-100·0)
Trinidad and Tobago	51·6 (48·7-54·1)	58·9 (55·3-62·2)	59·1 (55·3-62·4)	65·5 (58·4-71·9)	66·8 (55·3-71·0)	76·3 (63·8-82·5)	76·5 (64·0-83·0)	81·1 (65·4-93·7)	71·0 (66·2-75·3)	62·0 (57·4-66·5)	62·5 (57·7-67·3)	59·6 (46·7-70·4)	58·1 (52·8-62·9)	51·8 (41·7-61·2)	54·3 (43·4-64·3)	62·2 (37·8-80·2)
**Tropical Latin America**																
Brazil	39·8 (37·1-42·7)	51·9 (49·4-54·2)	52·4 (50·0-54·7)	64·3 (60·8-68·4)	70·2 (66·0-74·0)	81·9 (78·2-86·2)	82·7 (79·1-86·9)	94·6 (88·9-100·0)	50·1 (47·8-52·2)	84·2 (83·3-85·1)	85·4 (84·5-86·3)	98·0 (97·0-99·2)	92·6 (88·4-95·5)	99·6 (99·3-99·8)	99·8 (99·5-99·9)	100·0 (100·0-100·0)
Paraguay	37·9 (35·8-39·8)	52·7 (48·9-55·6)	53·4 (49·6-56·4)	64·0 (59·1-68·0)	59·6 (53·3-64·0)	72·7 (60·0-80·4)	73·2 (60·4-81·2)	81·4 (63·2-98·7)	62·5 (59·1-65·8)	76·4 (73·1-79·4)	76·6 (73·3-79·8)	86·0 (81·0-90·4)	77·3 (73·5-80·9)	68·0 (59·1-75·3)	69·8 (60·8-77·1)	68·9 (44·4-85·8)
**East Asia**																
China	53·6 (51·9-55·4)	75·0 (72·3-78·0)	76·0 (73·3-79·1)	91·4 (87·0-97·0)	21·9 (17·1-25·0)	34·3 (27·9-50·2)	35·6 (28·9-51·6)	50·0 (38·4-76·8)	24·2 (21·9-26·3)	37·5 (35·7-39·2)	38·2 (36·3-40·0)	48·1 (45·0-51·1)	97·7 (94·4-99·1)	100·0 (99·8-100·0)	100·0 (99·8-100·0)	100·0 (100·0-100·0)
North Korea	46·0 (39·5-52·2)	58·0 (52·1-63·9)	58·3 (52·4-64·2)	63·3 (57·5-69·2)	19·4 (13·1-26·6)	21·4 (14·6-29·6)	21·7 (15·1-29·8)	26·4 (15·5-38·6)	37·3 (31·0-43·5)	45·8 (38·9-52·2)	46·1 (39·0-52·7)	51·7 (38·6-63·4)	88·8 (84·6-91·9)	98·5 (96·7-99·4)	98·6 (96·8-99·5)	99·9 (98·9-100·0)
Taiwan (Province of China)	76·9 (71·3-81·9)	78·5 (71·5-85·6)	78·7 (71·7-85·9)	81·5 (73·7-88·8)	52·2 (47·3-58·3)	67·4 (63·0-73·0)	67·2 (62·4-73·4)	80·6 (70·1-91·4)	35·9 (31·1-40·8)	67·8 (61·4-73·3)	67·6 (60·9-73·8)	85·1 (76·6-92·1)	99·4 (98·7-99·7)	96·5 (96·2-96·7)	97·9 (97·6-98·1)	100·0 (100·0-100·0)
**Southeast Asia**																
Cambodia	14·6 (12·0-17·1)	30·7 (26·0-34·4)	31·7 (27·0-35·6)	46·5 (40·3-51·6)	27·3 (14·7-39·3)	45·9 (38·7-53·0)	47·1 (39·9-53·7)	63·8 (51·2-75·6)	28·2 (24·3-32·2)	46·5 (41·5-51·6)	46·4 (40·9-52·2)	55·7 (43·7-66·0)	33·9 (28·9-39·0)	84·8 (79·2-89·1)	87·0 (81·4-91·0)	98·1 (94·7-99·6)
Indonesia	17·8 (11·8-25·6)	27·3 (20·6-33·9)	27·9 (21·0-34·6)	36·4 (27·2-45·8)	56·8 (50·5-72·4)	63·5 (57·0-85·8)	64·3 (57·5-86·5)	72·2 (59·3-100·0)	20·4 (17·5-23·3)	16·5 (13·7-19·2)	17·1 (14·2-19·9)	16·1 (10·3-21·2)	46·0 (40·8-50·8)	62·6 (53·9-70·7)	55·4 (41·1-66·9)	72·4 (49·9-88·9)
Laos	16·2 (11·8-20·5)	30·4 (24·8-35·6)	31·5 (25·8-36·7)	45·4 (38·3-52·4)	23·4 (13·9-40·6)	41·9 (33·6-52·6)	42·9 (34·3-53·3)	61·8 (48·4-74·9)	5·1 (0·0-12·6)	14·0 (1·5-24·6)	14·5 (2·4-25·1)	22·5 (1·1-41·5)	14·4 (8·5-20·4)	68·1 (60·7-75·0)	70·1 (61·2-77·8)	94·8 (86·3-99·2)
Malaysia	56·2 (51·6-61·9)	60·3 (55·6-66·3)	60·6 (55·9-66·8)	65·6 (60·1-72·0)	34·5 (30·9-39·7)	47·2 (39·6-53·7)	48·4 (40·3-55·0)	61·8 (45·9-74·0)	41·3 (35·4-47·4)	46·6 (40·9-52·0)	47·1 (40·3-53·0)	53·6 (40·9-64·2)	90·7 (88·3-92·8)	96·6 (94·5-97·9)	97·2 (95·2-98·4)	99·6 (98·3-100·0)
Maldives	51·1 (46·7-55·1)	63·7 (59·2-68·6)	64·4 (59·9-69·4)	75·8 (70·4-82·6)	80·9 (71·7-91·2)	100·0 (100·0-100·0)	100·0 (100·0-100·0)	100·0 (100·0-100·0)	36·2 (27·4-44·5)	34·8 (28·3-41·4)	35·9 (28·6-43·0)	37·8 (24·0-50·4)	91·3 (88·8-93·3)	94·7 (91·3-97·1)	94·7 (90·9-97·2)	94·2 (79·5-99·1)
Mauritius	63·6 (59·7-71·4)	65·2 (60·3-75·6)	65·6 (60·6-76·1)	70·8 (64·9-83·0)	68·4 (65·2-76·3)	91·0 (81·7-94·9)	91·3 (82·3-95·7)	99·2 (91·9-100·0)	53·9 (49·7-57·5)	52·4 (44·6-60·2)	52·7 (44·8-60·6)	55·2 (36·5-70·2)	87·8 (84·5-90·5)	98·0 (96·3-99·0)	98·3 (96·6-99·2)	99·7 (98·4-100·0)
Myanmar	15·1 (12·4-17·7)	29·5 (25·5-33·2)	30·5 (26·3-34·2)	43·8 (38·0-49·2)	22·6 (14·5-33·3)	43·1 (36·2-50·9)	44·1 (37·2-52·1)	63·5 (53·9-73·1)	32·2 (25·4-38·8)	52·6 (47·1-57·7)	52·5 (46·4-58·1)	62·6 (50·8-71·9)	82·9 (80·2-85·6)	95·3 (91·8-97·3)	95·7 (92·3-97·8)	99·0 (95·2-100·0)
Philippines	34·6 (30·8-38·4)	37·2 (32·6-41·5)	37·6 (33·0-42·1)	44·2 (37·5-50·5)	64·4 (60·6-74·0)	75·5 (70·7-81·7)	76·1 (70·9-82·7)	85·6 (74·9-96·8)	21·2 (16·2-25·8)	37·4 (30·0-44·7)	37·8 (29·7-45·8)	48·6 (32·8-61·9)	59·0 (50·4-66·9)	79·4 (70·9-85·3)	77·7 (67·8-84·2)	82·8 (58·3-94·2)
Sri Lanka	41·4 (37·4-44·7)	56·6 (50·6-62·3)	57·6 (51·3-63·4)	72·4 (63·2-81·0)	46·0 (39·5-49·6)	68·6 (55·9-75·9)	69·4 (56·4-77·0)	86·4 (63·3-100·0)	72·3 (69·1-75·5)	78·1 (75·1-81·1)	77·8 (74·5-81·1)	82·9 (77·2-88·5)	99·3 (98·7-99·7)	100·0 (99·7-100·0)	100·0 (99·8-100·0)	100·0 (100·0-100·0)
Seychelles	57·2 (52·7-61·7)	60·2 (56·2-64·8)	60·5 (56·4-65·1)	64·8 (60·1-70·1)	38·4 (34·2-45·4)	54·1 (47·6-58·6)	54·6 (47·7-59·6)	65·8 (53·7-76·3)	59·1 (52·3-66·0)	62·2 (55·7-67·8)	62·2 (55·4-68·2)	64·6 (51·5-75·4)	99·2 (98·6-99·6)	99·9 (99·5-100·0)	99·9 (99·6-100·0)	100·0 (99·8-100·0)
Thailand	43·4 (40·0-47·1)	46·6 (42·5-51·2)	47·0 (42·9-51·7)	53·1 (48·4-58·8)	57·2 (52·8-64·1)	72·3 (66·4-84·9)	73·0 (66·6-84·9)	85·0 (71·9-100·0)	33·8 (30·8-36·8)	51·1 (44·8-57·0)	51·5 (45·1-58·0)	59·8 (47·2-71·1)	77·6 (73·2-81·3)	88·9 (85·2-91·9)	89·0 (84·4-92·5)	90·7 (76·7-97·3)
Timor-Leste	12·3 (5·6-21·6)	29·4 (21·7-39·0)	30·5 (22·7-40·2)	46·8 (36·8-57·9)	37·8 (28·0-50·5)	56·5 (44·5-67·9)	56·8 (45·1-68·5)	72·7 (55·5-90·7)	2·8 (0·0-10·4)	17·0 (9·9-24·5)	17·6 (10·0-25·3)	26·3 (12·2-39·0)	0·0 (0·0-0·0)	18·5 (6·0-32·6)	19·5 (5·5-34·6)	42·1 (8·0-73·6)
Vietnam	42·3 (36·8-47·2)	56·6 (50·5-61·6)	57·3 (51·1-62·3)	69·6 (60·6-78·0)	28·8 (18·1-52·2)	42·5 (28·4-68·5)	43·3 (28·7-69·4)	58·2 (33·0-88·0)	29·5 (26·3-32·7)	51·8 (49·0-54·7)	51·8 (48·4-55·1)	59·8 (51·6-67·8)	66·5 (61·7-70·6)	77·7 (70·8-83·8)	79·0 (71·4-85·4)	88·7 (74·5-96·0)
**Oceania**																
Federated States of Micronesia	27·6 (24·9-30·4)	44·0 (38·1-48·9)	44·4 (38·4-49·3)	49·1 (42·4-54·9)	22·4 (15·9-29·7)	27·6 (20·2-36·2)	28·4 (21·1-36·8)	34·0 (19·3-49·3)	33·3 (24·6-40·8)	36·8 (28·8-44·6)	37·0 (28·8-44·7)	40·4 (25·0-53·6)	51·9 (43·3-59·1)	69·5 (60·1-76·7)	72·8 (62·8-80·3)	94·6 (82·3-99·8)
Fiji	34·7 (29·7-39·9)	39·4 (35·0-45·4)	39·8 (35·3-45·9)	45·5 (39·9-53·2)	40·1 (35·1-46·9)	48·3 (40·5-55·7)	48·5 (40·3-56·5)	52·2 (32·8-70·0)	64·5 (61·0-67·9)	66·0 (61·5-70·0)	65·9 (61·3-70·3)	67·9 (59·2-75·5)	66·8 (60·2-72·5)	80·6 (75·5-84·9)	82·2 (76·7-86·6)	94·6 (86·4-98·8)

Location	Indicator 5.2.1:<br>Age-standardised prevalence of women aged 15 years and older who experienced physical or sexual violence by an intimate partner in the last 12 months (%)				Indicator 6.1.1:<br>Risk-weighted prevalence of populations using unsafe or unimproved water sources, as measured by the summary exposure value (SEV) for unsafe water (%)				Indicator 6.2.1a:<br>Risk-weighted prevalence of populations using unsafe or unimproved sanitation, as measured by the summary exposure value (SEV) for unsafe sanitation (%)				Indicator 6.2.1b:<br>Risk-weighted prevalence of populations without access to a handwashing facility, as measured by the summary exposure value (SEV) for unsafe hygiene (%)			
	**2000**	**2015**	**2016**	**2030**	**2000**	**2015**	**2016**	**2030**	**2000**	**2015**	**2016**	**2030**	**2000**	**2015**	**2016**	**2030**
Guyana	55·0<br>(50·3-59·6)	65·3<br>(61·7-68·7)	65·7<br>(62·1-69·1)	71·3<br>(65·3-76·8)	39·0<br>(32·2-51·4)	39·9<br>(32·0-49·9)	40·5<br>(32·6-50·4)	48·7<br>(39·5-58·2)	46·5<br>(31·6-63·0)	73·7<br>(58·5-85·3)	75·4<br>(60·4-86·5)	91·4<br>(82·6-96·9)	48·6<br>(42·4-54·5)	44·3<br>(39·0-50·0)	44·9<br>(39·6-50·6)	44·6<br>(38·9-51·8)
Haiti	35·7<br>(32·0-39·2)	45·4<br>(41·5-48·9)	45·8<br>(41·9-49·4)	51·4<br>(46·5-55·6)	11·5<br>(5·8-21·4)	14·3<br>(7·5-26·6)	14·6<br>(7·6-27·1)	17·6<br>(10·2-31·6)	11·8<br>(0·4-24·9)	20·2<br>(2·7-40·5)	20·9<br>(2·9-42·2)	32·4<br>(11·9-66·7)	20·0<br>(16·0-24·6)	19·4<br>(15·4-23·5)	19·5<br>(15·5-23·7)	18·3<br>(14·5-24·2)
Jamaica	68·5<br>(65·4-71·3)	71·7<br>(69·1-74·2)	72·1<br>(69·5-74·6)	75·7<br>(73·2-78·2)	51·8<br>(44·4-68·0)	58·3<br>(50·7-74·9)	58·6<br>(51·0-75·3)	62·8<br>(55·0-78·4)	61·0<br>(47·6-74·4)	58·0<br>(33·5-81·4)	57·6<br>(31·0-82·5)	51·5<br>(10·0-91·9)	53·2<br>(47·3-59·1)	57·8<br>(52·3-63·4)	58·1<br>(52·6-63·7)	62·5<br>(57·1-67·5)
Saint Lucia	66·3<br>(62·4-69·7)	73·1<br>(70·2-75·6)	73·5<br>(70·7-76·0)	79·4<br>(77·0-81·7)	53·7<br>(44·8-70·5)	61·6<br>(53·5-78·2)	62·0<br>(54·0-78·6)	67·5<br>(60·4-83·0)	56·0<br>(43·6-70·2)	79·1<br>(62·7-90·6)	79·7<br>(62·4-91·5)	90·6<br>(69·8-98·9)	81·0<br>(77·0-84·8)	82·4<br>(78·5-85·9)	82·5<br>(78·6-86·0)	83·6<br>(79·7-87·5)
Saint Vincent and the Grenadines	60·2<br>(55·5-64·0)	66·1<br>(62·3-69·1)	66·6<br>(62·9-69·6)	72·8<br>(69·5-75·7)	45·6<br>(38·2-57·8)	53·2<br>(45·1-65·9)	53·5<br>(45·5-66·3)	58·6<br>(50·0-71·4)	53·0<br>(40·6-66·0)	68·6<br>(34·5-90·9)	69·0<br>(34·6-91·2)	77·6<br>(41·7-97·6)	66·8<br>(60·8-72·3)	73·4<br>(67·5-78·5)	73·7<br>(67·8-78·8)	78·7<br>(72·6-84·3)
Suriname	64·1<br>(60·0-67·7)	72·6<br>(69·7-75·4)	73·3<br>(70·4-75·9)	81·5<br>(79·0-83·8)	38·6<br>(30·9-49·4)	51·4<br>(43·0-62·2)	52·3<br>(43·9-63·1)	63·9<br>(55·7-73·6)	70·1<br>(61·5-78·4)	82·8<br>(65·1-94·0)	83·1<br>(63·9-94·8)	89·1<br>(58·8-100·0)	61·5<br>(55·7-66·7)	68·1<br>(62·9-72·9)	68·7<br>(63·4-73·3)	75·4<br>(68·8-79·7)
Trinidad and Tobago	70·5<br>(66·7-73·6)	76·1<br>(73·0-78·8)	76·7<br>(73·6-79·3)	83·9<br>(79·7-87·3)	55·3<br>(47·4-66·2)	65·0<br>(56·9-76·7)	65·2<br>(57·0-77·0)	68·3<br>(39·3-89·9)	67·8<br>(58·0-77·6)	84·7<br>(65·0-95·0)	84·3<br>(62·9-95·1)	91·9<br>(72·7-99·7)	73·5<br>(68·4-78·2)	78·7<br>(74·2-82·9)	78·8<br>(74·2-83·0)	83·1<br>(76·8-87·3)
**Tropical Latin America**																
Brazil	68·6<br>(65·7-70·9)	77·1<br>(75·1-78·6)	77·6<br>(75·7-79·1)	84·9<br>(83·5-86·1)	74·5<br>(72·7-77·2)	81·6<br>(80·2-83·6)	82·0<br>(80·6-83·9)	86·8<br>(85·7-88·2)	59·6<br>(53·1-65·5)	66·3<br>(57·5-73·6)	66·9<br>(58·0-74·3)	74·7<br>(65·3-82·2)	68·6<br>(67·0-70·1)	76·5<br>(75·1-77·8)	76·9<br>(75·5-78·2)	82·1<br>(80·9-83·3)
Paraguay	61·8<br>(58·4-64·9)	70·9<br>(67·8-73·7)	71·4<br>(68·2-74·2)	78·7<br>(75·3-81·3)	55·0<br>(49·3-62·5)	78·9<br>(74·0-82·9)	79·6<br>(74·8-83·4)	87·3<br>(84·7-89·6)	57·5<br>(48·9-69·5)	81·6<br>(62·4-93·6)	81·9<br>(61·4-94·1)	93·4<br>(73·3-100·0)	51·2<br>(44·7-57·7)	75·9<br>(71·1-80·4)	76·4<br>(71·7-80·9)	85·4<br>(81·2-88·7)
**East Asia**																
China	52·3<br>(49·0-55·4)	69·0<br>(66·7-70·9)	70·1<br>(67·8-72·0)	83·5<br>(80·6-86·1)	64·2<br>(58·2-80·6)	74·2<br>(68·4-89·0)	74·8<br>(69·1-89·4)	82·0<br>(76·8-93·9)	46·6<br>(42·7-50·5)	73·8<br>(70·1-77·2)	75·0<br>(71·4-78·4)	89·8<br>(86·9-92·2)	80·0<br>(77·7-82·3)	87·9<br>(86·1-89·5)	88·4<br>(86·6-89·9)	93·9<br>(92·7-94·8)
North Korea	46·4<br>(42·2-50·2)	47·4<br>(43·8-50·9)	47·8<br>(44·2-51·3)	49·3<br>(44·4-54·0)	60·8<br>(45·2-81·7)	61·2<br>(46·3-81·2)	61·5<br>(46·5-81·3)	65·8<br>(31·5-90·5)	48·5<br>(35·5-61·9)	52·1<br>(36·1-68·1)	52·4<br>(36·2-68·7)	56·7<br>(35·5-76·0)	86·2<br>(76·9-93·1)	85·7<br>(76·2-93·0)	85·9<br>(76·3-93·1)	86·5<br>(77·0-93·5)
Taiwan (Province of China)	69·4<br>(66·6-72·1)	81·3<br>(79·2-83·0)	81·7<br>(79·7-83·5)	89·1<br>(86·9-91·0)	80·2<br>(64·1-92·5)	81·6<br>(65·7-94·0)	81·7<br>(66·2-94·1)	83·9<br>(63·1-97·6)	87·6<br>(76·9-95·3)	94·2<br>(88·2-98·1)	94·4<br>(88·6-98·2)	97·5<br>(93·8-99·7)	94·0<br>(88·1-98·3)	94·7<br>(88·8-98·6)	94·8<br>(88·8-98·6)	95·5<br>(89·3-98·9)
**Southeast Asia**																
Cambodia	37·8<br>(34·0-41·5)	49·5<br>(45·8-53·5)	50·1<br>(46·4-54·0)	58·6<br>(53·7-64·0)	39·1<br>(25·9-72·9)	49·6<br>(35·4-85·2)	50·2<br>(35·9-85·8)	58·1<br>(8·2-96·4)	11·5<br>(5·6-17·7)	50·5<br>(40·4-60·8)	52·8<br>(42·3-63·4)	83·8<br>(72·0-91·6)	56·7<br>(42·4-70·7)	67·5<br>(55·1-78·7)	68·1<br>(55·9-79·2)	76·3<br>(65·2-85·9)
Indonesia	56·0<br>(52·4-59·3)	64·7<br>(61·9-67·1)	65·4<br>(62·7-67·8)	74·2<br>(71·9-76·2)	63·3<br>(49·7-99·7)	60·7<br>(47·1-99·2)	60·8<br>(47·1-99·2)	62·3<br>(6·7-99·9)	51·2<br>(44·8-56·6)	75·4<br>(70·9-79·7)	76·7<br>(72·1-81·0)	90·1<br>(86·6-93·5)	75·7<br>(72·4-78·7)	75·4<br>(73·4-77·4)	75·6<br>(73·6-77·6)	78·8<br>(76·8-80·8)
Laos	44·3<br>(39·2-49·4)	54·2<br>(49·4-58·7)	54·9<br>(50·2-59·3)	63·5<br>(58·7-67·6)	42·8<br>(30·9-75·8)	47·7<br>(35·4-78·2)	48·2<br>(35·7-78·7)	55·4<br>(0·0-96·2)	11·6<br>(4·6-20·1)	29·1<br>(16·3-44·3)	30·1<br>(16·7-46·0)	45·9<br>(24·1-71·0)	57·9<br>(43·7-71·7)	56·7<br>(41·4-70·8)	57·2<br>(41·8-71·2)	54·7<br>(36·2-73·4)
Malaysia	55·7<br>(50·7-60·0)	65·6<br>(61·4-69·2)	66·4<br>(62·3-70·0)	74·0<br>(70·7-77·1)	76·2<br>(63·4-89·7)	78·2<br>(64·4-90·4)	78·5<br>(64·7-90·4)	81·6<br>(59·7-95·2)	91·8<br>(85·0-96·5)	96·2<br>(90·1-99·2)	96·3<br>(90·2-99·3)	98·1<br>(93·1-100·0)	97·0<br>(92·7-100·0)	97·2<br>(92·9-100·0)	97·2<br>(93·0-100·0)	97·7<br>(93·9-100·0)
Maldives	50·3<br>(44·3-55·0)	62·5<br>(57·8-66·4)	63·4<br>(58·8-67·3)	73·7<br>(68·5-77·9)	25·6<br>(19·1-35·5)	35·0<br>(25·9-46·8)	36·0<br>(26·6-47·9)	48·8<br>(35·7-63·6)	46·9<br>(32·0-63·7)	79·6<br>(68·0-88·4)	80·9<br>(69·4-89·5)	94·3<br>(86·8-98·4)	62·1<br>(47·8-75·4)	71·4<br>(58·0-83·1)	72·5<br>(59·4-83·9)	80·0<br>(68·3-89·3)
Mauritius	64·3<br>(60·7-67·9)	70·6<br>(67·9-73·3)	71·2<br>(68·5-73·8)	78·4<br>(75·7-80·7)	72·1<br>(55·5-95·1)	72·0<br>(55·7-92·6)	72·2<br>(56·0-92·6)	74·8<br>(58·7-96·9)	92·1<br>(85·2-96·5)	96·2<br>(90·3-99·0)	96·3<br>(90·3-99·1)	98·1<br>(92·9-100·0)	98·6<br>(95·3-100·0)	98·2<br>(94·6-100·0)	98·2<br>(94·6-100·0)	98·0<br>(93·7-100·0)
Myanmar	40·0<br>(36·5-43·6)	57·2<br>(54·1-60·3)	58·1<br>(55·0-61·3)	70·8<br>(67·8-74·0)	29·6<br>(21·6-50·9)	38·1<br>(27·9-61·4)	38·8<br>(28·6-62·4)	47·9<br>(8·7-83·1)	19·0<br>(0·0-36·4)	48·6<br>(28·3-67·0)	51·0<br>(30·8-69·1)	79·9<br>(70·7-89·4)	53·3<br>(37·7-67·7)	56·3<br>(42·0-70·3)	56·8<br>(42·5-70·7)	61·0<br>(45·7-74·5)
Philippines	43·3<br>(39·7-47·3)	51·0<br>(47·5-54·7)	51·4<br>(47·8-55·0)	55·8<br>(52·1-59·6)	40·6<br>(29·1-54·4)	46·0<br>(34·5-61·3)	46·8<br>(35·0-62·2)	57·8<br>(40·4-74·1)	68·5<br>(62·0-74·3)	86·4<br>(78·9-92·2)	87·0<br>(79·5-92·8)	95·1<br>(88·5-98·5)	76·9<br>(65·6-86·3)	76·0<br>(63·1-86·1)	76·6<br>(63·8-86·6)	75·3<br>(59·0-89·1)
Sri Lanka	42·2<br>(38·4-45·7)	58·4<br>(55·2-61·2)	59·5<br>(56·3-62·3)	73·3<br>(70·7-75·7)	49·1<br>(35·7-74·7)	60·6<br>(45·6-84·3)	61·5<br>(46·5-84·9)	73·1<br>(32·1-97·0)	30·9<br>(19·5-42·4)	61·5<br>(43·0-77·4)	63·5<br>(44·6-79·2)	85·7<br>(68·7-95·2)	74·8<br>(62·8-85·4)	84·0<br>(73·6-91·9)	84·7<br>(74·6-92·3)	91·2<br>(83·3-96·6)
Seychelles	52·9<br>(48·7-57·2)	58·7<br>(55·2-62·2)	59·4<br>(55·9-62·8)	66·4<br>(62·6-69·6)	70·4<br>(53·9-90·3)	69·8<br>(54·0-91·5)	70·1<br>(54·4-91·8)	74·3<br>(43·0-95·5)	81·1<br>(67·0-91·5)	87·5<br>(77·3-94·5)	88·0<br>(78·2-94·8)	93·9<br>(88·2-97·8)	95·6<br>(90·6-99·3)	95·2<br>(90·1-99·0)	95·3<br>(90·3-99·0)	94·9<br>(89·2-99·5)
Thailand	48·8<br>(44·4-53·1)	65·5<br>(62·1-68·7)	66·3<br>(63·0-69·3)	76·8<br>(73·7-79·4)	32·8<br>(26·2-40·8)	35·8<br>(28·0-46·6)	36·3<br>(28·2-47·1)	43·0<br>(31·8-55·0)	92·0<br>(85·0-96·5)	97·4<br>(94·7-99·3)	97·5<br>(95·0-99·4)	99·4<br>(98·5-100·0)	70·3<br>(56·0-82·3)	70·7<br>(55·6-82·6)	71·1<br>(56·1-82·9)	72·4<br>(55·2-86·0)
Timor-Leste	21·7<br>(17·1-25·8)	35·0<br>(30·7-39·2)	35·8<br>(31·3-40·1)	47·3<br>(40·7-53·7)	60·5<br>(48·0-98·5)	63·1<br>(49·0-100·0)	63·8<br>(49·6-100·0)	72·2<br>(0·0-100·0)	5·8<br>(0·1-13·5)	34·6<br>(20·9-49·8)	36·7<br>(22·4-52·3)	71·5<br>(54·8-85·0)	69·9<br>(56·2-82·3)	67·7<br>(53·2-80·1)	68·7<br>(54·2-80·9)	65·7<br>(48·8-86·7)
Vietnam	56·1<br>(52·3-59·7)	65·1<br>(62·0-68·0)	65·8<br>(62·6-68·6)	73·5<br>(70·1-76·5)	66·5<br>(53·6-100·0)	69·3<br>(56·8-100·0)	69·6<br>(57·0-100·0)	72·8<br>(31·5-100·0)	21·9<br>(16·4-28·7)	63·6<br>(49·9-76·8)	65·3<br>(51·3-78·7)	88·1<br>(75·0-96·5)	68·2<br>(54·2-79·7)	69·0<br>(57·5-79·3)	69·4<br>(58·0-79·7)	70·9<br>(59·8-83·9)
**Oceania**																
Federated States of Micronesia	38·8<br>(34·4-43·1)	46·4<br>(41·4-50·8)	46·6<br>(41·7-51·1)	53·0<br>(46·6-58·4)	41·2<br>(26·2-62·1)	54·0<br>(34·5-74·9)	54·5<br>(34·9-75·1)	61·5<br>(39·7-79·5)	61·7<br>(40·9-79·4)	78·8<br>(62·7-90·4)	79·3<br>(63·3-90·7)	88·8<br>(76·3-96·2)	78·8<br>(66·6-88·3)	85·3<br>(75·9-92·6)	85·5<br>(76·4-92·7)	89·7<br>(82·1-96·3)
Fiji	53·1<br>(48·8-56·6)	60·0<br>(56·6-63·0)	60·3<br>(56·9-63·3)	65·3<br>(62·1-68·0)	61·2<br>(45·1-75·7)	68·6<br>(48·2-84·2)	69·0<br>(48·1-84·6)	74·0<br>(46·6-89·7)	80·5<br>(65·1-91·2)	84·0<br>(68·1-94·1)	84·0<br>(68·3-94·1)	86·4<br>(74·1-95·8)	89·4<br>(80·9-95·2)	91·8<br>(84·7-97·0)	91·9<br>(84·8-97·1)	93·9<br>(87·6-98·5)

Location	**Indicator 7.1.2:** Risk-weighted prevalence of household air pollution, as measured by the summary exposure value (SEV) for household air pollution (%)				**Indicator 8.8.1:** Age-standardised all-cause disability-adjusted life year (DALY) rates attributable to occupational risks (per 100,000 population)				**Indicator 11.6.2:** Population-weighted mean levels of fine particulate matter smaller than 2.5 microns in diameter (PM2.5)				**Indicator 16.1.1:** Age-standardised death rate due to interpersonal violence (per 100,000 population)			
	**2000**	**2015**	**2016**	**2030**	**2000**	**2015**	**2016**	**2030**	**2000**	**2015**	**2016**	**2030**	**2000**	**2015**	**2016**	**2030**
Guyana	89·5<br>(86·6-92·1)	94·1<br>(91·4-96·3)	94·5<br>(91·8-96·6)	97·8<br>(95·9-99·0)	50·4<br>(30·5-64·8)	59·3<br>(40·8-73·5)	59·9<br>(41·5-74·0)	67·7<br>(50·0-81·5)	47·1<br>(34·7-56·0)	57·3<br>(46·0-66·0)	57·3<br>(46·0-66·3)	58·2<br>(41·4-73·7)	14·0<br>(6·9-18·8)	12·8<br>(6·4-20·8)	13·9<br>(7·3-21·7)	17·1<br>(6·8-27·6)
Haiti	15·4<br>(7·0-24·8)	26·4<br>(17·2-35·2)	27·4<br>(18·2-36·3)	41·5<br>(31·7-50·3)	60·1<br>(47·9-70·9)	66·1<br>(54·2-76·9)	66·5<br>(54·6-77·3)	71·2<br>(59·2-81·7)	42·0<br>(36·3-46·1)	50·9<br>(45·4-55·1)	51·0<br>(45·8-55·3)	51·6<br>(45·5-56·4)	26·8<br>(19·3-38·7)	38·1<br>(28·8-46·1)	38·0<br>(28·7-46·1)	37·7<br>(21·2-53·6)
Jamaica	88·0<br>(84·3-90·8)	93·8<br>(90·7-96·2)	94·1<br>(91·1-96·5)	97·2<br>(94·9-98·9)	57·2<br>(41·7-68·5)	67·8<br>(53·2-79·8)	68·3<br>(53·6-80·5)	74·4<br>(59·5-88·3)	55·6<br>(53·6-57·8)	65·9<br>(63·7-68·1)	66·0<br>(63·9-68·2)	67·3<br>(64·7-70·2)	18·2<br>(8·2-23·6)	9·3<br>(1·8-17·0)	9·8<br>(2·3-17·6)	17·0<br>(0·2-32·3)
Saint Lucia	90·2<br>(86·3-93·3)	97·5<br>(95·7-98·7)	97·7<br>(96·0-98·9)	99·4<br>(98·7-99·8)	74·7<br>(65·8-82·9)	93·0<br>(85·1-100·0)	93·7<br>(85·9-100·0)	99·7<br>(96·9-100·0)	58·1<br>(43·7-71·6)	62·4<br>(47·0-76·8)	62·1<br>(46·4-76·2)	62·2<br>(41·7-80·0)	22·0<br>(15·8-26·8)	19·5<br>(13·9-29·0)	19·8<br>(13·8-29·3)	22·4<br>(11·3-34·6)
Saint Vincent and the Grenadines	79·4<br>(69·2-87·3)	96·3<br>(93·5-98·2)	96·6<br>(93·9-98·4)	98·7<br>(97·1-99·6)	100·0<br>(100·0-100·0)	99·9<br>(98·6-100·0)	99·9<br>(98·2-100·0)	99·1<br>(93·6-100·0)	59·2<br>(39·7-76·3)	63·5<br>(47·7-79·9)	62·2<br>(45·6-78·0)	62·8<br>(43·1-81·7)	22·1<br>(13·9-27·3)	12·8<br>(7·3-22·0)	13·3<br>(7·7-22·4)	8·1<br>(0·0-22·4)
Suriname	85·0<br>(78·9-90·6)	92·0<br>(88·1-95·2)	92·4<br>(88·4-95·6)	96·3<br>(92·0-98·9)	83·5<br>(64·4-97·4)	85·7<br>(69·6-97·7)	85·3<br>(69·1-97·3)	79·8<br>(63·4-91·8)	45·8<br>(24·3-60·1)	54·6<br>(36·6-67·9)	54·9<br>(36·6-67·9)	56·0<br>(36·0-74·1)	35·7<br>(28·5-40·0)	31·2<br>(25·7-39·3)	31·4<br>(25·8-39·6)	29·8<br>(19·6-41·8)
Trinidad and Tobago	99·4<br>(98·8-99·8)	99·7<br>(99·4-99·9)	99·7<br>(99·5-99·9)	99·9<br>(99·7-99·9)	78·3<br>(67·4-89·2)	94·1<br>(82·9-100·0)	94·2<br>(82·9-100·0)	94·4<br>(82·7-100·0)	57·8<br>(51·1-63·9)	62·9<br>(56·4-68·4)	62·5<br>(56·4-68·4)	63·7<br>(53·3-79·3)	23·6<br>(16·2-28·2)	13·6<br>(6·7-25·1)	14·2<br>(7·0-25·8)	22·4<br>(10·4-35·1)
**Tropical Latin America**																
Brazil	89·6<br>(87·8-91·2)	97·0<br>(96·4-97·6)	97·3<br>(96·7-97·8)	99·3<br>(99·1-99·5)	41·7<br>(38·7-44·5)	52·2<br>(49·2-54·9)	52·9<br>(49·9-55·6)	61·9<br>(58·4-64·9)	63·7<br>(62·5-64·8)	72·1<br>(70·9-73·3)	71·9<br>(70·8-73·0)	68·7<br>(64·9-74·2)	4·0<br>(0·0-7·6)	5·3<br>(0·0-10·7)	5·6<br>(0·3-10·7)	6·9<br>(1·9-14·1)
Paraguay	59·7<br>(53·8-65·4)	78·8<br>(70·4-86·0)	80·3<br>(72·0-87·3)	93·5<br>(88·2-97·2)	35·0<br>(31·2-38·6)	43·3<br>(39·4-47·3)	43·9<br>(40·0-47·9)	52·9<br>(48·2-57·8)	51·4<br>(48·4-54·2)	51·6<br>(48·1-54·7)	50·8<br>(47·0-54·1)	50·4<br>(46·0-54·3)	17·0<br>(11·4-21·6)	20·8<br>(12·0-26·1)	20·8<br>(12·0-26·6)	21·5<br>(7·1-33·9)
**East Asia**																
China	52·2<br>(47·3-56·8)	77·6<br>(74·1-80·8)	79·0<br>(75·7-82·0)	92·3<br>(90·9-93·5)	19·4<br>(14·0-24·6)	49·0<br>(44·2-53·5)	50·4<br>(45·5-54·8)	69·4<br>(64·3-73·9)	24·4<br>(23·6-25·6)	21·4<br>(20·5-22·6)	21·5<br>(20·6-22·6)	19·3<br>(18·3-20·5)	55·4<br>(50·7-62·4)	78·4<br>(67·3-82·2)	78·6<br>(67·7-82·4)	97·0<br>(78·5-100·0)
North Korea	26·6<br>(17·6-35·5)	33·4<br>(23·0-46·9)	34·4<br>(23·9-47·9)	46·8<br>(35·4-61·1)	17·4<br>(8·5-26·0)	16·7<br>(8·7-24·3)	17·1<br>(9·0-24·6)	21·8<br>(13·9-29·4)	41·9<br>(38·6-44·8)	36·7<br>(33·9-39·3)	36·6<br>(33·8-39·1)	35·4<br>(32·2-38·6)	58·9<br>(51·5-65·6)	61·5<br>(51·6-69·1)	61·7<br>(51·9-69·2)	64·4<br>(52·4-75·8)
Taiwan (Province of China)	93·9<br>(87·7-97·5)	97·9<br>(95·5-99·3)	98·1<br>(95·9-99·4)	99·4<br>(98·7-99·8)	65·8<br>(60·7-70·6)	69·7<br>(63·4-75·4)	69·7<br>(63·4-75·4)	69·2<br>(62·5-75·9)	42·8<br>(41·1-44·6)	43·5<br>(41·6-45·2)	43·5<br>(41·6-45·4)	43·1<br>(39·1-45·8)	51·0<br>(45·2-66·9)	76·5<br>(66·7-81·4)	76·2<br>(66·3-81·7)	90·9<br>(73·3-100·0)
**Southeast Asia**																
Cambodia	15·4<br>(6·8-24·8)	37·4<br>(30·0-44·6)	39·0<br>(31·7-46·2)	60·0<br>(55·0-64·8)	0·3<br>(0·0-3·1)	11·7<br>(5·7-18·0)	12·3<br>(6·3-18·7)	21·9<br>(15·0-28·9)	48·9<br>(44·7-52·4)	47·4<br>(42·8-50·8)	47·5<br>(42·1-51·1)	46·9<br>(41·3-50·7)	47·9<br>(42·9-55·3)	51·7<br>(44·3-59·1)	51·7<br>(43·9-59·4)	53·0<br>(38·3-65·9)
Indonesia	63·3<br>(59·5-67·0)	79·9<br>(76·6-82·8)	81·9<br>(78·8-84·6)	96·2<br>(95·4-96·9)	14·3<br>(9·5-19·9)	14·9<br>(10·1-19·9)	15·6<br>(10·8-20·6)	24·9<br>(20·1-30·1)	63·0<br>(61·9-64·1)	62·6<br>(61·5-63·7)	62·5<br>(61·4-63·5)	62·3<br>(61·1-63·7)	68·0<br>(63·2-74·4)	71·0<br>(66·3-77·1)	71·3<br>(66·6-77·4)	74·3<br>(67·8-81·6)
Laos	16·9<br>(9·0-25·6)	28·5<br>(20·7-37·0)	29·5<br>(21·6-37·9)	43·0<br>(36·1-50·9)	1·8<br>(0·0-6·8)	11·7<br>(5·7-17·9)	12·5<br>(6·4-18·7)	23·2<br>(16·4-29·7)	46·3<br>(44·0-48·3)	45·2<br>(43·1-47·1)	45·5<br>(43·2-47·4)	44·4<br>(41·8-46·6)	92·8<br>(83·8-100·0)	96·7<br>(89·8-100·0)	96·8<br>(90·0-100·0)	97·8<br>(87·1-100·0)
Malaysia	97·9<br>(94·2-99·5)	99·6<br>(99·0-99·9)	99·7<br>(99·1-99·9)	100·0<br>(99·9-100·0)	45·6<br>(38·9-51·9)	58·8<br>(52·7-64·3)	59·5<br>(53·4-65·0)	69·3<br>(62·7-75·6)	64·1<br>(62·5-65·7)	61·0<br>(59·2-62·8)	60·8<br>(59·0-62·5)	59·5<br>(57·7-61·2)	62·9<br>(52·1-67·6)	69·1<br>(55·0-76·0)	69·7<br>(55·2-76·8)	78·8<br>(58·6-92·5)
Maldives	66·9<br>(58·6-74·5)	96·5<br>(92·4-98·8)	96·9<br>(93·0-99·0)	99·4<br>(97·8-100·0)	48·0<br>(41·3-54·7)	76·6<br>(68·7-84·4)	77·4<br>(69·4-85·3)	89·0<br>(79·6-97·9)	44·0<br>(30·3-57·5)	47·3<br>(33·4-60·4)	47·2<br>(33·0-59·8)	46·3<br>(31·7-59·9)	77·3<br>(71·8-87·2)	83·6<br>(74·5-100·0)	83·7<br>(74·3-100·0)	86·0<br>(64·8-100·0)
Mauritius	96·1<br>(92·0-98·5)	98·7<br>(96·6-99·7)	98·8<br>(96·8-99·7)	99·6<br>(98·6-100·0)	67·5<br>(61·3-73·7)	77·6<br>(69·1-86·1)	78·0<br>(69·5-86·7)	84·2<br>(74·3-95·2)	59·8<br>(52·9-65·7)	67·6<br>(60·8-73·9)	67·6<br>(61·1-73·7)	66·5<br>(59·8-72·8)	64·9<br>(53·1-69·6)	62·8<br>(56·1-69·8)	63·3<br>(55·6-70·2)	68·6<br>(55·9-81·7)
Myanmar	18·0<br>(8·4-27·6)	42·1<br>(29·8-58·5)	43·9<br>(31·4-60·9)	67·0<br>(54·8-82·8)	35·7<br>(25·8-42·9)	44·2<br>(37·5-50·8)	45·0<br>(38·4-51·6)	55·9<br>(49·5-62·6)	29·6<br>(28·5-30·9)	26·2<br>(25·0-27·5)	26·3<br>(25·0-27·6)	25·4<br>(23·9-26·9)	60·6<br>(54·7-66·7)	66·9<br>(59·5-74·0)	67·3<br>(59·7-74·6)	72·4<br>(58·9-85·4)
Philippines	66·7<br>(59·9-73·0)	62·5<br>(51·8-72·8)	63·5<br>(52·5-73·8)	75·6<br>(62·9-86·5)	29·3<br>(23·2-35·1)	36·5<br>(29·6-42·7)	37·1<br>(30·1-43·4)	45·7<br>(37·6-53·3)	48·6<br>(46·7-50·4)	51·4<br>(49·6-53·1)	51·4<br>(49·7-53·0)	51·4<br>(48·2-53·7)	15·9<br>(9·2-30·2)	20·1<br>(13·8-30·7)	20·2<br>(13·6-31·3)	22·1<br>(7·6-36·3)
Sri Lanka	44·3<br>(35·0-54·0)	64·2<br>(49·9-78·6)	66·2<br>(51·9-80·6)	85·7<br>(73·3-95·5)	61·3<br>(55·5-67·6)	71·7<br>(64·2-79·2)	72·7<br>(65·1-80·3)	87·3<br>(78·1-96·5)	41·9<br>(39·8-43·9)	47·9<br>(45·6-50·0)	48·1<br>(45·8-50·2)	48·2<br>(45·8-50·7)	33·6<br>(28·0-40·3)	47·5<br>(38·7-54·9)	47·7<br>(38·2-55·4)	52·7<br>(32·1-70·8)
Seychelles	97·7<br>(94·5-99·4)	99·3<br>(98·0-99·8)	99·3<br>(98·2-99·9)	99·8<br>(99·5-100·0)	51·9<br>(45·4-58·0)	66·3<br>(58·9-73·0)	67·0<br>(59·6-73·8)	77·5<br>(69·5-84·8)	59·7<br>(35·0-84·2)	68·6<br>(43·3-91·0)	68·7<br>(46·9-92·4)	69·6<br>(45·4-95·0)	31·0<br>(25·0-42·2)	41·4<br>(35·5-48·8)	41·5<br>(35·0-49·3)	49·9<br>(37·6-61·3)
Thailand	73·4<br>(66·9-79·7)	91·9<br>(85·4-96·0)	92·7<br>(86·5-96·6)	98·6<br>(95·8-99·7)	23·6<br>(17·1-29·7)	37·6<br>(30·7-44·1)	38·5<br>(31·5-45·0)	49·8<br>(42·0-57·1)	50·5<br>(49·6-51·6)	51·3<br>(50·3-52·4)	51·5<br>(50·5-52·5)	50·7<br>(49·7-51·9)	25·0<br>(18·9-37·1)	40·2<br>(34·0-45·9)	40·2<br>(34·0-46·4)	44·2<br>(31·2-56·7)
Timor-Leste	16·7<br>(7·9-26·3)	35·1<br>(24·4-47·8)	36·6<br>(26·0-49·4)	56·6<br>(47·4-67·8)	50·8<br>(38·7-70·6)	64·5<br>(55·3-80·9)	65·1<br>(56·0-81·2)	74·1<br>(65·1-87·2)	56·4<br>(48·4-63·1)	62·0<br>(53·9-68·8)	62·1<br>(54·8-68·9)	63·2<br>(55·4-70·3)	55·9<br>(47·2-69·1)	59·8<br>(49·2-75·9)	59·5<br>(49·3-75·1)	61·5<br>(44·5-83·8)
Vietnam	42·0<br>(33·5-50·0)	75·3<br>(65·6-83·1)	76·9<br>(67·1-84·5)	91·9<br>(84·4-96·1)	43·2<br>(36·5-49·7)	45·7<br>(39·9-51·4)	46·3<br>(40·6-52·0)	55·3<br>(49·2-61·4)	46·2<br>(44·9-47·5)	47·1<br>(45·8-48·4)	47·2<br>(45·8-48·5)	46·7<br>(45·2-48·1)	68·0<br>(60·1-75·2)	70·0<br>(62·6-77·9)	70·1<br>(62·6-78·1)	74·1<br>(58·8-88·8)
**Oceania**																
Federated States of Micronesia	64·5<br>(54·9-73·4)	81·2<br>(72·4-88·1)	82·1<br>(73·5-88·9)	91·6<br>(85·6-95·5)	37·0<br>(24·3-49·1)	36·6<br>(21·8-50·3)	36·6<br>(21·8-50·4)	37·0<br>(21·0-51·6)	84·0<br>(59·2-100·0)	86·8<br>(63·3-100·0)	86·2<br>(61·1-100·0)	85·7<br>(60·7-100·0)	37·9<br>(30·0-46·2)	37·4<br>(27·8-47·3)	37·9<br>(28·1-48·6)	38·5<br>(23·7-55·7)
Fiji	73·1<br>(63·5-81·7)	87·8<br>(81·0-92·7)	88·4<br>(81·8-93·1)	94·8<br>(91·0-97·0)	33·8<br>(19·8-48·0)	44·5<br>(24·6-62·3)	45·1<br>(24·7-63·1)	54·2<br>(27·4-76·3)	85·2<br>(72·0-95·8)	90·3<br>(76·9-100·0)	89·5<br>(75·0-100·0)	88·5<br>(71·8-100·0)	42·2<br>(36·2-51·0)	45·0<br>(37·0-55·7)	44·7<br>(36·3-55·3)	42·7<br>(23·9-63·4)

Location	Indicator 16.1.2: Death rate due to conflict and terrorism (per 100,000 population)				Indicator 16.1.3: Age-standardised prevalence of physical or sexual violence experienced by populations in the last 12 months (%)				Indicator 16.2.3: Age-standardised prevalence of women and men aged 18-29 years who experienced sexual violence by age 18 (%)				Indicator 17.19.2c: Percentage of well-certified deaths by a vital registration (VR) system among a country's total population (%)			
	**2000**	**2015**	**2016**	**2030**	**2000**	**2015**	**2016**	**2030**	**2000**	**2015**	**2016**	**2030**	**2000**	**2015**	**2016**	**2030**
Guyana	100·0 (100·0-100·0)	100·0 (100·0-100·0)	100·0 (100·0-100·0)	100·0 (100·0-100·0)	76·1 (72·8-78·8)	81·2 (78·3-83·7)	81·6 (78·7-84·0)	86·3 (83·9-88·4)	74·5 (71·1-78·2)	75·3 (71·8-79·0)	75·3 (71·8-79·1)	75·8 (72·0-79·5)	75·2 (70·3-79·5)	80·6 (72·0-87·8)	81·4 (72·4-89·0)	90·4 (73·4-100·0)
Haiti	70·5 (60·8-86·0)	70·6 (60·9-86·1)	100·0 (100·0-100·0)	100·0 (100·0-100·0)	46·6 (42·9-50·0)	59·7 (56·6-62·4)	60·1 (56·9-62·8)	66·0 (62·5-69·1)	75·2 (71·8-78·8)	74·7 (71·2-78·3)	74·6 (71·2-78·2)	74·5 (71·1-78·2)	9·4 (6·4-13·7)	9·5 (5·6-14·9)	9·7 (5·5-15·0)	15·9 (5·6-33·5)
Jamaica	100·0 (100·0-100·0)	100·0 (100·0-100·0)	100·0 (100·0-100·0)	100·0 (100·0-100·0)	88·6 (87·2-89·9)	89·5 (88·1-90·8)	89·7 (88·3-91·0)	91·9 (90·5-93·3)	67·0 (62·9-70·9)	66·7 (62·7-70·5)	66·6 (62·5-70·5)	65·8 (61·5-70·0)	67·8 (61·6-73·9)	79·2 (70·8-87·3)	79·3 (70·4-87·9)	87·2 (72·1-98·5)
Saint Lucia	100·0 (100·0-100·0)	100·0 (100·0-100·0)	100·0 (100·0-100·0)	100·0 (100·0-100·0)	81·6 (78·9-84·0)	84·4 (81·9-86·7)	84·7 (82·3-86·9)	88·2 (85·9-90·2)	74·8 (71·3-78·4)	74·8 (71·4-78·3)	74·8 (71·3-78·3)	74·7 (71·2-78·2)	76·4 (71·8-80·7)	87·8 (82·3-92·9)	88·1 (82·0-93·4)	93·0 (85·0-99·0)
Saint Vincent and the Grenadines	100·0 (100·0-100·0)	100·0 (100·0-100·0)	100·0 (100·0-100·0)	100·0 (100·0-100·0)	79·1 (75·8-81·8)	80·5 (77·5-83·1)	80·7 (77·8-83·3)	83·5 (80·9-85·9)	74·6 (71·2-78·2)	74·5 (71·1-78·1)	74·5 (71·1-78·1)	74·5 (70·9-78·2)	80·9 (76·7-84·8)	88·3 (83·2-92·6)	88·7 (83·2-93·3)	96·9 (88·5-100·0)
Suriname	100·0 (100·0-100·0)	100·0 (100·0-100·0)	100·0 (100·0-100·0)	100·0 (100·0-100·0)	82·1 (79·3-84·8)	86·0 (83·4-88·4)	86·3 (83·8-88·7)	90·8 (88·4-92·8)	74·2 (70·6-78·1)	74·3 (70·6-78·2)	74·3 (70·6-78·1)	74·1 (70·2-78·4)	62·3 (57·2-67·6)	68·9 (61·7-75·8)	69·7 (61·5-77·4)	77·9 (58·7-93·7)
Trinidad and Tobago	100·0 (100·0-100·0)	100·0 (100·0-100·0)	100·0 (100·0-100·0)	100·0 (100·0-100·0)	84·9 (82·2-87·1)	85·5 (82·8-87·7)	85·8 (83·1-88·0)	89·7 (87·3-91·6)	74·7 (71·3-78·2)	74·0 (70·2-78·2)	74·0 (70·2-78·2)	73·7 (69·5-78·2)	91·4 (88·0-94·3)	95·4 (90·1-99·6)	95·5 (90·2-99·8)	96·8 (87·4-100·0)
**Tropical Latin America**																
Brazil	100·0 (100·0-100·0)	100·0 (100·0-100·0)	100·0 (100·0-100·0)	100·0 (100·0-100·0)	81·9 (80·8-83·1)	86·3 (85·4-87·4)	86·7 (85·7-87·7)	90·9 (90·1-91·9)	82·1 (78·0-86·3)	81·0 (76·1-85·9)	81·0 (76·1-85·9)	80·4 (74·6-85·9)	73·6 (72·4-74·8)	87·2 (85·9-88·6)	87·5 (86·1-89·0)	95·6 (93·8-97·3)
Paraguay	100·0 (100·0-100·0)	100·0 (100·0-100·0)	100·0 (100·0-100·0)	100·0 (100·0-100·0)	83·6 (81·9-85·1)	89·5 (88·0-90·8)	89·7 (88·3-91·1)	93·6 (92·0-95·0)	89·9 (86·9-92·9)	90·8 (87·7-93·6)	90·9 (87·9-93·8)	91·0 (88·0-93·9)	58·8 (54·4-63·1)	71·1 (64·2-76·8)	72·1 (64·7-78·2)	81·4 (67·0-91·3)
**East Asia**																
China	92·6 (87·6-97·9)	92·8 (87·9-98·0)	100·0 (100·0-100·0)	100·0 (100·0-100·0)	67·6 (66·2-69·1)	77·2 (76·1-78·4)	77·8 (76·6-78·9)	84·8 (83·7-85·8)	68·9 (62·2-75·5)	68·1 (61·1-74·8)	67·8 (60·6-74·7)	67·2 (59·1-74·5)	80·5 (79·2-81·7)	79·7 (78·3-81·0)	80·5 (79·0-82·0)	81·7 (78·4-84·8)
North Korea	100·0 (100·0-100·0)	100·0 (100·0-100·0)	100·0 (100·0-100·0)	100·0 (100·0-100·0)	61·4 (59·4-63·5)	58·8 (56·7-61·1)	58·9 (56·8-61·1)	59·9 (57·6-62·3)	78·8 (73·3-83·8)	79·6 (75·0-84·0)	79·6 (75·0-84·0)	79·4 (74·6-84·1)	0·0 (0·0-0·0)	0·0 (0·0-0·0)	0·0 (0·0-0·0)	0·0 (0·0-0·0)
Taiwan (Province of China)	100·0 (100·0-100·0)	100·0 (100·0-100·0)	100·0 (100·0-100·0)	100·0 (100·0-100·0)	80·9 (79·4-82·2)	86·4 (85·1-87·6)	86·5 (85·3-87·7)	88·8 (87·5-90·0)	84·4 (80·4-88·5)	84·5 (80·4-88·5)	84·6 (80·5-88·6)	84·9 (80·9-88·9)	40·4 (34·1-46·9)	88·2 (81·7-93·8)	88·1 (81·0-94·1)	95·6 (86·4-100·0)
**Southeast Asia**																
Cambodia	100·0 (100·0-100·0)	100·0 (100·0-100·0)	100·0 (100·0-100·0)	100·0 (100·0-100·0)	71·0 (68·2-73·5)	80·0 (77·7-82·2)	80·4 (78·1-82·6)	85·9 (83·7-88·1)	59·4 (51·6-66·7)	59·8 (51·4-66·8)	59·8 (51·4-66·8)	60·3 (51·3-67·4)	0·0 (0·0-0·0)	0·0 (0·0-0·0)	0·0 (0·0-0·0)	0·0 (0·0-0·0)
Indonesia	59·7 (55·0-63·6)	100·0 (100·0-100·0)	100·0 (100·0-100·0)	100·0 (100·0-100·0)	78·0 (76·4-79·5)	84·6 (83·1-86·0)	85·1 (83·6-86·4)	91·1 (89·7-92·3)	72·7 (67·8-77·4)	72·5 (67·6-77·4)	72·6 (67·7-77·4)	72·7 (67·8-77·5)	0·0 (0·0-0·0)	0·0 (0·0-0·0)	0·0 (0·0-0·0)	0·0 (0·0-0·0)
Laos	100·0 (100·0-100·0)	100·0 (100·0-100·0)	100·0 (100·0-100·0)	100·0 (100·0-100·0)	73·1 (68·3-77·8)	82·7 (78·7-86·5)	83·3 (79·4-87·0)	90·5 (87·1-93·5)	70·4 (64·6-76·0)	70·5 (64·7-76·1)	70·4 (64·7-76·1)	70·4 (64·5-76·1)	0·0 (0·0-0·0)	0·0 (0·0-0·0)	0·0 (0·0-0·0)	0·0 (0·0-0·0)
Malaysia	100·0 (100·0-100·0)	100·0 (100·0-100·0)	100·0 (100·0-100·0)	100·0 (100·0-100·0)	79·5 (75·5-83·1)	85·9 (82·4-89·1)	86·4 (82·9-89·5)	92·3 (89·2-95·1)	59·8 (52·4-67·0)	59·7 (52·3-66·8)	59·7 (52·3-66·8)	59·7 (52·3-66·8)	35·7 (33·2-38·5)	52·6 (48·7-56·6)	53·7 (49·5-57·8)	71·3 (63·6-77·7)
Maldives	100·0 (100·0-100·0)	100·0 (100·0-100·0)	100·0 (100·0-100·0)	100·0 (100·0-100·0)	76·6 (71·9-81·0)	86·5 (82·7-89·9)	87·0 (83·2-90·4)	93·9 (90·7-96·5)	70·2 (64·2-76·0)	70·1 (64·2-76·1)	70·1 (64·2-76·1)	69·7 (62·3-76·5)	37·6 (34·0-41·2)	62·4 (58·5-66·1)	63·4 (59·5-67·3)	81·4 (75·6-86·6)
Mauritius	100·0 (100·0-100·0)	100·0 (100·0-100·0)	100·0 (100·0-100·0)	100·0 (100·0-100·0)	83·3 (79·6-86·8)	86·0 (82·6-89·2)	86·4 (83·0-89·5)	91·0 (88·0-93·7)	70·4 (64·5-76·0)	70·4 (64·5-76·1)	70·3 (64·4-76·1)	70·5 (64·7-76·1)	84·2 (81·9-86·4)	90·6 (88·5-92·5)	90·9 (88·8-92·9)	95·0 (91·4-97·8)
Myanmar	47·3 (43·3-50·6)	45·5 (35·2-64·9)	100·0 (100·0-100·0)	100·0 (100·0-100·0)	73·6 (71·5-75·4)	86·0 (84·6-87·4)	86·6 (85·2-88·0)	94·1 (92·9-95·2)	70·3 (64·3-76·1)	70·2 (64·2-76·1)	70·2 (64·2-76·1)	70·2 (64·3-76·1)	0·0 (0·0-0·0)	0·0 (0·0-0·0)	0·0 (0·0-0·0)	0·0 (0·0-0·0)
Philippines	35·7 (32·0-38·8)	54·4 (50·3-57·7)	37·5 (31·3-45·0)	32·7 (16·3-68·1)	79·9 (77·7-81·9)	86·9 (85·1-88·6)	87·0 (85·2-88·7)	88·7 (86·6-90·7)	76·6 (70·0-83·0)	76·7 (70·4-83·0)	76·7 (70·4-82·9)	76·8 (70·4-82·9)	70·7 (68·2-73·4)	79·3 (76·3-82·2)	79·9 (76·9-82·8)	86·8 (81·6-91·1)
Sri Lanka	11·7 (3·7-24·8)	100·0 (100·0-100·0)	100·0 (100·0-100·0)	100·0 (100·0-100·0)	57·7 (54·8-60·5)	68·8 (66·3-71·3)	69·6 (67·2-72·0)	79·1 (77·0-81·0)	62·3 (54·4-68·9)	62·7 (55·3-69·1)	62·8 (55·4-69·2)	63·0 (55·9-69·4)	62·0 (59·1-64·8)	77·7 (74·8-80·7)	78·7 (75·8-81·9)	91·5 (87·3-95·2)
Seychelles	100·0 (100·0-100·0)	100·0 (100·0-100·0)	100·0 (100·0-100·0)	100·0 (100·0-100·0)	74·0 (69·1-78·4)	79·3 (75·1-83·2)	79·5 (75·3-83·4)	82·4 (78·6-85·9)	70·3 (64·3-76·1)	70·4 (64·5-76·2)	70·4 (64·5-76·2)	70·3 (64·3-76·2)	75·4 (72·2-78·4)	82·6 (80·1-85·2)	83·3 (80·6-85·8)	90·4 (85·9-94·0)
Thailand	100·0 (100·0-100·0)	100·0 (100·0-100·0)	100·0 (100·0-100·0)	100·0 (100·0-100·0)	69·1 (63·5-73·7)	80·4 (76·0-84·1)	80·8 (76·4-84·5)	85·8 (82·1-89·2)	86·5 (83·3-90·0)	86·6 (83·3-90·0)	86·6 (83·3-90·0)	86·5 (83·0-90·0)	39·8 (37·0-42·6)	58·3 (54·6-62·0)	59·0 (55·4-62·7)	70·6 (64·0-76·9)
Timor-Leste	14·8 (10·6-18·9)	100·0 (100·0-100·0)	100·0 (100·0-100·0)	100·0 (100·0-100·0)	41·4 (36·2-46·4)	55·1 (50·6-59·3)	56·4 (51·9-60·6)	71·9 (67·2-76·5)	58·7 (54·0-63·1)	58·5 (53·6-62·7)	58·4 (53·5-62·6)	57·5 (52·6-62·0)	0·0 (0·0-0·0)	0·0 (0·0-0·0)	0·0 (0·0-0·0)	0·0 (0·0-0·0)
Vietnam	100·0 (100·0-100·0)	100·0 (100·0-100·0)	100·0 (100·0-100·0)	100·0 (100·0-100·0)	79·1 (76·7-81·4)	85·6 (83·6-87·5)	85·8 (83·8-87·7)	88·8 (86·6-90·9)	75·5 (68·6-81·9)	75·4 (68·5-82·0)	75·3 (68·5-81·9)	75·3 (68·4-81·9)	0·0 (0·0-0·0)	0·0 (0·0-0·0)	0·0 (0·0-0·0)	0·0 (0·0-0·0)
**Oceania**																
Federated States of Micronesia	100·0 (100·0-100·0)	100·0 (100·0-100·0)	100·0 (100·0-100·0)	100·0 (100·0-100·0)	60·9 (58·2-63·5)	68·3 (65·9-70·7)	68·7 (66·3-71·0)	74·0 (71·6-76·2)	22·5 (8·8-35·7)	20·1 (5·2-33·8)	20·2 (5·3-33·8)	18·6 (3·2-32·7)	0·0 (0·0-0·0)	0·0 (0·0-0·0)	0·0 (0·0-0·0)	0·0 (0·0-0·0)
Fiji	100·0 (100·0-100·0)	100·0 (100·0-100·0)	100·0 (100·0-100·0)	100·0 (100·0-100·0)	71·8 (69·8-73·9)	75·1 (73·0-77·0)	75·2 (73·1-77·2)	77·1 (74·9-79·1)	21·5 (7·1-34·9)	20·9 (6·2-34·4)	20·9 (6·2-34·4)	20·6 (6·1-34·3)	46·7 (40·3-53·1)	68·4 (58·5-77·6)	68·7 (57·7-79·1)	80·6 (61·3-94·5)

[redacted]

Location	SDG Index: Geometric mean of all health-related SDG indicators (scale of 0 to 100)				MDG Index: Geometric mean of all health-related SDG indicators with corresponding MDG indicators (scale of 0 to 100)				Non-MDG Index: Geometric mean of all health-related indicators without corresponding MDG indicators (scale of 0 to 100)				Indicator 1.5.1: Death rate due to exposure to forces of nature (per 100,000 population)			
	2000	2015	2016	2030	2000	2015	2016	2030	2000	2015	2016	2030	2000	2015	2016	2030
Kiribati	28·0 (25·7-30·0)	34·3 (29·9-38·9)	35·2 (30·6-40·2)	43·0 (35·2-49·8)	29·8 (26·0-34·1)	40·3 (35·0-44·9)	41·2 (36·0-46·0)	49·5 (37·3-56·3)	28·9 (23·1-32·2)	33·0 (25·4-40·0)	33·7 (25·9-40·8)	39·8 (29·8-51·2)	100·0 (100·0-100·0)	100·0 (100·0-100·0)	100·0 (100·0-100·0)	100·0 (100·0-100·0)
Marshall Islands	30·6 (27·9-33·0)	37·7 (34·1-41·5)	38·3 (34·7-42·4)	44·8 (39·0-51·0)	33·5 (29·9-37·1)	48·7 (44·5-52·8)	49·6 (45·4-53·9)	60·3 (52·7-66·3)	32·4 (25·6-36·0)	35·3 (27·8-40·3)	35·7 (28·4-41·2)	39·2 (30·5-48·4)	100·0 (100·0-100·0)	100·0 (100·0-100·0)	100·0 (100·0-100·0)	100·0 (100·0-100·0)
Papua New Guinea	13·0 (10·9-15·5)	21·8 (18·9-26·4)	22·5 (19·3-27·1)	29·4 (23·2-35·0)	13·5 (10·1-17·2)	22·3 (17·5-26·7)	23·3 (18·4-27·7)	33·0 (26·2-38·7)	14·6 (12·4-17·4)	23·4 (20·0-29·2)	24·0 (20·4-30·0)	29·0 (20·6-36·1)	0·0 (0·0-0·0)	41·1 (37·0-45·0)	46·2 (41·8-50·2)	25·5 (22·0-28·8)
Samoa	37·2 (34·3-39·8)	34·5 (31·6-37·1)	35·1 (32·0-38·1)	40·1 (35·8-44·0)	56·9 (52·5-61·4)	62·8 (58·1-67·5)	63·4 (58·7-68·1)	70·4 (64·0-76·0)	32·0 (26·3-36·7)	26·9 (22·6-31·8)	27·4 (23·0-32·5)	30·8 (25·4-38·1)	48·0 (43·6-52·3)	0·0 (0·0-0·0)	0·5 (0·0-2·7)	0·5 (0·0-2·6)
Solomon Islands	27·2 (24·6-30·3)	28·1 (25·2-31·0)	28·9 (26·0-31·9)	34·1 (29·3-38·6)	26·8 (23·2-30·6)	36·6 (32·3-40·8)	37·6 (33·2-41·7)	49·0 (43·6-54·1)	29·1 (25·2-32·9)	26·8 (21·4-30·3)	27·4 (21·8-31·1)	29·7 (22·4-36·0)	100·0 (100·0-100·0)	10·7 (7·8-13·5)	13·6 (10·6-16·5)	15·3 (12·3-18·2)
Tonga	49·1 (45·5-52·4)	56·1 (52·3-60·2)	56·5 (52·7-60·6)	61·5 (56·4-67·2)	51·3 (46·9-55·9)	59·6 (55·0-64·1)	60·1 (55·5-64·5)	66·9 (60·9-72·3)	44·3 (36·7-50·9)	49·0 (42·6-58·3)	49·3 (42·8-58·6)	52·8 (45·6-65·3)	100·0 (100·0-100·0)	100·0 (100·0-100·0)	100·0 (100·0-100·0)	100·0 (100·0-100·0)
Vanuatu	19·6 (17·6-22·7)	28·6 (24·1-32·8)	29·2 (24·6-33·5)	34·6 (29·4-39·2)	27·0 (22·7-30·9)	37·5 (33·7-41·7)	38·3 (34·6-42·6)	47·4 (42·5-52·7)	19·5 (17·4-23·1)	27·6 (22·7-32·3)	28·1 (23·0-32·8)	31·9 (24·0-37·3)	0·0 (0·0-0·0)	16·5 (13·2-19·8)	18·0 (14·7-21·3)	15·0 (11·8-18·1)
**North Africa and Middle East**																
Afghanistan	4·7 (4·2-5·5)	10·4 (9·3-11·5)	10·9 (9·6-11·9)	16·8 (14·4-19·3)	4·5 (3·4-5·9)	12·8 (9·4-16·7)	13·9 (10·0-17·6)	27·9 (22·2-32·7)	6·6 (6·0-7·7)	11·6 (11·0-12·2)	11·8 (11·2-12·5)	15·1 (11·3-18·4)	1·2 (0·0-10·8)	21·7 (16·0-28·3)	24·3 (18·4-30·9)	17·2 (10·6-25·4)
Algeria	44·8 (42·5-46·8)	57·0 (53·7-60·5)	57·9 (54·5-61·4)	65·2 (58·9-70·3)	39·7 (38·4-41·1)	55·5 (50·7-62·4)	56·6 (51·5-63·5)	70·5 (58·7-77·3)	51·4 (48·1-54·5)	60·2 (55·7-63·4)	60·8 (55·9-64·1)	60·0 (49·6-70·1)	56·5 (48·8-66·6)	60·5 (53·2-68·4)	64·4 (56·8-72·7)	38·4 (30·3-48·9)
Bahrain	57·6 (55·5-59·5)	63·9 (59·6-66·9)	64·1 (59·0-67·2)	69·0 (62·3-73·9)	68·7 (66·8-70·4)	78·7 (76·8-80·6)	79·1 (77·2-81·1)	84·9 (81·5-88·0)	55·2 (52·4-57·7)	60·0 (54·5-63·7)	60·0 (53·9-63·8)	63·2 (50·3-69·9)	100·0 (100·0-100·0)	100·0 (100·0-100·0)	100·0 (100·0-100·0)	100·0 (100·0-100·0)
Egypt	48·4 (46·6-50·0)	48·9 (47·2-50·6)	49·8 (47·8-51·7)	54·8 (50·9-58·1)	49·7 (45·8-52·2)	63·4 (60·2-65·9)	64·3 (61·0-66·8)	73·4 (69·3-77·0)	49·2 (46·9-51·4)	46·0 (43·9-47·9)	46·7 (44·4-48·9)	50·5 (46·0-54·2)	51·9 (43·5-62·3)	84·7 (76·8-94·3)	88·1 (81·0-95·9)	79·3 (72·6-85·8)
Iran	45·3 (43·6-47·0)	61·5 (58·1-64·2)	62·3 (58·9-65·0)	67·1 (61·5-71·9)	60·9 (57·2-63·7)	71·9 (68·7-74·6)	72·6 (69·6-75·3)	81·6 (75·8-86·5)	43·1 (40·9-45·2)	59·5 (55·3-62·9)	60·2 (55·8-63·7)	62·4 (52·2-68·9)	27·4 (18·3-41·5)	52·0 (44·0-62·2)	55·4 (46·8-66·2)	14·0 (5·2-29·0)
Iraq	37·4 (34·8-41·0)	35·9 (33·1-38·7)	36·3 (33·4-39·1)	40·3 (35·1-45·7)	43·1 (40·8-45·5)	46·9 (41·2-51·9)	47·5 (41·7-52·9)	54·5 (47·8-64·3)	39·1 (35·9-43·7)	35·1 (32·1-37·8)	35·5 (32·4-38·3)	36·8 (28·6-43·9)	93·2 (88·1-98·1)	69·4 (58·4-85·0)	70·6 (59·7-85·9)	76·4 (66·2-89·7)
Jordan	60·5 (58·2-62·5)	66·8 (64·5-69·0)	65·1 (62·4-67·7)	69·8 (64·8-73·8)	62·3 (60·5-63·9)	72·7 (70·6-74·6)	73·2 (71·0-75·1)	80·7 (77·1-83·8)	63·2 (60·5-65·7)	66·7 (63·9-69·3)	64·2 (61·0-67·3)	66·9 (59·3-72·0)	100·0 (100·0-100·0)	100·0 (100·0-100·0)	100·0 (100·0-100·0)	100·0 (100·0-100·0)
Kuwait	59·9 (56·6-62·3)	58·7 (56·3-62·6)	59·3 (56·6-63·2)	64·0 (59·1-69·2)	73·9 (72·0-75·5)	83·0 (81·0-85·0)	83·8 (81·7-86·1)	90·8 (86·1-94·4)	53·4 (44·4-58·7)	45·9 (42·1-54·1)	46·3 (42·2-54·6)	48·6 (43·5-59·5)	80·1 (72·2-92·3)	100·0 (100·0-100·0)	100·0 (100·0-100·0)	97·5 (95·5-99·4)
Lebanon	50·3 (48·2-52·0)	55·6 (50·8-58·0)	56·1 (52·3-58·5)	58·2 (50·6-64·3)	62·9 (60·7-65·2)	75·9 (72·6-78·8)	76·6 (73·3-79·4)	87·5 (81·2-92·2)	47·7 (44·6-50·2)	50·5 (44·8-53·4)	50·9 (46·3-53·8)	49·8 (40·3-57·6)	68·2 (58·3-83·4)	100·0 (100·0-100·0)	100·0 (100·0-100·0)	95·7 (92·0-98·9)
Libya	52·4 (49·4-55·1)	54·8 (51·2-58·4)	55·5 (51·6-59·3)	58·4 (53·2-64·2)	63·0 (61·1-65·0)	74·4 (72·2-76·3)	75·0 (72·9-77·1)	81·3 (77·4-84·5)	49·0 (40·5-53·3)	46·2 (38·7-52·5)	46·9 (39·0-53·3)	46·6 (39·1-57·8)	100·0 (100·0-100·0)	66·1 (56·7-81·7)	67·8 (58·3-83·0)	72·8 (63·7-86·9)
Morocco	46·7 (44·7-48·7)	56·2 (53·8-58·5)	56·9 (54·3-59·3)	63·4 (58·0-68·2)	42·4 (40·7-44·0)	59·5 (56·9-61·8)	60·4 (57·9-62·7)	71·8 (68·5-74·7)	52·0 (49·2-54·8)	56·1 (50·7-59·7)	56·5 (51·0-60·6)	57·7 (46·7-67·5)	38·9 (29·3-54·9)	64·0 (55·6-74·1)	65·8 (57·6-76·2)	50·7 (42·4-61·9)
Palestine	57·1 (54·9-59·1)	52·8 (49·6-55·5)	53·2 (50·1-55·9)	55·3 (49·9-59·9)	61·1 (57·8-64·7)	58·8 (55·7-62·3)	59·6 (56·2-63·3)	64·1 (53·7-71·9)	58·9 (56·4-61·3)	54·1 (50·3-57·1)	54·3 (50·7-57·5)	55·0 (49·0-59·9)	100·0 (100·0-100·0)	100·0 (100·0-100·0)	100·0 (100·0-100·0)	100·0 (100·0-100·0)
Oman	45·0 (42·4-47·2)	52·0 (49·1-56·0)	52·7 (49·6-57·3)	61·5 (55·6-65·5)	53·9 (47·1-59·2)	77·5 (74·8-80·0)	78·3 (75·6-80·7)	88·8 (85·5-91·6)	44·4 (42·1-46·3)	41·7 (35·7-48·7)	42·2 (36·0-49·4)	45·6 (39·6-54·4)	100·0 (100·0-100·0)	44·2 (36·6-54·7)	49·8 (42·2-60·0)	42·2 (35·2-52·5)
Qatar	45·7 (40·9-50·7)	53·5 (49·2-57·1)	53·5 (48·3-57·4)	56·4 (46·4-62·8)	69·6 (67·1-71·9)	81·0 (78·6-83·4)	81·3 (78·8-83·7)	87·7 (82·6-91·8)	39·7 (32·6-46·1)	42·8 (35·9-49·6)	42·6 (35·4-49·7)	42·6 (32·1-54·4)	100·0 (100·0-100·0)	100·0 (100·0-100·0)	100·0 (100·0-100·0)	100·0 (100·0-100·0)
Saudi Arabia	45·6 (43·2-48·8)	52·4 (49·8-54·9)	54·1 (51·5-56·7)	60·3 (57·9-64·0)	58·3 (56·0-60·5)	79·8 (77·6-81·8)	81·1 (78·8-83·2)	93·1 (91·3-94·6)	42·5 (37·4-46·9)	41·1 (36·2-47·2)	42·2 (37·5-48·9)	44·0 (41·1-53·7)	100·0 (100·0-100·0)	66·5 (59·5-75·1)	69·2 (62·3-77·5)	67·2 (60·4-74·8)
Sudan	17·8 (16·3-19·8)	27·6 (25·1-29·7)	28·1 (25·6-30·1)	34·9 (31·4-37·9)	11·2 (8·7-14·1)	24·0 (18·9-27·5)	25·1 (19·9-28·8)	37·1 (27·5-42·2)	24·1 (22·4-27·2)	31·3 (28·0-33·6)	31·3 (28·1-33·6)	35·7 (31·4-38·9)	56·0 (47·2-66·3)	59·1 (50·4-69·5)	52·8 (44·7-62·4)	54·8 (46·9-63·8)
Syria	53·4 (52·0-54·8)	60·3 (58·0-62·2)	60·9 (58·6-62·8)	64·3 (60·3-68·2)	55·8 (54·4-57·3)	67·8 (65·1-70·3)	68·5 (65·8-71·1)	76·8 (71·9-81·4)	54·6 (51·9-56·7)	57·7 (51·0-60·9)	58·1 (51·5-61·4)	56·4 (47·6-65·2)	52·5 (45·3-60·9)	93·6 (89·1-98·2)	100·0 (100·0-100·0)	68·9 (61·5-76·1)
Tunisia	57·5 (55·1-59·7)	62·1 (59·4-64·8)	63·0 (60·3-65·8)	68·2 (62·6-73·0)	63·9 (62·0-65·6)	72·4 (70·1-74·3)	73·1 (70·8-75·0)	81·6 (78·5-84·1)	57·3 (54·1-60·3)	59·3 (55·3-63·0)	60·1 (55·9-64·0)	61·3 (48·7-69·6)	100·0 (100·0-100·0)	75·1 (66·7-86·6)	79·4 (71·3-89·7)	75·5 (67·2-86·7)
Turkey	40·1 (36·0-44·9)	64·4 (62·3-66·4)	64·8 (62·5-66·9)	68·1 (62·7-73·3)	50·9 (48·9-52·7)	70·3 (67·4-72·6)	71·3 (68·5-73·7)	82·8 (78·4-86·5)	39·6 (34·3-45·8)	63·1 (59·6-66·0)	63·1 (59·5-66·3)	60·3 (48·7-69·2)	3·5 (0·0-20·3)	54·4 (44·3-71·5)	56·7 (46·6-73·6)	32·7 (22·7-50·4)
United Arab Emirates	49·7 (45·2-53·9)	46·1 (42·8-52·0)	45·9 (42·7-51·3)	48·3 (42·7-56·3)	66·4 (63·1-69·5)	73·7 (70·7-76·4)	74·0 (70·9-76·7)	79·2 (74·0-83·1)	46·5 (41·1-51·6)	39·3 (34·2-45·9)	39·0 (33·4-45·2)	38·8 (30·2-49·1)	100·0 (100·0-100·0)	100·0 (100·0-100·0)	100·0 (100·0-100·0)	100·0 (100·0-100·0)
Yemen	13·1 (11·7-14·4)	22·9 (20·3-25·6)	23·5 (20·7-26·2)	30·8 (26·8-33·9)	9·1 (7·5-10·9)	25·9 (22·1-28·9)	26·7 (22·8-29·8)	36·0 (30·9-41·8)	18·1 (15·9-19·7)	24·5 (21·4-27·8)	25·0 (21·7-28·3)	31·1 (25·9-34·7)	38·0 (29·7-50·1)	59·5 (51·2-69·1)	64·3 (56·2-73·4)	52·0 (44·4-59·9)
**South Asia**																

Location	Indicator 2.2.1: Prevalence of stunting in children under 5 (%)				Indicator 2.2.2a: Prevalence of wasting in children under 5 (%)				Indicator 2.2.2b: Prevalence of overweight in children aged 2-4 (%)				Indicator 3.1.1: Maternal mortality ratio (maternal deaths per 100,000 livebirths) in women aged 10-54 years			
	**2000**	**2015**	**2016**	**2030**	**2000**	**2015**	**2016**	**2030**	**2000**	**2015**	**2016**	**2030**	**2000**	**2015**	**2016**	**2030**
Kiribati	54·3 (44·1-64·8)	66·3 (57·1-74·8)	66·9 (57·9-75·4)	75·2 (67·2-82·7)	48·5 (30·6-63·8)	55·4 (38·0-69·9)	55·8 (38·3-70·4)	61·3 (44·1-75·2)	38·6 (0·0-82·2)	29·1 (0·0-75·4)	28·5 (0·0-75·7)	25·7 (0·0-92·7)	24·4 (19·6-29·4)	25·5 (18·8-32·4)	25·8 (18·9-32·9)	28·8 (15·3-42·3)
Marshall Islands	70·8 (61·1-79·2)	76·7 (68·0-83·8)	77·4 (68·9-84·3)	83·8 (75·4-89·9)	73·8 (62·4-82·4)	72·1 (60·1-81·5)	72·2 (60·3-81·6)	74·4 (62·6-83·5)	48·2 (0·0-86·5)	36·9 (0·0-79·2)	36·1 (0·0-78·6)	30·6 (0·0-92·5)	32·6 (26·3-39·1)	33·4 (25·2-41·5)	34·2 (26·2-42·1)	41·4 (25·3-58·3)
Papua New Guinea	5·0 (0·0-16·7)	13·0 (0·0-25·0)	14·1 (0·0-26·6)	29·1 (2·2-44·0)	27·1 (12·9-39·8)	36·1 (20·3-49·9)	38·5 (21·8-52·7)	32·7 (23·2-49·0)	80·2 (54·1-96·3)	71·8 (29·7-98·5)	70·9 (27·9-97·9)	62·0 (0·0-100·0)	7·8 (2·0-13·7)	9·9 (3·8-15·9)	10·6 (4·3-16·6)	16·7 (4·7-28·9)
Samoa	87·0 (82·7-90·6)	89·0 (84·5-92·8)	89·2 (84·8-92·9)	91·5 (88·3-94·8)	90·9 (86·4-94·8)	91·2 (86·2-95·5)	91·3 (86·2-95·5)	92·1 (86·9-96·4)	23·4 (0·0-69·8)	15·0 (0·0-59·9)	14·7 (0·0-60·9)	17·1 (0·0-81·7)	51·0 (44·6-57·9)	56·7 (50·2-63·9)	57·5 (51·1-64·7)	63·5 (47·9-79·4)
Solomon Islands	48·8 (37·2-59·5)	59·5 (49·4-69·0)	60·7 (50·6-70·3)	75·4 (63·8-84·5)	74·1 (65·8-80·6)	77·3 (68·3-83·7)	77·2 (68·0-83·9)	78·5 (66·1-86·8)	58·0 (11·2-91·7)	48·5 (0·0-87·1)	47·8 (0·0-86·9)	39·6 (0·0-96·3)	24·7 (17·2-31·4)	23·3 (15·3-30·3)	23·8 (15·8-31·0)	28·4 (14·1-42·7)
Tonga	83·1 (76·6-88·3)	86·9 (82·1-90·8)	87·2 (82·2-91·2)	90·7 (86·6-93·8)	65·3 (53·3-75·0)	73·5 (68·6-78·0)	73·6 (68·5-78·5)	80·7 (78·8-83·4)	22·5 (0·0-68·3)	12·5 (0·0-55·7)	12·2 (0·0-55·5)	14·0 (0·0-78·9)	25·0 (19·4-30·8)	28·9 (22·6-36·3)	29·1 (22·7-36·3)	31·5 (18·6-45·5)
Vanuatu	57·1 (47·9-66·8)	58·9 (51·2-67·2)	60·0 (52·0-68·4)	63·0 (53·6-67·5)	68·4 (62·6-74·2)	77·3 (72·7-81·7)	77·8 (73·1-82·2)	84·1 (78·6-87·5)	80·9 (46·8-100·0)	71·7 (27·5-96·4)	71·4 (28·3-96·4)	64·1 (0·0-100·0)	28·6 (21·8-36·3)	28·7 (20·8-36·6)	29·0 (20·8-36·6)	31·5 (18·0-45·7)
**North Africa and Middle East**																
Afghanistan	0·0 (0·0-0·0)	4·1 (0·0-15·7)	5·0 (0·0-17·2)	21·6 (0·1-38·3)	53·9 (48·4-60·3)	52·9 (42·7-63·5)	52·6 (42·3-63·6)	49·9 (35·8-64·0)	77·6 (55·0-93·8)	63·2 (32·8-86·4)	62·2 (32·4-85·3)	47·2 (0·0-92·6)	3·6 (0·0-8·3)	7·7 (2·3-13·2)	8·3 (2·9-13·6)	16·7 (5·2-28·4)
Algeria	38·4 (32·3-44·0)	80·4 (74·4-85·9)	81·2 (75·4-86·6)	89·8 (85·6-93·7)	43·0 (37·3-48·6)	73·9 (67·3-79·6)	75·1 (68·4-80·7)	87·5 (82·1-91·7)	78·8 (58·2-94·5)	49·6 (18·8-76·5)	48·2 (16·3-75·3)	21·1 (0·0-71·5)	35·1 (31·3-39·0)	43·6 (39·1-48·2)	44·7 (39·9-49·7)	53·9 (42·9-65·3)
Bahrain	85·5 (80·4-90·1)	96·4 (94·6-98·0)	96·5 (94·7-98·0)	99·7 (97·7-100·0)	74·3 (69·2-79·2)	88·7 (85·3-92·2)	89·0 (85·5-92·5)	94·9 (92·0-97·2)	72·7 (48·1-89·3)	72·6 (51·9-90·0)	71·3 (49·1-89·7)	58·3 (0·0-96·2)	57·8 (52·9-62·6)	65·4 (58·6-72·0)	65·2 (58·0-72·3)	73·8 (55·1-92·3)
Egypt	52·6 (46·6-58·5)	63·2 (56·8-69·9)	65·0 (58·7-71·5)	69·1 (62·2-75·9)	70·4 (67·2-73·5)	65·7 (61·2-70·2)	66·2 (61·6-70·8)	61·3 (54·7-67·8)	47·2 (32·8-60·6)	59·7 (42·2-74·0)	59·0 (40·2-74·5)	67·4 (35·7-89·4)	39·3 (35·5-43·7)	50·9 (45·5-56·5)	51·4 (45·4-57·5)	58·8 (44·5-73·0)
Iran	67·9 (62·7-72·8)	87·1 (82·5-91·0)	87·3 (82·7-91·1)	94·0 (89·9-96·5)	73·4 (70·2-76·4)	77·4 (68·9-84·1)	77·2 (68·2-84·2)	78·3 (59·8-89·7)	81·8 (61·5-96·2)	68·2 (40·5-87·8)	67·7 (40·5-87·3)	56·8 (4·3-93·0)	58·8 (53·1-64·9)	73·5 (67·3-79·7)	73·7 (67·1-80·5)	86·2 (69·0-100·0)
Iraq	48·4 (43·6-53·5)	55·9 (48·3-64·0)	56·6 (48·8-64·9)	63·9 (52·9-74·9)	46·9 (41·4-53·2)	63·9 (56·3-71·1)	64·3 (56·5-71·7)	70·0 (59·6-78·9)	76·3 (55·2-91·2)	48·7 (30·7-64·8)	47·7 (25·1-66·6)	28·3 (0·0-73·9)	37·6 (31·6-43·5)	48·5 (38·6-57·8)	49·0 (39·1-58·4)	59·8 (41·1-77·5)
Jordan	79·9 (75·1-84·5)	86·2 (81·5-90·2)	86·7 (81·8-90·8)	92·2 (86·0-96·4)	83·2 (78·0-87·1)	84·6 (79·3-88·8)	85·1 (79·7-89·4)	90·4 (84·9-93·8)	86·3 (75·1-95·3)	78·2 (59·9-91·4)	77·3 (57·0-91·3)	67·7 (17·3-97·4)	43·8 (37·0-50·7)	55·8 (47·3-63·3)	56·0 (47·4-63·4)	59·6 (39·9-77·8)
Kuwait	92·1 (88·5-94·8)	96·3 (94·2-97·8)	96·4 (94·4-97·9)	98·2 (97·0-99·3)	86·3 (83·2-89·3)	92·3 (88·6-95·4)	92·4 (88·6-95·6)	95·0 (90·7-98·2)	23·1 (0·0-52·8)	4·6 (0·0-27·5)	4·5 (0·0-28·0)	2·9 (0·0-30·8)	78·2 (73·6-83·1)	80·2 (72·8-87·9)	80·4 (71·6-90·0)	85·4 (56·0-100·0)
Lebanon	68·1 (60·3-75·5)	78·6 (72·1-84·5)	79·3 (72·7-85·1)	86·9 (80·3-92·4)	77·1 (70·8-82·2)	80·0 (74·1-84·9)	80·2 (74·1-85·2)	82·8 (75·5-89·1)	42·6 (18·0-64·5)	57·0 (33·9-76·6)	55·1 (30·2-76·8)	51·4 (0·0-90·4)	59·5 (53·1-65·3)	74·2 (67·4-80·8)	74·5 (67·4-81·4)	87·2 (69·8-100·0)
Libya	65·7 (57·8-74·1)	73·4 (65·5-80·8)	74·2 (66·2-81·6)	83·0 (74·0-90·6)	79·5 (75·7-83·1)	75·9 (67·9-82·3)	76·1 (68·0-82·7)	75·4 (64·2-83·4)	36·2 (0·0-67·1)	16·3 (0·0-48·4)	16·9 (0·0-49·1)	10·5 (0·0-55·1)	63·1 (56·9-69·4)	63·1 (56·3-70·2)	62·7 (55·2-70·4)	61·3 (43·3-78·0)
Morocco	55·7 (47·6-64·0)	69·6 (61·4-77·9)	70·4 (61·8-79·0)	80·2 (72·6-86·9)	64·1 (56·3-70·6)	79·0 (72·4-84·0)	79·6 (73·1-84·5)	86·5 (80·6-90·8)	63·4 (46·1-77·3)	42·9 (9·2-72·3)	41·7 (8·1-71·7)	26·0 (0·0-76·2)	25·2 (21·0-29·9)	38·8 (32·6-45·1)	39·0 (32·9-45·3)	43·9 (30·6-57·4)
Palestine	80·1 (76·2-83·8)	84·1 (79·1-88·3)	85·2 (80·1-89·3)	95·4 (91·5-98·0)	77·0 (72·4-81·2)	86·6 (82·2-90·7)	87·8 (83·5-91·8)	94·3 (90·8-97·2)	73·6 (61·2-84·1)	74·1 (59·2-86·1)	72·9 (56·6-86·4)	63·4 (22·0-91·6)	78·6 (74·3-83·1)	69·8 (65·4-74·4)	69·3 (64·7-74·3)	66·5 (51·4-82·7)
Oman	76·2 (71·2-81·1)	80·5 (75·9-85·2)	80·5 (75·7-85·2)	85·6 (80·0-88·2)	54·8 (48·2-61·1)	62·5 (56·7-67·9)	62·5 (56·7-68·0)	69·0 (65·3-72·2)	63·5 (36·4-85·0)	13·8 (0·0-46·3)	13·2 (0·0-45·9)	1·5 (0·0-20·3)	71·1 (64·8-77·6)	81·5 (74·3-88·0)	81·6 (74·4-88·1)	86·0 (70·9-100·0)
Qatar	83·9 (78·2-88·5)	88·1 (83·3-91·8)	88·3 (83·6-92·0)	91·6 (87·3-94·4)	87·7 (82·5-91·9)	88·9 (83·5-93·0)	89·0 (83·6-93·1)	90·3 (85·3-94·4)	34·0 (1·2-63·5)	12·7 (0·0-43·7)	12·2 (0·0-43·7)	7·4 (0·0-51·3)	49·8 (42·2-56·8)	65·6 (57·6-74·3)	65·3 (55·7-74·8)	78·4 (55·3-100·0)
Saudi Arabia	74·6 (72·1-77·3)	89·1 (87·4-90·6)	89·6 (88·0-91·0)	96·1 (95·1-96·9)	72·9 (68·9-76·5)	78·8 (75·0-82·0)	79·4 (75·7-82·6)	86·7 (83·7-89·0)	41·1 (5·8-70·7)	10·3 (0·0-41·1)	8·9 (0·0-38·2)	2·8 (0·0-32·1)	72·8 (69·0-76·6)	79·9 (75·3-84·4)	80·6 (75·7-85·3)	89·5 (78·0-100·0)
Sudan	17·2 (8·1-25·0)	40·4 (30·8-49·6)	41·7 (32·1-50·9)	57·4 (48·6-66·9)	2·9 (0·0-13·6)	26·9 (11·7-41·8)	28·5 (13·2-43·5)	46·9 (31·6-62·8)	89·4 (74·9-99·2)	85·9 (69·4-96·4)	85·1 (67·0-96·5)	81·5 (42·7-100·0)	8·7 (5·3-12·2)	13·3 (9·0-17·3)	13·8 (9·4-18·0)	19·1 (10·3-27·7)
Syria	42·1 (36·9-47·2)	56·4 (47·7-65·9)	58·4 (49·3-68·3)	66·4 (57·9-75·1)	54·1 (46·9-61·2)	52·8 (41·0-63·3)	53·4 (41·1-64·2)	61·8 (41·9-73·6)	57·7 (29·6-80·4)	37·1 (4·5-66·0)	36·8 (4·1-66·4)	21·0 (0·0-72·5)	55·8 (52·1-59·6)	72·1 (66·5-77·7)	72·3 (66·8-78·1)	76·9 (63·2-90·7)
Tunisia	72·9 (68·2-77·4)	85·4 (80·3-89·8)	85·8 (80·6-90·2)	91·8 (87·6-95·1)	77·4 (72·5-82·0)	84·7 (80·0-89·2)	84·9 (80·2-89·4)	89·0 (84·2-93·4)	72·2 (45·2-89·8)	54·1 (22·3-80·4)	53·0 (19·9-79·4)	35·3 (0·0-83·3)	48·2 (43·1-53·1)	58·2 (51·5-65·5)	58·6 (51·7-66·1)	67·4 (54·2-82·3)
Turkey	70·8 (64·7-77·0)	83·1 (77·2-88·3)	83·6 (77·6-88·7)	90·1 (84·5-94·4)	90·2 (86·3-93·3)	94·1 (89·7-97·5)	94·2 (89·8-97·6)	96·0 (91·7-99·3)	75·0 (61·0-85·9)	52·0 (22·8-76·4)	50·7 (20·4-75·1)	28·8 (0·0-76·0)	49·2 (43·8-54·6)	71·0 (64·4-77·0)	71·3 (64·2-78·1)	76·8 (59·2-94·2)
United Arab Emirates	73·2 (65·8-79·5)	81·4 (74·5-86·9)	81·7 (74·7-87·1)	85·6 (79·8-90·5)	36·5 (24·5-48·2)	60·3 (47·2-71·9)	61·0 (47·5-72·5)	74·0 (62·5-83·3)	63·2 (39·6-81·4)	40·2 (4·3-68·6)	39·4 (4·8-68·1)	25·3 (0·0-73·5)	76·2 (68·9-83·6)	80·0 (70·9-88·9)	79·9 (70·7-88·8)	74·7 (53·8-95·3)
Yemen	1·5 (0·0-9·8)	17·1 (2·0-28·7)	19·4 (3·6-31·4)	34·7 (19·4-42·9)	26·8 (14·9-37·8)	14·4 (4·1-24·2)	13·1 (2·6-23·0)	1·3 (0·0-9·7)	77·0 (55·3-92·4)	71·9 (46·9-91·3)	71·3 (46·1-91·0)	70·0 (24·0-99·5)	12·5 (9·1-15·8)	22·1 (17·1-27·1)	22·0 (16·9-27·3)	28·8 (18·4-39·3)
**South Asia**																

Location	Indicator 3.1.2: Proportion of births attended by skilled health personnel (%)				Indicator 3.2.1: Under-5 mortality rate (probability of dying before the age of 5 per 1,000 livebirths)				Indicator 3.2.2: Neonatal mortality rate (probability of dying during the first 28 days of life per 1,000 livebirths)				Indicator 3.3.1: Age-standardised rate of new HIV infections (per 1,000 population)			
	2000	2015	2016	2030	2000	2015	2016	2030	2000	2015	2016	2030	2000	2015	2016	2030
Kiribati	80·9 (68·9-89·6)	87·3 (78·3-93·3)	87·5 (78·1-93·6)	91·5 (70·4-100·0)	22·7 (17·3-28·1)	29·5 (16·6-40·9)	30·5 (17·5-42·8)	39·4 (10·8-66·2)	18·1 (9·4-27·5)	23·5 (6·8-38·4)	24·5 (7·7-39·7)	33·5 (0·0-65·8)	85·9 (80·7-90·5)	89·0 (81·7-94·0)	88·9 (79·2-94·9)	87·4 (77·4-93·9)
Marshall Islands	89·3 (83·2-93·9)	94·2 (89·9-97·2)	94·6 (90·3-97·5)	97·6 (89·9-100·0)	34·4 (23·5-45·5)	47·5 (34·4-60·6)	48·7 (35·4-61·9)	65·5 (32·3-100·0)	30·2 (16·7-44·7)	40·2 (24·3-56·3)	41·4 (26·0-57·5)	58·0 (19·3-100·0)	79·4 (49·6-99·6)	63·8 (34·4-86·6)	62·3 (32·8-85·3)	62·2 (32·7-85·7)
Papua New Guinea	40·6 (31·4-50·1)	56·0 (35·4-72·9)	57·9 (36·7-75·0)	76·7 (35·7-100·0)	20·0 (12·6-26·9)	28·6 (17·0-39·2)	29·6 (18·2-40·4)	42·9 (21·5-63·1)	15·0 (7·6-22·9)	22·3 (6·8-36·2)	23·2 (7·7-37·1)	35·7 (9·0-60·8)	38·9 (31·3-46·9)	43·6 (31·6-54·6)	43·5 (29·5-55·1)	42·5 (29·0-54·0)
Samoa	89·6 (82·6-94·4)	87·4 (78·6-93·6)	88·0 (78·8-94·1)	93·8 (73·7-100·0)	56·7 (42·8-70·3)	65·3 (50·9-77·9)	66·6 (52·2-79·7)	78·8 (53·7-100·0)	51·0 (35·9-67·0)	62·0 (43·3-77·7)	63·4 (44·4-79·6)	76·8 (42·5-100·0)	79·2 (49·5-99·9)	63·3 (33·7-85·9)	61·7 (31·9-84·6)	61·0 (31·3-84·2)
Solomon Islands	81·6 (72·8-88·6)	93·4 (88·1-96·9)	93·7 (88·5-97·1)	97·5 (91·8-99·9)	36·7 (30·4-43·5)	43·9 (35·8-50·8)	44·8 (36·8-52·0)	57·1 (38·9-73·4)	29·6 (19·9-39·6)	35·3 (23·6-45·6)	36·2 (24·2-46·5)	46·7 (23·4-68·2)	79·0 (49·6-99·5)	64·3 (34·9-87·3)	62·7 (33·2-85·9)	61·0 (31·7-83·9)
Tonga	92·3 (89·0-94·8)	98·4 (96·8-99·3)	98·4 (96·9-99·4)	99·2 (97·2-99·9)	46·8 (36·1-56·4)	52·1 (39·6-63·0)	52·7 (40·4-64·0)	61·9 (40·0-82·6)	37·3 (26·5-47·5)	42·2 (26·3-55·9)	43·0 (26·8-57·1)	52·1 (26·2-76·6)	79·1 (49·9-99·7)	63·1 (33·7-86·5)	61·5 (32·1-85·2)	61·0 (31·4-85·2)
Vanuatu	81·6 (73·0-88·5)	83·9 (71·9-92·0)	84·1 (72·1-92·3)	89·7 (63·3-100·0)	34·4 (28·5-40·1)	39·1 (29·4-48·2)	39·9 (29·7-48·8)	49·8 (28·1-70·9)	27·2 (18·1-36·9)	31·0 (25·6-36·9)	31·7 (26·3-37·6)	40·4 (23·2-60·4)	75·6 (44·0-98·9)	63·8 (34·2-86·1)	62·2 (32·4-84·5)	61·4 (31·8-83·7)
**North Africa and Middle East**																
Afghanistan	2·0 (0·0-10·9)	30·0 (16·5-44·8)	31·8 (17·6-47·9)	57·1 (24·4-82·2)	5·6 (3·6-7·6)	21·4 (16·2-26·2)	22·3 (16·7-27·2)	37·3 (26·5-47·3)	0·7 (0·0-2·7)	15·3 (9·0-21·1)	16·3 (9·5-22·4)	31·1 (18·2-42·9)	89·6 (74·4-100·0)	80·1 (60·3-99·3)	80·0 (60·2-99·3)	79·6 (60·1-98·6)
Algeria	0·0 (0·0-0·0)	4·7 (0·0-18·5)	5·1 (0·0-18·9)	23·0 (0·0-59·9)	38·4 (35·2-41·5)	50·9 (45·1-56·1)	52·4 (46·0-57·9)	64·1 (51·3-77·1)	27·0 (23·8-30·5)	37·6 (33·0-42·0)	39·3 (34·3-44·0)	48·9 (37·4-61·1)	85·0 (80·8-89·3)	78·9 (70·5-100·0)	82·0 (71·5-100·0)	81·5 (71·1-100·0)
Bahrain	92·2 (87·5-95·8)	98·6 (97·5-99·3)	98·6 (97·5-99·3)	99·8 (98·7-100·0)	61·3 (57·9-64·8)	74·1 (68·8-79·3)	75·0 (69·5-80·6)	87·3 (69·8-100·0)	59·8 (55·9-64·0)	75·6 (70·7-80·5)	76·5 (71·5-81·7)	89·8 (73·5-100·0)	78·8 (73·8-83·7)	76·3 (70·5-81·7)	76·5 (70·8-82·0)	82·0 (77·0-86·8)
Egypt	58·2 (54·3-61·9)	91·3 (88·9-93·1)	92·0 (89·4-93·9)	98·6 (97·3-99·5)	31·8 (28·7-34·7)	49·5 (43·4-54·6)	51·1 (44·2-57·2)	71·1 (51·7-89·2)	22·9 (19·0-27·1)	42·4 (34·2-50·0)	43·9 (35·0-51·8)	65·2 (41·6-86·5)	97·0 (94·4-98·7)	87·4 (81·2-92·6)	87·1 (80·5-92·6)	86·5 (79·8-92·0)
Iran	94·5 (91·1-97·0)	97·8 (96·3-98·9)	97·9 (96·4-98·9)	99·0 (97·0-99·9)	33·6 (28·7-38·6)	51·8 (44·2-59·3)	53·0 (45·5-60·5)	70·1 (50·1-90·5)	21·0 (15·6-26·6)	38·2 (29·8-46·8)	39·5 (31·0-48·3)	56·3 (33·9-80·2)	81·9 (74·7-88·9)	75·4 (69·2-81·2)	75·3 (68·8-81·4)	75·2 (68·2-81·1)
Iraq	57·5 (46·5-67·5)	85·1 (78·5-90·2)	85·7 (79·1-90·9)	93·5 (85·2-98·1)	32·7 (30·6-34·7)	41·5 (36·2-46·3)	42·0 (36·5-47·0)	54·2 (43·4-64·8)	21·8 (19·8-23·7)	30·6 (25·5-35·5)	31·4 (25·9-36·4)	42·6 (31·2-53·7)	85·4 (68·9-99·3)	81·2 (62·6-99·4)	81·2 (62·6-99·6)	80·8 (62·2-99·1)
Jordan	98·0 (97·5-98·4)	99·7 (99·4-99·9)	99·6 (99·4-99·9)	99·9 (99·6-100·0)	43·2 (40·6-45·6)	54·0 (49·2-58·3)	53·7 (48·6-58·2)	63·5 (53·8-73·3)	29·3 (26·9-31·6)	40·7 (35·6-45·1)	41·3 (35·9-46·0)	51·9 (41·1-62·2)	98·0 (95·6-99·8)	99·9 (98·9-100·0)	99·9 (98·9-100·0)	99·4 (97·5-100·0)
Kuwait	96·2 (93·9-97·9)	98·6 (97·6-99·2)	98·7 (97·9-99·3)	99·7 (98·8-100·0)	60·4 (57·2-63·7)	66·6 (62·1-71·1)	67·6 (61·7-73·5)	75·0 (57·1-92·4)	51·5 (48·4-54·6)	58·7 (54·0-63·5)	59·8 (53·2-66·5)	67·3 (48·0-87·8)	95·0 (91·9-97·5)	98·1 (95·7-99·4)	98·2 (95·9-99·6)	100·0 (99·5-100·0)
Lebanon	74·8 (62·8-84·3)	91·3 (85·9-95·2)	91·7 (86·6-95·6)	97·5 (92·6-99·9)	52·2 (45·7-58·5)	66·9 (60·0-73·8)	67·7 (60·5-74·8)	81·4 (59·3-100·0)	40·5 (33·5-47·5)	59·7 (52·3-67·3)	60·8 (53·0-68·8)	78·6 (55·5-100·0)	83·8 (66·1-98·4)	81·2 (63·1-98·5)	81·1 (62·9-98·4)	82·0 (63·2-99·0)
Libya	90·7 (84·7-95·0)	96·1 (93·3-97·9)	96·0 (93·2-97·9)	97·0 (91·0-99·6)	43·4 (39·8-47·0)	55·7 (48·8-61·7)	58·8 (52·1-64·3)	77·0 (64·2-87·3)	33·5 (29·2-37·8)	51·7 (43·8-58·1)	53·8 (45·9-60·4)	73·8 (59·6-86·0)	85·9 (69·2-99·7)	81·0 (62·7-99·5)	81·1 (62·7-99·7)	80·6 (62·3-99·1)
Morocco	52·1 (48·6-55·5)	76·1 (63·6-85·4)	76·9 (64·0-86·1)	89·4 (71·1-98·0)	31·0 (28·7-33·1)	47·1 (42·2-51·1)	48·3 (43·4-52·5)	64·5 (54·9-72·9)	18·3 (15·5-21·3)	32·5 (26·3-38·1)	33·8 (27·4-39·5)	47·9 (36·4-58·6)	74·0 (55·6-92·0)	79·8 (60·5-99·0)	80·0 (61·4-98·9)	78·9 (60·3-98·3)
Palestine	97·1 (96·3-97·7)	98·5 (97·7-99·1)	98·6 (97·8-99·2)	99·4 (97·9-100·0)	43·1 (39·0-47·3)	52·9 (45·8-60·6)	54·5 (47·2-62·2)	66·3 (49·5-84·3)	39·8 (35·0-44·6)	43·4 (35·0-52·5)	45·2 (36·4-54·6)	53·0 (30·7-76·3)	90·8 (86·7-94·2)	87·2 (82·1-91·7)	87·4 (82·2-91·9)	87·4 (82·4-91·7)
Oman	97·7 (96·1-98·8)	98·5 (97·3-99·2)	98·8 (97·9-99·4)	99·5 (98·2-100·0)	55·6 (51·6-59·4)	69·4 (65·7-73·0)	71·2 (67·3-75·2)	90·3 (74·3-100·0)	46·8 (42·6-50·7)	62·3 (58·8-65·9)	64·3 (60·4-68·5)	84·3 (66·2-100·0)	71·8 (66·7-77·3)	75·2 (69·0-86·2)	69·1 (62·0-79·9)	53·7 (37·4-69·1)
Qatar	95·7 (92·6-97·8)	99·4 (98·8-99·7)	99·4 (98·8-99·7)	99·9 (99·3-100·0)	54·8 (46·8-63·2)	67·0 (58·1-76·3)	68·0 (58·8-77·7)	82·4 (58·0-100·0)	45·3 (36·6-54·5)	60·2 (50·3-70·5)	61·2 (50·8-72·6)	76·4 (47·9-100·0)	91·7 (87·7-95·2)	98·2 (95·6-99·5)	98·4 (95·8-99·8)	86·6 (78·3-94·8)
Saudi Arabia	92·8 (89·3-95·4)	96·8 (94·2-98·3)	96·9 (94·4-98·4)	98·5 (95·9-99·8)	46·6 (39·4-53·4)	73·0 (63·0-81·4)	76·3 (66·1-84·9)	98·0 (86·4-100·0)	36·4 (28·2-44·3)	67·8 (56·9-77·3)	70·7 (59·4-80·3)	97·7 (84·3-100·0)	85·0 (68·3-99·0)	81·2 (62·5-99·5)	81·2 (62·7-99·5)	85·3 (67·8-100·0)
Sudan	65·8 (54·1-76·4)	73·1 (68·0-78·0)	74·9 (69·3-80·1)	87·5 (74·5-95·7)	14·1 (11·7-16·5)	23·0 (18·1-27·9)	24·1 (18·3-29·3)	34·0 (20·0-47·2)	7·9 (4·9-11·0)	14·4 (9·3-19·4)	15·6 (9·6-21·1)	24·0 (8·3-38·5)	49·3 (43·4-55·4)	54·4 (48·2-60·8)	56·8 (49·7-63·8)	55·3 (48·2-62·5)
Syria	76·6 (70·8-81·4)	92·1 (87·8-95·6)	92·0 (87·4-95·6)	95·7 (86·8-99·7)	52·3 (49·9-54·7)	43·7 (30·7-58·3)	44·2 (30·1-59·5)	50·0 (30·4-75·1)	45·9 (42·7-49·1)	51·4 (45·0-57·7)	53·1 (46·6-59·4)	67·6 (53·7-79·7)	99·4 (97·9-100·0)	92·7 (85·5-97·3)	94·9 (88·1-98·8)	83·9 (32·8-100·0)
Tunisia	90·8 (87·7-93·5)	98·4 (97·4-99·1)	98·5 (97·4-99·2)	99·8 (99·0-100·0)	43·4 (41·1-45·8)	59·6 (56·3-61·9)	60·9 (57·5-63·4)	77·3 (69·8-83·3)	31·1 (28·2-33·9)	48·0 (43·8-51·8)	49·6 (45·0-53·5)	65·9 (56·8-74·1)	84·8 (79·3-89·7)	74·8 (66·8-82·8)	75·2 (66·6-83·6)	73·5 (65·2-81·6)
Turkey	79·3 (76·2-82·3)	93·8 (89·8-96·4)	93·7 (89·7-96·4)	97·8 (93·7-99·8)	33·9 (30·4-37·7)	54·3 (47·7-59·8)	55·9 (49·6-61·4)	76·7 (58·0-93·2)	23·9 (19·9-28·1)	43·1 (34·6-50·2)	45·1 (36·9-52·1)	64·0 (43·3-82·6)	92·9 (87·7-96·6)	84·0 (77·1-91·4)	83·9 (77·1-91·4)	86·0 (79·5-92·8)
United Arab Emirates	98·6 (97·4-99·3)	99·5 (98·9-99·8)	99·5 (98·9-99·8)	99·6 (98·6-100·0)	65·8 (52·3-79·6)	78·7 (66·4-90·3)	80·0 (68·3-90·2)	89·2 (57·7-100·0)	60·4 (45·5-76·1)	75·6 (61·0-89·8)	77·1 (62·8-89·3)	88·2 (51·9-100·0)	87·1 (71·2-100·0)	73·8 (39·6-99·4)	73·1 (36·5-99·1)	74·5 (38·0-99·9)
Yemen	0·1 (0·0-1·1)	32·4 (20·7-43·8)	32·9 (20·2-44·8)	59·5 (26·8-83·7)	15·6 (14·1-17·1)	31·7 (27·3-35·7)	32·1 (27·3-36·4)	47·2 (37·6-56·0)	8·2 (6·1-10·5)	22·7 (16·5-28·3)	23·4 (16·9-29·3)	37·0 (23·9-49·6)	84·3 (67·3-98·6)	78·3 (57·9-99·1)	78·1 (57·5-99·1)	77·7 (57·5-98·7)
**South Asia**																

Location	Indicator 3.3.2: Age-standardised rate of tuberculosis cases (per 100,000 population)				Indicator 3.3.3: Age-standardised rate of malaria cases (per 1,000 population)				Indicator 3.3.4: Age-standardised rate of hepatitis B incidence (per 100,000 population)				Indicator 3.3.5: Age-standardised prevalence* of the sum of 15 neglected tropical diseases (NTDs) (%) *Prevalence estimates reported here may exceed 100% as they reflect the sum of prevalent cases of 15 NTDs.*			
	**2000**	**2015**	**2016**	**2030**	**2000**	**2015**	**2016**	**2030**	**2000**	**2015**	**2016**	**2030**	**2000**	**2015**	**2016**	**2030**
Kiribati	20·4<br>(18·6-22·3)	23·7<br>(21·9-25·7)	23·9<br>(22·1-25·9)	26·4<br>(24·6-28·5)	100·0<br>(100·0-100·0)	100·0<br>(100·0-100·0)	100·0<br>(100·0-100·0)	100·0<br>(100·0-100·0)	24·7<br>(21·5-27·9)	25·8<br>(22·3-29·2)	26·1<br>(22·5-29·5)	29·8<br>(26·0-33·5)	88·3<br>(85·6-90·7)	87·7<br>(85·2-89·8)	87·7<br>(85·2-89·8)	88·3<br>(85·8-90·3)
Marshall Islands	25·1<br>(22·7-27·4)	29·8<br>(27·7-32·2)	30·2<br>(28·2-32·6)	35·0<br>(32·7-38·2)	100·0<br>(100·0-100·0)	100·0<br>(100·0-100·0)	100·0<br>(100·0-100·0)	100·0<br>(100·0-100·0)	29·1<br>(26·8-31·5)	33·8<br>(31·5-36·2)	34·3<br>(32·0-36·7)	39·1<br>(36·4-41·8)	70·7<br>(65·0-75·6)	69·8<br>(64·4-74·4)	69·7<br>(64·3-74·4)	70·1<br>(64·9-74·6)
Papua New Guinea	31·4<br>(29·6-33·2)	35·8<br>(33·7-39·2)	36·5<br>(34·3-40·0)	42·6<br>(39·3-51·6)	1·4<br>(0·0-4·1)	9·2<br>(7·2-11·7)	9·7<br>(7·6-12·2)	16·1<br>(13·3-20·2)	26·3<br>(23·4-29·1)	29·5<br>(26·5-32·2)	29·8<br>(26·9-32·5)	34·7<br>(31·9-37·1)	18·7<br>(10·5-26·6)	20·0<br>(11·9-28·1)	20·0<br>(11·9-28·1)	20·1<br>(12·0-28·0)
Samoa	58·8<br>(56·6-61·1)	64·2<br>(62·0-66·5)	64·5<br>(62·2-66·8)	69·4<br>(67·1-71·8)	100·0<br>(100·0-100·0)	100·0<br>(100·0-100·0)	100·0<br>(100·0-100·0)	100·0<br>(100·0-100·0)	44·4<br>(42·3-46·5)	48·2<br>(46·2-50·2)	48·4<br>(46·4-50·5)	51·7<br>(49·7-53·9)	93·7<br>(91·3-95·2)	96·2<br>(95·3-97·0)	96·3<br>(95·5-97·1)	97·6<br>(96·8-98·2)
Solomon Islands	39·5<br>(37·6-41·6)	43·3<br>(41·4-45·4)	43·8<br>(41·9-45·9)	50·7<br>(48·7-52·8)	0·0<br>(0·0-0·0)	9·9<br>(8·5-11·3)	10·7<br>(9·4-12·1)	22·8<br>(18·8-26·9)	28·0<br>(25·3-30·8)	31·4<br>(28·7-34·3)	31·8<br>(29·1-34·7)	36·0<br>(33·2-39·0)	50·4<br>(42·2-57·9)	50·6<br>(42·4-58·0)	50·6<br>(42·4-58·0)	50·9<br>(42·8-58·3)
Tonga	66·6<br>(64·3-69·0)	69·4<br>(67·1-71·8)	69·5<br>(67·3-72·0)	72·2<br>(69·8-74·8)	100·0<br>(100·0-100·0)	100·0<br>(100·0-100·0)	100·0<br>(100·0-100·0)	100·0<br>(100·0-100·0)	0·9<br>(0·0-4·0)	4·8<br>(1·1-8·7)	5·1<br>(1·2-9·0)	8·6<br>(4·7-12·9)	87·3<br>(84·1-89·7)	88·4<br>(85·7-90·4)	88·4<br>(85·8-90·4)	88·7<br>(86·0-90·7)
Vanuatu	40·2<br>(38·2-42·2)	45·4<br>(43·4-47·4)	45·6<br>(43·7-47·7)	49·8<br>(47·8-51·9)	2·5<br>(0·0-6·6)	29·4<br>(27·4-31·0)	33·2<br>(31·0-34·9)	56·4<br>(52·5-59·5)	29·3<br>(26·5-31·8)	33·5<br>(30·8-35·9)	33·9<br>(31·2-36·3)	37·3<br>(34·5-39·9)	95·9<br>(94·3-96·9)	22·8<br>(10·2-34·2)	22·9<br>(10·3-34·3)	25·2<br>(12·4-36·5)
**North Africa and Middle East**																
Afghanistan	22·7<br>(20·3-24·7)	29·7<br>(27·4-31·6)	30·2<br>(27·9-32·2)	37·5<br>(34·2-39·8)	16·2<br>(12·4-20·5)	26·5<br>(22·1-33·8)	26·2<br>(21·7-34·3)	23·6<br>(17·2-42·0)	24·0<br>(22·1-26·1)	32·5<br>(30·3-34·9)	32·8<br>(30·6-35·2)	37·7<br>(35·5-40·3)	49·8<br>(39·1-58·6)	50·6<br>(38·9-60·2)	50·8<br>(39·0-60·3)	52·3<br>(40·5-61·5)
Algeria	48·3<br>(46·7-49·9)	55·7<br>(54·0-57·4)	56·0<br>(54·2-57·8)	63·1<br>(60·8-65·1)	91·3<br>(86·1-94·8)	96·1<br>(93·3-97·4)	95·8<br>(93·0-97·2)	90·8<br>(86·6-93·1)	41·4<br>(38·1-44·9)	52·2<br>(49·3-55·0)	52·9<br>(50·1-55·6)	62·6<br>(59·1-65·2)	91·5<br>(90·6-92·4)	91·5<br>(90·6-92·3)	91·5<br>(90·6-92·3)	91·7<br>(90·8-92·5)
Bahrain	61·0<br>(57·3-65·3)	70·2<br>(66·0-74·6)	70·5<br>(66·4-75·1)	75·4<br>(70·8-80·5)	100·0<br>(100·0-100·0)	100·0<br>(100·0-100·0)	100·0<br>(100·0-100·0)	100·0<br>(100·0-100·0)	26·1<br>(21·5-30·6)	44·5<br>(40·5-48·4)	45·0<br>(40·8-48·8)	51·5<br>(47·5-55·7)	99·8<br>(99·8-99·8)	99·8<br>(99·8-99·8)	99·8<br>(99·8-99·8)	99·8<br>(99·8-99·8)
Egypt	72·8<br>(71·2-74·6)	77·5<br>(75·5-79·4)	78·0<br>(75·9-79·9)	84·2<br>(81·1-86·8)	99·8<br>(99·5-100·0)	99·5<br>(98·6-99·9)	99·5<br>(98·6-99·9)	100·0<br>(100·0-100·0)	40·7<br>(38·1-43·2)	50·3<br>(47·4-52·8)	51·1<br>(48·1-53·7)	62·5<br>(58·9-65·6)	42·3<br>(39·2-45·2)	86·0<br>(84·3-87·5)	87·2<br>(85·5-88·6)	97·3<br>(96·8-97·9)
Iran	69·7<br>(67·5-72·0)	72·8<br>(70·6-75·1)	73·5<br>(71·3-75·8)	79·7<br>(77·4-82·2)	73·4<br>(21·0-96·9)	78·2<br>(73·6-81·2)	79·3<br>(74·6-82·3)	92·8<br>(88·4-95·6)	37·5<br>(34·0-41·4)	49·1<br>(45·0-53·1)	49·9<br>(45·9-54·0)	62·1<br>(57·3-66·8)	97·7<br>(97·4-97·9)	97·7<br>(97·2-98·0)	97·7<br>(97·2-98·0)	97·7<br>(97·3-98·0)
Iraq	46·9<br>(42·9-51·2)	55·7<br>(51·3-60·2)	56·2<br>(51·8-60·7)	63·1<br>(58·4-67·8)	92·6<br>(88·6-94·6)	100·0<br>(100·0-100·0)	100·0<br>(100·0-100·0)	100·0<br>(100·0-100·0)	34·1<br>(31·6-36·8)	40·4<br>(38·1-43·0)	41·1<br>(38·7-43·6)	49·7<br>(47·0-52·2)	96·7<br>(96·0-97·3)	96·9<br>(96·2-97·5)	96·9<br>(96·2-97·5)	96·8<br>(96·1-97·5)
Jordan	80·7<br>(77·7-83·6)	90·5<br>(87·6-93·4)	91·0<br>(88·0-93·9)	97·7<br>(93·7-100·0)	100·0<br>(100·0-100·0)	100·0<br>(100·0-100·0)	100·0<br>(100·0-100·0)	100·0<br>(100·0-100·0)	53·9<br>(51·4-56·7)	60·4<br>(57·9-63·1)	60·8<br>(58·4-63·5)	66·9<br>(64·7-69·1)	81·1<br>(78·3-83·6)	76·6<br>(72·3-80·4)	76·6<br>(72·3-80·4)	76·7<br>(72·4-80·5)
Kuwait	59·4<br>(55·5-63·9)	69·1<br>(65·0-73·8)	70·0<br>(65·9-74·7)	79·9<br>(75·5-84·9)	100·0<br>(100·0-100·0)	100·0<br>(100·0-100·0)	100·0<br>(100·0-100·0)	100·0<br>(100·0-100·0)	49·8<br>(47·3-52·3)	72·3<br>(70·1-74·2)	72·8<br>(70·6-74·7)	80·6<br>(78·3-82·8)	100·0<br>(100·0-100·0)	100·0<br>(100·0-100·0)	100·0<br>(100·0-100·0)	100·0<br>(100·0-100·0)
Lebanon	66·6<br>(65·0-68·1)	79·3<br>(77·5-81·0)	79·7<br>(77·9-81·5)	91·4<br>(88·8-93·4)	100·0<br>(100·0-100·0)	100·0<br>(100·0-100·0)	100·0<br>(100·0-100·0)	100·0<br>(100·0-100·0)	60·2<br>(58·1-62·0)	70·2<br>(68·2-71·9)	70·5<br>(68·6-72·2)	80·0<br>(77·9-82·0)	99·8<br>(99·7-99·8)	99·8<br>(99·8-99·8)	99·8<br>(99·8-99·8)	99·8<br>(99·8-99·8)
Libya	62·4<br>(58·4-67·2)	63·7<br>(59·2-68·8)	63·6<br>(59·1-68·7)	65·5<br>(60·4-71·2)	100·0<br>(100·0-100·0)	100·0<br>(100·0-100·0)	100·0<br>(100·0-100·0)	100·0<br>(100·0-100·0)	57·6<br>(55·7-59·6)	64·6<br>(62·5-66·6)	64·6<br>(62·5-66·7)	73·5<br>(70·9-75·9)	83·8<br>(82·0-85·5)	85·5<br>(83·5-87·6)	85·7<br>(83·7-87·7)	88·0<br>(86·4-89·8)
Morocco	26·4<br>(22·3-31·1)	31·9<br>(27·6-36·6)	32·3<br>(28·0-37·0)	37·6<br>(33·3-42·4)	98·4<br>(96·8-99·0)	100·0<br>(100·0-100·0)	100·0<br>(100·0-100·0)	100·0<br>(100·0-100·0)	53·4<br>(51·5-55·4)	62·0<br>(60·0-63·7)	62·4<br>(60·4-64·2)	69·9<br>(67·6-71·9)	89·3<br>(87·4-90·9)	86·8<br>(84·6-88·8)	86·8<br>(84·6-88·8)	86·9<br>(84·6-88·8)
Palestine	92·3<br>(88·2-96·7)	100·0<br>(99·6-100·0)	100·0<br>(99·9-100·0)	100·0<br>(100·0-100·0)	100·0<br>(100·0-100·0)	100·0<br>(100·0-100·0)	100·0<br>(100·0-100·0)	100·0<br>(100·0-100·0)	69·3<br>(66·8-72·1)	71·2<br>(68·5-74·1)	71·2<br>(68·5-74·1)	72·8<br>(69·6-76·3)	91·7<br>(89·5-93·3)	91·7<br>(89·3-93·5)	91·7<br>(89·3-93·5)	91·7<br>(89·3-93·5)
Oman	79·2<br>(75·1-83·5)	80·9<br>(76·5-85·4)	81·3<br>(76·8-85·8)	86·9<br>(81·9-91·8)	88·1<br>(83·0-91·0)	80·9<br>(69·6-100·0)	80·5<br>(68·1-100·0)	100·0<br>(100·0-100·0)	40·6<br>(37·1-44·0)	53·1<br>(49·3-56·7)	53·6<br>(49·9-57·2)	65·8<br>(61·5-69·7)	98·8<br>(98·5-99·0)	98·9<br>(98·7-99·1)	98·9<br>(98·7-99·1)	99·0<br>(98·7-99·2)
Qatar	53·6<br>(49·3-57·9)	63·7<br>(59·1-68·3)	63·8<br>(59·2-68·4)	65·4<br>(60·7-70·0)	100·0<br>(100·0-100·0)	100·0<br>(100·0-100·0)	100·0<br>(100·0-100·0)	100·0<br>(100·0-100·0)	63·9<br>(60·8-66·4)	68·5<br>(65·3-70·9)	68·9<br>(65·8-71·4)	76·0<br>(73·2-78·4)	99·8<br>(99·8-99·8)	99·8<br>(99·8-99·8)	99·8<br>(99·8-99·8)	99·8<br>(99·8-99·8)
Saudi Arabia	58·7<br>(56·3-61·2)	59·0<br>(56·8-61·4)	59·2<br>(57·0-61·6)	61·9<br>(59·6-64·4)	100·0<br>(100·0-100·0)	100·0<br>(100·0-100·0)	100·0<br>(100·0-100·0)	100·0<br>(100·0-100·0)	51·7<br>(48·0-55·1)	61·3<br>(57·5-64·9)	61·8<br>(58·0-65·4)	69·0<br>(65·3-72·6)	93·7<br>(89·0-96·5)	93·3<br>(92·6-93·8)	93·3<br>(92·6-93·8)	93·4<br>(92·8-94·0)
Sudan	32·2<br>(30·5-34·1)	38·5<br>(36·6-40·4)	38·8<br>(37·0-40·8)	43·6<br>(41·6-45·7)	12·9<br>(10·7-14·9)	18·5<br>(16·2-20·6)	18·4<br>(16·1-20·6)	18·1<br>(15·3-20·7)	38·4<br>(34·8-41·9)	48·2<br>(45·2-51·4)	49·0<br>(46·1-52·1)	61·0<br>(58·6-63·8)	82·1<br>(78·8-84·6)	84·6<br>(81·4-87·1)	84·8<br>(81·6-87·3)	87·5<br>(84·3-89·9)
Syria	66·2<br>(62·1-70·7)	71·9<br>(67·2-77·1)	71·9<br>(67·2-77·2)	78·5<br>(72·2-84·9)	96·2<br>(93·0-97·5)	100·0<br>(100·0-100·0)	100·0<br>(100·0-100·0)	100·0<br>(100·0-100·0)	57·7<br>(54·6-60·9)	78·6<br>(75·9-81·6)	78·7<br>(76·1-81·7)	90·1<br>(86·5-93·2)	75·0<br>(72·0-77·8)	73·7<br>(69·9-77·1)	73·5<br>(69·7-76·9)	72·2<br>(67·8-75·8)
Tunisia	59·8<br>(56·2-64·0)	65·8<br>(61·9-70·1)	66·1<br>(62·2-70·5)	71·8<br>(67·4-76·3)	100·0<br>(100·0-100·0)	100·0<br>(100·0-100·0)	100·0<br>(100·0-100·0)	100·0<br>(100·0-100·0)	45·4<br>(41·8-48·9)	62·2<br>(59·9-64·4)	63·1<br>(61·0-65·3)	78·3<br>(75·8-81·0)	91·1<br>(90·2-91·9)	91·2<br>(90·3-92·0)	91·2<br>(90·3-92·0)	91·2<br>(90·4-92·0)
Turkey	52·3<br>(50·8-53·8)	69·3<br>(67·6-71·0)	69·4<br>(67·7-71·1)	73·5<br>(71·7-75·2)	58·4<br>(54·6-60·8)	100·0<br>(100·0-100·0)	100·0<br>(100·0-100·0)	100·0<br>(100·0-100·0)	38·8<br>(34·8-43·0)	60·0<br>(56·4-63·0)	60·4<br>(56·9-63·4)	72·9<br>(68·5-76·9)	94·4<br>(93·4-95·2)	94·5<br>(93·5-95·4)	94·6<br>(93·5-95·4)	94·8<br>(93·8-95·6)
United Arab Emirates	72·9<br>(70·9-74·9)	79·3<br>(77·2-81·3)	79·1<br>(77·0-81·2)	83·5<br>(81·2-85·6)	100·0<br>(100·0-100·0)	100·0<br>(100·0-100·0)	100·0<br>(100·0-100·0)	100·0<br>(100·0-100·0)	59·2<br>(56·7-61·7)	62·1<br>(59·1-64·9)	62·2<br>(59·1-65·0)	63·2<br>(59·7-66·5)	99·8<br>(99·8-99·8)	99·8<br>(99·8-99·8)	99·8<br>(99·8-99·8)	99·8<br>(99·8-99·8)
Yemen	44·9<br>(40·8-49·7)	50·7<br>(46·3-55·5)	50·9<br>(46·4-55·7)	56·0<br>(50·8-61·4)	17·8<br>(3·1-25·2)	24·9<br>(20·8-29·9)	25·1<br>(21·1-30·1)	27·5<br>(24·4-33·4)	36·0<br>(32·3-39·1)	49·5<br>(46·5-52·2)	50·2<br>(47·3-52·9)	60·9<br>(57·2-64·0)	63·5<br>(58·6-67·6)	71·5<br>(67·0-75·8)	72·1<br>(67·6-76·3)	79·0<br>(74·8-82·8)
**South Asia**																

Location	Indicator 3.4.1: Age-standardised death rate due to cardiovascular disease, cancer, diabetes, and chronic respiratory disease in populations aged 30-70 (per 100,000 population)				Indicator 3.4.2: Age-standardised death rate due to self-harm (per 100,000 population)				Indicator 3.5.2: Risk-weighted prevalence of alcohol consumption, as measured by the summary exposure value (SEV) for alcohol use (%)				Indicator 3.6.1: Age-standardised death rate due to road injuries (per 100,000 population)			
	**2000**	**2015**	**2016**	**2030**	**2000**	**2015**	**2016**	**2030**	**2000**	**2015**	**2016**	**2030**	**2000**	**2015**	**2016**	**2030**
Kiribati	0·0 (0·0-0·0)	1·0 (0·0-5·8)	1·1 (0·0-6·3)	4·0 (0·0-15·4)	8·7 (2·0-16·6)	9·0 (0·0-19·6)	9·2 (0·0-19·6)	11·1 (0·0-27·9)	88·7 (86·1-91·2)	88·0 (82·9-92·2)	87·9 (82·7-92·2)	86·7 (78·1-92·7)	42·6 (37·9-48·4)	46·5 (39·6-54·7)	46·9 (40·1-54·8)	51·8 (38·7-65·6)
Marshall Islands	0·8 (0·0-4·6)	1·0 (0·0-7·3)	1·4 (0·0-8·1)	6·4 (0·0-24·0)	20·4 (12·1-31·5)	24·1 (14·0-37·5)	24·9 (15·1-38·7)	31·3 (13·8-50·9)	87·8 (77·3-94·3)	85·5 (73·7-93·1)	85·3 (73·3-93·0)	82·3 (67·1-91·4)	37·1 (29·7-43·0)	38·8 (30·4-47·2)	39·8 (31·4-48·4)	47·5 (32·9-63·1)
Papua New Guinea	0·1 (0·0-1·8)	0·6 (0·0-6·0)	0·8 (0·0-6·9)	6·7 (0·0-23·6)	27·9 (14·5-45·3)	29·2 (17·0-45·7)	29·5 (17·4-45·8)	33·6 (16·8-52·7)	50·7 (37·1-64·5)	58·6 (42·8-75·0)	58·6 (42·8-75·2)	59·4 (42·3-77·3)	13·0 (5·4-22·0)	15·8 (7·4-24·5)	16·2 (7·7-24·8)	22·1 (8·3-35·8)
Samoa	27·9 (20·4-36·1)	32·6 (24·4-42·3)	33·4 (25·4-43·1)	37·0 (20·3-53·2)	27·8 (19·0-38·0)	35·0 (26·7-45·2)	35·6 (27·0-45·7)	39·1 (23·9-53·9)	78·8 (72·9-84·0)	76·6 (66·5-85·5)	76·5 (66·2-85·6)	75·8 (62·4-87·4)	50·5 (42·3-58·5)	52·1 (44·5-61·4)	52·9 (45·1-61·8)	55·8 (42·5-70·0)
Solomon Islands	0·9 (0·0-6·7)	0·5 (0·0-5·9)	0·6 (0·0-6·5)	4·8 (0·0-19·7)	20·7 (8·2-36·4)	22·4 (10·7-38·2)	22·9 (11·0-38·8)	27·3 (10·5-44·6)	94·4 (92·2-96·4)	89·7 (84·7-93·6)	89·4 (84·1-93·5)	84·7 (75·1-91·6)	33·5 (23·3-42·3)	32·0 (21·7-40·8)	32·6 (22·1-41·5)	35·9 (20·7-50·8)
Tonga	26·2 (18·7-33·3)	31·4 (22·7-39·9)	31·8 (23·1-40·6)	36·2 (20·1-53·4)	54·7 (47·6-61·9)	56·2 (47·9-64·8)	56·2 (47·9-65·0)	56·3 (40·3-72·9)	91·1 (87·8-94·0)	89·1 (82·0-94·1)	89·0 (81·8-94·2)	88·8 (78·4-95·1)	41·6 (34·9-47·4)	45·4 (38·4-53·5)	45·8 (38·6-54·0)	49·4 (36·0-63·9)
Vanuatu	1·0 (0·0-7·1)	2·6 (0·0-11·1)	2·9 (0·0-11·6)	7·4 (0·0-24·6)	21·4 (9·1-35·6)	24·8 (13·5-38·3)	25·1 (13·8-38·2)	28·8 (12·8-45·2)	89·7 (85·6-93·1)	94·2 (89·6-97·5)	94·2 (89·5-97·6)	94·7 (87·8-98·7)	33·1 (24·2-40·9)	34·4 (24·8-42·6)	34·6 (25·2-42·6)	37·5 (24·0-51·4)
**North Africa and Middle East**																
Afghanistan	0·0 (0·0-0·0)	0·1 (0·0-0·8)	0·1 (0·0-1·1)	4·6 (0·0-15·1)	45·3 (27·0-53·5)	45·7 (29·3-53·0)	45·8 (29·5-53·2)	46·9 (29·4-60·2)	99·9 (99·6-100·0)	98·4 (96·9-99·4)	98·3 (96·8-99·3)	96·6 (93·1-98·7)	0·0 (0·0-0·0)	0·0 (0·0-0·0)	0·0 (0·0-0·0)	0·9 (0·0-6·4)
Algeria	58·9 (52·9-65·2)	73·0 (67·5-78·8)	72·4 (66·5-78·7)	86·9 (73·7-100·0)	83·1 (72·3-90·7)	86·2 (75·1-94·0)	86·5 (75·0-94·6)	89·9 (70·0-100·0)	94·7 (92·6-96·6)	94·0 (90·3-97·0)	94·0 (90·3-97·1)	94·6 (90·7-97·8)	14·4 (9·4-19·1)	25·2 (20·2-29·8)	25·8 (20·3-30·7)	35·5 (23·9-46·1)
Bahrain	33·9 (27·6-40·4)	65·2 (54·5-75·6)	65·2 (54·2-75·5)	85·4 (56·9-100·0)	48·0 (41·1-55·3)	61·2 (49·0-71·9)	60·9 (47·8-71·7)	70·5 (41·9-99·8)	82·5 (77·7-87·1)	87·3 (80·4-93·4)	87·3 (80·3-93·5)	88·0 (79·9-94·7)	26·1 (20·6-31·0)	46·5 (38·2-55·1)	46·5 (37·2-54·9)	62·1 (37·5-84·3)
Egypt	36·3 (32·3-40·0)	35·1 (29·0-41·2)	35·8 (28·7-43·0)	42·1 (24·3-59·5)	87·4 (76·2-100·0)	85·5 (72·6-98·6)	85·7 (72·1-98·7)	87·7 (67·2-100·0)	97·4 (96·5-98·2)	97·4 (96·5-98·2)	97·4 (96·5-98·2)	97·9 (97·1-98·6)	9·3 (5·2-13·6)	13·7 (8·5-19·6)	14·4 (8·6-21·2)	22·2 (7·4-36·5)
Iran	48·1 (41·0-55·6)	63·3 (53·4-73·2)	63·1 (53·1-73·3)	78·7 (50·8-100·0)	60·4 (53·3-68·0)	66·9 (58·2-74·6)	67·1 (58·2-74·8)	71·0 (49·2-91·3)	100·0 (100·0-100·0)	100·0 (100·0-100·0)	100·0 (100·0-100·0)	100·0 (100·0-100·0)	0·0 (0·0-0·0)	13·0 (5·7-20·2)	13·6 (6·1-21·2)	40·0 (18·2-60·3)
Iraq	14·6 (7·1-21·0)	24·1 (12·8-36·6)	24·8 (13·4-37·6)	36·0 (15·0-59·8)	47·4 (40·1-55·3)	47·5 (35·6-60·2)	47·5 (35·2-60·0)	48·7 (25·7-71·5)	92·4 (88·9-95·6)	93·9 (89·0-97·1)	93·7 (88·6-97·0)	90·8 (81·6-96·2)	18·6 (12·5-24·9)	23·5 (14·1-33·4)	23·9 (14·4-33·8)	30·4 (12·7-50·5)
Jordan	44·8 (34·3-55·6)	62·0 (48·5-74·8)	62·5 (48·1-76·0)	78·8 (47·9-100·0)	74·1 (64·7-83·9)	88·9 (78·2-99·5)	88·8 (77·8-99·6)	90·0 (66·3-100·0)	97·8 (96·6-98·8)	96·5 (93·7-98·5)	96·5 (93·6-98·6)	96·3 (92·0-98·7)	32·2 (24·4-39·5)	41·1 (32·2-50·0)	41·4 (32·0-50·7)	46·0 (25·9-65·4)
Kuwait	60·3 (53·0-68·0)	80·3 (68·5-93·5)	79·4 (64·3-94·2)	91·3 (56·3-100·0)	92·5 (85·0-100·0)	97·3 (87·6-100·0)	96·6 (85·0-100·0)	95·3 (71·7-100·0)	100·0 (100·0-100·0)	100·0 (100·0-100·0)	100·0 (100·0-100·0)	97·3 (72·9-100·0)	23·7 (18·5-29·4)	39·4 (30·6-48·7)	39·5 (28·1-51·0)	54·1 (20·7-84·9)
Lebanon	57·5 (50·9-64·4)	78·9 (72·0-85·3)	78·9 (71·6-85·9)	91·6 (71·7-100·0)	93·3 (78·7-100·0)	98·7 (89·6-100·0)	98·7 (89·4-100·0)	99·4 (92·1-100·0)	82·7 (78·6-86·8)	83·1 (75·1-89·6)	82·9 (74·6-89·7)	80·4 (68·2-90·1)	59·1 (53·1-65·6)	76·1 (69·3-83·3)	76·4 (69·4-83·6)	86·9 (70·0-100·0)
Libya	57·4 (51·9-63·3)	58·5 (51·4-66·1)	58·0 (50·8-65·4)	57·3 (43·8-69·9)	80·0 (69·7-88·8)	77·6 (64·2-88·9)	77·0 (63·0-88·5)	74·4 (51·1-94·3)	100·0 (100·0-100·0)	100·0 (99·8-100·0)	100·0 (99·8-100·0)	99·9 (99·5-100·0)	17·0 (12·1-22·4)	23·9 (17·4-30·4)	24·0 (17·4-30·8)	29·2 (16·7-41·2)
Morocco	51·7 (45·5-61·7)	58·6 (53·7-64·6)	59·1 (54·2-65·1)	66·2 (58·6-74·8)	66·0 (56·7-76·9)	70·5 (61·4-78·4)	71·3 (62·6-79·1)	83·0 (69·5-96·0)	95·3 (94·0-96·5)	96·0 (94·8-97·0)	96·0 (94·9-97·0)	96·7 (95·7-97·6)	16·3 (11·5-21·0)	23·3 (18·4-28·1)	23·7 (18·7-28·5)	30·3 (22·2-38·0)
Palestine	42·4 (39·7-45·4)	38·4 (35·2-41·6)	37·8 (34·2-41·5)	29·5 (20·3-39·6)	98·2 (89·7-100·0)	98·1 (90·2-100·0)	97·4 (88·2-100·0)	92·8 (76·7-100·0)	99·8 (99·9-99·8)	99·3 (98·6-99·8)	99·3 (98·6-99·8)	99·5 (98·8-100·0)	54·3 (50·2-58·6)	42·4 (34·4-51·5)	42·1 (34·0-51·3)	35·7 (21·9-49·8)
Oman	53·6 (48·2-58·7)	63·2 (58·3-68·9)	63·9 (59·1-69·2)	73·4 (65·9-82·6)	92·9 (82·5-100·0)	93·4 (83·1-100·0)	93·2 (83·1-100·0)	91·3 (69·9-100·0)	95·9 (94·0-97·4)	94·1 (89·9-97·1)	94·1 (89·9-97·1)	93·7 (88·1-97·4)	0·0 (0·0-0·0)	0·7 (0·0-4·2)	0·8 (0·0-5·0)	9·1 (0·4-18·7)
Qatar	56·0 (44·0-66·4)	87·8 (71·6-100·0)	86·9 (68·8-100·0)	95·7 (68·5-100·0)	57·5 (46·2-68·4)	76·1 (61·8-88·9)	74·8 (59·1-89·3)	84·3 (45·9-100·0)	91·9 (88·4-94·9)	85·2 (79·3-90·9)	85·1 (79·2-90·8)	83·4 (76·6-90·0)	5·9 (0·0-14·3)	18·3 (6·8-29·9)	18·1 (4·8-31·0)	30·8 (0·0-65·6)
Saudi Arabia	59·1 (54·9-62·9)	73·4 (68·2-78·4)	74·7 (69·4-79·8)	88·3 (78·1-99·3)	95·7 (87·5-100·0)	93·0 (86·5-100·0)	93·6 (86·5-100·0)	87·7 (69·5-100·0)	99·5 (99·0-99·9)	99·3 (98·7-99·8)	99·3 (98·7-99·8)	98·7 (97·7-99·4)	1·8 (0·0-5·9)	10·2 (5·8-15·2)	11·3 (6·9-16·3)	20·4 (11·5-29·9)
Sudan	28·7 (24·3-34·8)	42·3 (38·0-47·2)	42·8 (38·5-47·6)	53·0 (45·6-59·8)	73·6 (62·4-88·9)	72·7 (64·2-83·2)	72·6 (64·0-83·1)	71·8 (59·9-83·4)	79·6 (75·1-83·9)	80·1 (73·3-86·3)	80·1 (73·3-86·3)	80·2 (72·7-86·8)	0·5 (0·0-4·9)	5·0 (0·0-10·1)	5·6 (0·6-10·4)	11·1 (2·9-19·4)
Syria	37·8 (33·9-42·2)	55·4 (49·7-61·4)	55·9 (50·0-61·8)	67·1 (54·6-79·2)	99·0 (93·3-100·0)	99·7 (96·4-100·0)	99·6 (95·7-100·0)	97·5 (85·6-100·0)	95·0 (93·4-96·7)	95·8 (93·4-98·1)	95·9 (93·4-98·1)	96·8 (94·3-99·1)	40·5 (37·0-43·8)	50·2 (46·0-54·4)	50·8 (46·5-55·3)	58·9 (49·8-68·8)
Tunisia	58·9 (51·9-65·4)	67·6 (57·6-78·1)	67·9 (58·0-78·5)	75·7 (57·7-95·7)	85·6 (76·1-95·2)	85·9 (74·9-96·9)	86·2 (74·9-97·2)	88·7 (70·5-100·0)	94·6 (93·3-95·8)	94·0 (91·0-96·8)	94·0 (90·9-96·8)	93·1 (88·5-97·0)	19·3 (13·3-25·0)	27·0 (18·5-35·1)	27·5 (18·9-35·6)	35·8 (21·5-50·0)
Turkey	53·3 (49·5-57·1)	72·6 (64·1-80·5)	73·7 (63·8-82·8)	92·6 (67·0-100·0)	80·6 (72·0-87·9)	90·4 (82·3-97·9)	89·9 (80·9-98·4)	94·0 (75·9-100·0)	72·3 (66·1-78·2)	84·9 (76·3-91·5)	85·4 (76·8-92·0)	90·6 (81·9-96·1)	44·7 (40·5-49·0)	57·2 (50·9-63·7)	57·3 (50·3-64·4)	64·0 (47·0-81·0)
United Arab Emirates	46·9 (39·4-54·4)	51·4 (38·6-63·1)	51·6 (38·1-63·8)	52·3 (24·5-77·1)	81·2 (70·2-91·7)	84·7 (67·4-98·2)	85·0 (67·6-98·7)	83·7 (55·2-100·0)	71·9 (66·1-76·9)	71·4 (63·3-78·4)	71·7 (63·6-78·7)	75·2 (66·8-82·2)	4·8 (0·0-13·1)	1·8 (0·0-12·1)	2·0 (0·0-12·5)	3·0 (0·0-20·0)
Yemen	19·6 (12·6-27·4)	30·3 (22·8-37·1)	29·9 (22·5-36·9)	37·7 (25·0-49·3)	65·9 (54·3-83·5)	63·8 (54·1-75·8)	63·3 (53·3-75·4)	60·8 (46·9-74·8)	98·2 (97·3-99·0)	99·3 (98·4-99·8)	99·3 (98·4-99·8)	98·9 (97·4-99·7)	0·0 (0·0-0·0)	2·1 (0·0-6·6)	2·2 (0·0-6·7)	8·1 (0·0-17·3)
**South Asia**																

Location	Indicator 3.7.1: Proportion of women of reproductive age (15-49 years) who have their need for family planning satisfied with modern contraception methods (%)				Indicator 3.7.2: Number of livebirths per 1,000 women aged 10-14 years and women aged 15-19 years				Indicator 3.8.1: Coverage of essential health services, as defined by the UHC index comprised of the coverage of 9 tracer interventions and risk-standardised death rates from 32 causes amenable to personal healthcare (scale of 0 to 100)				Indicator 3.9.1: Age-standardised death rate attributable to household air pollution and ambient air pollution (per 100,000 population)			
	**2000**	**2015**	**2016**	**2030**	**2000**	**2015**	**2016**	**2030**	**2000**	**2015**	**2016**	**2030**	**2000**	**2015**	**2016**	**2030**
Kiribati	41·3 (30·1-51·4)	57·6 (47·6-67·7)	58·0 (48·0-67·8)	70·0 (50·5-86·1)	33·7 (31·7-35·9)	58·1 (55·3-61·0)	61·1 (58·2-64·0)	86·4 (79·8-93·0)	15·8 (11·3-20·2)	21·1 (15·6-26·4)	22·5 (16·8-27·8)	27·9 (21·2-34·0)	12·9 (8·6-17·0)	20·2 (14·9-26·1)	20·6 (15·3-26·6)	27·0 (20·5-34·6)
Marshall Islands	65·8 (56·5-74·8)	76·5 (67·7-85·0)	76·9 (68·3-85·3)	84·7 (69·2-97·4)	0·1 (0·0-1·1)	15·1 (12·3-18·5)	16·1 (13·2-19·6)	30·6 (24·0-38·1)	15·3 (10·2-20·1)	26·5 (20·5-32·7)	27·7 (21·5-34·0)	39·0 (29·9-47·8)	31·5 (20·4-47·8)	44·0 (28·6-67·2)	45·2 (29·4-69·2)	61·5 (41·5-96·0)
Papua New Guinea	39·1 (30·7-48·5)	51·5 (41·3-61·9)	52·4 (41·9-62·6)	63·3 (43·6-80·4)	19·7 (18·5-21·1)	24·2 (23·1-25·4)	24·2 (23·1-25·5)	25·9 (23·4-28·6)	8·2 (2·8-13·7)	17·2 (10·9-23·6)	18·3 (11·8-24·8)	33·5 (23·6-43·4)	0·0 (0·0-0·0)	0·8 (0·0-4·8)	1·1 (0·0-5·2)	5·8 (0·6-11·2)
Samoa	55·4 (46·0-64·6)	65·4 (55·0-74·5)	65·8 (55·5-74·8)	74·2 (55·2-88·6)	34·6 (32·3-37·1)	44·2 (41·7-47·0)	45·5 (43·0-48·4)	56·6 (50·6-62·9)	28·5 (23·6-33·8)	34·0 (28·9-40·1)	34·8 (29·6-40·8)	44·1 (36·9-51·2)	31·9 (26·3-38·1)	44·8 (36·8-52·9)	45·8 (37·5-54·1)	57·3 (46·8-68·6)
Solomon Islands	47·1 (36·7-57·3)	59·2 (48·5-69·1)	59·9 (49·3-69·7)	69·9 (51·9-86·1)	19·5 (17·5-21·9)	30·5 (27·4-33·9)	31·5 (28·3-34·9)	43·2 (35·9-51·4)	14·4 (8·4-20·0)	19·4 (13·5-25·2)	20·5 (14·5-26·2)	31·9 (25·1-38·6)	4·3 (0·0-9·4)	7·4 (1·9-13·0)	7·8 (2·3-13·4)	13·3 (7·5-19·0)
Tonga	59·8 (50·0-69·4)	74·4 (65·3-82·5)	74·7 (65·8-82·6)	84·8 (67·8-96·9)	45·7 (43·8-47·4)	58·7 (56·1-61·3)	59·6 (57·0-62·3)	71·9 (66·7-77·1)	38·2 (33·0-42·8)	44·8 (39·0-50·5)	45·4 (39·5-51·2)	50·3 (43·4-57·0)	31·4 (25·6-37·9)	52·4 (43·2-63·5)	53·6 (44·1-64·9)	69·9 (57·1-85·8)
Vanuatu	53·7 (43·8-63·8)	67·0 (57·4-75·6)	67·3 (57·5-76·0)	76·5 (58·4-90·6)	20·7 (18·8-22·6)	27·4 (25·7-29·2)	27·8 (26·1-29·8)	34·1 (30·6-38·1)	12·8 (7·3-17·6)	17·4 (10·8-22·9)	18·0 (11·4-23·5)	25·8 (18·0-33·1)	2·7 (0·0-7·5)	9·6 (3·3-15·9)	10·0 (3·7-16·4)	16·4 (9·4-24·0)
**North Africa and Middle East**																
Afghanistan	15·4 (8·5-22·9)	34·3 (31·2-37·5)	35·0 (31·2-38·9)	54·2 (40·9-67·5)	7·1 (3·0-12·9)	19·9 (13·8-28·1)	20·8 (14·5-29·4)	34·3 (19·0-53·4)	0·0 (0·0-0·0)	3·7 (0·0-10·1)	4·9 (0·0-11·3)	26·6 (18·5-37·0)	0·0 (0·0-0·0)	0·0 (0·0-0·0)	0·0 (0·0-0·0)	0·1 (0·0-1·3)
Algeria	76·0 (71·6-80·5)	77·8 (72·9-82·8)	78·7 (73·1-84·2)	82·8 (71·4-92·8)	52·2 (47·9-56·9)	68·0 (61·0-75·6)	69·6 (62·6-77·1)	85·8 (71·2-100·0)	45·6 (40·3-49·8)	62·5 (57·5-67·0)	63·3 (58·3-67·9)	76·3 (71·0-81·2)	46·5 (43·1-49·7)	57·0 (53·3-60·8)	56·7 (53·0-60·5)	70·1 (57·9-82·3)
Bahrain	59·7 (49·5-69·6)	71·4 (61·7-80·4)	72·0 (62·3-81·2)	81·0 (63·4-94·4)	57·4 (55·2-59·5)	61·5 (59·2-63·9)	62·3 (59·6-65·2)	60·8 (57·3-64·2)	51·7 (47·9-55·6)	70·1 (64·5-75·5)	70·8 (65·0-76·4)	80·3 (70·8-88·5)	30·5 (25·8-35·1)	47·3 (40·9-53·3)	47·8 (41·4-53·9)	60·1 (51·3-71·4)
Egypt	83·7 (81·9-85·5)	88·6 (86·4-90·9)	89·1 (86·0-91·9)	94·0 (88·5-98·7)	26·4 (24·4-28·4)	31·0 (29·0-33·3)	32·0 (29·7-34·5)	36·5 (31·2-43·0)	39·7 (36·9-42·5)	56·6 (52·5-60·9)	57·6 (53·5-62·1)	73·3 (67·6-79·6)	30·5 (26·8-33·9)	29·1 (24·2-33·5)	29·2 (24·3-33·6)	30·7 (25·5-35·4)
Iran	78·6 (71·0-85·8)	86·8 (79·4-93·1)	87·2 (79·9-93·1)	94·8 (83·3-100·0)	33·3 (24·4-43·0)	41·8 (23·8-60·0)	43·3 (24·5-61·4)	64·2 (27·4-100·0)	56·7 (51·4-61·5)	69·0 (62·8-74·9)	70·2 (63·9-76·1)	79·9 (73·3-86·4)	41·0 (36·3-45·4)	48·8 (43·6-53·9)	49·5 (44·3-54·6)	57·0 (51·3-62·3)
Iraq	48·0 (39·5-57·3)	55·4 (48·5-62·1)	56·5 (48·9-64·3)	69·6 (49·6-85·4)	22·5 (13·3-37·0)	9·2 (0·0-26·9)	9·2 (0·0-28·1)	8·7 (0·0-42·9)	27·2 (22·3-32·2)	41·1 (34·5-47·5)	42·4 (35·8-49·0)	56·7 (47·8-65·7)	24·3 (19·4-28·7)	27·5 (22·1-33·1)	28·5 (23·0-34·1)	31·0 (18·3-42·5)
Jordan	49·9 (46·8-53·0)	58·3 (53·0-63·5)	59·1 (52·5-65·3)	69·5 (55·9-80·1)	36·8 (34·3-39·6)	49·0 (46·1-52·0)	50·1 (46·9-53·3)	63·1 (56·2-69·6)	51·4 (45·7-56·7)	66·2 (59·5-72·8)	67·2 (60·3-74·0)	78·4 (69·0-87·5)	47·7 (42·6-53·0)	56·1 (49·2-62·5)	56·6 (49·6-63·0)	63·4 (55·5-70·9)
Kuwait	67·8 (58·7-76·4)	83·9 (75·5-90·6)	84·1 (76·4-90·7)	94·4 (82·3-100·0)	49·3 (47·3-51·1)	78·6 (75·6-81·2)	81·9 (78·7-84·8)	100·0 (100·0-100·0)	65·0 (61·1-69·5)	77·7 (71·1-84·6)	79·5 (72·6-86·8)	96·4 (86·7-100·0)	43·5 (38·4-48·4)	46·1 (38·4-54·1)	46·8 (38·8-55·1)	55·2 (43·3-67·4)
Lebanon	54·1 (46·4-61·5)	63·1 (53·5-72·6)	63·8 (54·3-73·2)	80·9 (60·9-96·3)	48·8 (45·1-52·9)	69·1 (63·9-74·3)	70·3 (65·1-75·5)	92·6 (81·1-100·0)	57·1 (52·6-61·0)	81·4 (76·1-86·3)	82·3 (77·2-87·1)	94·4 (89·1-99·4)	56·5 (52·5-60·7)	64·8 (59·9-69·6)	64·7 (59·8-69·5)	74·9 (66·3-87·0)
Libya	59·5 (49·3-68·8)	79·0 (70·7-86·6)	78·7 (70·2-86·1)	91·6 (78·3-100·0)	75·0 (70·1-80·1)	92·8 (87·0-98·6)	93·8 (87·8-99·7)	100·0 (100·0-100·0)	54·4 (49·9-58·3)	64·8 (60·3-68·8)	65·0 (60·5-69·1)	68·9 (62·9-74·4)	44·2 (40·3-48·2)	44·9 (40·7-49·5)	44·7 (40·5-49·3)	44·1 (39·5-49·8)
Morocco	71·9 (66·9-76·2)	83·2 (75·6-90·1)	83·7 (75·9-90·5)	91·5 (79·7-99·9)	36·9 (34·7-39·2)	49·7 (46·9-52·5)	50·6 (47·8-53·4)	63·0 (56·4-69·0)	35·1 (31·1-38·9)	52·6 (47·3-57·7)	53·6 (48·5-58·4)	69·3 (63·4-75·4)	41·7 (38·3-46·0)	53·0 (49·3-56·9)	53·2 (49·4-57·0)	67·6 (58·9-78·8)
Palestine	57·5 (48·5-66·6)	61·4 (58·4-64·3)	62·7 (58·5-67·0)	75·2 (64·2-85·1)	20·2 (8·7-41·1)	14·1 (6·6-28·0)	14·8 (6·7-30·5)	18·1 (0·0-52·6)	49·7 (46·5-53·3)	54·4 (51·3-57·6)	54·8 (51·5-58·0)	58·3 (53·6-62·7)	38·2 (35·2-41·3)	38·7 (35·3-42·1)	38·4 (35·1-41·9)	35·6 (32·1-39·2)
Oman	41·8 (32·1-51·5)	77·3 (67·7-85·3)	78·0 (68·8-85·9)	97·6 (86·8-100·0)	29·7 (27·6-32·4)	74·9 (70·4-79·8)	79·4 (74·7-84·8)	100·0 (100·0-100·0)	70·4 (66·8-74·2)	83·3 (80·0-86·6)	84·3 (80·9-87·6)	97·3 (92·9-100·0)	30·0 (25·9-33·6)	44·4 (40·5-48·3)	44·5 (40·6-48·5)	56·2 (48·6-64·9)
Qatar	61·6 (52·0-70·5)	76·8 (68·4-84·3)	77·1 (68·6-84·5)	87·5 (74·2-98·3)	51·0 (48·7-53·5)	70·0 (66·0-74·1)	70·5 (66·3-74·7)	84·5 (73·4-95·3)	64·3 (58·9-70·1)	86·8 (78·7-94·5)	87·7 (79·1-95·7)	96·6 (84·7-100·0)	40·4 (34·5-46·5)	54·8 (45·5-63·6)	54·9 (45·4-64·0)	57·5 (44·3-70·2)
Saudi Arabia	55·0 (45·4-65·0)	84·7 (77·7-91·4)	85·4 (78·5-91·7)	99·0 (92·6-100·0)	40·9 (38·0-44·2)	81·5 (75·8-86·9)	84·5 (78·8-90·0)	100·0 (100·0-100·0)	58·9 (56·6-61·5)	76·9 (74·0-80·2)	78·7 (75·7-82·1)	95·8 (91·7-99·9)	25·0 (22·1-28·1)	36·2 (31·9-40·1)	36·7 (32·4-40·7)	44·4 (39·8-48·5)
Sudan	2·6 (0·0-7·7)	19·2 (15·9-22·2)	20·2 (16·1-24·0)	39·1 (26·3-52·8)	13·2 (9·5-17·6)	30·5 (22·8-37·9)	31·9 (24·3-39·1)	53·3 (37·2-68·8)	16·0 (12·7-19·4)	31·6 (27·4-35·3)	33·3 (28·9-37·1)	49·6 (43·6-54·8)	4·7 (1·9-7·9)	16·0 (13·1-18·8)	16·4 (13·5-19·2)	22·8 (19·6-26·0)
Syria	53·3 (49·4-57·2)	74·2 (65·3-82·0)	74·6 (66·1-82·3)	87·4 (74·4-97·6)	24·2 (20·9-28·2)	41·9 (36·4-47·2)	42·5 (36·8-47·6)	58·2 (46·9-69·2)	54·1 (51·7-56·5)	69·7 (65·9-73·5)	70·0 (66·3-74·0)	80·4 (73·4-86·6)	37·8 (33·9-41·3)	43·0 (38·9-47·2)	43·1 (39·0-47·3)	45·7 (41·1-50·6)
Tunisia	79·8 (73·3-85·9)	74·7 (68·6-80·7)	75·8 (69·3-82·4)	88·5 (69·7-100·0)	76·5 (73·2-79·5)	83·4 (79·5-86·9)	84·5 (80·6-88·3)	95·6 (85·4-100·0)	53·4 (47·8-58·2)	66·8 (60·9-72·4)	67·6 (61·7-73·2)	78·8 (72·8-84·6)	46·0 (42·3-49·8)	54·2 (48·8-59·6)	54·3 (48·9-59·7)	56·5 (50·4-62·7)
Turkey	48·3 (45·2-51·4)	66·6 (57·0-75·2)	67·5 (58·1-76·0)	82·5 (68·2-94·2)	29·2 (26·4-31·9)	45·0 (41·7-48·4)	46·8 (42·9-50·7)	62·0 (53·6-70·7)	39·8 (36·2-43·4)	68·5 (63·7-73·6)	69·2 (64·1-74·6)	84·2 (75·5-92·5)	46·7 (43·4-49·7)	60·2 (55·0-65·5)	60·4 (55·1-65·8)	66·9 (59·1-73·9)
United Arab Emirates	57·3 (46·6-67·7)	72·6 (63·1-81·0)	72·9 (63·2-81·3)	86·8 (71·9-98·5)	35·3 (33·6-37·0)	28·2 (26·5-30·1)	28·1 (26·4-30·1)	32·8 (27·8-37·7)	56·3 (52·2-60·2)	66·7 (60·8-73·4)	67·3 (61·1-74·1)	72·6 (63·9-81·6)	35·0 (30·9-39·2)	37·0 (30·9-42·3)	36·4 (30·2-41·7)	32·6 (24·0-41·1)
Yemen	11·9 (8·3-15·7)	40·7 (36·1-45·1)	41·6 (35·5-47·5)	70·9 (54·8-83·9)	13·8 (6·5-28·7)	22·2 (9·9-40·7)	23·5 (9·3-45·4)	39·6 (5·9-80·3)	10·0 (5·8-14·4)	26·9 (21·6-31·9)	28·0 (22·7-33·2)	43·5 (36·1-50·5)	6·8 (3·6-10·2)	17·6 (13·6-21·3)	17·9 (13·8-21·6)	26·8 (20·7-33·7)
**South Asia**																

Location	Indicator 3.9.2: Age-standardised death rate attributable to unsafe water, sanitation, and hygiene (WaSH) (per 100,000 population)				Indicator 3.9.3: Age-standardised death rate due to unintentional poisonings (per 100,000 population)			
	**2000**	**2015**	**2016**	**2030**	**2000**	**2015**	**2016**	**2030**
Kiribati	10·6 (6·5-14·7)	17·1 (10·0-23·4)	17·4 (10·2-23·8)	22·3 (13·1-30·6)	9·7 (1·7-23·8)	9·8 (0·0-33·5)	10·0 (0·0-34·0)	13·5 (0·0-45·3)
Marshall Islands	29·6 (26·0-33·4)	45·0 (39·2-50·1)	45·7 (39·9-50·8)	55·8 (49·6-61·0)	22·6 (15·3-31·5)	30·1 (22·2-39·4)	31·0 (23·4-40·5)	42·2 (28·7-56·6)
Papua New Guinea	6·8 (3·5-10·1)	13·5 (9·5-17·2)	14·2 (10·2-17·9)	19·8 (13·1-25·4)	8·8 (0·0-25·6)	12·0 (3·9-27·7)	12·5 (4·3-28·0)	20·5 (5·8-37·0)
Samoa	44·6 (38·2-50·0)	51·1 (44·6-56·3)	51·4 (44·9-56·6)	56·0 (49·7-61·7)	33·2 (17·9-40·7)	38·6 (22·0-46·9)	39·0 (22·5-47·2)	44·4 (27·5-56·0)
Solomon Islands	18·8 (13·4-25·7)	26·3 (20·9-32·5)	27·0 (21·7-33·2)	36·1 (30·6-42·4)	11·0 (0·2-25·4)	13·9 (5·3-26·1)	14·6 (5·8-26·5)	23·2 (10·7-35·5)
Tonga	40·9 (37·0-44·6)	48·7 (44·3-53·4)	49·0 (44·7-53·8)	55·5 (50·4-61·6)	11·9 (6·4-16·5)	18·3 (9·4-24·8)	18·8 (9·7-25·3)	25·8 (10·4-38·8)
Vanuatu	17·9 (13·1-23·9)	26·1 (21·3-31·2)	26·7 (21·9-31·8)	35·0 (29·7-40·1)	10·9 (2·0-25·8)	15·9 (8·4-27·3)	16·1 (8·7-27·5)	22·4 (10·3-34·8)
**North Africa and Middle East**								
Afghanistan	21·7 (18·0-25·0)	31·7 (28·0-35·7)	32·2 (28·5-36·1)	38·9 (35·0-42·6)	19·3 (13·0-26·0)	27·4 (20·5-35·1)	27·9 (20·8-35·6)	35·9 (22·2-49·1)
Algeria	52·5 (49·5-55·9)	61·7 (57·9-66·4)	62·3 (58·3-66·9)	70·0 (65·2-75·7)	33·9 (18·6-48·6)	49·0 (34·1-65·2)	49·9 (34·5-66·1)	63·4 (44·0-83·5)
Bahrain	57·1 (52·6-61·6)	63·3 (58·7-67·9)	63·7 (59·0-68·3)	68·9 (63·6-74·3)	50·9 (46·4-56·7)	71·6 (61·8-78·4)	71·4 (61·7-78·1)	90·8 (72·0-100·0)
Egypt	34·7 (30·2-38·9)	43·3 (37·5-49·1)	44·1 (38·2-50·0)	55·4 (47·0-63·0)	73·4 (65·2-88·2)	85·0 (71·0-100·0)	86·0 (70·5-100·0)	96·2 (76·4-100·0)
Iran	53·9 (49·5-58·0)	64·5 (59·7-69·0)	64·9 (60·0-69·4)	74·9 (67·8-82·3)	3·3 (0·0-9·1)	27·2 (15·2-34·4)	27·7 (15·7-35·0)	52·0 (25·5-68·6)
Iraq	39·0 (34·8-44·1)	47·0 (42·4-52·0)	47·6 (43·0-52·6)	56·3 (50·2-62·2)	40·1 (32·9-49·2)	49·6 (40·8-57·8)	50·5 (41·5-58·7)	62·0 (45·7-77·8)
Jordan	61·3 (58·4-64·0)	69·5 (66·3-72·7)	70·0 (66·7-73·3)	77·3 (72·7-81·8)	30·8 (24·1-38·7)	52·3 (44·9-58·9)	52·3 (44·7-59·3)	68·5 (53·3-83·7)
Kuwait	71·1 (68·1-74·3)	70·4 (66·2-74·4)	70·6 (66·4-74·7)	74·1 (68·5-79·5)	71·0 (63·0-75·4)	83·4 (76·8-90·2)	83·9 (76·0-91·9)	96·0 (78·4-100·0)
Lebanon	58·6 (49·1-64·8)	67·7 (61·5-73·0)	68·3 (62·2-73·6)	77·3 (71·8-82·6)	57·2 (44·6-73·9)	77·1 (63·2-95·7)	77·8 (63·6-96·0)	92·5 (72·0-100·0)
Libya	56·5 (53·3-59·3)	65·0 (61·3-68·3)	65·2 (61·5-68·6)	75·5 (69·7-82·6)	45·0 (32·6-62·7)	56·5 (42·8-78·3)	56·6 (42·7-79·1)	65·3 (44·9-98·9)
Morocco	35·1 (31·2-38·9)	48·3 (44·0-52·8)	49·2 (45·0-53·7)	62·0 (57·2-66·9)	33·2 (21·6-47·8)	46·6 (36·4-61·8)	47·4 (37·1-62·3)	60·4 (46·8-77·9)
Palestine	51·3 (48·3-54·1)	53·0 (47·2-56·9)	53·1 (47·3-57·1)	55·6 (48·1-60·6)	50·9 (45·0-65·3)	69·0 (58·2-74·3)	69·4 (58·8-75·0)	80·0 (64·5-95·3)
Oman	49·5 (46·4-52·8)	63·6 (60·5-66·9)	64·4 (61·2-67·7)	78·5 (73·7-83·3)	54·4 (31·7-80·2)	66·0 (52·0-90·9)	67·8 (52·8-93·6)	84·5 (62·8-100·0)
Qatar	76·2 (72·3-79·6)	82·9 (79·0-86·8)	83·1 (79·2-87·0)	86·1 (81·3-90·6)	62·9 (52·5-70·4)	70·5 (59·8-86·1)	70·4 (59·1-86·3)	77·8 (49·3-100·0)
Saudi Arabia	44·2 (40·4-47·7)	55·4 (51·3-59·8)	56·4 (52·1-60·9)	68·3 (62·7-74·8)	33·9 (27·2-42·1)	53·2 (44·1-64·2)	54·4 (45·0-65·7)	73·3 (55·8-93·2)
Sudan	18·6 (14·0-25·2)	28·4 (22·9-35·0)	29·3 (23·6-36·0)	37·8 (30·3-45·6)	10·9 (0·0-30·3)	22·8 (7·0-41·4)	23·8 (7·7-42·5)	37·0 (12·3-59·7)
Syria	67·5 (64·3-70·9)	74·7 (71·5-78·1)	75·1 (71·9-78·6)	81·7 (77·3-86·3)	47·0 (40·9-50·2)	56·7 (50·3-63·9)	57·2 (50·6-64·7)	65·9 (51·7-81·8)
Tunisia	56·4 (51·2-60·9)	60·7 (54·5-66·2)	61·0 (54·8-66·5)	65·5 (58·0-71·7)	40·9 (28·4-56·8)	55·8 (44·3-73·3)	56·7 (45·4-74·3)	71·4 (54·3-92·0)
Turkey	47·7 (44·0-51·5)	70·7 (67·1-74·6)	71·4 (67·7-75·3)	82·7 (76·6-93·2)	58·6 (48·9-70·4)	82·9 (75·0-94·3)	83·4 (74·9-94·9)	94·3 (75·2-100·0)
United Arab Emirates	57·2 (48·1-64·9)	62·1 (52·8-70·1)	62·2 (52·9-70·3)	67·0 (56·7-75·8)	46·9 (40·2-54·8)	59·8 (51·7-69·0)	60·4 (52·4-69·5)	67·0 (49·7-84·4)
Yemen	16·5 (11·8-22·2)	29·5 (24·8-35·0)	30·4 (25·6-35·9)	43·5 (37·3-49·8)	9·0 (0·0-27·0)	24·0 (10·0-38·3)	24·4 (10·3-38·9)	39·8 (21·0-57·3)
**South Asia**								

Location	Indicator 3.a.1: Age-standardised prevalence of daily smoking in populations aged 10 and older (%)				Indicator 3.b.1: Geometric mean of the coverage of eight vaccines, conditional on inclusion in national vaccine schedules, in target populations (%)			
	**2000**	**2015**	**2016**	**2030**	**2000**	**2015**	**2016**	**2030**
Kiribati	0·0 (0·0-0·0)	4·6 (0·0-13·0)	5·7 (0·0-15·0)	25·3 (3·6-45·3)	75·7 (70·3-80·2)	85·6 (78·8-90·0)	85·7 (78·6-90·4)	90·1 (75·0-97·2)
Marshall Islands	57·2 (53·0-61·3)	61·0 (55·2-66·3)	61·1 (55·4-66·3)	64·1 (54·3-72·3)	14·1 (5·3-22·8)	32·8 (16·7-46·8)	35·8 (17·8-51·5)	66·4 (29·2-90·4)
Papua New Guinea	5·4 (0·0-12·9)	16·6 (7·4-25·6)	17·9 (8·5-26·7)	19·7 (3·0-35·8)	38·3 (31·7-45·0)	52·7 (42·2-62·5)	46·5 (32·5-58·6)	59·1 (29·3-79·7)
Samoa	14·6 (8·4-20·7)	25·0 (17·3-31·9)	25·5 (17·8-32·5)	33·9 (20·6-45·8)	16·6 (8·8-24·6)	15·6 (3·8-27·3)	17·0 (3·0-30·9)	44·2 (3·7-76·4)
Solomon Islands	29·7 (22·8-36·2)	35·5 (27·9-43·0)	35·9 (28·1-43·4)	40·1 (26·2-53·0)	67·3 (61·5-72·4)	76·6 (69·0-82·6)	77·9 (70·2-84·2)	89·2 (74·3-97·1)
Tonga	25·3 (18·8-31·6)	35·0 (28·1-41·3)	35·7 (28·6-42·0)	44·1 (32·1-54·6)	96·0 (94·0-97·4)	99·4 (98·7-99·8)	99·5 (98·8-99·9)	100·0 (99·6-100·0)
Vanuatu	51·5 (47·1-55·8)	61·0 (56·9-65·1)	60·9 (56·9-65·2)	60·4 (50·9-69·3)	43·3 (36·8-49·7)	50·0 (39·1-60·0)	49·5 (37·3-60·7)	51·0 (21·7-74·8)
**North Africa and Middle East**								
Afghanistan	76·1 (67·6-82·2)	76·4 (70·1-81·4)	76·5 (70·4-81·4)	76·2 (64·4-85·8)	0·0 (0·0-0·0)	46·2 (40·2-51·8)	50·3 (43·9-56·4)	88·2 (81·1-93·2)
Algeria	69·4 (63·7-74·4)	75·0 (69·9-79·6)	75·3 (70·0-79·8)	80·1 (71·9-87·4)	91·3 (89·1-93·0)	99·0 (98·6-99·3)	99·1 (98·7-99·4)	100·0 (99·9-100·0)
Bahrain	76·3 (72·2-79·9)	79·1 (73·3-83·9)	79·1 (73·0-84·1)	85·0 (75·5-92·5)	83·3 (75·8-88·7)	95·3 (93·6-96·6)	95·2 (93·3-96·6)	99·2 (98·0-99·9)
Egypt	54·8 (50·8-58·7)	49·6 (45·3-53·8)	50·3 (45·8-54·6)	49·7 (39·5-59·3)	92·3 (91·0-93·4)	95·9 (94·8-96·7)	96·4 (95·4-97·2)	98·2 (97·0-99·1)
Iran	68·3 (62·7-73·5)	73·4 (68·9-77·1)	73·5 (69·0-77·4)	74·3 (64·0-82·8)	98·4 (97·6-99·0)	100·0 (99·8-100·0)	100·0 (99·9-100·0)	100·0 (100·0-100·0)
Iraq	51·7 (45·3-57·0)	59·5 (51·6-66·0)	59·6 (51·6-65·9)	61·3 (47·5-72·7)	57·7 (52·6-62·0)	65·3 (58·9-70·5)	68·8 (62·7-74·2)	78·2 (68·7-86·1)
Jordan	33·3 (25·6-40·0)	30·4 (21·3-38·0)	31·2 (21·6-39·5)	34·3 (16·2-50·1)	88·1 (80·9-93·0)	99·3 (98·9-99·6)	99·3 (98·9-99·5)	100·0 (99·8-100·0)
Kuwait	47·3 (41·8-52·4)	53·5 (49·8-56·8)	53·9 (49·5-57·7)	59·0 (51·1-66·2)	77·9 (72·4-82·4)	90·7 (88·1-92·7)	92·1 (89·7-93·9)	98·2 (96·2-99·3)
Lebanon	24·3 (11·9-35·7)	16·6 (2·3-28·6)	18·6 (4·3-31·5)	11·9 (0·0-36·0)	96·7 (94·4-98·0)	99·5 (99·2-99·8)	99·6 (99·3-99·8)	100·0 (100·0-100·0)
Libya	57·3 (52·1-62·5)	59·8 (55·2-64·5)	60·6 (55·7-65·4)	60·0 (50·7-68·1)	94·1 (92·5-95·5)	97·7 (96·6-98·5)	97·5 (96·2-98·4)	97·3 (94·1-99·1)
Morocco	71·3 (68·0-74·4)	76·1 (71·5-80·1)	76·2 (71·7-80·3)	80·5 (72·9-86·8)	87·0 (85·0-88·7)	92·4 (90·3-94·1)	93·8 (92·0-95·3)	98·4 (97·3-99·2)
Palestine	39·7 (36·0-43·3)	46·0 (39·2-52·2)	45·9 (38·6-52·5)	48·9 (37·0-60·1)	94·9 (93·8-95·8)	95·2 (93·6-96·6)	95·5 (94·0-96·9)	97·5 (94·6-99·1)
Oman	85·2 (82·7-87·4)	84·7 (80·9-88·3)	84·9 (81·0-88·5)	83·6 (74·2-91·6)	99·8 (99·7-100·0)	100·0 (99·8-100·0)	100·0 (99·9-100·0)	100·0 (100·0-100·0)
Qatar	66·8 (62·4-70·9)	66·4 (61·9-70·4)	67·0 (62·3-71·3)	71·6 (61·1-81·2)	87·2 (84·4-89·6)	96·9 (95·8-97·6)	97·0 (95·9-97·8)	99·0 (97·9-99·6)
Saudi Arabia	74·3 (71·9-76·5)	69·7 (67·7-71·9)	70·1 (68·1-72·3)	64·4 (59·2-69·1)	96·5 (95·5-97·3)	99·3 (98·9-99·6)	98·7 (98·1-99·2)	99·8 (99·4-100·0)
Sudan	75·7 (69·9-80·5)	76·4 (69·5-81·6)	76·4 (69·4-81·7)	76·3 (62·8-85·9)	42·5 (38·0-46·8)	68·9 (63·5-73·3)	70·7 (64·7-75·6)	86·2 (78·2-91·8)
Syria	34·4 (28·7-39·7)	45·7 (32·2-55·1)	46·3 (32·4-55·5)	54·5 (34·1-70·2)	85·3 (82·6-87·8)	81·8 (77·7-85·8)	82·0 (77·4-86·0)	79·5 (65·7-89·2)
Tunisia	34·0 (29·4-38·1)	37·8 (29·8-44·1)	38·4 (29·9-44·7)	42·2 (25·9-54·0)	96·7 (95·5-97·6)	99·0 (98·1-99·5)	99·2 (98·5-99·6)	100·0 (99·9-100·0)
Turkey	9·7 (1·5-17·7)	39·9 (32·7-46·2)	40·1 (31·1-47·7)	58·1 (46·4-68·7)	63·9 (60·4-67·3)	78·8 (73·1-83·6)	78·5 (72·4-83·8)	87·7 (75·3-94·7)
United Arab Emirates	71·6 (67·2-75·8)	70·8 (64·8-76·4)	71·1 (65·2-76·7)	71·2 (60·3-80·5)	90·5 (88·4-92·3)	97·2 (96·0-98·2)	97·3 (96·0-98·2)	99·1 (97·9-99·7)
Yemen	48·2 (38·7-56·7)	55·5 (42·6-64·5)	55·6 (43·4-64·5)	55·8 (32·3-72·1)	39·6 (34·7-45·0)	53·1 (44·8-60·4)	54·6 (46·1-62·3)	72·3 (56·8-83·1)
**South Asia**								

Location	Indicator 5.2.1: Age-standardised prevalence of women aged 15 years and older who experienced physical or sexual violence by an intimate partner in the last 12 months (%)				Indicator 6.1.1: Risk-weighted prevalence of populations using unsafe or unimproved water sources, as measured by the summary exposure value (SEV) for unsafe water (%)				Indicator 6.2.1a: Risk-weighted prevalence of populations using unsafe or unimproved sanitation, as measured by the summary exposure value (SEV) for unsafe sanitation (%)				Indicator 6.2.1b: Risk-weighted prevalence of populations without access to a handwashing facility, as measured by the summary exposure value (SEV) for unsafe hygiene (%)			
	**2000**	**2015**	**2016**	**2030**	**2000**	**2015**	**2016**	**2030**	**2000**	**2015**	**2016**	**2030**	**2000**	**2015**	**2016**	**2030**
Kiribati	19·4 (15·6-23·3)	20·6 (17·0-24·3)	20·9 (17·3-24·7)	24·3 (20·2-28·7)	18·5 (11·5-32·7)	29·1 (19·1-46·7)	29·7 (19·6-47·1)	38·3 (26·3-56·5)	25·6 (11·1-45·6)	48·6 (29·3-70·1)	49·8 (30·5-71·2)	68·1 (52·2-85·6)	57·6 (42·9-71·5)	67·3 (52·6-79·9)	67·9 (53·4-80·4)	75·8 (62·1-86·4)
Marshall Islands	35·4 (30·8-39·5)	41·7 (37·5-45·8)	42·1 (37·9-46·2)	47·8 (43·5-51·8)	35·4 (22·4-54·4)	48·5 (31·6-69·3)	49·4 (32·4-70·3)	61·2 (42·0-80·8)	53·3 (33·5-73·0)	74·0 (56·9-87·1)	74·9 (58·0-87·6)	85·7 (73·2-93·0)	74·9 (61·3-85·4)	82·0 (70·6-90·6)	82·4 (71·0-90·9)	87·7 (77·8-94·5)
Papua New Guinea	16·4 (12·4-20·7)	21·3 (17·5-25·4)	21·8 (18·0-25·8)	26·3 (22·1-30·8)	13·0 (6·9-25·0)	18·7 (11·2-31·2)	19·2 (11·7-31·8)	26·9 (18·1-40·8)	14·4 (3·6-30·8)	23·8 (10·3-42·2)	24·6 (10·8-43·2)	37·8 (21·9-57·9)	48·4 (34·4-62·3)	52·8 (38·2-66·9)	53·3 (38·6-67·4)	58·3 (42·4-73·0)
Samoa	39·9 (35·2-44·1)	45·9 (41·9-49·5)	46·2 (42·3-49·8)	50·9 (46·7-54·7)	46·1 (29·5-71·4)	55·2 (35·1-81·3)	55·8 (35·4-81·8)	63·3 (38·9-88·2)	80·2 (64·1-91·1)	83·9 (68·2-94·4)	84·0 (68·3-94·5)	85·8 (69·7-95·8)	86·0 (76·7-93·0)	89·5 (81·6-95·5)	89·7 (81·8-95·6)	92·1 (85·0-97·3)
Solomon Islands	20·1 (15·9-24·1)	25·1 (21·0-28·9)	25·6 (21·5-29·4)	31·0 (26·3-35·5)	26·2 (17·0-45·5)	34·6 (22·6-56·0)	35·4 (23·2-56·9)	46·8 (32·2-68·4)	20·2 (8·3-36·4)	33·4 (16·8-53·6)	34·7 (17·7-55·4)	52·3 (31·4-75·2)	70·6 (56·8-82·4)	76·5 (64·4-86·4)	77·1 (65·3-86·9)	83·3 (72·4-91·5)
Tonga	43·7 (39·3-48·2)	50·5 (46·6-54·3)	50·7 (46·8-54·4)	54·3 (49·8-58·4)	37·5 (23·9-56·4)	49·6 (32·2-70·0)	50·4 (32·8-70·7)	60·1 (40·8-79·7)	57·1 (37·0-77·5)	75·5 (59·2-88·3)	76·2 (60·2-88·8)	87·6 (76·9-95·0)	75·9 (63·5-86·0)	83·0 (72·6-91·0)	83·4 (73·2-91·2)	88·8 (80·7-94·8)
Vanuatu	40·1 (35·9-44·3)	46·5 (42·5-50·3)	46·9 (42·9-50·7)	51·9 (47·5-55·6)	18·4 (12·4-29·3)	27·5 (18·6-41·0)	28·2 (19·1-41·7)	37·1 (26·1-51·1)	23·8 (7·8-41·1)	41·3 (21·7-61·7)	42·4 (23·0-62·9)	58·9 (41·8-79·5)	54·5 (38·4-69·4)	63·5 (48·5-77·0)	64·2 (49·3-77·4)	72·6 (58·8-83·5)
**North Africa and Middle East**																
Afghanistan	0·0 (0·0-0·0)	0·0 (0·0-0·0)	0·0 (0·0-0·0)	0·0 (0·0-0·0)	3·5 (0·0-8·0)	5·7 (1·4-10·1)	5·9 (1·6-10·3)	8·9 (4·0-14·0)	5·1 (0·0-12·7)	15·5 (6·0-23·6)	16·5 (6·5-24·9)	33·5 (22·9-44·8)	35·4 (33·6-37·1)	37·5 (35·8-39·3)	37·7 (35·9-39·4)	40·1 (37·6-42·1)
Algeria	30·2 (25·9-34·4)	42·7 (38·5-46·5)	43·6 (39·2-47·4)	55·1 (48·9-59·9)	51·4 (44·7-57·8)	74·4 (67·2-79·1)	75·6 (68·6-80·1)	88·3 (83·3-91·0)	76·0 (69·1-83·3)	91·4 (83·7-95·5)	91·9 (84·0-95·9)	96·8 (88·7-99·4)	80·0 (78·8-81·1)	85·8 (84·9-86·7)	86·2 (85·3-87·1)	91·4 (88·2-92·7)
Bahrain	26·3 (19·7-32·3)	24·6 (17·4-30·8)	24·9 (17·8-30·9)	26·5 (19·6-32·4)	69·3 (58·6-76·3)	77·4 (69·3-82·8)	77·8 (69·7-83·1)	81·8 (75·6-86·5)	86·2 (78·1-92·1)	91·3 (84·1-95·4)	91·5 (84·3-95·5)	94·0 (87·6-97·4)	86·9 (86·0-87·8)	86·9 (85·9-87·8)	87·0 (86·0-87·9)	86·8 (85·5-88·5)
Egypt	43·2 (39·5-46·5)	38·9 (35·1-42·5)	39·2 (35·4-42·8)	42·8 (37·3-47·8)	57·6 (53·3-61·7)	76·8 (73·0-79·4)	77·6 (73·8-80·1)	86·6 (83·4-88·6)	51·1 (27·0-69·4)	65·8 (45·8-80·3)	66·5 (46·6-80·9)	76·3 (62·2-87·8)	84·0 (82·9-85·0)	88·9 (88·1-89·7)	89·0 (88·2-89·8)	92·2 (90·4-93·3)
Iran	38·2 (33·5-42·7)	44·3 (39·7-48·6)	44·9 (40·3-49·2)	51·3 (42·9-58·2)	74·4 (68·6-79·2)	81·0 (75·8-85·4)	81·3 (76·1-85·7)	84·7 (79·7-88·7)	87·6 (78·4-93·2)	92·2 (84·3-96·4)	92·4 (84·6-96·5)	95·5 (89·4-98·6)	89·4 (88·6-90·3)	89·5 (88·6-90·3)	89·5 (88·7-90·3)	90·4 (89·6-91·2)
Iraq	1·2 (0·0-6·0)	0·0 (0·0-0·0)	0·0 (0·0-0·0)	0·0 (0·0-0·0)	48·8 (44·0-54·0)	43·0 (35·2-50·3)	43·8 (35·6-51·3)	54·5 (42·0-63·3)	67·1 (63·7-70·8)	67·2 (62·5-72·0)	68·6 (63·8-73·5)	84·6 (79·8-89·2)	94·8 (94·2-95·3)	91·8 (91·0-92·4)	91·9 (91·2-92·5)	94·0 (93·4-94·6)
Jordan	30·3 (25·7-35·0)	36·0 (32·1-39·9)	36·3 (32·4-40·3)	41·8 (37·4-46·0)	66·7 (59·6-72·9)	80·1 (71·7-85·0)	80·8 (72·7-85·6)	88·6 (81·2-92·1)	87·9 (80·1-92·9)	95·7 (91·3-98·3)	96·2 (92·0-98·6)	99·1 (96·7-100·0)	87·0 (86·1-87·9)	87·3 (86·4-88·2)	87·5 (86·5-88·4)	88·6 (87·6-90·0)
Kuwait	34·8 (28·6-41·3)	47·6 (41·7-53·2)	48·4 (42·6-53·8)	59·5 (53·9-65·0)	75·4 (67·2-80·7)	84·2 (79·2-88·3)	84·6 (79·7-88·7)	89·3 (85·6-92·7)	88·9 (78·9-94·7)	92·6 (84·5-97·3)	92·7 (84·6-97·4)	95·5 (88·5-99·1)	89·2 (88·4-89·9)	90·3 (89·4-91·0)	90·4 (89·6-91·2)	92·3 (90·6-93·2)
Lebanon	45·1 (41·0-48·7)	53·0 (49·5-56·2)	53·5 (50·0-56·6)	59·2 (55·4-62·6)	51·9 (44·3-59·2)	63·1 (46·7-75·1)	64·5 (47·7-76·3)	80·3 (51·1-94·3)	89·2 (78·9-95·0)	92·7 (84·5-97·5)	92·8 (84·6-97·5)	94·8 (87·9-98·5)	75·2 (73·7-76·6)	77·7 (76·3-78·9)	78·4 (77·1-79·7)	86·2 (82·8-87·8)
Libya	39·4 (34·7-43·7)	49·5 (45·3-53·2)	49·9 (45·8-53·5)	58·7 (53·5-63·3)	72·3 (66·2-77·7)	83·1 (78·0-87·5)	83·3 (78·2-87·7)	85·9 (71·9-94·7)	56·5 (49·8-63·7)	82·1 (77·1-87·8)	82·7 (77·9-88·3)	92·6 (88·8-95·9)	88·1 (87·1-89·0)	90·2 (89·4-91·0)	90·3 (89·4-91·1)	91·2 (90·4-92·0)
Morocco	29·5 (25·1-33·4)	38·6 (34·6-42·0)	39·3 (35·3-42·7)	47·8 (43·1-51·8)	30·7 (25·3-36·3)	56·4 (42·8-67·3)	57·9 (44·2-68·6)	75·0 (60·5-84·2)	64·5 (60·3-68·7)	84·2 (81·5-87·3)	85·1 (82·5-88·1)	94·4 (92·8-96·0)	67·3 (65·8-68·8)	77·1 (75·8-78·3)	77·7 (76·5-79·0)	86·1 (85·0-87·1)
Palestine	19·6 (15·0-23·9)	5·3 (1·7-8·9)	5·6 (1·9-9·2)	8·2 (3·6-12·7)	50·9 (41·8-57·9)	35·1 (28·5-42·1)	35·6 (28·9-42·7)	42·9 (33·7-50·9)	87·7 (85·5-89·6)	82·0 (79·2-84·5)	82·7 (80·1-85·2)	90·9 (89·5-92·4)	81·0 (79·9-82·2)	68·7 (67·1-70·2)	68·9 (67·3-70·4)	71·4 (69·9-73·0)
Oman	17·7 (12·4-23·0)	27·6 (19·0-35·0)	29·0 (20·6-36·4)	38·0 (22·6-49·9)	16·3 (5·3-30·4)	67·7 (53·8-78·9)	69·7 (56·3-80·5)	89·6 (82·2-94·4)	89·0 (79·9-94·5)	92·9 (84·7-97·5)	93·0 (84·8-97·5)	95·0 (88·3-98·6)	54·8 (53·0-56·5)	82·4 (81·2-83·6)	83·4 (82·2-84·5)	95·0 (93·8-96·3)
Qatar	34·6 (27·0-41·0)	27·5 (18·6-35·3)	28·4 (20·0-35·8)	36·0 (25·5-42·9)	74·4 (65·2-80·2)	81·5 (76·2-86·0)	81·9 (76·6-86·2)	85·8 (82·4-89·3)	88·5 (78·9-94·0)	92·3 (84·0-96·8)	92·4 (84·2-96·9)	95·0 (88·3-98·7)	88·8 (88·0-89·6)	88·8 (87·9-89·6)	88·9 (88·0-89·7)	89·4 (88·5-90·6)
Saudi Arabia	33·1 (28·2-38·0)	51·3 (47·3-54·9)	52·7 (48·9-56·2)	68·9 (64·7-72·4)	39·0 (32·6-45·4)	69·4 (64·7-73·1)	71·2 (66·8-74·7)	88·4 (86·2-90·1)	63·0 (57·8-67·6)	91·7 (86·7-95·5)	92·2 (87·2-95·9)	99·4 (97·4-100·0)	69·4 (68·8-70·1)	81·5 (81·0-82·0)	82·6 (82·2-83·1)	92·4 (91·4-93·0)
Sudan	19·5 (15·3-23·5)	21·6 (17·8-25·3)	22·4 (18·5-26·0)	27·4 (23·4-31·5)	14·0 (7·2-20·8)	24·9 (14·5-36·1)	26·2 (15·9-37·3)	43·2 (31·0-54·7)	6·1 (0·0-14·0)	10·9 (0·7-19·1)	11·6 (0·9-20·0)	20·4 (0·0-33·8)	22·7 (21·2-24·2)	27·2 (25·7-28·8)	28·0 (26·5-29·6)	33·6 (26·9-37·2)
Syria	16·3 (12·0-20·7)	29·3 (25·4-33·4)	29·8 (25·8-33·8)	41·0 (36·4-45·7)	51·3 (45·6-56·4)	71·3 (63·6-76·5)	72·3 (64·5-77·3)	83·0 (75·2-87·0)	72·7 (65·4-80·2)	88·6 (80·0-93·9)	88·9 (80·0-94·2)	95·9 (87·3-99·1)	85·1 (84·1-86·1)	86·4 (85·4-87·4)	86·7 (85·7-87·6)	89·8 (89·0-90·6)
Tunisia	40·7 (36·9-44·6)	45·6 (41·7-49·2)	46·3 (42·4-49·9)	54·9 (49·9-59·3)	55·4 (49·7-60·5)	62·8 (54·1-69·1)	63·6 (55·1-70·0)	73·8 (63·4-80·0)	82·9 (70·9-90·4)	84·4 (74·2-91·3)	84·6 (74·3-91·4)	87·3 (76·7-93·9)	81·9 (80·7-83·1)	81·8 (80·6-82·9)	82·3 (81·1-83·3)	86·2 (85·2-87·1)
Turkey	43·0 (39·0-46·4)	50·1 (46·6-53·5)	50·5 (46·9-54·0)	56·2 (51·9-60·0)	44·1 (37·1-52·7)	63·0 (49·4-73·0)	64·5 (50·7-74·3)	81·2 (66·3-87·8)	74·1 (66·9-80·2)	88·0 (79·7-93·1)	88·5 (79·9-93·5)	94·8 (84·9-98·1)	71·7 (70·2-73·2)	78·0 (76·7-79·3)	78·8 (77·6-80·1)	85·0 (83·9-86·1)
United Arab Emirates	26·0 (17·4-33·5)	25·3 (16·5-33·2)	26·4 (18·0-34·0)	34·6 (24·1-44·1)	69·2 (58·8-76·0)	78·2 (70·4-83·4)	78·6 (71·0-83·8)	84·2 (78·3-88·1)	90·4 (81·2-96·3)	92·9 (84·5-97·5)	93·0 (84·6-97·5)	94·6 (87·2-98·4)	85·4 (84·4-86·4)	87·2 (86·2-88·1)	87·4 (86·4-88·3)	90·1 (89·4-90·8)
Yemen	0·0 (0·0-0·0)	3·9 (0·0-9·3)	4·5 (0·0-9·9)	13·4 (6·6-19·6)	8·0 (3·2-12·2)	15·8 (8·7-23·6)	16·9 (9·6-24·7)	30·8 (21·9-39·2)	5·3 (2·0-8·8)	36·1 (32·0-40·4)	38·7 (34·5-43·1)	75·7 (70·9-80·5)	45·4 (43·6-47·2)	45·8 (44·0-47·6)	46·5 (44·7-48·3)	49·6 (47·9-51·4)
**South Asia**																

Location	**Indicator 7.1.2:** Risk-weighted prevalence of household air pollution, as measured by the summary exposure value (SEV) for household air pollution (%)				**Indicator 8.8.1:** Age-standardised all-cause disability-adjusted life year (DALY) rates attributable to occupational risks (per 100,000 population)				**Indicator 11.6.2:** Population-weighted mean levels of fine particulate matter smaller than 2.5 microns in diameter (PM2.5)				**Indicator 16.1.1:** Age-standardised death rate due to interpersonal violence (per 100,000 population)			
	**2000**	**2015**	**2016**	**2030**	**2000**	**2015**	**2016**	**2030**	**2000**	**2015**	**2016**	**2030**	**2000**	**2015**	**2016**	**2030**
Kiribati	19·2 (9·9-29·1)	33·6 (23·3-44·1)	34·8 (24·5-45·3)	50·7 (41·0-60·0)	65·8 (53·7-77·5)	50·6 (37·2-62·5)	50·6 (37·1-62·6)	50·9 (36·8-63·4)	99·6 (93·7-100·0)	99·8 (97·0-100·0)	99·8 (96·5-100·0)	99·7 (95·8-100·0)	55·2 (42·7-60·9)	54·9 (42·4-62·1)	55·0 (42·7-62·5)	56·4 (42·2-68·6)
Marshall Islands	73·9 (64·6-81·3)	85·1 (77·6-90·9)	85·9 (78·7-91·6)	93·9 (89·8-97·2)	35·6 (22·1-49·6)	32·6 (14·7-51·6)	33·0 (14·9-52·2)	38·4 (17·4-61·7)	81·5 (37·9-100·0)	81·5 (38·9-100·0)	81·3 (40·0-100·0)	80·7 (38·2-100·0)	40·4 (33·4-47·4)	40·8 (32·4-49·6)	41·1 (32·7-49·8)	44·0 (31·5-57·1)
Papua New Guinea	20·7 (10·5-32·0)	34·7 (23·8-47·0)	35·9 (25·0-48·3)	52·0 (42·0-63·6)	0·8 (0·0-7·1)	2·0 (0·0-11·1)	2·3 (0·0-11·7)	9·3 (0·0-21·1)	64·5 (60·6-67·3)	69·2 (66·0-71·8)	69·3 (66·2-72·0)	70·2 (64·2-80·1)	34·8 (27·4-43·7)	35·0 (27·5-44·0)	35·2 (27·8-44·3)	37·3 (25·1-49·3)
Samoa	53·0 (43·0-62·5)	67·8 (57·1-77·3)	69·1 (58·3-78·4)	83·4 (74·1-90·2)	43·4 (35·6-51·2)	65·4 (56·8-73·9)	66·7 (58·1-75·4)	86·2 (76·1-96·5)	100·0 (100·0-100·0)	100·0 (100·0-100·0)	100·0 (100·0-100·0)	100·0 (100·0-100·0)	45·2 (37·0-54·6)	47·5 (38·8-56·6)	47·8 (38·8-57·1)	49·2 (36·5-61·8)
Solomon Islands	20·9 (12·0-30·1)	31·7 (22·4-42·1)	32·8 (23·6-43·2)	48·6 (40·8-57·0)	50·7 (38·1-64·0)	43·3 (29·1-57·2)	42·9 (28·6-57·1)	38·2 (22·0-53·6)	91·9 (77·9-100·0)	87·0 (72·5-96·3)	85·9 (71·0-95·3)	85·1 (69·4-96·7)	40·6 (33·2-49·7)	39·2 (31·2-49·6)	39·4 (31·3-49·8)	40·9 (27·8-53·8)
Tonga	53·9 (43·0-65·0)	76·3 (66·8-85·1)	77·3 (68·0-85·9)	88·1 (81·7-93·7)	45·9 (38·2-53·7)	52·9 (43·8-62·2)	53·2 (44·0-62·4)	57·2 (47·9-66·8)	99·7 (96·6-100·0)	100·0 (100·0-100·0)	100·0 (100·0-100·0)	100·0 (100·0-100·0)	52·4 (30·6-61·1)	58·7 (32·3-69·4)	58·6 (32·3-69·5)	57·8 (31·9-73·7)
Vanuatu	28·0 (19·2-37·3)	42·8 (32·0-54·0)	43·9 (33·0-55·2)	58·8 (48·6-70·2)	1·4 (0·0-7·9)	7·2 (0·0-16·8)	8·0 (0·0-17·7)	18·8 (6·3-30·7)	79·9 (60·7-92·1)	85·5 (64·7-98·8)	85·1 (65·0-97·6)	83·5 (60·5-100·0)	75·4 (69·0-81·7)	75·9 (68·0-82·9)	76·0 (68·2-82·9)	77·0 (64·7-88·5)
**North Africa and Middle East**																
Afghanistan	3·0 (0·0-13·3)	25·8 (15·0-35·9)	27·2 (16·6-37·3)	48·4 (39·6-56·3)	0·0 (0·0-0·0)	0·1 (0·0-0·0)	0·1 (0·0-1·6)	14·6 (3·7-25·6)	21·6 (20·2-23·1)	18·2 (16·6-19·8)	17·8 (16·3-19·3)	17·9 (15·4-22·2)	10·3 (3·6-22·0)	11·5 (4·5-24·3)	11·6 (4·8-24·2)	13·3 (2·4-27·3)
Algeria	96·8 (94·9-98·2)	99·7 (99·5-99·9)	99·8 (99·6-99·9)	100·0 (100·0-100·0)	47·9 (35·8-59·2)	68·7 (57·7-78·0)	70·1 (58·8-79·6)	89·2 (75·7-100·0)	40·4 (37·8-42·2)	35·6 (33·6-37·4)	35·5 (33·2-37·3)	38·9 (36·2-41·3)	76·1 (69·4-85·4)	81·8 (75·0-89·8)	82·1 (75·2-90·4)	86·3 (73·6-98·3)
Bahrain	96·5 (94·0-98·1)	99·2 (98·6-99·6)	99·3 (98·7-99·6)	99·7 (99·6-99·9)	51·1 (42·6-59·6)	57·1 (48·2-65·7)	57·8 (48·7-66·7)	68·7 (57·4-79·8)	18·1 (10·4-25·0)	10·2 (2·3-17·9)	10·0 (2·0-17·8)	10·1 (1·6-18·2)	63·9 (58·1-69·6)	70·0 (62·1-78·2)	70·1 (62·2-78·7)	73·2 (55·2-88·9)
Egypt	98·5 (97·7-99·2)	99·8 (99·7-99·9)	99·8 (99·7-99·9)	100·0 (100·0-100·0)	75·3 (67·8-82·7)	69·0 (61·8-76·7)	70·4 (63·0-78·1)	89·4 (80·4-98·6)	12·5 (9·8-14·9)	0·0 (0·0-0·0)	0·0 (0·0-0·0)	0·0 (0·0-0·0)	98·9 (91·0-100·0)	93·1 (86·1-100·0)	93·4 (86·5-100·0)	95·3 (81·8-100·0)
Iran	98·7 (97·8-99·3)	99·7 (99·5-99·8)	99·7 (99·5-99·9)	99·9 (99·8-100·0)	74·0 (64·1-83·4)	82·7 (74·5-90·3)	83·6 (75·3-91·2)	95·6 (87·0-100·0)	26·9 (25·9-28·0)	26·4 (25·6-27·5)	26·2 (25·3-27·3)	30·5 (28·0-32·2)	59·1 (52·7-68·9)	67·8 (61·3-75·7)	68·4 (61·3-76·2)	82·1 (66·0-98·3)
Iraq	96·2 (94·3-97·6)	98·5 (97·6-99·2)	98·6 (97·7-99·2)	99·6 (99·2-99·8)	40·0 (29·2-50·7)	43·5 (32·2-54·4)	44·5 (33·3-55·4)	58·7 (47·7-69·5)	24·9 (22·4-27·0)	13·1 (10·7-15·5)	12·6 (10·2-15·0)	11·3 (6·4-14·9)	33·2 (25·6-40·6)	35·7 (27·1-45·1)	36·1 (27·4-45·4)	43·1 (27·6-59·5)
Jordan	99·9 (99·8-100·0)	100·0 (100·0-100·0)	100·0 (100·0-100·0)	100·0 (100·0-100·0)	92·6 (82·9-100·0)	100·0 (100·0-100·0)	100·0 (100·0-100·0)	100·0 (100·0-100·0)	43·1 (41·0-45·4)	35·5 (33·2-37·7)	35·5 (33·2-37·6)	37·6 (35·2-40·0)	50·1 (42·8-58·6)	53·5 (45·4-65·6)	53·6 (45·6-65·9)	56·0 (38·8-74·1)
Kuwait	98·0 (96·6-99·0)	99·9 (99·7-99·9)	99·9 (99·8-99·9)	100·0 (100·0-100·0)	48·7 (39·6-57·1)	57·4 (49·2-66·4)	58·4 (49·9-67·7)	72·9 (61·3-85·7)	16·1 (11·8-20·4)	0·6 (0·0-3·7)	0·4 (0·0-3·1)	0·4 (0·0-3·6)	74·1 (67·8-83·6)	86·1 (74·7-94·7)	86·2 (74·8-95·5)	96·6 (78·0-100·0)
Lebanon	100·0 (100·0-100·0)	100·0 (100·0-100·0)	100·0 (100·0-100·0)	100·0 (100·0-100·0)	77·6 (65·3-88·2)	84·5 (72·0-99·0)	84·7 (72·1-99·4)	88·3 (71·6-100·0)	49·4 (46·0-52·6)	40·2 (36·1-43·8)	40·0 (36·1-43·9)	36·2 (30·8-42·2)	50·1 (41·8-62·3)	60·2 (49·2-72·6)	60·2 (49·1-72·9)	64·6 (45·2-82·1)
Libya	98·5 (97·4-99·2)	99·9 (99·7-99·9)	99·9 (99·8-100·0)	100·0 (100·0-100·0)	84·8 (74·8-94·1)	93·5 (84·0-100·0)	93·3 (83·8-100·0)	91·3 (81·5-99·9)	22·8 (19·7-25·4)	16·9 (14·3-19·1)	17·3 (14·8-19·5)	17·7 (15·0-20·1)	69·3 (62·9-77·7)	70·9 (63·3-80·7)	70·8 (63·1-80·6)	72·1 (59·4-85·2)
Morocco	89·1 (85·6-92·0)	98·0 (96·6-98·9)	98·2 (96·9-99·0)	99·6 (99·2-99·8)	49·0 (41·2-57·0)	57·3 (49·4-64·9)	57·8 (49·8-65·5)	64·9 (54·1-74·6)	53·7 (52·3-55·1)	48·6 (47·2-50·1)	48·4 (46·8-49·9)	49·9 (48·3-51·5)	81·6 (75·2-92·4)	83·1 (76·1-95·7)	83·3 (76·2-96·0)	85·7 (73·3-100·0)
Palestine	98·0 (96·5-98·9)	99·4 (99·0-99·7)	99·5 (99·1-99·7)	99·9 (99·8-99·9)	65·8 (54·5-81·9)	59·7 (49·2-71·1)	58·8 (48·7-69·7)	50·6 (25·3-69·0)	61·8 (59·4-64·4)	58·3 (55·6-60·8)	58·4 (55·6-61·1)	58·9 (55·4-63·0)	62·5 (54·7-66·8)	57·1 (51·5-65·5)	56·9 (51·2-65·0)	60·3 (47·8-71·6)
Oman	81·1 (71·7-88·7)	99·1 (98·4-99·5)	99·2 (98·7-99·6)	100·0 (99·9-100·0)	48·5 (37·7-59·2)	68·0 (59·1-77·0)	68·4 (59·5-77·3)	73·8 (64·1-83·3)	16·1 (12·0-19·5)	11·2 (7·8-14·2)	10·5 (7·0-13·6)	11·2 (7·1-15·0)	96·4 (89·0-100·0)	97·9 (91·6-100·0)	97·9 (91·7-100·0)	98·0 (89·2-100·0)
Qatar	100·0 (100·0-100·0)	100·0 (100·0-100·0)	100·0 (100·0-100·0)	100·0 (100·0-100·0)	43·1 (34·3-51·4)	49·4 (40·6-58·5)	49·7 (40·8-58·8)	54·4 (43·8-64·4)	1·1 (0·0-5·4)	0·0 (0·0-0·0)	0·0 (0·0-0·0)	0·0 (0·0-0·0)	85·2 (71·8-92·8)	83·9 (75·1-95·3)	84·0 (74·4-95·6)	85·8 (63·4-100·0)
Saudi Arabia	87·0 (84·3-89·4)	99·6 (99·5-99·7)	99·7 (99·6-99·8)	100·0 (100·0-100·0)	68·6 (63·4-73·7)	91·7 (86·6-97·0)	92·9 (87·8-98·1)	100·0 (100·0-100·0)	4·4 (3·2-5·8)	0·0 (0·0-0·0)	0·0 (0·0-0·0)	0·0 (0·0-0·0)	99·7 (94·5-100·0)	99·8 (97·9-100·0)	99·9 (98·3-100·0)	99·2 (94·6-100·0)
Sudan	22·3 (14·8-30·6)	51·4 (44·7-57·9)	52·7 (46·1-59·1)	69·7 (64·3-74·6)	40·9 (29·5-52·1)	66·1 (55·2-76·7)	67·4 (56·4-77·9)	85·7 (74·1-96·6)	20·9 (19·1-22·6)	11·3 (9·5-13·1)	10·6 (8·6-12·3)	10·6 (8·5-12·6)	97·5 (90·1-100·0)	76·3 (67·2-100·0)	76·6 (67·3-100·0)	81·5 (66·2-100·0)
Syria	99·2 (98·7-99·6)	99·9 (99·9-100·0)	99·9 (99·9-100·0)	100·0 (100·0-100·0)	49·9 (41·9-57·9)	69·3 (59·4-79·1)	69·7 (59·7-79·7)	75·6 (62·0-88·5)	40·3 (35·9-43·6)	30·3 (26·6-33·3)	30·2 (26·4-33·6)	30·7 (23·0-36·1)	58·1 (52·1-69·6)	63·1 (56·5-75·6)	63·2 (56·5-76·0)	65·8 (53·3-81·8)
Tunisia	95·9 (94·0-97·3)	99·8 (99·6-99·9)	99·8 (99·7-99·9)	100·0 (100·0-100·0)	62·6 (53·8-71·2)	73·9 (64·9-82·9)	74·2 (65·1-83·3)	80·0 (68·3-91·5)	37·0 (35·2-38·7)	36·7 (34·9-38·3)	37·0 (35·3-38·8)	40·6 (38·0-42·8)	84·9 (78·4-92·1)	87·4 (79·5-97·0)	87·7 (79·5-97·6)	91·2 (77·0-100·0)
Turkey	93·0 (89·1-96·0)	99·1 (98·4-99·5)	99·2 (98·6-99·6)	99·9 (99·8-100·0)	27·5 (20·9-34·5)	51·0 (42·9-59·1)	52·0 (43·8-60·2)	66·5 (55·9-76·9)	42·5 (41·5-43·6)	35·5 (34·5-36·6)	35·4 (34·3-36·6)	33·5 (30·5-38·2)	58·0 (51·1-66·1)	66·1 (59·0-76·9)	66·0 (58·2-77·2)	73·6 (59·1-90·6)
United Arab Emirates	98·1 (96·6-99·0)	99·7 (99·5-99·9)	99·7 (99·5-99·9)	99·9 (99·9-100·0)	28·8 (20·3-37·1)	37·3 (27·8-45·9)	37·3 (27·7-45·9)	37·8 (26·9-47·6)	12·2 (10·6-13·9)	1·2 (0·0-3·1)	0·5 (0·0-2·1)	0·8 (0·0-3·2)	81·0 (71·8-88·7)	82·6 (73·1-91·2)	82·8 (72·9-91·6)	84·8 (68·4-100·0)
Yemen	55·2 (46·8-62·9)	76·2 (71·1-81·0)	77·4 (72·4-82·0)	89·6 (86·5-92·3)	9·9 (0·0-19·0)	43·6 (34·6-52·8)	44·6 (35·5-53·8)	58·7 (48·5-69·2)	22·7 (17·4-26·3)	13·4 (8·9-16·8)	13·0 (7·9-16·5)	10·9 (5·3-15·3)	55·0 (49·2-61·9)	55·4 (48·6-65·2)	55·3 (48·2-65·2)	56·3 (44·1-69·8)
**South Asia**																

Location	Indicator 16.1.2: Death rate due to conflict and terrorism (per 100,000 population)				Indicator 16.1.3: Age-standardised prevalence of physical or sexual violence experienced by populations in the last 12 months (%)				Indicator 16.2.3: Age-standardised prevalence of women and men aged 18-29 years who experienced sexual violence by age 18 (%)				Indicator 17.19.2c: Percentage of well-certified deaths by a vital registration (VR) system among a country's total population (%)			
	**2000**	**2015**	**2016**	**2030**	**2000**	**2015**	**2016**	**2030**	**2000**	**2015**	**2016**	**2030**	**2000**	**2015**	**2016**	**2030**
Kiribati	100·0 (100·0-100·0)	100·0 (100·0-100·0)	100·0 (100·0-100·0)	100·0 (100·0-100·0)	38·3 (34·8-41·9)	38·2 (34·5-41·8)	38·5 (34·8-42·0)	42·0 (38·0-45·6)	23·3 (9·5-36·3)	21·5 (7·1-34·8)	21·5 (7·0-34·8)	20·4 (5·8-34·1)	37·9 (29·8-46·0)	49·6 (35·4-64·3)	50·3 (36·1-65·4)	62·5 (33·5-89·5)
Marshall Islands	100·0 (100·0-100·0)	100·0 (100·0-100·0)	100·0 (100·0-100·0)	100·0 (100·0-100·0)	59·6 (56·9-62·0)	64·4 (61·9-66·7)	64·7 (62·3-67·1)	69·8 (67·3-72·2)	21·1 (6·5-34·6)	21·2 (6·7-34·6)	21·2 (6·6-34·6)	21·2 (6·7-34·6)	0·0 (0·0-0·0)	0·0 (0·0-0·0)	0·0 (0·0-0·0)	0·0 (0·0-0·0)
Papua New Guinea	100·0 (100·0-100·0)	100·0 (100·0-100·0)	100·0 (100·0-100·0)	100·0 (100·0-100·0)	42·4 (39·2-45·9)	46·5 (43·4-49·9)	47·1 (44·0-50·4)	54·3 (51·3-57·5)	21·6 (6·5-35·0)	21·7 (6·8-35·0)	21·7 (6·8-35·0)	21·9 (6·5-35·1)	23·7 (14·6-35·6)	25·0 (15·5-37·2)	25·7 (16·1-38·2)	26·0 (9·7-49·3)
Samoa	100·0 (100·0-100·0)	100·0 (100·0-100·0)	100·0 (100·0-100·0)	100·0 (100·0-100·0)	63·3 (60·9-65·6)	66·6 (64·2-68·6)	66·8 (64·5-68·9)	69·9 (67·7-72·0)	20·0 (5·1-33·8)	20·6 (6·5-34·4)	20·5 (6·3-34·3)	21·1 (6·5-34·9)	0·0 (0·0-0·0)	0·0 (0·0-0·0)	0·0 (0·0-0·0)	0·0 (0·0-0·0)
Solomon Islands	100·0 (100·0-100·0)	100·0 (100·0-100·0)	100·0 (100·0-100·0)	100·0 (100·0-100·0)	46·6 (43·4-49·7)	49·8 (46·5-52·8)	50·3 (47·1-53·3)	57·2 (53·8-60·1)	13·5 (0·0-30·1)	14·0 (0·0-30·5)	13·9 (0·0-30·3)	15·3 (0·0-31·9)	0·0 (0·0-0·0)	0·0 (0·0-0·0)	0·0 (0·0-0·0)	0·0 (0·0-0·0)
Tonga	100·0 (100·0-100·0)	100·0 (100·0-100·0)	100·0 (100·0-100·0)	100·0 (100·0-100·0)	63·9 (61·5-66·2)	68·7 (66·5-71·0)	69·0 (66·8-71·3)	73·0 (70·6-75·1)	26·7 (10·9-41·4)	27·1 (11·5-41·5)	26·9 (11·3-41·4)	26·4 (10·3-41·6)	53·4 (40·9-65·9)	60·4 (45·5-75·1)	61·0 (46·1-75·2)	67·5 (40·9-90·3)
Vanuatu	100·0 (100·0-100·0)	100·0 (100·0-100·0)	100·0 (100·0-100·0)	100·0 (100·0-100·0)	65·2 (62·9-67·5)	68·8 (66·5-71·0)	69·1 (66·8-71·3)	73·1 (70·7-75·2)	22·3 (8·1-35·5)	22·8 (8·6-36·0)	22·8 (8·7-35·9)	22·7 (8·5-36·0)	0·0 (0·0-0·0)	0·0 (0·0-0·0)	0·0 (0·0-0·0)	0·0 (0·0-0·0)
**North Africa and Middle East**																
Afghanistan	1·2 (0·0-4·3)	0·0 (0·0-0·0)	0·6 (0·0-8·1)	0·4 (0·0-5·5)	0·0 (0·0-0·0)	0·0 (0·0-0·0)	0·0 (0·0-0·0)	0·0 (0·0-0·0)	76·1 (71·3-80·7)	76·1 (71·3-80·7)	76·1 (71·2-80·6)	76·1 (71·2-80·7)	0·0 (0·0-0·0)	0·0 (0·0-0·0)	0·0 (0·0-0·0)	0·0 (0·0-0·0)
Algeria	29·3 (25·9-32·2)	67·1 (62·7-70·2)	73·3 (63·1-87·2)	98·3 (88·7-100·0)	51·6 (49·2-54·3)	64·9 (62·9-67·2)	65·6 (63·5-67·8)	74·1 (72·1-76·2)	76·0 (71·1-80·6)	75·7 (70·6-80·3)	75·6 (70·6-80·3)	75·3 (70·3-80·2)	15·3 (6·6-27·8)	31·8 (15·3-52·0)	32·8 (15·9-53·7)	54·2 (17·6-91·9)
Bahrain	100·0 (100·0-100·0)	100·0 (100·0-100·0)	100·0 (100·0-100·0)	100·0 (100·0-100·0)	58·1 (55·8-60·4)	67·3 (65·3-69·2)	67·5 (65·6-69·4)	70·8 (68·7-72·7)	75·9 (71·1-80·3)	76·1 (71·2-80·9)	76·2 (71·3-80·9)	76·7 (71·9-81·3)	57·3 (50·6-64·2)	60·2 (49·7-70·9)	61·4 (48·4-73·7)	71·0 (43·7-94·6)
Egypt	73·6 (64·0-87·6)	33·0 (22·4-50·7)	46·0 (35·4-65·4)	46·0 (35·4-65·4)	61·2 (59·1-63·6)	62·0 (59·7-64·4)	62·4 (60·1-64·7)	67·6 (64·7-70·2)	76·1 (71·1-80·7)	75·7 (70·7-80·3)	75·7 (70·7-80·3)	75·5 (70·5-80·1)	44·0 (37·1-50·9)	52·0 (43·0-61·7)	53·2 (41·5-64·8)	57·7 (36·9-76·3)
Iran	59·1 (48·6-76·2)	58·7 (48·4-75·1)	73·6 (64·4-88·2)	80·0 (46·7-94·2)	56·7 (54·2-59·1)	66·0 (63·8-68·1)	66·4 (64·2-68·4)	71·2 (69·2-73·2)	76·1 (71·1-80·8)	75·4 (70·5-80·1)	75·4 (70·5-80·2)	74·8 (68·5-80·6)	39·6 (29·1-50·2)	68·4 (55·5-80·0)	68·6 (54·9-81·5)	81·6 (53·3-98·1)
Iraq	50·0 (46·3-52·9)	0·7 (0·0-10·0)	1·0 (0·0-13·1)	10·4 (0·0-46·6)	19·2 (13·5-24·3)	13·3 (7·7-18·5)	13·9 (8·2-19·2)	22·1 (13·9-29·4)	76·0 (71·0-80·6)	76·0 (70·9-80·6)	76·0 (71·0-80·6)	75·9 (71·0-80·5)	33·8 (17·0-53·0)	33·7 (16·9-54·0)	35·1 (17·5-56·3)	48·0 (13·1-92·0)
Jordan	100·0 (100·0-100·0)	100·0 (100·0-100·0)	9·8 (0·0-28·6)	9·8 (0·0-28·6)	54·6 (51·8-57·3)	64·3 (62·2-66·6)	64·5 (62·3-66·8)	67·6 (64·3-70·3)	76·0 (71·0-80·5)	75·9 (70·9-80·5)	75·9 (70·9-80·5)	75·8 (70·7-80·4)	68·7 (53·0-82·8)	71·1 (56·9-83·7)	71·8 (56·3-85·7)	83·3 (49·8-100·0)
Kuwait	61·4 (51·3-77·6)	53·1 (42·9-67·8)	100·0 (100·0-100·0)	100·0 (100·0-100·0)	66·7 (64·4-68·7)	77·6 (75·6-79·3)	78·2 (76·3-79·9)	85·6 (84·0-87·1)	75·5 (70·5-80·4)	75·2 (70·1-80·1)	75·3 (70·3-80·2)	75·0 (69·7-80·1)	83·6 (79·4-87·6)	87·9 (80·7-94·0)	88·2 (80·0-95·1)	92·5 (77·7-100·0)
Lebanon	42·6 (32·2-62·0)	18·2 (8·6-37·3)	18·0 (7·7-37·9)	72·9 (0·0-99·9)	57·9 (55·5-60·3)	71·0 (69·1-73·0)	71·3 (69·4-73·2)	74·7 (72·6-76·7)	75·9 (70·9-80·5)	75·7 (70·6-80·4)	75·6 (70·6-80·4)	75·6 (70·5-80·3)	0·0 (0·0-0·0)	0·0 (0·0-0·0)	0·0 (0·0-0·0)	0·0 (0·0-0·0)
Libya	31·2 (21·3-49·6)	1·0 (0·0-10·2)	3·4 (0·0-19·3)	3·4 (0·0-19·3)	61·3 (58·7-63·5)	70·1 (68·0-72·1)	70·4 (68·2-72·3)	73·4 (71·1-75·4)	75·9 (70·9-80·5)	75·5 (70·5-80·2)	75·6 (70·5-80·2)	75·3 (70·2-80·2)	5·2 (2·0-10·4)	10·9 (4·4-20·9)	10·8 (4·4-21·5)	17·4 (3·3-44·9)
Morocco	100·0 (100·0-100·0)	100·0 (100·0-100·0)	100·0 (100·0-100·0)	100·0 (100·0-100·0)	47·8 (44·8-50·5)	58·7 (56·3-61·1)	59·3 (56·9-61·7)	67·6 (65·4-69·6)	70·0 (63·8-75·3)	67·2 (60·3-73·7)	66·6 (59·5-73·3)	66·3 (59·2-72·8)	26·8 (15·5-42·4)	18·3 (9·8-29·9)	19·1 (9·7-32·9)	34·7 (6·7-76·7)
Palestine	8·5 (0·0-25·2)	32·5 (21·2-53·5)	34·4 (24·1-54·5)	68·9 (22·8-98·0)	41·5 (38·5-44·3)	29·9 (26·4-33·2)	30·5 (27·0-33·8)	38·3 (34·9-41·3)	76·0 (71·1-80·6)	76·1 (71·1-80·6)	76·0 (71·1-80·6)	76·1 (71·2-80·9)	29·1 (19·8-40·2)	29·8 (18·0-43·2)	30·9 (18·2-46·2)	35·5 (11·4-65·9)
Oman	100·0 (100·0-100·0)	100·0 (100·0-100·0)	100·0 (100·0-100·0)	100·0 (100·0-100·0)	53·8 (51·3-56·2)	75·6 (73·9-77·3)	76·5 (74·8-78·1)	90·4 (88·9-91·7)	76·2 (71·4-80·6)	76·0 (71·0-80·8)	75·9 (70·8-80·8)	75·2 (68·7-81·8)	50·0 (31·0-69·2)	63·4 (43·9-80·7)	64·9 (44·5-82·4)	90·6 (57·2-100·0)
Qatar	100·0 (100·0-100·0)	100·0 (100·0-100·0)	100·0 (100·0-100·0)	100·0 (100·0-100·0)	72·7 (70·8-74·5)	79·8 (78·1-81·3)	80·1 (78·4-81·6)	84·8 (83·1-86·4)	75·9 (71·1-80·6)	76·5 (71·0-81·5)	76·6 (71·4-81·6)	76·6 (70·2-82·7)	13·4 (8·5-20·5)	45·1 (32·9-58·5)	45·0 (31·6-60·1)	43·7 (11·4-90·8)
Saudi Arabia	100·0 (100·0-100·0)	19·9 (16·6-22·9)	68·7 (63·3-73·9)	68·7 (63·3-73·9)	63·8 (62·4-65·4)	79·8 (78·7-81·0)	80·5 (79·4-81·7)	90·1 (89·1-91·1)	78·1 (73·5-82·7)	78·5 (74·0-83·1)	78·6 (74·1-83·1)	78·9 (74·5-83·4)	28·8 (26·2-31·4)	45·6 (39·7-52·0)	47·9 (41·3-55·0)	70·2 (57·9-82·0)
Sudan	22·8 (18·9-26·3)	30·7 (21·5-44·4)	22·5 (12·6-38·3)	14·1 (0·0-69·5)	43·0 (40·2-45·9)	48·9 (46·3-51·6)	49·9 (47·4-52·6)	63·3 (61·1-65·7)	76·0 (71·0-80·6)	76·0 (71·0-80·6)	76·0 (71·0-80·6)	75·9 (70·9-80·6)	0·0 (0·0-0·0)	0·0 (0·0-0·0)	0·0 (0·0-0·0)	0·0 (0·0-0·0)
Syria	100·0 (100·0-100·0)	0·2 (0·0-0·0)	0·1 (0·0-0·0)	0·1 (0·0-0·0)	37·8 (34·6-41·0)	54·1 (51·2-56·7)	54·7 (51·9-57·3)	62·7 (60·0-65·2)	76·0 (71·1-80·6)	76·0 (71·0-80·6)	76·1 (71·1-80·7)	76·1 (71·1-80·6)	54·9 (48·9-60·5)	73·3 (56·3-87·9)	74·0 (57·0-89·4)	88·2 (61·8-100·0)
Tunisia	100·0 (100·0-100·0)	35·7 (25·4-54·8)	43·7 (35·0-57·6)	43·7 (35·0-57·6)	57·4 (54·8-59·7)	63·4 (61·1-65·6)	64·0 (61·6-66·1)	71·3 (68·9-73·2)	75·9 (70·9-80·6)	75·6 (70·6-80·3)	75·6 (70·7-80·2)	75·3 (70·2-80·2)	27·1 (13·1-47·0)	33·0 (20·0-50·2)	34·3 (20·5-52·4)	45·6 (16·4-80·3)
Turkey	68·4 (64·4-71·3)	43·3 (39·2-46·8)	34·1 (23·8-51·6)	12·5 (0·0-67·0)	62·0 (58·8-64·8)	71·7 (69·1-74·0)	72·0 (69·3-74·3)	75·7 (72·1-78·8)	79·6 (69·1-89·8)	80·3 (69·8-89·8)	80·4 (69·9-89·8)	81·1 (70·0-90·4)	27·0 (20·3-34·2)	80·7 (67·0-91·0)	80·7 (66·4-92·2)	89·8 (76·2-97·9)
United Arab Emirates	100·0 (100·0-100·0)	100·0 (100·0-100·0)	100·0 (100·0-100·0)	100·0 (100·0-100·0)	71·3 (69·4-73·1)	77·9 (76·2-79·5)	78·1 (76·5-79·7)	81·2 (79·5-82·9)	76·3 (71·4-81·0)	76·1 (70·9-81·0)	76·1 (71·0-81·0)	75·2 (69·0-81·0)	35·1 (18·5-54·6)	48·6 (28·6-70·1)	49·4 (28·7-71·5)	65·3 (27·6-97·8)
Yemen	100·0 (100·0-100·0)	0·0 (0·0-0·0)	0·0 (0·0-0·0)	0·0 (0·0-0·0)	4·5 (0·3-9·1)	26·9 (23·6-30·5)	28·2 (25·0-31·8)	45·7 (42·7-48·8)	76·3 (71·2-81·1)	76·0 (71·1-80·6)	76·0 (71·0-80·6)	75·9 (70·9-80·5)	0·0 (0·0-0·0)	0·0 (0·0-0·0)	0·0 (0·0-0·0)	0·0 (0·0-0·0)
**South Asia**																

Location	SDG Index: Geometric mean of all health-related SDG indicators (scale of 0 to 100)				MDG Index: Geometric mean of all health-related SDG indicators with corresponding MDG indicators (scale of 0 to 100)				Non-MDG Index: Geometric mean of all health-related indicators without corresponding MDG indicators (scale of 0 to 100)				Indicator 1.5.1: Death rate due to exposure to forces of nature (per 100,000 population)			
	**2000**	**2015**	**2016**	**2030**	**2000**	**2015**	**2016**	**2030**	**2000**	**2015**	**2016**	**2030**	**2000**	**2015**	**2016**	**2030**
Bangladesh	16·8 (14·9-18·2)	27·1 (25·6-28·7)	27·8 (26·1-29·3)	32·9 (30·9-34·7)	11·2 (8·8-12·7)	30·9 (26·4-34·3)	32·0 (27·5-35·6)	47·8 (42·7-52·2)	22·1 (19·5-24·1)	27·8 (26·2-29·4)	28·3 (26·8-29·9)	30·2 (28·4-32·0)	8·1 (2·2-16·7)	46·1 (39·1-56·9)	53·7 (46·5-64·6)	36·5 (29·9-46·8)
Bhutan	22·1 (19·8-24·8)	42·2 (40·4-43·9)	43·0 (41·2-44·8)	49·0 (46·1-51·7)	21·8 (17·3-26·1)	50·7 (47·4-53·8)	52·2 (48·6-55·2)	69·9 (64·8-74·1)	24·2 (21·5-27·7)	41·2 (39·3-43·0)	41·7 (39·8-43·5)	43·4 (35·0-46·6)	0·6 (0·0-6·8)	42·2 (34·0-54·4)	46·0 (37·8-58·3)	25·0 (19·2-33·2)
India	24·2 (23·2-25·2)	37·6 (36·5-38·6)	38·6 (37·5-39·6)	46·8 (45·0-48·4)	15·4 (14·2-16·5)	34·4 (32·8-36·0)	36·0 (34·5-37·6)	51·1 (48·7-53·3)	30·1 (28·5-31·7)	40·0 (38·8-41·3)	40·8 (39·5-42·0)	45·7 (42·2-47·8)	34·0 (27·6-43·9)	48·9 (42·1-59·4)	49·9 (43·1-60·2)	41·8 (35·1-52·2)
Nepal	13·2 (12·4-14·4)	24·4 (22·7-27·0)	25·2 (23·3-28·0)	33·4 (30·1-36·6)	11·7 (10·3-13·4)	33·7 (30·7-36·1)	35·1 (31·9-37·7)	52·0 (44·5-56·6)	16·3 (15·3-17·9)	24·0 (22·0-27·2)	24·6 (22·5-27·9)	30·5 (27·1-33·8)	19·2 (12·9-29·1)	1·0 (0·0-8·8)	1·4 (0·0-10·4)	5·0 (0·0-14·9)
Pakistan	21·6 (20·3-22·9)	28·7 (27·2-30·1)	29·4 (27·9-30·9)	33·0 (30·3-35·8)	17·4 (14·7-19·9)	29·0 (26·0-31·8)	30·1 (27·0-32·9)	38·4 (32·3-44·4)	27·5 (26·2-28·9)	31·8 (30·0-33·3)	32·3 (30·6-33·9)	33·8 (30·9-36·3)	35·5 (28·8-45·9)	35·3 (28·7-45·7)	37·0 (30·3-47·4)	12·3 (6·2-21·4)
**Southern Sub-Saharan Africa**																
Botswana	28·1 (14·9-35·6)	39·1 (33·7-44·8)	40·0 (34·7-45·6)	48·3 (36·6-56·6)	23·5 (17·2-27·3)	38·9 (33·1-44·6)	41·5 (34·6-47·5)	60·7 (48·3-69·8)	30·8 (15·4-39·8)	38·3 (32·6-44·4)	38·6 (32·9-44·5)	41·7 (28·7-50·2)	51·2 (42·5-65·3)	56·5 (46·9-70·5)	58·2 (48·6-72·1)	61·6 (52·7-73·2)
Lesotho	15·4 (12·4-19·1)	17·8 (14·7-21·5)	18·3 (15·0-21·9)	21·6 (16·9-27·9)	10·2 (7·8-13·7)	19·2 (14·0-23·8)	19·6 (14·2-24·3)	26·4 (18·4-33·8)	17·6 (14·1-22·3)	16·8 (14·0-20·6)	17·2 (14·3-21·0)	19·0 (14·9-25·7)	100·0 (100·0-100·0)	48·9 (39·7-64·1)	51·3 (42·2-66·4)	54·7 (45·6-69·6)
Namibia	25·6 (20·7-31·0)	34·0 (30·3-37·9)	34·8 (31·1-38·6)	42·3 (35·8-48·0)	21·5 (17·9-24·8)	34·0 (29·6-38·2)	35·2 (30·7-39·5)	47·3 (34·6-55·9)	29·6 (23·7-36·2)	35·6 (30·9-39·6)	36·2 (31·8-40·3)	41·8 (35·2-47·3)	100·0 (100·0-100·0)	23·3 (15·1-36·5)	26·5 (18·1-39·9)	27·5 (19·3-40·4)
South Africa	31·2 (30·2-32·4)	42·2 (40·6-44·1)	43·2 (41·3-45·1)	50·9 (46·8-54·4)	31·3 (30·0-32·8)	37·7 (35·9-39·8)	40·4 (38·7-42·2)	51·4 (47·3-55·3)	32·0 (30·6-33·5)	43·4 (41·0-45·8)	43·6 (41·2-46·1)	47·4 (38·7-53·3)	35·0 (28·1-43·8)	38·2 (30·4-48·8)	41·6 (33·7-52·3)	36·4 (29·1-46·5)
Swaziland	22·2 (15·5-27·2)	29·7 (23·7-34·8)	30·9 (24·7-35·9)	40·4 (27·8-47·4)	18·6 (15·3-21·9)	27·9 (24·1-31·6)	30·2 (25·9-34·4)	39·9 (26·4-48·8)	24·1 (15·8-30·6)	30·0 (22·6-36·0)	30·8 (23·2-36·5)	39·0 (24·4-46·5)	100·0 (100·0-100·0)	49·7 (39·6-67·1)	51·2 (41·2-68·5)	57·4 (47·7-74·1)
Zimbabwe	28·0 (21·1-33·3)	31·2 (26·9-34·8)	31·8 (27·6-35·5)	38·2 (28·6-45·7)	19·2 (15·5-22·3)	23·0 (20·2-25·7)	23·6 (20·9-26·4)	32·4 (20·7-41·7)	33·9 (25·3-40·6)	36·0 (30·6-40·8)	36·5 (31·1-41·3)	40·7 (32·0-48·0)	43·7 (34·8-58·2)	48·8 (39·8-63·2)	50·5 (41·5-64·8)	53·4 (44·5-67·6)
**Western Sub-Saharan Africa**																
Benin	17·0 (15·1-18·7)	22·0 (19·9-24·1)	22·1 (19·7-24·3)	27·0 (23·3-30·3)	10·2 (8·2-13·0)	18·4 (13·5-22·5)	19·1 (14·0-23·4)	24·6 (16·8-31·8)	19·6 (16·8-21·9)	23·2 (20·5-25·9)	22·9 (20·0-25·7)	26·9 (21·6-31·3)	61·6 (53·6-72·0)	60·4 (52·1-72·4)	64·0 (55·4-76·4)	62·4 (54·9-71·6)
Burkina Faso	12·2 (10·9-13·8)	20·2 (17·8-22·6)	20·5 (18·0-22·4)	26·2 (23·1-28·6)	4·4 (3·6-5·8)	15·8 (11·3-19·6)	16·5 (11·7-20·3)	26·3 (17·2-32·4)	18·8 (16·5-21·9)	23·2 (19·8-25·9)	23·1 (20·0-25·5)	26·7 (23·7-29·3)	75·9 (66·8-90·5)	65·8 (58·0-74·2)	69·9 (62·3-79·1)	67·1 (59·7-75·2)
Cameroon	15·0 (13·2-16·8)	21·8 (19·7-23·7)	22·4 (20·3-24·4)	27·2 (24·1-30·0)	9·3 (7·3-11·3)	20·4 (15·4-23·4)	21·1 (15·9-24·2)	30·6 (23·1-35·3)	17·5 (15·0-19·7)	22·8 (19·9-25·3)	23·3 (20·4-25·9)	26·3 (22·5-29·7)	70·8 (61·0-87·6)	76·9 (67·9-91·7)	78·5 (69·8-92·6)	73·9 (67·5-81·9)
Cape Verde	42·3 (38·3-45·7)	52·8 (49·4-55·9)	53·3 (49·6-56·3)	60·0 (52·7-65·4)	33·7 (24·2-40·6)	52·3 (46·6-56·6)	53·2 (46·8-57·8)	66·1 (57·7-71·6)	46·8 (42·3-50·9)	53·5 (48·9-57·6)	53·7 (48·8-57·8)	57·2 (47·0-64·2)	100·0 (100·0-100·0)	100·0 (100·0-100·0)	100·0 (100·0-100·0)	100·0 (100·0-100·0)
Chad	7·8 (6·9-9·0)	13·7 (11·8-15·7)	14·2 (12·0-16·3)	20·0 (17·1-22·8)	2·3 (2·0-3·0)	6·3 (4·9-8·6)	6·8 (5·1-9·1)	12·0 (8·4-16·9)	15·6 (13·3-18·1)	20·6 (17·4-23·2)	20·7 (17·3-23·5)	24·6 (21·6-27·8)	79·3 (69·6-91·9)	60·0 (53·1-68·5)	65·7 (58·5-75·6)	55·0 (48·5-62·1)
Cote d'Ivoire	19·4 (17·3-21·3)	25·2 (22·8-27·3)	25·6 (23·2-27·8)	29·9 (26·1-33·5)	11·6 (9·6-13·6)	19·8 (15·9-23·5)	20·6 (16·6-24·6)	27·9 (20·9-34·7)	24·4 (21·7-26·9)	28·6 (25·9-31·1)	28·8 (26·0-31·2)	32·0 (27·8-36·0)	73·0 (62·5-87·9)	63·9 (54·7-77·9)	65·8 (56·7-79·9)	69·2 (60·8-81·5)
The Gambia	20·6 (17·3-23·1)	28·1 (24·6-31·4)	28·4 (25·0-31·8)	32·4 (28·5-36·4)	15·1 (11·4-17·8)	26·8 (22·8-30·3)	27·4 (23·3-31·1)	36·6 (31·8-41·4)	23·0 (18·8-26·6)	28·6 (23·8-33·2)	28·8 (23·8-33·3)	31·0 (26·3-35·9)	27·5 (18·0-47·2)	100·0 (100·0-100·0)	100·0 (100·0-100·0)	59·6 (50·7-76·7)
Ghana	27·8 (25·9-29·7)	34·8 (32·2-37·1)	35·3 (32·6-37·6)	39·7 (35·6-43·0)	16·3 (13·2-18·9)	26·3 (21·6-29·5)	27·1 (22·2-30·4)	34·0 (27·0-39·2)	34·6 (31·7-37·2)	38·0 (34·5-41·0)	38·2 (34·6-41·4)	40·2 (36·5-44·2)	50·0 (41·6-61·6)	63·3 (55·2-73·7)	67·4 (59·3-78·6)	62·5 (55·4-69·9)
Guinea	13·4 (11·8-15·1)	21·4 (19·0-23·5)	22·0 (19·6-24·1)	27·6 (24·8-30·5)	5·5 (4·2-6·8)	14·8 (12·0-17·5)	15·5 (12·6-18·3)	24·4 (20·0-28·5)	20·5 (17·6-24·0)	25·8 (22·2-28·8)	26·2 (22·8-29·3)	29·6 (25·8-32·9)	100·0 (100·0-100·0)	100·0 (100·0-100·0)	100·0 (100·0-100·0)	100·0 (100·0-100·0)
Guinea-Bissau	11·4 (9·6-13·6)	19·9 (17·3-22·7)	20·3 (17·5-23·2)	26·4 (21·4-31·3)	4·6 (3·3-6·5)	14·4 (10·3-19·0)	15·1 (10·9-19·8)	23·5 (14·5-33·3)	18·1 (15·2-21·2)	23·0 (19·2-26·1)	23·3 (19·5-26·4)	27·1 (22·8-31·4)	100·0 (100·0-100·0)	100·0 (100·0-100·0)	100·0 (100·0-100·0)	100·0 (100·0-100·0)
Liberia	11·1 (9·8-12·7)	22·6 (20·1-24·5)	23·0 (20·6-24·9)	29·3 (26·7-31·5)	5·8 (4·4-7·4)	19·0 (15·4-21·8)	19·8 (16·2-22·7)	30·2 (24·4-34·8)	14·5 (12·9-16·6)	22·2 (19·5-24·2)	22·4 (19·8-24·4)	26·9 (24·1-29·2)	60·7 (51·2-78·1)	100·0 (100·0-100·0)	100·0 (100·0-100·0)	87·8 (81·4-96·2)
Mali	11·3 (10·0-12·9)	21·3 (18·7-24·1)	21·6 (18·8-24·5)	29·2 (25·4-32·9)	4·1 (3·2-5·5)	12·8 (9·5-15·5)	13·4 (9·8-16·1)	20·0 (13·3-24·4)	18·4 (16·1-21·3)	27·8 (23·7-32·1)	28·0 (23·7-32·3)	35·0 (30·2-39·7)	71·2 (60·5-86·6)	63·3 (55·0-77·4)	65·5 (57·2-79·8)	67·8 (60·5-78·9)
Mauritania	21·1 (18·7-23·9)	28·9 (26·4-31·3)	29·5 (27·0-31·8)	34·9 (31·2-38·7)	10·2 (8·4-13·2)	23·0 (17·7-27·9)	24·3 (18·6-29·5)	34·8 (27·7-41·9)	30·1 (26·6-33·4)	34·2 (32·0-36·9)	34·4 (32·1-36·7)	37·4 (34·2-41·8)	100·0 (100·0-100·0)	100·0 (100·0-100·0)	100·0 (100·0-100·0)	72·5 (62·2-87·5)
Niger	7·6 (6·4-8·9)	14·5 (12·5-16·8)	15·0 (12·9-17·3)	20·4 (17·4-23·5)	2·5 (1·9-3·4)	8·9 (6·2-12·2)	9·4 (6·6-12·8)	18·0 (12·8-23·9)	13·9 (11·8-16·4)	19·3 (17·0-22·3)	19·6 (17·2-22·6)	22·7 (19·3-26·0)	62·9 (52·1-80·1)	47·4 (39·2-57·3)	49·2 (40·9-59·0)	54·4 (45·9-64·1)
Nigeria	14·5 (13·1-16·0)	24·9 (22·7-27·0)	25·4 (23·2-27·5)	34·9 (30·2-39·1)	7·5 (5·9-9·2)	17·9 (14·5-20·8)	18·8 (15·3-21·9)	25·4 (15·6-31·9)	20·9 (18·7-23·0)	30·0 (27·6-32·3)	30·1 (27·7-32·6)	39·9 (35·0-44·5)	70·6 (61·1-86·9)	64·9 (55·1-83·5)	66·9 (57·1-85·1)	68·1 (59·4-81·0)
Sao Tome and Principe	34·3 (31·1-37·7)	49·0 (46·3-52·0)	49·7 (46·9-52·5)	58·1 (53·8-62·1)	24·0 (19·9-27·6)	42·6 (38·3-46·2)	43·7 (39·4-47·5)	55·1 (48·1-60·0)	38·9 (34·8-43·0)	52·0 (48·0-55·7)	52·5 (48·5-56·3)	60·1 (55·2-64·5)	100·0 (100·0-100·0)	100·0 (100·0-100·0)	100·0 (100·0-100·0)	100·0 (100·0-100·0)
Senegal	24·0 (22·4-25·5)	33·1 (31·0-35·0)	33·4 (31·3-35·4)	37·7 (34·3-40·7)	16·8 (14·5-19·1)	31·1 (27·0-34·9)	31·7 (27·3-35·9)	41·0 (33·8-48·0)	29·1 (27·1-30·9)	35·4 (33·0-37·4)	35·5 (33·0-37·6)	37·2 (33·6-40·3)	38·3 (27·8-60·4)	75·0 (65·8-90·8)	76·7 (67·6-91·8)	63·3 (54·4-76·4)

Location	Indicator 2.2.1: Prevalence of stunting in children under 5 (%)				Indicator 2.2.2a: Prevalence of wasting in children under 5 (%)				Indicator 2.2.2b: Prevalence of overweight in children aged 2-4 (%)				Indicator 3.1.1: Maternal mortality ratio (maternal deaths per 100,000 livebirths) in women aged 10-54 years			
	**2000**	**2015**	**2016**	**2030**	**2000**	**2015**	**2016**	**2030**	**2000**	**2015**	**2016**	**2030**	**2000**	**2015**	**2016**	**2030**
Bangladesh	0·2 (0·0-2·9)	29·9 (10·5-45·5)	32·6 (12·0-48·9)	64·2 (39·0-79·3)	30·4 (24·5-36·7)	33·1 (24·5-41·4)	34·6 (25·7-43·3)	51·9 (42·8-60·2)	99·7 (96·9-100·0)	99·0 (93·9-100·0)	98·8 (93·0-100·0)	95·4 (77·6-100·0)	7·3 (4·3-10·6)	23·6 (18·6-28·9)	24·5 (19·1-29·8)	37·6 (23·1-51·5)
Bhutan	5·4 (0·0-17·8)	47·9 (27·5-64·2)	49·8 (28·4-66·9)	71·8 (53·9-89·9)	87·8 (83·4-91·4)	72·2 (64·4-78·6)	72·4 (64·1-79·1)	75·6 (59·9-85·5)	92·0 (78·8-100·0)	77·3 (54·5-91·7)	76·3 (52·9-91·4)	56·8 (0·2-91·7)	14·3 (9·7-19·5)	26·1 (18·2-34·0)	26·8 (18·5-35·1)	38·2 (22·2-54·0)
India	5·5 (1·8-9·4)	21·8 (18·0-25·6)	23·4 (19·8-27·2)	34·4 (29·4-40·0)	0·1 (0·0-1·3)	13·7 (8·6-18·8)	16·7 (11·7-21·6)	29·9 (25·8-34·1)	94·0 (84·9-100·0)	85·5 (73·5-94·9)	84·5 (71·5-94·7)	67·6 (24·1-94·2)	12·1 (10·3-14·0)	21·4 (19·0-23·5)	22·4 (19·7-24·6)	31·7 (26·5-36·1)
Nepal	0·1 (0·0-1·6)	33·9 (14·2-50·8)	36·6 (16·3-53·7)	67·5 (45·6-80·2)	37·6 (26·6-47·3)	45·5 (32·2-56·6)	47·4 (34·0-58·6)	57·4 (53·2-61·2)	99·5 (95·8-100·0)	98·2 (91·4-100·0)	97·7 (90·3-100·0)	89·8 (61·5-100·0)	9·4 (6·7-12·2)	18·8 (14·3-24·1)	19·6 (15·1-25·2)	30·5 (20·9-41·9)
Pakistan	13·5 (0·0-26·9)	22·5 (8·6-37·8)	24·4 (9·5-40·7)	48·4 (21·8-67·9)	42·8 (34·7-50·3)	13·6 (5·0-22·4)	14·6 (5·5-23·7)	5·8 (0·0-18·8)	85·8 (68·8-97·2)	96·3 (87·8-100·0)	96·2 (87·7-100·0)	96·7 (79·8-100·0)	7·5 (4·1-11·2)	15·0 (9·2-21·0)	15·6 (10·0-21·6)	24·5 (12·6-36·9)
**Southern Sub-Saharan Africa**																
Botswana	46·6 (39·8-52·0)	56·7 (44·1-68·8)	58·7 (45·8-71·1)	62·4 (53·5-73·3)	67·9 (62·3-72·7)	70·9 (62·9-77·4)	71·4 (63·0-78·1)	74·2 (64·6-80·3)	65·5 (54·5-75·0)	52·0 (35·1-66·9)	50·4 (32·8-66·5)	29·2 (0·0-62·3)	22·0 (0·0-38·3)	45·9 (28·7-71·4)	45·4 (29·1-71·9)	70·2 (31·7-100·0)
Lesotho	4·6 (0·0-12·8)	40·8 (26·9-52·2)	42·8 (28·8-54·4)	67·4 (53·0-78·7)	56·0 (47·8-62·9)	83·6 (78·2-88·2)	84·3 (78·9-88·8)	94·5 (91·2-97·1)	76·8 (66·1-84·7)	76·6 (67·3-84·8)	75·9 (66·2-84·5)	73·0 (52·3-89·1)	11·3 (2·7-22·4)	13·0 (2·1-25·4)	13·9 (1·9-27·1)	25·9 (0·0-57·3)
Namibia	47·5 (40·3-54·6)	61·1 (51·6-70·8)	62·9 (52·9-73·1)	72·5 (66·2-78·4)	51·1 (41·7-59·7)	58·2 (48·8-66·6)	58·9 (49·2-67·5)	67·2 (53·6-76·0)	95·1 (89·9-99·2)	85·6 (77·6-91·6)	85·0 (76·2-91·7)	75·9 (52·3-92·0)	16·2 (5·1-31·9)	33·1 (20·2-52·3)	34·0 (21·1-53·6)	51·4 (28·0-80·1)
South Africa	51·9 (47·5-56·0)	65·3 (62·0-68·3)	66·0 (62·6-69·1)	74·8 (70·8-78·4)	70·9 (67·5-73·9)	75·1 (72·0-77·6)	75·3 (72·3-77·9)	79·0 (76·1-81·5)	54·1 (40·0-66·8)	41·8 (24·4-58·7)	41·1 (23·8-57·6)	24·6 (0·0-56·9)	21·2 (16·5-26·6)	22·6 (18·8-26·9)	22·2 (18·1-26·5)	36·8 (21·2-50·2)
Swaziland	34·3 (24·1-43·3)	51·4 (39·4-64·2)	53·5 (41·3-66·6)	64·0 (51·0-78·2)	90·5 (86·8-93·7)	92·7 (88·4-96·2)	92·9 (88·6-96·4)	94·7 (90·0-98·3)	61·4 (47·3-73·5)	61·6 (45·4-74·5)	60·9 (44·6-74·4)	57·8 (26·8-82·4)	32·6 (16·6-43·6)	41·4 (24·6-58·5)	42·4 (24·9-60·9)	68·3 (31·1-100·0)
Zimbabwe	42·4 (33·6-51·4)	50·0 (40·6-59·2)	52·0 (42·6-61·3)	51·4 (43·2-59·8)	63·3 (56·9-68·8)	83·4 (79·2-87·0)	83·9 (79·8-87·5)	90·2 (86·4-93·1)	70·8 (63·3-77·3)	86·1 (79·5-92·0)	85·7 (78·8-91·8)	91·9 (79·3-100·0)	9·0 (0·0-17·7)	10·7 (3·1-18·4)	11·2 (4·0-18·7)	21·1 (1·4-39·0)
**Western Sub-Saharan Africa**																
Benin	25·6 (8·7-36·8)	37·6 (22·0-50·6)	41·9 (25·9-55·0)	33·9 (21·6-49·1)	41·7 (34·0-48·3)	52·5 (44·2-59·8)	55·1 (46·6-62·6)	66·1 (59·0-72·5)	86·2 (78·0-92·7)	65·8 (47·3-80·1)	65·5 (46·6-80·1)	38·8 (0·0-73·5)	7·5 (4·1-11·3)	13·5 (9·8-17·4)	14·2 (10·4-18·2)	21·7 (14·1-29·6)
Burkina Faso	10·0 (0·0-23·4)	43·6 (32·4-54·8)	45·4 (34·0-56·8)	66·2 (53·0-78·7)	2·4 (0·0-8·9)	30·3 (23·3-37·2)	33·7 (26·7-40·4)	67·5 (62·7-72·3)	84·9 (78·6-90·3)	80·9 (72·3-88·6)	80·1 (70·7-88·3)	70·4 (45·2-89·1)	9·3 (6·3-12·7)	13·8 (10·1-17·7)	14·2 (10·6-18·2)	18·9 (9·9-27·6)
Cameroon	38·5 (27·6-47·2)	45·2 (33·9-55·9)	46·5 (35·4-57·6)	59·5 (46·0-71·8)	63·6 (58·1-68·4)	74·5 (70·4-78·2)	75·2 (71·0-78·9)	83·6 (78·8-86·6)	67·1 (54·8-77·5)	63·0 (46·0-76·0)	61·6 (43·9-75·5)	53·3 (18·9-79·0)	4·1 (0·5-8·5)	8·2 (2·8-15·6)	8·8 (3·2-16·6)	14·7 (3·6-29·3)
Cape Verde	67·8 (51·3-81·4)	80·7 (69·0-89·6)	81·4 (69·9-90·1)	89·1 (80·8-94·9)	70·2 (62·0-77·5)	80·2 (72·6-86·4)	80·7 (73·1-86·7)	86·3 (80·0-92·0)	81·6 (68·5-91·6)	65·2 (46·5-80·3)	64·0 (46·0-79·6)	43·7 (2·8-76·1)	47·3 (40·3-55·0)	61·6 (55·8-67·1)	61·7 (55·6-67·5)	72·4 (57·2-85·1)
Chad	23·8 (10·0-33·6)	32·9 (18·8-46·1)	33·7 (19·2-47·5)	45·5 (28·5-59·1)	20·4 (11·9-28·3)	24·0 (13·8-34·0)	25·1 (14·7-35·3)	39·0 (24·7-52·5)	89·5 (82·6-94·9)	90·0 (83·6-95·2)	89·5 (82·6-95·1)	86·3 (69·4-97·6)	0·8 (0·0-3·9)	4·1 (0·9-8·5)	4·5 (1·2-8·8)	9·7 (2·5-18·1)
Cote d'Ivoire	38·8 (19·9-54·2)	45·8 (26·0-62·4)	47·4 (27·0-64·7)	67·0 (42·7-82·8)	51·4 (41·7-60·9)	53·0 (41·0-64·3)	54·0 (41·7-65·4)	58·2 (44·5-70·5)	82·2 (71·9-90·0)	79·9 (68·2-90·5)	79·0 (66·3-90·0)	72·9 (42·9-93·2)	2·2 (0·0-5·8)	7·2 (3·3-11·1)	7·9 (3·9-11·9)	14·8 (5·6-23·4)
The Gambia	52·0 (42·3-59·4)	59·7 (46·3-70·8)	60·1 (45·2-72·2)	66·5 (38·8-84·5)	50·4 (38·9-60·5)	37·1 (26·2-48·2)	36·7 (25·3-48·2)	30·8 (13·5-46·6)	76·6 (62·3-87·7)	82·5 (69·1-92·3)	82·1 (68·7-92·4)	76·1 (45·0-95·8)	14·4 (11·5-17·7)	14·4 (10·8-18·5)	14·8 (11·0-19·0)	20·1 (11·1-29·0)
Ghana	40·1 (25·3-51·7)	64·7 (54·7-73·9)	67·9 (57·9-77·2)	73·7 (68·9-77·3)	56·2 (50·8-61·6)	60·4 (52·7-67·2)	62·6 (54·7-69·6)	62·2 (54·3-69·0)	91·4 (85·7-96·0)	91·0 (83·5-97·1)	90·6 (82·6-96·8)	87·5 (70·4-99·0)	18·9 (15·7-22·6)	29·9 (25·4-35·1)	30·6 (25·6-36·0)	40·0 (28·0-52·1)
Guinea	29·6 (18·1-37·6)	43·0 (24·4-58·6)	45·1 (25·5-61·1)	60·6 (40·4-76·9)	41·4 (33·5-48·5)	58·8 (50·0-66·5)	59·4 (50·0-67·2)	70·4 (61·5-77·7)	85·6 (80·7-89·9)	79·5 (66·0-90·2)	78·9 (66·1-89·8)	70·8 (37·6-91·2)	0·3 (0·0-2·1)	4·6 (1·2-8·1)	5·1 (1·3-8·8)	11·2 (3·2-19·4)
Guinea-Bissau	29·8 (12·5-41·1)	49·4 (32·1-62·7)	51·0 (33·3-64·8)	70·3 (47·4-86·3)	40·3 (32·3-48·4)	65·8 (58·7-72·6)	66·6 (59·3-73·6)	77·2 (71·7-84·6)	85·0 (73·6-94·2)	69·2 (52·8-83·4)	68·8 (52·6-82·7)	60·2 (26·4-86·1)	5·4 (1·2-9·5)	11·9 (6·3-17·4)	12·4 (6·9-17·8)	19·7 (8·5-31·4)
Liberia	5·4 (0·0-16·3)	41·6 (21·5-57·8)	43·2 (22·8-59·4)	64·2 (43·5-78·7)	66·9 (59·2-73·8)	68·2 (60·2-75·4)	68·7 (60·7-76·0)	72·7 (65·1-80·0)	84·5 (73·3-92·8)	75·0 (60·6-86·7)	74·5 (59·9-86·1)	65·0 (27·8-90·2)	0·3 (0·0-2·4)	4·1 (1·1-7·1)	4·7 (1·5-8·0)	13·6 (6·2-21·3)
Mali	26·3 (11·2-40·4)	54·4 (38·6-69·4)	55·5 (39·1-71·1)	69·3 (45·9-88·2)	3·9 (0·0-15·2)	41·5 (29·8-52·3)	43·0 (31·2-54·1)	65·2 (53·9-74·1)	89·9 (84·3-95·0)	80·7 (67·2-91·2)	80·1 (66·2-90·9)	67·4 (32·9-90·9)	5·4 (3·1-8·3)	10·7 (5·6-17·0)	11·2 (6·0-17·5)	17·2 (6·8-29·5)
Mauritania	23·5 (10·4-34·0)	54·8 (46·5-63·5)	55·4 (47·0-64·4)	74·7 (65·7-80·1)	23·4 (13·3-33·2)	36·2 (27·6-44·7)	36·4 (27·4-45·1)	39·0 (31·3-50·1)	88·7 (80·3-95·4)	87·0 (76·3-94·8)	86·2 (74·7-94·3)	78·7 (50·0-96·5)	1·0 (0·0-8·7)	7·2 (0·8-16·2)	7·7 (1·3-16·7)	14·7 (1·4-29·4)
Niger	2·9 (0·0-12·5)	17·8 (0·0-38·0)	19·1 (0·2-40·5)	37·1 (11·7-61·1)	0·7 (0·0-7·1)	4·2 (0·0-17·4)	4·5 (0·0-18·2)	8·5 (0·0-30·7)	97·6 (92·9-100·0)	93·8 (87·5-98·8)	93·7 (86·9-98·8)	93·5 (79·8-100·0)	5·9 (2·3-9·8)	8·1 (3·9-13·8)	8·5 (4·2-14·3)	12·8 (4·1-23·6)
Nigeria	14·0 (1·5-25·1)	35·5 (21·1-49·4)	39·6 (24·5-53·9)	47·3 (33·8-58·6)	26·9 (18·1-35·1)	39·7 (32·7-46·2)	40·1 (32·8-46·8)	46·0 (36·5-54·6)	84·2 (74·4-91·5)	71·5 (56·5-83·7)	71·5 (56·1-84·3)	60·1 (22·2-85·4)	9·8 (4·2-16·8)	18·1 (11·7-25·4)	18·1 (11·7-25·7)	25·2 (9·4-41·8)
Sao Tome and Principe	33·5 (18·4-43·0)	68·5 (56·7-78·8)	70·9 (59·4-80·9)	84·9 (76·9-92·4)	68·6 (62·1-74·6)	72·9 (66·1-79·2)	74·9 (68·1-81·0)	85·9 (80·6-90·2)	80·8 (68·2-90·6)	87·6 (78·4-95·1)	87·4 (77·8-95·2)	86·1 (66·0-99·4)	22·1 (16·3-37·6)	34·5 (28·7-42·2)	34·5 (28·5-41·4)	38·6 (20·9-54·9)
Senegal	44·3 (34·2-53·7)	65·1 (55·9-74·7)	66·9 (57·4-76·5)	85·0 (76·1-92·8)	44·4 (34·6-52·9)	61·4 (53·7-68·2)	62·3 (54·6-69·2)	73·4 (63·9-81·2)	94·8 (87·9-100·0)	93·7 (86·5-99·2)	93·3 (85·4-99·0)	92·9 (77·8-100·0)	5·4 (3·0-7·8)	11·6 (7·8-15·4)	12·0 (8·1-15·8)	17·6 (9·0-25·8)

Location	Indicator 3.1.2: Proportion of births attended by skilled health personnel (%)				Indicator 3.2.1: Under-5 mortality rate (probability of dying before the age of 5 per 1,000 livebirths)				Indicator 3.2.2: Neonatal mortality rate (probability of dying during the first 28 days of life per 1,000 livebirths)				Indicator 3.3.1: Age-standardised rate of new HIV infections (per 1,000 population)			
	**2000**	**2015**	**2016**	**2030**	**2000**	**2015**	**2016**	**2030**	**2000**	**2015**	**2016**	**2030**	**2000**	**2015**	**2016**	**2030**
Bangladesh	0·0 (0·0-0·0)	30·2 (16·8-44·5)	31·5 (16·5-47·4)	69·0 (33·7-92·3)	16·2 (15·1-17·3)	36·1 (34·5-37·3)	37·8 (36·0-39·0)	58·4 (53·9-62·0)	2·7 (1·6-3·9)	20·1 (17·2-22·7)	21·8 (18·7-24·5)	40·1 (33·6-46·0)	98·7 (89·8-100·0)	95·0 (80·9-100·0)	95·2 (81·1-100·0)	94·7 (80·5-100·0)
Bhutan	18·3 (0·0-42·4)	53·9 (30·2-73·4)	54·8 (30·9-75·2)	78·2 (34·6-99·5)	16·4 (13·9-18·8)	37·1 (32·7-40·9)	38·6 (34·1-42·5)	59·1 (52·0-65·5)	3·3 (0·6-6·2)	21·7 (17·2-25·9)	23·3 (18·7-27·6)	41·9 (34·1-48·7)	68·3 (48·3-85·9)	63·3 (40·8-85·0)	63·6 (41·0-85·4)	63·9 (42·1-85·1)
India	32·0 (21·4-42·9)	64·8 (44·3-80·7)	66·6 (45·1-82·4)	86·2 (55·7-100·0)	17·0 (15·7-18·4)	33·1 (29·0-36·7)	34·5 (30·2-38·3)	52·1 (43·3-60·2)	4·9 (3·1-6·9)	19·4 (14·4-24·1)	20·9 (15·6-25·8)	36·8 (26·2-46·8)	40·3 (36·8-44·1)	55·7 (48·6-64·1)	55·7 (47·9-65·2)	57·4 (49·1-67·5)
Nepal	0·6 (0·0-4·1)	39·5 (24·8-54·4)	42·2 (25·8-58·0)	80·6 (53·5-97·8)	17·6 (16·2-18·9)	38·8 (37·0-40·1)	41·1 (39·2-42·5)	64·0 (59·1-68·1)	4·3 (2·1-6·8)	23·3 (19·8-26·5)	25·2 (21·5-28·4)	46·3 (39·0-52·8)	50·8 (29·5-69·5)	69·9 (48·4-90·7)	71·3 (50·2-91·4)	69·5 (47·5-90·7)
Pakistan	17·7 (10·6-25·1)	56·5 (48·1-64·1)	57·1 (46·3-66·2)	84·3 (65·9-95·0)	15·0 (13·7-16·2)	26·0 (23·7-27·8)	27·2 (24·8-29·1)	40·9 (35·4-45·5)	0·7 (0·0-2·4)	9·4 (5·9-12·7)	10·7 (7·0-14·0)	21·7 (14·3-28·0)	91·0 (74·3-100·0)	71·1 (50·4-91·7)	70·8 (50·0-91·3)	70·2 (49·5-90·1)
**Southern Sub-Saharan Africa**																
Botswana	91·0 (84·8-95·2)	95·5 (88·2-99·0)	95·3 (87·0-99·0)	96·8 (73·0-100·0)	23·3 (20·6-26·1)	56·1 (48·9-62·4)	57·1 (49·6-63·5)	88·9 (73·9-100·0)	24·6 (21·6-27·8)	49·1 (40·8-56·4)	50·3 (41·7-57·9)	73·9 (56·6-89·5)	0·0 (0·0-0·0)	2·3 (0·0-6·8)	6·6 (1·8-12·6)	10·7 (5·2-17·4)
Lesotho	45·8 (39·0-52·7)	76·9 (69·0-83·6)	76·6 (66·0-85·1)	92·6 (77·8-99·4)	12·8 (11·2-14·5)	21·9 (17·6-25·7)	22·2 (17·8-26·3)	30·8 (20·2-40·9)	5·8 (3·6-8·3)	13·9 (7·9-19·2)	14·3 (7·8-19·9)	20·0 (7·9-32·4)	0·0 (0·0-0·0)	0·0 (0·0-0·0)	0·0 (0·0-0·0)	2·9 (0·5-5·2)
Namibia	74·0 (69·9-77·9)	86·9 (79·6-92·0)	86·5 (76·5-93·0)	91·9 (73·6-99·9)	23·2 (19·7-26·4)	35·6 (27·8-43·8)	36·6 (27·4-45·6)	54·5 (32·4-78·6)	20·8 (16·7-24·6)	28·6 (19·7-37·9)	29·5 (19·2-40·0)	42·3 (17·8-68·2)	0·0 (0·0-0·0)	6·0 (2·3-9·5)	6·9 (2·7-10·8)	15·1 (10·9-19·6)
South Africa	86·9 (81·8-91·0)	98·9 (97·4-99·8)	98·9 (97·0-99·8)	99·6 (96·8-100·0)	19·2 (16·9-21·5)	31·0 (25·7-36·3)	32·3 (26·3-38·0)	50·0 (33·6-67·1)	19·2 (16·6-21·7)	29·3 (23·0-35·4)	30·5 (23·7-37·3)	44·9 (27·8-63·9)	0·0 (0·0-0·0)	1·7 (0·0-3·5)	5·2 (3·0-7·7)	14·3 (11·9-17·0)
Swaziland	71·0 (62·8-78·4)	85·2 (70·5-94·2)	85·2 (69·2-94·9)	92·6 (58·0-100·0)	14·2 (12·3-16·3)	28·8 (24·3-33·4)	29·3 (24·2-34·2)	47·7 (33·7-60·2)	20·8 (18·1-23·6)	28·7 (24·4-32·9)	29·1 (24·4-33·8)	40·2 (26·5-53·0)	0·0 (0·0-0·0)	0·3 (0·0-1·8)	3·3 (0·9-5·5)	9·3 (6·4-12·1)
Zimbabwe	68·6 (63·2-73·4)	78·6 (69·9-85·6)	81·9 (71·6-89·5)	96·8 (82·9-100·0)	18·7 (16·9-20·4)	25·5 (21·6-28·9)	26·0 (21·8-29·9)	34·3 (22·8-45·5)	18·4 (15·9-20·8)	18·2 (14·2-22·1)	18·7 (14·2-23·1)	26·7 (13·9-39·0)	0·0 (0·0-0·0)	6·3 (3·4-9·3)	6·2 (3·0-9·6)	9·6 (6·5-13·3)
**Western Sub-Saharan Africa**																
Benin	69·4 (65·4-73·2)	84·3 (75·5-90·8)	84·8 (74·9-91·9)	91·2 (71·8-99·2)	5·0 (3·5-6·5)	19·1 (15·3-22·8)	20·4 (16·2-24·2)	33·7 (25·3-41·7)	5·9 (4·1-7·8)	15·9 (12·4-19·3)	17·0 (13·4-20·7)	26·1 (18·3-33·4)	23·4 (20·2-27·4)	33·2 (30·0-37·0)	33·7 (30·5-37·5)	37·3 (33·7-41·4)
Burkina Faso	26·2 (20·0-32·9)	64·9 (46·2-79·8)	63·9 (42·3-80·2)	73·2 (20·9-98·5)	0·0 (0·0-0·5)	10·0 (6·9-12·8)	10·8 (7·7-13·8)	21·7 (15·4-27·6)	4·3 (3·3-5·5)	12·6 (8·7-16·4)	13·5 (9·3-17·4)	21·8 (13·7-29·2)	34·5 (26·9-46·9)	40·9 (33·4-49·9)	39·6 (29·8-50·1)	43·2 (33·1-54·2)
Cameroon	43·9 (40·0-48·1)	60·5 (46·9-72·8)	62·1 (46·5-75·9)	73·8 (36·6-96·2)	5·5 (3·8-7·2)	14·9 (10·4-19·2)	15·7 (11·0-20·2)	26·1 (16·3-35·4)	7·1 (5·0-9·4)	14·2 (9·0-19·1)	14·9 (9·3-20·1)	22·6 (11·1-33·7)	7·0 (5·1-9·1)	16·6 (13·8-19·9)	17·2 (14·1-20·8)	20·9 (17·8-24·9)
Cape Verde	51·6 (28·9-72·3)	79·8 (58·5-92·1)	80·8 (60·3-92·5)	92·4 (67·1-100·0)	31·2 (28·8-33·3)	52·4 (49·2-55·4)	53·8 (50·6-57·1)	73·6 (64·7-82·4)	27·9 (24·4-31·2)	43·2 (38·6-48·2)	44·7 (39·9-50·2)	61·6 (51·0-74·0)	32·7 (26·6-39·1)	33·0 (27·0-37·9)	32·4 (26·2-37·4)	36·6 (29·8-41·7)
Chad	1·8 (0·0-6·8)	2·8 (0·0-11·7)	3·6 (0·0-14·6)	11·6 (0·0-45·9)	0·0 (0·0-0·0)	9·0 (5·9-11·9)	9·9 (6·7-13·0)	21·2 (14·7-27·4)	2·5 (0·9-4·2)	10·7 (7·6-13·9)	11·5 (8·3-14·9)	19·7 (12·4-26·5)	11·7 (8·3-15·6)	23·2 (17·9-29·0)	24·0 (18·9-29·7)	27·9 (22·5-33·9)
Cote d'Ivoire	55·9 (50·0-61·2)	56·5 (39·2-70·6)	57·4 (38·3-73·1)	69·1 (21·5-97·9)	5·4 (3·9-6·9)	15·3 (12·1-18·2)	16·4 (13·1-19·5)	27·6 (20·2-34·1)	1·7 (0·0-3·6)	9·3 (5·5-13·1)	10·4 (6·5-14·3)	18·6 (10·2-26·9)	10·8 (8·6-13·5)	18·8 (16·5-21·3)	20·1 (17·6-22·8)	24·2 (21·4-27·2)
The Gambia	44·9 (37·8-51·6)	58·3 (49·4-67·1)	59·3 (48·7-70·1)	71·8 (46·1-91·1)	16·2 (14·6-18·0)	28·6 (24·8-31·8)	29·5 (25·6-33·0)	41·2 (34·0-47·8)	10·8 (9·1-12·5)	20·4 (16·5-24·1)	21·3 (17·2-25·2)	30·7 (22·6-38·5)	17·9 (13·1-23·5)	29·0 (23·3-36·2)	30·7 (24·5-39·1)	35·4 (29·0-44·2)
Ghana	25·6 (17·7-33·2)	66·1 (54·0-76·2)	67·1 (53·8-78·6)	89·1 (67·3-99·6)	12·5 (11·1-14·0)	26·5 (22·8-29·9)	27·8 (23·7-31·7)	44·3 (34·1-53·8)	7·6 (6·1-9·4)	17·5 (14·1-20·9)	18·8 (14·9-22·7)	32·3 (21·3-42·4)	16·9 (13·8-20·5)	31·2 (25·9-38·4)	31·6 (25·7-40·1)	35·5 (29·3-44·3)
Guinea	14·6 (9·6-19·2)	35·3 (21·2-49·6)	36·8 (20·5-52·7)	55·8 (12·5-88·0)	0·3 (0·0-1·4)	12·3 (8·2-16·1)	13·4 (9·1-17·5)	26·6 (17·5-35·6)	0·3 (0·0-1·8)	9·6 (4·6-14·3)	10·5 (5·2-15·4)	20·9 (10·4-31·1)	22·4 (19·3-26·1)	30·8 (24·4-38·0)	32·0 (24·6-40·9)	35·9 (28·0-45·7)
Guinea-Bissau	24·5 (14·9-34·8)	36·6 (13·3-60·3)	36·9 (12·4-62·6)	48·9 (0·0-90·1)	2·1 (0·6-3·8)	19·1 (16·1-22·2)	20·4 (17·2-23·7)	39·0 (32·1-45·7)	0·0 (0·0-0·1)	11·9 (8·6-15·2)	13·1 (9·8-16·6)	27·5 (20·3-34·8)	11·0 (7·3-14·9)	20·5 (14·0-28·2)	22·7 (14·6-32·7)	26·6 (18·3-37·8)
Liberia	23·7 (12·5-35·7)	59·1 (47·9-68·8)	60·8 (47·6-72·0)	81·7 (57·0-95·3)	0·3 (0·0-1·4)	21·2 (17·9-24·2)	22·7 (19·2-25·8)	41·3 (34·0-48·2)	1·8 (0·2-3·5)	18·6 (16·1-21·2)	20·0 (17·3-22·7)	34·8 (28·7-40·8)	16·5 (13·0-20·2)	31·9 (26·4-38·1)	32·1 (26·1-38·9)	35·6 (29·2-42·7)
Mali	15·7 (9·9-21·9)	41·4 (24·9-56·8)	43·9 (24·8-61·1)	72·3 (28·7-97·9)	0·0 (0·0-0·0)	8·3 (4·5-11·8)	9·1 (5·0-13·0)	20·0 (11·0-28·3)	0·0 (0·0-0·0)	5·2 (1·2-9·1)	6·0 (1·6-10·2)	14·6 (4·9-23·6)	25·5 (21·5-29·9)	32·3 (29·0-35·9)	32·5 (28·8-36·2)	36·7 (32·8-40·8)
Mauritania	56·1 (50·0-61·6)	64·1 (47·8-77·0)	65·8 (48·5-79·9)	82·6 (42·7-100·0)	14·5 (12·9-16·2)	27·5 (24·8-29·7)	28·7 (25·8-31·0)	43·4 (37·2-48·7)	6·5 (4·4-8·5)	17·1 (13·5-20·4)	18·3 (14·4-21·7)	30·6 (23·2-37·4)	41·6 (22·2-61·1)	55·1 (31·4-78·3)	55·6 (32·4-78·3)	54·6 (31·3-77·7)
Niger	0·4 (0·0-3·2)	18·5 (5·2-32·9)	18·5 (3·8-35·1)	46·4 (3·0-86·6)	0·0 (0·0-0·0)	9·9 (6·1-13·4)	11·0 (7·0-14·7)	25·8 (17·6-33·5)	2·8 (1·0-4·9)	14·9 (9·6-19·9)	15·9 (10·1-21·1)	26·3 (15·9-36·4)	24·3 (21·2-28·0)	46·5 (40·4-53·0)	45·5 (39·1-52·8)	49·0 (42·2-56·5)
Nigeria	17·8 (9·5-26·6)	25·9 (14·0-38·3)	24·5 (10·8-39·2)	24·1 (0·0-60·8)	0·0 (0·0-0·0)	9·4 (6·3-12·6)	10·8 (7·4-14·2)	21·4 (13·5-29·8)	0·0 (0·0-0·7)	6·4 (1·5-11·7)	7·7 (2·5-13·2)	15·7 (4·5-28·2)	12·1 (9·3-15·4)	22·7 (19·0-26·7)	24·3 (20·0-29·3)	28·7 (24·0-34·1)
Sao Tome and Principe	77·0 (67·0-85·5)	84·6 (73·4-92·5)	84·7 (72·6-92·8)	89·5 (64·7-99·9)	19·8 (17·8-21·9)	39·1 (36·4-41·1)	40·4 (37·5-42·5)	58·6 (50·7-65·1)	21·1 (18·6-23·7)	33·8 (30·2-36·9)	35·0 (31·3-38·2)	47·6 (38·7-55·6)	34·5 (14·0-54·5)	55·7 (33·9-79·5)	56·7 (34·3-80·3)	61·8 (39·7-85·5)
Senegal	52·0 (44·7-59·2)	45·1 (36·1-54·1)	46·4 (35·0-57·4)	45·3 (16·3-70·7)	9·5 (8·5-10·6)	27·9 (25·2-30·3)	29·1 (26·2-31·7)	47·3 (40·8-53·0)	7·2 (5·5-9·1)	21·7 (18·9-24·4)	22·9 (19·7-25·8)	38·1 (30·8-45·4)	31·5 (27·4-35·8)	45·4 (39·6-52·4)	44·8 (38·8-51·5)	43·0 (36·9-49·7)

Location	Indicator 3.3.2:<br>Age-standardised rate of tuberculosis cases (per 100,000 population)				Indicator 3.3.3:<br>Age-standardised rate of malaria cases (per 1,000 population)				Indicator 3.3.4:<br>Age-standardised rate of hepatitis B incidence (per 100,000 population)				Indicator 3.3.5:<br>Age-standardised prevalence* of the sum of 15 neglected tropical diseases (NTDs) (%)<br>**Prevalence estimates reported here may exceed 100% as they reflect the sum of prevalent cases of 15 NTDs.*			
	**2000**	**2015**	**2016**	**2030**	**2000**	**2015**	**2016**	**2030**	**2000**	**2015**	**2016**	**2030**	**2000**	**2015**	**2016**	**2030**
Bangladesh	27·7<br>(25·9-29·4)	38·7<br>(36·7-40·6)	38·5<br>(36·6-40·5)	42·4<br>(40·5-44·4)	28·5<br>(26·3-31·4)	56·6<br>(52·3-62·3)	59·3<br>(54·7-65·6)	89·5<br>(85·1-92·5)	12·0<br>(9·4-15·2)	28·7<br>(25·5-32·1)	29·0<br>(25·7-32·6)	35·1<br>(29·3-40·8)	14·4<br>(6·0-21·9)	38·4<br>(27·7-47·5)	39·9<br>(29·5-48·8)	58·0<br>(50·4-64·2)
Bhutan	29·1<br>(27·3-30·8)	36·2<br>(34·4-38·0)	36·6<br>(34·7-38·3)	42·2<br>(40·3-44·1)	20·8<br>(17·6-23·8)	64·6<br>(60·4-67·3)	68·1<br>(63·8-70·8)	97·1<br>(94·8-98·1)	1·3<br>(0·0-5·5)	20·6<br>(17·5-23·4)	21·8<br>(19·0-24·5)	40·4<br>(37·5-43·1)	83·7<br>(78·7-87·5)	83·7<br>(78·3-87·8)	83·7<br>(78·3-87·8)	83·8<br>(78·4-87·9)
India	22·3<br>(20·6-24·1)	27·0<br>(25·2-28·9)	27·3<br>(25·5-29·3)	31·5<br>(29·5-33·6)	20·7<br>(18·3-22·9)	25·3<br>(22·0-29·0)	25·8<br>(22·2-29·8)	31·7<br>(26·6-36·7)	1·5<br>(0·0-5·1)	9·5<br>(7·0-12·2)	10·5<br>(8·0-13·1)	22·6<br>(19·5-25·4)	72·7<br>(67·9-76·5)	73·8<br>(69·3-77·0)	73·9<br>(69·5-77·2)	76·2<br>(72·1-79·2)
Nepal	26·2<br>(24·6-28·0)	36·3<br>(34·6-38·2)	37·0<br>(35·3-38·9)	47·0<br>(45·3-49·0)	37·2<br>(33·9-40·3)	48·4<br>(45·1-50·5)	47·7<br>(44·6-49·8)	60·8<br>(56·2-64·5)	12·4<br>(9·2-15·4)	26·5<br>(22·9-29·9)	27·8<br>(24·1-31·2)	40·6<br>(36·1-45·2)	20·7<br>(9·7-29·3)	44·2<br>(34·7-51·5)	45·7<br>(36·4-52·8)	63·2<br>(56·7-68·2)
Pakistan	22·0<br>(20·1-24·0)	27·5<br>(25·5-29·5)	27·9<br>(25·9-30·0)	33·8<br>(31·7-35·9)	23·5<br>(20·9-26·2)	31·3<br>(29·0-33·2)	32·4<br>(30·2-34·4)	36·4<br>(31·7-44·3)	14·4<br>(11·5-17·2)	22·4<br>(19·2-25·2)	23·1<br>(19·8-25·8)	32·1<br>(27·6-35·5)	85·5<br>(82·2-88·2)	85·7<br>(82·0-88·7)	85·7<br>(82·0-88·8)	85·9<br>(82·1-88·9)
**Southern Sub-Saharan Africa**																
Botswana	0·0<br>(0·0-0·0)	0·1<br>(0·0-1·1)	0·4<br>(0·0-2·1)	14·5<br>(11·8-17·6)	36·1<br>(33·2-39·0)	57·7<br>(54·1-60·1)	60·6<br>(56·8-63·2)	95·3<br>(91·8-97·2)	47·2<br>(45·0-49·1)	56·7<br>(54·5-58·5)	57·2<br>(55·0-59·0)	63·7<br>(61·3-65·7)	45·6<br>(38·5-52·4)	44·0<br>(36·7-51·1)	43·6<br>(36·2-50·8)	37·8<br>(29·1-46·2)
Lesotho	0·0<br>(0·0-0·0)	0·0<br>(0·0-0·0)	0·0<br>(0·0-0·0)	0·0<br>(0·0-0·0)	100·0<br>(100·0-100·0)	100·0<br>(100·0-100·0)	100·0<br>(100·0-100·0)	100·0<br>(100·0-100·0)	43·5<br>(41·7-45·5)	45·4<br>(43·2-47·5)	45·9<br>(43·6-48·0)	47·5<br>(44·6-50·4)	30·7<br>(18·6-41·6)	30·8<br>(18·0-41·8)	30·8<br>(18·1-41·8)	31·4<br>(18·7-42·2)
Namibia	0·0<br>(0·0-0·0)	1·6<br>(0·0-3·4)	2·2<br>(0·4-4·1)	13·1<br>(10·6-15·9)	15·1<br>(9·3-21·3)	36·7<br>(34·2-38·5)	35·3<br>(32·9-37·0)	28·2<br>(22·5-36·1)	46·2<br>(44·2-48·0)	55·2<br>(53·4-56·9)	55·8<br>(54·0-57·6)	64·6<br>(62·5-66·6)	69·4<br>(61·5-77·4)	69·1<br>(61·2-77·4)	69·0<br>(61·1-77·4)	67·8<br>(58·9-76·7)
South Africa	8·3<br>(7·2-9·3)	2·7<br>(1·7-3·8)	3·7<br>(2·6-4·7)	5·5<br>(0·9-11·0)	30·3<br>(26·2-34·4)	34·2<br>(28·1-41·5)	33·5<br>(27·5-41·1)	33·7<br>(27·4-42·6)	43·0<br>(41·4-44·6)	52·3<br>(50·5-54·0)	52·8<br>(50·9-54·5)	60·6<br>(57·7-64·0)	25·3<br>(11·8-37·5)	29·2<br>(20·4-37·6)	29·8<br>(21·1-38·1)	37·4<br>(29·7-44·9)
Swaziland	0·0<br>(0·0-0·0)	0·0<br>(0·0-0·0)	0·0<br>(0·0-0·0)	1·7<br>(0·0-4·9)	40·1<br>(33·8-46·0)	54·3<br>(50·8-56·5)	55·1<br>(51·6-57·5)	69·4<br>(58·1-77·1)	44·6<br>(42·8-46·3)	49·0<br>(47·2-50·9)	49·8<br>(48·0-51·7)	56·9<br>(54·6-59·2)	8·8<br>(0·0-20·5)	10·5<br>(1·0-20·8)	10·5<br>(1·1-20·8)	11·6<br>(2·3-21·5)
Zimbabwe	0·0<br>(0·0-0·0)	0·0<br>(0·0-0·2)	0·3<br>(0·0-1·8)	12·2<br>(9·5-15·9)	7·7<br>(1·5-15·3)	10·7<br>(6·4-16·3)	11·2<br>(6·7-17·0)	17·6<br>(11·1-24·9)	45·1<br>(42·9-47·6)	42·4<br>(40·0-45·0)	42·8<br>(40·5-45·4)	42·7<br>(39·9-45·6)	41·4<br>(34·2-47·8)	50·4<br>(43·5-56·4)	51·5<br>(44·7-57·5)	64·6<br>(58·3-69·8)
**Western Sub-Saharan Africa**																
Benin	28·9<br>(27·4-30·5)	35·5<br>(34·0-37·2)	35·9<br>(34·3-37·6)	41·4<br>(39·6-43·4)	2·8<br>(1·1-4·9)	2·5<br>(1·2-4·2)	2·5<br>(1·2-4·2)	3·2<br>(1·4-5·3)	2·9<br>(1·0-5·4)	13·8<br>(11·6-16·7)	14·4<br>(12·2-17·3)	23·2<br>(20·7-26·0)	18·1<br>(12·7-23·4)	48·6<br>(44·5-52·5)	51·7<br>(47·7-55·5)	79·7<br>(77·0-81·9)
Burkina Faso	22·4<br>(20·9-24·0)	28·9<br>(27·3-30·3)	29·2<br>(27·6-30·7)	34·9<br>(32·8-37·0)	0·0<br>(0·0-0·0)	0·2<br>(0·0-1·0)	0·3<br>(0·0-1·3)	1·6<br>(0·5-2·6)	5·2<br>(3·5-7·3)	12·5<br>(10·7-14·6)	12·9<br>(11·0-15·0)	18·2<br>(15·7-20·6)	16·7<br>(11·2-22·7)	85·2<br>(82·6-87·2)	86·8<br>(84·3-88·7)	98·3<br>(97·7-98·7)
Cameroon	21·1<br>(19·9-22·2)	26·7<br>(25·4-27·8)	27·1<br>(25·9-28·3)	33·1<br>(31·8-34·4)	1·5<br>(0·5-2·7)	4·0<br>(2·8-5·4)	4·2<br>(2·9-5·6)	6·7<br>(4·7-8·5)	0·0<br>(0·0-0·6)	11·3<br>(9·5-13·0)	11·9<br>(10·2-13·7)	21·6<br>(19·7-23·4)	0·0<br>(0·0-0·0)	39·8<br>(32·2-46·1)	45·3<br>(38·3-51·1)	85·8<br>(83·8-87·6)
Cape Verde	39·6<br>(37·9-41·2)	47·7<br>(45·9-49·3)	48·2<br>(46·4-49·8)	55·1<br>(53·2-56·8)	48·7<br>(44·7-52·2)	74·2<br>(69·5-77·0)	77·4<br>(72·8-80·3)	92·7<br>(88·9-94·9)	29·5<br>(27·7-31·4)	38·8<br>(36·8-41·3)	39·2<br>(37·2-41·8)	48·1<br>(45·3-51·5)	98·3<br>(98·0-98·7)	98·6<br>(98·1-98·9)	98·6<br>(98·2-99·0)	98·9<br>(98·6-99·2)
Chad	21·0<br>(19·6-22·6)	28·8<br>(27·3-30·3)	29·3<br>(27·8-30·8)	36·0<br>(34·4-37·6)	6·2<br>(1·8-10·6)	8·2<br>(2·7-13·6)	8·4<br>(2·9-13·9)	10·8<br>(3·3-18·0)	1·8<br>(0·0-4·1)	10·6<br>(8·6-12·9)	11·2<br>(9·2-13·6)	20·1<br>(17·7-22·8)	44·3<br>(37·4-50·5)	46·1<br>(39·0-52·6)	45·9<br>(38·8-52·4)	44·5<br>(37·5-50·8)
Cote d'Ivoire	19·3<br>(18·0-20·5)	27·2<br>(25·9-28·5)	27·7<br>(26·3-28·9)	34·3<br>(32·8-35·8)	0·0<br>(0·0-0·0)	3·4<br>(2·5-4·5)	4·2<br>(3·2-5·4)	5·5<br>(4·3-6·9)	2·7<br>(0·1-5·7)	9·2<br>(6·7-12·3)	9·6<br>(7·1-12·6)	15·3<br>(13·0-17·9)	11·2<br>(4·4-17·9)	27·8<br>(22·1-33·6)	29·9<br>(24·2-35·4)	52·8<br>(48·0-57·2)
The Gambia	28·0<br>(26·6-29·4)	32·5<br>(31·0-33·9)	32·8<br>(31·4-34·3)	38·0<br>(36·6-39·5)	4·3<br>(1·0-7·2)	14·9<br>(10·7-18·4)	15·1<br>(10·8-18·7)	18·2<br>(12·0-22·5)	14·0<br>(11·9-16·8)	22·7<br>(20·4-25·7)	23·0<br>(20·7-26·0)	27·6<br>(24·7-31·1)	47·1<br>(42·6-51·4)	48·4<br>(44·7-52·0)	48·6<br>(45·1-52·1)	52·4<br>(48·1-57·1)
Ghana	16·8<br>(15·2-18·6)	26·2<br>(24·5-27·8)	26·8<br>(25·1-28·4)	35·5<br>(33·5-37·6)	0·2<br>(0·0-1·0)	2·1<br>(1·3-3·1)	2·3<br>(1·5-3·4)	4·6<br>(3·3-6·0)	7·9<br>(5·7-10·4)	16·7<br>(14·5-19·3)	17·6<br>(15·5-20·2)	25·0<br>(22·1-28·4)	61·5<br>(58·0-64·5)	78·2<br>(75·5-80·5)	79·4<br>(76·7-81·8)	90·9<br>(88·8-92·6)
Guinea	24·6<br>(23·1-26·1)	26·3<br>(24·8-27·8)	26·5<br>(25·0-28·0)	27·9<br>(26·4-29·4)	0·5<br>(0·0-2·0)	0·9<br>(0·0-2·4)	1·0<br>(0·0-2·5)	1·5<br>(0·3-2·9)	1·8<br>(0·1-4·3)	11·4<br>(9·4-14·2)	12·1<br>(10·1-14·9)	20·9<br>(18·2-24·6)	27·1<br>(18·1-35·4)	31·2<br>(22·2-39·7)	31·5<br>(22·6-40·0)	36·1<br>(27·1-44·5)
Guinea-Bissau	17·8<br>(16·5-19·2)	17·5<br>(16·2-18·9)	17·7<br>(16·5-19·0)	18·8<br>(17·4-20·4)	2·1<br>(0·0-5·4)	12·6<br>(6·2-19·4)	13·0<br>(6·1-20·2)	18·6<br>(7·1-32·2)	0·0<br>(0·0-0·0)	7·0<br>(4·4-9·8)	7·7<br>(5·1-10·5)	17·9<br>(14·7-21·2)	24·5<br>(16·4-32·8)	55·8<br>(49·3-62·7)	57·7<br>(51·3-64·3)	77·2<br>(72·3-81·6)
Liberia	20·6<br>(19·2-22·0)	28·3<br>(26·8-29·8)	28·8<br>(27·3-30·3)	35·9<br>(34·2-37·6)	0·8<br>(0·0-3·3)	3·6<br>(1·6-6·4)	3·9<br>(1·8-6·8)	6·3<br>(4·5-8·6)	5·3<br>(3·3-7·8)	15·1<br>(12·6-17·9)	15·6<br>(13·0-18·4)	22·5<br>(19·6-25·7)	0·0<br>(0·0-0·0)	0·5<br>(0·0-6·1)	0·9<br>(0·0-8·2)	20·1<br>(3·7-33·4)
Mali	35·8<br>(33·9-37·5)	46·6<br>(44·6-48·3)	47·0<br>(45·0-48·8)	53·9<br>(51·8-55·7)	0·5<br>(0·0-2·2)	0·5<br>(0·0-2·1)	0·4<br>(0·0-1·9)	0·0<br>(0·0-0·5)	3·3<br>(1·2-5·7)	16·5<br>(14·5-19·0)	17·3<br>(15·2-19·7)	28·4<br>(25·9-31·2)	16·3<br>(9·0-22·9)	79·6<br>(77·2-81·7)	82·2<br>(79·9-84·1)	97·2<br>(96·7-97·7)
Mauritania	33·9<br>(32·5-35·4)	42·5<br>(41·0-44·1)	42·9<br>(41·3-44·4)	47·8<br>(46·1-49·5)	12·8<br>(8·7-18·1)	17·5<br>(13·8-21·8)	17·6<br>(13·9-21·8)	19·6<br>(15·6-23·3)	11·2<br>(9·2-13·4)	24·7<br>(23·0-26·5)	25·4<br>(23·7-27·2)	35·5<br>(33·1-37·9)	70·9<br>(61·8-79·8)	74·9<br>(66·6-82·7)	75·4<br>(67·2-83·1)	81·4<br>(74·5-87·1)
Niger	28·0<br>(26·4-29·5)	34·9<br>(33·2-36·5)	35·2<br>(33·5-36·7)	39·3<br>(37·4-41·4)	2·5<br>(0·0-6·6)	1·7<br>(0·0-5·3)	1·8<br>(0·0-5·5)	1·6<br>(0·0-5·4)	2·3<br>(0·0-4·8)	12·5<br>(10·0-15·3)	12·9<br>(10·4-15·7)	19·0<br>(16·1-22·2)	60·8<br>(51·3-68·2)	90·7<br>(88·7-92·2)	92·0<br>(90·4-93·3)	99·1<br>(98·9-99·3)
Nigeria	15·0<br>(13·7-16·4)	26·8<br>(25·4-28·1)	27·4<br>(26·0-28·8)	36·6<br>(35·0-38·2)	0·7<br>(0·0-1·7)	2·2<br>(1·1-3·6)	2·4<br>(1·2-3·8)	4·6<br>(2·6-6·4)	6·0<br>(3·6-8·4)	21·1<br>(18·9-23·5)	21·7<br>(19·5-24·2)	30·2<br>(27·7-33·0)	27·2<br>(20·7-32·9)	39·7<br>(33·2-45·0)	40·9<br>(34·6-46·2)	56·1<br>(50·3-60·9)
Sao Tome and Principe	34·4<br>(32·9-36·0)	41·8<br>(40·2-43·3)	42·1<br>(40·5-43·7)	47·8<br>(46·0-49·7)	5·7<br>(0·0-12·2)	25·2<br>(23·2-26·8)	27·2<br>(25·3-28·7)	52·4<br>(41·1-65·0)	14·8<br>(12·9-16·9)	29·7<br>(27·7-31·8)	30·4<br>(28·4-32·6)	41·2<br>(38·4-43·8)	12·8<br>(0·0-32·2)	18·3<br>(5·6-33·4)	19·0<br>(6·4-33·6)	29·0<br>(16·6-40·4)
Senegal	23·1<br>(21·6-24·5)	30·3<br>(28·8-31·7)	30·6<br>(29·1-32·0)	35·2<br>(33·6-36·8)	4·0<br>(2·2-5·6)	13·0<br>(11·1-14·9)	13·6<br>(11·6-15·6)	21·8<br>(18·9-24·5)	0·1<br>(0·0-1·3)	12·2<br>(10·0-15·1)	12·6<br>(10·3-15·5)	18·5<br>(15·4-22·4)	43·2<br>(36·8-50·7)	69·4<br>(65·7-73·0)	72·0<br>(68·5-75·3)	92·0<br>(90·1-93·5)

Location	**Indicator 3.4.1:** Age-standardised death rate due to cardiovascular disease, cancer, diabetes, and chronic respiratory disease in populations aged 30-70 (per 100,000 population)				**Indicator 3.4.2:** Age-standardised death rate due to self-harm (per 100,000 population)				**Indicator 3.5.2:** Risk-weighted prevalence of alcohol consumption, as measured by the summary exposure value (SEV) for alcohol use (%)				**Indicator 3.6.1:** Age-standardised death rate due to road injuries (per 100,000 population)			
	**2000**	**2015**	**2016**	**2030**	**2000**	**2015**	**2016**	**2030**	**2000**	**2015**	**2016**	**2030**	**2000**	**2015**	**2016**	**2030**
Bangladesh	41·5 (36·7-46·2)	48·3 (41·9-54·6)	48·9 (42·1-55·4)	57·3 (42·0-72·0)	45·4 (38·9-50·6)	55·8 (49·3-62·2)	56·9 (50·1-63·6)	64·9 (50·4-80·3)	99·9 (99·7-100·0)	99·8 (99·5-100·0)	99·8 (99·5-100·0)	99·8 (99·4-100·0)	62·6 (54·7-70·6)	45·7 (39·7-51·9)	47·3 (41·0-53·8)	40·0 (25·7-55·7)
Bhutan	46·0 (39·2-52·8)	59·2 (50·0-67·9)	59·7 (50·3-68·7)	67·6 (48·9-85·7)	53·0 (43·3-62·6)	60·6 (50·7-70·0)	60·8 (50·4-70·4)	65·7 (46·9-82·9)	79·0 (70·9-86·3)	86·2 (77·4-93·8)	86·3 (77·5-93·9)	87·5 (78·4-95·0)	43·8 (37·1-51·7)	56·9 (47·8-66·7)	57·6 (48·5-67·6)	69·8 (53·8-85·8)
India	31·1 (27·8-35·6)	38·3 (35·3-41·6)	39·2 (36·3-42·4)	44·5 (39·7-49·4)	16·2 (11·5-25·0)	21·5 (17·1-29·7)	22·3 (17·6-30·5)	27·6 (19·2-35·0)	89·8 (87·8-91·6)	87·0 (84·9-89·0)	86·8 (84·6-88·9)	83·8 (80·8-86·6)	32·6 (29·9-35·7)	31·2 (28·7-34·6)	32·0 (29·4-35·5)	31·0 (27·4-35·9)
Nepal	33·8 (29·1-39·2)	42·5 (37·3-47·9)	43·1 (38·0-48·3)	52·1 (42·6-61·0)	31·3 (20·4-44·9)	40·9 (33·0-53·0)	41·7 (33·9-53·3)	53·8 (41·1-67·4)	92·4 (90·5-94·3)	89·4 (83·3-93·8)	89·4 (83·0-93·9)	89·8 (79·8-96·0)	29·0 (19·8-36·5)	34·2 (25·3-43·5)	34·8 (26·1-43·9)	40·2 (28·7-53·2)
Pakistan	22·7 (17·9-27·8)	30·1 (21·3-38·6)	30·8 (21·8-39·5)	39·1 (23·1-56·8)	75·7 (68·1-82·0)	84·5 (76·6-92·0)	85·1 (77·0-92·6)	92·8 (77·8-100·0)	100·0 (99·9-100·0)	100·0 (100·0-100·0)	100·0 (100·0-100·0)	100·0 (99·9-100·0)	29·7 (20·0-37·1)	28·1 (16·2-36·7)	28·6 (16·9-37·4)	31·1 (14·5-46·9)
**Southern Sub-Saharan Africa**																
Botswana	25·4 (0·0-62·6)	39·9 (14·8-83·1)	39·8 (15·9-79·8)	56·7 (0·2-100·0)	17·1 (0·0-46·2)	24·6 (4·6-57·4)	24·6 (5·0-54·4)	39·3 (0·0-93·4)	47·7 (36·2-58·5)	46·0 (28·5-64·6)	46·6 (29·0-65·3)	54·2 (36·2-74·3)	24·6 (0·0-48·1)	33·1 (16·4-59·8)	33·2 (17·4-59·5)	46·3 (8·9-100·0)
Lesotho	19·1 (6·7-38·5)	11·9 (0·0-27·1)	13·2 (0·0-28·9)	14·8 (0·0-46·9)	2·5 (0·0-15·6)	0·5 (0·0-6·2)	0·5 (0·0-6·7)	2·0 (0·0-19·2)	34·6 (20·7-50·0)	34·2 (16·3-55·0)	34·7 (16·5-55·7)	40·9 (20·7-64·1)	6·4 (0·0-16·8)	1·5 (0·0-9·2)	1·8 (0·0-9·6)	3·3 (0·0-19·2)
Namibia	24·6 (8·5-48·7)	51·6 (35·5-73·3)	52·6 (36·2-74·7)	77·2 (46·2-100·0)	22·4 (8·4-39·6)	35·3 (20·5-49·4)	35·7 (20·7-49·5)	43·0 (18·8-70·0)	69·9 (52·9-83·1)	33·7 (6·8-65·2)	34·1 (6·8-66·2)	39·4 (5·3-78·1)	5·7 (0·0-17·6)	17·0 (7·5-29·9)	17·6 (8·1-30·7)	28·3 (9·2-52·0)
South Africa	30·2 (26·2-34·4)	44·9 (41·4-48·3)	45·3 (41·5-49·0)	52·8 (43·2-62·4)	15·7 (7·9-26·6)	32·5 (27·0-38·6)	31·8 (26·2-38·1)	45·9 (32·9-58·9)	55·4 (46·4-64·4)	55·1 (46·0-64·5)	55·0 (45·9-64·5)	54·5 (44·8-64·9)	0·1 (0·0-0·9)	9·0 (5·5-13·5)	8·5 (4·9-13·0)	15·1 (6·4-23·7)
Swaziland	24·2 (0·0-46·9)	29·2 (7·0-55·9)	30·5 (8·7-56·5)	54·3 (0·0-100·0)	19·9 (0·0-38·8)	20·8 (4·8-39·6)	21·3 (5·3-39·4)	32·9 (0·0-66·8)	59·4 (51·6-66·9)	68·5 (55·9-82·0)	68·8 (56·0-82·6)	73·3 (59·7-89·1)	4·1 (0·0-15·3)	8·7 (0·0-23·3)	9·4 (0·0-24·2)	27·1 (0·0-55·7)
Zimbabwe	31·4 (10·5-51·8)	31·0 (18·1-44·3)	31·6 (18·9-45·0)	43·0 (14·4-69·6)	10·3 (0·0-25·1)	5·4 (0·0-15·5)	5·4 (0·0-15·7)	8·9 (0·0-29·7)	70·7 (63·3-77·5)	69·5 (53·2-82·9)	69·4 (52·2-83·3)	67·7 (38·4-88·0)	8·5 (0·0-21·1)	12·2 (4·2-20·7)	12·5 (4·0-21·4)	19·9 (2·5-36·4)
**Western Sub-Saharan Africa**																
Benin	46·5 (40·6-53·0)	46·5 (40·9-53·1)	46·8 (41·1-53·2)	47·9 (37·2-58·4)	32·9 (23·1-44·2)	32·7 (24·6-41·9)	32·7 (24·5-41·8)	32·7 (20·3-44·0)	85·2 (79·2-90·2)	83·2 (71·0-91·5)	83·0 (70·5-91·5)	81·0 (61·6-92·3)	22·7 (11·6-30·0)	28·5 (17·7-36·3)	28·9 (18·1-36·7)	33·5 (19·0-44·9)
Burkina Faso	44·9 (38·1-51·5)	45·0 (40·4-50·1)	45·3 (40·4-50·3)	45·8 (36·1-55·5)	35·8 (29·2-42·1)	35·9 (29·6-41·6)	35·7 (28·9-41·5)	34·3 (21·5-45·0)	44·7 (27·2-61·7)	54·1 (25·3-77·2)	54·7 (25·5-78·1)	63·0 (28·6-87·2)	25·1 (20·5-29·9)	27·8 (23·7-32·0)	28·1 (23·8-32·3)	31·6 (22·3-40·2)
Cameroon	39·6 (29·8-49·5)	43·0 (31·2-55·4)	43·5 (31·4-56·4)	47·5 (27·1-70·8)	26·0 (16·6-37·2)	24·6 (15·2-34·9)	24·7 (15·2-35·3)	25·0 (9·0-41·5)	65·0 (56·1-72·6)	64·7 (54·0-73·8)	65·1 (54·5-74·2)	70·7 (61·3-78·9)	25·8 (19·2-32·9)	28·7 (21·1-36·7)	29·0 (21·2-37·1)	32·9 (19·2-48·2)
Cape Verde	54·1 (44·5-65·2)	58·0 (50·0-66·7)	58·5 (50·0-68·0)	63·5 (44·3-85·4)	21·4 (10·6-32·4)	17·4 (9·3-26·9)	17·7 (9·1-27·1)	17·9 (0·0-38·2)	67·9 (60·6-75·0)	68·9 (59·2-76·6)	69·3 (59·6-76·9)	74·5 (65·7-81·3)	52·5 (44·5-60·3)	56·1 (49·7-63·2)	56·6 (49·9-63·9)	63·3 (47·5-80·1)
Chad	44·7 (37·5-52·7)	49·4 (41·7-57·3)	49·7 (42·1-57·8)	54·5 (40·7-69·1)	40·2 (27·9-56·6)	45·9 (30·5-61·7)	46·7 (31·2-61·9)	58·2 (35·9-76·0)	80·9 (77·6-84·1)	77·8 (69·1-85·4)	77·9 (69·0-85·9)	80·2 (66·3-90·9)	31·3 (25·5-37·3)	34·8 (28·7-41·3)	35·2 (29·2-41·7)	41·3 (31·0-52·6)
Cote d'Ivoire	29·6 (20·4-38·0)	33·6 (25·8-41·0)	34·2 (26·3-41·4)	39·4 (24·4-53·0)	25·5 (16·7-33·2)	25·6 (17·4-32·8)	25·4 (17·1-32·6)	24·8 (10·6-37·0)	62·8 (47·8-76·7)	68·8 (48·5-85·6)	69·2 (48·5-86·2)	75·0 (51·3-92·7)	18·6 (12·5-24·4)	23·9 (18·2-29·1)	24·1 (18·3-29·5)	27·9 (16·2-38·3)
The Gambia	49·1 (42·4-55·9)	51·4 (44·4-59·0)	51·7 (44·5-59·4)	53·6 (40·0-68·9)	55·6 (39·1-66·5)	58·6 (40·7-69·7)	58·6 (40·6-69·9)	58·9 (35·5-75·3)	83·1 (75·1-89·2)	83·2 (69·4-92·7)	83·5 (69·6-93·0)	87·5 (73·4-95·9)	37·5 (32·2-43·4)	41·8 (36·4-48·0)	42·2 (36·5-48·5)	45·9 (34·7-57·5)
Ghana	40·7 (32·5-50·6)	46·3 (39·7-54·3)	47·2 (40·5-55·3)	56·0 (42·5-72·2)	53·7 (46·4-61·3)	52·7 (45·4-60·1)	53·0 (45·2-60·4)	55·9 (41·0-71·1)	52·5 (38·0-67·7)	44·4 (28·0-64·9)	44·1 (27·1-65·2)	40·0 (16·4-68·5)	32·8 (27·5-38·8)	33·7 (28·3-39·4)	34·3 (28·6-39·8)	36·3 (24·8-47·7)
Guinea	41·0 (34·7-47·3)	37·6 (29·5-46·2)	37·6 (29·4-46·4)	36·8 (20·9-54·2)	50·6 (35·9-63·5)	48·2 (32·4-62·3)	48·2 (32·1-62·4)	48·0 (27·0-67·7)	97·0 (95·6-98·3)	97·3 (95·3-98·7)	97·3 (95·2-98·7)	96·7 (93·8-98·7)	32·3 (27·2-38·0)	36·0 (30·0-42·7)	36·2 (30·1-42·7)	39·6 (29·1-50·8)
Guinea-Bissau	20·3 (13·3-27·9)	22·0 (15·1-29·7)	22·5 (15·5-30·1)	26·7 (12·7-41·2)	37·5 (26·6-48·9)	37·1 (21·7-49·1)	37·3 (21·8-49·5)	38·5 (16·1-56·5)	74·1 (63·8-82·3)	74·4 (57·2-87·4)	74·5 (57·1-87·6)	75·8 (55·3-90·5)	17·3 (11·1-24·3)	23·8 (17·2-31·5)	24·2 (17·6-31·8)	30·8 (19·1-42·3)
Liberia	50·5 (43·0-59·0)	47·2 (41·7-53·8)	47·4 (42·0-53·8)	46·3 (35·5-57·2)	36·4 (27·6-46·6)	25·8 (18·5-34·0)	25·7 (18·4-34·1)	24·4 (11·5-39·8)	54·4 (44·2-64·7)	64·6 (45·2-80·4)	64·5 (44·2-80·9)	63·7 (30·3-87·1)	40·8 (35·6-47·6)	45·2 (40·2-51·2)	45·4 (40·5-51·6)	48·8 (39·2-59·5)
Mali	36·7 (30·2-43·9)	47·1 (37·0-56·8)	47·1 (36·4-57·5)	54·8 (30·6-77·2)	66·3 (48·4-80·2)	68·3 (46·4-84·1)	68·2 (46·0-84·1)	68·2 (37·8-90·1)	90·8 (88·4-93·5)	91·0 (87·2-95·2)	91·0 (87·2-95·3)	91·3 (87·0-96·1)	37·4 (32·0-44·2)	44·8 (37·9-52·8)	44·9 (37·7-53·5)	48·7 (33·7-63·5)
Mauritania	49·2 (40·4-63·3)	60·6 (48·7-75·0)	60·7 (49·1-75·1)	67·0 (41·0-93·4)	67·8 (44·9-83·7)	72·0 (44·3-90·0)	71·6 (43·9-89·6)	70·6 (36·0-98·6)	99·6 (99·3-99·9)	99·8 (99·3-100·0)	99·8 (99·2-100·0)	99·2 (97·3-100·0)	42·9 (35·1-53·2)	53·9 (45·5-66·3)	54·1 (45·4-66·0)	61·4 (45·0-79·5)
Niger	52·6 (44·5-60·5)	50·2 (40·8-59·5)	50·0 (40·4-60·1)	48·3 (29·2-66·9)	58·8 (42·2-74·8)	56·8 (36·2-73·7)	56·5 (35·7-73·3)	54·2 (28·6-75·6)	96·7 (94·8-98·4)	96·6 (93·9-98·5)	96·6 (93·8-98·5)	96·5 (92·8-98·8)	40·0 (33·4-46·1)	44·1 (37·3-51·6)	44·2 (37·6-51·6)	46·6 (34·2-60·4)
Nigeria	63·9 (51·6-78·7)	79·8 (68·7-92·4)	79·4 (68·5-92·0)	91·7 (72·9-100·0)	49·6 (33·6-66·9)	61·5 (40·2-79·3)	61·6 (40·6-78·7)	69·3 (38·4-95·4)	36·8 (23·2-49·9)	53·4 (31·1-75·0)	54·7 (32·2-76·3)	70·1 (45·7-89·8)	40·5 (31·3-52·9)	48·5 (39·0-59·4)	48·8 (39·1-59·5)	55·6 (39·0-72·2)
Sao Tome and Principe	45·7 (37·5-63·6)	53·1 (44·3-64·4)	53·6 (44·6-63·5)	61·4 (42·3-81·5)	98·0 (90·3-100·0)	94·7 (84·2-100·0)	94·6 (84·1-100·0)	91·6 (71·4-100·0)	63·9 (53·7-73·1)	66·3 (56·5-74·5)	66·8 (57·0-74·8)	72·3 (63·9-79·2)	45·6 (37·8-59·8)	45·3 (36·8-56·8)	45·4 (37·0-55·3)	49·6 (33·1-64·9)
Senegal	42·6 (37·2-48·2)	41·2 (36·3-46·3)	41·5 (36·5-46·6)	43·9 (34·5-52·4)	36·3 (30·0-42·7)	33·2 (27·1-39·6)	33·5 (27·5-40·0)	31·9 (19·8-44·5)	96·7 (95·5-97·8)	97·3 (95·3-98·9)	97·3 (95·2-98·9)	97·0 (94·0-99·2)	32·9 (27·0-40·3)	35·4 (29·6-43·2)	34·9 (29·2-43·4)	38·1 (28·6-49·3)

Location	**Indicator 3.7.1:** Proportion of women of reproductive age (15-49 years) who have their need for family planning satisfied with modern contraception methods (%)				**Indicator 3.7.2:** Number of livebirths per 1,000 women aged 10-14 years and women aged 15-19 years				**Indicator 3.8.1:** Coverage of essential health services, as defined by the UHC index comprised of the coverage of 9 tracer interventions and risk-standardised death rates from 32 causes amenable to personal healthcare (scale of 0 to 100)				**Indicator 3.9.1:** Age-standardised death rate attributable to household air pollution and ambient air pollution (per 100,000 population)			
	**2000**	**2015**	**2016**	**2030**	**2000**	**2015**	**2016**	**2030**	**2000**	**2015**	**2016**	**2030**	**2000**	**2015**	**2016**	**2030**
Bangladesh	65·6 (62·7-68·5)	79·6 (76·5-82·7)	80·0 (76·1-83·8)	88·0 (81·6-93·4)	10·3 (8·8-12·0)	17·2 (15·3-19·4)	17·6 (15·6-20·1)	24·9 (19·8-30·4)	19·1 (15·4-22·7)	45·6 (40·9-50·2)	47·0 (42·2-51·7)	70·1 (63·5-76·3)	8·8 (6·1-11·7)	14·2 (10·8-17·5)	14·6 (11·2-18·0)	21·1 (17·0-25·1)
Bhutan	59·2 (50·5-67·7)	80·0 (71·7-86·6)	80·7 (72·7-87·4)	93·5 (83·3-100·0)	11·3 (9·3-13·7)	47·8 (44·2-51·9)	51·2 (47·2-55·7)	94·8 (84·7-100·0)	24·8 (20·5-29·6)	48·1 (42·4-54·0)	49·1 (43·3-55·0)	67·0 (59·8-74·4)	14·7 (10·5-19·5)	30·7 (25·2-35·7)	31·4 (25·9-36·5)	42·8 (35·3-50·2)
India	70·9 (67·4-74·0)	80·9 (75·1-86·5)	80·7 (74·9-86·1)	84·7 (73·4-95·1)	22·1 (20·8-23·5)	51·6 (49·4-53·8)	54·5 (52·2-56·8)	82·9 (78·2-88·0)	15·5 (12·8-18·3)	36·8 (33·8-39·4)	38·3 (35·4-41·0)	59·2 (54·5-62·9)	6·0 (2·8-9·4)	14·6 (11·8-17·3)	15·2 (12·4-17·9)	23·3 (20·2-26·1)
Nepal	51·5 (48·2-54·6)	62·3 (58·8-65·9)	63·5 (59·2-68·0)	72·8 (60·9-83·3)	6·7 (2·2-12·1)	9·9 (4·5-15·7)	10·2 (4·3-17·2)	14·2 (0·0-29·8)	15·9 (12·0-19·5)	40·8 (35·8-45·5)	42·2 (37·2-46·8)	64·4 (58·0-69·9)	6·2 (2·7-10·2)	11·6 (8·2-15·3)	12·2 (8·7-15·9)	18·2 (13·6-22·4)
Pakistan	33·2 (26·4-40·3)	46·6 (43·3-50·3)	47·7 (43·0-52·4)	64·6 (54·3-74·8)	31·8 (29·3-34·2)	37·6 (33·7-41·8)	38·8 (34·4-43·4)	46·8 (36·1-56·9)	9·6 (5·4-13·4)	25·3 (20·0-30·4)	26·1 (20·7-31·1)	42·3 (34·8-50·0)	5·3 (2·7-8·1)	12·8 (9·2-16·3)	13·4 (9·7-16·9)	20·3 (15·4-24·8)
**Southern Sub-Saharan Africa**																
Botswana	73·5 (64·6-81·7)	91·2 (85·2-96·3)	91·3 (85·4-96·3)	99·2 (93·4-100·0)	22·4 (14·1-32·2)	41·1 (31·7-51·2)	43·1 (33·4-53·5)	67·1 (46·9-92·0)	31·6 (9·0-50·2)	51·0 (38·9-70·8)	52·0 (40·0-72·6)	65·8 (55·6-93·9)	15·6 (0·0-31·2)	34·7 (22·8-52·0)	35·7 (23·9-53·8)	50·6 (40·0-78·2)
Lesotho	51·5 (45·0-58·2)	85·7 (82·2-88·7)	85·9 (81·9-89·4)	99·9 (99·0-100·0)	15·8 (11·7-20·9)	13·6 (9·5-18·4)	13·2 (9·0-18·3)	9·8 (0·0-21·9)	18·8 (12·3-26·6)	23·7 (16·3-31·9)	24·3 (16·7-32·5)	35·2 (25·3-45·2)	6·7 (1·2-13·6)	10·5 (4·5-17·6)	11·1 (5·0-18·4)	17·0 (9·6-27·2)
Namibia	72·9 (69·8-76·2)	88·5 (85·1-91·6)	88·7 (84·5-92·5)	96·3 (90·1-100·0)	15·0 (12·9-17·3)	17·2 (14·8-19·9)	17·4 (14·9-20·4)	20·6 (14·1-27·6)	24·8 (15·8-35·0)	47·2 (39·2-58·2)	48·1 (40·0-59·6)	65·0 (55·2-80·1)	8·9 (1·6-18·6)	27·3 (19·3-37·9)	28·3 (20·3-39·2)	42·9 (33·2-57·3)
South Africa	85·9 (82·4-88·9)	94·4 (88·9-98·9)	94·5 (89·1-99·0)	98·7 (91·6-100·0)	20·7 (18·0-23·6)	30·1 (27·4-33·1)	30·8 (27·7-34·3)	41·0 (33·8-47·5)	29·2 (25·9-32·5)	43·2 (40·3-46·1)	43·9 (41·1-46·9)	59·1 (55·6-62·7)	24·5 (22·2-26·9)	41·6 (38·6-44·4)	42·8 (39·8-45·7)	60·4 (56·8-63·9)
Swaziland	50·8 (42·7-58·9)	91·4 (87·6-95·0)	91·4 (87·0-95·2)	99·8 (97·9-100·0)	11·4 (8·2-15·3)	16·6 (13·9-19·8)	18·9 (16·1-22·4)	21·4 (14·0-29·1)	26·0 (12·6-34·4)	37·4 (27·0-49·4)	38·9 (28·4-51·4)	62·6 (51·8-79·4)	10·1 (0·0-18·0)	24·2 (14·6-35·3)	25·7 (16·0-37·3)	47·8 (34·3-67·9)
Zimbabwe	77·4 (74·3-80·4)	92·8 (90·7-94·8)	93·1 (90·7-95·4)	100·0 (99·6-100·0)	10·1 (8·6-12·0)	8·2 (6·7-10·1)	8·4 (6·9-10·4)	10·2 (5·6-15·4)	22·5 (13·4-30·8)	28·8 (22·5-34·9)	30·6 (24·4-36·8)	56·2 (50·7-61·5)	13·8 (5·5-21·8)	18·1 (11·4-25·2)	19·1 (12·4-26·0)	29·3 (16·6-36·8)
**Western Sub-Saharan Africa**																
Benin	3·9 (1·7-6·2)	16·6 (11·5-22·2)	17·5 (11·8-24·3)	32·2 (17·7-49·4)	9·4 (7·8-11·0)	14·1 (12·5-16·2)	14·6 (12·8-16·8)	21·1 (16·3-26·8)	18·9 (15·5-22·3)	30·4 (27·0-33·7)	31·6 (28·2-34·9)	43·0 (38·3-47·5)	6·6 (3·4-10·2)	10·3 (7·1-13·5)	10·2 (7·0-13·4)	12·3 (8·5-17·6)
Burkina Faso	11·0 (7·6-14·3)	45·2 (42·6-48·0)	48·5 (45·5-51·5)	82·1 (75·7-87·9)	4·1 (0·3-9·7)	10·5 (4·7-18·3)	10·9 (4·9-18·8)	17·9 (4·2-36·2)	12·8 (9·4-16·3)	31·3 (27·8-34·8)	31·6 (28·0-35·1)	41·8 (36·0-47·3)	6·8 (3·8-10·6)	10·7 (7·9-14·1)	10·7 (7·9-14·1)	11·4 (8·2-15·0)
Cameroon	10·3 (7·4-13·3)	38·6 (34·6-42·7)	39·0 (33·8-44·1)	56·1 (39·2-72·4)	3·3 (2·1-4·7)	10·5 (9·4-12·0)	11·4 (10·2-13·0)	17·5 (14·3-21·1)	15·0 (10·0-19·8)	28·8 (23·2-34·9)	29·8 (24·0-36·0)	43·1 (35·2-51·3)	5·3 (1·0-9·7)	9·7 (4·4-15·1)	9·9 (4·5-15·3)	11·8 (5·4-18·6)
Cape Verde	85·9 (81·1-89·9)	84·0 (76·1-90·8)	84·7 (76·9-90·9)	93·7 (79·6-100·0)	9·3 (7·8-11·0)	8·5 (5·6-11·2)	8·9 (5·9-11·8)	13·6 (6·4-20·5)	41·1 (35·9-46·7)	59·0 (54·7-64·1)	60·1 (55·6-65·1)	76·3 (70·8-82·0)	24·2 (19·3-30·0)	32·9 (24·9-40·3)	33·1 (24·9-40·7)	38·8 (26·7-50·4)
Chad	0·0 (0·0-0·0)	4·0 (1·4-6·6)	4·2 (1·1-7·4)	10·2 (1·9-19·6)	0·0 (0·0-0·0)	6·0 (5·2-6·8)	7·0 (6·3-7·8)	14·4 (13·2-15·5)	0·5 (0·0-3·2)	13·5 (9·3-17·3)	14·5 (10·3-18·4)	29·5 (23·1-35·6)	3·4 (0·0-7·2)	7·2 (3·4-10·6)	7·4 (3·5-10·9)	10·4 (6·6-13·8)
Cote d'Ivoire	12·9 (8·7-17·3)	27·2 (21·0-34·6)	27·9 (21·0-36·0)	39·9 (24·5-60·3)	7·1 (3·7-10·9)	4·6 (0·6-9·6)	4·3 (0·0-9·4)	3·9 (0·0-15·1)	13·8 (9·2-17·9)	25·1 (20·4-29·4)	25·9 (21·2-30·2)	37·0 (30·9-42·5)	4·9 (0·7-8·6)	8·0 (3·8-12·5)	8·0 (3·7-12·5)	7·9 (3·0-13·6)
The Gambia	10·5 (5·0-16·7)	21·2 (16·9-25·8)	22·0 (16·6-27·7)	35·7 (21·3-50·8)	4·3 (1·9-7·2)	10·1 (7·4-13·6)	10·2 (7·2-14·0)	14·6 (6·1-23·6)	29·8 (25·5-33·9)	38·8 (34·2-43·3)	39·5 (34·8-44·1)	48·1 (42·6-53·6)	9·5 (5·6-13·6)	12·4 (8·2-16·7)	12·5 (8·3-16·8)	13·6 (8·9-18·3)
Ghana	19·8 (15·9-24·0)	35·0 (32·2-38·0)	35·7 (31·6-40·0)	52·9 (41·7-63·5)	18·2 (15·6-21·3)	20·5 (17·8-23·8)	20·8 (18·0-24·3)	25·3 (17·6-33·7)	25·1 (20·8-29·7)	41·3 (37·1-45·7)	42·4 (38·1-47·0)	58·4 (53·2-64·1)	8·6 (5·0-12·8)	14·3 (10·9-18·2)	14·7 (11·3-18·6)	20·2 (16·7-24·0)
Guinea	6·3 (3·7-9·1)	14·6 (9·6-20·0)	15·4 (9·9-21·7)	26·8 (14·0-42·3)	1·2 (0·4-2·2)	3·0 (2·2-4·0)	3·1 (2·3-4·1)	4·8 (3·9-5·8)	8·7 (5·1-12·2)	19·0 (14·5-23·5)	19·7 (15·1-24·4)	27·7 (21·3-34·3)	5·0 (1·4-8·5)	6·0 (2·2-10·0)	6·0 (2·1-10·0)	6·1 (2·1-10·5)
Guinea-Bissau	1·3 (0·0-5·3)	44·5 (37·4-51·2)	44·2 (37·2-51·6)	85·1 (66·9-100·0)	6·3 (5·6-7·1)	14·5 (13·5-15·4)	15·4 (14·4-16·3)	28·1 (26·5-29·6)	3·2 (0·0-7·5)	16·6 (12·5-21·0)	17·4 (13·3-21·8)	30·4 (24·5-36·3)	0·1 (0·0-1·0)	0·8 (0·0-3·7)	0·9 (0·0-3·8)	2·8 (0·0-6·3)
Liberia	9·7 (4·5-15·8)	38·6 (33·5-43·7)	39·0 (32·8-45·3)	74·0 (60·8-86·2)	0·9 (0·0-2·5)	10·3 (8·1-12·8)	10·7 (8·4-13·5)	19·2 (12·9-27·1)	14·0 (9·8-18·2)	30·9 (27·4-34·3)	31·8 (28·3-35·2)	43·4 (38·3-48·3)	13·1 (9·3-17·3)	15·9 (12·8-19·7)	16·0 (12·8-19·7)	16·9 (13·9-20·1)
Mali	6·0 (3·8-8·4)	23·1 (18·0-28·4)	23·5 (17·4-29·9)	37·9 (26·1-53·8)	0·0 (0·0-0·0)	0·0 (0·0-0·5)	0·1 (0·0-0·6)	1·3 (0·0-3·5)	8·5 (4·4-12·5)	26·8 (21·0-32·3)	28·1 (22·2-33·8)	42·1 (34·2-50·0)	11·1 (7·8-14·7)	17·6 (12·4-22·4)	17·7 (12·3-22·6)	22·7 (15·2-29·7)
Mauritania	1·8 (0·0-4·5)	2·6 (0·0-6·7)	3·9 (0·1-8·6)	4·8 (0·0-14·3)	17·2 (16·2-18·4)	22·7 (21·7-23·7)	23·1 (22·0-24·0)	27·7 (26·3-29·2)	20·2 (15·4-27·9)	38·2 (31·4-45·9)	39·3 (32·4-47·0)	54·8 (46·6-63·9)	10·8 (6·5-15·5)	21·0 (14·8-28·0)	21·2 (14·8-28·3)	26·1 (17·6-36·4)
Niger	3·5 (0·8-6·7)	34·9 (31·0-38·9)	38·9 (34·8-43·1)	77·0 (67·6-86·0)	0·0 (0·0-0·0)	0·0 (0·0-0·0)	0·0 (0·0-0·0)	1·8 (0·0-4·2)	4·7 (0·8-8·7)	24·5 (19·4-29·5)	25·0 (19·9-30·1)	38·5 (31·5-45·5)	2·1 (0·0-5·6)	5·8 (1·5-10·2)	5·7 (1·3-10·2)	9·9 (3·8-17·8)
Nigeria	16·3 (11·4-21·9)	29·6 (26·6-32·7)	29·0 (25·4-32·5)	47·3 (37·1-57·2)	6·0 (3·6-9·3)	11·6 (9·1-14·8)	12·1 (9·3-15·8)	17·0 (10·1-25·1)	15·2 (9·5-21·6)	34·4 (28·9-40·6)	35·0 (29·6-41·4)	50·0 (41·7-58·6)	13·0 (6·9-18·8)	23·0 (16·2-29·2)	23·1 (16·4-29·4)	26·6 (20·3-32·8)
Sao Tome and Principe	41·7 (32·4-50·6)	52·3 (48·1-56·5)	53·1 (47·7-58·5)	63·7 (50·9-75·8)	11·8 (11·0-12·7)	14·4 (13·4-15·5)	14·6 (13·5-15·8)	19·0 (17·0-21·0)	31·4 (26·3-42·4)	47·1 (41·7-53·5)	47·7 (42·3-53·7)	59·2 (52·9-64·4)	9·2 (5·6-14·2)	22·8 (14·9-34·3)	23·7 (15·3-35·6)	36·0 (22·2-56·8)
Senegal	5·7 (2·6-9·2)	40·6 (37·6-43·6)	41·0 (37·1-44·9)	73·9 (65·6-81·0)	12·3 (10·8-14·1)	19·1 (17·5-21·0)	20·0 (18·2-22·0)	28·9 (24·1-34·5)	15·6 (12·6-18·4)	28·0 (24·8-31·0)	29·0 (25·8-32·1)	41·7 (36·9-46·4)	12·2 (9·3-15·2)	14·7 (10·9-19·5)	14·8 (10·8-19·8)	16·1 (11·1-22·8)

Location	Indicator 3.9.2: Age-standardised death rate attributable to unsafe water, sanitation, and hygiene (WaSH) (per 100,000 population)				Indicator 3.9.3: Age-standardised death rate due to unintentional poisonings (per 100,000 population)				Indicator 3.a.1: Age-standardised prevalence of daily smoking in populations aged 10 and older (%)				Indicator 3.b.1: Geometric mean of the coverage of eight vaccines, conditional on inclusion in national vaccine schedules, in target populations (%)			
	**2000**	**2015**	**2016**	**2030**	**2000**	**2015**	**2016**	**2030**	**2000**	**2015**	**2016**	**2030**	**2000**	**2015**	**2016**	**2030**
Bangladesh	11·3 (8·7-15·4)	24·4 (18·9-28·7)	25·3 (19·7-29·4)	37·0 (31·0-40·9)	56·9 (45·8-65·5)	66·7 (58·8-82·3)	68·4 (60·4-83·2)	82·4 (60·1-100·0)	50·1 (43·7-56·0)	37·8 (30·1-45·2)	38·7 (30·4-46·1)	45·0 (27·2-59·9)	77·8 (74·2-81·0)	90·7 (87·2-93·2)	91·0 (87·1-93·8)	96·4 (90·3-99·2)
Bhutan	20·7 (10·9-39·5)	38·0 (26·4-55·1)	38·8 (27·2-55·9)	51·2 (37·4-67·8)	42·5 (28·0-53·2)	64·1 (50·1-76·6)	65·3 (51·3-77·9)	86·6 (60·7-100·0)	94·4 (89·6-98·4)	97·4 (95·5-99·2)	97·5 (95·1-99·4)	98·4 (93·9-100·0)	81·0 (76·7-84·6)	95·4 (92·9-97·0)	95·6 (93·1-97·2)	98·8 (96·5-99·8)
India	1·8 (0·0-4·5)	10·2 (8·0-13·0)	10·9 (8·7-13·7)	19·6 (16·9-23·1)	45·2 (41·0-53·2)	59·2 (53·6-68·1)	60·4 (54·4-69·5)	75·0 (63·0-87·5)	61·7 (59·6-63·6)	68·6 (66·6-70·6)	69·4 (67·4-71·4)	77·0 (72·4-81·1)	48·4 (44·9-51·7)	74·5 (72·4-76·6)	76·5 (74·3-78·7)	90·9 (88·3-93·0)
Nepal	2·7 (0·0-5·6)	17·1 (11·5-22·1)	18·3 (12·6-23·6)	35·7 (26·7-43·5)	46·1 (31·6-56·2)	63·3 (47·8-72·9)	64·6 (48·8-74·1)	82·4 (60·8-99·8)	0·3 (0·0-3·5)	48·0 (40·5-54·8)	47·1 (38·5-54·8)	59·7 (39·2-76·4)	65·4 (60·1-70·4)	81·1 (73·7-86·7)	82·7 (74·9-88·4)	94·4 (85·0-98·9)
Pakistan	14·2 (9·9-19·1)	22·3 (16·1-27·5)	22·9 (16·6-28·3)	32·2 (22·6-39·4)	37·0 (26·3-47·0)	49·2 (37·6-58·6)	50·0 (37·9-59·5)	65·1 (43·5-83·0)	48·7 (41·9-55·4)	66·4 (58·7-72·9)	66·6 (58·4-73·4)	77·9 (64·8-87·0)	58·6 (54·9-62·2)	61·4 (51·1-69·7)	61·2 (49·7-70·1)	60·2 (27·1-80·5)
**Southern Sub-Saharan Africa**																
Botswana	13·2 (3·2-21·9)	21·4 (14·7-29·0)	22·0 (15·5-29·7)	30·0 (23·4-39·9)	17·1 (0·0-34·4)	31·2 (16·0-50·1)	31·2 (15·5-50·1)	47·9 (19·4-85·7)	65·7 (60·7-70·2)	68·0 (63·5-72·4)	68·4 (63·8-72·9)	70·8 (62·2-78·3)	85·0 (80·0-88·9)	95·9 (93·1-97·7)	97·1 (94·9-98·5)	99·8 (98·7-100·0)
Lesotho	1·4 (0·0-6·4)	5·9 (0·5-12·5)	6·5 (0·9-13·3)	11·0 (2·6-20·6)	10·8 (0·0-21·7)	10·1 (0·0-21·5)	10·6 (0·0-22·3)	16·3 (0·0-39·8)	69·5 (66·4-72·6)	59·6 (56·5-62·6)	60·0 (56·9-63·2)	50·3 (42·6-58·7)	73·4 (70·0-76·4)	77·2 (71·9-81·6)	75·9 (69·4-81·3)	80·4 (61·7-92·1)
Namibia	11·4 (6·9-15·6)	18·9 (14·2-23·7)	19·6 (14·8-24·5)	27·3 (20·7-34·1)	13·6 (0·7-27·1)	31·9 (15·6-44·8)	32·4 (16·7-45·5)	49·6 (23·5-71·4)	62·7 (58·4-66·6)	70·5 (65·7-74·5)	70·7 (66·0-74·9)	75·2 (66·5-83·0)	59·9 (55·2-64·3)	80·8 (74·8-85·8)	80·2 (73·1-85·9)	90·1 (74·9-96·6)
South Africa	15·4 (13·5-17·1)	24·7 (22·5-26·8)	25·4 (23·1-27·5)	34·7 (32·0-37·9)	22·4 (17·8-26·5)	37·9 (32·4-42·1)	38·0 (32·1-42·4)	52·3 (41·4-62·2)	41·6 (39·5-43·9)	65·9 (63·8-67·8)	66·2 (64·0-68·1)	82·1 (79·3-85·1)	64·2 (59·3-68·6)	79·0 (71·0-85·1)	78·5 (69·0-85·5)	89·8 (74·8-97·4)
Swaziland	7·8 (2·9-12·3)	13·6 (9·0-17·8)	14·3 (9·7-18·6)	19·2 (11·1-27·0)	12·7 (0·0-24·7)	17·7 (4·6-29·7)	18·3 (5·4-30·5)	32·9 (5·1-57·6)	90·4 (88·0-92·7)	91·0 (89·1-92·8)	91·1 (89·1-92·9)	93·2 (89·1-96·7)	82·4 (77·3-86·3)	94·0 (90·7-96·2)	94·2 (90·7-96·5)	99·1 (96·3-100·0)
Zimbabwe	16·3 (12·3-19·5)	14·4 (10·7-17·6)	14·9 (11·2-18·2)	14·5 (2·9-22·2)	7·0 (0·0-16·6)	20·4 (0·0-31·4)	21·4 (0·0-33·0)	36·6 (5·3-60·5)	69·7 (66·3-72·8)	69·8 (66·6-72·7)	70·0 (66·9-73·1)	71·1 (64·7-77·0)	58·6 (51·8-65·0)	93·6 (90·6-95·7)	95·7 (93·4-97·3)	99·9 (99·5-100·0)
**Western Sub-Saharan Africa**																
Benin	4·6 (2·0-7·8)	8·9 (6·4-12·0)	9·3 (6·8-12·5)	13·3 (9·6-17·3)	11·5 (2·1-18·5)	20·5 (13·0-27·2)	21·1 (13·2-27·8)	28·9 (15·7-41·1)	92·9 (89·2-96·1)	95·4 (92·4-97·7)	95·3 (92·2-97·9)	96·1 (90·5-100·0)	55·7 (50·6-60·0)	66·3 (57·3-72·8)	68·4 (58·9-75·4)	87·2 (69·1-97·3)
Burkina Faso	1·4 (0·0-2·9)	7·8 (5·6-10·3)	8·1 (5·9-10·6)	12·7 (9·7-15·8)	7·4 (0·0-13·2)	15·4 (4·0-22·8)	15·6 (4·3-23·1)	21·9 (2·3-36·2)	66·7 (61·2-71·5)	80·8 (77·8-83·6)	81·5 (78·1-84·5)	87·6 (80·3-93·9)	46·5 (41·0-51·8)	83·8 (78·5-88·2)	81·7 (74·2-87·6)	83·3 (57·4-95·8)
Cameroon	5·1 (2·7-8·3)	12·5 (9·4-16·6)	13·1 (10·0-17·3)	19·6 (14·7-25·1)	14·3 (6·9-25·0)	21·2 (11·4-34·9)	21·7 (11·5-35·7)	30·4 (11·7-48·5)	88·2 (83·4-91·8)	90·7 (87·8-93·4)	90·9 (87·9-93·7)	91·8 (85·7-97·0)	42·0 (36·3-47·3)	79·5 (71·9-85·5)	81·4 (73·3-87·7)	93·1 (82·2-98·4)
Cape Verde	25·9 (23·1-29·1)	35·4 (31·8-39·2)	35·9 (32·2-39·8)	42·8 (38·3-47·6)	29·7 (22·2-37·0)	44·4 (37·1-50·2)	45·1 (37·4-50·9)	59·6 (40·9-74·7)	89·6 (85·3-93·3)	90·4 (86·0-94·1)	90·4 (86·0-94·1)	91·6 (83·6-98·0)	67·5 (60·3-73·6)	95·4 (92·4-97·4)	96·0 (93·0-97·8)	99·8 (98·7-100·0)
Chad	0·8 (0·0-3·3)	4·7 (2·0-7·5)	5·1 (2·4-7·9)	9·1 (5·4-12·5)	7·0 (0·0-16·0)	13·2 (4·1-22·4)	13·7 (4·6-23·0)	21·4 (3·2-36·1)	87·0 (83·6-90·1)	88·0 (83·6-92·0)	88·0 (83·5-92·1)	88·7 (80·5-96·0)	0·0 (0·0-0·0)	17·2 (4·2-29·2)	19·0 (4·5-33·1)	60·8 (32·3-83·5)
Cote d'Ivoire	9·6 (6·4-13·0)	12·4 (8·6-16·1)	12·7 (8·9-16·5)	16·9 (12·4-20·9)	8·5 (1·3-18·4)	16·0 (9·0-27·2)	16·4 (9·2-27·6)	23·4 (10·0-36·8)	85·9 (82·3-89·2)	78·9 (74·1-83·2)	79·4 (74·3-83·9)	79·9 (68·9-89·0)	51·8 (45·2-57·6)	86·8 (80·7-91·3)	87·3 (80·6-91·9)	97·0 (91·5-99·5)
The Gambia	12·4 (8·8-15·5)	16·6 (11·7-20·3)	16·8 (11·8-20·6)	20·6 (14·4-25·3)	19·0 (11·3-26·8)	27·1 (17·8-36·5)	27·7 (18·1-37·1)	36·3 (20·1-48·8)	71·3 (65·4-76·9)	77·1 (72·3-81·4)	77·5 (72·5-82·2)	82·2 (73·9-89·9)	68·4 (63·3-72·7)	80·0 (73·6-85·4)	80·2 (72·4-86·2)	87·7 (71·9-96·3)
Ghana	13·7 (11·7-15·8)	21·3 (18·1-24·7)	21·9 (18·7-25·3)	29·5 (25·7-33·4)	17·9 (10·2-25·5)	25·1 (16·1-36·0)	25·7 (16·5-36·9)	32·9 (16·0-48·9)	96·6 (94·5-98·6)	100·0 (99·6-100·0)	100·0 (99·5-100·0)	100·0 (100·0-100·0)	70·7 (66·8-74·1)	94·1 (90·4-96·5)	94·6 (90·8-97·0)	99·0 (96·7-99·9)
Guinea	3·8 (0·9-7·6)	11·2 (8·4-14·9)	11·8 (9·0-15·4)	19·3 (16·2-23·1)	9·2 (0·0-17·3)	15·3 (7·5-23·2)	15·8 (8·0-23·7)	22·9 (8·2-36·3)	82·5 (77·3-87·5)	83·2 (77·7-88·1)	83·2 (77·8-88·2)	84·0 (74·1-92·4)	38·6 (29·7-47·1)	61·1 (49·1-71·2)	62·0 (48·8-73·3)	66·8 (29·3-90·6)
Guinea-Bissau	3·7 (0·6-7·5)	10·4 (6·5-15·0)	10·7 (6·8-15·4)	16·2 (11·3-21·7)	3·4 (0·0-12·3)	9·5 (2·4-20·7)	9·8 (2·8-20·7)	17·5 (3·8-31·4)	86·4 (81·5-90·7)	86·9 (82·5-91·0)	86·9 (82·5-91·0)	87·5 (79·3-94·3)	52·1 (43·8-59·7)	82·7 (76·8-88·1)	82·6 (75·5-88·6)	95·2 (86·6-99·3)
Liberia	0·1 (0·0-1·4)	6·5 (2·1-9·4)	7·0 (2·5-10·0)	14·8 (8·1-19·1)	16·9 (2·8-25·5)	26·0 (15·5-35·4)	26·6 (16·0-35·9)	34·7 (20·6-46·4)	85·7 (81·4-89·6)	90·4 (87·3-93·2)	90·4 (87·2-93·5)	92·0 (85·6-97·3)	12·6 (4·2-20·3)	60·1 (48·6-68·9)	61·4 (48·9-71·0)	66·2 (36·9-84·5)
Mali	0·1 (0·0-1·0)	5·5 (2·4-8·9)	6·0 (2·9-9·5)	12·9 (8·5-18·0)	12·0 (0·0-21·6)	22·4 (6·0-32·7)	23·0 (6·4-33·7)	32·7 (9·6-49·4)	81·4 (77·6-85·0)	86·7 (83·1-89·9)	86·7 (82·9-90·2)	88·1 (81·1-94·3)	19·6 (13·8-25·1)	52·5 (43·1-60·8)	55·0 (44·3-64·6)	67·9 (36·7-88·4)
Mauritania	4·9 (2·4-7·7)	15·1 (10·1-19·6)	15·6 (10·5-20·2)	23·6 (16·6-29·2)	23·9 (12·7-34·2)	35·2 (20·7-48·0)	35·8 (21·1-48·8)	46·9 (23·8-65·6)	75·2 (70·7-79·4)	78·2 (72·5-83·6)	78·5 (72·4-83·9)	79·8 (68·5-88·8)	38·6 (33·1-44·4)	66·7 (58·4-73·3)	67·0 (57·0-74·9)	83·9 (64·0-94·7)
Niger	0·2 (0·0-1·8)	5·6 (2·4-9·4)	5·9 (2·6-9·8)	10·6 (6·5-15·3)	8·5 (0·0-18·6)	15·7 (0·0-25·4)	15·8 (0·0-25·3)	20·3 (0·0-36·6)	98·0 (95·0-100·0)	94·9 (92·2-97·3)	94·8 (91·9-97·5)	93·3 (86·2-98·7)	6·3 (0·1-12·8)	72·9 (64·8-79·5)	72·6 (63·3-80·3)	89·2 (73·6-97·2)
Nigeria	4·4 (1·7-7·3)	10·4 (7·5-13·5)	11·1 (8·2-14·3)	18·4 (13·7-23·3)	24·3 (12·7-33·6)	37·1 (24·2-47·8)	37·4 (25·0-48·2)	48·2 (24·9-68·2)	95·0 (92·3-97·4)	99·4 (98·0-100·0)	99·4 (97·8-100·0)	100·0 (99·8-100·0)	0·0 (0·0-0·0)	26·8 (16·1-36·4)	24·8 (11·9-36·3)	44·1 (19·3-65·8)
Sao Tome and Principe	14·9 (11·3-18·6)	27·0 (22·3-32·3)	27·7 (22·9-33·1)	37·8 (31·0-45·3)	35·7 (23·0-47·8)	44·2 (26·9-56·6)	44·4 (27·2-57·0)	53·7 (25·7-73·8)	98·9 (96·4-100·0)	99·7 (98·2-100·0)	99·6 (98·1-100·0)	99·7 (97·4-100·0)	73·2 (68·6-77·2)	90·1 (85·9-93·2)	90·1 (85·3-93·4)	96·5 (87·3-99·6)
Senegal	5·9 (3·2-8·6)	13·8 (10·8-16·4)	14·0 (11·0-16·7)	17·8 (14·0-21·1)	14·2 (4·7-20·5)	19·1 (8·0-26·2)	19·7 (8·8-26·9)	27·0 (10·0-39·4)	84·2 (80·2-88·1)	92·5 (89·3-95·2)	92·5 (89·2-95·5)	96·8 (91·5-100·0)	44·4 (38·4-50·1)	72·6 (64·4-79·2)	73·3 (63·5-81·1)	82·9 (59·7-94·6)

Location	Indicator 5.2.1: Age-standardised prevalence of women aged 15 years and older who experienced physical or sexual violence by an intimate partner in the last 12 months (%)				Indicator 6.1.1: Risk-weighted prevalence of populations using unsafe or unimproved water sources, as measured by the summary exposure value (SEV) for unsafe water (%)				Indicator 6.2.1a: Risk-weighted prevalence of populations using unsafe or unimproved sanitation, as measured by the summary exposure value (SEV) for unsafe sanitation (%)				Indicator 6.2.1b: Risk-weighted prevalence of populations without access to a handwashing facility, as measured by the summary exposure value (SEV) for unsafe hygiene (%)			
	**2000**	**2015**	**2016**	**2030**	**2000**	**2015**	**2016**	**2030**	**2000**	**2015**	**2016**	**2030**	**2000**	**2015**	**2016**	**2030**
Bangladesh	17·6 (13·6-22·1)	27·5 (23·6-31·6)	28·2 (24·3-32·3)	38·0 (33·6-42·0)	9·3 (1·2-15·5)	12·4 (5·8-18·7)	12·6 (6·2-19·0)	16·0 (10·1-23·0)	14·4 (8·4-22·5)	33·4 (16·0-50·4)	34·7 (16·8-52·4)	56·7 (31·9-78·0)	23·1 (21·1-25·2)	26·8 (24·7-28·9)	27·1 (24·9-29·2)	30·9 (28·4-33·2)
Bhutan	34·8 (30·0-39·8)	45·9 (41·4-50·2)	46·6 (41·9-50·7)	55·1 (49·3-59·8)	61·6 (47·7-92·3)	79·8 (69·5-99·4)	80·3 (69·9-99·3)	86·0 (66·2-99·0)	22·6 (14·0-33·9)	58·6 (46·5-71·4)	60·6 (48·6-73·5)	85·4 (78·0-93·2)	77·0 (75·1-79·0)	82·6 (80·8-84·1)	82·6 (80·9-84·2)	83·9 (79·5-91·3)
India	33·6 (31·4-36·0)	40·3 (38·0-42·7)	40·8 (38·5-43·2)	47·9 (44·9-50·6)	23·0 (20·8-26·4)	31·4 (28·5-34·7)	32·3 (29·5-35·6)	44·7 (42·0-47·9)	21·6 (19·9-23·5)	48·8 (46·7-50·9)	50·8 (48·6-53·0)	76·2 (74·1-78·6)	39·2 (38·5-39·9)	50·4 (49·8-51·1)	51·4 (50·8-52·1)	60·5 (59·9-61·2)
Nepal	34·9 (31·5-38·6)	42·7 (39·7-46·0)	42·9 (39·9-46·1)	48·6 (45·5-52·0)	10·9 (6·1-16·7)	24·3 (16·5-31·3)	25·5 (17·6-32·6)	41·6 (30·2-50·3)	18·6 (13·7-24·5)	46·1 (38·2-54·0)	47·9 (39·9-55·9)	73·0 (65·8-80·1)	38·1 (35·5-40·6)	54·9 (52·4-57·2)	56·9 (54·5-59·3)	68·2 (64·3-73·5)
Pakistan	14·8 (10·8-19·5)	22·7 (19·0-26·2)	23·4 (19·7-26·9)	32·0 (28·2-35·4)	18·8 (11·4-26·5)	26·8 (16·5-37·3)	27·1 (16·6-38·0)	32·5 (14·8-47·2)	41·5 (32·8-50·6)	62·9 (53·5-71·4)	64·6 (55·3-73·0)	83·6 (76·6-89·7)	49·1 (46·5-51·8)	54·9 (52·2-57·5)	55·1 (52·4-57·7)	58·1 (55·2-61·0)
**Southern Sub-Saharan Africa**																
Botswana	43·3 (37·7-47·6)	49·1 (44·6-52·8)	49·7 (45·3-53·4)	57·4 (52·6-61·4)	28·9 (17·4-47·3)	41·6 (25·8-60·1)	42·5 (26·4-60·8)	53·6 (12·2-81·7)	16·1 (10·4-24·3)	42·4 (13·9-75·1)	43·9 (12·2-78·8)	70·2 (24·6-99·7)	34·7 (14·0-61·0)	40·3 (16·9-65·4)	41·0 (17·4-66·1)	48·1 (20·4-73·3)
Lesotho	19·5 (15·1-23·9)	22·2 (17·5-27·6)	22·4 (17·8-27·7)	24·5 (18·7-30·4)	7·5 (2·8-13·4)	18·1 (10·8-26·1)	18·9 (11·6-27·2)	30·8 (20·9-41·0)	3·7 (0·0-17·6)	12·9 (0·0-36·0)	13·2 (0·0-37·5)	20·1 (0·4-62·2)	0·3 (0·0-3·0)	0·5 (0·0-3·5)	0·6 (0·0-3·8)	1·0 (0·0-4·4)
Namibia	28·1 (22·2-32·8)	40·0 (35·3-44·1)	40·5 (35·8-44·6)	47·8 (43·5-51·7)	21·4 (9·1-36·6)	38·2 (21·5-54·8)	39·2 (22·3-55·8)	52·4 (8·3-80·7)	33·4 (18·7-50·8)	39·7 (22·3-59·6)	40·8 (22·0-62·1)	52·4 (12·3-86·7)	34·3 (14·9-57·4)	45·0 (27·3-63·3)	45·8 (28·0-64·2)	56·5 (38·3-73·8)
South Africa	53·5 (50·3-56·4)	57·8 (54·9-60·5)	58·0 (55·1-60·6)	60·1 (57·3-62·6)	46·6 (37·3-55·1)	57·3 (47·6-65·3)	57·9 (48·1-65·9)	64·9 (55·4-72·4)	52·6 (48·5-56·5)	59·4 (56·6-62·2)	60·2 (57·2-63·3)	71·2 (65·8-76·0)	49·8 (41·3-58·1)	49·2 (40·1-58·5)	49·2 (40·1-58·5)	49·1 (39·9-59·1)
Swaziland	32·1 (26·8-37·0)	31·3 (25·6-36·4)	31·8 (26·3-36·8)	37·8 (30·9-44·4)	12·7 (3·8-23·2)	27·8 (13·8-42·4)	29·1 (14·7-43·6)	45·3 (27·6-60·4)	14·8 (5·5-29·0)	23·1 (9·6-41·4)	24·5 (10·1-43·7)	28·0 (0·0-65·1)	25·5 (10·6-44·5)	36·0 (19·9-54·6)	37·0 (20·6-55·9)	46·5 (29·6-65·0)
Zimbabwe	33·4 (28·4-37·6)	30·1 (25·3-34·2)	30·6 (25·8-34·7)	37·6 (31·9-42·7)	22·1 (14·3-32·3)	18·3 (11·8-27·3)	18·6 (12·0-27·4)	23·8 (0·0-58·4)	36·4 (20·7-55·2)	36·8 (20·0-56·3)	37·3 (19·8-57·9)	44·6 (17·8-76·5)	24·6 (10·8-43·6)	11·5 (4·7-21·1)	11·2 (4·4-20·9)	6·6 (1·1-15·9)
**Western Sub-Saharan Africa**																
Benin	28·6 (23·7-33·6)	30·4 (25·6-35·3)	30·5 (25·8-35·4)	32·7 (28·1-37·4)	13·6 (3·8-26·0)	11·3 (2·7-23·9)	11·2 (2·5-23·8)	10·1 (0·0-26·2)	2·2 (0·0-8·4)	9·8 (0·0-25·5)	10·3 (0·1-27·4)	20·1 (2·5-59·0)	0·8 (0·0-4·1)	3·3 (0·0-9·3)	3·4 (0·0-9·6)	6·0 (0·0-17·2)
Burkina Faso	39·1 (34·8-43·6)	43·0 (38·8-47·2)	43·3 (39·0-47·5)	46·9 (42·4-51·3)	0·8 (0·0-4·6)	6·8 (0·0-15·8)	7·3 (0·3-16·3)	14·1 (6·7-24·9)	1·0 (0·0-5·4)	10·6 (0·0-24·4)	11·2 (0·0-26·0)	24·4 (0·0-55·9)	4·3 (0·0-11·1)	7·8 (1·4-17·0)	8·1 (1·5-17·8)	12·6 (2·4-26·3)
Cameroon	28·0 (23·2-32·1)	28·6 (23·7-32·7)	28·9 (24·1-33·0)	32·9 (28·1-37·1)	4·9 (0·0-9·6)	12·7 (6·4-19·9)	13·2 (6·8-20·4)	20·1 (11·7-28·3)	7·4 (2·2-14·7)	21·9 (3·3-40·2)	22·7 (3·5-42·1)	37·9 (9·3-69·9)	7·8 (0·1-21·1)	9·2 (0·8-23·7)	9·4 (0·8-24·1)	11·4 (1·3-27·1)
Cape Verde	31·6 (25·9-36·6)	39·3 (34·4-43·6)	40·0 (35·2-44·3)	47·7 (41·9-52·9)	19·7 (4·6-36·4)	52·5 (28·1-72·5)	53·5 (28·6-73·6)	66·4 (37·3-86·6)	25·0 (2·1-63·4)	55·3 (25·3-80·7)	56·6 (23·1-83·1)	80·1 (33·9-99·4)	14·7 (3·7-31·5)	34·6 (14·4-60·7)	35·4 (14·9-61·4)	61·1 (32·7-85·8)
Chad	25·1 (21·0-29·4)	28·6 (24·6-32·9)	28·7 (24·7-33·0)	29·9 (24·2-35·2)	0·6 (0·0-3·8)	2·4 (0·0-9·5)	2·6 (0·0-9·9)	5·9 (0·0-15·3)	0·8 (0·0-5·3)	1·5 (0·0-8·1)	1·6 (0·0-8·6)	3·0 (0·0-17·6)	9·8 (2·0-23·1)	5·2 (0·7-11·9)	4·9 (0·4-11·6)	2·0 (0·0-7·4)
Cote d'Ivoire	35·5 (30·5-40·2)	40·4 (36·2-44·4)	40·6 (36·5-44·6)	44·1 (39·6-48·0)	20·8 (7·6-35·5)	25·8 (9·1-46·5)	26·6 (9·4-47·9)	36·6 (10·6-65·7)	16·3 (8·4-26·7)	30·1 (10·3-60·4)	31·2 (10·0-63·7)	47·7 (10·2-93·9)	12·0 (2·7-27·4)	12·9 (4·0-25·2)	13·4 (4·1-26·0)	20·6 (6·6-38·8)
The Gambia	46·5 (41·8-50·7)	50·5 (46·0-54·5)	50·6 (46·0-54·6)	52·1 (47·3-56·6)	10·9 (0·0-21·8)	25·3 (9·7-42·0)	26·1 (10·3-42·9)	36·9 (18·3-55·5)	7·4 (3·3-14·4)	27·2 (10·8-45·6)	27·7 (10·9-46·8)	53·3 (31·4-78·2)	1·6 (0·0-7·2)	4·5 (0·0-11·4)	4·7 (0·1-11·8)	8·9 (2·1-18·4)
Ghana	40·4 (36·0-45·1)	43·6 (39·6-47·7)	44·1 (40·0-48·1)	49·1 (44·3-53·7)	14·8 (7·1-21·9)	11·2 (4·1-18·4)	10·9 (3·9-18·2)	7·1 (0·0-15·6)	12·8 (2·1-27·9)	29·8 (10·6-50·5)	31·1 (12·0-52·3)	52·9 (40·8-76·6)	10·6 (3·3-21·2)	3·6 (0·7-8·1)	3·4 (0·5-7·9)	1·1 (0·0-4·3)
Guinea	25·8 (21·5-30·1)	26·4 (22·1-30·7)	26·6 (22·3-30·9)	27·9 (23·5-32·5)	6·7 (1·3-13·2)	13·4 (5·1-27·0)	14·0 (5·4-27·9)	22·8 (8·9-39·8)	6·7 (0·0-18·9)	25·2 (8·7-45·7)	26·4 (9·2-48·1)	50·9 (26·5-81·0)	3·6 (0·0-12·8)	5·1 (0·2-13·0)	5·3 (0·3-13·5)	7·0 (1·2-15·3)
Guinea-Bissau	15·9 (11·4-20·4)	18·4 (14·0-22·8)	18·8 (14·4-23·2)	22·3 (17·6-27·0)	1·3 (0·0-10·2)	4·0 (0·0-17·4)	4·3 (0·0-17·8)	14·4 (0·0-53·0)	6·0 (0·0-15·8)	6·6 (0·4-14·1)	7·0 (0·7-14·6)	8·4 (0·0-18·7)	6·0 (0·0-17·3)	5·6 (0·0-16·5)	5·7 (0·0-16·8)	5·7 (0·0-17·3)
Liberia	8·1 (3·2-12·7)	17·4 (12·7-21·7)	17·7 (12·9-22·2)	23·1 (15·4-29·9)	5·2 (1·2-9·9)	6·0 (1·5-12·0)	6·1 (1·5-12·0)	7·2 (1·6-14·2)	9·3 (0·7-27·2)	24·3 (10·8-42·9)	25·3 (11·1-45·5)	42·5 (18·0-77·3)	0·0 (0·0-0·0)	0·0 (0·0-0·0)	0·0 (0·0-0·0)	0·0 (0·0-0·0)
Mali	15·1 (10·5-19·3)	19·9 (15·7-23·7)	20·2 (16·0-24·0)	24·2 (19·6-28·4)	3·1 (0·0-9·0)	9·0 (4·2-15·7)	9·2 (4·3-15·8)	11·3 (5·8-18·6)	2·5 (0·0-8·3)	11·5 (0·0-23·9)	12·0 (0·0-25·0)	21·7 (0·2-45·3)	6·2 (0·7-13·9)	6·9 (1·4-16·2)	6·9 (1·4-16·6)	7·7 (1·6-19·8)
Mauritania	38·4 (33·6-43·4)	43·0 (38·6-47·5)	43·4 (39·1-47·8)	48·6 (44·6-52·5)	16·0 (6·8-27·6)	27·9 (11·2-50·5)	28·9 (11·6-52·2)	43·1 (17·4-71·9)	1·2 (0·0-8·2)	19·1 (3·4-42·3)	20·2 (3·7-45·5)	45·5 (18·4-87·5)	20·3 (7·2-40·0)	28·5 (14·3-46·5)	29·3 (14·9-47·7)	39·8 (22·3-57·8)
Niger	14·9 (10·3-19·9)	15·5 (11·2-20·0)	15·5 (11·2-20·0)	16·1 (11·6-20·8)	2·9 (0·0-9·4)	10·4 (0·2-24·9)	12·0 (1·7-26·7)	33·3 (18·4-49·3)	0·5 (0·0-4·3)	3·2 (0·0-12·5)	3·5 (0·0-13·4)	10·0 (0·3-31·2)	9·6 (1·4-23·7)	13·8 (4·8-26·5)	14·3 (5·3-27·1)	17·8 (7·0-32·5)
Nigeria	51·3 (47·6-54·6)	58·2 (55·1-61·1)	58·7 (55·5-61·6)	64·6 (61·1-67·9)	3·7 (0·0-8·3)	7·4 (1·7-15·8)	7·7 (2·0-16·3)	12·2 (3·4-22·3)	18·8 (7·2-35·5)	40·3 (25·6-57·3)	43·7 (28·9-61·2)	54·6 (0·0-89·3)	8·7 (1·4-19·7)	8·5 (3·3-15·7)	8·6 (3·4-15·9)	8·1 (2·9-16·2)
Sao Tome and Principe	21·7 (17·0-26·7)	25·8 (21·7-30·3)	26·0 (21·8-30·6)	28·6 (23·5-33·9)	18·4 (4·2-37·8)	28·4 (12·7-44·0)	28·8 (12·8-44·8)	33·4 (11·9-55·7)	14·5 (9·5-21·0)	59·1 (39·1-75·5)	63·2 (42·6-79·3)	95·3 (86·5-99·3)	25·2 (9·6-46·7)	33·0 (17·7-51·9)	33·5 (18·2-52·7)	40·8 (27·0-59·7)
Senegal	39·6 (34·5-44·7)	39·7 (34·7-44·5)	39·7 (34·8-44·6)	40·2 (35·7-45·0)	22·0 (9·4-35·7)	37·6 (17·3-59·2)	38·4 (17·5-60·6)	49·8 (19·2-76·1)	28·8 (13·3-51·1)	36·2 (14·4-62·4)	35·4 (12·7-63·0)	34·0 (9·0-73·7)	26·8 (12·2-43·8)	24·2 (12·8-38·2)	24·1 (12·5-38·5)	23·1 (10·4-40·6)

Location	Indicator 7.1.2: Risk-weighted prevalence of household air pollution, as measured by the summary exposure value (SEV) for household air pollution (%)				Indicator 8.8.1: Age-standardised all-cause disability-adjusted life year (DALY) rates attributable to occupational risks (per 100,000 population)				Indicator 11.6.2: Population-weighted mean levels of fine particulate matter smaller than 2.5 microns in diameter (PM2.5)				Indicator 16.1.1: Age-standardised death rate due to interpersonal violence (per 100,000 population)			
	**2000**	**2015**	**2016**	**2030**	**2000**	**2015**	**2016**	**2030**	**2000**	**2015**	**2016**	**2030**	**2000**	**2015**	**2016**	**2030**
Bangladesh	20·9 (13·0-29·6)	37·5 (29·3-46·4)	39·0 (30·9-47·8)	58·8 (52·6-65·6)	7·7 (1·0-14·0)	32·1 (25·9-38·5)	33·0 (26·7-39·4)	45·3 (38·1-52·5)	16·4 (15·2-17·8)	2·0 (0·7-3·4)	1·7 (0·4-3·3)	0·1 (0·0-1·1)	53·9 (42·6-58·7)	62·1 (52·1-68·0)	62·6 (52·4-68·6)	71·0 (57·8-82·0)
Bhutan	46·6 (37·4-55·7)	79·9 (72·3-86·2)	81·2 (73·8-87·3)	93·6 (89·1-96·6)	4·6 (0·0-11·1)	24·5 (18·1-31·4)	25·3 (18·9-32·2)	37·2 (30·2-44·5)	32·2 (30·1-34·4)	21·8 (19·7-24·0)	21·8 (19·7-24·0)	21·3 (18·9-23·7)	68·6 (61·2-78·2)	79·4 (70·1-86·9)	79·8 (70·6-87·6)	84·6 (68·3-98·7)
India	35·6 (31·8-39·7)	60·5 (56·6-64·2)	62·5 (58·8-66·2)	84·1 (82·5-85·8)	17·9 (13·0-22·9)	34·6 (30·2-39·1)	34·9 (30·6-39·3)	39·2 (35·0-43·6)	18·5 (17·6-19·7)	11·5 (10·6-12·7)	11·4 (10·5-12·7)	10·0 (9·0-11·3)	47·6 (42·7-53·9)	52·4 (46·4-56·6)	52·7 (46·7-56·8)	56·1 (49·4-62·0)
Nepal	27·0 (19·3-34·8)	42·5 (35·1-50·7)	44·7 (37·5-52·7)	70·8 (66·8-75·9)	0·2 (0·0-2·1)	9·6 (3·2-16·4)	10·2 (3·9-17·0)	19·4 (12·8-25·9)	21·6 (20·1-23·2)	10·5 (8·2-12·6)	10·3 (7·6-12·8)	6·6 (3·8-9·5)	62·6 (56·4-75·2)	65·3 (57·3-80·6)	65·5 (57·5-81·0)	68·6 (55·2-86·4)
Pakistan	35·8 (29·2-43·1)	53·7 (47·6-60·1)	55·5 (49·6-61·7)	76·2 (72·8-80·3)	33·1 (26·6-40·1)	41·0 (34·2-47·7)	41·4 (34·6-48·1)	47·0 (39·8-54·3)	14·3 (13·2-15·6)	11·8 (10·6-13·1)	11·5 (10·3-12·9)	12·5 (11·1-14·5)	44·2 (38·8-51·4)	45·9 (38·6-53·9)	46·2 (38·8-54·4)	49·2 (36·0-61·3)
**Southern Sub-Saharan Africa**																
Botswana	58·9 (50·3-66·4)	80·9 (70·6-89·2)	82·2 (72·0-90·3)	93·7 (86·7-98·1)	27·5 (0·0-51·3)	48·8 (35·3-61·8)	50·1 (36·9-63·0)	68·7 (58·1-80·1)	52·7 (49·6-55·5)	52·0 (49·0-54·7)	51·6 (48·3-54·2)	53·6 (50·1-56·8)	28·3 (6·9-55·0)	26·8 (12·3-54·2)	27·0 (12·9-54·2)	36·6 (6·9-75·3)
Lesotho	41·9 (34·5-49·4)	56·7 (48·8-63·9)	57·9 (49·9-65·0)	72·4 (64·5-78·7)	23·8 (10·5-37·6)	35·5 (21·8-49·1)	36·3 (22·5-50·0)	47·7 (33·3-62·5)	45·3 (36·3-51·7)	47·3 (37·5-53·8)	46·7 (37·2-53·3)	43·0 (30·5-53·0)	2·0 (0·0-15·8)	1·5 (0·0-14·4)	1·6 (0·0-15·1)	2·9 (0·0-18·7)
Namibia	47·3 (39·3-55·0)	64·2 (55·0-72·8)	65·5 (56·2-73·9)	79·7 (71·3-86·6)	51·9 (40·0-64·2)	74·4 (65·9-83·8)	75·4 (66·9-84·8)	89·3 (80·9-99·1)	46·2 (40·3-50·7)	48·3 (40·4-53·7)	47·9 (39·4-53·9)	50·9 (41·2-57·8)	11·4 (2·7-20·8)	15·8 (6·4-26·5)	16·1 (6·5-27·2)	21·1 (4·8-39·8)
South Africa	76·0 (73·3-78·9)	90·7 (88·8-92·4)	91·4 (89·5-93·0)	97·0 (96·1-97·7)	36·2 (30·6-41·4)	59·2 (54·3-64·1)	59·5 (54·6-64·5)	65·9 (59·7-71·5)	35·6 (34·5-37·0)	37·0 (35·9-38·4)	36·7 (35·3-38·1)	35·8 (32·2-39·8)	0·0 (0·0-0·0)	0·8 (0·0-4·0)	0·7 (0·0-3·8)	7·5 (0·0-15·0)
Swaziland	43·2 (35·6-50·7)	71·7 (61·4-80·7)	73·2 (62·9-82·3)	89·2 (80·8-95·3)	31·4 (10·0-47·5)	49·7 (34·8-65·2)	51·7 (36·8-67·3)	79·6 (65·6-95·2)	45·7 (39·6-51·2)	50·0 (44·1-55·0)	50·2 (44·7-55·3)	52·5 (44·9-58·8)	10·7 (0·0-22·4)	14·3 (0·2-25·1)	14·5 (0·4-25·5)	19·6 (0·0-41·5)
Zimbabwe	46·6 (39·2-54·3)	50·5 (40·6-59·5)	51·7 (41·9-60·8)	67·0 (57·3-74·8)	33·2 (21·9-44·0)	38·9 (31·2-46·8)	40·4 (32·7-48·2)	61·6 (54·1-69·1)	48·8 (44·6-52·1)	49·8 (46·2-52·8)	49·6 (46·0-52·5)	48·7 (42·9-54·4)	29·0 (19·1-38·7)	27·6 (19·3-34·5)	27·3 (19·4-34·3)	26·1 (10·7-40·9)
**Western Sub-Saharan Africa**																
Benin	8·2 (0·0-18·0)	18·3 (7·8-30·2)	19·3 (8·6-31·2)	32·9 (21·5-45·3)	31·2 (24·2-37·6)	35·9 (29·2-42·5)	36·5 (29·9-43·2)	45·9 (38·5-52·9)	20·1 (15·5-23·4)	5·5 (2·2-8·6)	3·5 (0·0-6·4)	8·3 (3·4-12·8)	59·0 (47·7-66·4)	56·3 (48·6-61·9)	56·3 (48·5-62·1)	55·1 (44·4-65·0)
Burkina Faso	1·5 (0·0-10·0)	12·0 (0·3-23·4)	13·1 (1·6-24·6)	31·0 (20·9-41·2)	53·2 (45·6-60·9)	56·4 (48·1-64·3)	56·9 (48·5-64·8)	63·1 (54·2-71·3)	15·7 (13·4-17·7)	1·0 (0·0-3·3)	0·1 (0·0-1·3)	0·3 (0·0-2·1)	84·5 (62·9-92·2)	84·8 (64·2-92·3)	85·2 (64·5-92·8)	87·9 (67·0-100·0)
Cameroon	25·0 (16·9-34·0)	38·1 (23·9-55·4)	39·2 (24·6-57·0)	53·9 (32·7-78·2)	41·2 (33·7-48·3)	45·5 (37·9-52·8)	46·0 (38·4-53·3)	53·2 (45·2-61·1)	2·2 (0·7-3·8)	0·0 (0·0-0·0)	0·0 (0·0-0·0)	0·0 (0·0-0·0)	51·9 (45·3-61·3)	53·1 (45·1-62·8)	53·1 (45·0-63·1)	54·1 (40·5-69·2)
Cape Verde	61·4 (49·5-71·9)	78·6 (55·2-94·0)	79·7 (55·6-95·0)	91·3 (64·6-99·8)	83·4 (74·6-91·6)	89·9 (81·6-98·3)	90·7 (82·4-99·1)	99·2 (94·2-100·0)	26·2 (16·5-34·4)	17·0 (7·2-26·4)	16·2 (6·1-26·4)	14·2 (2·5-26·5)	22·9 (14·3-30·1)	20·2 (12·7-26·9)	20·5 (13·0-27·5)	22·1 (8·8-35·9)
Chad	4·1 (0·0-14·5)	10·1 (0·0-21·0)	10·6 (0·1-21·4)	17·1 (7·0-28·2)	35·8 (27·9-43·1)	45·7 (37·7-53·7)	46·3 (38·3-54·3)	54·6 (46·4-62·4)	17·2 (13·7-19·6)	5·0 (1·6-7·4)	3·6 (0·2-6·1)	6·3 (1·8-9·6)	58·7 (46·2-66·6)	56·1 (48·3-62·0)	56·1 (48·3-61·8)	55·9 (45·6-65·6)
Cote d'Ivoire	27·8 (17·3-38·0)	34·8 (20·0-53·5)	36·2 (20·8-55·4)	54·3 (32·3-78·0)	34·3 (26·6-41·9)	40·9 (34·2-48·4)	41·3 (34·6-48·8)	46·4 (39·6-53·7)	40·2 (36·7-42·8)	22·2 (17·7-25·3)	20·7 (15·9-23·9)	22·6 (17·9-25·9)	71·5 (26·0-84·8)	68·9 (28·2-81·5)	69·4 (29·0-82·1)	68·4 (28·2-85·0)
The Gambia	7·9 (0·0-18·3)	14·2 (4·6-24·2)	14·8 (5·1-24·8)	23·2 (12·5-33·2)	51·0 (43·4-58·9)	59·9 (52·4-66·9)	60·5 (53·0-67·5)	68·8 (61·5-75·7)	18·0 (0·0-32·5)	8·0 (0·0-22·6)	7·5 (0·0-21·1)	6·6 (0·0-21·1)	84·4 (39·5-97·2)	86·3 (38·5-99·1)	86·8 (38·7-99·7)	90·3 (39·7-100·0)
Ghana	24·9 (17·2-33·3)	41·1 (32·5-50·4)	42·6 (33·9-52·0)	61·7 (53·2-70·6)	52·3 (44·5-60·1)	52·1 (44·8-59·3)	52·5 (45·1-59·8)	58·4 (50·4-66·2)	36·1 (34·4-37·7)	24·1 (22·1-26·1)	22·8 (20·5-24·9)	24·7 (22·2-26·9)	51·7 (24·6-62·0)	54·0 (25·6-64·7)	54·8 (26·0-66·0)	61·5 (27·5-80·3)
Guinea	4·0 (0·0-13·7)	9·4 (0·2-20·3)	10·0 (0·7-20·8)	17·6 (8·5-28·1)	27·6 (20·3-35·5)	36·5 (29·0-43·7)	37·4 (29·9-44·6)	49·2 (41·8-56·4)	42·1 (38·8-44·6)	29·4 (25·7-32·4)	28·2 (24·4-31·2)	28·4 (23·9-32·0)	60·9 (48·4-68·7)	56·0 (48·4-62·4)	55·9 (48·4-62·3)	54·5 (43·2-64·8)
Guinea-Bissau	4·9 (0·0-14·4)	12·2 (1·7-23·5)	12·9 (2·2-24·2)	22·7 (12·3-33·3)	55·5 (47·6-63·1)	56·5 (48·5-64·1)	56·9 (48·8-64·4)	61·7 (53·9-69·2)	32·0 (19·1-39·0)	21·6 (7·9-29·6)	20·6 (4·4-29·1)	20·6 (4·8-30·2)	36·7 (28·1-43·5)	36·8 (30·1-44·3)	36·9 (30·3-44·2)	38·8 (28·7-49·3)
Liberia	2·0 (0·0-10·8)	9·9 (0·4-19·8)	10·6 (1·2-20·5)	21·2 (12·3-29·9)	46·7 (38·2-55·5)	58·3 (50·5-66·2)	59·1 (51·4-67·0)	71·0 (63·4-79·0)	78·1 (74·3-81·0)	62·5 (57·2-66·1)	61·4 (56·1-65·4)	60·8 (55·4-65·5)	60·2 (53·5-71·3)	60·3 (53·6-71·9)	60·1 (53·3-71·7)	60·3 (47·8-73·7)
Mali	1·6 (0·0-10·7)	5·2 (0·0-15·8)	5·7 (0·0-16·3)	14·2 (4·9-24·4)	38·0 (29·4-47·8)	46·8 (39·3-53·8)	47·6 (40·1-54·7)	59·6 (52·0-66·8)	19·2 (16·1-21·6)	6·2 (2·0-9·1)	4·9 (0·8-8·0)	6·7 (1·6-10·9)	63·9 (51·5-71·3)	62·5 (54·0-70·2)	62·4 (53·7-70·0)	62·6 (48·3-75·3)
Mauritania	33·7 (24·1-43·1)	57·8 (41·6-74·2)	58·8 (41·8-76·2)	73·1 (45·5-93·6)	70·3 (61·5-80·1)	80·5 (71·3-91·0)	80·9 (71·7-91·3)	87·1 (78·3-96·4)	4·8 (0·0-11·8)	0·2 (0·0-2·5)	0·1 (0·0-1·2)	0·6 (0·0-5·2)	65·9 (53·4-77·6)	65·6 (54·9-77·0)	65·4 (54·7-76·8)	64·7 (47·4-82·4)
Niger	1·2 (0·0-9·9)	2·3 (0·0-12·4)	2·4 (0·0-12·7)	5·6 (0·0-17·2)	22·5 (11·9-36·0)	24·9 (17·3-33·6)	25·5 (18·1-33·9)	34·1 (25·9-41·8)	0·0 (0·0-0·0)	0·0 (0·0-0·0)	0·0 (0·0-0·0)	0·0 (0·0-0·0)	63·4 (51·3-71·2)	59·6 (50·3-67·2)	59·5 (50·6-67·1)	58·3 (44·6-70·4)
Nigeria	29·3 (19·4-38·5)	43·7 (31·7-56·3)	45·1 (32·8-58·0)	62·7 (46·2-77·6)	58·9 (49·1-67·9)	68·9 (60·3-77·1)	68·9 (60·3-77·1)	69·4 (60·3-78·3)	12·0 (11·0-13·3)	0·0 (0·0-0·0)	0·0 (0·0-0·0)	4·6 (1·6-8·0)	92·0 (84·5-99·5)	93·7 (85·9-100·0)	93·8 (85·8-100·0)	94·7 (81·8-100·0)
Sao Tome and Principe	21·2 (11·1-32·0)	53·0 (29·5-77·1)	54·8 (29·9-79·8)	77·1 (42·2-98·0)	72·5 (63·2-86·6)	82·9 (74·0-92·1)	83·6 (74·7-92·7)	93·3 (83·5-100·0)	65·3 (48·5-79·9)	66·3 (49·9-81·2)	66·2 (49·7-81·2)	67·3 (48·4-85·0)	45·5 (37·3-60·8)	45·0 (36·0-58·8)	44·8 (35·9-57·3)	45·0 (29·2-60·9)
Senegal	31·6 (22·4-40·6)	39·9 (22·4-60·9)	40·7 (22·0-62·8)	51·8 (17·1-85·8)	63·5 (55·7-70·6)	63·1 (55·8-70·3)	63·2 (55·9-70·5)	65·4 (57·7-73·1)	32·2 (29·9-34·3)	22·0 (19·7-24·0)	21·2 (18·8-23·4)	21·4 (18·9-23·8)	77·3 (70·9-82·7)	75·9 (69·3-82·2)	76·0 (69·5-82·2)	75·7 (64·4-86·1)

Location	Indicator 16.1.2: Death rate due to conflict and terrorism (per 100,000 population)				Indicator 16.1.3: Age-standardised prevalence of physical or sexual violence experienced by populations in the last 12 months (%)				Indicator 16.2.3: Age-standardised prevalence of women and men aged 18-29 years who experienced sexual violence by age 18 (%)				Indicator 17.19.2c: Percentage of well-certified deaths by a vital registration (VR) system among a country's total population (%)			
	2000	2015	2016	2030	2000	2015	2016	2030	2000	2015	2016	2030	2000	2015	2016	2030
Bangladesh	100·0 (100·0-100·0)	100·0 (100·0-100·0)	100·0 (100·0-100·0)	100·0 (100·0-100·0)	27·4 (23·4-31·5)	43·0 (39·6-46·5)	43·8 (40·4-47·4)	54·9 (51·1-59·1)	0·0 (0·0-0·0)	0·0 (0·0-0·0)	0·0 (0·0-0·0)	0·0 (0·0-0·0)	0·0 (0·0-0·0)	0·0 (0·0-0·0)	0·0 (0·0-0·0)	0·0 (0·0-0·0)
Bhutan	100·0 (100·0-100·0)	100·0 (100·0-100·0)	100·0 (100·0-100·0)	100·0 (100·0-100·0)	52·8 (49·3-55·9)	71·9 (69·3-74·0)	72·3 (69·8-74·5)	79·3 (76·4-81·8)	0·6 (0·0-5·5)	0·4 (0·0-5·2)	0·4 (0·0-5·3)	0·8 (0·0-6·4)	0·0 (0·0-0·0)	0·0 (0·0-0·0)	0·0 (0·0-0·0)	0·0 (0·0-0·0)
India	60·0 (55·0-63·2)	100·0 (100·0-100·0)	100·0 (100·0-100·0)	100·0 (100·0-100·0)	46·8 (44·7-49·1)	55·4 (53·3-57·5)	55·8 (53·7-57·9)	62·1 (59·3-64·8)	13·6 (5·2-21·3)	13·7 (4·7-22·0)	13·6 (4·6-22·0)	13·7 (4·5-22·9)	15·8 (12·8-19·6)	22·8 (18·7-27·6)	23·6 (19·1-28·5)	36·7 (24·4-50·3)
Nepal	40·9 (37·4-43·8)	100·0 (100·0-100·0)	100·0 (100·0-100·0)	100·0 (100·0-100·0)	53·0 (50·5-55·7)	64·1 (62·1-66·0)	64·7 (62·7-66·5)	72·4 (70·5-74·2)	0·6 (0·0-5·7)	1·0 (0·0-6·0)	0·9 (0·0-5·9)	1·3 (0·0-6·6)	0·0 (0·0-0·0)	0·0 (0·0-0·0)	0·0 (0·0-0·0)	0·0 (0·0-0·0)
Pakistan	100·0 (100·0-100·0)	43·9 (39·3-47·3)	65·0 (58·1-73·8)	65·0 (58·1-73·8)	19·3 (15·4-23·2)	36·8 (33·7-39·9)	37·9 (34·9-40·9)	52·1 (49·4-54·7)	0·4 (0·0-5·3)	0·4 (0·0-5·3)	0·4 (0·0-5·3)	0·4 (0·0-5·3)	0·0 (0·0-0·0)	0·0 (0·0-0·0)	0·0 (0·0-0·0)	0·0 (0·0-0·0)
**Southern Sub-Saharan Africa**																
Botswana	100·0 (100·0-100·0)	100·0 (100·0-100·0)	100·0 (100·0-100·0)	100·0 (100·0-100·0)	44·3 (38·2-50·8)	52·7 (47·3-58·5)	53·4 (48·0-59·2)	62·6 (57·7-67·9)	61·6 (54·1-69·7)	61·6 (54·3-70·0)	61·6 (54·3-70·0)	61·7 (54·1-70·2)	0·0 (0·0-0·0)	0·0 (0·0-0·0)	0·0 (0·0-0·0)	0·0 (0·0-0·0)
Lesotho	100·0 (100·0-100·0)	100·0 (100·0-100·0)	100·0 (100·0-100·0)	100·0 (100·0-100·0)	8·7 (0·0-17·8)	22·7 (14·3-30·9)	23·4 (15·1-31·6)	33·5 (25·6-40·9)	61·7 (54·0-69·9)	61·7 (54·4-70·1)	61·7 (54·4-70·1)	61·7 (54·3-70·0)	0·0 (0·0-0·0)	0·0 (0·0-0·0)	0·0 (0·0-0·0)	0·0 (0·0-0·0)
Namibia	24·8 (16·7-36·0)	100·0 (100·0-100·0)	100·0 (100·0-100·0)	100·0 (100·0-100·0)	47·6 (45·3-50·0)	61·1 (59·4-63·0)	61·6 (59·9-63·5)	68·6 (66·8-70·4)	61·6 (54·3-69·9)	61·6 (54·3-69·6)	61·6 (54·4-69·7)	61·6 (54·4-69·9)	0·0 (0·0-0·0)	0·0 (0·0-0·0)	0·0 (0·0-0·0)	0·0 (0·0-0·0)
South Africa	100·0 (100·0-100·0)	58·7 (51·6-69·5)	75·8 (69·0-84·3)	76·9 (61·8-86·7)	63·1 (61·9-64·4)	69·2 (68·0-70·4)	69·4 (68·3-70·6)	72·4 (71·2-73·6)	54·8 (49·9-58·8)	53·5 (48·5-57·6)	53·6 (48·5-57·7)	53·0 (47·9-57·2)	50·0 (48·5-51·6)	62·1 (60·0-64·1)	62·8 (60·5-65·0)	72·1 (67·9-76·2)
Swaziland	100·0 (100·0-100·0)	100·0 (100·0-100·0)	100·0 (100·0-100·0)	100·0 (100·0-100·0)	0·0 (0·0-0·5)	6·0 (1·3-10·3)	7·3 (2·7-11·5)	24·1 (19·9-27·9)	42·2 (27·6-56·5)	53·3 (42·5-64·2)	53·3 (42·5-64·1)	55·4 (44·7-65·5)	0·0 (0·0-0·0)	0·0 (0·0-0·0)	0·0 (0·0-0·0)	0·0 (0·0-0·0)
Zimbabwe	65·0 (57·0-78·2)	100·0 (100·0-100·0)	100·0 (100·0-100·0)	100·0 (100·0-100·0)	30·1 (25·8-34·4)	34·2 (30·3-38·2)	35·0 (31·1-39·0)	45·2 (41·4-49·0)	61·6 (53·7-69·9)	61·5 (54·3-69·7)	61·5 (54·3-69·7)	61·5 (54·3-69·9)	32·7 (26·7-39·1)	30·1 (24·4-36·4)	30·7 (25·0-37·0)	37·8 (25·6-52·3)
**Western Sub-Saharan Africa**																
Benin	100·0 (100·0-100·0)	100·0 (100·0-100·0)	100·0 (100·0-100·0)	100·0 (100·0-100·0)	28·7 (23·7-33·8)	36·8 (32·4-41·4)	37·3 (32·9-41·9)	44·2 (40·0-48·7)	10·5 (0·8-20·4)	10·6 (1·0-20·4)	10·6 (1·0-20·4)	10·6 (1·1-20·4)	0·0 (0·0-0·0)	0·0 (0·0-0·0)	0·0 (0·0-0·0)	0·0 (0·0-0·0)
Burkina Faso	100·0 (100·0-100·0)	72·8 (62·3-88·9)	59·0 (49·0-77·0)	59·0 (49·0-77·0)	52·3 (49·5-55·3)	61·9 (59·5-64·4)	62·5 (60·1-65·0)	70·1 (67·9-72·3)	10·6 (0·6-20·7)	10·7 (1·1-20·5)	10·7 (1·1-20·5)	10·8 (1·4-20·5)	0·0 (0·0-0·0)	0·0 (0·0-0·0)	0·0 (0·0-0·0)	0·0 (0·0-0·0)
Cameroon	100·0 (100·0-100·0)	27·3 (24·0-30·2)	42·3 (32·0-62·0)	47·1 (21·6-68·5)	26·9 (22·5-31·8)	34·2 (30·2-38·8)	34·9 (30·9-39·5)	43·8 (40·2-47·9)	10·6 (0·8-20·5)	10·6 (1·2-20·4)	10·6 (1·2-20·4)	10·7 (1·6-20·4)	0·0 (0·0-0·0)	0·0 (0·0-0·0)	0·0 (0·0-0·0)	0·0 (0·0-0·0)
Cape Verde	100·0 (100·0-100·0)	100·0 (100·0-100·0)	100·0 (100·0-100·0)	100·0 (100·0-100·0)	24·3 (18·8-29·5)	41·6 (37·2-45·7)	42·6 (38·3-46·8)	56·1 (52·5-59·6)	10·6 (0·8-20·5)	10·7 (1·5-20·3)	10·6 (1·5-20·3)	10·7 (1·4-20·6)	46·2 (38·2-54·5)	68·7 (62·0-75·2)	69·4 (62·1-75·8)	84·8 (74·9-92·9)
Chad	10·6 (7·5-13·6)	33·5 (24·9-44·9)	72·1 (62·5-87·1)	82·1 (45·3-95·5)	31·1 (27·2-35·2)	41·5 (38·4-45·1)	42·0 (38·8-45·4)	48·1 (43·3-52·6)	10·6 (0·7-20·6)	10·6 (0·7-20·6)	10·6 (0·7-20·6)	10·6 (0·8-20·6)	0·0 (0·0-0·0)	0·0 (0·0-0·0)	0·0 (0·0-0·0)	0·0 (0·0-0·0)
Cote d'Ivoire	50·1 (45·9-53·5)	74·8 (64·5-90·3)	66·2 (56·4-82·7)	33·5 (0·0-99·7)	40·4 (37·5-43·1)	47·5 (45·1-50·1)	47·8 (45·4-50·4)	51·6 (49·2-54·0)	19·0 (6·4-32·4)	19·7 (7·3-32·7)	19·9 (7·5-32·7)	19·8 (7·4-32·7)	0·0 (0·0-0·0)	0·0 (0·0-0·0)	0·0 (0·0-0·0)	0·0 (0·0-0·0)
The Gambia	38·2 (27·2-59·7)	100·0 (100·0-100·0)	100·0 (100·0-100·0)	100·0 (100·0-100·0)	58·3 (56·3-60·4)	66·4 (64·6-68·2)	66·7 (65·0-68·5)	71·4 (69·8-73·1)	10·2 (0·0-20·6)	10·4 (0·7-20·3)	10·4 (0·7-20·3)	10·6 (0·8-20·7)	0·0 (0·0-0·0)	0·0 (0·0-0·0)	0·0 (0·0-0·0)	0·0 (0·0-0·0)
Ghana	71·4 (61·8-86·7)	100·0 (100·0-100·0)	100·0 (100·0-100·0)	100·0 (100·0-100·0)	35·8 (32·2-39·3)	42·7 (39·4-45·9)	43·3 (40·0-46·6)	51·9 (47·7-55·6)	10·7 (1·2-20·4)	10·7 (1·4-20·5)	10·7 (1·4-20·4)	10·7 (1·5-20·4)	10·8 (8·1-14·0)	25·6 (19·4-32·0)	26·1 (20·1-32·7)	38·2 (25·0-53·9)
Guinea	33·8 (23·4-54·0)	76·4 (67·0-90·9)	100·0 (100·0-100·0)	100·0 (100·0-100·0)	47·9 (45·4-50·2)	52·8 (50·4-55·0)	53·2 (50·8-55·5)	59·3 (56·7-61·8)	10·6 (0·8-20·5)	10·7 (1·2-20·5)	10·7 (1·2-20·5)	10·8 (1·4-20·5)	0·0 (0·0-0·0)	0·0 (0·0-0·0)	0·0 (0·0-0·0)	0·0 (0·0-0·0)
Guinea-Bissau	100·0 (100·0-100·0)	100·0 (100·0-100·0)	100·0 (100·0-100·0)	100·0 (100·0-100·0)	10·6 (4·8-16·8)	20·1 (14·8-25·9)	20·9 (15·6-26·7)	31·5 (26·7-36·8)	10·6 (0·7-20·5)	10·6 (1·2-20·4)	10·6 (1·2-20·4)	10·6 (1·3-20·3)	0·0 (0·0-0·0)	0·0 (0·0-0·0)	0·0 (0·0-0·0)	0·0 (0·0-0·0)
Liberia	30·9 (19·4-52·3)	100·0 (100·0-100·0)	66·6 (56·6-84·4)	68·2 (57·8-85·7)	0·2 (0·0-1·9)	13·3 (7·7-18·7)	13·6 (7·9-19·0)	18·5 (9·1-26·3)	10·6 (0·9-20·5)	10·7 (1·4-20·5)	10·7 (1·3-20·5)	10·8 (1·6-20·5)	0·0 (0·0-0·0)	0·0 (0·0-0·0)	0·0 (0·0-0·0)	0·0 (0·0-0·0)
Mali	8·8 (0·0-30·0)	37·8 (31·3-45·4)	45·8 (34·9-66·9)	54·5 (35·4-89·9)	0·2 (0·0-2·2)	15·9 (12·6-19·7)	16·8 (13·5-20·5)	28·3 (25·0-31·8)	10·7 (0·9-20·7)	10·8 (1·2-20·6)	10·8 (1·1-20·6)	10·9 (1·6-20·5)	4·6 (3·3-6·0)	18·3 (13·9-24·0)	18·6 (14·0-24·3)	30·2 (18·8-44·5)
Mauritania	100·0 (100·0-100·0)	100·0 (100·0-100·0)	100·0 (100·0-100·0)	100·0 (100·0-100·0)	40·7 (36·3-45·5)	49·6 (45·7-53·8)	50·2 (46·3-54·4)	58·3 (54·5-61·8)	10·7 (1·3-20·5)	10·7 (1·3-20·5)	10·7 (1·3-20·5)	10·7 (1·3-20·5)	0·0 (0·0-0·0)	0·0 (0·0-0·0)	0·0 (0·0-0·0)	0·0 (0·0-0·0)
Niger	100·0 (100·0-100·0)	38·0 (32·4-45·1)	47·9 (38·2-62·5)	49·0 (37·0-69·8)	13·9 (8·1-19·9)	19·7 (14·3-25·3)	20·1 (14·6-25·6)	25·1 (19·4-30·4)	9·8 (0·0-20·2)	10·2 (0·0-20·5)	10·3 (0·0-20·6)	10·7 (0·5-20·8)	0·0 (0·0-0·0)	0·0 (0·0-0·0)	0·0 (0·0-0·0)	0·0 (0·0-0·0)
Nigeria	41·0 (30·6-60·7)	37·3 (33·6-40·2)	39·6 (28·5-61·5)	42·7 (0·0-82·3)	53·8 (50·6-57·2)	66·3 (63·8-68·9)	67·0 (64·5-69·6)	75·5 (72·6-78·2)	3·2 (0·0-13·6)	1·9 (0·0-11·7)	1·7 (0·0-11·4)	1·7 (0·0-11·4)	1·1 (0·8-1·4)	10·1 (7·5-13·5)	10·4 (7·6-13·7)	34·1 (20·0-50·8)
Sao Tome and Principe	100·0 (100·0-100·0)	100·0 (100·0-100·0)	100·0 (100·0-100·0)	100·0 (100·0-100·0)	6·9 (2·1-12·5)	20·3 (16·2-25·1)	20·7 (16·5-25·6)	25·9 (19·6-32·2)	10·6 (0·0-21·0)	10·7 (1·5-20·3)	10·7 (1·5-20·3)	10·7 (1·4-20·4)	11·2 (8·2-14·6)	37·3 (30·0-45·4)	38·0 (30·6-45·8)	56·5 (42·8-71·9)
Senegal	57·9 (46·7-76·8)	100·0 (100·0-100·0)	100·0 (100·0-100·0)	100·0 (100·0-100·0)	59·6 (57·2-61·7)	62·9 (60·7-64·9)	63·1 (60·9-65·1)	66·0 (63·8-68·0)	10·4 (0·4-20·6)	10·6 (1·1-20·4)	10·6 (1·2-20·4)	10·7 (1·6-20·4)	0·0 (0·0-0·0)	0·0 (0·0-0·0)	0·0 (0·0-0·0)	0·0 (0·0-0·0)

Location	SDG Index: Geometric mean of all health-related SDG indicators (scale of 0 to 100)				MDG Index: Geometric mean of all health-related SDG indicators with corresponding MDG indicators (scale of 0 to 100)				Non-MDG Index: Geometric mean of all health-related indicators without corresponding MDG indicators (scale of 0 to 100)				Indicator 1.5.1: Death rate due to exposure to forces of nature (per 100,000 population)			
	2000	2015	2016	2030	2000	2015	2016	2030	2000	2015	2016	2030	2000	2015	2016	2030
Sierra Leone	10·8 (9·4-12·4)	19·3 (17·2-21·6)	20·0 (17·8-22·2)	26·0 (21·9-30·4)	4·7 (3·7-6·1)	12·5 (8·8-16·0)	13·2 (9·4-16·9)	21·7 (14·1-30·2)	15·6 (13·0-18·2)	23·9 (21·0-26·4)	24·3 (21·5-26·9)	27·3 (22·9-30·6)	54·4 (45·7-64·8)	41·9 (34·7-51·8)	45·0 (37·9-54·5)	43·9 (37·0-52·4)
Togo	19·3 (16·7-21·7)	23·8 (21·1-26·2)	24·1 (21·5-26·6)	29·3 (24·7-34·3)	11·8 (9·7-13·9)	18·4 (14·2-22·9)	19·0 (14·6-23·6)	26·8 (15·5-38·5)	23·8 (20·0-26·9)	27·4 (24·1-30·2)	27·5 (24·0-30·4)	30·9 (27·0-34·4)	100·0 (100·0-100·0)	60·6 (52·9-72·4)	65·8 (58·0-77·0)	61·1 (53·1-73·5)
**Eastern Sub-Saharan Africa**																
Burundi	8·0 (6·7-9·9)	18·2 (15·3-21·4)	18·6 (15·7-21·8)	25·7 (21·8-29·4)	3·8 (2·8-5·2)	14·9 (10·0-20·3)	15·4 (10·4-21·0)	24·5 (18·2-31·6)	11·2 (9·3-13·9)	20·1 (16·3-23·8)	20·5 (16·9-24·2)	26·9 (22·5-30·9)	100·0 (100·0-100·0)	42·2 (36·9-49·1)	39·3 (33·9-46·5)	45·4 (39·9-52·8)
Comoros	23·4 (21·2-25·4)	34·4 (32·0-36·5)	34·9 (32·6-37·0)	41·1 (37·4-44·3)	14·3 (11·3-18·0)	29·7 (24·3-33·7)	30·6 (25·1-34·8)	41·9 (35·0-48·0)	27·7 (24·2-30·6)	35·6 (32·7-38·4)	35·9 (33·1-38·7)	39·6 (31·7-44·2)	100·0 (100·0-100·0)	100·0 (100·0-100·0)	100·0 (100·0-100·0)	100·0 (100·0-100·0)
Djibouti	22·1 (19·2-24·7)	31·1 (27·0-34·5)	32·0 (27·3-35·6)	34·4 (28·8-39·6)	15·5 (11·2-19·6)	25·2 (19·6-30·6)	25·9 (20·1-31·9)	34·2 (24·1-43·7)	26·6 (22·6-29·8)	34·0 (28·6-38·2)	34·8 (28·9-39·0)	32·6 (24·9-40·0)	16·1 (11·1-22·9)	100·0 (100·0-100·0)	100·0 (100·0-100·0)	39·2 (34·0-46·2)
Eritrea	16·9 (15·0-18·9)	25·3 (22·0-28·8)	25·7 (22·3-29·2)	31·3 (27·1-36·0)	8·8 (6·5-11·1)	17·0 (11·1-23·7)	17·6 (11·4-24·7)	25·7 (16·1-37·0)	23·9 (21·1-26·8)	31·2 (27·2-34·7)	31·3 (27·4-34·8)	35·2 (30·6-39·0)	100·0 (100·0-100·0)	100·0 (100·0-100·0)	100·0 (100·0-100·0)	100·0 (100·0-100·0)
Ethiopia	6·9 (5·8-8·2)	19·2 (17·0-21·5)	20·1 (17·7-22·4)	28·3 (24·6-31·9)	3·6 (2·8-4·4)	13·9 (11·0-17·4)	14·4 (11·4-18·0)	23·5 (17·2-31·3)	9·8 (8·2-11·9)	20·8 (18·4-23·0)	21·7 (19·2-23·9)	27·8 (24·3-31·5)	57·2 (51·6-64·0)	71·5 (65·6-78·4)	100·0 (100·0-100·0)	57·1 (51·4-64·0)
Kenya	25·1 (23·6-26·7)	31·1 (29·9-32·5)	31·7 (30·5-33·0)	36·8 (35·1-38·6)	19·0 (17·4-21·0)	30·0 (27·7-32·4)	30·5 (28·1-33·0)	40·3 (37·1-43·4)	27·9 (26·0-29·6)	31·2 (30·0-32·6)	31·9 (30·6-33·2)	34·7 (32·3-37·1)	59·7 (53·9-66·5)	46·3 (41·5-52·1)	47·0 (42·1-53·0)	46·2 (41·4-52·1)
Madagascar	15·8 (13·9-17·3)	20·2 (17·3-22·9)	20·9 (17·8-23·7)	25·8 (22·0-29·7)	10·5 (8·3-12·8)	10·0 (7·9-12·7)	10·4 (8·1-13·3)	13·8 (10·1-18·4)	18·7 (16·1-20·7)	27·0 (22·5-31·2)	27·8 (23·1-32·1)	33·4 (27·8-38·6)	32·6 (27·5-39·2)	38·2 (32·9-45·2)	40·9 (35·5-47·9)	36·2 (30·9-43·1)
Malawi	14·0 (11·5-17·1)	24·3 (21·8-26·4)	24·9 (22·4-27·0)	31·7 (28·1-34·9)	5·2 (4·2-6·6)	18·3 (13·9-21·7)	19·2 (14·4-22·9)	30·4 (20·8-37·4)	20·2 (16·5-24·4)	25·7 (22·8-28·8)	26·2 (23·4-29·2)	29·9 (26·8-33·2)	54·1 (48·4-61·4)	38·6 (33·2-45·8)	40·0 (34·6-47·2)	45·8 (40·2-53·1)
Mozambique	13·9 (11·9-16·5)	25·4 (22·4-28·5)	26·5 (23·4-29·5)	35·2 (29·1-41·0)	5·6 (4·1-7·6)	15·6 (11·7-20·1)	16·7 (12·4-21·3)	26·8 (17·5-37·5)	21·2 (17·7-25·6)	31·8 (28·0-35·2)	32·6 (29·0-35·9)	39·6 (34·2-43·8)	21·2 (16·6-26·8)	43·4 (38·0-50·5)	45·3 (39·8-52·4)	41·7 (36·4-48·4)
Rwanda	10·6 (8·8-12·8)	27·2 (24·3-29·6)	27·4 (24·5-29·8)	35·3 (28·7-39·4)	6·4 (4·6-8·9)	30·2 (21·7-35·8)	30·4 (21·7-36·1)	45·0 (30·9-53·4)	12·9 (10·7-15·5)	23·6 (20·6-25·8)	23·7 (20·6-26·0)	29·5 (24·0-33·5)	100·0 (100·0-100·0)	58·9 (52·7-66·8)	50·9 (44·7-58·9)	48·2 (42·4-55·8)
Somalia	6·0 (4·9-7·2)	11·3 (9·5-13·0)	11·3 (9·5-13·1)	16·1 (12·6-20·2)	4·1 (2·8-5·7)	9·2 (7·1-11·5)	9·4 (7·1-11·9)	15·0 (8·9-21·5)	8·3 (6·5-10·3)	14·3 (11·5-17·1)	14·2 (11·4-16·8)	18·6 (14·9-22·9)	1·0 (0·0-4·6)	37·2 (32·7-42·5)	40·0 (35·4-45·5)	26·7 (22·7-31·5)
South Sudan	7·4 (6·1-9·0)	11·0 (8·9-13·3)	11·5 (9·3-14·0)	14·0 (10·8-18·0)	3·6 (2·6-4·9)	6·8 (5·0-9·6)	7·1 (5·1-10·2)	10·5 (6·9-15·9)	11·7 (9·9-14·2)	14·3 (11·5-17·5)	14·9 (12·0-18·4)	16·3 (12·2-21·4)	45·1 (35·2-62·2)	45·2 (38·8-54·9)	47·3 (40·8-57·0)	50·5 (44·4-58·8)
Tanzania	16·8 (14·8-18·7)	27·1 (25·0-29·1)	27·6 (25·5-29·7)	33·6 (30·5-36·6)	10·0 (8·3-12·1)	25·2 (22·3-27·9)	26·0 (23·1-28·9)	36·6 (31·7-41·1)	19·8 (16·8-22·8)	27·6 (24·4-30·7)	28·0 (24·7-31·2)	32·5 (28·4-36·7)	53·4 (47·5-60·9)	68·2 (62·1-75·4)	70·4 (64·4-77·6)	69·3 (63·2-76·5)
Uganda	11·0 (9·5-12·6)	21·6 (19·7-23·5)	22·4 (20·4-24·4)	30·8 (28·1-33·4)	8·1 (7·0-9·3)	20·4 (16·2-24·4)	21·2 (16·8-25·4)	32·4 (28·3-38·0)	11·9 (9·8-14·3)	21·9 (19·6-24·2)	22·7 (20·3-24·9)	30·1 (27·0-32·8)	57·2 (51·3-64·6)	49·2 (43·5-56·4)	52·2 (46·5-59·5)	52·9 (47·2-60·3)
Zambia	16·4 (14·3-18·7)	26·1 (23·2-28·5)	26·8 (23·9-29·4)	33·9 (28·8-38·5)	8·7 (7·2-10·5)	23·0 (19·4-26·7)	24·1 (20·1-27·9)	36·3 (29·8-42·7)	20·4 (17·6-23·4)	25·7 (22·2-28·7)	26·2 (22·5-29·3)	29·5 (23·1-35·6)	100·0 (100·0-100·0)	74·7 (68·6-81·7)	77·9 (71·9-84·6)	77·3 (71·3-84·0)
**Central Sub-Saharan Africa**																
Angola	10·3 (8·2-12·7)	24·5 (21·5-27·5)	25·4 (22·3-28·4)	35·3 (30·7-39·9)	5·5 (4·0-7·4)	18·7 (14·5-23·2)	19·9 (15·3-24·5)	34·9 (26·6-42·2)	15·9 (12·5-19·5)	28·3 (24·1-32·0)	28·7 (24·4-32·5)	32·8 (24·9-39·8)	58·8 (49·9-76·2)	47·7 (38·7-66·2)	47·2 (38·1-65·7)	48·2 (39·2-66·6)
Central African Republic	8·1 (6·6-9·9)	10·8 (8·5-13·6)	11·0 (8·8-13·8)	11·9 (8·8-15·6)	2·8 (2·3-3·7)	5·1 (3·5-7·2)	5·2 (3·6-7·4)	7·2 (4·3-11·3)	14·3 (10·9-17·9)	16·6 (12·9-20·2)	16·8 (13·2-20·5)	16·5 (12·2-21·4)	100·0 (100·0-100·0)	100·0 (100·0-100·0)	100·0 (100·0-100·0)	100·0 (100·0-100·0)
Congo	17·3 (15·0-19·3)	27·9 (24·6-30·6)	28·3 (24·7-31·0)	33·4 (25·7-39·4)	12·2 (9·9-14·5)	23·7 (17·9-27·8)	24·4 (18·2-28·7)	29·9 (14·6-43·0)	20·0 (17·0-22·5)	28·8 (24·7-32·4)	29·2 (25·0-33·0)	34·1 (27·5-39·1)	100·0 (100·0-100·0)	64·8 (56·1-81·1)	66·7 (58·1-82·6)	71·0 (62·6-85·8)
Democratic Republic of the Congo	9·2 (7·9-10·9)	16·0 (14·1-18·0)	16·5 (14·6-18·5)	23·0 (18·8-26·5)	5·5 (4·0-7·3)	12·6 (9·4-16·2)	13·3 (9·9-17·2)	21·6 (14·9-28·4)	11·5 (9·7-13·8)	17·2 (14·7-19·6)	17·6 (15·1-20·1)	22·7 (18·1-26·1)	100·0 (100·0-100·0)	66·5 (57·9-82·4)	67·9 (59·4-83·5)	67·1 (58·6-82·9)
Equatorial Guinea	16·2 (12·6-19·7)	28·8 (24·3-33·2)	28·3 (23·3-33·4)	33·5 (27·1-40·4)	8·5 (5·7-11·6)	29·6 (23·8-34·2)	30·3 (24·5-34·9)	42·9 (32·1-49·7)	21·5 (16·9-25·5)	24·8 (19·5-31·5)	23·9 (18·6-31·7)	25·9 (20·1-33·6)	100·0 (100·0-100·0)	100·0 (100·0-100·0)	100·0 (100·0-100·0)	100·0 (100·0-100·0)
Gabon	23·2 (20·8-25·4)	35·3 (32·9-37·7)	35·7 (33·1-38·2)	41·6 (37·2-45·3)	17·6 (14·9-20·1)	33·3 (29·2-37·0)	34·1 (29·8-37·8)	43·5 (37·4-49·3)	26·6 (23·2-29·4)	35·4 (31·8-38·7)	35·6 (32·0-38·9)	39·3 (32·5-44·0)	100·0 (100·0-100·0)	100·0 (100·0-100·0)	100·0 (100·0-100·0)	100·0 (100·0-100·0)

Location	Indicator 2.2.1: Prevalence of stunting in children under 5 (%)				Indicator 2.2.2a: Prevalence of wasting in children under 5 (%)				Indicator 2.2.2b: Prevalence of overweight in children aged 2-4 (%)				Indicator 3.1.1: Maternal mortality ratio (maternal deaths per 100,000 livebirths) in women aged 10-54 years			
	**2000**	**2015**	**2016**	**2030**	**2000**	**2015**	**2016**	**2030**	**2000**	**2015**	**2016**	**2030**	**2000**	**2015**	**2016**	**2030**
Sierra Leone	28·2<br>(15·4-37·5)	31·6<br>(14·6-47·8)	33·4<br>(15·1-50·8)	43·0<br>(23·6-57·9)	36·0<br>(27·5-43·7)	56·9<br>(48·9-64·2)	57·4<br>(49·0-64·9)	65·6<br>(57·7-75·3)	80·6<br>(69·2-90·7)	64·4<br>(47·2-78·4)	63·6<br>(46·3-78·0)	44·2<br>(2·2-75·4)	0·4<br>(0·0-2·6)	4·5<br>(1·2-7·9)	5·0<br>(1·5-8·6)	11·5<br>(3·4-20·5)
Togo	40·5<br>(26·1-53·2)	62·4<br>(51·2-72·7)	64·4<br>(52·7-75·2)	77·5<br>(69·7-82·9)	39·3<br>(30·3-47·8)	65·4<br>(59·2-71·3)	66·2<br>(59·7-72·2)	75·9<br>(66·2-83·1)	92·7<br>(86·9-97·5)	89·0<br>(78·6-97·1)	88·5<br>(77·5-96·8)	79·9<br>(50·6-97·0)	7·0<br>(3·5-12·8)	16·3<br>(12·0-20·6)	16·9<br>(12·5-21·3)	25·9<br>(16·5-36·4)
**Eastern Sub-Saharan Africa**																
Burundi	0·2<br>(0·0-3·0)	7·1<br>(0·0-22·3)	8·4<br>(0·0-24·7)	24·1<br>(1·8-42·2)	52·1<br>(44·1-59·6)	70·1<br>(54·8-81·2)	70·3<br>(53·3-82·2)	77·1<br>(58·3-92·1)	84·7<br>(75·6-91·7)	89·7<br>(78·5-97·3)	89·5<br>(78·3-97·7)	92·1<br>(70·2-100·0)	0·1<br>(0·0-1·1)	12·7<br>(6·9-18·2)	12·9<br>(7·0-18·6)	21·6<br>(7·4-34·3)
Comoros	12·9<br>(0·5-22·1)	45·2<br>(29·8-57·7)	46·7<br>(31·2-59·4)	65·7<br>(50·8-78·4)	33·4<br>(20·8-44·9)	47·0<br>(26·6-63·1)	47·8<br>(26·7-64·4)	59·8<br>(30·9-76·8)	56·8<br>(38·7-72·4)	48·8<br>(24·2-70·3)	48·1<br>(22·8-69·7)	38·5<br>(0·0-76·6)	14·0<br>(10·4-17·8)	26·3<br>(20·7-32·4)	26·7<br>(20·9-32·9)	33·3<br>(20·2-46·2)
Djibouti	44·9<br>(34·7-55·1)	49·9<br>(38·5-60·6)	51·4<br>(39·7-62·4)	49·4<br>(39·2-64·9)	1·0<br>(0·0-9·5)	1·0<br>(0·0-11·3)	1·5<br>(0·0-14·7)	5·4<br>(0·0-28·4)	80·6<br>(64·5-91·8)	53·4<br>(29·0-73·4)	52·1<br>(28·1-72·7)	22·0<br>(0·0-65·2)	10·3<br>(5·0-18·0)	15·3<br>(9·0-23·5)	16·2<br>(9·6-24·7)	23·3<br>(12·2-36·6)
Eritrea	15·7<br>(1·6-26·8)	43·8<br>(22·7-58·9)	45·2<br>(24·5-60·1)	64·9<br>(40·7-81·3)	15·1<br>(5·5-24·7)	39·4<br>(0·0-65·7)	39·9<br>(0·0-66·6)	51·5<br>(0·0-84·8)	97·6<br>(91·7-100·0)	93·7<br>(84·0-100·0)	93·3<br>(83·3-100·0)	87·9<br>(60·2-100·0)	1·2<br>(0·0-4·6)	2·5<br>(0·0-7·3)	2·6<br>(0·0-7·5)	3·6<br>(0·0-12·6)
Ethiopia	9·6<br>(0·0-18·7)	24·0<br>(13·3-33·4)	24·8<br>(14·0-34·3)	35·8<br>(22·7-46·6)	33·8<br>(25·5-41·3)	44·0<br>(34·5-52·3)	44·0<br>(34·1-52·5)	44·5<br>(29·6-57·0)	96·4<br>(89·7-100·0)	81·9<br>(69·2-91·8)	80·9<br>(66·3-91·4)	62·7<br>(21·0-89·3)	0·0<br>(0·0-0·1)	11·9<br>(6·1-18·0)	12·4<br>(6·2-18·8)	25·6<br>(11·7-40·2)
Kenya	29·8<br>(26·7-32·7)	56·0<br>(53·5-58·4)	57·4<br>(54·9-59·8)	73·3<br>(71·6-75·0)	64·9<br>(61·7-67·7)	72·8<br>(69·8-75·5)	73·5<br>(70·5-76·1)	78·6<br>(75·9-81·1)	81·7<br>(73·4-88·1)	81·4<br>(73·6-88·4)	80·8<br>(71·2-88·6)	75·2<br>(50·4-92·3)	10·4<br>(7·0-14·1)	18·4<br>(13·3-23·0)	18·5<br>(13·3-23·2)	27·2<br>(18·4-35·9)
Madagascar	8·5<br>(0·0-19·6)	17·4<br>(1·2-31·8)	18·7<br>(1·9-33·7)	35·8<br>(13·0-51·3)	32·5<br>(17·3-44·8)	0·0<br>(0·0-0·0)	0·0<br>(0·0-0·0)	0·0<br>(0·0-0·0)	93·5<br>(88·0-97·6)	86·5<br>(72·9-96·4)	86·1<br>(72·5-96·6)	80·4<br>(49·2-99·3)	8·6<br>(5·5-11·3)	11·5<br>(5·3-17·8)	12·1<br>(5·6-18·9)	18·0<br>(5·0-32·0)
Malawi	2·7<br>(0·0-11·7)	31·4<br>(19·7-41·3)	33·1<br>(21·1-43·2)	54·0<br>(37·8-65·6)	59·5<br>(53·6-64·8)	84·1<br>(79·5-87·8)	84·8<br>(80·1-88·6)	92·5<br>(87·3-96·2)	53·0<br>(41·7-63·2)	71·1<br>(56·9-82·4)	70·4<br>(55·5-82·3)	79·1<br>(53·0-95·5)	4·6<br>(0·0-13·9)	18·0<br>(11·5-25·3)	18·6<br>(11·7-26·2)	28·6<br>(12·5-44·2)
Mozambique	12·7<br>(0·0-24·8)	43·5<br>(26·0-57·8)	46·5<br>(27·9-61·0)	62·5<br>(45·0-75·2)	54·2<br>(45·4-62·9)	77·7<br>(68·4-85·7)	78·1<br>(68·0-86·5)	85·0<br>(74·6-94·7)	72·7<br>(61·6-81·8)	69·3<br>(54·8-81·5)	68·4<br>(52·3-81·5)	60·3<br>(22·0-87·0)	11·9<br>(6·2-21·6)	15·8<br>(10·8-22·5)	16·3<br>(11·3-22·6)	21·1<br>(7·0-34·8)
Rwanda	11·8<br>(0·7-21·4)	33·4<br>(17·0-45·6)	35·3<br>(18·4-47·6)	58·4<br>(37·7-70·9)	57·1<br>(47·8-65·4)	85·1<br>(75·9-92·4)	85·3<br>(75·8-92·7)	88·9<br>(74·6-97·2)	71·2<br>(63·8-78·4)	66·9<br>(57·1-76·2)	65·5<br>(53·7-76·4)	55·9<br>(28·0-78·1)	3·6<br>(0·0-8·0)	20·1<br>(14·1-26·2)	20·4<br>(14·4-26·7)	36·3<br>(23·2-50·0)
Somalia	40·6<br>(28·9-48·2)	45·8<br>(27·4-59·4)	45·9<br>(27·2-60·0)	48·3<br>(27·1-67·6)	0·4<br>(0·0-4·4)	15·5<br>(0·0-44·0)	15·5<br>(0·0-45·6)	17·7<br>(0·0-64·4)	91·0<br>(79·6-99·4)	88·1<br>(75·3-97·3)	87·8<br>(74·1-97·2)	84·2<br>(52·9-100·0)	0·0<br>(0·0-0·0)	0·1<br>(0·0-1·5)	0·2<br>(0·0-2·0)	2·4<br>(0·0-10·3)
South Sudan	20·2<br>(10·2-28·8)	42·2<br>(30·5-54·0)	41·9<br>(28·7-55·1)	48·5<br>(39·4-70·3)	0·9<br>(0·0-10·8)	2·6<br>(0·0-19·0)	4·0<br>(0·0-24·4)	27·3<br>(0·0-54·2)	76·5<br>(59·0-88·8)	74·6<br>(54·5-89·7)	74·2<br>(53·1-89·7)	70·4<br>(28·6-95·5)	7·8<br>(2·4-15·6)	11·5<br>(6·0-16·9)	11·6<br>(6·1-16·8)	14·0<br>(5·7-23·2)
Tanzania	9·7<br>(0·0-22·3)	36·8<br>(21·3-48·0)	39·1<br>(23·2-50·5)	63·7<br>(46·9-71·8)	68·1<br>(60·1-75·6)	74·0<br>(65·0-81·5)	74·3<br>(64·6-82·1)	79·1<br>(64·6-89·1)	84·8<br>(77·1-91·2)	86·3<br>(77·5-93·2)	85·5<br>(75·6-93·3)	80·4<br>(54·6-96·7)	7·0<br>(3·5-11·5)	11·6<br>(7·4-16·4)	12·2<br>(7·8-16·8)	17·2<br>(7·7-26·5)
Uganda	16·5<br>(2·0-27·1)	45·3<br>(31·7-55·8)	47·0<br>(32·8-57·9)	67·5<br>(52·2-78·1)	72·1<br>(66·8-77·6)	77·9<br>(71·3-84·1)	78·3<br>(71·3-84·8)	84·1<br>(73·0-90·7)	76·0<br>(67·8-82·7)	80·8<br>(70·2-89·0)	79·8<br>(67·3-89·2)	85·1<br>(64·5-98·3)	16·5<br>(11·7-20·8)	20·3<br>(14·6-26·0)	21·0<br>(14·9-26·7)	27·2<br>(14·1-39·1)
Zambia	1·3<br>(0·0-8·2)	25·0<br>(8·5-37·7)	26·2<br>(9·7-39·0)	42·7<br>(24·8-55·4)	70·5<br>(65·6-74·8)	70·4<br>(61·0-77·5)	70·2<br>(60·4-77·5)	67·5<br>(54·9-77·9)	46·3<br>(33·7-57·4)	33·0<br>(10·4-54·8)	32·3<br>(8·2-56·3)	21·4<br>(0·0-62·3)	7·5<br>(1·2-14·1)	24·7<br>(15·1-34·3)	25·4<br>(15·6-35·6)	42·2<br>(20·7-64·9)
**Central Sub-Saharan Africa**																
Angola	6·1<br>(0·0-15·4)	44·7<br>(23·2-58·7)	45·8<br>(23·8-59·9)	61·0<br>(42·0-74·7)	28·5<br>(17·3-40·6)	56·1<br>(46·3-65·5)	57·5<br>(47·8-66·7)	74·3<br>(66·3-81·2)	77·2<br>(52·4-94·9)	52·9<br>(16·1-82·7)	50·9<br>(12·3-82·2)	29·0<br>(0·0-82·9)	11·9<br>(5·8-17·6)	23·8<br>(16·1-30·5)	25·0<br>(17·1-32·1)	40·5<br>(26·1-55·0)
Central African Republic	16·2<br>(5·5-23·9)	29·3<br>(12·8-42·8)	30·3<br>(13·2-44·8)	44·4<br>(19·6-59·2)	48·9<br>(42·5-56·1)	57·1<br>(49·3-65·3)	57·8<br>(49·7-66·1)	66·5<br>(55·5-76·8)	71·8<br>(48·0-88·9)	81·8<br>(56·6-98·4)	82·2<br>(58·0-98·3)	84·4<br>(41·6-100·0)	0·1<br>(0·0-1·0)	0·4<br>(0·0-3·7)	0·5<br>(0·0-4·0)	3·0<br>(0·0-14·7)
Congo	51·6<br>(39·2-62·6)	63·3<br>(54·3-71·1)	64·6<br>(55·7-72·3)	79·2<br>(71·4-85·5)	63·3<br>(56·4-69·1)	62·3<br>(56·2-67·7)	62·4<br>(56·3-67·8)	62·6<br>(56·7-67·4)	71·8<br>(47·9-89·3)	83·1<br>(61·6-96·8)	81·9<br>(56·8-96·9)	77·8<br>(29·0-100·0)	3·4<br>(0·0-8·2)	9·6<br>(2·8-16·9)	10·1<br>(3·2-17·8)	18·3<br>(2·5-33·7)
Democratic Republic of the Congo	18·0<br>(6·6-26·6)	28·8<br>(14·2-40·3)	30·5<br>(15·3-42·6)	33·5<br>(20·5-47·3)	24·5<br>(16·2-32·6)	56·7<br>(48·9-63·5)	57·9<br>(50·0-64·8)	71·7<br>(62·7-78·7)	72·4<br>(53·2-87·7)	75·9<br>(49·1-93·6)	75·3<br>(46·3-93·9)	73·5<br>(18·5-100·0)	9·8<br>(6·3-13·5)	7·2<br>(3·7-11·0)	8·2<br>(4·6-12·1)	11·7<br>(2·1-22·5)
Equatorial Guinea	13·5<br>(0·5-23·2)	61·3<br>(51·9-70·4)	62·7<br>(52·4-72·4)	80·6<br>(71·8-90·4)	74·5<br>(70·3-78·5)	84·9<br>(81·9-87·7)	85·5<br>(82·4-88·3)	92·0<br>(88·3-94·9)	47·0<br>(16·5-74·6)	5·3<br>(0·0-34·1)	5·1<br>(0·0-35·0)	1·9<br>(0·0-26·9)	2·3<br>(0·0-8·8)	21·5<br>(11·4-34·7)	22·2<br>(12·0-35·3)	39·2<br>(20·8-60·4)
Gabon	53·9<br>(47·2-59·4)	70·7<br>(61·8-78·4)	71·5<br>(62·4-79·1)	80·6<br>(70·9-88·1)	77·7<br>(72·5-81·9)	82·1<br>(77·6-85·8)	82·4<br>(77·9-86·1)	85·8<br>(81·9-89·1)	80·0<br>(67·2-90·3)	70·9<br>(51·3-86·6)	69·2<br>(46·9-86·7)	60·4<br>(10·2-93·6)	8·1<br>(3·7-12·6)	20·8<br>(14·3-27·1)	21·4<br>(14·8-27·9)	30·6<br>(14·8-46·5)

Location	Indicator 3.1.2: Proportion of births attended by skilled health personnel (%)				Indicator 3.2.1: Under-5 mortality rate (probability of dying before the age of 5 per 1,000 livebirths)				Indicator 3.2.2: Neonatal mortality rate (probability of dying during the first 28 days of life per 1,000 livebirths)				Indicator 3.3.1: Age-standardised rate of new HIV infections (per 1,000 population)			
	**2000**	**2015**	**2016**	**2030**	**2000**	**2015**	**2016**	**2030**	**2000**	**2015**	**2016**	**2030**	**2000**	**2015**	**2016**	**2030**
Sierra Leone	29·5 (20·1-39·4)	41·7 (27·2-55·5)	43·4 (26·8-58·9)	58·6 (18·8-89·0)	0·0 (0·0-0·0)	8·1 (5·3-10·8)	9·4 (6·5-12·3)	23·2 (17·1-29·6)	0·0 (0·0-0·0)	7·5 (4·8-10·3)	8·3 (5·5-11·2)	19·4 (13·2-25·8)	21·7 (18·1-25·6)	34·2 (27·6-43·8)	35·0 (27·7-46·1)	39·0 (31·3-51·0)
Togo	40·9 (33·5-48·4)	53·5 (42·0-65·1)	55·5 (41·4-69·0)	66·3 (24·5-94·0)	8·0 (6·6-9·4)	20·3 (17·1-23·3)	21·4 (17·9-24·6)	36·0 (29·0-42·5)	7·2 (5·2-9·4)	16·0 (12·1-19·6)	17·0 (13·0-20·9)	28·3 (19·9-36·7)	7·2 (4·3-10·4)	26·1 (21·0-32·0)	25·7 (19·8-32·3)	29·3 (23·3-36·2)
**Eastern Sub-Saharan Africa**																
Burundi	12·1 (2·1-23·5)	61·5 (37·7-81·1)	60·1 (32·5-81·8)	78·1 (17·5-100·0)	1·4 (0·0-4·0)	17·3 (9·8-24·5)	18·0 (9·6-25·5)	30·3 (9·4-48·0)	2·6 (0·0-6·1)	14·6 (3·8-24·3)	15·3 (3·5-25·6)	25·6 (0·0-49·2)	19·0 (14·0-26·0)	41·0 (22·5-61·1)	41·9 (19·2-65·7)	46·1 (22·3-73·0)
Comoros	62·7 (56·2-69·0)	81·9 (71·0-89·7)	81·7 (68·9-90·8)	88·3 (55·3-100·0)	13·3 (10·7-16·0)	26·7 (20·6-32·3)	27·8 (21·1-33·8)	43·3 (28·6-56·9)	4·0 (0·1-8·0)	14·6 (10·1-19·0)	15·7 (11·0-20·6)	26·4 (12·8-39·7)	63·4 (42·6-82·9)	60·9 (37·5-83·9)	57·1 (33·9-81·3)	56·6 (33·5-80·5)
Djibouti	79·2 (62·4-91·4)	90·7 (76·4-97·4)	90·6 (76·8-97·4)	93·1 (59·5-100·0)	12·2 (10·0-14·5)	28·0 (23·1-32·2)	29·8 (24·9-34·2)	46·7 (37·8-55·0)	12·2 (9·5-15·2)	24·3 (18·5-29·6)	25·9 (20·0-31·4)	39·0 (28·5-48·9)	12·4 (4·7-20·5)	29·4 (19·1-41·5)	29·8 (18·7-43·7)	35·0 (23·6-49·2)
Eritrea	9·8 (5·3-14·8)	13·8 (0·0-44·8)	14·8 (0·0-47·3)	24·0 (0·0-78·6)	14·5 (11·8-17·2)	27·2 (21·7-32·0)	28·4 (22·8-33·4)	42·6 (30·7-52·7)	16·8 (12·5-21·4)	25·3 (17·9-32·1)	26·5 (19·0-33·2)	36·5 (21·3-49·7)	28·8 (22·4-35·0)	38·2 (31·0-47·3)	37·4 (29·7-47·8)	41·3 (33·5-52·2)
Ethiopia	0·0 (0·0-0·0)	5·3 (0·0-24·9)	6·5 (0·0-28·2)	31·5 (0·0-91·3)	4·9 (3·5-6·2)	30·5 (26·7-33·8)	32·1 (28·2-35·7)	56·4 (48·7-63·7)	2·7 (1·4-4·2)	23·1 (18·5-27·2)	24·8 (19·9-29·2)	44·4 (34·8-53·0)	17·3 (13·9-22·0)	38·8 (28·9-51·5)	38·5 (26·7-55·5)	42·7 (30·3-61·1)
Kenya	35·2 (27·4-42·9)	57·4 (43·0-71·2)	58·5 (41·0-73·3)	78·3 (40·9-98·1)	15·1 (13·6-16·7)	31·5 (28·6-33·5)	32·1 (29·1-34·5)	47·0 (39·6-53·9)	14·4 (12·6-16·2)	25·3 (21·7-28·5)	25·9 (22·1-29·4)	36·0 (26·6-45·1)	10·2 (6·7-15·1)	15·2 (13·5-17·1)	15·1 (13·3-16·9)	20·5 (18·2-22·8)
Madagascar	31·7 (25·3-37·7)	10·3 (0·0-27·9)	12·0 (0·0-32·7)	9·9 (0·0-44·3)	9·0 (6·6-11·7)	17·2 (12·1-22·1)	18·1 (12·8-23·0)	28·2 (15·5-41·5)	9·1 (6·1-12·8)	14·6 (9·6-19·5)	15·5 (10·1-20·4)	23·3 (9·6-37·4)	39·9 (25·6-53·3)	51·5 (36·9-66·2)	52·0 (36·9-66·9)	50·1 (34·6-65·0)
Malawi	45·0 (39·4-50·4)	84·1 (75·3-90·3)	84·7 (74·8-91·5)	96·0 (82·9-99·9)	1·1 (0·0-2·9)	20·1 (15·1-24·5)	21·5 (16·3-26·2)	41·1 (27·2-53·5)	4·8 (2·4-7·1)	16·8 (11·5-21·7)	18·0 (12·2-23·4)	31·3 (17·5-44·9)	0·0 (0·0-0·0)	5·8 (3·9-7·8)	6·3 (4·4-8·4)	10·4 (8·3-12·9)
Mozambique	35·1 (31·8-38·5)	52·0 (33·5-69·0)	53·8 (33·2-73·1)	68·2 (23·6-95·6)	1·6 (0·5-2·7)	17·8 (14·7-20·8)	19·1 (15·7-22·3)	34·5 (27·6-41·2)	3·2 (1·9-4·8)	16·2 (13·2-19·2)	17·5 (14·3-20·6)	29·6 (23·3-36·4)	0·0 (0·0-0·0)	6·6 (3·6-9·8)	8·4 (4·6-12·1)	12·8 (9·0-16·8)
Rwanda	6·3 (0·0-14·4)	88·3 (80·8-93·5)	88·5 (80·0-94·0)	99·1 (94·0-100·0)	2·4 (0·3-4·6)	28·2 (23·7-32·7)	29·3 (24·0-33·9)	48·8 (34·9-61·7)	4·9 (1·5-8·0)	24·9 (20·5-29·2)	25·9 (21·0-30·5)	41·1 (26·7-54·4)	15·0 (10·0-26·1)	47·4 (35·5-68·0)	44·5 (33·0-63·8)	50·4 (36·5-75·1)
Somalia	12·0 (0·0-33·1)	27·3 (9·8-46·5)	27·7 (7·4-50·1)	34·7 (0·0-74·3)	2·5 (0·8-4·3)	12·9 (8·8-16·8)	13·5 (9·3-17·4)	22·9 (15·3-29·6)	4·9 (3·2-6·8)	12·7 (8·7-16·6)	13·4 (9·2-17·5)	21·1 (13·5-28·4)	30·1 (18·6-40·6)	40·7 (28·2-52·9)	41·4 (28·1-53·8)	45·6 (31·7-58·4)
South Sudan	1·3 (0·0-13·1)	26·1 (6·9-49·6)	25·3 (3·8-51·6)	54·6 (0·0-100·0)	4·3 (2·5-6·4)	13·5 (8·6-18·6)	14·4 (9·1-19·7)	22·7 (11·5-33·6)	5·1 (2·4-8·1)	11·2 (4·3-18·0)	11·8 (4·6-18·9)	17·3 (2·5-32·2)	14·8 (5·2-24·4)	22·8 (13·2-32·5)	24·5 (14·6-35·2)	28·6 (18·5-39·7)
Tanzania	26·0 (20·1-31·8)	47·9 (28·5-66·1)	49·2 (27·4-68·8)	70·0 (23·7-99·9)	7·8 (6·6-8·9)	25·1 (21·9-28·1)	26·3 (22·8-29·5)	43·3 (35·5-50·3)	8·8 (6·5-11·3)	20·7 (17·5-23·6)	21·8 (18·3-24·9)	34·3 (25·9-43·2)	7·2 (4·8-10·6)	18·5 (14·4-22·8)	19·0 (14·3-23·4)	23·3 (18·1-28·3)
Uganda	22·3 (17·2-27·0)	55·3 (37·0-71·4)	55·1 (34·0-73·2)	67·1 (24·1-92·8)	5·8 (4·7-7·0)	22·4 (19·6-25·0)	23·7 (20·6-26·5)	41·4 (34·6-47·8)	8·2 (7·0-9·3)	18·9 (15·8-21·9)	20·1 (16·7-23·3)	32·1 (24·4-39·6)	14·0 (10·7-19·1)	13·6 (8·1-19·8)	14·8 (7·4-23·6)	19·8 (12·2-29·5)
Zambia	34·9 (29·5-40·0)	66·4 (53·9-77·2)	68·9 (53·8-81·3)	90·4 (67·8-100·0)	4·5 (2·3-7·0)	23·9 (19·2-28·7)	24·8 (19·8-29·7)	43·8 (29·9-56·5)	9·2 (6·7-12·1)	21·2 (15·9-26·6)	22·0 (16·2-27·7)	33·6 (19·0-48·0)	0·0 (0·0-0·0)	5·3 (2·9-8·0)	6·3 (3·8-9·1)	12·0 (9·3-15·1)
**Central Sub-Saharan Africa**																
Angola	31·0 (25·0-36·9)	63·7 (36·1-84·9)	65·6 (37·5-86·2)	83·0 (29·2-100·0)	3·2 (0·1-6·3)	24·9 (18·6-31·4)	27·1 (20·6-33·3)	55·3 (38·8-73·3)	6·5 (2·8-10·0)	23·9 (16·8-31·0)	25·9 (18·8-33·2)	48·9 (29·9-69·1)	22·2 (15·0-29·4)	27·0 (16·4-39·7)	28·7 (16·7-43·0)	33·2 (20·8-48·2)
Central African Republic	31·5 (26·8-35·9)	39·3 (21·1-58·7)	38·8 (16·7-61·5)	35·0 (0·0-76·4)	0·4 (0·0-2·8)	6·0 (0·0-12·4)	6·8 (0·1-13·0)	14·9 (0·0-32·0)	1·0 (0·0-4·5)	4·6 (0·0-11·3)	5·2 (0·0-11·7)	10·7 (0·0-29·2)	4·8 (2·0-9·5)	18·2 (12·4-23·0)	18·3 (11·8-23·3)	21·8 (14·9-27·3)
Congo	85·6 (79·1-91·1)	94·1 (89·0-97·5)	94·6 (89·0-98·0)	98·2 (90·2-100·0)	12·0 (9·5-14·6)	26·8 (19·6-33·4)	27·8 (19·7-34·9)	43·4 (25·1-59·6)	13·7 (10·9-16·8)	23·9 (15·8-31·3)	24·9 (15·7-32·9)	37·4 (16·9-56·1)	14·1 (11·4-17·9)	21·7 (19·5-24·2)	22·1 (19·9-24·7)	27·0 (24·4-30·2)
Democratic Republic of the Congo	35·1 (8·9-61·1)	79·3 (62·5-90·4)	79·7 (62·3-91·0)	93·0 (67·7-100·0)	3·0 (0·0-6·2)	16·8 (10·5-23·3)	18·0 (11·4-24·7)	32·1 (13·0-50·1)	6·6 (2·8-10·6)	16·2 (9·2-23·6)	17·5 (10·1-25·3)	28·3 (6·4-48·4)	22·5 (18·5-26·8)	38·0 (31·3-47·2)	36·7 (29·1-45·5)	40·5 (32·4-50·1)
Equatorial Guinea	58·0 (36·6-75·0)	84·3 (64·1-95·8)	85·6 (65·8-96·2)	93·4 (64·2-100·0)	3·9 (0·0-8·2)	25·1 (16·1-33·0)	26·2 (16·8-34·3)	42·4 (26·6-56·6)	5·3 (0·0-11·5)	22·0 (13·5-29·7)	23·0 (14·1-30·9)	37·7 (21·9-51·7)	3·6 (0·2-7·8)	11·7 (6·0-19·9)	12·4 (6·0-22·3)	20·5 (13·8-30·9)
Gabon	83·6 (80·0-86·6)	90·2 (84·0-94·6)	90·2 (82·8-95·3)	93·6 (74·4-100·0)	21·0 (18·9-23·1)	31·2 (26·4-35·4)	32·0 (27·0-36·5)	44·4 (34·8-53·6)	16·3 (13·8-18·7)	23·3 (17·6-28·8)	24·2 (18·0-29·8)	34·9 (23·7-47·0)	8·6 (5·1-12·4)	18·8 (14·6-23·4)	19·6 (14·9-24·9)	26·2 (21·4-31·7)

Location	Indicator 3.3.2: Age-standardised rate of tuberculosis cases (per 100,000 population)				Indicator 3.3.3: Age-standardised rate of malaria cases (per 1,000 population)				Indicator 3.3.4: Age-standardised rate of hepatitis B incidence (per 100,000 population)				Indicator 3.3.5: Age-standardised prevalence* of the sum of 15 neglected tropical diseases (NTDs) (%) *Prevalence estimates reported here may exceed 100% as they reflect the sum of prevalent cases of 15 NTDs.*			
	**2000**	**2015**	**2016**	**2030**	**2000**	**2015**	**2016**	**2030**	**2000**	**2015**	**2016**	**2030**	**2000**	**2015**	**2016**	**2030**
Sierra Leone	20·0 (18·6-21·3)	24·3 (22·8-25·7)	24·9 (23·5-26·4)	30·1 (28·5-31·8)	0·6 (0·0-2·9)	1·2 (0·0-3·9)	1·5 (0·0-4·3)	0·9 (0·0-4·7)	0·0 (0·0-0·0)	5·2 (3·2-7·9)	5·8 (3·7-8·4)	13·9 (11·3-16·9)	0·0 (0·0-0·0)	57·7 (52·0-62·8)	60·6 (55·2-65·4)	86·3 (84·0-88·3)
Togo	22·9 (21·5-24·1)	28·3 (26·8-29·6)	28·7 (27·3-30·1)	33·4 (31·7-35·2)	0·3 (0·0-1·1)	0·9 (0·0-1·7)	1·0 (0·1-1·8)	2·2 (1·3-3·2)	3·5 (1·1-6·2)	10·4 (7·6-13·6)	11·0 (8·3-14·2)	16·8 (13·2-21·1)	25·3 (21·3-29·6)	79·1 (77·4-80·7)	82·1 (80·5-83·5)	97·9 (97·4-98·3)
**Eastern Sub-Saharan Africa**																
Burundi	6·9 (5·4-8·5)	14·5 (13·0-16·1)	14·7 (13·1-16·2)	18·7 (16·4-21·2)	1·1 (0·0-3·3)	7·5 (5·7-9·7)	8·0 (6·1-10·2)	13·9 (11·6-16·7)	7·0 (4·4-9·6)	14·9 (12·3-17·4)	15·3 (12·7-17·8)	21·1 (18·3-23·7)	0·0 (0·0-0·0)	50·6 (46·0-54·7)	53·9 (49·6-57·7)	82·9 (81·0-84·5)
Comoros	26·0 (24·4-27·4)	28·4 (26·8-29·9)	28·3 (26·7-29·8)	27·2 (25·6-28·8)	16·8 (9·3-23·8)	32·0 (28·4-36·6)	36·5 (32·9-41·2)	35·6 (29·0-44·6)	14·2 (12·1-16·6)	21·6 (19·4-23·9)	22·1 (19·8-24·4)	29·4 (26·9-31·6)	29·2 (16·2-40·2)	33·1 (19·6-44·0)	33·1 (19·6-44·0)	34·0 (20·4-44·9)
Djibouti	17·7 (16·5-19·0)	15·5 (14·2-16·8)	15·6 (14·4-17·0)	15·9 (14·5-17·5)	33·7 (29·9-37·3)	27·0 (19·3-34·5)	24·6 (16·3-32·6)	29·3 (21·9-38·3)	15·2 (13·3-16·7)	23·4 (21·1-25·5)	24·1 (21·8-26·3)	31·2 (28·0-34·6)	73·3 (65·9-77·6)	73·7 (66·5-78·0)	73·7 (66·6-78·0)	74·1 (67·0-78·4)
Eritrea	23·9 (22·2-25·4)	19·8 (18·2-21·3)	19·8 (18·2-21·2)	19·2 (17·6-20·8)	18·7 (12·0-28·1)	28·3 (25·6-31·2)	29·2 (26·4-32·2)	41·4 (37·6-45·4)	9·2 (6·8-11·2)	14·3 (11·6-16·6)	14·7 (12·0-17·1)	20·8 (17·6-24·0)	78·6 (72·3-84·0)	80·0 (74·1-84·9)	80·1 (74·3-85·0)	82·3 (76·9-86·3)
Ethiopia	11·5 (10·1-12·9)	22·5 (21·1-23·9)	23·2 (21·8-24·6)	34·2 (32·2-36·3)	11·8 (8·9-14·4)	27·5 (24·9-30·2)	27·7 (25·1-30·4)	30·7 (27·5-33·3)	0·6 (0·0-2·2)	14·2 (11·5-16·8)	15·0 (12·1-17·6)	27·0 (22·5-30·8)	32·7 (25·4-39·5)	38·9 (31·8-45·5)	39·8 (32·7-46·3)	50·6 (44·1-56·5)
Kenya	25·1 (23·5-26·8)	25·2 (23·6-26·8)	25·4 (23·8-27·1)	29·4 (27·5-31·2)	5·4 (4·1-6·9)	12·8 (11·2-14·8)	12·7 (11·0-14·7)	12·4 (10·8-14·4)	15·7 (13·8-17·5)	22·2 (20·2-24·3)	22·8 (20·7-25·0)	30·0 (27·6-32·4)	68·2 (64·3-71·7)	71·0 (67·8-73·8)	71·2 (68·1-74·0)	74·5 (71·9-76·8)
Madagascar	30·8 (28·3-33·3)	35·4 (32·9-38·0)	35·6 (33·2-38·3)	39·8 (37·5-42·2)	6·3 (4·0-8·5)	11·6 (9·6-13·8)	11·9 (9·8-14·1)	15·3 (13·0-17·9)	20·0 (17·5-22·4)	22·7 (20·0-25·3)	23·1 (20·3-25·6)	27·3 (24·3-30·0)	0·0 (0·0-0·0)	35·6 (27·7-42·5)	40·0 (32·7-46·5)	77·8 (75·2-80·2)
Malawi	8·9 (7·4-10·5)	17·7 (16·0-19·4)	18·4 (16·7-20·2)	29·2 (26·7-31·9)	0·9 (0·0-2·4)	3·3 (2·3-4·7)	3·7 (2·6-5·2)	8·0 (6·5-9·5)	0·0 (0·0-0·0)	9·7 (7·3-11·9)	10·6 (8·1-12·8)	23·1 (19·6-26·3)	34·8 (27·6-41·3)	78·2 (75·8-80·3)	80·9 (78·7-82·8)	96·9 (96·5-97·4)
Mozambique	13·2 (11·7-14·7)	14·6 (12·9-16·3)	15·0 (13·2-16·7)	16·6 (14·1-19·3)	0·0 (0·0-0·4)	2·5 (1·6-3·4)	2·6 (1·7-3·6)	4·9 (3·5-6·7)	3·7 (1·5-6·2)	13·9 (11·2-16·5)	14·5 (11·7-17·0)	22·7 (19·3-25·6)	36·2 (32·0-40·0)	42·0 (38·2-45·5)	42·6 (38·9-46·1)	51·4 (48·0-54·6)
Rwanda	6·2 (4·7-7·7)	23·5 (21·9-25·0)	23·8 (22·2-25·4)	32·0 (29·4-34·4)	8·5 (0·1-22·0)	4·3 (1·5-8·2)	3·4 (0·7-7·0)	3·6 (0·8-7·3)	0·0 (0·0-0·0)	16·0 (14·1-17·8)	16·6 (14·6-18·5)	25·3 (22·4-28·5)	30·5 (19·5-39·7)	37·1 (26·8-45·9)	37·6 (27·3-46·4)	45·0 (34·9-53·1)
Somalia	19·2 (17·7-20·5)	17·6 (16·2-18·9)	17·6 (16·2-18·9)	17·9 (16·3-19·4)	11·6 (9·2-14·4)	15·4 (12·1-18·6)	14·5 (11·0-17·9)	7·7 (5·8-9·5)	2·6 (0·9-4·1)	8·0 (5·9-9·8)	8·4 (6·3-10·3)	14·2 (11·4-16·6)	37·5 (29·9-44·6)	38·5 (31·2-45·5)	38·6 (31·3-45·6)	40·0 (32·7-46·9)
South Sudan	30·0 (28·4-31·6)	27·5 (25·8-29·1)	27·4 (25·8-29·0)	26·1 (24·4-27·7)	3·4 (0·7-6·1)	7·2 (3·6-11·1)	7·3 (3·7-11·2)	9·6 (4·8-14·0)	13·6 (11·9-15·2)	16·1 (14·3-18·0)	16·3 (14·5-18·2)	18·7 (16·6-21·0)	46·7 (38·1-54·5)	59·5 (52·5-65·1)	60·2 (53·3-65·7)	69·4 (63·4-74·0)
Tanzania	21·8 (20·5-23·2)	25·2 (23·8-26·6)	25·4 (24·0-26·8)	29·8 (27·8-31·5)	2·4 (1·5-3·7)	8·5 (7·3-10·2)	8·7 (7·5-10·4)	12·0 (10·4-14·5)	5·1 (2·9-7·0)	13·1 (10·6-15·5)	13·7 (11·2-16·2)	22·0 (18·7-25·5)	17·1 (9·7-23·3)	43·2 (36·4-49·1)	45·9 (39·2-51·5)	72·2 (67·0-76·4)
Uganda	5·5 (4·1-7·0)	12·0 (10·7-13·4)	12·4 (11·2-13·9)	18·4 (16·8-20·1)	0·3 (0·0-1·1)	5·3 (4·1-6·8)	6·1 (4·8-7·8)	7·6 (6·4-9·4)	10·5 (7·5-13·4)	16·7 (13·6-19·9)	17·2 (14·1-20·4)	22·9 (19·6-26·2)	3·4 (0·0-10·2)	57·5 (54·4-60·4)	60·8 (57·9-63·5)	87·4 (86·4-88·4)
Zambia	0·0 (0·0-0·0)	3·0 (1·6-4·6)	3·8 (2·3-5·4)	14·6 (12·2-17·1)	2·2 (0·9-3·9)	6·4 (4·9-8·1)	6·1 (4·6-8·0)	5·7 (4·2-7·5)	0·0 (0·0-0·0)	6·2 (3·4-8·6)	7·0 (4·2-9·4)	18·0 (14·5-21·3)	41·6 (33·5-48·5)	46·7 (38·9-53·5)	47·4 (39·6-54·1)	55·7 (48·1-62·2)
**Central Sub-Saharan Africa**																
Angola	9·5 (8·2-10·9)	16·1 (14·8-17·6)	16·6 (15·2-18·0)	22·9 (21·5-24·4)	4·5 (3·1-6·0)	8·8 (7·0-10·7)	8·8 (6·9-10·8)	8·8 (6·0-12·3)	10·2 (8·2-12·1)	22·3 (20·0-24·4)	23·3 (20·9-25·4)	36·1 (33·4-38·7)	40·9 (33·8-47·7)	44·1 (37·4-50·5)	44·5 (37·8-50·8)	49·7 (43·3-55·9)
Central African Republic	0·0 (0·0-0·0)	0·0 (0·0-0·0)	0·0 (0·0-0·0)	0·8 (0·0-2·3)	0·6 (0·0-2·8)	2·4 (0·6-5·6)	2·7 (0·7-5·9)	5·9 (2·8-10·3)	2·4 (0·2-4·8)	4·4 (2·0-6·8)	4·8 (2·3-7·3)	5·5 (2·9-8·4)	23·0 (17·6-28·1)	34·4 (29·7-38·7)	35·1 (30·4-39·4)	45·2 (40·8-49·0)
Congo	1·0 (0·0-2·4)	12·3 (11·2-13·7)	12·8 (11·7-14·2)	19·4 (18·1-21·0)	2·0 (0·2-4·3)	4·9 (2·7-7·6)	4·7 (2·6-7·5)	4·0 (1·9-6·8)	13·0 (10·6-15·1)	22·9 (20·6-24·9)	23·4 (21·1-25·5)	30·9 (27·8-34·2)	0·0 (0·0-0·0)	3·6 (0·0-11·9)	4·8 (0·0-13·4)	24·7 (14·1-33·6)
Democratic Republic of the Congo	6·4 (5·1-7·9)	11·9 (10·5-13·4)	12·3 (10·9-13·8)	17·5 (15·9-19·2)	0·2 (0·0-0·8)	2·8 (1·9-3·9)	3·3 (2·3-4·4)	6·2 (4·9-7·3)	8·5 (6·0-11·1)	15·0 (12·5-17·6)	15·5 (13·1-18·1)	23·0 (20·2-25·9)	0·0 (0·0-0·3)	3·6 (0·0-11·9)	4·4 (0·0-12·9)	17·2 (7·2-26·2)
Equatorial Guinea	4·5 (3·2-5·9)	14·4 (13·0-15·8)	14·9 (13·5-16·3)	22·2 (20·6-23·8)	0·2 (0·0-1·4)	2·1 (1·2-3·4)	1·8 (0·9-2·9)	1·2 (0·2-2·2)	18·5 (16·2-20·6)	38·2 (35·8-40·5)	39·3 (36·7-41·6)	54·7 (50·5-58·8)	6·2 (0·0-14·8)	10·8 (2·7-19·2)	11·1 (3·0-19·5)	15·3 (7·6-23·5)
Gabon	6·6 (5·4-7·8)	16·4 (15·2-17·8)	17·1 (15·8-18·4)	27·1 (25·1-29·0)	2·0 (0·0-4·7)	4·2 (0·8-8·0)	4·0 (0·5-7·5)	1·4 (0·0-6·6)	17·6 (15·2-20·0)	26·6 (24·4-28·8)	27·3 (25·0-29·5)	36·3 (33·6-38·9)	5·6 (0·0-14·4)	6·2 (0·1-14·8)	6·3 (0·2-14·8)	6·7 (0·6-15·2)

Location	Indicator 3.4.1: Age-standardised death rate due to cardiovascular disease, cancer, diabetes, and chronic respiratory disease in populations aged 30-70 (per 100,000 population)				Indicator 3.4.2: Age-standardised death rate due to self-harm (per 100,000 population)				Indicator 3.5.2: Risk-weighted prevalence of alcohol consumption, as measured by the summary exposure value (SEV) for alcohol use (%)				Indicator 3.6.1: Age-standardised death rate due to road injuries (per 100,000 population)			
	**2000**	**2015**	**2016**	**2030**	**2000**	**2015**	**2016**	**2030**	**2000**	**2015**	**2016**	**2030**	**2000**	**2015**	**2016**	**2030**
Sierra Leone	41·4<br>(35·5-48·4)	38·8<br>(31·0-46·2)	38·6<br>(31·0-45·9)	38·4<br>(23·9-52·6)	51·8<br>(39·1-63·1)	47·6<br>(33·7-59·1)	47·4<br>(33·2-58·9)	45·7<br>(26·2-63·0)	42·8<br>(28·5-55·1)	52·2<br>(35·5-71·3)	53·3<br>(36·7-72·3)	66·2<br>(50·8-83·6)	30·1<br>(24·1-38·7)	33·6<br>(27·2-40·9)	33·8<br>(27·4-41·2)	38·1<br>(26·9-50·2)
Togo	39·1<br>(30·6-49·7)	38·8<br>(31·8-46·2)	39·1<br>(32·1-45·9)	40·9<br>(26·2-54·8)	30·3<br>(20·7-40·6)	28·6<br>(20·0-37·7)	28·4<br>(19·9-37·3)	29·8<br>(16·2-44·4)	80·2<br>(74·5-86·0)	80·1<br>(71·3-88·5)	80·1<br>(71·1-88·5)	78·8<br>(68·6-88·5)	29·4<br>(23·8-35·8)	32·1<br>(26·5-38·0)	32·2<br>(26·6-38·4)	35·4<br>(24·8-46·8)
**Eastern Sub-Saharan Africa**																
Burundi	20·8<br>(12·2-28·4)	38·8<br>(29·1-49·3)	39·2<br>(29·4-49·5)	44·4<br>(26·6-63·5)	17·3<br>(6·0-29·9)	30·7<br>(20·8-40·8)	30·7<br>(20·3-41·1)	39·0<br>(22·4-53·1)	23·9<br>(8·9-36·7)	28·5<br>(12·4-43·1)	29·2<br>(13·2-43·9)	38·6<br>(23·6-52·6)	11·7<br>(4·3-18·5)	17·9<br>(10·3-26·1)	18·2<br>(10·6-26·6)	21·6<br>(8·1-36·1)
Comoros	37·4<br>(30·9-44·8)	49·5<br>(42·1-57·8)	49·8<br>(42·0-58·4)	54·3<br>(39·6-70·8)	58·5<br>(35·4-75·5)	53·9<br>(24·8-70·2)	53·9<br>(24·4-70·1)	53·5<br>(18·7-76·3)	98·8<br>(98·1-99·5)	98·1<br>(96·4-99·4)	98·1<br>(96·2-99·4)	97·4<br>(93·8-99·4)	34·8<br>(29·7-39·8)	36·6<br>(28·2-45·1)	37·1<br>(28·5-45·5)	44·2<br>(32·0-56·4)
Djibouti	47·7<br>(37·4-59·9)	51·2<br>(39·6-65·5)	51·8<br>(40·2-66·3)	58·0<br>(39·3-80·4)	64·1<br>(42·0-84·4)	63·8<br>(39·0-82·4)	63·9<br>(39·2-82·9)	64·4<br>(31·0-89·6)	94·3<br>(91·2-96·7)	93·1<br>(87·3-97·0)	93·0<br>(87·0-97·0)	91·8<br>(81·5-97·5)	34·0<br>(26·8-41·8)	39·6<br>(30·9-48·6)	40·1<br>(31·3-49·5)	46·4<br>(31·6-61·5)
Eritrea	33·7<br>(26·7-42·0)	37·9<br>(29·9-46·2)	38·3<br>(30·1-46·5)	42·2<br>(28·0-55·0)	32·9<br>(23·9-42·7)	35·9<br>(27·9-43·1)	35·8<br>(27·9-43·3)	35·3<br>(20·6-49·2)	82·3<br>(72·9-89·5)	87·4<br>(76·9-94·3)	87·2<br>(76·1-94·3)	84·1<br>(63·7-94·9)	26·2<br>(20·3-31·9)	30·5<br>(24·3-37·3)	30·7<br>(24·5-37·7)	34·0<br>(23·4-45·6)
Ethiopia	26·9<br>(20·4-33·2)	41·7<br>(32·9-51·3)	42·3<br>(33·6-52·1)	51·1<br>(34·3-68·3)	26·8<br>(20·0-33·9)	36·0<br>(27·6-44·2)	36·3<br>(27·7-44·7)	42·4<br>(24·4-58·7)	82·6<br>(78·2-86·7)	81·1<br>(70·9-88·7)	81·0<br>(70·0-89·0)	79·0<br>(56·0-91·9)	20·2<br>(15·5-25·1)	30·5<br>(23·7-37·4)	30·7<br>(23·7-38·0)	36·9<br>(23·3-51·4)
Kenya	65·1<br>(56·6-75·4)	72·0<br>(63·9-81·4)	72·4<br>(64·3-81·6)	77·2<br>(66·2-89·0)	45·5<br>(34·4-58·1)	50·3<br>(41·9-59·8)	50·2<br>(41·8-59·4)	53·1<br>(41·8-63·4)	78·8<br>(74·5-83·4)	76·7<br>(72·4-81·2)	76·5<br>(72·3-81·1)	74·2<br>(69·7-79·1)	42·4<br>(35·6-50·5)	46·2<br>(39·5-53·6)	46·1<br>(39·6-53·4)	50·5<br>(42·1-59·5)
Madagascar	21·3<br>(15·1-27·3)	27·3<br>(16·6-39·5)	27·7<br>(16·6-40·1)	32·1<br>(9·2-55·5)	40·9<br>(33·5-48·1)	43·5<br>(34·1-52·7)	43·6<br>(33·9-52·8)	44·9<br>(26·8-63·5)	83·8<br>(77·2-89·4)	83·9<br>(72·4-91·6)	83·3<br>(71·1-91·5)	72·7<br>(42·8-89·1)	35·2<br>(30·5-39·8)	39·8<br>(32·6-48·0)	40·1<br>(32·7-48·6)	43·1<br>(27·2-58·8)
Malawi	32·9<br>(15·5-58·3)	40·8<br>(28·8-54·7)	41·2<br>(29·5-55·5)	49·8<br>(23·6-76·7)	33·2<br>(20·8-48·5)	37·2<br>(28·1-46·5)	37·3<br>(28·0-47·0)	39·8<br>(19·7-59·8)	83·3<br>(75·7-89·7)	79·6<br>(65·3-90·3)	79·6<br>(64·7-90·6)	79·2<br>(56·5-93·6)	24·9<br>(15·6-38·2)	33·2<br>(25·7-41·5)	33·7<br>(26·1-42·1)	41·2<br>(24·5-58·4)
Mozambique	40·1<br>(27·0-56·0)	47·2<br>(35·5-58·2)	47·1<br>(35·8-57·8)	50·2<br>(26·6-73·2)	36·0<br>(26·8-47·4)	37·1<br>(27·9-46·7)	36·9<br>(27·5-46·2)	35·9<br>(17·2-53·7)	85·0<br>(78·2-90·5)	80·8<br>(66·6-90·0)	80·6<br>(65·6-90·1)	77·4<br>(50·2-91·3)	22·6<br>(14·3-32·4)	29·5<br>(21·5-37·6)	29·7<br>(21·8-37·9)	34·2<br>(19·3-48·5)
Rwanda	33·2<br>(25·0-41·3)	57·5<br>(48·5-66·3)	57·3<br>(48·5-66·3)	65·8<br>(44·1-86·3)	21·0<br>(10·5-31·2)	40·2<br>(29·9-50·0)	40·5<br>(30·3-50·1)	52·5<br>(34·2-69·8)	37·5<br>(12·6-59·3)	43·1<br>(5·4-72·5)	43·6<br>(5·4-73·4)	49·9<br>(2·1-82·7)	14·0<br>(4·5-21·3)	27·4<br>(16·1-37·7)	27·6<br>(16·7-37·7)	31·9<br>(14·6-48·5)
Somalia	21·0<br>(10·8-33·1)	26·5<br>(16·3-36·5)	26·7<br>(16·5-36·6)	31·4<br>(16·6-45·2)	47·7<br>(31·6-63·0)	49·2<br>(31·0-64·2)	48·9<br>(30·9-64·0)	49·6<br>(26·9-69·2)	98·1<br>(96·3-99·3)	97·4<br>(94·3-99·4)	97·4<br>(94·2-99·4)	96·7<br>(92·5-99·3)	22·7<br>(12·9-31·8)	25·2<br>(14·2-35·2)	25·1<br>(14·1-35·2)	28·7<br>(14·3-42·5)
South Sudan	45·9<br>(31·1-62·6)	48·9<br>(35·8-64·6)	48·4<br>(35·4-63·4)	45·4<br>(26·1-68·3)	63·5<br>(41·9-86·1)	63·5<br>(41·5-84·3)	63·1<br>(41·3-83·7)	58·5<br>(34·1-81·1)	96·5<br>(92·1-99·1)	96·0<br>(90·5-98·8)	96·0<br>(90·4-98·8)	95·4<br>(89·6-98·4)	36·1<br>(26·7-46·1)	38·0<br>(28·7-47·4)	37·9<br>(28·8-47·2)	35·7<br>(22·0-50·0)
Tanzania	40·2<br>(29·9-54·4)	46·3<br>(38·0-57·0)	46·7<br>(38·1-56·9)	50·8<br>(33·8-68·0)	38·8<br>(31·0-48·0)	40·8<br>(31·5-48·5)	40·8<br>(31·5-48·5)	40·5<br>(24·2-56·0)	47·4<br>(32·2-63·6)	52·4<br>(31·5-74·9)	53·1<br>(32·1-75·7)	62·0<br>(40·2-84·6)	30·7<br>(24·5-38·4)	34·3<br>(28·1-40·7)	34·6<br>(28·3-41·0)	37·9<br>(25·0-49·7)
Uganda	32·9<br>(25·5-41·0)	43·2<br>(34·2-52·3)	43·7<br>(34·8-53·3)	51·3<br>(35·0-69·5)	3·7<br>(0·0-13·5)	15·8<br>(6·3-25·5)	16·7<br>(7·4-26·0)	30·2<br>(14·0-45·2)	38·5<br>(18·1-58·1)	52·3<br>(21·8-77·4)	53·6<br>(22·9-78·7)	68·7<br>(37·0-90·3)	23·7<br>(18·0-29·6)	25·1<br>(18·4-31·7)	25·4<br>(18·5-32·1)	28·0<br>(15·1-39·9)
Zambia	20·0<br>(10·1-32·9)	33·4<br>(19·1-47·5)	34·2<br>(19·8-49·0)	52·6<br>(22·8-86·1)	23·5<br>(14·6-34·3)	23·0<br>(12·8-33·6)	23·0<br>(12·3-34·1)	27·3<br>(5·8-50·9)	69·0<br>(56·9-79·7)	70·3<br>(51·8-85·7)	70·3<br>(51·2-86·2)	70·7<br>(41·0-92·0)	17·9<br>(11·0-26·5)	22·5<br>(13·9-31·1)	23·0<br>(14·4-31·9)	31·5<br>(13·2-51·3)
**Central Sub-Saharan Africa**																
Angola	34·8<br>(19·8-51·3)	45·9<br>(28·2-61·6)	46·5<br>(28·8-62·1)	57·6<br>(31·1-84·4)	39·9<br>(27·9-53·1)	43·2<br>(30·3-53·9)	43·9<br>(30·8-55·0)	50·8<br>(30·1-70·1)	86·8<br>(81·4-91·4)	66·5<br>(47·1-82·5)	65·7<br>(45·6-82·4)	53·6<br>(21·5-80·7)	16·7<br>(7·2-25·5)	24·6<br>(13·4-35·1)	26·2<br>(15·2-36·8)	42·3<br>(23·0-61·9)
Central African Republic	19·1<br>(6·4-31·7)	22·7<br>(10·9-36·8)	22·7<br>(10·8-36·8)	26·0<br>(4·3-49·7)	30·5<br>(16·7-49·7)	30·9<br>(16·0-50·5)	30·9<br>(15·9-50·1)	32·3<br>(10·1-55·9)	63·0<br>(48·4-76·3)	65·2<br>(45·4-82·8)	65·2<br>(45·1-83·1)	66·1<br>(42·3-87·0)	4·8<br>(0·0-13·2)	6·2<br>(0·0-15·0)	6·8<br>(0·0-15·7)	13·4<br>(0·0-30·1)
Congo	22·2<br>(13·7-31·4)	41·3<br>(29·9-53·5)	41·7<br>(29·7-54·3)	51·5<br>(28·8-77·3)	33·4<br>(21·1-46·5)	43·7<br>(29·4-57·5)	43·4<br>(28·4-57·3)	44·1<br>(20·2-66·9)	68·2<br>(58·4-77·1)	61·3<br>(42·7-77·7)	60·5<br>(41·2-77·6)	48·4<br>(16·8-76·8)	14·7<br>(8·5-21·2)	28·7<br>(20·1-38·4)	29·0<br>(20·1-39·3)	36·3<br>(17·2-55·7)
Democratic Republic of the Congo	45·0<br>(37·9-52·1)	47·4<br>(40·9-53·5)	47·9<br>(41·5-54·1)	53·8<br>(44·3-63·9)	44·1<br>(34·3-57·2)	43·1<br>(32·3-55·6)	43·1<br>(32·3-55·7)	42·3<br>(27·2-57·4)	83·1<br>(76·5-88·9)	77·0<br>(59·6-89·5)	76·3<br>(57·9-89·5)	64·4<br>(27·1-88·8)	26·4<br>(19·9-34·1)	30·8<br>(25·0-37·6)	31·3<br>(25·3-38·1)	35·3<br>(24·1-46·3)
Equatorial Guinea	38·3<br>(20·4-60·1)	67·7<br>(40·4-96·3)	67·5<br>(40·6-96·5)	85·5<br>(47·7-100·0)	35·2<br>(20·5-52·0)	49·4<br>(29·8-70·2)	49·0<br>(29·4-70·3)	62·2<br>(30·1-97·6)	64·5<br>(54·1-73·9)	44·9<br>(27·0-64·1)	45·0<br>(27·0-64·4)	47·2<br>(27·4-68·5)	12·9<br>(2·0-25·1)	37·0<br>(19·9-54·6)	37·3<br>(20·2-55·1)	55·1<br>(26·6-84·7)
Gabon	32·2<br>(23·6-40·6)	50·5<br>(39·2-61·7)	51·6<br>(39·9-63·6)	67·3<br>(43·3-91·7)	36·1<br>(25·7-47·1)	42·7<br>(28·8-55·4)	43·1<br>(29·2-56·3)	49·3<br>(23·6-73·9)	55·1<br>(46·3-63·8)	53·6<br>(36·2-70·7)	53·5<br>(35·7-70·9)	51·4<br>(29·4-74·1)	15·0<br>(7·8-21·9)	26·1<br>(16·6-35·8)	27·0<br>(17·1-36·8)	38·1<br>(17·9-59·3)

Location	Indicator 3.7.1: Proportion of women of reproductive age (15-49 years) who have their need for family planning satisfied with modern contraception methods (%)				Indicator 3.7.2: Number of livebirths per 1,000 women aged 10-14 years and women aged 15-19 years				Indicator 3.8.1: Coverage of essential health services, as defined by the UHC index comprised of the coverage of 9 tracer interventions and risk-standardised death rates from 32 causes amenable to personal healthcare (scale of 0 to 100)				Indicator 3.9.1: Age-standardised death rate attributable to household air pollution and ambient air pollution (per 100,000 population)			
	**2000**	**2015**	**2016**	**2030**	**2000**	**2015**	**2016**	**2030**	**2000**	**2015**	**2016**	**2030**	**2000**	**2015**	**2016**	**2030**
Sierra Leone	3·7 (0·0-8·6)	40·0 (33·8-46·6)	39·8 (32·7-46·9)	71·5 (56·7-83·4)	3·7 (2·9-4·5)	6·5 (5·7-7·4)	6·7 (5·9-7·6)	10·3 (9·3-11·5)	9·9 (6·1-13·6)	26·3 (22·2-30·7)	27·6 (23·3-32·1)	44·4 (39·6-49·7)	3·8 (0·0-8·1)	6·3 (2·3-10·4)	6·4 (2·5-10·5)	8·7 (5·0-12·7)
Togo	23·5 (18·5-28·7)	33·8 (28·5-39·6)	34·8 (28·5-41·4)	52·8 (35·2-72·5)	14·9 (11·3-19·1)	15·7 (11·5-20·4)	15·4 (11·1-20·5)	16·8 (6·3-28·0)	14·1 (9·8-19·1)	28·8 (25·0-32·6)	29·9 (26·1-33·7)	44·7 (40·0-49·9)	4·8 (0·7-9·3)	7·7 (4·3-11·5)	7·7 (4·2-11·4)	8·1 (4·6-12·1)
**Eastern Sub-Saharan Africa**																
Burundi	11·3 (5·9-16·9)	28·3 (20·7-36·6)	28·4 (20·2-37·3)	39·5 (20·1-60·8)	37·2 (35·8-38·5)	44·6 (42·7-46·5)	45·4 (43·5-47·4)	55·1 (51·7-58·5)	2·9 (0·0-7·4)	25·8 (20·9-31·5)	26·1 (21·2-31·9)	35·3 (27·7-43·1)	0·5 (0·0-3·3)	10·0 (6·1-14·6)	10·3 (6·3-14·9)	13·7 (9·0-21·9)
Comoros	24·9 (18·8-31·6)	26·6 (20·5-33·0)	28·1 (21·2-35·2)	33·4 (19·2-51·0)	16·2 (15·3-17·2)	29·4 (28·1-30·6)	30·4 (29·2-31·7)	42·9 (40·3-45·6)	15·1 (10·2-19·4)	28·9 (23·6-34·0)	29·2 (24·1-34·5)	39·1 (31·1-47·4)	12·7 (9·4-16·4)	25·0 (20·1-31·0)	25·4 (20·4-31·5)	32·0 (25·4-40·3)
Djibouti	14·5 (9·2-20·3)	41·6 (30·9-52·2)	42·4 (32·3-52·9)	62·7 (42·8-80·7)	31·3 (28·6-34·9)	37·2 (32·4-42·7)	37·8 (32·8-43·2)	46·7 (36·6-58·4)	15·5 (8·1-22·8)	30·5 (23·1-38·8)	31·5 (24·2-39·9)	45·5 (37·0-54·9)	24·7 (17·1-33·3)	33·0 (21·0-44·4)	33·2 (20·9-44·9)	38·7 (21·6-54·3)
Eritrea	3·4 (0·7-6·0)	19·2 (11·0-27·9)	20·1 (11·7-29·0)	38·7 (21·0-57·1)	14·7 (12·7-17·1)	30·6 (27·1-34·6)	31·9 (28·3-36·2)	50·9 (41·5-61·7)	8·1 (3·3-12·5)	18·4 (13·2-23·8)	19·2 (13·8-24·7)	29·9 (22·2-38·0)	11·2 (7·0-16·0)	20·5 (11·3-32·9)	21·0 (11·5-33·8)	29·2 (15·9-46·5)
Ethiopia	5·8 (2·5-9·5)	60·6 (57·7-63·7)	63·7 (60·3-67·3)	95·6 (88·8-100·0)	13·1 (11·9-14·4)	24·2 (22·9-25·8)	25·0 (23·5-26·6)	35·7 (32·7-38·8)	0·0 (0·0-0·0)	19·1 (13·6-24·4)	20·5 (14·9-25·9)	46·4 (38·3-55·2)	3·6 (0·7-6·4)	14·6 (10·6-18·9)	15·1 (10·9-19·5)	23·2 (17·5-29·4)
Kenya	65·2 (63·5-67·0)	76·2 (75·1-77·3)	76·7 (75·5-77·8)	85·1 (83·1-87·0)	10·8 (9·2-12·3)	17·8 (16·1-19·7)	18·1 (16·2-20·4)	27·0 (22·3-32·4)	33·5 (29·0-39·1)	46·6 (42·1-51·8)	47·5 (42·9-52·9)	60·6 (54·7-66·4)	25·4 (20·1-32·6)	32·4 (27·5-39·4)	32·9 (28·1-39·8)	39·6 (34·8-45·1)
Madagascar	17·6 (13·6-22·1)	44·4 (35·9-52·7)	44·2 (35·5-52·7)	79·6 (63·1-93·3)	1·3 (0·3-2·9)	6·5 (5·0-8·3)	6·8 (5·2-8·8)	12·9 (8·5-17·7)	10·1 (6·0-14·1)	17·4 (11·3-23·6)	18·5 (12·0-24·8)	26·7 (16·9-36·7)	4·2 (1·3-7·1)	9·2 (4·1-14·5)	9·4 (4·3-14·8)	13·0 (6·5-20·1)
Malawi	37·6 (33·9-41·8)	77·9 (74·6-81·0)	78·0 (74·4-81·7)	99·6 (96·9-100·0)	0·4 (0·0-1·4)	2·4 (1·2-3·6)	2·8 (1·4-4·1)	5·8 (3·2-8·6)	17·4 (9·3-27·7)	35·5 (29·6-42·5)	36·6 (30·7-43·5)	53·7 (47·5-59·6)	6·6 (0·0-15·4)	17·8 (12·5-24·3)	18·4 (13·1-24·9)	27·0 (21·7-32·6)
Mozambique	18·0 (13·9-22·7)	33·9 (27·2-41·4)	34·9 (27·3-42·7)	48·9 (31·5-66·5)	0·0 (0·0-0·0)	3·9 (1·8-6·6)	4·8 (2·6-7·9)	11·7 (5·4-19·0)	14·9 (8·2-21·7)	29·8 (23·8-35·8)	30·9 (25·1-36·7)	46·5 (40·1-52·7)	12·4 (6·8-18·2)	21·2 (15·5-26·3)	21·5 (16·0-26·6)	27·1 (22·6-31·7)
Rwanda	2·5 (0·0-6·8)	62·5 (55·6-69·4)	62·0 (54·6-69·5)	91·8 (76·8-100·0)	28·4 (26·5-30·5)	44·4 (41·8-47·0)	45·4 (42·6-48·3)	63·1 (54·3-72·1)	5·6 (1·1-10·1)	40·6 (35·6-45·4)	41·5 (36·5-46·5)	58·5 (52·4-64·2)	3·8 (0·0-8·0)	20·5 (16·2-25·5)	20·9 (16·6-25·9)	26·7 (21·2-34·9)
Somalia	0·0 (0·0-0·0)	1·0 (0·0-4·9)	1·3 (0·0-5·5)	8·0 (0·0-23·3)	17·9 (13·6-23·2)	23·1 (17·7-29·6)	23·5 (18·2-30·1)	30·0 (17·7-43·7)	0·0 (0·0-0·0)	0·1 (0·0-0·7)	0·1 (0·0-1·7)	5·3 (0·0-12·2)	1·4 (0·0-5·5)	5·8 (1·1-10·3)	6·0 (1·2-10·5)	9·0 (4·0-13·6)
South Sudan	0·0 (0·0-0·0)	0·0 (0·0-0·0)	0·0 (0·0-0·0)	0·0 (0·0-0·0)	1·6 (0·2-3·8)	13·3 (10·8-16·3)	14·8 (12·4-17·9)	26·0 (19·8-32·8)	4·2 (0·0-12·0)	11·7 (4·2-19·3)	12·1 (4·5-19·8)	18·4 (8·6-28·1)	8·2 (1·6-15·4)	12·3 (6·7-18·7)	12·2 (6·7-18·6)	12·1 (7·1-19·9)
Tanzania	33·4 (30·1-37·1)	54·7 (51·4-58·0)	55·3 (51·7-59·0)	74·7 (66·3-82·2)	4·5 (2·5-6·6)	9·8 (7·2-13·3)	10·1 (7·3-13·9)	15·4 (8·4-22·9)	21·3 (16·5-27·2)	34·5 (29·7-39·5)	35·9 (31·1-40·9)	48·4 (42·2-54·5)	10·6 (6·6-15·8)	18·6 (14·5-23·7)	19·1 (14·9-24·2)	26·0 (21·3-31·7)
Uganda	21·9 (19·0-24·8)	44·7 (42·3-47·0)	46·6 (43·8-49·3)	72·3 (65·1-79·0)	0·0 (0·0-0·0)	10·3 (9·4-11·3)	11·5 (10·6-12·5)	22·5 (20·7-24·7)	9·7 (5·5-13·6)	26·4 (21·6-31·0)	27·0 (22·2-31·8)	42·4 (35·9-48·6)	6·8 (3·2-10·4)	15·2 (11·0-19·4)	15·6 (11·3-19·8)	21·0 (15·9-25·9)
Zambia	32·2 (28·7-35·9)	63·3 (57·8-68·5)	63·5 (57·1-69·7)	83·6 (74·1-91·9)	4·0 (3·1-5·0)	16·3 (15·2-17·8)	17·7 (16·5-19·1)	28·9 (26·2-32·2)	10·1 (4·9-16·7)	27·7 (20·7-34·9)	29·3 (22·2-36·7)	52·4 (43·0-62·3)	3·1 (0·0-8·7)	12·4 (6·5-18·6)	13·2 (7·0-19·4)	23·6 (15·8-31·5)
**Central Sub-Saharan Africa**																
Angola	1·1 (0·0-4·7)	22·9 (20·2-26·1)	23·5 (20·2-27·2)	56·6 (41·8-72·1)	0·0 (0·0-0·0)	1·2 (0·0-3·7)	1·6 (0·0-4·4)	7·2 (2·0-13·0)	3·5 (0·0-10·5)	27·3 (18·1-35·2)	29·3 (20·2-37·2)	56·0 (47·1-64·4)	7·0 (0·7-13·7)	23·8 (14·1-33·7)	25·0 (14·9-35·4)	42·2 (26·3-60·2)
Central African Republic	3·9 (0·2-8·0)	11·6 (6·1-17·8)	11·6 (5·9-18·1)	21·5 (6·2-38·8)	7·7 (6·7-8·7)	11·6 (10·6-12·7)	11·8 (10·9-12·9)	15·4 (14·3-16·6)	0·3 (0·0-3·6)	2·5 (0·0-9·1)	2·6 (0·0-9·3)	4·5 (0·0-13·0)	0·2 (0·0-2·5)	1·1 (0·0-5·7)	1·2 (0·0-5·8)	2·1 (0·0-7·6)
Congo	12·1 (6·2-18·0)	37·6 (30·6-44·7)	38·0 (30·1-45·7)	55·4 (37·7-73·2)	3·6 (1·8-6·0)	9·2 (7·2-11·7)	9·7 (7·5-12·4)	16·0 (10·4-22·2)	9·3 (4·4-14·8)	31·5 (25·5-38·2)	32·5 (26·4-39·3)	48·5 (40·2-56·6)	2·9 (0·0-7·0)	18·6 (11·6-26·7)	19·5 (12·2-27·9)	31·9 (20·3-44·6)
Democratic Republic of the Congo	1·5 (0·0-5·5)	17·8 (14·0-21·4)	18·8 (14·2-23·4)	34·9 (21·6-48·8)	9·6 (2·1-22·8)	7·5 (0·3-19·3)	7·3 (0·0-19·9)	8·8 (0·0-33·8)	7·2 (3·0-11·6)	25·3 (21·2-29·6)	26·3 (22·2-30·5)	42·0 (37·4-46·7)	8·0 (3·9-11·6)	12·1 (8·8-15·7)	12·4 (9·1-16·0)	16·8 (13·7-20·5)
Equatorial Guinea	6·5 (1·6-12·5)	28·3 (18·8-37·9)	28·9 (19·3-38·6)	50·9 (29·1-70·0)	13·5 (10·7-16·8)	16·5 (12·7-20·0)	16·8 (13·1-20·6)	21·3 (12·9-30·4)	11·5 (1·9-21·9)	40·5 (28·4-54·4)	42·5 (30·2-56·5)	66·3 (53·6-80·7)	8·9 (1·1-18·5)	30·7 (17·8-45·2)	31·4 (18·4-46·1)	43·7 (28·1-61·6)
Gabon	17·4 (14·5-20·5)	43·2 (37·3-49·5)	43·8 (36·8-50·9)	64·6 (48·3-78·9)	2·9 (0·0-7·1)	10·8 (5·6-17·0)	11·7 (6·5-18·2)	19·6 (7·2-33·9)	14·5 (9·7-19·1)	36·6 (30·4-43·0)	37·9 (31·5-44·4)	59·2 (50·7-67·2)	21·8 (15·7-28·0)	39·2 (31·3-46·5)	39·7 (31·7-47·3)	49·7 (38·7-60·0)

Location	Indicator 3.9.2: Age-standardised death rate attributable to unsafe water, sanitation, and hygiene (WaSH) (per 100,000 population)				Indicator 3.9.3: Age-standardised death rate due to unintentional poisonings (per 100,000 population)				Indicator 3.a.1: Age-standardised prevalence of daily smoking in populations aged 10 and older (%)				Indicator 3.b.1: Geometric mean of the coverage of eight vaccines, conditional on inclusion in national vaccine schedules, in target populations (%)			
	**2000**	**2015**	**2016**	**2030**	**2000**	**2015**	**2016**	**2030**	**2000**	**2015**	**2016**	**2030**	**2000**	**2015**	**2016**	**2030**
Sierra Leone	1·0<br>(0·0-3·5)	4·6<br>(2·0-7·7)	5·1<br>(2·4-8·2)	7·9<br>(2·5-12·9)	10·9<br>(0·0-20·5)	17·9<br>(8·9-28·0)	18·4<br>(9·8-28·6)	26·9<br>(8·9-42·2)	57·2<br>(49·7-64·4)	61·5<br>(55·9-67·2)	62·3<br>(56·5-68·2)	63·2<br>(50·9-74·3)	25·3<br>(18·8-31·2)	71·1<br>(63·2-77·6)	72·5<br>(63·8-79·8)	89·9<br>(77·8-96·3)
Togo	6·7<br>(3·7-10·0)	11·6<br>(7·6-15·8)	12·0<br>(8·0-16·2)	16·6<br>(11·4-21·6)	13·3<br>(6·6-20·5)	21·8<br>(15·4-31·9)	22·6<br>(15·9-33·1)	31·1<br>(17·1-46·0)	92·1<br>(88·3-95·6)	93·9<br>(91·3-96·2)	93·9<br>(91·0-96·5)	95·4<br>(90·3-99·7)	39·4<br>(34·9-43·7)	73·6<br>(66·8-79·4)	75·5<br>(68·1-81·6)	91·7<br>(81·6-97·6)
**Eastern Sub-Saharan Africa**																
Burundi	0·6<br>(0·0-3·0)	3·3<br>(0·0-7·5)	3·6<br>(0·2-7·9)	8·1<br>(4·0-13·5)	1·0<br>(0·0-7·9)	9·5<br>(0·0-18·5)	9·7<br>(0·0-18·9)	14·4<br>(0·0-30·8)	88·2<br>(82·4-93·2)	90·2<br>(85·3-94·2)	90·1<br>(85·1-94·3)	91·5<br>(82·2-98·6)	56·3<br>(49·9-62·2)	91·2<br>(87·0-94·2)	90·5<br>(85·3-94·1)	94·5<br>(84·3-99·4)
Comoros	2·6<br>(0·4-5·5)	8·8<br>(5·5-13·7)	9·2<br>(5·8-14·1)	14·4<br>(10·2-20·4)	11·6<br>(0·8-20·3)	23·4<br>(9·1-32·6)	24·0<br>(9·8-33·3)	34·3<br>(11·3-51·2)	72·4<br>(66·8-77·7)	79·8<br>(74·9-84·5)	79·8<br>(74·4-85·1)	83·2<br>(72·5-91·8)	47·9<br>(41·5-54·6)	61·1<br>(50·7-70·0)	61·0<br>(49·7-70·9)	65·0<br>(36·8-84·3)
Djibouti	8·2<br>(2·9-12·1)	16·6<br>(10·0-21·6)	17·1<br>(10·4-22·3)	25·1<br>(15·9-32·0)	10·2<br>(0·0-19·2)	21·0<br>(9·4-29·8)	21·4<br>(9·5-30·6)	33·1<br>(11·4-51·9)	57·0<br>(51·1-62·8)	53·7<br>(44·8-62·2)	55·0<br>(46·0-63·7)	58·7<br>(41·9-73·1)	25·0<br>(18·1-32·2)	60·5<br>(49·9-71·0)	60·2<br>(47·6-71·8)	71·0<br>(36·9-91·3)
Eritrea	2·5<br>(0·0-5·6)	7·1<br>(3·0-10·6)	7·5<br>(3·3-11·0)	12·8<br>(8·0-16·8)	5·4<br>(0·0-11·2)	10·5<br>(2·0-22·4)	11·1<br>(1·8-23·3)	17·2<br>(0·0-37·7)	89·1<br>(84·3-93·1)	96·0<br>(92·2-99·1)	95·6<br>(91·4-99·1)	97·6<br>(90·8-100·0)	60·8<br>(56·0-65·2)	95·1<br>(92·1-97·1)	95·5<br>(92·4-97·5)	99·1<br>(96·9-100·0)
Ethiopia	1·0<br>(0·0-2·7)	8·3<br>(5·9-11·0)	8·9<br>(6·4-11·6)	15·8<br>(12·3-20·2)	4·2<br>(0·0-10·0)	19·3<br>(12·0-25·1)	19·9<br>(12·4-25·9)	33·0<br>(16·9-46·3)	98·3<br>(95·5-100·0)	97·9<br>(94·9-100·0)	97·8<br>(94·3-100·0)	98·2<br>(92·2-100·0)	18·0<br>(7·0-28·6)	38·7<br>(20·7-54·5)	38·3<br>(17·7-56·4)	68·4<br>(32·1-92·7)
Kenya	0·0<br>(0·0-0·0)	0·2<br>(0·0-2·4)	0·4<br>(0·0-2·8)	3·4<br>(0·0-8·2)	13·7<br>(2·2-21·1)	27·0<br>(15·2-34·4)	27·4<br>(15·2-34·5)	36·3<br>(18·8-45·1)	77·5<br>(75·8-79·1)	83·7<br>(82·5-85·1)	83·8<br>(82·6-85·1)	88·2<br>(86·0-90·3)	73·4<br>(68·1-78·0)	88·7<br>(84·4-92·2)	89·4<br>(84·9-93·1)	95·8<br>(88·9-99·1)
Madagascar	4·4<br>(1·8-6·9)	7·6<br>(4·2-10·8)	7·9<br>(4·3-11·1)	11·7<br>(7·1-16·1)	11·6<br>(4·4-16·8)	19·2<br>(9·8-27·0)	19·7<br>(9·9-27·6)	27·3<br>(8·4-43·9)	69·9<br>(64·2-75·1)	74·2<br>(66·6-81·0)	74·4<br>(66·6-81·3)	78·1<br>(65·0-88·7)	47·1<br>(41·3-52·5)	69·2<br>(61·0-75·7)	70·9<br>(61·9-78·0)	83·6<br>(66·6-92·7)
Malawi	1·1<br>(0·0-4·0)	7·0<br>(4·1-10·1)	7·5<br>(4·5-10·6)	13·3<br>(9·7-17·0)	3·3<br>(0·0-12·6)	16·0<br>(5·3-24·0)	16·8<br>(6·3-24·8)	28·7<br>(7·6-44·5)	70·3<br>(66·1-74·2)	73·1<br>(67·0-78·8)	73·1<br>(66·3-79·0)	73·6<br>(61·5-84·0)	83·5<br>(80·7-85·9)	89·7<br>(86·5-92·3)	90·7<br>(87·3-93·3)	96·6<br>(91·4-99·2)
Mozambique	8·5<br>(4·2-11·9)	15·7<br>(9·9-20·4)	16·2<br>(10·3-21·0)	23·5<br>(16·0-29·6)	5·0<br>(0·0-11·7)	15·4<br>(5·5-23·0)	16·1<br>(6·0-23·8)	25·8<br>(5·1-40·8)	76·9<br>(71·0-82·2)	74·7<br>(67·7-80·9)	75·1<br>(67·8-81·4)	73·1<br>(57·7-85·1)	65·8<br>(61·1-70·1)	72·6<br>(65·6-78·2)	74·0<br>(65·9-80·4)	85·3<br>(68·7-93·8)
Rwanda	5·8<br>(3·1-8·6)	13·7<br>(9·7-18·9)	14·1<br>(10·0-19·5)	20·5<br>(14·6-27·5)	2·3<br>(0·0-10·0)	20·1<br>(7·9-29·6)	20·5<br>(8·3-30·0)	32·8<br>(8·1-51·2)	88·3<br>(82·6-93·6)	90·8<br>(88·0-93·4)	91·0<br>(87·9-93·9)	92·5<br>(86·1-97·5)	65·9<br>(59·3-71·8)	84·1<br>(75·7-90·3)	84·6<br>(75·6-90·9)	83·1<br>(55·2-96·1)
Somalia	3·1<br>(0·0-7·3)	6·0<br>(0·0-10·6)	6·3<br>(0·0-11·0)	9·5<br>(3·0-14·8)	0·6<br>(0·0-5·5)	4·7<br>(0·0-12·1)	5·0<br>(0·0-12·5)	11·6<br>(0·0-26·0)	78·7<br>(71·6-85·0)	83·3<br>(76·9-89·5)	83·4<br>(76·9-89·5)	86·9<br>(75·8-96·0)	0·0<br>(0·0-0·0)	0·2<br>(0·0-2·9)	0·2<br>(0·0-3·6)	6·9<br>(0·0-31·9)
South Sudan	1·8<br>(0·0-5·4)	4·8<br>(1·4-8·1)	4·9<br>(1·6-8·2)	6·7<br>(3·5-9·9)	6·4<br>(0·0-17·1)	9·6<br>(0·0-20·2)	9·7<br>(0·0-20·2)	12·8<br>(0·0-29·5)	82·1<br>(75·3-87·9)	85·5<br>(79·1-91·5)	85·6<br>(79·4-91·5)	87·8<br>(76·7-96·8)	1·0<br>(0·0-10·3)	23·3<br>(14·0-33·6)	24·5<br>(13·6-36·6)	41·9<br>(10·1-68·0)
Tanzania	3·1<br>(0·8-5·4)	9·1<br>(5·8-12·9)	9·7<br>(6·2-13·4)	16·7<br>(12·4-21·3)	9·2<br>(3·3-15·2)	16·7<br>(3·4-25·1)	17·3<br>(3·4-25·6)	26·8<br>(2·5-43·6)	73·2<br>(66·5-79·6)	79·2<br>(74·2-84·2)	79·3<br>(73·7-84·4)	81·9<br>(70·4-91·2)	78·4<br>(74·7-81·4)	90·4<br>(86·2-93·6)	89·7<br>(83·9-93·7)	93·3<br>(79·3-99·3)
Uganda	7·1<br>(4·1-10·4)	12·6<br>(8·8-17·3)	12·8<br>(9·0-17·5)	16·0<br>(11·5-22·0)	5·7<br>(0·0-13·2)	14·9<br>(4·8-23·1)	15·4<br>(5·0-23·7)	24·0<br>(5·7-39·0)	77·1<br>(72·4-81·0)	80·8<br>(77·5-83·9)	81·0<br>(77·0-84·6)	81·4<br>(73·3-88·6)	46·7<br>(40·4-52·6)	69·8<br>(60·2-77·9)	68·6<br>(56·6-78·7)	83·6<br>(60·6-95·9)
Zambia	0·0<br>(0·0-0·0)	7·0<br>(3·0-10·5)	7·9<br>(3·8-11·4)	18·8<br>(13·2-24·9)	0·7<br>(0·0-5·2)	9·0<br>(0·0-16·3)	10·0<br>(0·0-17·7)	24·0<br>(3·7-41·4)	75·3<br>(70·9-79·2)	68·7<br>(63·3-73·9)	69·2<br>(63·1-74·7)	71·2<br>(58·1-82·3)	81·5<br>(78·1-84·5)	82·8<br>(77·5-87·1)	84·5<br>(78·2-89·3)	87·2<br>(74·3-94·5)
**Central Sub-Saharan Africa**																
Angola	0·0<br>(0·0-0·0)	8·5<br>(4·9-11·7)	9·2<br>(5·5-12·6)	20·1<br>(14·9-24·8)	7·7<br>(0·0-15·6)	20·1<br>(9·8-29·1)	21·5<br>(11·3-30·8)	38·4<br>(17·8-56·1)	88·7<br>(84·4-92·7)	81·5<br>(76·1-86·7)	81·7<br>(76·2-86·7)	74·5<br>(61·5-85·4)	7·3<br>(0·0-15·8)	65·9<br>(55·2-75·3)	67·5<br>(54·6-78·0)	91·9<br>(78·0-98·4)
Central African Republic	1·5<br>(0·0-5·5)	1·2<br>(0·0-4·6)	1·2<br>(0·0-4·8)	1·8<br>(0·0-6·0)	4·0<br>(0·0-14·9)	6·6<br>(0·0-21·7)	6·9<br>(0·0-22·1)	11·5<br>(0·0-32·4)	91·6<br>(87·4-95·3)	86·0<br>(80·9-90·4)	86·0<br>(81·1-90·5)	80·7<br>(68·7-90·4)	14·7<br>(8·5-20·7)	22·0<br>(10·4-33·2)	20·2<br>(7·2-33·1)	5·4<br>(0·0-33·1)
Congo	5·2<br>(1·0-11·0)	8·3<br>(3·1-13·2)	8·7<br>(3·5-13·7)	11·8<br>(4·5-18·0)	14·7<br>(5·2-29·5)	28·8<br>(16·2-41·8)	29·5<br>(16·5-42·6)	40·4<br>(19·0-58·6)	94·4<br>(91·5-97·0)	84·4<br>(80·6-87·9)	84·8<br>(80·6-88·6)	78·6<br>(66·6-88·6)	27·7<br>(20·6-34·9)	74·9<br>(65·0-82·5)	76·9<br>(66·5-84·9)	87·7<br>(66·6-97·8)
Democratic Republic of the Congo	5·9<br>(3·7-8·7)	9·4<br>(6·7-12·4)	9·9<br>(7·1-12·8)	13·8<br>(9·4-17·4)	15·1<br>(4·3-25·1)	20·5<br>(10·0-31·0)	21·1<br>(10·8-31·6)	26·8<br>(9·6-40·4)	83·4<br>(78·7-87·9)	81·1<br>(77·7-84·6)	81·2<br>(77·4-85·0)	79·4<br>(70·2-86·9)	0·5<br>(0·0-4·3)	66·0<br>(57·5-73·6)	68·5<br>(58·2-76·6)	90·1<br>(74·0-97·2)
Equatorial Guinea	16·0<br>(7·3-22·8)	27·1<br>(16·1-35·7)	27·6<br>(16·5-36·3)	35·5<br>(22·8-46·9)	14·9<br>(3·6-30·5)	40·5<br>(24·6-57·0)	41·3<br>(25·1-58·0)	60·7<br>(32·4-85·4)	100·0<br>(100·0-100·0)	100·0<br>(100·0-100·0)	100·0<br>(100·0-100·0)	100·0<br>(99·4-100·0)	14·2<br>(5·9-21·8)	11·2<br>(0·5-23·1)	7·7<br>(0·0-23·7)	7·1<br>(0·0-35·5)
Gabon	11·9<br>(7·2-15·7)	18·9<br>(12·4-24·0)	19·5<br>(12·9-24·7)	26·5<br>(18·2-33·6)	22·7<br>(11·8-38·1)	34·7<br>(17·8-50·1)	35·4<br>(18·3-50·6)	45·8<br>(20·2-65·2)	80·5<br>(74·9-85·6)	77·8<br>(73·7-81·7)	77·9<br>(73·6-82·1)	75·2<br>(66·0-84·0)	7·9<br>(1·6-14·2)	60·3<br>(50·9-68·1)	60·6<br>(50·1-69·6)	83·2<br>(65·3-93·6)

Location	Indicator 5.2.1: Age-standardised prevalence of women aged 15 years and older who experienced physical or sexual violence by an intimate partner in the last 12 months (%)				Indicator 6.1.1: Risk-weighted prevalence of populations using unsafe or unimproved water sources, as measured by the summary exposure value (SEV) for unsafe water (%)				Indicator 6.2.1a: Risk-weighted prevalence of populations using unsafe or unimproved sanitation, as measured by the summary exposure value (SEV) for unsafe sanitation (%)				Indicator 6.2.1b: Risk-weighted prevalence of populations without access to a handwashing facility, as measured by the summary exposure value (SEV) for unsafe hygiene (%)			
	**2000**	**2015**	**2016**	**2030**	**2000**	**2015**	**2016**	**2030**	**2000**	**2015**	**2016**	**2030**	**2000**	**2015**	**2016**	**2030**
Sierra Leone	20·1 (14·8-25·1)	27·3 (22·7-31·4)	27·8 (23·3-31·9)	35·0 (30·8-39·3)	2·1 (0·0-6·3)	3·8 (0·0-11·1)	4·1 (0·0-11·3)	14·4 (0·0-52·5)	3·4 (0·0-10·5)	12·8 (0·0-28·4)	13·3 (0·0-29·7)	23·3 (0·5-50·8)	8·2 (1·1-19·5)	6·4 (1·3-13·8)	6·4 (1·2-13·8)	4·6 (0·0-12·3)
Togo	42·0 (37·3-46·2)	43·7 (39·4-47·6)	43·9 (39·6-47·8)	45·6 (41·2-49·7)	5·8 (1·5-12·1)	4·6 (0·0-13·9)	4·8 (0·0-14·2)	13·8 (0·0-49·5)	11·2 (5·1-19·4)	19·9 (4·4-42·4)	20·9 (4·6-45·4)	32·1 (1·1-80·1)	7·1 (0·4-18·6)	7·6 (1·3-17·4)	7·7 (1·3-17·8)	8·1 (1·1-19·2)
**Eastern Sub-Saharan Africa**																
Burundi	26·7 (21·9-31·6)	27·8 (23·3-32·2)	28·1 (23·6-32·4)	29·6 (24·6-34·8)	2·5 (0·0-7·8)	3·9 (0·0-11·9)	4·0 (0·0-12·0)	6·3 (0·6-15·4)	1·3 (0·0-7·5)	9·7 (0·0-26·5)	9·8 (0·0-27·4)	21·0 (6·9-50·1)	2·6 (0·0-9·7)	4·0 (0·0-13·0)	4·2 (0·0-13·2)	6·6 (0·0-16·0)
Comoros	30·5 (26·5-34·5)	37·0 (32·9-41·1)	37·3 (33·2-41·4)	41·8 (37·7-45·6)	18·7 (11·3-29·6)	31·9 (19·1-47·4)	32·9 (19·6-48·6)	46·1 (26·0-63·5)	3·5 (0·0-11·4)	17·6 (3·8-35·8)	18·6 (4·1-37·8)	38·9 (13·3-70·1)	6·3 (0·4-15·6)	16·7 (7·1-29·3)	17·8 (7·7-30·9)	35·7 (12·9-55·4)
Djibouti	13·7 (9·6-18·0)	24·3 (20·2-28·2)	25·1 (21·0-29·1)	35·9 (31·2-40·6)	13·1 (1·7-29·5)	23·7 (6·4-46·4)	24·1 (6·2-47·1)	29·3 (3·7-57·4)	21·7 (1·4-51·1)	41·3 (9·7-74·2)	42·2 (9·5-75·6)	59·5 (17·6-92·4)	10·0 (1·0-25·1)	19·0 (5·0-38·8)	19·4 (5·2-39·5)	28·9 (10·0-53·4)
Eritrea	12·3 (7·6-16·9)	15·6 (11·4-20·4)	15·9 (11·8-20·5)	20·5 (14·3-26·1)	8·5 (1·2-20·5)	16·5 (4·3-35·8)	17·1 (4·6-36·7)	25·2 (8·5-48·5)	6·0 (0·4-12·9)	15·5 (0·0-56·9)	16·4 (0·0-60·4)	31·0 (0·0-92·4)	5·8 (0·0-17·9)	8·6 (0·5-22·0)	8·9 (0·6-22·4)	12·2 (1·4-28·9)
Ethiopia	0·1 (0·0-1·2)	7·7 (4·0-11·0)	8·7 (4·9-12·1)	17·2 (13·1-21·2)	5·6 (0·0-15·2)	12·6 (3·2-25·6)	12·8 (3·3-26·0)	16·3 (4·5-31·7)	0·2 (0·0-1·8)	0·6 (0·0-4·3)	0·5 (0·0-4·1)	1·8 (0·0-8·7)	0·1 (0·0-0·8)	0·5 (0·0-2·9)	0·6 (0·0-3·3)	2·8 (0·0-8·0)
Kenya	29·7 (24·3-34·8)	32·6 (28·1-36·8)	32·8 (28·2-36·9)	34·8 (30·3-38·7)	18·6 (14·0-30·0)	26·0 (20·8-39·7)	26·5 (21·2-40·4)	33·1 (27·2-49·5)	8·0 (4·6-12·2)	15·8 (8·7-22·3)	16·4 (9·2-23·0)	27·0 (16·7-34·2)	5·0 (3·4-7·0)	8·7 (6·9-10·7)	9·5 (7·9-11·5)	10·1 (5·0-19·0)
Madagascar	25·6 (20·8-30·4)	32·0 (27·5-36·7)	32·3 (27·8-36·9)	36·2 (31·7-40·8)	22·1 (13·9-40·9)	15·3 (7·3-35·4)	15·1 (7·1-34·8)	12·3 (4·7-27·6)	4·2 (0·0-9·3)	1·6 (0·0-5·3)	1·8 (0·0-5·5)	4·2 (0·0-9·6)	9·1 (2·6-18·1)	8·2 (1·0-19·7)	8·2 (0·8-20·1)	9·0 (0·0-23·2)
Malawi	27·6 (23·2-31·7)	32·0 (27·4-36·0)	32·2 (27·5-36·2)	35·1 (29·8-39·6)	6·9 (3·8-10·6)	13·8 (9·8-19·1)	14·4 (10·4-19·6)	22·0 (17·8-27·6)	0·2 (0·0-1·6)	10·0 (0·0-23·2)	10·5 (0·0-24·7)	22·6 (0·2-58·7)	8·6 (3·5-15·9)	1·8 (0·0-5·7)	1·8 (0·0-5·7)	0·4 (0·0-4·5)
Mozambique	18·1 (14·6-21·6)	20·9 (17·2-24·6)	21·3 (17·6-25·0)	26·8 (22·6-31·2)	1·1 (0·0-6·0)	4·9 (0·0-16·6)	5·3 (0·0-17·4)	16·6 (0·0-55·1)	6·1 (0·0-20·4)	20·9 (3·7-46·6)	22·1 (3·4-50·0)	43·3 (7·7-90·2)	5·9 (0·0-15·0)	9·6 (3·3-19·1)	10·2 (3·5-20·1)	14·0 (4·7-23·7)
Rwanda	11·4 (6·6-16·0)	25·9 (21·4-29·7)	26·4 (22·0-30·3)	34·2 (28·7-39·1)	18·2 (10·5-34·7)	30·3 (21·1-50·5)	31·2 (21·9-51·6)	43·8 (1·6-80·9)	1·6 (0·0-10·2)	15·7 (0·0-36·7)	16·1 (0·0-37·4)	33·8 (10·7-65·2)	0·0 (0·0-0·0)	0·4 (0·0-2·3)	0·5 (0·0-2·7)	4·7 (0·4-10·6)
Somalia	1·9 (0·0-5·4)	2·8 (0·0-6·4)	2·9 (0·0-6·5)	4·2 (0·7-7·9)	7·2 (0·0-18·8)	18·0 (8·3-33·6)	18·6 (8·6-33·5)	26·8 (12·1-44·7)	4·0 (0·0-15·9)	21·3 (6·0-44·1)	22·4 (5·5-47·5)	44·0 (4·0-88·7)	4·8 (0·0-14·7)	9·0 (0·6-22·8)	9·3 (0·7-23·3)	14·2 (2·3-30·4)
South Sudan	0·0 (0·0-0·0)	0·1 (0·0-0·7)	0·1 (0·0-0·9)	0·6 (0·0-2·7)	1·7 (0·0-6·3)	2·8 (0·0-9·1)	2·9 (0·0-9·2)	4·9 (0·0-11·9)	6·2 (0·7-15·4)	2·3 (0·0-14·3)	2·4 (0·0-15·0)	3·0 (0·0-30·5)	3·0 (0·0-11·6)	2·8 (0·0-10·6)	2·8 (0·0-10·8)	2·6 (0·0-11·5)
Tanzania	7·9 (3·3-12·6)	21·3 (17·4-25·3)	21·8 (18·0-25·8)	29·2 (24·9-33·3)	19·2 (12·7-32·7)	24·0 (16·1-39·1)	23·6 (15·7-38·5)	18·3 (9·0-34·0)	0·6 (0·0-5·0)	21·3 (10·1-35·2)	23·2 (11·4-38·3)	64·4 (46·7-84·2)	5·4 (0·0-16·3)	6·3 (0·0-18·5)	6·4 (0·0-18·8)	7·3 (0·0-23·5)
Uganda	0·4 (0·0-2·9)	14·0 (9·6-17·8)	14·9 (10·5-18·8)	28·0 (22·0-33·4)	23·3 (16·2-42·7)	30·1 (22·8-48·4)	30·5 (23·1-49·1)	35·9 (26·6-56·7)	0·2 (0·0-2·4)	6·2 (0·0-15·7)	6·3 (0·0-16·0)	14·4 (6·1-37·0)	2·4 (0·0-6·8)	7·5 (3·1-13·5)	7·8 (3·3-14·0)	13·6 (5·9-23·3)
Zambia	12·5 (7·4-17·3)	21·0 (16·6-25·5)	21·9 (17·5-26·3)	33·5 (29·0-38·2)	20·1 (12·9-33·1)	23·7 (12·9-40·2)	24·1 (12·9-41·1)	30·3 (12·3-51·7)	13·1 (7·3-21·9)	25·8 (8·0-52·3)	26·6 (7·8-55·3)	39·8 (9·4-86·4)	10·3 (1·5-25·7)	10·7 (3·9-19·7)	10·9 (3·9-20·0)	10·1 (3·3-19·6)
**Central Sub-Saharan Africa**																
Angola	2·6 (0·0-7·8)	13·8 (8·3-19·2)	14·8 (9·3-20·2)	27·9 (22·1-33·2)	0·6 (0·0-4·8)	6·8 (0·0-17·0)	7·6 (0·1-18·1)	18·9 (7·8-29·9)	25·0 (18·1-37·9)	27·1 (9·4-49·1)	27·8 (8·6-52·0)	38·3 (5·5-84·5)	6·6 (0·0-19·5)	9·2 (0·6-25·1)	9·5 (0·7-25·7)	12·0 (1·1-30·7)
Central African Republic	19·9 (15·4-24·3)	18·0 (13·7-22·2)	17·4 (13·0-21·7)	14·4 (9·9-18·9)	0·3 (0·0-2·1)	0·4 (0·0-3·0)	0·4 (0·0-3·0)	0·4 (0·0-2·5)	2·4 (0·0-7·9)	5·5 (0·0-18·3)	5·5 (0·0-19·2)	8·9 (0·4-36·3)	9·3 (1·5-20·5)	9·3 (2·1-20·4)	9·2 (2·0-20·7)	9·5 (2·3-23·1)
Congo	20·4 (14·7-26·2)	26·8 (22·0-31·9)	27·3 (22·5-32·3)	33·1 (28·6-37·8)	16·0 (6·3-27·7)	21·3 (8·7-36·5)	22·2 (9·5-37·6)	34·3 (0·0-69·8)	16·5 (8·0-29·1)	19·7 (2·4-40·6)	21·0 (1·8-44·0)	24·9 (0·0-79·3)	13·8 (2·9-31·0)	14·5 (3·2-32·9)	14·9 (3·3-33·7)	15·9 (2·2-41·3)
Democratic Republic of the Congo	0·0 (0·0-0·0)	0·1 (0·0-2·2)	0·3 (0·0-3·3)	7·7 (0·0-15·9)	1·3 (0·0-5·1)	1·4 (0·0-6·2)	1·6 (0·0-6·6)	5·6 (0·3-11·8)	3·6 (0·0-10·9)	11·9 (0·0-24·3)	12·9 (0·0-26·2)	30·8 (0·0-56·6)	2·1 (0·0-8·1)	1·1 (0·0-4·5)	1·2 (0·0-4·7)	0·4 (0·0-2·7)
Equatorial Guinea	22·2 (16·1-27·9)	42·1 (37·4-46·3)	42·7 (38·0-47·0)	51·7 (47·0-56·5)	7·1 (0·0-17·7)	28·8 (11·4-47·5)	29·9 (12·1-48·7)	45·4 (4·9-77·1)	17·0 (8·7-24·7)	57·0 (14·8-89·1)	58·2 (14·8-89·7)	82·5 (43·4-99·0)	6·9 (0·0-19·7)	15·7 (3·4-34·4)	16·4 (3·7-35·3)	28·5 (8·7-49·8)
Gabon	28·2 (22·8-33·2)	34·2 (29·7-38·1)	34·6 (30·1-38·4)	38·7 (34·2-42·5)	26·9 (10·7-43·3)	50·0 (26·6-66·9)	50·8 (27·2-67·7)	62·1 (35·6-78·7)	27·0 (17·8-35·7)	44·6 (21·7-70·2)	46·0 (22·1-72·8)	62·5 (28·2-94·2)	21·9 (6·3-44·3)	33·3 (13·2-58·8)	34·0 (13·7-59·7)	46·0 (22·4-73·3)

Location	Indicator 7.1.2:<br>Risk-weighted prevalence of household air pollution, as measured by the summary exposure value (SEV) for household air pollution (%)				Indicator 8.8.1:<br>Age-standardised all-cause disability-adjusted life year (DALY) rates attributable to occupational risks (per 100,000 population)				Indicator 11.6.2:<br>Population-weighted mean levels of fine particulate matter smaller than 2.5 microns in diameter (PM2.5)				Indicator 16.1.1:<br>Age-standardised death rate due to interpersonal violence (per 100,000 population)			
	**2000**	**2015**	**2016**	**2030**	**2000**	**2015**	**2016**	**2030**	**2000**	**2015**	**2016**	**2030**	**2000**	**2015**	**2016**	**2030**
Sierra Leone	5·6<br>(0·0-16·4)	13·4<br>(3·9-23·7)	14·2<br>(4·7-24·4)	25·1<br>(16·4-35·1)	52·6<br>(45·2-60·0)	58·0<br>(50·7-65·2)	58·6<br>(51·2-65·8)	66·3<br>(59·1-73·4)	48·4<br>(39·2-53·2)	32·9<br>(21·5-38·4)	31·5<br>(22·1-37·3)	32·2<br>(19·5-39·5)	73·1<br>(67·0-80·3)	73·1<br>(66·8-80·3)	73·0<br>(66·4-80·5)	74·0<br>(62·0-85·1)
Togo	9·3<br>(0·0-19·9)	21·8<br>(12·7-32·5)	23·0<br>(13·7-33·7)	39·8<br>(30·5-50·3)	35·0<br>(26·9-42·9)	41·4<br>(34·2-47·7)	42·2<br>(34·9-48·5)	53·3<br>(45·9-59·8)	23·3<br>(16·2-28·0)	9·7<br>(2·6-14·6)	8·1<br>(0·6-13·0)	10·7<br>(3·8-16·5)	56·8<br>(45·4-65·7)	52·1<br>(43·6-59·3)	52·1<br>(43·7-59·2)	51·0<br>(39·3-62·2)
**Eastern Sub-Saharan Africa**																
Burundi	1·8<br>(0·0-10·7)	6·8<br>(0·0-17·5)	7·5<br>(0·0-18·1)	18·4<br>(8·5-28·6)	2·0<br>(0·0-10·6)	15·1<br>(4·7-24·8)	16·2<br>(5·9-25·9)	31·6<br>(22·0-40·9)	27·6<br>(22·6-31·9)	28·1<br>(22·4-32·8)	28·2<br>(22·7-32·6)	29·1<br>(20·9-37·0)	82·1<br>(74·7-89·8)	83·7<br>(71·7-94·4)	83·9<br>(71·8-94·5)	85·4<br>(67·1-100·0)
Comoros	16·1<br>(4·6-27·5)	38·7<br>(23·7-58·0)	39·6<br>(24·1-59·8)	52·8<br>(30·4-80·3)	43·4<br>(32·1-54·0)	53·9<br>(44·1-63·2)	54·5<br>(44·7-63·6)	62·0<br>(52·8-70·4)	54·9<br>(46·3-63·2)	58·4<br>(50·0-66·4)	58·2<br>(48·7-66·3)	58·0<br>(47·2-67·3)	36·1<br>(29·6-45·4)	34·1<br>(26·2-48·6)	34·1<br>(26·4-48·8)	33·8<br>(21·3-53·5)
Djibouti	70·4<br>(45·6-89·4)	82·9<br>(52·4-97·9)	83·2<br>(50·5-98·4)	88·4<br>(38·5-100·0)	67·3<br>(57·3-77·2)	73·0<br>(63·4-82·3)	73·4<br>(63·9-82·6)	78·4<br>(69·0-87·1)	27·5<br>(5·1-45·6)	15·3<br>(0·0-30·8)	15·4<br>(0·0-31·2)	11·9<br>(0·0-28·5)	37·4<br>(29·9-48·0)	34·3<br>(25·9-49·0)	34·4<br>(26·1-49·6)	34·5<br>(20·3-55·1)
Eritrea	31·4<br>(17·5-46·4)	56·6<br>(21·4-93·0)	57·9<br>(22·2-93·9)	74·6<br>(38·1-99·3)	39·4<br>(27·6-49·7)	48·0<br>(38·1-57·6)	48·8<br>(38·9-58·3)	59·5<br>(50·5-67·7)	30·4<br>(27·4-33·1)	25·4<br>(22·3-28·1)	25·2<br>(22·2-27·8)	26·2<br>(23·0-29·0)	33·1<br>(26·3-44·0)	29·5<br>(22·2-44·4)	29·4<br>(21·5-44·4)	27·4<br>(14·9-46·3)
Ethiopia	2·8<br>(0·0-13·2)	12·3<br>(2·2-23·7)	13·5<br>(3·3-24·8)	30·3<br>(19·2-40·8)	4·9<br>(0·0-21·8)	28·6<br>(18·7-38·1)	30·0<br>(20·3-39·3)	50·9<br>(41·6-59·3)	37·1<br>(34·8-38·8)	26·3<br>(24·2-27·9)	25·7<br>(23·3-27·4)	25·9<br>(23·5-27·9)	22·6<br>(6·7-30·0)	24·8<br>(12·0-33·3)	25·4<br>(12·0-34·2)	29·9<br>(13·7-45·5)
Kenya	26·3<br>(22·2-30·1)	36·6<br>(32·0-41·0)	37·7<br>(33·0-42·1)	51·9<br>(47·5-55·7)	29·4<br>(23·0-36·1)	33·2<br>(27·0-39·6)	33·6<br>(27·4-40·2)	40·4<br>(33·7-47·6)	63·6<br>(62·1-65·2)	63·4<br>(62·0-64·8)	63·4<br>(62·0-64·8)	63·9<br>(62·3-65·6)	28·8<br>(19·6-36·4)	28·3<br>(20·7-34·3)	28·3<br>(20·8-34·1)	28·3<br>(20·1-35·6)
Madagascar	7·6<br>(0·0-18·0)	12·7<br>(3·4-22·9)	13·2<br>(3·9-23·3)	20·4<br>(11·1-30·5)	0·0<br>(0·0-0·0)	6·2<br>(0·0-14·6)	7·1<br>(0·0-15·6)	20·6<br>(12·5-29·9)	57·6<br>(55·7-59·3)	54·2<br>(51·8-56·3)	54·1<br>(51·8-56·0)	53·1<br>(49·9-58·4)	58·4<br>(49·6-82·0)	66·8<br>(57·5-83·3)	67·0<br>(57·3-83·6)	70·5<br>(54·0-88·6)
Malawi	5·9<br>(0·0-17·2)	13·1<br>(3·5-23·0)	13·8<br>(4·1-23·6)	23·1<br>(13·6-32·4)	20·9<br>(10·1-31·6)	36·0<br>(26·3-45·9)	36·8<br>(27·2-46·8)	49·4<br>(39·7-58·8)	43·7<br>(39·9-46·6)	44·9<br>(41·7-47·8)	44·8<br>(41·6-47·6)	44·4<br>(40·8-47·6)	58·5<br>(29·0-73·8)	62·8<br>(31·9-76·6)	63·9<br>(31·9-77·6)	79·4<br>(34·8-100·0)
Mozambique	2·6<br>(0·0-12·0)	12·6<br>(1·7-25·7)	13·9<br>(2·9-27·2)	33·8<br>(21·4-46·4)	18·4<br>(7·0-29·5)	33·3<br>(23·9-42·1)	34·3<br>(24·9-43·1)	47·9<br>(39·2-56·5)	52·1<br>(48·7-54·7)	55·0<br>(52·3-57·0)	54·8<br>(52·3-57·0)	55·2<br>(52·4-57·5)	39·1<br>(30·8-47·1)	35·1<br>(27·6-44·0)	35·2<br>(27·8-44·0)	37·4<br>(23·1-51·4)
Rwanda	3·5<br>(0·0-14·4)	14·5<br>(5·5-24·0)	15·4<br>(6·4-24·9)	29·5<br>(20·9-37·5)	3·3<br>(0·0-11·4)	30·6<br>(21·8-39·4)	31·8<br>(22·9-40·5)	47·7<br>(38·9-56·2)	23·1<br>(18·5-27·4)	23·9<br>(19·0-28·2)	23·7<br>(18·3-27·9)	23·4<br>(15·1-32·2)	45·3<br>(37·1-54·6)	50·0<br>(43·0-57·1)	49·9<br>(42·5-57·1)	50·9<br>(36·1-64·1)
Somalia	1·7<br>(0·0-9·8)	8·0<br>(0·0-18·9)	8·8<br>(0·0-19·8)	21·6<br>(10·1-32·1)	8·8<br>(0·0-24·8)	16·3<br>(6·3-26·0)	16·9<br>(7·1-26·5)	26·1<br>(17·7-33·5)	52·8<br>(48·9-55·2)	50·8<br>(45·9-53·7)	50·5<br>(45·7-53·3)	49·7<br>(44·6-53·3)	33·1<br>(24·6-43·3)	29·3<br>(20·8-43·7)	29·2<br>(20·7-43·6)	28·1<br>(15·9-46·3)
South Sudan	0·2<br>(0·0-2·7)	1·3<br>(0·0-9·8)	1·6<br>(0·0-10·7)	10·4<br>(0·0-23·3)	32·8<br>(12·9-55·9)	16·1<br>(1·0-37·5)	15·3<br>(0·9-37·1)	9·5<br>(0·0-37·3)	30·8<br>(29·5-32·2)	26·8<br>(25·7-28·2)	26·4<br>(25·1-27·7)	24·9<br>(23·4-26·6)	43·2<br>(33·2-56·1)	38·2<br>(27·7-54·4)	37·9<br>(27·4-54·2)	33·3<br>(19·1-53·5)
Tanzania	11·9<br>(3·4-21·6)	21·0<br>(10·8-34·8)	22·1<br>(11·9-35·9)	37·0<br>(26·9-51·2)	13·3<br>(3·7-22·4)	25·1<br>(17·5-32·7)	25·6<br>(18·0-33·1)	32·4<br>(24·8-39·7)	52·7<br>(51·3-54·1)	52·7<br>(50·8-54·3)	52·7<br>(50·9-54·3)	52·5<br>(49·9-55·1)	30·9<br>(20·0-38·0)	31·8<br>(21·4-39·0)	31·9<br>(21·6-39·3)	32·6<br>(19·0-44·8)
Uganda	4·3<br>(0·0-14·5)	13·8<br>(5·3-23·4)	14·7<br>(6·0-24·4)	26·7<br>(15·9-36·7)	20·8<br>(10·4-30·8)	32·4<br>(24·0-40·6)	33·1<br>(24·7-41·3)	43·8<br>(35·6-51·7)	19·1<br>(17·6-20·7)	12·8<br>(11·4-14·4)	12·5<br>(10·9-14·0)	13·8<br>(11·9-16·8)	36·7<br>(30·6-46·1)	36·4<br>(29·2-47·8)	36·6<br>(29·3-48·0)	38·1<br>(24·3-52·3)
Zambia	26·8<br>(18·3-35·6)	29·1<br>(19·7-39·4)	28·9<br>(19·4-39·4)	27·3<br>(15·0-38·9)	30·6<br>(20·8-40·2)	42·1<br>(33·1-51·6)	43·4<br>(34·3-53·0)	62·0<br>(52·3-72·4)	41·8<br>(38·3-44·7)	42·4<br>(38·3-45·4)	42·2<br>(38·2-45·2)	47·7<br>(43·4-51·3)	27·9<br>(20·6-35·8)	28·8<br>(19·6-38·3)	29·0<br>(19·6-38·8)	32·7<br>(15·6-48·8)
**Central Sub-Saharan Africa**																
Angola	19·2<br>(7·8-31·0)	61·5<br>(37·3-83·3)	63·4<br>(37·7-86·0)	85·0<br>(50·6-99·3)	13·9<br>(2·5-36·2)	39·0<br>(30·3-47·5)	40·9<br>(32·3-49·3)	67·5<br>(57·7-76·5)	33·3<br>(31·1-35·3)	37·1<br>(25·5-43·2)	36·8<br>(24·8-43·4)	38·2<br>(23·3-49·6)	46·4<br>(29·0-56·4)	47·0<br>(29·0-57·7)	47·6<br>(29·5-59·0)	51·2<br>(29·5-72·0)
Central African Republic	6·5<br>(0·0-17·8)	11·0<br>(0·3-24·2)	11·3<br>(0·6-24·8)	16·3<br>(5·0-34·1)	7·6<br>(0·0-18·0)	18·2<br>(8·9-27·0)	18·4<br>(9·1-27·2)	20·7<br>(11·5-29·1)	24·5<br>(14·2-29·8)	16·6<br>(7·4-21·9)	16·2<br>(6·7-21·2)	15·9<br>(5·8-21·7)	39·5<br>(26·5-53·0)	38·3<br>(26·9-51·1)	38·4<br>(27·0-51·4)	39·6<br>(23·4-56·2)
Congo	25·8<br>(17·2-34·9)	52·3<br>(33·8-74·9)	54·2<br>(34·7-77·7)	75·8<br>(48·0-96·9)	26·6<br>(18·8-33·8)	42·6<br>(34·5-50·8)	43·5<br>(35·3-51·7)	55·6<br>(46·9-64·2)	19·6<br>(2·4-31·5)	22·2<br>(3·5-34·8)	22·9<br>(3·5-35·2)	23·0<br>(2·8-39·2)	41·9<br>(30·4-51·8)	46·6<br>(35·8-55·1)	46·4<br>(35·4-55·3)	47·1<br>(29·0-64·5)
Democratic Republic of the Congo	4·8<br>(0·0-15·5)	10·5<br>(0·0-21·8)	11·4<br>(0·6-22·6)	23·6<br>(11·8-34·2)	22·8<br>(9·3-49·8)	26·5<br>(15·0-45·1)	26·6<br>(15·7-45·3)	30·9<br>(0·0-54·0)	24·7<br>(18·0-29·2)	21·8<br>(17·4-24·9)	21·5<br>(17·3-24·4)	20·8<br>(14·9-28·6)	49·4<br>(42·6-55·5)	49·3<br>(42·6-54·8)	49·4<br>(42·6-55·0)	49·2<br>(38·1-59·5)
Equatorial Guinea	26·8<br>(13·4-52·3)	55·2<br>(34·4-89·0)	56·6<br>(35·6-90·1)	74·4<br>(52·7-98·0)	38·6<br>(29·6-47·7)	67·8<br>(59·1-76·2)	68·6<br>(59·9-76·9)	79·1<br>(70·2-87·5)	24·1<br>(5·7-34·9)	15·8<br>(0·0-27·2)	15·1<br>(0·0-26·9)	11·6<br>(0·0-23·6)	43·2<br>(30·5-54·3)	53·1<br>(38·2-67·5)	53·0<br>(38·1-67·6)	63·7<br>(39·1-88·6)
Gabon	74·2<br>(67·1-80·7)	90·7<br>(77·9-97·1)	91·2<br>(77·5-97·5)	96·4<br>(79·9-99·9)	51·1<br>(43·5-59·4)	63·5<br>(54·8-71·0)	64·0<br>(55·1-71·6)	71·2<br>(61·8-79·2)	33·2<br>(16·8-42·7)	28·2<br>(14·6-36·3)	27·8<br>(14·9-35·7)	26·7<br>(13·7-35·6)	43·4<br>(32·1-52·3)	45·9<br>(34·9-56·0)	46·3<br>(34·6-56·7)	49·7<br>(28·8-70·4)

Location	Indicator 16.1.2: Death rate due to conflict and terrorism (per 100,000 population)				Indicator 16.1.3: Age-standardised prevalence of physical or sexual violence experienced by populations in the last 12 months (%)				Indicator 16.2.3: Age-standardised prevalence of women and men aged 18-29 years who experienced sexual violence by age 18 (%)				Indicator 17.19.2c: Percentage of well-certified deaths by a vital registration (VR) system among a country's total population (%)			
	**2000**	**2015**	**2016**	**2030**	**2000**	**2015**	**2016**	**2030**	**2000**	**2015**	**2016**	**2030**	**2000**	**2015**	**2016**	**2030**
Sierra Leone	5·4 (0·0-15·8)	100·0 (100·0-100·0)	100·0 (100·0-100·0)	100·0 (100·0-100·0)	0·0 (0·0-0·0)	9·4 (4·8-14·1)	10·5 (6·0-15·2)	25·1 (20·7-29·3)	10·5 (0·6-20·6)	10·5 (1·0-20·3)	10·5 (1·0-20·3)	10·5 (1·2-20·3)	0·0 (0·0-0·0)	0·0 (0·0-0·0)	0·0 (0·0-0·0)	0·0 (0·0-0·0)
Togo	100·0 (100·0-100·0)	100·0 (100·0-100·0)	100·0 (100·0-100·0)	100·0 (100·0-100·0)	52·3 (49·9-54·9)	60·0 (57·8-62·3)	60·4 (58·3-62·7)	66·3 (64·2-68·5)	10·4 (0·3-20·6)	10·6 (1·3-20·4)	10·6 (1·3-20·4)	10·8 (1·7-20·4)	0·0 (0·0-0·0)	0·0 (0·0-0·0)	0·0 (0·0-0·0)	0·0 (0·0-0·0)
**Eastern Sub-Saharan Africa**																
Burundi	1·2 (0·0-6·6)	38·6 (35·0-41·4)	44·6 (39·4-50·4)	95·5 (82·1-99·9)	35·0 (31·6-38·4)	40·3 (37·1-43·7)	40·8 (37·6-44·1)	47·0 (44·0-50·3)	40·2 (33·7-46·8)	40·2 (33·9-46·4)	40·2 (33·9-46·4)	40·4 (33·7-46·8)	0·0 (0·0-0·0)	0·0 (0·0-0·0)	0·0 (0·0-0·0)	0·0 (0·0-0·0)
Comoros	100·0 (100·0-100·0)	100·0 (100·0-100·0)	100·0 (100·0-100·0)	100·0 (100·0-100·0)	49·3 (46·9-51·9)	59·6 (57·5-61·8)	60·1 (58·0-62·3)	66·5 (64·5-68·7)	39·8 (33·5-46·0)	39·7 (33·4-45·9)	39·7 (33·4-45·9)	39·6 (33·4-45·6)	0·0 (0·0-0·0)	0·0 (0·0-0·0)	0·0 (0·0-0·0)	0·0 (0·0-0·0)
Djibouti	100·0 (100·0-100·0)	33·1 (26·6-41·7)	100·0 (100·0-100·0)	100·0 (100·0-100·0)	18·3 (14·4-22·4)	32·3 (28·6-35·8)	33·2 (29·6-36·7)	45·4 (41·8-48·9)	39·8 (33·6-46·1)	39·7 (33·5-45·9)	39·7 (33·4-45·8)	39·6 (33·0-46·1)	0·0 (0·0-0·0)	0·0 (0·0-0·0)	0·0 (0·0-0·0)	0·0 (0·0-0·0)
Eritrea	0·0 (0·0-0·0)	100·0 (100·0-100·0)	61·4 (55·9-66·7)	61·4 (55·9-66·7)	15·3 (11·3-19·9)	23·5 (19·8-27·7)	24·2 (20·6-28·4)	34·1 (30·7-38·0)	40·1 (33·6-46·7)	39·7 (33·5-45·8)	39·7 (33·5-45·8)	39·4 (32·2-46·0)	0·0 (0·0-0·0)	0·0 (0·0-0·0)	0·0 (0·0-0·0)	0·0 (0·0-0·0)
Ethiopia	0·0 (0·0-0·0)	70·5 (60·8-86·0)	49·5 (44·1-55·1)	41·7 (21·8-58·0)	0·0 (0·0-0·1)	13·3 (9·4-17·7)	15·0 (11·1-19·4)	36·7 (33·1-40·5)	33·5 (25·2-41·6)	32·1 (23·6-39·9)	32·1 (23·6-39·9)	32·0 (23·5-39·6)	0·0 (0·0-0·0)	0·0 (0·0-0·0)	0·0 (0·0-0·0)	0·0 (0·0-0·0)
Kenya	53·7 (49·8-57·0)	58·6 (54·9-61·8)	100·0 (100·0-100·0)	100·0 (100·0-100·0)	38·0 (33·9-41·8)	37·8 (34·2-41·1)	37·8 (34·2-41·2)	38·1 (34·6-41·4)	1·2 (0·0-8·9)	1·0 (0·0-8·1)	1·0 (0·0-8·0)	1·0 (0·0-8·1)	0·0 (0·0-0·0)	0·0 (0·0-0·0)	0·0 (0·0-0·0)	0·0 (0·0-0·0)
Madagascar	100·0 (100·0-100·0)	49·2 (44·0-54·0)	59·5 (53·9-64·8)	59·5 (53·9-64·8)	32·5 (29·0-36·2)	43·3 (40·1-46·5)	43·9 (40·7-47·1)	51·0 (47·7-54·1)	39·9 (33·6-46·1)	39·9 (33·6-46·1)	39·9 (33·6-46·1)	39·9 (33·6-46·1)	2·8 (2·1-3·6)	4·1 (3·2-5·3)	4·2 (3·3-5·4)	5·9 (3·6-9·0)
Malawi	100·0 (100·0-100·0)	100·0 (100·0-100·0)	100·0 (100·0-100·0)	100·0 (100·0-100·0)	34·3 (29·1-39·4)	45·4 (41·1-49·9)	45·9 (41·6-50·4)	52·6 (48·2-57·0)	39·9 (33·6-46·2)	39·9 (33·7-46·1)	39·9 (33·7-46·1)	39·8 (33·5-46·0)	0·0 (0·0-0·0)	0·0 (0·0-0·0)	0·0 (0·0-0·0)	0·0 (0·0-0·0)
Mozambique	51·6 (46·7-56·1)	64·0 (59·0-68·5)	67·2 (61·8-72·2)	67·2 (61·8-72·2)	24·9 (21·5-28·3)	36·4 (32·9-40·3)	37·3 (33·8-41·2)	49·2 (46·0-52·5)	59·3 (54·3-63·9)	57·3 (52·4-62·1)	57·3 (52·4-62·0)	56·1 (51·2-60·7)	5·8 (4·6-7·2)	10·4 (7·9-13·2)	10·8 (8·4-13·8)	18·4 (11·6-26·4)
Rwanda	39·1 (28·1-58·9)	100·0 (100·0-100·0)	100·0 (100·0-100·0)	100·0 (100·0-100·0)	31·3 (27·6-34·7)	48·3 (45·3-51·2)	49·2 (46·2-52·0)	60·9 (58·2-63·4)	40·5 (34·1-46·9)	39·9 (33·4-46·2)	39·9 (33·4-46·3)	39·4 (32·5-46·0)	0·0 (0·0-0·0)	0·0 (0·0-0·0)	0·0 (0·0-0·0)	0·0 (0·0-0·0)
Somalia	16·8 (13·2-20·2)	15·1 (5·1-34·5)	1·1 (0·0-5·0)	0·4 (0·0-6·5)	1·2 (0·0-5·4)	6·8 (2·4-11·6)	7·4 (3·0-12·2)	15·5 (11·4-20·0)	39·9 (33·7-46·1)	39·9 (33·6-46·2)	39·9 (33·6-46·2)	39·9 (33·6-46·2)	0·0 (0·0-0·0)	0·0 (0·0-0·0)	0·0 (0·0-0·0)	0·0 (0·0-0·0)
South Sudan	3·4 (0·0-18·4)	3·7 (0·0-11·9)	22·4 (15·6-32·0)	17·3 (0·0-76·2)	0·0 (0·0-0·0)	1·8 (0·0-6·1)	2·3 (0·0-6·7)	10·1 (5·4-14·7)	39·9 (33·6-46·0)	39·8 (33·6-46·0)	39·8 (33·6-46·0)	39·7 (33·5-45·9)	0·0 (0·0-0·0)	0·0 (0·0-0·0)	0·0 (0·0-0·0)	0·0 (0·0-0·0)
Tanzania	62·5 (57·4-67·2)	100·0 (100·0-100·0)	100·0 (100·0-100·0)	100·0 (100·0-100·0)	15·9 (10·3-21·7)	36·2 (31·9-40·8)	37·2 (32·9-41·8)	50·6 (45·2-55·3)	27·4 (17·9-34·8)	24·7 (15·1-32·7)	24·1 (14·0-32·2)	23·9 (14·0-32·1)	0·0 (0·0-0·0)	0·0 (0·0-0·0)	0·0 (0·0-0·0)	0·0 (0·0-0·0)
Uganda	24·4 (20·9-27·6)	55·9 (51·9-58·8)	100·0 (100·0-100·0)	100·0 (100·0-100·0)	0·0 (0·0-0·0)	0·5 (0·0-4·4)	1·0 (0·0-5·7)	19·2 (12·2-25·6)	45·5 (37·4-52·7)	47·4 (39·4-54·3)	47·8 (39·8-54·6)	47·8 (39·7-54·7)	0·0 (0·0-0·0)	0·0 (0·0-0·0)	0·0 (0·0-0·0)	0·0 (0·0-0·0)
Zambia	100·0 (100·0-100·0)	100·0 (100·0-100·0)	100·0 (100·0-100·0)	100·0 (100·0-100·0)	18·5 (14·3-23·3)	32·9 (29·3-37·0)	34·3 (30·8-38·4)	52·0 (48·3-55·9)	41·2 (35·2-47·0)	41·3 (35·3-46·8)	41·3 (35·4-46·8)	41·3 (35·4-46·8)	0·0 (0·0-0·0)	0·0 (0·0-0·0)	0·0 (0·0-0·0)	0·0 (0·0-0·0)
**Central Sub-Saharan Africa**																
Angola	19·7 (9·3-40·5)	100·0 (100·0-100·0)	75·2 (66·1-89·3)	75·2 (66·1-89·3)	9·5 (5·2-13·3)	27·6 (24·0-30·8)	28·9 (25·3-32·1)	45·8 (42·7-48·5)	40·3 (32·1-48·5)	40·3 (32·1-48·4)	40·3 (32·1-48·4)	40·3 (32·1-48·4)	0·0 (0·0-0·0)	0·0 (0·0-0·0)	0·0 (0·0-0·0)	0·0 (0·0-0·0)
Central African Republic	100·0 (100·0-100·0)	18·1 (14·2-21·8)	30·2 (19·9-50·6)	89·3 (62·9-100·0)	26·6 (22·9-31·0)	31·6 (28·0-35·5)	31·2 (27·6-35·2)	26·4 (22·4-30·9)	40·3 (32·1-48·3)	40·3 (32·2-48·2)	40·3 (32·2-48·2)	40·4 (32·3-48·2)	0·0 (0·0-0·0)	0·0 (0·0-0·0)	0·0 (0·0-0·0)	0·0 (0·0-0·0)
Congo	10·2 (0·0-31·4)	100·0 (100·0-100·0)	48·1 (37·8-67·3)	99·9 (99·5-100·0)	27·6 (24·2-30·9)	38·6 (35·7-41·7)	39·3 (36·4-42·4)	48·5 (45·6-51·3)	40·4 (32·1-48·4)	40·4 (32·2-48·3)	40·4 (32·2-48·3)	40·4 (32·3-48·2)	0·0 (0·0-0·0)	0·0 (0·0-0·0)	0·0 (0·0-0·0)	0·0 (0·0-0·0)
Democratic Republic of the Congo	22·8 (17·1-29·0)	55·5 (51·5-58·5)	48·3 (38·1-67·5)	87·1 (42·4-100·0)	2·7 (0·0-8·9)	15·5 (9·4-21·2)	17·4 (11·4-22·9)	40·9 (36·2-45·3)	40·4 (32·1-48·4)	40·4 (32·2-48·4)	40·4 (32·1-48·4)	40·4 (32·1-48·4)	0·0 (0·0-0·0)	0·0 (0·0-0·0)	0·0 (0·0-0·0)	0·0 (0·0-0·0)
Equatorial Guinea	100·0 (100·0-100·0)	100·0 (100·0-100·0)	100·0 (100·0-100·0)	100·0 (100·0-100·0)	32·1 (29·0-35·0)	54·1 (51·6-56·3)	54·7 (52·3-56·9)	63·0 (61·0-65·0)	40·5 (32·7-48·3)	40·4 (32·3-48·3)	40·4 (32·3-48·2)	40·5 (32·5-48·2)	0·0 (0·0-0·0)	0·0 (0·0-0·0)	0·0 (0·0-0·0)	0·0 (0·0-0·0)
Gabon	100·0 (100·0-100·0)	100·0 (100·0-100·0)	100·0 (100·0-100·0)	100·0 (100·0-100·0)	30·5 (27·1-34·0)	46·2 (43·3-49·2)	46·8 (43·9-49·8)	54·7 (52·0-57·5)	40·4 (32·2-48·4)	40·4 (32·3-48·3)	40·4 (32·3-48·3)	40·5 (32·6-48·1)	0·0 (0·0-0·0)	0·0 (0·0-0·0)	0·0 (0·0-0·0)	0·0 (0·0-0·0)

Location	Indicator 1.5.1: Death rate due to exposure to forces of nature (per 100,000 population)				Indicator 2.2.1: Prevalence of stunting in children under 5 (%)				Indicator 2.2.2a: Prevalence of wasting in children under 5 (%)				Indicator 2.2.2b: Prevalence of overweight in children aged 2-4 (%)			
	2000	2015	2016	2030	2000	2015	2016	2030	2000	2015	2016	2030	2000	2015	2016	2030
**High-income North America**																
Canada	0·0 (0·0-0·0)	0·0 (0·0-0·0)	0·0 (0·0-0·0)	0·0 (0·0-0·0)	2·5 (2·1-2·9)	2·1 (1·8-2·4)	2·1 (1·8-2·4)	1·8 (1·6-2·1)	0·7 (0·3-1·5)	0·7 (0·3-1·4)	0·7 (0·3-1·4)	0·6 (0·3-1·5)	22·4 (17·7-28·2)	29·1 (22·3-36·4)	29·3 (22·4-36·7)	35·5 (22·5-52·0)
United States	0·09 (0·05-0·1)	0·08 (0·05-0·1)	0·07 (0·04-0·09)	0·09 (0·05-0·1)	2·4 (2·3-2·5)	1·9 (1·9-2·0)	1·9 (1·8-2·0)	1·6 (1·6-1·7)	0·6 (0·5-0·8)	0·6 (0·5-0·7)	0·6 (0·5-0·7)	0·5 (0·4-0·7)	20·6 (17·0-24·5)	23·6 (18·9-29·1)	24·0 (19·0-29·8)	27·5 (17·4-40·0)
**Australasia**																
Australia	0·02 (0·01-0·02)	0·02 (0·01-0·03)	0·01 (0·01-0·02)	0·01 (0·01-0·02)	3·4 (2·7-4·2)	2·9 (2·3-3·6)	2·9 (2·3-3·6)	2·5 (1·9-3·1)	1·2 (0·7-1·9)	0·9 (0·5-1·6)	0·9 (0·5-1·6)	0·8 (0·4-1·4)	18·9 (16·4-21·5)	25·5 (21·5-29·8)	25·8 (21·7-30·2)	33·8 (24·8-43·3)
New Zealand	0·03 (0·01-0·05)	0·4 (0·2-0·6)	0·3 (0·2-0·5)	0·3 (0·1-0·4)	3·6 (2·9-4·4)	3·2 (2·5-3·9)	3·1 (2·5-3·9)	2·7 (2·0-3·3)	1·2 (0·8-1·9)	1·0 (0·5-1·6)	1·0 (0·5-1·6)	0·8 (0·4-1·4)	31·3 (26·2-36·2)	32·6 (28·6-36·7)	32·8 (28·6-37·2)	35·7 (26·6-44·6)
**High-income Asia Pacific**																
Brunei	0·0 (0·0-0·0)	0·05 (0·01-0·09)	0·04 (0·01-0·07)	0·03 (0·01-0·06)	10·0 (8·5-11·7)	8·5 (7·2-10·0)	8·4 (7·0-9·8)	6·6 (5·4-7·9)	3·8 (2·6-5·3)	3·1 (2·2-4·3)	3·1 (2·2-4·3)	2·7 (1·9-3·5)	12·5 (8·2-18·4)	18·7 (12·4-25·9)	18·9 (12·7-26·1)	26·2 (13·0-42·7)
Japan	0·5 (0·08-0·9)	1·7 (0·3-3·2)	1·4 (0·2-2·7)	1·1 (0·2-2·1)	2·9 (2·8-3·0)	3·8 (3·6-3·9)	3·7 (3·6-3·9)	3·4 (3·3-3·6)	2·0 (1·9-2·2)	1·7 (1·5-1·8)	1·6 (1·5-1·8)	1·5 (1·4-1·7)	9·8 (7·2-13·0)	13·4 (8·9-19·4)	13·6 (9·0-19·7)	16·4 (7·9-28·5)
Singapore	0·01 (0·0-0·03)	0·0 (0·0-0·0)	0·0 (0·0-0·0)	0·0 (0·0-0·0)	3·3 (2·7-4·0)	3·1 (2·4-3·7)	3·0 (2·4-3·7)	2·6 (2·0-3·2)	4·2 (3·3-5·2)	3·5 (2·5-5·0)	3·5 (2·5-5·0)	2·9 (2·0-4·4)	17·2 (11·5-24·7)	23·3 (16·1-32·2)	23·6 (16·4-32·7)	31·2 (15·7-50·7)
South Korea	0·3 (0·04-0·5)	0·03 (0·0-0·05)	0·02 (0·0-0·04)	0·07 (0·01-0·1)	2·5 (1·9-3·1)	3·2 (2·5-3·9)	3·1 (2·5-3·8)	2·7 (2·1-3·6)	1·7 (1·0-2·4)	1·8 (1·1-2·6)	1·7 (1·1-2·6)	1·6 (0·9-2·4)	24·9 (17·4-33·9)	32·0 (23·0-41·7)	32·4 (23·4-42·2)	38·9 (22·4-56·9)
**Western Europe**																
Andorra	0·0 (0·0-0·0)	0·0 (0·0-0·0)	0·0 (0·0-0·0)	0·0 (0·0-0·0)	1·2 (0·9-1·6)	1·1 (0·9-1·6)	1·1 (0·8-1·6)	1·1 (0·8-1·6)	1·1 (0·7-1·7)	0·9 (0·6-1·5)	0·9 (0·6-1·5)	0·9 (0·5-1·5)	24·8 (18·9-31·3)	26·6 (20·3-33·0)	26·6 (20·4-33·3)	29·5 (18·1-43·5)
Austria	0·1 (0·05-0·2)	0·1 (0·05-0·2)	0·09 (0·04-0·1)	0·1 (0·05-0·2)	1·3 (1·0-1·8)	1·2 (0·9-1·7)	1·2 (0·9-1·7)	1·1 (0·8-1·7)	1·2 (0·7-1·8)	0·9 (0·6-1·6)	0·9 (0·6-1·6)	0·9 (0·5-1·5)	20·2 (15·2-25·5)	21·2 (16·1-27·2)	21·4 (16·3-27·4)	24·0 (14·2-36·9)
Belgium	0·0 (0·0-0·0)	0·0 (0·0-0·0)	0·0 (0·0-0·0)	0·0 (0·0-0·0)	1·2 (1·0-1·7)	1·2 (0·9-1·6)	1·2 (0·9-1·6)	1·1 (0·8-1·6)	1·1 (0·8-1·8)	0·9 (0·6-1·6)	0·9 (0·6-1·6)	0·8 (0·5-1·5)	18·7 (14·1-23·6)	18·9 (14·3-24·2)	19·0 (14·3-24·6)	19·7 (11·4-30·5)
Cyprus	0·0 (0·0-0·0)	0·01 (0·01-0·02)	0·01 (0·0-0·01)	0·02 (0·01-0·04)	1·4 (1·1-1·7)	1·2 (1·0-1·7)	1·2 (1·0-1·7)	1·1 (0·9-1·6)	1·2 (0·8-1·8)	1·0 (0·6-1·6)	0·9 (0·6-1·6)	0·8 (0·5-1·5)	25·1 (20·1-31·4)	29·7 (23·2-36·8)	29·9 (23·2-36·9)	35·3 (23·3-48·9)
Denmark	0·0 (0·0-0·0)	0·0 (0·0-0·0)	0·0 (0·0-0·0)	0·0 (0·0-0·0)	1·1 (0·9-1·6)	1·1 (0·8-1·6)	1·1 (0·8-1·6)	1·1 (0·7-1·7)	1·1 (0·7-1·7)	0·9 (0·5-1·5)	0·9 (0·5-1·5)	0·8 (0·5-1·4)	28·5 (22·4-35·2)	30·5 (24·4-37·7)	30·4 (24·2-37·8)	32·8 (21·4-46·4)
Finland	0·09 (0·04-0·1)	0·0 (0·0-0·0)	0·0 (0·0-0·0)	0·0 (0·0-0·01)	1·2 (1·0-1·7)	1·2 (0·9-1·6)	1·1 (0·9-1·6)	1·1 (0·8-1·6)	1·1 (0·8-1·7)	0·9 (0·6-1·6)	0·9 (0·6-1·5)	0·8 (0·5-1·5)	20·0 (14·8-25·6)	21·3 (16·5-26·8)	21·5 (16·7-27·3)	22·7 (13·8-34·3)
France	0·05 (0·02-0·07)	0·01 (0·0-0·02)	0·01 (0·0-0·01)	0·01 (0·01-0·02)	1·3 (1·1-1·7)	1·2 (1·0-1·6)	1·2 (1·0-1·6)	1·2 (0·9-1·6)	1·2 (0·8-1·8)	1·0 (0·6-1·6)	1·0 (0·6-1·5)	0·9 (0·5-1·5)	19·9 (15·2-25·8)	21·3 (16·1-27·1)	21·3 (16·0-27·4)	23·0 (14·0-34·9)
Germany	0·0 (0·0-0·0)	0·0 (0·0-0·0)	0·0 (0·0-0·0)	0·0 (0·0-0·0)	1·0 (0·8-1·6)	1·0 (0·7-1·6)	1·0 (0·7-1·6)	0·9 (0·6-1·6)	0·8 (0·5-1·5)	0·8 (0·5-1·4)	0·8 (0·5-1·4)	0·8 (0·5-1·4)	21·9 (17·0-27·5)	25·7 (19·9-32·8)	25·9 (20·0-32·9)	30·6 (19·7-44·1)
Greece	0·2 (0·09-0·3)	0·0 (0·0-0·0)	0·0 (0·0-0·0)	0·02 (0·01-0·03)	1·4 (1·2-1·8)	1·3 (1·0-1·7)	1·3 (1·0-1·7)	1·2 (0·9-1·7)	1·2 (0·8-1·8)	1·0 (0·6-1·7)	1·0 (0·6-1·6)	0·9 (0·5-1·6)	27·9 (22·4-33·9)	35·0 (28·4-41·8)	34·9 (28·3-41·6)	41·4 (28·9-54·1)
Iceland	1·3 (0·7-2·0)	0·01 (0·0-0·01)	0·0 (0·0-0·0)	0·1 (0·06-0·1)	1·2 (0·9-1·7)	1·1 (0·8-1·6)	1·1 (0·8-1·6)	1·0 (0·7-1·6)	1·1 (0·7-1·7)	0·9 (0·5-1·6)	0·9 (0·5-1·6)	0·8 (0·5-1·5)	30·6 (24·3-37·2)	36·0 (29·3-43·6)	36·1 (29·5-43·9)	41·1 (28·4-55·4)
Ireland	0·0 (0·0-0·0)	0·0 (0·0-0·0)	0·0 (0·0-0·0)	0·0 (0·0-0·0)	1·3 (1·1-1·7)	1·2 (0·9-1·7)	1·2 (0·9-1·7)	1·0 (0·8-1·6)	1·2 (0·8-1·8)	0·9 (0·6-1·6)	0·9 (0·6-1·6)	0·8 (0·5-1·4)	27·4 (21·7-32·9)	29·4 (23·3-36·7)	29·7 (23·6-36·7)	33·7 (21·7-48·2)
Israel	0·0 (0·0-0·0)	0·0 (0·0-0·0)	0·0 (0·0-0·0)	0·0 (0·0-0·0)	1·5 (1·3-1·8)	1·5 (1·2-1·8)	1·5 (1·2-1·8)	1·4 (1·1-1·6)	1·3 (0·9-1·9)	1·0 (0·6-1·7)	1·0 (0·6-1·7)	0·9 (0·6-1·5)	22·3 (17·3-28·2)	28·4 (22·2-35·5)	28·6 (22·1-35·7)	35·8 (23·5-50·0)
Italy	0·03 (0·02-0·05)	0·06 (0·03-0·09)	0·1 (0·07-0·2)	0·1 (0·05-0·1)	1·3 (1·1-1·7)	1·2 (1·0-1·7)	1·2 (1·0-1·7)	1·2 (0·9-1·6)	1·2 (0·8-1·8)	1·0 (0·6-1·6)	1·0 (0·6-1·6)	0·9 (0·6-1·5)	30·9 (24·4-37·4)	34·8 (27·5-41·6)	34·8 (27·6-41·7)	41·7 (27·7-55·6)
Luxembourg	0·08 (0·04-0·1)	0·0 (0·0-0·0)	0·0 (0·0-0·0)	0·01 (0·01-0·02)	1·1 (0·9-1·7)	1·1 (0·8-1·7)	1·1 (0·8-1·7)	1·1 (0·7-1·7)	1·1 (0·7-1·7)	0·9 (0·5-1·5)	0·9 (0·5-1·5)	0·8 (0·5-1·5)	23·4 (18·1-29·5)	29·9 (23·7-37·0)	30·0 (23·9-36·8)	36·8 (24·5-50·1)
Malta	0·04 (0·01-0·08)	0·03 (0·01-0·06)	0·03 (0·0-0·05)	0·02 (0·0-0·04)	1·4 (1·2-1·7)	1·3 (1·0-1·7)	1·3 (1·0-1·7)	1·2 (0·9-1·7)	1·2 (0·8-1·8)	1·0 (0·6-1·5)	0·9 (0·6-1·5)	0·8 (0·5-1·4)	36·9 (29·8-44·2)	39·0 (31·3-46·4)	39·4 (31·9-46·7)	42·5 (28·1-57·4)
Netherlands	0·0 (0·0-0·0)	0·0 (0·0-0·0)	0·0 (0·0-0·0)	0·0 (0·0-0·0)	1·2 (0·9-1·8)	1·1 (0·8-1·7)	1·1 (0·8-1·7)	1·0 (0·7-1·5)	1·1 (0·7-1·7)	0·9 (0·5-1·5)	0·9 (0·5-1·5)	0·8 (0·5-1·4)	16·3 (12·2-20·8)	19·7 (14·7-25·3)	19·8 (14·9-25·3)	23·6 (14·1-35·4)
Norway	0·05 (0·03-0·07)	0·09 (0·04-0·1)	0·08 (0·03-0·1)	0·08 (0·04-0·1)	1·2 (0·9-1·7)	1·1 (0·8-1·7)	1·1 (0·8-1·7)	1·1 (0·7-1·7)	1·1 (0·7-1·7)	0·9 (0·5-1·6)	0·9 (0·5-1·6)	0·8 (0·5-1·5)	20·4 (15·9-25·3)	26·3 (20·3-32·7)	26·5 (20·1-33·0)	32·5 (20·7-45·5)
Portugal	0·02 (0·01-0·03)	0·04 (0·02-0·06)	0·03 (0·01-0·05)	0·03 (0·01-0·04)	1·7 (1·4-2·0)	1·5 (1·3-1·8)	1·5 (1·2-1·8)	1·3 (1·1-1·6)	1·3 (0·9-1·9)	1·1 (0·6-1·7)	1·0 (0·6-1·7)	1·0 (0·6-1·6)	29·2 (23·3-35·5)	31·6 (25·0-39·0)	31·7 (25·2-38·8)	33·7 (22·1-47·1)
Spain	0·04 (0·02-0·07)	0·0 (0·0-0·0)	0·0 (0·0-0·0)	0·0 (0·0-0·0)	1·2 (1·0-1·6)	1·1 (0·8-1·5)	1·1 (0·8-1·5)	1·0 (0·7-1·5)	1·5 (1·1-2·2)	1·4 (1·0-2·1)	1·4 (1·0-2·1)	1·3 (0·9-2·0)	31·4 (24·7-39·0)	34·2 (27·5-41·3)	34·3 (27·5-41·3)	37·9 (25·1-51·1)

Location	Indicator 3.1.1: Maternal mortality ratio (maternal deaths per 100,000 livebirths) in women aged 10-54 years				Indicator 3.1.2: Proportion of births attended by skilled health personnel (%)				Indicator 3.2.1: Under-5 mortality rate (probability of dying before the age of 5 per 1,000 livebirths)				Indicator 3.2.2: Neonatal mortality rate (probability of dying during the first 28 days of life per 1,000 livebirths)			
	2000	2015	2016	2030	2000	2015	2016	2030	2000	2015	2016	2030	2000	2015	2016	2030
**High-income North America**																
Canada	7·0 (6·3-7·7)	8·2 (7·1-9·2)	8·0 (6·9-9·1)	6·8 (4·3-10·8)	98·9 (98·5-99·2)	99·2 (98·9-99·5)	99·3 (99·0-99·5)	99·5 (99·0-99·8)	6·2 (6·0-6·3)	5·4 (5·3-5·6)	5·4 (5·2-5·6)	4·6 (4·1-5·3)	3·5 (3·4-3·6)	3·2 (3·1-3·3)	3·1 (3·1-3·2)	2·8 (2·5-3·2)
United States	15·5 (14·7-16·2)	29·4 (27·3-31·5)	28·7 (26·4-30·9)	45·0 (38·1-52·7)	98·2 (97·6-98·7)	99·1 (98·7-99·3)	99·1 (98·7-99·3)	99·4 (98·9-99·8)	8·2 (8·1-8·3)	6·9 (6·8-7·1)	6·8 (6·7-7·0)	5·7 (5·1-6·2)	4·6 (4·4-4·7)	3·9 (3·8-4·1)	3·9 (3·7-4·1)	3·3 (2·8-3·9)
**Australasia**																
Australia	7·3 (6·4-8·1)	5·3 (4·7-6·1)	5·3 (4·5-6·2)	4·7 (2·4-8·1)	99·0 (98·5-99·3)	99·4 (99·0-99·6)	99·4 (99·1-99·6)	99·6 (99·1-99·9)	6·4 (6·2-6·6)	4·0 (3·7-4·3)	3·9 (3·6-4·2)	2·8 (2·1-3·5)	3·5 (3·4-3·7)	2·3 (2·1-2·4)	2·2 (2·1-2·4)	1·5 (1·2-1·9)
New Zealand	9·9 (8·7-11·2)	11·8 (10·0-13·7)	11·9 (9·6-14·4)	13·4 (5·8-24·2)	98·9 (98·4-99·3)	99·4 (99·1-99·7)	99·5 (99·2-99·7)	99·7 (99·2-99·9)	7·5 (7·3-7·8)	5·7 (5·4-6·1)	5·6 (5·2-6·0)	3·9 (3·2-4·7)	3·5 (3·3-3·7)	3·2 (3·1-3·4)	3·1 (2·9-3·3)	3·1 (2·5-3·9)
**High-income Asia Pacific**																
Brunei	40·0 (30·6-51·1)	36·1 (26·8-49·8)	35·8 (25·0-52·8)	31·2 (10·5-81·5)	98·8 (98·0-99·3)	99·0 (98·3-99·5)	99·0 (98·4-99·5)	99·2 (97·6-99·9)	8·8 (8·2-9·5)	9·2 (8·2-10·4)	9·2 (8·0-10·5)	9·7 (7·3-12·9)	4·0 (3·7-4·3)	4·3 (3·9-4·8)	4·3 (3·8-4·8)	4·5 (3·4-5·8)
Japan	8·8 (8·4-9·2)	5·9 (5·4-6·3)	5·8 (5·3-6·3)	4·4 (3·3-5·8)	98·9 (98·1-99·4)	99·3 (98·7-99·6)	99·3 (98·7-99·6)	99·5 (98·4-99·9)	4·5 (4·4-4·6)	2·7 (2·5-2·9)	2·6 (2·4-2·9)	1·7 (1·3-2·2)	1·8 (1·7-1·9)	1·0 (0·9-1·0)	0·9 (0·8-1·0)	0·6 (0·4-0·7)
Singapore	13·4 (10·8-16·4)	7·0 (5·3-8·9)	6·1 (4·1-8·4)	1·9 (0·6-4·6)	99·1 (98·6-99·5)	99·5 (99·2-99·7)	99·5 (99·2-99·7)	99·7 (99·1-99·9)	3·8 (3·6-4·1)	2·4 (2·2-2·6)	2·3 (2·0-2·5)	1·5 (1·1-2·0)	1·7 (1·6-1·8)	1·0 (0·9-1·1)	1·0 (0·9-1·1)	0·6 (0·4-0·8)
South Korea	15·0 (11·8-18·5)	11·1 (7·1-16·4)	11·0 (6·5-17·1)	8·0 (1·8-21·2)	98·9 (98·1-99·4)	99·5 (99·1-99·7)	99·5 (99·1-99·7)	99·7 (99·1-100·0)	7·1 (6·9-7·2)	3·6 (3·0-4·2)	3·5 (2·8-4·4)	2·3 (1·2-3·8)	3·2 (3·1-3·4)	1·5 (1·2-1·8)	1·5 (1·2-1·9)	0·9 (0·5-1·5)
**Western Europe**																
Andorra	3·9 (2·3-5·9)	4·2 (2·4-6·3)	4·2 (2·5-6·4)	4·7 (1·6-11·0)	99·5 (99·3-99·6)	99·6 (99·6-99·7)	99·6 (99·6-99·7)	99·7 (99·5-99·8)	3·2 (2·5-4·0)	3·3 (2·6-4·3)	3·3 (2·6-4·3)	3·1 (2·0-4·8)	1·1 (0·8-1·3)	1·2 (0·9-1·4)	1·2 (0·9-1·5)	1·1 (0·7-1·7)
Austria	6·5 (5·6-7·5)	4·0 (3·4-4·7)	3·8 (3·2-4·5)	1·8 (1·0-2·9)	99·3 (99·1-99·4)	99·7 (99·6-99·7)	99·7 (99·6-99·7)	99·8 (99·7-99·9)	5·7 (5·4-6·1)	3·5 (3·0-4·0)	3·4 (2·8-4·0)	2·0 (1·1-3·4)	3·1 (2·9-3·4)	2·0 (1·7-2·2)	1·9 (1·6-2·2)	1·1 (0·6-1·9)
Belgium	9·1 (8·1-10·2)	7·0 (5·9-8·3)	6·9 (5·5-8·4)	5·5 (2·3-10·7)	98·5 (98·2-98·8)	99·3 (99·1-99·4)	99·3 (99·1-99·4)	99·6 (99·3-99·7)	5·8 (5·7-6·0)	4·0 (3·8-4·3)	3·9 (3·6-4·3)	2·8 (2·1-3·7)	3·0 (3·0-3·1)	2·0 (1·9-2·2)	2·0 (1·8-2·1)	1·3 (1·0-1·7)
Cyprus	22·0 (15·8-30·1)	9·9 (7·6-13·0)	9·9 (7·5-12·9)	5·6 (2·8-10·0)	97·4 (96·8-97·9)	99·0 (98·8-99·2)	99·1 (98·8-99·3)	99·5 (99·3-99·7)	8·1 (7·6-8·6)	3·4 (2·9-3·9)	3·3 (2·8-3·8)	1·7 (1·3-2·4)	4·7 (4·4-5·1)	1·8 (1·5-2·1)	1·7 (1·4-2·1)	0·8 (0·5-1·1)
Denmark	4·6 (3·9-5·3)	4·3 (3·5-5·2)	4·2 (3·3-5·2)	2·5 (0·9-5·5)	98·2 (97·7-98·5)	99·1 (98·8-99·3)	99·1 (98·9-99·3)	99·5 (99·3-99·7)	5·6 (5·3-5·9)	4·2 (3·7-4·7)	4·1 (3·5-4·7)	3·3 (2·2-5·0)	3·2 (3·1-3·4)	2·5 (2·3-2·8)	2·5 (2·2-2·8)	2·0 (1·4-3·1)
Finland	7·4 (6·6-8·3)	3·5 (3·0-4·1)	3·4 (2·8-4·1)	2·0 (0·5-4·3)	99·2 (99·0-99·4)	99·7 (99·6-99·7)	99·7 (99·6-99·7)	99·8 (99·8-99·9)	4·2 (3·9-4·6)	2·2 (1·9-2·5)	2·2 (1·8-2·7)	1·3 (0·7-2·3)	2·4 (2·2-2·6)	1·2 (1·0-1·3)	1·2 (1·0-1·4)	0·6 (0·3-1·1)
France	10·5 (9·4-11·7)	7·6 (6·4-8·8)	7·5 (6·2-8·8)	5·6 (3·1-9·2)	99·1 (98·9-99·3)	99·6 (99·5-99·7)	99·6 (99·6-99·7)	99·8 (99·7-99·9)	5·4 (5·2-5·6)	3·9 (3·4-4·4)	3·8 (3·2-4·6)	2·9 (1·8-4·6)	2·8 (2·7-2·9)	2·1 (1·9-2·3)	2·1 (1·7-2·5)	1·7 (1·0-2·6)
Germany	11·0 (9·7-12·7)	8·3 (6·9-9·9)	8·2 (6·7-9·9)	6·4 (3·6-10·3)	98·4 (98·0-98·7)	99·1 (98·9-99·3)	99·2 (99·0-99·3)	99·5 (99·2-99·7)	5·4 (5·2-5·5)	3·7 (3·3-4·2)	3·6 (3·0-4·4)	2·5 (1·6-4·1)	2·8 (2·7-2·9)	2·0 (1·7-2·2)	1·9 (1·6-2·3)	1·4 (0·9-2·2)
Greece	6·5 (5·7-7·3)	8·9 (7·5-10·3)	8·8 (7·4-10·4)	8·6 (3·7-15·6)	98·6 (98·2-98·9)	99·2 (99·0-99·4)	99·2 (99·1-99·4)	99·6 (99·3-99·7)	6·3 (6·1-6·6)	4·0 (3·7-4·3)	3·9 (3·6-4·3)	2·8 (2·0-4·0)	3·8 (3·6-4·0)	2·3 (2·1-2·4)	2·2 (2·0-2·4)	1·6 (1·1-2·2)
Iceland	1·1 (1·0-1·3)	1·9 (1·6-2·2)	2·2 (1·8-2·6)	2·3 (1·9-2·9)	99·2 (99·0-99·4)	99·6 (99·5-99·7)	99·6 (99·5-99·7)	99·8 (99·7-99·9)	4·0 (3·5-4·6)	2·3 (2·0-2·7)	2·2 (1·9-2·6)	1·3 (0·9-1·9)	1·9 (1·7-2·2)	1·0 (0·9-1·2)	1·0 (0·8-1·1)	0·5 (0·3-0·8)
Ireland	3·7 (3·2-4·3)	5·2 (4·2-6·4)	5·1 (4·0-6·5)	4·2 (1·5-9·8)	98·6 (98·2-98·9)	99·4 (99·3-99·6)	99·5 (99·3-99·6)	99·8 (99·6-99·8)	7·2 (6·9-7·7)	4·0 (3·5-4·5)	3·9 (3·4-4·5)	2·9 (1·8-4·4)	4·1 (3·8-4·3)	2·3 (2·0-2·6)	2·2 (1·9-2·6)	1·6 (1·0-2·5)
Israel	7·1 (5·8-8·7)	6·1 (4·6-8·0)	6·0 (4·3-8·1)	2·4 (0·7-6·5)	97·7 (97·2-98·2)	99·0 (98·8-99·2)	99·0 (98·8-99·2)	99·5 (99·3-99·7)	7·1 (6·8-7·4)	3·8 (3·4-4·2)	3·6 (3·1-4·1)	1·7 (1·2-2·4)	3·7 (3·5-3·9)	1·8 (1·7-2·0)	1·8 (1·6-2·0)	0·9 (0·6-1·2)
Italy	6·1 (5·5-6·9)	3·9 (3·4-4·5)	3·9 (3·3-4·6)	3·5 (1·9-5·7)	98·7 (98·3-99·0)	99·3 (99·2-99·5)	99·3 (99·2-99·5)	99·6 (99·3-99·7)	5·3 (5·2-5·5)	3·3 (2·7-4·0)	3·2 (2·5-4·0)	2·1 (1·1-3·4)	3·4 (3·3-3·5)	1·9 (1·6-2·3)	1·9 (1·5-2·3)	1·2 (0·7-1·9)
Luxembourg	8·2 (7·1-9·4)	9·8 (8·2-11·7)	9·7 (7·9-11·5)	7·6 (3·7-14·1)	98·8 (98·5-99·0)	99·4 (99·3-99·5)	99·4 (99·3-99·5)	99·7 (99·5-99·8)	4·7 (4·2-5·2)	2·3 (1·9-2·7)	2·2 (1·8-2·6)	1·0 (0·6-1·5)	2·4 (2·2-2·7)	1·2 (1·0-1·3)	1·1 (0·9-1·3)	0·5 (0·3-0·8)
Malta	7·2 (6·0-8·6)	5·5 (4·2-7·1)	5·4 (3·9-7·3)	1·2 (0·2-3·5)	98·4 (98·0-98·7)	99·2 (99·0-99·3)	99·2 (99·0-99·4)	99·6 (99·4-99·7)	7·8 (7·0-8·5)	6·4 (5·6-7·5)	6·3 (5·4-7·3)	4·6 (2·7-7·1)	5·2 (4·8-5·8)	4·5 (4·0-5·2)	4·4 (3·8-5·1)	3·1 (1·9-4·7)
Netherlands	13·3 (11·5-15·2)	6·0 (5·1-7·0)	6·0 (4·9-7·3)	3·9 (2·1-6·7)	98·9 (98·7-99·1)	99·5 (99·4-99·6)	99·5 (99·4-99·6)	99·8 (99·6-99·9)	6·3 (6·1-6·6)	3·9 (3·7-4·1)	3·8 (3·4-4·2)	2·8 (2·0-3·7)	3·8 (3·7-4·0)	2·4 (2·3-2·5)	2·3 (2·2-2·5)	1·6 (1·3-2·1)
Norway	5·2 (4·5-6·1)	3·7 (3·0-4·4)	3·7 (3·0-4·5)	2·3 (0·9-5·2)	98·7 (98·4-99·0)	99·4 (99·3-99·6)	99·5 (99·4-99·6)	99·8 (99·6-99·9)	4·9 (4·6-5·2)	2·8 (2·5-3·2)	2·7 (2·3-3·2)	1·7 (1·1-2·7)	2·7 (2·5-2·9)	1·6 (1·4-1·8)	1·5 (1·3-1·7)	0·9 (0·6-1·4)
Portugal	11·0 (9·7-12·3)	10·6 (9·1-12·2)	10·2 (8·7-11·9)	10·2 (6·4-15·1)	98·6 (98·3-98·9)	99·5 (99·4-99·6)	99·5 (99·4-99·6)	99·8 (99·7-99·9)	7·0 (6·8-7·2)	3·3 (3·1-3·5)	3·1 (2·9-3·4)	1·6 (1·3-1·9)	3·6 (3·6-3·7)	1·6 (1·5-1·7)	1·5 (1·5-1·6)	0·8 (0·6-0·9)
Spain	6·6 (5·9-7·4)	5·0 (4·4-5·7)	5·0 (4·3-5·8)	3·4 (2·4-4·8)	88·4 (86·2-90·3)	94·9 (93·7-96·0)	95·1 (93·9-96·2)	97·6 (96·4-98·5)	5·3 (5·1-5·5)	3·3 (3·0-3·6)	3·3 (2·8-3·8)	2·2 (1·5-3·1)	2·9 (2·8-3·0)	1·7 (1·6-1·9)	1·7 (1·5-1·9)	1·1 (0·7-1·5)

Supplementary Table 3. Unscaled values for the 37 individual health-related indicators, by country, in 2000, 2015, 2016, and 2030. Projected estimates for 2030 were produced based on past trends and rates of change observed from 1990 to 2016.

Location	Indicator 3.3.1: Age-standardised rate of new HIV infections (per 1,000 population)				Indicator 3.3.2: Age-standardised rate of tuberculosis cases (per 100,000 population)				Indicator 3.3.3: Age-standardised rate of malaria cases (per 1,000 population)				Indicator 3.3.4: Age-standardised rate of hepatitis B incidence (per 100,000 population)			
	2000	2015	2016	2030	2000	2015	2016	2030	2000	2015	2016	2030	2000	2015	2016	2030
**High-income North America**																
Canada	0·1 (0·05-0·2)	0·1 (0·02-0·2)	0·1 (0·02-0·2)	0·09 (0·02-0·2)	5·3 (4·8-5·8)	4·3 (3·9-4·8)	4·4 (4·0-4·8)	3·8 (3·5-4·2)	0·0 (0·0-0·0)	0·0 (0·0-0·0)	0·0 (0·0-0·0)	0·0 (0·0-0·0)	369·4 (282·0-485·0)	178·0 (141·5-226·7)	178·5 (141·9-227·0)	183·8 (147·3-228·6)
United States	0·2 (0·1-0·3)	0·1 (0·04-0·2)	0·1 (0·04-0·2)	0·1 (0·03-0·2)	5·1 (4·7-5·6)	3·3 (3·1-3·6)	3·3 (3·1-3·6)	3·2 (2·9-3·5)	0·0 (0·0-0·0)	0·0 (0·0-0·0)	0·0 (0·0-0·0)	0·0 (0·0-0·0)	597·3 (430·6-779·2)	134·7 (104·5-168·2)	135·0 (104·7-168·8)	138·7 (107·1-172·9)
**Australasia**																
Australia	0·05 (0·03-0·09)	0·05 (0·03-0·08)	0·05 (0·03-0·08)	0·05 (0·02-0·08)	5·4 (4·6-6·3)	4·8 (4·1-5·5)	4·7 (4·0-5·4)	3·4 (3·0-3·9)	0·0 (0·0-0·0)	0·0 (0·0-0·0)	0·0 (0·0-0·0)	0·0 (0·0-0·0)	396·2 (307·4-503·0)	267·8 (210·0-335·8)	257·1 (202·1-321·5)	184·1 (144·7-234·5)
New Zealand	0·05 (0·02-0·07)	0·04 (0·02-0·07)	0·04 (0·02-0·07)	0·04 (0·02-0·07)	8·5 (7·2-9·9)	6·2 (5·3-7·3)	6·1 (5·2-7·2)	4·7 (4·0-5·5)	0·0 (0·0-0·0)	0·0 (0·0-0·0)	0·0 (0·0-0·0)	0·0 (0·0-0·0)	214·4 (167·3-276·8)	155·3 (122·4-196·1)	151·4 (119·5-191·2)	105·8 (84·0-134·0)
**High-income Asia Pacific**																
Brunei	0·2 (0·09-0·3)	0·2 (0·08-0·3)	0·2 (0·08-0·3)	0·1 (0·07-0·3)	96·5 (83·1-109·3)	61·1 (52·4-69·5)	59·9 (51·3-68·1)	40·8 (35·3-46·2)	0·0 (0·0-0·0)	0·0 (0·0-0·0)	0·0 (0·0-0·0)	0·0 (0·0-0·0)	1,590·3 (1,197·2-2,086·2)	352·4 (278·5-445·2)	347·9 (275·0-438·8)	288·3 (228·6-361·2)
Japan	0·01 (0·009-0·02)	0·02 (0·008-0·02)	0·02 (0·008-0·02)	0·01 (0·007-0·02)	24·0 (20·6-27·4)	12·4 (11·1-13·7)	12·1 (10·8-13·4)	8·0 (7·2-8·8)	0·0 (0·0-0·0)	0·0 (0·0-0·0)	0·0 (0·0-0·0)	0·0 (0·0-0·0)	811·1 (613·5-1,036·2)	239·5 (196·3-293·7)	237·7 (195·2-291·1)	214·5 (177·2-260·6)
Singapore	0·1 (0·05-0·2)	0·1 (0·05-0·2)	0·1 (0·05-0·2)	0·1 (0·04-0·2)	49·4 (43·1-56·2)	34·4 (30·0-38·9)	34·7 (30·4-39·4)	31·0 (27·2-35·2)	0·0 (0·0-0·0)	0·0 (0·0-0·0)	0·0 (0·0-0·0)	0·0 (0·0-0·0)	341·0 (275·1-418·5)	147·7 (124·1-178·3)	142·8 (120·1-172·0)	85·5 (71·7-103·8)
South Korea	0·08 (0·03-0·2)	0·02 (0·003-0·05)	0·02 (0·003-0·05)	0·02 (0·002-0·04)	163·5 (144·4-184·4)	72·7 (64·3-81·5)	70·9 (62·8-79·6)	33·9 (29·9-38·1)	0·06 (0·06-0·07)	0·01 (0·01-0·01)	0·009 (0·009-0·01)	0·006 (0·006-0·006)	472·6 (384·8-570·9)	253·7 (209·5-306·3)	249·3 (206·1-300·8)	194·4 (163·5-230·8)
**Western Europe**																
Andorra	0·06 (0·006-0·2)	0·06 (0·007-0·2)	0·06 (0·006-0·2)	0·06 (0·006-0·2)	14·9 (10·9-20·0)	10·1 (7·5-13·6)	10·0 (7·4-13·4)	7·9 (5·7-10·7)	0·0 (0·0-0·0)	0·0 (0·0-0·0)	0·0 (0·0-0·0)	0·0 (0·0-0·0)	104·7 (79·7-135·6)	87·0 (67·1-111·4)	86·7 (66·9-110·9)	80·8 (62·1-103·7)
Austria	0·1 (0·05-0·2)	0·1 (0·05-0·2)	0·1 (0·05-0·2)	0·1 (0·04-0·2)	11·7 (9·0-15·3)	7·3 (5·6-9·4)	7·3 (5·5-9·4)	4·3 (3·4-5·4)	0·0 (0·0-0·0)	0·0 (0·0-0·0)	0·0 (0·0-0·0)	0·0 (0·0-0·0)	150·1 (112·6-201·6)	64·7 (51·9-80·7)	64·3 (51·7-80·4)	59·6 (48·8-74·0)
Belgium	0·1 (0·05-0·2)	0·1 (0·04-0·2)	0·1 (0·04-0·2)	0·1 (0·04-0·2)	11·2 (8·7-14·2)	8·6 (6·7-11·0)	8·4 (6·6-10·8)	6·7 (5·1-8·7)	0·0 (0·0-0·0)	0·0 (0·0-0·0)	0·0 (0·0-0·0)	0·0 (0·0-0·0)	194·2 (146·1-248·1)	109·3 (85·5-135·6)	110·2 (86·1-137·0)	122·1 (93·0-152·9)
Cyprus	0·06 (0·03-0·1)	0·1 (0·05-0·2)	0·1 (0·05-0·2)	0·08 (0·04-0·1)	14·4 (11·1-18·9)	9·4 (7·1-12·2)	9·2 (7·0-12·1)	7·3 (5·5-9·7)	0·0 (0·0-0·0)	0·0 (0·0-0·0)	0·0 (0·0-0·0)	0·0 (0·0-0·0)	203·8 (152·7-262·7)	117·5 (91·9-145·2)	115·6 (90·6-142·6)	91·1 (73·6-110·8)
Denmark	0·08 (0·04-0·1)	0·06 (0·02-0·1)	0·06 (0·02-0·1)	0·06 (0·02-0·1)	10·1 (7·8-12·8)	6·3 (4·8-8·1)	6·2 (4·7-8·1)	4·9 (3·7-6·5)	0·0 (0·0-0·0)	0·0 (0·0-0·0)	0·0 (0·0-0·0)	0·0 (0·0-0·0)	100·3 (77·8-130·8)	92·2 (71·9-118·0)	90·8 (70·9-115·9)	72·8 (57·4-89·8)
Finland	0·04 (0·02-0·06)	0·03 (0·008-0·05)	0·03 (0·008-0·05)	0·03 (0·007-0·05)	8·0 (6·3-10·0)	5·5 (4·3-7·1)	5·4 (4·2-7·1)	4·4 (3·3-5·8)	0·0 (0·0-0·0)	0·0 (0·0-0·0)	0·0 (0·0-0·0)	0·0 (0·0-0·0)	111·7 (85·6-146·0)	65·6 (52·3-82·1)	64·7 (51·6-80·8)	52·4 (42·9-64·4)
France	0·1 (0·05-0·2)	0·05 (0·02-0·09)	0·05 (0·02-0·09)	0·05 (0·02-0·09)	11·9 (9·3-15·3)	7·6 (5·9-9·7)	7·5 (5·9-9·6)	5·1 (4·1-6·4)	0·0 (0·0-0·0)	0·0 (0·0-0·0)	0·0 (0·0-0·0)	0·0 (0·0-0·0)	194·5 (149·1-249·9)	83·0 (67·8-101·6)	82·6 (67·5-100·9)	76·6 (63·2-93·2)
Germany	0·06 (0·03-0·09)	0·07 (0·03-0·1)	0·07 (0·03-0·1)	0·06 (0·03-0·1)	9·7 (7·2-12·7)	6·3 (4·9-8·1)	6·6 (5·1-8·6)	3·9 (2·8-5·6)	0·0 (0·0-0·0)	0·0 (0·0-0·0)	0·0 (0·0-0·0)	0·0 (0·0-0·0)	187·4 (134·7-248·7)	86·2 (63·6-110·3)	83·0 (61·2-106·0)	48·3 (35·8-60·9)
Greece	0·04 (0·02-0·07)	0·06 (0·03-0·09)	0·06 (0·03-0·09)	0·05 (0·02-0·08)	6·1 (4·7-7·8)	4·9 (3·8-6·3)	4·8 (3·7-6·2)	4·0 (3·1-5·2)	0·0 (0·0-0·0)	0·0 (0·0-0·0)	0·0 (0·0-0·0)	0·0 (0·0-0·0)	251·6 (201·1-310·5)	331·0 (254·8-420·8)	323·2 (249·0-410·2)	279·2 (218·0-347·3)
Iceland	0·08 (0·04-0·1)	0·07 (0·02-0·1)	0·07 (0·02-0·1)	0·06 (0·01-0·1)	14·4 (10·9-18·8)	9·3 (7·0-12·3)	9·1 (6·8-12·1)	6·9 (5·1-9·3)	0·0 (0·0-0·0)	0·0 (0·0-0·0)	0·0 (0·0-0·0)	0·0 (0·0-0·0)	82·9 (64·2-104·7)	65·1 (51·0-81·4)	64·9 (50·8-81·1)	62·1 (48·5-77·0)
Ireland	0·09 (0·03-0·2)	0·1 (0·04-0·2)	0·1 (0·04-0·2)	0·1 (0·03-0·2)	10·4 (8·1-13·2)	7·2 (5·6-9·3)	7·0 (5·4-9·0)	4·6 (3·5-6·0)	0·0 (0·0-0·0)	0·0 (0·0-0·0)	0·0 (0·0-0·0)	0·0 (0·0-0·0)	219·4 (165·6-289·1)	135·6 (104·8-172·9)	132·1 (102·4-168·7)	91·5 (73·1-113·9)
Israel	0·07 (0·03-0·1)	0·08 (0·03-0·1)	0·08 (0·03-0·1)	0·07 (0·03-0·1)	8·2 (6·4-10·3)	4·6 (3·5-5·9)	4·5 (3·4-5·8)	3·2 (2·4-4·2)	0·0 (0·0-0·0)	0·0 (0·0-0·0)	0·0 (0·0-0·0)	0·0 (0·0-0·0)	243·4 (185·2-309·6)	114·4 (91·0-140·6)	113·2 (90·2-138·9)	97·7 (78·6-117·2)
Italy	0·1 (0·06-0·2)	0·1 (0·06-0·2)	0·1 (0·05-0·2)	0·1 (0·05-0·2)	7·0 (5·8-8·4)	6·1 (5·0-7·4)	5·9 (4·8-7·1)	3·5 (2·9-4·3)	0·0 (0·0-0·0)	0·0 (0·0-0·0)	0·0 (0·0-0·0)	0·0 (0·0-0·0)	540·4 (412·3-690·3)	199·0 (156·9-245·7)	198·3 (156·4-244·9)	187·9 (149·0-228·8)
Luxembourg	0·1 (0·06-0·2)	0·1 (0·05-0·2)	0·1 (0·05-0·2)	0·1 (0·04-0·2)	12·1 (9·1-15·9)	7·6 (5·7-10·1)	7·4 (5·6-9·9)	5·7 (4·3-7·7)	0·0 (0·0-0·0)	0·0 (0·0-0·0)	0·0 (0·0-0·0)	0·0 (0·0-0·0)	178·7 (133·0-232·3)	87·6 (69·9-108·4)	86·2 (68·9-106·5)	68·5 (55·5-83·6)
Malta	0·07 (0·03-0·1)	0·2 (0·07-0·3)	0·2 (0·07-0·3)	0·1 (0·04-0·2)	8·8 (6·6-11·5)	6·3 (4·7-8·4)	6·3 (4·7-8·3)	5·2 (3·9-6·9)	0·0 (0·0-0·0)	0·0 (0·0-0·0)	0·0 (0·0-0·0)	0·0 (0·0-0·0)	180·6 (138·3-232·0)	92·6 (74·0-114·5)	90·6 (72·5-111·7)	64·9 (53·3-77·3)
Netherlands	0·08 (0·04-0·1)	0·04 (0·02-0·07)	0·04 (0·02-0·07)	0·04 (0·02-0·06)	9·0 (7·0-11·5)	5·6 (4·4-7·2)	5·5 (4·3-7·1)	3·9 (3·0-5·0)	0·0 (0·0-0·0)	0·0 (0·0-0·0)	0·0 (0·0-0·0)	0·0 (0·0-0·0)	100·8 (78·9-126·3)	85·8 (67·5-107·3)	86·5 (67·9-108·2)	93·9 (72·4-119·6)
Norway	0·07 (0·03-0·1)	0·05 (0·02-0·09)	0·05 (0·02-0·09)	0·04 (0·02-0·07)	6·7 (5·2-8·6)	6·0 (4·6-7·8)	5·9 (4·5-7·7)	4·9 (3·7-6·5)	0·0 (0·0-0·0)	0·0 (0·0-0·0)	0·0 (0·0-0·0)	0·0 (0·0-0·0)	121·2 (91·1-155·4)	79·6 (62·2-98·5)	78·1 (61·2-96·6)	59·4 (47·7-72·4)
Portugal	1·0 (0·5-1·9)	0·6 (0·2-1·0)	0·6 (0·2-1·0)	0·4 (0·1-0·6)	35·7 (27·0-46·4)	18·5 (14·3-24·0)	18·0 (13·9-23·3)	11·2 (8·7-14·6)	0·0 (0·0-0·0)	0·0 (0·0-0·0)	0·0 (0·0-0·0)	0·0 (0·0-0·0)	647·3 (476·7-856·5)	167·7 (131·2-211·5)	163·0 (127·8-204·7)	107·4 (87·8-131·9)
Spain	0·1 (0·08-0·2)	0·1 (0·07-0·2)	0·1 (0·07-0·2)	0·08 (0·04-0·1)	17·1 (12·6-22·7)	9·1 (7·1-11·8)	8·7 (6·7-11·2)	4·1 (3·2-5·5)	0·0 (0·0-0·0)	0·0 (0·0-0·0)	0·0 (0·0-0·0)	0·0 (0·0-0·0)	240·0 (181·4-307·4)	104·7 (82·2-129·2)	102·6 (80·7-126·3)	74·9 (60·5-89·3)

Location	Indicator 3.3.5: Age-standardised prevalence* of the sum of 15 neglected tropical diseases (NTDs) (%) *Prevalence estimates reported here may exceed 100% as they reflect the sum of prevalent cases of 15 NTDs.				Indicator 3.4.1: Age-standardised death rate due to cardiovascular disease, cancer, diabetes, and chronic respiratory disease in populations aged 30-70 (per 100,000 population)				Indicator 3.4.2: Age-standardised death rate due to self-harm (per 100,000 population)				Indicator 3.5.2: Risk-weighted prevalence of alcohol consumption, as measured by the summary exposure value (SEV) for alcohol use (%)			
	2000	2015	2016	2030	2000	2015	2016	2030	2000	2015	2016	2030	2000	2015	2016	2030
**High-income North America**																
Canada	0·0 (0·0-0·0)	0·0 (0·0-0·0)	0·0 (0·0-0·0)	0·0 (0·0-0·0)	299·0 (291·7-306·8)	223·8 (217·3-230·4)	221·4 (212·8-230·4)	165·1 (150·0-182·3)	12·3 (9·7-13·2)	11·0 (9·0-12·2)	10·9 (9·0-12·3)	9·9 (7·7-12·3)	15·5 (11·8-19·2)	17·5 (12·8-22·9)	17·5 (12·7-22·9)	17·4 (12·0-23·7)
United States	0·1 (0·1-0·1)	0·1 (0·1-0·1)	0·1 (0·1-0·1)	0·1 (0·1-0·1)	380·0 (376·0-383·3)	304·3 (299·6-309·0)	305·2 (299·0-311·3)	257·9 (241·0-275·6)	11·7 (10·7-14·0)	12·9 (11·0-14·4)	12·9 (10·9-14·4)	14·1 (11·0-15·8)	14·9 (11·7-18·3)	16·2 (11·5-21·4)	16·2 (11·3-21·6)	16·3 (8·9-25·1)
**Australasia**																
Australia	0·0 (0·0-0·0)	0·0 (0·0-0·0)	0·0 (0·0-0·0)	0·0 (0·0-0·0)	269·1 (262·8-275·1)	195·9 (186·6-206·0)	196·1 (183·9-210·0)	150·0 (131·2-174·0)	12·5 (10·6-13·6)	10·5 (8·9-11·9)	10·5 (8·9-12·1)	9·5 (7·1-12·1)	19·4 (15·1-24·0)	21·0 (15·3-27·7)	21·1 (15·1-28·1)	22·3 (12·4-34·3)
New Zealand	0·0 (0·0-0·0)	0·0 (0·0-0·0)	0·0 (0·0-0·0)	0·0 (0·0-0·0)	328·7 (320·3-337·4)	227·3 (213·0-242·5)	227·7 (205·7-254·4)	166·8 (135·8-199·7)	12·9 (10·6-13·9)	11·1 (9·2-12·6)	11·1 (9·0-13·1)	9·9 (6·9-13·2)	17·9 (13·9-21·9)	19·6 (14·0-25·9)	19·6 (13·9-26·0)	19·4 (12·4-28·0)
**High-income Asia Pacific**																
Brunei	0·1 (0·1-0·1)	0·3 (0·2-0·4)	0·3 (0·2-0·4)	0·3 (0·1-0·5)	423·8 (389·4-462·8)	376·1 (317·8-428·4)	373·0 (300·4-443·6)	350·0 (215·5-540·4)	4·3 (3·7-5·1)	4·7 (3·7-5·8)	4·8 (3·5-6·2)	5·1 (2·3-9·7)	0·3 (0·0-0·9)	0·3 (0·0-1·0)	0·3 (0·0-1·0)	0·7 (0·0-6·3)
Japan	0·1 (0·1-0·1)	0·1 (0·1-0·1)	0·1 (0·1-0·1)	0·1 (0·1-0·1)	239·5 (237·6-243·2)	175·0 (170·9-179·1)	172·1 (167·1-177·3)	131·6 (123·7-138·2)	19·2 (16·3-20·3)	17·4 (14·9-19·0)	17·3 (14·7-19·0)	14·9 (12·3-18·3)	11·1 (8·4-14·1)	10·7 (7·4-14·5)	10·8 (7·4-14·7)	11·8 (7·5-17·0)
Singapore	0·0 (0·0-0·1)	0·1 (0·1-0·2)	0·2 (0·1-0·3)	0·2 (0·1-0·3)	336·5 (301·1-376·9)	215·2 (186·4-245·5)	211·3 (173·0-258·3)	141·1 (83·5-230·3)	12·4 (10·7-14·8)	8·3 (6·8-11·3)	8·4 (6·5-11·7)	5·9 (3·0-10·9)	3·9 (2·5-5·5)	3·6 (2·1-5·4)	3·6 (2·1-5·4)	3·7 (2·0-6·1)
South Korea	3·1 (2·9-3·4)	2·9 (2·6-3·2)	2·9 (2·6-3·2)	2·8 (2·5-3·1)	364·2 (329·4-399·3)	214·1 (163·8-269·6)	214·9 (156·1-281·9)	131·5 (63·0-237·6)	20·7 (17·9-24·2)	25·3 (17·1-33·3)	25·1 (16·5-33·7)	22·1 (8·2-45·5)	13·3 (9·7-17·3)	12·5 (8·2-18·1)	12·5 (8·1-18·1)	11·6 (6·8-18·9)
**Western Europe**																
Andorra	0·0 (0·0-0·0)	0·0 (0·0-0·1)	0·0 (0·0-0·1)	0·0 (0·0-0·1)	227·5 (183·0-277·3)	206·2 (162·8-254·3)	206·2 (162·0-256·9)	211·4 (126·6-336·4)	8·7 (6·3-12·0)	8·2 (5·9-11·2)	8·3 (6·0-11·2)	8·6 (4·6-14·8)	30·3 (24·5-36·5)	22·2 (14·9-30·3)	21·9 (14·6-30·1)	18·4 (10·9-27·3)
Austria	0·0 (0·0-0·0)	0·0 (0·0-0·0)	0·0 (0·0-0·0)	0·0 (0·0-0·0)	318·2 (310·2-325·3)	227·8 (217·2-239·3)	224·7 (211·3-238·3)	178·1 (146·2-211·8)	17·3 (16·0-22·0)	12·6 (11·2-18·1)	12·6 (10·9-18·0)	9·9 (7·3-15·8)	22·9 (18·2-28·2)	21·1 (14·3-29·1)	21·0 (14·1-29·3)	19·7 (11·1-30·8)
Belgium	0·0 (0·0-0·0)	0·0 (0·0-0·0)	0·0 (0·0-0·0)	0·0 (0·0-0·0)	325·3 (313·4-337·8)	243·6 (225·5-262·2)	242·4 (219·2-266·7)	184·3 (143·0-226·1)	19·9 (18·3-23·2)	16·0 (13·9-19·7)	16·0 (13·7-19·8)	14·1 (9·9-20·4)	14·7 (11·2-18·8)	18·0 (12·3-25·4)	18·0 (12·2-25·6)	17·9 (10·0-29·6)
Cyprus	0·0 (0·0-0·0)	0·0 (0·0-0·0)	0·0 (0·0-0·0)	0·0 (0·0-0·0)	328·9 (316·4-341·3)	238·3 (225·4-251·8)	238·1 (224·5-251·9)	184·5 (163·7-206·6)	6·0 (5·1-7·4)	4·6 (4·1-5·7)	4·7 (4·1-5·8)	3·9 (3·0-5·0)	16·0 (12·2-20·0)	15·0 (9·8-20·9)	14·9 (9·6-21·0)	14·1 (7·6-22·6)
Denmark	0·0 (0·0-0·0)	0·0 (0·0-0·0)	0·0 (0·0-0·0)	0·0 (0·0-0·0)	384·4 (361·9-407·5)	245·1 (220·5-271·0)	242·3 (214·4-271·0)	162·8 (121·7-212·2)	14·9 (13·5-19·1)	10·0 (8·3-15·0)	10·0 (8·1-15·1)	7·0 (4·5-12·2)	25·2 (19·4-31·2)	22·2 (14·5-30·7)	21·9 (14·1-30·6)	18·3 (9·4-29·0)
Finland	0·0 (0·0-0·0)	0·0 (0·0-0·0)	0·0 (0·0-0·0)	0·0 (0·0-0·0)	305·9 (296·4-316·6)	209·7 (200·2-219·0)	204·7 (188·4-220·4)	139·7 (115·4-165·2)	22·3 (20·2-25·5)	14·2 (12·4-19·5)	14·0 (11·9-19·6)	8·8 (5·8-15·1)	22·1 (17·3-27·0)	21·7 (15·6-29·2)	21·5 (15·3-29·1)	18·9 (11·6-28·4)
France	0·0 (0·0-0·0)	0·0 (0·0-0·0)	0·0 (0·0-0·0)	0·0 (0·0-0·0)	297·3 (287·7-306·0)	230·0 (219·8-240·5)	227·2 (214·8-239·9)	181·8 (160·2-205·0)	19·6 (18·1-24·4)	15·3 (13·5-20·3)	15·3 (13·4-20·4)	12·0 (9·3-17·8)	26·2 (19·6-33·2)	22·3 (14·2-31·7)	22·1 (13·9-31·7)	19·5 (10·0-31·6)
Germany	0·0 (0·0-0·0)	0·0 (0·0-0·0)	0·0 (0·0-0·0)	0·0 (0·0-0·0)	338·6 (322·8-353·7)	250·1 (231·8-269·3)	247·0 (227·1-268·1)	199·7 (163·7-246·3)	13·4 (12·3-16·7)	10·6 (9·2-14·3)	10·5 (9·1-14·3)	9·3 (6·7-13·9)	21·0 (16·4-26·0)	18·3 (12·3-25·8)	18·2 (12·1-25·8)	16·3 (9·1-26·0)
Greece	0·0 (0·0-0·0)	0·0 (0·0-0·0)	0·0 (0·0-0·0)	0·0 (0·0-0·0)	305·0 (296·2-313·6)	255·8 (242·2-270·7)	255·4 (236·0-275·9)	224·9 (189·0-266·1)	3·3 (2·9-5·2)	3·7 (3·2-4·9)	3·7 (3·1-5·0)	4·1 (2·9-5·6)	20·0 (15·6-25·0)	18·7 (12·4-25·7)	18·6 (12·2-25·7)	16·8 (9·0-26·2)
Iceland	0·0 (0·0-0·0)	0·0 (0·0-0·0)	0·0 (0·0-0·0)	0·0 (0·0-0·0)	269·3 (256·3-282·5)	197·0 (182·9-211·2)	197·5 (180·9-215·1)	169·2 (132·6-219·3)	12·7 (11·6-15·8)	10·9 (9·6-13·7)	10·8 (9·3-13·4)	9·9 (7·5-13·6)	8·9 (6·5-11·7)	11·9 (7·7-17·1)	12·1 (7·8-17·5)	14·3 (8·2-23·0)
Ireland	0·0 (0·0-0·0)	0·0 (0·0-0·0)	0·0 (0·0-0·0)	0·0 (0·0-0·0)	373·2 (354·0-393·1)	229·7 (209·4-254·3)	229·0 (202·2-260·6)	162·8 (118·7-211·8)	12·6 (9·1-14·1)	10·0 (7·2-11·9)	9·9 (7·1-12·2)	9·3 (5·6-14·3)	27·7 (22·5-33·5)	25·4 (17·1-34·7)	25·1 (16·6-34·6)	20·9 (11·3-33·1)
Israel	0·1 (0·0-0·1)	0·0 (0·0-0·1)	0·0 (0·0-0·0)	0·0 (0·0-0·0)	307·4 (275·7-339·6)	202·9 (173·6-235·8)	202·6 (168·5-245·8)	138·2 (83·4-209·4)	9·9 (8·5-11·7)	7·7 (6·1-10·7)	7·7 (5·8-10·8)	6·2 (3·2-11·4)	3·3 (2·2-4·5)	4·5 (2·9-6·4)	4·6 (2·9-6·6)	5·8 (3·3-8·9)
Italy	0·0 (0·0-0·0)	0·0 (0·0-0·0)	0·0 (0·0-0·0)	0·0 (0·0-0·0)	282·1 (274·9-288·0)	206·2 (195·1-217·8)	205·5 (192·3-219·9)	166·5 (142·5-190·7)	6·9 (6·3-9·2)	5·7 (4·8-8·0)	5·7 (4·7-8·0)	5·1 (3·7-7·5)	21·4 (15·9-27·3)	17·1 (10·6-24·7)	16·9 (10·3-24·7)	14·6 (7·0-24·8)
Luxembourg	0·0 (0·0-0·0)	0·0 (0·0-0·0)	0·0 (0·0-0·0)	0·0 (0·0-0·0)	316·6 (303·1-329·2)	214·2 (194·7-236·1)	211·5 (190·3-236·0)	155·7 (120·5-206·6)	13·4 (12·2-16·6)	9·4 (8·0-13·6)	9·4 (7·8-13·7)	7·3 (4·7-12·7)	22·9 (18·3-28·1)	31·6 (23·5-39·7)	31·3 (23·2-39·6)	27·2 (18·1-36·6)
Malta	0·0 (0·0-0·0)	0·0 (0·0-0·0)	0·0 (0·0-0·0)	0·0 (0·0-0·0)	337·0 (309·8-366·8)	218·9 (189·2-253·5)	218·0 (180·9-258·6)	163·5 (102·4-247·7)	5·5 (4·8-7·2)	4·7 (3·8-6·5)	4·7 (3·6-6·6)	4·1 (2·1-7·4)	8·2 (5·9-10·9)	9·7 (6·1-14·3)	9·8 (6·1-14·6)	12·1 (6·1-20·3)
Netherlands	0·0 (0·0-0·0)	0·0 (0·0-0·0)	0·0 (0·0-0·0)	0·0 (0·0-0·0)	342·0 (328·7-356·6)	227·4 (216·7-240·0)	227·6 (210·0-250·1)	168·7 (138·2-204·4)	10·5 (9·7-13·0)	9·3 (8·1-11·1)	9·2 (7·8-11·1)	8·4 (5·9-11·3)	14·7 (11·3-18·2)	14·1 (9·6-19·6)	14·1 (9·5-19·9)	14·5 (7·8-24·1)
Norway	0·0 (0·0-0·0)	0·0 (0·0-0·0)	0·0 (0·0-0·0)	0·0 (0·0-0·0)	296·5 (285·4-309·4)	202·0 (185·9-218·2)	201·3 (180·2-222·4)	143·7 (112·0-177·7)	12·7 (11·5-14·7)	9·8 (8·5-12·5)	9·8 (8·3-12·4)	8·8 (6·0-13·0)	11·4 (8·3-14·7)	12·8 (8·4-18·5)	12·8 (8·4-18·6)	12·8 (7·7-19·8)
Portugal	1·0 (0·7-1·3)	0·8 (0·6-1·1)	0·8 (0·6-1·1)	0·8 (0·6-1·1)	338·5 (328·1-348·1)	235·0 (221·2-249·1)	232·8 (216·8-250·4)	164·2 (140·4-194·3)	11·3 (10·2-15·1)	9·7 (8·5-12·0)	9·7 (8·3-12·0)	8·9 (6·2-12·1)	28·4 (23·9-32·6)	25·6 (18·7-32·0)	25·4 (18·3-31·9)	22·4 (13·2-31·4)
Spain	0·2 (0·1-0·3)	0·3 (0·2-0·3)	0·3 (0·2-0·3)	0·2 (0·2-0·3)	282·7 (276·2-288·8)	204·7 (197·1-213·1)	201·8 (192·0-212·3)	149·3 (133·6-167·5)	7·3 (6·7-9·6)	6·0 (5·3-8·0)	6·0 (5·2-7·9)	5·0 (3·6-7·2)	27·1 (22·1-31·8)	25·6 (17·6-33·0)	25·3 (17·2-33·0)	22·1 (12·0-32·3)

Location	Indicator 3.6.1: Age-standardised death rate due to road injuries (per 100,000 population)				Indicator 3.7.1: Proportion of women of reproductive age (15-49 years) who have their need for family planning satisfied with modern contraception methods (%)				Indicator 3.7.2: Number of livebirths per 1,000 women aged 10-14 years and women aged 15-19 years				Indicator 3.8.1: Coverage of essential health services, as defined by the UHC index comprised of the coverage of 9 tracer interventions and risk-standardised death rates from 32 causes amenable to personal healthcare (scale of 0 to 100)			
	**2000**	**2015**	**2016**	**2030**	**2000**	**2015**	**2016**	**2030**	**2000**	**2015**	**2016**	**2030**	**2000**	**2015**	**2016**	**2030**
**High-income North America**																
Canada	10·4 (9·9-11·1)	7·1 (6·7-7·8)	7·0 (6·6-7·7)	5·7 (4·7-7·0)	85·5 (81·9-88·9)	85·4 (81·0-89·1)	85·5 (81·1-89·3)	86·6 (77·5-93·7)	8·2 (8·1-8·4)	5·4 (4·6-6·2)	5·1 (4·3-6·0)	2·6 (1·8-3·9)	75·0 (73·9-76·0)	79·2 (77·9-80·5)	79·4 (78·1-80·8)	82·1 (80·3-83·8)
United States	16·4 (15·9-17·1)	12·7 (12·2-13·2)	12·8 (12·3-13·3)	10·9 (10·0-11·8)	83·3 (80·7-85·5)	76·3 (70·5-81·3)	76·5 (70·4-81·7)	79·5 (61·8-92·5)	23·3 (23·1-23·6)	9·9 (9·6-10·3)	8·7 (8·1-9·4)	3·8 (3·3-4·4)	70·0 (69·0-70·9)	72·6 (71·4-73·5)	72·6 (71·4-73·5)	72·4 (71·0-73·6)
**Australasia**																
Australia	10·6 (10·1-11·1)	6·2 (5·7-6·7)	6·2 (5·6-6·8)	3·7 (3·1-4·3)	90·3 (86·9-92·9)	91·6 (88·5-94·0)	91·7 (88·4-93·9)	93·0 (87·4-96·4)	8·8 (8·7-8·9)	6·7 (6·4-7·0)	6·5 (6·0-6·9)	4·6 (4·1-5·4)	74·7 (73·5-75·8)	81·5 (79·9-83·1)	81·9 (80·2-83·5)	86·2 (84·1-88·0)
New Zealand	13·0 (12·3-13·8)	7·4 (6·8-8·2)	7·5 (6·6-8·4)	4·4 (3·5-5·3)	84·0 (79·3-88·2)	86·2 (82·1-89·8)	86·3 (82·6-89·8)	88·4 (80·3-93·8)	13·8 (13·6-14·0)	11·4 (10·5-12·3)	11·2 (9·9-12·6)	7·2 (5·2-9·6)	70·7 (69·4-71·8)	77·6 (75·6-79·5)	77·9 (75·9-80·0)	82·7 (79·8-85·3)
**High-income Asia Pacific**																
Brunei	19·6 (17·4-21·9)	14·6 (11·9-17·3)	14·5 (11·3-18·0)	13·0 (6·9-23·1)	71·6 (64·0-78·0)	76·9 (70·3-82·6)	77·1 (70·6-82·5)	82·1 (70·7-90·7)	13·5 (13·2-13·7)	10·6 (10·4-10·8)	10·6 (10·3-10·8)	8·8 (8·3-9·3)	61·3 (59·6-63·1)	64·5 (61·7-67·6)	64·5 (61·6-67·8)	65·0 (59·3-71·1)
Japan	8·3 (8·1-8·6)	4·1 (3·9-4·4)	4·1 (3·9-4·4)	2·3 (1·9-2·6)	62·9 (56·9-68·5)	63·1 (55·4-70·7)	63·4 (55·9-71·0)	67·6 (50·7-83·6)	2·8 (2·8-2·9)	2·0 (1·8-2·1)	1·8 (1·7-2·0)	1·3 (1·1-1·5)	75·1 (73·8-76·0)	82·4 (80·9-83·5)	82·9 (81·4-84·1)	88·0 (86·3-89·4)
Singapore	6·3 (5·5-7·2)	3·6 (3·1-4·3)	3·6 (2·9-4·5)	2·3 (1·2-4·0)	67·7 (60·6-74·4)	74·0 (67·0-79·9)	74·3 (67·5-80·1)	79·2 (66·8-87·8)	3·8 (3·7-3·9)	1·9 (1·7-2·1)	1·8 (1·5-2·2)	1·3 (0·8-2·1)	71·0 (69·1-72·8)	80·7 (77·7-83·5)	81·2 (78·1-84·1)	87·5 (83·0-91·2)
South Korea	26·4 (23·9-29·2)	13·1 (10·1-16·2)	13·1 (9·8-16·9)	8·2 (3·9-14·8)	78·1 (72·8-82·9)	82·9 (77·8-87·2)	83·1 (78·0-87·5)	85·9 (76·9-92·4)	1·4 (1·4-1·5)	0·8 (0·7-0·9)	0·8 (0·6-0·9)	0·4 (0·2-0·6)	68·9 (66·7-70·7)	80·5 (76·5-84·2)	81·1 (76·7-85·0)	87·4 (80·6-93·2)
**Western Europe**																
Andorra	5·9 (4·5-7·6)	4·5 (3·5-5·8)	4·5 (3·4-5·8)	3·8 (2·2-6·1)	83·7 (79·0-88·0)	85·1 (81·0-89·1)	85·2 (81·0-89·2)	87·3 (79·0-93·6)	4·2 (4·0-4·5)	3·2 (3·1-3·4)	3·2 (3·0-3·4)	2·6 (2·1-3·0)	79·0 (75·7-82·3)	81·4 (78·0-84·9)	81·3 (77·9-84·9)	80·1 (75·2-84·8)
Austria	11·0 (9·9-11·7)	5·0 (4·3-5·5)	5·0 (4·3-5·5)	2·3 (1·8-2·8)	75·2 (69·0-80·7)	83·7 (78·9-87·7)	83·8 (78·8-88·0)	85·9 (77·1-92·1)	6·9 (6·8-7·0)	3·4 (3·2-3·6)	3·1 (2·8-3·4)	1·4 (1·2-1·6)	72·8 (71·6-73·9)	81·4 (79·9-83·0)	81·9 (80·4-83·6)	87·7 (85·8-89·8)
Belgium	15·9 (14·5-16·8)	7·9 (7·0-8·7)	7·9 (6·9-8·9)	4·4 (3·4-5·7)	81·6 (76·5-86·2)	83·3 (78·2-87·4)	83·3 (78·5-87·4)	85·4 (75·9-92·1)	5·4 (5·3-5·5)	4·0 (3·7-4·3)	3·8 (3·5-4·2)	2·4 (1·7-3·5)	72·2 (71·0-73·3)	79·5 (77·7-81·4)	79·9 (78·0-81·8)	84·5 (82·0-87·1)
Cyprus	22·6 (20·2-24·3)	10·6 (9·7-11·5)	10·7 (9·8-11·6)	5·9 (4·9-7·0)	79·1 (73·5-83·9)	82·5 (77·7-87·0)	82·5 (77·9-86·9)	85·4 (75·8-91·9)	4·4 (4·3-4·5)	2·5 (2·2-2·9)	2·5 (2·1-2·9)	1·5 (0·9-2·2)	67·8 (66·1-69·3)	77·2 (75·6-78·8)	77·7 (76·1-79·4)	83·2 (80·5-85·5)
Denmark	9·8 (8·8-10·5)	4·2 (3·6-4·8)	4·3 (3·6-4·9)	2·2 (1·5-2·9)	79·7 (73·8-84·5)	81·9 (77·0-86·4)	81·9 (77·2-86·4)	83·9 (74·4-90·7)	3·5 (3·4-3·5)	2·0 (1·9-2·1)	2·0 (1·8-2·1)	1·3 (1·0-1·6)	71·7 (70·4-73·1)	79·0 (76·9-81·1)	79·5 (77·3-81·7)	84·8 (81·9-87·7)
Finland	8·6 (8·1-9·1)	4·2 (3·8-4·7)	4·1 (3·7-4·7)	1·8 (1·3-2·4)	87·8 (84·1-90·9)	89·1 (85·4-91·9)	89·1 (85·4-91·9)	90·7 (83·9-95·1)	5·1 (5·1-5·2)	3·2 (3·0-3·4)	3·1 (2·8-3·3)	1·8 (1·5-2·3)	75·5 (74·1-76·6)	84·5 (82·8-86·1)	85·3 (83·5-86·9)	91·7 (89·8-93·5)
France	13·2 (11·6-14·0)	6·3 (5·3-6·8)	6·2 (5·3-6·9)	3·4 (2·6-4·1)	83·0 (78·5-87·0)	86·4 (82·1-89·8)	86·5 (82·2-90·0)	87·5 (78·6-93·3)	5·4 (5·3-5·4)	4·4 (4·1-4·6)	4·3 (3·9-4·6)	3·3 (2·7-4·1)	72·0 (70·8-73·3)	80·1 (78·5-81·6)	80·5 (78·8-82·0)	85·3 (83·3-87·2)
Germany	9·9 (9·1-10·5)	4·7 (4·1-5·2)	4·7 (4·0-5·2)	2·4 (1·9-3·1)	79·7 (73·6-84·4)	82·8 (78·1-87·0)	83·0 (78·3-87·2)	85·1 (76·4-91·3)	6·4 (6·3-6·5)	3·4 (3·3-3·6)	3·1 (2·9-3·3)	1·5 (1·3-1·7)	72·0 (70·5-73·3)	78·9 (77·0-80·7)	79·3 (77·4-81·2)	83·7 (81·5-85·9)
Greece	17·8 (16·9-18·6)	10·9 (10·2-11·9)	11·0 (10·0-12·2)	7·0 (5·7-8·4)	55·8 (47·8-62·9)	62·8 (55·5-70·1)	62·9 (55·6-70·0)	67·4 (53·7-80·2)	5·9 (5·8-6·0)	3·5 (3·1-4·1)	3·4 (2·8-4·0)	1·7 (0·9-2·8)	73·8 (72·6-74·9)	78·3 (76·5-80·0)	78·6 (76·8-80·3)	80·9 (78·6-83·3)
Iceland	7·5 (6·8-8·0)	3·8 (3·5-4·2)	4·0 (3·6-4·5)	2·4 (1·8-3·2)	83·0 (78·2-87·1)	85·3 (80·7-89·3)	85·4 (80·9-89·2)	87·8 (78·8-93·7)	9·9 (9·7-10·0)	2·6 (2·4-2·9)	2·3 (2·1-2·6)	0·5 (0·3-0·7)	78·6 (77·3-79·7)	85·1 (83·1-86·9)	85·4 (83·4-87·2)	89·0 (86·2-91·2)
Ireland	11·0 (10·2-12·1)	4·6 (4·1-5·2)	4·6 (4·0-5·3)	2·3 (1·6-3·1)	79·8 (74·2-84·4)	83·2 (78·2-87·6)	83·4 (78·6-87·7)	86·8 (77·6-93·3)	9·9 (9·7-10·1)	4·5 (3·8-5·3)	4·2 (3·4-5·1)	1·6 (0·9-2·8)	69·6 (68·1-70·8)	79·9 (77·5-82·0)	80·3 (77·8-82·5)	85·5 (82·0-88·5)
Israel	13·4 (11·8-14·9)	7·8 (6·7-9·2)	7·8 (6·5-9·5)	4·7 (2·8-7·4)	75·7 (69·7-81·4)	77·4 (71·3-82·6)	77·6 (71·5-82·7)	80·4 (69·1-88·6)	8·7 (8·6-8·8)	4·6 (4·5-4·7)	4·3 (4·1-4·4)	2·0 (1·9-2·2)	68·0 (66·1-69·8)	76·1 (72·9-79·1)	76·5 (73·2-79·7)	81·6 (76·2-85·9)
Italy	14·4 (13·5-15·0)	8·0 (7·3-8·7)	8·0 (7·2-8·8)	4·7 (4·0-5·6)	66·8 (60·2-73·5)	75·3 (68·9-80·8)	75·4 (69·1-81·1)	77·7 (65·5-86·7)	3·5 (3·5-3·6)	2·8 (2·5-3·1)	2·7 (2·3-3·0)	1·7 (1·3-2·4)	73·7 (72·6-74·7)	80·5 (78·8-82·1)	80·8 (79·1-82·5)	84·9 (82·5-87·1)
Luxembourg	12·0 (10·9-12·8)	5·2 (4·6-5·9)	5·3 (4·5-6·0)	2·7 (1·9-3·7)	84·5 (79·6-88·5)	86·2 (82·0-89·9)	86·3 (81·9-89·8)	88·2 (79·5-94·0)	5·5 (5·4-5·6)	2·8 (2·5-3·0)	2·6 (2·3-3·0)	1·2 (0·9-1·6)	73·5 (72·2-74·7)	82·2 (80·1-84·2)	82·6 (80·4-84·6)	86·8 (83·9-89·3)
Malta	6·1 (5·4-6·8)	3·4 (2·8-4·0)	3·3 (2·7-4·1)	2·1 (1·2-3·4)	66·7 (59·2-73·4)	71·1 (63·6-77·3)	71·4 (64·2-77·4)	75·5 (62·2-85·0)	9·2 (9·0-9·4)	9·3 (8·5-10·1)	8·9 (7·8-10·1)	7·8 (6·0-10·9)	67·8 (66·2-69·4)	76·6 (73·8-79·3)	77·2 (74·3-80·1)	83·6 (79·5-87·7)
Netherlands	8·4 (7·8-8·9)	4·0 (3·7-4·4)	4·1 (3·6-4·5)	2·6 (2·0-3·3)	76·3 (70·3-81·5)	83·6 (78·7-87·7)	83·6 (78·8-87·8)	85·4 (75·4-92·4)	3·4 (3·3-3·4)	1·8 (1·6-2·1)	1·7 (1·5-2·0)	0·9 (0·6-1·3)	73·5 (72·2-74·7)	81·9 (80·0-83·8)	82·3 (80·3-84·1)	86·6 (84·1-89·1)
Norway	8·0 (7·5-8·5)	3·6 (3·2-4·0)	3·7 (3·2-4·2)	1·8 (1·4-2·5)	79·4 (74·2-84·3)	82·1 (77·1-86·4)	82·1 (77·2-86·4)	84·5 (74·7-91·5)	5·3 (5·3-5·4)	3·0 (2·8-3·1)	3·0 (2·7-3·2)	1·9 (1·5-2·3)	74·9 (73·5-76·1)	83·1 (81·1-85·0)	83·6 (81·6-85·6)	88·9 (86·5-91·3)
Portugal	20·6 (18·8-21·7)	8·6 (7·7-9·3)	8·5 (7·6-9·4)	3·4 (2·7-4·1)	75·7 (69·6-81·4)	79·2 (73·3-84·4)	79·3 (73·4-84·5)	82·2 (71·0-90·1)	11·5 (11·3-11·7)	4·5 (4·2-4·8)	4·1 (3·8-4·6)	1·7 (1·3-2·2)	67·0 (65·7-68·1)	76·5 (74·9-77·9)	76·9 (75·4-78·4)	82·3 (80·5-84·2)
Spain	13·7 (12·7-14·2)	4·7 (4·2-5·1)	4·7 (4·2-5·2)	1·7 (1·4-2·2)	79·2 (74·2-83·8)	79·8 (73·6-84·7)	79·9 (73·9-85·0)	81·9 (69·7-90·4)	5·1 (5·0-5·2)	4·0 (3·5-4·6)	3·8 (3·1-4·7)	2·5 (1·2-5·4)	73·5 (72·6-74·5)	82·1 (80·6-83·5)	82·5 (81·1-84·1)	87·8 (85·8-89·6)

Location	Indicator 3.9.1: Age-standardised death rate attributable to household air pollution and ambient air pollution (per 100,000 population)				Indicator 3.9.2: Age-standardised death rate attributable to unsafe water, sanitation, and hygiene (WaSH) (per 100,000 population)				Indicator 3.9.3: Age-standardised death rate due to unintentional poisonings (per 100,000 population)				Indicator 3.a.1: Age-standardised prevalence of daily smoking in populations aged 10 and older (%)			
	**2000**	**2015**	**2016**	**2030**	**2000**	**2015**	**2016**	**2030**	**2000**	**2015**	**2016**	**2030**	**2000**	**2015**	**2016**	**2030**
**High-income North America**																
Canada	19·4 (15·0-23·7)	12·5 (9·9-15·7)	12·5 (9·8-15·6)	9·2 (6·5-12·6)	0·3 (0·2-0·4)	0·5 (0·3-0·8)	0·5 (0·3-0·7)	0·5 (0·2-1·2)	0·3 (0·3-0·4)	0·3 (0·2-0·3)	0·2 (0·2-0·3)	0·2 (0·2-0·3)	19·1 (18·6-19·6)	13·5 (12·9-14·1)	13·4 (12·8-14·0)	10·9 (9·6-12·0)
United States	32·2 (26·4-38·0)	21·4 (17·4-25·6)	21·5 (17·5-25·6)	17·0 (13·2-23·9)	0·3 (0·2-0·5)	0·4 (0·3-0·6)	0·4 (0·3-0·6)	0·5 (0·3-0·8)	0·5 (0·4-0·8)	0·5 (0·5-0·7)	0·5 (0·5-0·7)	0·5 (0·4-0·6)	19·0 (18·9-19·2)	12·7 (12·6-12·8)	12·6 (12·5-12·7)	9·8 (9·6-10·1)
**Australasia**																
Australia	15·3 (11·6-19·4)	8·2 (6·0-10·8)	8·1 (5·9-10·7)	4·6 (2·9-7·6)	0·2 (0·2-0·3)	0·3 (0·2-0·4)	0·3 (0·2-0·4)	0·3 (0·2-0·5)	0·2 (0·2-0·3)	0·2 (0·1-0·2)	0·2 (0·1-0·2)	0·1 (0·1-0·2)	17·6 (17·1-18·3)	13·7 (12·8-14·7)	13·6 (12·6-14·6)	10·2 (8·8-11·7)
New Zealand	15·6 (11·4-20·3)	8·2 (5·7-11·3)	8·2 (5·7-11·2)	4·7 (2·6-7·7)	0·2 (0·1-0·3)	0·4 (0·2-0·6)	0·4 (0·2-0·6)	0·3 (0·2-0·6)	0·2 (0·2-0·3)	0·2 (0·2-0·2)	0·2 (0·1-0·2)	0·2 (0·1-0·3)	21·0 (20·3-21·8)	16·5 (15·3-18·0)	16·5 (15·2-18·1)	14·3 (11·8-17·1)
**High-income Asia Pacific**																
Brunei	16·1 (9·6-23·2)	16·7 (10·8-23·3)	16·7 (10·8-23·4)	17·0 (10·8-24·1)	0·7 (0·5-1·1)	0·7 (0·4-1·1)	0·7 (0·4-1·1)	0·7 (0·4-1·2)	0·5 (0·4-0·6)	0·4 (0·3-0·5)	0·4 (0·3-0·5)	0·4 (0·2-0·6)	14·8 (14·0-15·6)	12·8 (11·8-13·8)	12·7 (11·7-13·7)	10·9 (9·2-12·8)
Japan	19·0 (15·4-23·1)	13·3 (10·8-16·2)	13·1 (10·6-15·9)	9·9 (8·1-12·2)	0·6 (0·4-0·8)	0·5 (0·3-0·7)	0·5 (0·3-0·7)	0·4 (0·2-0·6)	0·3 (0·3-0·5)	0·3 (0·2-0·4)	0·3 (0·2-0·4)	0·2 (0·1-0·3)	25·5 (25·1-25·8)	22·7 (22·4-23·0)	22·7 (22·4-23·0)	20·1 (19·5-20·7)
Singapore	40·9 (29·7-53·3)	27·3 (19·2-36·9)	27·5 (19·4-37·2)	28·7 (20·8-39·8)	1·0 (0·6-1·3)	0·7 (0·4-0·9)	0·6 (0·4-0·9)	0·5 (0·3-0·8)	0·1 (0·1-0·2)	0·1 (0·1-0·1)	0·1 (0·1-0·1)	0·0 (0·0-0·1)	8·7 (8·1-9·4)	7·6 (7·0-8·3)	7·5 (6·9-8·2)	6·1 (5·1-7·3)
South Korea	41·6 (35·1-49·1)	25·5 (19·3-32·7)	25·4 (19·1-33·0)	14·5 (7·3-23·0)	0·7 (0·4-1·3)	0·6 (0·3-1·2)	0·6 (0·3-1·2)	0·6 (0·2-1·3)	1·0 (0·8-1·3)	0·5 (0·3-0·7)	0·5 (0·3-0·7)	0·3 (0·1-0·6)	25·8 (24·8-27·0)	18·8 (17·9-19·9)	18·6 (17·6-19·7)	14·5 (12·5-16·7)
**Western Europe**																
Andorra	18·5 (13·3-24·5)	15·9 (11·1-21·3)	15·9 (11·0-21·3)	13·8 (8·8-19·2)	0·2 (0·1-0·4)	0·2 (0·1-0·4)	0·2 (0·1-0·4)	0·2 (0·1-0·3)	0·2 (0·1-0·3)	0·1 (0·1-0·2)	0·1 (0·1-0·2)	0·1 (0·1-0·3)	24·7 (23·4-26·1)	21·5 (20·2-23·0)	21·4 (20·1-22·9)	19·7 (17·1-22·5)
Austria	33·8 (28·4-39·4)	20·8 (17·1-24·3)	20·3 (16·8-23·8)	13·2 (10·2-16·2)	0·1 (0·1-0·1)	0·1 (0·1-0·1)	0·1 (0·1-0·1)	0·1 (0·0-0·1)	0·2 (0·2-0·4)	0·2 (0·2-0·3)	0·2 (0·2-0·3)	0·1 (0·1-0·2)	26·8 (25·8-27·7)	24·8 (23·7-26·0)	24·7 (23·4-25·9)	23·1 (20·8-25·6)
Belgium	36·2 (29·8-42·7)	23·8 (19·4-28·8)	23·3 (19·0-28·2)	16·7 (13·3-20·7)	0·4 (0·2-0·6)	0·5 (0·3-0·8)	0·5 (0·3-0·7)	0·6 (0·3-1·1)	0·3 (0·3-0·6)	0·2 (0·1-0·4)	0·2 (0·1-0·4)	0·1 (0·1-0·3)	24·4 (23·5-25·4)	18·7 (17·9-19·5)	18·6 (17·7-19·5)	14·5 (12·9-16·1)
Cyprus	40·1 (34·1-46·3)	27·0 (22·7-31·4)	26·9 (22·7-31·3)	17·4 (12·6-24·6)	0·5 (0·3-1·0)	0·4 (0·2-0·7)	0·4 (0·2-0·7)	0·2 (0·1-0·5)	0·2 (0·2-0·2)	0·1 (0·1-0·1)	0·1 (0·1-0·1)	0·1 (0·0-0·1)	26·8 (25·5-28·2)	24·8 (23·5-26·0)	24·4 (23·1-25·6)	24·7 (22·0-27·4)
Denmark	31·9 (25·3-38·9)	19·0 (14·5-24·2)	18·5 (14·1-23·5)	12·4 (8·9-16·2)	0·3 (0·2-0·4)	0·5 (0·3-0·7)	0·4 (0·3-0·7)	0·4 (0·2-0·7)	0·3 (0·2-0·3)	0·1 (0·1-0·2)	0·1 (0·1-0·2)	0·1 (0·0-0·1)	29·2 (28·5-30·0)	17·3 (16·5-18·2)	17·3 (16·4-18·3)	10·9 (9·5-12·5)
Finland	22·1 (17·6-27·2)	10·9 (8·4-14·0)	10·2 (7·8-13·2)	4·9 (3·4-6·8)	0·3 (0·2-0·4)	0·1 (0·1-0·2)	0·1 (0·1-0·2)	0·1 (0·0-0·1)	0·4 (0·3-0·5)	0·2 (0·1-0·3)	0·2 (0·1-0·3)	0·1 (0·1-0·2)	21·1 (20·7-21·5)	16·5 (15·7-17·4)	16·6 (15·7-17·5)	13·8 (12·2-15·4)
France	20·2 (16·6-23·9)	13·0 (10·5-15·7)	12·7 (10·2-15·3)	8·6 (6·7-10·8)	0·4 (0·2-0·6)	0·3 (0·2-0·6)	0·3 (0·2-0·6)	0·3 (0·2-0·5)	0·3 (0·3-0·4)	0·2 (0·2-0·3)	0·2 (0·2-0·3)	0·1 (0·1-0·2)	28·3 (27·4-29·2)	23·9 (22·8-25·1)	23·7 (22·5-24·9)	20·6 (18·6-22·8)
Germany	33·5 (27·8-38·9)	22·0 (18·0-26·2)	21·6 (17·7-25·8)	15·4 (12·3-19·0)	0·2 (0·1-0·2)	0·3 (0·2-0·6)	0·3 (0·2-0·6)	0·3 (0·2-0·5)	0·1 (0·1-0·2)	0·1 (0·1-0·1)	0·1 (0·1-0·1)	0·1 (0·0-0·1)	24·0 (23·3-24·7)	21·2 (20·1-22·2)	21·0 (19·9-22·0)	19·0 (17·1-21·1)
Greece	34·2 (28·9-40·3)	26·0 (21·2-30·9)	25·5 (20·8-30·3)	20·0 (16·0-24·2)	0·1 (0·1-0·2)	0·1 (0·1-0·1)	0·1 (0·1-0·1)	0·1 (0·1-0·1)	0·3 (0·3-0·4)	0·2 (0·2-0·3)	0·2 (0·2-0·3)	0·1 (0·1-0·2)	33·5 (32·6-34·5)	31·1 (30·0-32·3)	30·4 (29·2-31·7)	29·3 (26·5-31·8)
Iceland	20·6 (16·0-25·7)	12·5 (9·6-15·9)	12·2 (9·3-15·5)	8·0 (5·9-10·6)	0·2 (0·1-0·3)	0·2 (0·1-0·3)	0·2 (0·1-0·3)	0·2 (0·1-0·3)	0·3 (0·3-0·4)	0·2 (0·2-0·3)	0·2 (0·1-0·3)	0·1 (0·1-0·2)	21·1 (20·1-22·0)	13·9 (13·2-14·6)	13·9 (13·2-14·7)	8·2 (7·4-9·2)
Ireland	37·1 (29·4-45·4)	18·4 (14·2-23·1)	18·1 (13·9-22·7)	11·4 (7·6-15·9)	0·4 (0·2-0·5)	0·2 (0·1-0·3)	0·2 (0·1-0·3)	0·2 (0·1-0·2)	0·4 (0·4-0·5)	0·2 (0·2-0·3)	0·2 (0·2-0·3)	0·2 (0·1-0·3)	24·2 (23·3-25·1)	19·6 (18·6-20·7)	19·3 (18·3-20·5)	16·1 (14·5-17·8)
Israel	30·6 (25·3-36·5)	19·8 (15·7-24·4)	19·7 (15·5-24·3)	11·3 (6·6-15·9)	0·5 (0·3-0·8)	0·6 (0·3-0·9)	0·6 (0·3-0·9)	0·5 (0·2-0·8)	0·2 (0·2-0·3)	0·1 (0·1-0·2)	0·1 (0·1-0·2)	0·1 (0·1-0·2)	19·6 (18·8-20·5)	16·9 (15·8-18·1)	16·8 (15·7-18·0)	13·7 (12·1-15·6)
Italy	26·4 (21·9-30·9)	18·4 (15·1-21·9)	18·2 (14·9-21·7)	13·4 (10·3-17·4)	0·1 (0·1-0·1)	0·1 (0·1-0·2)	0·1 (0·1-0·2)	0·1 (0·0-0·1)	0·3 (0·2-0·3)	0·1 (0·1-0·2)	0·1 (0·1-0·2)	0·1 (0·0-0·2)	21·0 (20·4-21·7)	19·8 (18·8-20·8)	19·5 (18·4-20·5)	17·2 (15·2-19·5)
Luxembourg	34·2 (28·6-39·9)	20·9 (17·1-25·3)	20·5 (16·8-24·8)	12·2 (8·6-16·0)	0·2 (0·2-0·4)	0·3 (0·2-0·5)	0·3 (0·2-0·5)	0·2 (0·1-0·4)	0·2 (0·2-0·4)	0·1 (0·1-0·2)	0·1 (0·1-0·2)	0·1 (0·1-0·2)	24·7 (23·5-25·8)	19·6 (18·4-21·0)	19·5 (18·3-20·9)	16·3 (14·1-18·6)
Malta	41·5 (33·4-50·1)	22·8 (17·8-28·8)	21·9 (17·0-27·7)	12·3 (8·8-16·6)	0·3 (0·2-0·4)	0·2 (0·1-0·3)	0·2 (0·1-0·2)	0·1 (0·1-0·2)	0·2 (0·2-0·3)	0·1 (0·1-0·2)	0·1 (0·1-0·2)	0·1 (0·0-0·2)	19·0 (18·0-20·0)	17·6 (16·4-18·7)	17·4 (16·2-18·6)	14·9 (12·7-17·3)
Netherlands	36·9 (30·1-44·0)	22·1 (17·7-27·0)	21·6 (17·3-26·5)	16·4 (13·0-20·4)	0·3 (0·2-0·4)	0·3 (0·2-0·5)	0·3 (0·2-0·5)	0·4 (0·2-0·7)	0·1 (0·1-0·2)	0·1 (0·1-0·1)	0·1 (0·1-0·1)	0·1 (0·0-0·1)	24·5 (23·9-25·1)	16·6 (15·8-17·4)	16·5 (15·6-17·4)	11·4 (10·2-12·6)
Norway	25·4 (20·2-30·9)	13·5 (10·3-17·2)	12·9 (9·9-16·6)	7·1 (5·1-9·6)	0·4 (0·2-0·5)	0·5 (0·3-0·8)	0·5 (0·3-0·8)	0·3 (0·2-0·7)	0·2 (0·2-0·3)	0·2 (0·1-0·2)	0·2 (0·1-0·2)	0·1 (0·1-0·2)	25·2 (24·0-26·3)	14·4 (13·5-15·3)	14·4 (13·5-15·4)	8·7 (7·5-10·1)
Portugal	34·2 (28·0-41·0)	18·0 (14·3-22·4)	17·5 (13·8-21·8)	9·8 (7·0-13·1)	0·4 (0·2-0·5)	0·4 (0·3-0·5)	0·4 (0·2-0·5)	0·3 (0·2-0·5)	0·4 (0·4-0·5)	0·2 (0·2-0·4)	0·2 (0·2-0·3)	0·1 (0·1-0·2)	19·1 (18·4-19·8)	18·5 (17·5-19·6)	18·4 (17·3-19·5)	17·6 (15·6-19·8)
Spain	24·5 (19·9-29·1)	14·5 (11·6-17·6)	14·2 (11·3-17·2)	9·8 (7·5-12·2)	0·2 (0·1-0·3)	0·2 (0·1-0·3)	0·2 (0·1-0·3)	0·1 (0·1-0·2)	0·2 (0·2-0·3)	0·1 (0·1-0·2)	0·1 (0·1-0·2)	0·1 (0·0-0·1)	27·5 (26·8-28·1)	25·1 (23·9-26·3)	24·6 (23·4-25·8)	21·5 (19·2-23·9)

Location	Indicator 3.b.1: Geometric mean of the coverage of eight vaccines, conditional on inclusion in national vaccine schedules, in target populations (%)				Indicator 5.2.1: Age-standardised prevalence of women aged 15 years and older who experienced physical or sexual violence by an intimate partner in the last 12 months (%)				Indicator 6.1.1: Risk-weighted prevalence of populations using unsafe or unimproved water sources, as measured by the summary exposure value (SEV) for unsafe water (%)				Indicator 6.2.1a: Risk-weighted prevalence of populations using unsafe or unimproved sanitation, as measured by the summary exposure value (SEV) for unsafe sanitation (%)			
	**2000**	**2015**	**2016**	**2030**	**2000**	**2015**	**2016**	**2030**	**2000**	**2015**	**2016**	**2030**	**2000**	**2015**	**2016**	**2030**
**High-income North America**																
Canada	57·7 (52·6-63·2)	71·3 (61·3-80·2)	75·0 (65·6-83·0)	94·7 (88·4-98·5)	14·7 (12·9-16·6)	13·4 (11·8-15·1)	13·3 (11·8-15·0)	12·0 (10·6-13·6)	0·9 (0·5-1·5)	0·7 (0·4-0·8)	0·7 (0·4-0·8)	0·7 (0·4-0·8)	4·8 (2·1-9·0)	3·6 (1·6-7·0)	3·6 (1·5-7·0)	2·8 (1·2-5·9)
United States	91·3 (89·7-92·5)	91·7 (88·9-94·0)	92·3 (89·2-94·7)	98·2 (94·3-99·9)	15·2 (13·5-17·0)	13·6 (12·1-15·2)	13·5 (12·1-15·2)	12·1 (10·8-13·5)	1·1 (0·7-1·3)	0·5 (0·3-0·6)	0·5 (0·3-0·6)	0·5 (0·3-0·6)	6·2 (5·3-7·2)	4·9 (4·1-5·7)	4·8 (4·1-5·6)	4·0 (3·4-4·8)
**Australasia**																
Australia	92·4 (90·2-94·3)	95·6 (93·3-96·9)	95·8 (93·4-97·3)	98·6 (95·1-99·9)	14·6 (12·8-16·6)	13·1 (11·5-14·8)	13·0 (11·4-14·7)	11·8 (10·3-13·5)	1·6 (1·0-2·1)	2·2 (1·2-3·4)	2·2 (1·2-3·4)	2·4 (1·2-3·6)	3·3 (1·4-6·4)	1·8 (0·7-3·5)	1·8 (0·7-3·3)	1·0 (0·4-1·8)
New Zealand	86·6 (83·2-89·3)	93·8 (90·7-95·9)	94·1 (90·8-96·3)	97·4 (92·8-99·5)	17·6 (15·5-19·9)	15·6 (13·8-17·5)	15·4 (13·6-17·3)	13·3 (11·8-14·9)	1·9 (1·2-2·4)	2·4 (1·4-3·6)	2·4 (1·4-3·6)	2·6 (1·3-4·1)	4·1 (1·7-8·1)	2·4 (1·0-4·9)	2·3 (0·9-4·8)	1·3 (0·5-2·8)
**High-income Asia Pacific**																
Brunei	96·6 (94·6-98·0)	97·7 (94·9-99·0)	97·8 (94·6-99·1)	98·7 (90·9-100·0)	20·8 (17·7-24·3)	17·6 (15·3-20·3)	17·4 (15·1-20·0)	14·5 (12·8-16·3)	2·3 (1·6-2·9)	2·3 (1·3-3·7)	2·4 (1·3-3·7)	2·7 (1·3-4·4)	5·2 (2·2-10·0)	2·3 (0·9-4·7)	2·2 (0·9-4·6)	1·0 (0·3-2·5)
Japan	95·6 (93·2-97·3)	97·3 (95·2-98·6)	97·2 (94·2-98·7)	97·6 (89·5-100·0)	13·6 (12·2-15·2)	11·3 (10·2-12·4)	11·2 (10·1-12·3)	9·5 (8·6-10·4)	1·6 (1·1-1·8)	2·3 (1·6-2·7)	2·3 (1·6-2·7)	2·6 (1·8-3·1)	3·1 (2·7-3·6)	2·0 (1·7-2·4)	2·0 (1·6-2·4)	1·4 (1·1-1·7)
Singapore	96·9 (95·8-97·9)	96·4 (94·9-97·3)	96·7 (95·0-97·6)	98·8 (95·8-99·8)	16·8 (14·6-19·2)	12·7 (11·1-14·3)	12·5 (11·0-14·1)	9·4 (8·3-10·6)	2·1 (1·3-2·6)	2·3 (1·3-3·4)	2·3 (1·3-3·4)	2·5 (1·4-3·6)	4·6 (2·1-8·9)	2·0 (0·8-4·1)	1·9 (0·7-3·9)	0·8 (0·3-1·7)
South Korea	88·7 (83·9-92·3)	98·6 (96·4-99·4)	98·7 (96·7-99·4)	99·7 (98·3-100·0)	19·1 (16·6-21·8)	14·8 (13·0-16·8)	14·6 (12·8-16·5)	11·5 (10·2-12·8)	2·1 (1·4-2·5)	2·3 (1·3-3·6)	2·3 (1·3-3·6)	2·5 (1·3-4·3)	4·6 (1·9-8·6)	2·1 (0·9-4·1)	2·0 (0·8-4·0)	0·9 (0·3-2·0)
**Western Europe**																
Andorra	91·3 (89·4-92·9)	96·6 (94·9-97·8)	96·7 (94·9-97·8)	98·2 (94·8-99·6)	12·8 (11·2-14·5)	11·6 (10·2-13·1)	11·6 (10·1-13·0)	10·6 (9·4-11·9)	0·6 (0·4-0·7)	0·6 (0·4-0·7)	0·6 (0·4-0·7)	0·6 (0·3-0·7)	1·7 (0·7-3·1)	1·1 (0·5-2·3)	1·1 (0·5-2·3)	0·8 (0·3-1·8)
Austria	81·9 (78·4-85·0)	91·8 (86·1-95·4)	92·6 (86·8-96·1)	98·3 (93·7-99·9)	13·6 (11·9-15·4)	11·8 (10·5-13·2)	11·7 (10·4-13·1)	10·4 (9·2-11·5)	1·1 (0·7-1·4)	0·8 (0·5-1·0)	0·8 (0·5-1·0)	0·6 (0·4-0·8)	4·6 (2·4-8·1)	3·2 (1·4-5·8)	3·1 (1·4-5·8)	2·2 (0·9-4·3)
Belgium	81·9 (79·2-84·3)	97·2 (96·4-97·8)	97·6 (96·8-98·2)	99·9 (99·7-99·9)	15·8 (13·9-17·8)	14·0 (12·4-15·7)	13·9 (12·4-15·6)	12·5 (11·1-13·9)	0·7 (0·4-0·8)	0·6 (0·4-0·8)	0·6 (0·4-0·8)	0·6 (0·4-0·8)	2·0 (0·8-3·8)	1·3 (0·5-2·6)	1·3 (0·5-2·5)	0·9 (0·4-1·9)
Cyprus	93·9 (91·7-95·4)	95·8 (94·1-97·1)	96·2 (94·3-97·5)	99·4 (97·9-100·0)	16·1 (14·2-18·3)	14·1 (12·3-16·0)	14·0 (12·3-15·9)	12·3 (10·8-14·2)	1·2 (0·7-1·5)	0·8 (0·5-1·0)	0·8 (0·5-1·0)	0·8 (0·5-0·9)	3·2 (1·4-6·3)	1·7 (0·7-3·5)	1·7 (0·7-3·4)	0·9 (0·3-2·1)
Denmark	95·0 (93·2-96·3)	93·9 (91·1-95·9)	94·4 (91·6-96·4)	97·3 (90·4-99·9)	14·4 (12·7-16·2)	12·7 (11·3-14·1)	12·6 (11·2-14·0)	11·3 (10·1-12·6)	0·6 (0·3-0·7)	0·6 (0·4-0·7)	0·6 (0·4-0·7)	0·6 (0·4-0·7)	1·6 (0·7-3·0)	1·0 (0·4-2·1)	1·0 (0·4-2·1)	0·7 (0·2-1·6)
Finland	97·5 (95·7-98·3)	97·0 (96·0-97·7)	97·4 (96·4-98·1)	99·3 (98·1-99·9)	16·0 (14·2-18·1)	13·5 (12·0-15·1)	13·3 (11·9-14·9)	11·4 (10·2-12·6)	0·6 (0·4-0·8)	0·6 (0·4-0·7)	0·6 (0·4-0·7)	0·6 (0·4-0·7)	2·1 (0·8-4·0)	1·2 (0·5-2·5)	1·2 (0·5-2·5)	0·8 (0·3-1·5)
France	74·3 (72·2-76·3)	93·1 (91·5-94·2)	93·7 (92·1-94·8)	98·5 (97·3-99·2)	15·3 (13·5-17·3)	13·2 (11·8-14·8)	13·1 (11·7-14·7)	11·4 (10·3-12·8)	0·7 (0·4-0·9)	0·7 (0·4-0·8)	0·7 (0·4-0·8)	0·7 (0·4-0·8)	2·7 (1·2-5·2)	1·7 (0·7-3·6)	1·7 (0·7-3·6)	1·2 (0·5-2·7)
Germany	80·9 (77·2-83·9)	86·3 (81·7-90·2)	83·7 (80·0-86·8)	97·3 (95·0-98·8)	13·9 (12·2-15·6)	11·7 (10·4-13·1)	11·6 (10·3-13·0)	10·0 (8·8-11·3)	0·8 (0·5-1·0)	0·6 (0·3-0·7)	0·5 (0·3-0·7)	0·4 (0·2-0·6)	2·3 (1·0-4·3)	1·4 (0·6-2·8)	1·4 (0·6-2·8)	1·0 (0·4-2·1)
Greece	93·9 (90·2-96·5)	85·0 (83·7-85·8)	87·9 (86·7-88·7)	99·0 (97·8-99·8)	15·9 (14·1-17·9)	13·6 (12·2-15·2)	13·6 (12·1-15·1)	11·8 (10·5-13·1)	0·7 (0·5-0·8)	0·7 (0·5-0·9)	0·7 (0·5-0·9)	0·7 (0·5-0·9)	3·0 (1·4-5·8)	1·8 (0·7-3·6)	1·8 (0·7-3·6)	1·0 (0·3-2·2)
Iceland	96·4 (94·2-97·9)	93·7 (91·0-95·9)	93·8 (91·1-96·1)	89·9 (76·9-96·8)	14·9 (12·9-17·1)	12·9 (11·3-14·7)	12·8 (11·2-14·5)	11·1 (9·8-12·5)	0·6 (0·4-0·8)	0·6 (0·4-0·7)	0·6 (0·4-0·7)	0·6 (0·3-0·7)	1·9 (0·8-3·7)	1·1 (0·4-2·1)	1·0 (0·4-2·1)	0·6 (0·2-1·2)
Ireland	82·4 (78·5-85·5)	93·4 (90·9-95·3)	93·7 (90·5-95·8)	97·0 (90·9-99·4)	15·6 (13·7-17·8)	13·0 (11·5-14·6)	12·8 (11·3-14·3)	10·3 (9·2-11·5)	0·8 (0·5-1·1)	0·6 (0·4-0·7)	0·6 (0·4-0·7)	0·6 (0·4-0·7)	2·8 (1·2-5·2)	1·5 (0·7-2·9)	1·5 (0·6-2·8)	1·0 (0·4-1·9)
Israel	95·1 (93·7-96·3)	94·9 (92·2-96·6)	95·3 (92·4-97·2)	96·8 (90·9-99·3)	20·0 (17·4-23·0)	17·8 (15·6-20·4)	17·8 (15·5-20·3)	16·7 (14·7-18·9)	1·4 (0·9-1·7)	1·0 (0·7-1·3)	1·0 (0·7-1·3)	0·8 (0·5-1·1)	4·7 (2·2-8·9)	3·2 (1·3-6·2)	3·1 (1·3-6·1)	2·2 (0·8-4·2)
Italy	85·4 (66·6-89·7)	95·2 (93·2-96·7)	95·3 (93·3-96·9)	96·9 (91·2-99·1)	15·3 (13·5-17·2)	13·1 (11·7-14·6)	13·0 (11·6-14·5)	11·2 (10·0-12·5)	2·2 (1·3-2·7)	1·6 (1·0-2·1)	1·6 (1·0-2·1)	1·4 (0·8-1·8)	2·5 (1·1-4·7)	1·8 (0·7-3·6)	1·7 (0·7-3·5)	1·3 (0·5-2·7)
Luxembourg	97·2 (95·4-98·2)	97·5 (96·6-98·0)	97·8 (97·0-98·4)	99·8 (99·5-100·0)	13·8 (12·0-15·7)	12·4 (10·9-14·0)	12·3 (10·8-13·9)	11·3 (10·0-12·8)	0·6 (0·4-0·7)	0·6 (0·4-0·7)	0·6 (0·4-0·7)	0·6 (0·4-0·7)	1·5 (0·6-3·0)	0·9 (0·4-1·9)	0·9 (0·4-1·8)	0·6 (0·2-1·3)
Malta	82·9 (76·2-88·0)	95·2 (91·6-97·3)	95·6 (91·9-97·7)	99·0 (95·7-100·0)	16·4 (14·4-18·6)	13·9 (12·3-15·6)	13·7 (12·1-15·4)	11·7 (10·4-13·0)	0·9 (0·6-1·2)	0·7 (0·4-0·8)	0·6 (0·4-0·8)	0·5 (0·4-0·6)	3·3 (1·4-6·3)	1·9 (0·8-3·9)	1·9 (0·8-3·8)	1·3 (0·5-2·5)
Netherlands	96·0 (94·7-97·0)	90·6 (88·6-92·0)	91·0 (88·9-92·5)	92·6 (84·9-96·9)	15·4 (13·6-17·4)	13·0 (11·6-14·5)	12·9 (11·5-14·4)	11·3 (10·2-12·7)	0·6 (0·4-0·7)	0·6 (0·4-0·7)	0·6 (0·4-0·7)	0·6 (0·3-0·7)	1·8 (0·7-3·6)	1·1 (0·5-2·2)	1·1 (0·5-2·1)	0·8 (0·3-1·6)
Norway	91·8 (89·5-93·7)	95·1 (92·9-96·7)	95·5 (93·2-97·0)	97·5 (94·0-99·3)	14·1 (12·4-16·1)	12·8 (11·3-14·5)	12·7 (11·2-14·4)	11·7 (10·3-13·1)	0·6 (0·4-0·7)	0·6 (0·4-0·7)	0·6 (0·4-0·7)	0·6 (0·4-0·7)	1·7 (0·7-3·2)	1·0 (0·4-2·0)	1·0 (0·4-1·9)	0·7 (0·3-1·1)
Portugal	92·6 (87·8-95·2)	94·9 (82·7-98·9)	95·1 (82·5-99·0)	93·0 (39·0-100·0)	17·9 (15·7-20·3)	14·4 (12·8-16·2)	14·2 (12·7-16·0)	11·9 (10·6-13·2)	1·9 (1·3-2·4)	1·1 (0·8-1·5)	1·1 (0·7-1·4)	0·8 (0·5-1·1)	10·1 (5·8-16·0)	5·9 (2·7-11·0)	5·7 (2·7-10·7)	3·6 (1·3-7·3)
Spain	94·4 (93·0-95·7)	98·0 (96·7-98·9)	98·1 (96·7-99·0)	99·1 (96·7-100·0)	14·4 (12·7-16·4)	12·0 (10·7-13·5)	11·9 (10·5-13·3)	9·9 (8·8-11·0)	0·7 (0·5-0·9)	0·7 (0·5-0·9)	0·7 (0·5-0·9)	0·7 (0·5-0·9)	2·7 (1·4-4·9)	1·1 (0·5-2·2)	1·1 (0·4-2·2)	0·6 (0·2-1·2)

Location	Indicator 6.2.1b: Risk-weighted prevalence of populations without access to a handwashing facility, as measured by the summary exposure value (SEV) for unsafe hygiene (%)				Indicator 7.1.2: Risk-weighted prevalence of household air pollution, as measured by the summary exposure value (SEV) for household air pollution (%)				Indicator 8.8.1: Age-standardised all-cause disability-adjusted life year (DALY) rates attributable to occupational risks (per 100,000 population)				Indicator 11.6.2: Population-weighted mean levels of fine particulate matter smaller than 2.5 microns in diameter (PM2.5)			
	**2000**	**2015**	**2016**	**2030**	**2000**	**2015**	**2016**	**2030**	**2000**	**2015**	**2016**	**2030**	**2000**	**2015**	**2016**	**2030**
**High-income North America**																
Canada	3·8 (3·6-4·0)	3·6 (3·4-3·8)	3·6 (3·4-3·8)	3·4 (3·2-3·7)	0·1 (0·1-0·1)	0·0 (0·0-0·1)	0·0 (0·0-0·1)	0·0 (0·0-0·0)	1,001·1 (860·4-1,154·0)	906·8 (769·6-1,054·3)	904·8 (767·1-1,052·6)	876·0 (737·3-1,027·3)	7·8 (7·6-8·0)	7·5 (7·3-7·7)	7·5 (7·3-7·7)	7·4 (6·9-7·8)
United States	3·5 (3·5-3·6)	3·2 (3·2-3·2)	3·2 (3·2-3·2)	3·0 (2·9-3·0)	0·1 (0·1-0·1)	0·1 (0·1-0·1)	0·1 (0·1-0·1)	0·1 (0·0-0·1)	906·0 (787·5-1,039·6)	752·4 (648·9-863·4)	751·6 (648·3-862·5)	740·1 (639·1-848·2)	10·7 (10·6-10·8)	9·2 (9·1-9·2)	9·2 (9·1-9·3)	8·5 (8·3-8·7)
**Australasia**																
Australia	6·3 (6·0-6·6)	5·9 (5·6-6·2)	5·9 (5·6-6·2)	5·6 (5·2-5·9)	0·2 (0·1-0·3)	0·1 (0·1-0·2)	0·1 (0·1-0·2)	0·1 (0·0-0·1)	791·9 (677·7-920·2)	741·9 (626·7-876·8)	736·2 (620·8-871·9)	660·7 (541·2-798·1)	6·8 (6·6-7·0)	6·1 (5·9-6·3)	6·1 (6·0-6·3)	6·2 (5·8-6·6)
New Zealand	6·5 (6·2-6·9)	6·1 (5·8-6·5)	6·1 (5·8-6·4)	5·7 (5·4-6·1)	0·2 (0·1-0·3)	0·2 (0·1-0·2)	0·2 (0·1-0·2)	0·1 (0·0-0·1)	955·5 (797·2-1,139·0)	887·0 (732·3-1,070·1)	884·0 (729·2-1,067·1)	841·6 (688·2-1,028·7)	6·2 (6·0-6·5)	5·5 (5·3-5·8)	5·5 (5·3-5·8)	5·5 (5·1-5·9)
**High-income Asia Pacific**																
Brunei	6·8 (6·5-7·1)	6·1 (5·8-6·4)	6·1 (5·8-6·4)	5·6 (4·9-6·0)	0·2 (0·1-0·2)	0·0 (0·0-0·0)	0·0 (0·0-0·0)	0·0 (0·0-0·0)	1,566·2 (1,189·7-1,915·8)	1,327·3 (912·8-1,691·4)	1,322·2 (907·6-1,686·7)	1,249·5 (832·1-1,602·4)	5·7 (4·8-6·7)	6·4 (5·3-7·7)	6·4 (5·3-7·8)	6·1 (4·8-7·5)
Japan	6·3 (6·2-6·3)	6·0 (5·9-6·1)	6·0 (5·9-6·0)	5·8 (5·6-6·0)	0·0 (0·0-0·0)	0·0 (0·0-0·0)	0·0 (0·0-0·0)	0·0 (0·0-0·0)	860·7 (713·5-1,023·2)	824·6 (668·1-997·8)	827·2 (668·7-1,003·2)	861·4 (673·3-1,083·3)	12·4 (12·2-12·7)	13·1 (12·9-13·4)	13·2 (12·9-13·4)	12·9 (12·6-13·2)
Singapore	6·7 (6·3-7·0)	6·0 (5·7-6·3)	6·0 (5·7-6·3)	5·4 (4·9-5·7)	0·1 (0·1-0·1)	0·0 (0·0-0·0)	0·0 (0·0-0·0)	0·0 (0·0-0·0)	1,009·2 (809·9-1,230·9)	787·0 (605·4-975·9)	779·6 (597·8-969·4)	679·2 (484·1-876·7)	19·1 (13·1-26·6)	24·9 (17·4-35·0)	25·0 (17·3-34·6)	25·8 (17·4-36·2)
South Korea	6·7 (6·3-7·0)	6·0 (5·7-6·3)	6·0 (5·7-6·3)	5·5 (5·0-5·9)	0·0 (0·0-0·0)	0·0 (0·0-0·0)	0·0 (0·0-0·0)	0·0 (0·0-0·0)	1,249·9 (1,011·5-1,551·6)	747·6 (599·6-906·7)	732·3 (586·1-889·6)	544·8 (425·0-678·5)	25·8 (24·6-27·0)	28·7 (27·6-29·8)	28·7 (27·6-29·9)	29·1 (27·9-30·4)
**Western Europe**																
Andorra	3·7 (3·4-3·9)	3·5 (3·3-3·7)	3·5 (3·3-3·7)	3·4 (3·2-3·6)	0·5 (0·4-0·7)	0·3 (0·2-0·4)	0·3 (0·2-0·3)	0·1 (0·1-0·2)	884·8 (693·2-1,113·1)	884·7 (695·0-1,120·6)	884·2 (694·6-1,119·4)	878·2 (690·2-1,105·9)	11·1 (8·2-14·8)	10·9 (8·3-14·2)	10·9 (8·2-14·1)	10·5 (7·9-13·6)
Austria	3·9 (3·7-4·1)	3·7 (3·5-3·9)	3·7 (3·5-3·9)	3·5 (3·3-3·7)	0·7 (0·6-1·0)	0·4 (0·3-0·5)	0·4 (0·3-0·5)	0·2 (0·1-0·2)	759·6 (627·5-904·9)	730·3 (589·9-885·6)	728·5 (587·0-884·9)	700·9 (542·9-874·8)	15·3 (14·9-15·7)	15·0 (14·6-15·5)	15·0 (14·5-15·5)	14·3 (12·7-15·3)
Belgium	3·7 (3·5-3·9)	3·6 (3·4-3·8)	3·6 (3·4-3·8)	3·5 (3·3-3·6)	0·3 (0·2-0·3)	0·1 (0·1-0·2)	0·1 (0·1-0·2)	0·1 (0·1-0·1)	929·4 (798·4-1,072·8)	810·2 (679·0-946·5)	804·2 (672·1-941·7)	722·2 (583·7-873·3)	16·9 (16·4-17·3)	16·0 (15·6-16·5)	16·0 (15·6-16·5)	14·5 (13·5-15·1)
Cyprus	4·0 (3·7-4·2)	3·7 (3·5-3·9)	3·7 (3·5-3·9)	3·5 (3·2-3·7)	0·6 (0·4-0·8)	0·3 (0·2-0·3)	0·2 (0·2-0·3)	0·1 (0·1-0·2)	908·3 (784·7-1,050·0)	809·8 (665·9-995·0)	795·8 (659·4-968·4)	616·3 (553·6-677·0)	15·2 (13·9-16·5)	17·9 (16·3-19·6)	17·9 (16·2-19·5)	17·9 (15·5-20·1)
Denmark	3·6 (3·4-3·8)	3·5 (3·3-3·7)	3·5 (3·3-3·7)	3·4 (3·2-3·6)	0·2 (0·1-0·2)	0·1 (0·1-0·2)	0·1 (0·1-0·1)	0·1 (0·0-0·1)	1,030·1 (875·9-1,193·8)	986·5 (801·3-1,184·7)	982·7 (795·8-1,183·1)	928·6 (720·9-1,158·3)	11·0 (10·5-11·5)	10·4 (9·9-10·9)	10·3 (9·9-10·8)	8·7 (8·3-9·2)
Finland	3·7 (3·5-3·9)	3·6 (3·4-3·8)	3·6 (3·4-3·8)	3·4 (3·2-3·6)	0·3 (0·2-0·4)	0·2 (0·1-0·2)	0·2 (0·1-0·2)	0·1 (0·1-0·1)	701·5 (586·5-825·3)	633·9 (516·8-761·1)	631·4 (512·6-760·2)	591·6 (453·1-749·4)	7·1 (6·9-7·3)	6·3 (6·1-6·5)	6·2 (6·1-6·4)	5·7 (5·5-5·9)
France	3·8 (3·6-4·1)	3·7 (3·5-3·9)	3·7 (3·5-3·9)	3·6 (3·3-3·8)	0·4 (0·3-0·5)	0·2 (0·1-0·2)	0·2 (0·1-0·2)	0·1 (0·1-0·1)	859·1 (723·4-1,020·2)	896·4 (720·4-1,094·6)	884·8 (709·0-1,086·3)	738·3 (568·1-938·8)	12·6 (12·4-12·8)	11·9 (11·7-12·1)	11·9 (11·7-12·1)	11·0 (10·4-11·3)
Germany	3·3 (3·2-3·5)	3·2 (3·0-3·4)	3·2 (3·0-3·4)	3·1 (2·9-3·3)	0·6 (0·5-0·8)	0·4 (0·3-0·5)	0·4 (0·3-0·5)	0·2 (0·1-0·3)	854·7 (718·2-1,005·9)	806·7 (661·6-964·3)	806·2 (659·7-965·1)	797·0 (632·9-972·9)	14·1 (13·9-14·2)	13·5 (13·3-13·6)	13·5 (13·3-13·6)	11·7 (11·2-12·0)
Greece	3·8 (3·6-4·0)	3·6 (3·4-3·8)	3·6 (3·4-3·8)	3·4 (3·2-3·6)	0·6 (0·4-0·8)	0·2 (0·2-0·3)	0·2 (0·2-0·3)	0·1 (0·1-0·1)	837·5 (697·4-994·1)	712·4 (582·6-856·3)	704·4 (574·9-848·3)	600·9 (475·0-741·5)	12·0 (11·3-12·8)	11·4 (10·7-12·1)	11·3 (10·6-12·0)	9·6 (8·5-10·5)
Iceland	3·7 (3·5-3·9)	3·5 (3·3-3·7)	3·5 (3·3-3·7)	3·3 (3·2-3·5)	0·3 (0·2-0·4)	0·2 (0·1-0·2)	0·2 (0·1-0·2)	0·1 (0·1-0·1)	964·6 (783·6-1,182·4)	832·7 (664·6-1,021·3)	832·8 (663·5-1,023·8)	832·7 (647·0-1,045·1)	8·0 (7·3-8·8)	7·3 (6·6-8·1)	7·3 (6·7-8·1)	7·0 (6·3-7·8)
Ireland	3·9 (3·7-4·1)	3·6 (3·5-3·8)	3·6 (3·4-3·8)	3·5 (3·1-3·7)	0·5 (0·4-0·7)	0·3 (0·2-0·3)	0·2 (0·2-0·3)	0·1 (0·1-0·2)	833·8 (707·1-969·5)	674·0 (549·8-832·2)	673·3 (547·4-834·3)	660·5 (509·8-864·3)	9·8 (9·5-10·2)	9·2 (8·9-9·5)	9·2 (8·8-9·5)	8·1 (7·8-8·5)
Israel	4·2 (3·9-4·4)	4·0 (3·8-4·2)	4·0 (3·8-4·2)	3·9 (3·6-4·1)	0·5 (0·4-0·6)	0·3 (0·2-0·4)	0·3 (0·2-0·3)	0·1 (0·1-0·2)	632·1 (531·4-740·7)	584·6 (477·1-705·4)	585·2 (476·5-707·8)	590·2 (464·7-731·6)	17·0 (16·1-17·9)	18·8 (17·8-19·8)	18·7 (17·7-19·7)	18·2 (16·6-19·5)
Italy	4·2 (3·9-4·4)	3·9 (3·7-4·1)	3·9 (3·7-4·1)	3·7 (3·5-4·0)	0·6 (0·4-0·8)	0·3 (0·2-0·4)	0·3 (0·2-0·4)	0·2 (0·1-0·2)	852·5 (725·9-995·5)	758·2 (616·1-931·1)	758·4 (613·9-935·2)	758·1 (578·0-986·7)	15·4 (15·2-15·7)	15·6 (15·3-15·9)	15·5 (15·2-15·7)	12·3 (12·1-12·5)
Luxembourg	3·6 (3·4-3·8)	3·5 (3·3-3·6)	3·5 (3·3-3·6)	3·4 (3·2-3·5)	0·4 (0·3-0·5)	0·2 (0·1-0·2)	0·2 (0·1-0·2)	0·1 (0·1-0·1)	789·7 (671·1-933·2)	696·5 (571·3-843·3)	693·3 (567·2-842·0)	647·7 (512·7-818·5)	17·0 (15·5-18·6)	16·1 (14·7-17·7)	16·1 (14·7-17·7)	14·1 (12·7-15·7)
Malta	4·0 (3·7-4·2)	3·8 (3·6-4·0)	3·8 (3·6-4·0)	3·6 (3·3-3·8)	0·8 (0·6-1·0)	0·4 (0·3-0·5)	0·3 (0·3-0·5)	0·2 (0·1-0·2)	881·6 (742·0-1,053·8)	763·4 (625·9-931·1)	760·9 (621·8-931·2)	721·5 (567·7-918·5)	13·4 (10·9-16·5)	12·3 (10·1-15·1)	12·1 (9·9-14·8)	10·7 (8·7-13·2)
Netherlands	3·7 (3·5-3·9)	3·5 (3·3-3·7)	3·5 (3·3-3·7)	3·4 (3·2-3·6)	0·3 (0·2-0·3)	0·1 (0·1-0·2)	0·1 (0·1-0·2)	0·1 (0·0-0·1)	991·3 (850·0-1,142·2)	821·7 (692·8-965·9)	814·9 (686·2-959·4)	722·4 (593·1-870·5)	15·8 (15·4-16·2)	15·2 (14·8-15·6)	15·2 (14·8-15·6)	13·9 (12·9-14·4)
Norway	3·7 (3·5-3·9)	3·5 (3·3-3·7)	3·5 (3·3-3·7)	3·4 (3·1-3·5)	0·5 (0·3-0·6)	0·2 (0·2-0·3)	0·2 (0·2-0·3)	0·1 (0·1-0·2)	772·4 (650·9-910·9)	684·5 (564·2-822·2)	672·8 (553·2-809·0)	528·0 (420·0-650·5)	9·6 (9·3-9·8)	7·9 (7·7-8·1)	7·9 (7·7-8·1)	6·9 (6·6-7·2)
Portugal	4·4 (4·2-4·7)	4·0 (3·8-4·3)	4·0 (3·8-4·3)	3·7 (3·4-4·0)	2·4 (1·8-3·0)	0·7 (0·5-0·9)	0·6 (0·5-0·8)	0·2 (0·2-0·3)	921·3 (775·8-1,071·5)	753·5 (605·3-921·3)	746·8 (597·6-915·9)	658·6 (506·8-838·8)	9·7 (9·3-10·2)	9·5 (9·0-10·0)	9·5 (9·0-10·0)	9·3 (8·8-9·9)
Spain	3·9 (3·7-4·1)	3·7 (3·5-3·9)	3·7 (3·5-3·9)	3·5 (3·3-3·7)	1·0 (0·8-1·3)	0·4 (0·3-0·6)	0·4 (0·3-0·5)	0·2 (0·2-0·3)	756·1 (640·6-883·3)	580·3 (475·8-692·0)	575·4 (471·2-688·1)	509·2 (408·7-630·2)	10·1 (9·9-10·3)	10·0 (9·8-10·1)	10·0 (9·8-10·1)	9·6 (9·3-9·8)

Location	Indicator 16.1.1: Age-standardised death rate due to interpersonal violence (per 100,000 population)				Indicator 16.1.2: Death rate due to conflict and terrorism (per 100,000 population)				Indicator 16.1.3: Age-standardised prevalence of physical or sexual violence experienced by populations in the last 12 months (%)				Indicator 16.2.3: Age-standardised prevalence of women and men aged 18-29 years who experienced sexual violence by age 18 (%)			
	**2000**	**2015**	**2016**	**2030**	**2000**	**2015**	**2016**	**2030**	**2000**	**2015**	**2016**	**2030**	**2000**	**2015**	**2016**	**2030**
**High-income North America**																
Canada	1·5 (1·2-2·5)	1·5 (1·0-2·1)	1·5 (1·0-2·1)	1·3 (0·8-1·9)	0·0 (0·0-0·0)	0·0 (0·0-0·0)	0·0 (0·0-0·0)	0·0 (0·0-0·0)	4·8 (4·3-5·5)	4·7 (4·1-5·4)	4·7 (4·1-5·4)	4·5 (3·9-5·1)	10·8 (8·8-13·4)	11·4 (9·6-13·7)	11·5 (9·7-13·8)	12·5 (10·7-14·7)
United States	6·5 (4·2-7·9)	5·8 (3·9-7·5)	5·8 (3·8-7·5)	5·3 (3·4-7·4)	0·01 (0·01-0·01)	0·0 (0·0-0·0)	0·0 (0·0-0·0)	0·0 (0·0-0·0)	3·1 (2·7-3·5)	2·9 (2·5-3·3)	2·9 (2·5-3·4)	3·2 (2·8-3·7)	12·1 (9·3-15·7)	12·1 (9·3-15·7)	12·1 (9·3-15·7)	12·0 (9·3-15·6)
**Australasia**																
Australia	1·7 (1·2-2·3)	1·2 (0·9-1·7)	1·2 (0·9-1·7)	1·0 (0·7-1·4)	0·0 (0·0-0·0)	0·0 (0·0-0·0)	0·0 (0·0-0·0)	0·0 (0·0-0·0)	5·1 (4·5-5·8)	4·7 (4·2-5·4)	4·7 (4·1-5·3)	4·3 (3·8-4·9)	10·4 (8·5-12·9)	10·8 (8·5-13·9)	10·8 (8·4-14·0)	11·0 (8·3-14·9)
New Zealand	1·6 (1·2-2·3)	1·3 (0·9-1·7)	1·3 (0·9-1·7)	0·9 (0·6-1·4)	0·0 (0·0-0·0)	0·0 (0·0-0·0)	0·0 (0·0-0·0)	0·0 (0·0-0·0)	8·4 (7·4-9·6)	7·9 (6·9-8·9)	7·8 (6·9-8·8)	6·7 (5·9-7·6)	16·3 (14·2-18·8)	16·8 (14·6-19·2)	16·8 (14·6-19·3)	17·4 (15·0-19·9)
**High-income Asia Pacific**																
Brunei	1·5 (1·0-1·8)	1·2 (0·9-1·5)	1·2 (0·9-1·6)	1·1 (0·6-2·0)	0·0 (0·0-0·0)	0·0 (0·0-0·0)	0·0 (0·0-0·0)	0·0 (0·0-0·0)	5·6 (4·9-6·4)	5·1 (4·4-5·8)	5·0 (4·4-5·8)	4·7 (4·1-5·4)	11·4 (8·7-14·8)	11·4 (8·7-14·9)	11·4 (8·8-14·9)	11·4 (8·7-14·9)
Japan	0·8 (0·6-1·1)	0·6 (0·4-0·8)	0·6 (0·4-0·8)	0·4 (0·3-0·6)	0·0 (0·0-0·0)	0·0 (0·0-0·0)	0·0 (0·0-0·0)	0·0 (0·0-0·0)	4·9 (4·3-5·6)	4·5 (4·0-5·1)	4·5 (4·0-5·1)	4·2 (3·7-4·8)	11·9 (9·0-15·4)	11·9 (9·0-15·4)	11·9 (9·0-15·4)	11·9 (9·0-15·4)
Singapore	1·0 (0·6-1·2)	0·5 (0·3-0·8)	0·5 (0·3-0·8)	0·3 (0·1-0·7)	0·0 (0·0-0·0)	0·0 (0·0-0·0)	0·0 (0·0-0·0)	0·0 (0·0-0·0)	5·2 (4·5-6·0)	4·3 (3·7-4·9)	4·2 (3·7-4·9)	3·8 (3·3-4·4)	11·4 (8·7-14·8)	11·4 (8·8-14·9)	11·4 (8·7-14·9)	11·4 (8·7-14·8)
South Korea	2·1 (1·2-2·5)	1·4 (0·7-1·9)	1·4 (0·7-2·0)	0·9 (0·3-1·8)	0·0 (0·0-0·0)	0·0 (0·0-0·0)	0·0 (0·0-0·0)	0·0 (0·0-0·0)	5·8 (5·1-6·7)	5·1 (4·4-5·9)	5·0 (4·4-5·8)	4·4 (3·9-5·2)	11·4 (8·8-14·9)	11·5 (8·8-14·9)	11·5 (8·8-14·9)	11·5 (8·8-14·9)
**Western Europe**																
Andorra	0·7 (0·5-1·2)	0·7 (0·5-1·1)	0·7 (0·5-1·1)	0·7 (0·4-1·2)	0·0 (0·0-0·0)	0·0 (0·0-0·0)	0·0 (0·0-0·0)	0·0 (0·0-0·0)	4·6 (3·9-5·5)	4·5 (3·8-5·3)	4·5 (3·8-5·3)	4·4 (3·6-5·3)	11·2 (8·4-14·5)	11·2 (8·4-14·6)	11·2 (8·4-14·6)	11·2 (8·4-14·6)
Austria	1·0 (0·8-1·5)	0·7 (0·6-1·1)	0·7 (0·5-1·0)	0·5 (0·4-0·8)	0·0 (0·0-0·0)	0·0 (0·0-0·0)	0·0 (0·0-0·0)	0·0 (0·0-0·0)	4·1 (3·6-4·6)	3·7 (3·3-4·2)	3·7 (3·3-4·2)	3·4 (3·0-3·9)	11·1 (8·4-14·5)	11·2 (8·4-14·6)	11·2 (8·4-14·6)	11·2 (8·5-14·6)
Belgium	1·9 (1·3-2·4)	1·3 (0·9-1·6)	1·3 (0·8-1·6)	0·9 (0·6-1·3)	0·0 (0·0-0·0)	0·0 (0·0-0·0)	0·3 (0·1-0·5)	0·3 (0·1-0·5)	7·0 (6·2-8·0)	6·4 (5·7-7·2)	6·4 (5·6-7·2)	6·0 (5·3-6·8)	11·2 (8·4-14·6)	11·2 (8·4-14·6)	11·2 (8·4-14·6)	11·2 (8·4-14·6)
Cyprus	2·7 (2·1-3·2)	1·8 (1·4-2·1)	1·8 (1·4-2·1)	1·4 (1·0-1·8)	0·0 (0·0-0·0)	0·0 (0·0-0·0)	0·0 (0·0-0·0)	0·0 (0·0-0·0)	4·5 (3·9-5·0)	3·9 (3·5-4·5)	3·9 (3·5-4·4)	3·7 (3·2-4·1)	11·1 (8·4-14·5)	11·1 (8·4-14·5)	11·1 (8·4-14·5)	11·1 (8·4-14·5)
Denmark	1·4 (0·9-1·7)	0·8 (0·5-1·1)	0·8 (0·5-1·1)	0·5 (0·3-0·8)	0·0 (0·0-0·0)	0·0 (0·0-0·0)	0·0 (0·0-0·0)	0·0 (0·0-0·0)	6·8 (6·0-7·7)	6·3 (5·6-7·1)	6·3 (5·5-7·0)	5·7 (5·1-6·4)	10·3 (8·0-13·3)	10·3 (7·9-13·4)	10·3 (7·9-13·5)	10·4 (7·9-13·5)
Finland	2·5 (1·6-3·2)	1·4 (1·0-2·0)	1·4 (0·9-2·0)	0·8 (0·5-1·3)	0·0 (0·0-0·0)	0·0 (0·0-0·0)	0·0 (0·0-0·0)	0·0 (0·0-0·0)	7·1 (6·3-8·0)	6·3 (5·6-7·1)	6·2 (5·5-7·0)	5·4 (4·8-6·1)	10·4 (8·1-13·3)	10·4 (7·9-13·5)	10·4 (7·9-13·5)	10·4 (7·9-13·5)
France	1·3 (1·0-1·9)	0·9 (0·6-1·3)	0·9 (0·6-1·3)	0·7 (0·4-1·0)	0·0 (0·0-0·0)	0·4 (0·2-0·6)	0·0 (0·0-0·0)	0·0 (0·0-0·0)	7·0 (6·2-8·0)	6·5 (5·8-7·3)	6·5 (5·7-7·3)	6·1 (5·4-6·8)	7·4 (5·6-9·7)	7·5 (5·6-9·8)	7·5 (5·6-9·8)	7·5 (5·6-9·8)
Germany	1·1 (0·8-1·5)	0·7 (0·6-1·1)	0·7 (0·5-1·1)	0·5 (0·4-0·9)	0·0 (0·0-0·0)	0·0 (0·0-0·0)	0·0 (0·0-0·0)	0·0 (0·0-0·0)	5·7 (5·0-6·5)	5·2 (4·5-5·8)	5·1 (4·5-5·8)	4·7 (4·1-5·3)	12·5 (9·4-16·4)	12·5 (9·4-16·4)	12·5 (9·4-16·4)	12·5 (9·4-16·3)
Greece	1·0 (0·7-1·4)	1·0 (0·6-1·2)	1·0 (0·5-1·3)	0·9 (0·4-1·4)	0·0 (0·0-0·0)	0·0 (0·0-0·0)	0·0 (0·0-0·0)	0·0 (0·0-0·0)	5·3 (4·7-6·1)	5·0 (4·5-5·7)	5·0 (4·5-5·7)	5·0 (4·4-5·6)	11·2 (8·5-14·6)	11·2 (8·4-14·6)	11·2 (8·4-14·6)	11·2 (8·4-14·6)
Iceland	1·0 (0·7-1·3)	0·7 (0·5-1·0)	0·7 (0·5-1·0)	0·5 (0·4-0·8)	0·0 (0·0-0·0)	0·0 (0·0-0·0)	0·0 (0·0-0·0)	0·0 (0·0-0·0)	5·0 (4·2-6·0)	4·6 (3·8-5·5)	4·6 (3·8-5·5)	4·2 (3·6-5·1)	11·5 (9·1-14·7)	10·9 (8·5-14·1)	10·9 (8·5-14·0)	10·1 (7·7-13·1)
Ireland	1·0 (0·6-1·3)	0·7 (0·4-1·0)	0·7 (0·4-1·0)	0·6 (0·3-1·0)	0·0 (0·0-0·0)	0·0 (0·0-0·0)	0·0 (0·0-0·0)	0·0 (0·0-0·0)	5·3 (4·7-6·0)	4·8 (4·2-5·4)	4·8 (4·2-5·4)	4·4 (3·8-4·9)	11·8 (9·7-14·4)	12·4 (9·7-15·7)	12·5 (9·8-16·0)	12·5 (9·7-16·0)
Israel	3·2 (1·5-4·4)	2·2 (1·0-3·1)	2·2 (1·0-3·2)	1·5 (0·5-3·1)	1·3 (1·2-1·4)	1·2 (0·6-1·9)	0·1 (0·06-0·2)	0·2 (0·01-0·9)	6·8 (5·8-8·1)	6·3 (5·3-7·5)	6·2 (5·3-7·5)	5·9 (4·9-7·0)	13·6 (10·8-17·0)	13·7 (10·6-17·3)	13·7 (10·6-17·3)	13·7 (10·5-17·4)
Italy	1·2 (0·7-1·4)	0·8 (0·5-0·9)	0·8 (0·5-0·9)	0·5 (0·3-0·8)	0·0 (0·0-0·0)	0·0 (0·0-0·0)	0·0 (0·0-0·0)	0·0 (0·0-0·0)	5·8 (5·1-6·6)	5·3 (4·7-6·0)	5·3 (4·6-5·9)	5·1 (4·5-5·7)	11·1 (8·4-14·5)	11·2 (8·4-14·6)	11·2 (8·4-14·6)	11·2 (8·5-14·6)
Luxembourg	1·5 (1·0-1·9)	0·9 (0·7-1·2)	0·9 (0·6-1·2)	0·6 (0·4-0·9)	0·0 (0·0-0·0)	0·0 (0·0-0·0)	0·0 (0·0-0·0)	0·0 (0·0-0·0)	4·8 (4·2-5·4)	4·3 (3·8-4·8)	4·3 (3·7-4·8)	4·0 (3·5-4·5)	11·1 (8·4-14·5)	11·2 (8·4-14·5)	11·2 (8·4-14·5)	11·2 (8·4-14·6)
Malta	1·5 (1·0-2·1)	1·1 (0·8-1·6)	1·1 (0·8-1·6)	0·8 (0·5-1·4)	0·0 (0·0-0·0)	0·0 (0·0-0·0)	0·0 (0·0-0·0)	0·0 (0·0-0·0)	4·4 (3·9-4·9)	4·0 (3·5-4·5)	4·0 (3·5-4·5)	3·6 (3·2-4·1)	11·2 (8·4-14·6)	11·2 (8·4-14·6)	11·2 (8·4-14·6)	11·2 (8·5-14·6)
Netherlands	1·4 (0·8-1·6)	0·8 (0·5-1·0)	0·8 (0·5-1·0)	0·6 (0·4-0·8)	0·0 (0·0-0·0)	0·0 (0·0-0·0)	0·0 (0·0-0·0)	0·0 (0·0-0·0)	7·1 (6·2-8·0)	6·5 (5·7-7·3)	6·5 (5·7-7·3)	6·1 (5·4-6·9)	11·8 (9·9-13·9)	11·8 (10·0-13·9)	11·8 (10·0-13·9)	11·8 (10·0-13·9)
Norway	1·1 (0·8-1·5)	0·7 (0·5-1·0)	0·7 (0·5-1·0)	0·6 (0·3-0·9)	0·0 (0·0-0·0)	0·0 (0·0-0·0)	0·0 (0·0-0·0)	0·0 (0·0-0·0)	5·1 (4·3-6·0)	4·7 (3·9-5·5)	4·6 (3·9-5·5)	4·2 (3·5-4·9)	10·3 (7·7-13·7)	10·3 (7·7-13·7)	10·3 (7·7-13·7)	10·2 (7·7-13·8)
Portugal	2·2 (1·4-3·0)	1·3 (0·8-1·7)	1·3 (0·8-1·7)	1·0 (0·6-1·5)	0·0 (0·0-0·0)	0·0 (0·0-0·0)	0·0 (0·0-0·0)	0·0 (0·0-0·0)	5·7 (4·9-6·5)	5·0 (4·4-5·7)	5·0 (4·3-5·6)	4·6 (4·0-5·2)	11·2 (8·4-14·6)	11·2 (8·4-14·6)	11·2 (8·4-14·6)	11·2 (8·4-14·6)
Spain	0·9 (0·6-1·2)	0·6 (0·4-0·7)	0·6 (0·4-0·7)	0·4 (0·2-0·6)	0·0 (0·0-0·0)	0·0 (0·0-0·0)	0·0 (0·0-0·0)	0·0 (0·0-0·0)	3·9 (3·4-4·5)	3·6 (3·1-4·1)	3·6 (3·1-4·1)	3·4 (3·0-3·9)	10·4 (8·3-13·1)	10·5 (8·1-13·7)	10·6 (8·1-13·7)	10·6 (8·1-14·1)

Location	Indicator 17.19.2c: Percentage of well-certified deaths by a vital registration (VR) system among a country's total population (%)			
	**2000**	**2015**	**2016**	**2030**
**High-income North America**				
Canada	89·0 (87·5-90·2)	90·1 (88·4-91·6)	90·2 (88·4-91·7)	91·3 (88·5-93·7)
United States	88·2 (87·9-88·5)	87·7 (87·2-88·0)	87·7 (87·3-88·1)	87·5 (86·6-88·3)
**Australasia**				
Australia	91·1 (88·6-93·3)	90·0 (85·1-93·8)	90·0 (83·7-94·5)	88·6 (74·9-96·3)
New Zealand	95·4 (93·4-96·9)	95·1 (91·8-97·4)	95·1 (91·1-97·6)	95·5 (86·9-99·5)
**High-income Asia Pacific**				
Brunei	81·5 (78·2-84·3)	80·3 (76·0-84·2)	80·6 (76·0-84·7)	84·8 (75·9-91·8)
Japan	85·1 (84·6-85·5)	82·1 (81·3-82·9)	82·4 (81·6-83·2)	80·3 (78·1-82·7)
Singapore	93·9 (92·2-95·2)	95·6 (94·0-96·7)	95·5 (93·9-96·7)	97·3 (94·9-98·7)
South Korea	78·9 (76·0-81·8)	81·7 (77·4-85·5)	82·1 (77·1-86·0)	86·2 (75·9-92·6)
**Western Europe**				
Andorra	0·0 (0·0-0·0)	0·0 (0·0-0·0)	0·0 (0·0-0·0)	0·0 (0·0-0·0)
Austria	88·0 (85·5-90·2)	89·1 (85·7-92·1)	89·2 (85·5-92·4)	89·0 (80·4-94·5)
Belgium	82·7 (79·8-85·3)	79·8 (74·8-84·3)	79·9 (74·3-84·9)	78·2 (67·0-87·5)
Cyprus	35·5 (29·6-41·9)	65·7 (59·4-72·2)	65·9 (58·8-73·4)	70·2 (54·7-81·5)
Denmark	84·4 (81·3-87·0)	83·9 (79·4-87·7)	83·9 (79·1-88·1)	84·8 (74·5-91·8)
Finland	95·0 (93·6-96·2)	95·1 (92·9-96·7)	95·1 (92·9-96·7)	96·0 (91·8-98·3)
France	78·4 (75·2-81·3)	78·1 (72·5-83·0)	78·3 (71·8-83·5)	78·2 (66·2-86·8)
Germany	83·2 (80·3-85·7)	84·0 (79·7-87·7)	84·1 (79·2-88·2)	85·7 (77·0-92·6)
Greece	71·1 (67·8-74·2)	72·1 (66·6-77·2)	72·3 (66·0-78·0)	74·2 (63·4-83·7)
Iceland	92·1 (90·0-93·8)	88·7 (85·8-91·1)	88·8 (85·7-91·4)	86·2 (77·4-92·1)
Ireland	90·1 (88·0-92·0)	91·7 (88·5-94·2)	91·8 (88·4-94·4)	93·0 (86·7-97·1)
Israel	81·5 (78·5-84·3)	78·1 (73·2-82·5)	78·4 (72·6-83·1)	75·5 (64·1-84·5)
Italy	86·9 (84·1-89·3)	87·8 (83·3-91·3)	87·9 (83·0-91·6)	88·6 (79·6-94·7)
Luxembourg	81·4 (78·3-84·4)	80·6 (75·6-85·0)	80·7 (75·0-85·6)	81·5 (69·7-90·7)
Malta	88·1 (85·6-90·5)	89·3 (85·9-92·0)	89·5 (85·9-92·5)	91·8 (85·0-96·2)
Netherlands	82·5 (79·6-85·2)	83·0 (79·1-86·5)	83·1 (78·3-87·0)	83·7 (74·2-90·7)
Norway	86·5 (84·0-89·0)	82·5 (77·9-86·5)	82·7 (77·2-87·0)	80·5 (68·9-89·7)
Portugal	74·2 (70·7-77·5)	79·2 (74·1-83·5)	79·3 (73·5-84·2)	81·9 (71·3-89·5)
Spain	82·7 (80·1-85·1)	85·0 (81·0-88·5)	85·0 (80·9-88·9)	87·2 (79·8-93·1)

Location	Indicator 1.5.1: Death rate due to exposure to forces of nature (per 100,000 population)				Indicator 2.2.1: Prevalence of stunting in children under 5 (%)				Indicator 2.2.2a: Prevalence of wasting in children under 5 (%)				Indicator 2.2.2b: Prevalence of overweight in children aged 2-4 (%)			
	2000	2015	2016	2030	2000	2015	2016	2030	2000	2015	2016	2030	2000	2015	2016	2030
Sweden	0·0 (0·0-0·0)	0·0 (0·0-0·0)	0·0 (0·0-0·0)	0·0 (0·0-0·01)	1·3 (1·1-1·6)	1·2 (1·0-1·6)	1·2 (1·0-1·6)	1·1 (0·9-1·6)	1·1 (0·8-1·7)	0·9 (0·6-1·5)	0·9 (0·6-1·5)	0·8 (0·6-1·3)	22·2 (17·3-28·4)	26·1 (20·6-32·2)	26·4 (20·9-32·4)	31·1 (20·1-44·1)
Switzerland	0·2 (0·1-0·3)	0·1 (0·05-0·2)	0·09 (0·04-0·1)	0·1 (0·06-0·2)	1·2 (0·9-1·7)	1·2 (0·9-1·6)	1·1 (0·9-1·7)	1·1 (0·8-1·7)	1·1 (0·7-1·7)	0·9 (0·6-1·6)	0·9 (0·6-1·5)	0·8 (0·5-1·5)	18·2 (14·1-23·6)	22·4 (17·3-28·3)	22·6 (17·3-28·5)	26·3 (16·4-39·1)
United Kingdom	0·02 (0·01-0·02)	0·0 (0·0-0·01)	0·0 (0·0-0·01)	0·0 (0·0-0·01)	3·2 (3·1-3·2)	2·9 (2·9-3·0)	2·9 (2·8-2·9)	2·6 (2·6-2·7)	1·0 (0·9-1·1)	0·9 (0·8-1·0)	0·9 (0·8-1·0)	0·8 (0·8-0·9)	23·6 (20·3-26·7)	26·1 (21·5-30·9)	26·5 (21·7-31·6)	30·7 (21·5-41·8)
**Southern Latin America**																
Argentina	0·04 (0·01-0·07)	0·02 (0·0-0·04)	0·02 (0·0-0·04)	0·01 (0·0-0·03)	9·9 (7·5-12·6)	6·8 (5·0-9·0)	6·7 (4·9-8·9)	5·5 (3·8-7·5)	3·9 (2·9-5·1)	2·4 (1·4-3·8)	2·4 (1·4-3·7)	1·8 (0·8-3·2)	12·4 (8·6-17·8)	17·2 (11·6-24·9)	17·4 (11·7-25·0)	22·9 (11·3-40·0)
Chile	0·08 (0·01-0·2)	0·5 (0·08-0·9)	0·4 (0·07-0·8)	0·3 (0·05-0·6)	2·6 (2·0-3·1)	2·3 (1·6-3·0)	2·2 (1·5-2·9)	1·0 (0·6-1·4)	0·5 (0·2-1·3)	0·4 (0·1-1·2)	0·4 (0·1-1·2)	0·3 (0·0-1·0)	32·0 (23·7-41·4)	44·1 (34·3-53·9)	44·7 (34·6-54·4)	57·9 (39·4-74·9)
Uruguay	0·07 (0·01-0·1)	0·0 (0·0-0·0)	0·0 (0·0-0·0)	0·01 (0·0-0·02)	11·3 (8·7-14·1)	9·2 (6·8-12·1)	9·1 (6·7-11·9)	7·3 (5·2-10·0)	2·7 (2·0-3·6)	1·8 (1·1-2·9)	1·8 (1·0-2·8)	1·2 (0·6-2·3)	22·7 (16·4-30·5)	35·3 (26·5-45·0)	35·8 (27·1-45·6)	49·2 (31·9-66·5)
**Eastern Europe**																
Belarus	0·0 (0·0-0·0)	0·5 (0·2-0·8)	0·4 (0·2-0·6)	0·4 (0·2-0·6)	6·2 (3·7-9·7)	4·6 (2·7-7·5)	4·6 (2·7-7·4)	3·8 (2·2-6·8)	2·9 (2·3-3·7)	2·5 (1·9-3·3)	2·5 (1·9-3·2)	2·3 (1·7-3·0)	15·9 (9·9-24·4)	21·5 (14·2-31·4)	21·9 (14·5-31·2)	28·9 (12·6-51·2)
Estonia	0·02 (0·0-0·04)	0·0 (0·0-0·0)	0·0 (0·0-0·0)	0·0 (0·0-0·0)	10·4 (6·0-16·3)	5·9 (3·4-9·8)	5·8 (3·3-9·6)	4·1 (2·2-6·9)	3·1 (2·3-3·9)	2·3 (1·6-3·0)	2·3 (1·6-3·0)	1·9 (1·3-2·6)	15·9 (10·2-23·0)	26·7 (17·9-36·7)	27·0 (18·2-37·6)	38·4 (19·2-61·7)
Latvia	0·02 (0·01-0·04)	0·0 (0·0-0·0)	0·0 (0·0-0·0)	0·0 (0·0-0·0)	11·7 (6·8-18·1)	6·5 (3·7-10·7)	6·4 (3·6-10·7)	3·9 (2·2-6·8)	3·4 (2·6-4·3)	2·5 (1·8-3·2)	2·5 (1·8-3·2)	2·2 (1·6-2·8)	14·0 (9·0-20·6)	21·6 (14·1-30·4)	21·9 (14·4-31·3)	26·9 (13·0-47·2)
Lithuania	0·1 (0·04-0·2)	0·0 (0·0-0·0)	0·0 (0·0-0·0)	0·01 (0·01-0·01)	11·2 (6·5-17·2)	6·1 (3·4-9·9)	6·0 (3·3-9·8)	3·7 (2·1-6·0)	3·3 (2·5-4·1)	2·4 (1·8-3·1)	2·4 (1·8-3·1)	2·0 (1·4-2·6)	11·6 (7·8-16·9)	20·1 (13·1-29·6)	20·5 (13·5-30·2)	26·6 (12·1-49·1)
Moldova	0·1 (0·08-0·2)	0·05 (0·02-0·07)	0·04 (0·02-0·06)	0·07 (0·04-0·09)	13·8 (8·3-20·2)	7·8 (4·7-11·7)	7·7 (4·6-11·6)	5·7 (2·8-10·5)	6·2 (4·9-7·6)	3·0 (2·3-3·8)	2·9 (2·2-3·7)	2·0 (1·3-2·7)	8·0 (4·8-12·6)	10·2 (6·6-15·2)	10·4 (6·6-16·1)	13·3 (5·2-27·0)
Russia	0·5 (0·2-0·8)	0·05 (0·02-0·09)	0·04 (0·01-0·07)	0·1 (0·05-0·2)	14·1 (10·3-18·7)	10·4 (6·2-16·2)	10·2 (6·1-16·1)	8·2 (4·0-14·1)	4·6 (3·8-5·4)	2·3 (1·7-3·1)	2·3 (1·6-3·1)	1·5 (1·0-2·4)	22·6 (15·6-31·3)	24·6 (16·1-34·4)	24·8 (16·4-34·9)	28·1 (12·9-50·8)
Ukraine	0·0 (0·0-0·0)	0·3 (0·2-0·5)	0·3 (0·1-0·4)	0·3 (0·1-0·4)	22·7 (19·6-26·3)	16·8 (10·8-24·2)	16·7 (10·6-24·1)	15·0 (8·9-23·2)	6·5 (5·4-7·9)	5·3 (4·0-6·8)	5·2 (4·0-6·7)	4·6 (3·4-6·1)	10·9 (7·0-16·5)	16·7 (11·0-25·3)	16·7 (10·9-25·3)	19·9 (8·7-37·8)
**Central Europe**																
Albania	0·3 (0·2-0·5)	0·0 (0·0-0·0)	0·0 (0·0-0·0)	0·04 (0·03-0·06)	36·2 (33·1-39·6)	22·3 (17·7-27·3)	21·9 (17·1-27·1)	16·8 (10·3-24·0)	9·8 (8·6-11·2)	8·7 (6·5-11·6)	8·6 (6·3-11·8)	8·3 (4·5-14·4)	37·2 (28·7-45·7)	38·3 (29·5-47·3)	38·7 (29·9-48·1)	42·0 (25·0-60·2)
Bosnia and Herzegovina	0·0 (0·0-0·0)	0·1 (0·02-0·2)	0·1 (0·02-0·2)	0·06 (0·01-0·1)	11·8 (9·7-13·9)	14·3 (10·5-18·3)	14·0 (10·2-18·1)	14·9 (9·9-19·5)	6·7 (5·4-8·2)	7·3 (5·6-9·6)	7·3 (5·6-9·8)	9·0 (6·3-12·0)	24·2 (18·1-31·9)	31·3 (23·0-40·2)	31·6 (23·2-40·9)	35·7 (20·3-53·9)
Bulgaria	0·3 (0·1-0·4)	0·05 (0·02-0·08)	0·04 (0·01-0·06)	0·09 (0·05-0·1)	12·1 (8·8-16·2)	9·4 (6·8-12·8)	9·3 (6·7-12·7)	7·9 (5·7-10·6)	4·3 (2·7-6·4)	3·7 (2·2-5·9)	3·7 (2·2-5·9)	3·4 (2·0-5·6)	27·9 (20·4-37·0)	33·1 (25·0-43·1)	33·6 (25·3-43·9)	39·2 (23·0-60·4)
Croatia	0·01 (0·0-0·02)	0·0 (0·0-0·0)	0·0 (0·0-0·0)	0·0 (0·0-0·0)	10·2 (7·2-13·9)	7·8 (5·5-10·8)	7·7 (5·5-10·7)	6·8 (4·8-9·5)	4·5 (2·9-6·9)	3·6 (2·3-5·4)	3·6 (2·3-5·4)	3·1 (1·8-4·7)	20·4 (15·5-26·9)	29·2 (22·0-38·7)	29·4 (22·0-38·7)	36·4 (21·7-54·9)
Czech Republic	0·03 (0·01-0·06)	0·01 (0·0-0·01)	0·01 (0·0-0·01)	0·01 (0·0-0·01)	2·9 (2·0-3·9)	2·6 (1·8-3·6)	2·6 (1·8-3·6)	2·4 (1·6-3·3)	3·0 (2·3-4·0)	3·1 (1·9-4·7)	3·1 (1·9-4·8)	3·1 (1·6-5·5)	21·5 (15·6-29·3)	25·3 (18·5-33·6)	25·6 (18·8-33·7)	29·1 (16·8-45·3)
Hungary	0·06 (0·01-0·1)	0·0 (0·0-0·0)	0·0 (0·0-0·0)	0·01 (0·0-0·01)	3·9 (2·7-5·4)	3·3 (2·2-4·6)	3·2 (2·2-4·5)	2·9 (1·9-4·0)	2·0 (1·1-3·3)	1·9 (1·0-3·2)	1·9 (1·0-3·2)	1·9 (0·9-3·0)	24·0 (17·7-32·3)	27·1 (19·8-37·1)	27·4 (20·0-37·1)	29·4 (17·0-48·0)
Macedonia	0·3 (0·2-0·4)	0·06 (0·03-0·1)	0·04 (0·02-0·07)	0·08 (0·05-0·1)	7·7 (5·5-10·5)	8·1 (5·8-11·0)	7·8 (5·5-10·7)	8·7 (6·4-11·7)	3·1 (2·2-4·3)	4·1 (3·0-5·5)	4·0 (2·9-5·5)	4·5 (3·7-5·6)	22·7 (16·6-30·4)	28·6 (21·4-36·9)	29·0 (21·4-38·0)	35·6 (20·7-53·9)
Montenegro	0·0 (0·0-0·0)	0·02 (0·0-0·03)	0·01 (0·0-0·02)	0·02 (0·0-0·03)	10·7 (7·7-14·6)	13·8 (10·4-18·0)	13·8 (10·4-18·1)	14·1 (11·1-18·7)	5·2 (3·7-7·3)	3·6 (2·6-4·7)	3·5 (2·6-4·6)	2·8 (2·0-3·5)	26·0 (19·4-34·1)	35·0 (29·3-41·1)	35·3 (29·0-42·2)	42·9 (28·1-58·8)
Poland	0·04 (0·02-0·06)	0·0 (0·0-0·0)	0·0 (0·0-0·0)	0·0 (0·0-0·0)	10·2 (7·1-13·9)	7·4 (5·0-10·3)	7·3 (5·0-10·2)	5·9 (3·9-8·2)	4·6 (2·9-7·0)	3·6 (2·2-5·6)	3·5 (2·2-5·5)	2·9 (1·7-4·5)	18·9 (13·4-26·1)	22·4 (16·0-30·3)	22·7 (16·5-30·6)	25·8 (13·9-41·5)
Romania	0·3 (0·1-0·4)	0·1 (0·04-0·2)	0·07 (0·03-0·1)	0·1 (0·08-0·2)	17·2 (15·7-18·8)	12·5 (9·0-16·9)	12·3 (8·8-16·7)	9·9 (6·6-14·5)	4·2 (3·6-4·8)	4·0 (2·5-6·3)	4·0 (2·4-6·3)	3·5 (1·8-7·5)	16·8 (12·1-23·1)	23·7 (17·3-31·8)	24·0 (17·5-32·0)	32·5 (18·0-49·8)
Serbia	0·02 (0·01-0·04)	0·1 (0·02-0·2)	0·09 (0·02-0·2)	0·07 (0·03-0·1)	7·3 (5·1-10·2)	9·9 (7·6-12·5)	10·2 (7·9-12·9)	13·5 (10·9-16·6)	6·1 (4·5-8·7)	4·4 (3·5-5·5)	4·4 (3·5-5·6)	3·2 (2·8-3·6)	24·4 (17·6-33·9)	23·0 (19·5-26·9)	23·3 (19·2-27·7)	21·7 (13·8-31·4)
Slovakia	0·2 (0·05-0·3)	0·04 (0·02-0·06)	0·03 (0·01-0·05)	0·04 (0·02-0·06)	9·4 (6·6-12·9)	7·2 (4·9-10·2)	7·1 (4·8-10·0)	6·1 (4·1-8·8)	4·4 (2·7-6·8)	3·5 (2·1-5·3)	3·4 (2·1-5·2)	2·9 (1·8-4·4)	18·2 (13·1-25·1)	22·5 (17·0-29·5)	22·9 (17·2-30·1)	28·2 (16·2-42·6)
Slovenia	0·07 (0·01-0·1)	0·08 (0·04-0·1)	0·06 (0·03-0·1)	0·07 (0·04-0·1)	9·3 (6·6-12·6)	7·2 (5·0-9·8)	7·1 (5·0-9·7)	6·3 (4·2-8·6)	4·5 (2·9-7·0)	3·6 (2·2-5·5)	3·6 (2·2-5·5)	3·1 (1·8-5·2)	24·4 (17·5-33·1)	31·9 (23·6-41·2)	32·2 (24·0-41·5)	38·4 (22·2-57·3)
**Central Asia**																
Armenia	0·0 (0·0-0·0)	0·3 (0·1-0·4)	0·2 (0·1-0·3)	0·2 (0·1-0·3)	18·4 (15·5-21·6)	17·4 (12·0-23·6)	16·7 (11·1-23·6)	15·0 (9·1-21·8)	3·0 (2·3-3·9)	4·0 (2·9-5·3)	3·9 (2·8-5·3)	4·1 (2·8-6·0)	24·6 (21·0-28·5)	33·4 (28·1-39·6)	33·9 (28·3-40·2)	40·8 (29·1-53·5)

Location	Indicator 3.1.1: Maternal mortality ratio (maternal deaths per 100,000 livebirths) in women aged 10-54 years				Indicator 3.1.2: Proportion of births attended by skilled health personnel (%)				Indicator 3.2.1: Under-5 mortality rate (probability of dying before the age of 5 per 1,000 livebirths)				Indicator 3.2.2: Neonatal mortality rate (probability of dying during the first 28 days of life per 1,000 livebirths)			
	2000	2015	2016	2030	2000	2015	2016	2030	2000	2015	2016	2030	2000	2015	2016	2030
Sweden	6·3 (5·8-6·8)	3·3 (2·8-3·9)	3·8 (3·1-4·7)	2·0 (1·1-3·5)	99·0 (98·8-99·2)	99·4 (99·3-99·6)	99·5 (99·3-99·6)	99·7 (99·5-99·8)	3·9 (3·7-4·2)	2·7 (2·3-3·1)	2·6 (2·2-3·2)	1·8 (1·0-3·1)	2·1 (1·9-2·3)	1·4 (1·2-1·6)	1·4 (1·1-1·7)	0·9 (0·5-1·6)
Switzerland	8·2 (7·1-9·5)	5·4 (4·0-7·0)	5·4 (3·5-8·0)	4·6 (0·9-16·2)	99·0 (98·8-99·2)	99·5 (99·4-99·6)	99·5 (99·4-99·6)	99·7 (99·6-99·8)	5·8 (5·5-6·1)	4·1 (3·6-4·7)	3·9 (3·3-4·7)	3·0 (2·1-4·4)	3·5 (3·3-3·8)	2·7 (2·5-3·0)	2·6 (2·3-3·0)	2·2 (1·6-3·0)
United Kingdom	10·3 (9·9-10·7)	6·8 (6·5-7·2)	7·0 (6·7-7·4)	5·6 (4·8-6·4)	98·7 (98·4-98·9)	99·3 (99·1-99·4)	99·3 (99·2-99·5)	99·6 (99·4-99·8)	6·6 (6·4-6·8)	4·6 (4·3-4·9)	4·6 (4·2-5·0)	3·4 (2·8-4·2)	3·7 (3·6-3·8)	2·6 (2·4-2·8)	2·6 (2·4-2·8)	1·9 (1·5-2·3)
**Southern Latin America**																
Argentina	55·7 (50·3-61·7)	53·7 (46·8-61·8)	52·6 (44·9-61·6)	43·6 (26·8-67·6)	98·1 (97·5-98·7)	98·9 (98·5-99·2)	98·9 (98·6-99·2)	99·4 (98·8-99·8)	20·5 (20·3-20·6)	12·4 (12·1-12·8)	12·2 (11·7-12·7)	8·4 (7·4-9·3)	11·5 (11·0-12·0)	7·0 (6·8-7·2)	6·9 (6·6-7·1)	4·9 (4·2-5·7)
Chile	23·8 (20·6-27·5)	21·4 (15·8-28·6)	20·6 (13·3-30·6)	18·9 (7·0-46·4)	98·9 (98·4-99·3)	99·3 (99·0-99·6)	99·3 (99·0-99·6)	99·6 (99·1-99·9)	11·0 (10·9-11·2)	8·1 (7·8-8·5)	7·8 (7·4-8·3)	5·8 (4·8-6·8)	5·7 (5·4-5·9)	5·0 (4·8-5·2)	4·8 (4·6-5·1)	4·1 (3·4-4·8)
Uruguay	29·2 (25·4-33·2)	24·9 (21·1-29·5)	24·1 (20·1-29·0)	20·0 (10·1-35·1)	96·5 (94·8-97·6)	98·7 (98·2-99·2)	98·8 (98·2-99·2)	99·3 (98·5-99·8)	16·0 (15·2-16·8)	8·9 (7·5-10·6)	8·8 (6·8-11·3)	4·4 (2·0-8·7)	7·9 (7·5-8·3)	4·6 (3·9-5·3)	4·5 (3·5-5·6)	2·4 (1·1-4·6)
**Eastern Europe**																
Belarus	42·7 (37·5-48·1)	5·8 (4·6-7·2)	5·8 (4·1-7·9)	2·4 (0·7-5·2)	98·7 (97·8-99·3)	99·5 (99·0-99·8)	99·5 (99·0-99·8)	99·8 (99·0-100·0)	16·1 (13·2-19·3)	5·8 (4·5-7·7)	5·5 (4·2-7·4)	2·2 (1·3-3·8)	8·5 (6·8-10·3)	2·8 (2·3-3·6)	2·7 (2·1-3·4)	1·0 (0·6-1·7)
Estonia	27·1 (23·8-30·7)	5·5 (4·4-6·6)	5·3 (4·0-6·7)	3·6 (0·9-9·3)	99·0 (98·2-99·5)	99·7 (99·4-99·9)	99·7 (99·4-99·9)	99·9 (99·6-100·0)	11·3 (10·3-12·3)	3·3 (2·6-4·1)	3·1 (2·4-4·1)	1·6 (0·4-4·6)	5·4 (4·9-5·9)	1·4 (1·1-1·7)	1·3 (1·0-1·7)	0·6 (0·2-1·5)
Latvia	27·0 (23·6-31·0)	11·0 (8·6-13·7)	10·6 (7·9-14·1)	5·1 (1·5-10·6)	99·1 (98·4-99·5)	99·6 (99·0-99·8)	99·6 (99·1-99·8)	99·8 (99·2-100·0)	13·1 (12·1-14·3)	4·9 (3·7-6·3)	4·8 (3·3-6·8)	2·1 (0·7-4·3)	6·8 (6·2-7·4)	2·5 (2·0-3·1)	2·5 (1·8-3·4)	1·1 (0·4-2·1)
Lithuania	14·7 (13·1-16·7)	12·3 (10·5-14·2)	10·7 (8·9-12·5)	12·9 (6·9-22·8)	99·1 (98·4-99·5)	99·5 (98·9-99·8)	99·5 (98·9-99·8)	99·8 (98·9-100·0)	11·2 (10·6-11·9)	5·3 (4·8-5·8)	4·7 (4·1-5·3)	2·3 (1·5-3·5)	5·2 (4·8-5·6)	2·5 (2·3-2·8)	2·2 (1·9-2·5)	0·9 (0·6-1·4)
Moldova	34·7 (29·4-40·2)	23·1 (19·0-28·1)	20·4 (16·1-25·6)	14·2 (7·4-25·7)	99·0 (98·4-99·5)	99·5 (99·0-99·8)	99·6 (99·1-99·8)	99·9 (99·3-100·0)	31·4 (25·3-38·3)	12·1 (8·4-17·1)	11·6 (7·7-16·8)	6·5 (1·7-16·4)	18·8 (15·1-22·8)	8·0 (5·5-11·3)	7·7 (5·1-11·2)	4·3 (1·2-11·0)
Russia	49·7 (41·7-58·6)	17·2 (11·4-25·3)	17·9 (9·5-31·2)	7·4 (1·6-19·4)	98·9 (98·0-99·4)	99·6 (99·2-99·8)	99·6 (99·2-99·8)	99·8 (99·3-100·0)	19·8 (19·5-20·2)	8·8 (8·6-9·0)	8·4 (8·1-8·7)	3·7 (3·2-4·0)	10·4 (9·9-10·8)	4·5 (4·3-4·6)	4·2 (4·1-4·3)	1·9 (1·8-2·1)
Ukraine	56·5 (49·1-64·5)	22·2 (16·0-29·7)	22·9 (13·2-34·8)	9·8 (2·2-25·7)	99·0 (98·3-99·4)	99·4 (98·9-99·7)	99·3 (98·8-99·7)	99·6 (98·4-100·0)	20·9 (16·2-26·5)	9·4 (6·1-13·9)	9·2 (5·7-14·1)	4·7 (1·2-13·1)	13·3 (10·4-16·9)	5·3 (3·7-7·5)	5·2 (3·5-7·5)	2·6 (0·7-6·9)
**Central Europe**																
Albania	16·6 (13·6-20·7)	9·5 (6·8-12·8)	9·2 (6·5-12·5)	5·2 (2·0-10·4)	97·8 (97·0-98·4)	99·3 (98·8-99·7)	99·4 (98·8-99·7)	99·7 (99·1-100·0)	27·5 (22·6-33·1)	14·2 (9·6-20·9)	13·7 (9·1-20·2)	7·7 (2·5-18·0)	11·2 (9·2-13·6)	7·5 (5·2-10·7)	7·2 (4·9-10·4)	4·1 (1·3-9·4)
Bosnia and Herzegovina	22·0 (15·2-31·1)	7·1 (4·9-10·1)	7·2 (4·9-10·3)	2·6 (1·1-5·4)	99·0 (98·4-99·5)	99·5 (99·1-99·7)	99·5 (99·1-99·8)	99·7 (99·1-100·0)	10·9 (10·4-11·5)	5·3 (4·7-6·0)	5·2 (4·5-6·0)	2·6 (1·7-3·7)	7·4 (6·9-7·9)	3·6 (3·1-4·2)	3·5 (2·9-4·2)	1·7 (1·1-2·5)
Bulgaria	42·3 (37·7-47·6)	19·6 (15·6-24·8)	19·5 (14·6-26·3)	17·5 (5·4-41·3)	98·9 (98·2-99·4)	99·4 (98·8-99·7)	99·4 (98·9-99·7)	99·7 (98·9-100·0)	16·5 (15·7-17·4)	8·5 (6·2-11·3)	8·3 (5·7-12·0)	6·4 (2·3-14·2)	7·7 (7·3-8·2)	4·1 (2·9-5·4)	4·0 (2·7-5·7)	2·8 (1·0-6·2)
Croatia	15·7 (13·6-18·2)	8·5 (6·9-10·1)	8·7 (6·8-10·8)	4·2 (2·1-7·7)	98·9 (98·1-99·4)	99·4 (98·9-99·7)	99·5 (99·0-99·7)	99·7 (99·0-100·0)	9·1 (8·6-9·6)	4·6 (4·0-5·3)	4·5 (3·8-5·3)	2·6 (1·6-3·8)	5·7 (5·4-6·0)	2·8 (2·5-3·3)	2·8 (2·3-3·2)	1·6 (1·0-2·4)
Czech Republic	10·4 (9·3-11·6)	5·5 (4·8-6·3)	5·6 (4·8-6·6)	3·6 (2·1-5·8)	99·0 (98·3-99·4)	99·4 (99·0-99·7)	99·5 (99·0-99·8)	99·8 (99·1-100·0)	5·4 (5·0-5·8)	3·0 (2·7-3·2)	2·8 (2·4-3·3)	1·5 (0·9-2·5)	2·8 (2·6-3·0)	1·4 (1·3-1·6)	1·4 (1·2-1·6)	0·7 (0·5-1·1)
Hungary	11·5 (10·1-12·9)	13·4 (11·3-15·7)	12·4 (10·0-15·2)	8·8 (4·6-15·3)	97·8 (96·2-98·8)	99·1 (98·3-99·6)	99·2 (98·4-99·6)	99·7 (98·8-100·0)	10·6 (10·1-11·1)	5·6 (5·2-6·1)	5·6 (4·5-6·9)	3·9 (2·3-5·9)	6·1 (5·7-6·6)	3·0 (2·7-3·3)	3·0 (2·4-3·6)	1·9 (1·1-3·0)
Macedonia	13·6 (11·4-16·2)	7·8 (6·4-9·5)	7·7 (6·3-9·4)	6·9 (4·2-10·7)	97·0 (95·8-98·0)	99·0 (98·4-99·5)	99·0 (98·3-99·5)	99·6 (98·4-100·0)	14·4 (13·4-15·4)	10·8 (7·4-15·2)	10·1 (6·4-15·3)	5·4 (1·5-15·5)	8·9 (8·2-9·5)	7·0 (4·8-9·9)	6·5 (4·1-10·0)	3·5 (0·9-10·0)
Montenegro	14·2 (10·7-18·6)	6·0 (4·4-8·0)	6·0 (4·3-8·1)	3·5 (1·5-7·0)	98·5 (97·4-99·2)	99·3 (98·7-99·6)	99·3 (98·8-99·7)	99·6 (98·7-100·0)	16·9 (15·4-18·5)	5·2 (4·1-6·5)	5·1 (3·9-6·6)	1·9 (0·9-3·9)	10·6 (9·7-11·6)	3·1 (2·4-3·8)	3·0 (2·3-3·9)	1·1 (0·5-2·1)
Poland	13·3 (11·8-15·1)	4·7 (3·9-5·4)	4·6 (3·7-5·5)	3·0 (1·7-4·8)	99·1 (98·5-99·5)	99·6 (99·2-99·8)	99·6 (99·2-99·8)	99·8 (99·4-100·0)	9·6 (9·3-9·9)	4·7 (3·8-5·7)	4·5 (3·0-6·5)	2·2 (1·1-3·9)	5·8 (5·6-6·1)	2·7 (2·3-3·3)	2·6 (1·8-3·8)	1·3 (0·6-2·3)
Romania	40·4 (34·8-46·9)	20·2 (16·9-23·8)	19·7 (15·8-24·0)	13·0 (6·0-23·3)	98·5 (97·6-99·2)	99·3 (98·8-99·7)	99·4 (98·8-99·7)	99·8 (99·0-100·0)	23·3 (22·7-23·9)	9·9 (9·5-10·3)	9·5 (8·2-11·0)	4·0 (2·8-5·6)	9·9 (9·3-10·5)	4·7 (4·3-5·1)	4·5 (3·8-5·3)	2·3 (1·4-3·3)
Serbia	16·8 (14·0-20·1)	17·0 (14·0-20·7)	17·6 (14·4-21·5)	16·6 (10·5-26·5)	98·9 (98·1-99·4)	99·6 (99·3-99·8)	99·6 (99·3-99·8)	99·8 (99·3-100·0)	15·1 (12·5-18·2)	8·0 (7·1-9·0)	7·9 (6·6-9·3)	4·7 (2·5-7·4)	8·5 (7·0-10·1)	4·6 (4·0-5·1)	4·5 (3·7-5·3)	2·5 (1·4-4·0)
Slovakia	13·2 (11·3-15·2)	7·4 (5·9-9·2)	7·2 (5·6-9·4)	5·0 (2·4-9·0)	99·0 (98·2-99·4)	99·5 (99·0-99·8)	99·5 (99·1-99·8)	99·8 (99·3-100·0)	9·9 (9·3-10·6)	6·3 (5·1-7·5)	6·1 (4·6-8·0)	3·8 (1·7-7·7)	5·0 (4·7-5·4)	3·1 (2·6-3·7)	3·0 (2·3-3·9)	1·9 (0·9-3·7)
Slovenia	11·5 (9·8-13·5)	6·9 (5·5-8·5)	6·3 (5·0-7·9)	3·4 (1·6-6·9)	99·0 (98·3-99·4)	99·6 (99·2-99·8)	99·6 (99·2-99·8)	99·8 (99·4-100·0)	5·4 (5·0-5·9)	2·4 (2·2-2·8)	2·4 (2·0-2·8)	1·2 (0·8-1·8)	3·2 (2·9-3·5)	1·5 (1·4-1·7)	1·5 (1·3-1·7)	0·8 (0·6-1·1)
**Central Asia**																
Armenia	53·8 (46·5-61·7)	17·5 (14·7-20·9)	17·9 (14·4-22·2)	10·6 (4·8-19·1)	97·9 (96·8-98·7)	99·4 (98·1-99·9)	99·4 (98·0-99·9)	99·5 (95·7-100·0)	30·7 (26·1-36·4)	12·8 (9·5-16·7)	12·1 (8·9-16·1)	5·6 (2·4-11·4)	15·5 (13·2-18·3)	6·5 (4·8-8·5)	6·1 (4·5-8·2)	2·8 (1·1-5·6)

Location	Indicator 3.3.1: Age-standardised rate of new HIV infections (per 1,000 population)				Indicator 3.3.2: Age-standardised rate of tuberculosis cases (per 100,000 population)				Indicator 3.3.3: Age-standardised rate of malaria cases (per 1,000 population)				Indicator 3.3.4: Age-standardised rate of hepatitis B incidence (per 100,000 population)			
	**2000**	**2015**	**2016**	**2030**	**2000**	**2015**	**2016**	**2030**	**2000**	**2015**	**2016**	**2030**	**2000**	**2015**	**2016**	**2030**
Sweden	0·05 (0·03-0·09)	0·06 (0·02-0·09)	0·06 (0·02-0·09)	0·05 (0·01-0·08)	5·7 (4·8-6·8)	7·1 (5·8-8·5)	7·0 (5·7-8·5)	6·2 (4·8-7·9)	0·0 (0·0-0·0)	0·0 (0·0-0·0)	0·0 (0·0-0·0)	0·0 (0·0-0·0)	94·4 (73·2-121·3)	72·2 (58·0-89·6)	72·3 (58·1-89·7)	74·1 (59·6-92·5)
Switzerland	0·09 (0·04-0·1)	0·09 (0·03-0·1)	0·09 (0·03-0·1)	0·09 (0·03-0·1)	8·5 (6·4-10·9)	6·3 (4·8-8·1)	6·3 (4·8-8·1)	5·4 (4·1-7·0)	0·0 (0·0-0·0)	0·0 (0·0-0·0)	0·0 (0·0-0·0)	0·0 (0·0-0·0)	402·8 (303·9-517·7)	306·5 (231·0-391·4)	302·2 (227·3-385·2)	245·8 (182·8-312·7)
United Kingdom	0·1 (0·08-0·2)	0·1 (0·06-0·2)	0·1 (0·06-0·2)	0·09 (0·05-0·1)	12·6 (10·3-15·1)	11·3 (9·2-13·8)	11·1 (9·0-13·5)	8·2 (6·6-10·0)	0·0 (0·0-0·0)	0·0 (0·0-0·0)	0·0 (0·0-0·0)	0·0 (0·0-0·0)	186·4 (143·3-237·7)	97·2 (78·2-119·1)	93·7 (75·7-114·3)	55·6 (46·8-66·8)
**Southern Latin America**																
Argentina	0·2 (0·1-0·3)	0·2 (0·1-0·3)	0·2 (0·1-0·3)	0·1 (0·09-0·2)	31·5 (27·0-36·4)	21·4 (18·2-24·9)	21·8 (18·6-25·4)	20·5 (16·9-25·3)	0·01 (0·01-0·01)	0·0 (0·0-0·0)	0·0 (0·0-0·0)	0·0 (0·0-0·0)	330·9 (252·1-422·5)	210·9 (166·4-265·2)	207·1 (163·4-260·1)	158·9 (126·0-201·2)
Chile	0·1 (0·08-0·2)	0·07 (0·04-0·1)	0·06 (0·03-0·1)	0·05 (0·03-0·1)	22·6 (19·6-26·2)	15·0 (13·1-17·2)	14·8 (12·9-16·9)	11·0 (9·6-12·5)	0·0 (0·0-0·0)	0·0 (0·0-0·0)	0·0 (0·0-0·0)	0·0 (0·0-0·0)	189·4 (145·3-243·2)	113·4 (88·9-144·1)	112·9 (88·5-143·5)	82·9 (64·8-106·5)
Uruguay	0·2 (0·1-0·3)	0·2 (0·1-0·3)	0·2 (0·1-0·3)	0·1 (0·09-0·2)	20·7 (17·7-24·0)	19·6 (16·9-22·5)	19·8 (17·1-22·8)	23·3 (20·3-26·6)	0·0 (0·0-0·0)	0·0 (0·0-0·0)	0·0 (0·0-0·0)	0·0 (0·0-0·0)	209·3 (164·0-261·4)	163·9 (128·3-203·8)	161·1 (126·0-200·0)	125·6 (97·0-155·5)
**Eastern Europe**																
Belarus	0·08 (0·05-0·1)	0·2 (0·1-0·3)	0·2 (0·1-0·3)	0·2 (0·1-0·3)	49·5 (41·8-58·3)	36·8 (32·4-41·6)	35·3 (31·0-40·0)	24·0 (18·7-31·0)	0·0 (0·0-0·0)	0·0 (0·0-0·0)	0·0 (0·0-0·0)	0·0 (0·0-0·0)	536·3 (414·5-690·4)	356·5 (281·2-445·6)	343·0 (271·5-431·7)	225·7 (177·1-285·3)
Estonia	0·06 (0·04-0·08)	0·07 (0·05-0·1)	0·07 (0·05-0·1)	0·06 (0·04-0·09)	45·7 (39·5-52·5)	19·5 (16·6-22·7)	18·8 (16·0-21·9)	11·1 (9·3-13·1)	0·0 (0·0-0·0)	0·0 (0·0-0·0)	0·0 (0·0-0·0)	0·0 (0·0-0·0)	297·8 (225·6-394·9)	183·7 (143·8-234·1)	182·1 (142·9-231·6)	160·1 (126·4-202·5)
Latvia	0·06 (0·05-0·07)	0·08 (0·07-0·1)	0·08 (0·07-0·1)	0·07 (0·06-0·09)	66·1 (57·9-74·9)	34·2 (29·8-38·8)	33·2 (28·9-37·8)	21·7 (18·6-25·0)	0·0 (0·0-0·0)	0·0 (0·0-0·0)	0·0 (0·0-0·0)	0·0 (0·0-0·0)	421·2 (331·9-536·5)	463·8 (364·6-585·8)	445·5 (351·1-560·4)	253·5 (197·4-317·5)
Lithuania	0·03 (0·03-0·05)	0·02 (0·004-0·04)	0·02 (0·004-0·04)	0·02 (0·003-0·03)	64·0 (55·6-72·9)	44·6 (38·9-50·8)	43·4 (37·8-49·5)	28·8 (24·8-33·0)	0·0 (0·0-0·0)	0·0 (0·0-0·0)	0·0 (0·0-0·0)	0·0 (0·0-0·0)	352·5 (281·6-441·8)	314·3 (251·7-395·0)	302·6 (243·1-378·8)	179·1 (144·5-223·0)
Moldova	0·1 (0·08-0·1)	0·07 (0·05-0·1)	0·07 (0·04-0·1)	0·07 (0·05-0·1)	68·6 (59·9-78·7)	68·8 (60·3-78·7)	66·4 (58·0-76·0)	43·0 (37·5-48·8)	0·0 (0·0-0·0)	0·0 (0·0-0·0)	0·0 (0·0-0·0)	0·0 (0·0-0·0)	901·6 (660·9-1,276·0)	304·5 (236·1-403·8)	301·0 (233·4-398·6)	253·1 (195·9-332·9)
Russia	0·2 (0·1-0·3)	0·2 (0·1-0·4)	0·2 (0·1-0·4)	0·2 (0·1-0·4)	80·4 (70·3-92·3)	62·7 (54·6-72·3)	60·9 (52·9-70·3)	50·1 (43·0-57·9)	0·0 (0·0-0·0)	0·0 (0·0-0·0)	0·0 (0·0-0·0)	0·0 (0·0-0·0)	517·1 (387·5-709·0)	306·2 (239·2-397·1)	299·2 (234·3-386·1)	215·4 (173·9-267·1)
Ukraine	0·4 (0·3-0·6)	0·3 (0·2-0·4)	0·4 (0·3-0·5)	0·3 (0·3-0·5)	63·6 (55·8-71·9)	59·2 (52·0-67·3)	57·6 (50·8-65·2)	38·6 (35·1-43·4)	0·0 (0·0-0·0)	0·0 (0·0-0·0)	0·0 (0·0-0·0)	0·0 (0·0-0·0)	486·1 (372·3-628·8)	420·7 (324·6-538·0)	420·4 (324·5-537·4)	411·9 (316·5-534·8)
**Central Europe**																
Albania	0·001 (0·001-0·001)	0·001 (0·001-0·001)	0·001 (0·001-0·001)	0·001 (0·001-0·001)	20·8 (18·2-23·7)	13·0 (11·4-14·9)	12·8 (11·2-14·6)	9·2 (7·9-10·6)	0·0 (0·0-0·0)	0·0 (0·0-0·0)	0·0 (0·0-0·0)	0·0 (0·0-0·0)	336·3 (262·7-440·2)	163·0 (130·2-206·7)	161·8 (129·2-205·1)	72·3 (58·4-88·3)
Bosnia and Herzegovina	0·001 (0·001-0·001)	0·002 (0·001-0·002)	0·002 (0·001-0·002)	0·001 (0·001-0·002)	35·1 (31·2-39·1)	25·6 (22·6-28·6)	25·0 (22·0-27·9)	17·7 (15·5-19·9)	0·0 (0·0-0·0)	0·0 (0·0-0·0)	0·0 (0·0-0·0)	0·0 (0·0-0·0)	245·4 (190·6-315·2)	208·5 (162·3-269·4)	202·1 (157·4-260·5)	130·7 (103·4-163·7)
Bulgaria	0·01 (0·01-0·02)	0·01 (0·007-0·02)	0·01 (0·007-0·02)	0·02 (0·008-0·03)	33·6 (29·5-38·1)	21·0 (18·7-23·3)	19·9 (17·8-22·1)	11·8 (10·4-13·1)	0·0 (0·0-0·0)	0·0 (0·0-0·0)	0·0 (0·0-0·0)	0·0 (0·0-0·0)	667·0 (508·5-876·2)	387·5 (299·8-504·0)	378·0 (292·9-491·2)	264·6 (206·6-340·7)
Croatia	0·004 (0·002-0·007)	0·004 (0·002-0·007)	0·004 (0·002-0·007)	0·004 (0·001-0·006)	24·5 (20·9-28·6)	11·2 (9·8-12·7)	10·7 (9·4-12·2)	5·7 (4·9-6·5)	0·0 (0·0-0·0)	0·0 (0·0-0·0)	0·0 (0·0-0·0)	0·0 (0·0-0·0)	165·1 (129·7-206·2)	134·2 (107·2-166·5)	132·3 (105·9-164·5)	108·7 (87·9-133·8)
Czech Republic	0·005 (0·003-0·008)	0·008 (0·003-0·01)	0·008 (0·003-0·01)	0·007 (0·003-0·01)	11·0 (9·6-12·4)	5·4 (4·6-6·2)	5·2 (4·5-6·0)	3·6 (3·1-4·2)	0·0 (0·0-0·0)	0·0 (0·0-0·0)	0·0 (0·0-0·0)	0·0 (0·0-0·0)	138·6 (108·7-173·5)	135·5 (105·5-170·8)	133·4 (104·1-167·6)	106·9 (85·8-132·7)
Hungary	0·007 (0·006-0·009)	0·006 (0·004-0·008)	0·006 (0·004-0·008)	0·005 (0·004-0·007)	20·6 (18·0-23·5)	8·4 (7·4-9·6)	7·9 (7·0-9·0)	3·3 (2·8-3·9)	0·0 (0·0-0·0)	0·0 (0·0-0·0)	0·0 (0·0-0·0)	0·0 (0·0-0·0)	157·8 (125·1-200·7)	123·3 (98·8-154·5)	122·6 (98·2-153·5)	98·1 (79·2-122·9)
Macedonia	0·001 (0·001-0·002)	0·002 (0·001-0·003)	0·002 (0·001-0·003)	0·002 (0·001-0·003)	44·6 (39·5-50·2)	21·2 (18·6-24·0)	20·9 (18·3-23·6)	15·9 (13·8-18·3)	0·0 (0·0-0·0)	0·0 (0·0-0·0)	0·0 (0·0-0·0)	0·0 (0·0-0·0)	327·7 (255·7-418·3)	204·6 (161·3-256·4)	204·2 (161·1-256·1)	196·7 (155·8-249·7)
Montenegro	0·003 (0·002-0·004)	0·004 (0·002-0·006)	0·004 (0·002-0·006)	0·003 (0·002-0·005)	21·1 (18·0-24·3)	15·2 (12·9-17·7)	15·0 (12·7-17·4)	11·7 (9·9-13·6)	0·0 (0·0-0·0)	0·0 (0·0-0·0)	0·0 (0·0-0·0)	0·0 (0·0-0·0)	388·6 (298·6-496·6)	241·9 (188·2-305·9)	237·6 (185·1-300·2)	183·4 (144·8-226·9)
Poland	0·01 (0·007-0·02)	0·01 (0·005-0·02)	0·01 (0·005-0·02)	0·01 (0·004-0·02)	22·7 (19·8-25·7)	13·3 (11·7-15·0)	13·0 (11·4-14·6)	9·2 (8·0-10·3)	0·0 (0·0-0·0)	0·0 (0·0-0·0)	0·0 (0·0-0·0)	0·0 (0·0-0·0)	167·3 (126·6-216·8)	89·5 (71·2-110·9)	89·2 (71·0-110·5)	57·8 (46·6-70·1)
Romania	0·01 (0·008-0·02)	0·03 (0·01-0·05)	0·03 (0·02-0·05)	0·04 (0·02-0·05)	99·7 (87·3-112·3)	64·2 (56·6-71·7)	61·9 (54·7-69·2)	37·5 (33·1-41·9)	0·0 (0·0-0·0)	0·0 (0·0-0·0)	0·0 (0·0-0·0)	0·0 (0·0-0·0)	322·8 (249·9-416·7)	389·4 (299·0-502·3)	388·3 (298·4-501·6)	373·1 (279·5-485·3)
Serbia	0·008 (0·001-0·02)	0·004 (0·002-0·008)	0·004 (0·002-0·008)	0·005 (0·003-0·009)	33·5 (29·8-37·9)	19·7 (17·5-22·2)	19·3 (17·1-21·7)	13·9 (12·2-15·9)	0·0 (0·0-0·0)	0·0 (0·0-0·0)	0·0 (0·0-0·0)	0·0 (0·0-0·0)	223·2 (172·8-284·2)	143·8 (114·7-181·4)	143·1 (114·2-180·5)	131·6 (103·4-166·3)
Slovakia	0·002 (0·001-0·003)	0·003 (0·001-0·004)	0·003 (0·001-0·004)	0·003 (0·001-0·004)	12·3 (10·8-14·1)	7·4 (6·4-8·5)	7·1 (6·2-8·2)	4·2 (3·6-5·0)	0·0 (0·0-0·0)	0·0 (0·0-0·0)	0·0 (0·0-0·0)	0·0 (0·0-0·0)	178·0 (139·8-224·7)	128·7 (102·9-159·8)	127·1 (101·6-157·7)	106·3 (85·1-131·7)
Slovenia	0·003 (0·002-0·005)	0·003 (0·002-0·006)	0·003 (0·002-0·006)	0·003 (0·002-0·005)	14·9 (12·7-17·1)	7·3 (6·3-8·5)	7·1 (6·1-8·2)	4·5 (3·8-5·2)	0·0 (0·0-0·0)	0·0 (0·0-0·0)	0·0 (0·0-0·0)	0·0 (0·0-0·0)	144·4 (114·0-181·1)	110·6 (88·4-137·4)	109·4 (87·4-136·0)	93·2 (74·6-118·1)
**Central Asia**																
Armenia	0·008 (0·003-0·02)	0·02 (0·01-0·04)	0·03 (0·01-0·05)	0·02 (0·01-0·04)	47·4 (41·0-55·4)	37·0 (31·8-43·1)	35·6 (30·3-41·7)	22·3 (17·6-29·0)	0·0 (0·0-0·0)	0·0 (0·0-0·0)	0·0 (0·0-0·0)	0·0 (0·0-0·0)	485·5 (371·1-628·3)	434·6 (335·1-560·4)	405·7 (314·0-522·3)	276·0 (218·4-344·2)

Location	**Indicator 3.3.5:** **Age-standardised prevalence* of the sum of 15 neglected tropical diseases (NTDs) (%)** **Prevalence estimates reported here may exceed 100% as they reflect the sum of prevalent cases of 15 NTDs.*				**Indicator 3.4.1:** **Age-standardised death rate due to cardiovascular disease, cancer, diabetes, and chronic respiratory disease in populations aged 30-70 (per 100,000 population)**				**Indicator 3.4.2:** **Age-standardised death rate due to self-harm (per 100,000 population)**				**Indicator 3.5.2:** **Risk-weighted prevalence of alcohol consumption, as measured by the summary exposure value (SEV) for alcohol use (%)**			
	**2000**	**2015**	**2016**	**2030**	**2000**	**2015**	**2016**	**2030**	**2000**	**2015**	**2016**	**2030**	**2000**	**2015**	**2016**	**2030**
Sweden	0·0 (0·0-0·0)	0·0 (0·0-0·0)	0·0 (0·0-0·0)	0·0 (0·0-0·0)	271·0 (262·8-279·1)	196·8 (180·2-215·0)	197·7 (174·5-223·2)	153·0 (116·2-190·3)	13·6 (11·7-14·9)	12·1 (9·9-14·0)	12·1 (9·7-14·2)	11·0 (7·5-15·4)	17·3 (13·1-22·3)	16·5 (11·0-23·4)	16·6 (11·0-23·7)	17·6 (10·3-27·5)
Switzerland	0·0 (0·0-0·0)	0·0 (0·0-0·0)	0·0 (0·0-0·0)	0·0 (0·0-0·0)	251·5 (235·5-268·3)	175·6 (149·6-203·4)	175·0 (136·4-224·4)	126·5 (74·5-195·9)	18·2 (16·5-21·8)	11·8 (9·4-17·1)	11·8 (8·6-17·8)	7·7 (3·9-15·0)	16·9 (12·8-21·5)	15·4 (10·5-21·7)	15·4 (10·3-21·9)	14·8 (8·5-24·3)
United Kingdom	0·0 (0·0-0·0)	0·0 (0·0-0·0)	0·0 (0·0-0·0)	0·0 (0·0-0·0)	347·2 (343·4-351·0)	242·6 (238·4-247·1)	241·4 (237·0-246·2)	177·7 (168·1-188·2)	9·1 (7·5-9·9)	7·9 (6·6-8·6)	7·9 (6·6-8·5)	7·1 (5·9-8·2)	15·9 (9·9-23·8)	16·7 (10·1-25·2)	16·8 (10·2-25·2)	17·5 (10·6-26·1)
**Southern Latin America**																
Argentina	18·4 (17·2-19·7)	15·4 (14·2-16·6)	15·2 (14·0-16·4)	12·6 (11·6-13·7)	444·0 (428·8-461·5)	353·6 (331·9-375·8)	351·4 (325·0-379·5)	283·9 (236·4-336·6)	12·5 (9·9-13·6)	11·3 (9·3-13·0)	11·2 (9·2-12·9)	10·8 (8·0-14·3)	17·6 (13·0-22·3)	16·3 (10·5-23·4)	16·5 (10·5-23·8)	18·6 (10·3-29·3)
Chile	26·2 (21·8-30·9)	25·6 (21·0-30·8)	25·5 (21·0-30·7)	24·7 (20·1-29·9)	300·9 (283·3-318·8)	236·1 (201·3-273·0)	234·7 (182·2-295·9)	189·2 (115·0-296·9)	13·3 (11·2-15·1)	10·8 (8·6-14·0)	10·9 (7·9-15·4)	9·1 (4·2-15·8)	12·8 (9·8-16·1)	16·9 (11·7-23·2)	17·3 (11·8-23·8)	22·4 (14·2-32·3)
Uruguay	1·4 (1·1-1·7)	0·9 (0·7-1·0)	0·8 (0·7-1·0)	0·5 (0·4-0·6)	457·7 (444·6-471·3)	365·6 (347·1-384·7)	361·7 (338·6-384·4)	295·7 (249·1-346·0)	16·6 (12·9-18·0)	17·0 (13·1-18·9)	16·8 (12·8-18·9)	16·1 (11·8-21·1)	16·0 (12·7-19·9)	14·0 (9·1-19·9)	14·1 (9·1-20·1)	15·0 (8·6-23·1)
**Eastern Europe**																
Belarus	0·1 (0·0-0·2)	0·0 (0·0-0·1)	0·0 (0·0-0·1)	0·0 (0·0-0·0)	811·0 (772·5-852·3)	555·5 (495·6-617·1)	547·7 (458·7-637·6)	421·4 (249·2-613·5)	37·4 (27·0-41·6)	22·9 (18·8-28·9)	23·0 (17·8-30·6)	16·7 (8·2-28·6)	24·7 (20·0-29·3)	28·0 (21·7-34·6)	28·1 (21·7-34·7)	29·2 (21·7-36·6)
Estonia	0·0 (0·0-0·0)	0·0 (0·0-0·0)	0·0 (0·0-0·0)	0·0 (0·0-0·0)	618·8 (594·7-645·3)	364·1 (332·1-393·8)	359·4 (315·3-401·4)	285·0 (171·2-383·7)	30·0 (25·2-34·2)	15·6 (12·7-23·6)	15·5 (12·1-23·5)	12·3 (6·0-23·1)	16·2 (12·7-20·1)	24·8 (18·5-31·6)	24·9 (18·4-31·8)	25·8 (17·4-34·7)
Latvia	0·0 (0·0-0·1)	0·0 (0·0-0·0)	0·0 (0·0-0·0)	0·0 (0·0-0·0)	647·8 (614·0-682·6)	493·5 (445·9-538·1)	488·0 (428·6-543·9)	368·7 (246·5-493·6)	30·5 (25·1-34·2)	19·4 (16·1-26·3)	19·4 (15·5-26·7)	14·1 (8·0-25·0)	17·6 (13·9-21·3)	21·9 (15·9-27·6)	21·9 (15·8-27·7)	22·4 (14·8-30·0)
Lithuania	0·0 (0·0-0·1)	0·0 (0·0-0·0)	0·0 (0·0-0·0)	0·0 (0·0-0·0)	535·5 (513·7-558·5)	455·9 (431·4-481·6)	449·9 (417·5-483·9)	373·1 (286·8-474·1)	42·0 (31·6-46·4)	31·4 (26·0-35·8)	31·0 (25·5-36·2)	25·4 (17·6-35·1)	18·9 (15·6-22·5)	26·8 (21·7-32·1)	26·9 (21·7-32·3)	28·3 (21·7-35·3)
Moldova	0·1 (0·1-0·3)	0·1 (0·0-0·1)	0·1 (0·0-0·1)	0·0 (0·0-0·1)	713·7 (668·0-763·7)	558·8 (504·5-618·9)	553·2 (493·8-618·0)	479·6 (348·7-674·8)	18·5 (15·0-21·2)	15·0 (12·1-18·3)	15·1 (12·0-18·6)	15·1 (9·5-22·8)	23·8 (15·9-31·3)	20·3 (12·8-29·1)	20·3 (12·8-29·3)	20·5 (11·6-31·7)
Russia	1·3 (1·1-1·4)	1·2 (1·1-1·4)	1·2 (1·1-1·4)	1·2 (1·1-1·4)	888·5 (811·4-972·4)	612·0 (503·5-740·5)	611·0 (446·2-817·8)	454·2 (210·7-841·1)	45·9 (34·9-53·8)	30·5 (23·3-40·4)	30·6 (20·6-43·6)	26·0 (9·8-56·2)	16·9 (13·0-20·9)	21·2 (15·7-27·0)	21·2 (15·7-27·0)	21·2 (14·3-28·0)
Ukraine	0·4 (0·3-0·5)	0·4 (0·3-0·4)	0·4 (0·3-0·4)	0·3 (0·2-0·4)	810·9 (762·7-855·4)	578·7 (497·3-665·4)	579·5 (455·0-732·9)	545·3 (262·9-959·3)	32·3 (26·0-36·4)	20·0 (15·8-27·2)	20·2 (14·0-29·1)	17·5 (7·2-33·9)	26·7 (20·7-32·6)	34·0 (26·7-40·6)	33·9 (26·4-40·6)	32·6 (22·7-41·8)
**Central Europe**																
Albania	0·0 (0·0-0·1)	0·0 (0·0-0·0)	0·0 (0·0-0·0)	0·0 (0·0-0·0)	388·6 (376·5-401·7)	340·2 (301·5-378·8)	336·9 (296·3-377·8)	289·3 (210·0-375·0)	5·9 (5·2-6·8)	5·8 (4·3-7·0)	5·7 (4·2-6·9)	4·6 (3·0-7·1)	9·2 (6·6-12·0)	12·4 (8·5-17·3)	12·4 (8·4-17·5)	12·6 (7·5-19·8)
Bosnia and Herzegovina	0·1 (0·0-0·4)	0·0 (0·0-0·1)	0·0 (0·0-0·1)	0·0 (0·0-0·0)	511·2 (457·8-571·0)	423·6 (364·9-486·9)	415·4 (353·6-481·3)	342·3 (238·1-465·7)	10·0 (8·1-12·4)	7·3 (5·8-9·5)	7·3 (5·7-9·5)	5·6 (3·2-9·6)	16·2 (14·2-18·4)	15·1 (12·9-17·6)	15·1 (12·9-17·6)	14·8 (12·5-17·4)
Bulgaria	0·1 (0·0-0·2)	0·0 (0·0-0·1)	0·0 (0·0-0·1)	0·0 (0·0-0·0)	641·3 (619·7-663·9)	510·6 (453·0-575·8)	508·2 (431·9-597·7)	425·3 (294·1-568·2)	16·3 (14·3-18·7)	10·6 (8·5-15·4)	10·7 (8·2-15·2)	7·7 (4·4-14·0)	15·7 (12·2-19·1)	17·6 (12·6-22·7)	17·7 (12·6-23·1)	20·2 (13·0-28·1)
Croatia	0·0 (0·0-0·0)	0·0 (0·0-0·0)	0·0 (0·0-0·0)	0·0 (0·0-0·0)	518·1 (484·5-549·7)	372·9 (335·2-413·6)	372·8 (323·6-420·8)	278·9 (201·9-372·0)	19·5 (17·2-22·2)	14·2 (12·0-18·7)	14·3 (11·7-18·9)	10·9 (6·9-17·2)	23·0 (19·6-26·2)	19·2 (14·7-24·1)	19·0 (14·4-24·0)	16·3 (10·8-22·5)
Czech Republic	0·0 (0·0-0·0)	0·0 (0·0-0·0)	0·0 (0·0-0·0)	0·0 (0·0-0·0)	482·6 (469·8-493·8)	317·5 (305·9-329·0)	312·8 (295·6-330·2)	208·7 (184·3-233·9)	14·8 (13·1-17·1)	11·7 (9·5-15·0)	11·6 (9·4-14·8)	9·6 (6·9-13·8)	23·6 (19·9-27·7)	22·1 (16·9-28·0)	21·8 (16·5-27·8)	17·7 (12·3-24·2)
Hungary	0·0 (0·0-0·1)	0·0 (0·0-0·0)	0·0 (0·0-0·0)	0·0 (0·0-0·0)	658·3 (628·1-695·8)	503·9 (465·7-540·4)	502·5 (447·5-555·3)	395·4 (295·1-495·8)	25·9 (23·2-31·5)	18·2 (15·5-24·4)	18·5 (15·2-25·4)	13·3 (8·9-21·6)	24·7 (21·4-28·0)	21·7 (16·9-26·6)	21·3 (16·5-26·4)	16·8 (11·5-22·8)
Macedonia	0·0 (0·0-0·1)	0·0 (0·0-0·0)	0·0 (0·0-0·0)	0·0 (0·0-0·0)	629·2 (595·5-661·4)	505·1 (475·1-535·6)	497·4 (467·1-529·7)	403·3 (349·8-463·1)	9·5 (8·4-11·3)	8·4 (7·3-9·9)	8·4 (7·2-9·9)	7·8 (5·9-10·4)	7·0 (5·1-9·3)	5·4 (3·5-7·8)	5·4 (3·5-7·8)	5·8 (3·5-8·8)
Montenegro	0·0 (0·0-0·1)	0·0 (0·0-0·0)	0·0 (0·0-0·0)	0·0 (0·0-0·0)	532·3 (502·8-562·9)	432·3 (395·8-474·3)	427·7 (388·9-470·4)	362·5 (295·8-447·3)	11·1 (9·8-13·1)	10·8 (9·2-12·3)	11·1 (9·6-12·9)	10·1 (7·4-13·7)	16·6 (11·5-21·6)	19·8 (13·9-26·4)	20·0 (13·9-26·5)	22·2 (15·1-29·7)
Poland	0·0 (0·0-0·1)	0·0 (0·0-0·0)	0·0 (0·0-0·0)	0·0 (0·0-0·0)	544·9 (525·0-564·2)	387·4 (362·0-414·6)	380·0 (347·6-416·1)	288·5 (221·2-357·9)	16·9 (11·9-18·7)	14·7 (10·2-17·2)	14·6 (10·1-17·5)	12·4 (7·6-17·9)	15·3 (12·1-18·8)	17·7 (12·8-22·9)	17·8 (12·7-23·1)	18·5 (11·9-25·9)
Romania	0·1 (0·0-0·2)	0·0 (0·0-0·1)	0·0 (0·0-0·1)	0·0 (0·0-0·0)	602·6 (573·7-632·6)	463·1 (434·0-494·9)	458·0 (416·9-503·6)	372·5 (289·8-486·0)	11·1 (8·4-12·3)	10·1 (7·3-11·5)	10·0 (7·2-11·8)	9·3 (5·8-13·5)	25·8 (22·2-29·5)	26·1 (21·4-31·1)	26·0 (21·2-31·2)	25·3 (19·3-32·2)
Serbia	0·1 (0·1-0·2)	0·0 (0·0-0·1)	0·0 (0·0-0·1)	0·0 (0·0-0·0)	627·0 (585·8-663·8)	451·8 (425·1-479·2)	447·0 (419·5-474·0)	351·6 (303·9-401·2)	19·5 (17·4-21·6)	15·2 (13·7-17·8)	15·5 (13·9-18·2)	12·6 (9·8-16·3)	15·5 (11·4-19·7)	19·7 (13·8-25·7)	19·9 (13·9-25·9)	22·2 (15·1-29·1)
Slovakia	0·0 (0·0-0·0)	0·0 (0·0-0·0)	0·0 (0·0-0·0)	0·0 (0·0-0·0)	559·3 (536·4-583·5)	391·8 (359·1-427·9)	389·1 (344·7-436·5)	273·3 (203·3-350·2)	13·7 (11·4-15·0)	10·6 (8·8-12·4)	10·6 (8·6-12·8)	8·5 (5·5-12·9)	22·4 (18·6-26·3)	20·3 (15·0-26·1)	20·2 (14·8-26·2)	19·4 (12·7-27·4)
Slovenia	0·0 (0·0-0·0)	0·0 (0·0-0·0)	0·0 (0·0-0·0)	0·0 (0·0-0·0)	396·5 (365·4-428·4)	247·8 (216·3-278·4)	243·5 (208·9-278·0)	160·0 (114·9-206·9)	26·6 (22·3-29·9)	16·1 (13·3-21·7)	16·1 (13·0-21·9)	11·1 (7·0-18·8)	20·1 (16·4-24·1)	17·3 (12·4-23·0)	17·2 (12·2-23·0)	15·7 (9·7-23·1)
**Central Asia**																
Armenia	0·8 (0·6-1·1)	0·8 (0·6-1·0)	0·8 (0·6-1·0)	0·7 (0·6-1·0)	607·4 (575·8-642·4)	452·2 (422·6-485·2)	442·7 (398·0-490·1)	328·6 (224·4-443·3)	4·4 (3·9-6·0)	7·6 (4·8-8·8)	7·4 (4·7-9·0)	9·5 (3·9-16·4)	7·4 (5·3-9·6)	10·5 (6·9-14·7)	10·8 (7·1-15·2)	16·2 (10·1-23·1)

Location	Indicator 3.6.1: Age-standardised death rate due to road injuries (per 100,000 population)				Indicator 3.7.1: Proportion of women of reproductive age (15-49 years) who have their need for family planning satisfied with modern contraception methods (%)				Indicator 3.7.2: Number of livebirths per 1,000 women aged 10-14 years and women aged 15-19 years				Indicator 3.8.1: Coverage of essential health services, as defined by the UHC index comprised of the coverage of 9 tracer interventions and risk-standardised death rates from 32 causes amenable to personal healthcare (scale of 0 to 100)			
	**2000**	**2015**	**2016**	**2030**	**2000**	**2015**	**2016**	**2030**	**2000**	**2015**	**2016**	**2030**	**2000**	**2015**	**2016**	**2030**
Sweden	6·3 (5·9-6·7)	3·5 (3·1-3·9)	3·6 (3·1-4·1)	2·2 (1·6-2·9)	79·9 (74·5-84·6)	80·3 (75·1-85·1)	80·4 (75·0-85·0)	81·8 (71·0-90·0)	3·0 (3·0-3·1)	2·8 (2·6-3·0)	2·7 (2·4-3·1)	2·3 (1·6-3·1)	78·0 (76·7-79·0)	82·8 (80·6-84·8)	83·1 (80·7-85·2)	86·3 (83·1-89·3)
Switzerland	7·9 (7·1-8·6)	3·6 (3·0-4·4)	3·6 (2·8-4·8)	1·9 (1·0-3·1)	83·1 (78·2-87·6)	84·4 (80·1-88·4)	84·6 (80·1-88·4)	86·1 (77·5-92·6)	2·7 (2·7-2·8)	1·4 (1·3-1·6)	1·3 (1·2-1·5)	0·6 (0·5-0·8)	76·9 (75·6-78·2)	85·3 (81·9-88·5)	85·7 (82·1-89·3)	90·9 (84·7-95·6)
United Kingdom	7·1 (6·9-7·4)	3·8 (3·7-4·0)	3·8 (3·7-4·1)	2·3 (2·1-2·6)	86·8 (83·4-89·7)	88·5 (84·8-91·5)	88·6 (84·9-91·5)	89·8 (83·0-94·5)	13·3 (13·2-13·4)	6·8 (6·6-7·1)	6·3 (5·9-6·7)	2·1 (1·7-2·9)	70·3 (69·2-71·3)	77·1 (76·0-78·2)	77·5 (76·3-78·6)	82·0 (80·6-83·3)
**Southern Latin America**																
Argentina	15·3 (14·5-16·2)	14·9 (13·7-16·0)	14·4 (13·1-15·7)	14·4 (11·7-17·4)	67·3 (60·1-74·2)	74·0 (67·3-80·2)	74·2 (67·4-80·4)	79·6 (67·9-89·0)	31·6 (29·4-33·8)	30·3 (27·3-33·6)	30·5 (27·0-34·4)	29·6 (22·0-39·0)	56·1 (54·6-57·4)	61·0 (59·2-62·7)	61·4 (59·5-63·1)	66·1 (63·5-68·7)
Chile	15·5 (14·4-16·8)	11·9 (9·9-13·9)	11·7 (9·0-15·3)	9·0 (4·7-14·7)	69·5 (62·8-75·9)	76·2 (70·2-82·2)	76·5 (70·4-82·2)	81·8 (71·6-89·9)	27·4 (27·1-27·8)	25·2 (21·6-29·2)	24·8 (21·4-28·8)	20·8 (15·0-27·9)	62·3 (61·0-63·7)	70·4 (66·7-73·8)	70·8 (66·9-74·6)	77·0 (70·2-83·3)
Uruguay	16·1 (15·3-17·0)	16·5 (15·2-17·8)	16·0 (14·6-17·5)	16·5 (13·6-19·9)	66·3 (58·4-73·0)	72·9 (66·2-78·5)	73·2 (66·6-78·9)	78·4 (65·7-87·1)	31·7 (29·5-33·8)	28·5 (24·8-32·6)	28·3 (24·5-32·6)	26·7 (19·0-36·3)	59·4 (57·9-60·7)	64·2 (62·5-65·9)	64·6 (62·8-66·3)	69·8 (67·2-72·5)
**Eastern Europe**																
Belarus	23·4 (21·7-25·6)	13·5 (11·8-15·5)	13·4 (11·1-16·2)	9·3 (5·4-13·2)	71·8 (65·6-77·8)	71·7 (67·3-75·6)	72·2 (67·2-76·4)	78·2 (64·7-88·2)	14·1 (13·6-14·6)	9·1 (8·5-9·8)	8·2 (7·6-8·9)	5·3 (4·2-6·5)	58·6 (57·0-59·9)	69·2 (66·5-72·0)	70·1 (67·3-72·9)	80·3 (76·2-84·5)
Estonia	18·6 (17·3-20·2)	6·6 (5·7-7·5)	6·5 (5·5-7·7)	4·4 (2·7-6·3)	72·0 (65·2-78·1)	75·7 (69·5-81·5)	75·9 (69·3-81·6)	79·4 (67·0-89·0)	12·8 (12·7-13·0)	5·6 (5·2-5·9)	4·9 (4·5-5·3)	1·0 (0·8-1·4)	60·3 (58·9-61·6)	73·9 (71·8-76·0)	74·4 (72·2-76·6)	80·9 (77·7-84·1)
Latvia	25·8 (24·0-27·7)	10·2 (8·8-11·5)	10·2 (8·5-11·8)	6·0 (3·7-8·6)	66·0 (58·7-72·5)	73·1 (66·1-79·2)	73·2 (66·4-79·5)	76·8 (64·3-86·8)	9·3 (9·2-9·4)	6·9 (6·7-7·1)	6·2 (5·9-6·6)	3·4 (3·0-3·8)	60·9 (59·5-62·2)	68·6 (66·5-70·7)	69·4 (67·1-71·6)	78·1 (74·5-81·5)
Lithuania	23·0 (21·6-24·7)	11·4 (10·4-12·4)	11·4 (10·2-12·6)	6·0 (4·1-8·4)	65·6 (57·9-72·3)	73·8 (66·6-79·7)	74·1 (66·8-79·9)	79·1 (64·7-87·8)	12·1 (12·0-12·3)	5·6 (5·3-6·0)	4·9 (4·6-5·4)	1·8 (1·6-2·1)	61·7 (60·2-62·9)	67·4 (65·9-68·9)	67·9 (66·3-69·4)	74·2 (72·0-76·4)
Moldova	17·3 (15·9-19·0)	11·1 (9·7-12·6)	10·9 (9·3-12·7)	8·6 (5·7-12·1)	62·0 (58·0-65·7)	59·7 (55·1-64·4)	60·3 (55·0-65·9)	61·5 (50·4-72·4)	18·2 (17·5-18·9)	12·6 (12·0-13·3)	11·7 (11·1-12·4)	8·7 (7·3-10·1)	54·5 (52·9-56·1)	62·9 (60·8-65·0)	63·7 (61·6-65·9)	73·5 (70·5-76·6)
Russia	27·9 (24·8-31·4)	17·9 (14·2-22·2)	17·8 (12·7-24·2)	13·7 (5·4-26·6)	65·7 (59·3-71·7)	69·9 (62·5-76·5)	70·2 (62·6-76·8)	74·2 (60·5-85·0)	14·6 (14·3-14·9)	11·4 (10·6-12·2)	10·2 (9·3-11·3)	4·8 (4·1-5·7)	51·9 (50·0-53·7)	62·3 (57·9-66·6)	62·6 (57·8-67·2)	69·1 (59·0-77·6)
Ukraine	19·7 (18·2-21·4)	13·1 (10·9-15·6)	13·2 (9·9-17·2)	12·2 (4·5-23·8)	59·3 (54·8-63·5)	67·3 (62·5-71·6)	67·6 (61·9-72·5)	71·0 (57·5-83·8)	16·6 (15·9-17·5)	12·0 (11·0-13·1)	10·8 (9·8-11·9)	6·9 (5·3-9·1)	54·6 (53·2-56·1)	62·2 (58·2-66·0)	62·5 (58·3-66·7)	67·9 (59·1-75·3)
**Central Europe**																
Albania	9·7 (8·9-10·9)	7·9 (6·8-9·0)	7·7 (6·6-8·9)	5·7 (3·8-8·5)	21·2 (17·9-25·1)	24·2 (19·0-30·3)	25·2 (19·7-31·7)	40·4 (22·9-60·4)	9·2 (8·6-9·9)	12·2 (9·9-14·8)	12·6 (10·2-15·4)	18·3 (11·4-28·3)	58·1 (56·6-59·4)	66·2 (63·7-68·7)	66·6 (64·1-69·1)	72·8 (69·4-76·2)
Bosnia and Herzegovina	6·3 (5·4-7·4)	6·7 (5·6-8·0)	6·7 (5·5-8·0)	6·6 (4·5-9·4)	30·8 (24·9-37·2)	25·8 (21·1-30·8)	26·4 (21·4-32·0)	26·4 (16·1-39·0)	9·5 (9·3-9·7)	4·3 (3·9-4·8)	3·8 (3·3-4·5)	1·3 (0·9-1·9)	58·8 (56·8-60·7)	64·7 (62·0-67·2)	65·0 (62·3-67·7)	69·8 (66·4-73·2)
Bulgaria	12·3 (11·5-13·0)	8·4 (7·1-9·9)	8·4 (6·8-10·2)	6·2 (3·2-9·9)	53·9 (46·2-61·6)	56·5 (48·3-64·8)	56·8 (48·8-65·1)	60·7 (44·8-76·0)	23·7 (23·1-24·3)	17·6 (15·8-19·7)	16·1 (14·0-18·5)	9·9 (7·6-13·5)	57·1 (55·7-58·2)	62·7 (60·2-65·2)	63·2 (60·6-65·9)	69·6 (64·6-74·5)
Croatia	15·8 (14·6-17·1)	9·2 (8·1-10·5)	9·2 (7·9-10·7)	5·1 (3·5-7·2)	55·4 (47·4-63·7)	52·2 (44·2-60·5)	52·3 (44·4-60·5)	53·6 (37·0-69·8)	8·0 (7·8-8·1)	4·7 (4·4-5·0)	4·2 (3·9-4·6)	2·2 (1·7-2·6)	64·0 (62·5-65·4)	72·0 (70·2-74·2)	72·5 (70·5-74·8)	78·2 (75·4-81·6)
Czech Republic	13·5 (12·9-14·3)	7·2 (6·7-7·8)	7·0 (6·4-7·7)	4·0 (3·1-5·1)	61·9 (54·6-68·7)	65·0 (57·0-72·6)	65·2 (57·4-72·9)	68·3 (52·3-81·3)	6·7 (6·6-6·9)	4·9 (4·4-5·4)	4·4 (3·7-5·1)	2·1 (1·6-3·1)	68·2 (66·7-69·3)	75·8 (74·1-77·2)	76·3 (74·7-77·8)	82·7 (80·7-84·7)
Hungary	13·9 (12·9-14·9)	7·2 (6·4-7·9)	7·2 (6·3-8·3)	4·2 (3·0-5·7)	64·1 (56·8-71·0)	63·8 (55·8-71·2)	64·0 (55·9-71·9)	66·6 (49·4-80·7)	11·8 (11·6-12·0)	9·5 (8·8-10·2)	8·8 (7·9-9·8)	6·0 (4·8-7·8)	62·0 (60·8-63·3)	69·6 (67·7-71·5)	69·9 (68·0-71·9)	75·4 (72·2-78·4)
Macedonia	8·2 (7·6-9·0)	7·8 (7·0-8·6)	7·7 (7·0-8·6)	7·5 (5·9-9·3)	20·5 (15·6-26·4)	20·7 (16·6-25·1)	21·6 (17·1-26·6)	22·2 (13·9-32·0)	15·0 (14·7-15·2)	8·6 (7·9-9·4)	8·1 (7·3-9·1)	4·7 (3·7-6·2)	56·1 (54·8-57·5)	63·2 (61·3-65·0)	63·5 (61·5-65·4)	68·8 (65·6-72·2)
Montenegro	10·9 (9·9-12·2)	9·1 (8·0-10·2)	9·0 (7·9-10·1)	7·7 (5·6-10·1)	45·6 (38·5-52·9)	40·4 (33·8-47·3)	40·7 (33·7-48·2)	40·3 (26·1-55·5)	9·5 (9·3-9·6)	6·3 (6·0-6·5)	6·1 (5·8-6·4)	3·7 (3·0-4·7)	60·0 (58·5-61·5)	69·0 (67·1-70·8)	69·4 (67·4-71·2)	74·8 (72·1-77·5)
Poland	18·6 (17·5-19·9)	10·1 (9·1-11·2)	9·8 (8·8-11·2)	5·8 (4·3-7·4)	46·4 (38·6-54·8)	50·3 (42·6-58·5)	50·7 (42·6-58·9)	57·3 (41·1-72·1)	8·9 (8·7-9·1)	6·7 (6·0-7·4)	6·3 (5·4-7·3)	3·8 (2·7-5·5)	62·2 (60·6-63·6)	71·4 (69·2-73·4)	72·0 (69·7-74·1)	79·4 (75·9-82·6)
Romania	14·6 (13·7-15·6)	10·2 (9·3-11·1)	10·1 (9·1-11·4)	7·3 (5·2-9·7)	47·0 (39·6-54·8)	52·2 (43·5-60·4)	52·5 (44·4-60·8)	57·8 (41·6-73·4)	18·6 (18·1-19·1)	17·3 (15·2-19·6)	16·2 (13·4-19·7)	11·5 (8·1-18·4)	58·3 (56·8-59·6)	65·6 (63·8-67·4)	66·2 (64·3-68·0)	72·9 (70·2-75·5)
Serbia	12·1 (11·1-13·2)	9·6 (8·7-10·6)	10·1 (9·1-11·2)	7·7 (6·2-9·7)	47·0 (40·2-54·3)	28·6 (23·7-33·7)	29·0 (23·7-34·6)	22·6 (14·5-32·2)	11·7 (11·3-12·2)	8·8 (8·3-9·3)	8·4 (7·7-9·1)	6·0 (5·0-7·4)	56·2 (54·6-57·6)	64·7 (63·2-66·2)	64·9 (63·5-66·4)	68·6 (66·6-70·8)
Slovakia	15·3 (14·3-16·4)	7·9 (6·9-9·1)	7·9 (6·7-9·3)	4·7 (3·4-6·2)	67·3 (60·1-73·9)	67·5 (59·7-74·3)	67·7 (59·9-74·6)	70·7 (55·7-82·9)	11·8 (11·5-12·0)	10·5 (9·7-11·3)	9·8 (8·8-10·9)	5·3 (4·0-7·7)	62·7 (61·1-64·3)	69·4 (67·2-71·7)	69·9 (67·6-72·3)	76·0 (72·8-79·5)
Slovenia	15·5 (14·1-17·0)	6·0 (5·1-6·9)	6·0 (5·0-7·1)	2·9 (1·9-4·0)	65·0 (57·7-71·5)	63·8 (56·3-71·0)	64·1 (56·6-71·4)	65·6 (50·3-78·7)	3·8 (3·8-3·9)	1·8 (1·6-1·9)	1·6 (1·3-1·8)	0·7 (0·5-1·0)	67·9 (66·4-69·4)	79·4 (77·1-81·7)	79·9 (77·6-82·3)	86·7 (83·5-89·6)
**Central Asia**																
Armenia	11·8 (10·9-12·8)	8·4 (7·6-9·2)	8·3 (7·2-9·6)	6·0 (4·1-8·3)	27·4 (25·4-29·6)	42·4 (34·9-50·6)	43·3 (35·2-51·8)	61·1 (41·9-79·3)	17·0 (16·2-17·9)	13·5 (12·3-15·1)	12·3 (11·0-13·9)	11·9 (9·3-15·0)	55·0 (53·6-56·4)	64·2 (62·2-66·1)	64·8 (62·8-66·8)	73·7 (70·8-76·5)

Location	Indicator 3.9.1: Age-standardised death rate attributable to household air pollution and ambient air pollution (per 100,000 population)				Indicator 3.9.2: Age-standardised death rate attributable to unsafe water, sanitation, and hygiene (WaSH) (per 100,000 population)				Indicator 3.9.3: Age-standardised death rate due to unintentional poisonings (per 100,000 population)				Indicator 3.a.1: Age-standardised prevalence of daily smoking in populations aged 10 and older (%)			
	2000	2015	2016	2030	2000	2015	2016	2030	2000	2015	2016	2030	2000	2015	2016	2030
Sweden	15·0 (11·4-19·2)	8·2 (5·8-11·0)	7·9 (5·6-10·7)	5·1 (3·4-7·2)	0·2 (0·2-0·3)	0·4 (0·2-0·6)	0·4 (0·2-0·6)	0·4 (0·2-0·7)	0·4 (0·3-0·4)	0·3 (0·2-0·4)	0·3 (0·2-0·4)	0·3 (0·2-0·5)	16·0 (15·3-16·7)	10·0 (9·7-10·5)	10·0 (9·6-10·5)	6·9 (6·3-7·6)
Switzerland	23·2 (19·1-27·5)	13·9 (10·4-17·8)	13·6 (10·1-17·4)	8·6 (5·8-12·0)	0·2 (0·1-0·2)	0·2 (0·1-0·3)	0·2 (0·1-0·3)	0·1 (0·1-0·3)	0·1 (0·1-0·2)	0·1 (0·1-0·1)	0·1 (0·1-0·1)	0·1 (0·0-0·1)	27·0 (26·4-27·5)	19·2 (18·1-20·3)	19·1 (18·0-20·4)	17·7 (15·0-21·0)
United Kingdom	37·0 (30·1-44·3)	21·5 (17·2-26·5)	21·2 (16·9-26·1)	12·2 (8·7-17·5)	0·4 (0·3-0·6)	0·4 (0·2-0·6)	0·4 (0·2-0·6)	0·3 (0·2-0·4)	0·4 (0·3-0·4)	0·2 (0·2-0·2)	0·2 (0·2-0·2)	0·1 (0·1-0·2)	22·1 (21·4-22·8)	19·5 (18·5-20·5)	19·4 (18·4-20·3)	16·9 (15·2-18·8)
**Southern Latin America**																
Argentina	48·3 (40·6-56·7)	34·9 (28·4-42·2)	34·2 (27·7-41·4)	25·3 (20·2-31·0)	2·5 (1·8-3·3)	1·5 (1·0-2·1)	1·5 (1·0-2·0)	0·9 (0·6-1·4)	0·8 (0·7-1·0)	0·6 (0·5-0·7)	0·5 (0·5-0·7)	0·4 (0·3-0·5)	22·9 (20·8-25·1)	18·3 (17·0-19·5)	18·3 (16·9-19·7)	14·4 (12·1-16·9)
Chile	44·5 (38·9-50·9)	26·0 (20·8-33·0)	25·4 (20·1-32·6)	14·8 (10·0-22·5)	1·9 (1·3-2·5)	0·9 (0·6-1·5)	0·9 (0·5-1·5)	0·6 (0·3-1·1)	0·4 (0·2-0·4)	0·2 (0·2-0·2)	0·2 (0·1-0·3)	0·1 (0·1-0·2)	30·9 (29·0-32·9)	26·0 (23·9-28·4)	25·8 (23·7-28·4)	22·5 (19·0-26·8)
Uruguay	38·1 (31·6-45·1)	25·2 (20·2-30·7)	24·6 (19·7-29·9)	17·4 (13·6-21·6)	1·8 (1·2-2·6)	1·3 (0·8-2·0)	1·3 (0·7-2·0)	0·8 (0·4-1·5)	0·5 (0·5-0·6)	0·4 (0·3-0·5)	0·4 (0·3-0·4)	0·3 (0·2-0·4)	27·5 (25·9-29·1)	21·0 (19·0-23·1)	21·0 (18·9-23·2)	18·5 (14·8-22·4)
**Eastern Europe**																
Belarus	107·8 (91·6-123·5)	72·2 (57·8-86·9)	69·2 (55·2-83·4)	50·4 (35·5-72·1)	0·8 (0·5-1·1)	0·2 (0·1-0·3)	0·2 (0·1-0·3)	0·1 (0·0-0·1)	3·1 (2·4-3·5)	1·9 (1·3-2·4)	1·8 (1·2-2·5)	1·1 (0·6-2·0)	26·0 (24·3-27·9)	26·2 (24·1-28·2)	26·0 (23·8-28·2)	25·7 (22·0-29·6)
Estonia	68·8 (52·4-87·9)	25·6 (15·4-43·1)	23·7 (14·0-40·8)	8·2 (3·5-18·8)	0·4 (0·3-0·7)	0·2 (0·1-0·3)	0·2 (0·1-0·2)	0·1 (0·1-0·2)	1·9 (1·3-2·2)	0·5 (0·4-0·9)	0·5 (0·3-0·9)	0·1 (0·1-0·5)	26·6 (25·7-27·5)	21·2 (20·0-22·6)	21·2 (19·8-22·8)	19·0 (15·6-22·3)
Latvia	87·9 (74·3-102·7)	54·6 (42·0-75·9)	52·4 (40·0-73·5)	28·7 (20·2-45·6)	0·5 (0·3-0·7)	0·2 (0·2-0·4)	0·2 (0·1-0·4)	0·1 (0·1-0·2)	2·8 (1·5-3·2)	0·8 (0·7-1·3)	0·8 (0·6-1·3)	0·3 (0·2-0·9)	28·4 (27·6-29·2)	28·4 (27·0-29·6)	28·2 (26·7-29·7)	28·6 (25·7-31·8)
Lithuania	64·0 (53·9-76·0)	48·9 (40·9-57·5)	47·2 (39·4-55·7)	28·4 (23·4-34·5)	0·4 (0·2-0·5)	0·3 (0·2-0·4)	0·3 (0·2-0·4)	0·2 (0·1-0·4)	2·0 (1·3-2·3)	0·6 (0·5-1·2)	0·6 (0·5-1·2)	0·4 (0·2-0·9)	24·1 (23·2-25·0)	23·9 (22·2-25·5)	23·8 (22·0-25·7)	24·0 (20·3-28·0)
Moldova	181·0 (159·8-202·9)	81·3 (67·1-97·4)	78·1 (64·2-93·8)	44·0 (34·8-55·3)	2·7 (1·9-3·7)	0·9 (0·6-1·3)	0·8 (0·5-1·2)	0·3 (0·1-0·5)	5·8 (3·2-6·7)	1·1 (0·8-2·8)	1·1 (0·8-2·7)	0·3 (0·1-1·6)	20·5 (19·0-22·1)	18·6 (17·4-19·8)	18·5 (17·2-19·8)	16·9 (14·6-19·6)
Russia	92·8 (77·7-108·3)	63·9 (47·6-84·4)	61·9 (45·7-82·7)	39·8 (25·7-60·1)	1·4 (1·0-1·8)	0·5 (0·3-0·8)	0·5 (0·3-0·8)	0·3 (0·2-0·6)	2·5 (2·2-3·2)	1·5 (1·2-2·1)	1·5 (1·1-2·2)	1·0 (0·5-1·9)	31·9 (31·1-32·6)	27·1 (26·1-28·2)	26·9 (25·6-28·2)	25·1 (21·8-28·4)
Ukraine	120·3 (104·1-136·6)	82·3 (63·9-105·9)	80·4 (61·9-104·1)	57·0 (40·2-82·2)	0·9 (0·6-1·3)	0·3 (0·2-0·5)	0·3 (0·2-0·5)	0·2 (0·1-0·4)	3·3 (2·7-3·7)	2·0 (1·5-2·6)	2·0 (1·4-2·7)	1·5 (0·6-2·9)	28·5 (27·4-29·5)	27·8 (25·9-29·7)	27·8 (25·6-29·9)	28·1 (22·9-33·4)
**Central Europe**																
Albania	122·0 (109·2-135·9)	67·6 (55·9-79·4)	64·9 (53·5-76·6)	37·3 (28·4-46·7)	1·4 (0·9-2·1)	0·4 (0·2-0·6)	0·4 (0·2-0·6)	0·2 (0·1-0·3)	1·0 (0·7-1·2)	0·4 (0·3-0·6)	0·4 (0·3-0·6)	0·3 (0·2-0·6)	16·9 (16·1-17·7)	17·0 (15·7-18·1)	16·9 (15·6-18·0)	16·5 (13·9-19·2)
Bosnia and Herzegovina	139·1 (121·4-158·5)	86·9 (72·4-102·5)	85·0 (70·6-100·6)	62·3 (47·4-77·9)	0·3 (0·2-0·5)	0·2 (0·1-0·3)	0·2 (0·1-0·3)	0·1 (0·0-0·2)	1·2 (0·9-1·5)	0·4 (0·3-0·8)	0·4 (0·3-0·8)	0·2 (0·1-0·6)	29·5 (28·4-30·6)	31·9 (30·1-33·9)	31·6 (29·7-33·6)	34·5 (30·4-38·6)
Bulgaria	120·7 (105·2-135·0)	81·6 (68·3-95·5)	79·5 (66·3-93·4)	55·8 (44·5-68·3)	0·6 (0·3-0·9)	0·3 (0·2-0·5)	0·3 (0·2-0·5)	0·2 (0·1-0·3)	1·3 (1·0-1·5)	0·5 (0·4-0·9)	0·5 (0·4-0·9)	0·2 (0·1-0·6)	33·9 (31·7-36·1)	31·7 (30·1-33·4)	31·3 (29·6-33·1)	30·3 (26·8-33·9)
Croatia	75·9 (65·1-86·5)	50·7 (42·0-60·1)	49·8 (41·2-59·2)	35·1 (26·6-43·5)	0·4 (0·2-0·5)	0·2 (0·1-0·3)	0·2 (0·1-0·3)	0·1 (0·1-0·2)	0·3 (0·2-0·4)	0·1 (0·1-0·3)	0·1 (0·1-0·3)	0·1 (0·0-0·2)	26·9 (25·7-28·1)	29·1 (27·4-30·9)	28·8 (27·0-30·8)	30·6 (26·3-34·9)
Czech Republic	64·0 (55·1-72·7)	39·4 (33·4-45·1)	38·0 (32·2-43·6)	23·4 (19·7-27·0)	0·2 (0·1-0·4)	0·6 (0·3-0·9)	0·6 (0·3-0·9)	1·0 (0·4-1·6)	0·4 (0·3-0·6)	0·2 (0·2-0·4)	0·2 (0·2-0·4)	0·1 (0·1-0·3)	26·6 (25·6-27·8)	23·7 (22·2-25·3)	23·5 (21·8-25·2)	22·3 (19·1-25·9)
Hungary	91·0 (79·0-104·7)	64·8 (53·9-75·8)	63·7 (52·9-74·7)	46·9 (38·1-56·6)	0·2 (0·1-0·3)	0·6 (0·3-0·9)	0·6 (0·3-1·0)	1·5 (0·6-2·5)	0·3 (0·3-0·6)	0·2 (0·2-0·4)	0·2 (0·2-0·4)	0·1 (0·1-0·3)	29·4 (28·0-30·9)	26·3 (24·7-27·9)	26·0 (24·4-27·8)	22·8 (19·6-26·2)
Macedonia	141·0 (121·3-167·6)	90·5 (77·0-109·8)	87·3 (74·1-106·0)	59·6 (48·3-76·6)	1·3 (0·7-2·0)	0·3 (0·2-0·5)	0·3 (0·1-0·5)	0·1 (0·0-0·2)	0·5 (0·5-0·7)	0·4 (0·3-0·5)	0·4 (0·3-0·5)	0·3 (0·2-0·4)	27·6 (25·6-29·7)	30·9 (28·9-32·9)	30·6 (28·5-32·7)	33·9 (29·6-38·8)
Montenegro	102·3 (89·7-115·4)	81·9 (70·6-94·1)	80·8 (69·7-93·1)	67·2 (56·8-79·0)	0·3 (0·2-0·5)	0·1 (0·1-0·2)	0·1 (0·1-0·2)	0·1 (0·0-0·1)	1·1 (0·9-1·3)	0·6 (0·5-0·8)	0·6 (0·5-0·8)	0·4 (0·2-0·7)	28·2 (25·9-30·5)	34·8 (33·0-36·4)	34·4 (32·5-36·3)	38·8 (33·7-43·7)
Poland	79·8 (69·5-90·4)	48·9 (42·2-56·2)	47·1 (40·6-54·2)	27·8 (23·3-32·7)	0·3 (0·2-0·5)	0·3 (0·2-0·4)	0·3 (0·2-0·4)	0·3 (0·1-0·4)	0·4 (0·3-0·4)	0·2 (0·2-0·2)	0·2 (0·1-0·2)	0·1 (0·1-0·2)	31·0 (29·1-32·8)	23·9 (22·5-25·2)	23·6 (22·2-25·1)	19·0 (16·4-21·9)
Romania	122·6 (108·5-136·0)	68·5 (57·5-80·3)	65·9 (55·2-77·4)	38·4 (31·0-46·4)	2·0 (1·4-2·6)	0·6 (0·3-0·8)	0·5 (0·3-0·8)	0·2 (0·1-0·4)	0·9 (0·6-1·0)	0·3 (0·3-0·5)	0·3 (0·3-0·5)	0·2 (0·1-0·4)	25·2 (23·7-26·7)	23·4 (22·1-24·8)	23·1 (21·6-24·7)	22·9 (19·7-26·5)
Serbia	127·1 (109·4-148·5)	69·5 (59·4-84·1)	67·4 (57·5-81·5)	43·0 (36·5-52·6)	0·3 (0·2-0·5)	0·2 (0·1-0·4)	0·2 (0·1-0·4)	0·2 (0·1-0·6)	0·6 (0·5-0·8)	0·3 (0·3-0·4)	0·3 (0·3-0·4)	0·2 (0·1-0·3)	30·3 (29·0-31·5)	29·1 (26·8-31·4)	28·9 (26·5-31·2)	30·5 (25·6-35·5)
Slovakia	79·4 (68·3-89·6)	51·4 (43·2-59·5)	49·9 (41·9-57·8)	32·6 (27·0-38·7)	0·5 (0·3-0·7)	0·3 (0·2-0·5)	0·3 (0·2-0·5)	0·2 (0·1-0·4)	0·7 (0·5-0·8)	0·3 (0·3-0·5)	0·3 (0·3-0·5)	0·2 (0·1-0·4)	22·0 (20·5-23·6)	19·4 (18·2-20·8)	19·3 (18·0-20·8)	16·6 (14·1-19·3)
Slovenia	49·3 (41·3-58·4)	26·0 (21·2-31·2)	25·4 (20·7-30·5)	15·8 (11·0-20·4)	0·3 (0·2-0·5)	0·2 (0·1-0·3)	0·2 (0·1-0·3)	0·1 (0·1-0·2)	0·6 (0·4-0·7)	0·2 (0·2-0·4)	0·2 (0·2-0·3)	0·1 (0·1-0·3)	21·8 (20·2-23·4)	21·9 (20·5-23·3)	21·7 (20·2-23·3)	20·1 (16·9-24·0)
**Central Asia**																
Armenia	110·7 (95·1-127·3)	74·9 (58·4-98·7)	71·7 (55·6-95·2)	38·9 (27·3-57·1)	4·2 (2·9-5·6)	0·7 (0·5-1·0)	0·7 (0·4-1·0)	0·2 (0·1-0·4)	1·2 (1·0-1·6)	0·7 (0·6-1·0)	0·7 (0·6-1·0)	0·5 (0·3-0·8)	24·9 (23·5-26·5)	22·3 (20·4-24·1)	22·0 (20·0-24·0)	19·8 (16·7-23·1)

Location	Indicator 3.b.1: Geometric mean of the coverage of eight vaccines, conditional on inclusion in national vaccine schedules, in target populations (%)				Indicator 5.2.1: Age-standardised prevalence of women aged 15 years and older who experienced physical or sexual violence by an intimate partner in the last 12 months (%)				Indicator 6.1.1: Risk-weighted prevalence of populations using unsafe or unimproved water sources, as measured by the summary exposure value (SEV) for unsafe water (%)				Indicator 6.2.1a: Risk-weighted prevalence of populations using unsafe or unimproved sanitation, as measured by the summary exposure value (SEV) for unsafe sanitation (%)			
	**2000**	**2015**	**2016**	**2030**	**2000**	**2015**	**2016**	**2030**	**2000**	**2015**	**2016**	**2030**	**2000**	**2015**	**2016**	**2030**
Sweden	96·8 (95·8-97·7)	97·3 (95·8-98·4)	97·5 (95·8-98·5)	98·7 (95·5-100·0)	14·9 (13·3-16·5)	14·1 (12·7-15·7)	14·1 (12·6-15·6)	13·3 (11·9-14·8)	0·7 (0·4-0·9)	0·6 (0·4-0·8)	0·6 (0·4-0·8)	0·6 (0·4-0·7)	2·3 (1·0-4·3)	1·6 (0·8-3·0)	1·6 (0·8-2·9)	1·2 (0·6-2·1)
Switzerland	89·0 (86·7-90·9)	90·5 (87·3-93·0)	90·7 (87·1-93·3)	96·6 (91·7-99·1)	14·2 (12·4-16·0)	12·5 (11·0-14·1)	12·4 (10·9-14·0)	11·1 (9·8-12·5)	0·6 (0·4-0·8)	0·6 (0·4-0·8)	0·6 (0·4-0·8)	0·6 (0·2-1·3)	1·9 (0·8-3·8)	1·3 (0·5-2·4)	1·2 (0·5-2·4)	0·9 (0·3-1·6)
United Kingdom	56·2 (34·8-81·8)	96·4 (94·7-97·7)	95·3 (93·6-96·5)	98·8 (96·4-99·9)	14·5 (12·9-16·2)	13·2 (11·8-14·7)	13·1 (11·8-14·6)	11·9 (10·7-13·3)	1·0 (0·7-1·1)	0·7 (0·5-0·8)	0·7 (0·5-0·8)	0·5 (0·3-0·6)	0·6 (0·5-0·7)	0·4 (0·3-0·5)	0·4 (0·3-0·5)	0·3 (0·2-0·3)
**Southern Latin America**																
Argentina	87·9 (79·7-92·0)	94·0 (89·2-96·2)	94·8 (90·4-97·0)	98·0 (92·0-100·0)	24·4 (21·3-28·0)	21·9 (19·1-25·1)	21·7 (18·9-24·8)	19·3 (16·8-22·1)	11·2 (7·2-14·2)	5·3 (2·9-8·1)	5·1 (2·8-7·9)	3·0 (1·3-5·7)	18·7 (12·3-26·5)	5·6 (2·7-10·2)	5·3 (2·5-9·6)	1·5 (0·4-3·2)
Chile	94·3 (92·5-95·7)	94·1 (91·6-95·9)	94·6 (91·9-96·5)	95·0 (88·5-98·3)	22·7 (19·6-26·3)	19·0 (16·5-21·9)	18·7 (16·3-21·6)	15·7 (13·7-17·8)	5·7 (3·4-8·8)	3·4 (1·7-5·6)	3·2 (1·7-5·4)	1·9 (1·0-3·1)	15·7 (9·5-24·4)	6·7 (3·1-12·6)	6·4 (3·0-12·1)	2·9 (1·0-6·1)
Uruguay	94·9 (93·0-96·3)	97·0 (94·9-98·3)	97·1 (94·7-98·4)	98·6 (94·2-100·0)	21·6 (18·9-24·7)	19·6 (17·2-22·4)	19·4 (17·0-22·1)	17·5 (15·3-19·9)	5·1 (3·2-7·2)	3·4 (2·1-5·0)	3·2 (2·0-4·9)	1·9 (1·1-3·3)	8·1 (4·6-12·9)	2·4 (1·1-4·7)	2·2 (1·0-4·3)	0·7 (0·2-1·5)
**Eastern Europe**																
Belarus	97·3 (93·1-98·9)	98·8 (92·9-99·8)	99·0 (94·0-99·9)	99·5 (98·1-100·0)	22·9 (20·4-25·7)	18·9 (16·7-21·2)	18·6 (16·4-20·9)	14·9 (12·9-17·0)	11·3 (7·5-13·4)	6·4 (4·1-7·9)	6·4 (4·1-7·9)	5·7 (3·3-7·2)	28·7 (16·6-43·5)	19·6 (9·3-34·4)	19·5 (9·1-34·8)	17·4 (6·3-39·5)
Estonia	94·2 (92·6-95·6)	95·2 (91·4-97·2)	95·2 (91·6-97·3)	97·8 (89·3-100·0)	19·1 (16·9-21·3)	15·1 (13·4-16·9)	14·9 (13·2-16·7)	12·3 (10·9-13·9)	9·0 (6·0-11·1)	7·6 (4·8-9·4)	7·5 (4·8-9·4)	6·8 (4·1-8·5)	15·5 (6·7-30·6)	17·4 (6·6-36·9)	17·4 (6·6-37·3)	18·0 (6·1-41·8)
Latvia	92·0 (88·0-94·7)	94·1 (91·0-96·2)	94·8 (91·6-96·8)	96·4 (86·9-99·9)	21·2 (18·9-23·6)	17·1 (15·2-19·0)	16·9 (15·1-18·8)	14·1 (12·5-15·9)	9·8 (6·8-11·8)	8·0 (5·2-9·8)	8·0 (5·2-9·7)	7·5 (4·7-9·1)	21·3 (10·0-38·6)	19·7 (7·2-41·2)	19·8 (7·2-41·6)	19·7 (6·0-44·4)
Lithuania	96·2 (95·2-97·2)	95·0 (90·8-97·1)	95·1 (91·6-97·2)	96·6 (87·7-100·0)	19·8 (17·6-22·2)	16·1 (14·3-18·1)	15·9 (14·1-17·9)	13·0 (11·4-14·6)	9·5 (6·8-11·6)	7·9 (5·1-9·7)	7·8 (5·0-9·6)	7·0 (4·1-8·7)	22·0 (7·0-50·6)	19·3 (7·3-40·3)	19·2 (7·2-40·0)	17·2 (6·6-35·1)
Moldova	95·3 (93·2-96·8)	90·2 (81·6-94·4)	91·0 (84·2-94·5)	76·6 (50·7-90·8)	28·2 (25·2-31·3)	24·1 (21·4-27·1)	23·7 (21·0-26·6)	19·2 (16·4-22·0)	25·3 (18·5-28·9)	18·9 (13·4-22·5)	18·4 (13·0-21·9)	11·6 (8·2-15·0)	63·4 (47·8-78·4)	52·3 (30·4-76·3)	51·5 (29·1-75·9)	41·1 (14·9-69·3)
Russia	84·9 (82·1-87·4)	84·0 (74·9-88·9)	83·8 (73·9-89·2)	84·8 (65·0-94·8)	24·8 (21·9-27·8)	20·6 (18·1-23·2)	20·4 (17·9-22·9)	17·2 (14·9-19·9)	10·2 (7·1-12·4)	8·5 (5·6-10·6)	8·5 (5·6-10·5)	7·8 (5·0-9·8)	35·7 (19·3-52·7)	26·9 (10·0-53·2)	26·7 (9·8-53·8)	23·0 (6·9-61·4)
Ukraine	96·4 (94·7-97·5)	63·4 (48·9-74·3)	62·0 (46·6-74·5)	41·4 (8·8-83·1)	24·5 (21·9-27·2)	21·1 (18·7-23·5)	20·9 (18·6-23·4)	19·3 (16·8-21·8)	11·7 (8·1-14·0)	10·7 (7·3-12·9)	10·6 (7·3-12·9)	9·0 (6·3-11·7)	28·2 (15·9-43·6)	32·7 (16·9-54·5)	32·3 (16·1-55·1)	41·2 (11·3-90·1)
**Central Europe**																
Albania	98·1 (97·0-98·8)	99·8 (99·5-99·9)	99·8 (99·5-99·9)	100·0 (99·8-100·0)	25·0 (22·1-28·3)	19·0 (16·8-21·5)	18·8 (16·7-21·3)	16·0 (13·9-18·3)	18·5 (13·3-24·6)	13·4 (9·6-19·9)	13·2 (9·5-19·6)	9·8 (3·8-20·4)	32·4 (22·3-44·0)	17·5 (5·9-35·0)	17·0 (5·2-35·2)	10·2 (0·8-36·4)
Bosnia and Herzegovina	93·7 (92·4-94·9)	95·9 (93·7-97·5)	96·2 (93·7-97·8)	98·5 (94·7-100·0)	19·2 (16·9-21·7)	18·2 (16·1-20·5)	18·0 (15·9-20·2)	14·9 (13·0-17·0)	9·7 (7·3-16·8)	7·9 (6·3-12·4)	7·8 (6·2-12·2)	6·9 (5·6-9·6)	18·6 (13·2-25·3)	7·8 (2·7-17·6)	7·6 (2·5-17·9)	3·1 (0·4-13·0)
Bulgaria	94·6 (92·8-96·1)	94·8 (92·2-96·7)	95·5 (92·8-97·3)	95·4 (88·8-98·6)	20·2 (18·0-22·5)	16·6 (14·8-18·5)	16·4 (14·6-18·3)	13·9 (12·3-15·5)	8·5 (6·5-12·3)	7·1 (5·7-8·2)	7·1 (5·6-8·1)	6·4 (5·0-7·3)	4·1 (1·1-11·1)	5·0 (1·3-13·3)	5·0 (1·3-13·4)	5·8 (1·3-15·6)
Croatia	94·8 (93·5-96·0)	96·5 (94·7-97·7)	96·8 (95·1-98·0)	98·0 (93·8-99·7)	16·3 (14·5-18·3)	13·7 (12·1-15·2)	13·5 (12·0-15·1)	11·8 (10·5-13·1)	7·7 (6·1-10·4)	7·0 (5·6-9·0)	7·0 (5·5-9·0)	6·7 (5·2-9·4)	6·5 (1·8-17·6)	5·9 (1·8-15·2)	5·9 (1·8-15·0)	6·0 (1·8-13·6)
Czech Republic	97·5 (96·4-98·3)	98·8 (97·4-99·5)	98·9 (97·6-99·5)	99·6 (97·9-100·0)	16·8 (14·9-18·8)	14·5 (12·8-16·2)	14·3 (12·6-16·0)	12·2 (10·7-13·8)	6·7 (5·4-7·7)	6·2 (5·0-7·0)	6·2 (5·0-7·0)	5·9 (4·5-6·8)	3·2 (0·9-8·3)	4·7 (1·2-12·5)	4·7 (1·2-12·8)	6·8 (1·4-19·7)
Hungary	98·8 (98·1-99·3)	98·6 (97·8-99·1)	98·8 (98·1-99·3)	99·4 (98·1-99·9)	19·2 (17·1-21·6)	16·2 (14·3-18·1)	16·0 (14·2-17·9)	13·7 (12·2-15·3)	7·5 (5·9-9·6)	6·6 (5·3-7·5)	6·6 (5·2-7·5)	6·2 (4·9-7·1)	13·8 (5·9-25·4)	9·4 (2·4-25·3)	9·3 (2·4-25·1)	6·8 (1·1-20·7)
Macedonia	92·6 (91·0-94·1)	93·4 (90·5-95·4)	93·2 (90·0-95·5)	95·9 (87·3-99·8)	21·5 (18·8-24·4)	18·3 (16·1-20·7)	18·1 (15·9-20·5)	15·6 (13·7-17·8)	7·0 (4·9-12·0)	6·1 (4·8-9·5)	6·0 (4·8-9·4)	5·4 (4·4-7·7)	14·1 (4·2-32·4)	10·0 (3·5-21·7)	9·9 (3·4-21·9)	6·9 (1·6-19·2)
Montenegro	92·3 (88·2-95·4)	94·7 (92·3-96·3)	94·9 (92·1-96·6)	96·5 (89·1-99·6)	21·8 (19·3-24·7)	18·8 (16·6-21·2)	18·6 (16·5-21·0)	16·4 (14·5-18·6)	11·6 (7·2-18·8)	8·2 (6·3-12·2)	8·1 (6·3-12·0)	6·3 (5·4-9·4)	11·6 (3·5-26·5)	5·6 (1·4-14·4)	5·4 (1·4-14·2)	3·5 (0·8-11·1)
Poland	96·6 (95·4-97·6)	97·2 (96·0-98·2)	97·5 (96·2-98·4)	99·0 (96·7-100·0)	17·7 (15·7-20·0)	14·3 (12·6-16·2)	14·1 (12·4-15·9)	11·2 (9·8-12·9)	7·5 (6·0-8·8)	6·6 (5·2-7·5)	6·5 (5·2-7·5)	5·8 (4·7-6·6)	10·1 (2·3-27·7)	7·0 (1·9-18·6)	6·9 (1·9-18·3)	4·9 (1·4-12·0)
Romania	98·0 (96·9-98·9)	95·3 (92·1-97·2)	95·5 (92·2-97·5)	92·1 (77·6-99·0)	21·0 (18·5-23·6)	16·8 (14·9-18·8)	16·6 (14·7-18·5)	13·3 (11·8-15·0)	14·0 (9·4-19·0)	8·3 (6·2-12·4)	8·1 (6·1-12·2)	6·1 (2·3-12·5)	47·6 (35·3-59·8)	25·0 (6·9-51·8)	24·0 (6·2-51·3)	12·2 (1·2-41·9)
Serbia	91·4 (88·3-93·8)	95·9 (93·6-97·5)	96·0 (93·5-97·7)	98·5 (94·1-100·0)	20·9 (18·5-23·6)	18·0 (15·9-20·3)	17·9 (15·8-20·2)	16·2 (14·3-18·3)	15·6 (10·2-22·2)	9·6 (7·4-13·9)	9·3 (7·3-13·6)	6·6 (5·4-10·1)	14·2 (5·0-30·0)	7·3 (2·7-16·8)	7·2 (2·6-17·0)	3·8 (0·9-13·4)
Slovakia	97·7 (96·5-98·5)	98·4 (97·1-99·2)	98·6 (97·3-99·3)	99·3 (97·1-100·0)	20·1 (17·8-22·7)	17·2 (15·2-19·2)	17·0 (15·0-19·0)	14·1 (12·4-15·9)	7·2 (5·6-9·5)	6·4 (5·1-7·3)	6·4 (5·1-7·2)	5·9 (4·8-6·9)	5·2 (1·3-14·3)	5·3 (1·3-13·9)	5·2 (1·3-13·9)	5·2 (1·1-14·4)
Slovenia	94·4 (92·6-95·7)	96·5 (94·7-97·8)	96·6 (94·6-98·0)	97·8 (93·4-99·7)	15·7 (13·9-17·7)	12·8 (11·4-14·4)	12·7 (11·2-14·2)	10·4 (9·1-11·7)	7·2 (5·5-10·5)	6·3 (5·1-7·3)	6·3 (5·1-7·3)	6·1 (2·6-11·3)	6·2 (1·7-16·0)	5·6 (1·6-13·5)	5·6 (1·6-13·5)	5·4 (1·4-13·3)
**Central Asia**																
Armenia	90·4 (87·4-92·6)	95·6 (93·1-97·4)	96·2 (93·8-97·8)	99·3 (97·7-100·0)	22·9 (20·1-25·8)	19·3 (17·0-22·0)	18·9 (16·7-21·5)	16·2 (14·2-18·4)	11·4 (5·8-26·3)	6·7 (3·2-19·0)	6·5 (3·1-19·0)	4·6 (1·8-18·5)	32·9 (19·1-46·8)	23·0 (9·2-43·5)	22·7 (8·7-44·2)	18·2 (3·4-51·1)

Location	Indicator 6.2.1b:<br>Risk-weighted prevalence of populations without access to a handwashing facility, as measured by the summary exposure value (SEV) for unsafe hygiene (%)				Indicator 7.1.2:<br>Risk-weighted prevalence of household air pollution, as measured by the summary exposure value (SEV) for household air pollution (%)				Indicator 8.8.1:<br>Age-standardised all-cause disability-adjusted life year (DALY) rates attributable to occupational risks (per 100,000 population)				Indicator 11.6.2:<br>Population-weighted mean levels of fine particulate matter smaller than 2.5 microns in diameter (PM2.5)			
	2000	2015	2016	2030	2000	2015	2016	2030	2000	2015	2016	2030	2000	2015	2016	2030
Sweden	3·8<br>(3·6-4·0)	3·7<br>(3·5-3·9)	3·7<br>(3·5-3·9)	3·6<br>(3·4-3·7)	0·7<br>(0·5-0·9)	0·5<br>(0·4-0·7)	0·5<br>(0·4-0·6)	0·4<br>(0·3-0·5)	695·2<br>(575·0-825·9)	657·0<br>(535·4-794·3)	654·8<br>(533·0-792·0)	624·0<br>(498·2-761·1)	5·9<br>(5·8-6·1)	5·2<br>(5·1-5·4)	5·2<br>(5·1-5·3)	4·5<br>(4·3-4·8)
Switzerland	3·7<br>(3·5-3·9)	3·6<br>(3·4-3·8)	3·6<br>(3·4-3·8)	3·5<br>(3·3-3·6)	0·7<br>(0·5-0·9)	0·4<br>(0·3-0·6)	0·4<br>(0·3-0·5)	0·2<br>(0·1-0·3)	979·1<br>(810·7-1,172·3)	878·1<br>(712·1-1,064·7)	860·1<br>(694·4-1,048·6)	643·7<br>(483·1-835·8)	11·2<br>(10·8-11·6)	10·6<br>(10·2-11·0)	10·5<br>(10·2-10·9)	9·8<br>(9·0-10·3)
United Kingdom	3·9<br>(3·9-4·0)	3·7<br>(3·7-3·8)	3·7<br>(3·7-3·8)	3·6<br>(3·5-3·6)	0·6<br>(0·5-0·7)	0·2<br>(0·2-0·3)	0·2<br>(0·2-0·3)	0·1<br>(0·1-0·1)	886·2<br>(769·9-1,017·3)	786·7<br>(668·8-912·2)	785·7<br>(667·4-911·5)	769·7<br>(646·6-900·1)	12·7<br>(12·5-12·9)	11·5<br>(11·3-11·8)	11·5<br>(11·3-11·7)	10·0<br>(9·7-10·2)
**Southern Latin America**																
Argentina	12·3<br>(11·7-13·0)	8·5<br>(8·1-9·0)	8·4<br>(7·9-8·9)	6·0<br>(5·0-6·7)	2·4<br>(1·8-3·1)	0·9<br>(0·7-1·3)	0·9<br>(0·6-1·2)	0·4<br>(0·2-0·5)	1,208·4<br>(1,021·3-1,408·4)	1,120·0<br>(920·8-1,354·9)	1,114·3<br>(914·3-1,351·0)	1,037·2<br>(831·3-1,303·3)	15·6<br>(15·0-16·3)	14·1<br>(13·6-14·7)	14·1<br>(13·6-14·7)	13·1<br>(12·4-13·7)
Chile	8·9<br>(8·4-9·4)	7·3<br>(6·9-7·7)	7·2<br>(6·9-7·6)	6·1<br>(5·5-6·6)	5·7<br>(4·3-7·5)	1·8<br>(1·3-2·5)	1·7<br>(1·2-2·3)	0·6<br>(0·4-0·8)	742·9<br>(605·1-891·2)	760·8<br>(621·3-924·5)	757·3<br>(616·8-921·8)	709·9<br>(555·2-883·5)	22·0<br>(20·9-23·2)	21·9<br>(20·9-23·0)	22·0<br>(20·9-23·0)	23·8<br>(22·7-25·1)
Uruguay	8·9<br>(8·5-9·4)	7·6<br>(7·2-8·1)	7·5<br>(7·1-8·0)	6·2<br>(5·9-6·6)	1·4<br>(1·0-1·8)	0·8<br>(0·6-1·1)	0·8<br>(0·5-1·0)	0·3<br>(0·2-0·4)	907·8<br>(765·7-1,051·9)	969·6<br>(818·3-1,153·7)	964·3<br>(813·3-1,148·8)	892·9<br>(753·3-1,075·3)	13·3<br>(12·3-14·4)	11·5<br>(10·7-12·4)	11·5<br>(10·7-12·5)	11·2<br>(10·4-12·3)
**Eastern Europe**																
Belarus	12·2<br>(7·0-19·2)	7·4<br>(4·1-12·3)	7·3<br>(4·1-12·2)	5·0<br>(2·6-9·4)	1·7<br>(0·4-4·7)	0·5<br>(0·1-1·4)	0·4<br>(0·1-1·3)	0·2<br>(0·0-0·7)	1,102·8<br>(958·9-1,271·0)	870·6<br>(711·8-1,037·4)	850·8<br>(694·2-1,018·0)	616·1<br>(480·3-767·5)	20·3<br>(18·3-23·2)	19·7<br>(17·5-22·7)	19·8<br>(17·5-23·1)	16·3<br>(12·8-19·9)
Estonia	8·1<br>(4·8-13·0)	6·5<br>(3·7-10·4)	6·4<br>(3·7-10·3)	5·4<br>(3·0-8·7)	7·2<br>(3·2-13·8)	4·8<br>(0·7-14·3)	4·6<br>(0·6-14·2)	2·6<br>(0·1-11·6)	835·0<br>(704·4-976·2)	651·7<br>(527·1-795·3)	648·2<br>(522·9-792·5)	599·2<br>(467·5-754·3)	8·1<br>(7·5-8·7)	6·0<br>(5·6-6·6)	5·9<br>(5·5-6·4)	4·4<br>(3·5-5·5)
Latvia	8·9<br>(5·0-15·0)	7·0<br>(3·9-11·0)	7·0<br>(3·9-10·9)	5·6<br>(3·0-9·5)	4·8<br>(2·3-9·1)	3·5<br>(0·5-11·8)	3·4<br>(0·4-12·0)	2·3<br>(0·1-13·0)	921·1<br>(759·0-1,090·9)	807·4<br>(650·5-974·7)	803·3<br>(645·5-971·3)	743·6<br>(574·9-930·3)	18·2<br>(17·1-19·5)	14·8<br>(13·9-15·8)	14·6<br>(13·7-15·6)	10·5<br>(8·0-13·2)
Lithuania	8·9<br>(5·3-14·3)	7·0<br>(3·8-11·5)	7·0<br>(3·8-11·4)	5·7<br>(2·9-9·4)	0·6<br>(0·1-2·4)	0·8<br>(0·1-3·2)	0·8<br>(0·1-3·1)	0·5<br>(0·1-2·3)	913·6<br>(769·5-1,076·6)	750·6<br>(619·8-900·3)	744·2<br>(613·4-895·1)	656·8<br>(523·9-816·8)	18·5<br>(17·9-19·2)	16·8<br>(16·2-17·4)	16·7<br>(16·1-17·4)	14·3<br>(11·2-16·6)
Moldova	24·9<br>(16·5-35·4)	16·7<br>(11·7-22·7)	16·1<br>(11·3-22·0)	9·6<br>(6·2-16·8)	19·0<br>(14·3-24·4)	2·4<br>(0·9-5·2)	2·3<br>(0·8-5·3)	1·2<br>(0·1-4·8)	978·4<br>(805·3-1,171·3)	717·4<br>(590·7-863·3)	709·5<br>(582·7-856·2)	598·0<br>(475·3-743·8)	21·9<br>(19·4-25·1)	20·0<br>(17·8-22·7)	19·9<br>(17·7-22·8)	12·6<br>(11·1-14·5)
Russia	10·2<br>(5·6-15·9)	8·0<br>(4·6-12·7)	7·9<br>(4·6-12·6)	6·9<br>(3·9-11·3)	0·3<br>(0·1-0·9)	0·3<br>(0·0-0·9)	0·2<br>(0·0-0·8)	0·0<br>(0·0-0·2)	1,256·7<br>(1,093·8-1,442·5)	957·5<br>(791·3-1,152·4)	941·7<br>(774·7-1,137·8)	742·5<br>(573·7-952·5)	15·3<br>(15·0-15·6)	15·5<br>(15·2-15·9)	15·5<br>(15·2-15·9)	13·3<br>(11·9-14·9)
Ukraine	12·7<br>(7·5-20·3)	11·2<br>(6·9-17·6)	11·1<br>(6·9-17·3)	9·4<br>(6·1-15·4)	3·2<br>(1·7-5·6)	1·7<br>(0·6-3·8)	1·6<br>(0·5-3·9)	0·9<br>(0·1-4·2)	1,085·7<br>(938·9-1,235·6)	838·7<br>(688·7-1,010·3)	826·4<br>(676·0-1,000·1)	666·6<br>(514·5-848·4)	20·0<br>(19·3-21·0)	19·2<br>(18·4-20·2)	19·2<br>(18·4-20·1)	13·2<br>(12·4-13·9)
**Central Europe**																
Albania	11·9<br>(6·7-19·2)	8·3<br>(4·7-13·5)	8·1<br>(4·6-13·2)	6·1<br>(3·4-9·8)	30·9<br>(24·0-38·5)	12·8<br>(8·8-17·6)	12·3<br>(8·4-17·1)	6·8<br>(3·9-11·1)	1,039·3<br>(809·0-1,307·3)	942·4<br>(736·7-1,193·5)	929·1<br>(724·0-1,179·6)	762·3<br>(567·7-1,008·1)	14·3<br>(13·2-15·5)	14·7<br>(13·3-16·6)	14·6<br>(13·1-16·4)	12·7<br>(9·6-15·7)
Bosnia and Herzegovina	5·4<br>(3·2-8·6)	4·4<br>(2·9-6·3)	4·3<br>(2·8-6·3)	3·7<br>(2·2-5·4)	28·1<br>(21·7-35·6)	16·4<br>(11·3-22·0)	15·9<br>(10·9-21·5)	10·1<br>(6·1-15·5)	720·3<br>(570·4-912·5)	698·1<br>(554·1-866·3)	708·3<br>(561·2-880·6)	854·2<br>(658·7-1,092·1)	38·0<br>(36·1-40·3)	39·5<br>(37·6-42·0)	39·3<br>(37·4-41·9)	38·1<br>(31·8-44·3)
Bulgaria	5·9<br>(3·4-9·6)	4·8<br>(2·7-7·6)	4·7<br>(2·6-7·5)	3·9<br>(2·2-6·2)	6·1<br>(3·7-9·5)	3·9<br>(2·2-6·0)	3·7<br>(2·1-5·8)	2·3<br>(1·2-3·7)	818·6<br>(685·6-957·8)	856·4<br>(708·6-1,022·9)	851·0<br>(702·0-1,019·9)	779·1<br>(618·2-974·0)	29·2<br>(28·5-29·8)	25·7<br>(25·0-26·4)	25·6<br>(25·0-26·3)	16·1<br>(15·0-16·7)
Croatia	5·3<br>(3·0-8·8)	4·5<br>(2·6-7·3)	4·5<br>(2·6-7·3)	3·9<br>(2·3-6·2)	6·4<br>(4·3-9·1)	3·5<br>(2·1-5·7)	3·4<br>(2·0-5·5)	2·0<br>(1·0-3·7)	831·7<br>(692·7-987·6)	821·1<br>(681·0-970·9)	807·2<br>(667·3-956·5)	635·7<br>(503·4-784·1)	18·1<br>(17·4-18·8)	19·8<br>(19·0-20·7)	19·8<br>(19·0-20·7)	18·4<br>(16·2-19·5)
Czech Republic	4·3<br>(2·4-7·1)	3·8<br>(2·2-6·4)	3·8<br>(2·1-6·4)	3·5<br>(1·9-5·8)	1·1<br>(0·6-1·8)	0·7<br>(0·4-1·1)	0·6<br>(0·4-1·1)	0·4<br>(0·2-0·7)	912·9<br>(759·3-1,094·5)	883·8<br>(709·8-1,090·7)	885·8<br>(709·8-1,097·5)	905·5<br>(692·0-1,178·0)	21·0<br>(20·6-21·4)	19·3<br>(18·8-19·7)	19·2<br>(18·8-19·6)	14·7<br>(12·7-16·2)
Hungary	4·7<br>(2·6-7·6)	4·1<br>(2·3-6·6)	4·1<br>(2·3-6·6)	3·7<br>(1·9-6·2)	6·6<br>(3·8-10·5)	4·3<br>(2·4-6·7)	4·1<br>(2·3-6·5)	2·7<br>(1·4-4·3)	870·0<br>(735·2-1,013·1)	829·7<br>(692·8-980·5)	838·7<br>(698·0-993·6)	964·9<br>(765·6-1,179·0)	24·3<br>(23·6-25·0)	24·4<br>(23·7-25·2)	24·6<br>(23·8-25·4)	18·2<br>(16·8-19·0)
Macedonia	5·6<br>(3·1-9·2)	4·9<br>(2·8-8·0)	4·9<br>(2·8-7·9)	4·4<br>(2·5-6·9)	20·0<br>(15·0-26·0)	11·2<br>(8·0-14·9)	10·7<br>(7·5-14·3)	5·5<br>(3·3-7·8)	666·8<br>(538·5-814·7)	659·1<br>(541·3-797·8)	662·4<br>(542·8-802·8)	708·6<br>(565·7-876·4)	35·6<br>(34·0-37·3)	32·3<br>(30·7-34·0)	31·9<br>(30·4-33·6)	20·9<br>(19·0-22·6)
Montenegro	7·6<br>(4·2-12·3)	5·7<br>(3·2-9·0)	5·6<br>(3·1-8·9)	4·4<br>(2·4-6·8)	14·9<br>(10·9-19·8)	14·8<br>(10·8-19·5)	14·7<br>(10·7-19·4)	13·0<br>(8·8-18·0)	684·1<br>(544·7-845·0)	648·6<br>(522·4-787·9)	652·3<br>(524·1-793·9)	702·6<br>(545·2-888·5)	18·5<br>(17·6-19·5)	20·5<br>(19·4-21·7)	20·4<br>(19·3-21·5)	16·9<br>(15·9-17·9)
Poland	5·4<br>(3·0-8·7)	4·5<br>(2·5-7·4)	4·4<br>(2·5-7·3)	3·8<br>(2·1-6·4)	4·0<br>(2·4-6·3)	2·2<br>(1·2-3·4)	2·1<br>(1·2-3·2)	1·1<br>(0·6-1·7)	922·6<br>(774·4-1,082·0)	813·1<br>(669·6-967·5)	805·8<br>(661·3-960·8)	708·4<br>(558·3-875·1)	26·3<br>(26·0-26·7)	25·6<br>(25·2-25·9)	25·6<br>(25·3-26·0)	22·6<br>(20·8-23·3)
Romania	9·2<br>(5·5-14·8)	5·9<br>(3·2-9·8)	5·8<br>(3·2-9·6)	3·7<br>(2·0-6·5)	12·7<br>(9·6-16·6)	5·7<br>(3·6-8·8)	5·5<br>(3·3-8·4)	2·6<br>(1·3-4·6)	1,349·8<br>(1,124·4-1,600·4)	990·7<br>(802·4-1,191·3)	981·4<br>(792·5-1,183·8)	858·4<br>(661·0-1,079·3)	21·2<br>(20·7-21·7)	19·0<br>(18·6-19·4)	19·1<br>(18·6-19·5)	12·5<br>(11·8-12·9)
Serbia	5·9<br>(3·6-8·9)	3·8<br>(2·5-5·8)	3·8<br>(2·4-5·7)	2·7<br>(1·7-4·6)	21·5<br>(16·2-27·3)	12·2<br>(9·1-15·8)	12·2<br>(9·1-15·7)	11·4<br>(8·2-15·2)	930·5<br>(733·7-1,142·1)	844·9<br>(688·5-1,019·9)	841·7<br>(684·8-1,017·7)	798·4<br>(640·0-983·9)	19·1<br>(18·5-19·7)	18·9<br>(18·3-19·6)	18·8<br>(18·2-19·5)	16·8<br>(13·7-19·0)
Slovakia	4·5<br>(2·4-7·3)	3·9<br>(2·2-6·4)	3·8<br>(2·2-6·4)	3·4<br>(2·0-5·7)	1·6<br>(0·9-2·4)	1·0<br>(0·6-1·7)	1·0<br>(0·5-1·7)	0·7<br>(0·3-1·1)	780·7<br>(659·1-909·5)	716·5<br>(592·2-857·5)	713·6<br>(588·8-856·1)	670·9<br>(538·8-837·8)	21·7<br>(21·1-22·3)	20·3<br>(19·8-20·9)	20·3<br>(19·8-20·9)	16·4<br>(14·6-17·3)
Slovenia	4·0<br>(2·2-6·6)	3·6<br>(2·0-5·9)	3·6<br>(2·0-5·9)	3·4<br>(1·8-5·5)	4·3<br>(2·8-6·3)	2·0<br>(1·1-3·3)	1·9<br>(1·1-3·2)	1·0<br>(0·5-1·9)	963·7<br>(804·4-1,144·2)	835·4<br>(672·6-1,010·6)	829·8<br>(664·8-1,008·1)	752·0<br>(567·7-956·0)	16·8<br>(16·0-17·5)	17·9<br>(17·1-18·8)	17·8<br>(17·1-18·6)	15·6<br>(13·4-16·7)
**Central Asia**																
Armenia	16·8<br>(12·4-21·9)	13·5<br>(9·2-19·0)	13·3<br>(8·9-19·0)	11·1<br>(6·7-17·8)	13·6<br>(10·3-17·5)	6·6<br>(1·7-15·8)	6·4<br>(1·4-16·2)	3·7<br>(0·1-17·5)	1,031·6<br>(870·6-1,209·0)	1,006·6<br>(841·5-1,194·6)	993·5<br>(828·7-1,182·0)	827·3<br>(671·9-1,007·1)	18·6<br>(14·4-25·0)	27·1<br>(21·0-37·3)	27·0<br>(20·8-37·5)	33·8<br>(24·4-48·9)

Location	Indicator 16.1.1:<br>Age-standardised death rate due to interpersonal violence (per 100,000 population)				Indicator 16.1.2:<br>Death rate due to conflict and terrorism (per 100,000 population)				Indicator 16.1.3:<br>Age-standardised prevalence of physical or sexual violence experienced by populations in the last 12 months (%)				Indicator 16.2.3:<br>Age-standardised prevalence of women and men aged 18-29 years who experienced sexual violence by age 18 (%)			
	**2000**	**2015**	**2016**	**2030**	**2000**	**2015**	**2016**	**2030**	**2000**	**2015**	**2016**	**2030**	**2000**	**2015**	**2016**	**2030**
Sweden	1·3<br>(0·8-1·5)	1·1<br>(0·6-1·3)	1·0<br>(0·6-1·3)	0·9<br>(0·5-1·3)	0·0<br>(0·0-0·0)	0·0<br>(0·0-0·0)	0·0<br>(0·0-0·0)	0·0<br>(0·0-0·0)	7·4<br>(6·6-8·4)	7·5<br>(6·7-8·5)	7·5<br>(6·7-8·5)	7·4<br>(6·5-8·3)	11·4<br>(8·6-15·0)	13·0<br>(10·0-16·9)	13·0<br>(10·0-16·9)	13·3<br>(10·2-17·4)
Switzerland	1·2<br>(0·8-1·6)	0·7<br>(0·5-1·1)	0·7<br>(0·5-1·1)	0·5<br>(0·2-0·9)	0·0<br>(0·0-0·0)	0·0<br>(0·0-0·0)	0·0<br>(0·0-0·0)	0·0<br>(0·0-0·0)	5·2<br>(4·4-6·3)	4·7<br>(4·0-5·7)	4·7<br>(4·0-5·7)	4·4<br>(3·7-5·3)	11·1<br>(9·0-13·7)	11·1<br>(8·6-14·2)	11·3<br>(8·8-14·5)	11·3<br>(8·8-14·6)
United Kingdom	0·8<br>(0·5-1·0)	0·6<br>(0·4-0·8)	0·6<br>(0·4-0·8)	0·4<br>(0·3-0·6)	0·0<br>(0·0-0·0)	0·0<br>(0·0-0·0)	0·0<br>(0·0-0·0)	0·0<br>(0·0-0·0)	5·3<br>(4·7-6·0)	5·2<br>(4·6-5·8)	5·2<br>(4·6-5·8)	5·1<br>(4·5-5·8)	13·4<br>(10·5-17·1)	12·4<br>(9·6-16·0)	12·3<br>(9·5-15·7)	12·0<br>(9·2-15·4)
**Southern Latin America**																
Argentina	6·4<br>(4·2-8·9)	6·1<br>(3·9-8·2)	5·9<br>(3·8-8·0)	6·5<br>(3·6-10·0)	0·0<br>(0·0-0·0)	0·0<br>(0·0-0·0)	0·0<br>(0·0-0·0)	0·0<br>(0·0-0·0)	11·3<br>(9·8-13·0)	10·2<br>(8·8-11·9)	10·1<br>(8·8-11·8)	9·0<br>(7·8-10·5)	10·1<br>(7·7-13·1)	10·1<br>(7·7-13·2)	10·1<br>(7·7-13·2)	10·1<br>(7·6-13·3)
Chile	5·0<br>(3·3-7·6)	4·2<br>(2·6-6·1)	4·1<br>(2·5-6·6)	3·6<br>(1·6-7·2)	0·0<br>(0·0-0·0)	0·0<br>(0·0-0·0)	0·0<br>(0·0-0·0)	0·0<br>(0·0-0·0)	8·3<br>(6·8-10·1)	7·3<br>(6·0-9·0)	7·2<br>(5·9-8·9)	6·3<br>(5·2-7·9)	10·0<br>(7·6-13·0)	10·1<br>(7·6-13·1)	10·1<br>(7·6-13·1)	10·1<br>(7·7-13·1)
Uruguay	4·7<br>(2·9-6·3)	5·2<br>(2·8-6·7)	5·1<br>(2·7-6·6)	5·9<br>(2·7-9·3)	0·0<br>(0·0-0·0)	0·0<br>(0·0-0·0)	0·0<br>(0·0-0·0)	0·0<br>(0·0-0·0)	6·5<br>(5·6-7·6)	6·1<br>(5·2-7·1)	6·0<br>(5·2-7·0)	5·4<br>(4·6-6·3)	10·0<br>(7·8-12·8)	10·1<br>(7·8-13·0)	10·1<br>(7·8-13·0)	10·1<br>(7·7-13·1)
**Eastern Europe**																
Belarus	11·6<br>(6·9-14·1)	6·2<br>(4·5-9·8)	6·3<br>(4·3-9·9)	3·9<br>(2·1-8·2)	0·0<br>(0·0-0·0)	0·0<br>(0·0-0·0)	0·0<br>(0·0-0·0)	0·0<br>(0·0-0·0)	6·2<br>(5·5-6·9)	5·2<br>(4·6-5·8)	5·1<br>(4·6-5·7)	4·4<br>(3·9-4·9)	13·1<br>(10·0-16·7)	13·0<br>(9·9-16·7)	13·0<br>(9·9-16·8)	12·9<br>(9·9-16·7)
Estonia	12·8<br>(7·5-15·4)	4·7<br>(3·4-8·1)	4·7<br>(3·3-8·3)	2·6<br>(1·1-7·1)	0·0<br>(0·0-0·0)	0·0<br>(0·0-0·0)	0·0<br>(0·0-0·0)	0·0<br>(0·0-0·0)	5·2<br>(4·6-5·8)	4·1<br>(3·6-4·6)	4·0<br>(3·6-4·5)	3·5<br>(3·2-4·0)	13·0<br>(10·0-16·6)	13·0<br>(9·9-16·6)	13·0<br>(9·9-16·7)	12·9<br>(9·9-16·7)
Latvia	11·2<br>(6·6-13·6)	6·3<br>(4·4-9·4)	6·3<br>(4·3-9·3)	3·5<br>(1·9-6·9)	0·0<br>(0·0-0·0)	0·0<br>(0·0-0·0)	0·0<br>(0·0-0·0)	0·0<br>(0·0-0·0)	6·2<br>(5·5-6·9)	5·0<br>(4·5-5·6)	5·0<br>(4·5-5·6)	4·5<br>(4·1-5·1)	13·0<br>(10·0-16·7)	13·0<br>(9·9-16·7)	13·0<br>(9·9-16·7)	13·0<br>(9·9-16·8)
Lithuania	9·2<br>(5·9-12·2)	6·2<br>(4·6-10·7)	6·2<br>(4·6-10·6)	4·6<br>(2·7-9·5)	0·0<br>(0·0-0·0)	0·0<br>(0·0-0·0)	0·0<br>(0·0-0·0)	0·0<br>(0·0-0·0)	5·2<br>(4·6-5·8)	4·4<br>(3·9-4·9)	4·3<br>(3·9-4·9)	3·8<br>(3·3-4·2)	13·1<br>(10·0-16·8)	13·0<br>(9·9-16·6)	13·0<br>(9·9-16·6)	12·9<br>(9·9-16·6)
Moldova	11·0<br>(6·8-14·0)	5·8<br>(4·3-9·9)	5·7<br>(4·0-9·9)	3·5<br>(1·5-8·4)	0·0<br>(0·0-0·0)	0·0<br>(0·0-0·0)	0·0<br>(0·0-0·0)	0·0<br>(0·0-0·0)	7·6<br>(6·9-8·4)	6·4<br>(5·8-7·1)	6·4<br>(5·7-7·1)	5·5<br>(4·8-6·3)	13·1<br>(10·0-16·6)	13·0<br>(10·0-16·8)	13·0<br>(10·0-16·8)	13·0<br>(9·9-16·8)
Russia	29·1<br>(15·6-36·8)	14·6<br>(8·8-22·1)	14·7<br>(8·4-23·5)	9·4<br>(3·3-22·5)	3·0<br>(1·2-4·9)	0·0<br>(0·0-0·0)	0·0<br>(0·0-0·0)	0·0<br>(0·0-0·0)	6·9<br>(6·1-7·7)	5·8<br>(5·1-6·5)	5·7<br>(5·1-6·4)	5·2<br>(4·6-5·8)	13·1<br>(10·1-16·8)	13·1<br>(10·0-16·9)	13·1<br>(10·0-16·9)	13·1<br>(9·8-16·9)
Ukraine	13·7<br>(8·2-16·8)	7·1<br>(4·7-11·4)	7·3<br>(4·5-11·7)	5·4<br>(2·2-12·4)	0·0<br>(0·0-0·0)	2·9<br>(2·7-3·0)	0·0<br>(0·0-0·0)	0·0<br>(0·0-0·0)	6·6<br>(6·0-7·4)	5·7<br>(5·1-6·4)	5·7<br>(5·1-6·4)	5·8<br>(5·1-6·6)	13·1<br>(10·0-16·7)	13·0<br>(9·9-16·8)	13·0<br>(9·9-16·8)	13·0<br>(9·9-16·9)
**Central Europe**																
Albania	8·6<br>(3·0-10·4)	2·3<br>(1·8-3·4)	2·3<br>(1·8-3·3)	2·1<br>(1·2-3·5)	0·0<br>(0·0-0·0)	0·0<br>(0·0-0·0)	0·0<br>(0·0-0·0)	0·0<br>(0·0-0·0)	5·6<br>(5·0-6·4)	4·6<br>(4·0-5·2)	4·5<br>(4·0-5·2)	4·2<br>(3·7-4·8)	6·6<br>(4·9-8·6)	6·8<br>(5·1-8·8)	6·8<br>(5·1-8·8)	6·9<br>(5·1-8·9)
Bosnia and Herzegovina	3·3<br>(2·2-4·3)	2·3<br>(1·7-2·8)	2·3<br>(1·7-2·9)	1·8<br>(1·1-2·8)	0·0<br>(0·0-0·0)	0·0<br>(0·0-0·0)	0·0<br>(0·0-0·0)	0·0<br>(0·0-0·0)	5·1<br>(4·4-5·9)	5·3<br>(4·6-6·1)	5·2<br>(4·6-6·0)	4·6<br>(4·1-5·3)	7·5<br>(5·6-9·6)	7·6<br>(5·6-9·7)	7·5<br>(5·6-9·7)	7·6<br>(5·5-9·7)
Bulgaria	3·6<br>(2·2-4·5)	2·3<br>(1·7-3·7)	2·2<br>(1·6-3·7)	1·6<br>(0·8-3·4)	0·0<br>(0·0-0·0)	0·0<br>(0·0-0·0)	0·0<br>(0·0-0·0)	0·0<br>(0·0-0·0)	6·3<br>(5·7-7·0)	5·3<br>(4·7-5·9)	5·3<br>(4·7-5·8)	4·7<br>(4·2-5·2)	7·5<br>(5·6-9·6)	7·5<br>(5·6-9·6)	7·5<br>(5·6-9·6)	7·5<br>(5·6-9·6)
Croatia	2·3<br>(1·5-2·9)	1·3<br>(1·0-2·0)	1·3<br>(1·0-2·0)	0·7<br>(0·4-1·5)	0·1<br>(0·03-0·2)	0·0<br>(0·0-0·0)	0·0<br>(0·0-0·0)	0·0<br>(0·0-0·0)	4·9<br>(4·4-5·5)	4·2<br>(3·7-4·7)	4·1<br>(3·7-4·6)	3·8<br>(3·4-4·3)	7·5<br>(5·6-9·6)	7·5<br>(5·6-9·6)	7·5<br>(5·6-9·6)	7·5<br>(5·6-9·7)
Czech Republic	1·8<br>(1·2-2·4)	1·1<br>(0·8-1·7)	1·1<br>(0·8-1·7)	0·7<br>(0·5-1·2)	0·0<br>(0·0-0·0)	0·0<br>(0·0-0·0)	0·0<br>(0·0-0·0)	0·0<br>(0·0-0·0)	5·4<br>(4·9-6·1)	4·8<br>(4·3-5·4)	4·8<br>(4·3-5·4)	4·3<br>(3·9-4·9)	8·0<br>(6·7-9·3)	7·9<br>(6·3-9·7)	8·0<br>(6·2-9·8)	7·9<br>(6·2-9·9)
Hungary	2·7<br>(2·0-4·0)	1·6<br>(1·3-2·7)	1·6<br>(1·2-2·7)	1·0<br>(0·7-2·0)	0·0<br>(0·0-0·0)	0·0<br>(0·0-0·0)	0·0<br>(0·0-0·0)	0·0<br>(0·0-0·0)	6·6<br>(5·9-7·3)	5·6<br>(5·0-6·2)	5·5<br>(5·0-6·1)	5·0<br>(4·5-5·5)	7·5<br>(5·6-9·6)	7·5<br>(5·6-9·6)	7·5<br>(5·6-9·6)	7·5<br>(5·6-9·6)
Macedonia	3·2<br>(1·7-3·7)	1·9<br>(1·4-2·2)	1·9<br>(1·4-2·3)	1·3<br>(0·9-1·9)	0·0<br>(0·0-0·0)	0·0<br>(0·0-0·0)	0·0<br>(0·0-0·0)	0·0<br>(0·0-0·0)	5·7<br>(5·0-6·6)	5·1<br>(4·5-5·9)	5·1<br>(4·5-5·9)	4·7<br>(4·1-5·4)	8·8<br>(6·6-11·3)	8·9<br>(6·9-11·4)	8·9<br>(6·9-11·4)	8·9<br>(6·9-11·4)
Montenegro	5·4<br>(4·3-6·3)	3·9<br>(3·2-4·9)	3·9<br>(3·2-4·8)	3·2<br>(2·2-4·8)	0·0<br>(0·0-0·0)	0·0<br>(0·0-0·0)	0·0<br>(0·0-0·0)	0·0<br>(0·0-0·0)	6·3<br>(5·5-7·3)	5·5<br>(4·8-6·4)	5·5<br>(4·8-6·3)	5·0<br>(4·4-5·8)	7·5<br>(5·6-9·5)	7·5<br>(5·6-9·6)	7·5<br>(5·6-9·6)	7·5<br>(5·6-9·6)
Poland	2·7<br>(1·7-3·5)	1·7<br>(1·1-2·8)	1·6<br>(1·1-2·7)	1·1<br>(0·7-2·4)	0·0<br>(0·0-0·0)	0·0<br>(0·0-0·0)	0·0<br>(0·0-0·0)	0·0<br>(0·0-0·0)	4·3<br>(3·9-4·8)	3·6<br>(3·2-4·0)	3·5<br>(3·2-4·0)	3·0<br>(2·7-3·4)	7·7<br>(5·9-9·7)	7·9<br>(6·0-9·9)	7·9<br>(6·0-10·0)	8·0<br>(6·0-10·1)
Romania	3·2<br>(2·1-4·2)	2·0<br>(1·5-3·5)	2·0<br>(1·4-3·5)	1·3<br>(0·7-2·7)	0·0<br>(0·0-0·0)	0·0<br>(0·0-0·0)	0·0<br>(0·0-0·0)	0·0<br>(0·0-0·0)	5·7<br>(5·0-6·3)	4·8<br>(4·2-5·3)	4·7<br>(4·2-5·3)	4·2<br>(3·7-4·6)	7·5<br>(5·6-9·6)	7·6<br>(5·6-9·7)	7·6<br>(5·6-9·7)	7·6<br>(5·6-9·8)
Serbia	3·9<br>(2·5-4·5)	2·6<br>(2·1-3·2)	2·7<br>(2·2-3·2)	1·9<br>(1·4-2·7)	5·4<br>(1·3-9·6)	0·0<br>(0·0-0·0)	0·0<br>(0·0-0·0)	0·0<br>(0·0-0·0)	6·2<br>(5·4-7·2)	5·5<br>(4·8-6·3)	5·4<br>(4·7-6·3)	5·1<br>(4·4-5·9)	7·9<br>(6·0-10·2)	8·2<br>(6·2-10·3)	8·2<br>(6·2-10·3)	8·2<br>(6·2-10·4)
Slovakia	3·2<br>(2·7-3·8)	2·2<br>(1·9-2·9)	2·2<br>(1·8-2·9)	1·7<br>(1·1-2·7)	0·0<br>(0·0-0·0)	0·0<br>(0·0-0·0)	0·0<br>(0·0-0·0)	0·0<br>(0·0-0·0)	6·6<br>(5·9-7·3)	5·8<br>(5·2-6·4)	5·7<br>(5·1-6·4)	5·1<br>(4·6-5·7)	7·5<br>(5·6-9·6)	7·5<br>(5·6-9·6)	7·5<br>(5·6-9·6)	7·5<br>(5·6-9·6)
Slovenia	1·7<br>(1·2-2·5)	0·9<br>(0·7-1·5)	0·9<br>(0·7-1·5)	0·6<br>(0·4-1·1)	0·0<br>(0·0-0·0)	0·0<br>(0·0-0·0)	0·0<br>(0·0-0·0)	0·0<br>(0·0-0·0)	3·7<br>(3·3-4·1)	3·1<br>(2·8-3·5)	3·1<br>(2·8-3·5)	2·9<br>(2·6-3·2)	7·5<br>(5·6-9·6)	7·6<br>(5·6-9·7)	7·5<br>(5·6-9·7)	7·6<br>(5·5-9·8)
**Central Asia**																
Armenia	4·5<br>(2·3-5·7)	3·4<br>(2·1-4·9)	3·5<br>(2·2-4·9)	2·5<br>(1·3-4·5)	0·0<br>(0·0-0·0)	1·7<br>(0·6-2·7)	0·0<br>(0·0-0·0)	0·0<br>(0·0-0·0)	6·0<br>(5·2-6·9)	5·1<br>(4·5-5·9)	5·1<br>(4·4-5·8)	4·6<br>(4·0-5·3)	4·2<br>(3·2-5·3)	4·2<br>(3·2-5·4)	4·3<br>(3·2-5·4)	4·2<br>(3·1-5·3)

Location	Indicator 17.19.2c: Percentage of well-certified deaths by a vital registration (VR) system among a country's total population (%)															
	**2000**	**2015**	**2016**	**2030**												
Sweden	85·7 (83·5-87·6)	84·3 (81·3-86·8)	84·3 (81·1-87·1)	82·7 (76·2-88·4)												
Switzerland	83·9 (80·5-86·9)	86·0 (81·1-90·2)	86·1 (81·1-90·6)	88·8 (79·5-94·5)												
United Kingdom	90·3 (90·1-90·6)	89·8 (89·5-90·2)	89·9 (89·5-90·3)	89·4 (88·7-90·2)												
**Southern Latin America**																
Argentina	66·3 (62·6-69·9)	68·1 (62·1-73·7)	68·7 (61·8-75·3)	68·2 (56·4-77·9)												
Chile	86·3 (83·6-88·6)	89·7 (86·5-92·5)	89·9 (86·6-92·9)	92·6 (86·3-96·5)												
Uruguay	78·2 (75·0-81·2)	76·7 (71·6-81·2)	77·3 (71·6-82·3)	78·3 (67·2-86·7)												
**Eastern Europe**																
Belarus	79·3 (75·8-82·4)	81·9 (76·9-86·0)	82·2 (76·8-86·9)	83·9 (73·8-91·2)												
Estonia	91·0 (88·7-93·0)	92·5 (89·6-94·8)	92·6 (89·3-95·1)	94·2 (88·6-97·6)												
Latvia	89·9 (87·3-92·2)	92·0 (88·6-94·5)	92·0 (88·2-94·7)	94·4 (88·8-97·8)												
Lithuania	92·0 (89·6-93·8)	93·8 (91·4-95·8)	94·0 (91·5-96·0)	95·5 (91·1-98·3)												
Moldova	84·7 (81·8-87·4)	89·7 (86·6-92·5)	89·9 (86·3-93·0)	92·9 (87·5-96·5)												
Russia	84·7 (81·7-87·3)	86·7 (82·7-90·4)	86·9 (82·5-90·8)	88·9 (78·5-95·4)												
Ukraine	82·7 (79·4-85·6)	89·6 (85·8-92·7)	89·6 (85·4-92·9)	92·9 (87·2-96·6)												
**Central Europe**																
Albania	64·9 (60·7-69·2)	57·5 (48·5-66·1)	58·4 (49·2-67·4)	51·2 (36·4-64·8)												
Bosnia and Herzegovina	71·5 (63·0-79·2)	70·2 (63·3-76·0)	70·6 (63·4-76·6)	76·4 (62·0-87·4)												
Bulgaria	71·3 (67·7-74·7)	70·3 (64·0-75·6)	71·0 (64·0-76·8)	66·8 (53·6-77·1)												
Croatia	79·9 (76·8-82·8)	87·0 (83·2-90·2)	87·0 (82·9-90·5)	90·7 (84·2-95·2)												
Czech Republic	83·4 (80·7-86·0)	86·1 (82·6-89·0)	86·3 (82·4-89·6)	88·1 (80·8-93·8)												
Hungary	90·7 (88·7-92·5)	92·8 (90·7-94·7)	93·0 (90·6-94·9)	94·5 (90·8-97·2)												
Macedonia	79·2 (75·7-82·4)	74·7 (68·7-79·7)	75·2 (68·9-80·7)	72·5 (58·8-82·9)												
Montenegro	67·2 (62·5-71·6)	74·4 (66·8-81·2)	74·7 (67·0-81·6)	79·9 (66·4-90·2)												
Poland	71·9 (68·2-75·7)	71·9 (66·9-76·6)	72·6 (67·0-77·8)	74·0 (62·0-83·9)												
Romania	84·0 (81·6-86·4)	84·6 (81·0-87·6)	85·1 (81·1-88·2)	86·2 (79·1-91·6)												
Serbia	73·2 (69·6-76·6)	75·4 (71·1-79·1)	75·7 (70·9-80·0)	77·8 (68·1-85·8)												
Slovakia	83·1 (80·0-85·6)	90·5 (87·5-93·1)	90·6 (87·3-93·3)	92·5 (87·9-95·8)												
Slovenia	86·4 (84·0-88·8)	85·6 (82·4-88·4)	86·0 (82·5-89·1)	85·5 (77·7-91·6)												
**Central Asia**																
Armenia	82·8 (79·0-86·1)	91·4 (87·8-94·4)	91·5 (87·3-94·8)	94·9 (88·9-98·1)												

Location	Indicator 1.5.1: Death rate due to exposure to forces of nature (per 100,000 population)				Indicator 2.2.1: Prevalence of stunting in children under 5 (%)				Indicator 2.2.2a: Prevalence of wasting in children under 5 (%)				Indicator 2.2.2b: Prevalence of overweight in children aged 2-4 (%)			
	**2000**	**2015**	**2016**	**2030**	**2000**	**2015**	**2016**	**2030**	**2000**	**2015**	**2016**	**2030**	**2000**	**2015**	**2016**	**2030**
Azerbaijan	0·1 (0·06-0·2)	0·0 (0·0-0·0)	0·0 (0·0-0·0)	0·03 (0·01-0·04)	23·5 (21·3-25·9)	14·6 (9·6-20·5)	14·0 (8·9-20·1)	7·4 (2·5-16·3)	5·1 (4·5-5·8)	5·4 (4·2-6·9)	5·4 (4·1-7·0)	5·2 (4·0-8·0)	15·9 (12·6-19·7)	35·4 (29·5-41·4)	35·9 (30·0-42·1)	51·5 (38·2-65·2)
Georgia	0·0 (0·0-0·0)	0·2 (0·09-0·3)	0·2 (0·08-0·3)	0·1 (0·06-0·2)	13·5 (11·6-15·5)	10·6 (6·7-15·5)	10·5 (6·4-15·8)	9·1 (3·1-19·7)	3·0 (2·6-3·5)	2·6 (1·9-3·4)	2·5 (1·8-3·4)	2·3 (1·4-3·5)	24·6 (19·9-29·7)	32·5 (27·2-38·5)	33·1 (27·9-39·4)	39·2 (28·0-52·0)
Kazakhstan	0·06 (0·03-0·1)	0·3 (0·1-0·4)	0·2 (0·1-0·3)	0·3 (0·1-0·4)	15·1 (11·5-19·1)	9·1 (6·6-12·0)	8·4 (6·0-11·3)	4·4 (2·9-6·4)	4·2 (3·4-5·3)	4·3 (3·4-5·2)	4·2 (3·3-5·2)	3·5 (2·7-4·5)	18·2 (14·9-21·9)	26·8 (21·5-32·2)	27·3 (21·9-32·8)	34·8 (23·7-47·5)
Kyrgyzstan	0·4 (0·2-0·6)	0·4 (0·2-0·5)	0·3 (0·2-0·4)	0·4 (0·2-0·6)	24·6 (17·9-31·0)	14·2 (10·0-18·8)	13·4 (9·0-18·3)	5·8 (3·7-11·1)	3·0 (2·3-3·8)	2·8 (2·2-3·5)	2·9 (2·2-3·6)	2·5 (2·1-3·0)	20·6 (17·0-24·8)	17·0 (14·4-20·2)	17·4 (14·5-20·7)	14·6 (9·7-20·7)
Mongolia	0·3 (0·2-0·5)	0·2 (0·1-0·4)	0·2 (0·09-0·3)	0·3 (0·1-0·4)	34·3 (30·9-38·7)	14·8 (10·2-20·0)	14·4 (9·5-19·9)	8·2 (3·8-10·8)	6·1 (5·2-7·0)	2·7 (1·9-3·5)	2·6 (1·8-3·5)	1·8 (0·9-2·8)	21·7 (17·3-26·4)	23·4 (19·4-28·4)	24·1 (19·9-29·6)	26·0 (17·9-36·6)
Tajikistan	1·4 (0·6-2·2)	0·2 (0·08-0·3)	0·2 (0·06-0·2)	0·2 (0·1-0·4)	40·7 (35·8-46·4)	29·0 (21·6-36·9)	28·8 (20·8-37·1)	24·2 (11·9-33·4)	10·8 (9·7-11·8)	8·8 (6·9-10·8)	8·8 (6·8-10·9)	8·8 (5·7-11·6)	15·3 (12·1-18·9)	17·8 (14·2-21·7)	18·1 (14·5-22·3)	20·0 (12·9-29·3)
Turkmenistan	0·04 (0·02-0·06)	0·2 (0·1-0·3)	0·2 (0·1-0·3)	0·2 (0·1-0·3)	25·7 (22·3-29·9)	11·7 (8·6-15·0)	11·2 (8·4-14·0)	5·7 (5·0-6·5)	7·2 (6·7-7·8)	4·7 (3·9-5·6)	4·5 (3·7-5·4)	2·7 (1·9-3·4)	20·4 (16·0-25·2)	30·7 (25·0-37·0)	31·4 (25·6-38·0)	41·7 (30·4-53·9)
Uzbekistan	0·0 (0·0-0·0)	0·0 (0·0-0·0)	0·0 (0·0-0·0)	0·0 (0·0-0·0)	31·7 (26·1-37·7)	16·4 (10·5-23·8)	16·0 (10·2-23·4)	8·6 (3·4-17·9)	8·6 (6·9-10·5)	5·1 (3·6-7·2)	5·1 (3·5-7·2)	3·4 (1·9-5·5)	27·5 (22·8-32·6)	31·3 (25·6-37·3)	32·1 (26·1-38·2)	40·1 (27·3-55·0)
**Central Latin America**																
Colombia	0·7 (0·4-1·0)	0·3 (0·2-0·5)	0·3 (0·1-0·4)	0·3 (0·2-0·5)	18·4 (16·3-20·5)	11·2 (8·4-14·4)	11·0 (8·0-14·2)	7·8 (4·4-11·6)	1·8 (1·3-2·3)	1·4 (0·9-2·0)	1·4 (0·9-2·0)	1·1 (0·6-1·7)	11·9 (9·3-15·2)	12·1 (7·9-17·0)	12·5 (8·0-18·0)	12·4 (5·7-22·6)
Costa Rica	0·3 (0·2-0·5)	0·2 (0·09-0·2)	0·2 (0·09-0·2)	0·2 (0·09-0·2)	8·0 (5·9-10·8)	6·3 (4·6-8·5)	6·2 (4·5-8·4)	5·0 (3·6-6·5)	2·9 (2·0-4·0)	2·5 (1·7-3·5)	2·5 (1·7-3·5)	2·3 (1·5-3·2)	28·0 (19·3-38·6)	37·1 (26·7-48·9)	37·5 (27·0-49·6)	47·0 (27·1-68·1)
El Salvador	1·3 (0·7-2·0)	0·5 (0·3-0·8)	0·4 (0·2-0·6)	1·0 (0·5-1·5)	32·1 (28·0-36·4)	14·7 (11·2-18·5)	14·0 (10·6-17·7)	6·6 (4·4-8·7)	2·0 (1·4-2·6)	1·9 (1·3-2·6)	2·0 (1·4-2·7)	2·3 (1·6-3·0)	12·2 (9·3-15·7)	16·2 (10·6-23·2)	16·6 (10·8-24·0)	19·7 (9·2-34·9)
Guatemala	0·7 (0·4-1·0)	0·2 (0·09-0·2)	0·1 (0·07-0·2)	0·3 (0·2-0·5)	53·2 (48·0-58·8)	45·1 (39·0-53·1)	44·8 (38·8-52·8)	41·0 (34·9-50·0)	3·6 (3·0-4·2)	1·3 (0·8-2·0)	1·3 (0·8-1·9)	0·7 (0·3-1·3)	18·1 (15·5-20·9)	20·4 (13·7-28·6)	21·1 (14·0-29·6)	31·6 (14·4-53·1)
Honduras	37·1 (21·4-52·8)	0·3 (0·1-0·4)	0·2 (0·1-0·3)	3·2 (1·8-4·5)	40·8 (37·4-44·2)	23·0 (17·8-27·5)	22·6 (17·3-27·2)	11·5 (9·2-14·2)	1·9 (1·5-2·3)	1·6 (1·1-2·2)	1·6 (1·1-2·2)	1·4 (0·8-2·2)	10·1 (7·7-13·2)	14·2 (10·2-19·0)	14·6 (10·1-19·9)	21·1 (10·2-34·3)
Mexico	0·4 (0·2-0·5)	0·1 (0·07-0·2)	0·1 (0·06-0·1)	0·1 (0·08-0·2)	18·0 (17·2-18·8)	12·9 (12·2-13·7)	12·4 (11·8-13·2)	8·8 (8·0-9·6)	3·0 (2·8-3·2)	2·3 (2·2-2·5)	2·3 (2·1-2·5)	1·6 (1·4-1·8)	26·0 (17·7-35·5)	23·4 (16·9-31·4)	24·0 (17·0-32·3)	26·0 (12·5-45·2)
Nicaragua	10·5 (5·8-15·1)	0·4 (0·2-0·6)	0·3 (0·2-0·4)	1·2 (0·7-1·8)	25·7 (23·0-28·6)	16·7 (13·1-20·8)	16·3 (12·8-20·5)	10·4 (7·9-14·7)	2·7 (2·2-3·2)	2·0 (1·4-2·7)	2·0 (1·4-2·7)	1·8 (1·1-2·6)	16·7 (14·1-19·6)	20·7 (13·6-29·8)	21·3 (14·1-30·8)	31·5 (15·2-55·1)
Panama	0·3 (0·1-0·4)	0·3 (0·2-0·5)	0·3 (0·1-0·4)	0·3 (0·2-0·5)	25·3 (21·5-29·2)	16·3 (12·5-20·4)	15·8 (12·0-20·1)	10·4 (6·2-16·0)	1·8 (1·2-2·5)	1·6 (0·9-2·4)	1·6 (0·9-2·4)	1·3 (0·7-2·1)	11·8 (7·4-17·8)	17·2 (10·9-24·1)	18·0 (11·5-25·1)	24·0 (10·6-41·5)
Venezuela	20·9 (11·7-30·0)	0·01 (0·01-0·02)	0·01 (0·0-0·01)	1·9 (1·0-2·7)	16·4 (13·8-19·0)	11·0 (8·1-14·4)	10·7 (7·8-14·1)	7·8 (5·2-11·2)	4·0 (3·3-4·7)	5·0 (3·5-6·8)	5·0 (3·5-6·9)	5·5 (3·5-8·7)	26·7 (18·1-36·7)	37·1 (26·4-47·9)	37·6 (26·5-48·9)	47·1 (26·3-68·2)
**Andean Latin America**																
Bolivia	0·5 (0·08-1·0)	0·6 (0·09-1·1)	0·5 (0·07-0·9)	0·5 (0·08-0·9)	34·4 (27·5-42·9)	18·2 (12·3-25·7)	17·5 (11·6-25·2)	9·7 (4·4-17·8)	2·9 (2·1-3·7)	1·9 (1·1-2·8)	1·9 (1·1-2·8)	1·3 (0·6-2·2)	27·0 (24·3-29·9)	28·4 (23·2-34·1)	29·2 (23·9-35·2)	33·9 (22·4-47·0)
Ecuador	0·5 (0·08-0·9)	0·2 (0·03-0·3)	1·7 (0·3-3·2)	1·1 (0·2-2·1)	30·3 (24·7-36·4)	20·8 (14·6-27·7)	20·3 (14·1-27·4)	14·5 (8·0-21·2)	2·7 (2·2-3·3)	2·0 (1·3-2·9)	2·0 (1·3-2·9)	1·7 (0·9-2·8)	17·0 (13·6-20·9)	20·4 (16·7-24·8)	20·9 (17·0-25·5)	25·5 (17·2-35·6)
Peru	0·7 (0·1-1·3)	0·3 (0·06-0·7)	0·3 (0·04-0·5)	0·4 (0·06-0·8)	30·8 (24·8-37·5)	19·3 (14·3-24·8)	18·3 (13·0-24·0)	8·1 (5·7-13·6)	1·8 (1·2-2·6)	1·3 (0·8-2·2)	1·3 (0·7-2·2)	0·8 (0·4-1·7)	26·8 (24·0-29·8)	21·3 (17·9-25·3)	22·0 (18·4-26·4)	23·9 (15·0-35·0)
**Caribbean**																
Antigua and Barbuda	0·0 (0·0-0·0)	0·0 (0·0-0·0)	0·0 (0·0-0·0)	0·0 (0·0-0·0)	7·6 (5·2-10·2)	6·9 (4·8-9·4)	6·9 (4·8-9·3)	6·1 (4·1-8·4)	2·6 (1·9-3·4)	2·3 (1·7-2·9)	2·3 (1·6-2·9)	2·1 (1·5-2·7)	23·3 (15·9-32·8)	30·2 (20·9-41·0)	30·7 (21·7-41·4)	39·1 (20·8-60·0)
The Bahamas	0·3 (0·05-0·6)	1·7 (0·3-3·3)	1·5 (0·2-3·0)	1·0 (0·2-1·9)	6·8 (4·8-9·3)	6·8 (4·8-9·3)	6·8 (4·8-9·3)	6·8 (4·6-9·2)	2·5 (1·9-3·3)	2·3 (1·7-3·0)	2·3 (1·6-3·0)	2·1 (1·5-2·8)	25·5 (17·8-34·6)	23·1 (15·6-32·3)	23·2 (15·6-32·8)	23·2 (11·1-41·1)
Barbados	0·07 (0·01-0·1)	0·0 (0·0-0·0)	0·0 (0·0-0·0)	0·02 (0·0-0·03)	7·6 (5·4-10·4)	7·1 (5·1-9·5)	7·1 (5·0-9·4)	6·6 (4·5-8·8)	5·7 (4·5-7·1)	5·3 (4·3-6·4)	5·3 (4·2-6·4)	4·9 (3·9-5·9)	24·8 (17·1-33·6)	21·7 (15·6-29·2)	21·9 (15·3-29·9)	22·2 (10·2-38·6)
Belize	1·1 (0·2-2·1)	0·01 (0·0-0·01)	0·0 (0·0-0·0)	0·4 (0·06-0·7)	18·2 (13·7-23·6)	14·6 (13·2-15·9)	13·9 (12·7-15·1)	10·2 (9·2-11·2)	2·4 (1·9-3·1)	2·7 (2·3-3·3)	2·7 (2·3-3·2)	2·9 (2·4-3·3)	18·8 (12·9-26·7)	18·7 (12·8-26·1)	18·8 (12·8-26·5)	21·0 (9·8-37·2)
Cuba	0·5 (0·07-0·9)	0·4 (0·06-0·7)	0·3 (0·05-0·6)	0·4 (0·06-0·8)	5·8 (4·5-7·1)	8·1 (6·1-10·5)	8·5 (6·4-10·8)	14·0 (11·6-16·3)	2·5 (2·1-3·0)	2·2 (1·6-3·0)	2·2 (1·6-3·0)	2·3 (1·6-3·2)	17·7 (11·9-25·3)	24·5 (20·3-29·2)	25·0 (20·2-30·3)	30·4 (18·9-44·0)
Dominica	0·0 (0·0-0·0)	8·6 (1·4-16·4)	7·6 (1·2-14·6)	4·9 (0·8-9·4)	9·5 (6·7-12·7)	8·3 (5·9-11·2)	8·3 (5·8-11·1)	7·3 (5·1-9·8)	3·0 (2·3-4·0)	2·6 (1·9-3·3)	2·5 (1·9-3·3)	2·3 (1·7-2·9)	24·5 (16·8-33·9)	28·4 (19·3-38·3)	28·6 (19·6-38·8)	32·9 (16·6-51·7)
Dominican Republic	0·7 (0·1-1·4)	0·06 (0·01-0·1)	0·03 (0·01-0·06)	0·4 (0·06-0·8)	10·2 (8·6-12·0)	8·6 (6·5-11·2)	8·3 (6·1-10·8)	4·6 (2·9-6·8)	2·4 (2·0-2·9)	2·4 (2·0-3·0)	2·4 (1·9-3·0)	2·3 (1·8-3·0)	14·7 (11·6-18·1)	22·9 (16·0-32·0)	23·6 (16·4-32·9)	34·4 (18·8-53·7)
Grenada	0·0 (0·0-0·0)	0·1 (0·02-0·3)	0·1 (0·02-0·2)	0·7 (0·1-1·4)	11·3 (8·0-15·2)	8·7 (6·3-11·8)	8·6 (6·2-11·7)	6·5 (4·8-9·0)	3·1 (2·3-3·9)	2·5 (1·9-3·2)	2·5 (1·9-3·1)	2·2 (1·6-2·8)	21·1 (14·4-30·0)	25·8 (17·7-35·6)	26·2 (17·8-36·4)	32·1 (16·1-53·1)

Location	Indicator 3.1.1: Maternal mortality ratio (maternal deaths per 100,000 livebirths) in women aged 10-54 years				Indicator 3.1.2: Proportion of births attended by skilled health personnel (%)				Indicator 3.2.1: Under-5 mortality rate (probability of dying before the age of 5 per 1,000 livebirths)				Indicator 3.2.2: Neonatal mortality rate (probability of dying during the first 28 days of life per 1,000 livebirths)			
	**2000**	**2015**	**2016**	**2030**	**2000**	**2015**	**2016**	**2030**	**2000**	**2015**	**2016**	**2030**	**2000**	**2015**	**2016**	**2030**
Azerbaijan	49·5 (40·9-58·6)	24·6 (17·5-33·4)	24·7 (17·2-34·3)	18·1 (7·1-38·0)	88·2 (85·7-90·5)	94·1 (80·9-99·1)	94·3 (80·4-99·3)	96·3 (65·5-100·0)	62·9 (53·2-74·8)	31·3 (21·8-43·2)	30·0 (21·4-41·1)	16·5 (6·3-34·6)	29·3 (24·7-35·6)	17·3 (12·6-22·9)	16·7 (12·4-22·0)	10·4 (4·1-21·3)
Georgia	23·3 (19·7-27·2)	34·3 (27·4-43·0)	33·4 (25·0-44·5)	22·8 (8·0-63·0)	97·5 (96·4-98·2)	99·0 (97·9-99·6)	99·0 (97·6-99·7)	99·0 (93·6-100·0)	37·9 (33·3-43·0)	16·1 (12·1-21·2)	15·4 (11·4-20·4)	7·6 (3·9-13·2)	21·0 (18·5-23·7)	10·1 (7·5-13·3)	9·6 (7·1-12·8)	4·8 (2·4-8·3)
Kazakhstan	67·6 (56·5-80·1)	15·3 (12·2-18·8)	16·5 (12·1-22·0)	4·3 (1·9-8·9)	99·2 (98·5-99·6)	99·5 (98·4-99·9)	99·4 (98·1-99·9)	99·3 (94·0-100·0)	36·9 (30·8-43·6)	14·0 (10·5-18·1)	13·5 (9·9-18·0)	5·3 (2·2-10·9)	15·0 (11·9-18·5)	6·9 (5·3-8·8)	6·6 (5·0-8·6)	2·9 (1·1-6·1)
Kyrgyzstan	58·7 (51·5-65·5)	47·0 (40·7-53·8)	46·6 (38·3-55·7)	35·9 (18·9-60·5)	97·9 (95·9-99·1)	99·5 (98·9-99·8)	99·5 (98·8-99·8)	99·7 (97·8-100·0)	49·8 (46·0-53·6)	30·0 (25·6-35·1)	28·9 (24·2-34·4)	18·3 (12·0-26·6)	24·7 (23·3-26·2)	17·2 (14·8-19·9)	16·7 (14·1-19·5)	11·8 (8·1-16·4)
Mongolia	162·0 (135·5-194·5)	49·4 (36·4-64·5)	49·8 (35·7-67·1)	22·6 (9·1-48·8)	98·3 (97·8-98·8)	98·9 (97·7-99·6)	98·9 (97·5-99·6)	98·8 (91·5-100·0)	57·6 (52·2-63·2)	22·2 (17·8-27·4)	21·2 (16·7-26·6)	9·2 (5·2-15·6)	22·3 (19·7-25·3)	11·6 (10·0-13·4)	11·2 (9·5-13·0)	6·2 (3·9-9·1)
Tajikistan	75·5 (58·4-93·7)	36·4 (24·5-50·5)	35·8 (23·7-50·7)	25·1 (8·7-54·8)	81·6 (66·9-91·6)	93·1 (80·5-98·5)	93·2 (78·4-98·7)	92·7 (43·5-100·0)	74·7 (64·4-86·5)	38·8 (28·0-52·2)	37·6 (27·1-50·8)	24·9 (9·9-53·5)	27·6 (23·7-32·4)	17·4 (13·1-22·5)	16·9 (12·7-21·9)	12·6 (5·4-25·5)
Turkmenistan	22·3 (19·5-25·5)	11·5 (9·7-13·3)	11·2 (9·4-13·1)	7·7 (4·5-12·0)	97·6 (96·5-98·4)	99·4 (97·8-99·9)	99·4 (97·8-99·9)	99·5 (94·7-100·0)	80·9 (64·4-100·8)	39·1 (26·7-55·5)	37·0 (25·2-52·6)	17·4 (6·8-37·4)	30·5 (24·4-37·3)	17·9 (12·1-25·4)	17·1 (11·6-24·2)	9·8 (3·7-21·0)
Uzbekistan	52·6 (46·1-59·9)	28·7 (23·4-34·9)	28·3 (22·0-35·5)	22·4 (8·6-50·3)	98·3 (96·8-99·2)	99·7 (98·9-100·0)	99·7 (99·0-100·0)	99·7 (96·9-100·0)	42·6 (35·8-50·7)	24·5 (17·4-33·5)	23·5 (16·7-32·1)	12·9 (5·0-28·8)	18·1 (15·1-21·8)	12·4 (8·8-17·0)	12·0 (8·5-16·4)	7·8 (3·0-17·6)
**Central Latin America**																
Colombia	87·4 (77·7-101·2)	57·3 (48·1-67·4)	55·9 (45·8-67·0)	39·5 (23·9-59·6)	88·0 (86·7-89·3)	97·3 (95·5-98·6)	97·5 (95·7-98·7)	99·2 (96·9-99·9)	26·6 (23·8-29·5)	16·1 (13·3-19·6)	15·5 (12·6-19·0)	8·8 (5·4-13·8)	14·3 (12·5-16·2)	9·4 (7·9-11·1)	9·0 (7·5-10·7)	5·0 (3·2-7·6)
Costa Rica	38·7 (33·7-43·8)	29·9 (25·2-35·7)	29·3 (24·1-35·5)	21·5 (12·3-33·8)	98·1 (96·8-99·1)	99·1 (98·2-99·6)	99·1 (98·3-99·6)	99·6 (98·2-100·0)	15·4 (12·9-18·6)	10·9 (8·5-13·9)	10·6 (8·1-13·7)	7·5 (4·4-12·2)	8·9 (7·4-10·8)	6·8 (5·4-8·5)	6·5 (5·2-8·2)	4·9 (2·9-7·7)
El Salvador	48·2 (39·8-57·2)	38·4 (28·9-49·6)	38·1 (27·9-51·1)	35·9 (15·9-68·4)	99·1 (98·7-99·4)	99·5 (99·0-99·8)	99·5 (99·0-99·8)	99·8 (98·9-100·0)	29·3 (25·7-33·6)	12·6 (10·2-15·6)	11·9 (9·5-14·8)	5·7 (3·1-9·7)	12·5 (10·9-14·3)	5·7 (4·7-7·1)	5·4 (4·3-6·7)	2·6 (1·4-4·4)
Guatemala	111·4 (87·3-139·3)	92·4 (64·8-126·0)	90·4 (57·5-132·5)	67·2 (18·5-174·6)	49·7 (47·2-52·7)	65·7 (51·5-78·1)	67·2 (52·4-79·9)	83·9 (55·9-100·0)	51·1 (47·4-54·9)	28·0 (24·1-32·4)	26·7 (22·9-31·2)	15·1 (11·2-19·8)	16·2 (14·8-17·7)	11·2 (9·2-13·6)	10·8 (8·8-13·2)	7·5 (4·9-11·0)
Honduras	145·7 (101·8-208·6)	103·2 (58·6-161·8)	101·9 (56·9-160·2)	84·0 (25·1-193·4)	68·6 (61·0-75·2)	95·4 (92·3-97·6)	95·4 (91·9-97·7)	98·9 (94·5-100·0)	36·8 (34·0-39·6)	22·3 (19·2-26·1)	21·4 (18·3-25·4)	12·8 (9·7-17·4)	16·8 (15·2-18·5)	12·0 (10·0-14·5)	11·6 (9·6-14·2)	8·5 (6·2-12·2)
Mexico	54·3 (51·9-56·8)	47·1 (44·4-50·3)	46·6 (43·7-50·0)	40·0 (31·5-50·5)	92·5 (90·5-94·2)	98·0 (96·3-99·0)	98·1 (96·5-99·1)	99·0 (96·2-99·9)	27·7 (24·2-31·1)	16·1 (12·8-20·3)	15·5 (12·1-19·7)	9·4 (5·8-14·7)	11·7 (10·2-13·2)	7·6 (6·1-9·5)	7·3 (5·8-9·2)	4·8 (3·0-7·3)
Nicaragua	93·3 (79·4-107·4)	65·4 (48·6-86·2)	63·6 (44·9-87·0)	50·7 (21·0-105·0)	91·8 (89·4-93·7)	96·4 (93·4-98·3)	96·5 (93·7-98·4)	98·1 (93·3-99·8)	36·1 (33·6-38·7)	15·9 (13·5-19·0)	15·1 (12·7-18·2)	7·0 (5·0-9·9)	14·2 (12·8-15·7)	7·8 (6·6-9·3)	7·4 (6·2-8·9)	4·1 (2·9-5·9)
Panama	62·4 (52·8-73·9)	74·7 (59·1-92·9)	71·1 (53·8-90·7)	52·4 (22·4-107·0)	90·7 (88·6-92·4)	93·7 (90·6-95·9)	94·1 (90·9-96·4)	97·0 (91·3-99·8)	23·1 (20·0-27·0)	17·1 (13·7-21·4)	16·6 (13·1-20·9)	12·6 (7·8-19·0)	10·0 (8·6-11·7)	7·3 (5·9-9·2)	7·1 (5·7-8·9)	5·3 (3·3-8·2)
Venezuela	59·3 (51·0-68·8)	69·9 (52·0-91·7)	69·8 (50·0-93·7)	72·1 (31·3-148·6)	97·4 (95·8-98·5)	97·3 (94·8-98·7)	97·3 (94·8-98·8)	97·4 (89·8-100·0)	21·4 (21·2-21·6)	16·9 (16·3-17·5)	16·8 (16·0-17·7)	16·2 (13·1-19·2)	11·3 (10·7-11·8)	10·0 (9·7-10·3)	10·0 (9·6-10·4)	9·8 (8·1-11·6)
**Andean Latin America**																
Bolivia	219·6 (183·3-258·3)	154·4 (102·6-215·2)	150·4 (99·8-216·3)	99·6 (39·3-211·5)	63·7 (61·5-65·9)	82·5 (77·5-86·4)	83·4 (78·4-87·4)	92·9 (86·4-96·8)	72·6 (66·7-78·7)	35·5 (27·8-44·6)	33·8 (26·3-43·2)	16·8 (10·2-26·2)	26·8 (24·1-29·9)	15·9 (12·5-20·0)	15·2 (11·8-19·4)	9·1 (5·5-14·3)
Ecuador	97·3 (87·6-108·5)	60·2 (51·5-70·3)	58·8 (49·7-69·1)	48·4 (30·0-72·7)	96·3 (95·1-97·3)	98·8 (98·2-99·2)	98·8 (98·3-99·2)	99·6 (99·0-99·9)	32·8 (30·1-35·7)	17·6 (14·8-20·8)	17·3 (14·5-20·7)	9·7 (6·9-13·7)	15·5 (14·0-17·1)	8·5 (7·3-9·8)	8·2 (7·0-9·4)	4·6 (3·5-6·0)
Peru	176·7 (148·6-208·7)	76·7 (57·0-98·5)	71·7 (50·4-96·0)	30·0 (11·6-61·4)	68·0 (63·5-71·9)	91·8 (89·5-93·7)	92·4 (90·2-94·4)	97·8 (96·1-98·9)	38·2 (35·8-40·7)	17·3 (14·2-21·1)	16·6 (13·2-20·8)	8·4 (4·7-14·3)	15·4 (13·9-17·0)	8·2 (6·6-10·1)	7·8 (6·2-9·9)	4·3 (2·3-7·2)
**Caribbean**																
Antigua and Barbuda	36·4 (29·9-44·0)	39·6 (30·7-49·6)	37·8 (29·2-48·6)	39·0 (21·7-66·5)	98·9 (97·6-99·5)	99·1 (97·7-99·7)	99·1 (97·7-99·7)	99·2 (95·1-100·0)	16·4 (12·2-22·0)	11·4 (7·7-16·5)	11·2 (7·4-16·5)	8·8 (3·9-17·8)	9·8 (7·1-13·5)	5·8 (3·8-8·6)	5·7 (3·7-8·6)	3·9 (1·6-8·1)
The Bahamas	61·6 (52·1-73·2)	61·0 (49·9-74·3)	61·8 (49·4-76·7)	56·8 (27·3-104·8)	98·8 (97·6-99·5)	98·9 (97·5-99·6)	98·9 (97·5-99·7)	98·8 (93·2-100·0)	14·0 (9·2-19·9)	11·4 (6·4-20·0)	11·0 (5·9-20·3)	8·0 (1·5-25·5)	6·5 (4·2-9·2)	5·4 (3·0-9·5)	5·3 (2·8-9·7)	4·3 (0·7-13·9)
Barbados	50·8 (42·5-60·1)	60·5 (48·7-73·6)	58·7 (46·7-72·1)	35·3 (17·1-64·2)	97·9 (95·8-99·2)	98·8 (97·2-99·6)	98·9 (97·2-99·7)	99·0 (93·9-100·0)	17·8 (10·6-27·6)	14·8 (8·3-24·5)	14·7 (7·9-25·7)	12·7 (2·7-38·8)	11·7 (7·2-17·8)	9·9 (5·6-16·4)	9·9 (5·4-17·2)	8·6 (1·9-26·3)
Belize	57·6 (48·4-70·4)	18·0 (13·7-23·2)	18·2 (13·5-24·2)	11·6 (4·2-24·1)	93·0 (89·9-95·3)	96·6 (93·6-98·2)	96·7 (93·4-98·5)	97·8 (87·8-100·0)	28·1 (21·8-35·3)	15·2 (9·2-23·6)	15·1 (8·8-24·6)	9·7 (2·0-29·0)	14·1 (10·9-17·7)	8·4 (5·0-13·0)	8·3 (4·9-13·5)	5·3 (1·1-15·3)
Cuba	49·6 (43·2-57·1)	43·6 (36·7-51·8)	42·8 (35·2-51·6)	33·0 (18·8-54·6)	98·8 (97·7-99·5)	99·2 (98·5-99·6)	99·2 (98·4-99·7)	99·4 (96·7-100·0)	8·7 (8·5-8·8)	5·4 (5·2-5·7)	5·3 (5·0-5·7)	3·7 (3·2-4·5)	4·2 (4·0-4·4)	2·5 (2·3-2·6)	2·4 (2·2-2·6)	1·5 (1·2-2·0)
Dominica	22·5 (18·5-26·8)	17·3 (13·7-22·0)	18·3 (14·1-24·0)	6·8 (2·5-15·0)	98·3 (96·7-99·3)	98·5 (96·3-99·5)	98·5 (96·4-99·6)	98·5 (90·2-100·0)	18·7 (13·4-25·2)	27·8 (17·6-43·6)	25·0 (15·7-38·5)	31·7 (11·5-72·4)	12·3 (8·9-16·5)	18·9 (12·5-27·7)	18·2 (11·7-27·3)	26·7 (10·3-57·8)
Dominican Republic	59·8 (51·4-68·5)	67·3 (50·3-89·3)	65·9 (48·0-87·9)	61·5 (27·5-117·3)	98·2 (97·4-98·8)	96·7 (94·3-98·2)	96·7 (93·8-98·4)	94·4 (73·7-100·0)	37·4 (33·8-41·5)	28·6 (22·8-35·0)	27·4 (21·7-34·5)	18·1 (9·5-31·8)	21·1 (19·0-23·4)	18·3 (14·5-22·5)	17·6 (14·0-22·1)	13·6 (7·1-23·3)
Grenada	29·9 (23·2-37·9)	28·7 (21·3-38·2)	33·9 (24·5-45·6)	30·2 (12·7-58·6)	98·9 (97·7-99·5)	99·0 (97·6-99·7)	99·0 (97·5-99·7)	99·1 (92·6-100·0)	15·5 (9·9-22·4)	16·2 (9·1-27·6)	15·4 (8·4-27·9)	16·6 (3·0-55·5)	9·2 (5·8-13·8)	10·3 (6·0-17·4)	9·8 (5·5-17·1)	11·3 (2·2-38·2)

Location	Indicator 3.3.1: Age-standardised rate of new HIV infections (per 1,000 population)				Indicator 3.3.2: Age-standardised rate of tuberculosis cases (per 100,000 population)				Indicator 3.3.3: Age-standardised rate of malaria cases (per 1,000 population)				Indicator 3.3.4: Age-standardised rate of hepatitis B incidence (per 100,000 population)			
	**2000**	**2015**	**2016**	**2030**	**2000**	**2015**	**2016**	**2030**	**2000**	**2015**	**2016**	**2030**	**2000**	**2015**	**2016**	**2030**
Azerbaijan	0·02 (0·01-0·03)	0·03 (0·01-0·04)	0·03 (0·01-0·05)	0·02 (0·01-0·04)	202·5 (176·5-231·2)	123·8 (109·1-140·3)	121·3 (106·8-137·7)	85·8 (74·3-99·4)	0·02 (0·02-0·02)	0·0 (0·0-0·0)	0·0 (0·0-0·0)	0·0 (0·0-0·0)	1,055·9 (790·0-1,402·4)	846·8 (642·4-1,126·3)	819·4 (620·1-1,092·1)	496·4 (374·7-685·0)
Georgia	0·004 (0·003-0·007)	0·03 (0·02-0·04)	0·03 (0·02-0·04)	0·03 (0·02-0·05)	85·9 (74·6-97·9)	75·2 (66·3-84·3)	72·5 (63·8-81·3)	43·9 (37·1-51·5)	0·007 (0·007-0·008)	0·0 (0·0-0·0)	0·0 (0·0-0·0)	0·0 (0·0-0·0)	465·2 (358·0-605·4)	788·5 (600·7-1,039·8)	810·5 (616·3-1,074·8)	1,154·1 (846·0-1,615·4)
Kazakhstan	0·05 (0·04-0·07)	0·07 (0·04-0·1)	0·08 (0·05-0·1)	0·08 (0·05-0·1)	175·3 (152·1-200·5)	91·1 (81·4-102·3)	84·9 (75·8-95·4)	42·4 (38·8-46·8)	0·0 (0·0-0·0)	0·0 (0·0-0·0)	0·0 (0·0-0·0)	0·0 (0·0-0·0)	1,764·4 (1,318·4-2,365·4)	491·4 (383·3-637·0)	463·5 (363·5-600·5)	202·0 (164·4-254·6)
Kyrgyzstan	0·06 (0·04-0·08)	0·1 (0·06-0·1)	0·1 (0·06-0·2)	0·1 (0·07-0·2)	143·0 (123·5-163·7)	114·9 (103·0-127·7)	116·0 (103·5-129·7)	123·6 (99·4-151·3)	0·05 (0·05-0·05)	0·0 (0·0-0·0)	0·0 (0·0-0·0)	0·0 (0·0-0·0)	2,531·4 (1,873·2-3,434·8)	1,217·1 (913·6-1,645·3)	1,176·8 (884·2-1,590·0)	638·3 (481·6-838·0)
Mongolia	0·0 (0·0-0·002)	0·007 (0·001-0·02)	0·006 (0·001-0·02)	0·006 (0·001-0·02)	243·6 (216·4-273·5)	166·4 (145·4-189·5)	160·1 (139·5-183·5)	99·5 (85·0-117·3)	0·0 (0·0-0·0)	0·0 (0·0-0·0)	0·0 (0·0-0·0)	0·0 (0·0-0·0)	5,392·9 (3,928·6-7,440·9)	1,634·7 (1,181·7-2,302·3)	1,592·4 (1,150·1-2,250·2)	1,075·9 (737·2-1,604·2)
Tajikistan	0·05 (0·04-0·07)	0·03 (0·02-0·04)	0·03 (0·02-0·04)	0·03 (0·02-0·05)	139·4 (123·2-156·6)	82·4 (72·1-92·4)	78·9 (68·9-88·5)	41·9 (34·7-47·3)	8·3 (2·7-12·1)	0·001 (0·001-0·001)	0·0 (0·0-0·0)	0·0 (0·0-0·0)	3,984·7 (2,914·8-5,413·5)	2,131·6 (1,598·3-2,871·1)	2,103·1 (1,575·3-2,837·4)	1,604·1 (1,181·9-2,181·1)
Turkmenistan	0·1 (0·08-0·1)	0·05 (0·04-0·08)	0·05 (0·04-0·08)	0·05 (0·04-0·08)	243·8 (212·9-279·9)	164·0 (145·1-186·5)	159·1 (140·8-181·0)	105·4 (90·9-118·5)	0·0 (0·0-0·0)	0·0 (0·0-0·0)	0·0 (0·0-0·0)	0·0 (0·0-0·0)	4,378·2 (3,280·7-5,918·1)	2,030·5 (1,505·5-2,798·0)	1,951·4 (1,446·8-2,693·6)	1,070·4 (769·5-1,493·9)
Uzbekistan	0·06 (0·05-0·09)	0·007 (0·001-0·01)	0·006 (0·001-0·01)	0·005 (0·001-0·01)	93·4 (80·4-107·3)	69·4 (62·7-76·9)	68·0 (60·9-75·5)	48·7 (39·4-58·9)	0·0 (0·0-0·0)	0·0 (0·0-0·0)	0·0 (0·0-0·0)	0·0 (0·0-0·0)	3,339·4 (2,489·7-4,469·2)	1,565·7 (1,162·2-2,140·7)	1,502·2 (1,115·5-2,057·4)	817·2 (601·9-1,151·9)
**Central Latin America**																
Colombia	0·07 (0·05-0·1)	0·1 (0·07-0·2)	0·1 (0·07-0·2)	0·1 (0·07-0·1)	32·9 (28·8-37·2)	22·8 (19·8-25·9)	22·3 (19·4-25·4)	16·5 (14·2-19·0)	9·5 (6·6-10·9)	1·1 (0·9-1·6)	0·9 (0·8-1·3)	0·1 (0·1-0·2)	708·5 (520·1-945·0)	268·1 (202·9-344·7)	261·4 (199·0-334·8)	181·1 (142·5-223·7)
Costa Rica	0·07 (0·05-0·08)	0·09 (0·06-0·1)	0·08 (0·06-0·1)	0·08 (0·06-0·1)	17·6 (15·2-20·0)	10·2 (8·7-11·7)	9·9 (8·4-11·4)	6·6 (5·5-7·7)	0·004 (0·004-0·004)	0·0 (0·0-0·0)	0·0 (0·0-0·0)	0·0 (0·0-0·0)	452·9 (347·1-575·6)	294·9 (226·3-375·0)	288·5 (221·3-365·6)	211·1 (162·4-267·8)
El Salvador	0·2 (0·2-0·2)	0·07 (0·05-0·1)	0·06 (0·04-0·09)	0·06 (0·05-0·09)	40·3 (35·1-45·6)	24·3 (21·2-27·4)	24·0 (20·8-26·9)	15·3 (13·4-17·1)	0·006 (0·006-0·006)	0·0 (0·0-0·0)	0·0 (0·0-0·0)	0·0 (0·0-0·0)	164·8 (129·0-210·2)	154·6 (120·2-197·0)	153·3 (119·2-194·9)	134·7 (103·9-171·7)
Guatemala	0·2 (0·1-0·3)	0·1 (0·05-0·2)	0·1 (0·05-0·2)	0·1 (0·05-0·2)	37·0 (31·5-42·6)	26·0 (22·1-29·9)	25·4 (21·6-29·2)	17·9 (15·4-20·5)	18·9 (11·6-28·4)	0·5 (0·4-0·5)	0·4 (0·4-0·4)	0·02 (0·009-0·04)	205·0 (162·6-258·1)	427·1 (332·7-540·8)	407·3 (317·6-514·8)	209·9 (164·2-260·7)
Honduras	0·2 (0·1-0·2)	0·1 (0·08-0·2)	0·1 (0·07-0·2)	0·1 (0·07-0·2)	74·3 (64·3-84·3)	50·4 (43·8-57·0)	49·4 (43·0-56·0)	37·3 (32·3-42·4)	7·9 (5·5-10·9)	0·5 (0·5-0·5)	0·4 (0·4-0·4)	0·03 (0·03-0·04)	742·7 (556·1-978·8)	470·3 (363·1-598·8)	456·9 (353·8-578·1)	301·2 (240·1-369·0)
Mexico	0·07 (0·07-0·08)	0·1 (0·09-0·2)	0·1 (0·09-0·2)	0·1 (0·09-0·2)	31·5 (27·0-35·9)	20·0 (17·2-22·8)	20·3 (17·5-23·3)	21·6 (17·1-26·7)	0·1 (0·1-0·1)	0·006 (0·006-0·006)	0·005 (0·005-0·005)	0·0 (0·0-0·0)	672·1 (515·0-848·6)	295·7 (227·6-370·0)	291·3 (224·5-364·6)	231·8 (180·3-290·4)
Nicaragua	0·04 (0·03-0·06)	0·1 (0·06-0·2)	0·1 (0·06-0·2)	0·1 (0·06-0·2)	66·9 (57·4-76·0)	39·9 (34·1-45·6)	39·3 (33·6-44·9)	31·3 (26·8-36·0)	8·9 (6·9-11·2)	0·3 (0·3-0·3)	0·4 (0·4-0·4)	0·01 (0·005-0·02)	932·8 (694·4-1,239·5)	419·9 (328·2-538·0)	403·8 (316·3-516·8)	220·8 (176·3-274·6)
Panama	0·2 (0·2-0·3)	0·4 (0·3-0·5)	0·4 (0·3-0·5)	0·3 (0·2-0·4)	48·0 (41·1-55·2)	41·7 (36·0-47·9)	41·2 (35·5-47·3)	33·8 (29·2-39·0)	0·7 (0·4-1·0)	0·1 (0·1-0·1)	0·1 (0·1-0·1)	0·2 (0·02-0·5)	502·7 (385·9-649·5)	267·6 (208·8-344·3)	262·6 (204·6-338·1)	195·5 (152·9-249·1)
Venezuela	0·1 (0·1-0·2)	0·2 (0·1-0·2)	0·2 (0·1-0·2)	0·2 (0·1-0·2)	31·0 (26·2-35·7)	24·1 (20·8-27·3)	24·0 (20·8-27·2)	18·2 (16·5-20·1)	1·9 (1·3-2·8)	6·4 (4·4-8·6)	7·9 (5·3-10·6)	24·8 (19·8-29·5)	676·7 (526·8-846·0)	432·6 (337·5-542·0)	431·3 (336·3-541·6)	410·3 (318·5-515·1)
**Andean Latin America**																
Bolivia	0·08 (0·02-0·2)	0·1 (0·02-0·4)	0·1 (0·02-0·3)	0·1 (0·02-0·3)	189·3 (169·7-208·0)	104·9 (93·9-115·1)	101·8 (91·2-111·8)	62·3 (55·6-69·0)	7·0 (6·0-9·6)	0·9 (0·8-1·2)	0·9 (0·7-1·2)	0·6 (0·3-0·8)	928·6 (724·6-1,187·3)	502·9 (397·1-623·9)	484·9 (382·8-599·9)	282·5 (224·7-356·0)
Ecuador	0·09 (0·07-0·1)	0·1 (0·09-0·2)	0·1 (0·08-0·2)	0·1 (0·08-0·1)	69·0 (60·2-79·1)	35·7 (31·0-40·6)	35·4 (30·7-40·3)	24·5 (20·8-29·0)	14·2 (10·3-16·6)	0·05 (0·04-0·06)	0·04 (0·03-0·05)	0·001 (0·001-0·001)	424·1 (334·0-539·7)	274·7 (218·7-344·9)	267·8 (213·4-335·3)	177·8 (143·6-222·4)
Peru	0·07 (0·06-0·08)	0·05 (0·03-0·08)	0·05 (0·03-0·08)	0·05 (0·03-0·08)	147·3 (131·7-163·4)	62·3 (55·9-69·1)	59·3 (53·2-65·8)	29·4 (26·2-32·8)	5·2 (4·0-6·6)	2·9 (2·3-3·6)	3·2 (2·5-4·0)	2·0 (1·8-2·3)	623·4 (481·4-794·2)	262·0 (209·6-322·9)	249·4 (200·1-306·8)	124·0 (103·2-146·4)
**Caribbean**																
Antigua and Barbuda	0·2 (0·2-0·2)	0·1 (0·09-0·2)	0·1 (0·08-0·2)	0·06 (0·05-0·09)	17·0 (13·8-20·4)	12·3 (10·1-14·7)	12·2 (10·0-14·5)	10·0 (8·2-12·1)	0·0 (0·0-0·0)	0·0 (0·0-0·0)	0·0 (0·0-0·0)	0·0 (0·0-0·0)	256·4 (195·8-327·9)	168·9 (131·2-213·1)	165·1 (128·7-207·8)	118·5 (96·5-144·4)
The Bahamas	0·7 (0·6-0·7)	0·4 (0·3-0·6)	0·4 (0·3-0·5)	0·2 (0·1-0·3)	60·5 (50·6-71·0)	41·7 (35·7-48·4)	41·5 (35·6-48·2)	37·7 (32·1-44·1)	0·0 (0·0-0·0)	0·0 (0·0-0·0)	0·0 (0·0-0·0)	0·0 (0·0-0·0)	254·7 (191·8-333·6)	205·0 (155·2-268·1)	205·3 (155·4-267·7)	200·6 (150·2-260·9)
Barbados	0·2 (0·2-0·3)	0·2 (0·1-0·3)	0·2 (0·1-0·3)	0·2 (0·1-0·3)	15·3 (12·5-18·3)	11·9 (9·9-14·1)	11·8 (9·8-13·9)	9·9 (8·2-11·7)	0·0 (0·0-0·0)	0·0 (0·0-0·0)	0·0 (0·0-0·0)	0·0 (0·0-0·0)	256·0 (194·0-335·9)	193·5 (147·6-250·4)	193·3 (147·5-250·5)	188·9 (143·4-243·6)
Belize	0·9 (0·2-1·3)	0·4 (0·3-0·6)	0·4 (0·3-0·6)	0·3 (0·2-0·4)	64·7 (54·3-75·6)	42·9 (36·0-50·0)	40·9 (34·4-47·8)	23·8 (19·8-28·3)	19·9 (8·9-36·7)	0·05 (0·04-0·05)	0·03 (0·03-0·03)	0·0 (0·0-0·0)	510·9 (390·2-655·7)	311·0 (240·7-397·4)	304·9 (235·7-389·7)	228·5 (174·7-290·0)
Cuba	0·04 (0·03-0·06)	0·3 (0·2-0·4)	0·3 (0·2-0·5)	0·3 (0·2-0·5)	10·0 (8·5-11·6)	6·3 (5·3-7·4)	6·1 (5·1-7·2)	4·5 (3·7-5·3)	0·0 (0·0-0·0)	0·0 (0·0-0·0)	0·0 (0·0-0·0)	0·0 (0·0-0·0)	304·2 (227·9-399·7)	126·6 (99·9-158·5)	124·8 (98·4-156·0)	101·6 (79·6-126·8)
Dominica	0·1 (0·1-0·1)	0·1 (0·08-0·1)	0·1 (0·08-0·1)	0·1 (0·08-0·1)	55·0 (46·1-64·8)	42·5 (35·9-49·7)	42·1 (35·6-49·3)	37·2 (31·3-43·7)	0·0 (0·0-0·0)	0·0 (0·0-0·0)	0·0 (0·0-0·0)	0·0 (0·0-0·0)	217·9 (168·1-278·3)	166·9 (130·0-211·6)	166·3 (129·5-211·4)	136·8 (106·8-174·4)
Dominican Republic	1·1 (0·7-1·4)	0·5 (0·4-0·7)	0·5 (0·4-0·7)	0·3 (0·2-0·4)	82·5 (71·7-93·6)	55·3 (48·3-63·1)	54·3 (47·3-61·9)	37·2 (31·7-43·1)	0·3 (0·2-0·3)	0·07 (0·07-0·08)	0·06 (0·06-0·06)	0·002 (0·002-0·004)	945·0 (734·1-1,193·1)	451·2 (354·5-564·4)	441·2 (347·0-552·7)	314·1 (246·1-396·1)
Grenada	0·1 (0·1-0·2)	0·1 (0·08-0·1)	0·1 (0·07-0·1)	0·09 (0·07-0·1)	12·9 (10·4-15·4)	10·6 (8·7-12·8)	10·5 (8·5-12·6)	8·3 (6·7-10·0)	0·0 (0·0-0·0)	0·0 (0·0-0·0)	0·0 (0·0-0·0)	0·0 (0·0-0·0)	340·3 (255·6-441·7)	209·8 (162·3-265·9)	202·6 (156·9-256·2)	121·9 (97·7-150·6)

Location	Indicator 3.3.5: Age-standardised prevalence* of the sum of 15 neglected tropical diseases (NTDs) (%) *Prevalence estimates reported here may exceed 100% as they reflect the sum of prevalent cases of 15 NTDs.				Indicator 3.4.1: Age-standardised death rate due to cardiovascular disease, cancer, diabetes, and chronic respiratory disease in populations aged 30-70 (per 100,000 population)				Indicator 3.4.2: Age-standardised death rate due to self-harm (per 100,000 population)				Indicator 3.5.2: Risk-weighted prevalence of alcohol consumption, as measured by the summary exposure value (SEV) for alcohol use (%)			
	**2000**	**2015**	**2016**	**2030**	**2000**	**2015**	**2016**	**2030**	**2000**	**2015**	**2016**	**2030**	**2000**	**2015**	**2016**	**2030**
Azerbaijan	3·8 (2·7-5·4)	3·4 (2·5-4·7)	3·4 (2·5-4·7)	3·4 (2·4-4·6)	717·3 (659·9-775·5)	550·1 (466·3-633·5)	541·8 (446·8-646·3)	428·2 (254·6-664·9)	3·0 (2·6-3·8)	4·1 (2·9-5·4)	4·1 (2·8-5·5)	3·8 (1·7-7·9)	11·4 (8·0-15·4)	14·1 (9·1-19·4)	14·1 (9·0-19·5)	13·9 (7·6-20·8)
Georgia	0·7 (0·5-0·9)	0·7 (0·5-0·9)	0·7 (0·5-0·9)	0·6 (0·5-0·8)	552·0 (504·8-597·3)	528·3 (456·5-610·8)	517·6 (428·3-620·2)	417·9 (256·4-657·9)	6·2 (5·0-7·3)	6·8 (5·3-8·4)	6·7 (5·0-8·6)	7·8 (4·2-13·7)	9·4 (7·0-12·2)	13·8 (9·5-18·2)	14·0 (9·6-18·6)	18·4 (11·2-25·1)
Kazakhstan	23·9 (18·5-31·3)	24·0 (17·9-32·4)	24·0 (17·9-32·4)	24·0 (17·8-32·3)	999·2 (910·9-1,090·9)	569·1 (510·6-645·7)	572·2 (480·4-680·0)	297·9 (183·0-461·4)	38·1 (27·9-43·9)	24·9 (20·2-30·0)	25·0 (19·2-32·5)	14·2 (7·3-26·4)	18·7 (14·0-23·5)	20·7 (13·9-28·1)	20·6 (13·7-28·1)	19·8 (12·0-29·3)
Kyrgyzstan	24·6 (18·8-32·5)	24·8 (18·6-32·9)	24·8 (18·6-32·9)	24·7 (18·5-32·7)	757·3 (726·6-791·1)	537·2 (508·7-566·2)	534·4 (485·6-582·5)	319·3 (244·5-413·8)	17·7 (14·2-20·5)	12·4 (10·7-16·7)	12·4 (10·2-16·9)	8·9 (5·7-16·6)	13·8 (10·6-16·9)	13·5 (9·3-17·5)	13·3 (9·2-17·4)	11·6 (7·0-16·3)
Mongolia	0·5 (0·4-1·0)	0·4 (0·3-0·5)	0·4 (0·3-0·5)	0·3 (0·2-0·4)	1,007·6 (933·7-1,083·1)	812·5 (722·1-905·8)	800·6 (697·2-908·2)	622·8 (401·3-906·3)	22·3 (18·7-25·6)	19·9 (16·1-23·8)	20·1 (16·1-24·6)	17·6 (10·5-28·0)	7·7 (5·4-10·4)	12·0 (7·7-16·7)	12·2 (7·7-16·9)	14·3 (8·5-20·5)
Tajikistan	10·3 (7·7-13·8)	10·2 (7·5-13·8)	10·1 (7·5-13·8)	10·0 (7·4-13·6)	682·6 (619·1-751·1)	484·3 (426·9-564·7)	478·1 (418·4-560·0)	368·6 (252·1-515·3)	6·1 (5·2-7·9)	5·4 (4·3-6·8)	5·3 (4·3-6·8)	5·0 (3·0-7·8)	4·8 (3·3-6·6)	3·6 (2·3-5·1)	3·6 (2·3-5·0)	3·1 (1·9-4·5)
Turkmenistan	3·2 (2·3-4·5)	3·1 (2·3-4·4)	3·1 (2·3-4·4)	3·0 (2·2-4·3)	856·3 (832·8-882·4)	631·0 (600·3-659·6)	614·9 (582·6-645·3)	439·7 (380·4-501·2)	12·2 (8·0-13·6)	8·4 (6·7-10·0)	8·4 (6·7-10·0)	6·3 (4·3-9·1)	10·5 (7·0-14·4)	10·4 (6·6-15·2)	10·4 (6·5-15·2)	10·1 (5·9-15·6)
Uzbekistan	0·4 (0·3-0·8)	0·3 (0·2-0·5)	0·3 (0·2-0·5)	0·2 (0·2-0·3)	818·1 (770·6-869·2)	652·7 (595·4-717·7)	642·8 (578·3-721·8)	518·9 (369·8-727·1)	12·6 (8·8-14·2)	10·9 (8·1-13·0)	10·8 (8·1-13·0)	9·7 (6·2-14·3)	8·4 (5·7-11·4)	11·6 (7·5-16·7)	11·8 (7·6-17·1)	15·1 (9·2-22·2)
**Central Latin America**																
Colombia	57·9 (52·6-63·7)	57·9 (51·9-64·3)	57·9 (51·9-64·2)	57·5 (51·4-63·9)	352·9 (340·0-367·0)	219·6 (202·5-237·2)	217·7 (196·9-239·5)	166·8 (132·4-200·5)	7·8 (6·3-10·0)	5·7 (4·5-7·1)	5·7 (4·4-7·2)	5·4 (2·9-8·3)	13·4 (9·9-17·2)	13·8 (9·5-18·5)	13·8 (9·5-18·7)	14·8 (9·3-20·9)
Costa Rica	42·1 (34·3-51·9)	42·3 (34·6-51·7)	42·2 (34·5-51·7)	42·0 (34·4-51·5)	252·5 (243·3-262·9)	197·8 (183·6-212·6)	195·9 (179·9-212·5)	171·8 (134·1-217·1)	7·0 (5·0-7·8)	6·8 (4·9-7·9)	6·8 (4·9-8·0)	6·6 (4·4-9·3)	12·9 (10·1-16·0)	12·1 (8·2-16·7)	12·2 (8·2-16·8)	12·9 (8·1-18·5)
El Salvador	46·0 (41·2-51·3)	19·9 (16·3-24·6)	17·6 (14·4-21·8)	3·1 (2·5-3·9)	280·4 (264·8-299·9)	262·0 (236·2-291·9)	257·3 (228·4-289·0)	233·7 (184·1-292·4)	10·6 (9·4-12·7)	9·4 (7·9-11·8)	9·4 (7·7-11·8)	9·0 (5·9-12·9)	8·3 (6·3-10·4)	10·3 (7·0-13·8)	10·3 (7·1-13·9)	11·4 (7·5-15·6)
Guatemala	124·6 (114·8-134·6)	76·5 (65·5-88·3)	72·4 (62·2-83·5)	34·0 (29·5-38·9)	312·8 (269·3-360·0)	303·8 (242·2-373·8)	302·2 (232·5-386·3)	280·5 (142·1-503·9)	13·4 (7·7-17·0)	7·0 (5·1-10·8)	7·1 (4·9-11·1)	4·6 (1·5-11·5)	7·4 (6·5-8·3)	7·4 (6·2-8·6)	7·4 (6·2-8·6)	7·3 (6·1-8·6)
Honduras	73·6 (61·6-87·5)	61·0 (51·6-71·6)	59·8 (50·6-70·2)	45·7 (38·7-53·6)	467·5 (362·7-578·1)	392·8 (300·2-509·1)	389·0 (298·6-504·7)	338·6 (212·0-515·4)	4·8 (3·2-6·6)	4·6 (3·0-6·9)	4·6 (3·0-6·8)	4·4 (2·1-8·3)	6·9 (6·0-7·8)	7·4 (6·1-8·6)	7·4 (6·1-8·6)	7·3 (5·9-8·7)
Mexico	27·2 (23·6-31·5)	26·7 (24·5-29·0)	26·6 (24·5-28·9)	25·5 (23·4-27·7)	322·0 (316·2-327·3)	284·0 (277·5-290·8)	281·6 (274·1-289·6)	250·1 (222·9-280·3)	4·9 (3·8-5·3)	5·8 (4·4-6·5)	5·8 (4·3-6·5)	5·9 (4·2-7·1)	14·3 (12·3-16·3)	12·8 (10·0-15·7)	12·7 (9·9-15·8)	12·1 (8·5-16·1)
Nicaragua	35·1 (31·3-39·2)	16·2 (12·8-20·4)	14·5 (11·5-18·3)	3·1 (2·4-3·8)	260·9 (249·0-272·9)	239·2 (209·2-270·5)	235·2 (199·5-273·3)	211·4 (145·0-298·6)	9·0 (7·1-10·1)	7·4 (6·0-8·9)	7·3 (5·7-9·1)	6·7 (3·9-10·9)	8·9 (7·3-10·4)	8·8 (6·9-11·0)	8·9 (6·9-11·0)	8·7 (6·6-11·2)
Panama	16·1 (13·5-19·3)	12·8 (10·1-16·1)	12·7 (10·1-16·0)	11·6 (9·1-14·7)	263·3 (245·5-282·3)	234·7 (209·5-262·3)	229·5 (200·8-263·2)	196·9 (137·7-278·8)	6·6 (4·7-7·6)	5·9 (4·7-7·7)	5·9 (4·5-7·9)	5·6 (2·9-9·8)	11·1 (9·5-12·7)	12·3 (9·9-14·7)	12·4 (10·0-14·8)	13·8 (11·0-16·7)
Venezuela	75·8 (70·5-82·0)	72·4 (61·9-85·8)	71·8 (61·4-85·1)	63·7 (54·2-75·9)	370·9 (347·6-394·2)	328·7 (286·2-381·2)	327·6 (279·4-390·9)	313·3 (199·7-474·9)	9·6 (7·4-11·6)	9·4 (6·7-14·0)	9·4 (6·5-14·3)	10·6 (4·7-22·8)	14·0 (11·9-16·1)	13·4 (10·6-16·3)	13·3 (10·6-16·3)	13·0 (10·1-16·3)
**Andean Latin America**																
Bolivia	34·5 (30·9-38·6)	33·9 (29·9-38·4)	33·6 (29·6-38·1)	29·3 (25·5-33·7)	403·8 (372·9-432·4)	338·4 (272·5-413·3)	336·6 (268·9-415·8)	307·0 (192·3-468·6)	8·3 (6·8-9·6)	7·2 (5·5-9·1)	7·2 (5·5-9·1)	6·6 (3·9-10·4)	6·6 (4·5-8·8)	7·7 (5·0-11·1)	7·8 (5·0-11·4)	9·9 (5·8-15·7)
Ecuador	80·7 (72·1-90·4)	65·6 (55·2-77·6)	64·1 (53·9-75·8)	46·5 (39·4-55·4)	292·0 (279·3-305·2)	224·2 (209·3-240·1)	222·2 (205·4-239·6)	177·6 (149·5-212·4)	7·9 (6·3-8·9)	7·9 (5·8-9·2)	7·9 (5·6-9·2)	6·9 (4·5-9·5)	10·8 (9·4-12·5)	10·5 (8·8-12·2)	10·4 (8·7-12·1)	9·8 (8·3-11·5)
Peru	48·1 (40·9-57·0)	34·7 (28·0-43·5)	33·3 (26·9-41·7)	18·7 (15·3-23·2)	248·4 (228·4-267·8)	190·7 (160·7-226·5)	187·3 (152·8-229·5)	145·2 (80·0-237·2)	4·5 (3·7-5·2)	3·1 (2·4-3·8)	3·0 (2·3-3·9)	2·3 (1·2-4·0)	16·5 (12·5-20·9)	14·6 (9·8-20·4)	14·5 (9·7-20·4)	13·3 (8·1-20·1)
**Caribbean**																
Antigua and Barbuda	3·6 (0·3-5·7)	3·7 (0·4-5·8)	3·7 (0·4-5·9)	4·3 (0·6-6·7)	412·8 (382·6-443·7)	336·1 (300·6-374·7)	333·5 (296·4-375·5)	299·8 (226·1-389·9)	2·2 (1·8-2·6)	2·3 (1·7-2·8)	2·3 (1·7-2·8)	2·2 (1·3-3·4)	4·1 (2·5-5·8)	5·9 (2·8-10·0)	6·0 (2·8-10·2)	7·1 (2·6-13·9)
The Bahamas	0·3 (0·2-0·3)	0·4 (0·3-0·5)	0·4 (0·3-0·5)	0·6 (0·4-0·8)	480·4 (457·2-503·2)	423·5 (385·9-466·1)	428·1 (383·4-477·2)	432·0 (329·0-574·6)	2·7 (2·1-3·1)	3·1 (2·4-3·8)	3·1 (2·4-3·8)	3·2 (2·1-4·9)	14·3 (11·3-17·3)	11·2 (6·2-16·5)	11·0 (5·9-16·4)	8·2 (3·3-14·0)
Barbados	0·4 (0·2-0·5)	0·5 (0·4-0·6)	0·5 (0·4-0·6)	0·7 (0·5-0·9)	379·0 (350·9-409·2)	341·1 (314·5-372·6)	342·4 (314·1-375·8)	313·6 (255·1-391·1)	4·5 (3·7-5·3)	4·9 (3·9-5·8)	4·9 (3·9-5·8)	5·1 (3·5-7·2)	11·0 (8·9-13·0)	12·9 (9·2-16·8)	13·0 (9·2-16·9)	14·1 (9·5-19·0)
Belize	27·0 (22·1-32·5)	10·7 (8·4-13·2)	9·4 (7·4-11·6)	1·5 (1·2-1·9)	575·0 (533·5-624·7)	461·1 (407·0-518·2)	461·5 (396·4-523·2)	382·0 (260·6-523·7)	8·0 (5·7-9·1)	7·9 (5·9-9·7)	7·9 (5·8-10·0)	7·7 (4·4-12·8)	12·5 (9·7-15·3)	15·4 (11·3-19·2)	15·4 (11·3-19·3)	16·1 (10·7-21·5)
Cuba	31·7 (29·9-33·6)	31·9 (29·8-34·1)	31·9 (29·8-34·1)	31·9 (29·9-34·2)	401·2 (384·5-416·9)	334·9 (311·8-358·9)	332·1 (305·6-358·7)	286·0 (233·1-346·3)	16·7 (14·7-18·7)	12·0 (10·3-15·3)	12·0 (10·2-15·1)	10·7 (7·5-15·1)	8·6 (6·6-10·9)	11·4 (7·7-15·4)	11·5 (7·8-15·7)	14·3 (9·2-19·9)
Dominica	0·3 (0·3-0·4)	0·3 (0·2-0·4)	0·3 (0·2-0·4)	0·3 (0·2-0·5)	422·1 (393·6-452·2)	431·2 (388·3-480·9)	429·2 (380·2-485·6)	423·0 (311·1-582·5)	3·7 (3·0-4·4)	4·6 (3·2-5·7)	4·6 (3·2-5·8)	5·4 (3·0-9·0)	14·8 (12·2-17·6)	14·3 (10·2-18·9)	14·3 (10·2-19·0)	14·7 (9·8-20·2)
Dominican Republic	2·0 (1·8-2·4)	1·5 (1·3-1·8)	1·5 (1·3-1·7)	1·1 (0·9-1·4)	320·6 (277·1-354·4)	279·3 (228·5-328·6)	278·7 (227·8-329·2)	257·5 (170·7-369·2)	7·0 (5·4-8·4)	6·0 (4·6-7·5)	6·0 (4·5-7·6)	6·3 (3·5-10·4)	10·2 (7·8-12·8)	11·2 (7·6-15·3)	11·3 (7·6-15·5)	12·6 (7·7-18·6)
Grenada	29·9 (26·9-33·3)	33·1 (27·3-40·2)	33·0 (27·3-40·2)	32·8 (26·9-39·8)	571·4 (523·9-625·1)	559·3 (490·8-629·7)	553·3 (477·7-628·2)	517·6 (359·5-704·9)	6·7 (5·2-8·0)	7·1 (5·4-8·8)	7·1 (5·4-8·9)	7·7 (4·5-12·6)	15·5 (13·1-17·8)	13·5 (9·8-17·0)	13·4 (9·7-17·0)	12·5 (8·4-16·7)

Location	Indicator 3.6.1: Age-standardised death rate due to road injuries (per 100,000 population)				Indicator 3.7.1: Proportion of women of reproductive age (15-49 years) who have their need for family planning satisfied with modern contraception methods (%)				Indicator 3.7.2: Number of livebirths per 1,000 women aged 10-14 years and women aged 15-19 years				Indicator 3.8.1: Coverage of essential health services, as defined by the UHC index comprised of the coverage of 9 tracer interventions and risk-standardised death rates from 32 causes amenable to personal healthcare (scale of 0 to 100)			
	**2000**	**2015**	**2016**	**2030**	**2000**	**2015**	**2016**	**2030**	**2000**	**2015**	**2016**	**2030**	**2000**	**2015**	**2016**	**2030**
Azerbaijan	9·3 (8·4-10·3)	8·1 (6·7-9·8)	8·1 (6·5-10·1)	7·5 (4·0-13·1)	30·9 (25·2-36·5)	38·3 (31·1-45·8)	39·5 (32·3-46·9)	56·2 (36·5-76·1)	15·2 (14·6-15·9)	32·8 (30·6-35·2)	33·6 (31·0-36·3)	46·8 (35·7-59·5)	41·8 (40·2-43·3)	56·9 (53·6-59·9)	57·7 (54·3-60·7)	72·2 (66·7-77·0)
Georgia	9·5 (8·6-10·5)	16·3 (13·5-19·5)	15·8 (12·5-19·6)	22·0 (13·7-35·3)	45·9 (41·8-49·9)	55·8 (48·1-63·6)	56·2 (48·6-64·0)	63·5 (47·4-78·3)	23·2 (21·4-25·2)	26·9 (24·0-30·5)	24·1 (21·2-27·8)	24·5 (16·2-36·1)	54·0 (52·0-55·7)	58·8 (56·1-61·4)	59·2 (56·3-62·0)	64·3 (59·4-69·2)
Kazakhstan	19·1 (17·0-21·3)	17·3 (15·0-20·1)	17·2 (14·0-20·9)	9·0 (4·6-16·4)	66·8 (64·1-69·4)	78·5 (76·6-80·4)	78·7 (76·3-81·1)	84·3 (80·8-87·6)	15·6 (14·7-16·4)	14·3 (13·3-15·3)	12·9 (11·9-14·0)	11·9 (9·3-14·9)	49·4 (47·6-51·4)	61·8 (59·1-64·6)	62·7 (59·8-65·7)	75·8 (72·1-79·3)
Kyrgyzstan	16·1 (15·1-17·2)	17·9 (16·3-19·6)	17·3 (15·2-19·7)	13·0 (8·9-19·1)	66·2 (61·4-70·8)	63·3 (59·9-66·4)	63·6 (59·5-67·5)	59·3 (50·9-67·3)	19·0 (17·8-20·1)	20·7 (18·6-22·8)	19·8 (17·7-22·1)	17·2 (12·6-23·2)	51·0 (49·6-52·3)	58·9 (57·1-60·7)	59·6 (57·8-61·6)	68·5 (65·8-71·2)
Mongolia	16·9 (14·9-18·9)	21·1 (17·9-24·4)	21·2 (17·7-25·0)	19·0 (11·4-29·9)	66·4 (59·7-72·4)	67·0 (63·6-70·2)	67·4 (63·4-71·4)	71·9 (61·0-81·0)	11·7 (10·7-12·8)	8·6 (7·6-9·8)	7·7 (6·6-9·0)	6·5 (4·6-8·7)	46·1 (44·2-48·0)	58·6 (55·6-61·3)	59·0 (56·0-61·8)	67·1 (63·1-70·8)
Tajikistan	10·4 (9·2-11·7)	8·1 (6·7-9·7)	8·1 (6·7-9·7)	6·7 (4·2-10·1)	49·0 (42·4-55·7)	53·3 (48·0-59·1)	53·6 (47·6-60·2)	57·7 (43·7-70·7)	16·7 (15·8-17·8)	18·8 (16·3-21·4)	18·6 (16·1-21·3)	18·7 (12·5-25·8)	44·3 (42·2-46·3)	55·1 (52·4-57·9)	55·8 (53·0-58·4)	65·9 (62·1-69·6)
Turkmenistan	14·1 (13·1-15·0)	8·3 (7·5-9·1)	8·3 (7·5-9·1)	4·2 (3·0-5·7)	59·0 (51·4-66·3)	72·0 (69·6-74·6)	72·6 (69·9-75·3)	82·3 (76·0-87·7)	10·8 (9·8-11·9)	8·4 (7·3-9·8)	8·1 (7·0-9·4)	6·2 (4·5-8·2)	48·6 (47·0-50·1)	54·9 (53·0-56·9)	55·8 (53·8-57·8)	63·5 (60·6-66·2)
Uzbekistan	16·1 (14·9-17·6)	16·0 (13·9-18·5)	15·7 (13·2-18·3)	14·2 (9·3-20·0)	76·6 (73·1-80·1)	79·8 (73·8-84·3)	80·0 (74·1-84·8)	83·8 (72·9-91·1)	12·6 (11·5-13·7)	9·3 (7·0-11·9)	9·1 (6·9-11·7)	8·2 (4·0-14·1)	51·9 (49·9-53·5)	59·7 (57·0-62·2)	60·2 (57·4-62·7)	68·6 (65·0-71·7)
**Central Latin America**																
Colombia	24·5 (22·4-28·9)	14·9 (13·2-17·2)	14·6 (12·8-17·1)	10·6 (7·3-14·7)	73·7 (72·4-74·9)	87·3 (86·6-88·1)	87·4 (86·3-88·4)	93·8 (92·3-95·0)	42·5 (40·9-44·3)	22·5 (20·9-24·2)	20·9 (19·2-22·8)	9·9 (8·3-11·8)	53·4 (52·1-54·7)	64·9 (63·0-66·7)	65·4 (63·4-67·2)	74·5 (71·5-76·9)
Costa Rica	20·4 (19·3-22·0)	14·7 (13·3-16·5)	14·5 (13·0-16·3)	11·8 (8·9-15·6)	83·2 (78·7-86·9)	87·1 (83·1-90·2)	87·3 (83·3-90·4)	89·7 (82·3-94·8)	36·4 (33·5-39·9)	24·8 (21·0-29·1)	24·0 (20·2-28·5)	16·5 (11·1-23·6)	61·3 (59·9-62·6)	68·7 (66·7-70·5)	69·0 (67·0-70·9)	73·8 (70·9-76·4)
El Salvador	36·0 (33·4-39·7)	23·6 (20·9-27·1)	23·4 (20·4-27·4)	15·9 (11·8-21·4)	75·9 (74·2-77·7)	81·4 (79·7-83·2)	81·8 (79·6-84·0)	87·8 (83·5-91·3)	44·5 (40·8-48·3)	33·7 (29·1-38·8)	33·3 (28·5-38·7)	25·0 (18·0-33·4)	54·5 (52·5-56·1)	62·4 (59·7-64·9)	62·8 (60·0-65·4)	68·4 (64·8-72·0)
Guatemala	16·0 (13·5-19·0)	17·7 (13·7-22·4)	17·6 (12·9-23·1)	16·4 (7·8-31·6)	58·6 (55·9-61·2)	67·3 (64·8-69·6)	67·7 (64·5-70·8)	69·2 (60·4-78·0)	53·6 (49·5-57·7)	36·6 (33·4-40·0)	36·6 (33·0-40·4)	26·4 (20·5-33·3)	46·7 (44·2-49·3)	53·9 (50·2-57·9)	54·3 (50·4-58·5)	60·6 (54·5-67·0)
Honduras	20·2 (15·2-25·9)	17·2 (12·8-22·9)	17·0 (12·7-22·6)	14·4 (8·7-23·6)	71·1 (69·1-73·1)	77·7 (74·0-81·2)	78·0 (73·6-81·7)	87·1 (80·1-92·3)	46·1 (39·3-52·7)	37·4 (31·9-43·3)	37·1 (31·4-43·4)	31·1 (20·9-44·7)	46·0 (42·6-49·8)	54·3 (50·1-58·3)	54·7 (50·4-58·6)	60·2 (55·5-64·6)
Mexico	20·8 (19·8-21·6)	16·6 (15·8-17·7)	16·5 (15·5-17·6)	11·6 (10·4-13·2)	77·2 (71·7-82·2)	79·2 (77·3-81·0)	79·7 (77·5-81·7)	82·2 (75·0-87·5)	43·0 (41·0-44·9)	31·3 (29·2-33·6)	30·8 (28·4-33·3)	23·1 (19·3-27·7)	54·5 (53·4-55·5)	59·8 (58·4-61·1)	60·4 (58·9-61·6)	65·2 (63·3-67·0)
Nicaragua	16·6 (15·5-18·3)	13·4 (11·3-16·0)	13·2 (10·8-16·2)	11·2 (6·9-17·8)	76·6 (74·9-78·3)	87·7 (84·1-90·9)	87·8 (84·1-90·9)	88·6 (81·0-93·4)	62·1 (61·3-62·8)	43·7 (43·0-44·4)	42·8 (42·1-43·4)	28·7 (27·5-30·1)	55·5 (54·1-56·8)	64·4 (61·8-67·1)	64·9 (62·2-67·7)	71·7 (67·2-76·0)
Panama	20·8 (18·8-23·2)	15·6 (13·3-18·6)	15·3 (12·7-18·5)	11·9 (6·8-18·5)	79·4 (73·5-84·3)	84·9 (80·1-88·6)	85·4 (80·6-89·1)	89·2 (81·6-94·5)	42·1 (39·1-45·4)	34·1 (29·6-39·1)	32·8 (27·9-38·3)	28·8 (20·0-41·1)	58·5 (56·9-59·9)	62·3 (59·7-64·8)	62·8 (60·1-65·5)	68·3 (64·0-72·6)
Venezuela	25·8 (23·2-29·8)	24·3 (19·2-31·3)	24·1 (18·5-32·1)	21·2 (10·9-39·0)	76·2 (70·0-81·4)	82·5 (77·5-86·6)	82·8 (78·0-87·0)	86·0 (77·6-92·0)	42·5 (38·0-47·6)	37·6 (33·4-42·1)	37·0 (32·4-42·1)	31·8 (25·2-40·3)	53·9 (52·3-55·4)	59·5 (56·5-62·3)	59·7 (56·6-62·7)	63·2 (58·3-67·6)
**Andean Latin America**																
Bolivia	30·9 (27·3-34·4)	22·5 (18·0-27·5)	22·3 (17·7-27·5)	18·6 (11·7-28·1)	39·9 (37·7-42·2)	48·4 (41·3-55·4)	49·1 (41·3-56·6)	60·5 (44·7-75·6)	45·6 (43·1-48·0)	39·5 (36·2-43·0)	39·6 (36·2-43·1)	35·1 (29·3-42·1)	40·1 (38·3-42·1)	51·7 (48·1-55·2)	52·3 (48·6-56·0)	61·9 (56·5-67·4)
Ecuador	28·5 (26·6-30·8)	25·7 (23·4-28·1)	25·1 (22·7-27·7)	19·4 (14·6-25·4)	72·0 (69·7-74·2)	79·5 (73·9-84·2)	79·6 (74·1-84·5)	82·2 (71·5-89·9)	43·1 (38·7-47·6)	42·4 (37·6-48·0)	42·2 (37·6-47·8)	40·0 (31·0-50·3)	52·3 (50·6-53·7)	60·6 (58·9-62·4)	61·1 (59·4-63·0)	68·4 (66·2-70·7)
Peru	18·1 (16·5-19·8)	14·7 (12·3-17·4)	14·2 (11·5-17·3)	10·4 (5·8-17·0)	59·4 (57·7-60·9)	63·3 (59·1-66·9)	63·6 (58·7-67·9)	69·2 (60·3-78·4)	29·9 (28·4-31·5)	28·8 (27·4-30·3)	28·3 (26·8-29·9)	27·1 (23·1-31·2)	52·9 (51·1-54·7)	65·4 (62·3-68·3)	65·9 (62·6-69·0)	74·5 (69·5-79·1)
**Caribbean**																
Antigua and Barbuda	8·9 (7·9-9·8)	8·6 (7·2-10·3)	8·5 (7·0-10·3)	7·9 (5·0-11·7)	79·3 (73·3-84·2)	83·1 (78·2-87·2)	83·4 (78·4-87·3)	86·1 (76·7-92·2)	24·3 (19·5-29·3)	18·3 (14·5-22·8)	17·5 (13·7-21·8)	12·4 (7·0-20·3)	58·2 (56·3-60·1)	62·2 (60·0-64·6)	62·6 (60·3-65·0)	65·9 (62·8-68·9)
The Bahamas	19·4 (17·2-21·6)	15·7 (13·6-18·1)	15·6 (13·3-18·2)	14·2 (9·5-20·8)	81·0 (75·8-85·3)	83·6 (78·8-87·4)	83·7 (79·1-87·7)	85·2 (75·8-92·0)	26·9 (25·3-28·6)	18·2 (15·5-21·4)	16·9 (14·3-20·1)	12·2 (8·5-16·7)	56·3 (54·5-58·1)	60·5 (57·9-63·1)	60·5 (57·8-63·1)	62·6 (57·6-66·9)
Barbados	11·1 (9·9-12·2)	10·2 (8·9-11·7)	10·2 (8·8-11·7)	9·9 (7·2-13·6)	78·9 (73·4-84·0)	81·8 (76·1-86·2)	81·9 (76·2-86·3)	83·5 (73·1-90·2)	21·5 (18·5-24·8)	16·6 (14·1-19·3)	15·4 (13·0-18·2)	14·0 (9·2-20·1)	58·4 (56·2-60·3)	62·6 (60·0-65·0)	62·6 (60·0-65·1)	63·9 (59·6-67·9)
Belize	29·6 (26·5-32·4)	23·3 (19·9-27·6)	23·1 (19·2-27·9)	18·8 (11·3-29·3)	71·3 (64·7-76·9)	72·9 (67·6-78·0)	73·5 (67·9-78·8)	83·9 (71·4-92·3)	49·6 (41·8-57·8)	39·3 (31·9-47·7)	38·9 (31·4-47·5)	32·8 (20·9-48·0)	47·8 (46·0-49·6)	55·3 (52·3-57·9)	55·6 (52·4-58·3)	60·5 (55·4-65·0)
Cuba	15·1 (14·1-16·0)	8·0 (7·1-8·9)	7·7 (6·5-8·9)	5·1 (3·5-7·4)	85·6 (81·6-88·9)	88·4 (86·7-90·0)	88·9 (86·9-90·7)	90·9 (85·5-94·9)	27·0 (26·4-27·6)	23·2 (18·5-28·5)	22·0 (16·7-28·2)	15·1 (8·9-26·2)	60·3 (59·2-61·4)	67·1 (65·5-68·6)	67·5 (65·8-69·0)	72·5 (70·2-74·6)
Dominica	15·6 (14·2-17·1)	17·3 (15·0-20·1)	17·3 (14·6-20·4)	18·1 (11·9-26·1)	74·5 (68·5-80·2)	80·2 (74·5-84·7)	80·5 (75·0-85·0)	83·7 (73·5-91·0)	13·9 (12·7-15·0)	10·2 (9·0-11·4)	9·8 (8·5-11·2)	7·2 (5·0-10·0)	56·6 (54·8-58·3)	56·4 (54·0-58·7)	56·6 (54·1-59·0)	56·2 (52·3-59·9)
Dominican Republic	24·2 (21·4-26·6)	20·2 (16·7-23·7)	20·1 (16·5-23·9)	18·4 (12·3-26·9)	79·1 (77·4-80·6)	83·2 (81·5-84·8)	83·7 (81·6-85·7)	91·1 (86·7-94·4)	53·4 (46·9-60·3)	41·8 (33·6-52·9)	41·1 (32·0-52·9)	31·6 (17·9-53·0)	54·8 (52·7-56·8)	61·5 (58·9-64·4)	61·9 (59·3-64·9)	67·5 (64·2-71·3)
Grenada	13·8 (11·9-15·8)	16·4 (13·5-19·6)	15·6 (12·6-18·8)	17·9 (10·6-27·8)	70·6 (63·8-76·8)	79·8 (74·3-84·7)	80·2 (74·8-84·7)	85·4 (76·4-91·4)	27·7 (24·1-31·8)	12·9 (10·9-15·1)	11·9 (10·1-14·0)	5·3 (3·5-7·5)	52·9 (51·0-54·9)	54·5 (51·9-57·0)	54·9 (52·2-57·4)	56·7 (53·1-60·4)

Location	Indicator 3.9.1: Age-standardised death rate attributable to household air pollution and ambient air pollution (per 100,000 population)				Indicator 3.9.2: Age-standardised death rate attributable to unsafe water, sanitation, and hygiene (WaSH) (per 100,000 population)				Indicator 3.9.3: Age-standardised death rate due to unintentional poisonings (per 100,000 population)				Indicator 3.a.1: Age-standardised prevalence of daily smoking in populations aged 10 and older (%)			
	**2000**	**2015**	**2016**	**2030**	**2000**	**2015**	**2016**	**2030**	**2000**	**2015**	**2016**	**2030**	**2000**	**2015**	**2016**	**2030**
Azerbaijan	177·0 (146·6-218·4)	114·2 (86·9-155·2)	110·2 (83·5-150·4)	66·5 (48·2-97·0)	11·4 (8·4-15·2)	2·4 (1·4-3·9)	2·2 (1·2-3·6)	0·8 (0·4-1·5)	1·2 (1·0-1·7)	0·8 (0·5-1·4)	0·8 (0·5-1·3)	0·4 (0·2-1·1)	18·7 (16·7-20·8)	18·6 (16·7-20·6)	18·5 (16·4-20·6)	18·2 (14·6-22·5)
Georgia	145·2 (121·4-171·4)	111·6 (77·2-147·7)	107·7 (73·3-144·0)	78·6 (41·1-125·4)	3·1 (2·2-4·0)	0·6 (0·4-0·8)	0·6 (0·4-0·8)	0·3 (0·2-0·5)	0·8 (0·7-1·4)	0·8 (0·6-1·1)	0·8 (0·6-1·1)	0·7 (0·3-1·3)	21·1 (19·3-23·0)	22·8 (20·8-25·0)	22·8 (20·6-25·2)	23·5 (18·9-28·7)
Kazakhstan	146·2 (117·8-179·5)	92·6 (69·4-126·2)	89·3 (66·4-122·7)	53·1 (35·1-81·4)	5·2 (3·7-7·0)	0·8 (0·5-1·2)	0·7 (0·5-1·1)	0·2 (0·1-0·4)	4·1 (3·2-4·8)	2·5 (1·8-3·0)	2·4 (1·7-3·0)	1·5 (0·7-2·6)	19·4 (17·8-21·0)	19·4 (17·8-21·1)	19·2 (17·4-21·0)	16·1 (12·7-19·9)
Kyrgyzstan	194·7 (155·3-239·3)	127·5 (102·4-154·7)	121·7 (97·3-148·3)	68·0 (50·1-88·5)	9·3 (7·2-11·4)	2·9 (2·1-3·8)	2·7 (2·0-3·6)	1·1 (0·7-1·7)	1·6 (1·3-1·9)	0·8 (0·7-1·3)	0·8 (0·6-1·3)	0·5 (0·3-1·0)	16·2 (15·1-17·3)	19·0 (17·2-20·7)	18·9 (17·0-20·7)	20·6 (16·4-25·0)
Mongolia	255·4 (224·4-286·6)	170·4 (139·1-203·9)	165·9 (134·8-199·3)	113·6 (86·2-144·6)	5·6 (3·5-8·0)	1·3 (0·8-2·1)	1·2 (0·7-2·0)	0·4 (0·2-0·7)	9·4 (4·5-12·0)	2·4 (1·9-3·9)	2·4 (1·8-3·8)	1·4 (0·6-3·6)	23·3 (22·0-24·7)	22·8 (20·5-25·3)	23·0 (20·5-25·5)	22·9 (18·5-27·7)
Tajikistan	297·3 (259·1-338·0)	168·4 (136·0-205·5)	164·4 (132·1-201·3)	116·0 (86·6-151·8)	39·9 (29·6-50·4)	12·2 (7·8-18·5)	11·6 (7·3-17·9)	5·3 (2·6-10·2)	2·3 (1·5-2·8)	1·4 (0·9-2·1)	1·4 (0·8-2·1)	1·0 (0·4-2·2)	4·3 (3·3-5·3)	3·3 (2·6-4·1)	3·3 (2·6-4·1)	2·4 (1·4-3·9)
Turkmenistan	149·2 (130·3-167·9)	101·6 (87·7-114·7)	100·8 (87·1-114·0)	90·8 (79·3-102·6)	21·7 (15·2-29·8)	2·8 (1·7-4·2)	2·4 (1·5-3·8)	0·4 (0·2-0·8)	1·4 (1·2-1·8)	0·8 (0·6-1·1)	0·8 (0·6-1·0)	0·4 (0·2-0·6)	8·7 (7·1-10·6)	7·7 (6·2-9·3)	7·7 (6·2-9·3)	6·9 (4·6-9·7)
Uzbekistan	188·6 (157·6-226·1)	130·7 (106·1-172·2)	128·8 (104·3-170·2)	103·5 (80·0-144·8)	5·4 (3·7-7·5)	1·5 (0·8-2·4)	1·4 (0·7-2·2)	0·4 (0·2-0·8)	1·5 (1·3-1·7)	1·2 (0·9-1·5)	1·2 (0·8-1·5)	1·0 (0·5-1·6)	11·6 (10·2-12·9)	8·2 (7·4-9·1)	8·3 (7·3-9·2)	6·3 (4·8-8·1)
**Central Latin America**																
Colombia	62·9 (54·4-71·7)	39·2 (31·8-47·7)	37·3 (30·1-45·7)	22·7 (16·4-31·2)	6·7 (4·8-8·3)	2·0 (1·3-2·6)	1·9 (1·3-2·5)	0·8 (0·4-1·1)	0·4 (0·4-0·7)	0·2 (0·2-0·4)	0·2 (0·2-0·4)	0·1 (0·1-0·3)	13·4 (11·7-15·2)	9·9 (8·7-11·2)	9·9 (8·6-11·2)	8·1 (6·0-10·4)
Costa Rica	41·3 (35·8-47·3)	21·4 (17·8-25·2)	21·4 (17·8-25·2)	11·9 (7·8-19·1)	3·4 (2·3-4·5)	1·5 (0·9-2·1)	1·5 (0·9-2·1)	0·8 (0·3-1·4)	0·3 (0·3-0·4)	0·2 (0·2-0·3)	0·2 (0·2-0·3)	0·2 (0·1-0·3)	11·7 (10·8-12·7)	7·3 (6·2-8·5)	7·3 (6·2-8·4)	6·0 (3·9-8·6)
El Salvador	89·7 (79·8-100·0)	49·9 (41·5-59·4)	48·8 (40·3-58·3)	31·4 (22·4-41·3)	17·7 (13·3-24·0)	6·6 (4·4-10·3)	6·3 (4·2-10·0)	2·4 (1·0-4·3)	0·4 (0·3-0·5)	0·2 (0·1-0·2)	0·2 (0·1-0·2)	0·1 (0·1-0·2)	8·0 (6·9-9·3)	7·0 (6·0-8·1)	7·0 (5·9-8·1)	6·2 (4·6-8·2)
Guatemala	151·6 (129·7-174·6)	83·1 (64·2-105·0)	80·4 (61·4-102·7)	49·6 (31·3-74·5)	58·3 (48·1-67·4)	22·8 (17·0-28·4)	21·6 (15·9-27·2)	9·0 (5·8-12·8)	1·4 (1·2-2·0)	1·4 (0·9-1·8)	1·4 (0·9-1·8)	1·0 (0·4-2·1)	5·3 (4·7-6·1)	5·4 (4·6-6·4)	5·4 (4·5-6·3)	5·0 (3·6-7·0)
Honduras	145·8 (116·6-179·9)	102·0 (78·7-129·3)	99·3 (76·6-126·0)	68·0 (51·8-87·4)	36·6 (22·5-53·7)	15·8 (8·1-27·6)	15·1 (7·7-26·6)	7·4 (3·0-14·8)	1·1 (0·6-1·7)	0·8 (0·4-1·3)	0·8 (0·4-1·3)	0·5 (0·2-1·2)	11·5 (10·5-12·7)	9·4 (8·3-10·6)	9·4 (8·3-10·7)	8·0 (6·1-10·2)
Mexico	48·8 (43·5-54·0)	33·8 (29·7-38·4)	32·9 (28·9-37·5)	22·9 (19·5-26·8)	7·7 (5·8-9·4)	3·1 (2·1-4·0)	3·0 (2·0-3·9)	1·3 (0·7-2·0)	0·8 (0·7-1·1)	0·5 (0·4-0·7)	0·4 (0·4-0·7)	0·3 (0·2-0·5)	16·1 (15·7-16·6)	11·5 (11·1-11·8)	11·2 (10·8-11·5)	8·2 (7·5-8·8)
Nicaragua	76·0 (68·2-83·6)	51·6 (40·2-63·2)	50·0 (38·4-61·7)	31·9 (20·6-44·4)	16·7 (13·1-21·1)	5·1 (3·4-8·0)	4·9 (3·2-7·7)	2·0 (0·9-3·6)	0·7 (0·6-0·9)	0·4 (0·3-0·5)	0·4 (0·3-0·5)	0·3 (0·1-0·6)	10·5 (9·0-12·0)	9·9 (8·5-11·5)	9·9 (8·5-11·5)	9·4 (7·0-12·4)
Panama	42·5 (36·3-49·3)	27·1 (21·6-34·6)	26·6 (21·0-34·0)	19·4 (14·4-26·5)	5·5 (4·3-6·7)	4·4 (3·1-5·7)	4·2 (2·9-5·5)	2·1 (1·2-3·2)	0·7 (0·6-0·9)	0·4 (0·3-0·6)	0·4 (0·3-0·6)	0·2 (0·1-0·5)	7·0 (6·2-7·8)	3·2 (2·9-3·6)	3·3 (2·9-3·7)	1·8 (1·3-2·5)
Venezuela	53·1 (46·3-60·1)	40·1 (32·5-48·1)	40·3 (32·6-48·5)	29·0 (18·0-39·1)	7·8 (4·9-10·3)	3·5 (2·2-4·9)	3·4 (2·1-4·8)	2·3 (1·2-3·7)	0·5 (0·4-0·6)	0·3 (0·3-0·4)	0·3 (0·3-0·4)	0·2 (0·1-0·4)	15·1 (13·3-17·0)	10·7 (9·3-12·4)	10·5 (9·1-12·3)	8·2 (6·0-10·9)
**Andean Latin America**																
Bolivia	130·6 (114·3-146·7)	73·8 (58·1-92·2)	70·3 (54·7-88·6)	37·1 (24·9-52·4)	28·6 (19·6-39·5)	9·2 (5·8-13·9)	8·5 (5·4-13·0)	3·0 (1·8-4·7)	2·0 (1·3-3·1)	1·0 (0·7-1·4)	1·0 (0·7-1·4)	0·6 (0·3-1·1)	9·4 (8·4-10·3)	8·6 (7·8-9·6)	8·6 (7·8-9·5)	8·1 (6·7-9·8)
Ecuador	46·3 (39·9-53·0)	22·6 (18·4-27·8)	21·8 (17·7-26·9)	12·6 (9·8-16·3)	10·2 (7·5-12·6)	3·3 (2·4-4·2)	3·2 (2·3-4·0)	1·1 (0·7-1·7)	0·9 (0·8-1·1)	0·5 (0·5-0·7)	0·5 (0·4-0·6)	0·3 (0·2-0·5)	7·4 (7·1-7·8)	4·7 (4·3-5·1)	4·7 (4·3-5·2)	3·5 (2·7-4·5)
Peru	88·2 (77·1-100·1)	53·0 (41·2-65·9)	50·7 (38·8-63·7)	27·1 (17·1-39·3)	13·3 (9·7-17·4)	6·1 (4·1-8·8)	5·8 (3·8-8·4)	2·8 (1·7-4·4)	1·3 (1·1-1·6)	0·6 (0·5-0·8)	0·6 (0·5-0·8)	0·3 (0·2-0·6)	7·7 (7·0-8·5)	7·0 (6·3-7·7)	6·9 (6·3-7·7)	6·5 (5·3-7·9)
**Caribbean**																
Antigua and Barbuda	35·6 (24·8-46·7)	25·2 (16·8-34·5)	25·3 (16·9-34·6)	16·9 (8·7-25·7)	4·3 (3·2-5·4)	2·9 (2·0-3·8)	2·8 (2·0-3·8)	2·0 (1·3-2·9)	0·5 (0·5-0·8)	0·4 (0·3-0·6)	0·4 (0·3-0·6)	0·3 (0·2-0·5)	2·2 (1·9-2·6)	2·2 (1·9-2·6)	2·2 (1·9-2·6)	2·1 (1·6-2·8)
The Bahamas	44·2 (33·2-56·3)	29·7 (21·4-40·0)	29·9 (21·6-40·3)	22·9 (12·5-36·1)	3·3 (2·3-4·3)	2·5 (1·8-3·4)	2·5 (1·8-3·4)	2·1 (1·3-3·3)	0·3 (0·3-0·5)	0·3 (0·2-0·4)	0·3 (0·2-0·4)	0·2 (0·1-0·4)	7·1 (6·1-8·2)	5·2 (4·5-6·0)	5·2 (4·5-6·0)	4·6 (3·5-5·9)
Barbados	32·3 (20·6-45·7)	24·7 (14·7-35·1)	25·4 (15·2-35·8)	21·5 (9·4-39·8)	3·3 (2·4-4·3)	2·7 (1·9-3·6)	2·7 (1·8-3·6)	2·1 (1·2-3·1)	0·3 (0·2-0·5)	0·2 (0·2-0·4)	0·2 (0·2-0·4)	0·2 (0·1-0·4)	5·6 (4·7-6·5)	4·4 (3·8-5·1)	4·4 (3·8-5·1)	3·7 (2·7-5·0)
Belize	104·3 (90·5-118·5)	65·0 (53·1-77·9)	64·2 (52·1-77·2)	53·2 (40·0-68·2)	11·4 (9·1-13·9)	7·4 (5·6-9·3)	7·2 (5·5-9·2)	5·2 (3·6-7·4)	1·3 (1·1-1·8)	0·8 (0·7-1·4)	0·8 (0·7-1·4)	0·7 (0·4-1·4)	7·2 (6·3-8·0)	6·7 (5·9-7·7)	6·7 (5·9-7·7)	6·5 (5·0-8·2)
Cuba	54·7 (46·5-63·6)	34·6 (28·8-41·0)	34·3 (28·5-40·7)	30·4 (22·4-37·2)	4·5 (3·4-5·6)	2·6 (1·8-3·3)	2·5 (1·8-3·2)	1·8 (1·2-2·5)	0·4 (0·3-0·4)	0·2 (0·2-0·3)	0·2 (0·2-0·3)	0·2 (0·1-0·2)	23·8 (21·5-26·2)	15·3 (13·8-17·1)	15·1 (13·6-16·9)	10·0 (7·8-12·6)
Dominica	54·0 (42·2-66·7)	36·4 (26·8-46·8)	36·3 (26·6-46·7)	22·3 (12·2-32·7)	5·3 (4·1-6·7)	3·6 (2·6-4·7)	3·6 (2·6-4·7)	2·8 (1·9-3·9)	1·9 (1·6-2·8)	1·4 (1·1-2·3)	1·4 (1·1-2·3)	1·2 (0·6-2·3)	5·2 (4·4-6·0)	4·3 (3·7-5·1)	4·3 (3·7-5·1)	3·9 (2·9-5·2)
Dominican Republic	61·2 (51·9-70·6)	46·5 (38·0-55·5)	46·0 (37·6-55·0)	39·1 (31·2-47·6)	13·8 (10·1-19·0)	7·4 (5·2-10·7)	7·1 (5·0-10·4)	4·0 (2·6-6·0)	0·4 (0·4-0·7)	0·3 (0·2-0·5)	0·3 (0·2-0·5)	0·2 (0·1-0·4)	14·5 (13·6-15·5)	8·5 (7·5-9·7)	8·5 (7·5-9·7)	6·4 (4·5-9·0)
Grenada	67·6 (48·0-87·8)	48·4 (31·7-67·1)	49·0 (32·1-67·9)	28·5 (11·9-47·0)	6·3 (4·5-8·2)	4·5 (3·2-6·2)	4·5 (3·1-6·2)	3·3 (2·0-4·9)	1·0 (0·8-1·3)	0·6 (0·4-1·0)	0·6 (0·4-0·9)	0·3 (0·2-0·7)	8·0 (6·9-9·1)	7·7 (6·7-8·6)	7·7 (6·7-8·6)	7·3 (5·6-9·1)

Location	Indicator 3.b.1: Geometric mean of the coverage of eight vaccines, conditional on inclusion in national vaccine schedules, in target populations (%)				Indicator 5.2.1: Age-standardised prevalence of women aged 15 years and older who experienced physical or sexual violence by an intimate partner in the last 12 months (%)				Indicator 6.1.1: Risk-weighted prevalence of populations using unsafe or unimproved water sources, as measured by the summary exposure value (SEV) for unsafe water (%)				Indicator 6.2.1a: Risk-weighted prevalence of populations using unsafe or unimproved sanitation, as measured by the summary exposure value (SEV) for unsafe sanitation (%)			
	2000	2015	2016	2030	2000	2015	2016	2030	2000	2015	2016	2030	2000	2015	2016	2030
Azerbaijan	51·4<br>(47·2-55·1)	83·2<br>(75·2-89·1)	80·5<br>(72·1-86·5)	95·9<br>(88·4-99·2)	25·6<br>(22·5-29·0)	21·4<br>(18·9-24·2)	21·1<br>(18·6-23·9)	17·4<br>(14·9-20·0)	15·0<br>(5·4-22·1)	9·9<br>(3·1-15·0)	9·6<br>(3·0-14·5)	6·3<br>(1·6-13·1)	64·5<br>(48·2-78·7)	36·9<br>(16·0-65·8)	36·2<br>(15·3-65·9)	26·5<br>(7·2-64·5)
Georgia	80·0<br>(76·7-83·1)	87·3<br>(83·2-90·6)	87·1<br>(82·3-90·9)	94·6<br>(86·2-98·7)	19·1<br>(17·0-21·5)	17·6<br>(15·6-19·8)	17·4<br>(15·4-19·6)	15·7<br>(13·8-17·7)	17·9<br>(12·2-29·3)	12·7<br>(8·2-23·9)	12·4<br>(7·9-23·6)	9·1<br>(5·5-19·3)	43·2<br>(21·8-66·4)	41·2<br>(22·7-67·3)	41·0<br>(22·3-67·2)	37·6<br>(16·9-64·1)
Kazakhstan	94·6<br>(92·6-96·2)	92·8<br>(89·4-94·9)	93·8<br>(90·0-96·3)	98·8<br>(94·9-100·0)	25·2<br>(22·3-28·3)	22·3<br>(19·7-25·0)	22·0<br>(19·4-24·7)	18·2<br>(15·9-20·8)	10·9<br>(3·8-16·5)	9·0<br>(2·9-14·4)	8·8<br>(2·7-14·2)	6·3<br>(1·5-11·2)	47·6<br>(30·2-64·3)	37·1<br>(18·3-61·8)	36·5<br>(17·7-61·2)	27·8<br>(10·8-50·8)
Kyrgyzstan	85·3<br>(82·5-87·8)	88·4<br>(84·2-91·8)	88·6<br>(83·8-92·3)	91·5<br>(76·9-98·3)	32·1<br>(28·2-36·0)	30·8<br>(27·3-34·3)	30·5<br>(27·0-34·0)	29·4<br>(26·3-32·6)	19·4<br>(10·0-27·6)	13·7<br>(6·0-21·8)	13·4<br>(5·8-21·4)	9·6<br>(3·8-16·8)	62·2<br>(41·6-86·5)	63·2<br>(38·0-98·6)	63·0<br>(37·6-98·6)	59·4<br>(33·2-98·9)
Mongolia	90·0<br>(87·9-92·0)	96·1<br>(93·9-97·6)	96·4<br>(94·0-97·9)	97·9<br>(91·9-100·0)	31·2<br>(27·2-35·6)	27·8<br>(24·3-31·4)	27·5<br>(24·1-31·1)	24·1<br>(20·9-27·6)	15·4<br>(0·0-22·4)	19·8<br>(4·0-27·0)	19·8<br>(4·2-27·0)	19·7<br>(3·6-38·5)	73·8<br>(57·4-88·6)	59·7<br>(36·5-88·0)	59·2<br>(35·8-87·3)	49·4<br>(24·9-74·6)
Tajikistan	86·4<br>(83·9-88·6)	92·3<br>(89·0-94·8)	92·6<br>(89·1-95·3)	96·4<br>(90·4-99·3)	35·1<br>(31·0-39·5)	30·7<br>(27·2-34·6)	30·6<br>(27·1-34·4)	28·2<br>(24·8-31·8)	16·1<br>(1·1-23·4)	12·5<br>(0·1-18·4)	12·4<br>(0·1-18·4)	11·3<br>(0·0-18·5)	63·0<br>(38·4-93·9)	60·2<br>(34·3-96·1)	60·3<br>(34·1-96·2)	61·8<br>(31·6-97·1)
Turkmenistan	92·9<br>(91·3-94·4)	91·7<br>(87·8-94·6)	92·3<br>(88·2-95·2)	93·7<br>(84·8-98·0)	28·3<br>(24·6-32·4)	23·7<br>(20·5-27·0)	23·3<br>(20·2-26·6)	19·3<br>(16·7-22·0)	19·3<br>(9·5-27·1)	12·7<br>(6·0-19·5)	12·3<br>(5·8-19·0)	7·2<br>(3·0-12·1)	68·2<br>(43·2-86·8)	52·1<br>(30·6-73·1)	51·0<br>(29·4-72·2)	35·2<br>(15·8-57·9)
Uzbekistan	79·1<br>(68·7-87·8)	99·5<br>(99·0-99·8)	99·6<br>(99·1-99·8)	100·0<br>(99·9-100·0)	27·8<br>(24·3-31·7)	24·0<br>(21·0-27·4)	23·7<br>(20·7-27·1)	20·2<br>(17·4-23·0)	7·2<br>(0·0-13·2)	5·4<br>(0·0-10·5)	5·3<br>(0·0-10·2)	3·8<br>(0·0-9·9)	63·6<br>(39·2-93·6)	53·4<br>(28·8-86·5)	52·6<br>(28·2-85·6)	43·3<br>(20·8-76·3)
**Central Latin America**																
Colombia	83·8<br>(81·3-86·0)	95·9<br>(94·2-97·1)	96·5<br>(94·8-97·6)	99·2<br>(98·4-99·6)	36·4<br>(32·3-40·8)	29·3<br>(26·3-32·4)	28·9<br>(25·9-31·9)	23·4<br>(20·9-26·0)	14·5<br>(9·0-18·4)	10·1<br>(6·2-12·8)	9·7<br>(6·0-12·4)	5·7<br>(3·7-7·6)	24·6<br>(15·9-34·8)	8·6<br>(2·5-23·2)	8·3<br>(2·2-23·9)	4·7<br>(0·3-26·7)
Costa Rica	95·3<br>(93·9-96·4)	97·1<br>(95·9-98·1)	97·4<br>(96·2-98·3)	98·9<br>(97·7-99·6)	26·6<br>(23·5-30·0)	23·6<br>(20·8-26·4)	23·3<br>(20·6-26·0)	20·9<br>(18·4-23·3)	10·9<br>(6·4-14·6)	6·4<br>(3·9-9·1)	6·2<br>(3·8-8·8)	3·8<br>(2·1-5·6)	10·5<br>(2·8-24·9)	4·9<br>(0·9-14·5)	4·8<br>(0·9-14·0)	2·2<br>(0·3-6·7)
El Salvador	94·4<br>(92·9-95·7)	93·1<br>(90·4-95·3)	93·5<br>(90·6-95·8)	95·0<br>(89·4-98·4)	32·2<br>(28·6-35·9)	27·6<br>(24·4-31·0)	27·3<br>(24·2-30·7)	23·5<br>(20·6-26·6)	21·0<br>(14·7-25·2)	16·1<br>(11·2-19·8)	15·8<br>(11·0-19·5)	12·2<br>(8·4-15·3)	53·5<br>(40·4-66·5)	46·3<br>(32·6-59·6)	46·1<br>(31·6-60·1)	40·6<br>(18·1-62·8)
Guatemala	95·2<br>(93·8-96·4)	94·1<br>(91·7-95·8)	95·2<br>(93·1-96·8)	93·2<br>(85·7-97·3)	35·1<br>(31·1-39·1)	31·8<br>(27·9-35·4)	31·5<br>(27·7-35·2)	28·5<br>(24·7-32·1)	21·8<br>(8·8-27·3)	15·9<br>(6·8-20·2)	15·6<br>(6·7-19·8)	11·7<br>(4·7-23·5)	54·4<br>(48·6-60·2)	43·7<br>(32·2-56·2)	42·8<br>(30·8-56·1)	31·6<br>(15·3-53·0)
Honduras	94·4<br>(93·3-95·4)	95·4<br>(93·7-96·7)	95·5<br>(93·8-96·9)	96·8<br>(93·3-99·0)	36·3<br>(32·1-40·4)	31·8<br>(28·0-35·5)	31·5<br>(27·7-35·1)	27·3<br>(23·8-30·8)	22·6<br>(15·4-27·2)	18·9<br>(13·3-22·7)	18·6<br>(13·1-22·4)	14·6<br>(10·0-17·8)	56·2<br>(42·9-69·5)	36·3<br>(15·2-57·7)	35·5<br>(13·3-58·6)	22·7<br>(1·4-58·6)
Mexico	88·3<br>(85·7-90·6)	84·0<br>(79·3-88·3)	84·9<br>(79·7-89·5)	88·8<br>(76·1-96·7)	22·2<br>(19·5-25·2)	20·1<br>(17·8-22·6)	20·0<br>(17·7-22·4)	17·9<br>(15·9-20·2)	10·3<br>(6·1-12·1)	6·5<br>(3·5-7·7)	6·3<br>(3·4-7·5)	4·2<br>(2·1-5·1)	24·1<br>(20·8-28·9)	12·2<br>(9·2-16·5)	12·1<br>(9·0-16·3)	7·0<br>(4·2-10·9)
Nicaragua	93·7<br>(91·5-95·3)	98·6<br>(97·5-99·3)	98·7<br>(97·7-99·4)	99·8<br>(99·1-100·0)	32·1<br>(28·6-36·1)	27·2<br>(24·0-30·6)	26·9<br>(23·8-30·4)	23·7<br>(20·8-27·0)	25·6<br>(19·9-29·6)	21·3<br>(16·4-24·7)	21·0<br>(16·1-24·3)	17·1<br>(12·6-20·1)	73·1<br>(65·9-79·6)	51·0<br>(21·4-77·6)	49·6<br>(18·9-78·1)	29·7<br>(2·1-74·2)
Panama	88·8<br>(87·0-90·5)	81·9<br>(76·4-86·8)	83·7<br>(78·0-88·3)	82·8<br>(69·8-91·8)	25·6<br>(22·5-29·1)	23·1<br>(20·3-26·1)	22·7<br>(20·0-25·7)	20·8<br>(18·4-23·4)	13·1<br>(9·1-16·8)	10·4<br>(7·1-13·2)	10·2<br>(7·0-12·9)	7·5<br>(4·9-9·7)	46·2<br>(37·4-54·5)	24·9<br>(13·7-37·2)	23·5<br>(11·9-36·7)	9·8<br>(1·3-27·1)
Venezuela	74·9<br>(72·1-77·3)	77·0<br>(72·3-81·2)	77·6<br>(72·4-82·1)	85·3<br>(75·4-92·8)	29·0<br>(25·4-33·1)	25·9<br>(22·7-29·4)	25·6<br>(22·5-29·0)	21·6<br>(19·0-24·6)	10·4<br>(5·7-13·9)	6·7<br>(3·5-9·0)	6·5<br>(3·3-8·7)	4·0<br>(1·9-5·4)	12·4<br>(6·1-22·0)	10·0<br>(2·7-27·4)	9·8<br>(2·6-27·2)	7·5<br>(1·8-23·7)
**Andean Latin America**																
Bolivia	91·2<br>(89·6-92·6)	95·8<br>(93·8-97·3)	96·2<br>(94·3-97·7)	99·3<br>(97·8-99·9)	37·4<br>(32·9-42·2)	32·7<br>(28·7-36·9)	32·5<br>(28·5-36·7)	29·3<br>(25·6-33·1)	16·6<br>(7·0-22·1)	9·0<br>(2·9-13·9)	8·6<br>(2·7-13·5)	4·7<br>(0·7-13·1)	34·4<br>(12·8-60·6)	18·3<br>(4·1-48·0)	17·5<br>(3·9-46·7)	8·7<br>(1·8-29·7)
Ecuador	90·4<br>(87·0-93·0)	94·6<br>(91·9-96·7)	95·0<br>(92·2-97·1)	97·6<br>(93·0-99·8)	30·0<br>(26·7-33·5)	27·0<br>(23·8-30·4)	26·7<br>(23·5-30·1)	24·0<br>(21·0-27·0)	12·3<br>(4·7-16·1)	7·2<br>(2·2-11·2)	7·0<br>(2·1-11·0)	4·8<br>(1·6-8·9)	23·4<br>(12·9-37·5)	22·5<br>(9·4-41·2)	22·6<br>(9·4-41·3)	23·4<br>(9·3-43·3)
Peru	92·7<br>(91·2-94·0)	95·3<br>(93·3-96·8)	96·1<br>(94·3-97·4)	99·6<br>(98·9-100·0)	28·0<br>(25·0-31·2)	24·2<br>(21·8-27·0)	24·0<br>(21·5-26·7)	20·7<br>(18·5-23·3)	10·2<br>(1·2-14·4)	6·8<br>(0·7-9·9)	6·6<br>(0·6-9·7)	4·1<br>(0·2-7·2)	40·1<br>(24·0-61·5)	37·2<br>(20·6-62·6)	37·2<br>(20·4-63·3)	36·7<br>(18·4-71·0)
**Caribbean**																
Antigua and Barbuda	98·9<br>(98·1-99·4)	99·6<br>(99·2-99·8)	99·6<br>(99·3-99·8)	100·0<br>(99·8-100·0)	20·8<br>(18·0-23·7)	19·5<br>(17·2-22·1)	19·4<br>(17·0-21·9)	17·1<br>(15·1-19·5)	20·4<br>(11·1-25·2)	18·0<br>(10·4-22·9)	17·9<br>(10·4-22·8)	15·9<br>(5·8-31·6)	24·7<br>(13·7-36·5)	19·9<br>(6·4-44·3)	19·8<br>(6·3-44·4)	17·5<br>(4·0-42·4)
The Bahamas	92·7<br>(90·6-94·4)	96·5<br>(94·9-97·7)	97·2<br>(95·9-98·2)	99·6<br>(99·0-99·9)	24·5<br>(21·4-27·7)	23·0<br>(20·3-25·9)	22·7<br>(20·1-25·6)	20·3<br>(18·0-22·8)	16·2<br>(8·0-20·8)	15·2<br>(8·0-19·7)	15·1<br>(8·0-19·6)	14·3<br>(3·4-35·2)	14·6<br>(6·2-29·0)	16·3<br>(5·5-35·9)	16·4<br>(5·5-36·0)	17·3<br>(5·1-39·8)
Barbados	91·8<br>(88·8-93·9)	88·9<br>(84·3-92·2)	89·3<br>(84·4-92·8)	90·9<br>(77·8-97·4)	20·3<br>(17·8-22·9)	18·8<br>(16·6-21·1)	18·6<br>(16·5-20·9)	16·6<br>(14·8-18·6)	14·0<br>(6·8-19·0)	12·2<br>(5·7-16·9)	12·1<br>(5·6-16·8)	11·0<br>(3·7-22·3)	22·2<br>(5·9-51·6)	18·8<br>(4·2-47·2)	18·6<br>(4·2-46·9)	16·2<br>(3·3-42·3)
Belize	77·7<br>(74·9-80·4)	78·8<br>(73·5-83·5)	79·3<br>(73·4-84·6)	83·0<br>(66·2-93·4)	33·7<br>(29·3-38·4)	30·9<br>(27·0-35·3)	30·7<br>(26·8-35·0)	27·9<br>(24·4-32·0)	34·1<br>(26·5-38·4)	33·3<br>(25·8-37·5)	33·1<br>(25·7-37·3)	30·5<br>(23·1-35·0)	42·9<br>(29·2-55·5)	28·2<br>(13·5-46·9)	27·7<br>(12·4-47·2)	18·6<br>(3·1-44·5)
Cuba	93·6<br>(91·9-95·0)	96·5<br>(94·7-97·8)	96·7<br>(94·7-98·0)	98·5<br>(95·0-99·9)	22·0<br>(19·3-24·9)	18·3<br>(16·2-20·5)	18·0<br>(16·0-20·3)	15·0<br>(13·2-17·0)	23·5<br>(13·5-29·1)	16·6<br>(8·6-21·4)	16·1<br>(8·3-20·9)	11·0<br>(1·4-31·4)	30·1<br>(11·7-56·6)	20·6<br>(10·2-33·7)	19·9<br>(9·4-33·4)	12·0<br>(2·9-26·5)
Dominica	99·1<br>(98·5-99·5)	98·7<br>(97·9-99·3)	98·8<br>(98·1-99·3)	99·5<br>(97·8-100·0)	23·8<br>(20·8-27·0)	22·4<br>(19·6-25·3)	22·3<br>(19·5-25·2)	20·5<br>(17·9-23·3)	24·2<br>(14·4-29·2)	20·9<br>(12·3-25·7)	20·7<br>(12·2-25·4)	17·9<br>(9·8-22·1)	41·1<br>(14·3-72·2)	28·8<br>(8·5-65·3)	28·2<br>(8·4-65·0)	19·0<br>(4·8-53·8)
Dominican Republic	71·0<br>(67·6-74·2)	80·2<br>(75·9-84·0)	72·9<br>(62·8-80·8)	82·9<br>(70·3-91·7)	30·7<br>(27·1-34·4)	27·2<br>(24·1-30·3)	26·9<br>(23·9-30·0)	23·9<br>(21·2-26·7)	34·5<br>(23·4-40·1)	32·5<br>(23·2-37·3)	32·3<br>(23·2-37·1)	29·8<br>(22·6-34·2)	43·9<br>(31·9-54·7)	21·5<br>(11·2-36·3)	20·7<br>(10·6-35·2)	9·0<br>(3·7-17·9)
Grenada	95·5<br>(93·4-97·0)	98·3<br>(97·3-99·0)	98·4<br>(97·4-99·1)	99·3<br>(98·2-99·8)	27·0<br>(23·4-31·0)	24·1<br>(20·9-27·5)	23·9<br>(20·7-27·2)	21·1<br>(18·0-24·5)	25·6<br>(14·2-31·1)	18·9<br>(9·1-24·1)	18·6<br>(8·9-23·8)	14·8<br>(4·4-29·1)	44·1<br>(31·7-55·9)	26·4<br>(8·7-55·1)	26·0<br>(8·2-55·6)	15·4<br>(2·4-49·0)

Location	Indicator 6.2.1b: Risk-weighted prevalence of populations without access to a handwashing facility, as measured by the summary exposure value (SEV) for unsafe hygiene (%)				Indicator 7.1.2: Risk-weighted prevalence of household air pollution, as measured by the summary exposure value (SEV) for household air pollution (%)				Indicator 8.8.1: Age-standardised all-cause disability-adjusted life year (DALY) rates attributable to occupational risks (per 100,000 population)				Indicator 11.6.2: Population-weighted mean levels of fine particulate matter smaller than 2.5 microns in diameter (PM2.5)			
	**2000**	**2015**	**2016**	**2030**	**2000**	**2015**	**2016**	**2030**	**2000**	**2015**	**2016**	**2030**	**2000**	**2015**	**2016**	**2030**
Azerbaijan	24·8 (15·2-35·8)	14·0 (8·6-21·6)	13·5 (8·4-21·1)	8·6 (5·5-14·2)	13·4 (7·2-21·9)	6·1 (1·1-18·2)	6·0 (1·0-18·5)	4·8 (0·4-21·8)	1,046·9 (878·3-1,235·2)	1,006·1 (834·7-1,188·3)	992·3 (821·3-1,174·0)	817·2 (655·0-991·4)	23·9 (21·1-27·9)	32·7 (29·1-37·5)	33·0 (29·4-38·9)	36·5 (26·7-47·6)
Georgia	17·6 (10·2-26·9)	10·8 (6·1-17·0)	10·6 (5·9-16·6)	7·9 (4·1-12·6)	22·9 (13·7-33·6)	19·3 (6·0-34·7)	18·8 (5·4-34·5)	12·1 (1·0-30·8)	1,032·2 (854·4-1,232·1)	1,056·0 (869·5-1,261·0)	1,045·0 (858·4-1,249·3)	902·9 (723·2-1,101·8)	15·7 (14·5-17·2)	20·7 (18·8-22·8)	20·7 (18·8-22·9)	23·4 (21·0-26·3)
Kazakhstan	18·0 (10·9-26·7)	13·0 (7·9-20·7)	12·7 (7·7-20·3)	9·1 (5·4-14·8)	9·5 (4·7-16·6)	5·9 (1·4-14·8)	5·7 (1·2-14·9)	3·4 (0·1-15·5)	1,635·1 (1,407·8-1,872·1)	1,245·2 (1,053·2-1,447·9)	1,224·5 (1,033·4-1,425·8)	962·5 (796·5-1,146·5)	16·6 (15·6-17·9)	19·6 (18·2-21·5)	19·7 (18·4-21·6)	20·3 (16·8-23·0)
Kyrgyzstan	21·2 (13·8-30·4)	13·8 (10·0-18·2)	13·5 (9·8-17·9)	9·6 (6·8-13·1)	22·7 (10·4-36·9)	13·5 (7·0-22·9)	12·9 (6·5-22·3)	6·8 (2·2-15·4)	1,253·0 (1,058·5-1,463·6)	946·0 (785·3-1,118·7)	926·3 (767·1-1,096·7)	683·9 (549·6-833·7)	14·9 (14·0-16·1)	17·8 (16·6-19·4)	17·9 (16·7-19·4)	18·2 (16·8-19·9)
Mongolia	38·1 (27·1-50·5)	31·7 (24·7-39·1)	31·1 (24·1-38·7)	26·7 (18·5-33·7)	37·3 (27·8-47·5)	26·8 (16·3-38·8)	26·1 (15·3-38·4)	17·0 (6·3-31·3)	1,127·1 (944·7-1,311·0)	946·6 (780·8-1,119·0)	930·0 (766·8-1,100·7)	726·1 (591·7-871·6)	19·2 (18·2-20·3)	29·2 (27·2-31·5)	29·7 (27·7-32·3)	38·8 (31·5-43·9)
Tajikistan	33·4 (23·5-44·6)	26·4 (18·9-35·0)	26·2 (18·7-34·8)	20·0 (13·6-29·2)	35·9 (26·5-46·8)	18·0 (8·2-30·8)	17·9 (7·7-31·3)	16·0 (2·8-37·0)	1,141·7 (945·6-1,353·5)	864·4 (708·4-1,044·2)	861·5 (705·0-1,042·2)	816·5 (653·4-1,003·7)	62·7 (54·7-78·9)	60·7 (52·7-76·2)	61·4 (53·4-75·3)	63·0 (42·5-89·5)
Turkmenistan	27·5 (17·5-41·3)	15·6 (9·2-23·8)	15·1 (8·9-22·9)	8·6 (4·7-13·0)	0·6 (0·1-2·5)	0·2 (0·0-0·7)	0·2 (0·0-0·6)	0·0 (0·0-0·2)	1,320·2 (1,115·3-1,542·2)	1,075·6 (884·8-1,271·2)	1,067·5 (878·9-1,261·9)	959·1 (793·6-1,136·7)	35·0 (32·1-40·2)	37·3 (33·9-43·7)	37·7 (34·5-43·8)	38·5 (30·0-49·0)
Uzbekistan	25·5 (15·4-36·7)	15·8 (9·4-24·4)	15·3 (9·1-23·6)	9·3 (5·2-15·0)	11·3 (4·5-21·7)	6·3 (1·2-18·3)	6·1 (1·1-18·4)	3·5 (0·2-17·9)	1,163·5 (979·8-1,371·1)	949·2 (777·6-1,140·1)	939·3 (769·9-1,128·1)	810·6 (667·2-973·9)	40·2 (37·3-45·2)	46·2 (43·0-51·4)	46·7 (43·4-52·5)	38·4 (32·9-44·4)
**Central Latin America**																
Colombia	28·2 (23·5-32·8)	23·1 (19·0-27·4)	22·7 (18·6-26·9)	18·0 (14·5-21·9)	10·3 (7·5-13·6)	6·6 (3·6-11·1)	6·1 (3·2-10·7)	2·0 (0·5-5·7)	738·7 (635·5-857·2)	581·8 (488·9-684·0)	569·4 (477·9-670·5)	421·6 (347·3-508·1)	20·4 (19·9-20·9)	17·1 (16·7-17·6)	17·1 (16·7-17·6)	16·2 (15·1-17·6)
Costa Rica	26·6 (22·2-31·3)	22·2 (18·7-26·1)	21·9 (18·5-25·8)	18·6 (15·7-21·8)	6·6 (5·1-8·1)	2·7 (1·6-4·2)	2·5 (1·4-4·1)	1·2 (0·3-3·1)	727·4 (621·2-841·8)	619·6 (523·0-726·7)	616·0 (519·2-722·7)	566·4 (464·7-668·9)	22·0 (20·6-23·8)	18·5 (17·4-19·8)	18·5 (17·3-19·9)	18·0 (15·9-19·7)
El Salvador	36·5 (31·1-42·2)	27·5 (22·7-32·2)	27·0 (22·2-31·7)	20·3 (15·6-24·4)	23·1 (17·4-29·9)	7·9 (4·0-14·0)	7·4 (3·6-13·5)	2·7 (0·7-7·2)	539·8 (451·9-638·4)	523·7 (427·0-629·4)	520·2 (423·1-626·2)	472·9 (371·0-586·4)	42·8 (39·2-46·7)	33·6 (31·2-36·5)	33·4 (30·8-36·2)	32·7 (25·1-40·5)
Guatemala	44·0 (37·8-50·3)	34·2 (29·0-39·5)	33·6 (28·5-39·0)	25·3 (21·1-29·9)	38·5 (30·9-46·6)	24·6 (14·4-35·9)	23·4 (13·4-34·8)	11·4 (4·2-21·4)	1,537·5 (1,323·7-1,748·1)	1,197·8 (1,032·7-1,366·7)	1,186·5 (1,021·7-1,355·7)	1,038·1 (882·9-1,199·9)	38·7 (37·4-40·0)	28·7 (27·9-29·6)	28·5 (27·6-29·4)	28·0 (25·2-31·1)
Honduras	28·1 (23·7-32·9)	24·2 (20·5-28·1)	23·8 (20·2-27·7)	20·3 (16·4-23·8)	37·3 (29·4-45·8)	25·3 (18·2-33·4)	24·4 (17·3-32·7)	14·2 (7·7-23·7)	1,137·4 (850·2-1,343·5)	1,154·8 (814·2-1,389·2)	1,151·2 (811·3-1,385·3)	1,102·6 (767·8-1,331·5)	37·3 (35·2-39·7)	29·8 (28·1-31·8)	29·5 (27·8-31·4)	31·4 (28·8-34·9)
Mexico	21·3 (20·4-22·2)	17·6 (16·9-18·5)	17·5 (16·7-18·3)	14·9 (13·2-16·2)	9·5 (7·7-11·5)	5·0 (3·8-6·2)	4·6 (3·5-5·8)	1·6 (1·1-2·1)	905·5 (782·9-1,039·5)	784·6 (661·9-919·6)	779·8 (656·9-915·2)	714·2 (589·2-850·3)	28·9 (28·0-30·0)	18·9 (18·4-19·4)	18·8 (18·4-19·3)	16·5 (13·6-20·7)
Nicaragua	38·7 (33·1-44·5)	28·1 (23·4-33·0)	27·4 (22·8-32·2)	19·4 (15·5-23·3)	38·2 (30·9-46·5)	22·6 (13·5-34·0)	21·6 (12·5-33·2)	10·3 (3·5-21·2)	648·1 (550·4-757·5)	635·7 (528·8-751·0)	632·9 (525·6-748·6)	594·2 (485·4-713·5)	34·3 (29·6-42·3)	23·0 (20·0-27·7)	22·7 (19·8-26·8)	20·9 (16·7-26·4)
Panama	21·6 (17·9-25·9)	17·8 (14·5-21·3)	17·5 (14·3-21·1)	14·2 (11·3-17·2)	10·5 (7·8-13·8)	5·6 (2·8-9·5)	5·2 (2·5-9·2)	1·9 (0·4-5·3)	735·6 (632·2-855·6)	642·6 (540·2-755·7)	632·1 (530·6-744·2)	502·6 (412·9-604·4)	16·3 (14·5-18·8)	14·0 (12·5-16·0)	14·1 (12·6-16·2)	13·2 (9·8-15·7)
Venezuela	22·8 (18·7-27·0)	19·2 (15·6-22·8)	19·0 (15·4-22·6)	16·1 (12·9-19·5)	1·5 (0·6-2·8)	0·4 (0·2-1·0)	0·4 (0·2-0·9)	0·1 (0·0-0·3)	1,684·5 (1,322·0-1,941·2)	1,482·2 (1,178·6-1,719·8)	1,468·5 (1,164·4-1,708·6)	1,289·4 (934·0-1,560·1)	32·6 (30·6-35·2)	26·1 (24·7-27·8)	26·3 (24·4-28·4)	19·4 (17·6-21·2)
**Andean Latin America**																
Bolivia	37·8 (32·4-43·2)	28·0 (23·2-32·5)	27·5 (22·8-31·9)	20·2 (16·1-23·6)	21·5 (17·1-26·3)	10·4 (6·0-16·5)	9·7 (5·3-15·9)	3·5 (0·9-9·0)	1,071·5 (878·7-1,281·8)	999·3 (811·0-1,213·5)	996·3 (807·6-1,211·0)	955·5 (765·2-1,172·3)	30·2 (29·1-31·4)	22·3 (21·5-23·1)	22·0 (21·2-22·9)	24·7 (23·5-26·5)
Ecuador	34·8 (29·7-40·0)	26·1 (21·5-30·7)	25·8 (21·2-30·4)	19·4 (14·6-23·8)	7·9 (5·7-10·6)	2·4 (1·1-4·6)	2·2 (1·0-4·4)	0·8 (0·2-1·9)	1,028·1 (844·8-1,247·8)	948·1 (760·9-1,148·4)	944·5 (756·9-1,146·4)	893·1 (696·7-1,119·6)	18·0 (17·4-18·5)	13·1 (12·8-13·5)	13·2 (12·8-13·6)	13·4 (12·9-14·1)
Peru	37·0 (31·9-42·1)	27·9 (23·4-32·9)	27·3 (22·9-32·3)	20·3 (16·7-24·9)	20·6 (16·5-25·6)	12·8 (9·0-17·5)	12·2 (8·4-16·9)	5·7 (3·1-9·7)	896·5 (738·8-1,065·4)	819·4 (658·1-989·1)	812·4 (650·9-982·3)	719·8 (556·5-897·7)	35·4 (33·4-38·0)	26·1 (24·4-27·8)	26·1 (24·5-27·9)	35·5 (33·1-38·7)
**Caribbean**																
Antigua and Barbuda	29·3 (24·6-34·3)	25·3 (21·1-29·7)	25·0 (20·9-29·4)	21·9 (17·7-25·7)	1·5 (0·7-2·9)	0·7 (0·3-1·4)	0·7 (0·3-1·3)	0·3 (0·1-0·6)	775·5 (631·2-940·1)	668·3 (536·7-806·7)	664·4 (533·5-801·8)	602·7 (479·0-731·5)	17·3 (9·4-29·3)	15·5 (8·8-26·6)	15·7 (8·9-27·5)	15·4 (8·2-27·6)
The Bahamas	21·8 (18·3-26·3)	21·3 (17·6-26·0)	21·3 (17·6-26·0)	20·9 (16·9-25·7)	2·3 (1·0-4·4)	1·4 (0·6-2·7)	1·3 (0·6-2·6)	0·8 (0·3-1·5)	665·9 (550·8-796·1)	610·0 (491·3-739·5)	613·6 (493·5-742·8)	664·6 (528·7-806·1)	18·2 (11·6-29·2)	12·9 (8·1-20·0)	12·8 (8·1-20·5)	11·3 (7·1-17·9)
Barbados	25·2 (21·3-29·5)	24·2 (20·5-28·0)	24·2 (20·4-28·0)	23·2 (19·6-26·7)	0·1 (0·0-0·2)	0·0 (0·0-0·1)	0·0 (0·0-0·1)	0·0 (0·0-0·0)	609·0 (487·9-762·6)	614·0 (486·0-761·8)	619·0 (490·6-767·9)	683·5 (541·7-847·2)	20·7 (10·1-38·4)	18·1 (9·4-31·9)	18·4 (8·7-32·5)	18·1 (7·6-35·9)
Belize	32·5 (27·8-37·3)	33·2 (28·9-38·0)	32·9 (28·6-37·7)	34·5 (25·5-40·2)	10·0 (7·1-13·6)	6·7 (4·2-10·1)	6·5 (3·9-10·0)	3·9 (1·4-8·6)	1,100·5 (862·2-1,445·1)	993·0 (770·4-1,301·7)	995·0 (771·7-1,298·4)	1,023·0 (796·8-1,239·2)	29·5 (26·6-32·5)	20·4 (18·5-22·4)	20·2 (18·4-22·2)	18·6 (14·2-23·3)
Cuba	26·3 (22·3-30·8)	20·9 (17·6-24·4)	20·5 (17·3-24·0)	16·4 (13·6-19·5)	2·6 (1·3-4·6)	1·0 (0·5-1·8)	0·9 (0·4-1·7)	0·4 (0·1-1·0)	1,162·7 (875·1-1,465·5)	945·2 (659·6-1,243·3)	931·3 (654·5-1,228·3)	754·2 (565·6-1,036·1)	21·7 (20·2-23·9)	16·7 (15·5-18·2)	16·6 (15·4-18·2)	14·7 (13·6-16·1)
Dominica	36·2 (30·9-41·8)	30·1 (25·3-35·1)	29·7 (25·0-34·7)	24·9 (20·3-29·8)	9·1 (5·9-13·3)	3·2 (1·4-6·0)	2·9 (1·3-5·7)	1·1 (0·4-2·5)	667·3 (543·4-810·6)	628·4 (508·0-758·1)	627·3 (506·9-756·5)	611·9 (490·3-740·9)	17·9 (11·5-27·4)	16·8 (10·7-26·3)	16·9 (11·0-26·1)	15·8 (9·6-24·6)
Dominican Republic	62·0 (56·5-67·3)	63·3 (58·5-68·0)	63·1 (58·2-67·7)	66·3 (60·9-71·2)	8·7 (6·3-11·4)	4·8 (3·0-7·3)	4·6 (2·8-7·1)	2·2 (1·0-4·2)	910·4 (669·8-1,278·5)	867·9 (634·8-1,187·8)	862·8 (630·7-1,182·8)	793·7 (568·7-1,145·8)	29·7 (24·6-37·5)	23·0 (19·4-28·5)	23·7 (19·7-29·6)	24·0 (18·7-32·2)
Grenada	32·3 (27·5-37·5)	24·4 (20·2-29·0)	24·1 (19·9-28·8)	18·4 (13·5-22·7)	4·7 (2·8-7·1)	1·3 (0·6-2·6)	1·2 (0·5-2·5)	0·4 (0·2-1·1)	801·0 (663·5-960·2)	728·8 (596·4-897·2)	720·7 (589·5-887·7)	616·1 (498·4-768·5)	19·9 (10·2-35·0)	17·9 (9·2-31·1)	18·5 (9·4-34·6)	18·4 (7·8-36·4)

Location	Indicator 16.1.1: Age-standardised death rate due to interpersonal violence (per 100,000 population)				Indicator 16.1.2: Death rate due to conflict and terrorism (per 100,000 population)				Indicator 16.1.3: Age-standardised prevalence of physical or sexual violence experienced by populations in the last 12 months (%)				Indicator 16.2.3: Age-standardised prevalence of women and men aged 18-29 years who experienced sexual violence by age 18 (%)			
	**2000**	**2015**	**2016**	**2030**	**2000**	**2015**	**2016**	**2030**	**2000**	**2015**	**2016**	**2030**	**2000**	**2015**	**2016**	**2030**
Azerbaijan	5·8 (4·1-6·9)	3·9 (2·9-5·8)	3·9 (2·8-5·8)	4·5 (1·9-8·9)	0·0 (0·0-0·0)	0·6 (0·4-0·9)	0·0 (0·0-0·0)	0·0 (0·0-0·0)	6·1 (5·5-6·8)	5·0 (4·5-5·6)	5·0 (4·5-5·6)	4·8 (4·2-5·5)	4·2 (3·2-5·4)	4·2 (3·2-5·4)	4·2 (3·2-5·4)	4·2 (3·2-5·4)
Georgia	3·6 (1·8-4·7)	3·7 (2·3-5·6)	3·7 (2·2-5·6)	3·8 (1·7-8·1)	0·4 (0·2-0·7)	0·0 (0·0-0·0)	0·0 (0·0-0·0)	0·0 (0·0-0·0)	4·3 (3·8-4·8)	3·9 (3·5-4·4)	3·9 (3·4-4·3)	3·6 (3·2-4·0)	3·9 (3·0-4·9)	3·9 (3·0-5·0)	3·9 (3·0-5·0)	3·9 (3·0-5·0)
Kazakhstan	19·6 (12·8-27·0)	9·9 (7·3-17·8)	10·2 (7·1-18·0)	4·8 (2·1-11·4)	0·0 (0·0-0·0)	0·0 (0·0-0·0)	0·0 (0·0-0·0)	0·0 (0·0-0·0)	5·3 (4·7-5·9)	4·5 (4·0-5·0)	4·4 (4·0-5·0)	3·8 (3·3-4·2)	4·6 (3·6-5·8)	4·7 (3·6-5·9)	4·7 (3·6-5·9)	4·7 (3·6-6·0)
Kyrgyzstan	9·3 (6·0-13·3)	5·8 (4·1-9·1)	5·8 (4·2-9·0)	3·5 (2·2-6·2)	0·5 (0·2-0·8)	0·0 (0·0-0·0)	0·0 (0·0-0·0)	0·0 (0·0-0·0)	8·8 (7·9-9·8)	8·0 (7·3-8·9)	8·0 (7·2-8·8)	7·3 (6·6-8·0)	4·2 (3·2-5·3)	4·2 (3·2-5·4)	4·2 (3·2-5·4)	4·2 (3·2-5·3)
Mongolia	12·0 (9·0-14·6)	9·6 (7·3-12·4)	9·7 (7·1-12·6)	8·7 (4·7-15·4)	0·0 (0·0-0·0)	0·0 (0·0-0·0)	0·0 (0·0-0·0)	0·0 (0·0-0·0)	7·0 (6·1-8·0)	6·3 (5·5-7·2)	6·3 (5·4-7·2)	5·7 (4·9-6·5)	4·2 (3·2-5·3)	4·2 (3·2-5·4)	4·2 (3·2-5·4)	4·2 (3·1-5·4)
Tajikistan	7·1 (4·3-8·6)	2·8 (2·0-5·1)	2·9 (2·0-5·0)	2·5 (1·0-5·4)	0·8 (0·3-1·2)	0·0 (0·0-0·0)	0·0 (0·0-0·0)	0·0 (0·0-0·0)	9·2 (8·2-10·3)	7·5 (6·8-8·3)	7·5 (6·8-8·3)	7·1 (6·5-7·8)	4·2 (3·2-5·3)	4·2 (3·2-5·3)	4·2 (3·2-5·3)	4·2 (3·2-5·3)
Turkmenistan	6·4 (4·6-9·8)	5·0 (3·8-8·8)	5·0 (3·7-8·8)	4·2 (2·5-7·9)	0·0 (0·0-0·0)	0·0 (0·0-0·0)	0·0 (0·0-0·0)	0·0 (0·0-0·0)	6·4 (5·6-7·3)	5·3 (4·6-6·1)	5·2 (4·6-6·0)	4·4 (3·8-5·0)	4·2 (3·2-5·3)	4·2 (3·2-5·3)	4·2 (3·2-5·3)	4·2 (3·2-5·4)
Uzbekistan	4·3 (3·0-6·5)	3·5 (2·5-6·4)	3·5 (2·5-6·4)	3·0 (1·6-6·4)	1·2 (0·5-2·0)	0·0 (0·0-0·0)	0·0 (0·0-0·0)	0·0 (0·0-0·0)	6·3 (5·4-7·2)	5·4 (4·7-6·2)	5·3 (4·7-6·1)	4·6 (4·0-5·3)	4·2 (3·2-5·3)	4·2 (3·2-5·3)	4·2 (3·2-5·4)	4·2 (3·2-5·4)
**Central Latin America**																
Colombia	62·9 (44·4-71·2)	32·8 (24·8-40·6)	32·5 (24·3-40·5)	22·9 (15·3-32·8)	4·2 (2·8-5·7)	0·3 (0·3-0·3)	0·0 (0·0-0·0)	0·0 (0·0-0·0)	10·4 (9·3-11·6)	8·8 (8·0-9·6)	8·8 (8·0-9·6)	8·3 (7·5-9·1)	8·5 (6·2-11·3)	8·5 (6·2-11·4)	8·5 (6·2-11·4)	8·6 (6·3-11·4)
Costa Rica	6·4 (4·2-9·2)	7·5 (4·0-9·5)	7·3 (4·0-9·4)	7·4 (3·4-12·1)	0·0 (0·0-0·0)	0·0 (0·0-0·0)	0·0 (0·0-0·0)	0·0 (0·0-0·0)	7·0 (6·3-7·8)	6·5 (5·8-7·2)	6·5 (5·8-7·2)	5·7 (5·1-6·3)	14·3 (12·4-16·5)	14·6 (11·8-17·8)	14·7 (11·6-18·1)	14·7 (11·6-18·3)
El Salvador	56·1 (43·6-61·9)	53·1 (38·4-61·8)	52·7 (38·1-62·2)	47·4 (29·6-67·1)	0·0 (0·0-0·0)	0·6 (0·3-0·9)	0·0 (0·0-0·0)	0·0 (0·0-0·0)	6·6 (5·9-7·3)	5·7 (5·1-6·4)	5·7 (5·1-6·3)	5·1 (4·6-5·7)	7·7 (6·2-9·5)	7·7 (6·2-9·5)	7·7 (6·2-9·5)	7·8 (6·2-9·5)
Guatemala	38·9 (26·5-53·1)	37·1 (23·2-49·7)	37·1 (23·2-52·1)	35·0 (13·8-71·0)	0·1 (0·08-0·2)	0·1 (0·08-0·2)	0·0 (0·0-0·0)	0·0 (0·0-0·0)	5·3 (4·8-5·9)	4·6 (4·2-5·0)	4·5 (4·1-5·0)	3·8 (3·4-4·3)	7·1 (5·5-8·8)	7·2 (5·6-9·0)	7·3 (5·7-9·1)	7·4 (5·7-9·3)
Honduras	35·4 (24·3-47·6)	35·3 (23·5-49·7)	34·8 (23·4-49·3)	29·7 (14·8-52·5)	0·0 (0·0-0·0)	0·4 (0·2-0·6)	0·0 (0·0-0·0)	0·0 (0·0-0·0)	6·3 (5·7-7·1)	5·5 (5·0-6·0)	5·4 (4·9-6·0)	4·6 (4·2-5·1)	8·4 (6·8-10·3)	8·7 (7·0-10·6)	8·7 (7·0-10·6)	8·8 (7·1-10·7)
Mexico	14·4 (9·8-20·3)	16·5 (10·3-21·3)	16·2 (10·3-20·9)	13·4 (9·0-18·2)	0·02 (0·01-0·02)	1·4 (0·3-2·6)	0·0 (0·0-0·0)	0·0 (0·0-0·0)	3·0 (2·7-3·5)	2·9 (2·6-3·3)	2·9 (2·6-3·3)	2·9 (2·6-3·3)	6·7 (5·1-8·6)	6·8 (5·1-8·7)	6·8 (5·1-8·7)	6·9 (5·1-8·8)
Nicaragua	11·4 (9·2-13·0)	10·2 (7·9-12·6)	10·1 (7·6-13·0)	9·2 (5·2-16·3)	0·5 (0·2-0·7)	0·0 (0·0-0·0)	0·0 (0·0-0·0)	0·0 (0·0-0·0)	5·8 (5·3-6·3)	4·9 (4·4-5·4)	4·9 (4·4-5·4)	4·9 (4·2-5·6)	7·1 (5·7-8·6)	7·0 (5·6-8·7)	7·1 (5·6-8·8)	7·1 (5·6-8·8)
Panama	10·2 (6·6-13·9)	15·8 (8·0-21·5)	15·4 (7·8-21·3)	14·7 (5·7-28·1)	0·0 (0·0-0·0)	0·0 (0·0-0·0)	0·0 (0·0-0·0)	0·0 (0·0-0·0)	5·0 (4·2-5·9)	4·7 (4·0-5·6)	4·6 (3·9-5·5)	4·0 (3·4-4·8)	8·5 (6·2-11·3)	8·5 (6·2-11·3)	8·5 (6·2-11·3)	8·5 (6·3-11·4)
Venezuela	27·6 (17·1-33·4)	38·1 (18·3-53·6)	37·9 (18·5-55·3)	39·1 (14·7-86·1)	0·1 (0·02-0·2)	0·0 (0·0-0·0)	0·0 (0·0-0·0)	0·0 (0·0-0·0)	5·7 (4·9-6·8)	5·3 (4·5-6·3)	5·3 (4·5-6·3)	4·7 (4·0-5·6)	8·5 (6·2-11·3)	8·5 (6·2-11·4)	8·5 (6·2-11·4)	8·6 (6·3-11·4)
**Andean Latin America**																
Bolivia	7·2 (5·4-8·8)	6·4 (4·2-8·4)	6·3 (4·1-8·4)	6·0 (2·8-10·5)	0·6 (0·1-1·2)	0·0 (0·0-0·0)	0·0 (0·0-0·0)	0·0 (0·0-0·0)	16·8 (15·2-18·9)	14·7 (13·4-16·5)	14·6 (13·2-16·4)	13·2 (11·5-15·3)	10·9 (8·4-14·0)	10·9 (8·4-14·0)	10·9 (8·4-14·0)	10·9 (8·4-14·0)
Ecuador	19·2 (11·4-23·2)	13·7 (8·8-17·8)	13·6 (8·6-17·8)	10·5 (6·6-15·5)	0·0 (0·0-0·0)	0·0 (0·0-0·0)	0·0 (0·0-0·0)	0·0 (0·0-0·0)	8·4 (7·6-9·4)	7·8 (6·9-8·7)	7·7 (6·9-8·6)	6·7 (6·0-7·5)	10·9 (8·4-14·0)	10·9 (8·4-14·0)	10·9 (8·4-14·0)	10·9 (8·4-14·0)
Peru	4·2 (3·4-5·0)	3·3 (2·4-4·3)	3·3 (2·3-4·4)	2·6 (1·2-5·0)	0·0 (0·0-0·0)	0·0 (0·0-0·0)	0·0 (0·0-0·0)	0·0 (0·0-0·0)	7·6 (7·0-8·2)	6·5 (6·1-7·0)	6·5 (6·1-6·9)	5·7 (5·2-6·1)	10·7 (8·8-12·6)	10·8 (8·6-13·6)	10·9 (8·5-13·8)	10·9 (8·5-13·8)
**Caribbean**																
Antigua and Barbuda	4·3 (3·1-6·3)	5·2 (2·9-7·0)	5·1 (2·8-6·9)	4·4 (2·2-7·6)	0·0 (0·0-0·0)	0·0 (0·0-0·0)	0·0 (0·0-0·0)	0·0 (0·0-0·0)	5·2 (4·5-6·0)	4·9 (4·3-5·8)	4·9 (4·2-5·7)	4·5 (3·8-5·2)	7·4 (5·6-9·3)	7·3 (5·6-9·1)	7·3 (5·6-9·2)	7·3 (5·6-9·1)
The Bahamas	15·9 (11·2-21·3)	20·0 (10·1-26·7)	19·6 (10·0-26·7)	20·3 (7·1-36·3)	0·0 (0·0-0·0)	0·0 (0·0-0·0)	0·0 (0·0-0·0)	0·0 (0·0-0·0)	5·9 (5·2-7·0)	5·9 (5·1-6·9)	5·9 (5·1-6·9)	5·4 (4·7-6·3)	7·4 (5·6-9·3)	7·4 (5·6-9·3)	7·4 (5·6-9·3)	7·4 (5·6-9·3)
Barbados	8·2 (5·7-10·9)	9·7 (5·2-12·8)	9·6 (5·1-12·8)	8·5 (3·9-14·0)	0·0 (0·0-0·0)	0·0 (0·0-0·0)	0·0 (0·0-0·0)	0·0 (0·0-0·0)	5·3 (4·6-6·1)	5·3 (4·6-6·2)	5·3 (4·6-6·2)	5·1 (4·4-5·9)	7·4 (5·6-9·3)	7·4 (5·6-9·3)	7·4 (5·6-9·3)	7·4 (5·6-9·2)
Belize	19·4 (13·5-26·7)	21·4 (11·0-29·4)	21·1 (10·8-29·6)	19·1 (7·2-36·9)	0·0 (0·0-0·0)	0·0 (0·0-0·0)	0·0 (0·0-0·0)	0·0 (0·0-0·0)	7·5 (6·5-8·7)	7·1 (6·1-8·3)	7·1 (6·1-8·2)	6·5 (5·5-7·5)	6·7 (5·4-8·2)	6·7 (5·3-8·4)	6·7 (5·3-8·4)	6·8 (5·2-8·5)
Cuba	5·6 (4·1-8·2)	5·2 (3·2-6·7)	5·1 (3·2-6·7)	4·7 (2·6-6·9)	0·0 (0·0-0·0)	0·0 (0·0-0·0)	0·0 (0·0-0·0)	0·0 (0·0-0·0)	5·4 (4·6-6·3)	4·9 (4·2-5·7)	4·9 (4·2-5·7)	4·5 (3·8-5·2)	7·5 (5·7-9·5)	7·5 (5·7-9·4)	7·5 (5·7-9·4)	7·5 (5·7-9·5)
Dominica	5·3 (4·0-8·8)	10·0 (5·0-13·5)	9·9 (5·0-13·8)	12·6 (4·9-23·1)	0·0 (0·0-0·0)	0·0 (0·0-0·0)	0·0 (0·0-0·0)	0·0 (0·0-0·0)	5·6 (4·9-6·6)	5·6 (4·8-6·5)	5·5 (4·8-6·4)	5·2 (4·5-6·0)	7·4 (5·6-9·3)	7·4 (5·6-9·3)	7·4 (5·6-9·3)	7·4 (5·6-9·3)
Dominican Republic	11·8 (9·9-14·7)	13·7 (8·8-17·1)	13·6 (8·7-17·2)	13·2 (6·5-21·8)	0·0 (0·0-0·0)	0·0 (0·0-0·0)	0·0 (0·0-0·0)	0·0 (0·0-0·0)	7·7 (6·9-8·7)	7·1 (6·4-7·8)	7·0 (6·4-7·8)	6·5 (5·8-7·4)	7·4 (5·6-9·2)	7·4 (5·6-9·2)	7·4 (5·6-9·2)	7·4 (5·6-9·3)
Grenada	5·8 (3·5-8·1)	6·9 (3·9-9·5)	6·8 (3·9-9·3)	7·7 (3·3-14·7)	0·0 (0·0-0·0)	0·0 (0·0-0·0)	0·0 (0·0-0·0)	0·0 (0·0-0·0)	6·2 (5·4-7·2)	5·5 (4·8-6·4)	5·5 (4·7-6·4)	5·0 (4·3-5·8)	7·4 (5·6-9·2)	7·4 (5·6-9·3)	7·4 (5·6-9·3)	7·5 (5·7-9·5)

Location	Indicator 17.19.2c: Percentage of well-certified deaths by a vital registration (VR) system among a country's total population (%)															
	**2000**	**2015**	**2016**	**2030**												
Azerbaijan	69·9 (64·7-75·0)	67·8 (49·4-82·7)	69·0 (50·1-83·8)	74·2 (38·6-96·2)												
Georgia	73·6 (66·6-79·8)	54·7 (44·7-64·8)	56·0 (44·3-67·7)	42·5 (23·2-63·9)												
Kazakhstan	80·6 (76·5-84·5)	81·2 (74·6-86·4)	81·7 (73·5-87·4)	71·8 (58·7-82·6)												
Kyrgyzstan	78·0 (73·9-81·8)	88·9 (84·1-92·5)	89·0 (83·4-93·0)	92·8 (85·4-96·9)												
Mongolia	9·7 (4·5-18·7)	23·7 (11·3-41·0)	23·1 (10·8-39·5)	14·4 (1·5-45·8)												
Tajikistan	41·4 (36·1-47·1)	52·8 (32·7-72·8)	53·1 (32·4-73·4)	58·8 (19·9-92·0)												
Turkmenistan	62·8 (57·3-67·8)	71·1 (62·7-78·1)	72·0 (62·0-80·5)	76·2 (56·8-89·4)												
Uzbekistan	59·8 (54·5-65·1)	65·7 (56·7-73·8)	66·3 (55·8-75·9)	70·5 (43·8-90·8)												
**Central Latin America**																
Colombia	83·2 (80·8-85·5)	87·5 (84·7-89·7)	87·9 (85·0-90·2)	92·7 (87·9-95·6)												
Costa Rica	88·5 (86·5-90·4)	90·9 (88·8-92·7)	91·1 (89·0-93·0)	94·4 (90·7-97·1)												
El Salvador	63·2 (59·7-66·6)	66·1 (60·9-70·7)	67·1 (61·9-72·1)	71·8 (62·9-79·6)												
Guatemala	67·2 (63·9-70·5)	74·3 (69·9-78·2)	74·8 (70·5-78·8)	79·3 (71·5-85·5)												
Honduras	13·3 (10·1-17·0)	15·7 (12·9-19·0)	16·2 (13·2-19·8)	22·3 (13·3-34·7)												
Mexico	77·1 (76·5-77·7)	86·1 (85·6-86·7)	86·3 (85·7-86·9)	91·5 (90·8-92·1)												
Nicaragua	64·7 (61·3-68·0)	83·3 (79·8-86·4)	83·6 (80·0-86·8)	92·5 (88·4-95·5)												
Panama	78·5 (75·7-81·3)	85·0 (82·1-87·6)	85·6 (82·8-88·2)	90·7 (85·6-94·2)												
Venezuela	88·0 (85·9-89·7)	90·1 (87·9-92·0)	90·3 (88·1-92·3)	92·8 (89·0-95·5)												
**Andean Latin America**																
Bolivia	11·6 (5·8-20·1)	27·4 (7·3-60·4)	28·3 (7·3-61·6)	48·7 (4·8-100·0)												
Ecuador	60·6 (54·9-65·9)	68·6 (56·4-79·3)	68·8 (52·0-82·0)	73·0 (46·8-91·4)												
Peru	50·1 (42·0-57·8)	59·1 (44·5-71·4)	59·3 (41·3-74·5)	64·6 (37·4-82·8)												
**Caribbean**																
Antigua and Barbuda	74·3 (70·0-78·1)	73·4 (66·7-79·3)	74·1 (66·7-80·5)	76·7 (63·3-87·4)												
The Bahamas	72·9 (68·5-77·0)	74·4 (67·5-80·6)	74·6 (67·0-81·0)	77·8 (63·3-88·4)												
Barbados	69·5 (64·3-74·3)	79·7 (73·1-84·9)	79·7 (72·5-85·3)	82·3 (68·9-91·3)												
Belize	68·1 (63·2-72·9)	81·9 (76·1-86·9)	82·0 (75·9-87·4)	90·6 (84·1-95·1)												
Cuba	86·9 (84·3-89·3)	91·6 (88·7-94·0)	91·8 (88·6-94·2)	94·6 (90·3-97·4)												
Dominica	60·8 (56·3-65·2)	78·1 (72·5-83·2)	78·5 (72·5-84·2)	83·3 (72·5-91·3)												
Dominican Republic	52·0 (47·4-56·5)	65·1 (56·2-72·9)	65·8 (56·1-74·4)	76·7 (62·0-88·0)												
Grenada	64·6 (58·7-70·0)	80·9 (76·2-85·4)	81·3 (75·9-86·1)	89·0 (82·6-94·1)												

Location	Indicator 1.5.1: Death rate due to exposure to forces of nature (per 100,000 population)				Indicator 2.2.1: Prevalence of stunting in children under 5 (%)				Indicator 2.2.2a: Prevalence of wasting in children under 5 (%)				Indicator 2.2.2b: Prevalence of overweight in children aged 2-4 (%)			
	2000	2015	2016	2030	2000	2015	2016	2030	2000	2015	2016	2030	2000	2015	2016	2030
Guyana	0·08 (0·01-0·1)	0·02 (0·0-0·03)	0·01 (0·0-0·03)	0·2 (0·03-0·4)	14·3 (12·4-16·3)	13·2 (10·6-16·2)	12·5 (9·8-15·5)	11·3 (10·5-12·5)	11·2 (10·2-12·2)	6·4 (5·3-7·5)	6·3 (5·2-7·5)	4·0 (3·4-4·6)	12·3 (8·4-17·6)	16·0 (11·1-22·7)	16·5 (11·3-23·5)	22·5 (10·5-40·1)
Haiti	1·6 (0·3-3·1)	206·5 (32·8-395·7)	167·3 (28·5-319·5)	148·1 (24·6-283·1)	29·3 (25·6-33·1)	21·9 (17·6-26·5)	21·4 (17·1-25·9)	15·4 (12·1-19·5)	7·4 (6·6-8·3)	5·0 (4·2-5·9)	4·8 (4·0-5·7)	2·8 (2·1-3·5)	7·4 (5·6-9·8)	8·3 (5·9-11·7)	8·5 (5·8-12·0)	8·9 (4·2-16·2)
Jamaica	0·01 (0·0-0·02)	0·08 (0·01-0·1)	0·06 (0·01-0·1)	0·08 (0·01-0·1)	8·5 (7·1-10·0)	6·7 (4·9-8·9)	6·5 (4·7-8·7)	5·4 (3·4-7·9)	3·1 (2·6-3·6)	2·2 (1·7-2·9)	2·2 (1·6-2·8)	1·6 (1·0-2·3)	15·5 (9·9-23·2)	18·2 (12·2-26·0)	18·2 (12·2-26·0)	20·2 (9·2-36·0)
Saint Lucia	0·1 (0·02-0·2)	0·8 (0·1-1·5)	0·6 (0·1-1·2)	0·5 (0·09-1·0)	4·0 (2·7-5·7)	3·2 (2·1-4·7)	3·1 (2·0-4·6)	2·4 (1·6-3·6)	3·6 (2·7-4·8)	2·9 (2·2-3·7)	2·9 (2·2-3·7)	2·3 (1·8-3·0)	12·8 (8·3-18·8)	11·8 (8·1-16·4)	12·0 (8·0-17·2)	11·2 (4·7-20·7)
Saint Vincent and the Grenadines	0·0 (0·0-0·0)	1·7 (0·3-3·2)	1·4 (0·2-2·8)	1·1 (0·2-2·1)	11·4 (8·1-15·0)	9·7 (6·8-12·8)	9·6 (6·7-12·8)	8·2 (5·7-10·9)	3·2 (2·4-4·2)	2·6 (2·0-3·5)	2·6 (1·9-3·4)	2·3 (1·7-3·1)	31·5 (22·6-42·3)	45·0 (33·8-56·0)	45·5 (34·4-56·5)	55·5 (36·7-73·9)
Suriname	0·04 (0·01-0·08)	0·0 (0·0-0·0)	0·0 (0·0-0·0)	0·01 (0·0-0·02)	14·2 (12·1-16·5)	8·6 (6·3-11·5)	8·5 (6·2-11·5)	7·2 (4·1-9·5)	5·8 (4·7-6·9)	3·9 (3·0-4·9)	3·8 (2·9-4·8)	2·8 (2·1-3·9)	8·2 (5·3-12·2)	12·6 (8·2-18·6)	12·9 (8·5-19·1)	18·5 (8·7-34·9)
Trinidad and Tobago	0·0 (0·0-0·0)	0·04 (0·01-0·09)	0·03 (0·01-0·07)	0·06 (0·01-0·1)	2·6 (1·8-3·4)	1·5 (0·9-2·3)	1·5 (0·9-2·3)	1·0 (0·5-1·6)	4·7 (3·7-5·8)	4·0 (2·8-5·2)	3·9 (2·8-5·2)	3·5 (2·3-4·9)	12·6 (8·2-18·2)	19·5 (13·4-27·5)	19·7 (13·4-27·5)	29·1 (14·9-48·1)
**Tropical Latin America**																
Brazil	0·1 (0·1-0·1)	0·1 (0·1-0·1)	0·1 (0·1-0·1)	0·1 (0·1-0·1)	11·8 (11·1-12·6)	6·1 (5·7-6·6)	6·0 (5·6-6·5)	3·5 (3·2-3·8)	3·8 (3·5-4·2)	2·5 (2·3-2·8)	2·5 (2·2-2·8)	2·2 (1·9-2·5)	20·4 (17·9-23·0)	32·2 (28·5-36·3)	32·6 (28·9-36·9)	45·9 (37·2-55·2)
Paraguay	0·3 (0·3-0·4)	0·2 (0·2-0·2)	0·1 (0·1-0·2)	0·2 (0·2-0·2)	14·8 (11·3-19·1)	7·3 (5·4-9·9)	7·0 (5·2-9·5)	4·2 (3·0-5·6)	1·5 (1·0-2·4)	1·6 (1·0-2·6)	1·6 (0·9-2·6)	1·7 (0·8-2·8)	18·1 (15·7-20·7)	27·1 (23·8-30·6)	27·7 (24·4-31·2)	38·7 (32·1-46·4)
**East Asia**																
China	0·2 (0·03-0·4)	0·5 (0·08-0·9)	0·4 (0·06-0·7)	0·5 (0·08-0·9)	19·9 (18·8-20·9)	11·9 (11·2-12·6)	11·6 (10·9-12·3)	8·1 (7·6-8·7)	4·0 (3·8-4·3)	2·7 (2·5-2·9)	2·7 (2·5-2·9)	2·1 (1·9-2·3)	17·3 (12·0-24·5)	23·8 (16·2-32·6)	24·1 (16·4-33·0)	28·1 (13·0-46·4)
North Korea	0·2 (0·03-0·3)	0·3 (0·04-0·5)	0·6 (0·09-1·1)	0·5 (0·08-1·0)	50·6 (44·4-57·8)	30·9 (25·5-36·9)	30·7 (25·1-37·0)	27·9 (20·0-35·1)	12·1 (10·9-13·2)	5·9 (4·7-7·3)	5·8 (4·6-7·3)	3·0 (2·5-3·9)	1·6 (0·9-2·6)	1·7 (1·0-2·8)	1·7 (1·0-2·8)	1·9 (0·7-4·3)
Taiwan (Province of China)	1·9 (0·3-3·7)	0·2 (0·04-0·4)	0·4 (0·06-0·7)	0·5 (0·08-0·9)	6·1 (4·4-8·1)	4·7 (3·4-6·4)	4·7 (3·4-6·3)	3·9 (2·8-5·4)	2·3 (1·6-3·2)	1·9 (1·3-2·6)	1·9 (1·3-2·6)	1·6 (1·0-2·3)	26·3 (17·4-35·8)	29·7 (20·6-40·2)	30·1 (20·9-40·9)	34·0 (16·7-53·5)
**Southeast Asia**																
Cambodia	1·0 (0·6-1·5)	0·4 (0·2-0·7)	0·4 (0·2-0·6)	0·4 (0·2-0·5)	49·0 (45·5-53·1)	35·0 (27·7-44·5)	33·2 (25·6-43·1)	20·9 (13·5-32·0)	12·4 (11·2-13·4)	9·5 (8·0-11·0)	9·3 (7·7-10·9)	6·8 (5·3-9·4)	5·3 (3·8-7·2)	4·1 (2·4-6·6)	4·4 (2·5-7·1)	4·5 (1·3-10·7)
Indonesia	0·03 (0·02-0·04)	0·1 (0·06-0·1)	0·09 (0·05-0·1)	2·5 (1·5-3·5)	38·1 (36·5-39·8)	32·8 (31·5-34·2)	31·6 (30·2-33·0)	31·0 (30·3-31·9)	12·7 (12·1-13·3)	13·0 (12·5-13·5)	12·8 (12·3-13·4)	12·8 (12·6-12·9)	9·7 (6·6-14·2)	16·6 (8·8-27·3)	17·1 (9·0-28·4)	25·9 (8·5-52·6)
Laos	0·2 (0·1-0·3)	0·2 (0·07-0·2)	0·1 (0·06-0·2)	0·1 (0·06-0·2)	60·9 (55·1-66·7)	41·3 (30·5-53·5)	40·3 (28·9-53·0)	26·4 (13·0-45·1)	11·5 (9·6-13·3)	6·6 (4·7-8·8)	6·5 (4·6-8·7)	4·9 (3·3-6·4)	5·4 (2·6-10·1)	7·4 (3·3-14·1)	7·8 (3·4-14·5)	13·0 (2·6-32·1)
Malaysia	0·2 (0·1-0·3)	0·01 (0·0-0·01)	0·0 (0·0-0·0)	0·04 (0·02-0·06)	19·1 (15·8-22·7)	17·4 (14·6-20·3)	17·6 (15·4-20·0)	16·1 (14·4-17·5)	13·3 (11·7-15·2)	9·7 (8·4-11·4)	9·6 (8·4-11·2)	8·1 (7·1-9·0)	24·0 (12·9-39·4)	34·8 (19·9-51·4)	35·4 (20·4-52·8)	47·2 (19·1-75·8)
Maldives	0·0 (0·0-0·0)	0·04 (0·04-0·04)	0·03 (0·03-0·03)	1·2 (0·6-1·7)	39·5 (34·9-44·9)	20·3 (14·5-26·5)	20·1 (13·9-26·8)	10·3 (7·5-11·8)	17·9 (16·1-19·8)	9·8 (7·5-12·7)	9·7 (7·2-12·8)	5·9 (4·0-8·0)	7·5 (3·6-13·6)	14·2 (6·9-24·6)	14·8 (7·2-25·4)	25·0 (6·8-54·3)
Mauritius	0·01 (0·01-0·02)	0·1 (0·06-0·2)	0·1 (0·05-0·2)	0·07 (0·03-0·1)	12·5 (8·2-17·9)	10·1 (6·4-15·0)	10·0 (6·3-14·8)	8·1 (5·0-12·4)	13·6 (11·1-16·6)	11·2 (7·8-15·1)	11·1 (7·7-15·0)	9·3 (5·5-13·7)	22·5 (12·2-36·6)	30·0 (16·6-45·7)	30·3 (16·6-45·4)	40·5 (13·5-69·7)
Myanmar	0·03 (0·02-0·04)	15·1 (7·8-22·4)	10·1 (5·2-14·9)	14·4 (7·5-21·3)	44·5 (38·9-51·6)	29·5 (25·2-34·6)	28·4 (24·2-33·2)	17·6 (13·0-21·8)	11·2 (9·9-12·5)	7·0 (5·8-8·3)	6·8 (5·7-8·2)	4·3 (3·9-5·0)	6·0 (2·8-11·3)	14·3 (6·8-24·5)	14·9 (7·1-25·9)	28·6 (8·0-56·8)
Philippines	1·0 (0·5-1·4)	2·1 (1·1-3·1)	1·7 (0·9-2·6)	1·6 (0·8-2·4)	37·5 (31·9-44·1)	32·4 (23·9-42·4)	31·6 (22·6-42·2)	29·3 (19·9-41·5)	7·5 (6·2-8·8)	6·8 (5·1-8·8)	6·8 (5·0-8·9)	7·1 (4·4-9·6)	5·6 (2·5-10·2)	9·7 (4·5-18·0)	9·9 (4·6-18·6)	15·0 (3·6-36·7)
Sri Lanka	0·6 (0·3-0·9)	0·5 (0·3-0·7)	0·5 (0·2-0·7)	6·5 (3·3-9·8)	25·4 (23·8-27·1)	14·4 (9·7-19·6)	14·1 (9·2-19·5)	9·7 (4·6-15·2)	16·4 (15·7-17·2)	12·1 (9·1-15·5)	12·0 (8·9-15·6)	9·1 (5·5-13·4)	23·5 (17·5-30·3)	23·0 (12·2-37·3)	23·7 (12·8-37·4)	30·7 (9·4-61·9)
Seychelles	0·0 (0·0-0·0)	0·0 (0·0-0·0)	0·0 (0·0-0·0)	0·0 (0·0-0·0)	8·5 (5·5-12·5)	8·9 (6·0-12·7)	9·0 (6·0-13·0)	9·6 (5·4-16·8)	3·8 (2·8-5·1)	4·6 (3·7-5·7)	4·6 (3·7-5·7)	4·7 (3·8-6·1)	19·2 (9·7-32·4)	24·9 (13·4-39·0)	25·5 (14·0-40·3)	31·8 (10·3-60·9)
Thailand	0·3 (0·2-0·5)	0·4 (0·2-0·5)	0·3 (0·1-0·4)	0·7 (0·4-1·1)	15·4 (10·4-21·2)	13·6 (9·4-18·5)	13·3 (9·0-18·3)	9·8 (4·7-16·9)	5·7 (4·7-7·0)	7·0 (5·7-8·6)	7·2 (5·7-8·9)	9·2 (6·1-13·0)	12·4 (6·2-21·8)	19·8 (12·9-28·2)	20·4 (12·5-30·0)	30·3 (11·7-54·8)
Timor-Leste	0·0 (0·0-0·0)	0·0 (0·0-0·0)	0·0 (0·0-0·0)	0·0 (0·0-0·0)	60·0 (53·1-67·3)	48·1 (39·5-57·2)	46·8 (37·7-56·5)	37·6 (31·8-47·4)	16·4 (14·4-18·7)	15·1 (13·1-17·5)	14·6 (12·5-17·1)	11·8 (10·1-13·6)	11·8 (7·2-17·9)	11·6 (5·8-19·5)	11·8 (5·8-20·5)	15·5 (3·6-36·8)
Vietnam	1·3 (0·7-1·8)	0·2 (0·08-0·2)	0·1 (0·05-0·2)	0·3 (0·2-0·4)	43·0 (38·7-49·1)	21·2 (15·2-27·3)	20·0 (13·7-26·5)	8·1 (3·9-15·6)	10·4 (9·5-11·1)	5·7 (4·7-7·0)	5·6 (4·4-7·0)	3·8 (2·3-5·4)	4·5 (2·7-7·4)	10·2 (4·8-19·3)	10·5 (4·9-19·9)	19·9 (5·1-45·1)
**Oceania**																
Federated States of Micronesia	0·0 (0·0-0·0)	0·0 (0·0-0·0)	0·0 (0·0-0·0)	1·1 (0·9-1·3)	14·9 (10·7-19·6)	11·9 (8·4-15·9)	11·7 (8·3-15·6)	8·9 (6·0-12·4)	5·1 (3·5-7·1)	5·4 (3·8-7·5)	5·4 (3·8-7·5)	5·3 (3·7-7·5)	36·9 (15·5-62·2)	41·6 (19·4-66·3)	41·9 (19·4-65·2)	47·5 (11·3-84·9)
Fiji	0·8 (0·7-1·0)	0·3 (0·2-0·3)	0·2 (0·2-0·3)	0·4 (0·3-0·5)	6·8 (5·0-9·1)	6·2 (4·5-8·5)	6·1 (4·4-8·4)	4·7 (3·2-6·7)	7·0 (5·5-8·7)	5·9 (4·2-8·1)	5·8 (4·1-8·1)	5·4 (3·4-7·8)	28·1 (10·2-54·2)	31·0 (11·8-56·7)	31·3 (11·9-57·2)	35·8 (5·9-75·5)

Location	Indicator 3.1.1: Maternal mortality ratio (maternal deaths per 100,000 livebirths) in women aged 10-54 years				Indicator 3.1.2: Proportion of births attended by skilled health personnel (%)				Indicator 3.2.1: Under-5 mortality rate (probability of dying before the age of 5 per 1,000 livebirths)				Indicator 3.2.2: Neonatal mortality rate (probability of dying during the first 28 days of life per 1,000 livebirths)			
	**2000**	**2015**	**2016**	**2030**	**2000**	**2015**	**2016**	**2030**	**2000**	**2015**	**2016**	**2030**	**2000**	**2015**	**2016**	**2030**
Guyana	105·4 (87·5-125·5)	130·3 (103·7-160·8)	112·7 (86·3-141·9)	49·7 (22·8-90·9)	88·2 (84·7-91·2)	91·7 (87·0-94·9)	92·2 (86·9-95·6)	94·8 (82·2-99·3)	39·2 (36·0-42·8)	27·6 (22·4-34·0)	24·9 (19·7-31·2)	16·1 (9·5-25·6)	23·9 (22·0-26·1)	18·0 (14·6-22·0)	16·4 (13·1-20·5)	12·1 (7·2-18·9)
Haiti	511·5 (398·0-650·3)	374·3 (240·8-556·2)	363·6 (232·5-539·9)	225·6 (89·4-479·2)	36·4 (26·3-47·0)	79·5 (63·8-90·5)	79·8 (62·8-91·4)	85·8 (46·2-98·2)	100·0 (86·1-114·7)	57·8 (39·3-84·8)	55·4 (37·4-79·2)	30·7 (10·7-74·4)	32·5 (28·0-37·2)	23·1 (15·6-33·7)	22·1 (15·0-31·5)	12·4 (4·4-28·1)
Jamaica	61·8 (47·9-78·2)	48·0 (32·5-69·8)	47·0 (31·1-68·3)	38·4 (12·5-93·5)	98·0 (97·1-98·6)	98·6 (96·9-99·5)	98·7 (96·9-99·5)	98·9 (93·0-100·0)	24·7 (17·5-33·4)	20·3 (12·1-31·6)	20·1 (12·2-31·7)	16·7 (4·0-46·0)	13·5 (9·7-18·1)	11·6 (7·2-17·5)	11·4 (7·2-17·5)	9·8 (2·5-25·8)
Saint Lucia	103·5 (90·3-118·6)	89·9 (76·4-105·6)	85·7 (71·8-100·7)	74·0 (45·2-114·0)	98·5 (96·9-99·4)	98·6 (96·7-99·6)	98·6 (96·6-99·6)	98·6 (89·7-100·0)	18·1 (11·4-27·0)	17·9 (10·1-30·2)	17·5 (9·1-31·2)	13·6 (2·0-45·3)	12·2 (7·7-18·1)	13·4 (7·8-22·1)	13·0 (7·0-22·5)	11·3 (1·9-35·3)
Saint Vincent and the Grenadines	41·0 (33·8-49·9)	45·9 (36·9-56·5)	45·0 (35·9-56·1)	21·1 (7·3-52·1)	98·6 (97·2-99·4)	98·8 (97·3-99·6)	98·8 (97·2-99·6)	98·7 (91·2-100·0)	23·3 (16·5-31·5)	17·1 (11·0-26·5)	16·6 (10·4-26·8)	11·8 (3·8-27·4)	14·5 (10·1-19·8)	11·0 (7·3-16·5)	10·6 (6·9-16·5)	6·9 (2·3-15·8)
Suriname	69·5 (57·1-83·5)	62·3 (47·3-80·4)	61·7 (45·8-79·5)	54·6 (27·3-96·9)	91·9 (87·6-95·0)	96·2 (91·7-98·6)	96·4 (91·7-98·8)	98·3 (88·6-100·0)	45·4 (38·1-53·3)	35·4 (27·5-45·1)	34·8 (26·8-44·8)	27·2 (17·0-41·2)	24·9 (20·9-29·0)	20·7 (16·1-26·3)	20·4 (15·7-26·2)	16·8 (10·5-25·4)
Trinidad and Tobago	67·1 (57·7-77·8)	67·2 (53·3-84·7)	67·6 (53·3-86·0)	73·1 (38·8-136·1)	98·5 (97·1-99·3)	98·7 (97·1-99·6)	98·8 (97·1-99·7)	99·1 (94·4-100·0)	27·5 (22·3-33·8)	21·1 (12·7-33·4)	20·9 (12·3-34·2)	18·5 (5·7-45·9)	20·7 (16·7-25·7)	16·0 (9·7-25·1)	15·9 (9·4-25·6)	13·9 (4·3-34·4)
**Tropical Latin America**																
Brazil	64·9 (62·0-68·2)	70·8 (67·0-75·4)	68·3 (63·8-73·2)	69·1 (58·7-81·6)	96·9 (95·8-97·8)	99·0 (98·4-99·4)	99·1 (98·4-99·5)	99·6 (98·9-99·9)	31·7 (26·8-37·0)	17·4 (13·2-22·6)	16·9 (12·8-22·1)	9·9 (4·5-18·8)	14·4 (12·2-16·8)	9·0 (6·7-11·7)	8·7 (6·6-11·4)	5·5 (2·5-10·4)
Paraguay	105·4 (90·7-123·1)	101·1 (77·7-129·4)	98·7 (74·4-131·2)	82·5 (38·4-161·6)	77·1 (73·6-80·4)	92·9 (89·6-95·2)	93·1 (89·8-95·4)	96·9 (93·1-99·0)	28·9 (25·2-32·6)	19·5 (15·0-25·4)	18·9 (14·3-24·7)	12·2 (6·4-21·3)	15·3 (13·3-17·4)	11·0 (8·4-14·3)	10·6 (8·1-13·9)	7·4 (3·9-13·2)
**East Asia**																
China	89·4 (82·3-96·8)	21·4 (19·6-23·4)	20·9 (18·8-23·2)	6·0 (4·7-7·7)	85·5 (69·0-94·9)	96·8 (92·3-99·0)	97·0 (92·5-99·1)	99·2 (95·6-100·0)	34·9 (31·4-38·9)	12·5 (10·9-14·5)	11·8 (10·2-13·7)	5·1 (3·5-7·4)	16·0 (14·3-17·8)	6·3 (5·5-7·2)	5·9 (5·2-6·8)	2·4 (1·6-3·4)
North Korea	82·6 (60·3-109·4)	82·6 (61·1-106·7)	80·4 (58·6-105·0)	57·3 (26·4-105·2)	81·3 (59·9-93·2)	91·7 (79·6-97·4)	91·8 (80·3-97·4)	95·5 (75·1-100·0)	87·5 (42·2-146·4)	27·2 (21·2-35·4)	26·2 (20·4-34·3)	19·1 (10·6-33·1)	15·6 (12·2-19·5)	13·3 (10·6-17·0)	12·9 (10·2-16·5)	10·2 (5·8-17·3)
Taiwan (Province of China)	18·3 (14·2-23·1)	14·2 (11·3-17·9)	14·2 (10·8-18·5)	11·2 (5·6-20·9)	94·8 (87·5-98·5)	98·3 (95·7-99·5)	98·2 (95·4-99·5)	98·9 (92·7-100·0)	8·6 (8·4-8·9)	4·7 (4·3-5·2)	4·7 (4·2-5·4)	2·9 (2·0-4·0)	4·1 (3·9-4·3)	2·2 (2·1-2·4)	2·2 (2·0-2·4)	1·4 (1·0-1·9)
**Southeast Asia**																
Cambodia	447·7 (376·4-525·4)	137·8 (102·6-180·9)	134·4 (99·0-178·2)	55·1 (25·8-105·6)	34·2 (32·6-35·8)	93·4 (92·0-94·7)	94·3 (92·7-95·6)	99·7 (99·4-99·9)	94·8 (88·1-101·5)	33·8 (28·0-40·5)	31·9 (26·1-38·6)	12·7 (7·7-19·8)	36·0 (33·1-38·9)	16·5 (13·5-20·2)	15·7 (12·7-19·3)	7·9 (4·6-12·9)
Indonesia	418·3 (375·6-455·6)	289·0 (259·4-318·7)	282·8 (253·9-313·5)	199·3 (162·0-242·5)	58·1 (55·8-60·4)	83·7 (79·1-87·8)	84·0 (78·5-88·5)	94·3 (87·4-98·2)	54·1 (50·7-57·6)	26·5 (21·8-32·0)	25·2 (20·5-31·0)	12·6 (7·8-19·0)	24·1 (22·5-25·7)	14·1 (11·6-17·1)	13·5 (11·0-16·6)	8·2 (5·2-12·3)
Laos	455·9 (385·8-538·1)	187·9 (136·7-250·4)	177·3 (126·5-240·3)	73·0 (33·2-133·0)	26·7 (22·5-31·4)	53·9 (41·9-66·3)	56·3 (43·5-68·9)	85·9 (62·9-98·7)	140·9 (125·3-159·2)	61·4 (44·0-84·2)	58·2 (40·9-80·4)	26·0 (10·6-53·0)	52·3 (46·5-59·1)	27·1 (19·5-36·8)	25·9 (18·3-35·4)	13·3 (5·5-26·7)
Malaysia	70·1 (57·9-83·7)	51·2 (39·9-65·0)	49·4 (38·4-62·5)	34·4 (18·8-56·7)	95·8 (94·1-97·1)	98·9 (98·0-99·5)	99·0 (98·2-99·5)	99·7 (98·8-100·0)	9·3 (9·1-9·5)	6·4 (5·7-7·1)	6·0 (5·2-6·9)	3·7 (2·4-5·3)	4·3 (4·2-4·5)	3·1 (2·7-3·4)	2·9 (2·5-3·3)	1·8 (1·2-2·5)
Maldives	172·7 (132·7-215·8)	51·1 (33·8-75·2)	52·8 (33·6-81·4)	24·0 (7·5-60·8)	77·6 (68·3-84·7)	98·6 (97·5-99·2)	98·6 (97·5-99·3)	99·9 (99·3-100·0)	31·3 (29·9-32·7)	8·1 (7·3-9·1)	7·5 (6·6-8·6)	2·5 (1·9-3·4)	20·5 (19·9-21·1)	5·0 (4·4-5·7)	4·6 (4·0-5·3)	1·4 (1·0-2·0)
Mauritius	40·0 (35·1-45·4)	56·1 (46·1-67·9)	54·2 (41·7-70·3)	33·9 (10·1-84·8)	98·6 (97·7-99·3)	99·2 (98·4-99·6)	99·3 (98·5-99·7)	99·6 (98·5-100·0)	18·0 (17·4-18·7)	14·3 (13·0-15·6)	13·9 (12·5-15·6)	11·2 (8·3-14·7)	11·9 (11·3-12·6)	9·0 (8·3-9·8)	8·8 (8·0-9·6)	6·9 (5·4-8·9)
Myanmar	400·4 (331·3-489·2)	252·2 (200·3-309·5)	235·7 (185·5-290·3)	148·9 (79·3-250·2)	69·2 (63·7-74·6)	82·7 (74·2-89·0)	83·7 (75·1-90·1)	93·1 (79·7-99·3)	83·4 (67·8-100·9)	30·1 (22·7-40·2)	27·7 (20·8-37·5)	9·1 (4·1-17·2)	34·9 (28·3-42·5)	16·1 (12·1-21·5)	15·0 (11·3-20·3)	6·4 (3·1-11·8)
Philippines	84·4 (74·7-94·5)	79·1 (61·1-98·5)	76·5 (57·3-98·4)	48·0 (19·8-91·9)	61·2 (58·8-63·3)	72·4 (61·4-81·3)	73·1 (62·0-82·5)	83·9 (63·2-97·1)	38·7 (35·9-41·5)	25·1 (21·4-29·5)	24·1 (20·4-28·6)	15·8 (11·1-21·9)	16·3 (15·2-17·4)	12·0 (10·3-14·1)	11·6 (9·8-13·7)	8·4 (5·9-11·7)
Sri Lanka	61·0 (51·2-72·5)	36·8 (24·7-51·7)	35·2 (22·6-50·3)	21·2 (6·4-50·4)	99·3 (98·9-99·6)	99·9 (99·8-99·9)	99·9 (99·8-99·9)	100·0 (99·9-100·0)	16·9 (16·4-17·4)	7·4 (5·6-9·7)	6·8 (5·1-9·0)	2·0 (0·7-4·8)	9·7 (9·4-10·0)	4·4 (3·3-5·7)	4·0 (3·0-5·2)	1·1 (0·4-2·5)
Seychelles	41·4 (32·1-52·5)	30·0 (22·5-40·5)	30·1 (21·2-43·2)	26·2 (9·6-57·9)	93·1 (87·1-96·6)	97·3 (94·9-98·8)	97·4 (94·9-98·8)	98·7 (95·9-99·8)	13·4 (11·9-15·0)	12·1 (10·1-14·5)	11·4 (9·3-13·9)	10·4 (6·6-15·3)	9·3 (8·3-10·3)	8·3 (6·9-9·8)	7·8 (6·4-9·4)	7·3 (4·9-10·4)
Thailand	74·6 (52·1-103·8)	29·9 (20·4-41·5)	29·1 (19·4-40·8)	16·1 (6·1-34·7)	95·9 (93·9-97·5)	99·1 (98·6-99·4)	99·1 (98·6-99·5)	99·7 (99·0-100·0)	15·6 (13·1-18·7)	6·8 (5·4-8·4)	6·4 (5·1-8·0)	2·9 (1·8-4·5)	8·3 (7·1-9·8)	3·5 (2·8-4·4)	3·3 (2·6-4·1)	1·5 (1·0-2·3)
Timor-Leste	792·5 (470·7-989·7)	281·2 (123·7-408·7)	281·4 (123·3-417·0)	164·5 (41·5-348·7)	15·8 (13·0-18·8)	48·7 (36·8-61·2)	49·1 (36·4-62·1)	73·0 (44·9-92·1)	99·8 (83·6-117·5)	36·8 (22·0-58·7)	35·3 (21·1-55·4)	15·2 (3·7-42·3)	31·2 (25·2-38·9)	15·7 (8·4-26·8)	15·2 (8·1-25·4)	8·3 (1·6-25·5)
Vietnam	37·2 (25·2-54·1)	19·2 (12·9-29·1)	18·7 (12·5-28·1)	10·7 (4·1-25·9)	86·2 (83·2-89·1)	95·1 (92·7-96·9)	95·4 (92·9-97·2)	98·5 (95·3-99·8)	27·7 (25·5-30·0)	13·8 (11·6-16·7)	13·1 (10·9-16·1)	6·8 (4·7-9·8)	14·2 (13·2-15·3)	7·3 (6·2-8·8)	7·0 (5·8-8·5)	3·5 (2·4-5·1)
**Oceania**																
Federated States of Micronesia	147·5 (96·2-210·9)	133·6 (78·3-206·1)	128·9 (72·6-199·0)	121·2 (40·2-257·5)	91·8 (86·9-95·3)	97·9 (96·0-99·1)	98·1 (96·2-99·1)	99·2 (96·7-100·0)	35·2 (23·1-52·4)	18·3 (11·8-27·5)	17·3 (11·2-26·1)	12·6 (4·4-29·4)	14·5 (11·0-18·9)	9·5 (5·9-14·9)	9·0 (5·7-13·9)	6·8 (2·5-15·1)
Fiji	142·6 (101·4-193·5)	135·4 (80·9-213·7)	137·8 (81·7-222·0)	167·5 (38·0-489·9)	98·7 (97·5-99·4)	99·0 (98·0-99·5)	99·0 (98·0-99·5)	99·3 (96·9-100·0)	30·7 (24·3-38·4)	37·8 (23·1-58·4)	37·8 (22·4-61·0)	42·4 (10·9-110·7)	14·1 (12·0-16·4)	17·1 (11·0-25·6)	17·0 (10·6-26·3)	17·5 (4·9-43·8)

Location	Indicator 3.3.1: Age-standardised rate of new HIV infections (per 1,000 population)				Indicator 3.3.2: Age-standardised rate of tuberculosis cases (per 100,000 population)				Indicator 3.3.3: Age-standardised rate of malaria cases (per 1,000 population)				Indicator 3.3.4: Age-standardised rate of hepatitis B incidence (per 100,000 population)			
	**2000**	**2015**	**2016**	**2030**	**2000**	**2015**	**2016**	**2030**	**2000**	**2015**	**2016**	**2030**	**2000**	**2015**	**2016**	**2030**
Guyana	1·6 (1·1-2·4)	1·1 (0·7-2·1)	0·9 (0·6-1·4)	0·7 (0·4-1·0)	76·2 (65·9-87·3)	76·5 (65·4-88·8)	74·1 (63·4-86·1)	54·5 (45·3-63·3)	66·1 (50·1-85·2)	37·1 (30·1-46·5)	39·3 (33·2-47·3)	20·2 (14·5-22·3)	940·3 (705·1-1,245·9)	642·1 (484·5-842·5)	628·2 (475·3-821·5)	451·6 (332·7-592·2)
Haiti	3·1 (2·2-4·2)	0·1 (0·07-0·3)	0·2 (0·1-0·5)	0·1 (0·05-0·3)	168·5 (145·5-193·0)	107·4 (93·4-122·8)	103·8 (90·3-118·9)	69·1 (59·7-79·1)	11·2 (7·6-13·1)	5·2 (3·4-6·6)	3·9 (2·6-4·9)	2·7 (1·3-10·3)	1,160·9 (887·2-1,510·5)	756·5 (570·1-995·6)	736·3 (553·5-970·3)	498·1 (371·8-664·1)
Jamaica	0·3 (0·3-0·4)	0·3 (0·2-0·4)	0·2 (0·2-0·3)	0·2 (0·2-0·3)	16·1 (13·6-18·8)	9·8 (8·4-11·4)	9·7 (8·4-11·3)	8·7 (7·4-10·2)	0·0 (0·0-0·0)	0·0 (0·0-0·0)	0·0 (0·0-0·0)	0·0 (0·0-0·0)	230·5 (172·0-294·9)	211·7 (160·0-272·0)	210·8 (159·1-270·8)	198·2 (145·9-260·5)
Saint Lucia	0·09 (0·08-0·1)	0·06 (0·04-0·09)	0·06 (0·04-0·08)	0·06 (0·05-0·08)	42·5 (35·6-50·2)	29·2 (24·5-34·4)	28·4 (23·9-33·5)	19·8 (16·7-23·5)	0·0 (0·0-0·0)	0·0 (0·0-0·0)	0·0 (0·0-0·0)	0·0 (0·0-0·0)	232·4 (175·2-301·3)	156·0 (119·7-198·6)	153·5 (117·7-194·8)	121·2 (94·1-151·9)
Saint Vincent and the Grenadines	0·3 (0·3-0·4)	0·2 (0·2-0·3)	0·2 (0·2-0·3)	0·2 (0·2-0·3)	44·9 (37·3-53·2)	32·1 (27·0-37·9)	31·7 (26·7-37·5)	26·8 (22·5-31·7)	0·0 (0·0-0·0)	0·0 (0·0-0·0)	0·0 (0·0-0·0)	0·0 (0·0-0·0)	179·0 (139·9-228·8)	123·9 (98·3-154·8)	121·7 (96·7-151·7)	93·5 (76·7-112·8)
Suriname	0·6 (0·5-0·7)	0·4 (0·3-0·6)	0·4 (0·3-0·6)	0·3 (0·2-0·4)	43·3 (37·1-50·4)	31·9 (27·3-36·9)	30·7 (26·3-35·5)	19·8 (16·9-23·2)	40·8 (20·3-56·4)	1·2 (0·7-1·9)	0·8 (0·5-1·4)	0·06 (0·02-0·08)	456·1 (350·3-585·5)	287·2 (223·0-366·2)	279·9 (217·6-357·3)	193·9 (151·8-247·6)
Trinidad and Tobago	0·4 (0·4-0·6)	0·2 (0·1-0·3)	0·2 (0·1-0·3)	0·1 (0·08-0·2)	21·3 (17·9-24·8)	15·9 (13·6-18·4)	15·6 (13·4-18·1)	12·2 (10·5-14·2)	0·0 (0·0-0·0)	0·0 (0·0-0·0)	0·0 (0·0-0·0)	0·0 (0·0-0·0)	288·2 (219·9-374·6)	242·6 (182·5-320·8)	240·8 (181·4-317·8)	214·0 (160·9-278·8)
**Tropical Latin America**																
Brazil	0·1 (0·1-0·2)	0·2 (0·1-0·2)	0·2 (0·1-0·2)	0·1 (0·1-0·2)	47·7 (41·9-54·0)	36·0 (32·3-39·7)	35·3 (31·7-39·0)	26·9 (23·7-30·1)	14·6 (12·1-17·9)	2·1 (1·6-2·8)	1·9 (1·5-2·7)	0·6 (0·5-1·2)	839·4 (659·3-1,052·8)	585·3 (464·7-728·6)	572·5 (455·3-712·5)	418·0 (338·0-515·9)
Paraguay	0·08 (0·05-0·1)	0·06 (0·05-0·09)	0·06 (0·04-0·08)	0·06 (0·05-0·09)	49·4 (42·5-56·2)	46·0 (39·8-52·7)	45·6 (39·3-52·2)	39·2 (33·7-44·9)	0·0 (0·0-0·0)	0·0 (0·0-0·0)	0·0 (0·0-0·0)	0·0 (0·0-0·0)	303·6 (237·3-385·0)	340·1 (265·4-434·9)	336·1 (262·8-429·3)	284·9 (224·2-357·5)
**East Asia**																
China	0·03 (0·02-0·05)	0·03 (0·01-0·06)	0·03 (0·01-0·06)	0·02 (0·007-0·04)	129·1 (121·1-137·9)	80·8 (76·4-85·6)	80·8 (76·5-85·5)	53·7 (51·0-56·7)	0·07 (0·05-0·07)	0·0 (0·0-0·001)	0·0 (0·0-0·001)	0·0 (0·0-0·0)	1,552·0 (1,247·9-1,892·8)	559·9 (462·2-667·8)	546·9 (451·9-652·4)	312·0 (252·4-378·4)
North Korea	0·03 (0·002-0·2)	0·04 (0·002-0·2)	0·04 (0·002-0·2)	0·04 (0·002-0·2)	270·9 (236·1-307·4)	278·5 (247·2-312·1)	285·3 (253·5-319·3)	394·4 (354·0-436·0)	3·1 (3·1-3·1)	0·2 (0·2-0·2)	0·2 (0·2-0·2)	0·04 (0·04-0·04)	1,304·5 (1,000·1-1,654·3)	1,149·3 (886·0-1,451·1)	1,136·3 (876·5-1,433·5)	984·0 (763·0-1,250·4)
Taiwan (Province of China)	0·01 (0·007-0·02)	0·02 (0·006-0·03)	0·02 (0·006-0·03)	0·009 (0·003-0·02)	38·9 (35·6-42·6)	23·7 (21·6-26·1)	23·4 (21·4-25·8)	14·5 (13·2-16·1)	0·0 (0·0-0·0)	0·0 (0·0-0·0)	0·0 (0·0-0·0)	0·0 (0·0-0·0)	836·8 (668·3-1,025·6)	698·1 (556·2-859·3)	698·4 (555·8-859·1)	558·4 (443·6-698·3)
**Southeast Asia**																
Cambodia	0·5 (0·1-1·2)	0·06 (0·01-0·2)	0·05 (0·007-0·1)	0·03 (0·004-0·09)	579·4 (507·0-656·4)	278·3 (245·8-313·1)	262·5 (232·0-295·4)	119·3 (104·3-136·4)	33·9 (23·0-62·2)	2·9 (2·3-3·6)	2·7 (2·0-3·3)	0·5 (0·3-0·7)	6,969·0 (5,253·6-9,180·0)	3,860·4 (2,889·9-4,986·7)	3,725·3 (2,787·5-4,825·1)	2,216·7 (1,629·9-2,848·8)
Indonesia	0·02 (0·02-0·03)	0·08 (0·05-0·1)	0·07 (0·04-0·1)	0·07 (0·05-0·1)	504·3 (457·2-558·2)	357·1 (323·5-396·0)	351·3 (318·0-389·9)	270·1 (243·0-300·3)	6·3 (4·9-7·6)	4·1 (2·7-6·0)	3·6 (2·3-5·4)	1·7 (1·3-2·2)	3,306·6 (2,576·0-4,230·1)	1,807·8 (1,421·4-2,270·6)	1,744·7 (1,374·1-2,188·3)	1,024·2 (794·5-1,278·6)
Laos	0·1 (0·006-0·8)	0·09 (0·004-0·5)	0·09 (0·004-0·5)	0·09 (0·004-0·5)	328·3 (285·6-378·8)	174·6 (148·4-203·5)	166·5 (141·5-194·0)	85·1 (70·8-100·3)	26·4 (16·5-42·5)	9·2 (4·4-13·3)	10·4 (4·6-15·1)	2·9 (2·0-4·0)	1,953·2 (1,472·3-2,530·7)	1,099·3 (847·3-1,404·6)	1,051·1 (811·6-1,343·5)	583·4 (452·3-742·6)
Malaysia	0·1 (0·1-0·1)	0·05 (0·04-0·06)	0·05 (0·04-0·07)	0·04 (0·03-0·06)	89·2 (71·7-112·2)	61·9 (49·8-77·2)	60·1 (48·4-75·1)	40·4 (32·5-50·9)	1·5 (0·7-2·5)	0·01 (0·01-0·01)	0·007 (0·007-0·007)	0·0 (0·0-0·001)	687·6 (504·4-888·4)	675·5 (493·9-878·1)	668·6 (489·8-869·3)	579·4 (431·6-740·5)
Maldives	0·004 (0·003-0·004)	0·003 (0·002-0·005)	0·003 (0·002-0·005)	0·001 (0·001-0·002)	101·4 (81·2-128·1)	53·3 (42·4-67·1)	52·6 (41·8-66·4)	29·6 (23·3-38·0)	0·0 (0·0-0·0)	0·0 (0·0-0·0)	0·0 (0·0-0·0)	0·0 (0·0-0·0)	715·8 (561·9-904·7)	391·1 (311·3-486·8)	383·0 (304·7-476·2)	219·2 (174·9-272·6)
Mauritius	0·08 (0·05-0·1)	0·06 (0·04-0·1)	0·06 (0·03-0·09)	0·05 (0·03-0·09)	23·3 (18·7-28·7)	20·8 (16·4-25·9)	20·8 (16·4-25·9)	19·8 (15·3-25·1)	0·0 (0·0-0·0)	0·0 (0·0-0·0)	0·0 (0·0-0·0)	0·0 (0·0-0·0)	262·8 (211·4-327·8)	513·6 (392·1-681·6)	507·0 (387·9-672·1)	423·0 (327·5-548·1)
Myanmar	0·7 (0·6-0·8)	0·2 (0·1-0·2)	0·2 (0·1-0·2)	0·2 (0·1-0·2)	407·8 (328·3-509·7)	218·4 (176·0-275·5)	207·7 (167·2-262·3)	116·5 (92·4-148·3)	12·7 (9·8-15·5)	3·5 (2·6-4·4)	2·6 (1·9-3·2)	2·6 (2·1-3·0)	1,830·4 (1,388·3-2,448·8)	1,032·1 (795·1-1,345·9)	998·0 (770·3-1,299·4)	615·3 (476·0-795·2)
Philippines	0·07 (0·04-0·1)	0·2 (0·1-0·3)	0·2 (0·1-0·4)	0·2 (0·1-0·4)	301·6 (258·7-349·0)	251·0 (219·4-285·0)	260·3 (228·1-295·0)	374·2 (322·0-427·3)	1·0 (0·004-1·4)	0·06 (0·03-0·07)	0·05 (0·05-0·05)	0·004 (0·001-0·03)	1,890·6 (1,480·4-2,397·5)	992·6 (777·0-1,268·6)	981·1 (767·4-1,254·4)	828·2 (640·4-1,070·7)
Sri Lanka	0·006 (0·004-0·01)	0·009 (0·005-0·02)	0·01 (0·005-0·02)	0·01 (0·006-0·02)	73·7 (61·8-87·4)	41·8 (35·0-49·6)	40·6 (33·9-48·1)	26·0 (21·5-31·4)	9·0 (0·01-32·5)	0·0 (0·0-0·0)	0·0 (0·0-0·0)	0·0 (0·0-0·0)	264·1 (204·7-337·9)	171·5 (137·3-213·2)	169·4 (135·5-210·7)	141·6 (113·7-175·9)
Seychelles	0·07 (0·06-0·09)	0·1 (0·1-0·2)	0·1 (0·09-0·2)	0·1 (0·09-0·2)	45·8 (36·2-58·4)	33·2 (25·9-43·1)	32·9 (25·6-42·6)	27·8 (20·9-36·5)	0·0 (0·0-0·0)	0·0 (0·0-0·0)	0·0 (0·0-0·0)	0·0 (0·0-0·0)	1,041·3 (774·7-1,379·3)	748·5 (566·6-966·8)	751·2 (568·0-968·9)	725·3 (544·0-940·5)
Thailand	0·2 (0·06-0·4)	0·3 (0·2-0·5)	0·3 (0·2-0·4)	0·1 (0·09-0·2)	172·0 (137·5-216·0)	82·8 (66·1-102·4)	80·7 (64·6-99·8)	54·7 (44·1-67·7)	2·5 (1·6-3·5)	0·1 (0·1-0·1)	0·08 (0·08-0·08)	0·002 (0·001-0·004)	568·8 (446·2-723·7)	416·3 (336·6-518·7)	413·4 (334·7-514·0)	373·1 (307·8-456·1)
Timor-Leste	0·2 (0·01-1·3)	0·2 (0·008-0·9)	0·1 (0·007-0·8)	0·2 (0·008-0·8)	189·3 (152·4-231·1)	112·9 (90·7-139·5)	111·7 (89·7-138·2)	87·7 (70·1-109·1)	36·6 (24·7-48·8)	0·3 (0·2-0·3)	0·08 (0·05-0·1)	0·02 (0·01-0·02)	1,147·9 (885·8-1,488·5)	677·7 (538·0-862·2)	663·3 (526·8-842·2)	470·2 (372·5-593·5)
Vietnam	0·2 (0·1-0·3)	0·2 (0·1-0·3)	0·2 (0·1-0·2)	0·1 (0·09-0·2)	211·6 (185·0-242·7)	140·3 (120·8-163·5)	136·1 (116·9-158·7)	87·6 (73·4-105·0)	2·6 (0·002-4·0)	0·2 (0·07-0·2)	0·2 (0·2-0·2)	0·05 (0·02-0·1)	1,012·3 (788·2-1,283·7)	676·2 (536·9-843·2)	659·4 (523·4-819·1)	459·0 (364·7-565·4)
**Oceania**																
Federated States of Micronesia	0·05 (0·002-0·3)	0·7 (0·03-4·0)	0·6 (0·03-3·3)	1·8 (0·05-10·2)	184·1 (161·7-208·3)	145·0 (124·3-168·4)	147·1 (126·1-170·6)	112·4 (93·4-134·2)	0·0 (0·0-0·0)	0·0 (0·0-0·0)	0·0 (0·0-0·0)	0·0 (0·0-0·0)	1,063·8 (824·1-1,350·4)	952·3 (734·9-1,205·9)	945·9 (729·0-1,195·7)	847·9 (655·8-1,064·2)
Fiji	0·03 (0·02-0·04)	0·05 (0·03-0·09)	0·06 (0·03-0·1)	0·06 (0·04-0·1)	66·9 (59·4-75·1)	58·4 (51·7-65·4)	58·3 (51·6-65·4)	56·4 (49·5-64·1)	0·0 (0·0-0·0)	0·0 (0·0-0·0)	0·0 (0·0-0·0)	0·0 (0·0-0·0)	1,010·0 (780·6-1,289·0)	926·8 (730·5-1,173·0)	932·9 (735·1-1,180·4)	901·7 (707·3-1,150·8)

Location	**Indicator 3.3.5:**<br>**Age-standardised prevalence* of the sum of 15 neglected tropical diseases (NTDs) (%)**<br>**Prevalence estimates reported here may exceed 100% as they reflect the sum of prevalent cases of 15 NTDs.*				**Indicator 3.4.1:**<br>**Age-standardised death rate due to cardiovascular disease, cancer, diabetes, and chronic respiratory disease in populations aged 30-70 (per 100,000 population)**				**Indicator 3.4.2:**<br>**Age-standardised death rate due to self-harm (per 100,000 population)**				**Indicator 3.5.2:**<br>**Risk-weighted prevalence of alcohol consumption, as measured by the summary exposure value (SEV) for alcohol use (%)**			
	**2000**	**2015**	**2016**	**2030**	**2000**	**2015**	**2016**	**2030**	**2000**	**2015**	**2016**	**2030**	**2000**	**2015**	**2016**	**2030**
Guyana	33·3<br>(28·1-39·8)	28·2<br>(23·7-33·6)	28·0<br>(23·5-33·3)	24·6<br>(20·3-29·9)	797·6<br>(748·4-843·6)	686·3<br>(622·3-751·4)	651·2<br>(578·4-729·3)	569·2<br>(443·3-708·1)	24·7<br>(21·5-30·4)	31·9<br>(23·8-37·3)	30·4<br>(22·8-36·1)	26·9<br>(17·4-38·9)	15·5<br>(13·0-17·7)	12·5<br>(9·3-15·2)	12·5<br>(9·2-15·4)	12·4<br>(7·4-17·1)
Haiti	56·3<br>(51·7-61·2)	14·3<br>(12·2-16·6)	12·8<br>(10·9-14·9)	2·7<br>(2·2-3·2)	863·4<br>(760·1-971·6)	743·4<br>(591·1-906·5)	740·5<br>(595·0-900·3)	654·5<br>(435·2-929·7)	10·1<br>(7·2-13·4)	9·8<br>(7·1-13·3)	9·8<br>(7·0-13·3)	9·6<br>(5·5-15·7)	9·8<br>(7·8-11·8)	11·0<br>(7·8-14·3)	11·1<br>(7·9-14·5)	12·4<br>(8·2-17·0)
Jamaica	35·0<br>(32·2-37·9)	39·5<br>(35·9-43·4)	39·4<br>(35·8-43·3)	37·9<br>(34·4-41·9)	441·1<br>(407·1-477·8)	410·1<br>(350·0-471·2)	410·3<br>(342·9-477·2)	416·3<br>(254·2-610·9)	2·3<br>(1·9-2·7)	2·9<br>(2·2-3·7)	2·9<br>(2·2-3·7)	3·2<br>(1·6-5·7)	9·2<br>(6·8-11·5)	11·4<br>(7·8-14·8)	11·5<br>(7·9-15·0)	13·2<br>(8·7-17·6)
Saint Lucia	54·6<br>(49·9-59·1)	50·3<br>(42·6-58·9)	50·3<br>(42·6-58·8)	50·1<br>(42·3-58·7)	456·8<br>(438·2-477·3)	376·3<br>(354·7-400·9)	373·7<br>(351·3-398·9)	338·4<br>(291·8-390·8)	6·5<br>(5·0-7·5)	6·8<br>(5·2-8·0)	7·0<br>(5·1-8·9)	7·3<br>(4·7-10·6)	17·9<br>(15·6-20·5)	14·2<br>(9·9-18·4)	14·0<br>(9·8-18·2)	11·9<br>(7·4-16·5)
Saint Vincent and the Grenadines	24·2<br>(20·1-29·0)	30·7<br>(25·6-36·3)	30·7<br>(25·6-36·3)	30·6<br>(25·5-36·2)	567·5<br>(532·7-603·7)	533·2<br>(496·5-576·2)	529·2<br>(490·9-574·0)	503·9<br>(421·0-604·8)	6·9<br>(5·3-7·9)	7·5<br>(5·9-9·1)	7·6<br>(5·8-9·2)	8·2<br>(5·4-11·9)	8·8<br>(6·6-11·1)	10·9<br>(7·0-14·9)	10·9<br>(7·0-15·0)	11·9<br>(7·1-17·2)
Suriname	23·7<br>(21·4-26·4)	23·2<br>(20·6-26·0)	23·2<br>(20·5-26·0)	22·7<br>(20·1-25·4)	484·1<br>(465·4-504·4)	453·7<br>(420·2-483·2)	448·4<br>(411·7-481·0)	392·4<br>(327·2-464·4)	21·1<br>(18·6-23·8)	25·4<br>(20·6-28·6)	24·5<br>(19·6-28·0)	24·6<br>(16·9-33·3)	11·3<br>(8·7-14·2)	15·0<br>(10·7-19·0)	15·0<br>(10·8-19·1)	16·2<br>(11·3-20·9)
Trinidad and Tobago	4·6<br>(4·0-5·2)	4·6<br>(4·0-5·2)	4·6<br>(4·0-5·2)	4·8<br>(4·2-5·4)	652·3<br>(626·0-678·8)	472·5<br>(437·0-509·2)	470·9<br>(432·6-507·5)	444·2<br>(361·2-540·9)	13·8<br>(10·1-15·5)	12·0<br>(9·5-14·5)	12·0<br>(9·5-14·4)	11·5<br>(7·9-16·0)	6·1<br>(4·7-7·9)	7·8<br>(5·4-10·7)	8·0<br>(5·4-10·9)	9·9<br>(6·3-14·2)
**Tropical Latin America**																
Brazil	34·3<br>(27·3-42·8)	32·0<br>(27·3-37·3)	31·6<br>(26·9-36·9)	26·6<br>(22·2-31·7)	459·3<br>(448·0-469·5)	341·8<br>(332·2-352·2)	340·4<br>(329·5-352·4)	264·4<br>(246·2-285·2)	6·7<br>(5·8-8·2)	6·4<br>(5·4-8·0)	6·4<br>(5·3-7·9)	6·0<br>(4·5-7·9)	14·5<br>(11·9-16·9)	14·2<br>(11·1-17·6)	14·3<br>(11·1-17·7)	14·7<br>(10·9-19·0)
Paraguay	25·8<br>(22·7-29·5)	25·7<br>(22·6-29·6)	25·6<br>(22·5-29·5)	25·1<br>(22·0-28·9)	374·7<br>(356·1-392·4)	388·7<br>(358·2-423·0)	385·5<br>(350·1-426·2)	377·5<br>(287·0-475·2)	5·1<br>(4·2-5·8)	6·6<br>(4·8-7·9)	6·5<br>(4·7-7·9)	7·9<br>(4·7-11·7)	18·6<br>(14·9-22·4)	15·5<br>(10·7-20·3)	15·6<br>(10·7-20·5)	16·9<br>(10·4-23·3)
**East Asia**																
China	38·0<br>(30·9-47·5)	21·9<br>(19·1-25·5)	21·3<br>(18·7-24·8)	14·6<br>(13·2-16·2)	599·8<br>(588·0-611·8)	400·8<br>(391·1-410·4)	387·5<br>(378·1-397·2)	265·0<br>(247·4-283·4)	17·7<br>(15·5-18·5)	8·7<br>(8·2-9·7)	8·5<br>(8·0-9·5)	4·6<br>(4·0-5·9)	8·4<br>(6·8-10·1)	9·7<br>(8·3-11·2)	9·8<br>(8·3-11·3)	11·3<br>(9·3-13·3)
North Korea	27·8<br>(21·7-35·5)	14·6<br>(10·7-19·7)	13·8<br>(10·2-18·7)	6·6<br>(4·9-8·9)	621·2<br>(561·8-672·0)	630·5<br>(584·0-676·2)	627·2<br>(584·1-673·3)	600·7<br>(502·6-704·6)	13·2<br>(10·7-16·4)	12·3<br>(9·3-16·3)	12·2<br>(9·2-16·3)	11·5<br>(7·6-16·9)	6·7<br>(5·1-8·3)	5·5<br>(3·6-7·9)	5·6<br>(3·6-8·0)	6·1<br>(3·4-9·9)
Taiwan (Province of China)	41·9<br>(39·1-44·9)	28·0<br>(24·8-31·3)	27·8<br>(24·6-31·1)	24·8<br>(22·0-27·7)	380·9<br>(363·4-400·8)	284·1<br>(256·4-311·8)	283·1<br>(242·7-322·3)	225·3<br>(159·7-297·5)	13·9<br>(12·3-15·2)	13·6<br>(11·5-15·2)	13·6<br>(11·2-16·0)	11·9<br>(7·6-17·3)	8·8<br>(4·8-12·9)	9·4<br>(5·0-13·7)	9·5<br>(5·0-13·8)	10·4<br>(5·6-15·2)
**Southeast Asia**																
Cambodia	90·5<br>(84·1-97·6)	5·8<br>(5·4-6·4)	4·9<br>(4·5-5·4)	0·3<br>(0·3-0·4)	641·9<br>(594·6-691·6)	500·3<br>(457·7-543·6)	494·6<br>(451·4-539·9)	408·5<br>(340·6-489·6)	7·7<br>(6·4-8·8)	6·7<br>(5·6-7·7)	6·6<br>(5·5-7·6)	6·1<br>(4·3-8·1)	11·3<br>(9·1-13·8)	12·0<br>(9·7-14·8)	12·0<br>(9·7-14·8)	11·4<br>(9·1-14·1)
Indonesia	41·2<br>(36·9-46·2)	29·9<br>(26·7-33·7)	29·6<br>(26·3-33·3)	24·3<br>(21·4-27·7)	529·4<br>(501·8-562·0)	503·1<br>(480·0-527·2)	495·7<br>(472·9-520·6)	446·6<br>(394·5-499·8)	4·0<br>(3·6-4·5)	3·6<br>(3·2-4·2)	3·5<br>(3·2-4·2)	3·2<br>(2·7-4·1)	0·7<br>(0·6-0·9)	0·9<br>(0·6-1·1)	0·9<br>(0·7-1·1)	1·1<br>(0·8-1·4)
Laos	88·3<br>(78·2-99·9)	52·8<br>(45·3-61·2)	50·0<br>(42·9-57·9)	23·4<br>(19·7-27·4)	769·3<br>(684·7-826·0)	607·3<br>(553·0-647·7)	597·2<br>(545·2-636·8)	476·5<br>(407·1-536·9)	14·5<br>(9·4-18·1)	10·7<br>(7·4-13·5)	10·6<br>(7·3-13·3)	8·6<br>(5·2-12·0)	8·2<br>(6·1-10·6)	8·4<br>(5·3-12·1)	8·5<br>(5·3-12·3)	9·6<br>(4·9-16·0)
Malaysia	83·2<br>(74·8-91·5)	79·4<br>(70·4-89·1)	78·9<br>(70·0-88·5)	72·8<br>(63·6-82·1)	460·6<br>(443·1-488·8)	382·2<br>(358·3-411·7)	376·0<br>(351·1-405·9)	315·7<br>(274·2-358·4)	9·6<br>(8·6-11·0)	8·5<br>(7·3-10·2)	8·4<br>(7·2-10·2)	7·7<br>(5·7-10·5)	4·0<br>(3·3-4·7)	4·0<br>(3·2-4·7)	4·0<br>(3·2-4·7)	4·2<br>(3·4-5·0)
Maldives	0·4<br>(0·3-0·5)	0·4<br>(0·3-0·5)	0·6<br>(0·4-0·8)	0·6<br>(0·5-0·8)	481·4<br>(443·1-521·1)	265·6<br>(221·4-318·0)	264·6<br>(215·2-323·8)	202·7<br>(114·6-344·5)	6·3<br>(4·8-7·8)	3·9<br>(2·8-5·1)	3·9<br>(2·7-5·2)	3·1<br>(1·5-5·5)	2·7<br>(1·6-3·8)	2·5<br>(1·1-4·5)	2·4<br>(1·0-4·4)	1·9<br>(0·5-4·1)
Mauritius	5·9<br>(4·1-7·8)	6·8<br>(4·8-9·3)	6·9<br>(4·8-9·4)	8·1<br>(5·6-11·2)	634·6<br>(611·4-659·1)	448·9<br>(406·9-491·6)	446·4<br>(390·9-509·6)	348·3<br>(254·9-444·1)	11·5<br>(9·0-12·6)	8·4<br>(6·8-9·8)	8·3<br>(6·6-10·3)	7·1<br>(4·0-10·7)	9·2<br>(6·8-11·9)	7·9<br>(4·7-11·9)	7·9<br>(4·6-12·0)	7·8<br>(3·7-13·8)
Myanmar	101·5<br>(89·3-115·4)	41·7<br>(34·4-50·4)	37·0<br>(30·4-44·6)	6·8<br>(5·6-8·2)	700·3<br>(628·7-795·6)	512·8<br>(470·0-578·2)	503·1<br>(462·6-564·5)	377·8<br>(324·0-441·8)	8·4<br>(6·3-11·1)	6·0<br>(5·0-7·5)	5·9<br>(4·9-7·3)	4·5<br>(3·3-6·1)	2·5<br>(1·5-3·7)	3·6<br>(2·0-5·5)	3·7<br>(2·0-5·6)	5·6<br>(2·8-8·6)
Philippines	124·4<br>(117·3-132·2)	64·3<br>(56·3-73·4)	60·8<br>(53·2-69·4)	27·5<br>(24·2-31·6)	552·8<br>(521·6-583·8)	549·1<br>(480·9-618·1)	542·8<br>(470·7-620·4)	490·7<br>(336·8-670·9)	6·5<br>(5·5-10·0)	6·4<br>(5·0-10·2)	6·4<br>(4·9-10·0)	6·0<br>(3·3-10·8)	9·4<br>(7·3-11·6)	8·4<br>(5·3-11·6)	8·3<br>(5·2-11·7)	7·5<br>(3·9-11·9)
Sri Lanka	25·3<br>(22·2-29·0)	17·7<br>(15·1-20·8)	17·6<br>(14·9-20·7)	14·8<br>(12·3-17·9)	423·1<br>(393·7-452·6)	313·6<br>(254·6-381·3)	308·1<br>(246·6-378·3)	236·4<br>(136·8-363·9)	42·0<br>(37·3-46·1)	23·8<br>(18·5-31·6)	23·7<br>(18·1-31·9)	20·3<br>(9·9-39·1)	2·7<br>(1·8-3·9)	7·6<br>(4·9-10·2)	7·8<br>(5·0-10·6)	12·4<br>(7·9-16·5)
Seychelles	72·4<br>(60·4-86·2)	72·4<br>(60·2-86·3)	72·4<br>(60·2-86·3)	71·9<br>(59·8-85·6)	568·4<br>(535·3-608·5)	436·9<br>(389·4-489·5)	434·3<br>(382·5-502·7)	348·9<br>(251·1-492·3)	11·3<br>(9·9-13·3)	8·3<br>(7·0-10·9)	8·3<br>(6·9-10·9)	6·2<br>(4·0-10·1)	10·3<br>(8·7-12·0)	8·9<br>(5·7-11·8)	8·7<br>(5·6-11·6)	6·6<br>(3·6-9·5)
Thailand	34·5<br>(30·0-39·0)	28·9<br>(24·7-33·3)	28·9<br>(24·7-33·2)	28·3<br>(24·2-32·5)	404·5<br>(381·4-430·1)	288·0<br>(261·8-320·7)	285·8<br>(255·2-321·3)	237·4<br>(175·0-314·5)	16·0<br>(13·8-18·2)	10·9<br>(9·2-12·5)	10·9<br>(9·1-12·6)	9·3<br>(6·0-13·0)	11·2<br>(9·6-13·0)	11·4<br>(8·8-14·4)	11·4<br>(8·8-14·5)	11·3<br>(8·2-15·0)
Timor-Leste	48·4<br>(42·1-55·9)	42·6<br>(36·4-50·1)	42·2<br>(36·1-49·7)	37·3<br>(31·6-44·7)	486·5<br>(373·6-574·9)	388·9<br>(286·6-481·4)	392·1<br>(292·4-487·5)	350·3<br>(207·9-521·7)	10·1<br>(6·4-12·6)	7·5<br>(4·3-10·3)	7·6<br>(4·4-10·5)	6·5<br>(2·8-11·6)	0·6<br>(0·3-0·8)	0·6<br>(0·3-1·1)	0·6<br>(0·3-1·1)	0·7<br>(0·3-1·3)
Vietnam	98·3<br>(92·0-104·4)	42·6<br>(36·2-49·6)	37·8<br>(32·1-43·9)	7·0<br>(5·9-8·1)	521·2<br>(457·2-578·2)	433·7<br>(375·4-492·3)	427·7<br>(375·3-483·7)	354·2<br>(263·2-454·3)	10·6<br>(8·7-12·6)	8·3<br>(6·9-9·8)	8·2<br>(6·8-9·7)	6·8<br>(4·5-9·7)	9·1<br>(6·0-12·7)	18·0<br>(13·4-21·7)	18·1<br>(13·4-21·8)	18·7<br>(13·3-22·8)
**Oceania**																
Federated States of Micronesia	57·0<br>(48·3-66·4)	56·0<br>(45·5-68·7)	56·4<br>(45·9-69·3)	58·4<br>(47·4-71·9)	931·8<br>(773·8-1,119·4)	1,009·7<br>(773·1-1,290·1)	989·0<br>(751·2-1,250·1)	1,022·7<br>(631·1-1,538·7)	20·1<br>(14·9-25·2)	19·7<br>(13·2-26·7)	19·4<br>(12·9-26·2)	18·5<br>(9·9-30·2)	3·6<br>(2·6-4·8)	2·8<br>(1·7-4·5)	2·8<br>(1·6-4·5)	2·7<br>(1·3-5·0)
Fiji	65·8<br>(57·8-74·8)	64·1<br>(55·0-75·5)	63·9<br>(54·8-75·3)	58·9<br>(49·9-69·9)	1,145·4<br>(1,015·2-1,308·0)	1,010·2<br>(770·4-1,256·6)	1,006·9<br>(767·4-1,263·3)	960·9<br>(481·0-1,714·0)	13·9<br>(11·5-16·5)	12·5<br>(9·2-16·3)	12·6<br>(8·9-16·9)	13·8<br>(5·7-28·3)	5·2<br>(3·9-6·6)	7·2<br>(4·8-9·6)	7·3<br>(4·8-9·7)	8·7<br>(5·4-12·2)

Location	Indicator 3.6.1: Age-standardised death rate due to road injuries (per 100,000 population)				Indicator 3.7.1: Proportion of women of reproductive age (15-49 years) who have their need for family planning satisfied with modern contraception methods (%)				Indicator 3.7.2: Number of livebirths per 1,000 women aged 10-14 years and women aged 15-19 years				Indicator 3.8.1: Coverage of essential health services, as defined by the UHC index comprised of the coverage of 9 tracer interventions and risk-standardised death rates from 32 causes amenable to personal healthcare (scale of 0 to 100)			
	**2000**	**2015**	**2016**	**2030**	**2000**	**2015**	**2016**	**2030**	**2000**	**2015**	**2016**	**2030**	**2000**	**2015**	**2016**	**2030**
Guyana	20·9 (19·3-22·9)	23·4 (20·1-26·7)	22·1 (19·0-25·4)	16·0 (11·0-22·4)	60·0 (52·8-67·3)	51·3 (47·5-55·2)	52·5 (47·9-56·8)	46·9 (36·2-58·0)	47·8 (39·9-56·5)	41·2 (33·8-49·4)	41·9 (34·3-50·4)	41·6 (25·6-63·3)	43·9 (41·9-45·5)	49·6 (47·4-52·0)	50·6 (48·3-52·9)	59·7 (56·2-63·2)
Haiti	33·6 (28·0-42·2)	28·4 (22·0-37·7)	28·2 (21·8-37·4)	24·8 (15·6-39·2)	32·4 (29·4-35·5)	46·6 (41·7-51·7)	47·1 (41·4-52·6)	62·6 (52·5-73·1)	29·9 (23·9-35·7)	23·7 (16·8-31·1)	23·6 (16·4-31·5)	19·0 (8·6-34·8)	28·8 (26·2-31·5)	39·7 (36·3-43·4)	40·4 (36·9-44·1)	51·1 (47·1-55·3)
Jamaica	6·6 (5·8-7·5)	9·6 (7·9-11·5)	9·5 (7·7-11·5)	8·7 (4·8-14·6)	74·4 (71·0-77·4)	78·3 (71·8-83·3)	78·5 (72·4-83·7)	82·6 (70·9-91·0)	49·0 (36·7-63·8)	36·7 (26·1-50·3)	35·7 (25·5-49·6)	27·6 (12·7-52·5)	55·9 (53·7-58·2)	61·1 (57·9-64·3)	61·1 (57·9-64·4)	64·5 (60·4-69·0)
Saint Lucia	16·0 (14·3-17·5)	14·4 (12·9-16·1)	14·9 (12·8-17·2)	13·3 (9·9-17·5)	71·7 (65·2-77·8)	79·1 (73·3-83·7)	79·3 (73·6-84·0)	83·3 (73·0-90·7)	29·9 (27·2-32·9)	19·1 (14·3-25·6)	18·7 (14·0-25·1)	11·8 (6·3-21·1)	54·9 (53·1-56·7)	59·0 (56·7-61·2)	59·4 (57·0-61·5)	62·5 (59·3-65·5)
Saint Vincent and the Grenadines	10·8 (9·9-11·8)	12·8 (11·4-14·3)	12·3 (10·8-13·9)	13·5 (9·9-17·8)	67·6 (60·4-74·5)	75·6 (68·9-81·3)	75·9 (69·6-81·7)	80·1 (68·1-88·9)	35·8 (30·4-41·9)	27·2 (23·0-32·0)	26·4 (21·6-31·6)	18·0 (11·4-27·5)	52·1 (50·3-53·8)	54·7 (52·5-56·7)	55·0 (52·8-57·1)	56·5 (53·7-59·4)
Suriname	19·8 (18·5-21·3)	21·3 (19·2-23·6)	21·3 (19·0-23·8)	21·3 (15·7-27·6)	64·0 (57·8-70·4)	67·6 (62·0-72·9)	68·5 (62·3-74·3)	71·8 (60·8-80·8)	30·1 (23·8-37·4)	21·9 (16·7-28·2)	21·6 (16·4-28·0)	16·3 (8·9-27·6)	48·7 (46·8-50·5)	55·2 (52·9-57·5)	55·9 (53·5-58·1)	63·0 (59·9-65·8)
Trinidad and Tobago	17·5 (16·1-18·8)	17·0 (15·0-19·4)	16·8 (14·6-19·2)	14·2 (10·5-18·2)	59·6 (53·2-65·8)	70·7 (63·8-77·2)	71·0 (63·5-77·5)	76·5 (63·7-85·9)	21·7 (17·7-26·1)	10·4 (6·7-15·2)	9·7 (6·3-14·5)	4·0 (1·3-9·3)	51·1 (49·2-52·7)	58·1 (55·1-60·8)	58·4 (55·3-61·2)	64·4 (59·7-68·2)
**Tropical Latin America**																
Brazil	26·7 (25·3-28·6)	23·0 (21·6-25·6)	22·7 (21·2-25·3)	19·9 (17·3-23·5)	81·2 (80·2-82·3)	86·6 (85·5-87·5)	86·8 (85·7-87·8)	90·5 (89·0-91·9)	40·4 (40·0-40·8)	34·3 (33·2-35·4)	34·2 (32·9-35·4)	30·0 (28·0-32·2)	55·3 (54·2-56·3)	61·8 (60·4-62·8)	62·0 (60·7-63·0)	67·0 (65·5-68·4)
Paraguay	19·1 (17·7-20·7)	27·0 (23·8-30·7)	26·4 (22·9-30·4)	32·7 (22·2-46·6)	72·2 (68·5-75·7)	85·5 (81·7-88·9)	85·7 (81·9-89·1)	92·3 (87·7-95·6)	41·0 (38·4-43·4)	26·2 (23·7-28·8)	25·6 (23·1-28·2)	17·1 (13·7-21·1)	52·9 (51·1-54·5)	55·6 (53·3-57·9)	55·9 (53·5-58·3)	60·3 (56·8-63·6)
**East Asia**																
China	27·2 (26·4-28·5)	20·9 (20·2-21·6)	20·4 (19·7-21·1)	14·6 (13·1-16·1)	86·9 (82·8-90·3)	91·3 (88·3-93·7)	91·5 (88·7-93·9)	94·2 (89·9-97·1)	3·6 (3·2-4·0)	2·6 (2·0-3·3)	2·4 (1·8-3·2)	1·3 (0·6-2·4)	53·1 (51·8-54·4)	68·6 (67·3-69·7)	69·2 (67·9-70·4)	79·2 (77·4-80·7)
North Korea	25·2 (21·0-29·4)	27·9 (22·6-33·4)	27·8 (22·6-33·3)	26·9 (17·8-37·4)	72·8 (66·3-78·8)	73·2 (66·6-79·0)	73·4 (67·0-79·3)	73·8 (60·0-84·2)	1·0 (0·8-1·1)	0·4 (0·4-0·5)	0·4 (0·3-0·5)	0·2 (0·1-0·2)	52·1 (49·3-54·3)	56·2 (53·6-58·7)	56·4 (53·7-58·8)	58·9 (55·6-61·8)
Taiwan (Province of China)	22·9 (21·4-24·6)	14·6 (13·1-16·3)	14·7 (12·6-17·0)	10·8 (7·5-14·4)	89·5 (86·1-92·2)	91·2 (88·2-93·6)	91·3 (88·4-93·7)	93·1 (87·7-96·6)	8·1 (8·0-8·2)	3·0 (2·8-3·3)	2·9 (2·6-3·2)	1·6 (0·7-3·2)	64·4 (63·1-65·7)	72·3 (70·1-74·5)	72·4 (70·2-74·7)	76·9 (73·3-80·5)
**Southeast Asia**																
Cambodia	32·0 (28·2-38·2)	27·8 (24·2-31·8)	27·5 (23·9-31·4)	24·2 (18·1-30·4)	31·8 (30·0-33·6)	57·4 (55·1-59·7)	58·0 (54·8-61·2)	76·3 (69·4-82·1)	24·0 (22·3-25·6)	28·0 (25·4-30·8)	28·4 (25·6-31·4)	34·4 (25·2-45·5)	29·7 (28·0-31·6)	49·3 (47·5-51·2)	50·2 (48·3-52·0)	65·0 (62·0-67·5)
Indonesia	29·0 (27·0-31·8)	18·7 (17·4-20·9)	18·4 (17·1-20·6)	14·6 (12·7-17·2)	76·6 (75·6-77·7)	78·4 (77·6-79·2)	79·0 (78·0-79·8)	82·2 (79·9-84·4)	27·0 (24·7-29·6)	22·1 (18·8-25·5)	21·7 (18·4-25·4)	17·4 (12·0-24·4)	41·4 (40·4-42·5)	49·7 (48·4-51·0)	50·4 (49·0-51·8)	59·6 (57·1-61·8)
Laos	30·4 (26·1-36·5)	25·4 (21·4-29·9)	25·0 (21·1-29·4)	20·3 (15·3-26·1)	47·4 (40·4-54·7)	61·7 (53·4-68·9)	62·3 (54·2-69·7)	73·6 (60·4-83·9)	41·0 (33·3-48·5)	45·5 (33·3-58·1)	45·2 (33·3-58·0)	46·1 (23·7-77·6)	26·6 (24·8-28·7)	42·5 (39·7-45·4)	43·6 (40·8-46·6)	61·0 (57·1-64·8)
Malaysia	28·3 (26·2-30·5)	23·1 (20·5-25·8)	22·5 (19·8-25·3)	18·2 (14·2-23·3)	64·1 (56·7-71·1)	77·0 (69·8-82·9)	77·4 (70·3-83·1)	86·3 (76·5-93·2)	7·1 (7·0-7·2)	7·0 (6·4-7·8)	7·2 (6·4-8·1)	7·6 (5·7-10·1)	56·0 (54·8-57·3)	63·8 (62·3-65·2)	64·5 (62·9-66·0)	71·7 (69·8-73·7)
Maldives	15·8 (14·0-18·0)	9·2 (7·4-11·6)	9·1 (7·1-11·6)	6·5 (3·6-11·0)	56·4 (48·9-63·4)	72·4 (65·7-78·4)	72·9 (66·5-78·9)	83·9 (75·0-90·7)	11·9 (11·1-12·7)	2·4 (2·0-2·7)	1·9 (1·7-2·2)	0·4 (0·3-0·6)	57·3 (55·2-59·4)	72·0 (69·0-75·0)	72·5 (69·2-75·6)	79·3 (74·2-83·8)
Mauritius	14·9 (14·1-16·0)	11·1 (9·9-12·2)	10·8 (9·3-12·6)	8·2 (5·4-11·8)	66·9 (59·7-73·9)	75·6 (68·9-81·2)	75·8 (69·3-81·2)	81·9 (71·2-90·6)	18·3 (18·0-18·7)	13·8 (12·5-15·1)	13·8 (12·1-15·7)	10·9 (8·4-13·8)	57·4 (55·9-58·6)	64·6 (62·2-66·9)	65·0 (62·5-67·5)	70·8 (67·0-74·6)
Myanmar	36·8 (31·1-45·5)	28·2 (24·4-33·8)	27·6 (24·0-32·9)	20·8 (16·0-26·2)	53·5 (46·5-60·6)	76·2 (74·1-78·1)	76·8 (74·3-79·0)	89·8 (85·5-92·9)	12·3 (9·9-14·9)	7·8 (6·2-9·9)	7·6 (6·0-9·8)	5·5 (3·3-8·6)	34·9 (32·5-37·1)	49·0 (46·4-51·1)	50·0 (47·5-52·2)	64·5 (62·2-66·8)
Philippines	12·7 (11·8-13·6)	11·5 (10·0-13·2)	11·3 (9·7-13·0)	8·9 (5·9-12·9)	44·7 (42·2-47·1)	53·3 (50·0-56·7)	54·1 (49·6-58·5)	66·1 (55·1-75·4)	23·4 (22·3-24·5)	32·2 (30·2-34·2)	33·3 (31·1-35·5)	44·6 (38·2-52·5)	44·4 (42·8-45·8)	49·0 (46·7-51·2)	49·8 (47·3-52·1)	56·3 (51·9-60·6)
Sri Lanka	18·5 (16·9-20·2)	14·7 (11·7-18·1)	14·4 (11·3-18·2)	11·4 (6·3-18·8)	66·9 (60·1-73·2)	77·0 (71·0-82·3)	77·5 (71·4-82·8)	86·1 (76·7-92·5)	14·9 (14·7-15·1)	5·9 (5·1-6·7)	5·3 (4·6-6·1)	2·3 (1·5-3·0)	57·7 (56·1-59·3)	67·9 (64·6-71·2)	68·6 (65·1-72·1)	77·7 (72·2-82·5)
Seychelles	18·8 (16·9-20·8)	14·3 (12·5-16·4)	14·2 (12·1-16·7)	12·1 (8·2-17·1)	75·4 (68·9-80·8)	79·5 (73·9-84·5)	79·8 (74·1-84·7)	83·8 (74·1-91·2)	27·3 (26·9-27·6)	28·7 (27·5-30·0)	27·3 (25·5-29·2)	29·0 (26·2-32·0)	53·5 (51·9-55·1)	59·1 (56·6-61·5)	59·4 (56·9-61·8)	63·9 (59·8-67·5)
Thailand	43·8 (39·8-48·0)	26·2 (23·4-29·3)	26·0 (22·7-29·6)	17·6 (12·6-24·5)	85·5 (81·6-88·9)	86·4 (84·0-88·6)	86·7 (83·9-89·2)	90·2 (83·2-94·7)	25·2 (19·9-31·0)	18·7 (14·7-23·4)	18·4 (14·0-23·6)	13·6 (7·1-23·3)	56·9 (55·4-58·5)	67·7 (65·7-69·8)	68·2 (66·1-70·3)	74·6 (71·8-77·4)
Timor-Leste	14·5 (10·7-18·4)	12·3 (8·6-16·2)	12·3 (8·6-16·0)	10·7 (6·2-16·4)	31·2 (24·7-38·6)	48·0 (41·2-55·0)	48·9 (41·7-56·4)	66·9 (51·0-81·1)	18·8 (14·6-22·8)	16·1 (12·5-19·9)	15·4 (11·8-19·3)	11·3 (5·7-19·0)	30·1 (26·9-34·7)	45·3 (41·3-50·6)	45·7 (41·6-51·0)	55·3 (49·7-60·7)
Vietnam	29·0 (24·5-33·3)	26·2 (21·7-30·7)	25·7 (21·3-30·1)	20·4 (13·4-29·0)	69·0 (66·3-71·4)	72·0 (68·6-75·3)	72·9 (68·7-76·7)	80·2 (71·0-87·6)	14·0 (12·6-15·6)	19·0 (15·3-23·3)	19·0 (15·5-23·0)	20·8 (13·8-31·0)	49·2 (47·0-51·5)	60·4 (58·0-62·9)	61·1 (58·6-63·6)	70·4 (67·5-73·3)
**Oceania**																
Federated States of Micronesia	20·4 (16·5-24·8)	20·5 (15·3-27·1)	20·1 (14·8-26·3)	19·8 (11·7-30·8)	61·0 (52·9-68·7)	72·6 (65·5-78·9)	72·8 (65·8-79·0)	80·5 (67·3-89·0)	28·3 (25·9-30·6)	11·6 (9·0-14·9)	11·1 (8·6-14·5)	6·0 (3·3-10·2)	40·7 (37·2-44·0)	44·6 (40·2-49·4)	45·1 (40·7-50·0)	49·3 (43·5-56·0)
Fiji	16·2 (14·1-18·5)	13·0 (10·0-16·3)	13·1 (9·8-16·6)	14·4 (7·0-26·9)	73·4 (66·6-79·3)	81·8 (76·3-86·2)	82·0 (76·5-86·3)	87·3 (78·0-93·3)	14·8 (14·7-14·8)	12·8 (12·7-12·9)	12·9 (12·8-13·0)	12·0 (11·7-12·2)	43·6 (41·3-45·8)	46·5 (43·1-50·4)	46·5 (42·9-50·6)	48·6 (43·4-55·0)

Location	Indicator 3.9.1: Age-standardised death rate attributable to household air pollution and ambient air pollution (per 100,000 population)				Indicator 3.9.2: Age-standardised death rate attributable to unsafe water, sanitation, and hygiene (WaSH) (per 100,000 population)				Indicator 3.9.3: Age-standardised death rate due to unintentional poisonings (per 100,000 population)				Indicator 3.a.1: Age-standardised prevalence of daily smoking in populations aged 10 and older (%)			
	**2000**	**2015**	**2016**	**2030**	**2000**	**2015**	**2016**	**2030**	**2000**	**2015**	**2016**	**2030**	**2000**	**2015**	**2016**	**2030**
Guyana	98·1 (83·8-113·1)	75·3 (61·0-91·4)	73·9 (59·8-89·7)	56·5 (44·8-68·9)	26·7 (22·0-31·1)	14·4 (10·9-18·0)	13·8 (10·3-17·3)	7·3 (4·7-10·5)	1·0 (0·9-1·4)	0·7 (0·5-1·0)	0·6 (0·5-1·0)	0·4 (0·2-0·7)	12·0 (10·7-13·4)	11·1 (9·9-12·4)	11·1 (9·8-12·4)	10·4 (8·2-12·7)
Haiti	293·2 (255·9-332·1)	225·3 (189·0-268·6)	222·2 (186·3-265·2)	178·7 (143·8-219·8)	76·6 (54·6-108·0)	55·4 (36·9-83·6)	50·6 (33·1-76·9)	23·4 (10·5-52·8)	2·9 (1·6-4·8)	1·7 (0·9-2·8)	1·6 (0·9-2·7)	1·0 (0·4-1·9)	7·8 (7·0-8·7)	5·2 (4·6-5·7)	5·2 (4·6-5·8)	4·0 (3·0-5·1)
Jamaica	52·4 (45·3-60·0)	36·7 (29·2-44·9)	36·5 (28·9-44·8)	25·6 (16·8-34·9)	5·6 (3·9-8·2)	3·9 (2·5-6·5)	3·9 (2·4-6·4)	3·1 (1·8-5·3)	0·4 (0·3-0·5)	0·3 (0·2-0·4)	0·3 (0·2-0·4)	0·3 (0·2-0·4)	12·0 (10·9-13·1)	9·9 (8·8-11·1)	9·9 (8·7-11·1)	9·1 (7·1-11·5)
Saint Lucia	44·1 (35·7-53·9)	27·2 (20·8-34·3)	27·2 (20·8-34·4)	19·6 (12·2-28·6)	4·2 (3·2-5·3)	2·4 (1·7-3·1)	2·3 (1·6-3·1)	1·3 (0·8-2·1)	0·3 (0·2-0·4)	0·2 (0·1-0·3)	0·2 (0·1-0·3)	0·1 (0·1-0·2)	9·2 (8·1-10·5)	9·5 (8·4-10·9)	9·5 (8·4-10·7)	9·9 (7·6-12·4)
Saint Vincent and the Grenadines	80·9 (63·0-99·0)	47·6 (34·1-60·3)	47·8 (34·3-60·4)	34·4 (16·9-52·8)	8·2 (6·3-10·0)	5·2 (3·9-6·7)	5·2 (3·9-6·7)	3·6 (2·2-5·5)	0·3 (0·3-0·5)	0·2 (0·2-0·4)	0·2 (0·2-0·4)	0·1 (0·1-0·3)	9·3 (8·2-10·6)	8·3 (7·3-9·4)	8·3 (7·3-9·3)	7·8 (6·1-9·8)
Suriname	88·6 (71·0-106·7)	59·1 (46·0-74·0)	59·0 (45·8-73·9)	53·0 (39·4-67·7)	17·4 (12·2-24·3)	10·0 (6·4-15·1)	9·7 (6·1-14·8)	6·2 (3·5-10·1)	0·6 (0·5-0·9)	0·4 (0·4-0·7)	0·4 (0·3-0·7)	0·3 (0·2-0·6)	13·7 (12·2-15·3)	16·1 (14·9-17·3)	15·8 (14·6-17·2)	18·1 (15·2-21·3)
Trinidad and Tobago	57·6 (48·2-67·6)	35·4 (28·7-41·9)	36·1 (29·4-42·7)	31·7 (22·6-47·5)	4·8 (3·6-5·9)	2·8 (2·0-3·7)	2·7 (1·9-3·7)	1·8 (0·9-2·9)	0·3 (0·3-0·5)	0·2 (0·2-0·4)	0·2 (0·2-0·4)	0·2 (0·1-0·4)	11·7 (10·5-13·0)	14·2 (12·9-15·4)	14·0 (12·7-15·4)	14·9 (11·8-18·4)
**Tropical Latin America**																
Brazil	73·1 (63·2-83·4)	34·8 (29·1-41·1)	34·1 (28·6-40·3)	21·0 (15·1-28·0)	11·6 (9·5-13·5)	4·7 (3·7-5·7)	4·5 (3·5-5·5)	1·9 (1·2-2·6)	0·3 (0·3-0·4)	0·2 (0·2-0·2)	0·2 (0·2-0·2)	0·1 (0·1-0·2)	17·5 (17·0-18·1)	8·0 (7·8-8·1)	7·6 (7·4-7·8)	4·1 (3·8-4·3)
Paraguay	97·4 (86·7-108·2)	72·8 (60·4-87·0)	71·1 (58·7-85·3)	51·0 (38·7-65·2)	13·5 (9·8-18·1)	4·5 (3·0-6·6)	4·2 (2·8-6·3)	1·9 (1·2-3·1)	0·4 (0·4-0·6)	0·3 (0·2-0·4)	0·3 (0·2-0·4)	0·2 (0·1-0·4)	14·0 (13·2-15·0)	10·2 (9·3-11·1)	10·1 (9·2-11·0)	7·5 (6·3-8·9)
**East Asia**																
China	240·9 (216·9-263·2)	123·0 (110·9-134·8)	117·2 (105·5-128·6)	60·2 (53·3-67·0)	4·1 (3·1-5·1)	0·8 (0·6-1·2)	0·8 (0·5-1·1)	0·2 (0·1-0·4)	1·9 (1·6-2·3)	1·2 (0·7-1·4)	1·1 (0·6-1·3)	0·7 (0·3-1·0)	24·8 (24·3-25·3)	21·1 (20·7-21·4)	20·9 (20·5-21·3)	18·1 (17·3-18·9)
North Korea	264·3 (227·8-302·6)	251·4 (218·4-285·8)	249·2 (216·4-283·4)	229·8 (195·8-266·6)	7·5 (4·4-11·9)	3·0 (1·9-4·9)	3·0 (1·8-4·8)	2·0 (1·2-3·2)	2·1 (1·4-2·9)	2·0 (1·3-2·7)	2·0 (1·3-2·7)	1·7 (1·0-2·5)	21·1 (19·5-22·8)	18·7 (16·9-20·6)	18·7 (16·8-20·6)	17·1 (13·8-20·7)
Taiwan (Province of China)	49·7 (41·8-59·0)	35·3 (29·7-41·6)	34·6 (29·0-40·9)	25·0 (20·2-30·9)	0·7 (0·4-1·2)	0·7 (0·3-1·1)	0·7 (0·3-1·1)	0·5 (0·2-1·0)	0·6 (0·4-0·7)	0·3 (0·3-0·4)	0·3 (0·3-0·4)	0·2 (0·1-0·3)	21·5 (20·2-22·8)	12·6 (11·0-14·3)	12·6 (10·9-14·5)	7·7 (5·8-10·0)
**Southeast Asia**																
Cambodia	280·1 (250·2-311·5)	187·3 (167·1-209·2)	182·7 (162·8-204·1)	127·6 (111·8-144·0)	78·6 (51·6-115·2)	23·6 (14·8-36·3)	21·8 (13·6-33·8)	7·3 (4·2-12·7)	1·6 (0·9-2·4)	0·8 (0·5-1·0)	0·7 (0·5-1·0)	0·4 (0·2-0·6)	23·7 (22·6-24·7)	18·5 (17·1-19·9)	18·6 (17·0-20·1)	15·9 (13·1-19·3)
Indonesia	123·4 (110·4-136·4)	87·4 (77·3-98·6)	83·0 (73·2-93·9)	58·4 (40·9-76·6)	64·5 (27·5-103·4)	31·4 (14·9-51·4)	30·2 (13·9-50·0)	16·5 (6·0-31·3)	0·5 (0·3-0·6)	0·4 (0·2-0·5)	0·4 (0·2-0·5)	0·3 (0·1-0·4)	25·9 (25·1-26·5)	27·0 (26·3-27·6)	26·8 (26·1-27·5)	27·1 (25·7-28·6)
Laos	312·5 (280·0-347·0)	217·9 (194·6-244·0)	212·2 (189·3-237·8)	147·3 (129·5-167·8)	69·8 (44·8-98·4)	24·2 (14·5-36·4)	22·4 (13·3-33·8)	8·0 (4·0-13·1)	1·9 (0·9-2·8)	0·9 (0·5-1·3)	0·9 (0·5-1·2)	0·4 (0·2-0·7)	30·2 (28·1-32·4)	27·7 (24·7-31·1)	27·5 (24·5-30·9)	25·3 (20·1-31·5)
Malaysia	72·3 (59·5-88·1)	58·3 (48·6-69·9)	57·7 (48·2-69·0)	49·6 (42·3-58·0)	3·5 (1·8-5·8)	2·6 (1·2-4·2)	2·5 (1·2-4·1)	1·8 (0·8-3·0)	1·2 (0·9-1·4)	0·7 (0·5-0·9)	0·7 (0·5-0·9)	0·4 (0·3-0·7)	20·0 (18·3-21·6)	18·5 (17·0-20·1)	18·4 (16·8-20·2)	16·6 (13·7-20·1)
Maldives	148·5 (127·3-172·1)	51·6 (38·6-67·1)	50·1 (37·2-65·6)	26·5 (14·6-41·5)	5·1 (3·3-7·5)	2·0 (1·2-2·9)	1·9 (1·1-2·7)	0·8 (0·4-1·3)	0·2 (0·1-0·3)	0·1 (0·1-0·1)	0·1 (0·1-0·1)	0·0 (0·0-0·1)	21·4 (19·2-23·8)	21·8 (20·1-23·7)	21·5 (19·5-23·6)	21·0 (17·5-24·8)
Mauritius	84·1 (70·6-98·5)	35·6 (28·5-43·7)	34·9 (27·9-42·9)	20·8 (14·7-28·6)	2·0 (0·9-2·8)	1·8 (0·6-2·7)	1·8 (0·6-2·6)	1·2 (0·4-1·9)	0·3 (0·2-0·3)	0·1 (0·1-0·2)	0·1 (0·1-0·2)	0·1 (0·1-0·1)	16·5 (15·5-17·6)	16·9 (14·7-19·1)	16·8 (14·6-19·0)	16·1 (11·8-21·3)
Myanmar	236·9 (206·5-274·9)	152·9 (131·8-176·0)	148·2 (127·7-170·3)	94·5 (80·4-108·2)	75·7 (50·8-112·7)	25·8 (15·4-38·8)	24·1 (14·2-36·4)	9·0 (4·6-14·8)	1·9 (1·2-2·6)	0·9 (0·6-1·1)	0·8 (0·6-1·1)	0·4 (0·3-0·6)	22·6 (20·8-24·4)	16·8 (15·4-18·4)	16·8 (15·3-18·6)	14·0 (11·4-17·3)
Philippines	184·0 (161·9-208·4)	185·3 (154·8-215·8)	181·3 (150·3-212·5)	147·3 (106·3-200·9)	17·3 (12·8-22·0)	14·3 (9·6-19·5)	13·8 (9·1-19·0)	8·6 (4·6-14·3)	0·4 (0·3-0·4)	0·2 (0·2-0·3)	0·2 (0·2-0·3)	0·2 (0·1-0·3)	25·6 (24·4-27·0)	21·1 (19·0-23·2)	21·0 (18·8-23·3)	17·9 (14·3-22·4)
Sri Lanka	120·6 (106·5-137·6)	74·6 (57·1-92·9)	71·1 (53·6-89·5)	49·4 (28·4-77·6)	10·5 (6·9-16·7)	3·4 (1·9-6·1)	3·2 (1·7-5·8)	1·1 (0·5-2·3)	0·8 (0·7-1·0)	0·3 (0·2-0·5)	0·3 (0·2-0·5)	0·2 (0·1-0·4)	11·3 (10·4-12·2)	9·7 (8·9-10·5)	9·7 (8·8-10·7)	8·3 (6·7-10·0)
Seychelles	57·5 (35·5-80·9)	37·0 (20·6-57·0)	36·4 (20·0-56·3)	28·3 (13·8-48·1)	3·2 (1·8-5·1)	2·6 (1·4-4·0)	2·5 (1·4-3·9)	1·8 (0·9-3·0)	1·0 (0·7-1·2)	0·6 (0·4-0·7)	0·5 (0·4-0·7)	0·4 (0·2-0·5)	15·0 (13·2-16·9)	14·1 (12·6-16·0)	14·1 (12·5-16·1)	13·5 (10·4-17·2)
Thailand	85·4 (74·0-97·6)	46·2 (37·1-56·3)	44·4 (35·4-54·4)	25·2 (18·5-33·2)	9·1 (5·4-14·3)	7·2 (4·1-11·2)	7·0 (3·9-10·8)	4·4 (2·3-6·9)	0·5 (0·4-0·6)	0·3 (0·2-0·3)	0·3 (0·2-0·3)	0·2 (0·1-0·3)	22·1 (21·3-22·9)	17·2 (15·6-19·0)	17·1 (15·4-19·0)	14·8 (11·6-18·4)
Timor-Leste	206·8 (174·6-241·3)	136·0 (107·6-162·4)	133·3 (105·2-159·5)	100·5 (75·0-123·6)	96·6 (46·6-161·9)	27·0 (12·9-47·2)	24·8 (11·8-43·7)	7·6 (3·0-15·1)	1·1 (0·6-1·6)	0·5 (0·3-0·8)	0·5 (0·3-0·8)	0·3 (0·1-0·5)	31·1 (28·6-34·0)	26·8 (24·8-28·8)	26·6 (24·5-28·7)	24·2 (20·7-28·1)
Vietnam	155·1 (134·2-174·9)	91·5 (76·3-108·7)	87·9 (73·0-104·7)	49·8 (39·7-61·9)	9·9 (5·7-15·4)	3·4 (2·0-5·5)	3·2 (1·9-5·3)	1·3 (0·6-2·7)	1·6 (0·6-2·2)	0·9 (0·3-1·4)	0·9 (0·3-1·4)	0·5 (0·2-1·2)	23·3 (22·4-24·1)	17·0 (16·3-17·8)	17·0 (16·2-18·0)	14·8 (12·6-17·1)
**Oceania**																
Federated States of Micronesia	157·4 (117·4-204·3)	107·0 (68·2-152·5)	104·9 (66·5-150·3)	77·2 (44·1-117·4)	29·3 (21·5-41·5)	8·8 (5·0-15·8)	8·6 (4·9-15·4)	6·1 (3·2-11·2)	1·9 (1·3-2·5)	1·6 (1·0-2·1)	1·5 (1·0-2·0)	1·3 (0·6-2·1)	22·2 (20·2-24·6)	21·3 (19·1-23·6)	21·2 (19·0-23·6)	20·3 (16·5-24·4)
Fiji	115·2 (88·6-146·4)	60·8 (41·7-83·7)	59·1 (40·1-81·9)	38·8 (23·3-59·5)	17·8 (9·3-32·2)	12·4 (6·3-19·9)	12·1 (6·1-19·5)	8·0 (3·4-13·8)	1·0 (0·7-1·1)	0·7 (0·5-0·9)	0·7 (0·5-0·9)	0·6 (0·3-1·2)	13·5 (12·5-14·4)	13·1 (11·9-14·3)	13·1 (11·9-14·4)	12·5 (10·4-14·9)

Location	Indicator 3.b.1: Geometric mean of the coverage of eight vaccines, conditional on inclusion in national vaccine schedules, in target populations (%)				Indicator 5.2.1: Age-standardised prevalence of women aged 15 years and older who experienced physical or sexual violence by an intimate partner in the last 12 months (%)				Indicator 6.1.1: Risk-weighted prevalence of populations using unsafe or unimproved water sources, as measured by the summary exposure value (SEV) for unsafe water (%)				Indicator 6.2.1a: Risk-weighted prevalence of populations using unsafe or unimproved sanitation, as measured by the summary exposure value (SEV) for unsafe sanitation (%)			
	**2000**	**2015**	**2016**	**2030**	**2000**	**2015**	**2016**	**2030**	**2000**	**2015**	**2016**	**2030**	**2000**	**2015**	**2016**	**2030**
Guyana	89·9 (87·9-91·8)	95·2 (93·3-96·6)	95·7 (93·7-97·0)	98·9 (96·9-99·8)	32·3 (28·1-36·6)	27·6 (24·3-31·3)	27·5 (24·1-31·2)	24·9 (21·4-29·0)	29·1 (18·6-34·2)	28·6 (19·3-34·2)	28·3 (19·1-33·9)	24·5 (16·6-30·6)	51·3 (34·7-68·7)	25·7 (14·2-41·3)	24·2 (13·0-39·5)	9·0 (3·6-17·9)
Haiti	71·2 (68·0-74·4)	89·8 (86·6-92·6)	90·8 (87·4-93·5)	97·9 (95·6-99·3)	41·0 (36·7-45·3)	36·7 (32·6-40·7)	36·5 (32·3-40·5)	33·9 (29·7-37·9)	41·8 (29·3-47·7)	40·5 (27·7-46·9)	40·4 (27·5-46·8)	39·0 (25·6-45·7)	84·0 (69·7-99·5)	76·1 (55·4-97·2)	75·5 (53·8-97·0)	64·7 (31·3-87·6)
Jamaica	92·0 (90·2-93·6)	97·4 (95·9-98·4)	97·6 (96·1-98·5)	99·2 (97·8-99·8)	26·2 (23·1-29·4)	24·7 (21·8-27·7)	24·5 (21·7-27·5)	22·9 (20·2-25·7)	23·1 (12·0-28·4)	20·1 (9·8-25·0)	20·0 (9·6-24·9)	18·0 (8·2-22·8)	37·7 (24·1-52·1)	40·4 (17·9-64·2)	40·9 (17·0-66·5)	46·6 (8·2-87·0)
Saint Lucia	93·2 (91·2-94·9)	98·8 (98·1-99·3)	98·9 (98·2-99·4)	99·8 (99·4-100·0)	27·2 (23·9-30·9)	24·1 (21·3-27·3)	23·9 (21·1-27·0)	21·3 (18·8-23·8)	22·3 (11·5-27·6)	18·6 (8·4-23·6)	18·4 (8·3-23·3)	15·8 (6·6-19·8)	42·3 (28·4-54·8)	20·6 (9·5-36·8)	20·0 (8·7-37·1)	9·7 (1·8-29·9)
Saint Vincent and the Grenadines	98·3 (96·9-99·2)	99·4 (98·9-99·7)	99·5 (99·0-99·8)	99·9 (99·6-100·0)	30·0 (26·3-34·1)	27·3 (24·1-30·8)	27·1 (23·9-30·5)	24·2 (21·4-27·3)	26·0 (15·5-31·0)	22·5 (13·0-27·6)	22·3 (12·9-27·4)	20·0 (10·8-25·2)	45·2 (32·5-57·8)	30·5 (9·1-63·2)	30·1 (8·8-63·1)	22·0 (3·0-54·7)
Suriname	88·9 (86·5-91·1)	96·4 (94·6-97·7)	96·8 (95·1-98·0)	99·4 (98·2-100·0)	28·2 (24·5-31·9)	24·3 (21·3-27·4)	24·0 (21·0-27·1)	20·3 (17·8-22·9)	29·2 (19·5-34·4)	23·2 (14·9-28·4)	22·8 (14·6-27·9)	17·5 (10·9-22·3)	29·0 (20·8-37·9)	17·1 (6·4-33·6)	16·8 (5·7-34·7)	11·1 (0·9-40·1)
Trinidad and Tobago	73·7 (70·5-76·5)	69·7 (63·6-75·5)	71·3 (64·8-77·5)	76·2 (61·2-87·4)	25·3 (22·1-28·9)	22·7 (20·0-25·9)	22·5 (19·7-25·5)	19·2 (16·6-21·9)	21·5 (13·3-26·1)	16·9 (9·0-21·6)	16·8 (8·9-21·5)	15·4 (4·6-29·6)	31·3 (21·7-41·5)	15·3 (5·5-35·0)	15·6 (5·4-37·0)	8·5 (1·2-27·4)
**Tropical Latin America**																
Brazil	95·2 (92·7-97·1)	99·6 (99·5-99·7)	99·7 (99·6-99·8)	100·0 (100·0-100·0)	26·1 (23·1-29·5)	22·3 (19·9-25·0)	22·1 (19·7-24·7)	18·7 (16·7-20·8)	12·5 (8·8-14·2)	9·2 (6·5-10·5)	9·0 (6·4-10·2)	6·7 (4·7-7·7)	38·9 (32·4-46·8)	32·6 (25·1-42·1)	32·1 (24·5-41·7)	24·7 (17·0-35·2)
Paraguay	85·7 (83·3-87·9)	79·9 (74·6-84·3)	81·0 (75·5-85·5)	80·5 (64·8-90·9)	29·2 (25·9-32·9)	25·1 (22·1-28·5)	24·9 (21·9-28·2)	21·6 (18·8-24·6)	21·6 (14·6-25·3)	10·4 (7·0-13·3)	10·1 (6·8-12·9)	6·5 (4·3-8·1)	40·9 (28·9-50·7)	18·2 (6·7-36·6)	17·9 (6·2-37·4)	7·1 (0·9-26·4)
**East Asia**																
China	98·4 (96·4-99·3)	99·9 (99·8-100·0)	99·9 (99·8-100·0)	100·0 (100·0-100·0)	33·5 (29·9-37·1)	26·0 (23·2-28·7)	25·5 (22·7-28·2)	19·4 (17·2-21·7)	17·4 (7·5-21·5)	12·7 (4·4-16·4)	12·5 (4·2-16·1)	9·1 (2·5-12·2)	51·1 (47·2-54·9)	25·5 (22·1-29·4)	24·4 (21·0-28·2)	10·5 (8·1-13·5)
North Korea	92·9 (90·3-94·8)	99·0 (97·8-99·5)	99·0 (97·9-99·6)	99·9 (99·2-100·0)	36·2 (31·9-40·0)	35·7 (31·9-39·4)	35·5 (31·7-39·2)	34·9 (30·9-38·8)	19·0 (7·6-27·6)	18·8 (7·5-27·2)	18·7 (7·5-27·1)	16·7 (4·1-33·4)	49·4 (37·0-61·3)	45·9 (30·9-60·8)	45·6 (30·4-60·9)	41·6 (23·5-61·4)
Taiwan (Province of China)	99·5 (99·1-99·7)	97·7 (97·5-97·8)	98·6 (98·4-98·7)	100·0 (100·0-100·0)	25·8 (22·7-28·9)	20·4 (18·0-22·9)	20·2 (17·8-22·7)	16·8 (14·9-18·9)	9·9 (3·1-18·0)	9·3 (2·3-17·0)	9·2 (2·2-16·8)	8·2 (1·2-18·5)	12·5 (5·2-23·3)	6·3 (2·6-12·2)	6·1 (2·5-11·8)	3·2 (1·2-6·7)
**Southeast Asia**																
Cambodia	58·6 (55·7-61·6)	90·4 (87·0-93·0)	91·7 (88·4-94·3)	98·7 (96·6-99·6)	40·1 (35·8-44·0)	34·8 (30·8-38·7)	34·5 (30·4-38·4)	30·7 (26·5-34·7)	29·3 (10·3-37·7)	24·4 (5·7-32·7)	24·1 (5·5-32·5)	20·4 (1·5-44·8)	84·1 (78·5-88·5)	47·4 (38·0-56·9)	45·3 (35·6-55·2)	16·1 (8·7-27·1)
Indonesia	66·1 (63·2-68·9)	76·5 (71·2-81·5)	72·0 (63·3-79·2)	82·6 (68·4-93·1)	31·9 (28·0-35·8)	27·9 (24·8-31·3)	27·6 (24·5-31·0)	23·6 (21·0-26·2)	18·0 (0·3-26·2)	19·2 (0·6-27·4)	19·2 (0·6-27·4)	18·6 (0·3-45·7)	46·9 (40·9-54·8)	24·0 (19·4-29·5)	22·8 (18·2-28·4)	10·2 (6·7-14·0)
Laos	46·4 (43·0-49·7)	79·9 (75·3-84·2)	81·2 (75·6-85·9)	96·7 (91·2-99·3)	37·2 (32·6-41·9)	32·7 (28·4-37·1)	32·4 (28·1-36·7)	28·5 (24·6-32·4)	27·5 (9·3-35·1)	25·2 (8·3-33·0)	25·0 (8·1-32·8)	21·9 (1·8-52·9)	84·0 (76·9-89·8)	67·5 (53·5-78·9)	66·6 (51·9-78·5)	51·7 (28·0-71·8)
Malaysia	94·1 (92·6-95·4)	97·8 (96·5-98·6)	98·1 (96·9-98·9)	99·6 (98·8-100·0)	32·0 (27·9-36·5)	27·5 (23·9-31·5)	27·1 (23·6-31·0)	23·7 (20·5-27·2)	11·8 (4·3-18·3)	10·8 (4·1-17·7)	10·7 (4·0-17·6)	9·3 (2·6-20·4)	8·6 (4·0-15·4)	4·4 (1·5-10·6)	4·3 (1·5-10·5)	2·7 (0·5-7·5)
Maldives	94·4 (92·9-95·7)	96·6 (94·5-98·0)	96·5 (94·2-98·1)	96·2 (87·1-99·3)	34·5 (29·9-39·3)	28·9 (25·0-33·1)	28·5 (24·7-32·6)	23·8 (20·3-27·6)	35·2 (24·6-40·5)	30·8 (21·2-36·6)	30·4 (20·7-36·3)	24·5 (13·8-32·0)	50·8 (35·0-64·9)	20·1 (11·6-31·1)	18·8 (10·5-29·7)	6·2 (2·2-13·2)
Mauritius	92·2 (90·2-93·9)	98·6 (97·5-99·3)	98·8 (97·7-99·4)	99·7 (98·9-100·0)	28·1 (24·6-31·7)	25·2 (22·3-28·3)	25·0 (22·1-28·0)	21·7 (19·3-24·3)	13·7 (2·3-22·4)	13·8 (3·0-22·5)	13·7 (3·0-22·4)	12·5 (1·4-21·0)	8·3 (4·0-15·1)	4·4 (1·7-10·1)	4·4 (1·6-10·0)	2·6 (0·7-7·6)
Myanmar	89·2 (87·5-90·8)	96·9 (94·8-98·2)	97·2 (95·1-98·5)	99·3 (96·9-99·9)	39·1 (34·9-43·4)	31·3 (27·7-34·9)	30·9 (27·3-34·4)	25·1 (22·1-28·2)	33·5 (18·9-39·6)	29·6 (15·0-36·2)	29·3 (14·7-35·9)	25·1 (7·1-44·7)	77·3 (59·5-100·0)	49·4 (31·0-70·3)	47·2 (29·1-67·7)	19·7 (10·6-28·7)
Philippines	74·2 (68·9-78·9)	87·0 (81·8-90·7)	86·0 (80·1-90·0)	89·1 (73·9-96·2)	37·6 (33·4-41·6)	34·1 (30·2-37·8)	34·0 (30·1-37·6)	31·9 (28·0-35·7)	28·3 (17·3-35·1)	25·8 (15·2-32·6)	25·4 (14·8-32·4)	20·4 (10·0-29·9)	30·5 (24·7-36·7)	13·7 (8·1-20·9)	13·1 (7·6-20·4)	5·5 (2·2-11·9)
Sri Lanka	99·4 (99·1-99·7)	99·9 (99·7-100·0)	99·9 (99·7-100·0)	100·0 (99·9-100·0)	38·1 (33·9-42·3)	30·8 (27·4-34·1)	30·3 (27·0-33·6)	24·0 (21·3-26·8)	24·5 (9·7-32·2)	19·1 (6·4-27·5)	18·7 (6·2-27·1)	13·3 (1·7-33·0)	65·9 (54·4-76·5)	37·2 (22·1-55·1)	35·2 (20·4-53·6)	14·3 (5·3-30·4)
Seychelles	99·4 (99·0-99·6)	99·8 (99·6-99·9)	99·8 (99·6-99·9)	99·9 (99·7-100·0)	33·2 (29·2-37·6)	30·6 (27·2-34·3)	30·3 (26·9-33·9)	27·1 (24·0-30·3)	14·5 (3·9-23·0)	14·8 (3·4-23·1)	14·7 (3·2-22·9)	12·7 (2·1-27·7)	18·7 (8·8-31·9)	12·7 (5·9-22·4)	12·2 (5·7-21·5)	6·6 (2·9-12·0)
Thailand	85·9 (83·2-88·2)	92·9 (90·5-94·8)	93·0 (90·1-95·2)	94·1 (85·3-98·2)	35·1 (31·0-39·3)	27·5 (24·4-30·7)	27·2 (24·1-30·3)	22·4 (19·9-24·9)	31·9 (22·4-37·2)	30·5 (20·3-36·3)	30·3 (20·1-36·2)	27·2 (17·0-34·5)	8·4 (4·1-14·9)	3·3 (1·5-6·0)	3·2 (1·4-5·6)	1·4 (0·5-2·3)
Timor-Leste	22·9 (19·8-25·8)	49·0 (41·5-57·3)	49·6 (40·6-59·0)	63·7 (42·5-83·4)	47·4 (42·0-52·5)	41·4 (36·1-46·1)	41·0 (35·8-45·8)	35·9 (30·3-41·5)	19·3 (0·9-26·9)	18·1 (0·0-26·0)	17·8 (0·0-25·7)	14·0 (0·0-52·3)	89·6 (80·5-97·2)	62·4 (47·8-75·9)	60·5 (45·4-74·5)	27·7 (15·0-43·4)
Vietnam	78·9 (76·1-81·5)	85·9 (81·6-89·8)	86·8 (81·9-90·8)	92·8 (84·0-97·4)	31·8 (27·9-36·0)	27·7 (24·5-31·0)	27·4 (24·2-30·7)	23·9 (21·0-26·9)	16·5 (0·0-23·8)	15·2 (0·0-22·3)	15·0 (0·0-22·2)	13·5 (0·0-33·9)	74·4 (66·5-81·5)	35·2 (22·1-48·9)	33·6 (20·4-47·6)	12·1 (4·0-24·8)
**Oceania**																
Federated States of Micronesia	69·9 (64·6-74·3)	80·8 (75·0-85·3)	82·9 (76·6-87·6)	96·5 (88·8-99·8)	39·7 (35·1-44·3)	36·2 (31·9-40·9)	36·2 (31·8-40·8)	33·2 (28·7-38·3)	28·0 (16·4-36·3)	22·1 (11·5-31·9)	21·8 (11·4-31·8)	18·6 (9·4-29·9)	37·0 (19·8-56·7)	20·9 (9·8-37·0)	20·5 (9·5-36·4)	11·5 (4·2-23·8)
Fiji	79·1 (75·0-82·8)	87·8 (84·7-90·6)	88·8 (85·2-91·5)	96·5 (91·4-99·1)	33·2 (29·2-37·3)	30·0 (26·7-33·5)	29·9 (26·6-33·3)	27·6 (24·7-30·9)	18·7 (9·8-27·0)	15·3 (7·3-25·4)	15·1 (7·1-25·4)	12·8 (4·8-26·4)	19·2 (8·8-34·9)	16·0 (6·3-31·8)	16·0 (6·2-31·5)	13·7 (4·7-25·9)

Location	Indicator 6.2.1b: Risk-weighted prevalence of populations without access to a handwashing facility, as measured by the summary exposure value (SEV) for unsafe hygiene (%)				Indicator 7.1.2: Risk-weighted prevalence of household air pollution, as measured by the summary exposure value (SEV) for household air pollution (%)				Indicator 8.8.1: Age-standardised all-cause disability-adjusted life year (DALY) rates attributable to occupational risks (per 100,000 population)				Indicator 11.6.2: Population-weighted mean levels of fine particulate matter smaller than 2.5 microns in diameter (PM2.5)			
	**2000**	**2015**	**2016**	**2030**	**2000**	**2015**	**2016**	**2030**	**2000**	**2015**	**2016**	**2030**	**2000**	**2015**	**2016**	**2030**
Guyana	51·1<br>(45·7-56·7)	55·0<br>(49·8-59·8)	54·4<br>(49·2-59·2)	54·7<br>(48·1-60·0)	7·7<br>(5·4-10·5)	4·3<br>(2·7-6·5)	4·0<br>(2·5-6·2)	1·7<br>(0·8-3·1)	1,034·2<br>(835·9-1,324·6)	929·1<br>(737·6-1,169·9)	923·1<br>(733·4-1,162·9)	840·6<br>(671·2-1,051·7)	26·7<br>(20·4-37·8)	19·7<br>(15·0-27·3)	19·7<br>(15·1-27·3)	19·6<br>(12·0-30·9)
Haiti	77·5<br>(73·3-81·3)	78·2<br>(74·3-81·7)	78·1<br>(74·1-81·6)	79·1<br>(73·7-82·6)	62·0<br>(51·0-74·1)	54·0<br>(43·0-65·5)	53·2<br>(42·4-64·8)	43·0<br>(32·8-54·5)	918·8<br>(746·2-1,107·2)	854·8<br>(693·4-1,028·8)	851·4<br>(690·9-1,024·6)	804·9<br>(656·0-968·2)	30·8<br>(27·2-36·4)	23·6<br>(20·8-27·7)	23·5<br>(20·8-27·6)	23·2<br>(20·1-27·6)
Jamaica	46·8<br>(41·2-52·2)	42·5<br>(37·4-47·6)	42·2<br>(37·2-47·3)	38·2<br>(33·6-43·2)	8·8<br>(6·4-11·8)	4·6<br>(2·7-7·0)	4·3<br>(2·5-6·7)	2·0<br>(0·8-3·8)	951·2<br>(773·1-1,178·1)	838·4<br>(668·7-1,036·8)	834·2<br>(663·7-1,031·3)	776·4<br>(597·1-970·6)	20·5<br>(19·4-21·7)	15·1<br>(14·3-16·0)	15·1<br>(14·3-15·9)	14·5<br>(13·4-15·5)
Saint Lucia	21·0<br>(17·5-24·8)	19·7<br>(16·5-23·3)	19·6<br>(16·4-23·2)	18·6<br>(15·0-22·2)	7·2<br>(4·8-10·1)	1·8<br>(0·9-3·2)	1·7<br>(0·8-3·0)	0·5<br>(0·2-1·0)	770·4<br>(631·4-932·4)	619·3<br>(503·8-742·7)	613·4<br>(498·6-736·3)	535·5<br>(429·8-647·1)	19·5<br>(12·8-29·1)	17·2<br>(10·9-26·6)	17·3<br>(11·1-26·9)	17·6<br>(10·0-30·5)
Saint Vincent and the Grenadines	34·1<br>(29·1-39·7)	28·0<br>(23·3-33·6)	27·8<br>(23·1-33·3)	23·1<br>(17·9-28·7)	15·1<br>(9·0-22·8)	2·7<br>(1·3-4·9)	2·5<br>(1·2-4·7)	0·9<br>(0·3-2·2)	511·5<br>(406·5-622·6)	520·7<br>(409·6-637·4)	522·9<br>(411·8-640·8)	553·5<br>(438·0-678·5)	19·1<br>(11·2-32·7)	16·8<br>(10·0-26·1)	17·4<br>(10·4-27·5)	17·4<br>(9·4-29·7)
Suriname	39·1<br>(34·2-44·5)	32·9<br>(28·6-37·8)	32·4<br>(28·2-37·3)	26·2<br>(22·1-32·4)	11·0<br>(6·7-16·6)	5·9<br>(3·4-9·1)	5·6<br>(3·1-8·9)	2·7<br>(0·8-6·0)	695·9<br>(564·4-872·5)	677·0<br>(546·7-840·0)	680·3<br>(549·1-843·6)	726·7<br>(585·3-905·8)	28·5<br>(17·9-52·0)	21·7<br>(14·1-35·9)	21·5<br>(14·3-36·1)	21·3<br>(11·7-36·7)
Trinidad and Tobago	27·9<br>(23·6-32·8)	23·1<br>(19·2-27·3)	23·0<br>(19·1-27·2)	19·0<br>(15·1-24·9)	0·4<br>(0·2-0·9)	0·2<br>(0·1-0·4)	0·2<br>(0·1-0·4)	0·1<br>(0·1-0·2)	738·8<br>(583·2-901·2)	608·6<br>(472·9-751·4)	608·4<br>(472·2-751·3)	604·6<br>(458·0-754·0)	19·3<br>(16·1-23·3)	16·6<br>(14·0-19·8)	16·8<br>(14·0-20·2)	16·5<br>(10·2-22·1)
**Tropical Latin America**																
Brazil	32·5<br>(31·1-33·9)	25·2<br>(24·0-26·4)	24·8<br>(23·6-26·0)	19·9<br>(18·9-21·1)	7·6<br>(6·1-9·4)	2·2<br>(1·7-2·8)	2·0<br>(1·5-2·6)	0·5<br>(0·4-0·7)	1,140·9<br>(999·0-1,286·1)	1,006·6<br>(875·9-1,145·9)	998·9<br>(869·4-1,137·3)	897·1<br>(780·5-1,022·4)	16·2<br>(15·9-16·5)	12·6<br>(12·4-12·8)	12·7<br>(12·4-12·9)	13·9<br>(11·8-15·5)
Paraguay	48·6<br>(42·5-54·7)	25·7<br>(21·6-30·1)	25·3<br>(21·2-29·6)	16·9<br>(13·9-20·8)	29·6<br>(23·3-36·6)	15·6<br>(9·8-22·5)	14·5<br>(8·9-21·3)	4·8<br>(1·9-9·1)	1,236·6<br>(1,066·2-1,421·1)	1,121·2<br>(957·7-1,304·6)	1,112·7<br>(949·3-1,295·6)	999·3<br>(837·7-1,180·8)	23·3<br>(21·5-25·4)	23·1<br>(21·2-25·4)	23·7<br>(21·6-26·3)	24·0<br>(21·4-27·2)
**East Asia**																
China	22·0<br>(19·8-24·1)	14·6<br>(13·1-16·2)	14·1<br>(12·7-15·8)	9·0<br>(8·2-10·2)	35·1<br>(28·8-42·0)	16·5<br>(13·2-20·3)	15·4<br>(12·3-19·0)	5·7<br>(4·4-7·1)	1,489·1<br>(1,335·9-1,656·7)	1,045·6<br>(921·6-1,171·5)	1,029·2<br>(906·6-1,153·8)	819·9<br>(714·4-924·9)	51·6<br>(51·3-51·8)	56·4<br>(56·1-56·7)	56·3<br>(56·0-56·6)	60·1<br>(59·7-61·1)
North Korea	16·1<br>(9·8-24·7)	16·6<br>(9·9-25·5)	16·5<br>(9·8-25·3)	15·9<br>(9·4-24·7)	53·8<br>(43·5-65·3)	48·8<br>(37·4-60·8)	48·2<br>(36·7-60·2)	39·1<br>(26·7-51·3)	1,527·6<br>(1,326·8-1,733·4)	1,539·2<br>(1,329·7-1,744·9)	1,533·1<br>(1,325·1-1,738·6)	1,449·0<br>(1,264·6-1,639·4)	30·8<br>(28·4-33·8)	35·9<br>(33·4-38·8)	36·0<br>(33·4-39·1)	37·4<br>(34·0-41·0)
Taiwan (Province of China)	8·9<br>(4·9-14·5)	8·3<br>(4·7-13·7)	8·2<br>(4·7-13·7)	7·5<br>(4·4-13·3)	4·5<br>(1·7-8·9)	1·5<br>(0·5-3·4)	1·4<br>(0·5-3·1)	0·4<br>(0·1-1·0)	856·5<br>(717·9-999·5)	817·7<br>(687·4-967·0)	818·2<br>(687·0-968·9)	823·7<br>(677·9-987·0)	29·9<br>(28·6-31·4)	29·4<br>(28·0-30·8)	29·4<br>(28·0-30·8)	29·8<br>(27·6-33·4)
**Southeast Asia**																
Cambodia	43·5<br>(30·4-56·8)	33·5<br>(23·1-45·0)	32·9<br>(22·6-44·3)	25·4<br>(16·5-35·5)	62·0<br>(50·4-73·8)	45·9<br>(37·2-55·6)	44·7<br>(36·2-54·2)	29·4<br>(23·8-35·5)	1,951·6<br>(1,659·2-2,251·9)	1,636·4<br>(1,374·2-1,910·4)	1,623·4<br>(1,361·8-1,895·5)	1,448·3<br>(1,201·5-1,706·3)	25·0<br>(22·8-28·6)	26·2<br>(23·6-29·7)	26·1<br>(23·6-30·6)	26·6<br>(23·9-31·2)
Indonesia	25·9<br>(23·1-28·9)	26·2<br>(24·4-28·0)	26·0<br>(24·1-27·8)	23·0<br>(21·2-24·9)	26·9<br>(21·8-32·4)	14·7<br>(11·8-18·4)	13·3<br>(10·6-16·7)	2·8<br>(2·1-3·7)	1,583·7<br>(1,369·6-1,797·4)	1,572·7<br>(1,358·5-1,802·4)	1,560·3<br>(1,347·8-1,788·5)	1,396·4<br>(1,197·7-1,608·0)	16·5<br>(16·2-16·8)	16·7<br>(16·4-16·9)	16·7<br>(16·5-17·0)	16·8<br>(16·3-17·2)
Laos	42·5<br>(29·6-55·5)	43·5<br>(30·4-57·8)	43·0<br>(30·0-57·4)	45·4<br>(27·9-62·6)	60·9<br>(50·0-72·5)	52·4<br>(42·2-63·9)	51·7<br>(41·5-63·0)	41·9<br>(32·7-51·4)	1,854·6<br>(1,593·4-2,134·4)	1,634·9<br>(1,366·8-1,909·3)	1,620·2<br>(1,353·2-1,894·3)	1,426·5<br>(1,179·2-1,705·1)	27·0<br>(25·7-28·8)	27·9<br>(26·5-29·7)	27·7<br>(26·4-29·5)	28·6<br>(26·9-30·9)
Malaysia	6·2<br>(3·4-10·1)	6·0<br>(3·1-9·9)	5·9<br>(3·1-9·8)	5·4<br>(2·6-9·0)	1·6<br>(0·4-4·3)	0·3<br>(0·1-0·7)	0·3<br>(0·1-0·7)	0·0<br>(0·0-0·1)	1,090·3<br>(944·5-1,253·5)	930·8<br>(792·0-1,080·0)	923·2<br>(784·5-1,072·5)	821·5<br>(685·1-966·1)	15·9<br>(15·4-16·6)	17·5<br>(16·8-18·3)	17·6<br>(16·9-18·4)	18·3<br>(17·5-19·2)
Maldives	38·5<br>(26·2-51·8)	29·9<br>(19·1-42·3)	28·9<br>(18·4-41·1)	21·9<br>(13·3-32·9)	24·2<br>(17·9-31·4)	2·6<br>(0·8-5·6)	2·3<br>(0·7-5·2)	0·5<br>(0·0-1·6)	1,059·3<br>(904·1-1,245·1)	753·8<br>(626·9-889·9)	746·6<br>(619·7-881·9)	650·1<br>(529·3-787·3)	29·6<br>(19·4-43·6)	26·8<br>(17·8-39·5)	26·9<br>(18·0-39·9)	27·6<br>(18·1-41·5)
Mauritius	4·5<br>(2·6-7·7)	5·0<br>(2·7-8·4)	5·0<br>(2·7-8·4)	5·2<br>(2·6-9·1)	2·9<br>(1·1-6·1)	1·0<br>(0·3-2·6)	0·9<br>(0·2-2·4)	0·3<br>(0·0-1·0)	839·5<br>(707·6-989·5)	745·1<br>(593·4-897·4)	741·4<br>(588·8-895·1)	689·3<br>(527·2-860·7)	18·2<br>(15·2-22·1)	14·4<br>(12·0-17·5)	14·5<br>(12·0-17·4)	14·9<br>(12·3-18·1)
Myanmar	46·7<br>(33·2-61·0)	43·9<br>(31·0-57·3)	43·4<br>(30·6-56·8)	39·6<br>(26·9-53·7)	60·1<br>(48·5-72·0)	42·5<br>(29·2-55·2)	41·1<br>(27·7-53·9)	24·3<br>(11·8-36·1)	1,227·9<br>(1,036·6-1,434·1)	1,109·6<br>(936·2-1,306·2)	1,099·2<br>(927·3-1,293·5)	963·8<br>(818·7-1,112·4)	44·2<br>(43·3-45·3)	49·0<br>(47·9-50·4)	48·8<br>(47·6-50·2)	50·1<br>(48·6-51·8)
Philippines	24·8<br>(16·2-35·3)	25·6<br>(16·2-37·7)	25·1<br>(15·8-37·0)	26·3<br>(13·5-41·4)	24·5<br>(18·5-31·0)	27·6<br>(18·9-36·9)	26·8<br>(18·1-36·3)	18·0<br>(9·3-28·8)	1,325·3<br>(1,136·8-1,529·0)	1,215·4<br>(1,042·9-1,407·3)	1,206·7<br>(1,033·7-1,399·0)	1,089·6<br>(913·2-1,294·8)	25·3<br>(24·2-26·5)	23·2<br>(22·3-24·3)	23·2<br>(22·3-24·3)	23·2<br>(21·9-25·4)
Sri Lanka	26·7<br>(17·0-37·8)	18·2<br>(10·8-27·8)	17·5<br>(10·4-26·9)	11·5<br>(6·5-18·8)	40·8<br>(31·7-51·3)	26·2<br>(15·8-37·5)	24·8<br>(14·4-36·3)	10·6<br>(3·3-20·8)	904·1<br>(763·6-1,054·9)	799·4<br>(655·2-943·3)	789·5<br>(646·1-933·4)	664·2<br>(532·4-810·9)	30·8<br>(29·1-32·6)	25·8<br>(24·3-27·3)	25·6<br>(24·2-27·2)	25·5<br>(23·9-27·4)
Seychelles	7·5<br>(4·1-12·1)	7·8<br>(4·3-12·5)	7·7<br>(4·3-12·4)	8·1<br>(3·8-13·4)	1·7<br>(0·4-4·2)	0·5<br>(0·1-1·5)	0·5<br>(0·1-1·4)	0·1<br>(0·0-0·4)	1,011·4<br>(860·5-1,185·3)	852·4<br>(716·0-1,008·4)	845·1<br>(709·3-1,000·6)	745·9<br>(619·6-895·2)	19·4<br>(8·8-37·4)	14·9<br>(7·2-29·5)	14·8<br>(6·9-26·7)	14·5<br>(6·5-27·7)
Thailand	30·9<br>(19·7-44·1)	30·5<br>(19·5-44·6)	30·2<br>(19·2-44·1)	29·0<br>(16·4-44·9)	19·5<br>(14·5-25·6)	6·0<br>(2·8-10·5)	5·4<br>(2·4-9·8)	1·1<br>(0·2-3·1)	1,418·1<br>(1,211·3-1,642·1)	1,199·8<br>(1,000·5-1,408·2)	1,188·3<br>(989·8-1,396·2)	1,037·8<br>(852·0-1,236·6)	23·8<br>(23·5-24·2)	23·3<br>(22·9-23·7)	23·2<br>(22·8-23·6)	23·7<br>(23·2-24·2)
Timor-Leste	31·2<br>(19·8-44·2)	33·3<br>(21·9-46·6)	32·4<br>(21·2-45·7)	35·1<br>(15·7-51·0)	61·1<br>(50·3-72·7)	47·6<br>(36·6-59·3)	46·5<br>(35·5-58·2)	31·9<br>(22·2-42·1)	1,029·6<br>(766·0-1,252·6)	872·0<br>(682·9-1,045·5)	866·0<br>(681·1-1,037·2)	776·7<br>(646·2-916·5)	20·2<br>(16·4-25·3)	17·1<br>(13·9-21·3)	17·0<br>(13·9-21·1)	16·5<br>(13·4-20·5)
Vietnam	32·9<br>(22·2-45·9)	32·1<br>(22·5-42·9)	31·7<br>(22·1-42·4)	30·3<br>(18·3-40·6)	42·6<br>(33·5-52·4)	18·1<br>(12·0-26·0)	16·9<br>(10·9-24·9)	6·0<br>(2·7-11·9)	1,122·5<br>(948·5-1,306·8)	1,089·7<br>(921·5-1,268·6)	1,081·4<br>(914·4-1,259·7)	971·8<br>(817·1-1,139·5)	27·1<br>(26·4-27·8)	26·4<br>(25·7-27·2)	26·3<br>(25·5-27·2)	26·7<br>(25·9-27·6)
**Oceania**																
Federated States of Micronesia	23·0<br>(14·1-34·2)	17·0<br>(10·4-25·7)	16·8<br>(10·3-25·3)	12·9<br>(6·8-20·0)	26·0<br>(18·2-34·4)	13·8<br>(8·3-21·1)	13·1<br>(7·8-20·2)	6·2<br>(3·2-10·9)	1,210·9<br>(997·4-1,451·2)	1,217·1<br>(981·0-1,484·6)	1,216·9<br>(979·7-1,485·9)	1,212·4<br>(958·6-1,495·2)	9·3<br>(4·6-18·7)	8·5<br>(4·1-16·3)	8·7<br>(3·8-17·3)	8·8<br>(3·8-17·7)
Fiji	13·2<br>(7·9-21·1)	11·0<br>(6·2-17·7)	10·9<br>(6·1-17·6)	9·0<br>(4·7-14·8)	19·8<br>(12·9-27·9)	9·0<br>(5·1-14·3)	8·5<br>(4·9-13·7)	3·8<br>(2·1-6·9)	1,258·4<br>(1,006·9-1,524·5)	1,110·1<br>(841·9-1,426·6)	1,102·1<br>(832·9-1,426·0)	995·2<br>(704·1-1,415·7)	8·7<br>(6·3-12·7)	7·5<br>(5·4-11·0)	7·7<br>(5·3-11·6)	7·9<br>(4·5-12·7)

Location	Indicator 16.1.1: Age-standardised death rate due to interpersonal violence (per 100,000 population)				Indicator 16.1.2: Death rate due to conflict and terrorism (per 100,000 population)				Indicator 16.1.3: Age-standardised prevalence of physical or sexual violence experienced by populations in the last 12 months (%)				Indicator 16.2.3: Age-standardised prevalence of women and men aged 18-29 years who experienced sexual violence by age 18 (%)			
	**2000**	**2015**	**2016**	**2030**	**2000**	**2015**	**2016**	**2030**	**2000**	**2015**	**2016**	**2030**	**2000**	**2015**	**2016**	**2030**
Guyana	19·0 (13·3-26·5)	20·1 (12·5-26·4)	19·2 (12·0-25·3)	17·1 (9·9-25·5)	0·0 (0·0-0·0)	0·0 (0·0-0·0)	0·0 (0·0-0·0)	0·0 (0·0-0·0)	7·4 (6·3-8·6)	6·6 (5·7-7·7)	6·5 (5·6-7·6)	5·8 (5·0-6·7)	7·4 (5·6-9·3)	7·3 (5·6-9·1)	7·3 (5·6-9·1)	7·2 (5·6-9·0)
Haiti	11·3 (6·1-14·7)	7·0 (5·0-9·5)	7·1 (5·0-9·6)	7·4 (3·7-13·5)	0·2 (0·03-0·3)	0·2 (0·03-0·3)	0·0 (0·0-0·0)	0·0 (0·0-0·0)	11·9 (10·8-13·0)	9·9 (8·9-11·0)	9·8 (8·8-11·0)	8·9 (7·8-10·2)	7·3 (5·6-9·1)	7·4 (5·6-9·2)	7·4 (5·6-9·2)	7·4 (5·6-9·3)
Jamaica	15·9 (13·3-20·3)	23·1 (15·2-29·2)	22·6 (15·1-29·0)	17·6 (8·9-32·2)	0·0 (0·0-0·0)	0·0 (0·0-0·0)	0·0 (0·0-0·0)	0·0 (0·0-0·0)	5·5 (4·9-6·1)	5·3 (4·8-5·9)	5·3 (4·7-5·9)	5·0 (4·4-5·5)	8·3 (6·4-10·4)	8·4 (6·4-10·4)	8·4 (6·4-10·5)	8·5 (6·4-10·7)
Saint Lucia	13·6 (9·3-18·4)	15·3 (8·6-19·3)	15·2 (8·5-19·3)	13·9 (7·2-21·9)	0·0 (0·0-0·0)	0·0 (0·0-0·0)	0·0 (0·0-0·0)	0·0 (0·0-0·0)	6·5 (5·6-7·6)	6·1 (5·3-7·1)	6·1 (5·2-7·1)	5·5 (4·8-6·4)	7·4 (5·6-9·2)	7·4 (5·6-9·2)	7·4 (5·6-9·2)	7·4 (5·6-9·2)
Saint Vincent and the Grenadines	13·6 (10·0-20·0)	20·2 (11·6-25·6)	19·8 (11·3-25·2)	26·1 (11·5-44·2)	0·0 (0·0-0·0)	0·0 (0·0-0·0)	0·0 (0·0-0·0)	0·0 (0·0-0·0)	6·9 (6·0-8·1)	6·7 (5·8-7·8)	6·7 (5·8-7·7)	6·2 (5·4-7·2)	7·4 (5·6-9·2)	7·4 (5·6-9·3)	7·4 (5·6-9·3)	7·4 (5·6-9·3)
Suriname	7·7 (6·3-9·1)	9·3 (6·1-11·1)	9·3 (6·0-11·1)	10·1 (5·3-14·8)	0·0 (0·0-0·0)	0·0 (0·0-0·0)	0·0 (0·0-0·0)	0·0 (0·0-0·0)	6·5 (5·6-7·5)	5·9 (5·1-6·9)	5·8 (5·0-6·8)	5·1 (4·4-6·0)	7·4 (5·6-9·3)	7·4 (5·6-9·3)	7·4 (5·6-9·3)	7·4 (5·6-9·3)
Trinidad and Tobago	12·7 (9·1-18·1)	19·9 (10·0-26·4)	19·4 (9·8-26·0)	13·9 (6·9-22·7)	0·0 (0·0-0·0)	0·0 (0·0-0·0)	0·0 (0·0-0·0)	0·0 (0·0-0·0)	6·0 (5·2-7·0)	5·9 (5·1-6·9)	5·9 (5·1-6·9)	5·3 (4·6-6·2)	7·4 (5·6-9·2)	7·5 (5·6-9·4)	7·5 (5·6-9·4)	7·5 (5·7-9·5)
**Tropical Latin America**																
Brazil	28·8 (20·8-36·8)	27·2 (18·7-33·6)	26·8 (18·5-33·2)	25·5 (16·6-31·3)	0·0 (0·0-0·0)	0·0 (0·0-0·0)	0·0 (0·0-0·0)	0·0 (0·0-0·0)	6·5 (5·8-7·3)	5·8 (5·2-6·5)	5·8 (5·2-6·4)	5·1 (4·6-5·7)	6·4 (5·0-8·2)	6·6 (5·1-8·4)	6·6 (5·1-8·4)	6·7 (5·1-8·6)
Paraguay	16·7 (12·6-18·8)	14·2 (11·7-18·2)	14·3 (11·5-18·1)	14·2 (8·3-23·0)	0·0 (0·0-0·0)	0·0 (0·0-0·0)	0·0 (0·0-0·0)	0·0 (0·0-0·0)	6·2 (5·6-7·0)	5·3 (4·7-6·0)	5·3 (4·7-5·9)	4·7 (4·1-5·3)	5·5 (4·3-6·8)	5·4 (4·1-6·7)	5·3 (4·1-6·6)	5·3 (4·1-6·6)
**East Asia**																
China	3·4 (2·4-3·7)	1·3 (1·2-2·0)	1·3 (1·1-1·9)	0·6 (0·4-1·3)	0·02 (0·0-0·03)	0·02 (0·0-0·03)	0·0 (0·0-0·0)	0·0 (0·0-0·0)	8·7 (7·8-9·7)	7·2 (6·4-8·0)	7·1 (6·4-7·9)	6·0 (5·4-6·7)	8·1 (6·1-10·3)	8·2 (6·2-10·4)	8·2 (6·2-10·5)	8·3 (6·2-10·7)
North Korea	3·0 (2·3-3·8)	2·7 (2·0-3·6)	2·6 (2·0-3·6)	2·4 (1·5-3·6)	0·0 (0·0-0·0)	0·0 (0·0-0·0)	0·0 (0·0-0·0)	0·0 (0·0-0·0)	9·6 (8·5-10·8)	10·0 (8·9-11·2)	10·0 (8·9-11·2)	9·8 (8·7-11·1)	6·8 (5·1-8·8)	6·8 (5·1-8·5)	6·8 (5·1-8·5)	6·8 (5·1-8·6)
Taiwan (Province of China)	4·2 (1·9-5·0)	1·4 (1·2-2·0)	1·4 (1·2-2·0)	0·8 (0·5-1·5)	0·0 (0·0-0·0)	0·0 (0·0-0·0)	0·0 (0·0-0·0)	0·0 (0·0-0·0)	6·6 (5·9-7·5)	5·8 (5·2-6·6)	5·8 (5·1-6·5)	5·4 (4·8-6·1)	6·2 (4·7-7·8)	6·1 (4·7-7·8)	6·1 (4·7-7·8)	6·1 (4·6-7·8)
**Southeast Asia**																
Cambodia	4·7 (3·0-5·4)	4·0 (2·6-5·3)	4·0 (2·6-5·3)	3·9 (2·1-6·7)	0·0 (0·0-0·0)	0·0 (0·0-0·0)	0·0 (0·0-0·0)	0·0 (0·0-0·0)	8·2 (7·3-9·2)	6·8 (6·0-7·6)	6·7 (6·0-7·5)	5·9 (5·2-6·7)	9·2 (7·4-11·3)	9·2 (7·5-11·1)	9·2 (7·5-11·1)	9·1 (7·6-10·9)
Indonesia	2·0 (1·4-2·3)	1·8 (1·3-2·0)	1·8 (1·2-2·0)	1·6 (1·1-2·0)	0·4 (0·2-0·5)	0·0 (0·0-0·0)	0·0 (0·0-0·0)	0·0 (0·0-0·0)	7·1 (6·2-8·0)	6·1 (5·3-6·9)	6·0 (5·2-6·8)	5·1 (4·4-5·8)	7·6 (5·7-9·7)	7·6 (5·8-9·7)	7·6 (5·7-9·7)	7·6 (5·8-9·7)
Laos	0·7 (0·5-1·0)	0·6 (0·4-0·8)	0·6 (0·4-0·8)	0·5 (0·3-0·9)	0·0 (0·0-0·0)	0·0 (0·0-0·0)	0·0 (0·0-0·0)	0·0 (0·0-0·0)	7·8 (6·7-9·3)	6·4 (5·4-7·6)	6·3 (5·3-7·5)	5·2 (4·4-6·2)	7·9 (5·9-10·1)	7·9 (5·9-10·1)	7·9 (5·9-10·1)	7·9 (5·9-10·2)
Malaysia	2·5 (2·1-3·6)	2·0 (1·5-3·2)	1·9 (1·4-3·2)	1·4 (0·7-2·8)	0·0 (0·0-0·0)	0·0 (0·0-0·0)	0·0 (0·0-0·0)	0·0 (0·0-0·0)	6·9 (5·9-8·1)	5·9 (5·0-7·0)	5·8 (4·9-6·9)	4·9 (4·2-5·8)	9·2 (6·9-11·7)	9·2 (6·9-11·8)	9·2 (6·9-11·8)	9·2 (6·9-11·8)
Maldives	1·4 (0·8-1·7)	1·1 (0·5-1·5)	1·1 (0·5-1·5)	1·0 (0·3-2·3)	0·0 (0·0-0·0)	0·0 (0·0-0·0)	0·0 (0·0-0·0)	0·0 (0·0-0·0)	7·3 (6·2-8·6)	5·8 (4·9-6·9)	5·7 (4·8-6·8)	4·7 (3·9-5·5)	7·9 (6·0-10·1)	7·9 (5·9-10·2)	7·9 (5·9-10·2)	8·0 (5·8-10·3)
Mauritius	2·3 (1·7-4·1)	2·5 (1·6-3·5)	2·5 (1·6-3·5)	2·1 (1·1-3·5)	0·0 (0·0-0·0)	0·0 (0·0-0·0)	0·0 (0·0-0·0)	0·0 (0·0-0·0)	6·3 (5·4-7·5)	5·9 (5·0-7·0)	5·8 (5·0-6·9)	5·1 (4·4-6·0)	7·9 (5·9-10·1)	7·9 (5·9-10·1)	7·9 (5·9-10·2)	7·9 (5·9-10·1)
Myanmar	2·8 (2·0-3·3)	2·1 (1·5-2·8)	2·1 (1·4-2·8)	1·7 (0·9-2·9)	1·0 (0·7-1·3)	1·3 (0·2-2·4)	0·0 (0·0-0·0)	0·0 (0·0-0·0)	7·8 (6·9-8·7)	5·9 (5·3-6·5)	5·8 (5·2-6·4)	4·6 (4·2-5·2)	7·9 (5·9-10·2)	7·9 (5·9-10·2)	7·9 (5·9-10·2)	7·9 (5·9-10·2)
Philippines	18·2 (7·9-23·4)	15·0 (7·7-19·7)	15·0 (7·6-20·0)	14·2 (6·6-26·6)	2·2 (2·1-2·4)	0·6 (0·4-0·7)	2·0 (1·0-3·1)	3·7 (0·2-8·7)	6·8 (6·1-7·6)	5·7 (5·1-6·4)	5·7 (5·1-6·4)	5·4 (4·8-6·2)	7·1 (5·4-9·2)	7·1 (5·4-9·2)	7·1 (5·4-9·2)	7·1 (5·3-9·2)
Sri Lanka	8·4 (5·5-9·8)	4·7 (3·4-6·4)	4·7 (3·3-6·4)	4·1 (1·8-8·3)	13·7 (5·3-22·0)	0·0 (0·0-0·0)	0·0 (0·0-0·0)	0·0 (0·0-0·0)	10·2 (9·0-11·5)	8·5 (7·5-9·6)	8·4 (7·4-9·5)	6·9 (6·1-7·8)	8·9 (7·4-10·5)	8·8 (7·2-10·5)	8·8 (7·2-10·5)	8·8 (7·2-10·5)
Seychelles	9·5 (5·0-11·5)	6·1 (4·1-7·5)	6·1 (4·0-7·6)	4·4 (2·6-6·9)	0·0 (0·0-0·0)	0·0 (0·0-0·0)	0·0 (0·0-0·0)	0·0 (0·0-0·0)	7·7 (6·6-9·1)	6·9 (5·9-8·2)	6·9 (5·9-8·1)	6·4 (5·5-7·6)	7·9 (5·9-10·2)	7·9 (5·9-10·2)	7·9 (5·9-10·2)	7·9 (5·9-10·2)
Thailand	12·1 (6·4-14·9)	6·4 (4·6-7·7)	6·4 (4·5-8·0)	5·6 (3·2-9·2)	0·0 (0·0-0·0)	0·0 (0·0-0·0)	0·0 (0·0-0·0)	0·0 (0·0-0·0)	8·5 (7·2-10·1)	6·7 (5·7-8·0)	6·7 (5·7-8·0)	5·9 (5·0-7·0)	5·9 (4·5-7·4)	5·9 (4·5-7·4)	5·9 (4·5-7·4)	5·9 (4·5-7·5)
Timor-Leste	3·4 (1·9-4·6)	2·9 (1·4-4·1)	2·9 (1·5-4·2)	2·8 (1·0-5·3)	10·5 (6·7-14·3)	0·0 (0·0-0·0)	0·0 (0·0-0·0)	0·0 (0·0-0·0)	12·7 (11·3-14·0)	10·6 (9·4-11·7)	10·4 (9·2-11·6)	8·0 (6·8-9·3)	9·4 (7·2-11·8)	9·4 (7·2-11·8)	9·4 (7·2-11·8)	9·5 (7·3-12·0)
Vietnam	2·0 (1·5-2·7)	1·9 (1·3-2·5)	1·9 (1·3-2·5)	1·6 (0·8-2·9)	0·0 (0·0-0·0)	0·0 (0·0-0·0)	0·0 (0·0-0·0)	0·0 (0·0-0·0)	6·9 (6·1-7·8)	5·9 (5·3-6·6)	5·9 (5·2-6·6)	5·4 (4·8-6·1)	7·3 (5·5-9·3)	7·3 (5·5-9·3)	7·3 (5·5-9·3)	7·3 (5·5-9·3)
**Oceania**																
Federated States of Micronesia	7·1 (4·5-9·4)	7·3 (4·7-10·3)	7·1 (4·4-10·1)	7·2 (3·4-12·3)	0·0 (0·0-0·0)	0·0 (0·0-0·0)	0·0 (0·0-0·0)	0·0 (0·0-0·0)	9·7 (8·5-10·9)	8·6 (7·5-9·7)	8·5 (7·4-9·6)	7·7 (6·7-8·7)	13·9 (10·5-17·6)	14·2 (10·7-18·0)	14·2 (10·7-18·0)	14·4 (10·8-18·3)
Fiji	5·9 (3·7-7·4)	5·3 (3·1-7·2)	5·4 (3·1-7·3)	6·3 (2·4-12·9)	0·0 (0·0-0·0)	0·0 (0·0-0·0)	0·0 (0·0-0·0)	0·0 (0·0-0·0)	8·0 (7·1-9·1)	7·5 (6·6-8·6)	7·5 (6·6-8·5)	7·2 (6·3-8·3)	14·0 (10·6-17·8)	14·1 (10·6-17·9)	14·1 (10·6-17·9)	14·1 (10·6-17·9)

Location	Indicator 17.19.2c: Percentage of well-certified deaths by a vital registration (VR) system among a country's total population (%)															
	2000	2015	2016	2030												
Guyana	70·3 (65·9-74·5)	75·4 (67·5-82·1)	76·1 (67·8-83·3)	84·7 (68·7-94·6)												
Haiti	8·8 (5·9-12·8)	8·9 (5·2-13·9)	9·0 (5·2-14·0)	14·8 (5·3-31·3)												
Jamaica	63·4 (57·6-69·0)	74·1 (66·1-81·8)	74·2 (65·9-82·3)	81·6 (67·4-92·3)												
Saint Lucia	71·5 (67·2-75·4)	82·2 (76·9-86·7)	82·4 (76·7-87·4)	87·0 (79·4-92·8)												
Saint Vincent and the Grenadines	75·7 (71·8-79·2)	82·6 (78·0-86·6)	83·0 (78·0-87·2)	91·0 (82·9-96·0)												
Suriname	58·3 (53·6-63·3)	64·5 (57·8-71·0)	65·2 (57·5-72·4)	72·9 (54·9-87·8)												
Trinidad and Tobago	85·5 (82·4-88·3)	89·3 (84·4-93·3)	89·4 (84·2-93·3)	90·9 (81·6-96·0)												
**Tropical Latin America**																
Brazil	68·9 (67·8-69·8)	81·6 (80·5-82·7)	81·9 (80·6-83·1)	89·4 (87·9-90·8)												
Paraguay	55·0 (50·9-59·0)	66·5 (60·0-72·0)	67·4 (60·5-73·2)	76·2 (62·6-85·5)												
**East Asia**																
China	75·3 (74·2-76·4)	74·5 (73·4-75·6)	75·4 (74·0-76·5)	76·4 (73·5-79·2)												
North Korea	0·0 (0·0-0·0)	0·0 (0·0-0·0)	0·0 (0·0-0·0)	0·0 (0·0-0·0)												
Taiwan (Province of China)	37·8 (31·9-43·8)	82·5 (76·5-87·7)	82·4 (75·7-88·0)	89·5 (80·8-94·9)												
**Southeast Asia**																
Cambodia	0·0 (0·0-0·0)	0·0 (0·0-0·0)	0·0 (0·0-0·0)	0·0 (0·0-0·0)												
Indonesia	0·0 (0·0-0·0)	0·0 (0·0-0·0)	0·0 (0·0-0·0)	0·0 (0·0-0·0)												
Laos	0·0 (0·0-0·0)	0·0 (0·0-0·0)	0·0 (0·0-0·0)	0·0 (0·0-0·0)												
Malaysia	33·4 (31·1-36·0)	49·2 (45·6-52·9)	50·2 (46·3-54·0)	66·7 (59·6-72·7)												
Maldives	35·2 (31·9-38·5)	58·3 (54·8-61·9)	59·3 (55·8-62·9)	76·2 (70·8-81·0)												
Mauritius	78·7 (76·7-80·7)	84·7 (83·0-86·5)	85·1 (83·2-86·8)	88·9 (85·7-91·6)												
Myanmar	0·0 (0·0-0·0)	0·0 (0·0-0·0)	0·0 (0·0-0·0)	0·0 (0·0-0·0)												
Philippines	66·2 (63·8-68·6)	74·2 (71·4-76·9)	74·7 (72·0-77·5)	81·2 (76·5-85·4)												
Sri Lanka	58·0 (55·3-60·5)	72·7 (70·0-75·5)	73·7 (71·0-76·6)	85·6 (81·5-88·9)												
Seychelles	70·5 (67·6-73·4)	77·3 (75·0-79·6)	77·9 (75·6-80·3)	84·5 (80·3-87·9)												
Thailand	37·3 (34·6-39·9)	54·6 (51·1-58·0)	55·2 (51·8-58·7)	66·0 (59·9-72·2)												
Timor-Leste	0·0 (0·0-0·0)	0·0 (0·0-0·0)	0·0 (0·0-0·0)	0·0 (0·0-0·0)												
Vietnam	0·0 (0·0-0·0)	0·0 (0·0-0·0)	0·0 (0·0-0·0)	0·0 (0·0-0·0)												
**Oceania**																
Federated States of Micronesia	0·0 (0·0-0·0)	0·0 (0·0-0·0)	0·0 (0·0-0·0)	0·0 (0·0-0·0)												
Fiji	43·7 (37·7-49·8)	64·0 (54·6-72·6)	64·3 (54·1-73·9)	75·4 (57·6-88·5)												

Location	Indicator 1.5.1: Death rate due to exposure to forces of nature (per 100,000 population)				Indicator 2.2.1: Prevalence of stunting in children under 5 (%)				Indicator 2.2.2a: Prevalence of wasting in children under 5 (%)				Indicator 2.2.2b: Prevalence of overweight in children aged 2-4 (%)			
	**2000**	**2015**	**2016**	**2030**	**2000**	**2015**	**2016**	**2030**	**2000**	**2015**	**2016**	**2030**	**2000**	**2015**	**2016**	**2030**
Kiribati	0·0 (0·0-0·0)	0·0 (0·0-0·0)	0·0 (0·0-0·0)	0·0 (0·0-0·0)	25·6 (20·1-30·9)	19·2 (14·4-23·9)	18·8 (14·2-23·6)	14·4 (10·3-18·5)	10·1 (7·4-13·2)	8·8 (6·2-12·0)	8·7 (6·2-12·0)	7·7 (5·2-11·0)	33·5 (12·7-58·8)	38·4 (15·9-61·7)	38·8 (15·4-63·3)	44·8 (8·2-84·4)
Marshall Islands	0·0 (0·0-0·0)	0·0 (0·0-0·0)	0·0 (0·0-0·0)	0·0 (0·0-0·0)	16·7 (12·3-21·8)	13·5 (9·8-18·0)	13·2 (9·5-17·6)	9·7 (6·5-14·2)	5·5 (3·9-7·5)	5·8 (4·1-8·0)	5·7 (4·1-8·0)	5·3 (3·7-7·5)	28·7 (10·8-53·3)	34·4 (14·4-59·9)	34·8 (14·3-59·9)	41·5 (8·0-82·3)
Papua New Guinea	6·5 (5·2-7·9)	0·2 (0·2-0·3)	0·2 (0·1-0·2)	0·8 (0·6-0·9)	53·0 (45·9-61·6)	47·9 (41·5-57·0)	47·3 (40·5-56·9)	39·2 (31·3-53·9)	14·0 (11·8-16·4)	12·4 (9·8-15·2)	11·9 (9·3-14·9)	13·0 (10·1-14·4)	13·7 (6·4-25·8)	17·6 (5·3-37·7)	18·0 (5·5-38·6)	22·7 (2·5-60·6)
Samoa	0·1 (0·1-0·2)	6·6 (5·1-8·0)	5·0 (3·9-6·1)	5·1 (4·0-6·2)	8·0 (6·1-10·3)	6·9 (4·9-9·3)	6·8 (4·8-9·2)	5·6 (3·8-7·3)	2·3 (1·6-3·2)	2·3 (1·5-3·2)	2·3 (1·5-3·2)	2·1 (1·3-3·0)	41·9 (18·5-66·9)	47·5 (22·5-72·8)	47·8 (22·1-73·6)	53·0 (13·6-90·3)
Solomon Islands	0·0 (0·0-0·0)	2·3 (1·8-2·8)	1·8 (1·5-2·2)	1·6 (1·3-1·9)	28·5 (23·1-34·8)	22·8 (17·7-28·1)	22·1 (17·0-27·4)	14·3 (9·3-20·5)	5·4 (4·2-6·9)	4·8 (3·7-6·4)	4·8 (3·7-6·5)	4·6 (3·1-6·9)	24·1 (8·5-46·2)	28·6 (10·6-53·9)	29·0 (10·7-54·8)	36·2 (6·0-79·9)
Tonga	0·0 (0·0-0·0)	0·0 (0·0-0·0)	0·0 (0·0-0·0)	0·0 (0·0-0·0)	10·1 (7·3-13·7)	8·1 (6·0-10·7)	7·9 (5·8-10·6)	6·0 (4·4-8·3)	7·0 (5·2-9·2)	5·5 (4·7-6·4)	5·5 (4·6-6·4)	4·2 (3·7-4·5)	42·7 (19·3-68·6)	49·7 (24·5-73·7)	50·1 (24·8-75·0)	56·3 (14·3-90·6)
Vanuatu	11·0 (8·5-13·5)	1·5 (1·1-1·9)	1·3 (1·0-1·7)	1·7 (1·3-2·1)	24·1 (19·1-28·9)	23·1 (18·8-27·2)	22·5 (18·1-26·8)	20·9 (18·6-26·0)	6·4 (5·4-7·5)	4·8 (4·0-5·6)	4·7 (4·0-5·5)	3·6 (3·0-4·5)	13·4 (4·2-29·2)	17·7 (6·0-38·2)	17·8 (6·1-37·6)	21·6 (2·9-58·5)
**North Africa and Middle East**																
Afghanistan	6·8 (2·1-11·6)	1·0 (0·7-1·4)	0·8 (0·5-1·1)	1·4 (0·8-2·1)	63·8 (59·3-68·0)	53·8 (46·1-62·3)	53·1 (45·2-61·8)	43·2 (34·0-54·9)	9·1 (8·0-10·0)	9·3 (7·5-11·1)	9·3 (7·5-11·2)	9·8 (7·4-12·4)	14·9 (7·5-25·4)	21·6 (11·2-35·8)	22·1 (11·4-37·1)	30·0 (8·0-65·1)
Algeria	0·08 (0·03-0·1)	0·06 (0·03-0·08)	0·04 (0·02-0·06)	0·3 (0·1-0·5)	34·1 (31·1-37·2)	11·6 (8·6-14·7)	11·1 (8·2-14·2)	6·5 (4·4-8·7)	11·1 (10·2-12·0)	5·4 (4·4-6·6)	5·2 (4·2-6·4)	2·9 (2·2-4·0)	14·4 (7·2-24·3)	27·9 (15·7-42·9)	28·6 (16·3-44·0)	44·7 (18·0-74·9)
Bahrain	0·0 (0·0-0·0)	0·0 (0·0-0·0)	0·0 (0·0-0·0)	0·0 (0·0-0·0)	8·8 (6·4-11·5)	3·0 (2·1-3·9)	2·9 (2·1-3·9)	1·2 (0·8-2·3)	5·4 (4·5-6·3)	2·7 (2·1-3·3)	2·7 (2·0-3·3)	1·6 (1·2-2·2)	17·2 (9·5-28·2)	17·2 (9·3-27·4)	17·9 (9·4-28·1)	24·1 (6·5-51·2)
Egypt	0·1 (0·05-0·2)	0·01 (0·0-0·01)	0·0 (0·0-0·01)	0·01 (0·01-0·02)	26·5 (23·4-29·6)	20·8 (17·3-24·2)	19·8 (16·4-23·2)	17·7 (14·0-21·2)	6·1 (5·6-6·6)	6·9 (6·1-7·7)	6·9 (6·0-7·6)	7·7 (6·6-8·9)	29·0 (23·0-35·0)	23·2 (16·7-31·3)	23·5 (16·4-32·0)	19·7 (9·6-34·1)
Iran	0·7 (0·2-1·2)	0·1 (0·05-0·2)	0·08 (0·03-0·1)	1·9 (0·6-3·2)	18·3 (15·7-20·9)	8·0 (5·9-10·5)	7·9 (5·8-10·4)	4·3 (2·9-6·5)	5·5 (5·1-6·0)	4·8 (3·6-6·3)	4·8 (3·6-6·4)	4·6 (2·5-7·9)	13·0 (6·4-22·3)	19·3 (10·4-31·9)	19·5 (10·5-31·9)	24·7 (7·6-48·3)
Iraq	0·0 (0·0-0·0)	0·03 (0·01-0·05)	0·03 (0·01-0·05)	0·02 (0·0-0·03)	28·8 (26·2-31·1)	24·7 (20·3-28·8)	24·4 (19·9-28·5)	20·4 (14·4-26·3)	10·4 (9·3-11·2)	7·3 (6·0-8·7)	7·2 (5·9-8·6)	6·2 (4·6-8·0)	15·5 (8·8-24·8)	28·3 (21·1-36·0)	28·8 (20·2-38·8)	39·1 (17·1-63·9)
Jordan	0·0 (0·0-0·0)	0·0 (0·0-0·0)	0·0 (0·0-0·0)	0·0 (0·0-0·0)	11·8 (9·4-14·5)	8·4 (6·3-11·0)	8·2 (6·0-10·8)	5·2 (3·0-8·6)	3·7 (3·1-4·6)	3·5 (2·7-4·4)	3·4 (2·6-4·4)	2·4 (1·8-3·4)	10·9 (6·9-16·2)	14·7 (8·7-23·3)	15·1 (8·7-24·3)	19·6 (5·7-42·9)
Kuwait	0·01 (0·0-0·02)	0·0 (0·0-0·0)	0·0 (0·0-0·0)	0·0 (0·0-0·0)	5·3 (3·8-7·1)	3·0 (2·2-4·1)	3·0 (2·2-4·0)	2·0 (1·4-2·6)	3·2 (2·7-3·7)	2·1 (1·5-2·8)	2·0 (1·5-2·7)	1·6 (1·0-2·3)	40·7 (25·9-57·7)	53·6 (38·1-68·9)	53·7 (37·1-68·9)	65·2 (36·3-90·6)
Lebanon	0·03 (0·01-0·06)	0·0 (0·0-0·0)	0·0 (0·0-0·0)	0·0 (0·0-0·0)	18·2 (14·2-22·1)	12·5 (9·4-15·9)	12·2 (9·1-15·6)	8·1 (5·1-11·6)	4·9 (3·9-6·0)	4·3 (3·4-5·4)	4·3 (3·4-5·4)	3·8 (2·7-5·1)	31·1 (20·9-41·8)	24·5 (15·2-35·1)	25·3 (15·4-36·6)	27·3 (9·1-52·2)
Libya	0·0 (0·0-0·0)	0·04 (0·01-0·07)	0·03 (0·01-0·06)	0·02 (0·01-0·04)	19·4 (15·1-23·8)	15·3 (11·4-19·6)	14·9 (11·0-19·2)	10·2 (6·1-14·9)	4·4 (3·8-5·1)	5·1 (3·9-6·5)	5·0 (3·8-6·5)	5·2 (3·7-7·2)	34·2 (20·1-50·9)	44·3 (27·9-60·6)	44·0 (28·4-60·1)	53·0 (25·5-79·7)
Morocco	0·3 (0·08-0·5)	0·04 (0·02-0·07)	0·04 (0·02-0·06)	0·1 (0·05-0·2)	24·8 (20·5-29·1)	17·4 (12·9-21·8)	16·9 (12·4-21·6)	11·7 (8·1-15·7)	7·2 (6·1-8·6)	4·5 (3·6-5·7)	4·4 (3·5-5·6)	3·1 (2·3-4·2)	21·5 (15·1-29·2)	31·1 (17·7-47·1)	31·6 (17·6-47·1)	41·6 (15·6-72·2)
Palestine	0·0 (0·0-0·0)	0·0 (0·0-0·0)	0·0 (0·0-0·0)	0·0 (0·0-0·0)	11·7 (9·7-13·9)	9·6 (7·3-12·3)	9·0 (6·8-11·7)	3·5 (2·1-5·6)	4·9 (4·1-5·7)	3·1 (2·4-3·9)	2·9 (2·1-3·7)	1·7 (1·2-2·4)	16·8 (12·2-22·1)	16·6 (11·0-23·2)	17·2 (10·9-24·6)	21·5 (8·7-40·1)
Oman	0·0 (0·0-0·0)	0·2 (0·08-0·3)	0·1 (0·05-0·2)	0·2 (0·1-0·4)	13·8 (11·3-16·3)	11·5 (9·0-13·9)	11·5 (9·1-13·9)	8·8 (7·4-11·7)	8·9 (7·9-10·1)	7·5 (6·6-8·5)	7·5 (6·6-8·5)	6·3 (5·8-6·9)	21·4 (11·6-34·4)	46·2 (29·5-63·2)	46·6 (29·3-64·0)	70·8 (41·8-92·6)
Qatar	0·0 (0·0-0·0)	0·0 (0·0-0·0)	0·0 (0·0-0·0)	0·0 (0·0-0·0)	9·7 (7·2-12·6)	7·4 (5·4-10·0)	7·3 (5·4-9·8)	5·6 (4·0-7·8)	2·9 (2·1-3·9)	2·7 (2·0-3·7)	2·7 (1·9-3·6)	2·4 (1·7-3·3)	35·2 (21·7-50·0)	46·6 (30·4-63·2)	47·0 (30·3-63·5)	57·0 (27·0-83·4)
Saudi Arabia	0·0 (0·0-0·0)	0·03 (0·02-0·05)	0·03 (0·01-0·04)	0·03 (0·02-0·05)	14·7 (13·3-16·1)	6·9 (6·1-7·8)	6·6 (5·9-7·5)	3·1 (2·7-3·7)	5·6 (5·0-6·3)	4·5 (4·0-5·2)	4·4 (3·9-5·0)	3·1 (2·7-3·6)	31·8 (18·2-47·7)	48·5 (31·6-66·0)	49·7 (33·2-67·7)	66·4 (35·1-90·5)
Sudan	0·08 (0·03-0·1)	0·06 (0·02-0·1)	0·1 (0·05-0·2)	0·09 (0·04-0·1)	45·5 (41·4-50·1)	33·0 (28·1-38·1)	32·4 (27·4-37·4)	23·9 (18·9-28·4)	18·9 (16·5-21·3)	14·0 (11·3-16·9)	13·7 (11·0-16·6)	10·4 (7·4-13·3)	9·5 (5·0-16·2)	11·1 (6·3-18·6)	11·5 (6·3-19·7)	13·1 (3·7-31·4)
Syria	0·1 (0·05-0·2)	0·0 (0·0-0·0)	0·0 (0·0-0·0)	0·03 (0·01-0·04)	32·1 (29·4-34·8)	24·5 (19·3-29·1)	23·4 (18·1-28·3)	19·1 (14·4-23·8)	9·1 (7·9-10·2)	9·3 (7·5-11·3)	9·2 (7·3-11·3)	7·7 (5·5-11·1)	24·2 (13·8-37·4)	33·8 (20·3-49·0)	33·9 (20·1-49·3)	44·4 (17·6-73·9)
Tunisia	0·0 (0·0-0·0)	0·02 (0·01-0·03)	0·01 (0·0-0·02)	0·02 (0·0-0·03)	15·6 (13·2-18·1)	8·9 (6·5-11·6)	8·7 (6·3-11·4)	5·4 (3·7-7·6)	4·8 (4·0-5·7)	3·5 (2·6-4·3)	3·4 (2·6-4·3)	2·7 (1·9-3·5)	17·4 (9·4-29·3)	25·8 (13·6-40·8)	26·4 (14·3-42·0)	35·8 (12·3-64·6)
Turkey	4·9 (1·1-8·7)	0·1 (0·02-0·2)	0·08 (0·02-0·1)	0·5 (0·1-0·9)	16·7 (13·5-20·0)	10·1 (7·4-13·3)	9·9 (7·1-13·0)	6·3 (4·0-9·4)	2·5 (1·9-3·2)	1·7 (1·1-2·6)	1·7 (1·1-2·5)	1·4 (0·8-2·2)	16·1 (11·1-22·7)	26·8 (15·6-40·6)	27·4 (16·3-41·4)	39·0 (15·7-66·8)
United Arab Emirates	0·0 (0·0-0·0)	0·0 (0·0-0·0)	0·0 (0·0-0·0)	0·0 (0·0-0·0)	15·4 (12·0-19·4)	11·0 (8·1-14·8)	10·9 (7·9-14·7)	8·8 (6·2-11·9)	12·3 (10·3-14·4)	7·9 (5·9-10·3)	7·8 (5·8-10·2)	5·4 (3·7-7·5)	21·6 (12·9-32·6)	32·3 (19·3-48·4)	32·7 (19·3-49·0)	41·0 (16·7-67·2)
Yemen	0·3 (0·1-0·5)	0·06 (0·03-0·1)	0·04 (0·02-0·07)	0·1 (0·05-0·2)	55·6 (49·6-61·5)	45·6 (39·4-53·8)	44·3 (38·1-52·9)	36·1 (31·8-44·2)	14·1 (12·0-16·1)	16·3 (14·6-18·0)	16·6 (14·8-18·3)	19·5 (17·3-21·2)	15·2 (8·1-25·2)	17·6 (8·8-29·0)	17·9 (8·7-29·3)	18·4 (4·8-39·2)
**South Asia**																

Location	Indicator 3.1.1: Maternal mortality ratio (maternal deaths per 100,000 livebirths) in women aged 10-54 years				Indicator 3.1.2: Proportion of births attended by skilled health personnel (%)				Indicator 3.2.1: Under-5 mortality rate (probability of dying before the age of 5 per 1,000 livebirths)				Indicator 3.2.2: Neonatal mortality rate (probability of dying during the first 28 days of life per 1,000 livebirths)			
	**2000**	**2015**	**2016**	**2030**	**2000**	**2015**	**2016**	**2030**	**2000**	**2015**	**2016**	**2030**	**2000**	**2015**	**2016**	**2030**
Kiribati	187·0 (143·1-239·3)	178·0 (122·3-248·9)	175·8 (119·4-246·1)	157·8 (70·0-303·4)	84·8 (75·7-91·6)	89·9 (82·6-94·6)	90·0 (82·6-94·9)	93·2 (76·7-100·0)	65·5 (51·5-82·1)	50·4 (27·4-87·1)	48·4 (25·3-85·4)	38·7 (8·9-111·6)	24·3 (16·9-32·9)	20·5 (10·7-36·3)	19·8 (10·1-35·7)	17·0 (3·7-51·7)
Marshall Islands	122·2 (85·2-169·2)	118·1 (76·4-176·8)	113·1 (74·1-169·9)	83·2 (31·4-179·3)	91·4 (86·7-95·0)	95·3 (92·0-97·6)	95·6 (92·3-97·9)	98·0 (91·9-100·0)	40·3 (24·5-60·4)	23·3 (12·4-40·1)	22·2 (11·8-37·9)	13·6 (2·2-44·0)	15·7 (8·9-24·8)	11·0 (5·6-19·5)	10·5 (5·5-18·0)	7·2 (1·1-23·5)
Papua New Guinea	455·5 (327·5-611·5)	405·9 (295·2-552·7)	392·1 (282·6-534·0)	294·0 (147·0-527·2)	53·2 (45·9-60·4)	65·3 (49·7-78·7)	66·8 (50·8-80·1)	81·6 (49·9-100·0)	74·1 (52·3-101·8)	52·0 (29·6-86·7)	49·9 (28·3-83·0)	30·6 (10·4-70·1)	27·3 (19·8-35·6)	21·4 (11·7-36·3)	20·6 (11·3-35·3)	14·2 (4·5-33·0)
Samoa	46·0 (31·3-62·5)	34·1 (23·0-47·0)	32·7 (21·7-45·2)	25·6 (10·0-53·9)	91·7 (86·1-95·4)	90·0 (83·2-94·8)	90·4 (83·3-95·3)	95·1 (79·4-100·0)	15·8 (7·6-28·9)	11·0 (5·2-21·0)	10·4 (4·8-20·2)	6·8 (1·8-17·8)	7·3 (3·6-13·0)	4·9 (2·3-9·6)	4·7 (2·1-9·2)	3·2 (0·7-9·6)
Solomon Islands	186·7 (126·1-267·1)	201·3 (137·0-300·6)	195·9 (132·3-293·5)	162·2 (72·3-327·4)	85·4 (78·6-90·9)	94·7 (90·4-97·4)	95·0 (90·9-97·6)	97·9 (93·6-99·8)	35·9 (26·4-47·7)	26·6 (17·5-39·3)	25·5 (16·7-37·9)	16·0 (6·4-34·1)	15·9 (10·6-22·6)	12·9 (8·1-19·7)	12·5 (7·9-19·1)	9·0 (3·5-20·1)
Tonga	182·0 (133·5-243·2)	149·2 (100·0-203·7)	147·6 (98·7-204·3)	136·4 (60·0-252·2)	93·8 (91·1-95·8)	98·6 (97·4-99·3)	98·6 (97·4-99·4)	99·3 (97·7-99·8)	23·6 (13·9-38·1)	19·1 (10·1-32·9)	18·6 (9·8-32·9)	13·6 (4·3-32·0)	11·9 (7·5-17·9)	10·2 (5·3-17·8)	9·9 (5·1-17·6)	7·6 (2·4-18·1)
Vanuatu	151·8 (101·2-216·6)	151·4 (96·8-230·0)	148·8 (96·9-222·6)	136·6 (60·3-264·7)	85·4 (78·5-90·8)	87·2 (78·0-93·6)	87·4 (78·0-93·9)	91·8 (70·8-100·0)	39·6 (30·7-50·5)	32·7 (21·4-49·7)	31·6 (20·7-48·6)	22·5 (7·9-49·9)	17·3 (12·1-23·8)	14·9 (11·6-18·6)	14·5 (11·4-18·0)	11·1 (4·7-19·9)
**North Africa and Middle East**																
Afghanistan	567·5 (439·4-713·6)	455·9 (338·2-606·0)	442·8 (328·3-595·8)	294·2 (157·8-509·7)	20·9 (13·2-29·8)	44·8 (34·3-56·1)	46·3 (35·2-58·6)	66·1 (40·5-86·0)	135·9 (122·9-149·0)	69·1 (55·4-87·3)	66·6 (52·9-85·7)	35·5 (22·4-56·3)	46·8 (42·0-51·9)	26·9 (21·1-34·8)	25·9 (20·2-33·9)	15·2 (9·2-24·2)
Algeria	105·8 (86·4-128·3)	67·7 (52·8-85·6)	63·9 (49·0-81·6)	40·7 (21·4-69·2)	12·2 (8·0-17·7)	24·2 (15·1-36·4)	24·6 (15·4-37·0)	39·1 (17·2-68·5)	33·1 (29·4-36·8)	19·4 (15·3-24·7)	18·2 (14·2-23·9)	11·4 (6·2-19·2)	17·2 (15·3-19·4)	11·6 (9·6-13·9)	10·9 (9·0-13·3)	7·8 (4·6-12·0)
Bahrain	31·8 (24·8-40·6)	21·4 (14·7-30·1)	21·7 (14·8-30·6)	15·5 (5·1-36·9)	93·8 (90·0-96·6)	98·8 (97·9-99·4)	98·7 (97·9-99·3)	99·7 (98·9-100·0)	12·3 (11·3-13·4)	7·1 (5·6-8·7)	6·9 (5·4-8·7)	4·3 (1·9-8·7)	5·0 (4·5-5·6)	2·8 (2·3-3·3)	2·7 (2·2-3·2)	1·7 (0·9-3·1)
Egypt	85·0 (67·5-103·9)	46·0 (34·2-60·6)	45·0 (32·3-61·5)	32·4 (14·0-65·5)	67·0 (64·1-69·8)	93·1 (91·2-94·5)	93·6 (91·6-95·1)	98·8 (97·7-99·5)	44·1 (38·9-49·8)	20·6 (14·9-28·2)	19·3 (13·2-27·8)	8·9 (3·2-20·3)	20·1 (17·3-23·5)	9·8 (6·8-13·7)	9·2 (6·3-13·4)	4·5 (1·7-10·5)
Iran	30·3 (22·2-39·7)	13·9 (10·1-19·0)	13·8 (9·6-18·9)	7·7 (2·8-17·2)	95·6 (92·8-97·5)	98·2 (96·9-99·0)	98·2 (97·0-99·0)	99·1 (97·5-99·8)	40·8 (33·0-50·1)	18·8 (13·2-25·5)	17·8 (12·6-24·5)	9·3 (3·4-19·9)	21·7 (17·6-26·6)	11·4 (8·1-15·5)	10·9 (7·7-15·0)	6·3 (2·3-13·5)
Iraq	93·3 (67·6-126·6)	53·7 (31·0-87·2)	52·3 (30·2-83·9)	32·3 (10·9-76·5)	66·5 (57·7-74·4)	88·2 (82·9-92·1)	88·7 (83·3-92·7)	94·8 (88·2-98·4)	42·2 (39·3-45·3)	29·1 (23·5-36·4)	28·5 (22·5-36·5)	17·2 (10·5-27·2)	20·9 (19·3-22·7)	15·1 (12·3-18·5)	14·6 (12·0-18·3)	9·8 (6·2-15·0)
Jordan	67·5 (46·3-94·6)	35·9 (23·8-54·9)	35·7 (23·5-55·8)	32·6 (10·6-82·7)	98·3 (97·9-98·6)	99·6 (99·4-99·8)	99·6 (99·4-99·7)	99·9 (99·6-100·0)	26·9 (25·0-28·9)	16·9 (14·2-20·5)	17·2 (14·2-21·3)	11·4 (7·5-17·1)	15·8 (14·5-17·1)	10·3 (8·6-12·7)	10·1 (8·3-12·6)	6·9 (4·5-10·4)
Kuwait	10·8 (8·4-13·4)	9·8 (6·6-14·2)	9·8 (5·9-15·1)	9·6 (1·2-34·3)	96·9 (95·1-98·2)	98·8 (98·0-99·3)	98·9 (98·2-99·3)	99·7 (99·0-100·0)	12·8 (11·9-13·7)	9·8 (8·1-11·8)	9·4 (7·2-12·2)	7·4 (3·1-15·2)	6·9 (6·4-7·3)	5·2 (4·3-6·3)	5·1 (3·9-6·5)	4·1 (1·8-8·0)
Lebanon	29·2 (21·3-40·1)	13·5 (9·4-19·1)	13·3 (9·1-18·8)	7·3 (3·0-16·4)	80·1 (70·8-87·5)	93·0 (88·9-96·2)	93·4 (89·4-96·4)	97·9 (94·0-99·8)	18·4 (14·1-23·3)	9·7 (7·3-12·6)	9·5 (7·0-12·5)	5·8 (2·1-13·8)	10·4 (8·0-13·3)	5·1 (3·9-6·6)	4·9 (3·6-6·5)	2·7 (1·1-5·8)
Libya	24·1 (17·4-32·6)	24·3 (16·5-34·6)	24·9 (16·5-35·9)	29·3 (10·8-68·3)	92·6 (87·7-95·9)	96·8 (94·6-98·2)	96·7 (94·5-98·2)	97·5 (92·8-99·6)	26·7 (21·9-32·3)	15·9 (10·9-22·4)	13·9 (9·7-19·7)	6·5 (3·5-11·8)	13·5 (11·2-16·3)	6·9 (4·9-9·5)	6·4 (4·5-8·8)	3·1 (1·7-5·3)
Morocco	178·8 (138·2-218·9)	87·9 (60·4-120·1)	86·9 (60·3-118·0)	70·6 (31·5-134·7)	62·2 (59·7-64·8)	81·1 (71·1-88·4)	81·7 (71·5-89·0)	91·5 (77·2-98·3)	45·5 (40·0-51·5)	22·9 (17·5-29·9)	21·7 (16·4-28·6)	11·0 (6·7-17·2)	23·9 (20·9-27·0)	14·1 (10·7-18·4)	13·4 (10·1-17·7)	8·1 (4·9-12·7)
Palestine	10·6 (8·4-13·1)	16·9 (13·3-21·0)	17·3 (13·5-21·8)	22·0 (8·6-44·0)	97·6 (97·0-98·1)	98·7 (98·1-99·2)	98·8 (98·1-99·2)	99·4 (98·2-99·9)	27·1 (23·3-31·8)	17·9 (13·1-23·7)	16·7 (12·0-22·8)	10·7 (4·6-20·8)	10·7 (9·0-12·7)	9·4 (6·5-12·9)	8·8 (6·1-12·3)	7·1 (2·6-15·2)
Oman	15·8 (11·2-21·7)	9·1 (6·5-12·6)	9·1 (6·4-12·7)	7·7 (3·1-15·8)	98·1 (96·8-98·9)	98·7 (97·8-99·3)	98·9 (98·2-99·4)	99·5 (98·5-99·9)	15·8 (13·5-18·4)	8·7 (7·8-9·6)	8·0 (7·0-9·2)	3·7 (1·8-7·0)	8·2 (7·0-9·6)	4·6 (4·1-5·1)	4·2 (3·7-4·8)	2·1 (1·0-3·9)
Qatar	49·3 (33·5-72·0)	21·4 (12·9-32·1)	22·0 (12·8-35·2)	12·9 (2·6-36·7)	96·5 (94·1-98·1)	99·4 (98·9-99·7)	99·4 (98·9-99·7)	99·8 (99·3-100·0)	16·5 (11·7-22·5)	9·8 (6·6-14·0)	9·4 (6·2-13·8)	5·6 (1·6-14·0)	8·8 (6·2-11·9)	5·0 (3·4-7·2)	4·9 (3·2-7·1)	3·1 (0·9-7·8)
Saudi Arabia	14·4 (11·9-17·5)	9·9 (7·7-12·4)	9·5 (7·3-12·2)	6·1 (3·0-10·8)	94·2 (91·4-96·2)	97·3 (95·3-98·5)	97·4 (95·6-98·6)	98·7 (96·6-99·7)	23·5 (17·0-32·1)	7·6 (5·1-11·3)	6·6 (4·4-9·8)	2·2 (1·1-4·0)	12·2 (8·8-16·9)	3·8 (2·5-5·7)	3·4 (2·3-5·1)	1·1 (0·5-2·0)
Sudan	430·5 (351·3-515·8)	337·9 (269·8-418·1)	327·8 (258·9-406·6)	253·1 (155·8-383·3)	73·0 (64·1-81·3)	78·7 (74·8-82·5)	80·2 (75·7-84·2)	90·0 (79·8-96·6)	94·4 (85·2-104·5)	64·5 (52·2-80·8)	61·6 (48·3-79·8)	41·7 (22·3-73·8)	35·4 (31·3-39·7)	27·8 (22·6-34·3)	26·6 (21·1-33·9)	20·1 (11·0-35·3)
Syria	35·4 (28·9-42·5)	15·0 (11·2-19·5)	14·8 (10·9-19·5)	12·3 (5·5-24·0)	81·5 (77·1-85·4)	93·7 (90·1-96·4)	93·6 (89·9-96·4)	96·5 (89·5-99·6)	18·1 (17·8-18·4)	27·4 (14·6-45·8)	27·0 (13·7-46·9)	22·8 (7·1-47·4)	8·5 (7·8-9·1)	6·9 (5·5-8·5)	6·5 (5·1-7·9)	3·9 (2·4-6·3)
Tunisia	53·2 (40·5-68·9)	31·5 (20·9-44·2)	30·8 (20·1-43·8)	20·4 (8·7-38·6)	92·7 (90·3-94·7)	98·6 (97·8-99·2)	98·7 (97·8-99·2)	99·7 (99·1-100·0)	26·6 (23·8-29·6)	13·3 (10·7-16·5)	12·5 (10·0-15·7)	6·3 (4·2-9·2)	14·8 (13·0-16·8)	7·8 (6·3-9·7)	7·4 (5·9-9·2)	4·1 (2·7-6·0)
Turkey	50·4 (37·7-66·6)	16·0 (11·5-21·9)	15·7 (10·9-22·3)	13·0 (4·6-29·2)	83·6 (81·1-86·0)	95·0 (91·9-97·0)	94·9 (91·9-97·1)	98·2 (94·9-99·7)	40·2 (34·5-46·7)	16·8 (11·8-24·0)	15·7 (11·0-22·1)	6·9 (2·7-15·1)	19·4 (16·6-22·4)	9·5 (6·7-13·5)	8·8 (6·2-12·3)	4·7 (1·9-9·9)
United Arab Emirates	12·2 (8·1-17·2)	10·0 (6·1-15·8)	10·1 (6·1-15·9)	15·2 (4·3-39·2)	98·8 (97·9-99·3)	99·4 (99·0-99·7)	99·4 (99·0-99·7)	99·6 (98·8-99·9)	10·6 (5·7-17·9)	6·1 (3·0-10·7)	5·7 (3·0-10·3)	4·3 (0·5-15·0)	5·1 (2·7-8·6)	2·9 (1·4-5·1)	2·7 (1·4-4·9)	2·0 (0·2-7·2)
Yemen	351·1 (293·8-418·9)	212·5 (161·6-276·0)	213·4 (160·0-278·6)	152·7 (87·1-255·1)	17·9 (14·6-21·8)	46·8 (37·8-55·7)	47·1 (37·2-56·6)	68·1 (42·0-87·0)	88·4 (82·2-95·0)	44·2 (36·7-53·7)	43·6 (36·1-53·8)	23·0 (15·4-34·7)	34·9 (31·5-38·7)	20·3 (16·0-25·9)	19·8 (15·5-25·6)	12·2 (7·3-19·6)
**South Asia**																

Location	Indicator 3.3.1: Age-standardised rate of new HIV infections (per 1,000 population)				Indicator 3.3.2: Age-standardised rate of tuberculosis cases (per 100,000 population)				Indicator 3.3.3: Age-standardised rate of malaria cases (per 1,000 population)				Indicator 3.3.4: Age-standardised rate of hepatitis B incidence (per 100,000 population)			
	2000	2015	2016	2030	2000	2015	2016	2030	2000	2015	2016	2030	2000	2015	2016	2030
Kiribati	0·007<br>(0·005-0·01)	0·005<br>(0·003-0·01)	0·006<br>(0·003-0·01)	0·007<br>(0·003-0·02)	354·6<br>(315·6-397·6)	295·3<br>(261·8-331·9)	292·6<br>(259·3-328·9)	255·7<br>(226·5-289·5)	0·0<br>(0·0-0·0)	0·0<br>(0·0-0·0)	0·0<br>(0·0-0·0)	0·0<br>(0·0-0·0)	2,283·2<br>(1,685·5-3,042·7)	2,169·5<br>(1,592·9-2,880·0)	2,144·1<br>(1,577·0-2,839·1)	1,819·6<br>(1,353·7-2,424·0)
Marshall Islands	0·03<br>(0·001-0·1)	0·09<br>(0·005-0·5)	0·1<br>(0·006-0·6)	0·1<br>(0·006-0·6)	274·7<br>(236·4-320·4)	213·3<br>(184·0-245·4)	208·4<br>(179·8-239·9)	161·1<br>(130·6-186·5)	0·0<br>(0·0-0·0)	0·0<br>(0·0-0·0)	0·0<br>(0·0-0·0)	0·0<br>(0·0-0·0)	1,874·9<br>(1,421·3-2,483·5)	1,522·9<br>(1,162·6-2,007·8)	1,487·9<br>(1,136·7-1,957·3)	1,199·0<br>(915·6-1,571·2)
Papua New Guinea	0·3<br>(0·2-0·6)	0·3<br>(0·1-0·6)	0·3<br>(0·1-0·7)	0·3<br>(0·1-0·7)	195·4<br>(173·0-220·4)	153·8<br>(128·2-172·1)	148·6<br>(122·3-166·5)	107·7<br>(65·3-128·4)	276·9<br>(199·0-351·3)	97·9<br>(74·7-119·6)	92·3<br>(69·3-113·7)	40·5<br>(24·6-55·9)	2,124·3<br>(1,575·2-2,861·6)	1,842·9<br>(1,387·1-2,454·1)	1,814·7<br>(1,369·0-2,402·5)	1,462·5<br>(1,120·5-1,918·0)
Samoa	0·03<br>(0·001-0·2)	0·1<br>(0·005-0·5)	0·1<br>(0·006-0·6)	0·1<br>(0·006-0·6)	44·3<br>(38·1-50·9)	33·1<br>(28·3-38·3)	32·7<br>(28·0-37·8)	25·0<br>(21·2-29·3)	0·0<br>(0·0-0·0)	0·0<br>(0·0-0·0)	0·0<br>(0·0-0·0)	0·0<br>(0·0-0·0)	946·6<br>(726·9-1,226·9)	799·2<br>(618·8-1,027·1)	790·7<br>(613·6-1,015·2)	680·9<br>(535·2-855·9)
Solomon Islands	0·03<br>(0·001-0·2)	0·09<br>(0·005-0·5)	0·1<br>(0·006-0·5)	0·1<br>(0·007-0·6)	125·8<br>(112·7-140·9)	102·7<br>(91·5-115·4)	100·0<br>(89·1-112·5)	68·8<br>(61·0-77·6)	592·9<br>(426·0-740·7)	89·2<br>(86·1-92·0)	79·9<br>(79·9-79·9)	17·2<br>(10·5-27·7)	1,970·4<br>(1,464·2-2,584·9)	1,695·3<br>(1,272·5-2,203·0)	1,660·5<br>(1,247·9-2,158·1)	1,380·9<br>(1,045·8-1,793·6)
Tonga	0·03<br>(0·001-0·2)	0·1<br>(0·005-0·5)	0·1<br>(0·006-0·6)	0·1<br>(0·006-0·6)	29·1<br>(24·8-34·3)	25·1<br>(21·2-29·5)	24·8<br>(21·0-29·3)	21·5<br>(17·9-25·7)	0·0<br>(0·0-0·0)	0·0<br>(0·0-0·0)	0·0<br>(0·0-0·0)	0·0<br>(0·0-0·0)	6,799·2<br>(4,993·0-8,806·0)	5,523·1<br>(4,048·4-7,157·7)	5,464·8<br>(4,003·5-7,084·2)	4,664·0<br>(3,435·3-6,055·0)
Vanuatu	0·04<br>(0·001-0·3)	0·1<br>(0·005-0·5)	0·1<br>(0·006-0·6)	0·1<br>(0·007-0·6)	121·5<br>(107·6-136·5)	91·5<br>(81·0-103·0)	90·4<br>(80·0-101·7)	72·2<br>(64·2-81·1)	244·8<br>(138·2-385·3)	7·0<br>(6·6-7·4)	4·3<br>(4·3-4·3)	0·2<br>(0·2-0·3)	1,859·0<br>(1,379·2-2,422·8)	1,539·5<br>(1,159·6-2,008·1)	1,514·9<br>(1,142·4-1,975·6)	1,296·9<br>(987·2-1,656·6)
**North Africa and Middle East**																
Afghanistan	0·007<br>(0·001-0·02)	0·02<br>(0·001-0·08)	0·02<br>(0·002-0·08)	0·02<br>(0·002-0·08)	313·1<br>(263·4-368·2)	214·7<br>(181·6-251·0)	208·9<br>(176·2-245·4)	140·7<br>(112·9-171·1)	40·5<br>(21·6-61·3)	10·9<br>(3·9-17·3)	11·4<br>(3·6-18·1)	18·1<br>(1·3-32·9)	2,355·9<br>(1,761·3-3,026·1)	1,609·8<br>(1,202·7-2,062·7)	1,585·8<br>(1,185·8-2,026·4)	1,273·2<br>(968·4-1,623·1)
Algeria	0·008<br>(0·006-0·01)	0·01<br>(0·0-0·02)	0·01<br>(0·0-0·02)	0·01<br>(0·0-0·02)	78·3<br>(64·9-93·4)	52·5<br>(43·3-62·8)	51·6<br>(42·5-61·8)	35·2<br>(28·8-43·0)	0·001<br>(0·001-0·002)	0·0<br>(0·0-0·0)	0·0<br>(0·0-0·0)	0·002<br>(0·002-0·002)	1,080·7<br>(851·0-1,377·2)	666·3<br>(527·0-824·7)	646·8<br>(511·3-800·8)	420·1<br>(325·3-529·2)
Bahrain	0·01<br>(0·01-0·02)	0·02<br>(0·01-0·03)	0·02<br>(0·01-0·03)	0·01<br>(0·008-0·01)	39·7<br>(28·5-52·9)	24·2<br>(17·3-33·1)	23·8<br>(16·9-32·6)	18·3<br>(12·4-25·9)	0·0<br>(0·0-0·0)	0·0<br>(0·0-0·0)	0·0<br>(0·0-0·0)	0·0<br>(0·0-0·0)	2,141·4<br>(1,613·0-2,707·1)	939·1<br>(711·5-1,174·0)	921·7<br>(697·9-1,153·5)	687·3<br>(514·5-868·7)
Egypt	0·002<br>(0·002-0·003)	0·006<br>(0·004-0·01)	0·007<br>(0·004-0·01)	0·007<br>(0·004-0·01)	20·8<br>(16·9-25·2)	16·1<br>(12·9-19·8)	15·8<br>(12·6-19·4)	11·3<br>(8·6-14·6)	0·0<br>(0·0-0·0)	0·0<br>(0·0-0·0)	0·0<br>(0·0-0·0)	0·0<br>(0·0-0·0)	1,111·6<br>(885·7-1,379·6)	725·7<br>(571·7-895·6)	699·9<br>(551·2-860·8)	421·9<br>(326·5-524·1)
Iran	0·01<br>(0·006-0·02)	0·02<br>(0·01-0·03)	0·02<br>(0·01-0·03)	0·02<br>(0·01-0·03)	24·6<br>(22·1-27·4)	20·7<br>(18·7-23·1)	20·0<br>(18·0-22·2)	14·3<br>(12·6-16·1)	2·9<br>(0·0-21·1)	0·01<br>(0·01-0·01)	0·01<br>(0·01-0·01)	0·001<br>(0·001-0·002)	1,283·0<br>(1,024·1-1,568·6)	766·1<br>(600·7-940·8)	737·0<br>(577·5-907·9)	428·6<br>(327·7-540·9)
Iraq	0·01<br>(0·001-0·04)	0·02<br>(0·001-0·06)	0·02<br>(0·001-0·06)	0·02<br>(0·002-0·06)	85·5<br>(60·9-115·0)	53·1<br>(37·2-72·7)	51·7<br>(36·1-70·9)	35·6<br>(24·1-50·2)	0·001<br>(0·001-0·001)	0·0<br>(0·0-0·0)	0·0<br>(0·0-0·0)	0·0<br>(0·0-0·0)	1,497·4<br>(1,164·9-1,855·8)	1,127·3<br>(876·9-1,401·2)	1,096·6<br>(852·7-1,366·9)	744·7<br>(583·6-938·0)
Jordan	0·002<br>(0·002-0·002)	0·001<br>(0·001-0·002)	0·001<br>(0·001-0·002)	0·001<br>(0·001-0·002)	13·7<br>(10·3-18·1)	8·0<br>(6·0-10·6)	7·8<br>(5·9-10·4)	5·4<br>(3·9-7·5)	0·0<br>(0·0-0·0)	0·0<br>(0·0-0·0)	0·0<br>(0·0-0·0)	0·0<br>(0·0-0·0)	617·8<br>(487·6-771·9)	462·2<br>(364·3-576·8)	453·8<br>(357·1-565·9)	346·9<br>(272·8-432·8)
Kuwait	0·003<br>(0·002-0·003)	0·002<br>(0·001-0·003)	0·002<br>(0·001-0·002)	0·001<br>(0·001-0·002)	43·5<br>(30·1-58·8)	25·8<br>(17·5-35·4)	24·5<br>(16·6-33·6)	14·3<br>(9·6-19·9)	0·0<br>(0·0-0·0)	0·0<br>(0·0-0·0)	0·0<br>(0·0-0·0)	0·0<br>(0·0-0·0)	741·2<br>(579·3-929·7)	272·8<br>(217·2-335·1)	266·8<br>(212·3-328·3)	187·6<br>(150·6-230·6)
Lebanon	0·01<br>(0·002-0·05)	0·02<br>(0·002-0·06)	0·02<br>(0·002-0·06)	0·02<br>(0·002-0·06)	29·1<br>(24·9-33·5)	14·6<br>(12·6-16·9)	14·3<br>(12·2-16·5)	7·6<br>(6·4-9·1)	0·0<br>(0·0-0·0)	0·0<br>(0·0-0·0)	0·0<br>(0·0-0·0)	0·0<br>(0·0-0·0)	467·5<br>(369·9-590·2)	299·3<br>(238·6-372·9)	295·2<br>(235·7-367·7)	192·7<br>(156·5-233·9)
Libya	0·01<br>(0·001-0·04)	0·02<br>(0·001-0·06)	0·02<br>(0·001-0·06)	0·02<br>(0·002-0·06)	36·9<br>(26·0-49·9)	34·4<br>(23·6-47·6)	34·7<br>(23·8-48·1)	31·3<br>(20·9-44·7)	0·0<br>(0·0-0·0)	0·0<br>(0·0-0·0)	0·0<br>(0·0-0·0)	0·0<br>(0·0-0·0)	523·7<br>(413·6-662·6)	384·3<br>(305·8-483·5)	383·1<br>(304·0-482·3)	258·0<br>(204·3-325·2)
Morocco	0·03<br>(0·004-0·1)	0·02<br>(0·002-0·07)	0·02<br>(0·002-0·07)	0·02<br>(0·002-0·07)	258·0<br>(185·3-347·8)	192·4<br>(135·8-262·6)	188·5<br>(133·0-258·2)	141·2<br>(98·6-196·3)	0·0<br>(0·0-0·0)	0·0<br>(0·0-0·0)	0·0<br>(0·0-0·0)	0·0<br>(0·0-0·0)	632·1<br>(496·5-794·6)	432·0<br>(340·1-537·7)	423·3<br>(333·2-527·3)	302·9<br>(238·7-378·2)
Palestine	0·004<br>(0·003-0·006)	0·006<br>(0·004-0·01)	0·006<br>(0·004-0·01)	0·006<br>(0·004-0·01)	7·3<br>(5·1-10·0)	3·9<br>(2·6-5·4)	3·8<br>(2·5-5·3)	2·6<br>(1·7-3·8)	0·0<br>(0·0-0·0)	0·0<br>(0·0-0·0)	0·0<br>(0·0-0·0)	0·0<br>(0·0-0·0)	312·5<br>(243·8-401·0)	287·2<br>(222·1-369·6)	286·8<br>(221·8-369·3)	267·5<br>(204·6-349·7)
Oman	0·02<br>(0·02-0·03)	0·02<br>(0·007-0·03)	0·03<br>(0·01-0·05)	0·1<br>(0·03-0·4)	14·9<br>(10·5-20·5)	13·6<br>(9·4-18·9)	13·3<br>(9·2-18·5)	9·8<br>(6·7-14·1)	0·003<br>(0·002-0·003)	0·01<br>(0·0-0·03)	0·01<br>(0·0-0·04)	0·0<br>(0·0-0·0)	1,118·3<br>(863·4-1,412·3)	640·8<br>(494·8-811·3)	626·5<br>(484·1-792·8)	364·0<br>(279·0-466·5)
Qatar	0·004<br>(0·003-0·005)	0·002<br>(0·001-0·003)	0·002<br>(0·001-0·003)	0·007<br>(0·003-0·01)	59·3<br>(41·6-82·0)	34·4<br>(23·7-48·6)	34·3<br>(23·6-48·3)	31·5<br>(21·7-44·0)	0·0<br>(0·0-0·0)	0·0<br>(0·0-0·0)	0·0<br>(0·0-0·0)	0·0<br>(0·0-0·0)	396·1<br>(305·6-502·4)	323·2<br>(251·9-406·7)	316·7<br>(247·6-397·4)	231·0<br>(184·5-286·1)
Saudi Arabia	0·01<br>(0·002-0·04)	0·02<br>(0·001-0·06)	0·02<br>(0·001-0·06)	0·01<br>(0·001-0·04)	44·5<br>(40·6-48·7)	43·8<br>(39·9-48·1)	43·3<br>(39·5-47·6)	37·5<br>(33·9-41·5)	0·0<br>(0·0-0·0)	0·0<br>(0·0-0·0)	0·0<br>(0·0-0·0)	0·0<br>(0·0-0·0)	683·4<br>(537·1-868·2)	444·9<br>(351·1-565·7)	435·5<br>(343·6-552·0)	314·6<br>(249·6-391·2)
Sudan	0·2<br>(0·1-0·2)	0·1<br>(0·07-0·2)	0·09<br>(0·05-0·1)	0·1<br>(0·06-0·2)	187·3<br>(164·9-210·2)	133·2<br>(115·1-152·4)	130·9<br>(112·9-150·1)	101·2<br>(85·6-118·4)	61·1<br>(45·3-82·1)	29·5<br>(21·3-38·4)	29·6<br>(21·3-38·8)	31·1<br>(21·5-44·2)	1,243·6<br>(907·1-1,646·4)	801·9<br>(604·4-1,030·4)	771·8<br>(584·2-988·9)	451·4<br>(349·0-569·5)
Syria	0·001<br>(0·001-0·002)	0·004<br>(0·002-0·008)	0·003<br>(0·002-0·006)	0·08<br>(0·001-0·5)	30·1<br>(21·1-41·4)	22·2<br>(14·9-31·4)	22·2<br>(14·9-31·4)	15·6<br>(9·8-23·5)	0·0<br>(0·0-0·001)	0·0<br>(0·0-0·0)	0·0<br>(0·0-0·0)	0·0<br>(0·0-0·0)	523·5<br>(402·8-690·9)	206·3<br>(163·7-266·2)	204·9<br>(162·3-264·4)	123·6<br>(96·0-161·2)
Tunisia	0·008<br>(0·006-0·01)	0·02<br>(0·01-0·04)	0·02<br>(0·01-0·04)	0·02<br>(0·01-0·04)	42·4<br>(30·5-56·2)	30·6<br>(21·7-41·6)	30·2<br>(21·3-41·0)	22·3<br>(15·4-30·9)	0·0<br>(0·0-0·0)	0·0<br>(0·0-0·0)	0·0<br>(0·0-0·0)	0·0<br>(0·0-0·0)	904·2<br>(694·6-1,151·5)	427·9<br>(342·0-524·3)	409·2<br>(327·0-502·6)	208·3<br>(166·1-257·6)
Turkey	0·004<br>(0·002-0·006)	0·009<br>(0·004-0·02)	0·009<br>(0·004-0·02)	0·007<br>(0·004-0·01)	63·0<br>(54·6-72·3)	25·1<br>(21·5-28·9)	25·0<br>(21·4-28·8)	20·1<br>(17·3-23·0)	0·2<br>(0·2-0·2)	0·0<br>(0·0-0·0)	0·0<br>(0·0-0·0)	0·0<br>(0·0-0·0)	1,211·5<br>(982·7-1,499·8)	471·3<br>(382·8-577·1)	461·9<br>(375·0-567·1)	265·1<br>(205·8-349·6)
United Arab Emirates	0·009<br>(0·001-0·03)	0·06<br>(0·001-0·4)	0·06<br>(0·002-0·5)	0·06<br>(0·001-0·4)	20·7<br>(18·1-23·9)	14·6<br>(12·7-16·8)	14·8<br>(12·9-17·0)	11·7<br>(10·2-13·4)	0·0<br>(0·0-0·0)	0·0<br>(0·0-0·0)	0·0<br>(0·0-0·0)	0·0<br>(0·0-0·0)	487·9<br>(369·5-615·7)	429·3<br>(323·5-547·2)	427·9<br>(322·4-543·1)	408·9<br>(304·5-515·6)
Yemen	0·01<br>(0·002-0·04)	0·02<br>(0·002-0·09)	0·02<br>(0·001-0·09)	0·02<br>(0·002-0·09)	94·8<br>(66·4-128·7)	69·5<br>(47·7-96·6)	69·0<br>(47·4-96·0)	52·4<br>(35·2-75·5)	43·0<br>(12·8-211·2)	13·1<br>(7·1-20·5)	12·8<br>(6·9-19·7)	9·2<br>(4·6-12·0)	1,381·4<br>(1,034·2-1,772·9)	755·1<br>(586·2-968·9)	730·8<br>(565·8-938·8)	453·8<br>(351·3-585·6)
**South Asia**																

Location	Indicator 3.3.5:<br>Age-standardised prevalence* of the sum of 15 neglected tropical diseases (NTDs) (%)<br>*Prevalence estimates reported here may exceed 100% as they reflect the sum of prevalent cases of 15 NTDs.*				Indicator 3.4.1:<br>Age-standardised death rate due to cardiovascular disease, cancer, diabetes, and chronic respiratory disease in populations aged 30-70 (per 100,000 population)				Indicator 3.4.2:<br>Age-standardised death rate due to self-harm (per 100,000 population)				Indicator 3.5.2:<br>Risk-weighted prevalence of alcohol consumption, as measured by the summary exposure value (SEV) for alcohol use (%)			
	2000	2015	2016	2030	2000	2015	2016	2030	2000	2015	2016	2030	2000	2015	2016	2030
Kiribati	13·9<br>(11·1-17·0)	14·7<br>(12·3-17·5)	14·6<br>(12·2-17·5)	14·0<br>(11·6-16·8)	1,180·2<br>(1,093·5-1,266·8)	1,112·8<br>(979·1-1,273·7)	1,104·8<br>(971·6-1,261·0)	1,048·7<br>(820·2-1,328·4)	25·0<br>(20·9-28·9)	25·0<br>(19·4-31·9)	24·9<br>(19·3-32·1)	24·6<br>(15·3-39·4)	3·3<br>(2·4-4·2)	3·4<br>(2·2-4·9)	3·5<br>(2·2-5·0)	3·8<br>(2·1-6·4)
Marshall Islands	34·8<br>(29·2-41·8)	36·0<br>(30·6-42·4)	36·0<br>(30·6-42·4)	35·5<br>(30·3-41·8)	1,103·8<br>(1,011·9-1,197·0)	1,135·1<br>(956·1-1,321·8)	1,114·9<br>(942·4-1,297·4)	1,019·2<br>(701·4-1,392·2)	18·8<br>(14·4-22·8)	17·2<br>(12·2-21·5)	16·9<br>(12·0-21·2)	14·6<br>(8·8-21·7)	3·5<br>(1·6-6·4)	4·1<br>(2·0-7·5)	4·2<br>(2·0-7·6)	5·0<br>(2·4-9·2)
Papua New Guinea	96·6<br>(89·0-105·5)	95·1<br>(87·1-103·7)	95·1<br>(87·1-103·7)	95·0<br>(87·1-103·6)	1,264·9<br>(1,052·5-1,481·1)	1,181·7<br>(960·9-1,425·0)	1,164·5<br>(957·1-1,402·8)	1,009·1<br>(703·7-1,365·5)	15·8<br>(10·2-21·3)	15·3<br>(10·1-20·1)	15·2<br>(10·1-20·0)	13·9<br>(8·4-20·5)	13·6<br>(9·5-17·4)	11·4<br>(6·7-15·9)	11·4<br>(6·7-15·9)	11·2<br>(6·2-16·2)
Samoa	7·5<br>(5·7-10·4)	4·5<br>(3·6-5·5)	4·4<br>(3·5-5·3)	2·8<br>(2·1-3·8)	658·3<br>(570·5-751·1)	604·1<br>(505·1-693·0)	595·1<br>(500·2-680·4)	562·0<br>(413·5-745·8)	15·7<br>(11·7-19·2)	13·1<br>(10·1-16·0)	12·9<br>(10·0-15·8)	12·0<br>(8·2-17·1)	6·0<br>(4·3-7·8)	6·5<br>(4·0-9·5)	6·6<br>(3·9-9·6)	6·8<br>(3·6-10·8)
Solomon Islands	59·0<br>(50·4-68·5)	58·7<br>(50·1-68·2)	58·7<br>(50·1-68·2)	58·3<br>(49·8-67·8)	1,151·6<br>(953·2-1,381·4)	1,188·8<br>(963·0-1,481·8)	1,173·0<br>(959·3-1,460·3)	1,061·6<br>(767·8-1,483·0)	18·8<br>(12·8-24·9)	18·0<br>(12·2-23·8)	17·8<br>(12·1-23·4)	16·1<br>(10·3-23·8)	1·7<br>(1·1-2·4)	3·0<br>(1·8-4·5)	3·1<br>(1·8-4·6)	4·3<br>(2·4-7·3)
Tonga	15·1<br>(12·4-18·8)	13·8<br>(11·5-17·0)	13·8<br>(11·5-16·9)	13·5<br>(11·2-16·6)	678·1<br>(599·5-771·5)	617·7<br>(531·6-717·9)	613·4<br>(525·9-713·2)	569·5<br>(413·0-757·8)	8·1<br>(6·6-9·5)	7·8<br>(6·1-9·5)	7·8<br>(6·1-9·5)	7·9<br>(5·1-11·3)	2·6<br>(1·7-3·6)	3·1<br>(1·7-5·2)	3·2<br>(1·7-5·3)	3·2<br>(1·5-6·2)
Vanuatu	4·9<br>(3·7-6·8)	91·8<br>(79·4-106·5)	91·7<br>(79·3-106·4)	88·9<br>(76·4-103·5)	1,132·7<br>(960·0-1,323·6)	1,082·0<br>(893·6-1,354·3)	1,068·9<br>(885·8-1,339·6)	994·0<br>(692·1-1,411·4)	18·5<br>(13·0-24·3)	17·0<br>(12·1-22·0)	16·9<br>(12·1-21·8)	15·5<br>(10·2-22·3)	3·0<br>(1·9-4·2)	1·8<br>(0·8-3·0)	1·8<br>(0·8-3·1)	1·6<br>(0·5-3·5)
**North Africa and Middle East**																
Afghanistan	59·7<br>(49·8-71·9)	58·7<br>(47·8-72·2)	58·5<br>(47·7-72·1)	56·7<br>(46·4-69·5)	1,368·4<br>(1,202·0-1,532·8)	1,189·1<br>(1,082·0-1,308·0)	1,182·1<br>(1,076·1-1,297·9)	1,026·0<br>(832·6-1,234·7)	10·3<br>(8·4-16·1)	10·1<br>(8·4-15·3)	10·1<br>(8·4-15·2)	9·9<br>(6·9-15·2)	0·2<br>(0·1-0·3)	0·6<br>(0·3-1·0)	0·6<br>(0·3-1·1)	1·1<br>(0·5-2·1)
Algeria	10·1<br>(9·1-11·1)	10·1<br>(9·2-11·1)	10·1<br>(9·2-11·1)	9·9<br>(9·0-10·9)	372·8<br>(333·3-416·2)	287·9<br>(259·1-317·3)	291·1<br>(259·4-324·1)	224·3<br>(174·8-284·4)	4·0<br>(3·3-5·2)	3·7<br>(3·0-5·1)	3·7<br>(3·0-5·0)	3·4<br>(2·2-5·6)	1·6<br>(1·0-2·3)	1·8<br>(1·0-2·9)	1·8<br>(0·9-2·9)	1·6<br>(0·8-2·8)
Bahrain	0·2<br>(0·2-0·3)	0·2<br>(0·2-0·3)	0·2<br>(0·2-0·3)	0·2<br>(0·2-0·3)	588·5<br>(528·4-656·9)	333·0<br>(274·4-403·6)	333·5<br>(274·4-407·6)	231·8<br>(134·3-386·0)	9·5<br>(7·9-11·2)	6·9<br>(5·2-9·3)	7·0<br>(5·2-9·5)	5·8<br>(2·6-11·0)	4·9<br>(3·6-6·3)	3·6<br>(1·9-5·5)	3·6<br>(1·9-5·5)	3·4<br>(1·6-5·5)
Egypt	68·6<br>(66·5-70·7)	16·6<br>(14·9-18·5)	15·2<br>(13·6-17·1)	3·2<br>(2·5-3·8)	563·0<br>(531·2-598·6)	576·0<br>(518·3-636·7)	569·4<br>(499·9-641·0)	513·5<br>(369·4-698·4)	3·6<br>(2·6-4·9)	3·8<br>(2·7-5·2)	3·8<br>(2·7-5·3)	3·6<br>(2·1-5·9)	0·9<br>(0·7-1·1)	0·9<br>(0·7-1·1)	0·9<br>(0·7-1·1)	0·7<br>(0·6-1·0)
Iran	2·8<br>(2·5-3·1)	2·8<br>(2·4-3·3)	2·8<br>(2·4-3·3)	2·7<br>(2·3-3·2)	454·1<br>(396·2-514·5)	344·5<br>(285·9-409·3)	346·1<br>(285·8-410·7)	265·3<br>(157·4-428·2)	7·0<br>(5·8-8·2)	6·0<br>(4·9-7·4)	5·9<br>(4·8-7·4)	5·6<br>(3·1-9·3)	0·0<br>(0·0-0·0)	0·0<br>(0·0-0·0)	0·0<br>(0·0-0·0)	0·0<br>(0·0-0·0)
Iraq	3·9<br>(3·2-4·7)	3·7<br>(3·0-4·5)	3·7<br>(3·0-4·5)	3·8<br>(3·0-4·7)	838·2<br>(749·9-946·8)	707·8<br>(562·8-867·8)	699·2<br>(557·1-864·2)	577·0<br>(369·4-832·9)	9·7<br>(7·8-11·5)	9·7<br>(6·9-12·6)	9·7<br>(7·0-12·7)	9·7<br>(5·2-16·4)	2·2<br>(1·3-3·3)	1·8<br>(0·9-3·2)	1·9<br>(1·0-3·4)	2·7<br>(1·2-5·3)
Jordan	22·5<br>(19·5-25·8)	27·8<br>(23·4-32·9)	27·8<br>(23·4-32·9)	27·7<br>(23·3-32·8)	483·5<br>(398·2-579·0)	354·4<br>(275·0-449·3)	351·4<br>(270·4-451·6)	265·6<br>(142·3-450·2)	5·0<br>(3·9-6·4)	3·5<br>(2·6-4·6)	3·5<br>(2·6-4·7)	3·4<br>(1·7-6·2)	0·8<br>(0·5-1·1)	1·1<br>(0·5-2·0)	1·1<br>(0·5-2·0)	1·2<br>(0·5-2·4)
Kuwait	0·0<br>(0·0-0·0)	0·0<br>(0·0-0·0)	0·0<br>(0·0-0·0)	0·0<br>(0·0-0·0)	363·5<br>(316·8-409·5)	253·3<br>(195·5-315·3)	257·9<br>(193·0-336·6)	191·8<br>(83·7-389·8)	3·2<br>(2·4-3·8)	2·7<br>(1·7-3·5)	2·7<br>(1·7-3·8)	2·5<br>(1·0-5·5)	0·0<br>(0·0-0·1)	0·1<br>(0·0-0·2)	0·1<br>(0·0-0·2)	0·8<br>(0·0-7·4)
Lebanon	0·3<br>(0·2-0·4)	0·2<br>(0·2-0·3)	0·2<br>(0·2-0·3)	0·2<br>(0·2-0·3)	382·4<br>(339·8-430·1)	258·6<br>(228·3-292·8)	258·4<br>(227·6-294·8)	202·9<br>(141·8-293·9)	3·1<br>(2·3-4·7)	2·4<br>(1·6-3·6)	2·4<br>(1·6-3·5)	1·9<br>(1·1-3·3)	4·9<br>(3·7-6·2)	4·8<br>(2·9-6·9)	4·8<br>(2·9-7·0)	5·5<br>(2·8-8·8)
Libya	19·3<br>(17·4-21·2)	17·3<br>(14·9-19·6)	17·0<br>(14·7-19·4)	14·3<br>(12·2-16·1)	382·8<br>(345·2-422·0)	375·7<br>(328·0-425·9)	379·3<br>(329·9-432·7)	385·5<br>(305·1-491·7)	4·3<br>(3·4-5·8)	4·6<br>(3·4-6·6)	4·7<br>(3·4-6·8)	5·2<br>(2·9-9·0)	0·1<br>(0·0-0·2)	0·1<br>(0·1-0·3)	0·2<br>(0·1-0·3)	0·2<br>(0·1-0·3)
Morocco	12·7<br>(10·9-14·8)	15·7<br>(13·4-18·2)	15·7<br>(13·4-18·2)	15·6<br>(13·4-18·2)	425·8<br>(350·0-472·3)	374·4<br>(335·5-408·0)	370·9<br>(332·5-404·4)	326·3<br>(281·4-370·5)	6·1<br>(4·7-7·5)	5·5<br>(4·5-6·8)	5·4<br>(4·4-6·7)	4·1<br>(2·9-5·6)	1·4<br>(1·2-1·7)	1·3<br>(1·0-1·5)	1·3<br>(1·0-1·5)	1·1<br>(0·8-1·3)
Palestine	9·9<br>(7·9-12·5)	9·9<br>(7·8-12·7)	9·9<br>(7·8-12·7)	9·9<br>(7·7-12·7)	502·9<br>(478·7-522·5)	541·8<br>(517·0-565·2)	547·8<br>(514·2-576·6)	639·8<br>(533·7-747·6)	2·7<br>(2·4-3·5)	2·7<br>(2·1-3·4)	2·7<br>(2·1-3·6)	3·1<br>(2·1-4·7)	0·3<br>(0·2-0·5)	0·4<br>(0·2-0·6)	0·4<br>(0·2-0·6)	0·3<br>(0·1-0·6)
Oman	1·4<br>(1·2-1·7)	1·3<br>(1·1-1·6)	1·3<br>(1·1-1·6)	1·2<br>(1·0-1·5)	410·7<br>(376·3-449·0)	344·1<br>(312·8-373·6)	339·9<br>(310·5-367·9)	285·9<br>(244·3-328·2)	3·1<br>(2·4-4·1)	3·1<br>(2·4-4·1)	3·1<br>(2·5-4·2)	3·3<br>(1·9-5·7)	1·3<br>(0·8-1·8)	1·8<br>(0·9-2·9)	1·8<br>(0·9-3·0)	1·9<br>(0·9-3·5)
Qatar	0·2<br>(0·2-0·3)	0·2<br>(0·2-0·3)	0·2<br>(0·2-0·3)	0·2<br>(0·2-0·3)	394·0<br>(323·6-481·9)	220·8<br>(163·2-297·2)	224·5<br>(159·3-312·0)	150·6<br>(59·4-310·2)	7·6<br>(5·7-9·7)	4·8<br>(3·4-6·7)	5·0<br>(3·4-7·2)	4·1<br>(1·4-9·9)	2·4<br>(1·5-3·4)	4·2<br>(2·6-5·9)	4·2<br>(2·6-5·9)	4·7<br>(2·8-6·6)
Saudi Arabia	7·5<br>(4·1-13·2)	8·0<br>(7·4-8·7)	8·0<br>(7·4-8·7)	7·8<br>(7·2-8·6)	370·6<br>(347·7-397·0)	285·6<br>(262·1-312·7)	278·8<br>(254·7-305·9)	218·2<br>(178·4-262·3)	2·9<br>(2·3-3·5)	3·1<br>(2·4-3·7)	3·1<br>(2·4-3·7)	3·6<br>(2·3-5·6)	0·3<br>(0·2-0·5)	0·4<br>(0·2-0·6)	0·4<br>(0·2-0·6)	0·5<br>(0·3-0·8)
Sudan	21·3<br>(18·4-25·1)	18·4<br>(15·4-22·2)	18·1<br>(15·2-21·9)	14·9<br>(12·1-18·7)	647·5<br>(579·1-698·3)	504·9<br>(461·2-542·4)	499·9<br>(457·0-538·0)	415·7<br>(367·2-473·7)	5·1<br>(3·5-6·6)	5·2<br>(3·9-6·5)	5·2<br>(4·0-6·5)	5·3<br>(3·8-7·0)	5·7<br>(4·5-7·1)	5·6<br>(3·9-7·5)	5·6<br>(3·8-7·5)	5·6<br>(3·7-7·6)
Syria	29·7<br>(26·3-33·3)	31·3<br>(27·3-35·9)	31·5<br>(27·5-36·1)	33·1<br>(28·8-38·4)	547·4<br>(509·2-582·5)	397·1<br>(356·5-438·4)	393·8<br>(353·6-434·8)	322·1<br>(255·1-401·9)	2·6<br>(2·3-3·1)	2·5<br>(2·1-3·0)	2·5<br>(2·0-3·0)	2·6<br>(1·7-3·8)	1·5<br>(1·1-2·0)	1·3<br>(0·7-1·9)	1·3<br>(0·7-1·9)	1·0<br>(0·4-1·7)
Tunisia	10·6<br>(9·7-11·6)	10·5<br>(9·6-11·5)	10·5<br>(9·6-11·5)	10·4<br>(9·5-11·4)	372·6<br>(331·5-422·9)	318·6<br>(262·8-383·7)	316·9<br>(260·7-380·1)	277·5<br>(189·0-380·0)	3·8<br>(3·0-4·8)	3·8<br>(2·7-5·0)	3·7<br>(2·7-5·0)	3·5<br>(2·1-5·5)	1·6<br>(1·2-2·1)	1·8<br>(1·0-2·6)	1·8<br>(1·0-2·7)	2·0<br>(1·0-3·3)
Turkey	6·7<br>(5·7-7·8)	6·5<br>(5·4-7·7)	6·5<br>(5·4-7·7)	6·2<br>(5·2-7·4)	412·2<br>(386·3-438·9)	290·1<br>(250·0-337·9)	284·7<br>(239·6-339·2)	192·2<br>(112·7-318·4)	4·3<br>(3·5-5·1)	3·4<br>(2·7-4·1)	3·4<br>(2·6-4·2)	3·0<br>(1·7-4·8)	7·7<br>(5·9-9·6)	4·3<br>(2·4-6·8)	4·2<br>(2·3-6·7)	2·7<br>(1·2-5·1)
United Arab Emirates	0·2<br>(0·2-0·3)	0·2<br>(0·2-0·3)	0·2<br>(0·2-0·3)	0·2<br>(0·2-0·3)	464·4<br>(402·4-529·6)	429·5<br>(343·2-538·7)	428·4<br>(338·7-541·6)	432·9<br>(267·0-687·7)	4·2<br>(3·1-5·6)	3·9<br>(2·6-6·0)	3·9<br>(2·6-6·0)	4·1<br>(2·0-7·9)	7·8<br>(6·5-9·3)	7·9<br>(5·9-10·0)	7·9<br>(5·8-9·9)	6·9<br>(4·9-9·0)
Yemen	43·4<br>(38·7-48·7)	33·8<br>(29·0-39·1)	33·2<br>(28·3-38·4)	25·0<br>(20·5-29·9)	766·2<br>(668·9-859·6)	629·1<br>(557·3-715·5)	634·2<br>(557·9-721·6)	552·2<br>(439·8-694·3)	6·2<br>(4·0-8·0)	6·5<br>(4·8-8·1)	6·5<br>(4·9-8·3)	7·0<br>(4·8-9·8)	0·6<br>(0·4-1·0)	0·4<br>(0·2-0·6)	0·4<br>(0·2-0·6)	0·5<br>(0·2-0·9)
**South Asia**																

Location	Indicator 3.6.1: Age-standardised death rate due to road injuries (per 100,000 population)				Indicator 3.7.1: Proportion of women of reproductive age (15-49 years) who have their need for family planning satisfied with modern contraception methods (%)				Indicator 3.7.2: Number of livebirths per 1,000 women aged 10-14 years and women aged 15-19 years				Indicator 3.8.1: Coverage of essential health services, as defined by the UHC index comprised of the coverage of 9 tracer interventions and risk-standardised death rates from 32 causes amenable to personal healthcare (scale of 0 to 100)			
	2000	2015	2016	2030	2000	2015	2016	2030	2000	2015	2016	2030	2000	2015	2016	2030
Kiribati	16·1 (14·2-18·2)	14·6 (12·1-17·3)	14·4 (11·9-17·1)	12·8 (8·9-17·5)	44·1 (35·1-52·0)	56·8 (49·1-64·8)	57·2 (49·3-64·9)	66·6 (51·2-79·2)	19·6 (18·2-20·9)	7·5 (6·9-8·1)	6·6 (6·1-7·2)	2·4 (2·0-3·0)	37·5 (35·3-39·7)	40·5 (37·4-43·1)	41·2 (38·1-43·9)	44·2 (40·6-47·6)
Marshall Islands	18·7 (15·9-22·7)	17·9 (14·3-22·4)	17·4 (14·0-21·9)	14·4 (9·5-20·8)	63·3 (56·0-70·4)	71·7 (64·7-78·3)	72·0 (65·3-78·4)	78·2 (66·1-87·8)	76·5 (72·1-81·1)	41·1 (35·8-46·9)	39·5 (34·2-45·2)	22·5 (16·3-29·1)	37·2 (34·3-40·1)	43·4 (39·9-47·1)	44·0 (40·4-47·8)	50·3 (45·1-55·4)
Papua New Guinea	35·4 (28·0-42·9)	32·9 (26·5-40·3)	32·5 (26·2-39·8)	28·1 (19·8-38·7)	42·3 (35·9-49·6)	52·1 (44·0-60·2)	52·7 (44·4-60·8)	61·3 (45·8-74·9)	34·1 (32·8-35·3)	28·6 (27·2-29·9)	28·5 (27·2-29·9)	26·7 (23·8-29·8)	33·3 (30·3-36·8)	38·3 (34·7-42·1)	38·9 (35·3-42·7)	47·3 (41·6-52·6)
Samoa	13·1 (10·7-16·0)	12·6 (10·0-15·3)	12·3 (9·9-14·9)	11·5 (7·9-16·2)	55·1 (47·6-62·4)	63·0 (54·9-70·2)	63·3 (55·4-70·2)	69·9 (55·2-81·2)	19·0 (17·1-20·6)	13·0 (11·8-14·1)	12·3 (11·1-13·5)	8·0 (6·3-9·9)	44·5 (41·7-47·5)	47·6 (44·5-50·9)	48·0 (45·0-51·4)	53·1 (49·0-57·1)
Solomon Islands	20·7 (16·1-27·6)	21·5 (16·9-28·2)	21·2 (16·6-27·9)	19·6 (13·0-28·7)	48·6 (40·4-56·7)	58·1 (49·8-65·8)	58·6 (50·3-66·3)	66·5 (52·4-79·2)	34·5 (31·2-37·6)	22·3 (19·1-25·6)	21·5 (18·4-24·8)	13·7 (9·7-18·4)	36·8 (33·1-40·2)	39·5 (36·0-43·2)	40·1 (36·6-43·7)	46·4 (42·5-50·6)
Tonga	16·6 (14·0-19·5)	15·0 (12·1-17·8)	14·9 (11·9-17·6)	13·7 (9·2-18·9)	58·6 (50·9-66·0)	70·0 (62·7-76·3)	70·3 (63·1-76·5)	78·2 (64·8-87·8)	12·2 (11·3-13·0)	7·3 (6·8-7·8)	7·0 (6·5-7·6)	4·3 (3·6-5·1)	49·8 (47·1-52·5)	53·5 (50·5-56·6)	53·8 (50·8-57·0)	56·5 (52·9-60·3)
Vanuatu	20·8 (16·9-26·4)	20·1 (16·1-26·1)	20·0 (16·0-25·8)	18·7 (12·8-26·5)	53·8 (46·0-61·6)	64·2 (56·6-70·9)	64·5 (56·7-71·3)	71·7 (57·7-82·6)	32·9 (30·7-35·1)	25·2 (23·6-27·0)	24·8 (23·1-26·6)	19·3 (16·6-22·3)	35·8 (32·8-38·6)	38·4 (34·6-41·5)	38·7 (35·0-41·8)	43·0 (38·4-46·9)
**North Africa and Middle East**																
Afghanistan	65·4 (56·6-75·4)	58·5 (51·7-66·5)	58·3 (51·4-66·1)	54·3 (41·4-71·4)	23·7 (18·2-29·7)	38·5 (36·1-41·1)	39·1 (36·1-42·1)	54·2 (43·8-64·5)	56·7 (44·3-67·5)	34·3 (24·5-44·3)	33·1 (23·2-43·2)	20·4 (9·1-35·5)	18·9 (15·9-23·3)	30·7 (27·5-35·2)	31·5 (28·3-35·8)	43·4 (38·7-49·7)
Algeria	34·1 (29·9-39·1)	25·5 (22·8-28·8)	25·1 (22·3-28·8)	19·6 (14·7-26·2)	71·3 (67·8-74·8)	72·7 (69·0-76·5)	73·4 (69·0-77·7)	76·6 (67·5-84·3)	9·5 (7·9-11·1)	5·1 (3·6-6·8)	4·8 (3·4-6·4)	2·6 (1·3-4·6)	53·9 (51·6-56·0)	63·2 (60·7-65·6)	63·7 (61·1-66·0)	70·8 (68·0-73·5)
Bahrain	24·9 (21·9-28·5)	14·6 (11·7-18·2)	14·6 (11·7-18·5)	10·1 (5·2-18·3)	58·5 (50·4-66·1)	67·7 (60·2-74·7)	68·1 (60·7-75·3)	75·3 (61·4-85·7)	7·7 (7·6-7·7)	6·5 (6·2-6·8)	6·3 (5·8-6·8)	6·7 (6·0-7·7)	57·3 (55·0-59·6)	67·4 (64·5-70·5)	67·8 (64·7-70·9)	73·1 (67·9-77·7)
Egypt	38·9 (34·6-44·1)	34·7 (29·5-40·4)	34·1 (28·2-40·0)	28·2 (19·2-41·0)	77·3 (76·0-78·6)	81·2 (79·5-82·8)	81·6 (79·2-83·8)	85·4 (81·3-89·0)	26·2 (24·7-27·7)	21·9 (20·0-23·7)	21·0 (19·2-23·0)	17·7 (13·6-21·7)	50·7 (49·3-52·0)	60·0 (57·7-62·5)	60·5 (58·2-63·1)	69·2 (66·1-72·6)
Iran	61·7 (53·2-70·9)	35·3 (29·4-42·5)	34·8 (29·0-42·3)	18·0 (10·0-30·8)	73·4 (67·4-78·9)	79·8 (73·9-84·7)	80·1 (74·4-85·0)	86·3 (76·9-92·6)	20·3 (13·7-28·2)	15·2 (6·6-29·4)	14·4 (6·2-28·8)	7·7 (1·2-25·4)	60·0 (57·1-62·9)	66·8 (63·4-70·1)	67·5 (64·0-70·7)	72·8 (69·1-76·2)
Iraq	30·5 (25·8-35·8)	26·9 (20·8-33·6)	26·6 (20·4-33·4)	22·9 (13·1-35·2)	49·3 (42·8-56·7)	55·1 (49·8-60·4)	56·0 (50·1-62·0)	66·2 (50·4-78·5)	31·5 (17·3-44·6)	54·0 (25·2-76·8)	54·4 (24·3-78·8)	77·4 (13·7-172·1)	43·8 (41·2-46·5)	51·4 (48·0-55·1)	52·2 (48·7-56·0)	60·0 (55·2-64·9)
Jordan	21·2 (17·6-25·6)	16·8 (13·3-21·1)	16·7 (13·0-21·3)	15·2 (8·8-25·2)	50·8 (48·3-53·2)	57·4 (53·2-61·5)	58·0 (52·9-62·9)	66·2 (55·7-74·8)	17·4 (16·0-18·9)	10·7 (9·4-12·1)	10·3 (8·9-11·7)	6·2 (4·6-8·3)	57·1 (53·9-60·1)	65·3 (61·5-68·9)	65·8 (61·9-69·5)	72·0 (66·9-77·0)
Kuwait	26·6 (23·1-30·3)	17·6 (13·6-22·1)	17·6 (12·9-23·5)	13·0 (5·1-28·5)	64·9 (57·6-71·6)	77·5 (70·7-82·8)	77·7 (71·5-82·9)	85·9 (76·0-92·6)	10·6 (10·5-10·7)	3·3 (3·0-3·6)	2·9 (2·6-3·2)	1·0 (0·9-1·2)	64·6 (62·3-67·2)	71·6 (68·0-75·7)	72·6 (68·7-77·0)	82·5 (76·7-88·5)
Lebanon	10·4 (8·9-12·1)	6·6 (5·5-8·0)	6·6 (5·5-8·0)	5·1 (3·3-7·7)	54·1 (48·1-59·9)	61·1 (53·8-68·5)	61·7 (54·4-69·0)	75·2 (59·3-87·3)	10·8 (9·2-12·6)	4·9 (3·8-6·0)	4·6 (3·7-5·7)	1·9 (1·2-3·1)	60·3 (58·2-62·2)	73·6 (70·9-76·0)	74·2 (71·6-76·5)	80·8 (78·0-83·4)
Libya	31·7 (27·4-36·1)	26·5 (22·2-31·3)	26·4 (22·0-31·4)	23·3 (16·8-32·2)	58·3 (50·3-65·5)	73·6 (67·1-79·7)	73·5 (66·7-79·5)	83·6 (73·2-91·4)	3·8 (3·2-4·5)	1·9 (1·5-2·4)	1·8 (1·4-2·4)	0·8 (0·5-1·3)	58·8 (56·6-61·0)	64·5 (62·0-66·8)	64·6 (62·1-67·0)	66·7 (63·5-69·8)
Morocco	32·3 (29·0-36·3)	26·9 (24·1-30·3)	26·6 (23·8-30·0)	22·4 (18·3-27·5)	68·0 (64·3-71·5)	76·9 (71·1-82·6)	77·3 (71·2-82·9)	83·5 (74·3-90·0)	17·3 (16·1-18·5)	10·4 (9·3-11·8)	10·0 (9·0-11·4)	6·2 (4·8-8·0)	48·1 (46·2-49·8)	57·8 (55·0-60·2)	58·3 (55·7-60·7)	67·0 (63·9-70·0)
Palestine	11·8 (10·7-13·0)	16·3 (12·7-19·7)	16·4 (12·8-19·9)	19·6 (13·3-27·5)	56·8 (49·7-63·7)	59·8 (57·6-62·2)	60·9 (57·6-64·1)	70·6 (62·1-78·6)	35·1 (14·5-52·9)	43·8 (24·5-58·8)	42·8 (22·1-58·8)	44·1 (9·0-102·5)	56·2 (54·5-58·3)	58·8 (56·8-60·6)	59·0 (56·9-60·9)	60·9 (58·0-63·4)
Oman	65·0 (58·5-72·0)	50·7 (44·6-56·6)	50·3 (44·2-56·2)	39·4 (30·3-49·5)	44·5 (36·9-52·1)	72·4 (64·9-78·8)	72·8 (65·7-79·2)	89·1 (79·7-94·8)	23·0 (20·6-25·3)	3·8 (3·2-4·5)	3·2 (2·7-3·8)	0·7 (0·5-1·0)	67·6 (65·4-69·8)	74·7 (72·8-76·6)	75·2 (73·2-77·1)	82·4 (80·0-84·6)
Qatar	43·0 (34·0-53·1)	30·9 (22·8-41·6)	31·2 (22·0-43·7)	25·1 (8·8-60·4)	60·0 (52·6-67·1)	71·9 (65·5-77·6)	72·2 (65·7-77·8)	80·4 (70·1-88·8)	9·9 (9·3-10·4)	4·7 (4·1-5·3)	4·6 (4·0-5·3)	2·7 (1·7-4·1)	64·3 (61·1-67·4)	76·6 (72·2-80·9)	77·1 (72·4-81·6)	83·0 (75·8-89·4)
Saudi Arabia	47·8 (43·7-52·3)	38·0 (33·9-41·6)	36·9 (32·8-40·6)	29·1 (22·7-37·0)	54·8 (47·3-62·6)	78·2 (72·5-83·4)	78·7 (73·2-83·8)	90·8 (84·4-95·0)	14·8 (13·0-16·4)	3·0 (2·4-3·7)	2·6 (2·1-3·4)	0·5 (0·3-0·7)	61·3 (59·9-62·7)	71·1 (69·5-72·8)	72·1 (70·4-73·8)	81·6 (79·4-83·7)
Sudan	51·6 (43·9-60·4)	43·7 (38·1-51·0)	43·0 (37·6-50·2)	37·3 (29·6-47·5)	13·5 (10·1-17·6)	26·7 (24·2-29·0)	27·5 (24·5-30·5)	42·3 (32·2-53·1)	44·4 (36·7-51·5)	22·5 (16·3-30·5)	21·3 (15·4-28·9)	9·6 (4·6-17·4)	37·6 (35·7-39·8)	46·2 (44·0-48·3)	47·1 (44·9-49·2)	56·2 (53·0-59·0)
Syria	17·0 (15·8-18·4)	13·2 (11·9-14·6)	13·0 (11·6-14·5)	10·5 (8·1-13·3)	53·5 (50·4-56·5)	69·9 (63·1-75·8)	70·2 (63·6-76·1)	80·2 (70·1-88·2)	28·6 (24·2-33·2)	14·3 (11·2-18·0)	13·9 (11·1-17·6)	7·6 (4·6-11·8)	58·6 (57·3-60·0)	67·2 (65·1-69·4)	67·4 (65·4-69·7)	73·1 (69·2-76·6)
Tunisia	29·9 (25·8-35·0)	24·4 (19·9-30·1)	24·1 (19·7-29·7)	19·6 (13·3-27·7)	74·3 (68·9-79·1)	70·3 (65·5-74·9)	71·1 (65·9-76·3)	81·2 (66·6-92·6)	3·6 (3·4-3·9)	2·7 (2·4-3·2)	2·6 (2·3-3·0)	1·7 (1·0-2·6)	58·2 (55·5-60·5)	65·6 (62·6-68·7)	66·1 (63·0-69·1)	72·2 (69·2-75·3)
Turkey	15·2 (13·7-17·0)	10·9 (9·2-12·7)	10·9 (9·0-12·8)	9·3 (5·8-14·2)	49·6 (47·1-51·9)	63·9 (56·3-70·6)	64·6 (57·3-71·2)	76·4 (65·2-85·4)	23·5 (21·2-25·9)	12·6 (11·0-14·2)	11·7 (10·1-13·5)	6·5 (4·6-8·9)	50·7 (48·9-52·6)	66·5 (63·6-69·3)	66·9 (63·8-69·8)	75·2 (70·3-79·8)
United Arab Emirates	44·3 (35·5-54·0)	52·6 (35·7-71·3)	52·3 (35·6-71·3)	56·2 (28·5-94·2)	56·6 (48·4-64·7)	68·6 (61·3-75·2)	68·8 (61·4-75·7)	79·8 (68·0-89·0)	18·4 (17·6-19·3)	24·4 (22·8-26·0)	24·5 (22·8-26·2)	20·5 (16·6-25·1)	59·8 (57·3-62·1)	65·6 (61·9-69·2)	65·9 (62·1-69·6)	68·8 (63·9-73·9)
Yemen	58·4 (50·8-68·0)	47·8 (41·3-57·3)	47·6 (41·2-57·7)	40·6 (31·3-54·9)	21·0 (18·1-24·0)	43·6 (40·0-47·0)	44·3 (39·5-48·8)	67·3 (54·8-77·7)	44·3 (23·9-58·8)	32·4 (14·5-51·5)	31·4 (12·2-52·1)	20·1 (2·9-58·8)	34·3 (31·9-36·9)	43·6 (40·8-46·7)	44·2 (41·3-47·1)	52·8 (48·7-56·6)
**South Asia**																

Location	Indicator 3.9.1: Age-standardised death rate attributable to household air pollution and ambient air pollution (per 100,000 population)				Indicator 3.9.2: Age-standardised death rate attributable to unsafe water, sanitation, and hygiene (WaSH) (per 100,000 population)				Indicator 3.9.3: Age-standardised death rate due to unintentional poisonings (per 100,000 population)				Indicator 3.a.1: Age-standardised prevalence of daily smoking in populations aged 10 and older (%)			
	**2000**	**2015**	**2016**	**2030**	**2000**	**2015**	**2016**	**2030**	**2000**	**2015**	**2016**	**2030**	**2000**	**2015**	**2016**	**2030**
Kiribati	210·4 (179·1-245·0)	165·8 (134·4-199·2)	163·3 (132·0-196·7)	132·5 (101·5-165·9)	107·1 (62·3-161·3)	67·6 (33·3-116·3)	66·0 (32·1-114·5)	47·1 (19·7-92·7)	3·1 (1·6-3·8)	3·3 (1·2-4·9)	3·3 (1·2-4·9)	3·1 (0·8-6·2)	36·9 (34·4-39·6)	30·5 (27·9-33·2)	30·1 (27·5-32·7)	24·5 (18·8-30·8)
Marshall Islands	116·1 (66·5-164·6)	78·5 (34·8-126·5)	75·8 (32·6-123·1)	45·9 (13·4-82·3)	25·5 (17·5-39·5)	8·2 (4·6-14·9)	7·7 (4·4-14·2)	3·6 (2·0-6·6)	1·9 (1·2-2·6)	1·4 (0·9-2·0)	1·4 (0·9-1·9)	0·9 (0·5-1·5)	15·5 (14·5-16·6)	14·5 (13·0-16·1)	14·4 (13·0-16·0)	13·6 (11·3-16·4)
Papua New Guinea	392·1 (323·5-461·7)	327·9 (266·8-393·5)	322·4 (262·4-387·2)	267·1 (215·8-327·0)	142·6 (88·0-234·5)	86·5 (52·4-141·6)	81·9 (49·6-134·4)	55·0 (29·1-101·6)	3·3 (1·5-4·7)	2·9 (1·4-4·1)	2·8 (1·4-4·1)	2·1 (1·0-3·7)	30·1 (28·0-32·6)	26·9 (24·4-29·5)	26·6 (24·1-29·1)	26·1 (21·7-30·8)
Samoa	112·3 (90·0-135·9)	73·6 (53·5-95·8)	71·3 (51·5-93·7)	49·0 (31·5-68·7)	8·5 (4·6-15·3)	5·2 (2·8-9·4)	5·1 (2·7-9·2)	3·6 (1·9-6·5)	1·3 (0·9-2·4)	1·0 (0·7-2·0)	1·0 (0·7-2·0)	0·8 (0·5-1·6)	27·5 (25·8-29·2)	24·6 (22·6-26·6)	24·4 (22·4-26·5)	22·1 (18·7-25·8)
Solomon Islands	280·5 (232·5-332·4)	253·2 (209·4-308·9)	250·0 (206·4-305·3)	208·6 (170·5-258·0)	59·4 (31·5-104·8)	33·6 (18·7-59·1)	31·7 (17·8-55·7)	15·9 (9·4-27·6)	3·0 (1·5-4·7)	2·7 (1·5-3·9)	2·6 (1·4-3·8)	1·9 (1·0-3·0)	23·2 (21·5-25·1)	21·6 (19·5-23·8)	21·5 (19·3-23·7)	20·3 (16·7-24·3)
Tonga	114·3 (89·7-139·5)	57·5 (39·8-78·4)	55·3 (37·9-76·0)	32·7 (19·1-48·8)	11·0 (6·8-17·5)	6·2 (3·6-9·9)	6·0 (3·5-9·7)	3·7 (2·0-6·2)	2·8 (2·3-3·5)	2·2 (1·7-2·8)	2·2 (1·6-2·8)	1·7 (1·0-2·7)	24·5 (22·8-26·2)	21·8 (20·0-23·7)	21·6 (19·8-23·6)	19·2 (16·2-22·5)
Vanuatu	297·1 (249·4-352·5)	236·0 (190·8-292·6)	232·5 (187·4-288·7)	188·1 (145·2-238·0)	62·9 (35·8-109·3)	33·8 (20·3-58·1)	32·3 (19·4-55·5)	17·3 (10·6-30·0)	3·0 (1·5-4·5)	2·5 (1·4-3·3)	2·4 (1·4-3·3)	1·9 (1·0-3·0)	17·1 (16·0-18·3)	14·5 (13·4-15·6)	14·5 (13·4-15·7)	14·6 (12·1-17·3)
**North Africa and Middle East**																
Afghanistan	527·4 (466·4-585·6)	411·2 (368·6-452·7)	406·1 (364·2-447·1)	339·8 (305·8-374·4)	45·9 (32·6-63·2)	21·6 (15·0-30·2)	20·9 (14·5-29·1)	12·6 (9·2-16·7)	2·1 (1·5-2·7)	1·5 (1·1-1·9)	1·5 (1·1-1·9)	1·1 (0·7-1·8)	10·2 (8·5-12·6)	10·1 (8·7-11·9)	10·1 (8·7-11·9)	10·2 (7·5-13·4)
Algeria	68·9 (59·0-78·4)	48·7 (42·1-56·0)	49·2 (42·6-56·6)	32·5 (20·5-47·3)	4·5 (2·9-6·6)	2·3 (1·3-3·5)	2·2 (1·3-3·4)	1·2 (0·7-2·0)	1·3 (0·7-2·2)	0·7 (0·4-1·2)	0·7 (0·3-1·2)	0·4 (0·2-0·8)	12·1 (10·8-13·7)	10·5 (9·2-12·0)	10·5 (9·2-11·9)	9·1 (7·1-11·4)
Bahrain	117·3 (98·0-137·0)	67·4 (54·2-82·6)	66·4 (53·2-81·7)	44·4 (31·5-58·8)	3·3 (1·8-5·1)	2·0 (1·1-3·2)	2·0 (1·1-3·1)	1·4 (0·7-2·3)	0·6 (0·5-0·8)	0·3 (0·2-0·4)	0·3 (0·2-0·4)	0·1 (0·1-0·3)	10·2 (9·2-11·3)	9·4 (8·0-11·0)	9·4 (8·0-11·1)	7·7 (5·6-10·5)
Egypt	117·3 (104·6-130·1)	123·1 (106·5-142·0)	122·6 (106·1-141·6)	116·5 (100·1-136·2)	17·2 (11·5-23·8)	9·2 (5·6-14·6)	8·6 (5·2-13·9)	3·8 (1·9-7·4)	0·3 (0·2-0·3)	0·2 (0·1-0·3)	0·2 (0·1-0·3)	0·1 (0·0-0·2)	16·2 (15·2-17·3)	17·7 (16·5-18·8)	17·5 (16·3-18·7)	17·6 (15·0-20·5)
Iran	82·9 (70·8-97·6)	64·0 (52·3-75·4)	62·6 (51·2-73·8)	48·9 (39·9-58·4)	4·1 (2·4-6·6)	1·9 (1·0-3·1)	1·8 (1·0-3·0)	0·9 (0·4-1·6)	4·0 (2·7-5·0)	1·6 (1·2-2·4)	1·5 (1·2-2·4)	0·7 (0·3-1·5)	12·4 (11·0-14·0)	11·0 (9·9-12·2)	11·0 (9·8-12·2)	10·7 (8·3-13·6)
Iraq	144·4 (123·8-168·0)	129·9 (105·4-155·8)	125·7 (101·6-151·2)	118·5 (77·6-176·5)	12·7 (7·7-19·9)	7·0 (4·2-11·4)	6·7 (4·0-11·0)	3·5 (1·9-6·3)	1·0 (0·7-1·3)	0·7 (0·5-0·9)	0·6 (0·5-0·9)	0·4 (0·2-0·8)	17·1 (15·6-18·8)	14·9 (13·1-17·0)	14·9 (13·1-17·1)	14·4 (11·2-18·3)
Jordan	66·3 (53·9-80·4)	50·4 (39·2-62·8)	49·6 (38·5-61·9)	39·6 (29·4-51·0)	2·3 (1·5-3·4)	1·3 (0·8-1·9)	1·2 (0·8-1·8)	0·7 (0·4-1·2)	1·4 (0·9-1·8)	0·6 (0·5-0·8)	0·6 (0·5-0·8)	0·3 (0·2-0·6)	22·2 (20·4-24·3)	23·0 (20·9-25·6)	22·8 (20·5-25·5)	22·0 (17·6-27·2)
Kuwait	76·3 (64·6-89·2)	70·2 (54·2-89·9)	68·7 (52·5-88·7)	52·5 (34·5-76·6)	1·1 (0·8-1·5)	1·2 (0·8-1·7)	1·2 (0·7-1·7)	0·9 (0·5-1·5)	0·3 (0·2-0·4)	0·2 (0·1-0·2)	0·2 (0·1-0·3)	0·1 (0·0-0·2)	18·3 (16·9-19·8)	16·6 (15·6-17·5)	16·5 (15·4-17·6)	15·0 (13·0-17·3)
Lebanon	49·5 (40·7-58·7)	37·6 (31·1-45·2)	37·7 (31·2-45·3)	27·3 (17·8-36·5)	3·0 (1·6-6·5)	1·5 (0·8-2·6)	1·4 (0·8-2·5)	0·7 (0·4-1·1)	0·5 (0·3-0·8)	0·2 (0·1-0·4)	0·2 (0·1-0·4)	0·1 (0·1-0·3)	24·8 (21·6-28·2)	26·9 (23·5-31·2)	26·4 (22·6-30·6)	28·9 (21·5-37·8)
Libya	74·5 (65·0-84·9)	72·7 (62·1-83·5)	73·1 (62·4-84·0)	74·8 (61·9-86·8)	3·4 (2·3-4·9)	1·8 (1·1-2·8)	1·8 (1·1-2·7)	0·8 (0·4-1·4)	0·8 (0·4-1·3)	0·5 (0·2-0·9)	0·5 (0·2-0·9)	0·4 (0·1-0·8)	15·5 (14·1-16·9)	14·8 (13·5-16·1)	14·6 (13·3-15·9)	14·7 (12·5-17·4)
Morocco	80·8 (67·7-93·3)	55·5 (46·3-64·7)	55·3 (46·1-64·4)	34·8 (23·4-46·3)	16·7 (11·6-22·3)	6·3 (3·8-9·1)	5·8 (3·5-8·5)	2·3 (1·3-3·6)	1·3 (0·7-1·9)	0·8 (0·4-1·1)	0·7 (0·4-1·1)	0·5 (0·2-0·7)	11·6 (10·8-12·5)	10·2 (9·1-11·5)	10·2 (9·1-11·5)	9·0 (7·2-11·1)
Palestine	90·7 (77·4-103·6)	89·5 (75·2-105·3)	90·1 (75·7-106·1)	99·1 (83·4-116·5)	4·9 (3·4-7·0)	4·4 (2·9-7·2)	4·4 (2·8-7·1)	3·7 (2·1-6·8)	0·6 (0·4-0·8)	0·3 (0·3-0·5)	0·3 (0·2-0·5)	0·2 (0·1-0·4)	20·4 (19·6-21·4)	18·7 (16·9-20·5)	18·7 (16·9-20·7)	17·9 (14·7-21·3)
Oman	119·4 (105·4-134·5)	74·0 (64·8-82·6)	73·6 (64·5-82·2)	50·5 (37·4-63·5)	5·7 (3·7-7·7)	2·0 (1·2-2·8)	1·9 (1·2-2·7)	0·7 (0·4-1·1)	0·6 (0·2-1·4)	0·4 (0·1-0·6)	0·3 (0·1-0·6)	0·2 (0·0-0·4)	7·7 (7·1-8·3)	7·8 (6·9-8·9)	7·8 (6·8-8·9)	8·1 (5·9-10·7)
Qatar	84·7 (69·1-101·3)	52·9 (39·0-70·9)	52·7 (38·4-71·1)	49·0 (31·1-73·9)	0·8 (0·5-1·1)	0·5 (0·3-0·7)	0·5 (0·3-0·7)	0·4 (0·2-0·6)	0·4 (0·3-0·6)	0·3 (0·2-0·4)	0·3 (0·2-0·4)	0·3 (0·1-0·7)	12·8 (11·7-14·0)	13·0 (11·8-14·2)	12·8 (11·5-14·1)	11·5 (8·8-14·3)
Saudi Arabia	140·6 (128·4-153·7)	97·1 (86·6-108·6)	95·4 (85·0-106·7)	74·0 (66·0-82·6)	8·5 (5·5-12·1)	3·7 (2·2-5·4)	3·4 (2·0-5·1)	1·4 (0·7-2·3)	1·2 (0·9-1·6)	0·6 (0·4-0·8)	0·6 (0·4-0·7)	0·3 (0·1-0·5)	10·7 (10·2-11·4)	12·0 (11·5-12·6)	11·9 (11·4-12·4)	13·5 (12·2-14·9)
Sudan	276·0 (248·2-304·2)	189·9 (171·0-209·8)	187·1 (168·4-206·8)	151·3 (134·7-169·0)	58·8 (33·7-92·6)	28·4 (15·2-47·0)	26·7 (14·2-44·2)	14·3 (7·0-26·3)	3·2 (1·2-5·6)	2·0 (0·8-3·5)	1·9 (0·8-3·4)	1·2 (0·4-2·9)	10·3 (9·0-12·0)	10·1 (8·7-12·0)	10·1 (8·7-12·1)	10·2 (7·5-13·9)
Syria	92·1 (79·3-104·8)	77·4 (65·9-88·5)	77·2 (65·8-88·4)	70·9 (60·0-81·9)	1·5 (1·0-2·1)	0·9 (0·6-1·3)	0·8 (0·5-1·2)	0·5 (0·3-0·8)	0·7 (0·6-1·0)	0·5 (0·4-0·6)	0·5 (0·4-0·6)	0·4 (0·2-0·6)	21·9 (20·4-23·5)	18·8 (16·1-22·5)	18·6 (15·9-22·5)	16·3 (11·9-22·0)
Tunisia	70·2 (60·8-80·3)	53·6 (43·5-65·0)	53·4 (43·2-64·8)	49·7 (39·4-61·7)	3·4 (2·0-5·6)	2·5 (1·4-4·5)	2·5 (1·4-4·4)	1·8 (0·9-3·4)	1·0 (0·5-1·5)	0·5 (0·3-0·8)	0·5 (0·3-0·8)	0·3 (0·1-0·5)	22·0 (21·0-23·3)	21·0 (19·2-23·4)	20·8 (19·0-23·1)	19·7 (16·4-24·3)
Turkey	68·6 (59·3-78·4)	43·9 (35·5-52·8)	43·6 (35·1-52·6)	35·2 (26·3-46·3)	6·5 (4·3-8·8)	1·2 (0·8-1·7)	1·1 (0·7-1·6)	0·5 (0·2-0·8)	0·5 (0·3-0·7)	0·2 (0·1-0·2)	0·2 (0·1-0·2)	0·1 (0·1-0·2)	28·9 (26·6-31·1)	20·4 (18·7-22·3)	20·3 (18·2-22·8)	15·3 (12·2-18·5)
United Arab Emirates	100·9 (88·5-114·2)	94·9 (79·4-113·6)	96·8 (80·8-116·0)	110·4 (83·1-144·7)	3·4 (1·5-6·8)	2·4 (1·0-4·9)	2·4 (1·0-4·8)	1·7 (0·7-3·6)	0·7 (0·5-0·9)	0·4 (0·3-0·6)	0·4 (0·3-0·6)	0·4 (0·2-0·6)	11·5 (10·3-12·7)	11·7 (10·2-13·4)	11·6 (10·1-13·3)	11·6 (9·0-14·7)
Yemen	257·3 (227·5-288·6)	179·9 (157·5-204·4)	178·3 (156·0-202·8)	133·1 (105·1-161·1)	68·5 (42·9-100·2)	25·8 (15·8-38·5)	24·1 (14·7-36·0)	9·1 (5·2-14·8)	3·4 (1·4-5·5)	1·9 (0·9-3·1)	1·8 (0·9-3·0)	1·0 (0·5-2·0)	18·1 (15·8-20·7)	16·0 (13·5-19·5)	16·0 (13·5-19·4)	15·9 (11·3-22·6)
**South Asia**																

Location	Indicator 3.b.1: Geometric mean of the coverage of eight vaccines, conditional on inclusion in national vaccine schedules, in target populations (%)				Indicator 5.2.1: Age-standardised prevalence of women aged 15 years and older who experienced physical or sexual violence by an intimate partner in the last 12 months (%)				Indicator 6.1.1: Risk-weighted prevalence of populations using unsafe or unimproved water sources, as measured by the summary exposure value (SEV) for unsafe water (%)				Indicator 6.2.1a: Risk-weighted prevalence of populations using unsafe or unimproved sanitation, as measured by the summary exposure value (SEV) for unsafe sanitation (%)			
	**2000**	**2015**	**2016**	**2030**	**2000**	**2015**	**2016**	**2030**	**2000**	**2015**	**2016**	**2030**	**2000**	**2015**	**2016**	**2030**
Kiribati	84·7 (81·3-87·5)	90·9 (86·8-93·6)	91·0 (86·4-93·8)	93·7 (84·4-98·1)	48·5 (43·8-52·9)	47·9 (43·3-52·3)	47·8 (43·2-52·1)	46·2 (41·7-50·4)	38·6 (26·4-44·5)	33·6 (21·3-40·6)	33·3 (21·0-40·4)	29·4 (17·3-37·0)	71·0 (51·0-86·7)	49·4 (28·6-68·7)	48·2 (27·6-67·6)	31·0 (14·2-46·0)
Marshall Islands	46·2 (40·8-51·5)	57·9 (47·9-66·8)	59·7 (48·2-69·6)	78·9 (55·4-93·9)	41·2 (36·3-45·8)	38·3 (33·6-42·6)	38·2 (33·5-42·4)	35·6 (31·0-39·8)	30·7 (17·7-38·4)	24·6 (12·2-33·5)	24·2 (11·7-33·2)	18·8 (7·2-28·9)	44·9 (25·6-64·4)	25·4 (12·7-42·1)	24·6 (12·2-41·0)	14·4 (7·2-26·4)
Papua New Guinea	61·3 (57·4-65·2)	70·3 (63·9-76·5)	66·4 (57·6-74·0)	74·3 (55·8-87·2)	49·8 (43·9-54·8)	47·6 (42·0-52·3)	47·4 (41·9-52·1)	45·3 (40·1-49·9)	41·1 (27·6-47·3)	38·5 (25·6-44·6)	38·2 (25·4-44·4)	34·7 (21·8-41·1)	81·6 (64·7-94·8)	72·7 (54·1-87·8)	71·9 (53·1-87·3)	59·5 (39·6-77·0)
Samoa	47·8 (43·1-52·6)	47·1 (39·4-54·3)	48·0 (39·4-56·2)	64·9 (39·6-85·0)	39·2 (34·5-44·0)	36·4 (32·2-40·9)	36·3 (32·1-40·7)	34·2 (30·0-38·3)	25·7 (12·9-34·8)	21·5 (8·5-32·5)	21·3 (8·2-32·3)	17·8 (5·3-30·2)	19·5 (9·0-35·6)	16·1 (6·2-31·8)	16·0 (6·1-31·8)	14·3 (4·6-30·0)
Solomon Islands	79·5 (75·8-82·6)	85·3 (80·8-89·0)	86·1 (81·5-90·0)	93·1 (83·9-98·1)	48·2 (42·7-53·4)	45·9 (40·7-50·5)	45·6 (40·5-50·3)	43·2 (38·2-47·7)	35·0 (22·2-41·7)	31·1 (18·7-38·6)	30·7 (18·4-38·4)	25·4 (13·8-34·2)	76·1 (59·1-90·1)	63·6 (43·6-81·5)	62·4 (41·9-80·8)	45·8 (23·5-67·8)
Tonga	97·4 (96·1-98·3)	99·5 (99·1-99·8)	99·6 (99·1-99·8)	99·9 (99·6-100·0)	37·4 (32·9-42·0)	34·4 (30·3-38·5)	34·3 (30·2-38·5)	32·6 (28·7-36·7)	29·7 (17·7-37·7)	24·1 (13·1-33·3)	23·7 (12·8-33·0)	19·2 (9·1-28·8)	41·4 (21·5-61·4)	24·0 (11·4-40·5)	23·3 (11·0-39·4)	12·6 (5·4-23·0)
Vanuatu	64·5 (60·5-68·3)	68·7 (62·0-74·9)	68·3 (60·7-75·2)	69·3 (51·1-84·2)	39·1 (34·4-43·9)	36·1 (31·9-40·5)	36·0 (31·7-40·3)	33·7 (29·7-37·9)	38·6 (28·2-43·8)	34·3 (23·9-40·5)	34·0 (23·5-40·3)	29·9 (19·0-36·9)	72·8 (54·8-92·2)	56·3 (35·8-77·2)	55·2 (34·5-76·2)	39·6 (19·4-56·7)
**North Africa and Middle East**																
Afghanistan	23·5 (21·4-25·8)	66·3 (62·7-69·9)	68·8 (65·0-72·7)	92·5 (88·1-95·6)	74·3 (69·9-78·9)	68·0 (64·3-71·6)	67·7 (64·1-71·4)	64·9 (61·4-68·6)	45·5 (34·8-50·7)	44·4 (34·5-49·5)	44·3 (34·5-49·4)	42·9 (34·1-48·1)	90·2 (80·5-100·0)	80·5 (70·8-94·1)	79·6 (69·7-93·5)	63·6 (51·1-75·4)
Algeria	94·4 (93·1-95·5)	99·3 (99·0-99·5)	99·3 (99·1-99·5)	99·9 (99·8-100·0)	43·6 (38·5-48·4)	37·9 (33·3-42·1)	37·5 (33·0-41·7)	32·3 (28·4-36·1)	23·2 (17·5-27·0)	12·5 (9·1-15·9)	11·9 (8·8-15·3)	6·0 (4·6-8·4)	23·5 (16·2-31·3)	9·0 (4·9-16·8)	8·5 (4·5-16·5)	3·9 (1·4-11·8)
Bahrain	89·5 (84·9-92·9)	96·9 (95·9-97·8)	96·9 (95·7-97·8)	99·4 (98·7-99·8)	45·3 (39·9-50·9)	46·1 (40·6-51·7)	46·0 (40·5-51·5)	45·2 (40·0-50·3)	14·8 (10·9-20·1)	11·1 (7·7-15·3)	10·9 (7·6-15·1)	9·0 (6·3-12·2)	13·9 (8·0-22·2)	9·1 (5·0-16·2)	8·9 (4·9-16·0)	6·5 (3·2-13·0)
Egypt	95·1 (94·3-95·7)	97·3 (96·6-97·8)	97·6 (97·0-98·1)	98·8 (98·0-99·3)	37·7 (33·8-41·6)	39·6 (35·7-43·2)	39·5 (35·5-43·1)	37·8 (33·6-41·8)	20·2 (16·2-22·6)	11·3 (8·6-13·4)	11·0 (8·3-13·0)	6·8 (4·8-8·6)	47·1 (28·6-73·3)	33·2 (18·8-54·6)	32·5 (18·2-53·9)	23·3 (11·9-38·2)
Iran	98·9 (98·4-99·3)	99·9 (99·8-99·9)	99·9 (99·8-99·9)	100·0 (99·9-100·0)	40·0 (35·3-44·8)	37·1 (32·9-41·4)	36·9 (32·7-41·2)	34·0 (29·5-38·8)	12·5 (9·3-15·6)	9·4 (6·8-12·0)	9·3 (6·8-11·9)	7·7 (5·6-10·3)	12·6 (7·0-21·7)	8·2 (4·1-16·2)	8·1 (4·0-15·9)	5·1 (2·1-11·2)
Iraq	73·5 (70·5-76·0)	78·2 (74·5-81·4)	80·4 (76·7-83·7)	86·3 (80·2-91·2)	57·1 (51·8-62·3)	60·5 (56·0-64·8)	60·5 (55·9-64·9)	60·9 (55·0-67·0)	24·4 (18·4-27·4)	27·1 (20·0-31·9)	26·7 (19·7-31·7)	21·8 (15·4-29·2)	31·8 (28·0-35·7)	31·7 (26·7-37·5)	30·4 (25·3-36·2)	15·4 (10·8-20·7)
Jordan	92·4 (87·9-95·4)	99·4 (99·2-99·6)	99·4 (99·2-99·6)	99·9 (99·8-100·0)	43·5 (38·4-48·3)	40·9 (36·5-45·0)	40·8 (36·3-44·9)	38·3 (34·5-41·9)	16·1 (11·0-20·4)	9·8 (6·5-13·9)	9·5 (6·2-13·5)	5·9 (3·8-9·4)	12·3 (7·2-20·6)	4·9 (2·3-9·5)	4·4 (2·1-8·7)	1·7 (0·5-4·1)
Kuwait	86·1 (82·6-88·8)	94·0 (92·5-95·3)	94·9 (93·5-96·0)	98·7 (97·5-99·4)	41·5 (36·2-46·5)	35·7 (31·0-40·1)	35·3 (30·7-39·6)	30·3 (26·5-33·9)	12·0 (8·5-16·2)	7·9 (5·4-10·4)	7·7 (5·3-10·2)	5·5 (3·6-7·5)	11·3 (5·7-21·5)	7·9 (3·2-16·0)	7·8 (3·2-15·9)	5·1 (1·7-12·0)
Lebanon	97·8 (96·4-98·6)	99·6 (99·4-99·7)	99·6 (99·4-99·8)	100·0 (99·9-100·0)	36·8 (32·7-41·2)	33·2 (29·4-37·0)	33·0 (29·2-36·8)	30·4 (27·0-33·8)	22·9 (17·0-27·5)	17·7 (11·5-25·8)	17·0 (11·0-25·3)	9·7 (3·0-22·9)	11·1 (5·3-21·8)	7·7 (3·1-16·0)	7·7 (3·0-15·9)	5·8 (2·2-12·6)
Libya	96·2 (95·2-97·0)	98·5 (97·7-99·0)	98·3 (97·5-98·9)	98·2 (96·2-99·3)	39·4 (34·7-44·2)	34·8 (30·5-38·9)	34·6 (30·4-38·6)	30·6 (27·0-34·2)	13·4 (10·1-16·6)	8·4 (5·8-11·0)	8·3 (5·8-10·9)	7·2 (3·0-14·0)	41·9 (34·5-49·6)	17·8 (12·0-23·4)	17·1 (11·5-22·7)	7·8 (4·6-11·8)
Morocco	91·7 (90·5-92·8)	95·1 (93·8-96·2)	96·0 (94·8-96·9)	98·9 (98·2-99·4)	43·9 (39·0-48·8)	39·7 (35·3-43·9)	39·4 (35·0-43·5)	35·5 (31·6-39·4)	32·8 (24·3-36·8)	20·9 (14·4-27·3)	20·2 (13·8-26·7)	12·2 (7·4-19·2)	34·3 (30·2-38·4)	15·7 (12·7-18·7)	14·9 (11·9-17·8)	6·2 (4·5-7·8)
Palestine	96·7 (96·0-97·2)	96·9 (95·9-97·7)	97·1 (96·1-97·9)	98·3 (96·5-99·3)	48·4 (42·9-53·6)	54·8 (50·0-59·1)	54·7 (50·0-58·9)	53·5 (49·0-57·6)	23·4 (16·9-29·1)	30·8 (22·5-35·4)	30·5 (22·1-35·2)	27·2 (18·4-33·1)	12·4 (10·7-14·4)	17·8 (15·5-20·3)	17·1 (14·9-19·4)	9·4 (8·1-10·5)
Oman	99·8 (99·7-99·8)	99·9 (99·8-99·9)	99·9 (99·8-99·9)	100·0 (99·9-100·0)	49·2 (43·5-54·7)	44·8 (38·7-50·6)	44·1 (38·2-49·7)	40·0 (33·0-47·8)	39·5 (28·2-46·1)	15·6 (9·5-22·5)	14·7 (8·8-21·3)	5·4 (3·0-9·1)	11·2 (5·8-20·7)	7·6 (3·0-15·8)	7·5 (3·0-15·7)	5·6 (2·0-12·1)
Qatar	91·9 (90·1-93·3)	97·9 (97·3-98·4)	98·0 (97·4-98·5)	99·2 (98·6-99·6)	41·5 (36·3-46·7)	44·8 (38·8-50·9)	44·4 (38·5-50·3)	40·9 (35·5-47·5)	12·4 (9·0-17·1)	9·1 (6·4-11·9)	9·0 (6·3-11·7)	7·2 (4·9-8·9)	11·7 (6·3-21·5)	8·1 (3·7-16·7)	8·0 (3·6-16·5)	5·6 (1·9-12·2)
Saudi Arabia	97·7 (97·1-98·2)	99·4 (99·2-99·6)	99·1 (98·7-99·4)	99·8 (99·5-99·9)	42·2 (37·4-46·9)	34·0 (30·0-37·7)	33·4 (29·5-37·0)	26·0 (23·0-29·1)	28·9 (22·7-33·1)	14·8 (11·5-17·4)	13·9 (10·9-16·5)	5·9 (4·9-7·0)	35·7 (30·5-42·7)	8·7 (4·8-14·0)	8·3 (4·5-13·5)	1·3 (0·3-3·6)
Sudan	64·0 (61·5-66·4)	80·4 (77·2-83·2)	81·5 (77·9-84·6)	91·2 (86·3-94·8)	48·4 (42·9-53·5)	47·5 (42·2-52·1)	47·1 (41·9-51·8)	44·8 (40·2-49·0)	40·4 (33·2-45·2)	35·4 (27·8-41·3)	34·8 (27·3-40·8)	26·9 (20·5-33·8)	89·3 (79·3-100·0)	84·8 (74·5-99·3)	84·2 (73·6-99·1)	75·9 (61·4-100·0)
Syria	90·7 (89·0-92·3)	88·5 (85·8-91·0)	88·6 (85·9-91·2)	87·1 (78·6-93·2)	49·9 (44·4-55·2)	43·9 (38·9-48·6)	43·7 (38·8-48·4)	38·6 (34·2-42·8)	23·2 (18·0-26·7)	13·9 (10·6-17·7)	13·4 (10·2-17·3)	8·5 (6·2-12·3)	26·6 (18·6-35·0)	11·6 (6·4-20·3)	11·4 (6·1-20·3)	4·8 (1·6-13·2)
Tunisia	97·8 (97·0-98·4)	99·2 (98·7-99·6)	99·4 (98·9-99·6)	99·9 (99·8-100·0)	38·8 (34·5-42·9)	36·6 (32·6-40·5)	36·3 (32·3-40·2)	32·4 (28·7-36·0)	21·3 (17·5-24·2)	17·8 (13·6-22·0)	17·4 (13·3-21·7)	12·7 (8·8-18·3)	17·1 (9·5-29·4)	15·6 (8·8-26·1)	15·4 (8·7-25·9)	12·9 (6·4-23·6)
Turkey	77·3 (75·2-79·4)	86·7 (83·1-89·6)	86·5 (82·7-89·7)	92·2 (84·8-96·6)	37·8 (33·7-41·9)	34·5 (30·8-38·2)	34·3 (30·6-38·0)	31·8 (28·0-35·8)	26·6 (19·8-30·9)	17·8 (12·4-24·9)	17·0 (11·9-24·3)	9·3 (5·9-17·0)	25·3 (19·0-33·5)	12·2 (7·0-20·9)	11·8 (6·6-20·8)	5·8 (2·5-15·5)
United Arab Emirates	94·0 (92·7-95·1)	98·1 (97·4-98·7)	98·2 (97·4-98·8)	99·3 (98·6-99·7)	45·5 (39·5-51·7)	45·8 (40·1-51·7)	45·3 (39·8-51·0)	41·5 (36·2-47·5)	14·9 (10·6-20·2)	10·7 (7·5-14·6)	10·5 (7·3-14·3)	7·9 (5·3-10·9)	10·0 (4·2-19·5)	7·6 (3·1-15·9)	7·5 (3·2-15·8)	6·0 (2·3-13·6)
Yemen	62·2 (59·0-65·3)	70·6 (65·5-75·1)	71·5 (66·2-76·2)	82·6 (72·9-89·4)	59·6 (55·0-63·9)	55·5 (50·9-59·5)	55·2 (50·7-59·3)	51·1 (46·3-55·5)	43·4 (33·5-48·3)	39·7 (30·1-45·0)	39·2 (29·7-44·6)	32·8 (24·6-39·0)	90·0 (87·6-92·5)	61·0 (56·6-65·1)	58·6 (54·0-62·7)	23·7 (19·1-28·3)
**South Asia**																

Location	Indicator 6.2.1b: Risk-weighted prevalence of populations without access to a handwashing facility, as measured by the summary exposure value (SEV) for unsafe hygiene (%)				Indicator 7.1.2: Risk-weighted prevalence of household air pollution, as measured by the summary exposure value (SEV) for household air pollution (%)				Indicator 8.8.1: Age-standardised all-cause disability-adjusted life year (DALY) rates attributable to occupational risks (per 100,000 population)				Indicator 11.6.2: Population-weighted mean levels of fine particulate matter smaller than 2.5 microns in diameter (PM2.5)			
	**2000**	**2015**	**2016**	**2030**	**2000**	**2015**	**2016**	**2030**	**2000**	**2015**	**2016**	**2030**	**2000**	**2015**	**2016**	**2030**
Kiribati	42·7<br>(29·9-56·5)	33·7<br>(22·0-47·1)	33·1<br>(21·5-46·3)	25·8<br>(16·0-38·5)	59·2<br>(48·0-70·8)	48·7<br>(38·7-60·4)	47·8<br>(37·9-59·5)	36·2<br>(27·7-47·0)	858·0<br>(719·0-1,019·2)	1,029·0<br>(834·4-1,251·1)	1,028·8<br>(833·7-1,250·6)	1,025·9<br>(828·9-1,248·4)	4·4<br>(2·8-6·6)	4·1<br>(2·6-6·0)	4·1<br>(2·5-6·2)	4·0<br>(2·2-6·4)
Marshall Islands	26·6<br>(16·8-39·4)	20·0<br>(12·1-30·6)	19·6<br>(11·8-30·2)	14·8<br>(8·4-23·9)	19·2<br>(12·7-26·8)	11·0<br>(6·5-16·8)	10·3<br>(6·0-16·0)	4·5<br>(2·0-7·7)	1,231·5<br>(973·8-1,490·0)	1,279·0<br>(941·3-1,628·8)	1,273·4<br>(933·9-1,626·6)	1,197·0<br>(837·7-1,600·4)	11·0<br>(2·2-35·3)	10·7<br>(2·0-33·6)	11·1<br>(2·2-32·5)	11·4<br>(2·2-34·4)
Papua New Guinea	51·2<br>(38·4-64·3)	47·2<br>(34·0-60·8)	46·7<br>(33·5-60·4)	42·0<br>(28·5-56·9)	58·2<br>(46·8-70·5)	47·9<br>(36·7-59·0)	47·0<br>(35·9-58·1)	35·3<br>(25·2-45·3)	1,974·7<br>(1,672·0-2,309·2)	1,901·1<br>(1,586·3-2,258·9)	1,886·2<br>(1,573·9-2,241·4)	1,689·3<br>(1,409·7-2,009·3)	15·8<br>(14·6-17·6)	13·7<br>(12·8-15·0)	13·7<br>(12·7-14·9)	13·4<br>(10·0-15·9)
Samoa	16·3<br>(9·9-25·0)	13·1<br>(7·6-20·4)	12·9<br>(7·5-20·2)	10·7<br>(5·9-17·2)	34·5<br>(25·9-43·6)	23·6<br>(16·0-32·6)	22·7<br>(15·2-31·6)	12·2<br>(6·9-19·9)	1,120·3<br>(947·8-1,308·1)	861·7<br>(718·3-1,017·6)	847·7<br>(705·1-1,002·5)	672·5<br>(540·9-815·1)	4·4<br>(3·8-5·1)	3·9<br>(3·4-4·5)	3·9<br>(3·3-4·5)	3·9<br>(3·3-4·5)
Solomon Islands	30·7<br>(19·7-43·3)	25·2<br>(16·0-36·5)	24·6<br>(15·5-35·7)	18·9<br>(11·2-29·0)	58·0<br>(46·8-69·3)	50·1<br>(39·8-62·3)	49·3<br>(39·0-61·3)	37·8<br>(29·2-46·9)	1,026·9<br>(863·2-1,212·5)	1,123·6<br>(914·3-1,356·7)	1,128·2<br>(916·3-1,365·0)	1,195·7<br>(945·3-1,490·9)	7·1<br>(5·4-10·4)	8·2<br>(6·2-12·4)	8·5<br>(6·3-13·0)	8·8<br>(6·1-13·6)
Tonga	25·7<br>(16·3-37·3)	19·1<br>(11·7-28·8)	18·7<br>(11·5-28·2)	13·8<br>(8·1-21·3)	33·8<br>(24·8-43·3)	17·4<br>(10·8-25·3)	16·7<br>(10·2-24·4)	8·7<br>(4·5-14·1)	1,087·5<br>(917·4-1,288·3)	1,000·1<br>(821·7-1,191·9)	996·8<br>(819·0-1,188·6)	951·0<br>(777·8-1,145·5)	4·9<br>(4·0-6·1)	4·0<br>(3·2-5·0)	4·0<br>(3·1-5·0)	4·0<br>(3·1-5·0)
Vanuatu	45·6<br>(31·6-60·6)	37·2<br>(24·8-50·9)	36·6<br>(24·4-50·1)	28·8<br>(18·7-41·5)	52·8<br>(42·9-64·0)	42·0<br>(31·1-52·8)	41·2<br>(30·3-52·0)	30·3<br>(19·9-40·3)	1,904·4<br>(1,604·0-2,218·8)	1,732·1<br>(1,417·1-2,028·9)	1,716·1<br>(1,399·3-2,014·2)	1,505·8<br>(1,181·0-1,813·1)	10·3<br>(6·9-17·8)	8·8<br>(5·8-15·8)	8·8<br>(5·9-15·7)	9·4<br>(5·0-18·1)
**North Africa and Middle East**																
Afghanistan	63·3<br>(61·8-64·9)	61·3<br>(59·8-62·9)	61·1<br>(59·6-62·8)	58·9<br>(57·0-61·4)	72·5<br>(58·8-84·7)	54·4<br>(43·9-65·6)	53·3<br>(43·0-64·5)	37·8<br>(29·8-46·0)	3,088·7<br>(2,634·1-3,624·6)	2,117·4<br>(1,811·6-2,426·3)	2,077·5<br>(1,777·3-2,381·7)	1,580·9<br>(1,345·4-1,827·7)	56·1<br>(54·5-57·9)	62·1<br>(60·1-64·4)	62·9<br>(60·8-65·1)	62·7<br>(55·1-66·9)
Algeria	21·9<br>(20·8-23·1)	16·5<br>(15·7-17·4)	16·1<br>(15·3-17·0)	11·3<br>(10·1-14·3)	2·4<br>(1·3-3·9)	0·2<br>(0·1-0·4)	0·2<br>(0·1-0·3)	0·0<br>(0·0-0·0)	1,061·6<br>(900·1-1,244·3)	827·9<br>(704·0-974·8)	814·7<br>(691·5-962·8)	648·4<br>(526·0-798·3)	32·2<br>(30·6-34·6)	37·0<br>(35·3-39·2)	37·2<br>(35·6-39·5)	33·6<br>(31·4-36·2)
Bahrain	15·5<br>(14·7-16·4)	15·5<br>(14·7-16·4)	15·4<br>(14·6-16·3)	15·6<br>(14·1-16·8)	2·6<br>(1·3-4·5)	0·6<br>(0·3-1·0)	0·6<br>(0·3-1·0)	0·2<br>(0·1-0·3)	1,021·9<br>(863·6-1,191·0)	951·9<br>(773·3-1,147·0)	943·2<br>(763·9-1,138·7)	829·7<br>(642·7-1,036·4)	62·6<br>(50·5-77·9)	79·1<br>(62·8-98·6)	79·7<br>(62·6-100·6)	79·6<br>(62·0-101·6)
Egypt	18·2<br>(17·3-19·1)	13·6<br>(12·9-14·4)	13·5<br>(12·8-14·3)	10·6<br>(9·6-12·3)	1·1<br>(0·6-1·8)	0·1<br>(0·1-0·3)	0·1<br>(0·1-0·2)	0·0<br>(0·0-0·0)	764·6<br>(647·9-896·5)	824·9<br>(689·4-970·1)	811·7<br>(677·4-954·5)	646·9<br>(523·7-782·8)	73·5<br>(68·7-79·2)	124·9<br>(117·3-133·9)	126·0<br>(118·4-135·5)	150·1<br>(128·7-166·3)
Iran	13·2<br>(12·4-13·9)	13·1<br>(12·4-13·9)	13·1<br>(12·4-13·9)	12·2<br>(11·6-13·0)	1·0<br>(0·5-1·7)	0·2<br>(0·1-0·4)	0·2<br>(0·1-0·4)	0·1<br>(0·0-0·1)	777·6<br>(642·0-936·6)	699·9<br>(595·0-810·3)	692·7<br>(588·6-802·6)	597·6<br>(500·8-702·5)	48·0<br>(47·5-48·6)	48·6<br>(48·1-49·1)	49·0<br>(48·5-49·4)	43·1<br>(41·6-46·3)
Iraq	8·2<br>(7·7-8·7)	11·0<br>(10·4-11·7)	10·9<br>(10·2-11·5)	8·9<br>(8·3-9·4)	2·8<br>(1·7-4·4)	1·1<br>(0·6-1·8)	1·0<br>(0·6-1·7)	0·3<br>(0·1-0·6)	1,167·0<br>(994·2-1,368·5)	1,118·9<br>(945·4-1,307·5)	1,105·4<br>(934·4-1,292·9)	932·8<br>(788·5-1,093·1)	50·9<br>(48·1-54·7)	72·3<br>(67·9-77·2)	73·3<br>(69·0-78·8)	76·2<br>(68·8-87·7)
Jordan	15·4<br>(14·6-16·3)	15·1<br>(14·3-16·0)	14·9<br>(14·1-15·8)	13·9<br>(12·6-14·9)	0·1<br>(0·0-0·1)	0·0<br>(0·0-0·0)	0·0<br>(0·0-0·0)	0·0<br>(0·0-0·0)	621·5<br>(512·8-738·8)	497·0<br>(409·3-592·8)	488·7<br>(402·3-583·4)	385·4<br>(312·0-465·3)	29·8<br>(28·1-31·6)	37·3<br>(35·1-39·5)	37·2<br>(35·0-39·6)	35·0<br>(32·6-37·4)
Kuwait	13·4<br>(12·7-14·1)	12·4<br>(11·7-13·1)	12·3<br>(11·6-12·9)	10·5<br>(9·7-12·0)	1·5<br>(0·8-2·5)	0·1<br>(0·1-0·2)	0·1<br>(0·1-0·2)	0·0<br>(0·0-0·0)	1,052·2<br>(865·5-1,251·9)	948·3<br>(766·2-1,138·4)	936·9<br>(754·5-1,127·4)	790·4<br>(602·0-991·4)	66·2<br>(58·3-74·9)	109·1<br>(95·7-125·3)	111·0<br>(96·5-129·3)	117·4<br>(95·2-144·8)
Lebanon	26·4<br>(25·1-27·7)	24·1<br>(22·9-25·3)	23·4<br>(22·2-24·5)	16·2<br>(14·7-19·3)	0·0<br>(0·0-0·0)	0·0<br>(0·0-0·0)	0·0<br>(0·0-0·0)	0·0<br>(0·0-0·0)	744·9<br>(615·3-901·5)	686·2<br>(539·2-857·4)	684·8<br>(533·4-859·7)	649·9<br>(428·3-875·6)	24·6<br>(22·4-27·1)	32·4<br>(29·1-36·4)	32·6<br>(29·0-36·6)	36·6<br>(30·6-42·7)
Libya	14·4<br>(13·6-15·2)	12·4<br>(11·7-13·2)	12·4<br>(11·7-13·1)	11·5<br>(10·8-12·2)	1·1<br>(0·6-2·0)	0·1<br>(0·1-0·2)	0·1<br>(0·1-0·2)	0·0<br>(0·0-0·0)	683·5<br>(562·2-813·8)	615·0<br>(507·8-740·7)	616·3<br>(508·7-743·4)	632·5<br>(521·3-769·6)	54·2<br>(50·6-59·1)	64·4<br>(61·0-69·3)	63·7<br>(60·2-68·1)	63·0<br>(58·9-67·9)
Morocco	33·7<br>(32·4-35·1)	24·6<br>(23·5-25·8)	24·0<br>(22·9-25·2)	16·3<br>(15·4-17·3)	8·0<br>(5·6-11·1)	1·5<br>(0·8-2·5)	1·3<br>(0·7-2·3)	0·3<br>(0·1-0·6)	1,047·6<br>(866·0-1,234·3)	948·6<br>(790·4-1,131·2)	943·1<br>(783·7-1,128·4)	868·4<br>(691·0-1,088·8)	21·7<br>(21·1-22·4)	25·2<br>(24·4-26·1)	25·4<br>(24·6-26·3)	24·3<br>(23·4-25·2)
Palestine	20·9<br>(19·9-22·0)	32·4<br>(31·0-33·9)	32·2<br>(30·8-33·7)	29·9<br>(28·5-31·2)	1·5<br>(0·8-2·5)	0·5<br>(0·2-0·8)	0·4<br>(0·2-0·7)	0·1<br>(0·1-0·2)	860·7<br>(638·6-1,069·9)	922·8<br>(738·1-1,095·0)	932·2<br>(760·9-1,099·5)	1,031·5<br>(799·5-1,278·6)	17·1<br>(15·9-18·3)	19·0<br>(17·7-20·5)	18·9<br>(17·6-20·4)	18·6<br>(16·5-20·6)
Oman	45·3<br>(43·7-47·0)	19·6<br>(18·6-20·8)	18·7<br>(17·7-19·8)	8·0<br>(6·8-9·1)	13·9<br>(7·9-21·6)	0·7<br>(0·4-1·2)	0·6<br>(0·3-1·0)	0·0<br>(0·0-0·1)	1,054·1<br>(894·5-1,233·7)	834·3<br>(703·8-980·6)	831·0<br>(700·7-978·4)	779·6<br>(650·8-926·8)	66·0<br>(60·3-74·0)	76·4<br>(70·4-83·9)	78·0<br>(71·5-85·7)	76·6<br>(68·5-85·7)
Qatar	13·8<br>(13·0-14·5)	13·7<br>(13·0-14·6)	13·7<br>(12·9-14·5)	13·2<br>(12·0-14·1)	0·0<br>(0·0-0·0)	0·0<br>(0·0-0·0)	0·0<br>(0·0-0·0)	0·0<br>(0·0-0·0)	1,124·5<br>(937·1-1,328·4)	1,043·1<br>(864·2-1,267·5)	1,039·4<br>(859·5-1,266·0)	984·2<br>(802·2-1,229·5)	106·4<br>(91·2-123·4)	147·5<br>(129·4-168·4)	148·2<br>(131·3-169·8)	144·7<br>(123·9-174·6)
Saudi Arabia	31·7<br>(31·2-32·3)	20·5<br>(20·1-20·9)	19·5<br>(19·1-19·9)	10·4<br>(9·8-11·4)	9·6<br>(7·4-12·2)	0·3<br>(0·2-0·4)	0·2<br>(0·2-0·3)	0·0<br>(0·0-0·0)	827·9<br>(717·8-946·8)	629·0<br>(525·5-743·4)	620·2<br>(517·6-734·3)	503·3<br>(408·2-612·2)	93·2<br>(91·6-95·1)	182·9<br>(179·5-186·4)	187·9<br>(184·2-191·9)	228·3<br>(196·3-257·6)
Sudan	75·1<br>(73·8-76·3)	70·9<br>(69·5-72·2)	70·2<br>(68·8-71·5)	64·9<br>(61·7-71·0)	57·0<br>(46·1-68·2)	35·6<br>(28·3-43·9)	34·7<br>(27·5-43·0)	22·3<br>(17·0-28·5)	1,154·2<br>(967·8-1,358·4)	854·5<br>(709·6-1,018·3)	841·5<br>(698·8-1,003·7)	676·6<br>(561·8-813·6)	57·2<br>(55·1-59·8)	76·1<br>(73·2-79·9)	77·7<br>(74·7-81·8)	77·7<br>(73·9-81·9)
Syria	17·2<br>(16·2-18·1)	16·0<br>(15·1-16·9)	15·7<br>(14·8-16·6)	12·8<br>(12·0-13·6)	0·6<br>(0·3-1·0)	0·1<br>(0·0-0·1)	0·1<br>(0·0-0·1)	0·0<br>(0·0-0·0)	1,036·8<br>(878·3-1,215·7)	822·6<br>(683·5-978·4)	818·8<br>(679·4-977·4)	764·8<br>(617·5-964·9)	32·4<br>(29·3-36·7)	43·4<br>(39·9-48·4)	43·5<br>(39·7-48·1)	43·0<br>(36·5-53·8)
Tunisia	20·1<br>(19·0-21·2)	20·2<br>(19·2-21·3)	19·8<br>(18·8-20·9)	16·2<br>(15·3-17·0)	3·0<br>(1·9-4·6)	0·2<br>(0·1-0·3)	0·2<br>(0·1-0·3)	0·0<br>(0·0-0·0)	890·3<br>(753·0-1,044·7)	778·5<br>(650·8-920·3)	775·0<br>(646·5-919·8)	725·0<br>(583·2-895·3)	35·6<br>(34·2-37·3)	35·9<br>(34·4-37·5)	35·6<br>(34·1-37·1)	32·0<br>(30·1-34·2)
Turkey	29·6<br>(28·2-30·9)	23·8<br>(22·6-24·9)	23·0<br>(21·8-24·1)	17·3<br>(16·2-18·3)	5·2<br>(2·8-8·4)	0·7<br>(0·4-1·2)	0·6<br>(0·3-1·0)	0·1<br>(0·0-0·1)	1,353·4<br>(1,157·3-1,569·5)	1,022·8<br>(859·2-1,209·0)	1,010·5<br>(846·8-1,197·3)	850·9<br>(685·5-1,049·9)	30·2<br>(29·7-30·8)	37·2<br>(36·4-37·9)	37·3<br>(36·5-38·1)	39·5<br>(34·4-42·8)
United Arab Emirates	16·9<br>(16·0-17·8)	15·3<br>(14·5-16·1)	15·1<br>(14·3-15·9)	12·5<br>(11·9-13·2)	1·4<br>(0·7-2·5)	0·2<br>(0·1-0·4)	0·2<br>(0·1-0·4)	0·1<br>(0·0-0·1)	1,332·9<br>(1,116·9-1,569·6)	1,204·3<br>(1,018·4-1,427·2)	1,204·5<br>(1,017·1-1,428·8)	1,199·1<br>(996·6-1,442·5)	74·2<br>(71·8-77·1)	102·9<br>(98·4-107·7)	105·1<br>(100·8-109·9)	104·7<br>(96·8-112·5)
Yemen	54·0<br>(52·3-55·6)	53·6<br>(52·1-55·2)	53·0<br>(51·4-54·5)	50·1<br>(48·5-51·6)	32·9<br>(25·2-41·8)	17·5<br>(13·5-22·1)	16·6<br>(12·7-21·1)	7·6<br>(5·4-10·5)	1,672·6<br>(1,410·4-1,944·8)	1,116·9<br>(944·3-1,290·0)	1,104·3<br>(931·7-1,277·1)	933·5<br>(765·7-1,109·4)	54·5<br>(49·0-63·3)	71·7<br>(64·9-81·8)	72·6<br>(65·8-84·2)	77·3<br>(67·6-91·0)
**South Asia**																

Location	Indicator 16.1.1: Age-standardised death rate due to interpersonal violence (per 100,000 population)				Indicator 16.1.2: Death rate due to conflict and terrorism (per 100,000 population)				Indicator 16.1.3: Age-standardised prevalence of physical or sexual violence experienced by populations in the last 12 months (%)				Indicator 16.2.3: Age-standardised prevalence of women and men aged 18-29 years who experienced sexual violence by age 18 (%)			
	**2000**	**2015**	**2016**	**2030**	**2000**	**2015**	**2016**	**2030**	**2000**	**2015**	**2016**	**2030**	**2000**	**2015**	**2016**	**2030**
Kiribati	3·5<br>(2·8-5·2)	3·5<br>(2·6-5·3)	3·5<br>(2·6-5·2)	3·4<br>(2·0-5·4)	0·0<br>(0·0-0·0)	0·0<br>(0·0-0·0)	0·0<br>(0·0-0·0)	0·0<br>(0·0-0·0)	13·1<br>(11·5-14·8)	13·2<br>(11·6-14·9)	13·1<br>(11·5-14·8)	12·6<br>(11·0-14·3)	13·8<br>(10·4-17·5)	14·0<br>(10·6-17·8)	14·0<br>(10·6-17·8)	14·1<br>(10·6-17·9)
Marshall Islands	6·4<br>(4·3-8·0)	6·3<br>(4·0-8·4)	6·2<br>(3·9-8·2)	5·7<br>(3·0-9·3)	0·0<br>(0·0-0·0)	0·0<br>(0·0-0·0)	0·0<br>(0·0-0·0)	0·0<br>(0·0-0·0)	9·9<br>(8·6-11·3)	9·2<br>(8·0-10·4)	9·1<br>(8·0-10·4)	8·3<br>(7·2-9·5)	14·1<br>(10·6-17·8)	14·0<br>(10·6-17·8)	14·0<br>(10·6-17·8)	14·0<br>(10·6-17·8)
Papua New Guinea	8·1<br>(5·0-10·8)	8·0<br>(4·9-10·4)	7·9<br>(4·9-10·4)	7·4<br>(4·1-11·6)	0·0<br>(0·0-0·0)	0·0<br>(0·0-0·0)	0·0<br>(0·0-0·0)	0·0<br>(0·0-0·0)	12·5<br>(10·9-14·2)	11·9<br>(10·3-13·5)	11·8<br>(10·3-13·4)	10·7<br>(9·3-12·1)	13·9<br>(11·5-16·5)	13·9<br>(11·5-16·5)	13·9<br>(11·5-16·5)	13·9<br>(11·5-16·4)
Samoa	5·2<br>(3·3-7·2)	4·8<br>(2·9-6·7)	4·7<br>(2·9-6·6)	4·6<br>(2·5-7·4)	0·0<br>(0·0-0·0)	0·0<br>(0·0-0·0)	0·0<br>(0·0-0·0)	0·0<br>(0·0-0·0)	9·3<br>(8·2-10·6)	8·8<br>(7·7-10·1)	8·8<br>(7·7-10·0)	8·3<br>(7·3-9·5)	14·2<br>(10·7-18·0)	14·1<br>(10·7-17·9)	14·1<br>(10·7-17·9)	14·0<br>(10·7-17·8)
Solomon Islands	6·4<br>(3·8-8·5)	6·8<br>(3·9-9·1)	6·7<br>(3·9-9·0)	6·4<br>(3·3-10·6)	0·0<br>(0·0-0·0)	0·0<br>(0·0-0·0)	0·0<br>(0·0-0·0)	0·0<br>(0·0-0·0)	11·9<br>(10·4-13·5)	11·4<br>(10·0-13·0)	11·3<br>(9·9-12·9)	10·3<br>(9·0-11·7)	15·0<br>(11·8-18·9)	14·9<br>(11·8-18·7)	14·9<br>(11·8-18·7)	14·8<br>(11·7-18·5)
Tonga	4·0<br>(2·7-8·6)	3·2<br>(1·9-8·0)	3·2<br>(1·9-8·0)	3·4<br>(1·6-8·2)	0·0<br>(0·0-0·0)	0·0<br>(0·0-0·0)	0·0<br>(0·0-0·0)	0·0<br>(0·0-0·0)	9·2<br>(8·1-10·5)	8·5<br>(7·5-9·7)	8·5<br>(7·4-9·6)	7·8<br>(6·9-8·9)	13·4<br>(10·1-17·1)	13·3<br>(10·1-17·1)	13·3<br>(10·1-17·1)	13·4<br>(10·2-17·1)
Vanuatu	1·5<br>(1·1-1·9)	1·5<br>(1·0-1·9)	1·5<br>(1·0-1·9)	1·4<br>(0·8-2·3)	0·0<br>(0·0-0·0)	0·0<br>(0·0-0·0)	0·0<br>(0·0-0·0)	0·0<br>(0·0-0·0)	9·0<br>(8·0-10·2)	8·5<br>(7·5-9·6)	8·4<br>(7·4-9·5)	7·8<br>(6·9-8·9)	13·9<br>(10·5-17·6)	13·8<br>(10·4-17·5)	13·8<br>(10·4-17·5)	13·8<br>(10·4-17·5)
**North Africa and Middle East**																
Afghanistan	22·4<br>(11·9-28·4)	21·3<br>(10·8-26·8)	21·2<br>(10·9-26·5)	20·1<br>(9·9-30·6)	28·6<br>(21·5-35·8)	55·8<br>(45·2-66·5)	67·7<br>(14·5-121·1)	189·1<br>(18·0-674·7)	28·8<br>(25·9-32·0)	23·9<br>(21·5-26·7)	23·7<br>(21·3-26·4)	20·7<br>(18·7-23·2)	7·2<br>(5·5-9·2)	7·2<br>(5·5-9·2)	7·2<br>(5·5-9·2)	7·2<br>(5·5-9·2)
Algeria	1·5<br>(0·9-1·9)	1·1<br>(0·7-1·4)	1·1<br>(0·7-1·4)	1·0<br>(0·5-1·6)	3·6<br>(3·1-4·1)	0·2<br>(0·2-0·2)	0·1<br>(0·03-0·2)	0·0<br>(0·0-0·03)	11·1<br>(9·7-12·6)	9·1<br>(7·9-10·3)	9·0<br>(7·9-10·2)	7·7<br>(6·7-8·7)	7·2<br>(5·5-9·2)	7·2<br>(5·5-9·3)	7·3<br>(5·5-9·3)	7·3<br>(5·5-9·4)
Bahrain	2·4<br>(1·8-2·9)	1·9<br>(1·3-2·4)	1·9<br>(1·3-2·5)	1·7<br>(0·8-3·5)	0·0<br>(0·0-0·0)	0·0<br>(0·0-0·0)	0·0<br>(0·0-0·0)	0·0<br>(0·0-0·0)	10·1<br>(8·9-11·4)	8·7<br>(7·6-9·8)	8·7<br>(7·6-9·8)	8·2<br>(7·2-9·2)	7·2<br>(5·5-9·2)	7·2<br>(5·4-9·2)	7·2<br>(5·4-9·1)	7·1<br>(5·4-9·0)
Egypt	0·5<br>(0·4-0·7)	0·7<br>(0·4-0·9)	0·7<br>(0·4-0·9)	0·6<br>(0·3-1·2)	0·1<br>(0·03-0·2)	3·1<br>(0·7-5·4)	1·2<br>(0·3-2·1)	1·2<br>(0·3-2·1)	9·6<br>(8·7-10·6)	9·5<br>(8·6-10·6)	9·5<br>(8·5-10·5)	8·7<br>(7·7-9·8)	7·2<br>(5·5-9·2)	7·2<br>(5·5-9·3)	7·2<br>(5·5-9·3)	7·3<br>(5·5-9·3)
Iran	2·9<br>(1·7-3·7)	2·1<br>(1·4-2·7)	2·0<br>(1·4-2·6)	1·2<br>(0·6-2·1)	0·4<br>(0·1-0·8)	0·4<br>(0·1-0·8)	0·1<br>(0·03-0·2)	0·1<br>(0·01-1·1)	10·3<br>(9·1-11·7)	8·9<br>(7·8-10·1)	8·9<br>(7·8-10·0)	8·1<br>(7·1-9·2)	7·2<br>(5·5-9·2)	7·3<br>(5·5-9·4)	7·3<br>(5·5-9·4)	7·4<br>(5·5-9·6)
Iraq	8·6<br>(5·9-11·1)	7·8<br>(4·9-10·6)	7·7<br>(4·8-10·5)	5·9<br>(2·8-10·6)	0·8<br>(0·6-0·9)	59·9<br>(15·0-107·3)	64·9<br>(11·5-120·8)	160·3<br>(1·0-1,485·4)	16·0<br>(14·4-18·0)	16·9<br>(15·2-18·9)	16·9<br>(15·1-18·9)	15·6<br>(13·6-18·1)	7·2<br>(5·5-9·2)	7·2<br>(5·5-9·2)	7·2<br>(5·5-9·2)	7·2<br>(5·5-9·2)
Jordan	4·3<br>(2·7-5·5)	3·8<br>(2·1-5·0)	3·7<br>(2·0-5·0)	3·5<br>(1·5-6·5)	0·0<br>(0·0-0·0)	0·0<br>(0·0-0·0)	16·5<br>(3·8-29·4)	16·5<br>(3·8-29·4)	10·6<br>(9·5-11·9)	9·2<br>(8·3-10·2)	9·1<br>(8·2-10·2)	8·7<br>(7·7-9·9)	7·2<br>(5·5-9·2)	7·2<br>(5·5-9·2)	7·2<br>(5·5-9·2)	7·2<br>(5·5-9·2)
Kuwait	1·6<br>(0·9-2·2)	1·0<br>(0·6-1·6)	1·0<br>(0·6-1·6)	0·5<br>(0·1-1·3)	0·4<br>(0·08-0·7)	0·7<br>(0·2-1·2)	0·0<br>(0·0-0·0)	0·0<br>(0·0-0·0)	8·8<br>(7·7-10·0)	7·1<br>(6·3-8·1)	7·1<br>(6·2-8·0)	5·9<br>(5·2-6·7)	7·3<br>(5·5-9·3)	7·3<br>(5·5-9·4)	7·3<br>(5·5-9·4)	7·3<br>(5·5-9·4)
Lebanon	4·3<br>(2·3-6·0)	2·9<br>(1·5-4·5)	2·9<br>(1·5-4·5)	2·5<br>(1·1-5·2)	1·5<br>(0·3-2·7)	8·9<br>(2·1-15·4)	9·2<br>(2·1-16·2)	5·2<br>(0·0-37·1)	10·1<br>(9·0-11·5)	8·1<br>(7·2-9·2)	8·1<br>(7·2-9·2)	7·6<br>(6·7-8·6)	7·2<br>(5·5-9·2)	7·2<br>(5·5-9·3)	7·3<br>(5·5-9·3)	7·3<br>(5·5-9·3)
Libya	1·9<br>(1·2-2·4)	1·8<br>(1·1-2·4)	1·8<br>(1·1-2·4)	1·8<br>(0·9-2·9)	3·5<br>(0·8-6·2)	46·6<br>(14·7-80·8)	28·5<br>(7·6-50·6)	28·5<br>(7·6-50·6)	9·6<br>(8·5-10·9)	8·3<br>(7·3-9·4)	8·2<br>(7·3-9·3)	7·8<br>(6·8-8·8)	7·2<br>(5·5-9·2)	7·3<br>(5·5-9·3)	7·3<br>(5·5-9·3)	7·3<br>(5·5-9·4)
Morocco	1·2<br>(0·7-1·4)	1·1<br>(0·6-1·4)	1·1<br>(0·6-1·4)	1·0<br>(0·5-1·6)	0·0<br>(0·0-0·0)	0·0<br>(0·0-0·0)	0·0<br>(0·0-0·0)	0·0<br>(0·0-0·0)	11·7<br>(10·3-13·3)	10·0<br>(8·8-11·4)	9·9<br>(8·8-11·2)	8·7<br>(7·7-9·8)	7·9<br>(6·2-10·0)	8·3<br>(6·4-10·6)	8·4<br>(6·5-10·7)	8·4<br>(6·5-10·8)
Palestine	2·5<br>(2·1-3·2)	3·2<br>(2·0-3·9)	3·2<br>(2·1-3·9)	2·9<br>(1·7-4·5)	18·3<br>(4·9-32·5)	3·2<br>(0·6-5·9)	2·8<br>(0·5-5·4)	1·5<br>(0·0-6·2)	12·7<br>(11·2-14·3)	14·4<br>(12·7-16·4)	14·3<br>(12·7-16·3)	13·1<br>(11·6-14·9)	7·2<br>(5·5-9·2)	7·2<br>(5·5-9·2)	7·2<br>(5·5-9·2)	7·2<br>(5·5-9·2)
Oman	0·6<br>(0·5-0·8)	0·6<br>(0·4-0·7)	0·6<br>(0·4-0·7)	0·5<br>(0·3-0·8)	0·0<br>(0·0-0·0)	0·0<br>(0·0-0·0)	0·0<br>(0·0-0·0)	0·0<br>(0·0-0·0)	10·8<br>(9·5-12·2)	7·4<br>(6·6-8·4)	7·3<br>(6·4-8·3)	5·2<br>(4·6-5·9)	7·2<br>(5·5-9·1)	7·2<br>(5·5-9·2)	7·2<br>(5·5-9·2)	7·3<br>(5·4-9·5)
Qatar	1·0<br>(0·8-1·6)	1·1<br>(0·6-1·5)	1·1<br>(0·6-1·5)	1·1<br>(0·3-2·4)	0·0<br>(0·0-0·0)	0·0<br>(0·0-0·0)	0·0<br>(0·0-0·0)	0·0<br>(0·0-0·0)	7·9<br>(7·0-8·9)	6·8<br>(6·0-7·7)	6·8<br>(6·0-7·6)	6·0<br>(5·3-6·8)	7·2<br>(5·5-9·2)	7·1<br>(5·3-9·1)	7·1<br>(5·4-9·1)	7·1<br>(5·3-9·2)
Saudi Arabia	0·4<br>(0·4-0·6)	0·5<br>(0·4-0·6)	0·5<br>(0·3-0·6)	0·5<br>(0·3-0·7)	0·0<br>(0·0-0·0)	7·1<br>(6·3-8·0)	0·2<br>(0·1-0·3)	0·2<br>(0·1-0·3)	9·2<br>(8·3-10·3)	6·8<br>(6·1-7·6)	6·7<br>(6·0-7·4)	5·2<br>(4·7-5·8)	6·9<br>(5·3-8·8)	6·9<br>(5·3-8·7)	6·9<br>(5·3-8·7)	6·8<br>(5·2-8·7)
Sudan	0·6<br>(0·5-0·8)	1·5<br>(0·4-2·1)	1·5<br>(0·4-2·1)	1·2<br>(0·4-2·2)	5·8<br>(3·9-7·7)	3·5<br>(1·2-6·0)	6·5<br>(1·9-11·4)	72·5<br>(0·2-281·4)	12·4<br>(11·0-14·1)	11·5<br>(10·2-13·0)	11·4<br>(10·1-12·9)	9·3<br>(8·2-10·6)	7·2<br>(5·5-9·2)	7·2<br>(5·5-9·2)	7·2<br>(5·5-9·2)	7·2<br>(5·5-9·2)
Syria	3·1<br>(1·7-3·7)	2·5<br>(1·3-3·2)	2·5<br>(1·3-3·2)	2·3<br>(1·0-3·7)	0·0<br>(0·0-0·0)	295·9<br>(79·6-514·4)	303·3<br>(84·3-531·4)	303·3<br>(83·5-524·2)	13·2<br>(11·6-15·0)	10·7<br>(9·4-12·2)	10·6<br>(9·3-12·1)	9·4<br>(8·2-10·7)	7·2<br>(5·5-9·2)	7·2<br>(5·5-9·2)	7·2<br>(5·5-9·2)	7·2<br>(5·5-9·2)
Tunisia	1·0<br>(0·7-1·2)	0·9<br>(0·6-1·3)	0·9<br>(0·6-1·3)	0·8<br>(0·4-1·4)	0·0<br>(0·0-0·0)	2·5<br>(0·6-4·5)	1·3<br>(0·5-2·2)	1·3<br>(0·5-2·2)	10·2<br>(9·0-11·6)	9·3<br>(8·2-10·6)	9·2<br>(8·1-10·5)	8·1<br>(7·1-9·2)	7·2<br>(5·5-9·2)	7·3<br>(5·5-9·3)	7·3<br>(5·5-9·3)	7·3<br>(5·5-9·4)
Turkey	3·1<br>(2·0-3·9)	2·2<br>(1·3-2·8)	2·2<br>(1·2-2·9)	1·7<br>(0·8-2·9)	0·2<br>(0·2-0·2)	1·3<br>(1·0-1·6)	2·8<br>(0·6-5·1)	358·1<br>(0·2-1,883·3)	9·5<br>(8·5-10·7)	8·0<br>(7·2-9·0)	8·0<br>(7·2-8·9)	7·4<br>(6·5-8·5)	6·7<br>(5·4-8·2)	6·6<br>(5·8-7·6)	6·6<br>(5·8-7·6)	6·5<br>(5·8-7·5)
United Arab Emirates	1·2<br>(0·9-1·6)	1·1<br>(0·8-1·5)	1·1<br>(0·8-1·6)	1·1<br>(0·5-2·0)	0·0<br>(0·0-0·0)	0·0<br>(0·0-0·0)	0·0<br>(0·0-0·0)	0·0<br>(0·0-0·0)	8·1<br>(7·1-9·2)	7·1<br>(6·2-8·0)	7·1<br>(6·2-8·0)	6·6<br>(5·8-7·5)	7·2<br>(5·4-9·1)	7·2<br>(5·4-9·2)	7·2<br>(5·4-9·2)	7·3<br>(5·4-9·4)
Yemen	3·5<br>(2·4-4·3)	3·4<br>(2·1-4·4)	3·5<br>(2·0-4·5)	3·4<br>(1·8-5·4)	0·0<br>(0·0-0·0)	61·3<br>(53·7-69·0)	83·1<br>(68·5-107·2)	5,837·6<br>(231·8-56,532·3)	18·3<br>(16·1-20·8)	14·9<br>(13·1-16·9)	14·7<br>(12·9-16·6)	12·0<br>(10·5-13·6)	7·2<br>(5·5-9·1)	7·2<br>(5·5-9·2)	7·2<br>(5·5-9·2)	7·2<br>(5·5-9·2)
**South Asia**																

Location	Indicator 17.19.2c: Percentage of well-certified deaths by a vital registration (VR) system among a country's total population (%)			
	**2000**	**2015**	**2016**	**2030**
Kiribati	35·4 (28·0-43·1)	46·4 (33·2-60·1)	47·1 (33·8-61·2)	58·4 (31·3-83·6)
Marshall Islands	0·0 (0·0-0·0)	0·0 (0·0-0·0)	0·0 (0·0-0·0)	0·0 (0·0-0·0)
Papua New Guinea	22·2 (13·7-33·3)	23·4 (14·5-34·8)	24·1 (15·0-35·7)	24·3 (9·1-46·1)
Samoa	0·0 (0·0-0·0)	0·0 (0·0-0·0)	0·0 (0·0-0·0)	0·0 (0·0-0·0)
Solomon Islands	0·0 (0·0-0·0)	0·0 (0·0-0·0)	0·0 (0·0-0·0)	0·0 (0·0-0·0)
Tonga	50·0 (38·3-61·6)	56·6 (42·5-70·1)	57·1 (43·1-70·3)	63·1 (38·3-84·6)
Vanuatu	0·0 (0·0-0·0)	0·0 (0·0-0·0)	0·0 (0·0-0·0)	0·0 (0·0-0·0)
**North Africa and Middle East**				
Afghanistan	0·0 (0·0-0·0)	0·0 (0·0-0·0)	0·0 (0·0-0·0)	0·0 (0·0-0·0)
Algeria	14·3 (6·2-25·9)	29·8 (14·3-48·8)	30·6 (14·9-49·9)	50·8 (16·4-86·0)
Bahrain	53·6 (47·3-60·0)	56·3 (46·4-66·1)	57·5 (45·3-69·1)	66·4 (41·0-88·7)
Egypt	41·2 (34·6-47·6)	48·7 (40·2-57·7)	49·7 (38·8-60·7)	54·0 (34·5-71·4)
Iran	37·0 (27·2-47·0)	64·0 (51·9-74·8)	64·2 (51·4-76·3)	76·4 (50·0-91·8)
Iraq	31·6 (15·9-49·5)	31·5 (15·8-50·5)	32·8 (16·3-52·8)	44·9 (12·3-86·2)
Jordan	64·3 (49·6-77·6)	66·6 (53·3-78·3)	67·2 (52·7-80·0)	78·3 (46·6-98·1)
Kuwait	78·2 (74·3-82·0)	82·2 (75·3-87·9)	82·6 (74·8-89·1)	86·6 (72·7-95·3)
Lebanon	0·0 (0·0-0·0)	0·0 (0·0-0·0)	0·0 (0·0-0·0)	0·0 (0·0-0·0)
Libya	4·9 (1·9-9·7)	10·2 (4·1-19·6)	10·1 (4·1-19·9)	16·3 (3·1-42·1)
Morocco	25·1 (14·5-39·8)	17·2 (9·2-28·0)	17·9 (9·0-30·6)	32·5 (6·2-71·7)
Palestine	27·2 (18·5-37·5)	27·9 (16·8-40·5)	28·9 (17·0-43·2)	33·3 (10·7-61·5)
Oman	46·8 (29·1-64·6)	59·3 (41·1-75·4)	60·7 (41·6-77·2)	86·0 (53·5-100·0)
Qatar	12·5 (8·0-19·1)	42·2 (30·9-54·6)	42·1 (29·6-56·1)	40·9 (10·7-84·9)
Saudi Arabia	26·9 (24·5-29·4)	42·7 (37·2-48·7)	44·8 (38·6-51·4)	65·7 (54·3-76·7)
Sudan	0·0 (0·0-0·0)	0·0 (0·0-0·0)	0·0 (0·0-0·0)	0·0 (0·0-0·0)
Syria	51·4 (45·8-56·5)	68·6 (52·6-82·2)	69·2 (53·4-83·7)	82·8 (57·7-97·6)
Tunisia	25·4 (12·3-44·0)	30·9 (18·6-47·0)	32·0 (19·2-49·3)	42·6 (15·3-75·1)
Turkey	25·3 (19·0-31·9)	75·5 (62·8-85·2)	75·5 (62·2-86·3)	84·0 (71·0-91·5)
United Arab Emirates	32·8 (17·3-51·1)	45·5 (26·7-65·6)	46·3 (26·8-66·9)	61·1 (25·9-91·8)
Yemen	0·0 (0·0-0·0)	0·0 (0·0-0·0)	0·0 (0·0-0·0)	0·0 (0·0-0·0)
**South Asia**				

Location	Indicator 1.5.1: Death rate due to exposure to forces of nature (per 100,000 population)				Indicator 2.2.1: Prevalence of stunting in children under 5 (%)				Indicator 2.2.2a: Prevalence of wasting in children under 5 (%)				Indicator 2.2.2b: Prevalence of overweight in children aged 2-4 (%)			
	2000	2015	2016	2030	2000	2015	2016	2030	2000	2015	2016	2030	2000	2015	2016	2030
Bangladesh	2·9 (1·3-4·5)	0·2 (0·07-0·3)	0·1 (0·04-0·2)	0·3 (0·2-0·5)	57·8 (53·4-62·0)	38·7 (30·1-49·0)	37·3 (28·3-48·1)	20·3 (12·1-34·3)	13·4 (12·3-14·2)	12·9 (11·5-14·4)	12·6 (11·1-14·2)	9·5 (8·0-11·2)	3·8 (2·2-6·0)	4·5 (2·4-7·5)	4·7 (2·4-8·0)	6·2 (1·6-15·2)
Bhutan	6·9 (2·7-11·2)	0·2 (0·09-0·4)	0·2 (0·07-0·3)	0·8 (0·4-1·2)	53·2 (44·9-63·7)	29·0 (20·1-40·3)	28·0 (18·8-39·9)	16·2 (6·4-26·0)	2·9 (2·3-3·7)	5·7 (4·6-7·1)	5·7 (4·5-7·2)	5·1 (3·4-7·9)	8·3 (4·3-14·3)	15·1 (8·5-25·6)	15·5 (8·5-25·6)	24·7 (8·5-48·7)
India	0·4 (0·2-0·7)	0·1 (0·06-0·2)	0·1 (0·06-0·2)	0·2 (0·1-0·4)	51·8 (50·4-53·1)	43·0 (41·4-44·6)	42·1 (40·5-43·7)	36·3 (33·6-38·8)	19·9 (19·4-20·3)	16·4 (16·1-16·8)	15·9 (15·5-16·2)	13·5 (13·2-13·8)	7·3 (4·2-11·5)	11·3 (7·0-16·9)	11·8 (7·1-17·9)	19·6 (7·3-40·2)
Nepal	1·3 (0·5-2·0)	6·2 (2·5-10·1)	5·6 (2·3-9·1)	3·7 (1·6-5·8)	60·7 (53·6-67·3)	36·5 (27·4-47·1)	35·1 (25·7-46·0)	18·5 (11·7-30·3)	12·1 (10·4-14·0)	10·6 (8·7-12·9)	10·3 (8·3-12·6)	8·4 (7·8-9·1)	4·0 (2·3-6·6)	5·2 (3·1-8·5)	5·4 (3·1-9·1)	9·2 (2·8-22·5)
Pakistan	0·4 (0·2-0·6)	0·4 (0·2-0·6)	0·3 (0·1-0·5)	2·1 (0·9-3·3)	47·6 (40·2-54·9)	42·7 (34·7-50·2)	41·7 (33·1-49·9)	28·8 (18·2-42·8)	11·1 (9·9-12·5)	16·5 (15·0-17·9)	16·3 (14·8-17·8)	18·0 (15·8-19·6)	11·1 (6·0-18·8)	6·2 (3·6-10·2)	6·2 (3·4-10·5)	5·4 (1·4-13·7)
**Southern Sub-Saharan Africa**																
Botswana	0·1 (0·04-0·2)	0·08 (0·02-0·1)	0·07 (0·02-0·1)	0·05 (0·02-0·09)	29·7 (26·8-33·2)	24·3 (17·8-31·1)	23·2 (16·5-30·1)	21·2 (15·3-26·0)	6·5 (5·7-7·5)	6·0 (4·8-7·4)	5·9 (4·7-7·4)	5·4 (4·3-7·1)	20·5 (16·4-25·4)	26·8 (20·0-34·3)	27·5 (20·5-35·1)	37·6 (21·9-54·3)
Lesotho	0·0 (0·0-0·0)	0·1 (0·04-0·2)	0·1 (0·04-0·2)	0·09 (0·03-0·2)	52·7 (48·2-58·9)	32·8 (26·7-40·4)	31·7 (25·5-39·4)	18·5 (12·5-26·1)	8·7 (7·5-10·1)	3·7 (2·8-4·6)	3·5 (2·7-4·5)	1·7 (1·2-2·3)	15·3 (11·6-20·2)	15·4 (11·8-19·5)	15·7 (11·9-20·2)	17·1 (9·8-26·2)
Namibia	0·0 (0·0-0·0)	1·0 (0·3-1·5)	0·8 (0·3-1·2)	0·7 (0·2-1·1)	29·2 (25·5-33·1)	21·9 (16·6-27·1)	20·9 (15·4-26·5)	15·8 (12·9-19·2)	9·6 (8·1-11·2)	8·3 (6·8-9·9)	8·2 (6·6-9·9)	6·7 (5·1-9·2)	6·9 (5·0-9·3)	11·3 (8·5-14·8)	11·5 (8·5-15·2)	15·7 (8·1-26·1)
South Africa	0·4 (0·2-0·6)	0·3 (0·1-0·5)	0·2 (0·1-0·4)	0·3 (0·2-0·5)	26·9 (24·6-29·0)	19·7 (18·1-21·3)	19·3 (17·7-20·9)	14·6 (12·7-16·7)	6·0 (5·5-6·5)	5·2 (4·8-5·7)	5·2 (4·8-5·6)	4·5 (4·1-5·0)	25·8 (20·4-32·1)	31·5 (23·9-39·6)	31·8 (24·2-39·9)	39·9 (24·8-56·9)
Swaziland	0·0 (0·0-0·0)	0·1 (0·03-0·2)	0·1 (0·03-0·2)	0·08 (0·02-0·1)	36·3 (31·5-41·4)	27·1 (20·3-33·3)	26·0 (19·1-32·4)	20·4 (12·7-27·2)	2·4 (1·8-3·1)	2·0 (1·4-2·8)	2·0 (1·3-2·7)	1·6 (1·0-2·5)	22·4 (16·9-28·8)	22·4 (16·5-29·7)	22·7 (16·5-30·1)	24·1 (12·8-38·5)
Zimbabwe	0·2 (0·07-0·3)	0·1 (0·05-0·2)	0·1 (0·04-0·2)	0·1 (0·03-0·2)	31·9 (27·3-36·6)	27·9 (23·1-32·8)	26·8 (21·9-31·8)	27·1 (22·5-31·4)	7·4 (6·4-8·4)	3·7 (3·1-4·4)	3·6 (3·0-4·3)	2·5 (1·9-3·1)	18·1 (15·3-21·4)	11·0 (8·3-14·0)	11·2 (8·4-14·4)	8·3 (4·5-13·9)
**Western Sub-Saharan Africa**																
Benin	0·05 (0·02-0·08)	0·06 (0·02-0·1)	0·04 (0·01-0·07)	0·05 (0·02-0·08)	41·0 (35·4-49·9)	34·6 (27·9-42·8)	32·3 (25·4-40·9)	36·5 (28·9-42·9)	11·3 (10·3-12·4)	9·3 (8·0-10·7)	8·9 (7·5-10·3)	6·9 (5·7-8·0)	11·0 (7·9-14·7)	20·4 (13·8-29·1)	20·5 (13·9-29·2)	33·1 (16·7-53·6)
Burkina Faso	0·02 (0·0-0·03)	0·04 (0·02-0·06)	0·03 (0·01-0·04)	0·03 (0·01-0·05)	49·5 (42·6-56·8)	31·3 (25·4-37·6)	30·4 (24·4-36·9)	19·2 (12·5-26·5)	18·7 (17·6-19·6)	13·4 (12·3-14·4)	12·8 (11·6-13·8)	6·6 (5·7-7·4)	11·6 (9·0-14·5)	13·4 (9·9-17·2)	13·8 (10·0-18·1)	18·3 (9·9-29·7)
Cameroon	0·03 (0·0-0·05)	0·02 (0·0-0·03)	0·01 (0·0-0·02)	0·02 (0·01-0·03)	34·1 (29·6-39·8)	30·5 (24·9-36·4)	29·7 (24·0-35·7)	22·8 (16·1-30·2)	7·3 (6·5-8·2)	5·3 (4·7-6·1)	5·2 (4·6-5·9)	3·7 (3·1-4·5)	19·8 (14·9-25·2)	21·7 (15·7-29·3)	22·4 (16·0-30·5)	26·2 (14·2-42·3)
Cape Verde	0·0 (0·0-0·0)	0·0 (0·0-0·0)	0·0 (0·0-0·0)	0·0 (0·0-0·0)	18·3 (11·0-27·0)	11·4 (6·5-17·6)	11·0 (6·3-17·1)	6·9 (3·8-11·3)	6·1 (4·8-7·6)	4·3 (3·2-5·6)	4·2 (3·1-5·5)	3·2 (2·1-4·3)	13·1 (8·6-19·0)	20·7 (13·7-29·0)	21·3 (14·2-29·3)	30·7 (15·6-49·4)
Chad	0·01 (0·0-0·02)	0·06 (0·03-0·09)	0·04 (0·01-0·06)	0·08 (0·05-0·1)	41·9 (36·7-49·5)	37·1 (30·1-44·7)	36·6 (29·3-44·4)	30·3 (23·0-39·3)	15·2 (13·9-16·5)	14·6 (12·8-16·3)	14·4 (12·5-16·2)	11·8 (9·5-14·5)	9·4 (7·1-12·4)	9·2 (6·9-12·2)	9·4 (6·9-12·7)	10·9 (5·6-18·6)
Cote d'Ivoire	0·02 (0·0-0·04)	0·04 (0·01-0·08)	0·04 (0·01-0·07)	0·03 (0·01-0·05)	33·9 (25·9-43·9)	30·1 (21·0-41·0)	29·3 (19·9-40·3)	18·7 (10·2-31·6)	9·5 (7·9-11·2)	9·3 (7·3-11·3)	9·1 (7·1-11·2)	8·3 (6·1-10·7)	12·8 (9·2-17·5)	13·9 (9·2-19·2)	14·3 (9·5-20·2)	17·1 (7·8-30·5)
The Gambia	0·7 (0·1-1·4)	0·0 (0·0-0·0)	0·0 (0·0-0·0)	0·07 (0·01-0·1)	26·8 (22·9-31·9)	22·7 (16·6-30·0)	22·5 (15·9-30·4)	19·0 (9·4-34·0)	9·7 (8·0-11·9)	12·2 (10·3-14·2)	12·2 (10·3-14·3)	13·3 (10·5-16·5)	15·4 (10·3-21·9)	12·7 (8·2-18·6)	12·9 (8·3-19·0)	15·7 (6·4-30·1)
Ghana	0·1 (0·05-0·2)	0·05 (0·01-0·08)	0·03 (0·01-0·06)	0·05 (0·02-0·08)	33·2 (26·9-41·1)	20·0 (15·1-25·5)	18·3 (13·3-23·8)	15·2 (13·3-17·6)	8·7 (7·8-9·6)	7·9 (6·7-9·2)	7·5 (6·3-8·9)	7·6 (6·4-8·9)	8·6 (6·4-11·2)	8·7 (6·0-12·1)	8·9 (6·1-12·6)	10·4 (5·1-18·4)
Guinea	0·0 (0·0-0·0)	0·0 (0·0-0·0)	0·0 (0·0-0·0)	0·0 (0·0-0·0)	38·8 (34·8-44·6)	31·6 (23·4-41·4)	30·5 (21·9-40·9)	22·2 (13·4-33·0)	11·4 (10·2-12·6)	8·2 (6·8-9·7)	8·1 (6·7-9·6)	6·1 (4·8-7·6)	11·3 (9·3-13·4)	14·1 (9·2-20·2)	14·3 (9·4-20·5)	18·1 (8·5-33·5)
Guinea-Bissau	0·0 (0·0-0·0)	0·0 (0·0-0·0)	0·0 (0·0-0·0)	0·0 (0·0-0·0)	38·7 (32·9-47·6)	28·2 (21·2-37·0)	27·3 (20·1-36·3)	17·0 (8·4-29·3)	11·6 (10·2-12·9)	6·9 (5·7-8·2)	6·8 (5·5-8·1)	4·8 (3·5-5·8)	11·5 (7·5-16·9)	18·8 (12·6-26·5)	19·0 (12·6-26·9)	23·0 (11·1-38·5)
Liberia	0·06 (0·01-0·1)	0·0 (0·0-0·0)	0·0 (0·0-0·0)	0·0 (0·0-0·01)	52·4 (46·2-59·7)	32·4 (23·7-43·2)	31·5 (22·9-42·4)	20·3 (12·3-31·6)	6·7 (5·5-8·0)	6·5 (5·2-7·9)	6·4 (5·1-7·8)	5·6 (4·3-7·0)	11·8 (8·0-16·7)	16·2 (10·9-22·8)	16·4 (11·1-23·1)	20·8 (9·2-38·0)
Mali	0·03 (0·01-0·05)	0·05 (0·01-0·08)	0·04 (0·01-0·07)	0·03 (0·01-0·05)	40·6 (33·3-48·7)	25·5 (17·5-33·8)	24·9 (16·5-33·5)	17·5 (7·4-29·9)	18·5 (16·3-20·7)	11·4 (9·5-13·3)	11·1 (9·2-13·1)	7·0 (5·4-9·1)	9·3 (7·0-11·8)	13·5 (8·7-19·7)	13·8 (8·8-20·1)	19·7 (8·7-35·6)
Mauritania	0·0 (0·0-0·0)	0·0 (0·0-0·0)	0·0 (0·0-0·0)	0·02 (0·0-0·04)	42·1 (36·8-49·1)	25·3 (20·7-29·8)	25·0 (20·3-29·6)	14·6 (11·8-19·3)	14·7 (13·0-16·3)	12·3 (10·9-13·8)	12·3 (10·8-13·8)	11·8 (9·8-13·0)	9·8 (6·8-13·7)	10·6 (6·9-15·5)	11·0 (7·2-16·2)	14·4 (6·2-27·6)
Niger	0·05 (0·01-0·09)	0·2 (0·07-0·2)	0·1 (0·06-0·2)	0·09 (0·04-0·1)	54·3 (48·4-61·0)	45·3 (34·2-55·6)	44·6 (32·9-55·1)	34·9 (21·9-48·9)	19·7 (17·5-21·9)	18·5 (15·6-21·5)	18·4 (15·5-21·6)	17·8 (13·3-22·5)	5·7 (4·0-7·9)	7·5 (5·1-10·2)	7·5 (5·2-10·5)	7·5 (3·4-14·0)
Nigeria	0·03 (0·0-0·05)	0·04 (0·01-0·08)	0·04 (0·01-0·07)	0·03 (0·01-0·06)	47·2 (41·7-53·6)	35·7 (28·5-43·3)	33·5 (26·2-41·6)	29·4 (23·4-36·9)	14·0 (12·7-15·4)	11·7 (10·6-12·7)	11·6 (10·5-12·7)	10·5 (9·1-12·2)	11·9 (8·6-16·1)	17·8 (12·2-24·5)	17·8 (12·1-24·8)	23·0 (11·4-39·8)
Sao Tome and Principe	0·0 (0·0-0·0)	0·0 (0·0-0·0)	0·0 (0·0-0·0)	0·0 (0·0-0·0)	36·7 (31·8-45·1)	17·9 (12·6-24·5)	16·7 (11·4-22·9)	9·2 (5·2-13·4)	6·4 (5·4-7·5)	5·6 (4·5-6·8)	5·3 (4·2-6·5)	3·2 (2·5-4·2)	13·5 (9·0-19·0)	10·3 (7·0-14·6)	10·4 (7·0-14·7)	11·0 (4·9-20·2)
Senegal	0·3 (0·05-0·6)	0·02 (0·0-0·03)	0·02 (0·0-0·03)	0·05 (0·01-0·08)	30·9 (25·9-36·3)	19·8 (14·6-24·6)	18·8 (13·6-23·8)	9·1 (4·9-13·8)	10·8 (9·4-12·5)	7·7 (6·5-9·1)	7·6 (6·3-8·9)	5·5 (4·1-7·3)	7·0 (4·6-10·2)	7·5 (4·9-10·8)	7·7 (5·0-11·4)	7·8 (3·3-15·0)

Location	Indicator 3.1.1: Maternal mortality ratio (maternal deaths per 100,000 livebirths) in women aged 10-54 years				Indicator 3.1.2: Proportion of births attended by skilled health personnel (%)				Indicator 3.2.1: Under-5 mortality rate (probability of dying before the age of 5 per 1,000 livebirths)				Indicator 3.2.2: Neonatal mortality rate (probability of dying during the first 28 days of life per 1,000 livebirths)			
	**2000**	**2015**	**2016**	**2030**	**2000**	**2015**	**2016**	**2030**	**2000**	**2015**	**2016**	**2030**	**2000**	**2015**	**2016**	**2030**
Bangladesh	461·7 (386·2-539·2)	195·5 (147·8-252·3)	187·4 (140·2-246·4)	99·0 (44·7-192·5)	16·8 (13·8-20·0)	45·0 (34·6-55·6)	46·0 (34·4-58·2)	75·5 (47·3-93·8)	86·1 (81·7-90·5)	36·5 (32·7-40·6)	34·0 (30·3-38·2)	14·0 (10·9-18·2)	42·9 (40·5-45·4)	22·3 (19·6-25·3)	21·0 (18·4-23·8)	10·6 (8·1-13·7)
Bhutan	320·9 (244·9-402·2)	173·8 (111·6-256·1)	167·2 (105·3-248·7)	97·8 (38·4-209·9)	35·5 (18·6-54·7)	63·6 (45·2-79·0)	64·4 (45·7-80·5)	82·8 (48·4-99·4)	85·4 (76·0-96·0)	35·1 (29·6-42·4)	32·9 (27·6-39·8)	13·7 (10·2-18·8)	42·0 (37·3-47·1)	21·0 (17·7-25·4)	19·8 (16·7-24·0)	9·9 (7·4-13·7)
India	357·4 (316·7-390·7)	217·9 (193·7-245·9)	207·0 (183·0-235·2)	127·2 (100·9-164·3)	46·4 (37·9-54·7)	72·2 (55·8-84·8)	73·7 (56·8-86·2)	89·0 (64·6-100·0)	82·9 (78·0-88·1)	41·7 (35·5-49·7)	39·2 (33·0-47·3)	18·6 (12·8-27·5)	39·5 (36·5-42·7)	23·0 (19·0-28·1)	21·8 (17·8-27·1)	12·1 (8·0-18·4)
Nepal	413·4 (351·9-472·8)	252·3 (190·1-312·8)	241·2 (182·2-300·6)	139·2 (73·9-220·6)	20·0 (16·0-24·3)	52·3 (41·0-63·9)	54·4 (41·5-66·8)	84·6 (63·4-98·1)	81·0 (76·0-86·2)	32·5 (28·7-36·8)	29·4 (25·8-33·5)	11·0 (8·4-14·4)	40·4 (37·4-43·6)	19·8 (17·2-22·8)	18·5 (16·0-21·3)	8·4 (6·3-11·3)
Pakistan	458·7 (376·0-542·8)	309·2 (226·1-406·6)	300·2 (218·0-395·8)	195·0 (94·3-349·2)	35·2 (29·9-41·1)	65·7 (59·3-71·6)	66·1 (57·8-73·3)	87·5 (73·2-96·0)	90·7 (86·0-95·7)	56·4 (49·8-64·1)	53·4 (46·9-61·1)	29·8 (22·7-38·9)	46·5 (43·7-49·4)	33·4 (29·3-38·1)	31·8 (27·8-36·4)	21·1 (16·1-28·0)
**Southern Sub-Saharan Africa**																
Botswana	261·0 (89·1-741·5)	68·6 (15·1-149·5)	69·9 (14·5-147·0)	27·6 (0·8-124·3)	92·8 (88·1-96·1)	96·4 (90·6-99·0)	96·2 (89·8-99·1)	97·4 (78·6-100·0)	63·5 (55·9-72·3)	15·6 (11·7-21·0)	14·9 (11·1-20·4)	3·9 (2·0-7·2)	18·9 (16·6-21·6)	7·6 (5·6-10·4)	7·2 (5·3-10·1)	3·1 (1·7-5·8)
Lesotho	384·9 (205·9-588·9)	358·5 (177·4-607·2)	342·9 (159·7-615·8)	227·0 (32·9-702·1)	57·3 (52·1-62·6)	81·7 (75·5-86·9)	81·5 (73·1-88·1)	94·1 (82·6-99·4)	99·6 (91·5-108·1)	67·6 (54·8-82·7)	66·6 (53·2-83·0)	47·1 (28·0-74·2)	38·1 (34·8-41·3)	28·3 (22·6-35·2)	27·9 (22·1-35·0)	23·0 (13·8-36·5)
Namibia	312·8 (124·3-516·8)	128·8 (42·5-233·2)	123·3 (39·2-224·6)	56·1 (9·8-152·9)	79·4 (76·3-82·5)	89·6 (83·6-93·6)	89·2 (81·5-94·3)	93·5 (79·4-99·8)	63·8 (55·5-73·4)	37·8 (25·5-53·0)	36·5 (24·0-53·3)	18·7 (5·8-43·5)	21·7 (19·0-25·0)	16·5 (11·1-23·0)	16·0 (10·5-23·3)	10·9 (3·6-24·2)
South Africa	222·8 (165·4-282·0)	205·8 (160·6-252·2)	210·6 (164·6-257·9)	104·3 (46·5-224·8)	89·6 (85·6-92·8)	99·0 (97·8-99·7)	99·0 (97·5-99·8)	99·6 (97·4-100·0)	75·7 (69·1-83·1)	45·7 (35·7-57·6)	43·4 (33·2-55·9)	21·5 (9·4-41·0)	23·1 (20·8-25·6)	15·9 (12·4-20·2)	15·2 (11·6-19·8)	9·3 (4·3-16·9)
Swaziland	130·0 (66·1-281·1)	84·5 (29·5-187·3)	80·7 (26·8-181·0)	30·5 (2·1-133·2)	77·0 (70·5-82·9)	88·2 (76·6-95·3)	88·2 (75·5-95·9)	94·1 (66·8-100·0)	93·7 (86·4-101·2)	50·3 (40·6-61·3)	49·2 (39·2-61·1)	23·1 (12·5-41·1)	21·7 (20·0-23·7)	16·2 (13·6-19·1)	15·9 (13·2-19·1)	10·8 (6·4-17·7)
Zimbabwe	445·1 (264·7-750·0)	393·0 (257·3-570·9)	381·5 (253·9-553·8)	251·8 (85·4-615·1)	75·2 (71·1-78·9)	83·1 (76·3-88·6)	85·6 (77·6-91·6)	97·4 (86·6-100·0)	77·4 (72·5-82·3)	57·8 (49·2-68·7)	56·5 (47·3-68·6)	40·7 (23·6-65·3)	23·8 (21·6-26·1)	24·0 (20·4-28·4)	23·5 (19·7-28·4)	17·9 (10·8-28·3)
**Western Sub-Saharan Africa**																
Benin	457·4 (372·5-544·6)	333·7 (270·7-407·3)	320·7 (257·2-388·3)	219·8 (141·0-320·3)	75·8 (72·7-78·7)	87·6 (80·6-92·7)	87·9 (79·9-93·5)	93·0 (77·7-99·2)	139·5 (130·0-148·8)	76·1 (64·2-91·1)	72·2 (60·5-87·5)	41·0 (28·9-58·1)	38·1 (35·5-41·0)	26·2 (22·7-30·5)	25·1 (21·6-29·4)	18·0 (13·4-24·3)
Burkina Faso	416·7 (344·3-488·8)	328·5 (263·2-396·9)	321·0 (254·6-389·3)	256·2 (154·9-395·7)	41·9 (37·2-47·1)	72·3 (57·6-84·0)	71·5 (54·8-84·3)	78·8 (38·1-98·7)	178·1 (168·5-188·1)	112·8 (99·1-129·3)	108·8 (94·7-126·7)	68·6 (52·2-89·9)	40·3 (38·1-42·6)	29·6 (25·5-34·8)	28·7 (24·5-34·3)	21·2 (15·6-29·1)
Cameroon	548·0 (428·6-658·9)	445·0 (299·3-580·5)	432·9 (287·7-570·7)	327·9 (143·9-563·1)	55·8 (52·7-58·8)	68·8 (58·2-78·5)	70·1 (58·0-81·2)	79·2 (50·4-96·9)	136·2 (126·5-147·8)	91·1 (73·7-112·0)	88·1 (70·1-110·1)	57·5 (36·0-88·0)	36·3 (33·2-39·9)	28·0 (22·6-34·6)	27·2 (21·6-34·0)	20·8 (12·9-31·3)
Cape Verde	56·1 (37·1-80·0)	26·2 (19·4-35·4)	26·1 (19·0-35·1)	15·7 (7·5-32·3)	61·8 (44·2-78·0)	84·0 (67·2-93·8)	84·7 (68·6-93·9)	93·9 (74·2-100·0)	45·0 (39·1-51·4)	18·1 (16·0-20·4)	17·0 (14·9-19·5)	7·4 (5·2-10·4)	16·6 (14·3-19·3)	9·4 (7·9-11·1)	8·9 (7·3-10·6)	4·8 (3·0-7·0)
Chad	669·6 (552·7-772·3)	549·5 (430·7-648·4)	536·4 (423·9-632·7)	414·8 (263·5-594·6)	22·2 (18·3-26·3)	22·9 (16·7-30·3)	23·5 (16·1-32·4)	28·8 (10·1-56·7)	182·7 (170·7-194·9)	117·5 (102·7-135·9)	113·1 (97·9-132·3)	69·8 (53·0-91·5)	43·3 (40·1-46·6)	31·7 (27·9-36·5)	30·8 (26·9-35·6)	22·8 (17·3-30·1)
Cote d'Ivoire	612·0 (505·8-745·9)	466·5 (378·5-578·5)	448·0 (363·6-561·3)	320·2 (194·6-500·6)	65·2 (60·7-69·4)	65·6 (51·9-76·7)	66·4 (51·4-78·6)	75·6 (38·5-98·2)	137·0 (128·2-146·5)	89·5 (76·9-104·8)	85·3 (72·5-100·4)	53·1 (37·9-74·8)	44·5 (40·8-48·6)	33·5 (28·5-39·1)	32·1 (27·1-37·9)	23·8 (16·7-32·8)
The Gambia	316·4 (263·6-364·5)	318·3 (255·6-378·4)	311·5 (249·9-373·2)	240·0 (146·1-372·2)	56·5 (51·2-61·8)	67·1 (60·4-74·0)	67·9 (59·5-76·1)	77·7 (57·6-92·8)	86·0 (78·9-93·2)	50·6 (43·6-59·7)	48·7 (41·5-57·9)	29·7 (21·8-40·3)	31·7 (29·0-34·4)	22·1 (19·0-26·1)	21·3 (18·2-25·4)	15·1 (11·1-20·7)
Ghana	250·5 (204·1-291·3)	139·7 (106·2-174·8)	135·2 (101·1-170·9)	85·6 (42·2-152·9)	41·4 (35·4-47·0)	73·2 (63·9-81·1)	74·0 (63·6-83·1)	91·3 (74·2-99·6)	101·1 (96·0-106·8)	55·4 (47·4-65·8)	52·3 (44·0-63·2)	26·2 (16·7-40·1)	35·6 (33·6-37·9)	24·6 (21·3-28·5)	23·4 (20·0-27·7)	14·4 (9·5-21·7)
Guinea	698·9 (596·6-794·0)	532·5 (435·5-640·6)	520·0 (421·1-631·4)	384·7 (246·3-565·9)	32·7 (29·3-36·1)	49·0 (38·1-59·7)	50·2 (37·3-62·8)	65·1 (31·3-90·3)	172·8 (160·1-186·0)	102·1 (85·2-123·8)	97·5 (80·6-119·6)	56·1 (37·6-82·7)	48·0 (43·9-52·3)	33·3 (27·4-40·8)	32·1 (26·2-39·9)	22·0 (14·5-32·5)
Guinea-Bissau	513·0 (412·8-621·4)	364·7 (269·9-486·1)	355·3 (262·7-471·6)	249·2 (127·6-429·7)	40·5 (33·1-48·5)	50·1 (31·5-68·7)	50·2 (30·8-70·1)	59·6 (20·3-92·0)	157·8 (145·9-170·0)	76·0 (66·0-87·0)	71·8 (62·0-82·7)	32·5 (23·9-43·5)	50·0 (46·5-53·5)	30·4 (26·7-34·5)	29·0 (25·3-33·1)	17·1 (12·8-22·5)
Liberia	717·6 (580·6-831·9)	548·3 (460·7-652·1)	530·4 (439·7-636·4)	336·9 (218·1-492·9)	39·9 (31·1-49·0)	67·7 (59·0-75·1)	69·0 (58·6-77·7)	85·5 (65·9-96·2)	171·5 (162·6-180·4)	69·4 (60·2-80·6)	65·3 (56·3-76·7)	29·5 (21·6-39·9)	44·3 (42·0-46·8)	23·6 (21·3-26·4)	22·4 (20·1-25·3)	12·9 (10·1-16·4)
Mali	509·4 (434·1-579·4)	388·3 (277·1-505·1)	379·6 (268·0-493·6)	286·3 (138·7-478·0)	33·6 (29·6-38·2)	53·8 (40·2-65·8)	55·7 (40·8-69·6)	78·1 (44·1-98·2)	192·4 (181·5-205·5)	121·6 (102·8-145·5)	117·1 (97·6-142·4)	74·4 (50·4-109·5)	53·6 (50·4-57·3)	39·2 (33·6-46·4)	38·0 (32·0-45·8)	27·9 (19·1-40·2)
Mauritania	684·1 (429·8-817·1)	473·5 (283·5-642·2)	461·8 (277·9-632·8)	333·7 (139·9-631·9)	65·4 (60·6-69·8)	71·7 (59·0-81·7)	73·0 (59·1-84·2)	86·2 (54·7-100·0)	92·5 (85·6-100·0)	52·9 (45·6-61·0)	50·3 (43·1-58·8)	26·9 (19·6-36·6)	37·2 (34·3-40·4)	25·0 (21·5-28·9)	23·9 (20·4-28·0)	15·2 (11·0-20·5)
Niger	498·6 (404·1-597·1)	444·8 (327·2-554·8)	436·5 (321·9-540·5)	355·7 (192·1-545·7)	20·0 (16·8-23·3)	35·8 (25·5-46·9)	35·9 (24·2-48·7)	57·7 (23·9-89·4)	224·8 (210·3-239·8)	113·2 (96·2-134·0)	108·2 (90·9-130·6)	57·7 (40·3-81·7)	42·7 (39·0-46·6)	27·2 (22·2-33·6)	26·3 (21·2-33·1)	18·0 (11·9-26·9)
Nigeria	410·0 (279·7-538·0)	264·5 (176·2-364·9)	265·0 (170·8-366·4)	195·1 (74·6-407·4)	35·3 (29·2-41·8)	41·6 (32·2-51·1)	40·5 (29·7-52·0)	39·8 (14·6-68·6)	183·8 (174·4-193·3)	115·4 (99·6-132·3)	108·7 (93·0-126·2)	70·0 (47·1-97·2)	49·5 (46·3-52·7)	37·4 (30·6-45·3)	35·7 (28·8-43·5)	27·0 (16·2-40·6)
Sao Tome and Principe	217·2 (93·1-284·4)	111·1 (73·3-150·4)	110·2 (76·3-149·9)	98·7 (37·4-232·2)	81·8 (73·9-88·4)	87·7 (79·0-94·0)	87·9 (78·4-94·3)	91·6 (72·1-99·7)	73·8 (66·9-80·7)	32·1 (27·3-37·6)	30·4 (25·6-36·1)	14·1 (9·6-20·3)	21·5 (19·3-23·7)	13·4 (11·3-15·8)	12·8 (10·7-15·2)	8·1 (5·4-11·5)
Senegal	510·7 (441·1-576·8)	369·5 (295·0-450·1)	361·8 (287·7-443·0)	273·5 (170·9-424·6)	62·1 (56·5-67·6)	56·7 (49·8-63·5)	57·7 (49·1-66·1)	56·9 (34·6-76·7)	114·9 (109·6-120·6)	52·0 (46·7-58·5)	49·4 (43·9-56·3)	22·7 (17·6-29·8)	36·2 (33·6-38·9)	21·0 (18·8-23·8)	20·1 (17·8-23·0)	11·4 (8·5-15·2)

Location	Indicator 3.3.1: Age-standardised rate of new HIV infections (per 1,000 population)				Indicator 3.3.2: Age-standardised rate of tuberculosis cases (per 100,000 population)				Indicator 3.3.3: Age-standardised rate of malaria cases (per 1,000 population)				Indicator 3.3.4: Age-standardised rate of hepatitis B incidence (per 100,000 population)			
	2000	2015	2016	2030	2000	2015	2016	2030	2000	2015	2016	2030	2000	2015	2016	2030
Bangladesh	0·001 (0·0-0·005)	0·003 (0·0-0·01)	0·003 (0·0-0·01)	0·003 (0·0-0·01)	238·8 (211·5-266·9)	131·8 (116·9-147·7)	132·6 (117·6-148·6)	107·6 (95·3-120·7)	7·9 (5·8-9·8)	0·2 (0·1-0·3)	0·1 (0·06-0·2)	0·002 (0·001-0·003)	4,011·0 (3,004·3-5,153·8)	1,909·0 (1,431·6-2,434·1)	1,882·5 (1,412·0-2,400·8)	1,433·2 (1,054·2-1,904·9)
Bhutan	0·05 (0·007-0·2)	0·07 (0·006-0·3)	0·07 (0·006-0·3)	0·07 (0·006-0·3)	221·2 (190·7-256·0)	150·5 (130·1-173·6)	147·8 (127·7-170·6)	108·6 (93·1-127·0)	22·0 (15·0-31·4)	0·07 (0·07-0·07)	0·04 (0·04-0·04)	0·0 (0·0-0·0)	6,765·3 (5,008·6-9,227·5)	2,736·7 (2,124·8-3,504·2)	2,584·5 (2,011·4-3,288·8)	1,132·6 (878·6-1,466·4)
India	0·3 (0·2-0·4)	0·09 (0·05-0·1)	0·09 (0·05-0·2)	0·08 (0·04-0·1)	319·4 (295·2-346·2)	247·8 (230·3-267·1)	242·8 (225·7-261·7)	194·3 (182·0-208·1)	22·0 (17·1-26·3)	12·1 (7·7-16·9)	11·5 (7·0-16·3)	5·5 (2·8-9·6)	6,461·7 (4,931·4-8,210·9)	4,467·2 (3,494·4-5,606·4)	4,281·0 (3,348·7-5,371·5)	2,498·8 (1,924·7-3,139·5)
Nepal	0·2 (0·03-0·6)	0·04 (0·004-0·2)	0·04 (0·004-0·1)	0·05 (0·004-0·2)	258·7 (229·1-292·0)	149·7 (132·1-169·8)	144·1 (127·1-163·4)	83·8 (73·5-95·0)	2·6 (1·8-3·5)	0·6 (0·5-0·7)	0·6 (0·6-0·6)	0·1 (0·08-0·2)	3,940·7 (3,072·4-4,963·5)	2,094·1 (1,613·5-2,635·0)	1,981·9 (1,528·3-2,495·4)	1,121·2 (845·1-1,459·8)
Pakistan	0·006 (0·001-0·02)	0·04 (0·004-0·1)	0·04 (0·004-0·1)	0·04 (0·004-0·1)	325·2 (264·5-399·0)	241·9 (195·5-298·9)	236·5 (190·7-292·9)	172·2 (137·2-215·9)	15·2 (10·7-19·8)	5·5 (4·6-6·7)	4·7 (3·9-5·7)	3·0 (1·0-4·7)	3,590·3 (2,821·3-4,464·4)	2,516·1 (1,959·8-3,199·1)	2,445·3 (1,904·1-3,113·8)	1,639·1 (1,226·6-2,186·3)
**Southern Sub-Saharan Africa**																
Botswana	19·8 (14·7-25·8)	5·9 (4·1-8·0)	4·2 (2·6-6·1)	3·1 (1·8-4·6)	2,126·4 (1,735·0-2,591·4)	1,119·9 (899·8-1,385·3)	1,068·5 (855·2-1,324·2)	491·1 (369·6-632·7)	3·0 (2·1-4·0)	0·2 (0·2-0·2)	0·1 (0·1-0·1)	0·001 (0·0-0·001)	833·6 (642·1-1,069·9)	546·0 (428·3-688·9)	535·0 (420·6-675·0)	398·7 (317·8-501·3)
Lesotho	19·1 (16·3-22·5)	8·9 (6·9-11·3)	8·4 (6·4-10·9)	5·5 (4·3-7·1)	1,802·0 (1,447·5-2,238·0)	2,221·5 (1,727·4-2,824·8)	2,203·4 (1,708·9-2,805·0)	1,849·6 (1,344·2-2,459·2)	0·0 (0·0-0·0)	0·0 (0·0-0·0)	0·0 (0·0-0·0)	0·0 (0·0-0·0)	985·3 (746·0-1,247·7)	904·6 (679·2-1,148·4)	885·2 (664·3-1,123·0)	824·5 (617·5-1,055·2)
Namibia	15·2 (12·3-19·1)	4·4 (3·2-5·7)	4·1 (2·9-5·5)	2·2 (1·5-3·0)	1,637·9 (1,318·6-2,024·4)	985·5 (787·5-1,221·1)	951·7 (758·8-1,178·3)	528·0 (404·6-666·9)	48·7 (21·7-96·4)	2·7 (2·6-2·8)	3·3 (3·3-3·3)	9·1 (3·0-16·6)	874·7 (682·6-1,115·1)	584·0 (463·4-733·4)	567·4 (449·9-712·4)	384·0 (310·5-471·7)
South Africa	16·8 (14·6-19·7)	6·0 (5·0-7·3)	4·6 (3·6-5·8)	2·3 (1·8-2·9)	683·8 (588·5-797·7)	922·4 (790·8-1,069·7)	876·8 (750·5-1,017·0)	805·6 (562·5-1,090·1)	6·5 (3·7-10·2)	4·2 (1·4-8·3)	4·5 (1·5-9·2)	4·5 (1·3-9·2)	1,006·8 (778·0-1,302·9)	665·9 (528·4-833·5)	651·4 (517·6-813·9)	458·4 (364·1-571·6)
Swaziland	21·3 (16·0-27·2)	7·0 (5·8-8·6)	5·3 (4·3-6·8)	3·4 (2·7-4·4)	2,061·7 (1,650·8-2,575·0)	1,643·7 (1,276·4-2,080·8)	1,562·3 (1,210·9-1,981·1)	985·0 (737·6-1,275·7)	1·9 (0·8-3·9)	0·3 (0·2-0·3)	0·2 (0·2-0·2)	0·05 (0·02-0·2)	936·5 (722·1-1,180·5)	769·3 (594·0-962·2)	742·8 (574·3-928·5)	541·9 (418·4-678·0)
Zimbabwe	9·8 (6·7-13·3)	4·2 (3·3-5·4)	4·3 (3·2-5·5)	3·3 (2·4-4·2)	2,031·9 (1,665·7-2,486·9)	1,187·1 (950·9-1,470·6)	1,098·6 (876·5-1,361·5)	556·5 (419·8-711·3)	132·3 (45·6-262·7)	83·7 (40·6-136·7)	79·2 (37·0-131·7)	36·6 (13·5-74·0)	920·2 (681·0-1,209·0)	1,039·0 (764·0-1,375·9)	1,020·4 (750·8-1,348·2)	1,026·4 (759·8-1,366·0)
**Western Sub-Saharan Africa**																
Benin	1·1 (0·8-1·5)	0·5 (0·4-0·7)	0·5 (0·4-0·6)	0·4 (0·3-0·5)	224·0 (188·9-265·6)	156·5 (132·0-185·1)	153·4 (129·3-181·4)	113·6 (94·9-135·6)	227·6 (153·6-306·2)	237·6 (178·5-308·4)	236·0 (176·9-307·1)	215·5 (153·2-296·2)	6,068·3 (4,273·7-8,494·5)	3,719·6 (2,632·3-5,190·6)	3,620·9 (2,567·9-5,043·5)	2,446·8 (1,769·6-3,330·8)
Burkina Faso	0·5 (0·2-0·9)	0·3 (0·1-0·5)	0·3 (0·1-0·7)	0·3 (0·1-0·5)	317·4 (266·8-376·4)	224·2 (192·8-263·5)	219·9 (189·4-258·3)	161·9 (137·5-191·7)	397·0 (322·0-478·2)	326·5 (268·1-394·8)	315·5 (259·4-381·9)	266·1 (223·0-325·2)	5,465·8 (3,920·5-7,278·7)	3,930·4 (2,845·9-5,188·7)	3,867·8 (2,804·7-5,096·0)	3,044·8 (2,251·3-3,967·9)
Cameroon	4·0 (3·1-5·0)	1·9 (1·4-2·5)	1·8 (1·4-2·5)	1·4 (1·0-1·9)	342·1 (286·9-402·4)	252·5 (211·6-297·3)	246·7 (206·8-291·0)	178·2 (149·2-210·9)	268·8 (204·7-340·9)	193·0 (146·9-252·8)	188·7 (143·2-248·2)	137·7 (98·2-195·1)	7,350·9 (5,344·1-10,366·9)	4,157·8 (3,096·0-5,616·7)	4,034·9 (3,007·6-5,433·5)	2,626·6 (1,977·4-3,424·4)
Cape Verde	0·6 (0·3-0·9)	0·5 (0·4-0·8)	0·6 (0·4-0·9)	0·4 (0·3-0·7)	125·5 (104·7-150·7)	81·0 (67·1-97·9)	78·9 (65·3-95·4)	54·3 (44·9-66·3)	0·6 (0·4-0·7)	0·02 (0·02-0·02)	0·01 (0·01-0·01)	0·001 (0·001-0·001)	1,839·9 (1,402·0-2,438·1)	1,215·8 (919·6-1,615·2)	1,193·8 (902·6-1,586·0)	804·3 (601·9-1,084·6)
Chad	2·8 (2·0-3·8)	1·2 (0·7-1·7)	1·1 (0·7-1·6)	0·8 (0·5-1·2)	342·9 (289·0-401·6)	224·9 (189·2-264·8)	219·3 (184·5-258·0)	152·5 (127·7-180·9)	152·3 (77·6-256·8)	118·6 (50·8-218·5)	116·2 (49·1-217·2)	88·2 (28·9-194·1)	6,379·4 (4,474·8-9,007·0)	4,303·1 (3,061·6-6,086·9)	4,183·2 (2,978·4-5,922·9)	2,810·3 (2,006·1-3,943·4)
Cote d'Ivoire	3·0 (2·2-3·9)	1·6 (1·3-2·0)	1·4 (1·2-1·8)	1·1 (0·8-1·4)	376·9 (317·5-444·5)	244·9 (206·9-288·4)	239·4 (202·1-281·7)	166·8 (140·1-197·7)	357·0 (295·6-444·4)	208·3 (164·1-262·9)	188·3 (146·5-237·5)	159·8 (120·1-203·1)	6,132·1 (4,339·8-8,769·2)	4,568·3 (3,251·8-6,473·1)	4,487·1 (3,197·6-6,322·0)	3,479·5 (2,517·2-4,730·5)
The Gambia	1·7 (1·1-2·5)	0·7 (0·4-1·1)	0·7 (0·3-1·1)	0·5 (0·2-0·7)	234·7 (205·2-264·0)	184·5 (159·9-209·1)	181·0 (157·0-205·2)	136·3 (117·9-155·1)	189·0 (119·8-263·9)	48·1 (29·6-78·3)	46·9 (28·6-77·2)	32·4 (17·4-65·3)	3,686·9 (2,645·9-5,160·5)	2,502·6 (1,790·7-3,447·3)	2,468·4 (1,765·6-3,412·5)	2,013·5 (1,449·3-2,792·1)
Ghana	1·9 (1·3-2·5)	0·6 (0·4-0·9)	0·6 (0·3-1·0)	0·5 (0·2-0·7)	430·6 (357·9-518·1)	259·8 (217·0-312·5)	251·4 (210·3-302·5)	156·6 (130·6-190·3)	323·9 (250·1-402·0)	248·4 (186·7-303·8)	240·8 (180·5-295·0)	180·1 (134·2-228·9)	4,846·3 (3,529·3-6,701·8)	3,272·5 (2,395·5-4,479·0)	3,133·2 (2,294·8-4,270·6)	2,261·5 (1,645·8-3,151·2)
Guinea	1·2 (0·9-1·6)	0·7 (0·4-1·0)	0·6 (0·3-1·0)	0·4 (0·2-0·8)	282·5 (237·7-332·0)	257·1 (215·6-303·4)	254·4 (213·2-300·6)	236·9 (197·4-280·1)	314·1 (239·0-394·3)	291·6 (227·3-358·3)	288·3 (225·9-354·1)	266·7 (208·3-331·4)	6,371·0 (4,503·2-8,950·9)	4,140·8 (2,948·3-5,818·6)	4,013·0 (2,852·4-5,626·0)	2,712·1 (1,911·1-3,757·9)
Guinea-Bissau	2·9 (2·1-4·0)	1·5 (0·7-2·4)	1·3 (0·5-2·3)	0·9 (0·4-1·7)	407·0 (343·7-479·7)	414·7 (347·4-490·7)	410·0 (343·6-484·9)	386·1 (319·7-464·3)	256·4 (162·6-375·7)	67·3 (25·9-145·3)	65·0 (23·3-148·6)	37·7 (4·9-131·8)	8,905·1 (6,347·6-12,760·9)	5,045·0 (3,625·0-7,102·5)	4,887·8 (3,512·7-6,887·9)	3,103·9 (2,224·8-4,237·7)
Liberia	1·9 (1·4-2·7)	0·6 (0·4-0·9)	0·6 (0·3-0·9)	0·4 (0·2-0·7)	351·0 (295·7-415·5)	231·5 (194·0-274·3)	225·3 (188·8-266·8)	153·5 (128·3-182·9)	310·1 (200·8-419·2)	208·0 (134·0-288·5)	199·2 (126·1-281·6)	144·1 (101·8-194·5)	5,448·7 (3,843·4-7,692·2)	3,524·8 (2,499·3-5,026·3)	3,448·2 (2,444·5-4,905·4)	2,525·3 (1,773·3-3,509·4)
Mali	1·0 (0·7-1·3)	0·6 (0·4-0·7)	0·6 (0·4-0·7)	0·4 (0·3-0·5)	154·2 (128·9-182·4)	86·0 (72·0-101·1)	83·8 (70·2-98·5)	58·0 (48·5-68·4)	307·8 (235·0-390·4)	306·7 (237·4-388·8)	316·7 (245·3-401·4)	389·2 (295·3-508·0)	5,947·2 (4,159·2-8,216·4)	3,296·1 (2,325·8-4,526·9)	3,184·9 (2,243·7-4,374·0)	1,937·2 (1,392·3-2,621·1)
Mauritania	0·4 (0·05-1·2)	0·1 (0·01-0·6)	0·1 (0·01-0·6)	0·2 (0·01-0·6)	170·3 (143·3-201·8)	107·1 (89·9-127·6)	105·2 (88·2-125·3)	80·7 (66·6-96·8)	64·0 (32·5-107·3)	34·3 (19·0-52·8)	33·7 (18·5-51·8)	26·1 (15·6-41·2)	4,183·8 (3,019·1-5,806·0)	2,280·4 (1,686·7-3,046·5)	2,212·3 (1,636·7-2,950·2)	1,407·4 (1,044·5-1,855·7)
Niger	1·1 (0·8-1·4)	0·2 (0·1-0·3)	0·2 (0·1-0·3)	0·2 (0·09-0·2)	235·5 (196·7-277·7)	161·9 (135·4-190·5)	159·6 (133·5-187·8)	127·3 (106·7-150·0)	244·9 (129·9-374·3)	272·1 (162·2-398·0)	267·6 (157·7-395·2)	276·8 (159·5-412·8)	6,241·2 (4,441·1-8,515·8)	3,957·1 (2,792·5-5,507·4)	3,886·1 (2,745·5-5,427·6)	2,956·5 (2,086·8-4,227·7)
Nigeria	2·7 (1·9-3·6)	1·2 (0·9-1·6)	1·1 (0·7-1·5)	0·8 (0·5-1·1)	473·9 (399·8-563·3)	250·7 (211·9-298·1)	242·4 (205·2-287·6)	147·2 (124·7-175·1)	296·7 (239·2-378·8)	244·1 (195·4-315·1)	239·3 (189·5-310·8)	181·4 (134·2-255·5)	5,259·5 (3,980·5-6,821·3)	2,676·1 (2,048·4-3,369·0)	2,606·6 (2,000·2-3,278·7)	1,779·5 (1,376·5-2,270·7)
Sao Tome and Principe	0·7 (0·09-2·3)	0·1 (0·01-0·5)	0·1 (0·01-0·5)	0·09 (0·006-0·3)	166·2 (137·8-199·5)	111·6 (93·0-134·0)	109·4 (91·2-131·5)	80·6 (66·5-97·4)	169·1 (69·3-315·9)	12·2 (11·3-12·8)	9·4 (9·4-9·4)	0·5 (0·06-1·6)	3,550·9 (2,575·8-4,894·8)	1,827·8 (1,345·7-2,435·9)	1,768·8 (1,302·2-2,352·0)	1,093·4 (805·3-1,452·7)
Senegal	0·6 (0·4-0·8)	0·2 (0·1-0·3)	0·2 (0·1-0·3)	0·3 (0·1-0·4)	306·4 (257·2-362·4)	207·6 (174·2-246·9)	204·1 (171·2-242·9)	159·0 (132·1-190·2)	193·5 (151·6-246·0)	59·8 (45·5-77·5)	55·5 (42·0-71·9)	19·3 (13·3-27·3)	7,316·2 (5,196·9-10,626·1)	3,993·9 (2,901·8-5,603·0)	3,927·6 (2,851·8-5,500·2)	3,020·3 (2,118·3-4,286·8)

Location	**Indicator 3.3.5:** Age-standardised prevalence* of the sum of 15 neglected tropical diseases (NTDs) (%) **Prevalence estimates reported here may exceed 100% as they reflect the sum of prevalent cases of 15 NTDs.*				**Indicator 3.4.1:** Age-standardised death rate due to cardiovascular disease, cancer, diabetes, and chronic respiratory disease in populations aged 30-70 (per 100,000 population)				**Indicator 3.4.2:** Age-standardised death rate due to self-harm (per 100,000 population)				**Indicator 3.5.2:** Risk-weighted prevalence of alcohol consumption, as measured by the summary exposure value (SEV) for alcohol use (%)			
	**2000**	**2015**	**2016**	**2030**	**2000**	**2015**	**2016**	**2030**	**2000**	**2015**	**2016**	**2030**	**2000**	**2015**	**2016**	**2030**
Bangladesh	101·8<br>(94·3-110·1)	73·2<br>(63·4-85·2)	71·4<br>(61·9-83·2)	50·0<br>(43·1-59·0)	512·0<br>(471·5-554·8)	452·3<br>(403·6-504·2)	448·0<br>(398·1-503·7)	386·5<br>(291·6-504·6)	10·1<br>(8·9-11·7)	7·8<br>(6·6-9·0)	7·6<br>(6·3-8·9)	6·3<br>(4·2-8·9)	0·2<br>(0·2-0·2)	0·2<br>(0·2-0·3)	0·2<br>(0·2-0·3)	0·2<br>(0·2-0·3)
Bhutan	19·4<br>(15·0-25·1)	19·4<br>(14·7-25·6)	19·4<br>(14·7-25·6)	19·3<br>(14·5-25·5)	471·7<br>(417·5-532·3)	371·4<br>(316·6-436·3)	367·9<br>(311·5-433·3)	322·1<br>(228·7-449·0)	8·4<br>(6·7-10·4)	7·0<br>(5·4-8·9)	7·0<br>(5·4-8·9)	6·3<br>(3·8-9·8)	5·9<br>(3·8-8·0)	3·9<br>(1·9-6·4)	3·9<br>(1·9-6·4)	3·6<br>(1·5-6·2)
India	32·5<br>(28·0-37·9)	31·2<br>(27·1-35·9)	31·0<br>(27·0-35·7)	28·3<br>(24·6-32·6)	618·8<br>(571·7-649·0)	542·7<br>(515·5-565·1)	534·2<br>(508·1-556·6)	485·0<br>(445·3-523·2)	20·8<br>(17·4-22·3)	18·2<br>(15·2-19·7)	17·9<br>(15·0-19·4)	15·7<br>(13·0-19·1)	3·0<br>(2·4-3·6)	3·7<br>(3·0-4·4)	3·8<br>(3·1-4·5)	4·6<br>(3·6-5·6)
Nepal	94·3<br>(84·3-106·1)	66·3<br>(57·7-76·9)	64·5<br>(56·1-74·9)	43·8<br>(37·9-51·0)	589·5<br>(538·4-635·8)	502·8<br>(459·1-547·2)	497·0<br>(453·5-541·0)	423·2<br>(360·2-497·1)	14·4<br>(10·3-18·1)	11·4<br>(8·6-13·5)	11·1<br>(8·5-13·1)	8·3<br>(5·8-11·1)	2·2<br>(1·6-2·9)	3·0<br>(1·8-4·8)	3·0<br>(1·8-4·9)	3·0<br>(1·2-5·9)
Pakistan	17·3<br>(14·2-21·2)	17·0<br>(13·5-21·3)	17·0<br>(13·5-21·3)	16·8<br>(13·3-21·2)	722·1<br>(659·5-782·3)	631·6<br>(545·8-736·3)	624·4<br>(537·8-727·0)	541·0<br>(385·2-715·2)	4·8<br>(4·1-5·6)	3·9<br>(3·2-4·7)	3·8<br>(3·1-4·7)	3·1<br>(2·0-4·6)	0·2<br>(0·1-0·2)	0·1<br>(0·0-0·2)	0·1<br>(0·0-0·2)	0·1<br>(0·0-0·2)
**Southern Sub-Saharan Africa**																
Botswana	64·7<br>(56·9-72·4)	66·6<br>(58·3-75·0)	67·1<br>(58·7-75·6)	74·0<br>(64·1-83·9)	792·8<br>(347·6-1,681·7)	548·7<br>(236·6-828·8)	548·9<br>(250·3-816·0)	442·0<br>(83·4-1,068·1)	23·5<br>(9·9-48·3)	17·7<br>(7·6-28·1)	17·6<br>(8·0-27·6)	14·1<br>(3·1-35·7)	14·4<br>(11·4-17·4)	14·8<br>(9·4-19·5)	14·7<br>(9·2-19·4)	12·6<br>(6·8-17·7)
Lesotho	82·3<br>(69·7-96·2)	82·3<br>(69·4-96·8)	82·2<br>(69·4-96·7)	81·6<br>(68·9-96·0)	781·2<br>(540·9-962·7)	889·8<br>(664·1-1,136·7)	869·5<br>(647·5-1,132·4)	901·9<br>(461·8-1,497·2)	32·4<br>(20·8-43·4)	39·4<br>(25·9-55·6)	39·0<br>(25·5-55·7)	42·5<br>(19·3-76·8)	17·9<br>(13·8-21·5)	18·0<br>(12·2-22·4)	17·9<br>(12·0-22·3)	16·2<br>(9·6-21·6)
Namibia	36·4<br>(27·5-45·6)	36·7<br>(27·4-46·2)	36·8<br>(27·5-46·3)	38·3<br>(28·2-48·6)	714·2<br>(452·5-928·8)	432·3<br>(288·7-570·1)	425·2<br>(279·9-559·7)	275·1<br>(140·8-473·4)	18·2<br>(11·8-24·8)	13·1<br>(9·1-18·2)	13·0<br>(9·0-18·0)	11·3<br>(5·5-19·0)	8·4<br>(4·7-13·2)	18·2<br>(9·7-25·6)	18·1<br>(9·5-25·6)	16·7<br>(6·1-25·8)
South Africa	88·8<br>(74·4-105·1)	84·2<br>(74·8-94·7)	83·5<br>(74·2-93·9)	74·4<br>(66·0-83·7)	629·1<br>(588·5-669·8)	481·1<br>(456·1-507·7)	477·5<br>(449·6-507·0)	417·7<br>(351·2-495·2)	21·1<br>(16·4-24·3)	13·9<br>(11·8-15·7)	14·2<br>(12·0-16·1)	10·1<br>(7·1-13·7)	12·3<br>(9·6-14·9)	12·4<br>(9·8-15·1)	12·4<br>(9·8-15·2)	12·5<br>(9·8-15·3)
Swaziland	108·7<br>(95·2-123·7)	106·5<br>(94·5-119·2)	106·4<br>(94·4-119·0)	105·1<br>(93·7-117·3)	731·8<br>(461·6-1,191·5)	659·8<br>(389·7-971·7)	644·2<br>(384·2-941·0)	461·3<br>(149·5-1,078·4)	19·7<br>(11·9-31·7)	19·0<br>(11·7-27·6)	18·7<br>(11·7-27·0)	15·4<br>(5·9-35·9)	11·2<br>(9·2-13·1)	8·7<br>(4·9-12·0)	8·6<br>(4·7-12·0)	7·4<br>(3·1-11·2)
Zimbabwe	69·7<br>(61·8-78·5)	59·0<br>(52·2-67·0)	57·7<br>(50·9-65·6)	42·1<br>(36·1-49·2)	631·1<br>(424·1-905·1)	624·6<br>(485·8-781·8)	618·1<br>(476·7-777·7)	515·5<br>(307·3-851·4)	24·8<br>(16·8-35·8)	27·8<br>(21·0-37·3)	27·8<br>(21·1-37·2)	27·3<br>(14·7-46·1)	8·1<br>(6·1-10·5)	8·5<br>(4·7-13·1)	8·5<br>(4·6-13·3)	9·0<br>(3·4-17·0)
**Western Sub-Saharan Africa**																
Benin	97·4<br>(92·3-103·2)	61·1<br>(57·1-66·0)	57·4<br>(53·6-62·2)	24·1<br>(21·5-27·2)	467·9<br>(417·7-518·6)	467·5<br>(417·6-515·9)	465·1<br>(415·7-511·8)	457·2<br>(377·7-553·5)	13·9<br>(10·4-17·1)	13·9<br>(11·1-16·5)	13·9<br>(11·1-16·4)	14·0<br>(10·6-18·4)	4·2<br>(2·7-6·0)	4·7<br>(2·5-8·1)	4·8<br>(2·5-8·3)	5·3<br>(2·2-10·7)
Burkina Faso	99·0<br>(92·2-105·2)	17·6<br>(15·2-20·7)	15·7<br>(13·4-18·7)	2·1<br>(1·6-2·7)	482·1<br>(427·8-541·7)	479·9<br>(439·4-517·8)	477·9<br>(435·4-516·3)	475·1<br>(397·3-561·0)	12·8<br>(10·9-14·9)	12·8<br>(11·0-14·9)	12·9<br>(11·1-15·1)	13·4<br>(10·1-18·1)	15·2<br>(10·5-20·0)	12·7<br>(6·5-20·5)	12·5<br>(6·2-20·4)	10·2<br>(3·7-19·7)
Cameroon	191·0<br>(167·8-217·4)	71·5<br>(63·4-80·9)	65·0<br>(57·5-73·8)	16·9<br>(14·6-19·4)	532·8<br>(441·7-633·4)	500·9<br>(399·4-621·2)	496·9<br>(393·1-617·7)	468·4<br>(299·7-671·9)	16·4<br>(12·5-20·5)	17·0<br>(13·3-21·2)	16·9<br>(13·0-21·2)	17·0<br>(11·1-24·9)	9·7<br>(7·6-11·9)	9·8<br>(7·2-12·6)	9·6<br>(7·1-12·4)	8·1<br>(6·0-10·6)
Cape Verde	2·0<br>(1·6-2·4)	1·7<br>(1·3-2·2)	1·7<br>(1·2-2·2)	1·3<br>(0·9-1·7)	407·9<br>(334·2-487·2)	379·4<br>(325·1-435·8)	376·0<br>(316·7-433·4)	348·2<br>(227·2-483·4)	18·4<br>(14·2-23·6)	20·3<br>(15·9-24·4)	20·1<br>(15·6-24·5)	20·7<br>(12·0-33·8)	8·9<br>(7·1-10·8)	8·6<br>(6·6-11·1)	8·5<br>(6·5-11·0)	7·1<br>(5·3-9·4)
Chad	66·3<br>(59·0-74·6)	64·1<br>(56·7-72·5)	64·3<br>(56·9-72·6)	66·0<br>(58·9-74·1)	484·0<br>(420·8-547·5)	443·9<br>(382·4-509·8)	441·3<br>(380·5-508·4)	406·3<br>(307·5-519·1)	11·7<br>(7·8-15·6)	10·2<br>(6·9-14·7)	10·0<br>(6·9-14·5)	7·6<br>(4·8-12·7)	5·4<br>(4·3-6·6)	6·2<br>(4·0-8·7)	6·2<br>(3·9-8·7)	5·6<br>(2·5-9·6)
Cote d'Ivoire	105·5<br>(98·1-113·1)	85·8<br>(79·6-92·4)	83·4<br>(77·3-89·8)	56·2<br>(51·3-61·7)	638·5<br>(550·0-747·0)	592·3<br>(520·2-679·5)	586·8<br>(515·6-675·6)	536·8<br>(416·1-697·4)	16·6<br>(13·7-20·0)	16·5<br>(13·7-19·8)	16·6<br>(13·8-19·9)	17·0<br>(12·3-23·5)	10·3<br>(6·5-14·5)	8·7<br>(4·0-14·2)	8·5<br>(3·8-14·1)	7·0<br>(2·1-13·3)
The Gambia	62·9<br>(58·1-68·3)	61·4<br>(57·8-65·4)	61·1<br>(57·7-64·8)	56·6<br>(52·0-60·8)	445·6<br>(394·4-501·1)	427·4<br>(372·7-482·7)	425·8<br>(369·6-482·8)	413·7<br>(309·4-526·4)	8·0<br>(6·1-11·8)	7·5<br>(5·5-11·5)	7·5<br>(5·5-11·5)	7·5<br>(4·7-13·2)	4·8<br>(3·0-7·0)	4·8<br>(2·2-8·6)	4·7<br>(2·1-8·5)	3·6<br>(1·3-7·6)
Ghana	45·8<br>(42·1-49·8)	26·0<br>(23·2-29·1)	24·5<br>(21·7-27·6)	10·8<br>(8·8-13·2)	520·9<br>(435·2-605·7)	469·4<br>(402·9-528·2)	462·0<br>(395·7-520·0)	395·8<br>(292·8-499·6)	8·3<br>(6·7-9·6)	8·5<br>(6·8-10·0)	8·4<br>(6·8-10·0)	7·9<br>(5·3-11·3)	13·1<br>(8·8-16·8)	15·3<br>(9·8-19·2)	15·3<br>(9·7-19·3)	16·5<br>(8·8-22·4)
Guinea	86·7<br>(77·6-97·6)	81·8<br>(72·1-92·7)	81·4<br>(71·7-92·3)	76·0<br>(66·1-86·9)	517·3<br>(461·5-576·6)	551·6<br>(470·6-636·6)	551·6<br>(468·5-638·6)	563·8<br>(408·5-746·0)	9·0<br>(6·5-12·9)	9·6<br>(6·6-14·0)	9·7<br>(6·6-14·1)	9·8<br>(5·8-15·9)	1·0<br>(0·6-1·5)	0·9<br>(0·5-1·5)	0·9<br>(0·5-1·5)	1·1<br>(0·5-1·9)
Guinea-Bissau	89·8<br>(80·3-98·8)	52·5<br>(45·0-60·0)	50·3<br>(42·9-57·7)	27·1<br>(21·9-32·8)	756·4<br>(653·2-851·0)	732·7<br>(634·0-826·4)	726·4<br>(628·1-821·7)	676·9<br>(514·8-864·2)	12·4<br>(9·4-16·2)	12·6<br>(9·1-18·1)	12·5<br>(9·1-18·2)	12·4<br>(7·6-20·8)	7·2<br>(4·8-10·0)	7·1<br>(3·5-11·8)	7·1<br>(3·5-11·8)	6·8<br>(2·8-12·3)
Liberia	152·6<br>(135·5-171·0)	128·5<br>(110·5-148·8)	125·9<br>(108·0-146·5)	95·1<br>(78·3-116·8)	434·9<br>(371·4-496·2)	461·4<br>(410·2-506·2)	459·5<br>(409·8-503·3)	471·0<br>(384·6-575·8)	12·7<br>(9·9-15·4)	16·5<br>(13·3-19·6)	16·5<br>(13·3-19·6)	17·2<br>(11·6-23·0)	12·6<br>(9·4-15·8)	9·8<br>(5·5-15·2)	9·8<br>(5·4-15·5)	10·1<br>(3·7-19·7)
Mali	99·5<br>(92·6-107·1)	24·2<br>(21·8-26·9)	21·2<br>(19·0-23·7)	3·3<br>(2·7-3·9)	559·3<br>(489·7-624·3)	464·2<br>(387·1-555·0)	463·9<br>(383·1-562·4)	410·1<br>(266·9-626·7)	6·2<br>(4·3-9·5)	5·9<br>(3·9-9·9)	6·0<br>(3·9-10·1)	6·1<br>(3·3-12·2)	2·7<br>(1·9-3·3)	2·6<br>(1·5-3·6)	2·6<br>(1·4-3·6)	2·5<br>(1·2-3·7)
Mauritania	34·6<br>(24·0-45·2)	29·8<br>(20·7-39·6)	29·2<br>(20·3-38·9)	22·1<br>(15·4-30·1)	446·0<br>(341·7-515·7)	363·4<br>(278·6-442·4)	362·5<br>(279·1-443·8)	330·7<br>(196·6-508·9)	6·0<br>(3·8-10·3)	5·6<br>(3·3-10·5)	5·6<br>(3·3-10·6)	6·0<br>(2·7-12·9)	0·3<br>(0·2-0·4)	0·2<br>(0·1-0·4)	0·2<br>(0·1-0·4)	0·4<br>(0·1-0·9)
Niger	46·6<br>(37·6-58·6)	11·1<br>(9·3-13·4)	9·5<br>(8·0-11·4)	1·0<br>(0·8-1·3)	418·7<br>(362·3-483·3)	438·2<br>(367·5-522·4)	439·7<br>(366·0-524·8)	459·3<br>(320·8-643·0)	7·4<br>(5·0-11·0)	7·9<br>(5·2-12·8)	7·9<br>(5·2-12·9)	8·5<br>(4·8-15·4)	1·1<br>(0·6-1·7)	1·1<br>(0·5-1·9)	1·1<br>(0·5-1·9)	1·1<br>(0·5-2·2)
Nigeria	86·5<br>(80·0-93·8)	71·7<br>(65·8-78·9)	70·2<br>(64·3-77·4)	52·2<br>(46·6-59·0)	342·4<br>(259·3-425·0)	255·1<br>(201·0-312·4)	257·0<br>(202·6-312·0)	199·7<br>(126·6-289·2)	9·3<br>(5·9-13·5)	7·0<br>(4·4-11·6)	7·0<br>(4·4-11·5)	6·0<br>(2·9-12·1)	17·3<br>(13·8-20·8)	12·9<br>(7·1-19·0)	12·5<br>(6·7-18·7)	8·3<br>(2·9-15·2)
Sao Tome and Principe	103·8<br>(80·9-121·6)	97·2<br>(79·4-114·2)	96·3<br>(79·2-113·1)	84·4<br>(71·6-99·7)	477·5<br>(340·6-545·9)	415·1<br>(338·5-482·0)	411·8<br>(342·8-478·2)	361·7<br>(247·6-501·1)	2·6<br>(1·5-3·4)	3·0<br>(2·1-4·0)	3·0<br>(2·2-4·0)	3·2<br>(1·9-5·3)	10·0<br>(7·6-12·5)	9·3<br>(7·1-11·7)	9·2<br>(7·0-11·6)	7·7<br>(5·8-9·9)
Senegal	67·5<br>(59·0-74·8)	36·4<br>(32·3-40·7)	33·3<br>(29·4-37·3)	9·5<br>(7·8-11·7)	502·0<br>(455·7-552·9)	515·0<br>(470·7-558·7)	512·2<br>(467·6-556·2)	491·6<br>(418·5-583·0)	12·7<br>(10·7-14·4)	13·7<br>(11·6-16·1)	13·6<br>(11·4-16·0)	14·2<br>(10·3-19·1)	1·1<br>(0·7-1·5)	0·9<br>(0·5-1·4)	0·9<br>(0·5-1·5)	1·0<br>(0·4-1·9)

Location	Indicator 3.6.1:<br>Age-standardised death rate due to road injuries (per 100,000 population)				Indicator 3.7.1:<br>Proportion of women of reproductive age (15-49 years) who have their need for family planning satisfied with modern contraception methods (%)				Indicator 3.7.2:<br>Number of livebirths per 1,000 women aged 10-14 years and women aged 15-19 years				Indicator 3.8.1:<br>Coverage of essential health services, as defined by the UHC index comprised of the coverage of 9 tracer interventions and risk-standardised death rates from 32 causes amenable to personal healthcare (scale of 0 to 100)			
	**2000**	**2015**	**2016**	**2030**	**2000**	**2015**	**2016**	**2030**	**2000**	**2015**	**2016**	**2030**	**2000**	**2015**	**2016**	**2030**
Bangladesh	9·5<br>(7·6-11·6)	14·9<br>(12·7-17·4)	14·3<br>(12·0-16·7)	17·6<br>(11·7-25·0)	63·2<br>(60·9-65·4)	74·1<br>(71·7-76·4)	74·5<br>(71·4-77·4)	80·7<br>(75·7-84·9)	49·5<br>(46·9-52·1)	37·8<br>(34·6-41·2)	37·2<br>(33·7-40·9)	28·0<br>(22·4-34·2)	39·4<br>(37·4-41·2)	53·9<br>(51·5-56·3)	54·7<br>(52·2-57·1)	67·4<br>(63·8-70·8)
Bhutan	15·6<br>(13·0-18·3)	11·1<br>(8·8-13·9)	10·9<br>(8·5-13·6)	8·0<br>(5·1-11·9)	58·1<br>(51·3-64·8)	74·4<br>(68·0-79·7)	75·0<br>(68·7-80·4)	85·1<br>(76·9-90·8)	47·7<br>(43·3-51·9)	11·2<br>(9·6-12·9)	9·8<br>(8·3-11·5)	1·7<br>(1·1-2·7)	42·5<br>(40·1-45·0)	55·3<br>(52·0-58·6)	55·9<br>(52·5-59·2)	65·7<br>(61·6-69·9)
India	21·0<br>(19·5-22·3)	21·8<br>(20·6-22·9)	21·3<br>(20·1-22·4)	21·9<br>(19·8-24·0)	67·3<br>(64·5-69·7)	75·2<br>(70·7-79·6)	75·0<br>(70·5-79·2)	78·2<br>(69·1-86·1)	31·1<br>(30·2-31·9)	9·7<br>(9·2-10·1)	8·6<br>(8·1-9·1)	2·8<br>(2·4-3·3)	37·3<br>(36·0-38·8)	49·1<br>(47·1-50·6)	49·9<br>(47·9-51·5)	61·4<br>(58·6-63·6)
Nepal	23·2<br>(18·9-29·6)	20·2<br>(15·8-25·9)	19·9<br>(15·7-25·5)	17·4<br>(12·4-23·5)	52·0<br>(49·5-54·5)	60·5<br>(57·8-63·3)	61·5<br>(58·2-65·0)	68·8<br>(59·4-77·0)	57·5<br>(45·9-69·8)	50·8<br>(39·5-63·6)	50·3<br>(37·5-64·7)	45·2<br>(22·9-82·1)	37·6<br>(35·6-39·6)	51·3<br>(48·5-54·0)	52·0<br>(49·2-54·7)	64·3<br>(60·5-67·4)
Pakistan	22·8<br>(18·7-29·9)	23·8<br>(18·7-32·3)	23·5<br>(18·4-31·9)	22·3<br>(14·3-33·8)	37·7<br>(32·4-43·3)	48·2<br>(45·5-51·0)	49·1<br>(45·2-52·8)	62·3<br>(54·3-70·3)	21·2<br>(19·5-22·8)	16·9<br>(13·9-20·0)	16·1<br>(13·1-19·5)	12·0<br>(7·6-18·0)	34·1<br>(32·4-35·9)	42·8<br>(40·1-45·4)	43·2<br>(40·5-45·9)	52·1<br>(48·2-56·2)
**Southern Sub-Saharan Africa**																
Botswana	28·1<br>(13·8-54·2)	21·4<br>(10·1-32·0)	21·3<br>(10·4-31·4)	16·9<br>(3·5-39·3)	69·3<br>(62·4-75·7)	83·2<br>(78·5-87·2)	83·4<br>(78·6-87·3)	91·5<br>(84·9-95·7)	31·2<br>(20·9-42·5)	15·0<br>(9·6-21·9)	13·8<br>(8·6-20·2)	5·8<br>(1·9-12·2)	46·2<br>(34·3-56·1)	56·9<br>(50·1-67·4)	57·5<br>(50·8-68·4)	65·1<br>(59·4-79·8)
Lesotho	42·5<br>(31·7-53·4)	51·1<br>(38·3-64·6)	50·3<br>(37·4-64·4)	53·3<br>(29·7-88·9)	52·0<br>(47·1-57·2)	78·9<br>(76·2-81·3)	79·1<br>(76·0-81·9)	93·1<br>(89·5-95·9)	40·1<br>(32·5-47·6)	43·6<br>(36·0-52·2)	44·4<br>(36·0-53·6)	52·4<br>(31·1-80·4)	39·1<br>(35·3-43·8)	41·9<br>(37·6-46·8)	42·2<br>(37·8-47·2)	48·2<br>(42·6-53·9)
Namibia	44·0<br>(30·9-56·8)	31·9<br>(23·0-40·1)	31·5<br>(22·3-39·7)	24·4<br>(12·6-38·2)	68·9<br>(66·4-71·3)	81·1<br>(78·5-83·5)	81·2<br>(78·1-84·2)	87·3<br>(82·4-91·1)	41·2<br>(37·4-45·4)	37·8<br>(33·9-41·8)	37·5<br>(33·3-41·7)	33·3<br>(25·0-42·9)	42·5<br>(37·5-48·4)	54·8<br>(50·2-61·0)	55·3<br>(50·7-61·7)	64·6<br>(59·3-72·7)
South Africa	54·8<br>(49·2-61·0)	39·2<br>(35·9-43·1)	39·7<br>(36·1-43·6)	33·5<br>(26·7-41·7)	79·1<br>(76·4-81·4)	85·8<br>(81·4-89·2)	85·8<br>(81·5-89·3)	90·4<br>(83·5-95·0)	32·9<br>(29·6-36·1)	22·7<br>(20·5-25·1)	22·0<br>(19·3-24·8)	14·8<br>(11·5-19·6)	44·9<br>(42·9-46·7)	52·6<br>(51·0-54·2)	53·0<br>(51·5-54·7)	61·3<br>(59·4-63·4)
Swaziland	49·2<br>(32·8-74·8)	40·4<br>(26·9-57·1)	39·6<br>(26·6-55·2)	26·7<br>(11·4-61·0)	51·5<br>(45·2-57·8)	83·4<br>(80·4-86·1)	83·4<br>(79·9-86·3)	93·2<br>(88·5-96·7)	47·5<br>(40·4-54·6)	38·7<br>(33·8-43·7)	35·3<br>(30·7-40·2)	32·4<br>(23·4-43·3)	43·1<br>(35·9-48·0)	49·4<br>(43·6-56·1)	50·2<br>(44·4-57·2)	63·3<br>(57·1-72·3)
Zimbabwe	40·6<br>(28·3-54·9)	36·2<br>(29·1-44·8)	35·9<br>(28·7-44·8)	30·2<br>(19·0-46·4)	72·4<br>(70·0-74·5)	84·5<br>(82·9-86·0)	84·8<br>(82·8-86·5)	92·3<br>(89·9-94·3)	50·0<br>(46·5-53·5)	54·0<br>(50·2-57·9)	53·5<br>(49·5-57·7)	50·1<br>(40·5-61·1)	41·2<br>(36·3-45·8)	44·6<br>(41·2-48·3)	45·7<br>(42·3-49·2)	59·8<br>(56·8-62·8)
**Western Sub-Saharan Africa**																
Benin	27·5<br>(22·4-37·0)	23·5<br>(19·0-31·8)	23·3<br>(18·7-31·6)	20·8<br>(15·0-31·0)	14·7<br>(13·1-16·3)	24·7<br>(20·9-29·1)	25·4<br>(20·9-30·7)	36·9<br>(25·5-50·3)	51·4<br>(48·4-54·3)	42·6<br>(39·4-45·7)	41·9<br>(38·5-45·2)	32·5<br>(26·1-39·2)	39·2<br>(37·2-41·3)	45·6<br>(43·6-47·5)	46·2<br>(44·1-48·3)	52·5<br>(49·9-55·1)
Burkina Faso	25·6<br>(22·7-28·5)	23·8<br>(21·4-26·4)	23·6<br>(21·2-26·3)	21·7<br>(17·2-27·5)	20·2<br>(17·7-22·9)	47·1<br>(45·0-49·2)	49·7<br>(47·4-52·0)	76·1<br>(71·2-80·7)	63·6<br>(50·4-75·2)	49·6<br>(35·4-61·8)	48·9<br>(34·6-62·3)	38·6<br>(17·9-64·1)	35·9<br>(33·9-38·1)	46·0<br>(44·0-48·3)	46·2<br>(44·1-48·5)	51·8<br>(48·6-55·1)
Cameroon	25·2<br>(20·9-29·6)	23·4<br>(18·9-28·1)	23·2<br>(18·5-28·0)	21·1<br>(14·1-29·4)	19·7<br>(17·5-22·1)	41·9<br>(38·7-45·0)	42·3<br>(38·1-46·3)	55·7<br>(42·3-68·3)	65·5<br>(61·6-69·2)	49·2<br>(46·4-52·0)	47·5<br>(44·7-50·4)	37·4<br>(32·4-42·3)	37·1<br>(34·2-39·9)	44·7<br>(41·5-48·0)	45·2<br>(42·0-48·6)	52·6<br>(48·1-57·1)
Cape Verde	12·4<br>(10·1-15·4)	11·3<br>(9·4-13·2)	11·1<br>(9·3-13·2)	9·5<br>(6·0-14·0)	79·1<br>(75·4-82·3)	77·6<br>(71·4-82·8)	78·1<br>(72·1-83·2)	85·4<br>(74·2-92·8)	51·6<br>(48·5-54·8)	53·3<br>(47·5-59·9)	52·5<br>(46·8-59·4)	43·9<br>(33·1-58·1)	51·5<br>(48·6-54·5)	61·3<br>(58·7-64·2)	61·9<br>(59·2-64·8)	70·8<br>(67·6-74·0)
Chad	21·7<br>(18·7-25·0)	19·8<br>(16·8-22·9)	19·6<br>(16·7-22·7)	16·8<br>(12·5-22·0)	5·5<br>(4·3-6·7)	14·7<br>(12·8-16·7)	14·9<br>(12·6-17·4)	19·6<br>(13·2-26·9)	82·5<br>(81·5-83·3)	58·8<br>(58·1-59·6)	56·5<br>(55·7-57·2)	42·2<br>(40·5-43·8)	28·5<br>(26·6-30·8)	36·2<br>(34·0-38·3)	36·8<br>(34·5-39·0)	45·1<br>(41·5-48·3)
Cote d'Ivoire	30·4<br>(26·1-36·1)	26·4<br>(23·0-30·8)	26·3<br>(22·9-30·6)	24·0<br>(18·2-32·1)	21·8<br>(18·5-25·2)	33·0<br>(28·2-38·8)	33·6<br>(28·2-40·1)	43·0<br>(30·7-58·8)	56·5<br>(48·3-64·3)	62·3<br>(50·3-74·6)	63·2<br>(50·5-76·1)	68·7<br>(40·9-106·0)	36·4<br>(34·0-38·8)	42·6<br>(40·2-44·8)	43·1<br>(40·6-45·3)	49·2<br>(45·9-52·2)
The Gambia	18·4<br>(16·1-20·8)	16·4<br>(14·3-18·8)	16·3<br>(14·1-18·7)	14·9<br>(11·0-19·7)	19·8<br>(15·6-24·8)	28·3<br>(24·8-32·0)	28·9<br>(24·7-33·4)	39·6<br>(27·9-51·4)	63·1<br>(55·2-70·4)	50·1<br>(43·3-56·5)	49·8<br>(42·5-56·5)	42·5<br>(29·4-58·4)	45·2<br>(43·0-47·3)	50·2<br>(47·6-52·5)	50·6<br>(48·0-52·9)	55·3<br>(52·4-58·4)
Ghana	20·9<br>(18·0-23·7)	20·4<br>(17·9-23·1)	20·1<br>(17·6-22·8)	19·2<br>(14·4-25·3)	27·2<br>(24·1-30·5)	39·1<br>(36·8-41·4)	39·7<br>(36·4-43·0)	53·2<br>(44·5-61·4)	36·3<br>(32·0-40·4)	33·2<br>(29·1-37·3)	32·8<br>(28·5-37·3)	27·7<br>(19·6-37·8)	42·6<br>(40·4-45·0)	51·5<br>(49·2-54·0)	52·2<br>(49·8-54·8)	61·0<br>(58·0-64·0)
Guinea	21·2<br>(18·3-24·1)	19·2<br>(16·3-22·5)	19·1<br>(16·1-22·2)	17·6<br>(13·0-23·2)	16·6<br>(14·6-18·7)	23·1<br>(19·3-27·3)	23·7<br>(19·5-28·6)	32·7<br>(22·6-45·0)	71·0<br>(71·0-71·0)	66·1<br>(66·0-66·1)	65·8<br>(65·8-65·9)	61·7<br>(61·6-61·8)	33·6<br>(31·6-35·6)	39·3<br>(36·7-41·5)	39·7<br>(37·1-42·0)	44·1<br>(40·5-47·8)
Guinea-Bissau	31·5<br>(26·5-36·9)	26·6<br>(21·9-31·4)	26·3<br>(21·7-31·2)	22·2<br>(16·3-29·5)	12·2<br>(9·4-15·8)	46·6<br>(41·3-51·9)	46·4<br>(40·7-52·1)	78·5<br>(63·8-90·2)	58·0<br>(57·3-58·9)	42·0<br>(41·4-42·6)	40·6<br>(40·0-41·2)	24·5<br>(23·6-25·5)	30·6<br>(28·1-33·3)	37·9<br>(35·4-40·7)	38·4<br>(35·9-41·1)	45·5<br>(42·2-48·9)
Liberia	16·9<br>(14·3-19·4)	15·1<br>(13·0-17·0)	15·0<br>(12·9-16·9)	13·7<br>(10·4-17·6)	19·2<br>(15·1-24·0)	41·9<br>(37·9-45·7)	42·2<br>(37·5-47·1)	69·7<br>(59·3-79·7)	72·1<br>(66·9-77·2)	49·7<br>(44·8-54·5)	48·9<br>(43·8-53·9)	35·2<br>(25·3-45·0)	36·5<br>(34·1-38·8)	45·8<br>(43·8-47·9)	46·3<br>(44·3-48·4)	52·7<br>(49·9-55·6)
Mali	18·5<br>(15·9-21·2)	15·2<br>(12·4-18·2)	15·2<br>(12·3-18·2)	13·9<br>(9·3-20·2)	16·3<br>(14·8-18·0)	29·7<br>(25·8-33·7)	30·0<br>(25·3-34·9)	41·4<br>(32·0-53·7)	84·9<br>(82·9-86·9)	76·2<br>(73·7-78·5)	76·2<br>(73·6-78·6)	71·0<br>(65·4-77·0)	33·5<br>(31·4-35·9)	43·6<br>(40·6-46·8)	44·3<br>(41·2-47·6)	52·0<br>(47·8-56·5)
Mauritania	16·0<br>(12·3-19·5)	12·0<br>(8·8-14·8)	12·0<br>(8·8-14·7)	10·0<br>(6·1-14·7)	13·0<br>(11·1-15·2)	13·6<br>(10·9-16·8)	14·7<br>(11·5-18·3)	15·2<br>(9·2-22·8)	37·7<br>(37·1-38·2)	30·3<br>(29·5-31·1)	29·9<br>(29·1-30·7)	24·9<br>(23·5-26·3)	40·0<br>(37·3-44·2)	49·9<br>(46·3-54·1)	50·5<br>(46·9-54·6)	59·0<br>(54·3-63·9)
Niger	17·3<br>(14·7-20·3)	15·5<br>(12·9-18·5)	15·5<br>(12·7-18·5)	14·7<br>(10·1-20·0)	14·3<br>(12·2-16·9)	39·0<br>(36·0-42·2)	42·2<br>(38·9-45·5)	72·1<br>(64·7-79·0)	91·1<br>(88·1-94·0)	79·8<br>(75·7-83·7)	79·1<br>(75·1-83·0)	69·7<br>(62·6-77·3)	31·4<br>(29·3-33·6)	42·3<br>(39·6-45·1)	42·6<br>(39·9-45·4)	50·0<br>(46·1-53·7)
Nigeria	17·1<br>(12·5-21·4)	13·9<br>(10·6-17·3)	13·8<br>(10·5-17·3)	11·7<br>(7·4-17·7)	24·4<br>(20·6-28·8)	34·9<br>(32·6-37·2)	34·3<br>(31·5-37·1)	48·8<br>(40·6-56·3)	58·8<br>(51·2-65·9)	47·1<br>(41·3-52·4)	46·2<br>(40·1-52·1)	38·5<br>(27·4-50·3)	37·2<br>(34·0-41·0)	47·7<br>(44·7-51·3)	48·1<br>(45·0-51·7)	56·4<br>(51·8-61·2)
Sao Tome and Principe	15·0<br>(10·2-18·0)	15·1<br>(11·3-18·7)	15·0<br>(11·5-18·7)	13·7<br>(9·0-20·9)	44·4<br>(37·1-51·3)	52·7<br>(49·3-56·1)	53·3<br>(49·0-57·6)	61·6<br>(51·8-71·0)	46·7<br>(45·7-47·7)	42·1<br>(41·2-43·0)	41·8<br>(40·9-42·7)	35·2<br>(33·2-37·3)	46·1<br>(43·4-51·9)	54·8<br>(51·7-58·2)	55·1<br>(52·1-58·3)	61·4<br>(57·8-64·4)
Senegal	20·8<br>(17·4-24·0)	19·5<br>(16·2-22·5)	19·7<br>(16·0-22·8)	18·3<br>(13·5-23·2)	16·1<br>(13·7-18·7)	43·5<br>(41·3-45·7)	43·8<br>(40·8-46·8)	69·7<br>(63·2-75·1)	45·7<br>(43·1-48·2)	35·0<br>(32·6-37·3)	33·8<br>(31·3-36·3)	23·8<br>(19·3-28·9)	37·4<br>(35·7-39·2)	44·2<br>(42·4-46·1)	44·8<br>(42·9-46·6)	51·8<br>(49·0-54·2)

Location	Indicator 3.9.1: Age-standardised death rate attributable to household air pollution and ambient air pollution (per 100,000 population)				Indicator 3.9.2: Age-standardised death rate attributable to unsafe water, sanitation, and hygiene (WaSH) (per 100,000 population)				Indicator 3.9.3: Age-standardised death rate due to unintentional poisonings (per 100,000 population)				Indicator 3.a.1: Age-standardised prevalence of daily smoking in populations aged 10 and older (%)			
	**2000**	**2015**	**2016**	**2030**	**2000**	**2015**	**2016**	**2030**	**2000**	**2015**	**2016**	**2030**	**2000**	**2015**	**2016**	**2030**
Bangladesh	240·6 (215·8-263·9)	201·6 (178·6-228·3)	198·6 (175·6-225·3)	160·5 (138·6-187·5)	101·5 (71·9-156·3)	38·3 (24·6-67·1)	36·0 (23·3-63·0)	14·8 (10·0-25·5)	0·5 (0·3-0·8)	0·3 (0·2-0·5)	0·3 (0·2-0·4)	0·2 (0·1-0·4)	17·5 (15·9-19·2)	21·0 (19·0-23·1)	20·7 (18·7-23·2)	19·0 (14·8-24·0)
Bhutan	198·4 (169·2-228·7)	116·8 (98·1-140·1)	114·0 (95·6-137·2)	78·5 (60·0-100·3)	56·5 (12·3-113·8)	15·8 (3·5-35·9)	14·8 (3·3-33·9)	6·1 (1·3-15·8)	0·9 (0·6-1·5)	0·4 (0·2-0·6)	0·4 (0·2-0·6)	0·2 (0·1-0·4)	5·1 (4·0-6·4)	4·2 (3·7-4·8)	4·2 (3·7-4·9)	3·9 (2·9-5·2)
India	264·4 (230·7-295·8)	198·5 (177·9-218·9)	194·7 (174·6-214·9)	148·9 (134·2-165·5)	210·6 (145·2-317·7)	109·8 (76·0-169·2)	104·2 (71·8-160·8)	54·2 (34·7-85·6)	0·8 (0·5-0·9)	0·5 (0·3-0·5)	0·4 (0·3-0·5)	0·3 (0·2-0·4)	14·3 (13·8-14·8)	12·3 (11·8-12·9)	12·1 (11·6-12·6)	10·0 (8·9-11·2)
Nepal	262·9 (225·0-302·0)	219·7 (192·1-248·0)	215·6 (188·5-243·0)	176·8 (152·7-206·9)	194·0 (117·7-285·9)	66·6 (36·2-108·5)	60·7 (32·5-100·5)	17·0 (7·3-34·2)	0·8 (0·5-1·4)	0·4 (0·3-0·7)	0·4 (0·3-0·7)	0·2 (0·1-0·4)	32·7 (30·6-34·8)	18·1 (16·3-20·2)	18·4 (16·2-20·8)	14·8 (10·3-20·7)
Pakistan	270·9 (243·4-298·3)	211·2 (186·1-240·9)	207·1 (182·2-236·6)	164·7 (140·4-195·1)	82·1 (51·4-138·7)	45·2 (27·2-82·4)	43·1 (25·6-79·7)	22·3 (11·1-50·3)	1·1 (0·7-1·6)	0·7 (0·5-1·0)	0·7 (0·4-1·0)	0·4 (0·2-0·8)	17·9 (16·0-19·8)	13·0 (11·1-15·1)	12·9 (11·0-15·1)	9·7 (7·1-13·4)
**Southern Sub-Saharan Africa**																
Botswana	203·2 (113·3-362·6)	104·8 (58·2-153·5)	101·3 (55·0-147·4)	62·5 (24·9-85·5)	93·1 (41·3-194·3)	48·5 (24·5-81·8)	46·2 (23·1-77·4)	25·5 (10·8-41·8)	2·5 (1·2-5·2)	1·4 (0·7-2·3)	1·4 (0·6-2·3)	0·8 (0·2-2·1)	13·2 (11·9-14·5)	12·5 (11·3-13·7)	12·4 (11·1-13·6)	11·7 (9·6-14·1)
Lesotho	260·2 (200·5-320·3)	229·1 (176·5-283·8)	224·7 (172·3-279·6)	185·8 (129·3-240·5)	227·6 (135·9-341·6)	153·9 (86·3-246·0)	147·4 (81·8-237·3)	108·5 (47·4-207·4)	3·0 (1·9-4·7)	3·1 (1·8-4·9)	3·1 (1·8-4·8)	2·6 (1·0-5·3)	12·1 (11·2-13·0)	14·9 (14·0-15·7)	14·7 (13·9-15·6)	17·5 (15·2-19·7)
Namibia	243·4 (170·1-312·7)	132·1 (90·5-172·3)	127·8 (86·6-167·3)	79·2 (47·6-109·9)	101·0 (61·7-149·8)	57·3 (35·4-84·2)	54·4 (33·3-80·6)	31·1 (15·9-51·4)	2·7 (1·5-4·3)	1·3 (0·8-2·4)	1·3 (0·8-2·3)	0·7 (0·3-1·7)	14·0 (12·9-15·2)	11·8 (10·7-13·1)	11·7 (10·6-13·0)	10·5 (8·3-12·9)
South Africa	143·1 (129·5-158·9)	81·2 (71·6-91·3)	78·0 (68·8-87·6)	43·5 (38·4-48·9)	73·7 (54·1-107·3)	36·6 (24·8-53·8)	34·9 (23·5-51·3)	17·5 (10·9-25·5)	1·9 (1·5-2·2)	1·0 (0·9-1·2)	1·0 (0·8-1·2)	0·6 (0·4-0·9)	19·9 (19·3-20·5)	13·1 (12·6-13·6)	13·0 (12·5-13·6)	8·5 (7·8-9·3)
Swaziland	234·4 (171·1-332·8)	146·8 (97·2-200·8)	139·6 (90·6-192·3)	68·6 (33·5-104·5)	132·0 (82·6-201·8)	85·3 (56·0-127·6)	80·8 (52·5-121·7)	58·3 (28·3-109·2)	2·8 (1·7-4·8)	2·3 (1·4-3·7)	2·3 (1·3-3·6)	1·4 (0·5-3·4)	6·2 (5·6-6·8)	6·0 (5·6-6·6)	6·0 (5·5-6·5)	5·4 (4·4-6·5)
Zimbabwe	206·5 (151·4-276·7)	178·1 (134·9-224·1)	172·4 (131·4-216·7)	124·0 (94·8-188·9)	69·2 (47·6-100·3)	79·4 (56·3-112·7)	76·5 (54·0-108·9)	84·5 (40·5-186·3)	3·5 (2·3-5·1)	2·1 (1·4-4·6)	2·1 (1·3-4·4)	1·2 (0·4-3·3)	12·0 (11·2-13·0)	12·0 (11·2-12·9)	11·9 (11·1-12·8)	11·6 (10·0-13·4)
**Western Sub-Saharan Africa**																
Benin	259·4 (225·2-290·1)	229·5 (202·0-256·8)	230·2 (202·9-257·7)	214·7 (178·1-245·0)	166·8 (120·0-248·6)	121·0 (86·8-184·2)	117·4 (84·1-179·2)	87·7 (59·5-139·9)	2·9 (1·9-4·2)	2·0 (1·4-2·7)	2·0 (1·4-2·7)	1·5 (0·9-2·4)	5·5 (4·6-6·5)	4·8 (4·2-5·7)	4·8 (4·1-5·7)	4·6 (3·3-6·2)
Burkina Faso	257·6 (224·4-289·1)	226·3 (197·8-252·8)	226·0 (197·4-252·7)	221·0 (192·1-250·1)	211·5 (151·9-286·0)	130·7 (89·2-192·1)	127·8 (86·7-188·7)	91·1 (57·2-144·3)	3·4 (2·5-4·6)	2·5 (1·8-3·6)	2·5 (1·7-3·5)	2·0 (1·1-3·9)	12·9 (11·5-14·4)	8·9 (8·2-9·7)	8·7 (7·8-9·7)	7·0 (5·3-9·1)
Cameroon	271·2 (230·7-314·1)	234·4 (193·0-278·1)	233·5 (191·4-277·9)	219·8 (171·2-272·0)	159·8 (117·0-236·1)	92·2 (63·4-140·2)	88·1 (60·4-134·3)	55·2 (34·1-89·8)	2·6 (1·6-3·5)	2·0 (1·1-2·9)	2·0 (1·1-2·8)	1·5 (0·7-2·8)	6·8 (5·8-8·1)	6·1 (5·4-6·9)	6·1 (5·3-6·9)	5·8 (4·3-7·6)
Cape Verde	145·0 (117·1-172·5)	109·0 (83·4-141·7)	108·3 (82·3-141·8)	90·8 (59·9-135·3)	33·4 (23·4-46·2)	16·5 (11·2-23·5)	15·9 (10·7-22·7)	9·5 (5·8-14·3)	1·4 (1·0-2·0)	0·8 (0·6-1·0)	0·8 (0·6-1·0)	0·5 (0·2-0·9)	6·5 (5·4-7·6)	6·2 (5·2-7·5)	6·2 (5·2-7·5)	5·9 (4·0-8·1)
Chad	288·4 (247·0-329·3)	254·1 (220·4-290·5)	252·4 (219·1-288·5)	228·6 (199·6-261·9)	227·2 (167·5-325·5)	165·5 (120·0-237·4)	160·8 (116·4-231·9)	119·6 (82·4-187·0)	3·5 (2·0-5·0)	2·7 (1·7-4·0)	2·7 (1·6-3·9)	2·1 (1·1-4·1)	7·2 (6·3-8·1)	6·9 (5·8-8·1)	6·9 (5·8-8·1)	6·7 (4·7-9·0)
Cote d'Ivoire	274·7 (236·7-320·4)	247·8 (211·0-290·2)	247·9 (210·8-290·8)	249·2 (201·9-296·8)	113·9 (83·8-169·6)	92·6 (65·3-142·4)	90·6 (63·7-139·7)	66·7 (45·3-106·5)	3·3 (2·0-4·4)	2·4 (1·5-3·2)	2·4 (1·5-3·2)	1·9 (1·1-3·0)	7·5 (6·5-8·5)	9·4 (8·2-10·8)	9·3 (8·0-10·7)	9·2 (6·6-12·3)
The Gambia	235·7 (204·5-269·1)	213·8 (183·2-245·6)	213·4 (182·7-245·2)	206·0 (174·0-239·8)	92·6 (65·7-138·0)	68·2 (45·6-107·8)	67·0 (44·6-106·4)	51·0 (31·0-89·4)	2·2 (1·4-3·0)	1·6 (1·0-2·2)	1·5 (1·0-2·1)	1·1 (0·7-2·0)	11·6 (10·0-13·2)	9·9 (8·8-11·3)	9·8 (8·6-11·2)	8·5 (6·4-10·9)
Ghana	242·4 (206·8-275·1)	200·7 (174·5-226·4)	198·2 (172·3-223·5)	165·3 (144·1-186·8)	83·5 (63·3-115·4)	47·5 (33·6-71·2)	45·5 (32·0-68·1)	25·8 (17·2-38·6)	2·3 (1·5-3·0)	1·7 (1·1-2·3)	1·7 (1·0-2·3)	1·3 (0·6-2·3)	4·5 (3·9-5·0)	3·2 (2·7-3·7)	3·2 (2·7-3·7)	2·6 (1·9-3·5)
Guinea	273·9 (238·9-310·2)	264·6 (226·5-302·6)	264·6 (226·4-302·6)	263·8 (222·4-304·3)	176·8 (125·4-262·1)	101·4 (71·0-152·9)	97·5 (68·2-147·2)	55·7 (38·3-84·8)	3·2 (1·9-4·6)	2·5 (1·6-3·4)	2·5 (1·6-3·3)	1·9 (1·1-3·2)	8·4 (7·1-9·9)	8·2 (6·9-9·8)	8·2 (6·8-9·8)	8·0 (5·7-10·8)
Guinea-Bissau	353·7 (306·1-399·7)	320·4 (275·6-365·2)	318·7 (274·2-363·0)	295·0 (251·8-334·6)	178·2 (127·4-262·9)	109·1 (72·5-172·2)	106·2 (70·2-168·3)	71·3 (44·0-122·3)	4·0 (2·4-5·7)	3·1 (1·9-4·2)	3·1 (1·8-4·2)	2·4 (1·3-3·9)	7·3 (6·1-8·7)	7·2 (6·1-8·4)	7·2 (6·1-8·4)	7·0 (5·1-9·3)
Liberia	209·2 (175·9-240·7)	190·3 (162·2-218·0)	190·0 (162·2-217·3)	184·5 (160·3-208·4)	253·5 (183·0-355·6)	145·5 (96·8-230·5)	139·9 (92·5-224·1)	80·1 (46·1-151·3)	2·4 (1·5-4·3)	1·7 (1·1-2·6)	1·6 (1·1-2·5)	1·2 (0·7-2·0)	7·6 (6·4-8·8)	6·2 (5·5-7·1)	6·2 (5·4-7·2)	5·8 (4·3-7·6)
Mali	223·3 (194·6-252·7)	180·4 (151·6-214·2)	179·7 (150·5-214·3)	152·8 (120·4-196·4)	261·6 (186·5-356·1)	156·4 (101·5-241·1)	150·7 (96·9-233·8)	91·4 (51·7-161·1)	2·9 (1·7-5·0)	2·0 (1·2-3·6)	1·9 (1·2-3·6)	1·4 (0·7-3·0)	8·7 (7·7-9·8)	7·3 (6·3-8·3)	7·2 (6·3-8·3)	6·9 (5·1-8·9)
Mauritania	225·7 (187·9-261·4)	161·7 (124·4-197·1)	160·7 (122·8-196·7)	137·4 (94·7-180·1)	162·6 (119·1-239·6)	76·9 (49·1-128·4)	73·9 (46·8-124·2)	41·3 (23·5-79·5)	1·8 (1·2-2·6)	1·2 (0·7-1·9)	1·1 (0·7-1·9)	0·8 (0·4-1·6)	10·5 (9·3-11·7)	9·6 (8·1-11·2)	9·6 (8·0-11·3)	9·2 (6·7-12·4)
Niger	302·4 (263·2-344·0)	266·9 (228·2-308·5)	267·3 (228·0-309·9)	233·6 (175·5-285·3)	253·8 (188·9-352·1)	154·7 (106·5-235·9)	151·2 (103·6-231·7)	108·0 (66·8-176·9)	3·4 (1·9-6·0)	2·5 (1·5-5·1)	2·5 (1·5-5·0)	2·2 (1·1-4·8)	4·1 (3·3-4·9)	5·0 (4·3-5·6)	5·0 (4·3-5·7)	5·4 (3·9-7·3)
Nigeria	210·4 (169·5-255·4)	151·2 (123·0-185·5)	150·4 (122·4-184·2)	134·0 (107·9-162·8)	168·0 (116·4-236·6)	107·1 (73·4-151·0)	101·9 (69·3-144·8)	59·6 (35·3-91·3)	1·8 (1·2-2·7)	1·1 (0·7-1·7)	1·1 (0·7-1·7)	0·8 (0·3-1·7)	4·9 (4·3-5·7)	3·6 (3·2-4·1)	3·6 (3·2-4·2)	2·7 (2·0-3·6)
Sao Tome and Principe	237·8 (199·5-276·1)	153·1 (100·5-195·9)	149·1 (95·4-193·1)	102·1 (45·6-156·0)	76·5 (55·4-104·1)	31·2 (19·7-48·3)	29·7 (18·6-46·5)	14·2 (7·5-26·2)	1·1 (0·7-1·7)	0·8 (0·5-1·4)	0·8 (0·5-1·4)	0·6 (0·3-1·6)	3·8 (3·1-4·6)	3·5 (3·1-4·1)	3·6 (3·0-4·1)	3·3 (2·5-4·3)
Senegal	215·4 (187·7-244·9)	198·5 (166·6-230·3)	198·0 (165·1-230·4)	190·2 (147·1-231·7)	150·5 (107·7-219·5)	83·8 (57·9-127·7)	82·4 (56·5-125·8)	62·5 (39·8-99·4)	2·6 (1·8-3·7)	2·2 (1·5-3·2)	2·1 (1·5-3·1)	1·6 (1·0-2·9)	7·9 (6·9-9·0)	5·6 (4·9-6·5)	5·6 (4·8-6·5)	4·4 (3·1-6·0)

Location	Indicator 3.b.1: Geometric mean of the coverage of eight vaccines, conditional on inclusion in national vaccine schedules, in target populations (%)				Indicator 5.2.1: Age-standardised prevalence of women aged 15 years and older who experienced physical or sexual violence by an intimate partner in the last 12 months (%)				Indicator 6.1.1: Risk-weighted prevalence of populations using unsafe or unimproved water sources, as measured by the summary exposure value (SEV) for unsafe water (%)				Indicator 6.2.1a: Risk-weighted prevalence of populations using unsafe or unimproved sanitation, as measured by the summary exposure value (SEV) for unsafe sanitation (%)			
	**2000**	**2015**	**2016**	**2030**	**2000**	**2015**	**2016**	**2030**	**2000**	**2015**	**2016**	**2030**	**2000**	**2015**	**2016**	**2030**
Bangladesh	86·0 (83·8-88·0)	94·1 (91·9-95·6)	94·3 (91·8-96·0)	97·6 (93·8-99·4)	49·3 (43·6-54·4)	44·8 (39·9-49·3)	44·4 (39·5-48·9)	40·0 (35·6-44·2)	42·6 (36·8-46·1)	41·2 (34·8-44·8)	41·0 (34·7-44·8)	39·5 (32·5-44·1)	81·5 (71·9-91·3)	63·7 (46·2-83·7)	62·5 (44·3-83·0)	41·9 (20·9-68·4)
Bhutan	88·0 (85·3-90·3)	97·0 (95·5-98·0)	97·1 (95·6-98·2)	99·1 (97·7-99·8)	41·5 (36·5-46·5)	36·4 (32·1-40·6)	36·1 (31·9-40·4)	32·2 (28·0-36·6)	18·7 (3·0-26·6)	10·1 (0·6-15·5)	9·9 (0·6-15·3)	7·2 (0·7-17·0)	73·8 (62·2-83·7)	39·9 (27·1-52·4)	38·0 (25·1-50·5)	14·6 (7·2-22·0)
India	67·6 (65·8-69·4)	84·0 (82·7-85·2)	85·2 (83·8-86·5)	94·2 (92·7-95·5)	42·0 (37·6-46·0)	39·0 (34·8-42·9)	38·7 (34·5-42·7)	35·5 (31·4-39·6)	36·3 (28·7-40·1)	32·4 (25·3-35·9)	32·0 (24·9-35·4)	26·3 (19·5-29·3)	74·7 (72·2-77·8)	49·1 (46·0-52·9)	47·2 (44·1-51·1)	23·3 (20·6-26·3)
Nepal	78·3 (74·8-81·4)	88·1 (83·5-91·6)	89·0 (84·3-92·7)	96·4 (90·5-99·2)	41·4 (36·7-45·7)	37·9 (33·8-41·5)	37·8 (33·7-41·4)	35·2 (31·4-38·6)	41·9 (33·8-46·2)	35·7 (27·9-40·8)	35·2 (27·3-40·4)	27·7 (20·0-35·0)	77·5 (71·6-82·7)	51·6 (43·5-59·5)	49·9 (41·7-57·9)	26·3 (19·2-33·2)
Pakistan	74·0 (71·8-76·1)	75·7 (69·6-80·8)	75·6 (68·5-81·3)	75·0 (54·8-87·7)	50·5 (45·0-55·8)	47·0 (42·1-51·5)	46·6 (41·9-51·2)	42·7 (38·3-46·8)	38·2 (30·8-43·3)	34·5 (27·9-40·2)	34·3 (27·6-40·4)	31·9 (23·2-42·4)	55·9 (47·6-64·2)	35·8 (27·6-44·7)	34·2 (26·1-43·0)	16·3 (10·5-22·8)
**Southern Sub-Saharan Africa**																
Botswana	90·5 (87·4-92·9)	97·3 (95·6-98·5)	98·1 (96·7-99·0)	99·7 (99·1-100·0)	37·6 (33·0-42·7)	35·0 (30·8-39·3)	34·7 (30·6-38·9)	31·2 (27·5-34·9)	33·6 (23·7-40·1)	27·7 (17·5-36·1)	27·3 (17·2-35·9)	22·2 (8·5-42·2)	79·9 (70·7-85·8)	55·1 (24·4-82·6)	53·6 (20·9-84·4)	28·9 (1·1-72·3)
Lesotho	83·2 (81·3-85·1)	85·6 (82·4-88·3)	84·8 (80·9-88·2)	87·7 (76·2-95·0)	48·4 (42·8-53·7)	47·2 (41·6-52·7)	47·1 (41·6-52·6)	46·2 (40·6-51·8)	43·5 (34·6-48·0)	38·6 (29·8-44·1)	38·2 (29·3-43·8)	32·8 (23·2-39·7)	91·5 (78·2-100·0)	83·0 (61·0-100·0)	82·7 (59·6-100·0)	76·2 (35·9-99·6)
Namibia	74·8 (71·9-77·5)	87·9 (84·2-91·0)	87·5 (83·1-91·1)	93·7 (84·1-97·8)	44·5 (39·4-50·0)	39·1 (34·8-43·8)	38·9 (34·6-43·5)	35·6 (31·7-39·6)	37·1 (27·5-43·3)	29·3 (20·5-38·4)	28·8 (20·1-38·1)	22·8 (9·1-43·6)	63·6 (46·4-77·6)	57·6 (39·0-74·0)	56·6 (36·7-74·3)	45·7 (13·4-83·7)
South Africa	77·5 (74·6-80·2)	86·8 (81·9-90·5)	86·5 (80·4-90·9)	93·5 (83·9-98·3)	33·0 (29·3-36·8)	31·0 (27·5-34·7)	31·0 (27·5-34·6)	30·0 (26·7-33·4)	25·4 (17·8-30·8)	20·4 (13·9-25·8)	20·2 (13·7-25·5)	16·9 (11·3-22·0)	45·5 (41·6-49·5)	39·1 (37·0-41·3)	38·3 (36·0-40·8)	28·0 (23·3-33·2)
Swaziland	88·9 (85·7-91·3)	96·1 (94·1-97·5)	96·2 (94·2-97·7)	99·3 (97·6-100·0)	42·7 (37·5-48·1)	43·1 (37·9-48·8)	42·8 (37·6-48·5)	40·1 (34·5-45·7)	41·2 (30·4-47·1)	34·1 (24·1-42·0)	33·5 (23·5-41·6)	26·0 (17·8-35·7)	81·1 (67·0-92·1)	73·3 (55·6-87·8)	72·0 (53·6-87·2)	69·0 (33·3-100·0)
Zimbabwe	74·0 (69·8-78·0)	95·9 (94·0-97·2)	97·2 (95·8-98·2)	99·8 (99·5-100·0)	42·1 (37·7-47·2)	43·6 (39·1-48·4)	43·4 (38·9-48·1)	40·2 (35·6-45·3)	36·8 (26·7-42·6)	38·5 (29·1-43·9)	38·4 (29·0-43·9)	37·0 (19·6-57·4)	60·8 (42·1-78·5)	60·5 (41·2-79·3)	60·1 (39·6-79·5)	53·2 (22·8-81·3)
**Western Sub-Saharan Africa**																
Benin	72·2 (69·2-74·7)	78·9 (73·4-82·9)	80·1 (74·2-84·5)	91·9 (80·7-98·2)	44·3 (39·0-49·5)	43·5 (38·3-48·5)	43·4 (38·3-48·4)	42·4 (37·5-47·3)	40·7 (31·2-46·9)	41·7 (32·9-47·5)	41·7 (32·8-47·7)	42·3 (32·5-50·1)	93·2 (85·1-100·0)	85·9 (69·8-99·1)	85·4 (68·0-99·0)	76·3 (38·9-96·4)
Burkina Faso	66·5 (63·4-69·5)	89·7 (86·6-92·4)	88·5 (83·8-92·1)	89·4 (73·5-97·2)	39·5 (34·8-44·0)	37·7 (33·3-42·2)	37·6 (33·2-42·1)	36·0 (31·6-40·4)	47·0 (37·9-51·5)	43·8 (36·1-48·1)	43·5 (35·8-47·9)	40·4 (32·3-45·4)	94·3 (87·4-99·3)	85·2 (70·0-100·0)	84·6 (68·5-100·0)	72·4 (41·0-100·0)
Cameroon	63·6 (60·1-66·9)	87·0 (82·3-90·8)	88·2 (83·3-92·1)	95·6 (88·7-98·9)	44·5 (40·0-49·5)	44·3 (39·7-49·1)	44·1 (39·6-49·0)	42·3 (38·0-47·1)	44·8 (34·1-49·7)	41·1 (32·4-46·3)	40·9 (32·1-46·1)	37·7 (29·1-44·1)	88·1 (79·3-97·8)	74·6 (55·8-96·7)	73·8 (54·2-96·5)	59·5 (28·5-90·7)
Cape Verde	79·6 (75·2-83·4)	97·0 (95·1-98·2)	97·3 (95·5-98·5)	99·8 (99·1-100·0)	42·9 (37·9-48·2)	39·4 (34·8-44·3)	39·1 (34·5-44·0)	35·6 (31·2-40·2)	37·8 (28·2-45·4)	22·6 (12·9-34·5)	22·2 (12·3-34·2)	16·2 (6·6-30·0)	71·5 (35·3-93·6)	43·0 (18·7-71·2)	41·7 (16·6-73·4)	19·7 (1·4-64·2)
Chad	25·5 (23·1-28·0)	48·1 (40·3-55·5)	49·3 (40·4-57·9)	75·4 (57·7-89·6)	45·9 (40·9-50·7)	44·3 (39·5-48·8)	44·2 (39·5-48·9)	43·7 (38·3-49·1)	47·2 (38·4-51·8)	46·1 (36·2-51·5)	46·0 (36·1-51·4)	44·4 (33·9-50·4)	95·3 (87·8-100·0)	94·0 (85·7-100·0)	93·9 (85·2-100·0)	92·7 (76·9-100·0)
Cote d'Ivoire	69·8 (66·0-73·4)	91·6 (87·9-94·4)	91·9 (87·9-94·8)	98·0 (94·7-99·6)	41·2 (36·4-45·9)	38·9 (34·6-43·1)	38·8 (34·5-43·0)	37·3 (33·0-41·6)	37·3 (28·0-44·6)	35·0 (24·5-43·7)	34·6 (23·8-43·7)	30·1 (15·8-43·7)	79·7 (69·0-88·6)	66·7 (38·2-87·3)	65·7 (35·1-87·6)	50·2 (6·7-87·1)
The Gambia	80·1 (77·0-82·8)	87·4 (83·4-90·8)	87·5 (82·9-91·3)	92·2 (82·4-97·5)	36·2 (32·0-40·7)	34·4 (30·3-38·8)	34·3 (30·3-38·7)	33·6 (29·4-38·1)	41·8 (34·8-46·8)	35·1 (27·3-42·7)	34·8 (26·9-42·5)	29·8 (21·0-39·8)	88·1 (79·8-94·8)	69·5 (51·2-88·7)	69·0 (50·1-88·6)	45·0 (20·9-67·6)
Ghana	81·6 (79·3-83·7)	96·2 (93·9-97·7)	96·5 (94·1-98·0)	99·2 (97·8-99·8)	38·9 (34·4-43·5)	37·5 (33·3-41·6)	37·3 (33·1-41·4)	35·0 (30·6-39·2)	40·1 (32·3-45·5)	41·7 (34·6-45·9)	41·8 (34·7-46·0)	43·6 (35·9-47·5)	83·1 (67·3-97·9)	67·1 (46·5-88·8)	65·9 (44·9-87·5)	45·3 (22·4-57·7)
Guinea	61·6 (56·3-66·6)	75·6 (68·2-81·7)	76·1 (68·0-83·1)	79·1 (55·7-94·0)	45·6 (40·3-50·9)	45·3 (39·9-50·5)	45·2 (39·8-50·4)	44·6 (39·2-49·7)	43·9 (34·8-49·2)	40·8 (30·7-47·0)	40·5 (30·3-46·9)	36·5 (24·1-45·5)	88·8 (75·3-100·0)	71·4 (51·5-88·8)	70·2 (49·3-88·3)	47·2 (18·6-71·2)
Guinea-Bissau	69·9 (64·8-74·6)	89·1 (85·4-92·4)	89·0 (84·6-92·7)	96·9 (91·5-99·4)	50·1 (44·4-55·8)	48·9 (43·2-54·4)	48·8 (43·1-54·2)	47·1 (41·6-52·7)	48·4 (35·4-55·5)	45·5 (33·8-51·9)	45·3 (33·4-51·9)	43·1 (21·5-66·7)	89·5 (79·1-96·0)	88·8 (81·9-93·8)	88·4 (81·4-93·5)	87·1 (78·0-96·2)
Liberia	45·3 (40·3-50·0)	74·9 (67·8-80·5)	75·8 (68·1-81·8)	78·8 (60·9-90·2)	53·6 (47·8-59·3)	49·4 (44·0-54·6)	49·2 (43·8-54·6)	46·8 (40·5-53·7)	44·6 (35·9-49·3)	44·2 (35·8-48·9)	44·1 (35·8-48·9)	43·6 (35·6-49·0)	86·4 (67·6-97·9)	72·2 (53·9-87·4)	71·3 (51·6-87·1)	55·1 (21·8-79·9)
Mali	49·6 (46·5-53·0)	70·2 (64·6-75·5)	71·8 (65·2-77·8)	79·8 (60·7-92·6)	50·4 (44·9-55·7)	48·2 (43·2-52·9)	48·1 (43·1-52·8)	46·3 (41·3-51·1)	45·7 (35·5-51·2)	42·8 (34·1-47·7)	42·8 (34·0-47·7)	41·8 (32·9-46·7)	92·7 (85·4-98·7)	84·3 (70·3-100·0)	83·8 (69·3-100·0)	74·8 (51·1-99·8)
Mauritania	61·5 (58·4-64·9)	79·1 (74·0-83·3)	79·3 (73·0-84·2)	89·8 (77·7-96·5)	39·8 (35·0-45·0)	37·7 (33·3-42·6)	37·6 (33·1-42·3)	35·2 (31·2-39·5)	39·6 (29·1-46·1)	34·1 (21·2-43·5)	33·7 (20·5-43·3)	27·1 (12·6-40·9)	94·8 (86·2-99·7)	77·1 (54·3-95·1)	76·1 (51·3-94·9)	52·4 (12·3-80·9)
Niger	41·3 (37·6-45·1)	83·0 (77·7-86·9)	82·8 (76·8-87·6)	93·1 (83·5-98·1)	50·5 (44·8-55·9)	50·2 (44·6-55·4)	50·2 (44·6-55·4)	49·9 (44·4-55·0)	45·7 (36·3-50·9)	42·1 (33·0-48·2)	41·3 (32·2-47·7)	31·5 (23·5-40·8)	95·9 (89·1-100·0)	92·1 (82·1-99·8)	91·8 (81·2-99·7)	85·7 (64·3-98·0)
Nigeria	32·5 (29·2-35·8)	54·2 (48·1-60·2)	52·9 (45·6-60·1)	65·0 (49·2-78·5)	34·0 (30·1-38·2)	30·9 (27·4-34·5)	30·7 (27·2-34·4)	28·0 (24·7-31·7)	45·4 (35·4-50·5)	43·6 (34·2-48·8)	43·4 (34·0-48·7)	41·4 (31·6-48·1)	77·4 (60·0-92·5)	57·1 (40·3-72·7)	53·9 (36·7-69·5)	43·8 (10·8-100·0)
Sao Tome and Principe	83·2 (80·2-85·6)	93·7 (91·1-95·6)	93·7 (90·8-95·7)	97·7 (92·0-99·6)	47·4 (41·9-53·0)	45·5 (40·3-50·5)	45·5 (40·1-50·4)	44·3 (38·8-49·5)	38·4 (27·3-46·0)	33·7 (25·5-41·4)	33·6 (25·1-41·5)	31·4 (20·3-42·5)	81·4 (74·1-87·4)	39·5 (23·2-61·3)	35·7 (19·7-57·8)	5·3 (1·4-14·3)
Senegal	65·2 (61·6-68·5)	82·7 (77·5-86·8)	83·2 (77·1-88·0)	89·2 (74·2-96·5)	39·3 (34·4-44·5)	39·3 (34·4-44·4)	39·3 (34·4-44·3)	39·0 (34·2-43·6)	36·8 (26·6-43·6)	29·6 (18·9-40·3)	29·2 (18·2-40·3)	24·0 (10·8-39·8)	68·0 (46·0-84·9)	61·1 (35·2-85·1)	61·8 (34·5-86·8)	63·2 (25·0-89·7)

Location	Indicator 6.2.1b: Risk-weighted prevalence of populations without access to a handwashing facility, as measured by the summary exposure value (SEV) for unsafe hygiene (%)				Indicator 7.1.2: Risk-weighted prevalence of household air pollution, as measured by the summary exposure value (SEV) for household air pollution (%)				Indicator 8.8.1: Age-standardised all-cause disability-adjusted life year (DALY) rates attributable to occupational risks (per 100,000 population)				Indicator 11.6.2: Population-weighted mean levels of fine particulate matter smaller than 2.5 microns in diameter (PM2.5)			
	2000	2015	2016	2030	2000	2015	2016	2030	2000	2015	2016	2030	2000	2015	2016	2030
Bangladesh	74·7 (72·9-76·5)	71·3 (69·3-73·1)	71·0 (69·1-72·9)	67·5 (65·4-69·8)	58·0 (46·9-69·5)	45·8 (36·5-56·0)	44·7 (35·5-54·8)	30·3 (23·5-37·7)	1,716·8 (1,444·0-2,015·5)	1,282·0 (1,085·7-1,496·9)	1,268·8 (1,073·9-1,483·2)	1,095·1 (914·1-1,301·2)	65·5 (64·2-66·9)	100·3 (98·0-102·8)	101·0 (98·5-103·8)	108·1 (104·9-111·4)
Bhutan	24·7 (22·9-26·5)	19·5 (18·1-21·1)	19·5 (18·1-21·1)	18·3 (11·4-22·3)	39·1 (30·8-48·6)	14·8 (10·0-20·6)	13·8 (9·1-19·5)	4·7 (2·4-8·2)	1,782·9 (1,507·5-2,094·0)	1,404·5 (1,163·6-1,653·9)	1,391·1 (1,151·5-1,638·1)	1,207·6 (990·1-1,447·2)	41·0 (38·7-43·4)	55·8 (52·7-59·2)	55·8 (52·7-59·1)	56·7 (53·1-60·7)
India	59·7 (59·3-60·1)	49·4 (48·9-49·8)	48·4 (48·0-48·9)	40·0 (39·5-40·4)	47·2 (39·8-55·4)	29·0 (23·9-34·9)	27·5 (22·6-33·1)	11·7 (9·6-14·1)	1,516·9 (1,339·9-1,694·6)	1,242·8 (1,088·3-1,400·1)	1,238·4 (1,083·4-1,396·2)	1,176·5 (1,015·1-1,342·9)	61·5 (61·1-61·9)	75·6 (75·2-76·0)	75·8 (75·4-76·2)	79·0 (78·5-79·5)
Nepal	60·8 (58·6-63·1)	45·2 (43·1-47·4)	43·3 (41·2-45·5)	32·9 (28·0-36·5)	53·5 (43·9-63·8)	42·2 (33·1-51·6)	40·6 (31·7-49·7)	21·5 (16·4-26·3)	1,990·8 (1,700·5-2,316·7)	1,677·4 (1,416·6-1,953·9)	1,664·5 (1,407·7-1,939·4)	1,492·0 (1,269·5-1,745·6)	56·1 (54·0-58·2)	77·9 (73·6-82·8)	78·5 (73·9-83·9)	87·4 (81·6-94·0)
Pakistan	50·6 (48·2-52·9)	45·2 (42·8-47·7)	45·0 (42·6-47·5)	42·2 (39·6-44·9)	47·1 (37·9-56·5)	34·0 (27·1-41·6)	32·6 (25·9-40·0)	17·5 (13·2-21·7)	1,266·0 (1,085·3-1,465·1)	1,152·2 (973·8-1,333·4)	1,146·8 (968·4-1,328·5)	1,072·5 (901·7-1,257·2)	69·6 (68·4-71·0)	75·1 (73·7-76·5)	75·8 (74·4-77·3)	73·5 (70·0-75·5)
**Southern Sub-Saharan Africa**																
Botswana	63·9 (39·5-82·8)	58·7 (35·3-80·5)	58·1 (34·7-80·1)	51·5 (28·2-77·6)	30·2 (23·1-38·2)	14·0 (7·4-22·0)	13·1 (6·7-21·1)	4·7 (1·3-10·1)	1,387·1 (963·8-2,086·7)	1,052·9 (837·7-1,289·3)	1,036·2 (825·7-1,263·9)	828·1 (679·0-952·8)	22·3 (20·7-24·4)	22·8 (21·2-24·9)	23·1 (21·5-25·3)	21·8 (19·8-23·9)
Lesotho	96·8 (93·4-98·9)	96·2 (92·8-98·3)	96·1 (92·6-98·2)	95·4 (92·0-97·9)	42·6 (34·1-52·0)	31·7 (24·9-40·0)	30·9 (24·1-39·1)	20·3 (14·6-27·8)	1,418·0 (1,156·1-1,703·0)	1,233·5 (1,009·3-1,476·7)	1,221·8 (998·5-1,462·2)	1,066·5 (859·3-1,288·0)	28·0 (23·1-36·2)	26·5 (21·7-35·0)	26·9 (22·1-35·7)	30·3 (22·1-43·0)
Namibia	64·3 (42·9-82·2)	54·4 (37·4-70·7)	53·7 (36·6-70·1)	43·8 (27·5-60·8)	38·6 (30·7-47·2)	26·2 (19·1-34·9)	25·3 (18·3-34·0)	14·9 (9·4-22·6)	1,013·7 (818·6-1,231·5)	774·3 (631·1-933·5)	765·2 (623·3-923·0)	647·8 (523·6-784·4)	27·2 (23·8-32·4)	25·6 (21·8-32·1)	25·9 (21·5-32·8)	23·7 (19·4-31·2)
South Africa	49·9 (42·1-58·0)	50·5 (41·9-58·8)	50·5 (41·8-58·8)	50·6 (41·4-59·0)	17·6 (14·1-21·3)	6·8 (5·2-8·7)	6·3 (4·8-8·1)	2·2 (1·6-3·1)	1,218·8 (1,087·2-1,355·4)	926·1 (819·3-1,043·6)	922·9 (817·2-1,039·3)	854·5 (768·6-954·4)	37·1 (36·1-38·0)	35·5 (34·6-36·5)	35·9 (34·9-37·0)	36·9 (32·9-40·9)
Swaziland	72·5 (55·0-86·2)	62·7 (45·4-77·5)	61·8 (44·2-76·9)	53·0 (35·8-68·8)	41·6 (33·6-50·8)	20·8 (13·7-29·0)	19·6 (12·6-27·9)	8·0 (3·4-14·9)	1,300·4 (1,020·7-1,699·4)	1,041·2 (827·3-1,278·3)	1,016·7 (808·1-1,246·0)	728·3 (572·3-872·6)	27·6 (23·5-32·8)	24·3 (20·9-29·0)	24·2 (20·9-28·5)	22·6 (18·6-28·0)
Zimbabwe	73·3 (55·4-86·0)	85·5 (76·3-91·7)	85·7 (76·5-91·9)	90·0 (81·5-94·8)	39·2 (31·1-47·8)	36·3 (28·2-45·4)	35·4 (27·4-44·5)	24·3 (17·3-33·1)	1,267·5 (1,021·0-1,519·8)	1,182·2 (980·9-1,398·4)	1,161·0 (964·0-1,374·0)	901·3 (747·8-1,069·5)	25·1 (22·8-28·2)	24·4 (22·3-27·0)	24·5 (22·6-27·2)	25·3 (21·3-29·7)
**Western Sub-Saharan Africa**																
Benin	95·7 (92·3-98·1)	93·1 (87·5-96·7)	93·0 (87·2-96·7)	90·6 (80·3-96·6)	67·4 (55·1-80·6)	59·9 (47·8-72·8)	59·2 (47·0-72·2)	49·3 (36·7-62·8)	1,296·2 (1,092·4-1,525·6)	1,225·7 (1,021·1-1,467·0)	1,215·9 (1,012·7-1,456·3)	1,087·5 (899·5-1,316·1)	58·8 (53·3-67·4)	90·5 (83·1-99·3)	96·0 (88·7-106·2)	83·3 (73·2-95·7)
Burkina Faso	92·1 (85·9-96·1)	88·9 (80·4-94·7)	88·6 (79·7-94·7)	84·5 (72·1-93·8)	74·6 (62·0-86·0)	64·6 (51·8-77·5)	63·7 (51·0-76·7)	50·6 (39·8-61·8)	997·2 (807·1-1,210·1)	959·9 (771·0-1,179·6)	954·9 (766·5-1,173·1)	887·2 (710·0-1,097·3)	66·8 (63·5-71·3)	104·9 (97·2-116·9)	110·7 (102·0-124·9)	108·7 (99·3-123·3)
Cameroon	88·9 (76·3-96·0)	87·6 (74·4-95·3)	87·4 (74·0-95·3)	85·6 (71·1-94·9)	55·0 (44·2-66·1)	45·4 (32·1-58·9)	44·6 (30·7-58·6)	34·0 (15·5-53·1)	1,150·8 (957·4-1,365·9)	1,092·8 (900·1-1,305·4)	1,086·1 (893·9-1,297·8)	996·8 (808·3-1,197·5)	99·6 (96·7-102·8)	134·8 (130·2-140·3)	139·7 (134·3-145·3)	126·6 (107·8-139·6)
Cape Verde	82·5 (67·0-92·6)	64·0 (39·8-82·9)	63·3 (39·2-82·4)	39·4 (16·6-65·6)	28·4 (19·3-38·9)	15·7 (4·1-33·2)	14·9 (3·4-33·0)	6·4 (0·2-26·3)	695·7 (571·1-843·0)	643·4 (520·7-786·2)	637·0 (515·3-778·7)	554·3 (445·3-681·3)	49·5 (38·3-64·8)	65·0 (49·0-86·1)	66·6 (49·2-88·1)	71·1 (48·3-98·1)
Chad	87·0 (74·7-94·2)	91·4 (85·4-95·5)	91·6 (85·7-95·7)	94·4 (89·0-97·5)	71·1 (57·6-83·8)	65·9 (53·8-78·1)	65·6 (53·4-77·8)	60·8 (48·9-73·2)	1,226·9 (1,012·5-1,454·8)	1,090·9 (894·4-1,305·4)	1,083·2 (888·1-1,296·5)	980·5 (801·7-1,179·3)	64·0 (60·1-70·6)	91·7 (86·4-100·7)	95·7 (90·0-105·1)	88·3 (80·7-99·9)
Cote d'Ivoire	85·0 (71·0-93·9)	84·1 (72·6-92·3)	83·7 (71·8-92·2)	77·0 (59·9-89·9)	53·0 (41·5-65·3)	47·8 (33·8-61·6)	46·8 (32·3-61·0)	33·7 (15·8-52·3)	1,248·3 (1,046·3-1,464·9)	1,154·2 (963·0-1,362·3)	1,149·2 (959·5-1,355·8)	1,080·5 (906·1-1,274·1)	32·3 (30·1-35·8)	55·3 (50·5-62·7)	57·8 (52·8-65·9)	54·6 (49·6-62·3)
The Gambia	95·0 (89·5-98·2)	92·0 (85·4-96·3)	91·7 (85·0-96·2)	87·9 (79·0-94·5)	67·7 (55·9-80·5)	62·9 (51·5-75·2)	62·5 (51·1-74·8)	56·4 (45·6-69·6)	1,023·1 (854·9-1,214·1)	920·7 (755·0-1,095·1)	914·2 (750·0-1,087·2)	827·7 (682·9-984·3)	66·9 (41·0-136·1)	93·5 (54·1-174·4)	95·0 (56·4-187·9)	99·8 (57·2-202·6)
Ghana	86·3 (76·6-93·1)	92·8 (88·6-95·5)	93·0 (88·8-95·7)	95·3 (92·1-97·3)	55·1 (44·7-65·9)	43·2 (34·0-53·7)	42·2 (32·9-52·6)	28·2 (20·0-37·6)	1,007·7 (835·2-1,201·9)	1,010·6 (826·3-1,204·8)	1,005·5 (821·5-1,199·2)	937·5 (757·1-1,128·7)	36·5 (35·1-38·2)	52·1 (49·6-55·0)	54·2 (51·2-57·7)	51·3 (48·1-54·7)
Guinea	92·8 (84·1-97·2)	91·4 (84·1-95·9)	91·2 (83·7-95·8)	89·6 (81·9-95·2)	71·1 (58·0-83·7)	66·4 (54·4-79·0)	66·1 (54·0-78·6)	60·4 (49·1-72·9)	1,353·0 (1,134·6-1,575·9)	1,216·1 (1,018·7-1,436·9)	1,204·0 (1,006·8-1,422·8)	1,045·6 (872·5-1,238·8)	30·6 (28·5-33·6)	44·5 (41·0-49·7)	46·1 (42·5-51·5)	46·0 (41·5-52·6)
Guinea-Bissau	90·6 (80·0-96·7)	91·0 (80·7-96·8)	90·9 (80·4-96·8)	90·9 (80·0-96·9)	70·2 (57·8-83·0)	64·4 (51·7-77·1)	63·9 (51·3-76·6)	56·7 (45·3-69·3)	969·5 (799·4-1,138·2)	958·4 (779·1-1,140·5)	954·4 (776·3-1,135·1)	900·7 (738·3-1,073·4)	41·8 (33·7-61·1)	57·0 (44·2-83·7)	59·2 (45·2-92·8)	59·2 (43·7-92·8)
Liberia	98·3 (96·4-99·4)	98·4 (97·0-99·3)	98·4 (97·0-99·3)	98·7 (97·4-99·4)	73·6 (60·1-85·6)	66·1 (53·9-78·4)	65·6 (53·5-77·8)	57·8 (47·8-68·6)	1,077·2 (899·7-1,274·7)	938·5 (776·0-1,112·2)	929·1 (768·1-1,101·1)	806·5 (663·6-959·7)	10·6 (9·7-11·8)	16·8 (15·0-19·6)	17·3 (15·4-20·1)	17·6 (15·4-20·6)
Mali	90·4 (83·3-95·3)	89·7 (81·1-94·8)	89·7 (80·7-94·9)	89·0 (77·7-94·8)	74·7 (61·3-86·0)	69·9 (57·3-82·6)	69·4 (56·9-82·2)	62·9 (50·7-76·6)	1,195·3 (979·7-1,404·1)	1,076·4 (901·6-1,257·9)	1,065·4 (893·0-1,245·8)	923·0 (771·9-1,088·3)	60·2 (56·6-65·8)	88·6 (81·7-100·3)	92·2 (85·0-103·5)	87·4 (78·0-101·0)
Mauritania	77·3 (59·2-89·4)	69·7 (52·9-83·1)	68·9 (51·8-82·6)	59·2 (42·4-75·6)	48·6 (38·7-60·2)	31·0 (18·2-44·0)	30·2 (16·7-44·1)	19·9 (4·4-41·8)	813·4 (656·3-984·0)	720·5 (567·8-878·5)	716·7 (566·3-872·9)	665·5 (537·1-803·0)	95·8 (76·4-134·2)	121·3 (98·6-161·2)	123·6 (102·0-169·3)	122·0 (89·8-180·9)
Niger	87·2 (74·0-94·9)	83·4 (71·7-91·5)	82·8 (71·1-91·1)	79·6 (65·9-89·4)	75·9 (61·8-87·6)	73·5 (60·3-85·7)	73·3 (60·0-85·5)	69·6 (57·1-81·2)	1,441·7 (1,121·5-1,737·2)	1,397·9 (1,148·9-1,649·7)	1,387·6 (1,150·4-1,634·8)	1,251·7 (1,094·6-1,441·0)	128·9 (122·2-137·3)	190·9 (181·3-203·1)	203·7 (191·9-219·1)	196·6 (181·9-211·6)
Nigeria	88·0 (78·0-94·8)	88·3 (81·5-93·1)	88·2 (81·3-93·0)	88·6 (81·4-93·5)	51·9 (40·7-63·9)	41·3 (30·2-53·0)	40·3 (29·0-52·4)	27·4 (15·1-42·1)	932·3 (737·3-1,143·4)	827·8 (661·1-1,006·9)	827·4 (659·9-1,007·8)	822·6 (643·8-1,013·8)	74·5 (73·4-75·7)	115·1 (113·0-117·6)	122·5 (119·9-125·3)	93·0 (84·4-100·6)
Sao Tome and Principe	72·8 (52·8-87·3)	65·5 (47·9-79·6)	65·1 (47·2-79·1)	58·3 (40·7-71·1)	57·7 (46·8-69·3)	34·5 (16·6-53·4)	33·2 (14·6-53·1)	17·0 (1·5-44·9)	791·8 (635·4-936·8)	699·1 (568·0-834·4)	693·2 (565·1-826·3)	615·4 (528·2-722·8)	15·9 (10·0-25·2)	15·4 (9·6-24·6)	15·4 (9·6-24·7)	15·1 (8·7-25·4)
Senegal	71·2 (55·6-84·7)	73·7 (60·9-84·2)	73·8 (60·6-84·5)	74·7 (58·4-86·3)	50·2 (40·4-60·1)	44·0 (28·0-59·5)	43·5 (26·4-60·0)	35·6 (10·1-64·1)	880·6 (734·9-1,029·7)	885·9 (721·2-1,051·3)	884·3 (719·5-1,050·3)	862·0 (696·9-1,027·2)	41·0 (38·8-43·6)	55·5 (52·5-58·8)	56·8 (53·6-60·7)	56·4 (53·0-60·5)

Location	Indicator 16.1.1: Age-standardised death rate due to interpersonal violence (per 100,000 population)				Indicator 16.1.2: Death rate due to conflict and terrorism (per 100,000 population)				Indicator 16.1.3: Age-standardised prevalence of physical or sexual violence experienced by populations in the last 12 months (%)				Indicator 16.2.3: Age-standardised prevalence of women and men aged 18-29 years who experienced sexual violence by age 18 (%)			
	**2000**	**2015**	**2016**	**2030**	**2000**	**2015**	**2016**	**2030**	**2000**	**2015**	**2016**	**2030**	**2000**	**2015**	**2016**	**2030**
Bangladesh	3·6 (3·1-5·2)	2·6 (2·0-3·7)	2·5 (1·9-3·7)	1·8 (1·1-3·1)	0·0 (0·0-0·0)	0·0 (0·0-0·0)	0·0 (0·0-0·0)	0·0 (0·0-0·0)	14·8 (13·3-16·5)	12·4 (11·1-13·8)	12·3 (11·0-13·7)	10·6 (9·3-12·0)	20·1 (16·4-24·6)	20·1 (16·3-24·6)	20·1 (16·4-24·6)	20·1 (16·3-24·7)
Bhutan	2·0 (1·2-2·5)	1·3 (0·8-1·7)	1·3 (0·8-1·7)	1·1 (0·5-1·9)	0·0 (0·0-0·0)	0·0 (0·0-0·0)	0·0 (0·0-0·0)	0·0 (0·0-0·0)	10·9 (9·8-12·3)	8·0 (7·2-9·0)	7·9 (7·1-8·9)	6·9 (6·0-7·8)	16·9 (12·9-21·6)	17·0 (12·9-21·8)	17·0 (12·9-21·8)	16·9 (12·8-21·7)
India	4·7 (3·3-5·3)	3·8 (3·1-4·4)	3·8 (3·1-4·4)	3·3 (2·5-4·2)	0·4 (0·4-0·4)	0·0 (0·0-0·0)	0·0 (0·0-0·0)	0·0 (0·0-0·0)	11·8 (10·8-13·0)	10·5 (9·5-11·7)	10·5 (9·5-11·6)	9·5 (8·4-10·8)	15·0 (11·7-18·9)	15·0 (11·7-18·9)	15·0 (11·7-18·9)	15·0 (11·6-19·1)
Nepal	2·6 (1·3-3·1)	2·3 (1·1-3·0)	2·3 (1·1-3·0)	2·1 (0·9-3·4)	1·5 (1·3-1·8)	0·0 (0·0-0·0)	0·0 (0·0-0·0)	0·0 (0·0-0·0)	10·9 (9·7-12·1)	9·2 (8·3-10·2)	9·1 (8·2-10·1)	7·9 (7·1-8·8)	16·9 (12·8-21·5)	16·8 (12·8-21·4)	16·8 (12·8-21·4)	16·7 (12·8-21·2)
Pakistan	5·4 (3·7-6·4)	5·1 (3·2-6·7)	5·0 (3·2-6·6)	4·6 (2·4-7·8)	0·0 (0·0-0·0)	1·2 (1·2-1·3)	0·3 (0·1-0·4)	0·3 (0·1-0·4)	16·0 (14·4-18·0)	13·4 (12·0-14·9)	13·2 (11·9-14·7)	11·0 (10·0-12·3)	17·0 (12·9-21·6)	17·0 (12·9-21·7)	17·0 (12·9-21·7)	17·0 (12·9-21·7)
**Southern Sub-Saharan Africa**																
Botswana	11·8 (3·2-25·8)	12·4 (3·1-20·3)	12·2 (3·1-19·9)	9·3 (1·5-25·0)	0·0 (0·0-0·0)	0·0 (0·0-0·0)	0·0 (0·0-0·0)	0·0 (0·0-0·0)	12·2 (10·5-14·3)	10·9 (9·3-12·8)	10·8 (9·2-12·7)	9·4 (8·0-11·0)	9·0 (6·7-11·9)	9·0 (6·7-11·9)	9·0 (6·7-11·9)	9·0 (6·6-11·9)
Lesotho	37·0 (15·6-52·4)	37·0 (16·2-51·7)	36·5 (16·0-52·2)	36·6 (13·8-65·0)	0·0 (0·0-0·0)	0·0 (0·0-0·0)	0·0 (0·0-0·0)	0·0 (0·0-0·0)	17·7 (15·2-20·7)	15·5 (13·3-18·2)	15·4 (13·2-18·1)	13·9 (11·9-16·3)	9·0 (6·7-11·9)	9·0 (6·7-11·9)	9·0 (6·7-11·9)	9·0 (6·7-11·9)
Namibia	21·3 (13·3-28·4)	17·8 (10·8-24·7)	17·6 (10·6-24·5)	15·0 (6·3-27·5)	5·3 (2·0-8·6)	0·0 (0·0-0·0)	0·0 (0·0-0·0)	0·0 (0·0-0·0)	11·7 (10·5-13·1)	9·7 (8·7-10·7)	9·6 (8·7-10·6)	8·5 (7·7-9·5)	9·0 (6·7-11·9)	9·0 (6·7-11·9)	9·0 (6·7-11·9)	9·0 (6·7-11·9)
South Africa	52·4 (36·9-61·8)	34·2 (27·3-39·5)	34·7 (27·9-40·0)	25·1 (18·2-34·3)	0·0 (0·0-0·0)	0·4 (0·2-0·7)	0·1 (0·05-0·2)	0·1 (0·04-0·3)	9·4 (8·4-10·5)	8·4 (7·5-9·5)	8·4 (7·5-9·4)	7·9 (7·1-8·9)	9·9 (7·7-12·3)	10·0 (7·8-12·5)	10·0 (7·8-12·5)	10·1 (7·8-12·6)
Swaziland	22·4 (13·6-35·0)	19·1 (12·0-30·0)	18·9 (12·0-29·5)	16·6 (6·4-37·5)	0·0 (0·0-0·0)	0·0 (0·0-0·0)	0·0 (0·0-0·0)	0·0 (0·0-0·0)	19·6 (17·6-21·9)	18·1 (16·2-20·1)	17·9 (16·0-19·9)	15·3 (13·7-17·1)	11·4 (8·8-14·6)	10·0 (7·9-12·7)	10·0 (7·9-12·7)	9·8 (7·6-12·5)
Zimbabwe	10·3 (6·6-14·2)	10·8 (7·8-14·4)	11·0 (7·8-14·5)	12·0 (6·2-21·2)	0·3 (0·09-0·4)	0·0 (0·0-0·0)	0·0 (0·0-0·0)	0·0 (0·0-0·0)	14·4 (13·1-15·8)	13·8 (12·6-15·1)	13·6 (12·5-15·0)	12·1 (10·9-13·5)	9·0 (6·7-11·9)	9·0 (6·7-11·9)	9·0 (6·7-11·9)	9·0 (6·7-11·9)
**Western Sub-Saharan Africa**																
Benin	2·9 (2·2-4·1)	3·3 (2·5-4·2)	3·3 (2·5-4·2)	3·5 (2·2-5·3)	0·0 (0·0-0·0)	0·0 (0·0-0·0)	0·0 (0·0-0·0)	0·0 (0·0-0·0)	14·6 (12·7-16·7)	13·4 (11·6-15·3)	13·3 (11·5-15·2)	12·2 (10·6-14·0)	15·4 (11·9-19·4)	15·4 (11·9-19·4)	15·4 (11·9-19·4)	15·4 (11·9-19·4)
Burkina Faso	1·1 (0·8-2·3)	1·1 (0·7-2·2)	1·0 (0·7-2·2)	0·9 (0·5-2·0)	0·0 (0·0-0·0)	0·1 (0·03-0·3)	0·5 (0·07-0·9)	0·5 (0·07-0·9)	11·0 (9·8-12·3)	9·5 (8·5-10·6)	9·4 (8·5-10·5)	8·3 (7·4-9·2)	15·4 (11·9-19·3)	15·4 (11·9-19·3)	15·4 (11·9-19·3)	15·3 (11·9-19·3)
Cameroon	4·0 (2·4-5·1)	3·8 (2·3-5·1)	3·8 (2·3-5·1)	3·8 (1·8-6·3)	0·0 (0·0-0·0)	4·2 (3·5-4·9)	1·6 (0·3-3·0)	1·6 (0·1-6·0)	14·9 (13·4-16·5)	13·8 (12·6-15·0)	13·7 (12·5-14·9)	12·3 (11·2-13·5)	15·4 (11·9-19·3)	15·4 (11·9-19·4)	15·4 (11·9-19·4)	15·4 (11·9-19·4)
Cape Verde	13·2 (9·8-17·6)	14·7 (10·1-18·3)	14·5 (10·0-18·3)	14·0 (7·3-23·4)	0·0 (0·0-0·0)	0·0 (0·0-0·0)	0·0 (0·0-0·0)	0·0 (0·0-0·0)	15·3 (13·3-17·7)	12·6 (11·1-14·6)	12·5 (10·9-14·4)	10·4 (9·1-12·0)	15·4 (11·9-19·3)	15·4 (11·9-19·4)	15·4 (11·9-19·4)	15·4 (11·9-19·4)
Chad	3·0 (2·2-4·5)	3·3 (2·6-4·2)	3·3 (2·6-4·2)	3·4 (2·2-5·1)	14·1 (11·6-16·6)	2·8 (1·2-4·6)	0·2 (0·03-0·3)	0·2 (0·01-1·1)	14·2 (12·8-15·9)	12·6 (11·5-14·0)	12·6 (11·4-13·9)	11·6 (10·3-13·4)	15·4 (11·9-19·3)	15·4 (11·9-19·3)	15·4 (11·9-19·3)	15·4 (11·9-19·3)
Cote d'Ivoire	2·4 (1·1-10·3)	2·5 (1·2-9·5)	2·4 (1·2-9·4)	2·5 (1·0-9·3)	0·8 (0·5-1·0)	0·1 (0·02-0·2)	0·3 (0·04-0·5)	4,044·5 (0·0-37,504·7)	12·8 (11·5-14·3)	11·7 (10·6-13·0)	11·7 (10·6-13·0)	11·1 (10·0-12·3)	14·3 (11·2-18·1)	14·2 (11·0-18·2)	14·2 (11·0-18·2)	14·2 (11·0-18·2)
The Gambia	1·4 (0·6-6·1)	1·4 (0·6-6·3)	1·4 (0·5-6·3)	1·2 (0·3-6·0)	2·1 (0·4-4·1)	0·0 (0·0-0·0)	0·0 (0·0-0·0)	0·0 (0·0-0·0)	10·1 (9·0-11·2)	8·9 (8·0-9·8)	8·8 (7·9-9·8)	8·1 (7·3-9·0)	15·4 (12·0-19·4)	15·4 (11·9-19·4)	15·4 (11·9-19·4)	15·4 (11·9-19·3)
Ghana	4·4 (2·7-11·2)	4·0 (2·3-10·3)	3·9 (2·2-10·2)	3·2 (1·2-9·8)	0·2 (0·03-0·3)	0·0 (0·0-0·0)	0·0 (0·0-0·0)	0·0 (0·0-0·0)	13·5 (12·1-15·0)	12·5 (11·2-13·8)	12·4 (11·1-13·8)	11·1 (9·6-12·6)	15·4 (11·9-19·3)	15·4 (11·9-19·3)	15·4 (11·9-19·3)	15·4 (11·9-19·4)
Guinea	2·7 (2·1-4·0)	3·3 (2·6-4·3)	3·3 (2·6-4·3)	3·6 (2·2-5·5)	3·0 (0·5-5·7)	0·1 (0·02-0·2)	0·0 (0·0-0·0)	0·0 (0·0-0·0)	11·7 (10·4-13·1)	10·9 (9·7-12·3)	10·9 (9·6-12·3)	9·9 (8·7-11·3)	15·4 (11·9-19·3)	15·4 (11·9-19·3)	15·4 (11·9-19·3)	15·3 (11·9-19·3)
Guinea-Bissau	7·4 (5·7-9·5)	7·4 (5·3-9·2)	7·4 (5·3-9·2)	6·9 (4·2-10·3)	0·0 (0·0-0·0)	0·0 (0·0-0·0)	0·0 (0·0-0·0)	0·0 (0·0-0·0)	17·4 (15·2-19·9)	15·9 (13·9-18·3)	15·8 (13·8-18·2)	14·2 (12·4-16·3)	15·4 (11·9-19·4)	15·4 (11·9-19·4)	15·4 (11·9-19·4)	15·4 (11·9-19·4)
Liberia	2·8 (1·6-3·6)	2·8 (1·6-3·6)	2·8 (1·6-3·6)	2·9 (1·4-4·5)	3·6 (0·6-6·7)	0·0 (0·0-0·0)	0·2 (0·04-0·5)	0·2 (0·04-0·4)	19·6 (17·8-21·9)	16·9 (15·4-18·8)	16·9 (15·3-18·8)	16·2 (14·1-18·6)	15·4 (11·9-19·3)	15·4 (11·9-19·3)	15·4 (11·9-19·3)	15·3 (11·9-19·3)
Mali	2·4 (1·8-3·7)	2·6 (1·9-3·5)	2·6 (1·9-3·5)	2·6 (1·5-4·5)	18·4 (3·5-34·6)	2·0 (1·0-2·9)	1·2 (0·2-2·4)	0·8 (0·02-2·1)	19·3 (17·3-21·4)	16·5 (15·1-18·1)	16·4 (15·0-17·9)	14·7 (13·4-16·1)	15·4 (11·9-19·3)	15·3 (11·9-19·3)	15·3 (11·9-19·3)	15·3 (11·9-19·3)
Mauritania	2·2 (1·4-3·4)	2·3 (1·4-3·4)	2·3 (1·4-3·4)	2·5 (1·1-4·7)	0·0 (0·0-0·0)	0·0 (0·0-0·0)	0·0 (0·0-0·0)	0·0 (0·0-0·0)	12·8 (11·1-14·7)	11·4 (9·9-13·1)	11·3 (9·9-13·0)	10·1 (8·8-11·6)	15·4 (11·9-19·3)	15·4 (11·9-19·3)	15·4 (11·9-19·3)	15·4 (11·9-19·3)
Niger	2·5 (1·9-3·7)	2·9 (2·1-3·9)	2·9 (2·1-3·9)	3·1 (1·8-5·3)	0·0 (0·0-0·0)	1·9 (1·1-2·8)	1·0 (0·3-1·8)	1·0 (0·1-1·9)	16·9 (14·6-19·4)	16·0 (13·9-18·4)	15·9 (13·8-18·3)	15·2 (13·1-17·5)	15·5 (12·0-19·5)	15·4 (11·9-19·4)	15·4 (11·9-19·4)	15·4 (11·9-19·3)
Nigeria	0·8 (0·6-1·0)	0·7 (0·5-0·9)	0·7 (0·5-1·0)	0·7 (0·3-1·1)	1·8 (0·3-3·4)	2·0 (1·8-2·2)	1·9 (0·3-3·7)	7·9 (0·06-37·5)	10·8 (9·8-11·9)	8·9 (8·1-9·8)	8·8 (8·0-9·7)	7·5 (6·7-8·5)	16·6 (13·8-19·9)	16·9 (13·6-20·9)	17·0 (13·5-21·1)	17·0 (13·5-21·1)
Sao Tome and Principe	5·3 (2·6-7·2)	5·4 (2·6-7·3)	5·4 (2·8-7·3)	5·5 (2·5-10·3)	0·0 (0·0-0·0)	0·0 (0·0-0·0)	0·0 (0·0-0·0)	0·0 (0·0-0·0)	17·9 (16·1-20·0)	15·9 (14·3-17·7)	15·8 (14·2-17·7)	15·0 (13·1-17·2)	15·4 (11·9-19·4)	15·4 (11·9-19·4)	15·4 (11·9-19·4)	15·4 (11·9-19·5)
Senegal	1·4 (1·1-1·7)	1·5 (1·0-1·8)	1·5 (1·0-1·8)	1·5 (0·9-2·4)	0·5 (0·08-0·9)	0·0 (0·0-0·0)	0·0 (0·0-0·0)	0·0 (0·0-0·0)	9·9 (8·9-11·0)	9·4 (8·4-10·5)	9·3 (8·4-10·4)	8·9 (7·9-10·0)	15·4 (12·0-19·4)	15·4 (11·9-19·4)	15·4 (11·9-19·4)	15·4 (11·9-19·4)

Location	Indicator 17.19.2c: Percentage of well-certified deaths by a vital registration (VR) system among a country's total population (%)															
	**2000**	**2015**	**2016**	**2030**												
Bangladesh	0·0 (0·0-0·0)	0·0 (0·0-0·0)	0·0 (0·0-0·0)	0·0 (0·0-0·0)												
Bhutan	0·0 (0·0-0·0)	0·0 (0·0-0·0)	0·0 (0·0-0·0)	0·0 (0·0-0·0)												
India	14·7 (12·0-18·3)	21·4 (17·5-25·9)	22·1 (18·0-26·5)	34·3 (22·7-46·9)												
Nepal	0·0 (0·0-0·0)	0·0 (0·0-0·0)	0·0 (0·0-0·0)	0·0 (0·0-0·0)												
Pakistan	0·0 (0·0-0·0)	0·0 (0·0-0·0)	0·0 (0·0-0·0)	0·0 (0·0-0·0)												
**Southern Sub-Saharan Africa**																
Botswana	0·0 (0·0-0·0)	0·0 (0·0-0·0)	0·0 (0·0-0·0)	0·0 (0·0-0·0)												
Lesotho	0·0 (0·0-0·0)	0·0 (0·0-0·0)	0·0 (0·0-0·0)	0·0 (0·0-0·0)												
Namibia	0·0 (0·0-0·0)	0·0 (0·0-0·0)	0·0 (0·0-0·0)	0·0 (0·0-0·0)												
South Africa	46·8 (45·4-48·3)	58·1 (56·2-59·9)	58·7 (56·6-60·8)	67·5 (63·5-71·3)												
Swaziland	0·0 (0·0-0·0)	0·0 (0·0-0·0)	0·0 (0·0-0·0)	0·0 (0·0-0·0)												
Zimbabwe	30·6 (25·0-36·7)	28·1 (22·9-34·1)	28·7 (23·4-34·6)	35·4 (24·0-49·1)												
**Western Sub-Saharan Africa**																
Benin	0·0 (0·0-0·0)	0·0 (0·0-0·0)	0·0 (0·0-0·0)	0·0 (0·0-0·0)												
Burkina Faso	0·0 (0·0-0·0)	0·0 (0·0-0·0)	0·0 (0·0-0·0)	0·0 (0·0-0·0)												
Cameroon	0·0 (0·0-0·0)	0·0 (0·0-0·0)	0·0 (0·0-0·0)	0·0 (0·0-0·0)												
Cape Verde	43·2 (35·6-50·9)	64·3 (57·9-70·2)	64·9 (58·2-70·9)	79·4 (70·2-86·8)												
Chad	0·0 (0·0-0·0)	0·0 (0·0-0·0)	0·0 (0·0-0·0)	0·0 (0·0-0·0)												
Cote d'Ivoire	0·0 (0·0-0·0)	0·0 (0·0-0·0)	0·0 (0·0-0·0)	0·0 (0·0-0·0)												
The Gambia	0·0 (0·0-0·0)	0·0 (0·0-0·0)	0·0 (0·0-0·0)	0·0 (0·0-0·0)												
Ghana	10·1 (7·6-13·0)	24·0 (18·1-30·0)	24·4 (18·8-30·7)	35·7 (23·4-50·3)												
Guinea	0·0 (0·0-0·0)	0·0 (0·0-0·0)	0·0 (0·0-0·0)	0·0 (0·0-0·0)												
Guinea-Bissau	0·0 (0·0-0·0)	0·0 (0·0-0·0)	0·0 (0·0-0·0)	0·0 (0·0-0·0)												
Liberia	0·0 (0·0-0·0)	0·0 (0·0-0·0)	0·0 (0·0-0·0)	0·0 (0·0-0·0)												
Mali	4·3 (3·1-5·6)	17·1 (12·9-22·6)	17·4 (13·1-22·8)	28·2 (17·5-41·7)												
Mauritania	0·0 (0·0-0·0)	0·0 (0·0-0·0)	0·0 (0·0-0·0)	0·0 (0·0-0·0)												
Niger	0·0 (0·0-0·0)	0·0 (0·0-0·0)	0·0 (0·0-0·0)	0·0 (0·0-0·0)												
Nigeria	1·0 (0·7-1·4)	9·5 (7·0-12·6)	9·7 (7·1-12·8)	31·9 (18·7-47·5)												
Sao Tome and Principe	10·5 (7·6-13·6)	34·9 (27·9-42·4)	35·5 (28·6-42·9)	52·9 (40·1-67·4)												
Senegal	0·0 (0·0-0·0)	0·0 (0·0-0·0)	0·0 (0·0-0·0)	0·0 (0·0-0·0)												

Location	Indicator 1.5.1: Death rate due to exposure to forces of nature (per 100,000 population)				Indicator 2.2.1: Prevalence of stunting in children under 5 (%)				Indicator 2.2.2a: Prevalence of wasting in children under 5 (%)				Indicator 2.2.2b: Prevalence of overweight in children aged 2-4 (%)			
	**2000**	**2015**	**2016**	**2030**	**2000**	**2015**	**2016**	**2030**	**2000**	**2015**	**2016**	**2030**	**2000**	**2015**	**2016**	**2030**
Sierra Leone	0·09<br>(0·04-0·1)	0·2<br>(0·1-0·4)	0·2<br>(0·08-0·3)	0·2<br>(0·09-0·3)	39·6<br>(34·8-46·1)	37·7<br>(29·0-46·8)	36·8<br>(27·5-46·6)	31·6<br>(23·6-42·2)	12·4<br>(11·1-13·7)	8·6<br>(7·3-9·8)	8·5<br>(7·1-9·8)	7·0<br>(5·2-8·3)	13·6<br>(9·0-18·7)	21·1<br>(14·7-29·2)	21·4<br>(15·0-29·6)	30·5<br>(16·4-49·4)
Togo	0·0<br>(0·0-0·0)	0·06<br>(0·02-0·1)	0·04<br>(0·01-0·06)	0·06<br>(0·02-0·09)	33·0<br>(25·9-40·7)	21·2<br>(15·7-27·4)	20·1<br>(14·4-26·6)	13·1<br>(10·2-17·6)	11·8<br>(10·3-13·4)	7·0<br>(5·9-8·1)	6·8<br>(5·8-8·0)	5·1<br>(3·8-6·8)	8·0<br>(5·8-10·5)	9·7<br>(6·1-14·3)	9·9<br>(6·3-14·7)	13·9<br>(5·9-27·1)
**Eastern Sub-Saharan Africa**																
Burundi	0·0<br>(0·0-0·0)	0·2<br>(0·1-0·3)	0·3<br>(0·2-0·4)	0·2<br>(0·1-0·3)	59·0<br>(53·0-65·7)	51·7<br>(42·5-61·7)	50·9<br>(41·3-61·2)	41·9<br>(31·9-54·3)	9·4<br>(8·2-10·8)	6·1<br>(4·1-9·0)	6·1<br>(3·9-9·3)	4·9<br>(2·1-8·2)	11·7<br>(8·4-15·8)	9·4<br>(5·8-14·5)	9·5<br>(5·6-14·7)	8·1<br>(2·7-18·1)
Comoros	0·0<br>(0·0-0·0)	0·0<br>(0·0-0·0)	0·0<br>(0·0-0·0)	0·0<br>(0·0-0·0)	47·8<br>(43·3-54·2)	30·5<br>(23·8-38·5)	29·6<br>(23·0-37·8)	19·5<br>(12·7-27·5)	12·9<br>(10·9-15·0)	10·4<br>(7·5-14·0)	10·2<br>(7·3-13·9)	8·0<br>(4·8-13·3)	24·6<br>(17·6-33·0)	28·3<br>(18·5-39·4)	28·6<br>(18·6-39·9)	33·2<br>(15·3-53·8)
Djibouti	1·6<br>(0·8-2·3)	0·0<br>(0·0-0·0)	0·0<br>(0·0-0·0)	0·3<br>(0·2-0·4)	30·6<br>(25·1-36·1)	27·9<br>(22·1-34·0)	27·1<br>(21·1-33·4)	28·2<br>(20·0-33·7)	20·5<br>(17·0-24·6)	21·5<br>(16·7-27·0)	21·2<br>(16·1-27·2)	19·8<br>(13·6-28·7)	13·6<br>(8·4-21·1)	26·1<br>(17·2-37·2)	26·8<br>(17·5-37·3)	42·5<br>(21·1-66·1)
Eritrea	0·0<br>(0·0-0·0)	0·0<br>(0·0-0·0)	0·0<br>(0·0-0·0)	0·0<br>(0·0-0·0)	46·3<br>(40·4-53·7)	31·2<br>(22·9-42·6)	30·5<br>(22·3-41·7)	19·9<br>(11·1-32·7)	16·2<br>(14·6-18·0)	11·8<br>(6·9-19·5)	11·7<br>(6·8-19·9)	9·8<br>(3·4-23·3)	5·6<br>(3·6-8·2)	7·5<br>(4·4-12·0)	7·7<br>(4·5-12·4)	10·1<br>(3·7-22·8)
Ethiopia	0·07<br>(0·04-0·1)	0·02<br>(0·01-0·03)	0·0<br>(0·0-0·0)	0·07<br>(0·04-0·1)	49·6<br>(45·0-55·4)	41·9<br>(36·8-47·8)	41·4<br>(36·3-47·5)	35·5<br>(29·7-42·8)	12·8<br>(11·5-14·2)	10·9<br>(9·4-12·5)	10·9<br>(9·4-12·6)	10·8<br>(8·5-13·5)	6·2<br>(4·0-9·4)	13·0<br>(8·4-18·8)	13·4<br>(8·6-20·2)	21·8<br>(9·6-40·7)
Kenya	0·06<br>(0·03-0·08)	0·2<br>(0·1-0·2)	0·2<br>(0·1-0·2)	0·2<br>(0·1-0·2)	38·8<br>(37·6-39·9)	24·7<br>(23·5-25·8)	23·9<br>(22·8-25·0)	15·4<br>(14·6-16·2)	7·1<br>(6·7-7·5)	5·6<br>(5·2-6·1)	5·5<br>(5·1-6·0)	4·6<br>(4·2-5·0)	13·1<br>(10·0-16·8)	13·2<br>(10·1-16·7)	13·5<br>(10·0-17·6)	16·0<br>(8·2-27·1)
Madagascar	0·5<br>(0·3-0·7)	0·3<br>(0·2-0·4)	0·2<br>(0·1-0·4)	0·4<br>(0·2-0·5)	50·4<br>(44·4-57·6)	45·5<br>(37·5-54·5)	44·7<br>(36·4-54·1)	35·5<br>(27·1-48·0)	13·0<br>(10·7-15·6)	29·5<br>(22·1-37·4)	29·2<br>(21·2-37·9)	43·8<br>(37·1-51·2)	7·6<br>(5·8-10·1)	10·8<br>(6·2-17·0)	11·0<br>(6·2-17·1)	13·6<br>(4·8-28·0)
Malawi	0·09<br>(0·05-0·1)	0·3<br>(0·2-0·4)	0·3<br>(0·1-0·4)	0·2<br>(0·09-0·2)	54·3<br>(48·7-60·9)	37·9<br>(32·7-44·5)	37·0<br>(31·7-43·8)	25·8<br>(19·6-34·4)	8·1<br>(7·2-9·0)	3·6<br>(2·9-4·3)	3·4<br>(2·8-4·2)	2·0<br>(1·4-3·0)	26·3<br>(22·1-31·6)	17·9<br>(13·0-24·2)	18·3<br>(12·8-24·9)	14·3<br>(6·8-26·0)
Mozambique	1·1<br>(0·6-1·5)	0·2<br>(0·1-0·3)	0·2<br>(0·1-0·3)	0·2<br>(0·1-0·3)	48·0<br>(41·6-55·4)	31·4<br>(23·8-40·9)	29·8<br>(22·0-39·8)	21·2<br>(14·5-30·6)	9·0<br>(7·6-10·7)	4·7<br>(3·3-6·5)	4·7<br>(3·1-6·5)	3·4<br>(1·6-5·3)	17·2<br>(13·1-22·0)	18·8<br>(13·1-25·6)	19·2<br>(13·0-26·5)	22·9<br>(10·8-40·9)
Rwanda	0·0<br>(0·0-0·0)	0·06<br>(0·03-0·09)	0·1<br>(0·06-0·2)	0·1<br>(0·08-0·2)	48·4<br>(43·6-54·5)	36·8<br>(30·4-45·4)	35·8<br>(29·2-44·7)	23·4<br>(16·7-34·4)	8·5<br>(7·0-10·3)	3·4<br>(2·1-5·1)	3·3<br>(2·0-5·2)	2·7<br>(1·1-5·4)	17·9<br>(14·9-21·3)	19·9<br>(15·9-24·1)	20·6<br>(15·7-26·0)	25·0<br>(14·8-37·7)
Somalia	4·9<br>(3·1-6·6)	0·3<br>(0·2-0·4)	0·3<br>(0·2-0·3)	0·7<br>(0·5-1·0)	32·9<br>(28·9-38·8)	30·1<br>(22·7-39·8)	30·1<br>(22·4-39·8)	28·8<br>(18·5-40·3)	19·6<br>(18·2-21·1)	16·7<br>(10·7-24·0)	16·8<br>(10·4-24·8)	17·8<br>(7·1-32·4)	8·7<br>(4·9-14·2)	10·1<br>(5·9-15·9)	10·2<br>(6·0-16·2)	11·8<br>(4·2-26·4)
South Sudan	0·2<br>(0·05-0·3)	0·2<br>(0·09-0·3)	0·2<br>(0·07-0·2)	0·1<br>(0·06-0·2)	43·9<br>(39·4-49·1)	32·1<br>(25·7-38·3)	32·2<br>(25·1-39·2)	28·7<br>(17·0-33·5)	20·8<br>(16·8-25·1)	20·1<br>(15·2-25·4)	19·7<br>(14·3-25·9)	14·7<br>(8·9-27·4)	15·5<br>(9·6-23·6)	16·3<br>(9·4-25·5)	16·5<br>(9·3-25·9)	18·3<br>(6·7-37·5)
Tanzania	0·1<br>(0·05-0·1)	0·03<br>(0·02-0·04)	0·02<br>(0·01-0·04)	0·03<br>(0·02-0·04)	49·8<br>(43·0-58·2)	35·0<br>(28·9-43·3)	33·7<br>(27·6-42·3)	20·5<br>(16·2-29·5)	6·5<br>(5·2-8·0)	5·4<br>(4·1-7·1)	5·4<br>(4·0-7·2)	4·5<br>(2·7-7·2)	11·6<br>(8·7-15·0)	10·9<br>(7·8-15·1)	11·3<br>(7·7-15·9)	13·7<br>(6·1-25·7)
Uganda	0·07<br>(0·04-0·1)	0·1<br>(0·07-0·2)	0·1<br>(0·06-0·2)	0·1<br>(0·06-0·1)	45·9<br>(40·1-53·6)	30·4<br>(24·7-37·8)	29·5<br>(23·5-37·1)	18·5<br>(12·8-26·8)	5·8<br>(4·8-6·7)	4·7<br>(3·6-5·8)	4·6<br>(3·4-5·8)	3·6<br>(2·4-5·5)	15·7<br>(12·7-19·2)	13·5<br>(9·7-18·2)	13·9<br>(9·6-19·6)	11·5<br>(5·4-20·5)
Zambia	0·0<br>(0·0-0·0)	0·02<br>(0·01-0·02)	0·01<br>(0·01-0·02)	0·01<br>(0·01-0·02)	55·8<br>(50·3-62·0)	41·3<br>(34·3-49·9)	40·7<br>(33·6-49·4)	31·8<br>(24·8-41·6)	6·1<br>(5·3-6·9)	6·1<br>(4·8-7·8)	6·1<br>(4·8-7·9)	6·6<br>(4·7-8·9)	29·4<br>(24·7-34·5)	35·6<br>(25·3-45·7)	35·9<br>(24·8-47·1)	42·2<br>(22·7-63·3)
**Central Sub-Saharan Africa**																
Angola	0·07<br>(0·01-0·1)	0·2<br>(0·03-0·3)	0·2<br>(0·03-0·3)	0·2<br>(0·02-0·3)	51·8<br>(46·5-58·4)	30·8<br>(23·1-42·7)	30·1<br>(22·4-42·3)	22·0<br>(14·6-32·3)	13·7<br>(11·6-15·7)	8·7<br>(7·0-10·5)	8·4<br>(6·8-10·2)	5·4<br>(4·1-6·8)	15·1<br>(7·0-26·0)	26·4<br>(13·0-43·8)	27·3<br>(13·2-45·3)	40·5<br>(12·3-74·8)
Central African Republic	0·0<br>(0·0-0·0)	0·0<br>(0·0-0·0)	0·0<br>(0·0-0·0)	0·0<br>(0·0-0·0)	46·0<br>(42·1-51·8)	39·0<br>(31·7-47·8)	38·5<br>(30·7-47·6)	30·9<br>(23·0-44·3)	10·0<br>(8·9-11·1)	8·5<br>(7·1-9·9)	8·4<br>(7·0-9·8)	6·8<br>(5·0-8·7)	17·6<br>(9·9-28·2)	13·0<br>(5·3-24·6)	12·8<br>(5·3-24·0)	11·6<br>(1·9-31·4)
Congo	0·0<br>(0·0-0·0)	0·04<br>(0·01-0·08)	0·04<br>(0·01-0·07)	0·03<br>(0·0-0·05)	27·0<br>(21·2-33·7)	20·8<br>(16·6-25·5)	20·1<br>(15·9-24·8)	12·2<br>(8·9-16·4)	7·4<br>(6·3-8·6)	7·6<br>(6·6-8·6)	7·5<br>(6·6-8·6)	7·5<br>(6·7-8·6)	17·6<br>(9·6-28·7)	12·4<br>(6·1-22·7)	13·0<br>(6·1-24·4)	14·8<br>(3·1-37·2)
Democratic Republic of the Congo	0·0<br>(0·0-0·0)	0·04<br>(0·01-0·07)	0·03<br>(0·01-0·06)	0·04<br>(0·01-0·07)	45·1<br>(40·7-50·9)	39·2<br>(33·2-47·0)	38·4<br>(32·1-46·5)	36·7<br>(29·4-43·6)	14·5<br>(13·1-15·7)	8·6<br>(7·4-9·8)	8·4<br>(7·2-9·7)	5·8<br>(4·5-7·4)	17·3<br>(10·5-26·8)	15·8<br>(7·5-27·9)	16·0<br>(7·5-29·1)	16·8<br>(3·7-41·8)
Equatorial Guinea	0·0<br>(0·0-0·0)	0·0<br>(0·0-0·0)	0·0<br>(0·0-0·0)	0·0<br>(0·0-0·0)	47·5<br>(42·4-54·5)	21·8<br>(17·0-26·8)	21·1<br>(15·9-26·7)	11·4<br>(6·2-16·1)	5·3<br>(4·6-6·1)	3·4<br>(2·9-4·0)	3·3<br>(2·8-3·9)	2·1<br>(1·6-2·8)	29·1<br>(16·3-43·5)	55·0<br>(34·0-74·4)	55·4<br>(34·3-75·2)	72·3<br>(37·2-92·5)
Gabon	0·0<br>(0·0-0·0)	0·0<br>(0·0-0·0)	0·0<br>(0·0-0·0)	0·0<br>(0·0-0·0)	25·8<br>(22·9-29·4)	16·8<br>(12·7-21·5)	16·4<br>(12·3-21·2)	11·4<br>(7·5-16·6)	4·7<br>(4·0-5·6)	3·9<br>(3·3-4·7)	3·9<br>(3·2-4·7)	3·3<br>(2·7-4·0)	13·9<br>(9·1-19·7)	18·1<br>(10·8-27·3)	18·8<br>(10·8-29·5)	23·0<br>(7·5-46·2)

Location	Indicator 3.1.1: Maternal mortality ratio (maternal deaths per 100,000 livebirths) in women aged 10-54 years				Indicator 3.1.2: Proportion of births attended by skilled health personnel (%)				Indicator 3.2.1: Under-5 mortality rate (probability of dying before the age of 5 per 1,000 livebirths)				Indicator 3.2.2: Neonatal mortality rate (probability of dying during the first 28 days of life per 1,000 livebirths)			
	**2000**	**2015**	**2016**	**2030**	**2000**	**2015**	**2016**	**2030**	**2000**	**2015**	**2016**	**2030**	**2000**	**2015**	**2016**	**2030**
Sierra Leone	699·6<br>(579·7-823·3)	537·3<br>(441·1-642·0)	523·4<br>(428·9-630·1)	377·9<br>(228·2-566·6)	44·5<br>(37·2-52·3)	54·1<br>(43·1-65·0)	55·4<br>(42·5-67·5)	67·3<br>(36·0-91·2)	215·2<br>(202·9-226·6)	122·4<br>(108·2-137·9)	115·6<br>(101·5-131·2)	64·2<br>(48·5-82·9)	54·8<br>(52·0-57·6)	35·9<br>(32·2-40·0)	34·8<br>(31·0-39·0)	23·0<br>(17·9-29·2)
Togo	472·0<br>(341·2-561·9)	287·6<br>(223·4-359·3)	278·5<br>(216·1-348·5)	178·1<br>(98·6-285·0)	53·4<br>(47·8-58·9)	63·4<br>(54·0-72·5)	64·9<br>(53·9-75·3)	73·4<br>(40·0-95·2)	122·4<br>(113·7-131·3)	72·1<br>(62·8-83·6)	69·0<br>(59·7-80·8)	37·1<br>(27·2-50·1)	36·2<br>(33·1-39·4)	26·1<br>(22·5-30·7)	25·1<br>(21·5-29·7)	16·6<br>(11·8-22·8)
**Eastern Sub-Saharan Africa**																
Burundi	810·3<br>(635·3-1,025·2)	350·7<br>(254·2-469·4)	346·3<br>(247·3-469·9)	229·4<br>(109·3-459·8)	30·7<br>(23·0-39·6)	69·6<br>(50·7-84·9)	68·5<br>(46·9-85·4)	82·6<br>(35·8-100·0)	164·3<br>(145·6-182·8)	83·3<br>(59·1-117·0)	80·8<br>(56·5-116·7)	51·6<br>(21·2-114·9)	43·2<br>(37·2-49·4)	28·0<br>(18·5-42·5)	27·3<br>(17·6-42·5)	20·5<br>(7·5-49·6)
Comoros	324·8<br>(262·1-390·1)	170·8<br>(119·4-229·0)	167·1<br>(116·3-225·1)	123·8<br>(58·0-230·5)	70·6<br>(65·6-75·3)	85·7<br>(77·2-91·8)	85·4<br>(75·3-92·7)	90·7<br>(65·2-100·0)	97·7<br>(86·5-110·2)	55·2<br>(42·6-72·0)	52·6<br>(39·9-70·6)	28·0<br>(14·3-50·9)	41·0<br>(35·3-47·3)	27·5<br>(22·9-33·1)	26·4<br>(21·6-32·1)	18·2<br>(10·4-29·4)
Djibouti	397·7<br>(264·7-523·2)	306·1<br>(196·3-424·0)	292·3<br>(183·6-406·2)	208·0<br>(96·8-354·2)	83·5<br>(70·0-93·0)	92·5<br>(81·5-97·8)	92·5<br>(81·7-97·8)	94·5<br>(68·2-100·0)	102·4<br>(91·4-113·6)	51·9<br>(42·9-64·0)	48·0<br>(39·2-59·8)	23·5<br>(16·1-33·8)	30·1<br>(26·5-33·9)	19·2<br>(15·5-24·0)	18·0<br>(14·4-22·9)	11·2<br>(7·4-16·5)
Eritrea	649·9<br>(525·2-775·8)	606·5<br>(454·9-777·2)	601·3<br>(451·2-766·6)	603·3<br>(349·7-969·2)	29·0<br>(25·6-32·2)	31·2<br>(12·0-56·3)	32·1<br>(12·1-58·7)	37·6<br>(4·2-82·9)	92·8<br>(80·8-105·5)	54·0<br>(42·8-68·7)	51·2<br>(40·5-65·8)	28·4<br>(17·6-46·9)	25·3<br>(21·0-30·0)	18·5<br>(14·0-24·5)	17·7<br>(13·4-23·8)	12·5<br>(7·2-21·9)
Ethiopia	817·7<br>(666·2-975·2)	366·1<br>(260·3-494·8)	357·1<br>(248·7-487·4)	187·8<br>(78·3-364·0)	13·1<br>(9·1-17·8)	23·4<br>(10·5-41·7)	24·5<br>(10·4-44·5)	44·5<br>(7·9-93·1)	140·1<br>(131·6-148·9)	46·6<br>(40·1-55·0)	43·4<br>(37·0-51·7)	15·4<br>(11·2-21·0)	42·8<br>(39·7-46·1)	20·0<br>(16·8-24·2)	18·8<br>(15·7-23·0)	9·1<br>(6·4-13·1)
Kenya	392·3<br>(311·6-474·0)	258·0<br>(196·7-335·1)	257·6<br>(195·1-335·3)	165·1<br>(101·7-258·5)	48·9<br>(43·0-54·9)	66·4<br>(54·9-77·1)	67·3<br>(53·7-78·8)	82·8<br>(53·9-98·4)	90·3<br>(84·2-96·6)	44·6<br>(38·2-51·9)	43·4<br>(36·9-51·2)	23·1<br>(15·6-33·0)	27·7<br>(25·3-30·3)	18·4<br>(15·6-21·7)	18·0<br>(15·0-21·3)	12·5<br>(8·2-18·3)
Madagascar	432·1<br>(369·2-505·1)	374·4<br>(263·7-511·4)	362·8<br>(248·4-506·1)	278·8<br>(122·2-522·7)	46·2<br>(41·5-50·9)	29·2<br>(18·2-43·7)	30·5<br>(18·0-47·2)	25·9<br>(5·5-56·2)	117·3<br>(104·8-129·2)	82·8<br>(65·5-104·0)	79·7<br>(63·4-101·9)	53·4<br>(28·8-89·5)	33·7<br>(30·0-37·2)	27·5<br>(22·4-33·6)	26·7<br>(21·8-33·2)	20·5<br>(11·3-33·7)
Malawi	557·7<br>(326·0-797·5)	265·1<br>(176·0-365·1)	258·0<br>(169·2-361·8)	163·9<br>(65·7-352·3)	56·6<br>(52·6-60·7)	87·4<br>(80·7-92·2)	87·9<br>(80·1-93·1)	96·7<br>(86·5-99·8)	165·2<br>(151·7-178·8)	73·1<br>(59·7-90·8)	69·0<br>(55·3-87·4)	30·7<br>(17·1-52·6)	39·7<br>(36·6-43·1)	25·4<br>(20·8-31·5)	24·2<br>(19·5-30·6)	15·2<br>(8·6-25·2)
Mozambique	367·8<br>(221·9-486·7)	296·2<br>(205·8-380·6)	288·7<br>(208·1-369·6)	237·6<br>(106·1-470·2)	48·9<br>(46·5-51·3)	62·1<br>(47·5-75·4)	63·6<br>(47·2-78·4)	74·9<br>(40·0-96·4)	161·5<br>(153·4-170·2)	80·5<br>(70·0-93·4)	76·1<br>(65·9-89·2)	39·4<br>(29·2-53·2)	42·1<br>(39·2-45·1)	25·8<br>(22·9-29·4)	24·6<br>(21·6-28·2)	15·7<br>(12·2-20·1)
Rwanda	566·9<br>(446·2-719·7)	237·0<br>(168·7-317·1)	232·9<br>(164·1-316·6)	105·7<br>(48·3-198·4)	26·2<br>(20·2-32·7)	90·7<br>(84·9-94·8)	90·8<br>(84·1-95·2)	99·2<br>(95·2-100·0)	156·2<br>(142·4-170·2)	51·5<br>(42·2-62·9)	49·2<br>(39·6-62·3)	22·0<br>(12·1-38·4)	39·6<br>(35·6-44·1)	18·7<br>(15·8-22·3)	18·0<br>(14·9-22·0)	10·5<br>(6·1-17·7)
Somalia	1,064·1<br>(748·5-1,394·3)	850·4<br>(605·2-1,164·7)	836·6<br>(593·8-1,146·2)	671·3<br>(384·6-1,080·3)	30·3<br>(16·3-47·3)	42·7<br>(28·7-57·6)	43·0<br>(26·9-60·6)	48·0<br>(12·5-79·6)	155·6<br>(141·2-170·5)	99·4<br>(83·1-120·1)	97·2<br>(81·3-117·8)	65·1<br>(47·6-90·4)	39·5<br>(36·1-43·0)	29·5<br>(25·1-34·9)	28·8<br>(24·5-34·4)	21·6<br>(16·1-29·0)
South Sudan	454·1<br>(300·0-592·5)	373·5<br>(273·7-493·4)	370·7<br>(275·1-488·3)	333·2<br>(197·7-499·8)	16·5<br>(6·6-32·1)	41·8<br>(26·4-59·9)	41·1<br>(24·1-61·7)	63·8<br>(13·6-100·0)	143·6<br>(131·3-156·0)	97·0<br>(77·0-120·2)	93·5<br>(73·4-117·4)	66·9<br>(39·8-105·9)	39·2<br>(34·3-44·2)	31·5<br>(24·0-40·6)	30·7<br>(23·0-40·3)	25·7<br>(13·9-43·0)
Tanzania	471·6<br>(370·2-570·3)	369·3<br>(278·8-462·1)	357·8<br>(269·7-450·0)	282·1<br>(170·3-458·8)	41·7<br>(37·5-45·8)	58·9<br>(43·6-73·4)	59·9<br>(43·5-75·3)	76·2<br>(39·9-99·8)	123·8<br>(117·8-130·0)	58·6<br>(51·0-67·8)	55·7<br>(48·2-65·3)	27·1<br>(19·6-37·7)	34·1<br>(30·8-37·9)	21·9<br>(19·4-25·2)	21·0<br>(18·4-24·4)	13·2<br>(9·3-18·4)
Uganda	285·2<br>(224·5-369·5)	233·7<br>(172·0-310·5)	225·4<br>(164·1-307·7)	170·1<br>(84·2-331·7)	38·8<br>(35·1-42·3)	64·7<br>(50·7-77·1)	64·6<br>(48·1-78·6)	74·0<br>(40·2-94·2)	134·5<br>(128·3-140·7)	65·9<br>(58·3-75·0)	62·4<br>(55·0-71·8)	29·3<br>(22·0-38·7)	34·9<br>(33·2-36·5)	23·3<br>(20·6-26·7)	22·3<br>(19·6-25·8)	14·3<br>(10·6-19·3)
Zambia	463·4<br>(319·5-634·4)	189·3<br>(109·8-303·6)	183·5<br>(101·7-298·3)	86·8<br>(21·4-226·8)	48·7<br>(44·9-52·5)	73·5<br>(63·6-81·8)	75·4<br>(63·8-85·0)	92·3<br>(74·9-100·0)	142·3<br>(128·6-155·2)	61·9<br>(49·4-75·8)	59·7<br>(47·7-73·9)	27·3<br>(14·9-47·1)	33·6<br>(30·1-37·3)	21·5<br>(17·0-26·8)	20·9<br>(16·5-26·3)	14·0<br>(7·7-23·6)
**Central Sub-Saharan Africa**																
Angola	365·8<br>(266·7-504·6)	195·9<br>(134·0-289·6)	183·5<br>(124·0-274·0)	85·6<br>(35·7-170·3)	45·6<br>(41·2-50·1)	71·4<br>(49·6-87·7)	72·8<br>(50·7-89·0)	86·5<br>(43·4-100·0)	151·2<br>(132·3-172·1)	59·7<br>(44·6-77·7)	54·5<br>(40·4-71·8)	17·1<br>(7·2-33·4)	37·2<br>(32·7-42·3)	19·5<br>(14·6-25·4)	18·1<br>(13·4-23·9)	8·1<br>(3·5-15·4)
Central African Republic	935·7<br>(640·8-1,355·2)	814·1<br>(544·8-1,201·7)	808·4<br>(534·8-1,196·7)	722·7<br>(310·9-1,484·3)	46·0<br>(42·6-49·4)	52·1<br>(37·2-67·4)	51·7<br>(34·4-69·5)	48·3<br>(11·8-81·1)	179·3<br>(152·2-210·1)	135·0<br>(100·9-177·7)	130·5<br>(97·2-176·9)	98·0<br>(43·9-190·8)	47·3<br>(40·2-55·4)	40·6<br>(30·5-52·7)	39·6<br>(29·9-53·0)	34·8<br>(15·5-67·5)
Congo	576·8<br>(434·5-745·8)	415·4<br>(276·6-586·0)	404·6<br>(263·9-576·2)	282·3<br>(111·0-596·8)	88·5<br>(83·5-92·9)	95·2<br>(91·2-97·9)	95·6<br>(91·2-98·2)	98·5<br>(92·2-100·0)	103·4<br>(92·0-115·7)	55·2<br>(40·6-76·2)	52·8<br>(37·8-74·9)	28·7<br>(12·9-59·5)	28·5<br>(25·2-31·9)	19·6<br>(14·4-27·1)	18·9<br>(13·4-26·6)	12·5<br>(5·5-25·2)
Democratic Republic of the Congo	404·9<br>(336·1-488·3)	465·2<br>(380·2-558·5)	442·0<br>(360·8-534·0)	378·7<br>(209·2-606·6)	48·8<br>(28·2-69·6)	83·6<br>(70·5-92·2)	83·9<br>(70·1-92·8)	94·4<br>(74·5-100·0)	152·4<br>(131·9-176·3)	84·9<br>(62·8-111·0)	80·5<br>(58·5-107·7)	46·9<br>(20·2-98·9)	37·1<br>(32·1-42·9)	26·1<br>(19·3-34·2)	24·9<br>(18·2-33·3)	17·8<br>(7·5-38·4)
Equatorial Guinea	630·0<br>(422·6-868·0)	227·5<br>(108·4-374·8)	219·4<br>(104·1-362·8)	97·7<br>(27·8-227·6)	66·9<br>(50·4-80·3)	87·6<br>(71·5-96·5)	88·6<br>(73·0-96·9)	94·7<br>(71·8-100·0)	146·8<br>(118·8-177·1)	59·7<br>(41·2-87·5)	57·0<br>(38·6-85·0)	29·5<br>(14·9-56·0)	39·2<br>(30·6-49·2)	21·0<br>(15·2-29·2)	20·3<br>(14·5-28·5)	12·0<br>(6·6-21·4)
Gabon	445·9<br>(348·0-560·4)	228·2<br>(161·2-317·8)	221·8<br>(154·1-309·4)	145·8<br>(57·1-304·8)	87·0<br>(84·2-89·4)	92·2<br>(87·3-95·6)	92·2<br>(86·1-96·2)	94·9<br>(79·4-100·0)	69·9<br>(63·5-76·5)	45·3<br>(37·3-55·9)	43·7<br>(35·5-54·6)	26·0<br>(17·0-38·6)	25·7<br>(23·4-28·2)	19·8<br>(16·0-25·2)	19·2<br>(15·4-24·5)	13·1<br>(8·0-19·9)

Location	Indicator 3.3.1: Age-standardised rate of new HIV infections (per 1,000 population)				Indicator 3.3.2: Age-standardised rate of tuberculosis cases (per 100,000 population)				Indicator 3.3.3: Age-standardised rate of malaria cases (per 1,000 population)				Indicator 3.3.4: Age-standardised rate of hepatitis B incidence (per 100,000 population)			
	**2000**	**2015**	**2016**	**2030**	**2000**	**2015**	**2016**	**2030**	**2000**	**2015**	**2016**	**2030**	**2000**	**2015**	**2016**	**2030**
Sierra Leone	1·3 (1·0-1·7)	0·5 (0·2-0·8)	0·5 (0·2-0·8)	0·4 (0·1-0·6)	362·4 (307·8-422·1)	287·5 (240·7-339·6)	277·3 (232·2-327·3)	209·5 (172·0-250·3)	319·1 (205·2-453·3)	282·6 (180·7-398·0)	272·5 (171·5-386·0)	297·8 (179·6-410·9)	7,845·8 (5,592·4-11,081·2)	5,459·5 (3,888·8-7,714·9)	5,321·6 (3,791·6-7,513·1)	3,713·0 (2,631·4-5,244·2)
Togo	4·0 (2·9-5·3)	0·9 (0·6-1·4)	1·0 (0·5-1·5)	0·7 (0·4-1·2)	310·5 (261·7-366·2)	231·6 (194·9-273·2)	225·8 (189·9-266·4)	175·2 (146·0-209·7)	320·4 (259·4-397·7)	289·5 (240·8-352·7)	286·2 (238·5-347·8)	243·0 (201·0-294·9)	5,914·5 (4,165·9-8,559·8)	4,349·3 (3,075·8-6,261·6)	4,225·0 (2,985·4-6,094·3)	3,275·3 (2,248·4-4,751·9)
**Eastern Sub-Saharan Africa**																
Burundi	1·6 (0·9-2·5)	0·4 (0·06-1·2)	0·4 (0·04-1·5)	0·3 (0·02-1·2)	734·8 (607·1-883·0)	487·8 (409·3-586·7)	483·8 (406·7-581·6)	389·5 (319·7-473·3)	288·2 (193·5-399·5)	123·2 (86·0-168·8)	116·6 (81·1-159·4)	54·0 (35·9-72·3)	5,013·1 (3,798·8-6,530·6)	3,520·0 (2,695·1-4,581·2)	3,457·3 (2,646·2-4,495·0)	2,670·8 (2,045·0-3,367·7)
Comoros	0·07 (0·008-0·3)	0·09 (0·007-0·4)	0·1 (0·009-0·5)	0·1 (0·01-0·5)	262·4 (217·3-315·9)	230·8 (190·1-279·7)	231·9 (191·1-281·0)	246·0 (199·9-300·4)	40·3 (15·1-92·5)	5·1 (2·9-7·3)	2·8 (1·6-4·0)	3·5 (0·9-7·2)	3,639·0 (2,746·7-4,839·7)	2,618·4 (2,018·1-3,409·9)	2,556·8 (1,971·7-3,340·0)	1,848·3 (1,447·0-2,365·6)
Djibouti	2·7 (1·4-4·9)	0·8 (0·3-1·6)	0·8 (0·2-1·6)	0·5 (0·2-1·1)	410·0 (357·3-466·2)	462·2 (400·7-527·8)	458·1 (397·7-523·5)	451·8 (387·2-520·3)	4·1 (2·9-5·8)	11·0 (3·9-23·9)	15·6 (5·1-36·2)	8·4 (2·2-17·5)	3,478·3 (2,666·1-4,469·6)	2,411·8 (1,856·2-3,132·1)	2,337·7 (1,797·1-3,030·3)	1,708·1 (1,284·3-2,232·4)
Eritrea	0·8 (0·4-1·2)	0·4 (0·2-0·6)	0·4 (0·2-0·7)	0·3 (0·1-0·5)	294·1 (244·9-354·6)	366·5 (308·3-441·4)	367·3 (308·9-442·9)	378·8 (316·2-457·3)	33·3 (8·8-67·0)	8·2 (5·8-10·6)	7·3 (5·1-9·5)	1·5 (0·9-2·0)	4,557·8 (3,458·9-6,004·1)	3,630·5 (2,751·7-4,797·6)	3,560·5 (2,691·7-4,716·6)	2,710·2 (2,020·1-3,660·2)
Ethiopia	1·8 (1·2-2·6)	0·4 (0·1-0·7)	0·4 (0·09-0·9)	0·3 (0·06-0·7)	572·5 (482·1-671·9)	316·7 (267·7-369·8)	304·6 (257·3-355·6)	168·2 (140·3-202·4)	71·1 (46·0-99·4)	9·1 (6·4-12·7)	8·8 (6·2-12·4)	6·0 (4·1-9·0)	6,764·2 (5,119·1-8,854·1)	3,639·1 (2,714·7-4,817·9)	3,520·4 (2,618·9-4,667·7)	2,064·2 (1,511·5-2,849·8)
Kenya	3·2 (1·9-4·8)	2·1 (1·8-2·5)	2·1 (1·9-2·5)	1·4 (1·2-1·7)	274·1 (248·7-301·8)	273·6 (246·6-304·1)	269·4 (242·9-299·3)	216·9 (195·3-242·2)	162·1 (120·8-199·8)	61·5 (44·8-79·1)	62·8 (45·6-81·3)	64·6 (46·6-82·9)	3,397·1 (2,643·3-4,329·5)	2,538·9 (1,979·5-3,223·1)	2,475·3 (1,928·5-3,138·9)	1,799·2 (1,418·9-2,267·8)
Madagascar	0·4 (0·1-0·9)	0·1 (0·04-0·4)	0·1 (0·04-0·4)	0·2 (0·05-0·5)	202·8 (159·6-258·0)	158·4 (123·7-202·7)	155·9 (122·2-199·2)	124·6 (101·2-154·9)	144·4 (105·5-192·9)	72·0 (51·8-94·0)	69·7 (50·2-91·1)	44·4 (30·6-59·3)	2,811·9 (2,125·9-3,726·9)	2,488·5 (1,886·3-3,285·8)	2,455·0 (1,861·5-3,236·9)	2,030·2 (1,544·6-2,677·3)
Malawi	9·3 (6·9-12·3)	4·4 (3·7-5·4)	4·2 (3·5-5·2)	3·1 (2·5-3·8)	658·8 (588·7-733·3)	410·3 (357·0-468·9)	394·8 (342·7-452·7)	220·0 (179·9-266·0)	290·3 (217·8-371·8)	212·6 (162·2-270·4)	200·6 (152·5-256·2)	114·6 (90·9-143·8)	7,680·3 (5,699·4-9,965·8)	4,443·2 (3,271·9-5,854·7)	4,272·8 (3,143·9-5,622·4)	2,451·9 (1,836·6-3,200·4)
Mozambique	8·6 (7·0-10·4)	4·2 (3·0-5·6)	3·6 (2·5-5·0)	2·6 (1·8-3·6)	524·3 (433·5-633·1)	484·9 (400·8-590·0)	476·0 (391·7-579·6)	437·2 (348·7-545·5)	338·2 (275·7-411·9)	235·8 (197·3-297·1)	231·2 (192·7-293·5)	172·6 (127·4-230·4)	5,822·2 (4,376·8-7,606·0)	3,686·8 (2,794·9-4,919·5)	3,595·7 (2,729·8-4,797·8)	2,499·0 (1,882·6-3,316·5)
Rwanda	2·3 (0·9-3·5)	0·2 (0·03-0·4)	0·3 (0·05-0·5)	0·2 (0·02-0·4)	763·5 (635·2-909·7)	299·6 (248·9-359·3)	294·7 (245·2-354·2)	189·7 (151·2-235·8)	149·9 (19·3-302·3)	189·9 (114·3-246·2)	212·3 (132·6-272·0)	207·4 (128·2-267·8)	7,632·6 (5,789·1-9,861·4)	3,355·1 (2,592·3-4,290·6)	3,271·8 (2,532·1-4,197·8)	2,216·0 (1,689·9-2,881·1)
Somalia	0·7 (0·3-1·6)	0·3 (0·1-0·8)	0·3 (0·1-0·8)	0·2 (0·07-0·6)	378·8 (320·3-444·0)	412·9 (349·0-482·5)	412·5 (348·5-482·5)	405·5 (341·4-479·6)	72·9 (44·9-104·2)	45·0 (26·1-73·8)	50·6 (28·4-84·8)	120·5 (84·2-163·1)	6,112·9 (4,592·3-7,954·2)	4,805·6 (3,624·5-6,204·1)	4,719·6 (3,556·5-6,071·4)	3,641·9 (2,759·1-4,716·8)
South Sudan	2·3 (1·0-4·7)	1·3 (0·5-2·5)	1·1 (0·4-2·2)	0·8 (0·3-1·6)	210·6 (175·6-251·9)	241·5 (202·1-289·0)	242·8 (203·2-290·2)	261·0 (219·3-312·3)	213·2 (143·7-294·1)	131·3 (77·4-204·6)	128·8 (75·5-203·0)	97·6 (51·7-177·1)	3,743·0 (2,793·3-4,862·4)	3,348·1 (2,483·4-4,400·7)	3,315·1 (2,464·0-4,359·1)	2,976·9 (2,218·6-3,858·9)
Tanzania	4·0 (2·7-5·4)	1·7 (1·2-2·4)	1·6 (1·1-2·3)	1·2 (0·8-1·7)	328·5 (289·7-374·2)	273·1 (238·5-311·0)	269·6 (235·8-307·4)	213·2 (181·0-248·4)	238·0 (182·3-294·7)	107·9 (79·4-140·9)	104·8 (77·1-137·2)	68·7 (45·5-89·0)	5,446·8 (4,285·4-6,897·7)	3,819·3 (2,998·7-4,813·9)	3,716·0 (2,913·4-4,682·6)	2,566·3 (1,988·5-3,264·1)
Uganda	2·4 (1·4-3·3)	2·5 (1·4-3·8)	2·3 (1·1-4·0)	1·6 (0·7-2·8)	794·3 (690·3-907·3)	557·7 (479·4-643·1)	545·0 (467·5-628·9)	395·3 (330·3-463·3)	317·9 (237·9-398·8)	163·6 (120·9-204·9)	147·5 (107·4-186·3)	121·1 (90·5-151·4)	4,293·2 (3,316·6-5,509·0)	3,247·9 (2,493·9-4,196·7)	3,177·2 (2,437·7-4,112·8)	2,470·6 (1,908·0-3,179·0)
Zambia	8·7 (6·1-11·7)	4·6 (3·7-5·6)	4·2 (3·3-5·3)	2·7 (2·1-3·4)	1,280·7 (1,122·2-1,454·4)	906·3 (779·9-1,041·3)	869·9 (747·0-1,001·2)	484·9 (396·6-581·9)	246·2 (181·8-316·5)	142·5 (107·0-185·7)	146·8 (110·1-193·0)	156·0 (117·7-204·1)	7,847·4 (6,020·3-10,123·7)	5,202·1 (3,972·6-6,777·5)	5,021·3 (3,834·4-6,541·8)	3,073·2 (2,289·4-4,056·1)
**Central Sub-Saharan Africa**																
Angola	1·3 (0·7-2·1)	0·9 (0·3-1·9)	0·8 (0·3-1·8)	0·6 (0·2-1·4)	639·5 (564·2-723·7)	446·8 (391·7-509·8)	436·0 (381·9-497·6)	309·7 (269·8-355·7)	181·0 (139·9-235·4)	104·4 (76·9-142·9)	104·6 (75·7-145·3)	106·6 (59·0-160·1)	4,363·9 (3,203·9-5,821·8)	2,543·8 (1,889·3-3,356·7)	2,434·2 (1,807·6-3,207·5)	1,372·7 (1,049·8-1,745·0)
Central African Republic	4·8 (3·0-6·7)	1·7 (1·1-2·8)	1·7 (1·1-2·9)	1·3 (0·8-2·3)	1,443·9 (1,263·4-1,640·3)	1,237·5 (1,090·2-1,404·3)	1,223·7 (1,078·1-1,387·8)	1,028·1 (902·0-1,174·5)	311·5 (203·7-395·5)	239·3 (149·7-312·0)	232·5 (144·7-304·9)	155·7 (86·6-240·5)	6,173·0 (4,514·8-8,324·6)	5,664·0 (4,135·7-7,696·8)	5,564·7 (4,058·5-7,595·0)	5,374·2 (3,952·7-7,239·6)
Congo	2·3 (1·6-3·2)	1·3 (1·1-1·6)	1·2 (1·0-1·6)	0·8 (0·7-1·1)	1,014·0 (886·7-1,149·7)	548·2 (480·5-621·4)	535·3 (469·1-606·8)	374·2 (326·3-428·3)	253·4 (178·7-335·9)	175·6 (112·0-251·7)	177·9 (113·1-253·6)	195·0 (126·3-280·0)	3,853·0 (2,816·5-5,109·9)	2,476·4 (1,831·2-3,255·8)	2,423·0 (1,792·8-3,196·0)	1,732·3 (1,282·4-2,314·7)
Democratic Republic of the Congo	1·2 (0·8-1·7)	0·4 (0·2-0·6)	0·4 (0·2-0·7)	0·3 (0·1-0·5)	754·8 (664·7-858·2)	560·3 (495·7-633·0)	548·3 (485·0-619·0)	413·2 (362·2-470·0)	327·2 (263·7-409·4)	225·1 (180·0-280·0)	212·9 (169·2-265·0)	145·7 (113·4-185·5)	4,716·3 (3,380·4-6,412·1)	3,533·0 (2,545·9-4,775·0)	3,449·1 (2,490·6-4,652·8)	2,463·8 (1,800·7-3,377·4)
Equatorial Guinea	5·2 (3·7-7·1)	2·9 (1·4-4·4)	2·7 (1·3-4·4)	1·5 (0·6-2·4)	839·2 (740·1-939·4)	491·0 (428·1-559·7)	477·8 (416·6-544·7)	321·1 (277·6-370·9)	334·9 (255·6-426·9)	248·3 (191·0-315·0)	258·7 (206·7-326·4)	281·0 (225·5-349·1)	3,005·4 (2,248·1-3,855·8)	1,245·9 (944·8-1,601·2)	1,190·1 (907·0-1,530·7)	598·4 (450·0-802·4)
Gabon	3·5 (2·5-4·8)	1·6 (1·1-2·3)	1·5 (1·0-2·2)	0·9 (0·6-1·3)	748·4 (658·6-849·4)	439·0 (386·3-497·5)	424·0 (373·2-480·3)	246·9 (213·8-283·8)	255·2 (165·6-370·3)	192·7 (109·0-289·4)	199·7 (117·4-299·9)	307·4 (127·2-505·9)	3,129·0 (2,370·0-4,101·4)	2,091·1 (1,607·1-2,707·9)	2,032·9 (1,561·8-2,631·5)	1,355·7 (1,041·1-1,740·0)

Location	**Indicator 3.3.5:** **Age-standardised prevalence* of the sum of 15 neglected tropical diseases (NTDs) (%)** **Prevalence estimates reported here may exceed 100% as they reflect the sum of prevalent cases of 15 NTDs.*				**Indicator 3.4.1:** **Age-standardised death rate due to cardiovascular disease, cancer, diabetes, and chronic respiratory disease in populations aged 30-70 (per 100,000 population)**				**Indicator 3.4.2:** **Age-standardised death rate due to self-harm (per 100,000 population)**				**Indicator 3.5.2:** **Risk-weighted prevalence of alcohol consumption, as measured by the summary exposure value (SEV) for alcohol use (%)**			
	**2000**	**2015**	**2016**	**2030**	**2000**	**2015**	**2016**	**2030**	**2000**	**2015**	**2016**	**2030**	**2000**	**2015**	**2016**	**2030**
Sierra Leone	136·7 (121·7-153·4)	50·3 (44·5-56·7)	46·8 (41·4-52·7)	16·3 (14·0-18·8)	513·1 (449·7-569·8)	539·1 (470·2-616·8)	541·3 (473·2-618·0)	546·8 (416·6-698·1)	8·7 (6·5-11·8)	9·7 (7·2-13·6)	9·8 (7·2-13·7)	10·3 (6·6-16·4)	15·7 (12·2-19·3)	13·2 (7·8-17·8)	12·9 (7·5-17·5)	9·4 (4·6-13·7)
Togo	88·8 (85·0-92·4)	24·9 (23·2-26·7)	21·3 (19·8-23·1)	2·5 (2·0-3·0)	537·0 (442·1-620·3)	538·4 (471·2-612·3)	535·9 (471·6-607·6)	521·1 (403·8-672·1)	14·8 (11·4-18·3)	15·4 (12·2-18·7)	15·5 (12·3-18·9)	15·1 (10·2-21·3)	5·6 (4·0-7·2)	5·6 (3·2-8·0)	5·6 (3·2-8·1)	5·9 (3·2-8·8)
**Eastern Sub-Saharan Africa**																
Burundi	139·6 (127·0-153·0)	58·7 (53·7-63·9)	54·8 (50·4-59·7)	20·3 (18·5-22·5)	750·1 (651·8-867·4)	539·4 (444·1-640·3)	536·2 (440·6-637·0)	491·8 (345·1-664·1)	20·4 (14·8-25·4)	14·6 (11·5-18·2)	14·6 (11·4-18·3)	12·1 (8·3-17·8)	20·8 (17·7-24·6)	19·6 (15·6-23·5)	19·4 (15·4-23·2)	16·8 (12·9-20·5)
Comoros	84·2 (71·6-99·5)	79·6 (67·0-95·1)	79·5 (66·9-95·0)	78·5 (65·9-94·0)	552·3 (483·1-616·6)	442·8 (379·5-504·4)	440·5 (377·8-503·7)	408·8 (300·6-530·3)	7·6 (4·9-13·0)	8·7 (5·5-17·0)	8·7 (5·4-17·1)	9·0 (4·7-19·5)	0·5 (0·3-0·8)	0·7 (0·3-1·2)	0·7 (0·3-1·2)	0·9 (0·3-1·9)
Djibouti	31·7 (26·7-41·3)	31·3 (26·3-40·6)	31·3 (26·3-40·6)	30·8 (25·9-40·0)	458·6 (367·7-547·2)	431·4 (329·2-528·0)	427·2 (326·5-520·1)	385·4 (253·0-536·2)	6·6 (3·9-11·3)	6·8 (4·1-11·8)	6·7 (4·0-11·8)	6·9 (3·3-14·4)	1·7 (1·0-2·6)	2·1 (1·0-3·8)	2·1 (0·9-3·9)	2·4 (0·8-5·4)
Eritrea	25·5 (19·0-32·9)	23·8 (17·9-30·8)	23·6 (17·7-30·5)	21·1 (16·4-27·0)	591·3 (504·9-667·0)	547·6 (472·8-630·3)	544·4 (470·3-625·7)	509·1 (399·4-655·4)	13·8 (11·0-16·8)	12·8 (10·7-15·5)	12·9 (10·6-15·5)	13·1 (9·2-18·7)	5·0 (2·9-7·7)	3·6 (1·7-6·6)	3·6 (1·7-6·8)	4·5 (1·5-10·3)
Ethiopia	80·0 (72·0-88·7)	72·6 (65·2-80·9)	71·6 (64·1-79·8)	58·8 (52·0-66·4)	669·6 (598·0-749·6)	511·2 (430·2-596·2)	505·7 (422·9-589·2)	434·6 (315·0-584·8)	16·0 (13·6-18·4)	12·8 (10·3-15·5)	12·7 (10·1-15·5)	11·1 (7·1-16·9)	4·9 (3·6-6·4)	5·3 (3·1-8·2)	5·4 (3·0-8·4)	5·9 (2·3-12·3)
Kenya	37·8 (33·9-42·2)	34·5 (31·2-38·1)	34·2 (31·0-37·8)	30·3 (27·7-33·2)	333·4 (273·3-388·2)	293·6 (247·0-338·9)	291·8 (246·1-336·4)	267·6 (216·4-324·7)	10·2 (7·5-13·2)	9·0 (7·0-11·1)	9·0 (7·1-11·1)	8·4 (6·3-11·2)	6·0 (4·6-7·3)	6·5 (5·1-7·9)	6·6 (5·2-7·9)	7·2 (5·7-8·6)
Madagascar	185·8 (165·9-207·3)	76·5 (68·9-85·2)	71·3 (64·2-79·3)	26·4 (23·6-29·3)	741·2 (666·5-819·6)	668·0 (528·0-806·4)	663·3 (523·4-804·1)	622·5 (395·7-912·9)	11·3 (9·6-13·1)	10·7 (8·4-13·2)	10·6 (8·3-13·1)	10·5 (6·5-16·2)	4·6 (3·0-6·5)	4·6 (2·4-7·9)	4·7 (2·4-8·3)	7·6 (3·0-16·1)
Malawi	77·5 (70·1-85·9)	25·9 (23·4-28·6)	22·8 (20·5-25·2)	3·6 (3·1-4·2)	613·0 (374·8-818·4)	522·1 (403·5-642·0)	517·9 (399·1-638·1)	454·0 (265·4-712·6)	13·9 (9·3-18·0)	12·5 (9·8-15·5)	12·4 (9·8-15·6)	12·0 (7·1-19·3)	4·7 (2·9-6·8)	5·7 (2·8-10·0)	5·7 (2·7-10·2)	5·9 (1·9-12·5)
Mozambique	75·8 (72·2-79·9)	69·0 (65·7-72·8)	68·2 (64·9-71·9)	57·8 (54·8-61·2)	530·3 (393·0-662·3)	463·4 (378·7-568·5)	464·2 (381·9-565·7)	446·7 (286·5-667·7)	12·9 (9·6-16·0)	12·5 (9·8-15·4)	12·5 (9·9-15·5)	13·1 (8·2-20·5)	4·3 (2·6-6·4)	5·4 (2·8-9·5)	5·5 (2·8-9·8)	6·3 (2·4-14·5)
Rwanda	82·6 (71·5-96·1)	74·7 (64·2-87·0)	74·2 (63·7-86·3)	65·4 (55·3-77·4)	596·9 (509·8-691·5)	383·0 (326·0-445·2)	384·2 (327·6-448·1)	334·3 (223·9-490·5)	18·6 (14·5-23·0)	11·6 (9·1-14·6)	11·5 (9·0-14·5)	8·7 (5·6-13·2)	17·2 (10·8-24·4)	15·7 (7·5-26·3)	15·6 (7·3-26·3)	13·9 (4·8-27·1)
Somalia	74·3 (66·8-82·4)	73·1 (65·7-81·0)	73·0 (65·6-80·9)	71·4 (64·0-79·2)	748·4 (597·5-893·2)	676·3 (564·1-813·8)	673·8 (562·8-809·9)	621·6 (478·5-806·1)	9·8 (6·5-14·3)	9·4 (6·2-14·4)	9·5 (6·3-14·5)	9·5 (5·5-16·3)	0·7 (0·3-1·2)	0·9 (0·3-1·8)	0·9 (0·3-1·8)	1·1 (0·3-2·3)
South Sudan	63·4 (53·4-74·8)	48·2 (41·1-56·9)	47·4 (40·4-56·0)	36·4 (30·9-44·0)	477·7 (346·9-617·6)	450·8 (335·0-568·1)	454·6 (337·2-572·1)	485·5 (309·9-679·1)	6·7 (3·8-11·2)	6·7 (3·9-11·3)	6·8 (4·0-11·3)	7·6 (4·2-13·5)	1·1 (0·4-2·4)	1·3 (0·5-2·8)	1·3 (0·5-2·8)	1·4 (0·6-3·1)
Tanzania	98·6 (91·9-106·5)	67·5 (61·2-75·1)	64·3 (58·1-71·8)	33·1 (28·1-38·8)	527·4 (407·3-630·2)	470·0 (386·8-546·2)	466·7 (386·4-542·8)	437·3 (314·2-590·6)	12·0 (9·5-14·3)	11·4 (9·4-14·0)	11·4 (9·3-14·0)	11·6 (7·7-16·9)	14·5 (10·1-18·4)	13·1 (6·9-18·7)	12·9 (6·7-18·5)	10·5 (4·3-16·3)
Uganda	115·5 (106·6-126·8)	50·6 (47·1-55·0)	46·6 (43·4-50·7)	14·9 (13·7-16·4)	600·9 (517·7-681·9)	497·7 (419·0-583·2)	493·1 (415·5-579·3)	433·0 (308·1-572·1)	28·9 (22·6-34·8)	21·1 (16·5-25·5)	20·6 (16·0-25·1)	15·0 (10·2-21·9)	16·9 (11·3-22·6)	13·2 (6·3-21·7)	12·8 (6·0-21·5)	8·7 (2·8-17·9)
Zambia	69·5 (61·3-78·8)	63·3 (55·4-72·6)	62·6 (54·6-71·7)	52·7 (45·0-61·5)	763·7 (601·1-908·5)	598·9 (458·0-766·7)	590·6 (447·2-760·3)	435·1 (227·3-721·1)	17·4 (13·5-21·4)	17·7 (13·5-22·3)	17·7 (13·4-22·8)	16·5 (8·7-27·1)	8·6 (5·6-12·4)	8·3 (4·0-13·6)	8·3 (3·9-13·7)	8·2 (2·4-16·1)
**Central Sub-Saharan Africa**																
Angola	70·3 (62·7-78·7)	66·5 (59·3-74·5)	66·0 (58·9-74·0)	59·8 (53·2-67·5)	584·3 (430·3-762·4)	477·3 (352·8-646·4)	471·5 (351·3-640·1)	392·5 (232·0-613·4)	11·7 (8·5-15·3)	10·8 (8·2-14·6)	10·6 (8·0-14·5)	9·1 (5·5-15·0)	3·8 (2·4-5·4)	9·3 (4·8-14·6)	9·5 (4·8-15·0)	12·8 (5·3-21·6)
Central African Republic	91·5 (85·6-98·7)	78·1 (73·4-83·7)	77·2 (72·5-82·8)	65·2 (61·0-69·9)	777·0 (603·9-965·7)	727·6 (557·0-894·5)	727·4 (554·1-895·6)	694·2 (440·8-1,006·0)	14·9 (9·1-20·6)	14·8 (8·9-20·8)	14·8 (9·0-21·0)	14·6 (7·7-23·9)	10·2 (6·5-14·3)	9·6 (4·8-15·2)	9·6 (4·7-15·2)	9·4 (3·7-16·0)
Congo	131·5 (120·0-145·2)	116·0 (105·0-129·2)	114·0 (103·0-127·1)	89·6 (79·4-102·0)	730·3 (618·8-852·2)	516·2 (412·9-636·2)	513·1 (406·0-636·1)	437·1 (265·6-649·1)	13·7 (9·8-18·8)	10·7 (7·5-15·2)	10·8 (7·4-15·5)	10·9 (5·8-19·0)	8·8 (6·1-11·9)	10·7 (6·1-15·8)	10·9 (6·1-16·3)	14·2 (6·3-22·9)
Democratic Republic of the Congo	129·5 (117·6-143·1)	115·8 (104·0-129·8)	114·6 (102·7-128·5)	98·5 (87·3-112·3)	480·8 (422·2-541·5)	459·9 (413·6-516·0)	456·0 (409·9-508·1)	410·4 (338·8-484·8)	10·6 (7·6-13·3)	10·8 (7·8-14·0)	10·8 (7·8-14·0)	11·1 (7·3-15·7)	4·8 (3·1-6·8)	6·4 (3·0-11·3)	6·6 (3·0-11·8)	9·9 (3·2-20·6)
Equatorial Guinea	111·8 (101·7-124·1)	106·1 (96·4-117·9)	105·8 (96·1-117·6)	100·8 (91·4-111·9)	551·7 (364·9-747·8)	327·0 (183·2-519·6)	327·7 (185·0-516·9)	225·2 (89·8-453·6)	13·2 (8·5-18·6)	9·4 (5·4-14·9)	9·5 (5·4-15·0)	7·3 (2·8-14·7)	9·8 (7·1-12·7)	15·2 (10·1-20·0)	15·1 (10·1-20·0)	14·5 (8·9-19·9)
Gabon	112·3 (102·3-121·4)	111·5 (101·7-120·4)	111·5 (101·7-120·3)	111·0 (101·4-119·7)	608·2 (522·6-708·9)	436·1 (353·1-528·9)	427·9 (343·5-523·1)	327·9 (203·8-491·8)	12·8 (9·7-16·5)	10·9 (7·7-15·2)	10·8 (7·6-15·2)	9·7 (4·9-17·4)	12·4 (9·7-15·2)	12·8 (7·9-17·5)	12·8 (7·9-17·6)	13·4 (7·0-19·6)

Location	Indicator 3.6.1: Age-standardised death rate due to road injuries (per 100,000 population)				Indicator 3.7.1: Proportion of women of reproductive age (15-49 years) who have their need for family planning satisfied with modern contraception methods (%)				Indicator 3.7.2: Number of livebirths per 1,000 women aged 10-14 years and women aged 15-19 years				Indicator 3.8.1: Coverage of essential health services, as defined by the UHC index comprised of the coverage of 9 tracer interventions and risk-standardised death rates from 32 causes amenable to personal healthcare (scale of 0 to 100)			
	**2000**	**2015**	**2016**	**2030**	**2000**	**2015**	**2016**	**2030**	**2000**	**2015**	**2016**	**2030**	**2000**	**2015**	**2016**	**2030**
Sierra Leone	22·4 (18·2-26·1)	20·5 (17·3-23·9)	20·4 (17·1-23·7)	18·3 (13·5-24·2)	14·5 (11·2-18·3)	43·0 (38·1-48·2)	42·8 (37·4-48·6)	67·8 (56·0-77·1)	64·4 (63·9-65·0)	57·6 (57·1-58·1)	57·1 (56·7-57·6)	49·5 (48·3-50·7)	34·3 (31·9-36·9)	43·3 (40·6-46·3)	44·0 (41·3-47·0)	53·3 (50·3-56·4)
Togo	22·9 (19·3-26·5)	21·3 (18·2-25·0)	21·2 (18·1-24·9)	19·6 (14·6-25·7)	30·0 (26·1-34·2)	38·1 (34·0-42·6)	38·9 (34·0-44·2)	53·0 (39·0-68·5)	41·5 (35·1-47·9)	40·2 (33·0-47·6)	40·7 (32·9-48·7)	39·2 (24·5-58·5)	36·6 (34·2-39·4)	44·7 (42·5-47·0)	45·3 (43·0-47·6)	53·4 (50·6-56·3)
**Eastern Sub-Saharan Africa**																
Burundi	36·6 (30·7-44·5)	31·1 (25·3-37·7)	30·8 (25·0-37·5)	28·5 (19·2-40·5)	20·5 (16·1-24·9)	33·9 (27·8-40·3)	33·9 (27·4-40·7)	42·6 (27·5-59·6)	17·1 (16·6-17·6)	12·8 (12·3-13·2)	12·3 (11·9-12·8)	8·4 (7·6-9·3)	30·3 (27·7-33·0)	43·0 (40·4-46·0)	43·2 (40·5-46·3)	48·3 (44·0-52·5)
Comoros	19·8 (17·3-22·7)	18·9 (15·2-23·6)	18·7 (14·9-23·3)	15·6 (11·2-21·2)	31·1 (26·3-36·4)	32·5 (27·8-37·3)	33·6 (28·3-39·1)	37·8 (26·6-51·7)	39·2 (38·1-40·3)	23·3 (22·4-24·3)	22·3 (21·4-23·3)	13·7 (12·3-15·1)	37·1 (35·0-39·3)	44·7 (42·1-47·5)	44·9 (42·2-47·7)	50·4 (46·0-54·9)
Djibouti	20·3 (16·2-24·6)	17·6 (13·7-22·3)	17·3 (13·3-22·0)	14·8 (9·7-21·7)	23·0 (18·9-27·6)	44·2 (36·0-52·6)	44·9 (37·0-53·2)	60·9 (45·3-75·1)	21·6 (18·6-24·4)	17·1 (13·6-21·0)	16·7 (13·2-20·7)	12·0 (7·2-17·8)	37·4 (33·7-41·3)	45·6 (42·0-50·1)	46·2 (42·5-50·7)	53·9 (49·4-59·0)
Eritrea	24·9 (21·5-29·5)	22·2 (18·4-26·6)	22·1 (18·3-26·4)	20·4 (14·8-27·0)	14·3 (12·2-16·4)	26·7 (20·3-33·5)	27·4 (20·7-34·3)	42·0 (27·9-56·6)	41·6 (37·9-45·1)	22·2 (18·8-25·9)	21·1 (17·8-24·7)	10·1 (6·5-14·6)	33·3 (31·1-35·5)	38·9 (36·4-41·9)	39·4 (36·8-42·4)	45·3 (41·3-49·8)
Ethiopia	29·2 (25·9-32·7)	22·2 (18·7-26·3)	22·1 (18·4-26·2)	19·0 (12·9-26·7)	16·1 (13·6-18·9)	59·2 (57·0-61·6)	61·6 (59·0-64·4)	86·8 (81·3-90·8)	44·3 (42·8-45·9)	28·5 (27·2-29·9)	27·7 (26·3-29·0)	18·2 (16·2-20·4)	25·0 (23·0-27·1)	39·3 (36·5-42·2)	40·1 (37·2-42·9)	54·4 (50·1-59·2)
Kenya	16·2 (13·2-19·5)	14·7 (12·1-17·6)	14·7 (12·0-17·6)	13·1 (10·4-16·4)	62·8 (61·5-64·2)	71·4 (70·8-72·1)	71·8 (71·1-72·5)	78·5 (77·0-79·9)	48·7 (46·4-51·3)	36·8 (34·1-39·5)	36·4 (33·4-39·4)	25·7 (20·7-31·0)	47·3 (44·9-50·3)	54·5 (52·0-57·5)	55·0 (52·5-58·0)	62·2 (59·0-65·6)
Madagascar	19·6 (17·3-22·0)	17·4 (14·2-21·0)	17·3 (13·9-21·0)	16·3 (10·6-24·5)	25·4 (22·3-28·9)	46·5 (39·8-53·0)	46·3 (39·6-53·1)	74·1 (61·1-84·8)	70·8 (66·6-74·9)	57·7 (53·6-61·5)	56·9 (52·7-61·0)	44·9 (36·9-53·0)	34·4 (32·3-36·4)	38·4 (35·3-41·7)	39·0 (35·7-42·4)	43·5 (38·4-49·0)
Malawi	26·0 (18·1-33·2)	20·8 (16·5-25·0)	20·5 (16·2-24·8)	17·1 (10·7-26·0)	41·1 (38·2-44·3)	72·8 (70·4-75·2)	72·9 (69·9-75·9)	90·9 (87·7-93·5)	73·6 (71·2-76·1)	67·8 (65·4-70·1)	66·8 (64·1-69·5)	59·3 (53·1-65·1)	38·4 (34·0-44·1)	48·4 (45·0-52·2)	49·0 (45·6-52·7)	58·4 (55·0-61·5)
Mozambique	27·5 (21·2-33·7)	22·9 (18·5-28·0)	22·7 (18·5-27·5)	20·5 (13·8-30·1)	25·7 (22·5-29·4)	38·3 (33·0-44·1)	39·0 (33·2-45·1)	50·0 (36·3-64·0)	82·8 (75·3-89·9)	64·1 (57·0-71·1)	61·7 (54·4-69·0)	47·4 (35·1-60·8)	37·0 (33·3-40·8)	45·2 (42·1-48·2)	45·8 (42·8-48·8)	54·4 (51·0-57·8)
Rwanda	34·5 (28·5-45·3)	24·3 (18·7-32·7)	24·1 (18·7-32·5)	21·9 (13·8-34·4)	13·5 (10·7-16·9)	60·7 (55·2-65·9)	60·3 (54·5-66·0)	83·9 (71·9-92·2)	24·3 (22·6-25·7)	12·9 (11·7-14·0)	12·4 (11·2-13·6)	6·2 (4·4-8·7)	31·9 (29·6-34·4)	51·2 (48·6-53·8)	51·7 (49·0-54·4)	61·1 (58·0-64·2)
Somalia	27·4 (21·2-35·7)	25·8 (19·4-34·1)	25·8 (19·4-34·3)	23·7 (16·0-35·5)	6·8 (5·0-9·2)	11·8 (8·9-15·6)	12·2 (9·1-16·2)	17·8 (9·3-29·8)	36·9 (29·4-44·2)	30·1 (22·9-37·7)	29·6 (22·5-37·2)	23·7 (13·1-37·7)	21·6 (18·7-24·9)	26·5 (23·8-29·6)	26·9 (24·2-30·0)	31·7 (28·2-35·6)
South Sudan	19·2 (14·7-24·3)	18·3 (14·1-23·4)	18·3 (14·3-23·3)	19·6 (13·2-28·1)	3·9 (2·7-5·3)	5·3 (3·9-6·9)	5·6 (4·1-7·4)	6·9 (3·7-11·4)	70·1 (63·6-75·5)	44·1 (39·0-48·9)	41·5 (36·6-46·1)	26·9 (20·1-34·2)	30·9 (26·8-35·3)	35·2 (31·2-39·4)	35·5 (31·4-39·6)	39·0 (33·6-44·3)
Tanzania	22·1 (18·3-26·0)	20·1 (17·1-23·7)	19·9 (16·9-23·7)	18·5 (13·3-25·9)	37·9 (35·2-40·6)	54·5 (52·1-57·0)	55·0 (52·2-57·9)	70·3 (63·9-76·1)	62·5 (57·9-67·1)	50·6 (43·7-57·3)	50·0 (42·9-57·3)	41·0 (29·9-54·5)	40·6 (37·9-43·7)	47·8 (45·3-50·7)	48·6 (46·0-51·3)	55·4 (52·2-58·8)
Uganda	26·6 (22·7-31·7)	25·6 (21·6-30·9)	25·4 (21·5-30·7)	24·0 (17·0-33·6)	28·8 (26·5-31·0)	46·7 (44·9-48·5)	48·2 (46·1-50·3)	68·4 (62·7-73·7)	82·2 (81·1-83·2)	49·6 (48·4-50·7)	47·3 (46·1-48·5)	30·6 (28·2-32·7)	34·1 (32·0-36·2)	43·3 (40·8-45·9)	43·7 (41·1-46·3)	52·2 (48·6-55·6)
Zambia	31·1 (24·6-37·0)	27·5 (21·9-34·4)	27·2 (21·3-33·9)	22·3 (12·9-35·2)	36·9 (34·2-39·8)	61·3 (57·2-65·3)	61·5 (56·5-66·2)	77·3 (69·9-83·6)	63·7 (61·7-65·6)	39·0 (37·3-40·7)	37·0 (35·2-38·7)	23·8 (21·0-26·5)	34·3 (31·4-38·1)	44·1 (40·2-48·0)	45·0 (41·0-49·0)	57·7 (52·5-63·4)
**Central Sub-Saharan Africa**																
Angola	32·2 (25·3-40·5)	26·1 (19·7-34·6)	25·1 (18·9-33·4)	16·8 (9·7-27·3)	12·0 (9·1-15·5)	29·6 (27·4-32·0)	30·1 (27·6-32·9)	56·0 (44·6-67·9)	89·3 (80·4-97·4)	71·6 (64·2-78·6)	70·0 (62·5-77·4)	56·4 (44·0-70·3)	30·5 (26·3-34·9)	43·8 (38·6-48·2)	44·9 (39·8-49·2)	59·7 (54·6-64·3)
Central African Republic	44·4 (34·6-56·5)	42·9 (33·2-57·3)	42·2 (32·5-56·9)	36·0 (22·2-55·4)	14·6 (11·8-17·9)	20·7 (16·5-25·5)	20·7 (16·1-25·9)	28·5 (16·5-42·3)	55·0 (54·7-55·4)	47·1 (46·7-47·4)	46·7 (46·3-47·1)	40·4 (39·8-41·2)	26·5 (22·3-31·8)	29·9 (25·9-34·7)	30·0 (25·9-34·7)	31·1 (26·4-36·4)
Congo	33·8 (28·2-39·3)	23·4 (18·1-29·1)	23·2 (17·8-29·2)	19·6 (11·4-31·2)	21·1 (16·5-25·9)	41·2 (35·5-46·7)	41·5 (35·2-47·5)	55·1 (41·3-68·9)	64·6 (57·7-71·0)	51·9 (47·1-56·8)	50·8 (45·7-56·0)	39·9 (31·2-49·6)	33·9 (30·9-37·4)	46·1 (42·5-50·0)	46·7 (43·1-50·7)	55·5 (50·8-59·9)
Democratic Republic of the Congo	24·8 (20·4-29·1)	22·0 (18·7-25·6)	21·8 (18·6-25·3)	19·7 (14·5-25·9)	12·6 (9·7-15·8)	25·6 (22·5-28·6)	26·4 (22·8-30·1)	39·0 (28·7-49·9)	52·2 (29·0-70·6)	56·5 (34·8-75·2)	57·1 (34·2-76·6)	62·6 (19·7-127·9)	32·7 (30·2-35·8)	42·7 (40·4-45·4)	43·3 (41·0-46·0)	51·9 (49·4-54·6)
Equatorial Guinea	35·7 (25·5-46·9)	19·1 (11·8-29·1)	18·9 (11·8-29·1)	12·5 (5·4-24·5)	16·7 (12·6-21·5)	33·8 (26·4-41·6)	34·3 (26·7-41·9)	51·6 (34·4-66·7)	43·7 (37·7-49·8)	38·9 (32·9-45·6)	38·5 (32·5-45·1)	32·5 (21·9-44·5)	35·1 (29·7-41·1)	51·1 (44·3-58·8)	52·2 (45·4-59·9)	65·4 (58·5-73·3)
Gabon	33·6 (28·0-39·9)	25·1 (19·3-31·9)	24·5 (18·7-31·5)	18·8 (10·2-30·8)	25·3 (23·1-27·7)	45·5 (40·9-50·3)	46·0 (40·5-51·6)	62·4 (49·3-73·6)	67·0 (55·6-78·9)	49·0 (38·0-60·8)	47·3 (36·0-59·3)	35·7 (19·3-56·6)	36·8 (33·9-39·6)	48·9 (45·5-52·5)	49·7 (46·2-53·3)	61·4 (56·9-65·9)

Location	Indicator 3.9.1: Age-standardised death rate attributable to household air pollution and ambient air pollution (per 100,000 population)				Indicator 3.9.2: Age-standardised death rate attributable to unsafe water, sanitation, and hygiene (WaSH) (per 100,000 population)				Indicator 3.9.3: Age-standardised death rate due to unintentional poisonings (per 100,000 population)				Indicator 3.a.1: Age-standardised prevalence of daily smoking in populations aged 10 and older (%)			
	**2000**	**2015**	**2016**	**2030**	**2000**	**2015**	**2016**	**2030**	**2000**	**2015**	**2016**	**2030**	**2000**	**2015**	**2016**	**2030**
Sierra Leone	285·0 (240·9-328·2)	262·2 (226·0-300·7)	260·8 (224·9-298·7)	241·6 (209·9-275·7)	218·5 (155·9-302·8)	166·4 (110·9-233·2)	160·8 (106·3-226·7)	131·7 (76·1-213·1)	3·0 (1·7-4·8)	2·3 (1·4-3·2)	2·2 (1·4-3·1)	1·7 (0·9-3·1)	15·5 (13·5-17·7)	14·3 (12·7-15·9)	14·1 (12·5-15·7)	13·8 (10·7-17·3)
Togo	275·8 (235·1-315·7)	249·8 (217·3-283·6)	250·2 (217·9-283·7)	247·0 (211·6-278·4)	142·7 (101·8-215·6)	99·3 (66·7-163·5)	96·4 (64·5-158·8)	68·8 (43·7-118·8)	2·7 (1·8-3·6)	1·9 (1·3-2·5)	1·9 (1·2-2·4)	1·4 (0·7-2·3)	5·7 (4·8-6·8)	5·2 (4·6-5·9)	5·2 (4·6-6·0)	4·8 (3·6-6·3)
**Eastern Sub-Saharan Africa**																
Burundi	329·3 (283·6-377·4)	231·7 (197·4-270·0)	229·9 (195·7-268·0)	205·6 (152·1-245·4)	236·7 (165·2-364·3)	184·9 (123·0-281·6)	180·6 (119·0-276·4)	130·3 (75·0-211·7)	5·3 (2·9-8·6)	3·1 (2·2-5·1)	3·1 (2·2-5·0)	2·7 (1·4-5·6)	6·8 (5·4-8·5)	6·3 (5·1-7·7)	6·3 (5·1-7·8)	5·9 (3·9-8·5)
Comoros	211·7 (181·9-241·2)	141·5 (112·0-168·9)	139·5 (110·0-167·1)	112·3 (83·1-143·3)	192·7 (135·7-283·0)	122·8 (74·5-201·8)	119·7 (72·1-197·2)	81·4 (44·7-139·6)	2·9 (2·1-4·3)	1·8 (1·3-2·9)	1·8 (1·3-2·8)	1·3 (0·6-2·6)	11·3 (9·8-12·8)	9·2 (7·9-10·5)	9·2 (7·7-10·7)	8·2 (5·8-11·3)
Djibouti	143·7 (103·8-185·3)	110·1 (72·4-159·7)	109·5 (71·3-160·2)	93·0 (51·4-159·6)	128·7 (81·8-215·7)	69·3 (41·4-122·6)	66·6 (39·4-119·5)	37·7 (19·1-79·1)	3·1 (2·1-4·7)	2·0 (1·4-2·9)	2·0 (1·4-2·9)	1·3 (0·6-2·6)	15·6 (14·0-17·2)	16·5 (14·2-19·0)	16·1 (13·8-18·7)	15·1 (11·0-19·8)
Eritrea	223·2 (186·6-260·3)	166·6 (104·9-228·7)	163·7 (102·0-226·6)	127·3 (67·4-197·0)	196·8 (129·9-301·2)	139·2 (90·2-220·2)	135·4 (87·2-214·6)	91·3 (56·1-151·3)	3·7 (2·7-5·2)	3·0 (1·9-4·2)	2·9 (1·9-4·1)	2·4 (1·1-4·4)	6·6 (5·5-7·9)	4·7 (3·7-5·8)	4·7 (3·7-6·0)	4·1 (2·5-6·1)
Ethiopia	286·9 (252·3-323·2)	198·8 (169·9-233·2)	195·8 (166·5-230·4)	150·1 (121·4-184·6)	218·4 (149·3-314·9)	125·8 (81·1-179·8)	121·1 (77·3-174·2)	72·7 (40·6-111·8)	3·9 (2·8-5·3)	2·1 (1·7-2·6)	2·1 (1·6-2·6)	1·3 (0·8-2·2)	4·0 (3·3-4·8)	4·1 (3·3-5·0)	4·1 (3·3-5·1)	3·9 (2·4-5·7)
Kenya	139·7 (106·2-168·8)	110·5 (85·7-131·6)	108·6 (84·7-129·0)	86·8 (71·8-102·4)	373·4 (259·4-561·3)	262·6 (175·6-395·0)	253·0 (168·8-380·6)	186·5 (111·2-303·9)	2·7 (2·0-3·8)	1·6 (1·2-2·3)	1·6 (1·1-2·3)	1·1 (0·8-1·9)	9·8 (9·4-10·3)	8·1 (7·8-8·4)	8·1 (7·7-8·4)	6·8 (6·3-7·4)
Madagascar	281·1 (247·0-317·7)	238·8 (195·4-283·3)	236·9 (193·0-282·0)	211·4 (160·2-265·3)	168·1 (126·1-232·8)	133·1 (89·8-202·3)	130·6 (87·4-199·7)	99·2 (59·7-167·2)	2·9 (2·2-4·0)	2·1 (1·5-3·0)	2·1 (1·5-2·9)	1·7 (0·8-3·1)	12·0 (10·6-13·6)	10·8 (8·9-12·9)	10·7 (8·8-12·9)	9·7 (6·7-13·4)
Malawi	262·0 (193·0-330·8)	179·5 (143·8-218·7)	176·0 (141·3-214·6)	132·4 (108·8-161·4)	221·9 (148·3-330·1)	139·0 (91·3-205·6)	134·9 (87·9-200·4)	87·4 (51·5-137·7)	4·1 (2·7-5·9)	2·4 (1·7-3·3)	2·3 (1·7-3·3)	1·6 (0·8-3·2)	11·9 (10·8-13·0)	11·1 (9·5-12·8)	11·1 (9·4-13·0)	10·9 (8·0-14·4)
Mozambique	215·1 (173·7-263·5)	160·4 (129·7-197·5)	158·4 (128·4-194·5)	131·4 (109·3-154·1)	124·7 (83·0-172·3)	73·6 (42·9-113·3)	70·9 (41·0-110·1)	42·1 (21·3-71·5)	3·7 (2·7-5·0)	2·5 (1·8-3·5)	2·4 (1·8-3·4)	1·7 (0·9-3·3)	10·0 (8·5-11·6)	10·6 (8·9-12·6)	10·5 (8·7-12·6)	11·1 (7·7-15·3)
Rwanda	284·5 (244·1-326·9)	163·8 (136·5-191·9)	161·7 (134·7-189·8)	134·0 (99·7-162·4)	151·8 (106·4-217·5)	85·0 (51·0-134·0)	82·4 (48·9-130·9)	52·1 (26·3-93·9)	4·6 (2·8-7·2)	2·1 (1·4-3·2)	2·1 (1·4-3·1)	1·4 (0·6-3·2)	6·8 (5·4-8·4)	6·1 (5·4-6·9)	6·1 (5·2-6·9)	5·6 (4·2-7·4)
Somalia	315·1 (261·8-373·5)	267·1 (222·7-318·2)	265·4 (221·2-316·0)	240·5 (198·9-290·0)	196·4 (119·9-341·6)	153·9 (91·7-274·8)	150·6 (89·8-268·6)	118·2 (67·7-222·7)	5·0 (3·2-7·4)	3·8 (2·6-5·7)	3·8 (2·5-5·6)	3·1 (1·6-5·9)	9·5 (7·7-11·4)	8·2 (6·5-10·0)	8·2 (6·5-10·1)	7·2 (4·6-10·4)
South Sudan	247·2 (193·2-308·1)	215·8 (171·4-262·1)	216·0 (171·9-261·7)	216·6 (166·1-255·2)	209·6 (138·7-329·9)	165·9 (107·0-258·4)	164·3 (105·9-255·9)	143·5 (93·5-218·9)	3·8 (2·2-7·2)	3·2 (2·0-5·4)	3·2 (1·9-5·3)	2·9 (1·4-5·9)	8·6 (6·9-10·5)	7·6 (5·9-9·4)	7·6 (5·9-9·3)	7·0 (4·4-10·1)
Tanzania	227·4 (186·8-266·1)	174·5 (144·4-204·7)	171·7 (141·9-201·7)	136·6 (110·9-163·3)	186·0 (127·0-263·3)	118·9 (76·3-175·1)	114·5 (73·2-169·4)	67·9 (40·5-107·2)	3·1 (2·4-3·9)	2·4 (1·7-3·6)	2·3 (1·7-3·5)	1·7 (0·8-3·8)	11·1 (9·3-12·9)	9·4 (8·0-10·8)	9·3 (7·9-10·9)	8·6 (6·0-11·8)
Uganda	257·6 (225·0-293·6)	195·2 (168·0-226·7)	192·8 (165·6-224·4)	161·3 (132·7-193·3)	138·1 (95·2-202·0)	91·8 (58·3-139·4)	90·3 (57·1-137·4)	71·5 (41·6-114·7)	3·6 (2·5-5·1)	2·5 (1·8-3·7)	2·5 (1·8-3·7)	1·9 (1·0-3·5)	10·0 (8·8-11·2)	8·9 (8·1-9·8)	8·9 (7·9-10·0)	8·7 (6·7-11·0)
Zambia	292·3 (237·9-340·8)	214·5 (173·1-262·2)	209·4 (168·3-256·9)	148·6 (112·2-192·3)	285·2 (192·6-401·0)	139·4 (87·1-205·1)	130·5 (80·6-193·9)	59·0 (29·9-95·7)	4·8 (3·4-6·2)	3·2 (2·3-4·2)	3·1 (2·2-4·2)	1·9 (0·9-3·6)	10·4 (9·3-11·7)	12·3 (10·9-13·8)	12·2 (10·6-13·8)	11·6 (8·6-15·3)
**Central Sub-Saharan Africa**																
Angola	257·2 (201·9-317·5)	148·5 (103·1-203·7)	142·9 (97·5-198·8)	82·6 (42·8-136·0)	303·8 (220·5-418·8)	125·0 (82·3-195·1)	118·3 (77·2-186·6)	53·1 (30·7-96·1)	3·4 (2·2-4·8)	2·1 (1·4-2·8)	2·0 (1·3-2·8)	1·1 (0·5-2·1)	6·7 (5·6-7·9)	8·7 (7·3-10·2)	8·7 (7·3-10·2)	10·7 (7·6-14·3)
Central African Republic	350·9 (284·7-416·0)	321·2 (259·6-386·3)	320·4 (259·1-385·6)	307·0 (245·1-373·2)	215·4 (136·9-320·1)	225·2 (141·0-333·7)	223·6 (139·5-332·9)	215·4 (127·0-337·7)	4·2 (2·3-6·4)	3·7 (1·8-5·9)	3·6 (1·8-5·7)	3·1 (1·2-6·0)	5·9 (4·9-7·0)	7·5 (6·2-8·9)	7·4 (6·2-8·8)	8·9 (6·2-12·2)
Congo	294·3 (248·5-342·3)	175·1 (131·3-223·4)	170·3 (126·3-219·5)	114·0 (72·3-167·4)	160·9 (93·9-244·3)	128·5 (73·5-197·2)	124·3 (70·9-191·1)	100·7 (52·0-174·8)	2·6 (1·4-3·7)	1·5 (0·9-2·2)	1·5 (0·9-2·1)	1·0 (0·5-2·0)	5·1 (4·4-5·9)	7·9 (6·9-9·0)	7·8 (6·8-8·9)	9·5 (6·7-12·9)
Democratic Republic of the Congo	247·8 (213·6-290·0)	216·4 (187·3-246·5)	214·2 (185·6-243·7)	185·2 (160·8-208·2)	150·6 (104·8-223·5)	116·2 (79·9-177·2)	112·5 (77·5-171·9)	84·4 (55·4-142·3)	2·5 (1·5-3·8)	2·1 (1·3-3·1)	2·0 (1·2-3·0)	1·7 (0·9-3·0)	8·2 (6·9-9·5)	8·8 (7·9-9·8)	8·8 (7·7-9·9)	9·3 (7·2-11·9)
Equatorial Guinea	242·8 (172·1-317·4)	119·6 (71·6-177·1)	116·8 (69·3-173·3)	78·7 (41·0-126·7)	73·7 (40·3-155·2)	33·2 (14·4-75·4)	32·1 (13·8-72·9)	18·4 (6·2-44·5)	2·6 (1·4-3·8)	1·0 (0·5-1·6)	0·9 (0·5-1·6)	0·5 (0·2-1·2)	2·4 (2·0-2·9)	2·6 (2·1-3·1)	2·6 (2·1-3·1)	2·6 (1·8-3·7)
Gabon	157·2 (126·9-193·7)	88·6 (67·4-115·0)	87·1 (65·8-113·8)	63·1 (42·5-91·2)	96·8 (60·9-144·8)	58·3 (32·3-96·4)	55·8 (30·6-93·0)	33·7 (15·7-65·2)	1·9 (1·0-2·6)	1·2 (0·6-2·0)	1·2 (0·6-2·0)	0·8 (0·3-1·8)	9·0 (7·6-10·6)	9·8 (8·7-10·9)	9·7 (8·6-10·9)	10·5 (8·0-13·0)

Location	Indicator 3.b.1:<br>Geometric mean of the coverage of eight vaccines, conditional on inclusion in national vaccine schedules, in target populations (%)				Indicator 5.2.1:<br>Age-standardised prevalence of women aged 15 years and older who experienced physical or sexual violence by an intimate partner in the last 12 months (%)				Indicator 6.1.1:<br>Risk-weighted prevalence of populations using unsafe or unimproved water sources, as measured by the summary exposure value (SEV) for unsafe water (%)				Indicator 6.2.1a:<br>Risk-weighted prevalence of populations using unsafe or unimproved sanitation, as measured by the summary exposure value (SEV) for unsafe sanitation (%)			
	**2000**	**2015**	**2016**	**2030**	**2000**	**2015**	**2016**	**2030**	**2000**	**2015**	**2016**	**2030**	**2000**	**2015**	**2016**	**2030**
Sierra Leone	53·2 (49·4-56·6)	81·8 (77·0-85·9)	82·7 (77·4-87·5)	93·6 (86·0-97·5)	48·2 (42·6-53·9)	44·9 (40·0-49·8)	44·6 (39·7-49·6)	41·4 (37·2-46·0)	46·1 (36·3-51·1)	45·3 (35·6-50·6)	45·1 (35·5-50·5)	43·0 (21·9-66·1)	91·9 (82·8-99·7)	83·1 (66·2-100·0)	82·6 (65·0-100·0)	73·3 (45·8-99·5)
Togo	62·0 (59·5-64·5)	83·4 (79·1-87·0)	84·6 (79·9-88·3)	94·7 (88·2-98·4)	38·2 (33·6-42·4)	37·4 (33·1-41·4)	37·4 (33·1-41·4)	36·6 (32·6-40·6)	44·3 (35·1-49·3)	44·9 (34·8-50·3)	44·8 (34·6-50·3)	43·4 (22·2-65·6)	84·6 (75·0-94·9)	76·4 (54·1-92·7)	75·4 (51·2-92·7)	65·0 (19·5-95·6)
**Eastern Sub-Saharan Africa**																
Burundi	72·6 (68·7-76·2)	94·4 (91·7-96·3)	93·9 (90·6-96·2)	96·4 (90·1-99·5)	45·1 (39·8-50·2)	44·7 (39·5-49·7)	44·5 (39·3-49·5)	43·8 (38·5-48·8)	45·9 (37·9-50·0)	45·2 (37·0-49·9)	45·1 (36·9-49·9)	44·1 (35·8-50·1)	94·0 (85·4-100·0)	86·0 (68·9-100·0)	85·9 (68·0-100·0)	75·4 (46·7-93·2)
Comoros	67·4 (63·2-71·3)	75·6 (69·3-81·1)	75·5 (68·6-81·7)	78·0 (60·2-89·9)	43·4 (38·9-47·7)	40·5 (36·1-44·7)	40·3 (36·0-44·5)	38·3 (34·2-42·2)	38·3 (29·5-43·9)	32·2 (22·4-39·5)	31·8 (21·9-39·3)	25·8 (15·4-36·5)	91·9 (83·2-100·0)	78·5 (60·5-94·5)	77·6 (58·6-94·3)	58·5 (28·8-84·8)
Djibouti	53·0 (49·2-57·2)	75·2 (68·5-81·7)	75·0 (67·2-82·2)	81·7 (60·2-94·4)	51·0 (45·4-56·5)	46·3 (41·2-51·2)	45·9 (40·9-50·8)	41·0 (36·3-45·4)	40·9 (31·3-46·8)	35·9 (24·7-45·0)	35·8 (24·4-45·0)	33·4 (19·8-46·8)	74·8 (46·6-98·5)	56·3 (24·8-88·2)	55·4 (23·4-88·4)	39·2 (7·7-80·2)
Eritrea	75·4 (72·5-78·2)	96·8 (95·0-98·1)	97·0 (95·2-98·3)	99·3 (98·0-99·8)	51·7 (46·2-57·4)	50·2 (44·7-55·5)	50·1 (44·6-55·3)	48·0 (42·4-53·1)	43·1 (31·6-49·2)	39·4 (26·4-47·8)	39·2 (26·0-47·7)	35·5 (21·0-46·1)	89·4 (83·3-93·8)	80·6 (41·8-96·8)	79·7 (38·5-96·8)	66·0 (8·1-96·7)
Ethiopia	48·6 (42·2-55·3)	61·6 (50·7-71·3)	61·4 (48·7-72·6)	80·2 (57·9-95·4)	58·1 (52·0-63·5)	53·7 (47·8-59·2)	53·3 (47·4-58·7)	49·5 (43·7-54·6)	44·6 (32·5-50·9)	41·2 (30·3-47·8)	41·1 (30·2-47·7)	39·6 (28·3-47·1)	96·2 (92·5-98·8)	95·2 (90·0-99·6)	95·4 (90·1-99·5)	94·1 (85·4-97·0)
Kenya	83·3 (80·0-86·1)	92·8 (90·1-95·0)	93·3 (90·3-95·5)	97·3 (92·9-99·3)	43·8 (38·5-49·3)	42·5 (37·6-47·5)	42·4 (37·5-47·3)	41·5 (36·8-45·9)	38·5 (26·1-44·0)	35·1 (22·4-40·5)	34·9 (22·2-40·3)	31·9 (18·9-37·4)	87·5 (81·3-94·2)	80·2 (72·0-91·1)	79·7 (71·4-90·7)	69·7 (60·8-83·3)
Madagascar	66·8 (63·3-70·3)	80·6 (75·4-84·7)	81·7 (75·9-86·1)	89·6 (79·1-95·3)	45·6 (40·5-50·9)	42·7 (37·7-47·8)	42·6 (37·6-47·6)	40·8 (36·0-45·7)	37·0 (22·0-43·5)	40·1 (24·5-47·0)	40·2 (24·6-47·1)	41·5 (26·8-48·3)	91·1 (85·9-95·2)	93·6 (88·5-98·7)	93·4 (88·3-98·7)	91·1 (84·7-99·4)
Malawi	89·6 (87·9-91·0)	93·5 (91·5-95·0)	94·1 (92·0-95·7)	97·8 (94·5-99·4)	44·7 (39·8-49·6)	42·7 (38·0-47·5)	42·6 (37·9-47·4)	41·3 (36·4-46·5)	43·8 (35·1-48·5)	40·6 (32·3-45·1)	40·3 (32·0-44·9)	36·8 (29·1-41·8)	96·0 (92·1-98·7)	85·7 (71·2-100·0)	85·2 (69·7-100·0)	74·0 (38·5-99·6)
Mozambique	78·5 (75·7-81·2)	82·8 (78·6-86·2)	83·7 (78·4-87·6)	90·7 (80·3-96·0)	49·0 (44·0-54·1)	47·8 (42·9-52·4)	47·6 (42·7-52·3)	45·1 (40·2-49·9)	47·3 (35·8-53·0)	44·9 (33·1-51·4)	44·7 (32·7-51·4)	41·8 (20·4-65·9)	89·3 (74·3-100·0)	75·4 (50·0-93·7)	74·3 (46·6-94·0)	54·4 (9·8-90·1)
Rwanda	78·5 (74·6-82·1)	90·0 (85·0-93·9)	90·2 (84·6-94·2)	89·3 (72·3-97·4)	52·1 (46·5-57·6)	45·5 (40·3-50·3)	45·3 (40·0-50·0)	41·8 (36·6-46·5)	38·8 (24·7-45·1)	33·2 (19·0-39·9)	32·8 (18·5-39·5)	27·0 (7·9-47·6)	93·9 (82·6-100·0)	80·4 (58·4-100·0)	80·1 (57·7-100·0)	63·4 (33·0-89·3)
Somalia	21·9 (18·0-26·4)	32·9 (27·3-39·7)	31·0 (23·6-40·1)	38·9 (22·8-57·8)	56·4 (50·6-62·1)	56·0 (50·2-61·5)	55·9 (50·2-61·5)	55·4 (49·7-61·0)	43·9 (30·4-51·3)	38·8 (26·2-45·9)	38·5 (25·9-45·8)	34·7 (21·6-44·1)	91·4 (79·1-98·1)	75·0 (53·6-89·7)	73·9 (50·4-90·3)	53·6 (11·3-92·1)
South Sudan	32·8 (23·0-44·5)	52·0 (46·4-58·4)	52·7 (46·0-60·2)	63·6 (44·3-79·7)	58·7 (53·1-63·9)	58·1 (52·5-63·2)	58·0 (52·5-63·1)	57·3 (52·0-62·3)	46·4 (36·2-51·8)	45·8 (36·2-51·4)	45·8 (36·2-51·4)	44·8 (35·2-51·4)	89·2 (79·0-96·0)	93·5 (80·5-100·0)	93·4 (79·7-100·0)	94·4 (65·4-100·0)
Tanzania	86·4 (84·1-88·2)	93·9 (91·2-95·9)	93·4 (89·8-96·0)	95·7 (87·0-99·4)	53·7 (47·9-59·3)	47·6 (42·7-52·3)	47·4 (42·6-52·1)	44·0 (39·2-48·9)	38·3 (25·5-44·1)	36·1 (23·1-42·2)	36·3 (23·3-42·5)	38·7 (24·3-46·0)	95·6 (89·8-98·8)	75·1 (60·5-88·8)	73·3 (57·7-87·7)	34·5 (15·3-52·1)
Uganda	66·6 (62·8-70·2)	81·0 (75·0-86·1)	80·3 (73·0-86·4)	89·6 (75·3-97·3)	57·9 (51·6-64·4)	50·9 (45·4-56·1)	50·5 (44·9-55·7)	44·6 (38·7-50·5)	36·4 (21·6-42·9)	33·3 (19·5-39·4)	33·1 (19·1-39·3)	30·6 (16·1-37·6)	96·0 (90·3-99·9)	89·3 (78·1-100·0)	89·2 (77·8-100·0)	81·6 (59·2-93·9)
Zambia	88·3 (86·3-90·2)	89·1 (85·8-91·8)	90·2 (86·4-93·1)	91·9 (83·8-96·4)	51·6 (45·9-57·2)	47·7 (42·6-52·9)	47·3 (42·2-52·5)	42·1 (37·3-47·0)	37·8 (25·0-44·2)	36·2 (22·9-43·6)	36·0 (22·5-43·5)	33·2 (18·3-44·2)	82·7 (73·9-90·0)	70·8 (45·1-90·6)	70·1 (42·4-90·8)	57·7 (13·5-89·2)
**Central Sub-Saharan Africa**																
Angola	42·0 (37·1-47·1)	78·6 (72·0-84·4)	79·6 (71·6-86·1)	94·8 (86·0-98·8)	56·3 (49·8-62·8)	51·0 (45·1-57·3)	50·6 (44·7-56·9)	44·6 (39·2-50·4)	48·3 (36·3-54·1)	43·9 (33·1-50·0)	43·6 (32·8-49·7)	38·3 (28·3-46·2)	71·5 (58·0-81·5)	69·5 (48·3-87·2)	68·9 (45·6-88·1)	59·1 (15·0-92·0)
Central African Republic	46·6 (43·2-49·8)	51·2 (43·7-58·2)	50·0 (41·7-58·3)	34·3 (15·3-58·0)	48·2 (42·9-53·8)	49·1 (43·8-54·4)	49·4 (44·0-54·8)	50·7 (45·3-56·0)	47·5 (38·6-52·0)	47·2 (38·4-51·7)	47·2 (38·4-51·7)	47·5 (37·9-52·3)	92·9 (85·6-100·0)	90·0 (76·0-100·0)	89·9 (75·1-100·0)	86·8 (60·3-97·9)
Congo	54·7 (50·2-59·1)	84·2 (78·0-88·9)	85·4 (78·9-90·4)	92·2 (78·8-98·5)	48·0 (42·3-54·1)	45·1 (39·7-50·5)	44·9 (39·5-50·2)	42·3 (37·3-47·1)	39·5 (30·5-46·0)	37·1 (27·5-44·1)	36·7 (27·0-43·9)	31·5 (14·1-54·0)	79·5 (66·4-90·0)	76·6 (55·0-97·3)	75·4 (51·8-97·9)	72·4 (20·1-100·0)
Democratic Republic of the Congo	36·1 (32·7-39·8)	78·7 (73·2-83·4)	80·2 (73·9-85·3)	93·7 (83·4-98·2)	60·5 (54·1-66·7)	58·5 (52·4-64·5)	58·0 (52·0-64·0)	53·9 (46·4-61·6)	46·6 (36·6-51·6)	46·7 (36·9-52·2)	46·5 (36·8-52·0)	44·4 (35·2-50·5)	91·7 (82·7-98·9)	83·9 (69·8-100·0)	83·0 (68·0-100·0)	66·2 (40·5-100·0)
Equatorial Guinea	46·3 (41·5-50·8)	44·3 (37·1-51·9)	41·7 (32·0-52·7)	38·3 (20·7-59·4)	47·2 (41·6-52·5)	38·2 (33·7-42·8)	37·9 (33·5-42·5)	33·8 (29·5-38·2)	43·8 (33·4-49·5)	33·7 (23·1-43·3)	33·2 (22·6-43·0)	26·0 (10·6-45·6)	79·0 (71·6-87·7)	41·4 (11·2-83·2)	40·3 (10·6-83·2)	17·4 (1·8-56·0)
Gabon	42·3 (39·0-45·8)	75·1 (69·5-79·9)	75·3 (69·0-80·8)	89·4 (78·6-95·9)	44·4 (39·4-50·0)	41·7 (37·4-46·4)	41·6 (37·3-46·2)	39·7 (35·5-44·1)	34·4 (25·7-42·3)	23·7 (15·5-35·3)	23·3 (15·1-35·0)	18·2 (10·3-30·7)	69·6 (60·3-80·0)	53·1 (28·0-76·2)	51·8 (25·6-75·9)	36·4 (6·2-70·1)

Location	Indicator 6.2.1b: Risk-weighted prevalence of populations without access to a handwashing facility, as measured by the summary exposure value (SEV) for unsafe hygiene (%)				Indicator 7.1.2: Risk-weighted prevalence of household air pollution, as measured by the summary exposure value (SEV) for household air pollution (%)				Indicator 8.8.1: Age-standardised all-cause disability-adjusted life year (DALY) rates attributable to occupational risks (per 100,000 population)				Indicator 11.6.2: Population-weighted mean levels of fine particulate matter smaller than 2.5 microns in diameter (PM2.5)			
	**2000**	**2015**	**2016**	**2030**	**2000**	**2015**	**2016**	**2030**	**2000**	**2015**	**2016**	**2030**	**2000**	**2015**	**2016**	**2030**
Sierra Leone	88·5 (78·2-95·2)	90·2 (83·6-94·9)	90·2 (83·5-95·0)	91·9 (84·7-96·3)	69·6 (56·8-82·0)	63·5 (51·9-75·4)	62·9 (51·3-74·8)	54·9 (43·7-64·5)	1,003·8 (845·5-1,182·7)	940·8 (784·8-1,114·8)	934·5 (780·7-1,107·7)	851·7 (717·6-1,011·5)	25·6 (22·1-33·3)	40·5 (34·0-56·4)	42·1 (35·3-55·0)	41·5 (32·9-59·3)
Togo	89·5 (78·9-95·6)	89·1 (80·0-95·0)	89·0 (79·6-95·0)	88·6 (78·4-95·2)	66·5 (54·0-78·5)	57·3 (46·1-69·4)	56·5 (45·2-68·5)	44·2 (33·9-55·6)	1,238·3 (1,043·2-1,445·8)	1,147·5 (961·7-1,346·1)	1,136·7 (953·2-1,332·9)	995·2 (834·8-1,172·7)	53·5 (46·3-66·5)	80·1 (69·5-99·2)	84·1 (72·8-102·8)	77·8 (65·3-95·8)
**Eastern Sub-Saharan Africa**																
Burundi	93·9 (86·8-97·8)	92·5 (84·1-97·4)	92·3 (84·0-97·4)	90·1 (81·5-96·7)	74·1 (60·9-86·3)	68·5 (56·0-81·1)	67·9 (55·5-80·5)	59·8 (48·7-71·2)	1,893·5 (1,583·9-2,251·6)	1,572·6 (1,296·3-1,891·8)	1,552·3 (1,279·9-1,868·7)	1,290·3 (1,065·8-1,564·4)	47·1 (41·6-54·3)	46·5 (40·6-54·8)	46·3 (40·7-54·1)	45·3 (35·5-56·7)
Comoros	90·3 (81·9-95·8)	80·6 (68·7-89·6)	79·7 (67·2-89·1)	63·0 (44·7-83·8)	61·5 (49·1-73·6)	45·0 (29·9-58·7)	44·3 (28·6-58·4)	34·8 (14·0-54·1)	1,122·5 (903·3-1,361·9)	989·0 (796·6-1,199·5)	982·7 (792·4-1,191·1)	898·1 (741·5-1,083·5)	21·1 (16·6-27·1)	19·0 (14·9-24·2)	19·1 (15·0-25·0)	19·3 (14·6-26·1)
Djibouti	86·9 (73·0-95·2)	78·5 (60·1-91·5)	78·1 (59·5-91·3)	69·3 (46·6-87·2)	22·0 (7·4-42·8)	12·5 (1·5-35·3)	12·3 (1·2-36·8)	8·6 (0·0-44·8)	842·4 (699·7-1,012·8)	787·4 (634·1-970·8)	784·1 (632·4-966·6)	738·4 (607·9-900·6)	49·8 (27·6-90·3)	71·1 (42·8-121·3)	71·7 (42·0-135·2)	81·7 (45·9-160·0)
Eritrea	90·8 (79·5-96·6)	88·2 (75·7-95·8)	87·9 (75·3-95·7)	84·8 (69·3-94·9)	50·2 (39·0-61·8)	31·8 (5·0-58·8)	30·9 (4·3-58·2)	18·7 (0·5-47·5)	1,176·6 (968·0-1,405·1)	1,060·1 (881·8-1,252·3)	1,050·6 (876·2-1,241·5)	923·9 (787·7-1,092·0)	43·3 (40·0-47·2)	50·2 (46·6-54·6)	50·5 (47·0-55·0)	49·1 (45·2-53·5)
Ethiopia	97·7 (95·3-99·1)	96·1 (93·4-97·9)	95·9 (93·1-97·8)	93·6 (88·6-96·9)	72·7 (59·5-85·3)	64·3 (52·2-77·1)	63·5 (51·4-76·4)	51·1 (40·0-64·7)	1,800·4 (1,358·7-2,136·1)	1,338·5 (1,095·6-1,604·8)	1,315·4 (1,079·6-1,576·2)	1,024·8 (856·1-1,237·9)	35·5 (34·1-37·7)	48·9 (47·0-51·6)	49·7 (47·8-52·7)	49·4 (47·1-52·7)
Kenya	91·5 (89·8-92·8)	88·1 (86·4-89·5)	87·3 (85·6-88·6)	86·8 (78·7-91·3)	54·0 (45·8-63·1)	46·5 (39·0-54·8)	45·7 (38·3-54·0)	35·3 (29·2-42·4)	1,325·3 (1,094·9-1,569·1)	1,266·7 (1,037·2-1,517·9)	1,259·4 (1,030·7-1,509·7)	1,162·0 (940·0-1,407·0)	16·2 (15·7-16·8)	16·3 (15·8-16·8)	16·3 (15·8-16·8)	16·1 (15·4-16·7)
Madagascar	87·7 (79·4-93·7)	88·5 (78·2-95·3)	88·5 (77·9-95·4)	87·8 (74·6-96·4)	67·8 (55·5-80·4)	64·0 (52·4-76·0)	63·6 (52·1-75·6)	58·4 (47·1-70·2)	2,149·8 (1,794·0-2,516·8)	1,751·1 (1,451·6-2,065·3)	1,731·0 (1,433·8-2,041·5)	1,471·5 (1,197·6-1,741·2)	19·4 (18·5-20·3)	21·4 (20·2-22·7)	21·5 (20·3-22·8)	22·1 (18·9-24·1)
Malawi	88·2 (81·5-93·1)	94·6 (90·9-97·2)	94·6 (90·9-97·3)	97·0 (91·7-98·9)	69·3 (56·2-81·9)	63·7 (51·6-76·6)	63·2 (51·2-76·0)	56·5 (45·4-68·4)	1,468·0 (1,184·2-1,758·8)	1,226·1 (988·9-1,491·1)	1,213·1 (979·6-1,477·1)	1,044·1 (848·7-1,279·6)	29·2 (26·9-32·2)	28·1 (26·0-30·9)	28·3 (26·2-31·1)	28·6 (26·2-31·7)
Mozambique	90·7 (82·3-96·3)	87·2 (78·5-93·2)	86·7 (77·5-93·0)	83·1 (74·2-91·7)	72·8 (59·4-85·2)	64·1 (51·6-78·1)	63·1 (50·6-77·2)	48·6 (36·8-62·2)	1,512·5 (1,211·6-1,841·0)	1,265·1 (1,038·2-1,521·1)	1,250·6 (1,026·6-1,504·0)	1,061·9 (871·8-1,264·9)	22·8 (21·3-25·0)	20·9 (19·7-22·5)	21·0 (19·8-22·5)	20·8 (19·5-22·4)
Rwanda	98·5 (97·4-99·2)	96·3 (93·9-97·9)	96·1 (93·5-97·8)	91·8 (86·2-95·9)	71·6 (57·9-83·8)	62·7 (50·9-75·0)	62·0 (50·3-74·2)	51·7 (41·5-62·7)	1,831·7 (1,525·5-2,164·5)	1,305·8 (1,066·0-1,557·9)	1,288·5 (1,052·9-1,536·3)	1,064·6 (864·1-1,274·7)	53·8 (47·2-62·0)	52·5 (46·2-60·0)	52·8 (46·8-61·8)	53·7 (41·2-67·6)
Somalia	91·8 (82·4-97·1)	87·8 (75·0-95·6)	87·5 (74·6-95·6)	83·0 (68·1-94·0)	74·2 (61·7-85·8)	67·6 (55·1-80·7)	67·0 (54·5-80·2)	57·5 (46·3-71·8)	1,707·9 (1,289·3-2,100·9)	1,549·9 (1,275·9-1,838·3)	1,537·9 (1,272·5-1,822·2)	1,377·1 (1,187·0-1,611·1)	22·3 (20·9-25·0)	23·7 (21·9-27·4)	23·9 (22·1-27·4)	24·5 (22·2-28·3)
South Sudan	93·5 (85·3-97·9)	93·7 (86·2-97·8)	93·6 (86·0-97·8)	93·9 (85·6-98·1)	81·3 (66·8-91·3)	75·5 (61·5-87·6)	74·9 (61·0-87·3)	66·0 (52·6-81·5)	1,282·0 (909·2-1,674·4)	1,560·8 (1,168·6-1,947·3)	1,575·0 (1,172·3-1,948·4)	1,754·1 (1,180·7-2,199·7)	42·7 (41·7-43·8)	48·1 (47·0-49·1)	48·7 (47·6-49·9)	50·9 (48·8-52·6)
Tanzania	91·2 (80·9-96·9)	90·3 (78·8-96·8)	90·2 (78·5-96·8)	89·4 (74·4-96·7)	64·6 (52·7-76·5)	57·9 (45·5-70·4)	57·2 (44·7-69·6)	46·2 (33·1-57·5)	1,606·1 (1,319·2-1,917·5)	1,394·4 (1,149·9-1,675·2)	1,386·5 (1,144·2-1,665·7)	1,278·4 (1,057·9-1,529·7)	22·4 (21·7-23·1)	22·4 (21·5-23·6)	22·3 (21·5-23·5)	22·5 (21·1-24·1)
Uganda	94·0 (89·8-96·7)	89·1 (83·9-93·1)	88·9 (83·4-93·0)	83·5 (74·7-90·6)	70·7 (58·3-83·2)	63·2 (51·5-75·8)	62·6 (50·9-75·3)	53·8 (42·6-66·5)	1,469·1 (1,208·0-1,779·1)	1,278·4 (1,050·8-1,529·7)	1,266·9 (1,041·9-1,517·4)	1,115·3 (924·4-1,339·8)	60·4 (58·6-62·5)	72·8 (70·5-75·3)	73·5 (71·2-76·2)	70·6 (65·0-73·9)
Zambia	86·5 (72·5-94·5)	86·2 (77·9-92·5)	86·0 (77·8-92·4)	86·8 (77·6-93·1)	53·7 (43·8-64·7)	52·0 (41·1-63·3)	52·1 (41·2-63·6)	53·4 (41·0-67·1)	1,306·4 (1,083·1-1,557·7)	1,138·3 (941·0-1,340·8)	1,120·4 (925·3-1,319·4)	898·2 (732·9-1,071·9)	30·9 (28·4-34·1)	30·4 (27·8-34·1)	30·6 (28·0-34·3)	26·0 (23·5-29·5)
**Central Sub-Saharan Africa**																
Angola	90·0 (78·0-96·2)	87·6 (73·0-95·8)	87·3 (72·5-95·7)	85·0 (68·1-95·3)	59·2 (47·5-71·0)	28·2 (11·6-46·5)	26·8 (9·7-46·5)	11·1 (0·5-37·8)	1,599·4 (1,188·5-1,909·1)	1,180·1 (999·6-1,375·0)	1,153·6 (982·4-1,343·0)	839·4 (735·7-967·2)	39·7 (37·7-42·2)	35·8 (29·7-50·1)	36·2 (29·7-51·5)	35·0 (24·6-53·5)
Central African Republic	87·5 (76·9-94·6)	87·5 (77·2-94·1)	87·6 (76·9-94·2)	87·3 (74·6-93·8)	68·9 (55·5-81·3)	65·3 (52·1-78·3)	65·1 (51·6-78·1)	61·5 (45·1-75·4)	1,720·4 (1,470·4-1,989·2)	1,512·8 (1,297·2-1,742·8)	1,509·8 (1,296·2-1,738·9)	1,468·5 (1,277·6-1,688·7)	51·9 (44·2-69·3)	65·6 (55·7-84·6)	66·3 (57·1-87·8)	66·9 (56·1-89·5)
Congo	83·3 (67·6-93·5)	82·6 (65·6-93·2)	82·3 (64·9-93·1)	81·4 (57·7-94·2)	54·5 (43·8-66·2)	35·0 (18·4-50·2)	33·6 (16·4-49·7)	17·9 (2·3-39·8)	1,367·8 (1,173·4-1,589·2)	1,130·5 (951·6-1,319·0)	1,119·0 (941·2-1,306·2)	968·7 (800·7-1,139·3)	61·3 (41·6-98·4)	56·9 (37·8-94·8)	55·8 (37·5-95·3)	56·5 (33·4-98·0)
Democratic Republic of the Congo	94·3 (88·6-97·6)	95·3 (91·9-97·5)	95·2 (91·7-97·5)	96·4 (93·5-98·2)	70·4 (57·6-83·8)	65·6 (53·7-78·7)	65·0 (53·1-78·2)	56·1 (45·8-70·0)	1,440·7 (1,028·6-1,731·3)	1,374·1 (1,065·6-1,651·0)	1,371·4 (1,074·3-1,633·7)	1,320·4 (978·5-1,854·0)	51·4 (45·4-62·3)	55·9 (51·4-63·6)	56·3 (52·0-63·3)	57·8 (45·7-68·6)
Equatorial Guinea	89·7 (78·0-96·4)	81·5 (64·3-92·8)	80·9 (63·5-92·6)	69·7 (49·9-88·1)	53·6 (35·2-67·3)	32·9 (8·4-50·2)	31·9 (7·6-49·4)	18·9 (1·5-36·8)	1,186·6 (1,010·9-1,366·9)	836·7 (693·8-988·3)	829·3 (687·7-978·0)	731·1 (607·9-859·2)	53·6 (38·1-90·7)	69·1 (47·3-114·7)	71·2 (48·0-125·9)	79·8 (52·8-145·0)
Gabon	75·8 (55·3-90·2)	65·2 (41·6-84·0)	64·6 (40·8-83·6)	53·5 (28·0-75·4)	18·8 (14·3-23·8)	6·8 (2·1-16·3)	6·5 (1·8-16·6)	2·7 (0·1-15·2)	1,020·7 (876·5-1,185·9)	880·9 (741·3-1,034·0)	875·5 (735·9-1,028·4)	803·5 (672·9-953·5)	40·7 (30·0-64·2)	46·8 (36·4-69·3)	47·4 (37·1-68·6)	49·1 (37·3-70·6)

Location	Indicator 16.1.1: Age-standardised death rate due to interpersonal violence (per 100,000 population)				Indicator 16.1.2: Death rate due to conflict and terrorism (per 100,000 population)				Indicator 16.1.3: Age-standardised prevalence of physical or sexual violence experienced by populations in the last 12 months (%)				Indicator 16.2.3: Age-standardised prevalence of women and men aged 18-29 years who experienced sexual violence by age 18 (%)			
	**2000**	**2015**	**2016**	**2030**	**2000**	**2015**	**2016**	**2030**	**2000**	**2015**	**2016**	**2030**	**2000**	**2015**	**2016**	**2030**
Sierra Leone	1·6 (1·1-2·1)	1·6 (1·1-2·1)	1·7 (1·1-2·1)	1·6 (0·9-2·6)	21·5 (10·4-34·1)	0·0 (0·0-0·0)	0·0 (0·0-0·0)	0·0 (0·0-0·0)	20·1 (18·0-22·4)	17·5 (16·2-19·0)	17·4 (16·0-18·8)	15·1 (14·0-16·5)	15·4 (11·9-19·4)	15·4 (11·9-19·4)	15·4 (11·9-19·4)	15·4 (12·0-19·4)
Togo	3·2 (2·3-4·8)	3·9 (2·8-5·2)	3·9 (2·8-5·1)	4·2 (2·4-6·5)	0·0 (0·0-0·0)	0·0 (0·0-0·0)	0·0 (0·0-0·0)	0·0 (0·0-0·0)	11·0 (9·9-12·2)	9·8 (8·9-10·8)	9·8 (8·8-10·7)	8·9 (8·0-9·8)	15·4 (12·0-19·4)	15·4 (11·9-19·4)	15·4 (11·9-19·4)	15·3 (11·9-19·4)
**Eastern Sub-Saharan Africa**																
Burundi	1·1 (0·8-1·5)	1·1 (0·6-1·8)	1·1 (0·6-1·8)	1·1 (0·5-2·1)	31·1 (16·9-45·8)	1·8 (1·6-2·1)	1·2 (0·6-1·8)	0·01 (0·0-0·05)	13·6 (11·9-15·5)	12·8 (11·2-14·6)	12·8 (11·1-14·6)	11·8 (10·4-13·4)	11·7 (9·0-14·7)	11·7 (9·0-14·8)	11·7 (9·0-14·8)	11·7 (8·9-14·8)
Comoros	7·6 (4·6-9·6)	8·4 (4·1-11·3)	8·4 (4·0-11·3)	8·8 (3·3-13·9)	0·0 (0·0-0·0)	0·0 (0·0-0·0)	0·0 (0·0-0·0)	0·0 (0·0-0·0)	11·5 (10·3-12·7)	9·9 (8·9-10·9)	9·8 (8·8-10·8)	8·8 (7·9-9·7)	11·7 (9·0-14·8)	11·7 (9·0-14·9)	11·7 (9·0-14·9)	11·8 (9·0-14·9)
Djibouti	7·3 (4·0-9·7)	8·4 (3·9-11·5)	8·3 (3·8-11·5)	8·6 (3·1-14·5)	0·0 (0·0-0·0)	2·8 (1·3-4·4)	0·0 (0·0-0·0)	0·0 (0·0-0·0)	16·2 (14·2-18·4)	14·1 (12·3-16·0)	13·9 (12·2-15·8)	12·1 (10·5-13·8)	11·7 (9·0-14·8)	11·7 (9·0-14·9)	11·7 (9·0-14·9)	11·8 (9·0-14·9)
Eritrea	8·7 (4·8-11·1)	10·2 (4·8-13·4)	10·3 (4·8-13·4)	11·4 (4·4-18·2)	50·8 (47·4-54·1)	0·0 (0·0-0·0)	0·3 (0·2-0·5)	0·3 (0·2-0·5)	16·7 (14·6-18·9)	15·4 (13·5-17·5)	15·3 (13·4-17·3)	13·8 (12·1-15·6)	11·7 (9·0-14·7)	11·7 (9·0-14·9)	11·7 (9·0-14·9)	11·8 (9·0-15·0)
Ethiopia	13·4 (10·2-22·7)	12·2 (8·8-18·1)	11·9 (8·5-18·2)	10·2 (5·2-18·1)	75·7 (69·6-81·8)	0·2 (0·03-0·3)	0·8 (0·4-1·2)	2·0 (0·4-6·5)	19·7 (17·4-22·3)	17·0 (15·0-19·2)	16·7 (14·7-18·9)	13·4 (11·8-15·2)	12·5 (9·7-15·9)	12·7 (9·8-16·0)	12·7 (9·8-16·0)	12·7 (9·8-16·0)
Kenya	10·3 (7·6-13·3)	10·5 (8·0-13·0)	10·5 (8·0-13·0)	10·5 (7·5-13·8)	0·6 (0·4-0·8)	0·4 (0·3-0·5)	0·0 (0·0-0·0)	0·0 (0·0-0·0)	13·2 (11·6-15·1)	13·2 (11·8-15·0)	13·2 (11·7-15·0)	13·2 (11·7-14·9)	16·9 (13·3-21·2)	16·9 (13·2-21·3)	16·9 (13·2-21·3)	16·9 (13·2-21·3)
Madagascar	3·2 (1·0-4·3)	2·2 (1·0-3·1)	2·2 (1·0-3·1)	1·9 (0·8-3·5)	0·0 (0·0-0·0)	0·9 (0·5-1·2)	0·4 (0·2-0·6)	0·4 (0·2-0·6)	14·0 (12·3-15·9)	12·4 (10·9-14·1)	12·3 (10·8-14·0)	11·2 (9·8-12·8)	11·7 (9·0-14·8)	11·7 (9·0-14·8)	11·7 (9·0-14·8)	11·7 (9·0-14·8)
Malawi	3·3 (1·6-9·4)	2·8 (1·4-8·0)	2·7 (1·4-8·0)	1·7 (0·4-7·4)	0·0 (0·0-0·0)	0·0 (0·0-0·0)	0·0 (0·0-0·0)	0·0 (0·0-0·0)	13·7 (12·4-15·1)	12·0 (11·1-13·1)	12·0 (11·0-13·0)	10·9 (9·9-12·2)	11·7 (9·0-14·8)	11·7 (9·0-14·8)	11·7 (9·0-14·8)	11·7 (9·0-14·8)
Mozambique	6·7 (4·9-8·8)	8·0 (5·0-10·7)	8·0 (5·0-10·7)	7·5 (3·7-13·0)	0·7 (0·4-1·0)	0·3 (0·2-0·4)	0·2 (0·1-0·3)	0·2 (0·1-0·3)	15·2 (13·6-16·8)	13·4 (12·3-14·6)	13·3 (12·2-14·4)	11·5 (10·6-12·4)	9·3 (7·2-11·7)	9·5 (7·4-11·9)	9·5 (7·4-11·9)	9·7 (7·5-12·1)
Rwanda	5·2 (3·5-6·9)	4·3 (3·0-5·6)	4·3 (3·0-5·6)	4·3 (2·2-7·2)	2·0 (0·4-3·6)	0·0 (0·0-0·0)	0·0 (0·0-0·0)	0·0 (0·0-0·0)	14·2 (12·8-15·8)	11·6 (10·5-12·8)	11·5 (10·4-12·7)	9·7 (8·7-10·8)	11·6 (9·0-14·7)	11·7 (9·0-14·8)	11·7 (9·0-14·8)	11·8 (9·0-15·0)
Somalia	8·7 (5·1-12·1)	10·3 (4·9-14·3)	10·4 (4·9-14·2)	11·1 (4·4-17·3)	9·0 (6·3-11·7)	11·4 (2·1-21·7)	30·3 (18·6-41·9)	162·5 (15·9-434·1)	18·9 (16·6-21·5)	17·9 (15·7-20·5)	17·9 (15·7-20·4)	16·6 (14·6-18·9)	11·7 (9·0-14·8)	11·7 (9·0-14·8)	11·7 (9·0-14·8)	11·7 (9·0-14·8)
South Sudan	5·8 (3·0-8·6)	7·2 (3·1-10·8)	7·3 (3·1-11·0)	9·1 (3·2-15·6)	28·4 (8·0-49·5)	24·5 (11·5-37·7)	6·3 (2·6-10·0)	115·2 (0·09-854·9)	19·8 (17·4-22·5)	18·8 (16·5-21·3)	18·7 (16·4-21·2)	17·4 (15·3-19·9)	11·7 (9·0-14·8)	11·7 (9·0-14·8)	11·7 (9·0-14·8)	11·7 (9·0-14·9)
Tanzania	9·4 (7·3-12·9)	9·1 (6·8-12·5)	9·1 (6·8-12·6)	9·0 (5·2-14·5)	0·3 (0·2-0·5)	0·0 (0·0-0·0)	0·0 (0·0-0·0)	0·0 (0·0-0·0)	16·5 (14·9-18·4)	13·4 (12·3-14·8)	13·3 (12·1-14·7)	11·3 (9·9-13·0)	13·3 (10·7-16·4)	13·6 (10·9-17·1)	13·7 (10·9-17·2)	13·7 (10·9-17·2)
Uganda	7·5 (4·3-9·3)	7·6 (4·3-10·0)	7·5 (4·2-10·0)	7·3 (3·5-12·1)	5·2 (3·8-6·5)	0·5 (0·4-0·6)	0·0 (0·0-0·0)	0·0 (0·0-0·0)	23·8 (21·7-26·4)	19·3 (17·9-21·0)	19·1 (17·7-20·8)	16·1 (14·3-18·3)	11·0 (8·8-13·3)	10·7 (8·6-13·1)	10·7 (8·6-13·0)	10·7 (8·6-13·0)
Zambia	10·7 (7·1-13·7)	10·4 (6·1-14·3)	10·3 (6·1-14·4)	9·2 (4·2-17·4)	0·0 (0·0-0·0)	0·0 (0·0-0·0)	0·0 (0·0-0·0)	0·0 (0·0-0·0)	16·1 (14·6-17·9)	13·9 (12·6-15·4)	13·7 (12·4-15·2)	11·0 (9·8-12·5)	11·5 (8·9-14·6)	11·5 (8·9-14·6)	11·5 (8·9-14·6)	11·5 (8·9-14·7)
**Central Sub-Saharan Africa**																
Angola	5·1 (3·4-9·2)	5·0 (3·1-8·9)	4·8 (3·0-8·7)	4·4 (1·8-9·4)	8·3 (1·3-16·0)	0·0 (0·0-0·0)	0·1 (0·02-0·2)	0·1 (0·02-0·2)	17·5 (15·8-19·4)	14·8 (13·3-16·4)	14·6 (13·1-16·1)	12·0 (10·8-13·3)	11·7 (9·1-14·8)	11·7 (9·1-14·8)	11·7 (9·1-14·8)	11·7 (9·1-14·8)
Central African Republic	6·8 (3·9-10·7)	7·1 (4·1-10·4)	7·1 (4·1-10·4)	6·9 (3·4-12·8)	0·0 (0·0-0·0)	8·2 (5·6-11·1)	3·9 (0·6-7·4)	0·05 (0·0-0·3)	14·9 (13·5-16·6)	14·2 (12·8-15·8)	14·2 (12·8-15·8)	14·9 (13·4-16·7)	11·7 (9·1-14·8)	11·7 (9·1-14·8)	11·7 (9·1-14·8)	11·7 (9·1-14·8)
Congo	6·0 (4·1-8·9)	5·0 (3·4-7·3)	5·0 (3·4-7·4)	5·1 (2·3-10·2)	16·6 (2·6-31·9)	0·0 (0·0-0·0)	1·0 (0·2-2·0)	0·0 (0·0-0·0)	14·8 (13·3-16·4)	13·1 (11·8-14·5)	13·0 (11·7-14·4)	11·6 (10·4-12·8)	11·7 (9·1-14·8)	11·7 (9·1-14·7)	11·7 (9·1-14·7)	11·7 (9·1-14·8)
Democratic Republic of the Congo	4·4 (3·4-5·4)	4·4 (3·3-5·5)	4·4 (3·3-5·5)	4·5 (2·8-6·8)	5·9 (3·2-8·7)	0·5 (0·5-0·6)	1·0 (0·2-1·9)	0·2 (0·0-1·3)	18·7 (16·7-20·8)	16·6 (15·1-18·3)	16·3 (14·9-18·0)	12·7 (11·5-14·2)	11·7 (9·1-14·8)	11·7 (9·1-14·8)	11·7 (9·1-14·8)	11·7 (9·1-14·8)
Equatorial Guinea	5·8 (3·6-8·6)	3·9 (2·1-6·8)	3·9 (2·0-6·8)	2·8 (0·8-6·9)	0·0 (0·0-0·0)	0·0 (0·0-0·0)	0·0 (0·0-0·0)	0·0 (0·0-0·0)	14·1 (12·7-15·6)	10·7 (9·6-11·9)	10·6 (9·5-11·8)	9·4 (8·4-10·4)	11·6 (9·0-14·8)	11·6 (9·1-14·7)	11·6 (9·1-14·8)	11·6 (9·0-14·8)
Gabon	5·7 (3·8-8·4)	5·1 (3·1-7·9)	5·1 (3·1-7·8)	4·7 (1·8-10·3)	0·0 (0·0-0·0)	0·0 (0·0-0·0)	0·0 (0·0-0·0)	0·0 (0·0-0·0)	14·3 (12·9-15·9)	11·9 (10·9-13·1)	11·8 (10·8-13·0)	10·6 (9·7-11·7)	11·7 (9·1-14·7)	11·6 (9·1-14·7)	11·6 (9·1-14·7)	11·6 (9·0-14·8)

Location	Indicator 17.19.2c: Percentage of well-certified deaths by a vital registration (VR) system among a country's total population (%)															
	2000	2015	2016	2030												
Sierra Leone	0·0 (0·0-0·0)	0·0 (0·0-0·0)	0·0 (0·0-0·0)	0·0 (0·0-0·0)												
Togo	0·0 (0·0-0·0)	0·0 (0·0-0·0)	0·0 (0·0-0·0)	0·0 (0·0-0·0)												
**Eastern Sub-Saharan Africa**																
Burundi	0·0 (0·0-0·0)	0·0 (0·0-0·0)	0·0 (0·0-0·0)	0·0 (0·0-0·0)												
Comoros	0·0 (0·0-0·0)	0·0 (0·0-0·0)	0·0 (0·0-0·0)	0·0 (0·0-0·0)												
Djibouti	0·0 (0·0-0·0)	0·0 (0·0-0·0)	0·0 (0·0-0·0)	0·0 (0·0-0·0)												
Eritrea	0·0 (0·0-0·0)	0·0 (0·0-0·0)	0·0 (0·0-0·0)	0·0 (0·0-0·0)												
Ethiopia	0·0 (0·0-0·0)	0·0 (0·0-0·0)	0·0 (0·0-0·0)	0·0 (0·0-0·0)												
Kenya	0·0 (0·0-0·0)	0·0 (0·0-0·0)	0·0 (0·0-0·0)	0·0 (0·0-0·0)												
Madagascar	2·6 (2·0-3·4)	3·9 (3·0-4·9)	4·0 (3·1-5·0)	5·5 (3·4-8·5)												
Malawi	0·0 (0·0-0·0)	0·0 (0·0-0·0)	0·0 (0·0-0·0)	0·0 (0·0-0·0)												
Mozambique	5·4 (4·3-6·8)	9·7 (7·4-12·4)	10·1 (7·8-13·0)	17·2 (10·9-24·7)												
Rwanda	0·0 (0·0-0·0)	0·0 (0·0-0·0)	0·0 (0·0-0·0)	0·0 (0·0-0·0)												
Somalia	0·0 (0·0-0·0)	0·0 (0·0-0·0)	0·0 (0·0-0·0)	0·0 (0·0-0·0)												
South Sudan	0·0 (0·0-0·0)	0·0 (0·0-0·0)	0·0 (0·0-0·0)	0·0 (0·0-0·0)												
Tanzania	0·0 (0·0-0·0)	0·0 (0·0-0·0)	0·0 (0·0-0·0)	0·0 (0·0-0·0)												
Uganda	0·0 (0·0-0·0)	0·0 (0·0-0·0)	0·0 (0·0-0·0)	0·0 (0·0-0·0)												
Zambia	0·0 (0·0-0·0)	0·0 (0·0-0·0)	0·0 (0·0-0·0)	0·0 (0·0-0·0)												
**Central Sub-Saharan Africa**																
Angola	0·0 (0·0-0·0)	0·0 (0·0-0·0)	0·0 (0·0-0·0)	0·0 (0·0-0·0)												
Central African Republic	0·0 (0·0-0·0)	0·0 (0·0-0·0)	0·0 (0·0-0·0)	0·0 (0·0-0·0)												
Congo	0·0 (0·0-0·0)	0·0 (0·0-0·0)	0·0 (0·0-0·0)	0·0 (0·0-0·0)												
Democratic Republic of the Congo	0·0 (0·0-0·0)	0·0 (0·0-0·0)	0·0 (0·0-0·0)	0·0 (0·0-0·0)												
Equatorial Guinea	0·0 (0·0-0·0)	0·0 (0·0-0·0)	0·0 (0·0-0·0)	0·0 (0·0-0·0)												
Gabon	0·0 (0·0-0·0)	0·0 (0·0-0·0)	0·0 (0·0-0·0)	0·0 (0·0-0·0)												
